# Effectiveness of smoking cessation interventions among adults: an overview of systematic reviews

**DOI:** 10.1186/s13643-024-02570-9

**Published:** 2024-07-12

**Authors:** Mona Hersi, Andrew Beck, Candyce Hamel, Leila Esmaeilisaraji, Kusala Pussegoda, Bradley Austin, Nadera Ahmadzai, Misty Pratt, Micere Thuku, Fatemeh Yazdi, Alexandria Bennett, Nicole Shaver, Niyati Vyas, Becky Skidmore, Brian Hutton, Douglas Manuel, Matt Morrow, Smita Pakhale, Justin Presseau, Beverley J. Shea, Julian Little, David Moher, Adrienne Stevens

**Affiliations:** 1 Knowledge Synthesis Group, Clinical Epidemiology Program, Ottawa Hospital Research Institute, Centre for Practice-Changing Research, 501 Smyth Road, Box 201, Ottawa, Ontario K1H 8L6 Canada; 2 Knowledge Synthesis and Application Unit, School of Epidemiology and Public Health, Faculty of Medicine, University of Ottawa, Ottawa, Ontario Canada; 3 Ottawa Hospital Research Institute, Ottawa, Ontario Canada; 4 Independent Information Specialist, Ottawa, ON Canada; 5 Department of Otolaryngology, University of Ottawa, Ottawa, Ontario Canada; 6 The Ottawa Hospital, Ottawa, Ontario Canada; 7 Department of Family Medicine, University of Ottawa, Ottawa, Ontario Canada; 8 School of Epidemiology and Public Health, Faculty of Medicine, University of Ottawa, 600 Peter Morand Crescent, Ottawa, Ontario K1G 5Z3 Canada; 9 Patient Representative, British Columbia, Vancouver, Canada; 10 School of Psychology, University of Ottawa, Ottawa, Ontario Canada

**Keywords:** Smoking cessation, Tobacco cessation, Pharmacotherapies, Behavioural therapies, Electronic cigarettes, Other therapies, Systematic review

## Abstract

**Background:**

This overview of reviews aims to identify evidence on the benefits (i.e. tobacco use abstinence and reduction in smoking frequency) and harms (i.e. possible adverse events/outcomes) of smoking cessation interventions among adults aged 18 years and older.

**Methods:**

We searched Medline, Embase, PsycINFO, Cochrane Database of Systematic Reviews, Database of Abstracts of Reviews of Effects, the CADTH Health Technology Assessment Database and several other websites for grey literature. Searches were conducted on November 12, 2018, updated on September 24, 2020, with publication years 2008 to 2020. Two reviewers independently performed title-abstract and full-text screening considering pre-determined inclusion criteria. Data extraction and quality assessments were initially completed by two reviewers independently (i.e. 73% of included studies (*n* = 22)) using A Measurement Tool to Assess Systematic Reviews-2 (AMSTAR 2), and the remainder done by one reviewer and verified by another due to resources and feasibility. The application of Grading of Recommendations Assessment, Development and Evaluation (GRADE) was performed by one independent reviewer and verified by another.

**Results:**

A total of 22 Cochrane systematic reviews evaluating the impact of smoking cessation interventions on outcomes such as tobacco use abstinence, reduction in smoking frequency, quality of life and possible adverse events were included. Pharmaceutical (i.e. varenicline, cytisine, nicotine replacement therapy (NRT), bupropion) and behavioural interventions (i.e. physician advice, non-tailored print-based self-help materials, stage-based individual counselling, etc.) showed to have increased smoking cessation; whereas, data for mobile phone-based interventions including text messaging, hypnotherapy, acupuncture, continuous auricular stimulation, laser therapy, electrostimulation, acupressure, St John’s wort, S-adenosyl-L-methionine (SAMe), interactive voice response systems and other combination treatments were unclear. Considering harms related to smoking cessation interventions, small/mild harms (i.e. increased palpitations, chest pain, nausea, insomnia, headache) were observed following NRT, varenicline and cytisine use. There were no data on harms related to behavioural therapies (i.e. individual or group counselling self-help materials, internet interventions), combination therapies or other therapies (i.e. laser therapy, electrostimulation, acupressure, St John’s wort, SAMe).

**Conclusion:**

Results suggest that pharmacological and behavioural interventions may help the general smoking population quit smoking with observed small/mild harms following NRT or varenicline. Consequently, evidence regarding ideal intervention strategies and the long-term impact of these interventions for preventing smoking was unclear.

**Systematic review registration:**

PROSPERO CRD42018099691

**Supplementary Information:**

The online version contains supplementary material available at 10.1186/s13643-024-02570-9.

## Background

### Prevalence and burden of tobacco smoking

The World Health Organization (WHO), under their 2013 Framework Convention on Tobacco Control, was able to commit 194 countries to a global target reduction in smoking rates of 30% by 2025 [1, 2]. In 2010, the global prevalence of current smokers was 22.1% and estimated to decrease to 18.9% in 2025 [2]. In Canada, between 1999 and 2020, there has been an overall reduction in the prevalence of current smokers (i.e. defined as those being daily and non-daily smokers) [3, 4]. According to the 2022 Canadian Tobacco and Nicotine Survey (CTNS), the prevalence of current cigarette smoking among adults aged 25 years and older was 11.7% (95% CI: 10.8 to 12.7), unchanged from 2021; however, the prevalence was higher among adult men compared to adult women (13.8% versus 9.8%) [5]. The prevalence of past-30-day use of at least one tobacco product was 10.7% (95% CI: 9.3 to 12.1) among young adults aged 20 to 24 years and 13.6% (95% CI: 12.6 to 14.6) among adults aged 25 years and older, all unchanged from 2021 [5]. The health economic burden due to the prevalence of smoking was approximately $16.2 billion Canadian dollars in 2012, comprising $9.5 billion in indirect costs (e.g. economic costs associated with increased morbidity and mortality) and $6.5 billion in direct costs (e.g. hospital expenditures, physician care, medications) [6].

Smoking continues to be one of the leading causes of preventable deaths globally [7]. In 2012, 45,464 deaths in Canada were attributable to smoking (approximately 599,390 potential years of life lost) and 993 deaths were due to secondhand smoke exposure [6]. In addition to various cancers (e.g. mouth, lung, bladder, cervix, colon, rectum) and cardiovascular disease (e.g. coronary heart disease, stroke, atherosclerosis, aortic aneurysm, peripheral heart disease), smoking can cause reproductive issues (e.g. infertility, spontaneous abortion, premature birth, low birth weight), neonatal death, sudden infant death syndrome, early menopause and osteoporosis, among other conditions like lung diseases (e.g. emphysema, chronic bronchitis, etc.) [8–15].

Smoking cessation, the act of discontinuing the use of tobacco smoking, has been shown to improve general, mental and physical health aspects of quality of life within a week of quitting and these improvements are maintained over time [7, 16–18]. A Canadian study found that men who had quit for 20 years had the same quality of life as those who had never smoked; this observation was even more beneficial for females, who only had to quit for 10 years [19]. Quitting smoking is beneficial at any age and reduces the excess morbidity risk experienced by smokers for a multitude of cancers, as well as outcomes related to cardiovascular disease, chronic respiratory disease/COPD, asthma and reproductive health [20].

As per the 2022 CTNS survey, the prevalence of former smoking was 23.2% among Canadians aged 15 years and older, unchanged from 2021 (22.7%) [5]. Among those reporting as former smoker, 4.2% had quit less than one year ago, 5.6% had quit between 1 and 2 years ago, 9.8% between 3 and 5 years ago and 80.4% had quit over 5 years ago [5]. About 62.4% of respondents reported independently quitting smoking as one of the cessation methods, whereas 39.5% reported reducing the number of cigarettes smoked, 28.2% reported switching to vaping and 26.3% reported using nicotine replacement products within the past 12 months [5]. Information on various smoking cessation interventions, including pharmacotherapies, behavioural therapies, exercise and other interventions can be found in Additional file 1. Different stop smoking interventions have been offered in randomized trials; however, the outcome criteria (e.g. measuring smoking abstinence, point prevalence abstinence, or continuous abstinence) and aspects of follow-up and analysis vary across trials leading to difficulties in interpretation [21]. West et al. proposed standardization based on six criteria (duration of abstinence of 6 or 12 months, self-report of abstinence, biochemical verification, following up with protocol violators, collecting follow-up data blind to specific allocation groups, using intention-to-treat (ITT) approach in analysis) to define smoking abstinence and provide guidance for analysis and trial data reporting, to address this issue arising from such variation between trials [21]. These have become known as the Russell Standard criteria enabling a meaningful comparison across trial results assessing smoking cessation interventions [21].

### Current guideline recommendations

#### Canadian guidelines

In 2011, the Canadian Action Network for the Advancement, Dissemination and Adoption of Practice-informed Tobacco Treatment (CAN-ADAPTT) released its guidelines for smoking cessation [22]. For guideline development, CAN-ADAPTT adopted the ADAPTE process consisting of three phases: planning and set-up, adaptation and development of the final product [23]. The guideline development group reviewed the extracted evidence from relevant pre-existing high-quality clinical practice guidelines (CPGs) and developed summary statements based on clarity of risk, benefits and quality of the supporting evidence. The grades of recommendations and levels of evidence were assigned using a modified GRADE approach [22], using the GRADE guiding table compiled by UpToDate [24]. Briefly, the guideline was separated into two cessation methods: (a) counselling and psychosocial approaches and (b) pharmacotherapy [22]. CAN-ADAPTT recommends combining counselling (i.e. self-help, individual/group, telephone quitline, web-based) or motivational interviewing and pharmacotherapy, when feasible. Counselling should be provided for a minimum of four sessions with incorporated problem-solving, skills training and providing support. Follow-up by healthcare professionals is recommended [22]. CAN-ADAPTT also provided separate guideline recommendations for specific sub-group populations: pregnant and breastfeeding individuals, youth, indigenous communities, hospitalized patients, people living with mental health conditions and people with addictions other than to tobacco [22].

The 2017 Registered Nurses Association of Ontario based their guideline recommendations from a systematic review capturing evidence from 6 international clinical practice guidelines and 53 peer-reviewed articles [25]. Briefly, the guidelines advocate for recommendations based on the level of evidence separated into the following areas: assessment (screen smoking patients), planning (develop intervention plan), implementation (provide intensive intervention and counselling on the use of pharmacotherapies and for pregnant individuals to provide intensive interventions in conjunction with NRT), evaluation (evaluate effectiveness until goals are attained) and education (continually updating education for health care professionals) [25]. The Association considered evidence for the effectiveness of e-cigarettes, hypnotherapy, laser therapy, electrostimulation, acupressure and acupuncture to be insufficient [25].

### Guidelines from international organizations

Various international guidelines have been published, all of which generally recommend behavioural interventions either alone or in combination with pharmacotherapies, such as NRT, bupropion and varenicline. The United Kingdom (UK) National Institute for Health and Care Excellence (NICE, 2023) recommends very brief advice, individual or group behavioural support, bupropion, short- and long-acting NRT, varenicline, nicotine containing e-cigarettes and/or in-person group seminar [26]. New Zealand’s Ministry of Health (2021) recommends the combination use of behavioural interventions and pharmacotherapies. It also stipulates that brief advice (approximately 30 s), behavioural support, NRT, bupropion, varenicline and nortriptyline are the most effective cessation aids [27]. The Scottish Intercollegiate Guidelines Network (2017) recommends varenicline or combination NRT alone or as part of a behavioural intervention and bupropion combined with a single NRT [28]. The United States Preventive Services Task Force (USPSTF) released their 2021 guideline, which recommends alone or the combined use of behavioural interventions and FDA-approved pharmacotherapies for tobacco cessation with high certainty of evidence [29]. The Royal Australian College of General Practitioners (RACGP) recommends brief smoking cessation advice and pharmacotherapies (NRT, varenicline, bupropion) accompanied by behavioural support based on efficacy, clinical suitability and patient preference [30]. The American Thoracic Society Clinical Practice Guideline (2020) also recommends pharmacotherapy choices to improve patient-centred care of tobacco dependence [31].

To address smoking cessation strategies relevant to the Canadian context and given a large amount of rapidly accumulating evidence, an approach capitalizing on available systematic review evidence was developed.

## Objective

The aim of this overview of reviews is to inform the development of the Canadian Task Force on Preventive Health Care’s (CTFPHC) clinical practice guideline on smoking cessation interventions in adults. This overview of reviews addresses the following key question (KQ): what are the benefits and harms of interventions to promote cessation of tobacco smoking in adults?

## Methods

We conducted an evidence review that occurred in two stages. First, the subject of this paper is an overview of systematic reviews that aims to evaluate the benefits and harms of various smoking cessation interventions for adults. Second is a systematic review update that aims to synthesize recent evidence on the benefits and harms of e-cigarettes as a smoking cessation intervention. The results of the present review were used to identify the candidate systematic review for the second stage and the results of stage two are reported elsewhere. For the purposes of this evidence review, tobacco smoking refers to any form of smoked tobacco (e.g. cigarettes, pipes, cigars, cigarillos, via water pipe or hookah) but excludes tobacco use for traditional or ceremonial purposes. We used the Preferred Reporting Items for Overviews of systematic reviews (PRIOR) (Additional file 2) as a guide for reporting, where relevant [32], and other reports to help us make informed decisions while conducting overview of reviews of healthcare interventions [33–35].

### Literature sources and search strategy

The search strategy was developed through an iterative process by an experienced medical information specialist in consultation with the review team. The MEDLINE search strategy was peer-reviewed prior to execution using the Peer Review of Electronic Search Strategies Checklist (Additional file 3) [36]. Using the multifile option and deduplication tool on Ovid, we searched Ovid MEDLINE® ALL, Embase Classic + Embase, PsycINFO, Cochrane Database of Systematic Reviews, Database of Abstracts of Reviews of Effects and the Health Technology Assessment database (Additional file 4) on November 13, 2018. We updated the search on September 24, 2020. The strategies used a combination of controlled vocabulary (e.g. ‘Smoking Cessation’, ‘Tobacco Use Disorder/drug therapy’, ‘Tobacco Use Cessation Products’) and free-text terms (e.g. quit smoking, nicotine replacement, vaping). The results were limited to the publication years 2008 to 2020 and, where possible, animal-only and opinion pieces were removed.

We searched for unpublished English or French language literature and reports using the Canadian Agency for Drugs and Technologies in Health (CADTH) Grey Matters checklist [37]. The list of websites searched for grey literature are included in Additional file 5. We also scanned reference lists of relevant systematic reviews and overviews for grey literature sources as well as references not captured by the electronic database search.

### Eligibility criteria

Cochrane systematic reviews with or without meta-analysis were selected for inclusion according to criteria specified in Additional file 6. Briefly, the overview focuses on general populations, those either specifically motivated or not motivated to quit or those living with mental illness in whom various interventions are compared with an inactive, minimally active or usual care control. To determine eligibility of comparators for a given analysis, we relied on information reported at face value by review authors. For example, we included comparators explicitly reported as ‘placebo’ by review authors even if it was apparent from review evidence tables that some trials in the analysis used a control condition other than placebo, including active controls (e.g. smoking cessation advice).We excluded analyses if authors explicitly signalled that trials with varying control conditions were included in the analysis (e.g. comparators identified as ‘placebo or no NRT’ where the latter could include various control conditions such as advice or counselling). Where comparators were not clearly reported by review authors (e.g. labelled only as ‘control’), we scrutinized review evidence tables to determine control conditions across trials; we excluded analyses where all trials had an active control condition or if there was a mix of both active and inactive control conditions across trials. Analyses that combined trials of various interventions, such that the effect of each cannot be isolated, were excluded post hoc due to limited utility of these data. For example, we would exclude analyses of ‘smoking cessation pharmacotherapies versus placebo’ where individual trials of different pharmacotherapy interventions (e.g. NRT, varenicline, bupropion) are combined in a meta-analysis. This is distinct from combination interventions where the intent is to test the synergistic effect of interventions; these interventions were relevant for inclusion as we restricted inclusion to Cochrane reviews, and the potential for overlapping outcome data was minimized [33]. Therefore, the extent of primary study overlap across reviews was not assessed and no attempt was made to assess the extent of concordance or discordance across potentially overlapping reviews. However, any instances of duplicate analyses across reviews are noted in the results section. To align with other smoking outcomes, we only included smoking reduction data if reported a minimum of 6 months from quit date or intervention initiation. To be consistent with included reviews, we considered smoking reduction data reported from quit date or intervention initiation between 24 and 26 weeks to match the 6-month criterion as the timing of outcome assessment.

### Study selection

Literature search results were downloaded and deduplicated in a reference manager (Reference Manager 12, Thomson Reuters, New York, USA) [38]. Remaining citations were uploaded to an online systematic review management software (DistillerSR, Evidence Partners, Ottawa, Canada) for study selection [39]. Two reviewers independently screened titles and abstracts of records according to the liberal accelerated method whereby a second reviewer only verifies records excluded by the first reviewer. References were randomized to ensure that one reviewer could not determine whether a given reference was excluded by another reviewer. Full-text screening was conducted by two reviewers independently. Disagreements were resolved by consensus or third-party arbitration.

Screening forms were piloted by all reviewers prior to screening commencement. Where necessary, articles were ordered via interlibrary loan and included if received within 30 days. The exclusion of records due to unavailability is noted in Additional file 7.

### Data extraction

Data extraction was conducted using DistillerSR following a calibration exercise. Data extraction items in detail are available in Additional file 8. Of the reviews included (*n* = 22), data were extracted independently by two reviewers for 73% of reviews. For the remainder of reviews, data were extracted by one reviewer and verified by a second reviewer due to resources and feasibility. For both procedures, disagreements were resolved by consensus or third-party arbitration.

We collected review characteristics and outcome data as reported by review authors. For meta-analyses, we reported the pooled effect estimates along with corresponding confidence intervals and heterogeneity statistics (e.g. *I*^2^). We also redrew forest plots for all relevant analyses (Additional file 9) using the Cochrane Review Manager software [40]. Where a meta-analysis was not performed, we collected outcome data as reported by review authors (e.g. vote count, narrative synthesis). Data were collected for all relevant timepoints of follow-up. We did not consult primary studies for the purposes of data extraction, risk of bias assessments or for checking the accuracy of data reported within a review.

Primary study characteristics were nearly always reported in aggregate at the review level (i.e. across all included studies) rather than at the analysis level, impeding their utility for this overview. Using data reported in review evidence tables, we looked across studies to produce analysis-specific aggregate information on populations and outcome measurement methods including biochemical validation of smoking outcomes, use of co-interventions, proportion of participants receiving specialized behavioural counselling, as well as any sources of indirectness which was used to inform assessment of the GRADE indirectness domain. As risk of bias information was also typically reported in aggregate across all studies rather than for each analysis, we developed risk of bias figures for each according to review authors’ assessments (Additional file 10). These figures were used to inform rating of the GRADE risk of bias domain.

Populations were coded as ‘general/mixed’ if all included studies recruited an unrestricted population of smokers or where populations across trials varied with no specific subpopulation forming a majority. If most trials were of a specific subpopulation of smokers (e.g. smokers motivated to quit), we coded accordingly. Where review authors made explicit statements regarding the applicability of the evidence to a specific subpopulation of smokers or where they restricted inclusion to a subpopulation, we coded the population in accordance with this information even if it was not made explicit in review evidence tables. Population details for each analysis are outlined in the GRADE tables (Additional file 11).

### Quality assessment

Quality assessment of reviews was assessed using the 16-item AMSTAR 2 instrument [41] (Additional file 12). Overall rating of quality was determined according to the fulfilment of critical and non-critical items, as either high (no critical flaws and a maximum of one non-critical weakness), moderate (no critical flaws and more than one non-critical weakness), low (one critical flaw with/without non-critical weaknesses) or critically low (more than one critical flaw with/without non-critical weaknesses). Quality assessment was conducted independently by two reviewers for 73% of included reviews (*n* = 22). For the remainder, assessments were made by one reviewer and verified by another due to resources and feasibility. For both procedures, disagreements were resolved by consensus or third-party arbitration.

### Subgroup analysis

Subgroup analyses have been reported in Additional file 13 and variables of interest for data extraction are listed in Table 1.

**Table 1:** Variables of interest for subgroup analysis

Category of analysis	Variables of interest
Populations	• Fewer versus more quit attempts • Opportunistic versus individuals seeking treatment (or based on motivation to quit) • Baseline level of nicotine dependence (e.g. using a validated scale or cigarettes per day as a proxy) • By demographic factors (age, socio-economic status (SES), sex, ethnicity, (lesbian, gay, bisexual, transgender, queer and others (LGBTQ+)) • By comorbid conditions (e.g. mental illness, Human immunodeficiency virus infection, cardiovascular disease, chronic obstructive pulmonary disease (COPD), obesity, substance use disorder)
Intervention-related variables	• Dose, type, duration, number of sessions • Specific forms of an intervention (e.g. yoga as a form of exercise) • Behavioural change technique (e.g. providing information on consequences of smoking, explaining the importance of abrupt cessation, receiving prompt commitment from the patient)
Settings	• Family medicine clinics • Walk-in clinics • Smoking cessation clinics • Urgent care facilities • Emergency departments • Public health units • Pharmacies • Dental offices • Behavioural health/substance use treatment facilities (ambulatory or outpatient) • Telehealth • Academic research settings
Other variables	• By industry funding status

### Evidence synthesis

We describe the results for each included analysis, along with reasons for downrating the certainty of evidence, narratively and within GRADE tables (Additional file 11). Given that we did not consult primary studies and relied solely on data as reported by review authors, indirect comparisons of treatments using simple (e.g. comparing 95% confidence intervals) or complex (e.g. network meta-analysis) methods were not performed, as stated in our protocol. As aforementioned, no attempt was made to assess concordance or discordance across reviews with similar scope.

For binary data meta-analyses, review authors typically reported relative effects (i.e. risk or odds ratios) which were used to calculate the absolute measure of intervention effects based on control event rates presented in the reviews. In the narrative summary of results, we report absolute effects as number per 1000 patients. Corresponding relative measures of effect are reported in GRADE tables (Additional file 11). Where there was insufficient information to calculate absolute effects, we relied on the reported relative effect and did not seek additional sources to inform baseline risks.

We followed authors’ main analyses or subgroups in lieu of main analyses. Further, where review authors performed GRADE assessments for subgroups in lieu of the main analysis, we followed suit. Credibility of subgroup assessments were guided by the GRADE working paper on inconsistency and criteria developed by Sun and colleagues (2010 and 2014) [42–44]. Credibility was only performed for subgroups that served as the main analysis (i.e. those for which we performed GRADE assessments). Results of subgroup credibility assessments are reported in GRADE Summary of Findings tables (Additional file 11).

### Grading the certainty of the evidence

We operationalized GRADE methods to determine the certainty of evidence in light of how information was presented in the Cochrane reviews (e.g. risk of bias ratings) and incorporating guidance for overviews made available by Cochrane in 2019 [45]. Assessments were performed by one person and verified by a second person; any remaining discrepancies were discussed for consensus. GRADE guidance for footnotes was consulted and implemented [46]; a detailed approach was used to elaborate on assessments and for additional information such as whether co-interventions were provided. Maximum downrating for a given domain was two levels, considering partial demerit where warranted, except for publication bias which was downrated by a maximum of one level.

For risk of bias, we used authors’ ratings for individual domains at face value to inform our assessments and any additional information provided by authors in the text of their review. For example, if performance bias was not assessed but authors state that it was not possible due to the nature of the intervention, we interpreted this as high risk for that domain. Where not already incorporated by review authors, we included an assessment of whether biochemical validation was used for smoking outcomes. For domains not assessed by authors, excluding selective reporting bias, we interpreted these as an unclear risk. Typically, most biases outlined in the Cochrane risk of bias tool [47] were considered to inform the rating with the exception of selective reporting (considered only if issues were identified by authors) and the ‘other’ bias domain (typically only considered issues of baseline imbalance to reduce variability across reviews, except where noted).

Eligibility criteria were used to guide our rating of indirectness except for specialized behavioural interventions which were considered direct evidence because the mix of specialized and general interventions were considered by the guideline Working Group as either reflective of practice or referral to counselling; as this information was not provided in aggregate, we extracted it from reviews. The proportion of indirectness of a given analysis, along a continuum, was used to inform the rating.

To assess imprecision and to establish the target of certainty ratings, extracted outcome data (i.e. including relative and absolute effects) were provided to the guideline Working Group to make their partially contextualized judgements on effect sizes (i.e. trivial, small, moderate, or large) for a given intervention or comparator and considering other contextual factors as necessary. Information on the procedure of effect size ratings and final effect size judgements can be found in Additional file 14.

For syntheses without meta-analysis, additional guidance made available by GRADE was used [48]. Guidance typically used for inconsistency and publication bias were followed without special considerations [44].

If information was missing to facilitate the rating of a particular domain, we provided a rating range, which was also reflected in the overall certainty rating. If authors performed a GRADE assessment, we recorded that rating and any accompanying footnote information for downrating in the Summary of Findings (SoF) table (Additional file 11) for readers’ comparison.

### Changes from protocol

Considering the substantial amount of evidence that was encountered during the conduct of the overview and potential for several hundred GRADE analyses, several protocol amendments were made in consultation with the guideline Working Group, for feasibility. First, we limited inclusion to Cochrane systematic reviews as evidence suggests that review findings from this source are more likely to have GRADE ratings than non-Cochrane reviews [49]. Given the substantial number of analyses reported within relevant Cochrane reviews, we further limited to comparisons and populations of greatest relevance to the Working Group and their recommendations on the suite of effective and suitable interventions available to physicians and patients. We further excluded comparative effectiveness data as well as data for specific subpopulations (e.g. pregnant smokers, smokers defined by demographic factors, smokers with co-morbidities other than those living with mental illness) as mentioned under the key questions 1b and 1c of the protocol [50].

## Results

### Search results

We identified 4436 citations from database searches and an additional 43 from the grey literature following the previous search (i.e. until November 2018) and updated search (i.e. until September 2020). We screened the titles and abstracts of 4276 unique records. In total, 2244 records advanced to full-text screening including 1 newly published citation brought forth by the guideline Working Group. We excluded 1890 citations at full-text screening; a list of excluded citations with reason for exclusion is provided in Additional file 7.

**Figure 1:**
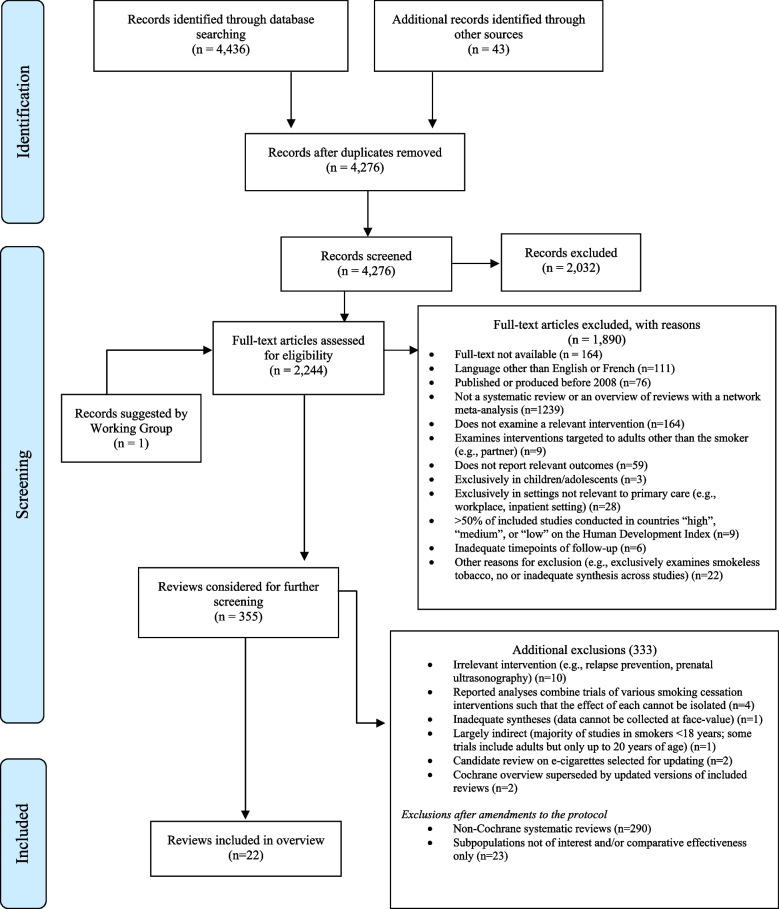
PRISMA flow diagram

Following protocol amendments, we excluded an additional 333 citations (Fig. 1, bottom right). Of these, 290 were non-Cochrane systematic reviews. Some excluded reviews (*n* = 23) were Cochrane systematic reviews that only reported comparative effectiveness data or examined subpopulations of smokers excluded by the guideline Working Group (e.g. smokers with COPD, pregnant/postpartum smokers). From the overview, we also excluded the candidate Cochrane electronic cigarette review as it was updated in the second part of this report [51]. A Cochrane overview of reviews and network meta-analysis, which would have contributed solely placebo-controlled analyses, was also excluded as it was superseded by updated versions of the included reviews of the pairwise placebo-controlled comparisons [52]. Reasons for exclusion of the remaining nine reviews are outlined in Fig. 1. Twenty-two Cochrane systematic reviews were included (i.e. inclusive of four Cochrane systematic reviews identified through an updated search) in the overview.

### Characteristics of included reviews

Twenty-two systematic reviews reporting 131 relevant analyses involving pharmacotherapies, behavioural interventions, other therapies and combination interventions were included. Reviews were published between 2010 and 2020 and the number of included studies ranged from none to 136. Of the 13 reviews reporting the total number of participants across included studies, the range of participants was 1120 to approximately 4.7 million. The largest review of 4.7 million participants examined the effects of automated telephone communication systems for prevention and management of various conditions, only one of which was smoking cessation; therefore, only a subset of studies (i.e. 10 of 132) was relevant to this overview. Review characteristics are summarized in Additional file 15.

A majority (68%) of systematic reviews typically included general (i.e. unrestricted) smokers as well as specific smoking subpopulations, as per eligibility. Three reviews sought studies of smokers motivated or wishing to quit [53–55]. One review of harm reduction strategies included studies of smokers with no immediate intention to quit [56]. Another review intended to examine the effect of advice in smokers with severe mental illness but found no studies [57]. Remaining reviews included studies of smokers with current and past depression [58] and schizophrenia or schizoaffective disorder [59]. Four (18%) reviews excluded studies of adolescents and five (23%) excluded studies of pregnant smokers. None of the reviews restricted inclusion to studies conducted in a specific setting; they typically included studies from various settings including workplaces, schools, primary care, smoking cessation clinics, medical specialist settings, and in-patient settings including hospital and substance use treatment facilities.

Of the 22 reviews reporting outcome data, all but one review [60] reported on smoking abstinence. Three reviews (14%, *n* = 3) reported data on smoking reduction, nine (41%, *n* = 9) on adverse events [53, 54, 56, 59, 61–65], two (9%, *n* = 2) on weight gain [56, 60] and three (14%, *n* = 3) on changes in emotional or mental state [59, 66, 67]. None of the reviews reported on quality of life or loss of social group.

### AMSTAR 2 rating

Most reviews (i.e. 12 studies; (54%)) were rated as having critically low quality. The remainder were of low (i.e. 5 studies; 23%) or moderate (i.e. 5 studies; 23%) quality. Regarding the critical domains, deficiencies were primarily observed with item 11 with only 24% of reviews reported using appropriate methods for meta-analysis of randomized controlled trials (RCTs) and non-randomized studies. Two (10%) reviews had inadequate search strategies, seven (33%) failed to use adequate methods for assessing risk of bias for RCTs and/or non-randomized studies, eight (38%) did not consider risk of bias in the interpretation of results and eight (38%) did not investigate publication bias or at least the intention to do so (Additional file 16).

### Risk of bias of primary studies

All reviews assessed the risk of bias of included studies using the Cochrane risk of bias tool [47]. However, there was variation in terms of which domains of the tool were assessed as well as how the domains were operationalized across reviews. For example, some but not all reviews assessed attrition and selective reporting biases. There was variation across reviews in the assessment of blinding with some combining performance and detection bias and others assessing them as separate domains. Some reviews added domains in relation to special considerations; for example, Farley [60] examined post-cessation weight gain in abstinent smokers and evaluated the definition of abstinence as part of the risk of bias assessment. Use of biochemical validation for smoking outcomes was inconsistently accounted for in risk of bias assessments across reviews.

As mentioned above, risk of bias figures was re-drawn for each analysis to inform GRADE assessments (Additional file 10). The risk of bias across studies for each analysis is specified in the GRADE table footnotes (Additional file 11).

### Included analyses

Pharmacotherapies were examined in 71 analyses from seven reviews. Data were available for all pharmacotherapies of interest, namely, varenicline, cytisine, bupropion and nicotine replacement therapy (NRT), all of which were compared to placebo (Fig 2a). Twenty-two (31%) analyses covered smoking abstinence, 9 (13%) smoking reduction, 22 (31%) adverse events, 10 (14%) post-cessation weight gain and eight (11%) changes in emotional or mental state.

Thirty-one analyses from eleven reviews compared a behavioural intervention to no intervention, assessment only, usual care or a minimal intervention. Behavioural interventions included self-help materials, physician advice, individual counselling, group therapy, internet interventions, telephone-based interventions (e.g. telephone counselling, interactive voice response systems, short messaging service) and various stage-based interventions including expert systems, interactive computer programmes, advice and counselling (Additional file 17: Figure 2b). A majority (87%) of the analyses were specific to smoking abstinence; three analyses were of adverse events and one of change in emotional state.

Three reviews, contributing eight analyses, examined the effect of other therapies on smoking abstinence (Fig 2c). Interventions included hypnotherapy, acupuncture, continuous auricular stimulation, laser therapy, electrostimulation, St. John’s wort and S-adenosyl-L-methionine (SAMe). Inactive control conditions included placebo, sham and wait-list or no intervention. The effect of e-cigarettes on smoking abstinence, reduction, adverse events and weight gain in smokers not interested in quitting was examined in one review contributing seven analyses [56].

Combination interventions were from 6 reviews covered 14 analyses and included combination behavioural interventions (e.g. telephone counselling plus self-help materials, internet interventions plus behavioural support) or a combination of behavioural and pharmacotherapy interventions (e.g. behavioural support and phone calls plus NRT) (Additional file 17: Figure 2d). These interventions were compared to no intervention, usual care or a minimal intervention. Eight analyses (57%) were of smoking abstinence and six (43%) of smoking reduction. Information on included analyses for different smoking interventions can be found in Additional file 17.

1. Pharmacotherapies

*Varenicline versus placebo*

Varenicline—abstinence/cessation in general/mixed population of smokers

Most trials reported biochemically validated continuous/sustained abstinence and provided behavioural co-interventions to both trial arms. Except where noted, varenicline was typically provided as 2 mg/day for 12 weeks.

At longest follow-up of 6 or more months, 138 more people per 1000 (95% CI: 118 to 159 more per 1000; 27 trials, 12,625 participants; *I*^2^ = 60%) on varenicline compared with placebo stopped smoking (rating down once for inconsistency). The certainty of the evidence was rated as moderate. Effectiveness was also shown with the 6-month follow-up results and the certainty of the evidence was also rated as moderate (Additional file 11: Table 9; Figure 2a (Additional file 17)).

For extended (long-term) varenicline treatment (2 mg/day for 6–12 months) compared to placebo, 177 more per 1000 (95% CI: 121 to 249 more; 4 trials, 2170 participants; *I*^2^ = 78%) stopped smoking (rating down twice for inconsistency and once for indirectness, the latter mainly from specialized medical settings). The certainty of evidence was rated as very low. Also, for the low (<2 mg/day) dose of varenicline compared to placebo at 12 months follow-up, 111 more per 1000 (95% CI: 57 to 182 more; *n* = 4, 1266 participants; *I*^2^ = 68%) stopped smoking (rating down for risk of bias, and/or inconsistency, and imprecision). The certainty of evidence was rated as very low. At 12 months follow-up, variable dose varenicline compared to placebo resulted in 125 more people per 1000 abstinent (95% CI: 78 to 183 more; trials 6, 1789 participants) being (rating down for inconsistency and imprecision). The certainty of evidence was low (Additional file 11: Table 10, 11, and 12; Figure 2a (Additional file 17)).

Varenicline—abstinence/cessation in smokers reducing to quit

With an approach to reduce-to-quit, 179 more per 1000 participants (95% CI: 116 to 266 more; *n* = 1, 1510 participants) taking varenicline for 6 months were abstinent at 12 months compared to placebo (rating down once for imprecision). Participants in both study arms received behavioural co-interventions [62]. The certainty of the evidence was rated as moderate (Additional file 11: Table 13; Figure 2a (Additional file 17)).

Varenicline—abstinence/cessation in smokers not motivated to quit

Among smokers with no immediate intention to quit all tobacco use, 68 more participants per 1000 (95% CI: 10 fewer to 245 more; *n* = 1, 218 participants) taking varenicline (2 mg/day for 2–8 weeks) were point prevalence abstinent at 6-month follow-up as compared to placebo (rating down once for risk of bias and twice for imprecision; very low certainty). Participants in both groups received a behavioural co-intervention (Additional file 11: Table 32; Figure 2a (Additional file 17)) [56].

Varenicline—abstinence/cessation in smokers with schizophrenia, bipolar or other psychiatric disorder

At 6-month follow-up, 104 more people per 1000 (95% CI: 67 to 152 more; 4 trials, 2332 participants; *I*^2^ = 0%) quit smoking on varenicline (2 mg/day). Behavioural co-intervention(s) were provided in all studies and all but one trial reported continuous/sustained abstinence [62]. The certainty of the evidence was rated as high (Additional file 11: Table 14; Figure 2a (Additional file 17)).

Among smokers with schizophrenia or schizoaffective disorder who were interested in quitting, 94 more participants per 1000 on varenicline (2 mg/day for about 12 weeks) (95% CI: 8 fewer to 866 more; single trial, 128 participants) were point prevalence abstinent at 6-month follow-up (rating down once for risk of bias and twice for imprecision) (Additional file 11: Table 55; Figure 2a (Additional file 17)) [59]. The certainty of the evidence was rated as very low.

Varenicline—abstinence/cessation in smokers with depression who were motivated/wishing to quit

More participants receiving varenicline (2 mg/day), 101 more per 1000 participants (95% CI: 29 to 209 more; single trial, 523 participants) were abstinent at 12 months follow-up (rating down once for imprecision). All participants received a behavioural co-intervention [62]. The certainty of the evidence was rated as moderate (Additional file 11: Table 15; Figure 2a (Additional file 17)).

Varenicline—abstinence/cessation in smokers who previously failed to quit on varenicline but are motivated/wishing to try quitting again

At 12 months follow-up in smokers who previously failed to quit on varenicline, 168 more per 1000 (95% CI: 65 to 382 more; single trial, 494 participants) remained abstinent (rating down once for risk of bias and imprecision). Participants in both arms received a behavioural co-intervention [62]. The certainty of the evidence was rated as low (Additional file 11: Table 16; Figure 2a (Additional file 17)).

Varenicline—reduction in number of cigarettes/day from baseline in smokers with schizophrenia or schizoaffective disorder

Among continued smokers with schizophrenia or schizoaffective disorder who were interested in quitting, review authors report no statistically significant difference between groups at 6 months regarding reduction in number of cigarettes per day from baseline. It is unclear whether this is based on confidence intervals or *p*-values; we interpret this to mean that there may be little to no difference between groups (rating down once for risk of bias) (Additional file 11: Table 55; Figure 2a (Additional file 17)) [59]. We were unable to assess imprecision and provide an overall certainty rating due to missing information (i.e. number of events or sample size).

Varenicline—weight gain in smokers motivated/wishing to quit

Across analyses, weight gain was assessed in biochemically validated abstinent smokers. Most trials provided behavioural co-intervention(s) to both study groups.

Compared to placebo, varenicline (2 mg/day) likely results in little to no difference in post-cessation weight gain at end of treatment (MD −0.41 kg, 95% CI: 0.63 to 0.19 lower; 11 trials, 2008 participants; *I*^2^ = 42%) (rating down once for risk of bias to also reflect concerns with inconsistency) in smokers motivated to quit. The certainty of the evidence was rated as moderate. These effects were sustained at 6 or 12 months follow-up (Additional file 11: Table 20; Figure 2a (Additional file 17)) [60]. The certainty of the evidence was rated as low (rating down once for risk of bias and imprecision) and moderate (rating down once for imprecision).

Low-dose varenicline (1 mg/day) may result in little to no difference in post-cessation weight gain by 0.12 kg at end of treatment (MD 0.12 kg, 95% CI: 0.68 lower to 0.43 higher; 3 trials, 254 participants; *I*^2^ = 60%) (rating down once for inconsistency and imprecision). The certainty of the evidence was rated as low (Additional file 11: Table 21; Figure 2a (Additional file 17)) [60].

Varenicline—change in mental state in smokers with schizophrenia or schizoaffective disorder

Most trials examined varenicline 2 mg/day for about 8 to 12 weeks and behavioural co-interventions were provided to both groups in most trials. Change in mental state outcomes included positive (e.g. hallucinations, delusions), negative (e.g. anhedonia, avolition), depressive and general symptoms of schizophrenia. Various outcome measurement tools were used including, but not limited to, the Positive and Negative Syndrome Scale (PANSS), Hamilton Depression Rating Scale (HAM-D) and Brief Psychiatric Rating Scale. Participants were interested in quitting in a few of the trials. Review authors analysed studies according to the primary study objective.

In trials with a primary aim of smoking cessation (*n* = 2), two reported on positive symptoms and one trial each on negative and depressive symptoms. Review authors report that there was no significant difference between groups at end of treatment for all symptoms, but it is unclear whether this is based on confidence intervals or *p*-values; we interpret this to mean that there may be little to no difference between groups [59]. We were unable to assess imprecision and provide an overall certainty rating due to missing information (i.e. number of events or sample size).

Three trials had a primary aim other than smoking cessation, reduction or relapse. These trials examined the effect of varenicline on cognitive function (*n* = 2) or alcohol dependence (*n* = 1). A single trial included both smokers and non-smokers; whereas the other trials either only recruited smokers or reported symptom data for smokers only. One trial had a small sample size (ten participants) due to recruitment issues. For all symptoms (i.e. positive, negative, depressive and general symptoms of schizophrenia), review authors reported either no significant changes during varenicline treatment or no significant difference between groups. Regarding the latter, it is unclear whether this is based on confidence intervals or *p*-values; we interpret this to mean that the confidence interval around the best estimate of effect includes the possibility of little to no difference between groups and more participants experiencing symptoms in one group over another (rating down once for risk of bias) (Additional file 11: Table 55; Figure 2a (Additional file 17)) [59]. We were unable to assess imprecision and provide an overall certainty rating due to unclear reporting of the sample size.

Varenicline—adverse events in general/mixed population of smokers

Most trials examined varenicline 2 mg/day for 12 weeks, provided both study groups with behavioural co-intervention and measured the outcome over a range of timepoints.

Nausea, insomnia, abnormal dreams, headache and serious adverse events occurred more frequently in those taking varenicline. Compared to placebo:

- 192 more per 1000 people on varenicline experienced nausea (95% CI: 169 to 216 more; *n* = 32, 14,963 participants; *I*^2^ = 22%). The certainty of the evidence was rated as high.
- 41 more per 1000 experienced insomnia (95% CI: 29 to 54 more; *n* = 29, 14,447 participants; *I*^2^ = 0%). The certainty of the evidence was rated as high.
- 64 more per 1000 had abnormal dreams (95% CI: 50 to 79 more; *n* = 26, 13,682 participants; *I*^2^ = 62%). The certainty of the evidence was rated as moderate (rating down once for inconsistency).
- 17 more per 1000 experienced headaches (95% CI: 7 to 30 more; *n* = 25, 13,835 participants; 27%). The certainty of the evidence was rated as high.
- 7 more per 1000 had at least one serious adverse event (defined as ‘any untoward medical occurrence that resulted in death, was life-threatening, required inpatient hospitalization or prolongation of existing hospitalization, resulted in persistent or significant disability or incapacity, or resulted in a congenital anomaly or birth defect’) (95% CI: 1 to 13 more; *n* = 29, 15,370 participants; *I*^2^ = 0%). The certainty of the evidence was rated as high.

Compared to placebo, 3 more people per 1000 (95% CI: ,1 fewer to 9 more; 21 trials, 8587 participants; *I*^2^ = 0%) taking varenicline had a cardiac adverse event including death (rating down once for risk of bias and once for indirectness due to setting and inclusion of quitters) as one of the serious adverse events. The certainty of the evidence was rated as low. Six more participants per 1000 (95% CI: 0 fewer to 12 more; 26 trials, 15,000 participants; *I*^2^ = 0%) taking varenicline experienced at least one serious adverse event during or immediately after treatment. The certainty of the evidence was rated as high (Additional file 11: Table 17; Figure 2a (Additional file 17)) [62].

One fewer participant per 1000 (95% CI: 6 fewer to 3 more; *n* = 36, 16,189 participants; *I*^2^ = 0%) taking varenicline experienced depression compared to placebo. The certainty of the evidence was rated as high. Two fewer participants per 1000 (95% CI: 4 fewer to 0 fewer; *n* = 24, 11,193 participants; *I*^2^ = 0%) on varenicline had suicidal ideation. The certainty of the evidence was rated as high (Additional file 11: Table 17; Figure 2a (Additional file 17)) [62].

Four studies reported discontinuation data narratively. Across three studies, treatment discontinuation ranged from 9.5 to 28% with varenicline and from 8 to 10% in the placebo group. In the fourth study, where study discontinuation was assessed during the 12-week varenicline open label phase, 32% exited the study because of discontinuation, non-adherence to protocol and relapse. We were unable to assess imprecision and provide an overall certainty rating due to missing information (i.e. number of events or sample size) (Additional file 11: Table 17; Figure 2a (Additional file 17)).

Varenicline—adverse events in smokers not motivated to quit

Review authors indicate that there was no difference between groups regarding treatment discontinuation due to adverse events, but it is unclear whether this is based on confidence intervals or *p*-values; we interpret this to mean that the confidence interval around the best estimate of effect includes the possibility of little to no difference between groups and more participants experiencing the outcome in one group over another (Additional file 11: Table 32; Figure 2a (Additional file 17)) (rating down once for risk of bias and once or twice for imprecision). We were unable to assess imprecision definitively because the sample size was unclear. Participants in both groups received a behavioural co-intervention [56].

Varenicline—adverse events in smokers with schizophrenia or schizoaffective disorder

Most trials examined varenicline 2 mg/day for about 8 to 12 weeks and behavioural co-interventions were provided to both groups in most trials. Participants were interested in quitting in a few of the trials. Review authors analysed studies according to the primary study objective.

Two trials had a primary aim of smoking cessation. One trial reported no suicidal ideation. This study reported exacerbation of side effects in the varenicline arm, namely, constipation, insomnia and nausea. In the second trial, two participants with a history of suicide attempts assigned to varenicline were hospitalized; one of the participants overdosed and had a seizure resulting in hospitalization. In total, the trial reported 13 serious adverse events occurring in 9 participants assigned to varenicline and 1 participant assigned to placebo (2 varenicline participants experienced 3 serious adverse events related to treatment). One death from accidental drowning occurred in the varenicline arm during the long-term follow-up period (off-treatment); the event was not related to treatment according to authors. No treatment-related adverse events occurred in placebo arm. This second trial also reported no difference between groups regarding other adverse events including neuropsychiatric serious adverse events and study discontinuation. The most common adverse events occurring in the varenicline arm were nausea (23.8%), headache (10.7%) and vomiting (10.7%) (Additional file 11: Table 55; Figure 2a (Additional file 17)) (rating down once for risk of bias and once or twice for imprecision) [59]. We were unable to assess imprecision definitively because the sample size was unclear.

Three trials had a primary aim other than smoking cessation, reduction or relapse. These trials examined the effect of varenicline on cognitive function (*n* = 2) or alcohol dependence (*n* = 1). Adverse events data from two trials included both smokers and non-smokers; the remaining trial recruited smokers with alcohol dependence and had a small sample size (ten participants) due to recruitment issues). In one trial recruiting participants with both smoking and alcohol dependence, one participant assigned to varenicline withdrew from the trial due to passive suicidal ideation (7 days after starting varenicline), vomiting and irritability. The other two trials reported that among smokers and non-smokers, no participants had suicidal ideation and no increase in suicidal ideation occurred in those assigned varenicline, respectively. One trial reported a trend toward reduced psychosis in the varenicline arm compared to placebo; both smokers and non-smokers were included in this analysis, but study authors report no difference in treatment effect related to smoking status. In the second trial of smokers and non-smokers, two participants in each study arm withdrew due to exacerbation of psychotic symptoms. One trial reported that among smokers and non-smokers, there was no difference between varenicline and placebo groups regarding common side effects of varenicline. However, the two other trials reported higher rates of common side effects in the varenicline arm (e.g. nausea, headache, vomiting, abdominal pain). In one of these studies, one patient withdrew due to nausea and vomiting. Given what was reported, the one trial in smokers with alcohol dependence raised concerns for review authors regarding the safety and tolerability of varenicline in schizophrenic patients (rating down once for risk of bias, once for inconsistency ) (Additional file 11: Table 55; Figure 2a (Additional file 17)) [59]. We were unable to assess imprecision or provide an overall certainty rating because the sample size was unclear.

Review authors note that across all varenicline trials in the review, 2 of 144 participants on varenicline experienced suicidal ideation or behaviour. This includes one participant who overdosed in a smoking cessation trial as well as one participant with passive suicidal ideation in a trial examining varenicline for purposes other than smoking cessation [59].

*Cytisine versus placebo*

Cytisine—abstinence/cessation in general/mixed population of smokers

At 2 years of follow-up, 79 more participants per 1000 taking cytisine (1.5 mg tablets for 25 days with variability in daily dose) were abstinent compared to placebo (95% CI: 31 to 141 more; *n* = 1, 1214 participants) (rating down twice for risk of bias and once for imprecision). The certainty of the evidence was rated as very low. Abstinence was not biochemically validated and review authors state that behavioural support was kept to a minimum (Additional file 11: Table 8; Figure 2a (Additional file 17)) [62].

Cytisine—abstinence/cessation in smokers motivated/wishing to quit

Two trials reported biochemically validated continuous/sustained abstinence in participants receiving cytisine (1.5 mg tablets for 25 days with variability in daily dose) compared to placebo. While review authors state that behavioural support was kept to a minimum, counselling or support was provided to both groups in both trials. Compared to placebo, 64 more participants per 1000 (95% CI: 22 to 147 more; *n* = 2, 937 participants; *I*^2^ = 0%) on cytisine were abstinent at 6 or more months follow-up (rating down once for imprecision). The certainty of the evidence was rated as moderate (Additional file 11: Table 7; Figure 2a (Additional file 17)) [62].

Cytisine—adverse events in general/mixed population of smokers

Adverse events were largely similar between cytisine and placebo groups across studies (*n* = 3). At 4 weeks, there were similar rates of mild adverse events (nausea, restlessness, insomnia, irritability) between groups in abstinent smokers (23.4% versus 20%); longer term information was not reported. A total of 10 events (e.g. dyspepsia, nausea and headache) from 4 people in each group were reported in a second study. The third study reported higher rates of gastrointestinal disorders with cytisine (13.8% versus 8.1%, *p* = 0.02) (Additional file 11: Table 8; Figure 2a (Additional file 17)) [62]. We were unable to assess imprecision or provide an overall certainty rating because the sample size was not reported.

*Nicotine replacement therapy (NRT) versus placebo*

NRT—abstinence/cessation in relapsed smokers motivated to quit

Nicotine patch (decreasing dose over 12 weeks: 21 mg/24 h to 7 mg/24 h) or placebo was provided to participants along with minimal additional support in one trial. Included participants had relapsed after transdermal patch and behavioural counselling in an earlier phase of the trial but were motivated to make a second attempt. Absolute effects could not be calculated for this study. Compared to placebo, NRT patch increased continuous abstinence by 25% but the confidence interval included possibility of a potential larger increase or large decrease in cessation (RR 1.25, 95% CI: 0.34 to 4.60; *n* = 1, 629 participants) (rating down 0.5 for risk of bias and twice for imprecision). The certainty of the evidence was rated as very low. Review authors indicate that there was a greater relative effect in 28-day point prevalence abstinence with NRT (RR 2.49, 95% CI: 1.11 to 5.57) although quit rates were low irrespective of the definition of abstinence used (Additional file 11: Table 22; Figure 2a (Additional file 17)) [54].

NRT—abstinence/cessation in smokers not motivated/wishing to quit

Lindson-Hawley [56] included studies of smokers who have no immediate intention to quit all tobacco use. Type of NRT varied across trials; studies examined inhaler (*n* = 2), gum (*n* = 4) or offered participants a choice of NRT type (*n* = 2). Dosing and treatment duration varied across studies within each NRT type (Additional file 11: Table 30). Most studies provided behavioural co-intervention to each group and biochemically validated smoking abstinence. For smoking cessation, authors preferred point prevalence over sustained/continuous abstinence as participants were not expected to quit at start of intervention.

Compared to placebo, 44 more participants per 1000 taking NRT were abstinent at 12 to 24 months follow-up (95% CI: 22 to 73 more; *n* = 8, 3081 participants; *I*^2^ = 30%) (rating down once for risk of bias). The certainty of the evidence was rated as moderate. Subgroup differences by NRT type was not detected (test for subgroup differences: *p* = 0.42, *I*^2^ = 0.0%) (Additional file 13) [56].

NRT—reduction in cigarettes per day of >50% of baseline or cessation in smokers not motivated/wishing to quit

Again in the review of Lindson-Hawley [56], studies of smokers who have no immediate intention to quit all tobacco use were included. NRT type, dose and treatment duration varied across studies (Additional file 11: Table 30). For smoking reduction, review authors preferred sustained/continuous rates over point prevalence.

At longest follow-up (12+ months), 60 more participants per 1000 (95% CI: 35 to 91 more; *n* = 8, 3081 participants; *I*^2^ = 45%) (rating down once for risk of bias) on NRT reduced the number of cigarettes smoked per day by more than 50% of baseline or quit entirely as compared to placebo. The certainty of the evidence was rated as moderate. Subgroup analysis indicated that compared to placebo, more participants reduced smoking with gum and inhaler than with choice of NRT (test for subgroup differences: *p* = 0.01, *I*^2^ = 79%) (Additional file 13) [56].

NRT—abstinence/cessation in smokers with current depression

A single study reporting post hoc subgroup data for smokers with current depression was identified by van der Meer [58]. Participants received NRT gum (2 or 4 mg with recommendation of 9 to 15 pieces per day for 2 months followed by weaning) or placebo plus a behavioural co-intervention provided to all participants. Abstinence was biochemically verified and measured continuously. Compared to placebo, 94 more participants per 1000 (95% CI: 4 fewer to 369 more; *n* = 1, 196 participants) receiving NRT gum were abstinent at 12 months follow-up (rating down once for risk of bias and twice for imprecision) (Additional file 11: Table 60; Figure 2a (Additional file 17)) [58]. The certainty of the evidence was rated as very low.

NRT—abstinence/cessation in smokers with past depression

Three studies recruited general smokers but reported pre-stated or post hoc subgroup data for smokers with past depression. One study each examined NRT gum (2 mg for 8 weeks and tapering to week 11) and patch (21, 14 and 7 mg titrated down during 8 weeks after quit date). The remaining study examined patch (21, 14 and 7 mg titrated down during 8 weeks after quit date), lozenge (2 or 4 mg according to dependence for 12 weeks) and patch plus lozenge with each of the treatment arms entered separately in the meta-analysis. All studies provided behavioural co-intervention to both arms; one four arm trial also provided bupropion or placebo tablets. Abstinence was biochemically validated in all studies, but measures varied with most reporting point prevalence.

Compared to placebo, 42 more participants per 1000 (95% CI: 38 fewer to 150 more; *n* = 3, 432 participants; *I*^2^ = 0%) on NRT were abstinent at longest follow-up (6+ months) (rating down twice for imprecision) (Additional file 11: Table 61; Figure 2a (Additional file 17)) [58]. The certainty of the evidence was rated as low.

NRT—abstinence/cessation in smokers with schizophrenia or schizoaffective disorder

No studies were found [59].

NRT—reduction in smokers with schizophrenia or schizoaffective disorder

No studies were found [59].

NRT—weight gain in smokers motivated/wishing to quit

Across analyses, weight gain was assessed in biochemically validated abstinent smokers. Type of NRT varied across studies and included patch, gum, inhaler, sublingual tablet and intranasal spray. Within each type, there was variation across trials with respect to dose and treatment duration. Control groups received placebo in all but two trials; one provided group therapy while the control condition was described as ‘no gum’ in the second. Most trials provided behavioural co-intervention to both study groups.

Compared to placebo, participants on NRT had 0.69 kg fewer kg gained post-cessation (95% CI: 0.88 to 0.51 lower; *n* = 19, 2600 participants; *I*^2^ = 82%) (rating down once for risk of bias and twice for inconsistency) at the end of treatment. The certainty of the evidence was rated as very low. There was little to no change in weight gain at 6 months (−0.37 kg; 95% CI: 0.88 lower to 0.14 higher; *n* = 9; 771 participants, *I*^2^ = 0%) and at 12 months of follow-up (−0.42 kg; CI: 0.92 lower to 0.08 higher; *n* = 15, 1334 participants, *I*^2^ = 0%) (Additional file 11: Table 19; Figure 2a (Additional file 17)). The certainty of the evidence was rated as moderate for both (rating down once for risk of bias). Subgroup differences by NRT type were not detected for all timepoints of follow-up (test for subgroup differences: end of treatment *p* = 0.38, *I*^2^ = 5%; 6 months *p* = 0.89, *I*^2^ = 0%; 12 months *p* = 0.34, *I*^2^ = 12%) (Additional file 13) [60].

NRT—change in mental state in smokers with schizophrenia or schizoaffective disorder

Two trials reported a change in mental state following NRT use, with the treatment duration of 7 h (8 mg/day) in one RCT and 32 h (22mg/day) in the second trial. One trial reported no difference in psychiatric symptoms between NRT patch and placebo phases. In the second trial, no participant experienced a change in subjective experience or mental status (rating down once for risk of bias and once or twice for imprecision) (Additional file 11: Table 56) [59]. We were unable to assess imprecision or provide an overall certainty rating lack of clarity regarding because the sample size was unclear.

NRT—adverse events in smokers with schizophrenia or schizoaffective disorder

One cross-over trial reported that 60% of participants experienced an increase in abnormal involuntary movement when using NRT patch (22 mg/day for 32 h). Review authors report the increase as statistically significant when participants were smoking and using NRT patch; it is unclear whether this is based on *p*-values or confidence intervals (rating down twice for risk of bias and once or twice for imprecision) (Additional file 11: Table 56) [59]. The certainty of the evidence was rated as very low.

NRT—adverse events in smokers motivated/wishing to quit

The type and dosing of NRT varied across studies or was not reported. Some studies provided either high- or low-intensity behavioural support to both study arms.

Compared to placebo, 12 more participants per 1000 (95% CI: 5 to 21 more; *n* = 15, 11,074 participants; *I*^2^ = 10%) taking NRT experienced palpitations/chest pains (rating down once for risk of bias). The certainty of the evidence was rated as moderate.

By type of formulation, the most common adverse events were as follows:

- Nicotine gum: hiccups gastrointestinal disturbances, jaw pain and orodental problems
- Nicotine patch: mild skin sensitivity and local skin irritation in up to 54% of patch users
- Nicotine inhalator: throat irritation, coughing and oral burning
- Nicotine nasal spray: irritation and runny nose
- Nicotine oral spray: hiccoughs and throat irritation
- Nicotine sublingual tablets: hiccoughs, burning and smarting sensation in the mouth, sore throat, coughing, dry lips and mouth ulcers

Review authors reported that reactions to NRT were usually not severe enough to prompt discontinuation of treatment. Trials could not be pooled due to heterogeneity with respect to the nature, timing and duration of symptoms.

Authors considered attrition as an adverse event in the review. They reported that attrition rates in NRT groups were generally similar to or lower than in control groups among included studies (Additional file 11: Table 23) [54].

*Bupropion versus placebo*

Bupropion—abstinence/cessation in smokers not motivated/wishing to quit

There was overlap across two reviews which included the same trial of smokers who were interested in reducing smoking but not quitting and who had at least two failed quit attempts, one of which was with NRT (Howes 2020 [67], Lindson-Hawley 2016 [56]). In the trial, those who became willing to quit entered the cessation phase of the trial, which included weekly counselling for seven weeks and then 19 weeks of follow-up. Both study arms received behavioural co-interventions and abstinence was biochemically confirmed.

Compared to placebo, 14 more participants per 1000 (95% CI: 18 fewer to 75 more; *n* = 1, 594 participants) on bupropion (300 mg/day for 26 weeks) were abstinent at 6 months follow-up, although there is uncertainty. Reviews differed in their rating of the attrition bias domain for this evidence; one review rated the trial as being at high risk while the other rated it at low risk of bias. As such, our GRADE assessments differed across reviews for this same evidence; we downrated once due to risk of bias and twice for imprecision for one review and twice for risk of bias and twice for imprecision for the other (Additional file 11: Tables 25 and 31) [56, 67]. The certainty of the evidence was rated as very low for both reviews.

Bupropion—abstinence/cessation in smokers with current depression

Five studies reported pre-stated or post hoc subgroup data for smokers with current depression. Abstinence was biochemically validated in all trials with most reporting continuous/sustained rates. Dosing of bupropion treatment was 300 mg/day for 7 weeks to 6 months across trials. All studies provided both arms with behavioural co-intervention and one also provided NRT patch.

At longest follow-up (6 to 12 months), 41 more participants per 1000 (95% CI: 19 fewer to 142 more; *n* = 5, 410 participants, *I* ^2^= 29%) were abstinent compared to placebo. We rated down once for risk of bias to also reflect concerns with indirectness and twice for imprecision (Additional file 11: Table 58; Figure 2a (Additional file 17)). The certainty of the evidence was rated as very low.

Review authors performed subgroup analysis by sole use of bupropion or bupropion used in adjunct to NRT; subgroup differences were not detected (test for subgroup differences: *p* = 0.66, *I*^2^ = 0%) (Additional file 13) [58].

Bupropion—abstinence/cessation in smokers with past depression

Four studies recruited general smokers but reported post hoc subgroup data for smokers with past depression. Bupropion treatment was 300 mg/day for 7 to 12 weeks across trials. All studies provided behavioural co-intervention to both arms; one four arm trial also provided NRT or placebo patch. The outcome was biochemically validated in all trials and most reported point prevalence abstinence.

Compared to placebo, 128 more participants per 1000 (95% CI: 38 to 268 more; *n* = 4, 404 participants; *I*^2^ = 44%) on bupropion stopped smoking at longest follow-up of 6 to 12 months (rating down once for risk of bias to also reflect concerns with inconsistency and once for imprecision) (Additional file 11: Table 59; Figure 2a (Additional file 17)). The certainty of the evidence was rated as low. Review authors signal the need for caution in interpreting these results as data for participants with past depression were derived from post-hoc subgroup analyses in all studies. Review authors performed subgroup analysis by sole use of bupropion or bupropion used in adjunct to NRT; they report no strong evidence of a difference between subgroups (test for subgroup differences: *p* = 0.05, *I*^2^ = 73.3%) (Additional file 13) [58].

Bupropion—abstinence/cessation in smokers with schizophrenia or schizoaffective disorder

Most trials examined the effect of offering 300 mg/day for about 10 or 12 weeks; 1 trial offered 150 mg/day for 12 weeks. Behavioural co-interventions were provided to both study groups in all trials; co-interventions included specialized behavioural counselling which was received by about 70% of participants in each study arm, across trials. Participants in two trials also received NRT patch with or without NRT gum. Abstinence was biochemically validated in all trials with most reporting continuous/sustained rates. Participants in all trials were interested in quitting.

At 6-month follow-up, 66 more participants per 1000 (95% CI: 1 to 244 more; *n* = 5, 214 participants; *I*^2^ = 0%) on bupropion were abstinent (rating down once for risk of bias and twice for imprecision) (Additional file 11: Table 54; Figure 2a (Additional file 17)). The certainty of the evidence was rated as very low. Review authors performed subgroup analysis by sole use of bupropion or bupropion used in adjunct to NRT; subgroup differences were not detected (test for subgroup differences: *p* = 0.67, *I*^2^ = 0%) (Additional file 13). Review authors also report data at longer follow-up. In one trial, three additional participants were abstinent at 2 years; of the four total participants that quit, three received bupropion during the trial or during the follow-up period. One other study reported that of the five bupropion participants that were abstinent at 6 months, two relapsed at 12-month follow-up. Including this 12-month follow-up data would reduce the effect estimate and the confidence interval would suggest little to no difference between groups or fewer events with bupropion [59].

Bupropion—reduction in number of cigarettes per day in smokers not motivated/wishing to quit

Participants were interested in reducing smoking but not quitting and had at least two failed quit attempts, one of which was with NRT (*n* = 1; 594 participants). At 12-month follow-up, 1 more participant per 1000 (95% CI: 36 fewer to 63 more; *n* = 1, 594 participants) on bupropion (300 mg/day for 26 weeks) quit or reduced the number of cigarettes smoked per day by more than 50% (rating down once for risk of bias and twice for imprecision) (Additional file 11: Table 31; Figure 2a (Additional file 17)) [56]. The certainty of the evidence was rated as very low.

Based on data from the same trial, Howes [67] reported no significant difference between groups in reduction of cigarettes per day at 12-month follow-up. It is unclear whether this is based on confidence intervals or *p*-values; we interpret this to mean that the confidence interval around the best estimate of effect includes the possibility of little to no difference between groups and greater reduction in one group over another (rating down twice for risk of bias) (Additional file 11: Table 25) [67]. We were unable to assess imprecision and provide an overall certainty rating due to missing information (i.e. number of events or sample size).

Bupropion—reduction in cotinine in smokers not motivated/wishing to quit

Two reviews reported data from the same single trial of the effects of bupropion compared with placebo on concentrations of biomarkers, like nicotine and its metabolites (i.e. cotinine), tobacco-specific nitrosamines, carbon monoxide (CO) and tobacco alkaloids (i.e. anabasine) [68, 69] assessed as their exposure in fluids , i.e. plasma, saliva and urine to determine approaches bound to achieve smoking cessation. Among participants who were interested in reducing but not quitting smoking and with at least two failed quit attempts (*n* = 1, 327 participants), 26 fewer participants per 1000 (95% CI: 40 fewer to 27 more; *n* = 1, 327 participants) on bupropion (300 mg/day for 26 weeks) had reduction in cotinine levels greater than 50%. Reviews differed in their rating of attrition bias for the same evidence resulting in different GRADE ratings across reviews; we downrated once due to risk of bias and twice for imprecision for one review and twice due to risk of bias and twice for imprecision for the other (Additional file 11: Tables 25 and 31) [56, 67]. The certainty of the evidence was rated as very low for both reviews.

Lindson-Hawley [56] also report no statistically significant difference between groups in mean urinary cotinine decrease from baseline at 12-month follow-up (mean decrease: bupropion 82 ng/mL versus placebo 28 ng/mL, *p* = 0.25) (rating down once for risk of bias and once or twice for imprecision). We were unable to rate the certainty of the evidence due to sample size analysed not being reported, and so the imprecision domain could not be assessed.

Bupropion—reduction in number of cigarettes per day in smokers with schizophrenia or schizoaffective disorder

Participants in all trials were interested in quitting and specialized behavioural counselling was provided to both groups. In one trial, participants in both groups also received NRT patch and NRT gum.

At 6-month follow-up, those on bupropion (300 mg/day for about 12 weeks) reduced their smoking by 0.4 cigarettes per day compared to those on placebo*(95% CI: 5.72 lower to 6.53 higher; *n* = 2, 104 participants; *I*^2^ = 0%) (rating down once for risk of bias and once for imprecision) (Additional file 11: Table 54; Figure 2a (Additional file 17)) [59]. The certainty of the evidence was rated as low.

Bupropion—expired carbon monoxide levels in smokers with schizophrenia or schizoaffective disorder

Participants in all trials were interested in quitting and specialized behavioural counselling was provided to both groups. In one trial, participants in both groups also received NRT patch with or without NRT gum.

Compared to placebo, expired CO mean was 5.55 ppm lower with bupropion (150 or 300 mg/day for about 12 weeks) at 6 months follow-up (95% CI: 17.89 lower to 6.78 higher; *n* = 3, 123 participants, *I*^2^ = 83%) (rating down once for risk of bias, twice for inconsistency and once for imprecision) (Additional file 11: Table 54; Figure 2a (Additional file 17)) [59]. The certainty of the evidence was rated as very low.

Bupropion—weight gain in smokers motivated/wishing to quit

Across analyses, weight gain was assessed in biochemically validated abstinent smokers. All trials examined bupropion 300 mg/day for 7 to 12 weeks and provided behavioural co-intervention to both study arms. Control groups received placebo in all but one trial in which participants received advice.

Compared to placebo, bupropion reduced post-cessation weight gain by 1.12 kg at end of treatment (95% CI: 1.47 to 0.77 lower; *n* = 7, 869 participants; *I*^2^ = 0%) (rating down once for risk of bias). The certainty of the evidence was rated as moderate. There was a little to no change in weight gain at 6 months of follow-up (0.87 kg lower; 95% CI: 2.21 lower to 0.47 higher, *n* = 4, 218 participants, *I*^2^ = 0%) and at 12 months of follow-up (0.38 kg lower; 95% CI: 2 lower to 1.24 higher, *n* = 4, 252 participants, *I*^2^ = 0%) (Additional file 11: Table 18; Figure 2a (Additional file 17)) [60]. The certainty of evidence was rated as low at both the follow-ups (rating down once for risk of bias and once for imprecision at both the follow-ups).

Bupropion—change in emotional state (depressive symptoms) in general/mixed population of smokers

A single trial included by Howes [67] reported on depressive symptoms. During treatment, most participants in both arms experienced reduction in depressive symptoms and this was sustained at follow-up. A between-group difference was observed for highly dependent smokers with greater reduction in the bupropion arm (300 mg/day for 10 weeks), but the reduction was not sustained at follow-up (Additional file 11: Table 26; Figure 2a (Additional file 17)). Behavioural co-interventions were provided to both study groups. We were unable to rate the certainty of the evidence due to sample size analysed not being reported for imprecision domain.

Bupropion—change in mental state in smokers with schizophrenia or schizoaffective disorder

Change in mental state outcomes included positive (e.g. hallucinations, delusions), negative (e.g. anhedonia, avolition), depressive and psychiatric symptoms. Various outcome measurement tools were used including, but not limited to, the PANSS, HAM-D and Beck Depression Inventory (BDI) [70–72]. Participants were interested in quitting in most trials. Review authors analysed studies according to the primary study objective.

Among studies with a primary aim of cessation, bupropion dosing was 300 mg/day for about 10 to 12 weeks and specialized behavioural counselling was provided to both groups in all trials. In one trial, participants in both arms also received NRT patch and NRT gum as a co-intervention. The positive symptoms score was 0.24 lower in those with bupropion at the end of treatment (95% CI: 0.66 lower to 0.19 higher; *n* = 2, 85 participants, *I*^2^ = 0%) (rating down once for risk of bias and once for imprecision) compared to placebo. The negative symptoms score was 0.12 lower in those with bupropion at the end of treatment (95% CI: 0.46 lower to 0.22 higher; *n* = 3, 136 participants, *I*^2^ = 0%) (rating down once for risk of bias and twice for imprecision) compared to placebo. Lastly, the depressive symptoms score was 0.16 lower in those with bupropion at the end of treatment (95% CI: 0.5 lower to 0.18 higher; *n* = 3, 136 participants, *I*^2^ = 0%) (Additional file 11: Table 54; Figure 2a (Additional file 17)) compared to placebo. The certainty of the evidence was rated as low or very low. Review authors indicate that three additional trials provided corroborating evidence of no significant difference between groups in symptoms, but data were incompletely reported. One trial not included in the meta-analysis reported improvement in negative symptoms and greater stability of psychotic and depressive symptoms in the bupropion arm during quit attempt. Three additional studies reported no effect of smoking abstinence on symptoms [59].

Three studies had a primary aim of smoking reduction. Intervention participants received bupropion 300 mg/day for about 22 days to 14 weeks across two studies; dosing and treatment duration was unclear for one study. One trial each provided specialized behavioural counselling and non-contingent reinforcement as co-interventions to both study groups. As reported by review authors, one study reported no worsening of positive and negative symptoms in the bupropion arm, a second study reported no significant difference between groups regarding change in positive and negative symptoms, and the third study reported no increase in psychiatric symptoms in the bupropion arm (rating down twice for risk of bias and once or twice for imprecision) (Additional file 11: Table 54; Figure 2a (Additional file 17)) [59]. The certainty of the evidence was rated as very low.

Bupropion—adverse events in smokers motivated/wishing to quit

Among participants who were interested in reducing but not quitting smoking and with at least two failed quit attempts (*n* = 1, 594 participants), 17 more participants per 1000 (95% CI: 3 fewer to 91 more; *n* = 1, 594 participants) on bupropion (300 mg/day for 26 weeks) experienced a serious adverse event (rating down once for risk of bias and twice for imprecision). The certainty of the evidence was rated as very low. Review authors state that one of the events was potentially attributed to bupropion treatment (Additional file 11: Table 31; Figure 2a (Additional file 17)) [56].

Bupropion—adverse events in smokers with schizophrenia or schizoaffective disorder

In nearly all studies, participants were interested in quitting or reducing smoking and received either placebo or bupropion 300 mg/day for 22 days to 14 weeks, across studies. Behavioural co-interventions were provided to both study groups in most studies; in a few trials, all participants also received pharmacotherapy (NRT patch with or without NRT gum). Review authors analysed studies according to the primary study objective.

Seven studies with a primary aim of smoking cessation reported adverse events. In one study, a participant who took bupropion had a seizure; authors reported that this was likely unrelated to bupropion treatment. No seizures were reported in remaining trials.

One study reported that one participant receiving bupropion (3%) and two receiving placebo (7%) experienced a psychotic breakdown; authors did not consider this related to study treatment. Another study reported recurrence of psychotic symptoms in two participants, but results were not reported by study arm. One study reported no serious adverse events.

One trial reported higher rates of dry mouth in the bupropion arm compared to placebo and another study reported that significantly more bupropion participants (also receiving NRT) experienced poor concentration, jitteriness, light-headedness, muscle stiffness and frequent nocturnal awakening. A third study reported that, compared to placebo, the bupropion arm had higher rates of insomnia, dry mouth and sweatiness. A fourth study reported an allergic reaction in one participant receiving bupropion. A fifth study reported no significant differences in major adverse events measured by the Side Effect Checklist (e.g. restlessness, insomnia, dry mouth, sedation); however, five participants on bupropion withdrew from the trial due to side effects, including rash (*n* = 1), restlessness and increased anxiety (*n* = 2), worsening of psychosis (*n* = 1) and the aforementioned seizure. The sixth study reported two participant withdrawals in the bupropion arm (also receiving NRT) due to insomnia and dizziness (rating down once for risk of bias ) (Additional file 11: Table 54; Figure 2a (Additional file 17)) [59]. We were unable to rate the certainty of the evidence due to sample size analysed not being reported for imprecision domain.

Among studies with a primary aim of smoking reduction (*n* = 3), two reported no adverse events related to bupropion. The remaining study reported no significant difference between groups in adverse events and no seizures or suicidal behaviour in the bupropion arm (rating down once for risk of bias, once for indirectness and once or twice for imprecision) (Additional file 11: Table 54) [59]. The certainty of the evidence was rated as very low.

2. Behavioural interventions

*Stage-based expert systems or tailored self-help materials versus assessment only*

Abstinence/cessation in general/mixed population of smokers

Stage-based expert systems are personalized reports or letters, often produced electronically according to questionnaires or interviews, that are matched to a participant’s stage of change [73]. In one trial, a behavioural co-intervention was provided only to those receiving the intervention. In another, 25% of participants in the intervention arm and 21% in the control arm used NRT by 2-year follow-up. Most trials reported either prolonged or point prevalence abstinence; none biochemically validated smoking abstinence.

At longest follow-up (6+ months), 22 more people per 1000 (95% CI: 12 to 33 more; *n* = 10, 13,597 participants; *I*^2^ = 46%) given stage-based expert systems or tailored self-help materials compared with assessment only stopped smoking (rating down twice for risk of bias and once for inconsistency) (Additional file 11: Table 2; Figure 2b (Additional file 17)) [73]. The certainty of the evidence was rated as very low.

One trial examined the effect of a computer-generated tailored letter addressing outcomes of smoking and quitting, self-efficacy, active skills to quit, boosting confidence and coping skills; control participants received a letter confirming no self-help intervention would be sent. Review authors state that there was a significant difference between groups at 14 months with more continuous/sustained abstainers in the intervention arm (OR 3.74, 95% CI: NR) (rating down twice for risk of bias) (Additional file 11: Table 2; Figure 2b (Additional file 17)) [73]. The certainty of the evidence was rated as very low.

*Stage-based interactive computer programmes compared to usual care*

Abstinence/cessation in general/mixed population of smokers

Two cluster randomized trials, one in students 13 to 14 years of age and another in pregnant individuals, examined the effect of stage-based interactive computer programmes. One trial each reported point prevalence abstinence and continuous/sustained abstinence; neither used biochemical confirmation.

At longest follow-up (12+ months), 10 more people per 1000 (95% CI: 13 fewer to 41 more; *n* = 2, 1702 participants, *I*^2^ = 0%) given stage-based interactive computer programmes stopped smoking in comparison to usual care (rating down twice for risk of bias, once for indirectness and twice for imprecision) (Additional file 11: Table 3; Figure 2b (Additional file 17)) [73]. The certainty of the evidence was rated as very low.

*Stage-based telephone counselling compared to usual care*

Abstinence/cessation in general/mixed population of smokers

One trial of stage-based telephone counselling based on specialized approaches (5As, motivational interviewing, 5Rs) reported that at 12 months follow-up, 16 more people per 1000 (95% CI: 27 fewer to 114 more; *n* = 1, 318 participants) given stage-based telephone counselling compared to usual care were point prevalent abstinent (rating down once for risk of bias and twice for imprecision) (Additional file 11: Table 4; Figure 2b (Additional file 17)). The certainty of the evidence was rated as very low. The intervention group received additional co-interventions depending on readiness to quit [73].

*Stage-based individual counselling and/or advice compared to usual care*

Abstinence/cessation in general/mixed population of smokers

One trial provided both stage-based counselling and advice; other trials offered either counselling or advice tailored to stage of change. About half of the studies provided a behavioural co-intervention to those in the active arm with a few also recommending pharmacotherapy. Usual care varied across studies with some trials providing active smoking cessation interventions in conjunction with usual care. Trials varied with respect to how abstinence was measured. Biochemical confirmation of abstinence was conducted in less than half of trials.

Compared to usual care, 21 more people per 1000 (95% CI: 1 fewer to 46 more; *n* = 7, 3293 participants, *I*^2^ = 36%) receiving stage-based individual counselling and/or advice were abstinent at longest follow-up (6+ months) (rating down once for risk of bias) (Additional file 11: Table 5; Figure 2b (Additional file 17)) [73]. The certainty of the evidence was rated as moderate.

*Stage-based individual counselling or advice compared to assessment only*

Abstinence/cessation in general/mixed population of smokers

Compared to receiving assessment only (i.e. no intervention), 12 more people per 1000 (95% CI: 2 fewer to 31 more; *n* = 3, 3056 participants, *I*^2^ = 80%) receiving stage-based individual counselling or advice were abstinent at 6 or more months (rating down once for risk of bias and twice for inconsistency) (Additional file 11: Table 6; Figure 2b (Additional file 17)). The certainty of the evidence was rated as very low. Behavioural co-interventions were provided to intervention participants in all trials; no co-interventions were provided to the control groups. Measures of abstinence and use of biochemical validation varied across trials [73].

*Interventions to increase adherence to medications for tobacco dependence compared to usual or standard care*

Abstinence/cessation in smokers motivated/wishing to quit or reduce smoking

Four trials reported on the effects of interventions aimed to increase adherence to tobacco cessation medications. All trials provided some behavioural support to those receiving usual care. Relative to usual care, the intervention typically included an additional component focused on medication adherence with additional contact time. In most studies, the intervention involved specialized behavioural counselling (e.g. counselling based on motivational interviewing techniques and 4R approach). All participants in each of the trials were receiving NRT. Biochemical verification was used in most trials and most reported point prevalence rates.

Compared to usual care, 33 more people per 1000 (95% CI: 8 fewer to 81 more; *n* = 5, 3593 participants; *I*^2^ = 72%) receiving interventions to increase adherence to tobacco cessation medications were abstinent at 6-month follow-up (rating down twice for risk of bias and once for inconsistency) (Additional file 11: Table 24; Figure 2b (Additional file 17)) [66]. The certainty of the evidence was rated as very low.

Adverse events in smokers motivated/wishing to quit or reduce smoking

One study each reported no serious adverse events, or no treatment related adverse events. The third study reported no difference in adverse events between groups, but it is unclear whether this is based on confidence intervals or *p*-values (rating down once for risk of bias and once or twice for imprecision) (Additional file 11: Table 24; Figure 2b (Additional file 17)) [66]. We were unable to rate the certainty of the evidence due to sample size analysed not being reported for imprecision domain.

Change in emotional state—anxiety in smokers motivated/wishing to quit or reduce smoking

One trial reported no difference between groups regarding levels of anxiety at 1 week and 6 months, but it is unclear whether this is based on confidence intervals or *p*-values; (Additional file 11: Table 24; Figure 2b (Additional file 17)) [66]. We were unable to rate the certainty of the evidence due to sample size not being reported for imprecision domain.

*Individual counselling (no systematic pharmacotherapy) compared to minimal contact control (no systematic pharmacotherapy)*

Abstinence/cessation in general/mixed population of smokers

Twenty-seven studies examined the effect of individual counselling delivered by a smoking cessation specialist outside of routine clinical care. Additional behavioural and/or other (e.g. computer-guided nicotine fading with contingent contract, cigarette substitute) co-interventions were provided in the majority of studies. Review authors state that no systematic pharmacotherapy was provided; however, a few trials did offer NRT or a prescription for NRT to those receiving counselling. Minimal contact control was usual care or brief advice (up to 15 min) with or without self-help materials. Some trials provided additional behavioural or other (e.g. monetary rewards for cessation) co-interventions to control participants; NRT was made available in three trials. Trials varied with respect to how abstinence was measured and whether biochemical validation was conducted.

At follow-up of 6 or more months, 40 more people per 1000 (95% CI: 28 to 54 more; *n* = 27, 11,100 participants; *I*^2^ = 50%) receiving individual counselling compared to minimal contact were abstinent (rating down once for inconsistency and twice for indirectness) (Additional file 11: Table 29; Figure 2b (Additional file 17)). This was performed as a subgroup analysis by review authors; our assessment of subgroup credibility suggests that the subgroup analysis is plausible [74]. However, the certainty of the evidence was rated as very low.

*Non-tailored print-based self-help materials (no face-to-face contact) versus no materials/no intervention*

Abstinence/cessation in general/mixed population of smokers

All trials in this analysis sent non-tailored materials to participants without personal contact. Control conditions varied across trials and included no intervention, usual care, wait-list and a letter apologizing for shortage of smoking cessation kits. Trials varied with respect to how abstinence was measured, and it was unclear or not reported in almost half of the studies. Biochemical validation was used in nearly half of studies. Behavioural co-interventions were provided to intervention participants in one trial.

Compared to control, 10 more people per 1000 (95% CI: 2 to 19 more; *n* = 11, 13,241 participants; *I*^2^ = 0%) receiving non-tailored print-based self-help materials (with no face-to-face contact) were abstinent at 6+ month follow-up (Additional file 11: Table 36; Figure 2b (Additional file 17)). The certainty of the evidence was rated as high. This was performed as a subgroup analysis by review authors; our assessment of credibility suggests that the subgroup analysis is plausible. Review authors indicate that one trial not included in the meta-analysis due to inadequate reporting found no difference between groups [75].

Abstinence/cessation in smokers motivated to quit/wishing to quit

Two studies of treatment-seeking smokers examined the effect of non-tailored print-based materials without face-to-face contact. One trial each reported point prevalence and continuous/sustained abstinence. Only one of the trials biochemically validated abstinence. No co-interventions were provided.

At 6 months follow-up, 174 more people per 1000 (95% CI: 71 to 398 more; *n* = 2, 924 participants; *I*^2^ = 0%) receiving non-tailored print-based self-help materials (no face-to-face contact) were abstinent compared to those receiving no materials/no intervention (rating down twice for risk of bias and once for imprecision) (Additional file 11: Table 37; Figure 2b (Additional file 17)) [75]. The certainty of the evidence was rated as very low.

*Non-tailored print-based self-help materials (no face-to-face contact) compared to brief leaflet*

Abstinence/cessation in general/mixed population of smokers

All trials in this analysis sent non-tailored materials to participants without personal contact. Control participants received a brief leaflet which was considered a minimal print-based intervention by review authors. Additional behavioural co-interventions were provided to control participants in all studies. No additional co-interventions were given to active arms. Half of the studies reported continuous/sustained abstinence rates. Very few studies biochemically confirmed abstinence.

Compared with a brief leaflet control, 10 fewer people per 1000 (95% CI: from 23 fewer to 5 more; *n* = 6, 7023 participants, *I*^2^ = 21%) given non-tailored print-based self-help materials (no face-to-face) were abstinent at 6+ months follow-up (rating down once for risk of bias) (Additional file 11: Table 38; Figure 2b (Additional file 17)). This was performed as a subgroup analysis by review authors; our assessment of credibility suggests that the subgroup analysis is plausible [75]. The certainty of the evidence was rated as moderate.

*Non-tailored print-based self-help materials (with face-to-face contact) compared to no intervention or leaflet only*

Abstinence/cessation in general/mixed population of smokers

Materials were given to participants in-person, but investigators did not provide advice to smoking cessation. Most studies provided additional behavioural co-interventions to those in the active arm. No intervention was provided to control participants in most studies; in one study, participants received materials and a video not specific to smoking. Trials varied with respect to how abstinence was measured. Only one study used biochemical validation.

At longest follow-up (6+ months), 18 more people per 1000 (95% CI: 1 to 41 more; *n* = 4, 2822 participants; *I*^2^ = 21%) receiving non-tailored print-based self-help materials (with face-to-face contact) were abstinent compared to those receiving no intervention or leaflet only (rating down twice for risk of bias) (Additional file 11: Table 39; Figure 2b (Additional file 17)). The certainty of the evidence was rated as low. Subgroup differences by control condition (no intervention or leaflet only) was not detected (test for subgroup differences: *p* = 0.88, *I*^2^ = 0%) [75].

*Individually tailored print-based self-help materials (no face-to-face contact) compared to no materials/no intervention*

Abstinence/cessation in general/mixed population of smokers

Materials were tailored to the individual’s characteristics; several trials used computerized expert systems with tailoring according to baseline data. In all trials, materials were sent to participants without personal contact. Control conditions varied across studies and included assessment only, thank you letters only, and no intervention or information. No co-interventions were provided to either arm in all trials. Majority of studies reported continuous/sustained abstinence rates. No studies used biochemical validation.

Compared to those receiving no materials/no intervention, 20 more people per 1000 (95% CI: 11 to 31 more; *n* = 10, 14,359 participants; *I*^2^ = 0%) receiving individually tailored print-based self-help materials (no face-to-face contact) were abstinent at longest follow-up (6+ months) (rating down twice for risk of bias) (Additional file 11: Table 40; Figure 2b (Additional file 17)) [75]. The certainty of the evidence was rated as low.

*Intensive telephone counselling compared to minimal telephone counselling*

Abstinence/cessation in general/mixed population of smokers

Three trials compared intensive telephone counselling, defined as three to five calls, to minimal telephone counselling consisting of a single call. Two trials provided a behavioural co-intervention to both arms, and one of these trials also provided pharmacotherapy to all study participants including those receiving control. Most trials reported point prevalence rates. Only one trial biochemically validated smoking abstinence.

At 6 months or more follow-up, 64 more people per 1000 (95% CI: 28 to 104 more; *n* = 3, 2602 participants; *I*^2^ = 0%) receiving intensive telephone counselling were abstinent compared to those receiving minimal telephone counselling (rating down twice for risk of bias and half for each indirectness and imprecision) (Additional file 11: Table 42; Figure 2b (Additional file 17)) [76]. The certainty of the evidence was rated as very low.

*Brief motivational telephone counselling compared to usual care telephone call*

Abstinence/cessation in general/mixed population of smokers

One trial compared brief motivational telephone counselling (three 15-min calls) to a usual care telephone call (one 5-min call). No co-interventions were provided to either arm.

At 12 months follow-up, 60 more people per 1000 (95% CI: 4 to 190 more; *n* = 1, 374 participants) receiving brief motivational telephone counselling were point prevalence abstinent compared to those receiving usual care telephone call (rating down twice for risk of bias and twice for imprecision) (Additional file 11: Table 43; Figure 2b (Additional file 17)) [76]. The certainty of the evidence was rated as very low.

*Telephone counselling for smoking reduction compared to usual care telephone call*

Abstinence/cessation in general/mixed population of smokers

Compared to a usual care telephone call (1 5-min call), 49 more people per 1000 (95% CI: 1 fewer to 167 more; *n* = 1, 375 participants) receiving telephone counselling (3 15-min calls) for smoking reduction were point prevalence abstinent at 12-month follow-up (rating down twice for risk of bias and twice for imprecision) (Additional file 11: Table 44; Figure 2b (Additional file 17)) [76]. The certainty of the evidence was rated as very low.

*Interactive voice response (IVR) systems compared to no intervention*

Abstinence/cessation in general/mixed population of smokers

One trial compared IVR to no intervention. Prior to randomization, all participants received varenicline for 12 weeks and IVR. After randomization, the control group no longer received IVR. Review authors reported little to no difference between groups at 24 months follow-up (21.7% of intervention group versus 42.9% of control group; *p* = 0.13) (rating down once for risk of bias) (Additional file 11: Table 45; Figure 2b (Additional file 17)) [77]. We were unable to rate the certainty of the evidence due to sample size analysed not being reported for imprecision domain.

*Physician advice (minimal or intensive interventions) compared to no advice (or usual care)*

Abstinence/cessation in general/mixed population of smokers

Physician advice involved a ‘stop smoking’ message. Interventions were defined as minimal if delivered during a single session of less than 20-min duration plus up to 1 follow-up visit with or without a leaflet. Intensive interventions involved a longer initial consultation, use of additional materials other than a leaflet, or more than one follow-up visit. Pharmacotherapy co-intervention was provided to intervention participants in some of the studies. Additional behavioural and/or pharmacotherapy co-interventions were provided to control participants in some studies. Trials varied with respect to how abstinence was measured and only a few biochemically confirmed smoking abstinence.

At longest follow-up, 36 more people per 1000 (95% CI: 28 to 46 more; *n* = 26, 22,239 participants; *I*^2^ = 40%) receiving physician advice were abstinent in comparison to those receiving no advice (or usual care) (rating down twice for risk of bias) (Additional file 11: Table 46; Figure 2b (Additional file 17)). The certainty of the evidence was rated as low. Subgroup differences by advice intensity (minimal or intensive) was not detected (test for subgroup differences: *p* = 0.31, *I*^2^ = 3%) (Additional file 13). Review authors state that indirect comparisons across subgroups defined by number of advice sessions was suggestive of greater effects with multiple visits compared to a single visit. Authors indicate no important differences between subgroups according to use of aids [78].

*Physician advice with follow-up compared to minimal intervention/advice with single visit*

Abstinence/cessation in general/mixed population of smokers

Five studies compared physician advice with follow-up to a minimal intervention/advice with a single visit. Most studies reported continuous/sustained abstinence. Abstinence was biochemically confirmed in 60% of studies. One study provided a pharmacotherapy co-intervention to both arms.

Compared to control, 47 more people per 1000 (95% CI: 7 to 103 more; *n* = 5, 1254 participants; *I*^2^ = 0%) receiving physician advice with follow-up were abstinent at 6+ months (rating down twice for risk of bias and once for imprecision) (Additional file 11: Table 47; Figure 2b (Additional file 17)) [78]. The certainty of the evidence was rated as very low.

*Intensive advice compared to minimal advice*

Abstinence/cessation in general/mixed population of smokers

Intensive interventions involved a longer initial consultation, use of additional materials other than a leaflet or more than one follow-up visit. Trials varied with respect to how abstinence was measured; nearly half reported point prevalence rates and the reminder continuous/sustained rates. Biochemical validation was used in 60% of studies. One study provided a pharmacotherapy co-intervention to both arms.

Compared to minimal advice, 28 more people per 1000 (95% CI: 15 to 42 more; *n* = 15, 9775 participants; *I*^2^ = 32%) receiving intensive advice were abstinent at longest follow-up (6+ months) (rating down twice for risk of bias and once for indirectness) (Additional file 11: Table 48; Figure 2b (Additional file 17)). The certainty of the evidence was rated as very low. The effect estimate was larger for the subgroup of participants at high risk of smoking-related diseases as compared to unselected smokers, but confidence intervals overlapped (test for subgroup differences: *p* = 0.02, *I*^2^ = 82%) (Additional file 13) [78].

*Group behaviour therapy compared to no intervention*

Abstinence/cessation in general/mixed population of smokers

Group therapy was delivered over at least two sessions. Measures of abstinence were unclear or not reported for two-thirds of trials. Some studies provided both groups with co-interventions including pharmacotherapy; however, provision of co-interventions was unclear for most studies.

Compared to those receiving no intervention, 108 more people per 1000 (95% CI: 54 to 186 more; *n* = 9, 1098 participants; *I*^2^ = 55%) given group behaviour therapy were abstinent at 6+ months follow-up (rating down twice for risk of bias, once for inconsistency, once for imprecision) (Additional file 11: Table 51; Figure 2b (Additional file 17)) [79]. The certainty of the evidence was rated as very low.

*Interactive and tailored internet interventions compared to non-active controls*

Abstinence/cessation in general/mixed population of smokers

Interactive interventions ‘involved a two-way flow of information between the internet and the participant’ and programmes were tailored to a participant's characteristics [61]. Across studies, control conditions consisted of printed self-help guides or usual care. One study provided NRT as an adjunct to the internet intervention. Another study offered free nicotine patches and bupropion to all participants, including those receiving control, and their partners who wanted to quit. Trials varied with respect to how abstinence was measured and only 25% used biochemical validation.

At longest follow-up (6 to 12 months), 19 more people per 1000 (95% CI: 1 to 39 more; *n* = 8, 6786 participants; *I*^2^ = 58%) receiving interactive and tailored internet interventions were abstinent compared to non-active controls (rating down twice for risk of bias, once for indirectness and inconsistency) (Additional file 11: Table 52; Figure 2b (Additional file 17)) [61]. The certainty of the evidence was rated as very low.

*Mobile phone short message service compared to control*

Abstinence/cessation in smokers motivated/wishing to quit

One trial examined the effect of personalized text messaging with smoking cessation advice, support and distraction sent between a health care provide or buddy (i.e. lay health worker or peer support) and the participant. The control group received biweekly text messages thanking them for their involvement and reminder of a free month of text messaging should they complete the study. The trial used various measures of abstinence.

Compared to control, 17 more people per 1000 (95% CI: 21 fewer to 62 more; *n* = 1, 1705 participants) receiving the active text messaging intervention were point prevalent abstinent at 6-month follow-up (rating down once for risk of bias and twice for imprecision). The certainty of the evidence was rated as very low. However, when point prevalence using last outcome carried forward, 76 more people per 1000 receiving the active intervention were point prevalent abstinent in comparison to controls (95% CI: 30 to 131 more; *n* = 1, 1705 participants) (rating down once for risk of bias and once for imprecision). The certainty of the evidence was rated as low.

When allowing for three or fewer lapses of two or fewer cigarettes per lapse, 29 more people per 1000 (95% CI: 5 to 65 more; *n* = 1, 1705 participants) receiving the active text messaging intervention were continuously abstinent compared to controls (rating down once for risk of bias and once for imprecision). The certainty of the evidence was rated as low. These effects were not sustained when allowing for no lapses (Additional file 11: Table 63; Figure 2b (Additional file 17)) [65].

Adverse events in smokers motivated/wishing to quit

Compared to control, six fewer people per 1000 receiving the active text messaging intervention had been in a car crash during the 6-month follow-up period (rating down twice for imprecision). The certainty of the evidence was rated as low. However, it is possible that there is little to no difference between groups or that more participants receiving the intervention were in a car crash (Additional file 11: Table 63; Figure 2b (Additional file 17)) [65].

Compared to control, 5 more people per 1000 (95% CI: 15 fewer to 33 more; *n* = 1, 1705 participants) receiving the active text messaging intervention experienced pain in their thumb/finger joints during the 6-month follow-up (rating down twice for imprecision). The certainty of the evidence was rated as low (Additional file 11: Table 63; Figure 2b (Additional file 17)) [65].

*Mobile phone-based interventions compared to usual care*

Abstinence/cessation in smokers motivated/wishing to quit

Nearly all studies had text messaging (SMS) as the main component of the intervention; however, one trial differed in that participants received mobile phone-based counselling (cognitive behavioural therapy (CBT) and motivational). In-person visits or assessments were provided in addition to SMS in five studies. Control conditions varied across studies and included no intervention, text messages, written/internet untailored materials, untailored messages and standard cessation advice and treatment. Trials varied with respect to how abstinence was measured with slightly more reporting point prevalence rates. Abstinence was biochemically confirmed in half of the studies.

At longest follow-up (6+ months), 37 more people per 1000 (95% CI: 26 to 50 more; *n* = 12, 11,885 participants; *I*^2^ = 59%) receiving mobile phone-based interventions were abstinent in comparison to those receiving usual care (rating down once for risk of bias and once for inconsistency) (Additional file 11: Table 69; Figure 2b (Additional file 17)). The certainty of the evidence was rated as low. Sensitivity analyses according to abstinence definition, biochemical validation and intervention characteristics showed no appreciable change in the relative effect estimate (Additional file 13) [80].

3. Other therapy interventions

*Hypnotherapy compared to placebo drug alone*

Abstinence/cessation in smokers motivated/wishing to quit

Intervention details, including the number and duration of sessions, was not reported in the only included trial. At 12 months follow-up, 18 fewer people per 1000 (95% CI: 77 fewer to 166 more; *n* = 1, 114 participants) receiving hypnotherapy were point prevalent abstinent compared to those receiving the placebo drug (rating down twice for risk of bias and twice for imprecision) (Additional file 11: Table 1; Figure 2c (Additional file 17)) [53]. The certainty of the evidence was rated as very low.

*Acupuncture compared to sham acupuncture*

Abstinence/cessation in smokers motivated/wishing to quit

All trials selected acupuncture points (i.e. anatomic sites) for smoking cessation. Two studies used facial acupuncture, five used auricular acupuncture alone with/without continuous stimulation (i.e. needle or pressure device) and four used combined body and auricular acupuncture with/without continuous stimulation (i.e. indwelling needle or seed). Two trials, representing 4% of the evidence, used potentially active acupuncture points for the sham arm. Behavioural co-interventions were provided to both study arms in some trials and pharmacotherapy to all participants in one study. Trials varied with respect to how abstinence was measured. Few studies used biochemical validation.

At longest follow-up (6–12 months), 11 more people per 1000 (95% CI: 15 fewer to 43 more; *n* = 11, 1892 participants, *I*^2^ = 23%) who received acupuncture were abstinent compared to those receiving sham (rating down twice for risk of bias and twice for imprecision) (Additional file 11: Table 64; Figure 2c (Additional file 17)). The certainty of the evidence was rated as low. Sensitivity analysis excluding studies which used potentially active acupuncture points for the sham arm yielded similar results [55].

*Acupuncture compared to waiting list/no intervention*

Abstinence/cessation in smokers motivated/wishing to quit

All trials selected acupuncture points (i.e. anatomic sites) for smoking cessation. One study used facial acupuncture and two used auricular acupuncture alone with or without continuous stimulation (i.e. needle or pressure device). One study provided a behavioural co-intervention to both study arms, with no co-interventions in the remaining studies. No studies biochemically verified abstinence.

At 6 to 12 months follow-up, 60 more people per 1000 (95% CI: 2 fewer to 174 more; *n* = 2, 393 participants, *I*^2^ = 57%) reported being smoking abstinent compared to those in the waitlist/no intervention group. We rated down twice for risk of bias, once for inconsistency and twice for imprecision (Additional file 11: Table 65; Figure 2c (Additional file 17)) [55]. The certainty of the evidence was rated as very low.

*Continuous auricular stimulation compared to sham stimulation*

Abstinence/cessation in smokers motivated/wishing to quit

Four studies used indwelling needles and the remainder used continuous acupressure. One trial, representing 5% of the evidence, used potentially active acupuncture points for the sham arm. Two studies provided behavioural co-intervention to both groups. Half of the studies reported use of biochemical confirmation for smoking abstinence. The measure of smoking abstinence was unclear or not reported for half of the studies.

At 6 to 12 months follow-up, 26 more people per 1000 (95% CI: 12 fewer to 98 more; *n* = 6, 570 participants, *I*^2^ = 22%) given continuous auricular stimulation compared to sham stimulation stopped smoking (rating down twice for risk of bias to also reflect concerns with publication bias and twice for imprecision) (Additional file 11: Table 66; Figure 2c (Additional file 17)). The certainty of the evidence was rated as very low. Subgroup differences by type of stimulation (indwelling needles or continuous acupressure) was not detected (test for subgroup differences: *p* = 0.06, *I*^2^ = 72%) (Additional file 13) [55].

*Laser therapy compared to sham laser*

Abstinence/cessation in smokers motivated/wishing to quit

Only one of the two studies reported the dose of laser used which was 50 mW for 14 min. One study provided behavioural co-intervention to both arms. Abstinence measures were unclear or not reported for both studies.

Studies were heterogeneous and could not be quantitatively synthesized (*I*^2^ = 97%). Review authors report that heterogeneity was possibly attributable to populations recruited and dose of laser administered. Results from one study were null-inclusive (RD 3 more per 1000, 95% CI: 45 fewer to 94 more). The other study reported results favouring the intervention (RD 515 more per 1000, 95% CI: 192 to 1000 more) (rating down once for risk of bias, twice for inconsistency and twice for imprecision) (Additional file 11: Table 67; Figure 2c (Additional file 17)) [55]. However, the certainty of the evidence was rated as very low.

*Electrostimulation compared to sham electrostimulation*

Abstinence/cessation in smokers motivated/wishing to quit

Electrostimulation was administered through surface electrodes over the mastoid bone in one study and to the ear in the other. No co-interventions were provided to either arm in both studies. Abstinence measures were unclear or not reported for both studies. Biochemical validation was used in one study.

Compared to sham electrostimulation, 34 fewer people per 1000 (95% CI: 102 fewer to 60 more; *n* = 2, 405 participants, *I*^2^ = 46%) receiving electrostimulation were abstinent at longest follow-up (6 to 12 months) (rating down twice for risk of bias and twice for imprecision) (Additional file 11: Table 68; Figure 2c (Additional file 17)) [55]. The certainty of the evidence was rated as very low.

*Acupressure versus sham*

No studies were found [55].

*Laser therapy versus wait-list/no intervention*

No studies were found [55].

*Electrostimulation versus wait-list/no intervention*

No studies were found [55].

St John’s wort versus placebo

Abstinence/cessation in smokers motivated/wishing to quit

One trial examined 900 mg/day of St John’s wort for 14 weeks and the other examined both 900 and 1800 mg/day for 12 weeks; treatment arms in the latter study were combined in the analysis. Behavioural co-interventions were provided to all groups in both studies. The studies reported biochemically confirmed prolonged abstinence.

Compared to placebo, 10 fewer participants per 1000 (95% CI: 40 fewer to 83 more; *n* = 2, 261 participants, *I*^2^ = 29%) on St. John’s wort were abstinent at 6-month follow-up (rating down twice for imprecision) (Additional file 11: Table 27; Figure 2c (Additional file 17)) [67]. The certainty of the evidence was rated as low.

*S-Adenosyl-L-methionine (SAMe) versus placebo*

Abstinence/cessation in smokers motivated/wishing to quit

A single study reported on 800 and 1600 mg/day SAMe; doses were combined in analysis by review authors. All study groups received a behavioural co-intervention.

Compared to placebo, 38 fewer participants per 1000 (95% CI: 95 fewer to 134 more; *n* = 1, 120 participants) receiving SAMe were point prevalence abstinent at 6 months follow-up (rating down once for risk of bias and twice for imprecision) (Additional file 11: Table 28; Figure 2c (Additional file 17)) [67]. The certainty of the evidence was rated as very low.

*Exercise interventions versus no intervention*

No studies were found [60].

4. Combination interventions

*Behavioural support (advice) plus NRT plus phone calls versus no intervention*

Abstinence/cessation in smokers not motivated/wishing to quit

In one trial, participants received an initial advice intervention aimed at encouraging reduction. Participants were also advised to quit; those who agreed received the cessation intervention. Intervention participants were offered a choice of NRT gum or patch. Control participants received assessment calls only. Co-interventions were not provided to either group. Smoking cessation was not biochemically verified.

Review authors report that point prevalence abstinence rates were significantly higher in the intervention group as compared to control at 6 months follow-up (rating down twice for risk of bias and once or twice for imprecision) (Additional file 11: Table 34; Figure 2d (Additional file 17)) [56]. The certainty of the evidence was rated as very low.

Reduction in the number of cigarettes/day in smokers not motivated/wishing to quit

The same trial also reported on smoking reduction. Review authors indicate that the reduction rate was significantly higher in the intervention group compared to those in the control arm (rating down twice for risk of bias and once or twice for imprecision) (Additional file 11: Table 34; Figure 2d (Additional file 17)) [56]. The certainty of the evidence was rated as very low.

*Telephone counselling plus self-help materials versus usual care*

Abstinence/cessation in smokers not motivated/wishing to quit

Participants were instructed to reduce smoking by 50% or more; cessation was subsequently encouraged. Self-help materials were individually tailored newsletter and a targeted newsletter. Usual care consisted of usual care plus generic health mailings. Co-interventions were not provided to either group. Smoking abstinence was biochemically verified.

At 12-month follow-up, 22 more people per 1000 (95% CI: 18 fewer to 124 more; *n* = 1, 320 participants) receiving telephone counselling plus self-help were point prevalence abstinent as compared to those receiving usual care (rating down twice for imprecision) (Additional file 11: Table 33; Figure 2d (Additional file 17)) [56]. The certainty of the evidence was rated as low.

Reduction in cigarettes/day of >50% of baseline or cessation

In the same trial, 63 more people per 1000 (95% CI: 22 fewer to 195 more; *n* = 1, 320 participants) receiving telephone counselling plus self-help were abstinent or reduced the number of cigarettes smoked by more than 50% at 12 months compared to usual care (rating down once for risk of bias and twice for imprecision) (Additional file 11: Table 33; Figure 2d (Additional file 17)) [56]. The certainty of the evidence was rated as very low.

Reduction in carbon monoxide >50%

At 12 months, 1 fewer person per 1000 (95% CI: 59 fewer to 100 more; *n* = 1, 320 participants) receiving telephone counselling plus self-help reduced their carbon monoxide levels by more than 50% as compared to those receiving usual care (rating down twice for imprecision) (Additional file 11: Table 33; Figure 2d (Additional file 17)) [56]. The certainty of the evidence was rated as low.

Reduction in number of cigarettes per day from baseline

Review authors state that the number of cigarettes per day decreased from baseline in both groups (mean change from baseline (SD): intervention: 21.2 (9.4) and usual care: 20.1 (8.9). There was no difference between groups at 12 months follow-up (mean (SD): intervention, 15.8 (10. 3) and usual care, 15.3 (9.2) (rating down once for risk of bias) (Additional file 11: Table 33; Figure 2d (Additional file 17)) [56]. Unable to assess the certainty of the evidence and imprecision domain based on information reported.

Reduction in carbon monoxide from baseline

Carbon monoxide levels decreased from baseline in both groups. Review authors state that there was no significant between-group difference in the change from baseline but it is unclear whether this is based on confidence intervals or *p*-values; we interpret this to mean that the confidence interval around the best estimate of effect includes the possibility of little to no difference between groups and greater reduction in one group over another (Additional file 11: Table 33; Figure 2d (Additional file 17)) [56]. Unable to assess the certainty of the evidence and imprecision domain based on information reported.

*Hotline and self-help materials versus minimal intervention*

Abstinence/cessation in general/mixed population of smokers

Two trials examined the effects of telephone hotline and self-help materials. One study promoted a 24-h hotline, daytime access to counsellors and use of the American Lung Association self-help manual. The second trial consisted of a Quitline proactive contract, quit kits (national Quitline printed resources), individual counselling with a practice nurse (face-to-face) and three proactive telephone calls from counsellors. Control participants received a minimal intervention; this was usual care delivered by a primary care provider (i.e. advice, referral to Quitline, or both) in one study and a self-help manual in the second (American Lung Association). One study offered pharmacotherapy co-intervention (NRT patch) to both arms. Both trials reported continuous/sustained abstinence rates. Only one used biochemical validation.

At longest follow-up (12 to 18 months), 21 more people per 1000 (95% CI: 5 more to 42 more; *n* = 2, 3327 participants; *I*^2^ = 0%) receiving hotline and self-help materials were abstinent compared to those receiving minimal intervention (rating down by 1.5 for risk of bias and 0.5 for imprecision) (Additional file 11: Table 41; Figure 2d (Additional file 17)) [76]. The certainty of the evidence was rated as low.

*Internet intervention plus behavioural support versus non-internet-based non-active control*

Abstinence/cessation in general/mixed population of smokers

Participants in five trials received an internet intervention plus behavioural support which was provided by nurses, peer coaches or tobacco treatment specialists. Control participants received a non-active control condition which varied across trials (usual care, printed self-help guides, standard smoking cessation advice). In two trials, all participants (including controls) were using or offered pharmacotherapy. Trials varied with respect to how smoking abstinence was measured, most reported prolonged rates. Biochemical validation was used in 40% of studies.

Compared to non-active controls, 54 more people per 1000 (95% CI: 23 more to 92 more; *n* = 5, 2334 participants; *I*^2^ = 60%) receiving an internet intervention plus behavioural support were abstinent at longest follow-up of 6 to 12 months (rating down one to two times for risk of bias, once for inconsistency and 0.5 for imprecision) (Additional file 11: Table 53; Figure 2d (Additional file 17)) [61]. The certainty of the evidence was rated as very low.

*Individual smoking cessation intervention based on cognitive behavioural therapy and motivational interviewing plus NRT patch compared to routine care*

Abstinence/cessation in smokers with schizophrenia or schizoaffective disorder

A single trial reported on the effects of a combination intervention which involved 8 h of individual contact for 8 weeks and NRT patch for about 10 weeks (21 mg, 14 mg, 7 mg titrated down). Participants in both study groups received booklets on smoking cessation as a co-intervention. Review authors report no statistically significant difference between groups in biochemically validated point prevalence or continuous abstinence rates at 6 months, 12 months, or 4 years (threshold for statistical significance was *p* < 0.01) (rating down once for risk of bias) (Additional file 11: Table 57) [59]. We were unable to assess imprecision and provide an overall certainty rating due to missing information.

Reduction in cigarettes per day of >50% of baseline in smokers with schizophrenia or schizoaffective disorder

Review authors report no statistically significant difference between groups in smoking reduction at 6 months, 12 months, or 4 years (rating down once for risk of bias) (Additional file 11: Table 57) [59]. We were unable to assess imprecision and provide an overall certainty rating due to missing information.

*Standard treatment plus extended NRT and extended CBT compared to standard treatment in smokers with past depression*

Abstinence/cessation in smokers with past depression

The only trial in this analysis recruited general smokers but provided pre-stated subgroup data for participants with past depression. Standard treatment consisted of sustained release bupropion (300 mg/day) for 12 weeks, nicotine gum (2 mg and 4 mg) for 10 weeks, 5 group counselling sessions from a counsellor and a self-help manual; no further treatment was provided after week 12. In addition to standard treatment, those in the active arm received extended NRT (i.e. until week 52) and extended CBT (i.e. 11 individual CBT sessions from week 10 to 52).

Compared to standard treatment, 259 more people per 1000 (95% CI: 6 fewer to 786 more; *n* = 1, 57 participants) receiving standard treatment plus extended NRT and extended CBT were point prevalence abstinent (follow-up timepoint unclear) (rating down once for risk of bias and twice for imprecision) (Additional file 11: Table 62; Figure 2d (Additional file 17)) [58]. The certainty of the evidence was rated as very low.

*Combined pharmacotherapy and behavioural interventions compared to usual care or minimal intervention*

Abstinence/cessation in general/mixed population of smokers

The typical intervention involved multiple contacts with a specialist cessation counsellor combined with pharmacotherapy. Review authors reported that most of the trials offered one or more types of NRT or bupropion. Usual care or minimal intervention was typically brief advice and self-help materials. Additional co-intervention (behavioural with or without pharmacotherapy) was provided to intervention participants in a few trials. Some trials provided behavioural and/or pharmacotherapy co-interventions to control participants. Trials varied with respect to how abstinence was measured but most used point prevalence. Biochemical confirmation of abstinence was used in 65% of the studies.

At longest follow-up (6+ months), 71 more people per 1000 (95% CI: 58 more to 84 more; *n* = 52, 19,488 participants; *I*^2^ = 36%) receiving combination pharmacotherapy and behavioural support were abstinent in comparison to those receiving usual care or minimal intervention (rating down twice for risk of bias to also reflect concerns for indirectness) (Additional file 11: Table 49; Figure 2d (Additional file 17)). The certainty of the evidence was rated as low. Review authors reported a larger effect in the subgroup of trials recruiting participants from health care settings (Additional file 13). Other variables tested in subgroup and meta-regression analyses did not modify the effect; this included participants’ motivation to quit and intensity of behavioural support [81].

*Combined pharmacotherapy and intensive behavioural interventions compared to usual care or no intervention*

Abstinence/cessation in general/mixed population of smokers

Review authors excluded one study with a more intensive intervention from the main analysis presented above [81]. The study intervention consisted of 12 group sessions over 10 weeks, with advice from physician on risk for COPD, and 2 mg of nicotine gum for 6 months. Intervention participants were also randomized to bronchodilator or placebo. The control group received no intervention or usual care. No co-interventions were provided to either arm. The study reported biochemically confirmed point prevalence rates.

Compared to usual care or no intervention, 260 more people per 1000 (95% CI: 212 more to 315 more; *n* = 1, 5887 participants) receiving the combination intervention were abstinent at 12-month follow-up (rating down once for indirectness) (Additional file 11: Table 50) [81]. The certainty of the evidence was rated as moderate.

5. Electronic cigarette intervention

*E-cigarette versus placebo*

Although the stage II of this evidence review aims at synthesizing evidence on the harms and benefits of e-cigs, we have included results from the Lindson-Hawley systematic review [56] in this overview of reviews for completeness. In the single trial identified by Lindson-Hawley [56], participants in the active treatment group received e-cigarettes containing either 7.2 or 5.4 mg of nicotine for 12 weeks to assist smoking reduction; active arms were combined in analyses. Control participants received e-cigarettes without nicotine. Neither group received co-interventions.

Abstinence/cessation in smokers not motivated/wishing to quit

The trial reported biochemically validated abstinence defined as ‘not even a puff’ since the previous visit. At 12 months follow-up, 70 more people per 1000 (95% CI: 1 fewer to 270 more; *n* = 1, 300 participants) using e-cigarette with nicotine were abstinent in comparison to those receiving e-cigarettes without nicotine, but it is possible that there is little to no difference between groups or that more participants in the active treatment arm stopped smoking (rating down twice for imprecision) (Additional file 11: Table 35; Figure 2e (Additional file 17)) [56]. The certainty of the evidence was rated as low.

Reduction in cigarettes/day of >50% of baseline or cessation in smokers not motivated/wishing to quit

At 12 months follow-up, 45 more people per 1000 (95% CI: 38 fewer to 187 more; *n* = 1, 300 participants) using e-cigarette with nicotine reduced the number of cigarettes per day by more than 50% (including those who quit) (rating down once for risk of bias and twice for imprecision) (Additional file 11: Table 35; Figure 2e (Additional file 17)) [56]. The certainty of the evidence was rated as very low.

Reduction in number cigarettes/day

Review authors indicate that there was no statistically significant difference between groups (median cigarettes per day = 12–14 in all groups), but it is unclear whether this is based on confidence intervals or *p*-values; we interpret this to mean that the confidence interval around the best estimate of effect includes the possibility of little to no difference between groups and a greater reduction in one group over another (rating down once for risk of bias) (Additional file 11: Table 35; Figure 2e (Additional file 17)) [56]. Unable to assess the certainty of the evidence because number of participants included in analysis not reported for imprecision domain.

Reduction in carbon monoxide

Authors indicate that there was no significant difference between groups (median CO = 15–17 ppm in all groups), but it is unclear whether this is based on confidence intervals or *p*-values; we interpret this to mean that the confidence interval around the best estimate of effect includes the possibility of little to no difference between groups and greater reduction in one group over another (Additional file 11: Table 35; Figure 2e (Additional file 17)) [56]. Unable to assess the certainty of the evidence because number of participants included in analysis not reported for imprecision domain.

Adverse events in smokers not motivated/wishing to quit

Review authors indicate that the frequency of adverse events was similar across groups at baseline, 12 weeks and 52 weeks. There was a reduction in reported symptoms from baseline to 12-month follow-up across groups (*p* < 0.001). Rates of shortness of breath were reduced from 20 to 4% from baseline to 2 weeks (Additional file 11: Table 35; Figure 2e (Additional file 17)) [56]. We were unable to assess imprecision and provide an overall certainty rating due to missing information (i.e. number of participants included in the analysis). Authors stated that no serious adverse events occurred [56].

Weight gain in smokers not motivated/wishing to quit

Review authors indicate that there was no significant difference in weight change within or between groups. Regarding the latter, it is unclear whether this is based on confidence intervals or *p*-values; we interpret this to mean that the confidence interval around the best estimate of effect includes the possibility of little to no difference between groups and more weight gain in one group over another (Additional file 11: Table 35; Figure 2e (Additional file 17)) [56]. Unable to assess the certainty of the evidence because number of participants included in analysis not reported for imprecision domain. Based on reporting, it is uncertain but unlikely that the outcome is post-cessation weight gain (i.e. does not appear to be assessed in only abstinent smokers).

## Discussion

Several stop-smoking interventions were identified that demonstrated an effect on sustained abstinence from tobacco smoking. Varenicline increased the chances of successful smoking cessation between two- and threefold compared with placebo in the general population of smokers and smokers with depression. The effect sizes included a large benefit for sustained abstinence for 6 or more months. There was a notable benefit for using varenicline in smokers with schizophrenia, bipolar disorder or other psychiatric disorders, given the high certainty of evidence based on four RCTs. Similarly, there is likely a benefit of cytisine over placebo in smokers motivated to quit; however, the estimates for benefit ranged from small but important to a large benefit with relatively low quit rates (9% in those receiving cytisine and 2% for placebo) at 1 year follow-up. NRT also demonstrated a twofold moderate benefit in smokers not motivated to quit, with 44 more per 1000 individuals more likely to stop smoking after following up for 1 to 2 years. Despite an observed benefit in smokers motivated to quit, the evidence in smokers with depression or past depression was less clear. The effect estimates for NRT gum versus placebo in smokers with depression encompassed little to no difference to a large benefit and was based on low certainty evidence, while the effect estimates for smokers with past depression encompassed both harm and benefit and was also based on low evidence. Lastly, evidence on bupropion suggested that it may increase smoking cessation among smokers with depression; however, we are very uncertain about the evidence as the effect estimates encompassed both benefits and harms. Although benefits were observed for smoking cessation with bupropion, varenicline and NRT, it is important to note little to no difference on long-term post-cessation weight gain. We also observed small harms like increased palpitations/chest pain with NRT, increased adverse events with varenicline (i.e. nausea, insomnia, abnormal dreams, headache) and mild harms (i.e. nausea, insomnia, irritability, headache, etc.) with cytisine compared to placebo. Lastly, bupropion showed little to no harm between groups due to insufficient information and low evidence certainty. The results from our review aligned with a 2023 systematic review and network meta-analysis (NMA) where high certainty evidence surrounding varenicline and cytisine as pharmacotherapy treatments showed higher smoking cessation rates compared to no pharmacotherapy. The NMA results also showed high-certainty evidence that nicotine patches, fast-acting nicotine and bupropion were more effective than control. For harm, low certainty evidence showed no difference between comparator groups [82]. The results also aligned with the review; an evidence update for the U.S. Preventive Services Task Force (USPSTF) having a very similar methodology showed strong evidence that a range of pharmacological and behavioural interventions offered individually or in combination can effectively increase smoking cessation in adults [83].

Other interventions that also showed a benefit on smoking cessation include physician advice, non-tailored print-based self-help materials, stage-based individual counselling, stage-based expert systems, individual counselling and group therapy. There was high certainty in the evidence for non-tailored print-based self-help materials (not face-to-face) compared to no materials in the general population of smokers suggesting a small but important benefit in smoking cessation; however, the effect was unclear in smokers motivated/wishing to quit. The effect of physician advice suggested moderate benefit; however, the certainty of the evidence was low due to very serious risk of bias. The effect of stage-based individual counselling and/or advice versus usual care in the general population of smokers had little to no difference on smoking cessation with moderate certainty of evidence. Lastly, stage-based expert systems, individual counselling versus minimal contact control and group therapy all showed a small but important benefit in smoking cessation; however, the certainty of evidence was very low.

The effects of some interventions on smoking cessation were unclear which included interventions to increase adherence to medications, telephone counselling plus self-help materials, interactive and tailored internet interventions, mobile phone-based interventions including text messaging, hypnotherapy, acupuncture, continuous auricular stimulation, laser therapy, electrostimulation, acupressure, St John’s wort, SAMe, electronic cigarettes, interactive voice response systems, standard treatment plus extended NRT plus extended CBT, individual counselling plus self-help and other combinations of interventions (Figure 2e). Unclear effects of interventions encompassed both a benefit and a harm and were usually based on low or very low certainty of evidence. For electronic cigarettes, the NMA results differed from our review results as there was a high certainty of evidence showing higher smoking abstinence rates in the nicotine e-cigs group than in the control group [82].

There are some important limitations to consider in our overview. For feasibility, we limited our study inclusion to Cochrane systematic reviews. Doing so may have resulted in a loss of outcome data as a more up-to-date non-Cochrane review with overlapping scope may have been available [33]. We also relied solely on information reported in the reviews (e.g. outcome measures, risk of bias assessments) and did not consult primary studies. This may have resulted in conflicting information reported within the text of reviews and the review evidence tables risk the possibility of propagating errors by carrying-over those extractions (i.e. two systematic reviews that had reported different GRADE ratings while assessing the same evidence from a single trial [56, 67]). Additionally, reporting across reviews varied. For example, assessment of co-interventions was a function of review authors’ reporting and presentation of the information. The reporting of usual care also varied and often included smoking cessation interventions; this could attenuate the effect of the intervention. Whether authors across reviews would have assessed and presented the same information in the same manner is unknown. There were also differences among reviews in whether or how they used GRADE methods. For example, variation in whether biochemical validation was considered in the assessment. Many reviews considered both performance and detection bias together, which would be reflective of early Cochrane standards. Determining indirectness was an arduous task as individual study information had to be extracted and collated to determine the weighted aggregate. This is an important aspect of feasibility that others should consider when considering an overview for clinical practice guidelines. Another limitation to note is that our exclusion of data combining placebo and non-placebo controls may have led to a few potentially useful analyses being excluded. For example, we did not include the main analyses from the reviews focusing on bupropion and NRT, which are the main analyses used in the USPSTF tobacco cessation guideline, due to the comparator being labelled as placebo and ‘other’ controls. One last limitation to consider is related to the timeframe of the searches of the included reviews and the updates. There has been a search update for three of the included Cochrane reviews (i.e. Cahill 2016 [84], Farley 2012 [85], and Howes 2020 [86]) with the inclusion of new studies; however, the main conclusions remained unchanged for two of the reviews (i.e. Farley 2012 and Howes 2020). For Cahill, conclusions were updated in terms of high evidence certainty surrounding varenicline and moderate evidence certainty surrounding cytisine helping more people quit smoking in comparison to placebo at 6 months plus follow-up. For the results on SAEs, there was no difference in the number of individuals reporting SAEs in the cytisine groups compared to placebo; however, there was moderate evidence certainty that those in the varenicline group are more likely to report SAE than those not taking it [84].

Overall, the evidence included in this overview of systematic reviews was based on systematic reviews rated low or very low AMSTAR 2 quality. AMSTAR 2 was used to measure quality, but low ratings may be reflective of poor reporting. Most of the reviews were rated as critically low. For critical domains like the risk of bias, there may be some studies where they were unassessed; however, we considered the authors’ statements within the text of their review to help fill gaps. For some domains, we determined as ‘unclear risk of bias’ by default when information was missing. For the 38% of reviews that did not consider the risk of bias in the interpretation of results, this would not strongly influence the understanding of the evidence here as we undertook GRADE assessments (that include an assessment of the risk of bias for the body of evidence of an analysis) as part of our process.

## Conclusions

This overview of reviews provides a comprehensive synthesis of the current evidence on the interventions helping adults aged 18 years and older to quit smoking. Results of this review, which included low, moderate and high-quality evidence, suggest that pharmacological (i.e. varenicline, NRT, cytisine, bupropion) and behavioural interventions (i.e. physician advice, non-tailored print-based self-help materials, stage-based individual counselling, stage-based expert systems, individual counselling and group therapy) can help the general smoking population quit smoking, however, with some small or mild harms to consider following NRT or varenicline use and will need to be assessed in the context of continued smoking. It is also important to note that the evidence examined does not provide clarity regarding ideal intervention strategies, nor the long-term impact of these interventions for preventing smoking. We also caution readers to avoid indirect comparisons across the analyses reported within this document, whether across categories or within a category where differences in dose and duration of treatment may be reported.

### Supplementary Information

Additional file 1. Various smoking cessation interventions.

Additional file 2. Preferred Reporting Items for Overview of Reviews (PRIOR) checklist.

Additional file 3. PRESS –peer review assessment of search strategy.

Additional file 4. Database search strategy.

Additional file 5. Grey literature sources.

Additional file 6. Eligibility criteria (PICO criteria).

Additional file 7. Reasons for exclusion at full-text and post-hoc exclusions.

Additional file 8. Overview of reviews data extraction items.

Additional file 9. Forest plots for included analyses.

Additional file 10. Risk of bias figures for included analyses.

Additional file 11. Grading of Recommendations, Assessment, Development and Evaluation (GRADE) Evidence Profile and Summary of Findings (SoF) tables.

Additional file 12. AMSTAR 2 instrument.

Additional file 13. Subgroup data for included analyses.

Additional file 14. Tobacco effect judgements.

Additional file 15. Review characteristics.

Additional file 16. AMSTAR 2 rating of included reviews.

Additional file 17. Included analyses and results.

Additional file 18. Stakeholders’ Feedback.

## Data Availability

All data generated or analysed during this study are included in this published article (and its supplementary information files).

## Abbreviations

AMSTAR: A MeaSurement Tool to Assess Systematic Reviews
CBT: Cognitive behavioural therapy
CI: Confidence interval
CO: Carbon monoxide
eCO/exCO: Exhaled carbon monoxide
GRADE: Grading of Recommendations, Assessment, Development and Evaluation
HAM-D: Hamilton Depression Rating Scale
IVR: Interactive voice response
MD: Mean difference
NRT: Nicotine replacement therapy
OR: Odds ratio
PANSS: Positive and Negative Syndrome Scale
PRISMA: Preferred Reporting Items for Systematic Reviews and Meta-Analyses
RCT: Randomized controlled trial
RD: Risk difference
RR: Risk ratio
SAE: Serious adverse event
SAMe: S-Adenosyl-L-methionine
SD: Standard deviation
SMD: Standardized mean difference
SMS: Short messaging service
