# Supplementary material for: Effectiveness of smoking cessation interventions among adults: an overview of systematic reviews
Source: Syst Rev. 2024 Jul 12;13:179. doi: 10.1186/s13643-024-02570-9 (PMC11242003; doi:10.1186/s13643-024-02570-9)
Supplement: Supplementary file 1 — Additional file 1. Various smoking cessation interventions. [file 13643_2024_2570_MOESM1_ESM.docx]

### **Additional file 1. Various Smoking cessation interventions**

#### Approved pharmacotherapies

Varenicline, cytisine, nicotine replacement therapy (NRT) and bupropion are all approved pharmacotherapies for use in Canada [1, 2]. Bupropion (Zyban) and varenicline (Champix) are available by prescription only, while NRT and cytisine can be purchased over the counter [3]. NRTs, acting temporarily by replacing nicotine to reduce cravings, can be administered as a patch, gum, inhaler, lozenge, or oral spray [1]. Cytisine is a plant-derived alkaloid that is regulated as a licensed natural health product in Canada [2]. It is similar in structure to nicotine and inexpensively produced [4, 5].

Bupropion was originally approved as an anti-depressant but has since also been approved as a cessation aid. It acts as a weak inhibitor for the neuronal uptake of the following neurotransmitters: norepinephrine, serotonin, and dopamine [1]. Varenicline, a nicotine receptor partial agonist and antagonist, stimulates dopamine, and reduces the ability of nicotine to attach to receptors, leading to a reduction in withdrawal symptoms [1, 3]. Bupropion can be used in combination with NRT, but information on the safety and efficacy of other combinations of pharmacotherapies is limited [3].

#### Behavioural therapies

Behavioural therapies for smoking cessation consist of a heterogeneous group of interventions. They can act directly by promoting cessation (i.e., encouraging adherence to smoking cessation pharmacotherapies) or other behaviours alongside smoking cessation (e.g., healthy eating, increase in exercise). Behavioural therapies can be delivered to groups or individuals, and are generally characterized by intensity, frequency of contact, method of contact, type of provider, and content.

The active components driving the change in behavioural smoking cessation interventions, referred to as behavior change techniques, have been identified and organized into taxonomies. They include addressing motivation (through incentives for abstinence), promoting self-regulatory skills (identifying barriers and problem solving), promoting other behaviours simultaneously (i.e., adherence to pharmacotherapies), and supporting other behavior change techniques (e.g., building rapport) [6].

Some of the most common behavioural therapies for smoking cessation include stage-based interventions, brief advice, individual or group counselling, face-to-face counselling, telephone counselling, and self-help. Stage-based interventions largely use the Transtheoretical Model of Change (also referred to as the ‘Stages of Change’ model), but this model may also be incorporated into other behavioural therapies [7]. The Transtheoretical Model of Change involves a sequence of motivational stages: precontemplation (not considering quitting), contemplation (considering quitting), preparation (planning to quit), action (quitting), and maintenance (staying tobacco free) [7, 8]. This model is based around the idea that interventions should be tailored to the current stage that an individual is in and help them move to the next stage. However, systematic reviews have not supported the effectiveness of stage-based intervention for smoking cessations due to the following reasons: varied methodological quality of the included studies, lack of reporting of validation of the instruments used in assessing participants' stage of change, little consistency in the types of interventions employed, limited description of the intervention, limited evidence on other types of staged interventions, including interactive computer programs and training of physicians or lay supporters for smoking cessation [7, 8]. Brief advice is usually delivered by a health care professional who provides a “stop smoking message”, usually a few minutes in duration [9]. Individual or group counselling is provided and led by facilitators (often physicians, nurses, or counsellors) and may consist of one or more sessions [10, 11]. In group counselling, an opportunity is provided for smokers and their fellow peers to share experiences and promote encouragement [10]. Face-to-face counselling is often more resource-intensive than other counselling options and can often only serve a small portion of the intended population. Telephone counselling is a potential alternative to some of the interventions mentioned above, and often has an opportunity to reach a large number of patients [12, 13]. Self-help interventions usually come in the form of self-guided manuals/pamphlets or programmes and are provided without the guidance of healthcare professionals. They come in various forms such as print and audio, video recordings or internet application (i.e., NHS Quit smoking app) [13 – 15].

#### Exercise interventions

Exercise has been considered as a potential smoking cessation aid. It can be offered alone or in conjunction with another smoking cessation intervention (such as counselling or pharmacotherapy) [16]. Various proposed mechanisms of action include increasing beta-endorphins through exercise (similarly to nicotine) to curb nicotine withdrawal and cravings [16, 17]. It has also been hypothesized that individuals who partake in routine exercise are more likely to be more health conscious and therefore likely to develop negative associations with smoking [16, 17].

#### Other therapies

Various other therapies have been investigated for their potential value in smoking cessation. Hypnotherapy is proposed to strengthen impulse control and is therefore potentially able to prevent an individual from actively participating in negative behaviours such as smoking [18]. Acupuncture and laser therapy have been used to stimulate the peripheral nerves, releasing neurotransmitters such as opioid peptides, dopamine, enkaphalin, and serotonin, which may be involved in the suppression of withdrawal symptoms [19]. Similarly, auricular stimulation involves the stimulation of acupuncture points on the earlobe and is thought to generate activation in higher areas of the brain that affect emotional lability often seen during the withdrawal phase of smoking dependence [20, 21]. St. John’s wort, an herbal product typically used as an anti-depressant, has been promoted as a treatment option for smoking cessation in the belief that it may reduce nicotine withdrawal symptoms [22]. Its mechanism is thought to inhibit the uptake of monoamine oxidase A and B, dopamine, and noradrenaline, which are all responsible for the reinforcing effects of nicotine and contribute to tobacco dependence [22]. S-Adenosylmethionine (SAMe), also a natural health product, produces dopamine and norepinephrine, which may alleviate nicotine withdrawal [23].

#### Electronic cigarettes

Electronic cigarettes (i.e., e-cigarettes, electronic nicotine delivery systems, or vapes) are battery-powered devices that heat a solution to deliver an aerosolized vapour with or without nicotine [24] and are gaining popularity amongst those who wish to quit cigarette smoking, especially among youth and young adults who were never smokers [25]. Electronic cigarettes may act as a cessation aid as a form of nicotine replacement, since they also satisfy the sensory and behavioral cues of holding and smoking a cigarette without providing the combustible harms associated with them (that is, formaldehyde, acrolein, or acetaldehyde) [25 - 27]. Since e-cigarettes are still relatively new on the market, the scientific data on their safety may not yet be fully known [28]. The second stage of this evidence review (i.e., de novo systematic review) synthesizes evidence on the benefits and harms of e-cigarettes as smoking cessation intervention in adults.

#### References:

1. Reid R, Pritchard G, Walker K, Aitken D, Mullen K-A, Pipe A. Managing smoking cessation. CMAJ 2016; 188:17–8.
2. Government of Canada HC. Licensed Natural Health Products Database (LNHPD). 2014.
3. McIvor A. Tobacco Control and Nicotine Addiction in Canada: Current trends, Management and Challenges. Can Respir J 2009; 16:21–6. https://doi.org/10.1155/2009/485953.
4. Etter J-F. Cytisine for Smoking Cessation. Arch Intern Med 2006;166.
5. Prochaska JJ, Das S, Benowitz NL. Cytisine, the world’s oldest smoking cessation aid. BMJ 2013;347: f5198–f5198. https://doi.org/10.1136/bmj.f5198.
6. Michie S, Hyder N, Walia A, West R. Development of a taxonomy of behaviour change techniques used in individual behavioural support for smoking cessation. Addict Behav 2011; 36:315–9. <https://doi.org/10.1016/j.addbeh.2010.11.016>.
7. Riemsma RP. Systematic review of the effectiveness of stage-based interventions to promote smoking cessation. BMJ 2003; 326:1175–7. https://doi.org/10.1136/bmj.326.7400.1175.
8. Cahill K, Lancaster T, Green N. Stage‐based interventions for smoking cessation. Cochrane Database Syst Rev 2010. <https://doi.org/10.1002/14651858.CD004492.pub4>.
9. Stead LF, Buitrago D, Preciado N, Sanchez G, Hartmann-Boyce J, Lancaster T. Physician advice for smoking cessation. Cochrane Database Syst Rev 2013:CD000165. https://doi.org/10.1002/14651858.CD000165.pub4.
10. Stead LF, Carroll AJ, Lancaster T. Group behaviour therapy programmes for smoking cessation. Cochrane Database Syst Rev 2017. https://doi.org/10.1002/14651858.CD001007.pub3.
11. Lancaster T, Stead LF. Individual behavioural counselling for smoking cessation. Cochrane Database Syst Rev 2017;3:CD001292.
12. Zhu S-H, Anderson CM, Tedeschi GJ, Rosbrook B, Johnson CE, Byrd M, et al. Evidence of real-world effectiveness of a telephone quitline for smokers. N Engl J Med 2002; 347:1087–93. https://doi.org/10.1056/NEJMsa020660.
13. Hartmann‐Boyce J, Lancaster T, Stead LF. Print‐based self‐help interventions for smoking cessation. Cochrane Database Syst Rev 2014. https://doi.org/10.1002/14651858.CD001118.pub3.
14. Taylor GMJ, Dalili MN, Semwal M, Civljak M, Sheikh A, Car J. Internet-based interventions for smoking cessation. Cochrane Database Syst Rev 2017;9:CD007078. <https://doi.org/10.1002/14651858.CD007078.pub5>.
15. https://www.facebook.com/nhswebsite. Quit smoking - Better Health. NhsUk 2020. https://www.nhs.uk/better-health/quit-smoking/ (accessed June 27, 2023).
16. Ussher MH, Taylor AH, Faulkner GEJ. Exercise interventions for smoking cessation. Cochrane Database Syst Rev 2014:CD002295. <https://doi.org/10.1002/14651858.CD002295.pub5>.
17. Hassandra M, Goudas M, Theodorakis Y. Exercise and Smoking: A Literature Overview. Health (N Y) 2015;07:1477–91. https://doi.org/10.4236/health.2015.711162.
18. Barnes J, McRobbie H, Dong CY, Walker N, Hartmann-Boyce J. Hypnotherapy for smoking cessation. Cochrane Database Syst Rev 2019.
19. White AR, Rampes H, Liu JP, Stead LF, Campbell J. Acupuncture and related interventions for smoking cessation. Cochrane Database Syst Rev 2014. https://doi.org/10.1002/14651858.CD000009.pub4.
20. Qureshi IS, Datta-Chaudhuri T, Tracey KJ, Pavlov VA, Chen ACH. Auricular neural stimulation as a new non-invasive treatment for opioid detoxification. Bioelectron Med 2020; 6:7. https://doi.org/10.1186/s42234-020-00044-6.
21. Bier ID, Wilson J, Studt P, Shakleton M. Auricular Acupuncture, Education, and Smoking Cessation: A Randomized, Sham-Controlled Trial. Am J Public Health 2002; 92:1642–7.
22. Sood A, Ebbert JO, Prasad K, Croghan IT, Bauer B, Schroeder DR. A Randomized Clinical Trial of St. John’s Wort for Smoking Cessation. J Altern Complement Med 2010; 16:761–7. <https://doi.org/10.1089/acm.2009.0445>.
23. Sood A, Prasad K, Croghan IT, Schroeder DR, Ehlers SL, Ebbert JO. S-Adenosyl-l-Methionine (SAMe) for Smoking Abstinence: A Randomized Clinical Trial. J Altern Complement Med 2012; 18:854–9. <https://doi.org/10.1089/acm.2011.0462>.
24. Kalkhoran S, Glantz SA. E-cigarettes and smoking cessation in real-world and clinical settings: a systematic review and meta-analysis. Lancet Respir Med 2016;4:116–28. https://doi.org/10.1016/S2213-2600(15)00521-4.
25. Bhatnagar A, Payne TJ, Robertson RM. Is There A Role for Electronic Cigarettes in Tobacco Cessation? J Am Heart Assoc 2019;8. <https://doi.org/10.1161/JAHA.119.012742>.
26. Farsalinos KE, Gillman G. Carbonyl Emissions in E-cigarette Aerosol: A Systematic Review and Methodological Considerations. Front Physiol 2018;8. <https://doi.org/10.3389/fphys.2017.01119>.
27. Information NC for B, Pike USNL of M 8600 R, MD B, Usa 20894. Smoking: E-cigarettes: An alternative to tobacco, or a quitting aid? Institute for Quality and Efficiency in Health Care (IQWiG); 2017.
28. Hartmann‐Boyce J, McRobbie H, Bullen C, Begh R, Stead LF, Hajek P. Electronic cigarettes for smoking cessation. Cochrane Libr 2016.
