# Supplementary material for: Effectiveness of smoking cessation interventions among adults: an overview of systematic reviews
Source: Syst Rev. 2024 Jul 12;13:179. doi: 10.1186/s13643-024-02570-9 (PMC11242003; doi:10.1186/s13643-024-02570-9)
Supplement: Supplementary file 4 — Additional file 4. Database search strategy. [file 13643_2024_2570_MOESM4_ESM.docx]

#### **Additional File 4. Database search strategy (original and updated)**

Database: Embase Classic+Embase <1947 to 2018 November 12>, Ovid MEDLINE(R) ALL <1946 to November 12, 2018>, PsycINFO <1806 to November Week 1 2018>

Search Strategy:

--------------------------------------------------------------------------------

1 Smoking Cessation/ (91359)

2 "Tobacco Use Cessation"/ (54104)

3 Smoking/dt [drug therapy] (1126)

4 Smoking/th [therapy] (1654)

5 exp Tobacco Smoking/dt [drug therapy] (180)

6 exp Tobacco Smoking/th [therapy] (58)

7 "Tobacco Use Disorder"/dt [drug therapy] (2679)

8 "Tobacco Use Disorder"/th [therapy] (1631)

9 ((smoking or smoker* or tobacco* or nicotine or cigar? or cigarette* or cigarillo?) adj5 (abstain* or abstinen* or cease or ceased or ceases or cessation* or dehabituat* or desist* or discontinu* or end or ended or ending or ends or "give up" or "giving up" or "gives up" or "gave up" or halt* or quit* or stop*)).tw,kf. (102746)

10 or/1-9 [SMOKING CESSATION] (133908)

11 "Tobacco Use Cessation Products"/ (4332)

12 (nicotine adj2 replac*).tw,kf. (8674)

13 (nrt? and (abstain* or abstinen* or cease or ceased or ceases or cessation* or dehabituat* or desist* or discontinu* or end or ended or ending or ends or "give up" or "giving up" or "gives up" or "gave up" or halt* or quit* or stop*)).tw,kf. (4004)

14 (nrt? and (smoking or smoke? or smoker? or cigar? or cigarette* or cigarillo? or nicotine or tobacco*)).tw,kf. (3397)

15 (nicotine adj3 (gum? or lozenge? or mist* or patch* or spray* or tablet* or transdermal* or inhalant? or inhaler? or inhalator? or inhalation*)).tw,kf. (7942)

16 (nrt? adj3 (gum? or lozenge? or mist* or patch* or spray* or tablet* or transdermal* or inhalant? or inhaler? or inhalator? or inhalation*)).tw,kf. (406)

17 (nicoderm* or nicorette*).tw,kf. (1135)

18 ((commit$2 or thrive$2) adj5 (gum? or lozenge? or nicotine or nrt?)).tw,kf. (28)

19 Varenicline/ (5117)

20 (varenicline or champix* or chantix* or CP 526555-18 or UNII-82269ASB48).tw,kf. (5040)

21 (cytisine or baptitoxin? or cravv or cystisine or cytiton? or cytizin or desmoxan or laburnin or sophorin? or tabax or tabex or tsitizin or ulexin? or "BRN 0083882" or EINECS 207-616-0 or HSDB 3560 or NSC 407282 or UNII-53S5U404NU).tw,kf. (1927)

22 (Zyban* or Zyntabac*).tw,kf. (1248)

23 Electronic Nicotine Delivery Systems/ (5924)

24 (electronic adj (cigar* or nicotine* or smok* or tobacco*)).tw,kf. (5652)

25 (ecig* or e-cig*).tw,kf. (6917)

26 Vaping/ (780)

27 (vape or vaped or vapes or vaping).tw,kf. (1337)

28 or/11-27 [SMOKING CESSATION PRODUCTS] (33478)

29 10 or 28 [SMOKING CESSATION, INCLUDING PRODUCTS] (146785)

30 limit 29 to systematic reviews [Limit not valid in Embase; records were retained] (78649)

31 meta analysis.pt. (94115)

32 exp meta-analysis as topic/ (55956)

33 (meta-analy* or metanaly* or metaanaly* or met analy* or integrative research or integrative review* or integrative overview* or research integration or research overview* or collaborative review*).tw,kf. (358837)

34 (systematic review* or systematic overview* or evidence-based review* or evidence-based overview* or (evidence adj3 (review* or overview*)) or meta-review* or meta-overview* or meta-synthes* or "review of reviews" or technology assessment* or HTA or HTAs).tw,kf. (425119)

35 exp Technology assessment, biomedical/ (23662)

36 (cochrane or health technology assessment or evidence report).jw. (38421)

37 (network adj (MA or MAs)).tw,kf. (16)

38 (NMA or NMAs).tw,kf. (4389)

39 indirect* compar*.tw,kf. (4695)

40 (indirect treatment* adj1 compar*).tw,kf. (631)

41 (mixed treatment* adj1 compar*).tw,kf. (1280)

42 (multiple treatment* adj1 compar*).tw,kf. (343)

43 (multi-treatment* adj1 compar*).tw,kf. (4)

44 simultaneous* compar*.tw,kf. (2370)

45 mixed comparison?.tw,kf. (63)

46 or/31-45 (743377)

47 29 and 46 (5778)

48 30 or 47 (79511)

49 exp Child/ not (exp Child/ and exp Adult/) (3195605)

50 exp Child/ not (exp Child/ and Adolescent/) (2941855)

51 exp Infant/ not (exp Infant/ and exp Adult/) (1656133)

52 exp Infant/ not (exp Infant/ and Adolescent/) (1673819)

53 or/49-52 (4161263)

54 48 not 53 [CHILD-/INFANT-ONLY REMOVED] (77185)

55 (editorial or news or newspaper article).pt. (1266918)

56 (letter not (letter and randomized controlled trial)).pt. (2044486)

57 54 not (55 or 56) [OPINION PIECES REMOVED] (71846)

58 57 use medall (3001)

59 smoking cessation/ (91359)

60 smoking cessation program/ (2966)

61 exp smoking/dt, th [Drug Therapy, Therapy] (2827)

62 tobacco dependence/dm, dt, th [Disease Management, Drug Therapy, Therapy] (4073)

63 ((smoking or smoker* or tobacco* or nicotine or cigar? or cigarette* or cigarillo?) adj5 (abstain* or abstinen* or cease or ceased or ceases or cessation* or dehabituat* or desist* or discontinu* or end or ended or ending or ends or "give up" or "giving up" or "gives up" or "gave up" or halt* or quit* or stop*)).tw,kw. (103080)

64 or/59-63 [SMOKING CESSATION] (134502)

65 nicotine gum/ (2825)

66 (nicotine adj2 replac*).tw,kw. (8783)

67 (nrt? and (abstain* or abstinen* or cease or ceased or ceases or cessation* or dehabituat* or desist* or discontinu* or end or ended or ending or ends or "give up" or "giving up" or "gives up" or "gave up" or halt* or quit* or stop*)).tw,kw. (4037)

68 (nrt? and (smoking or smoke? or smoker? or cigar? or cigarette* or cigarillo? or nicotine or tobacco*)).tw,kw. (3423)

69 (nicotine adj3 (gum? or lozenge? or mist* or patch* or spray* or tablet* or transdermal* or inhalant? or inhaler? or inhalator? or inhalation*)).tw,kw. (7968)

70 (nrt? adj3 (gum? or lozenge? or mist* or patch* or spray* or tablet* or transdermal* or inhalant? or inhaler? or inhalator? or inhalation*)).tw,kw. (406)

71 (nicoderm* or nicorette*).tw,kw. (1135)

72 ((commit$2 or thrive$2) adj5 (gum? or lozenge? or nicotine or nrt?)).tw,kw. (28)

73 commit.tn. (43)

74 thrive.tn. (3)

75 varenicline/ (5117)

76 (varenicline or champix* or chantix* or CP 526555-18 or UNII-82269ASB48).tw,kw. (5085)

77 (cytisine or baptitoxin? or cravv or cystisine or cytiton? or cytizin or desmoxan or laburnin or sophorin? or tabax or tabex or tsitizin or ulexin? or "BRN 0083882" or EINECS 207-616-0 or HSDB 3560 or NSC 407282 or UNII-53S5U404NU).tw,kw. (1942)

78 (Zyban* or Zyntabac*).tw,kw. (1251)

79 electronic cigarette/ (6789)

80 (electronic adj (cigar* or nicotine* or smok* or tobacco*)).tw,kw. (5623)

81 (ecig* or e-cig*).tw,kw. (7015)

82 vaping/ (780)

83 (vape or vaped or vapes or vaping).tw,kw. (1408)

84 or/65-83 [SMOKING CESSATION PRODUCTS] (33260)

85 64 or 84 [SMOKING CESSATION & PRODUCTS] (147233)

86 meta-analysis/ (250468)

87 "systematic review"/ (184128)

88 "meta analysis (topic)"/ (38909)

89 (meta-analy* or metanaly* or metaanaly* or met analy* or integrative research or integrative review* or integrative overview* or research integration or research overview* or collaborative review*).tw,kw. (361612)

90 (systematic review* or systematic overview* or evidence-based review* or evidence-based overview* or (evidence adj3 (review* or overview*)) or meta-review* or meta-overview* or meta-synthes* or "review of reviews" or technology assessment* or HTA or HTAs).tw,kw. (428278)

91 biomedical technology assessment/ (22555)

92 (cochrane or health technology assessment or evidence report).jw. (38421)

93 (network adj (MA or MAs)).tw,kw. (16)

94 (NMA or NMAs).tw,kw. (4408)

95 indirect* compar*.tw,kw. (4757)

96 (indirect treatment* adj1 compar*).tw,kw. (635)

97 (mixed treatment* adj1 compar*).tw,kw. (1305)

98 (multiple treatment* adj1 compar*).tw,kw. (348)

99 (multi-treatment* adj1 compar*).tw,kw. (4)

100 simultaneous* compar*.tw,kw. (2370)

101 mixed comparison?.tw,kw. (64)

102 or/86-101 (803440)

103 85 and 102 [REVIEWS] (6482)

104 exp child/ not (exp child/ and exp adult/) (3195605)

105 exp child/ not (exp child/ and adolescent/) (2941855)

106 fetus/ not (fetus/ and exp adult/) (237951)

107 fetus/ not (fetus/ and adolescent/) (274120)

108 or/104-107 (3868885)

109 103 not 108 [CHILD-/INFANT-/FETUS-ONLY REMOVED] (6342)

110 exp animal experimentation/ or exp animal model/ or exp animal experiment/ or nonhuman/ or exp vertebrate/ (48238195)

111 exp human/ or exp human experimentation/ or exp human experiment/ (37723542)

112 110 not 111 (10516358)

113 109 not 112 [ANIMAL-ONLY REMOVED] (6330)

114 editorial.pt. (1057883)

115 letter.pt. not (randomized controlled trial/ and letter.pt.) (2039541)

116 113 not (114 or 115) [OPINION PIECES REMOVED] (6184)

117 116 use emczd [EMBASE RECORDS] (3579)

118 smoking cessation/ (91359)

119 ((smoking or smoker* or tobacco* or nicotine or cigar? or cigarette* or cigarillo?) adj5 (abstain* or abstinen* or cease or ceased or ceases or cessation* or dehabituat* or desist* or discontinu* or end or ended or ending or ends or "give up" or "giving up" or "gives up" or "gave up" or halt* or quit* or stop*)).tw. (102515)

120 118 or 119 [SMOKING CESSATION] (132256)

121 (nicotine adj2 replac*).tw. (8614)

122 (nrt? and (abstain* or abstinen* or cease or ceased or ceases or cessation* or dehabituat* or desist* or discontinu* or end or ended or ending or ends or "give up" or "giving up" or "gives up" or "gave up" or halt* or quit* or stop*)).tw. (3983)

123 (nrt? and (smoking or smoke? or smoker? or cigar? or cigarette* or cigarillo? or nicotine or tobacco*)).tw. (3382)

124 (nicotine adj3 (gum? or lozenge? or mist* or patch* or spray* or tablet* or transdermal* or inhalant? or inhaler? or inhalator? or inhalation*)).tw. (7933)

125 (nrt? adj3 (gum? or lozenge? or mist* or patch* or spray* or tablet* or transdermal* or inhalant? or inhaler? or inhalator? or inhalation*)).tw. (406)

126 (nicoderm* or nicorette*).tw. (1134)

127 ((commit$2 or thrive$2) adj5 (gum? or lozenge? or nicotine or nrt?)).tw. (28)

128 (varenicline or champix* or chantix* or CP 526555-18 or UNII-82269ASB48).tw. (5015)

129 (cytisine or baptitoxin? or cravv or cystisine or cytiton? or cytizin or desmoxan or laburnin or sophorin? or tabax or tabex or tsitizin or ulexin? or "BRN 0083882" or EINECS 207-616-0 or HSDB 3560 or NSC 407282 or UNII-53S5U404NU).tw. (1919)

130 (Zyban* or Zyntabac*).tw. (1245)

131 electronic cigarettes/ (6303)

132 (electronic adj (cigar* or nicotine* or smok* or tobacco*)).tw. (5356)

133 (ecig* or e-cig*).tw. (6824)

134 (vape or vaped or vapes or vaping).tw. (1261)

135 or/121-134 (30937)

136 120 or 135 (144636)

137 limit 136 to ("0830 systematic review" or 1200 meta analysis or 1300 metasynthesis) [Limit not valid in Embase,Ovid MEDLINE(R),Ovid MEDLINE(R) Daily Update,Ovid MEDLINE(R) In-Process,Ovid MEDLINE(R) Publisher; records were retained] (125050)

138 meta analysis/ (250468)

139 (meta-analy* or metanaly* or metaanaly* or met analy* or integrative research or integrative review* or integrative overview* or research integration or research overview* or collaborative review*).tw. (357711)

140 (systematic review* or systematic overview* or evidence-based review* or evidence-based overview* or (evidence adj3 (review* or overview*)) or meta-review* or meta-overview* or meta-synthes* or "review of reviews" or technology assessment* or HTA or HTAs).tw. (423550)

141 (network adj (MA or MAs)).tw. (16)

142 (NMA or NMAs).tw. (4378)

143 indirect* compar*.tw. (4674)

144 (indirect treatment* adj1 compar*).tw. (617)

145 (mixed treatment* adj1 compar*).tw. (1229)

146 (multiple treatment* adj1 compar*).tw. (330)

147 (multi-treatment* adj1 compar*).tw. (4)

148 simultaneous* compar*.tw. (2370)

149 mixed comparison?.tw. (63)

150 or/138-149 (707269)

151 136 and 150 (5535)

152 137 or 151 (125364)

153 152 use medall,emczd (124633)

154 152 not 153 [PSCYINFO RECORDS] (731)

155 58 or 117 or 154 (7311)

156 limit 155 to yr="2008-current" (5273)

157 remove duplicates from 156 (3525) [TOTAL UNIQUE RECORDS]

158 157 use medall (2044) [MEDLINE UNIQUE RECORDS]

159 157 use emczd (1304) [EMBASE UNIQUE RECORDS]

160 157 not (158 or 159) (177) [PSYCINFO UNIQUE RECORDS]

***************************

Database: EBM Reviews - Cochrane Database of Systematic Reviews <2005 to November 8, 2018>, EBM Reviews - Database of Abstracts of Reviews of Effects <1st Quarter 2016>, EBM Reviews - Health Technology Assessment <4th Quarter 2016>

Search Strategy:

--------------------------------------------------------------------------------

1 Smoking Cessation/ (81)

2 "Tobacco Use Cessation"/ (3)

3 Smoking/dt [drug therapy] (2)

4 Smoking/th [therapy] (0)

5 exp Tobacco Smoking/dt [drug therapy] (0)

6 exp Tobacco Smoking/th [therapy] (0)

7 "Tobacco Use Disorder"/dt [drug therapy] (1)

8 "Tobacco Use Disorder"/th [therapy] (2)

9 ((smoking or smoker* or tobacco* or nicotine or cigar? or cigarette* or cigarillo?) adj5 (abstain* or abstinen* or cease or ceased or ceases or cessation* or dehabituat* or desist* or discontinu* or end or ended or ending or ends or "give up" or "giving up" or "gives up" or "gave up" or halt* or quit* or stop*)).ti,ab,kw. (390)

10 or/1-9 [SMOKING CESSATION] (406)

11 "Tobacco Use Cessation Products"/ (3)

12 (nicotine adj2 replac*).ti,ab,kw. (46)

13 (nrt? and (abstain* or abstinen* or cease or ceased or ceases or cessation* or dehabituat* or desist* or discontinu* or end or ended or ending or ends or "give up" or "giving up" or "gives up" or "gave up" or halt* or quit* or stop*)).ti,ab,kw. (24)

14 (nrt? and (smoking or smoke? or smoker? or cigar? or cigarette* or cigarillo? or nicotine or tobacco*)).ti,ab,kw. (23)

15 (nicotine adj3 (gum? or lozenge? or mist* or patch* or spray* or tablet* or transdermal* or inhalant? or inhaler? or inhalator? or inhalation*)).ti,ab,kw. (31)

16 (nrt? adj3 (gum? or lozenge? or mist* or patch* or spray* or tablet* or transdermal* or inhalant? or inhaler? or inhalator? or inhalation*)).ti,ab,kw. (2)

17 (nicoderm* or nicorette*).ti,ab,kw. (0)

18 ((commit$2 or thrive$2) adj5 (gum? or lozenge? or nicotine or nrt?)).ti,ab,kw. (0)

19 Varenicline/ (1)

20 (varenicline or champix* or chantix* or CP 526555-18 or UNII-82269ASB48).ti,ab,kw. (33)

21 (cytisine or baptitoxin? or cravv or cystisine or cytiton? or cytizin or desmoxan or laburnin or sophorin? or tabax or tabex or tsitizin or ulexin? or "BRN 0083882" or EINECS 207-616-0 or HSDB 3560 or NSC 407282 or UNII-53S5U404NU).ti,ab,kw. (6)

22 (Zyban* or Zyntabac*).ti,ab,kw. (1)

23 Electronic Nicotine Delivery Systems/ (0)

24 (electronic adj (cigar* or nicotine* or smok* or tobacco*)).ti,ab,kw. (7)

25 (ecig* or e-cig*).ti,ab,kw. (3)

26 Vaping/ (0)

27 (vape or vaped or vapes or vaping).ti,ab,kw. (0)

28 or/11-27 [SMOKING CESSATION PRODUCTS] (94)

29 10 or 28 [SMOKING CESSATION, INCLUDING PRODUCTS] (416)

30 limit 29 to yr="2008-current" [Limit not valid in DARE; records were retained] (377)

31 remove duplicates from 30 (377)

32 31 use coch (70)

33 31 use dare (223)

34 31 use clhta (84)

***************************

Smoking Cessation

Overview – Updated search

2020 Sep 24

Ovid Multifile

Database: Embase Classic+Embase <1947 to 2020 September 23> , Ovid MEDLINE(R) ALL <1946 to September 23, 2020>, APA PsycInfo <1806 to September Week 2 2020>

Search Strategy:

--------------------------------------------------------------------------------

1 Smoking Cessation/ (101800)

2 "Tobacco Use Cessation"/ (60843)

3 Smoking/dt [drug therapy] (1182)

4 Smoking/th [therapy] (2216)

5 exp Tobacco Smoking/dt [drug therapy] (209)

6 exp Tobacco Smoking/th [therapy] (490)

7 "Tobacco Use Disorder"/dt [drug therapy] (3122)

8 "Tobacco Use Disorder"/th [therapy] (3121)

9 ((smoking or smoker* or tobacco* or nicotine or cigar? or cigarette* or cigarillo?) adj5 (abstain* or abstinen* or cease or ceased or ceases or cessation* or dehabituat* or desist* or discontinu* or end or ended or ending or ends or "give up" or "giving up" or "gives up" or "gave up" or halt* or quit* or stop*)).tw,kf. (116469)

10 or/1-9 [SMOKING CESSATION] (150282)

11 "Tobacco Use Cessation Products"/ (4770)

12 (nicotine adj2 replac*).tw,kf. (9757)

13 (nrt? and (abstain* or abstinen* or cease or ceased or ceases or cessation* or dehabituat* or desist* or discontinu* or end or ended or ending or ends or "give up" or "giving up" or "gives up" or "gave up" or halt* or quit* or stop*)).tw,kf. (4639)

14 (nrt? and (smoking or smoke? or smoker? or cigar? or cigarette* or cigarillo? or nicotine or tobacco*)).tw,kf. (3987)

15 (nicotine adj3 (gum? or lozenge? or mist* or patch* or spray* or tablet* or transdermal* or inhalant? or inhaler? or inhalator? or inhalation*)).tw,kf. (8452)

16 (nrt? adj3 (gum? or lozenge? or mist* or patch* or spray* or tablet* or transdermal* or inhalant? or inhaler? or inhalator? or inhalation*)).tw,kf. (476)

17 (nicoderm* or nicorette*).tw,kf. (1162)

18 ((commit$2 or thrive$2) adj5 (gum? or lozenge? or nicotine or nrt?)).tw,kf. (29)

19 Varenicline/ (5782)

20 (varenicline or champix* or chantix* or CP 526555-18 or UNII-82269ASB48).tw,kf. (5732)

21 (cytisine or baptitoxin? or cravv or cystisine or cytiton? or cytizin or desmoxan or laburnin or sophorin? or tabax or tabex or tsitizin or ulexin? or "BRN 0083882" or EINECS 207-616-0 or HSDB 3560 or NSC 407282 or UNII-53S5U404NU).tw,kf. (2044)

22 (Zyban* or Zyntabac*).tw,kf. (1269)

23 Electronic Nicotine Delivery Systems/ (10224)

24 (electronic adj (cigar* or nicotine* or smok* or tobacco*)).tw,kf. (9273)

25 (ecig* or e-cig*).tw,kf. (12298)

26 Vaping/ (3329)

27 (vape or vaped or vapes or vaping).tw,kf. (3933)

28 or/11-27 [SMOKING CESSATION PRODUCTS] (43220)

29 10 or 28 [SMOKING CESSATION, INCLUDING PRODUCTS] (168738)

30 limit 29 to systematic reviews [Limit not valid in Embase; records were retained] (89247)

31 meta analysis.pt. (119996)

32 exp meta-analysis as topic/ (63151)

33 (meta-analy* or metanaly* or metaanaly* or met analy* or integrative research or integrative review* or integrative overview* or research integration or research overview* or collaborative review*).tw,kf. (470756)

34 (systematic review* or systematic overview* or evidence-based review* or evidence-based overview* or (evidence adj3 (review* or overview*)) or meta-review* or meta-overview* or meta-synthes* or "review of reviews" or technology assessment* or HTA or HTAs).tw,kf. (562297)

35 exp Technology assessment, biomedical/ (25802)

36 (cochrane or health technology assessment or evidence report).jw. (41005)

37 (network adj (MA or MAs)).tw,kf. (30)

38 (NMA or NMAs).tw,kf. (5980)

39 indirect* compar*.tw,kf. (5931)

40 (indirect treatment* adj1 compar*).tw,kf. (972)

41 (mixed treatment* adj1 compar*).tw,kf. (1393)

42 (multiple treatment* adj1 compar*).tw,kf. (432)

43 (multi-treatment* adj1 compar*).tw,kf. (9)

44 simultaneous* compar*.tw,kf. (2677)

45 mixed comparison?.tw,kf. (84)

46 or/31-45 (934628)

47 29 and 46 (6941)

48 30 or 47 (91005)

49 exp Child/ not (exp Child/ and exp Adult/) (3463176)

50 exp Child/ not (exp Child/ and Adolescent/) (3194636)

51 exp Infant/ not (exp Infant/ and exp Adult/) (1763309)

52 exp Infant/ not (exp Infant/ and Adolescent/) (1791292)

53 or/49-52 (4509248)

54 48 not 53 [CHILD-/INFANT-ONLY REMOVED] (88190)

55 (editorial or news or newspaper article).pt. (1429543)

56 (letter not (letter and randomized controlled trial)).pt. (2235203)

57 54 not (55 or 56) [OPINION PIECES REMOVED] (82045)

58 57 use medall (2399)

59 smoking cessation/ (101800)

60 smoking cessation program/ (3374)

61 exp smoking/dt, th [Drug Therapy, Therapy] (3615)

62 tobacco dependence/dm, dt, th [Disease Management, Drug Therapy, Therapy] (5567)

63 ((smoking or smoker* or tobacco* or nicotine or cigar? or cigarette* or cigarillo?) adj5 (abstain* or abstinen* or cease or ceased or ceases or cessation* or dehabituat* or desist* or discontinu* or end or ended or ending or ends or "give up" or "giving up" or "gives up" or "gave up" or halt* or quit* or stop*)).tw,kw. (116843)

64 or/59-63 [SMOKING CESSATION] (150974)

65 nicotine gum/ (2936)

66 (nicotine adj2 replac*).tw,kw. (9874)

67 (nrt? and (abstain* or abstinen* or cease or ceased or ceases or cessation* or dehabituat* or desist* or discontinu* or end or ended or ending or ends or "give up" or "giving up" or "gives up" or "gave up" or halt* or quit* or stop*)).tw,kw. (4673)

68 (nrt? and (smoking or smoke? or smoker? or cigar? or cigarette* or cigarillo? or nicotine or tobacco*)).tw,kw. (4015)

69 (nicotine adj3 (gum? or lozenge? or mist* or patch* or spray* or tablet* or transdermal* or inhalant? or inhaler? or inhalator? or inhalation*)).tw,kw. (8483)

70 (nrt? adj3 (gum? or lozenge? or mist* or patch* or spray* or tablet* or transdermal* or inhalant? or inhaler? or inhalator? or inhalation*)).tw,kw. (476)

71 (nicoderm* or nicorette*).tw,kw. (1164)

72 ((commit$2 or thrive$2) adj5 (gum? or lozenge? or nicotine or nrt?)).tw,kw. (31)

73 commit.tn. (44)

74 thrive.tn. (3)

75 varenicline/ (5782)

76 (varenicline or champix* or chantix* or CP 526555-18 or UNII-82269ASB48).tw,kw. (5785)

77 (cytisine or baptitoxin? or cravv or cystisine or cytiton? or cytizin or desmoxan or laburnin or sophorin? or tabax or tabex or tsitizin or ulexin? or "BRN 0083882" or EINECS 207-616-0 or HSDB 3560 or NSC 407282 or UNII-53S5U404NU).tw,kw. (2060)

78 (Zyban* or Zyntabac*).tw,kw. (1274)

79 electronic cigarette/ (11807)

80 (electronic adj (cigar* or nicotine* or smok* or tobacco*)).tw,kw. (9263)

81 (ecig* or e-cig*).tw,kw. (12514)

82 vaping/ (3329)

83 (vape or vaped or vapes or vaping).tw,kw. (4111)

84 or/65-83 [SMOKING CESSATION PRODUCTS] (43009)

85 64 or 84 [SMOKING CESSATION & PRODUCTS] (169320)

86 meta-analysis/ (321129)

87 "systematic review"/ (398041)

88 "meta analysis (topic)"/ (43072)

89 (meta-analy* or metanaly* or metaanaly* or met analy* or integrative research or integrative review* or integrative overview* or research integration or research overview* or collaborative review*).tw,kw. (473989)

90 (systematic review* or systematic overview* or evidence-based review* or evidence-based overview* or (evidence adj3 (review* or overview*)) or meta-review* or meta-overview* or meta-synthes* or "review of reviews" or technology assessment* or HTA or HTAs).tw,kw. (566269)

91 biomedical technology assessment/ (24690)

92 (cochrane or health technology assessment or evidence report).jw. (41005)

93 (network adj (MA or MAs)).tw,kw. (30)

94 (NMA or NMAs).tw,kw. (6000)

95 indirect* compar*.tw,kw. (6018)

96 (indirect treatment* adj1 compar*).tw,kw. (977)

97 (mixed treatment* adj1 compar*).tw,kw. (1416)

98 (multiple treatment* adj1 compar*).tw,kw. (440)

99 (multi-treatment* adj1 compar*).tw,kw. (9)

100 simultaneous* compar*.tw,kw. (2677)

101 mixed comparison?.tw,kw. (85)

102 or/86-101 (1020934)

103 85 and 102 [REVIEWS] (7805)

104 exp child/ not (exp child/ and exp adult/) (3463176)

105 exp child/ not (exp child/ and adolescent/) (3194636)

106 fetus/ not (fetus/ and exp adult/) (248303)

107 fetus/ not (fetus/ and adolescent/) (289691)

108 or/104-107 (4199035)

109 103 not 108 [CHILD-/INFANT-/FETUS-ONLY REMOVED] (7635)

110 exp animal experimentation/ or exp animal model/ or exp animal experiment/ or nonhuman/ or exp vertebrate/ (52651753)

111 exp human/ or exp human experimentation/ or exp human experiment/ (41458032)

112 110 not 111 (11195541)

113 109 not 112 [ANIMAL-ONLY REMOVED] (7619)

114 editorial.pt. (1209888)

115 letter.pt. not (randomized controlled trial/ and letter.pt.) (2229803)

116 113 not (114 or 115) [OPINION PIECES REMOVED] (7460)

117 116 use emczd [EMBASE RECORDS] (4291)

118 smoking cessation/ (101800)

119 ((smoking or smoker* or tobacco* or nicotine or cigar? or cigarette* or cigarillo?) adj5 (abstain* or abstinen* or cease or ceased or ceases or cessation* or dehabituat* or desist* or discontinu* or end or ended or ending or ends or "give up" or "giving up" or "gives up" or "gave up" or halt* or quit* or stop*)).tw. (116115)

120 118 or 119 [SMOKING CESSATION] (148177)

121 (nicotine adj2 replac*).tw. (9680)

122 (nrt? and (abstain* or abstinen* or cease or ceased or ceases or cessation* or dehabituat* or desist* or discontinu* or end or ended or ending or ends or "give up" or "giving up" or "gives up" or "gave up" or halt* or quit* or stop*)).tw. (4613)

123 (nrt? and (smoking or smoke? or smoker? or cigar? or cigarette* or cigarillo? or nicotine or tobacco*)).tw. (3968)

124 (nicotine adj3 (gum? or lozenge? or mist* or patch* or spray* or tablet* or transdermal* or inhalant? or inhaler? or inhalator? or inhalation*)).tw. (8439)

125 (nrt? adj3 (gum? or lozenge? or mist* or patch* or spray* or tablet* or transdermal* or inhalant? or inhaler? or inhalator? or inhalation*)).tw. (476)

126 (nicoderm* or nicorette*).tw. (1159)

127 ((commit$2 or thrive$2) adj5 (gum? or lozenge? or nicotine or nrt?)).tw. (29)

128 (varenicline or champix* or chantix* or CP 526555-18 or UNII-82269ASB48).tw. (5698)

129 (cytisine or baptitoxin? or cravv or cystisine or cytiton? or cytizin or desmoxan or laburnin or sophorin? or tabax or tabex or tsitizin or ulexin? or "BRN 0083882" or EINECS 207-616-0 or HSDB 3560 or NSC 407282 or UNII-53S5U404NU).tw. (2035)

130 (Zyban* or Zyntabac*).tw. (1263)

131 electronic cigarettes/ (11330)

132 (electronic adj (cigar* or nicotine* or smok* or tobacco*)).tw. (8736)

133 (ecig* or e-cig*).tw. (12077)

134 (vape or vaped or vapes or vaping).tw. (3747)

135 or/121-134 (40317)

136 120 or 135 (166023)

137 limit 136 to ("0830 systematic review" or 1200 meta analysis or 1300 metasynthesis) [Limit not valid in Embase,Ovid MEDLINE(R),Ovid MEDLINE(R) Daily Update,Ovid MEDLINE(R) In-Process,Ovid MEDLINE(R) Publisher; records were retained] (144104)

138 meta analysis/ (321129)

139 (meta-analy* or metanaly* or metaanaly* or met analy* or integrative research or integrative review* or integrative overview* or research integration or research overview* or collaborative review*).tw. (469167)

140 (systematic review* or systematic overview* or evidence-based review* or evidence-based overview* or (evidence adj3 (review* or overview*)) or meta-review* or meta-overview* or meta-synthes* or "review of reviews" or technology assessment* or HTA or HTAs).tw. (559938)

141 (network adj (MA or MAs)).tw. (30)

142 (NMA or NMAs).tw. (5960)

143 indirect* compar*.tw. (5900)

144 (indirect treatment* adj1 compar*).tw. (947)

145 (mixed treatment* adj1 compar*).tw. (1328)

146 (multiple treatment* adj1 compar*).tw. (416)

147 (multi-treatment* adj1 compar*).tw. (9)

148 simultaneous* compar*.tw. (2677)

149 mixed comparison?.tw. (84)

150 or/138-149 (896419)

151 136 and 150 (6633)

152 137 or 151 (144456)

153 152 use medall,emczd (143600)

154 152 not 153 [PSCYINFO RECORDS] (856)

155 58 or 117 or 154 (7546)

156 limit 155 to yr="2008-current" (5894)

157 remove duplicates from 156 (3708)

158 157 use medall [MEDLINE UNIQUE RECORDS] (1825)

159 (201811* or 201812* or 2019* or 2020*).dt. (2562848)

160 158 and 159 [UNIQUE MEDLINE RECORDS - UPDATE PERIOD] (419)

161 157 use emczd [EMBASE UNIQUE RECORDS] (1673)

162 (201811* or 201812* or 2019* or 2020*).dc. (3681573)

163 161 and 162 [EMBASE UNIQUE RECORDS - UPDATE PERIOD] (318)

164 157 not (158 or 161) [PSYCINFO UNIQUE RECORDS] (210)

165 (201811* or 201812* or 2019* or 2020*).up. (36682421)

166 164 and 165 [PSYCINFO UNIQUE RECORDS - UPDATE PERIOD] (33)

167 160 or 163 or 166 (770) [TOTAL UNIQUE RECORDS – UPDATE PERIOD]

***************************

Cochrane Library

Database: EBM Reviews - Cochrane Database of Systematic Reviews <2005 to September 23, 2020>, EBM Reviews - Database of Abstracts of Reviews of Effects <1st Quarter 2016>, EBM Reviews - Health Technology Assessment <4th Quarter 2016>

Search Strategy:

--------------------------------------------------------------------------------

1 Smoking Cessation/ (81)

2 "Tobacco Use Cessation"/ (3)

3 Smoking/dt [drug therapy] (2)

4 Smoking/th [therapy] (0)

5 exp Tobacco Smoking/dt [drug therapy] (0)

6 exp Tobacco Smoking/th [therapy] (0)

7 "Tobacco Use Disorder"/dt [drug therapy] (1)

8 "Tobacco Use Disorder"/th [therapy] (2)

9 ((smoking or smoker* or tobacco* or nicotine or cigar? or cigarette* or cigarillo?) adj5 (abstain* or abstinen* or cease or ceased or ceases or cessation* or dehabituat* or desist* or discontinu* or end or ended or ending or ends or "give up" or "giving up" or "gives up" or "gave up" or halt* or quit* or stop*)).ti,ab,kw. (405)

10 or/1-9 [SMOKING CESSATION] (421)

11 "Tobacco Use Cessation Products"/ (3)

12 (nicotine adj2 replac*).ti,ab,kw. (49)

13 (nrt? and (abstain* or abstinen* or cease or ceased or ceases or cessation* or dehabituat* or desist* or discontinu* or end or ended or ending or ends or "give up" or "giving up" or "gives up" or "gave up" or halt* or quit* or stop*)).ti,ab,kw. (26)

14 (nrt? and (smoking or smoke? or smoker? or cigar? or cigarette* or cigarillo? or nicotine or tobacco*)).ti,ab,kw. (25)

15 (nicotine adj3 (gum? or lozenge? or mist* or patch* or spray* or tablet* or transdermal* or inhalant? or inhaler? or inhalator? or inhalation*)).ti,ab,kw. (34)

16 (nrt? adj3 (gum? or lozenge? or mist* or patch* or spray* or tablet* or transdermal* or inhalant? or inhaler? or inhalator? or inhalation*)).ti,ab,kw. (4)

17 (nicoderm* or nicorette*).ti,ab,kw. (0)

18 ((commit$2 or thrive$2) adj5 (gum? or lozenge? or nicotine or nrt?)).ti,ab,kw. (0)

19 Varenicline/ (1)

20 (varenicline or champix* or chantix* or CP 526555-18 or UNII-82269ASB48).ti,ab,kw. (34)

21 (cytisine or baptitoxin? or cravv or cystisine or cytiton? or cytizin or desmoxan or laburnin or sophorin? or tabax or tabex or tsitizin or ulexin? or "BRN 0083882" or EINECS 207-616-0 or HSDB 3560 or NSC 407282 or UNII-53S5U404NU).ti,ab,kw. (6)

22 (Zyban* or Zyntabac*).ti,ab,kw. (1)

23 Electronic Nicotine Delivery Systems/ (0)

24 (electronic adj (cigar* or nicotine* or smok* or tobacco*)).ti,ab,kw. (8)

25 (ecig* or e-cig*).ti,ab,kw. (3)

26 Vaping/ (0)

27 (vape or vaped or vapes or vaping).ti,ab,kw. (0)

28 or/11-27 [SMOKING CESSATION PRODUCTS] (95)

29 10 or 28 [SMOKING CESSATION, INCLUDING PRODUCTS] (431)

30 limit 29 to yr="2008-current" [Limit not valid in DARE; records were retained] (406)

31 (201811* or 201812* or 2019* or 2020*).up. (4633)

32 30 and 31 [UPDATE PERIOD] (64)

33 32 use dare,clhta (0)

34 32 use coch [DSR UPDATE RECORDS] (64)

***************************
