# Supplementary material for: Effectiveness of smoking cessation interventions among adults: an overview of systematic reviews
Source: Syst Rev. 2024 Jul 12;13:179. doi: 10.1186/s13643-024-02570-9 (PMC11242003; doi:10.1186/s13643-024-02570-9)
Supplement: Supplementary file 5 — Additional file 5. Grey literature sources. [file 13643_2024_2570_MOESM5_ESM.docx]

### **Additional file 5. Grey literature sources**

Several websites were searched including American Cancer Society,

American Thoracic Society,

CADTH,

Canadian Cancer Society, Foundation for a Smoke-Free World, International Prevention Research Institute, NICE, North American Quitline Consortium, Ontario Tobacco Research Unit, OpenTrials, Ottawa Heart Institute’s Ottawa Model for Smoking Cessation, Philip Morris, Physicians for a Smoke-Free Canada, Public Health England, The Canadian Partnership Against Cancer, Tobacco.org, Truth Initiative, US Centers for Disease Control and Prevention Smoking and Health Resource Library, US National Cancer Institute, US National Comprehensive Cancer Network, US Office of the Surgeon General, World Health Organization Framework Convention on Tobacco Control, and World Health Organization’s International Clinical Trials Registry Platform.
