# Supplementary material for: Effectiveness of smoking cessation interventions among adults: an overview of systematic reviews
Source: Syst Rev. 2024 Jul 12;13:179. doi: 10.1186/s13643-024-02570-9 (PMC11242003; doi:10.1186/s13643-024-02570-9)
Supplement: Supplementary file 6 — Additional file 6. Eligibility criteria (PICO criteria). [file 13643_2024_2570_MOESM6_ESM.docx]

### **Additional file 6 - Eligibility criteria**

|  | **Inclusion** | **Exclusion** |
| --- | --- | --- |
| Population | Adults (≥18 years) who are current tobacco smokers (as defined by a given study/review)  The overview of reviews will seek information on the following population groups:   - General/mixed population of smokers - Smokers motivated to quit - Smokers not motivated to quit - Smokers with mental illness | - Reviews exclusively in children/adolescents (i.e., under 18 years old) - Reviews that focus solely on interventions targeted to adults other than the tobacco smoker (e.g., partners, healthcare providers) - Pregnant individuals - Smokers with comorbidities other than mental illness |
| Intervention | Interventions to promote abrupt (i.e., ‘all at once’) or gradual (reducing smoking to quit) tobacco smoking cessation that can be directly delivered or referred to by primary care practitioners and are available in Canada^1^   - Practitioner advice (of varying length/intensity, and by various provider types)   - Very brief/minimal advice (as defined by a given review)   - Brief advice (as defined by a given review) - Intensive individual counselling (of varying length, of varying number of sessions, and by various provider types) - Intensive group counselling (of varying length, of varying number of sessions, and by various provider types) - Self-help interventions^3^ (print-based or web/computer-based) - Internet or computer-based interventions with counselling/support^3^ - Telephone-based interventions (e.g., mobile phone-based, quit lines/help lines) with counselling/support ^3^ - Nicotine receptor partial agonists (varenicline and cytisine^4^) - Bupropion - Nicotine replacement therapy^5^ (e.g., patch, gum, lozenge, mist, inhaler) - E-cigarettes^6^ - Exercise interventions - ‘Alternative’ therapies (e.g., acupuncture, acupressure, electrostimulation, hypnosis, St. John’s Wort^4,^ S-adenosylmethionine^4^) - Combinations of interventions   Other interventions encountered in the literature were assessed on a case-by-case basis in consultation with the Working Group (WG).  Information on the effects of variations in the delivery of stop smoking interventions (e.g., dose, duration of intervention, number of session) was also included, where reported. | Interventions that cannot feasibly or readily be delivered or referred to by a wide variety of primary care practitioners:   - Quit-to-win contests - Biomedical risk assessment - Aversive smoking (e.g., rapid smoking) - Incentivized cessation   Reviews that focus solely on specialized behavioural counselling interventions (e.g., motivational interviewing, stage of change-based interventions).^2^  Pharmacotherapies that are not approved by Health Canada as smoking cessation aids (e.g., clonidine, lobeline, anxiolytics, nortriptyline, opioid antagonists, silver acetate, rimonabant) or not available in Canada (e.g., Nicobrevin, Nicobloc, nicotine vaccines, mecamylamine)  Broader public health interventions (e.g., mass media, taxation, packaging restrictions)  Reviews on broad lifestyle interventions not specific to tobacco smoking behaviour, and that do not attempt to isolate for the effect of our included interventions (when delivered as part of a multifaceted lifestyle intervention, for example) |
| Comparator | - Placebo - No intervention - Usual care - Waitlist - Minimal intervention | - Other intervention (e.g., head-to-head comparisons, comparisons of types or intensities of advice/counselling) - Other combination of interventions - The same intervention, but used to promote cessation by reducing smoking to quit as opposed to quitting abruptly or vice versa |
| Outcomes | **Benefits**  Critical   - Tobacco use abstinence (as defined in a given review)   Important   - Reduction in tobacco smoking frequency/quantity - Quality of life (using validated scales)   **Harms**  Important   - Adverse events (as defined in a given review) - Possible adverse outcomes: - Weight gain - Changes in emotional state (e.g., increases in anxiety, changes in mood, irritability) - Loss of social group^7^ | N/A |
| Timing of outcome assessment | For abstinence, reduction, and quality of life outcomes:  Minimum six months from quit date (if reported) or from initiation of intervention (if quit date not specified)  All other outcomes:  Any point after initiation of intervention | N/A |
| Setting | Reviews in which some or all of the included studies are in settings that could serve as the primary point of contact for individuals to receive smoking cessation advice, including:   - - Family medicine clinics   - Walk-in clinics   - Smoking cessation clinics   - Urgent care facilities   - Emergency departments   - Public health units   - Pharmacies   - Dental offices   - Behavioural health/substance use treatment facilities (ambulatory or outpatient)   - Telehealth   - Academic research settings   Information on the effects of various settings was included, where reported. | - Reviews exclusively in settings not relevant to primary care including workplaces, schools, inpatient settings, and medical specialist settings - Reviews in which >50% of included studies took place in countries “high”, “medium”, or “low” on the Human Development Index <http://hdr.undp.org/en/composite/HDI> |
| Study design | Systematic^9^ reviews  Overviews^10^ of systematic^8^ reviews that include a network meta-analysis | - Primary studies - Editorials - Commentaries |
| Language | - English - French |  |
| Dates of publications | 2008 – 2020 |  |

1 In this context, primary care practitioners refer to the provider of first contact for the delivery or referral to stop smoking interventions. This could include physicians, nurses, pharmacists, oral health professionals, counsellors, etc.

2 Reviews solely examining specialized behavioural counselling interventions will be excluded, as the target audience for this guideline is primary care. These interventions require specialized training, the amount of which has been shown to vary but can be substantial [Hollands et al., 2019](https://www.cochranelibrary.com/cdsr/doi/10.1002/14651858.CD009164.pub3/full) and may not be readily available for many primary care practitioners.

3 We define ‘Self-help interventions’ to include “any manual or programme to be used by individuals to assist a quit attempt not aided by health professionals, counsellors or group support” as per the definition in [Hartmann-Boyce et al., 2014](http://onlinelibrary.wiley.com/doi/10.1002/14651858.CD001118.pub3/full) . This differs from interventions that utilize computers, the web, or mobile phones to deliver interventions that involve counselling/support, although the platform of delivery may be the same.

4 Certain products are relevant for inclusion despite not being approved for use as smoking cessation aids by Health Canada, due to their ease of access. These include St. John’s Wort (sold in various forms in pharmacies and health stores across Canada), cytisine and S-adenosylmethionine (licensed natural health products).

5 Patches, gums, mists/sprays, and inhalers are the available forms of NRT in Canada

6 The practice of using e-cigarettes (‘vaping’; including e-cigarettes with nicotine) is increasingly popular, with use being higher among tobacco smokers [Vaping in Canada](https://www.canada.ca/en/health-canada/services/smoking-tobacco/surveys-statistics-research/vaping-what-we-know.html#a1) . Data from the CDC suggest that it was the most commonly used method to quit smoking in 2014-2016 after simply giving up cigarettes all at once or gradually cutting back [CTNS, 2022](https://www.canada.ca/en/health-canada/services/canadian-tobacco-nicotine-survey/2022-summary.html) . The massive interest in these products from the public and tobacco smokers, as well as the evolving evidence base surrounding them make them essential to include.

7 Although initially rated as being of limited importance by the WG, based on discussions with WG members it was decided that this outcome should be considered as important. Clinical experts and patients rated this outcome as important.

8 Reviews will be considered systematic if they meet the four following criteria: (1) searches at least one database; (2) reports their selection criteria; (3) conducts quality or risk of bias assessment on included studies; and (4) provides a list and synthesis of included studies.

9 Overviews will included if they meet the following criteria: (1) search at least one database; (2) report their selection criteria and how they will handling the inclusion of overlapping reviews; (3) provide information on the quality or risk of bias assessment of studies included in reviews; (4) provide a list of relevant reviews; (5) report the synthesized evidence from the included reviews; and (6) explicit declaration that the decision to undertake the network meta-analysis was made with firsthand knowledge of the primary studies, to ensure appropriateness of the analysis.
