# Supplementary material for: Effectiveness of smoking cessation interventions among adults: an overview of systematic reviews
Source: Syst Rev. 2024 Jul 12;13:179. doi: 10.1186/s13643-024-02570-9 (PMC11242003; doi:10.1186/s13643-024-02570-9)
Supplement: Supplementary file 7 — Additional file 7. Reasons for exclusion at full-text and post-hoc exclusions. [file 13643_2024_2570_MOESM7_ESM.doc]

### Additional file 7. Reasons for exclusion at full-text and post-hoc exclusions

## Full-text not available (n=164)

1. RefID: 2060. 11th Annual Canadian Respiratory Conference. 2018 Canadian Respiratory Conference Abstracts. Canadian Journal of Respiratory Critical Care and Sleep Medicine 2018;2(2).
2. RefID: 2781. Alarcon JA, Arrazola X, Lavado M, Perez A, Rocandio M, Reparaz J, Madruga F, Barrera J, San VJ, De La Cuesta P. Efficacy of tobacco cessation program in a cardiac rehabilitation unit. Eur J Prev Cardiolog 2014;21(1 SUPPL. 1):S70
3. RefID: 2486. Altman D, Clement F, Barnieh L, Manns B, Penz E. Cost-effectiveness of universally funding smoking cessation pharmacotherapy. Chest 2016;150(4 Supplement 1):1303A
4. RefID: 3064. American Association for the Advancement of Science. Patient Reported Outcomes and Pearson Centered Care in Mental Health. J Mental Health Policy Econ 2012;15(SUPPL. 1).
5. RefID: 3834. Andalusian Agency for Health Technology Assessment. Programs for smoking cessation - non-systematic review (Structured abstract). Health Technology Assessment Database 2016;(2016 Issue 4).
6. RefID: 2356. Aziz N, Rahman A. Lifestyle health behaviour interventions among long-term cancer survivors. Psycho-Oncology 2017;26(Supplement 2):26-7.
7. RefID: 3827. Basque Office for Health Technology Assessment, Health Department. Evaluation of the impact of different interventions in smoking cessation in the Basque Country and comparison between primary and specialised care (Project record). Health Technology Assessment Database 2016;(2016 Issue 4).
8. RefID: 2786. Bhatia SC, Ramaswamy S, Kolli V. Neurobiology of aging and dementias. Indian J Psychiatry 2014;56(SUPPL. 1):S17
9. RefID: 3018. Bogenschutz MP. Effects of psilocybin in the treatment of addictions: A review and preliminary results from two ongoing trials. Neuropsychopharmacology 2012;38(SUPPL. 1):S15-S16
10. RefID: 3822. Broman G, Bahtsevani C, Berg T, Eliasson M, Agens I. Treating asthma and COPD - a systematic review (Structured abstract). Health Technology Assessment Database 2016;(2016 Issue 4).
11. RefID: 3164. Bryg R, Bryg DJ. Do interventions in acute myocardial infarction improve outcome past 30 days? Circ Cardiovasc Qual Outcomes 2011;4(6 MeetingAbstracts2011).
12. RefID: 3763. Buiatti E. Intervention trials of cancer prevention: results and new research programmes (Structured abstract). Database of Abstracts of Reviews of Effects 2015;(2).
13. RefID: 2536. Burke LE, Ma J, Azar KMJ, Bennett GG, Peterson ED, Zheng Y, Riley W, Stephens J, Shah SH, Suffoletto B, Turan TN, Spring B, Steinberger J, Quinn CC. Current Science on Consumer Use of Mobile Health for Cardiovascular Disease Prevention: A Scientific Statement from the American Heart Association. Circulation 2015;132(12):1157-213.
14. RefID: 2415. Carlson NS. From cochrane database of systematic reviews (CDSR), issue 12 (2015) and issue 1 (2016). J Midwifery Women's Health 2016;61(3):384-6.
15. RefID: 3832. Catalan Agency for Health Technology Assessment and Research. Tobacco control: a review of the strategies (Structured abstract). Health Technology Assessment Database 2016;(2016 Issue 4).
16. RefID: 2353. Chen L-S. Can we clear the smoke? from genes to the bedside. J Neuroimmune Pharmacol 2017;12(2 Supplement 1):S93
17. RefID: 3819. Cohen D, Eliasson M, Eriksson C, Gilljam H, Hedin A, Hellnius ML, Hjalmarson A, Nilsson P, Tillgren P. Smoking cessation methods (Structured abstract). Health Technology Assessment Database 2016;(2016 Issue 4).
18. RefID: 2055. Cummings KM. PRO- Electronic Cigarettes: A Cessation Tool. J Thorac Oncol 2018;13(10 Supplement):S270-S271
19. RefID: 3823. Danish Centre for Evaluation and Health Technology Assessment (DACEHTA). Smoking cessation in patients with chronic obstructive lung disease admitted to medical department, Copenhagen County Hospital, Glostrup (co-funded by DIHTA) (Project record). Health Technology Assessment Database 2016;(2016 Issue 4).
20. RefID: 3839. Danish Institute for Health Services Research. Smoking cessation interventions in pharmacies. Demand from smokers and the ability of general practitioners to recruit participants (Structured abstract). Health Technology Assessment Database 2016;(2016 Issue 4).
21. RefID: 2653. Dawood B, Mulder J, Reynald L. An audit of smoking prevalence and cessation amongst patients admitted to the respiratory unit in peninsula health. Respirology 2015;20(SUPPL. 2):84
22. RefID: 2481. Dawson KL. Overview of pharmacological interventions for adolescent tobacco use disorder. J Am Acad Child Adolesc Psychiatry 2016;55(10 Supplement 1):S20
23. RefID: 3864. Department of Science and Technology - Brazilian Health Technology Assessment General Coordination (DECIT-CGATS), National Health Surveillance Agency (ANVISA) and National Supplementary Health Agency (ANS). BRATS 12: Clinical strategies for smoking cessation (Structured abstract). Health Technology Assessment Database 2016;(2016 Issue 4).
24. RefID: 3798. Donath C, Metz K, Kroger C. Do smoking bans really help: the effect of tobacco-control policy on patients in substance-abuse treatment (Provisional abstract). Database of Abstracts of Reviews of Effects 2015;(2).
25. RefID: 3169. Edjoc RK, Reid RD, Sharma M. Smoking cessation in cerebrovascular disease: A systematic review on the effects of smoking cessation on stroke recurrence, morbidity and mortality in patients with cerebrovascular disease. Stroke 2011;42(11):e595
26. RefID: 1058. Erwin BL, Slaton RM. Varenicline in the treatment of alcohol use disorders. Ann Pharmacother 2014;48(11):1445-55.
27. RefID: 3848. Fayter D, Main C, Misso K, Ogilvie D, Petticrew M, Sowden A, Stirk L, Thomas S, Whitehead M, Worthy G. Population tobacco control interventions and their effects on social inequalities in smoking (Structured abstract). Health Technology Assessment Database 2016;(2016 Issue 4).
28. RefID: 2487. Feemster L. E-cigarettes update. Respirology 2016;21(Supplement 3):7
29. RefID: 3792. Fichtenberg C, Glantz S. Effect of smoke-free workplaces on smoking behavior: systematic review (Structured abstract). Database of Abstracts of Reviews of Effects 2015;(2).
30. RefID: 2793. Filion KB, Dell'Aniello S, Eberg M, Renoux C, Daskalopoulou SS, Suissa S. Varenicline and the risk of adverse cardiovascular events: A population-based cohort study. Circulation 2014;129(SUPPL. 1).
31. RefID: 2779. Filion KB, Eberg M, Azoulay L. The impact of the publication of cardiovascular safety signals on the prescribing of varenicline: An interrupted time-series analysis. Circulation 2014;130(SUPPL. 2).
32. RefID: 3706. France E, Glasgow R, Marcus A. Smoking cessation interventions among hospitalized patients: what have we learned? (Structured abstract). Database of Abstracts of Reviews of Effects 2015;(2).
33. RefID: 2125. Galiatsatos P, Koehl R, Caufield-Noll C, Brigham E, Leone F, Hansel N, Rand C, McCormack M. Proposal for smoke-free public housing: A review of attitudes and preferences from residents of multi-unit housing. Am J Respir Crit Care Med 2018;197(MeetingAbstracts).
34. RefID: 2338. Galling B, Correll CU. Antidepressant augmentation of antipsychotics in schizophrenia: A systematic review, meta-analysis and metaregression analysis. Eur Neuropsychopharmacol 2017;27(Supplement 4):S946-S947
35. RefID: 3239. Gehricke J. Self-medicating with tobacco in individuals with adhd and schizophrenia. Neuropsychopharmacology 2010;35(SUPPL. 1):S41
36. RefID: 2075. Germovsek E, Hansson A, Kjellsson MC, Ruixo JJP, Westin A, Soons PA, Vermeulen A, Karlsson MO. An exposure-response (ER) model relating nicotine plasma concentration to momentary craving across different nicotine replacement therapy (NRT) formulations. J Pharmacokinet Pharmacodyn 2018;45(Supplement 1):S28-S29
37. RefID: 2341. Ghaffari DM, Khorasani E, Fatehi Z. Smoking cessation interv entions based on social marketing principles: A review. Value in Health 2017;20(9):A652
38. RefID: 2124. Golden S, Ono S, Wiener RS, Melzer AC, Davis J, Slatore CG. Patient and clinician perspectives on smoking cessation in three lung cancer screening programs. Am J Respir Crit Care Med 2018;197(MeetingAbstracts).
39. RefID: 3831. Gorgojo Jimenez L, Gonzalez Enriquez J, Salvador Llivina T. Efficacy, effectiveness and cost-effectiveness of interventions for smoking cessation IPE-03/40 (Public report) (Structured abstract). Health Technology Assessment Database 2016;(2016 Issue 4).
40. RefID: 2196. Gould GS, Bar-Zeev Y, Twyman T, Stevenson L, Palazzi K, Bonevski B. Health providers' performance of the 5as for smoking cessation care during pregnancy: A systematic review and meta-analysis. J Paediatr Child Health 2018;54(Supplement 1):78
41. RefID: 2776. Grills N, Shrivastav R, Singh D, Arora M. Impact of plain packaging in Australia: Lessons for India. Asia-Pac J Clin Oncol 2014;10(SUPPL. 9):14
42. RefID: 2778. Hahn B, Harvey A, Fischer B, Keller W, Ross T, Stein E. Nicotinic modulation of the default network of resting brain function in non-smokers. Neuropsychopharmacology 2014;39(SUPPL. 1):S255
43. RefID: 3896. Health Technology Assessment. A randomised controlled trial to examine the efficacy of e-cigarettes compared with nicotine replacement therapy, when used within the UK stop smoking service (Project record). Health Technology Assessment Database 2016;(2016 Issue 4).
44. RefID: 3897. Health Technology Assessment. What is the effectiveness of varenicline compared with nicotine replacement therapy for long term smoking cessation and clinically important outcomes such as mortality, myocardial infarction and frequency of service use? Evidence from the Clinical Practice Research Datalink (Project record). Health Technology Assessment Database 2016;(2016 Issue 4).
45. RefID: 2505. Henao Nieto DE, Gomez F. Multi-modal intervention to enhance the implementation of evidence-based tobacco use cessation treatments in non-physician healthcare workers in rural zones of Colombia. Glo Heart 2016;11(2 SUPPL. 1):e162
46. RefID: 3296. Heredia I, Valencia A, Bertozzi S. Cost-effectiveness of pharmacological interventions for smoking cessation: Current evidence and policy implications. Value in Health 2009;12(7):A526
47. RefID: 2123. Hirota JA, Tamminga A, Aguiar JA, Lobb B, Dvorkin-Gheva A, Doxey AC. ATP binding cassette (ABC) transporter expression in human airway epithelial cells: A meta-analysis of gene expression from bronchial brushings. Am J Respir Crit Care Med 2018;197(MeetingAbstracts).
48. RefID: 2916. Hoddinott P, Morgan H, Thomson G, Crossland N, Craig L, Britten J, Farrar S, Newlands R, Kiezebrink K, Coyle J. Intervention vignettes as a qualitative tool to refine complex intervention design. Trials 2013;14(SUPPL. 1):58DUMMY
49. RefID: 3735. Hughes J, Stead L, Lancaster T. Nortriptyline for smoking cessation: a review (Structured abstract). Database of Abstracts of Reviews of Effects 2015;(2).
50. RefID: 2792. Inglis SC, Du H, Newton PJ, DiGiacomo M, Omari A, Davidson PM. Disease management interventions for improving self-management in lower-limb peripheral arterial disease: A cochrane review. Glo Heart 2014;9(1 SUPPL. 1):e262
51. RefID: 2472. Johns DA. Randomised controlled trial comparing nicotine replacement therapy (NRT) plus brief counselling and brief counselling alone on smoking cessation in patients prone to lung cancer using a breath carbon monoxide (CO) monitor. Ann Oncol 2016;27(Supplement 9).
52. RefID: 2640. Lasiter S, Oles SK, Mundell J, London S, Khan BA. Critical care follow-up clinics: A systematic review. Am J Respir Crit Care Med 2015;191(MeetingAbstracts).
53. RefID: 3253. Le FAL. What works for smoking cessation? Cardiovasc Intervent Radiol 2010;33(SUPPL. 2):153-4.
54. RefID: 2782. Lee SY, Said N, See KC. Brief versus intensive smoking cessation counselling: Should patients be given a choice? Respirology 2014;19(SUPPL. 3):174
55. RefID: 795. Leuthard JL, Beebe LA, Halstead L, Olson KD, Roysdon JW. Increased Evidence-Based Tobacco Treatment Through Oklahoma Hospital System Changes. J Okla State Med Assoc 2015;108(11):471-6.
56. RefID: 3177. Manninen H. Endovascular is the first choice for long SFA occlusion in claudication: Con. Cardiovasc Intervent Radiol 2011;34(SUPPL. 3):381-2.
57. RefID: 2476. Matharoo J, Arshad A, Sadhra S, Norton-Wangford R, Jawad M. How does knowledge, perceptions and attitudes towards shisha pipe smoking vary amongst university students? Thorax 2016;71(Supplement 3):A74
58. RefID: 3289. Mead A, Jennings C, Jones J, Turner EL, Connolly SB, Kotseva K, De BG, De BD, Frost G, Wood D. Less weight gain and a healthier lifestyle after quitting smoking in EUROACTION: A family based preventive cardiology programme for coronary patients. Eur Heart J 2009;30(SUPPL. 1):77
59. RefID: 293. Merry S, Bullen CR. E-cigarette use in New Zealand-a systematic review and narrative synthesis. N Z Med J 2018;131(1470):37-50.
60. RefID: 3602. Momin B, Neri A, McCausland K, Duke J, Hansen H, Kahende J, Zhang L, Stewart S. Traditional and innovative promotional strategies of tobacco cessation services: a review of the literature (Provisional abstract). Database of Abstracts of Reviews of Effects 2015;(2).
61. RefID: 2084. Murphy RP. Review of reviews on the effectiveness of workplace wellbeing programmes. Occup Environ Med 2018;75(Supplement 2):A214
62. RefID: 3295. Narvaez J, Alvis N, De La Hoz F, Orozco J, Porras A. An economic evaluation of a pharmacological intervention using varenicline as therapy for smoking cessation. Value in Health 2009;12(3):A123
63. RefID: 3824. National Institute for Clinical Excellence. Guidance on the use of nicotine replacement therapy (NRT) and bupropion for smoking cessation (Structured abstract). Health Technology Assessment Database 2016;(2016 Issue 4).
64. RefID: 2939. Nduka C, Ngianga-Bakwin K, Suri G, Stranges S. Risk factor interventions are effective for preventing cardiovascular events in hiv-infected patients on antiretroviral therapy: A systematic review and meta-analysis. Circulation 2013;127(12 Meeting Abstracts).
65. RefID: 3821. NHS Centre for Reviews and Dissemination. Smoking cessation: what the health service can do (Structured abstract). Health Technology Assessment Database 2016;(2016 Issue 4).
66. RefID: 3833. NHSC. Rimonabant for smoking cessation, weight loss and cardiovascular risk factors of overweight/obesity - horizon scanning review (Structured abstract). Health Technology Assessment Database 2016;(2016 Issue 4).
67. RefID: 3509. No ai. Selected abstracts. American Journal of Health Promotion 2009;24(1):TAHP11-TAHP12
68. RefID: 2630. Ostroff J. Smoking cessation integrated with screening (window of opportunity). J Thorac Oncol 2015;10(9 SUPPL. 2):S158-S159
69. RefID: 2133. Paraschakis A, Goulas K. Profile of suicide victims in non-psychiatric units of general hospitals. Eur Psychiatry 2018;48(Supplement 1):S261
70. RefID: 2361. Perez G. Smoking cessation program needs among Black and Hispanic cancer patients. Psycho-Oncology 2017;26(Supplement 1):25
71. RefID: 2508. Phelan M, Eikhoff N, Chamberlin J, Hustey F, Meldon S. Frequency of smoking cessation counseling and smoking cessation discharge instructions in the emergency department. Acad Emerg Med 2016;23(SUPPL. 1):S200
72. RefID: 3589. Pointer L, Cerahill C, Freeman S, Johnson K, Hester E, Kozak K, Schilling L, Dellavalle R. Tobacco smoking cessation for treating acne. cdsr 2011;(6).
73. RefID: 2094. Poudel N, Kavookjian J. Motivational interviewing as a strategy for smoking cessation among adolescents-a systematic review. Value in Health 2018;21(Supplement 1):S238-S239
74. RefID: 2644. Pratt-Chapman M, Hatcher E, Brooks J, Davies M. Tailoring tobacco cessation interventions for special populations served by federally qualified health centers. Psycho-Oncology 2015;24(SUPPL. 2):50
75. RefID: 2923. Pratt A, Su L, Audera-Lopez C, Rarick J, Mercado S. The rise of e-cigarettes: An emerging threat to the tobacco endgame? Respir Med 2013;107(SUPPL. 1):S9
76. RefID: 2270. Rankin N, Weber M, Luo Q, Wade S, Hughes S, Fong K, Canfell K. Lung cancer pathways: A five-year program to reduce impact through epidemiological modelling and investment in prioritized interventions. J Thorac Oncol 2017;12(11 Supplement 2):S2297
77. RefID: 2350. Rankin NM, Slatore CG, Stone EC. Implementation science and challenges in lung cancer screening: A critical review of the literature. Am J Respir Crit Care Med 2017;195.
78. RefID: 3849. Rasch A, Mueller-Riemenschneider F, Vauth C, Willich SN, Greiner W. Federal structures and associated behavioural interventions in prevention of cigarette smoking (Structured abstract). Health Technology Assessment Database 2016;(2016 Issue 4).
79. RefID: 796. Rhoades RR, Beebe LA. Tobacco Control and Prevention in Oklahoma: Best Practices in a Preemptive State. J Okla State Med Assoc 2015;108(11):434-40.
80. RefID: 3604. Rice N, Godfrey C, Slack R, Sowden A, Worthy G. A systematic review of the effects of price on the smoking behaviour of young people (Structured abstract). Database of Abstracts of Reviews of Effects 2015;(2).
81. RefID: 2339. Richards AK, Jackson N, Cheng EM, Towfighi A, Byrg R, Brown A, Sanossian N, Barry F, Na L, Vickrey B. Derivation and application of a quantitative approach to estimate global stroke risk reduction for multi-faceted interventions to prevent recurrent stroke. Ann Neurol 2017;82(Supplement 21):S175
82. RefID: 2921. Rigotti NA. Smoking cessation. J Thorac Oncol 2013;8(SUPPL. 2):S95
83. RefID: 2504. Rizwani W, Jyothi D, Perumal D, Eslavath R, Singh SS. E-cigarettes upregulate alpha-enolase in cervical cancer cells, similar to conventional cigarettes, potentially promoting tumorigenesis. Eur J Cancer 2016;54(SUPPL. 1):S68
84. RefID: 2479. Rodrigues C, Andrade M, Bettencourt M, Palma V, Cardoso C. Varenicline suicidal ideation in psychiatric patients-a case report and systematic review. Eur Neuropsychopharmacol 2016;26(Supplement 2):S710
85. RefID: 3814. San L, Arranz B, Martinez-Raga J. Antipsychotic drug treatment of schizophrenic patients with substance abuse disorders (Provisional abstract). Database of Abstracts of Reviews of Effects 2015;(2).
86. RefID: 2056. Sarna L. Enhancing the Nurse's Role in Tobacco Prevention and Cessation: New Challenges. J Thorac Oncol 2018;13(10 Supplement):S214
87. RefID: 3505. Sarna L, Bialous SA. Annual review of nursing research, 2009: Advancing nursing science in tobacco control. Annual review of nursing research, 2009;
88. RefID: 2648. Scholl J, Kurz P. Long term benefits of a contemporary health check-up with evidence-based risk communication and motivational coaching-the PF study. Eur J Prev Cardiolog 2015;22(1 SUPPL. 1):S38
89. RefID: 2172. Singh N, Dornadula I, Ramezani A, Sheth S, De MC. Psychological preparation for neuromodulation: A systematic review. Neuromodulation 2018;21(3):e72
90. RefID: 2480. Sparks G. Behavioral interventions for tobacco cessation. J Am Acad Child Adolesc Psychiatry 2016;55(10 Supplement 1):S20-S21
91. RefID: 1026. Sullivan DH, Rees MA. Smoking bans in secure psychiatric hospitals and prisons. J Law Med 2014;22(1):22-30.
92. RefID: 3825. Swedish Council on Technology Assessment in Health Care. Bupropion (Zyban) in smoking cessation - early assessment briefs (Alert) (Structured abstract). Health Technology Assessment Database 2016;(2016 Issue 4).
93. RefID: 2070. Tam L-S. 2018 APLAR axial spondyloarthritis treatment recommendations. Int J Rheum Dis 2018;21(Supplement 1):20
94. RefID: 3837. The Netherlands Organisation for Health Research and Development (ZonMw). Implementation of an evidence based smoking cessation strategy (SMOCC) for patients with COPD in primary care (Project record). Health Technology Assessment Database 2016;(2016 Issue 4).
95. RefID: 2788. Theocharidis V, Economopoulos KP. Smoking cessation prior to elective plastic surgery: Why, when and how? Tob induc dis 2014;12(SUPPL. 1).
96. RefID: 2345. Treciokiene I, Postma MJ, Nuygen T, Fens T, Petkevicius J, Taxis K. A systematic review on lifestyle change interventions performed by health care professionals targeting blood pressure in hypertensive patients. Value in Health 2017;20(9):A603
97. RefID: 2627. Van Osch FHM, Jochems SHJ, Van SFJ, Bryan RT, Zeegers MPA. Urothelial cell carcinoma incidence, lifetime smoking behaviour and exposure to environmental tobacco smoke: A meta-analysis. Eur J Epidemiol 2015;30(8):888-9.
98. RefID: 2915. Van NK, Calvo-Ayala E, Khan BA. Smoking cessation interventions in critical care survivors-a systematic evidence review. Am J Respir Crit Care Med 2013;187(MeetingAbstracts).
99. RefID: 3851. Vassey J, Mitchell MD. Smoking cessation for inpatients (Structured abstract). Health Technology Assessment Database 2016;(2016 Issue 4).
100. RefID: 2932. Vatsalya V, Momenan R, Schwandt ML, Coe M, Bartlett S, Hommer DW, Heilig M, Ramchandani VA. Effects of varenicline on neural correlates of motivation for alcohol in heavy drinkers: The alcoholfood incentive delay task. Neuropsychopharmacology 2013;38(SUPPL. 2):S512
101. RefID: 2097. Wakhlu S, Chapman L, Dela Cruz AM. Electronic cigarettes: Review of safety and use. Am J Addict 2018;27(4):307
102. RefID: 119. Walker SP, Bibby AC, Halford P, Stadon L, White P, Maskell NA. Recurrence rates in primary spontaneous pneumothorax: a systematic review and meta-analysis. Eur Respir J 2018;52(3).
103. RefID: 2926. Warren GW, Marshall JR, Cummings KM. Smoking, cancer treatment, and design of clinical trials. Cancer Res 2013;73(8 SUPPL. 1).
104. RefID: 2358. Watsula-Morley A, Gillespie C, Altshuler L, Hanley K, Kalet A, Porter B, Wallach AB, Zabar S. Putting out the flame: Our trainees need to learn patient activation skills. J Gen Intern Med 2017;32(2 Supplement 1):S294-S295
105. RefID: 3065. Whelton SP, Nasir K, Blaha MJ, Berman DS, Blumenthal RS. Effect of non-invasive imaging on cardiovascular risk factors and downstream testing: A meta-analysis of randomized controlled trials. Circulation 2012;125(10 SUPPL. 1).
106. White MA, O'Malley SS. Interactions between smoking, eating, and body weight. In: Brownell K, Gold M, editors. Food and addiction: A comprehensive handbook, New York, NY, US: Oxford University Press; 2012. p. 244-8.
107. RefID: 2933. Windle SB, Bata I, Madan M, Abramson BL, Dehghani P, Sharma S, Roy N, Lauzon C, Grondin FR, Old W, Clarke A, Eisenberg MJ. Incidence of cardiovascular events with varenicline for smoking cessation post-acute coronary syndrome. Can J Cardiol 2013;29(10 SUPPL. 1):S278-S279
108. RefID: 2093. Winzenberg T. Is bone density testing an under-utilised educational tool in youngerwomen? Osteoporosis Int 2018;29(1 Supplement 1):S125
109. RefID: 2216. Yablonsky PK, Sukhovskaya OA. Smoking influence on the outcomes and complications of coronary bypass surgery. Russ J Cardiol 2018;153(1):66-71.
110. RefID: 2503. Agarwal S. Yoga and stroke: An evidence based review. J Altern Complement Med 2016;22(6):A68
111. RefID: 2641. Allehebi RO, Khan M, Stanbrook MB. Efficacy and safety of electronic cigarettes for smoking cessation: A systematic review. Am J Respir Crit Care Med 2015;191(MeetingAbstracts).
112. RefID: 720. Augustine JM, Taylor AM, Pelger M, Schiefer D, Warholak TL. Smoking quit rates among patients receiving pharmacist-provided pharmacotherapy and telephonic smoking cessation counseling. J Am Pharm Assoc (2003) 2016;56(2):129-36.
113. RefID: 1990. BMJ Group. Varenicline for smoking cessation. Drug Ther Bull 2008;46(5):33-6.
114. Brandon TH, Unrod M. Tobacco use and cessation. New York, NY, US: Oxford University Press; 2010.
115. RefID: 336. Camenga DR, Tindle HA. Weighing the Risks and Benefits of Electronic Cigarette Use in High-Risk Populations. Med Clin North Am 2018;102(4):765-79.
116. RefID: 2656. Carson K, To AN, Robertson M, King C, Smith B. Community pharmacy personnel interventions for smoking cessation: A cochrane systematic review and meta-analysis. Respirology 2015;20(SUPPL. 2):16
117. RefID: 2917. Chan GR, Bagazin P, Bautista M, Gison CB. Effects of smoking cessation in asthmatic patients: A systematic review. Pharmacotherapy 2013;33(10):e293
118. RefID: 2126. Charron CB, Hayes T, Pakhale S. A systematic review of interventions for smoking tobacco in low socio-economic populations. Am J Respir Crit Care Med 2018;197(MeetingAbstracts).
119. RefID: 2171. Cooper V, Clatworthy J, Whetham J. Mobile health (mHealth) interventions to support self-management in HIV: A systematic review. HIV Med 2018;19(Supplement 2):S105
120. RefID: 776. Drovandi AD, Chen CC, Glass BD. Adverse Effects Cause Varenicline Discontinuation: A Meta-Analysis. Curr Drug Saf 2016;11(1):78-85.
121. RefID: 3784. Edwards N. The effectiveness of postpartum smoking relapse prevention strategies (Provisional abstract). Database of Abstracts of Reviews of Effects 2015;(2).
122. RefID: 3718. Ferron J, Alterman A, McHugo G, Brunette M, Drake R. A review of research on smoking cessation interventions for adults with schizophrenia spectrum disorders (Structured abstract). Database of Abstracts of Reviews of Effects 2015;(2).
123. RefID: 2626. Foley NC, Lindsay P. The use of electronic cigarettes is not associated with cessation of smoking: A systematic review and meta-analysis. Int J Stroke 2015;10(SUPPL. 4):64-5.
124. RefID: 3692. Gervaise A. A systematic review of randomized controlled trials of youth smoking cessation interventions (Structured abstract). Database of Abstracts of Reviews of Effects 2015;(2).
125. RefID: 3723. Gourlay S. The pros and cons of transdermal nicotine therapy (Structured abstract). Database of Abstracts of Reviews of Effects 2015;(2).
126. RefID: 2076. Hamee RH. Human health effects of electronic cigarettes: A review. Indian J Public Health Res Dev 2018;9(8):1387-91.
127. RefID: 3650. Harrell P. Electronic nicotine delivery systems ('e-cigarettes'): review of safety and smoking cessation efficacy (Provisional abstract). Database of Abstracts of Reviews of Effects 2015;(2).
128. RefID: 3875. HAYES, Inc. Nicotine replacement therapy for smoking cessation: nicotine patch (Structured abstract). Health Technology Assessment Database 2016;(2016 Issue 4).
129. RefID: 3877. HAYES, Inc. Bupropion for smoking cessation (Structured abstract). Health Technology Assessment Database 2016;(2016 Issue 4).
130. RefID: 3873. HAYES, Inc. Nicotine replacement therapy for smoking cessation: nicotine gum and nicotine lozenge (Structured abstract). Health Technology Assessment Database 2016;(2016 Issue 4).
131. RefID: 3872. HAYES, Inc. Chantix (Varenicline Tartrate) (Pfizer Inc.) for smoking cessation (Structured abstract). Health Technology Assessment Database 2016;(2016 Issue 4).
132. RefID: 3891. HAYES, Inc. Smoking cessation in patients with schizophrenia (Structured abstract). Health Technology Assessment Database 2016;(2016 Issue 4).
133. RefID: 3874. HAYES, Inc. Nicotine replacement therapy for smoking cessation: nicotine nasal spray and nicotine inhaler (Structured abstract). Health Technology Assessment Database 2016;(2016 Issue 4).
134. RefID: 3829. Hermes-DeSantis E. Smoking cessation therapies (Structured abstract). Health Technology Assessment Database 2016;(2016 Issue 4).
135. RefID: 2654. Jayasinghe H, Carson K, Ali A, Singh K, Peters M, Esterman A, Veale A, Newchurch J, Smith B. Community interventions for preventing smoking in young people: A cochrane systematic review. Respirology 2015;20(SUPPL. 2):60
136. RefID: 2204. Johns DJ, Langley TE, Lewis S. Use of social media for the delivery of health promotion on smoking, nutrition, and physical activity: A systematic review. The Lancet 2017;390(SPEC.ISS 1):S49
137. RefID: 2363. Katangwe T, Bhattacharya D, Twigg MJ. A systematic review of diet and lifestyle interventions in newly diagnosed, medication nayve patients with type 2 diabetes. Int J Pharm Pract 2017;25(Supplement 1):62-3.
138. RefID: 3681. Kerr J. Varenicline: a novel nicotinic receptor partial agonist for smoking cessation (Provisional abstract). Database of Abstracts of Reviews of Effects 2015;(2).
139. RefID: 2636. Kohli IS. Effects of clinical computing systems during consultations on smoking cessation-evidence landscape from randomized and quasi randomized trials. Value in Health 2015;18(7):A507
140. RefID: 2343. Lee B, Lee T, Jang S, Lee Y, Choi N. The effect of smoking cessation interventions in patients with COPD: A systematic review. Value in Health 2017;20(9):A652
141. RefID: 3615. Likis F. Smoking cessation interventions in pregnancy and postpartum care (Provisional abstract). Database of Abstracts of Reviews of Effects 2015;(2).
142. RefID: 2497. Marques FM, Costa L, Duarte D, Moniz A. Smoking cessation in elderly: Is there a better way? Eur Geriatr Med 2016;7(Supplement 1):S183-S184
143. RefID: 2057. Mayel M. Are vapes an effective device for smoking cessation or a gateway to conventional tobacco smoking? Can J Resp Ther 2018;54(2):55.
144. RefID: 3682. McRobbie H. Using nicotine replacement therapy to assist in reducing cigarette consumption before quitting: another strategy for smoking cessation? (Structured abstract). Database of Abstracts of Reviews of Effects 2015;(2).
145. RefID: 3852. Mitchell MD, Leone F, Williams K. Varenicline for smoking cessation (Structured abstract). Health Technology Assessment Database 2016;(2016 Issue 4).
146. RefID: 3757. Mullen P. Maternal smoking during pregnancy and evidence based intervention to promote cessation (Structured abstract). Database of Abstracts of Reviews of Effects 2015;(2).
147. National Institute for Health and Care Excellence. Smoking: Harm reduction [Internet]. Available at: nice.org.uk/guidance/ph45. Last Accessed: 3-2-2020.
148. RefID: 2988. No authors listed. Varenicline: Long-term cardiovascular events. Prescrire Int 2012;21(123):15.
149. RefID: 798. No authors listed. Cardiac adverse effects of nicotine replacement therapy. Prescrire Int 2015;24(166):292-3.
150. RefID: 3755. Okoli C. Men's smoking cessation interventions: a brief review (Structured abstract). Database of Abstracts of Reviews of Effects 2015;(2).
151. RefID: 2344. OMurchu E, Cullinane F, Moran P, Harrington P, Ryan M. Clinical effectiveness of smoking cessation therapy during pregnancy. Value in Health 2017;20(9):A640
152. RefID: 3074. Prochaska JJ, Burtner JL, Delucchi K, Hall SM. Tobacco treatment effects on long-term abstinence from alcohol and illicit drugs: A meta-analysis. Alcohol Clin Exp Res 2012;36(SUPPL. 1):72A
153. RefID: 2275. Rani LD. Systematic review of RCT's in mhealth interventions focusing on maternal and child health. Indian J Public Health Res Dev 2017;8(4):463-7.
154. RefID: 3801. Ritvo P, Irvine M, Lindsay E, Kraetschmer N, Blair N, Schnek Z. A critical review of research related to family physician-assisted smoking cessation interventions (Structured abstract). Database of Abstracts of Reviews of Effects 2015;(2).
155. RefID: 306. Rosembaun A, Rojas P, Rodriguez MV, Barticevic N, Rivera Mercado S. Brief interventions to promote behavioral change in primary care settings, a review of their effectiveness for smoking, alcohol and physical inactivity. Medwave 2018;18(1):e7148
156. RefID: 2937. Shi H, Yang X, Huang C, Liu Z, Xu X, Lin C. Effectiveness of smoking cessation methods among chinese smokers: A systematic reviewand meta-analysis of randomized controlled trials. Am J Epidemiol 2013;177(SUPPL. 11):S34
157. RefID: 660. Siddiqui F, Huque R, Dogar O. Updated evidence-based guide to smoking cessation therapies. Br J Community Nurs 2016;21(12):607-11.
158. RefID: 2362. Strauss N, Rabin J, Temel B, Philpotts L, Ostroff J, Park ER, Perez GK. Smoking cessation interventions for Black and Hispanic cancer patients: A systematic review of tobacco trials. Psycho-Oncology 2017;26(Supplement 1):87
159. RefID: 3657. Sussman S. Effects of sixty six adolescent tobacco use cessation trials and seventeen prospective studies of self-initiated quitting (Provisional abstract). Database of Abstracts of Reviews of Effects 2015;(2).
160. RefID: 3835. Van denBruel A, Cleemput I, VanLinden A, Schoefs D, Ramaekers D, Bonneux L. Effectiveness and cost-effectiveness of treatments for smoking cessation (Structured abstract). Health Technology Assessment Database 2016;(2016 Issue 4).
161. RefID: 3298. Walczak J, Nogas G, Czarny I, Baginski P, Borowiack M. Systematic review of the efficacy and safety of varenicline for smoking cessation compared with placebo, nicotine replacement therapy or sustained-release bupropion. Value in Health 2009;12(7):A298
162. RefID: 2635. Wang X, Huang Y. Systematic review of nursing interventions of smoking cessation for patients with cardiovascular disease. J Am Coll Cardiol 2015;66(16 SUPPL. 1):C123
163. RefID: 2789. Wu IH, Abughosh S. Smoking cessation pharmacological interventions among schizophrenia smokers-a systematic review. Value in Health 2014;17(3):A170
164. RefID: 2495. Yemchuk SM, Boldt RG, Palma DA, Louie AV. Are we helping cancer patients quit smoking using smoking cessation programs? A systematic review and meta-synthesis of the literature. Radiother Oncol 2016;120(Supplement 1):S92

## Language other than English or French (n=111)

1. RefID: 3516. Aguirre EC, del Rocio Hernandez-Pozo M. Impulsivity assessment in smokers: A systematic review. Revista Mexicana de Analisis de la Conducta 2008;34(2):293-311.
2. RefID: 1270. Alba LH, Murillo R, Castillo JS, Grupo elaborador de guias de cesacion de tabaco del INC. [Counseling interventions for smoking cessation: systematic review]. Salud Publica Mex 2013;55(2):196-206.
3. RefID: 1580. Albornos Munoz L, Sanz Hernanz E. [Evidence based information sheet for consumer. Strategies to help smokers quit]. ENFERM CLIN 2011;21(2):119-21.
4. RefID: 2725. Alessi A, Brandao AA, de Paiva AMG, Nogueira AR, Feitosa A, Gonzaga CC, et al. I Brazilian position paper on prehypertension, white coat hypertension and masked hypertension: Diagnosis and management. Arq Bras Cardiol 2014;102(2):110-8.
5. RefID: 590. Alvarez FV, Trueba IM, Sanchis JB, Lopez-Rodo LM, Rodriguez Suarez PM, de Cos Escuin JS, et al. Recommendations of the Spanish Society of Pneumology and Thoracic Surgery on the diagnosis and treatment of non-small-cell lung cancer. Arch Bronconeumol 2016;52 Suppl 1:2-62.
6. RefID: 1991. Andreas S, Batra A, Behr J, Berck H, Chenot JF, Gillissen A, et al. [Guidelines for smoking cessation in patients with COPD issued by the Deutsche Gesellschaft fur Pneumologie und Beatmungsmedizin]. Pneumologie 2008;62(5):255-72.
7. RefID: 1351. Astrid Becerra N, Alba LH, Castillo JS, Murillo R, Canas A, Garcia-Herreros P. [Alternative therapies for smoking cessation: clinical practice guidelines review]. Gac Med Mex 2012;148(5):457-66.
8. RefID: 669. Aumann I, Rozanski K, Damm K, Graf von der Schulenburg JM. [Cost-Effectiveness of Pharmacological Smoking Cessation Therapies - A Systematic Literature Review]. Gesundheitswesen 2016;78(10):660-71.
9. RefID: 2999. Aveyard P, Lycett D, Farley A. Managing smoking cessation-related weight gain. Pol Arch Med Wewn 2012;122(10):494-8.
10. RefID: 765. Batra A, Petersen KU, Hoch E, Mann K, Kroger C, Schweizer C, et al. [Psychotherapy and pharmacotherapy for harmful tobacco use and tobacco dependency]. Nervenarzt 2016;87(1):35-45.
11. RefID: 3004. Becerra NA, Alba LH, Castillo JS, Murillo R, Canas A, Garcia-Herreros P. Alternative therapies for the cessation of addiction to snuff: Review of clinical practice guidelines. Gac Med Mex 2012;148(5):457-66.
12. RefID: 1584. Bergqvist D, Sawe J, Wahlberg E. [Lower limb arterial disease--nothing new since the SBU report]. Lakartidningen 2011;108(8):403-5.
13. RefID: 1868. Bolte G, Kuhn J, Twardella D, Fromme H. [Smoking bans in public places: current epidemiological evidence of cardiovascular health impacts at the population level]. Gesundheitswesen 2009;71(3):140-51.
14. RefID: 1219. Boveda Fontan J, Perula de Torres LA, Campinez Navarro M, Bosch Fontcuberta JM, Barragan Brun N, Prados Castillejo JA, et al. [Current evidence on the motivational interview in the approach to health care problems in primary care]. Aten Primaria 2013;45(9):486-95.
15. RefID: 1874. Bredesen H, Lous J. [Smoking cessation with special focus on primary health care]. Ugeskr Laeger 2009;171(9):683-8.
16. RefID: 2951. Buhler A, Thrul J. Smoking cessation in adolescents and young adults - Scientific basis and intervention. Sucht 2012;58(5):297-316.
17. RefID: 1822. Camarelles Guillem F, Salvador Llivina T, Ramon Torell JM, Cordoba Garcia R, Jimenez Ruiz C, Lopez Garcia-Aranda V, et al. [Consensus on health assistance for smoking control in Spain]. Rev Esp Salud Publica 2009;83(2):175-200.
18. RefID: 1309. Camarelles Guillem F, Dalmau Gonzalez-Gallarza R, Clemente Jimenez L, Diaz-Maroto Munoz JL, Lozano Polo A, Pinet Ogue MC, et al. [Consensus report for the clinical care of smoking cessation in Spain. Comite Nacional para la Prevencion del Tabaquismo]. Med Clin (Barc) 2013;140(6):272.
19. RefID: 1008. Canas A, Alba LH, Becerra N, Murillo R, Paez N, Mosquera C. [Efficacy and safety of medication use for the cessation of tobacco addiction: A review of Clinical Practice Guidelines]. Rev Salud Publica (Bogota) 2014;16(5):772-85.
20. RefID: 1916. Cases Amenos A, Goicoechea Diezhandino M, de Alvaro Moreno F. [Arterial hypertension and dyslipidemia in patients with chronic kidney disease (CKD). Anti-platelet aggregation. Goal oriented treatment]. Nefrologia 2008;28 Suppl 3:39-48.
21. RefID: 3738. Centre for Reviews and Dissemination. Nederland beschikbaar zijn: een systematische review op basis van Cochrane-gegevens [The efficacy of smoking cessation methods available in the Netherlands: a systematic review based on Cochrane data] (Provisional abstract). Database of Abstracts of Reviews of Effects 2015;(2).
22. RefID: 348. Corvalan B, Vejar M, Bambs S C, Pavie G, Zagolin B, Cerda L. [Clinical Practice Guidelines for Smoking Cessation]. Rev Med Chil 2017;145(11):1471-9.
23. RefID: 696. Cselko Z, Penzes M. [Summary of the existing knowledge about electronic cigarettes]. Orv Hetil 2016;157(25):979-86.
24. RefID: 1035. Dautzenberg B, Adler M, Garelik D, Loubrieu JF, Peiffer G, Perriot J, et al. [Adaptations to the management of smoking cessation with the arrival of e-cigarette? Expert opinion from the Office francais de prevention du tabagisme (OFT) 2014]. Rev Mal Respir 2014;31(7):641-5.
25. RefID: 3100. de Focchi GRA, da Silva IDCG, Scivoletto S. Association between polymorphism SLC6A3 3'UTR VNTR and response to treatment of nicotine dependence. J Bras Psiquiatr 2011;60(4):259-65.
26. RefID: 3420. de Oliveira RM, Furegato ARF. Impact and barriers for the restriction of smoking during psychiatric hospitalization: An integrative review. Paideia 2014;24(58):261-9.
27. RefID: 3008. De HJ, Bleda S, Varela C, Acin A. Role of statins, smoking cessation, blood pressure control and life style in abdominal aortic aneurysms. Ital J Vasc Endovasc Surg 2012;19(4):199-208.
28. RefID: 3362. Ervilha RR, de Andrade BABB, Gomide HP, Machado NM, Formagini TDB, Ronzani TM. Use of cell phone messages for smoking cessation: A systematic review. Psicologia em Estudo 2017;22(2):199-208.
29. RefID: 3267. Exarchos X, Kyriakou K, Lili E, Hainoglou A, Karakiulakis G. Pharmacological agents for smoking cessation treatments: Evidence-based medicine comparative evaluation. Epitheorese Klin Farmakol Farmakokinet 2009;27(3):237-56.
30. RefID: 1513. Ferrario MM, Borsani A. [Workplace health promotion: what evidence of effectiveness?]. G Ital Med Lav Ergon 2011;33(2 Suppl):44-7.
31. RefID: 1369. Figueroa Casas JC, Schiavi E, Mazzei JA, Lopez AM, Rhodius E, Ciruzzi J, et al. [Guidelines for COPD prevention, diagnosis and treatment in Argentina]. Medicina (B Aires ) 2012;72 Suppl 1:1-33.
32. RefID: 3359. Formagini TDB, Machado NM, Richter KP, Ronzani TM. Smoking cessation interventions in light smokers: A systematic review. Psicologia em Estudo 2015;20(2):201-11.
33. RefID: 3219. Forsetlund L, Eike MC, Vist GE. Effect of interventions to improve health care services for ethnic minority populations. Nor Epidemiol 2010;20(1):41-52.
34. RefID: 1789. Friederich HM, Batra A. [Drug treatment of tobacco dependence]. Dtsch Med Wochenschr 2009;134(47):2402-5.
35. RefID: 1666. Galeone D. [Activities for the prevention of smoking]. G ital cardiol 2010;11(5 Suppl 3):68S-70S.
36. RefID: 1094. Glatz U. [Electronic Cigarettes: "Healthy" smoke and help to abstinence?]. Dtsch Med Wochenschr 2014;139(11):520-1.
37. RefID: 1959. Griesam N, Meyer C, Haug S, Ruge J, Schumann A, Rumpf HJ, et al. [Diversity and accessibility of smoking cessation aids in the internet]. Gesundheitswesen 2008;70(6):372-6.
38. RefID: 3034. Hanewinkel R, Isensee B, Morgenstern M. Tobacco prevention measures. Pneumologe 2012;9(3):191-6.
39. RefID: 1503. Haug S, Sannemann J, Meyer C, John U. [Internet and mobile phone interventions to decrease alcohol consumption and to support smoking cessation in adolescents: a review]. Gesundheitswesen 2012;74(3):160-77.
40. RefID: 3467. Haug S, Schaub M. Efficacy of Internet programs for tobacco smoking: A systematic review. Zeitschrift fur Gesundheitspsychologie 2011;19(4):181-96.
41. RefID: 2754. He Z-Y, Yang L, Zhang J, Yao Y-L. Effectiveness and safety of bupropion for smoking cessation in smokers with cardiovascular disease: A meta-analysis. Chin J Evid -Based Med 2014;14(3):292-8.
42. RefID: 1226. Hering T. [Nicotine withdrawal - don't leave your COPD patients alone]. MMW Fortschr Med 2013;155(9):53-4.
43. RefID: 762. Hoch E, Petersen K, Kopp I, Batra A, Mann K. [Methodology of the S3 guidelines on alcohol and tobacco-related disorders]. Nervenarzt 2016;87(1):6-12.
44. RefID: 1034. Holzinger F, Beck S, Deutschen Gesellschaft fur Allgemeinmedizin und Familienmedizin (DEGAM). [Diagnostics and treatment of cough]. Dtsch Med Wochenschr 2014;139(40):2025.
45. RefID: 1481. Horak FJ, Fazekas T, Zacharasiewicz A, Eber E, Kiss H, Lichtenschopf A, et al. [The Fetal Tobacco Syndrome - A statement of the Austrian Societies for General- and Family Medicine (OGAM), Gynecology and Obstetrics (OGGG), Hygiene, Microbiology and Preventive Medicine (OGHMP), Pediatrics and Adolescence Medicine (OGKJ) as well as Pneumology (OGP)]. Wien Klin Wochenschr 2012;124(5-6):129-45.
46. RefID: 1735. Jahne A, Cohrs S, Rodenbeck A, Andreas S, Loessl B, Feige B, et al. [Nicotine. Influence on sleep and its relevance for psychiatry and psychotherapy]. Nervenarzt 2010;81(7):844-59.
47. RefID: 1918. Jankowski P, Brzozowska-Kiszka M, Debicka-Dabrowska D, Kawecka-Jaszcz K. [The influence of ban on smoking on admissions due to acute coronary syndromes - a systematic review]. Kardiol Pol 2008;66(10):1107-12.
48. RefID: 2280. Kala P, Mates M, Zelizko M, Rokyta R, Ostadal P. 2017 ESC Guidelines for the management of acute myocardial infarction in patients presenting with ST-segment elevation. Summary of the document prepared by the Czech Society of Cardiology. Cor Vasa 2017;59(6):e613-e644.
49. RefID: 2553. Katanoda K. Scientific evidence regarding tobacco control policies. Jpn J Lung Cancer 2015;55(4):273-6.
50. RefID: 1992. Kim Y, Park I, Park JS. [Meta-analysis of effects on adolescent smoking cessation programs in Korea]. Taehan Kanho Hakhoe chi 2008;38(2):204-16.
51. RefID: 1821. Kralikova E, Sochor O. [10th Conference of the European Society for Research on Nicotine and Tobacco: evidence based treatment and prevention]. Cas Lek Cesk 2009;148(2):102-3.
52. RefID: 3230. Kreutzkamp B. Smoking cessation: Nicotine replacement therapy improves long-term abstinence from cigarettes. Med Monatsschr Pharm 2010;33(3):104-5.
53. RefID: 1913. Kull I, Johansson GS, Lisspers K, Jagorstrand B, Romberg K, Tilling B, et al. [Efficient care in asthma/COPD primary health care clinics]. Lakartidningen 2008;105(42):2937-40.
54. RefID: 3207. Laizo A. Chronic obstructive pulmonary disease - A review. Rev Port Pneumol 2009;15(6):1157-66.
55. RefID: 3436. Ledochowski L, Taylor AH, Haasova M, Faulkner GE, Ussher MH, Kopp M. Immediate effects of single bouts of exercise on cravings to smoke: A systematic review of the literature. Zeitschrift fur Gesundheitspsychologie 2013;21(3):122-37.
56. RefID: 2101. Li Y, Pan Y, Pan J, Wei L, Shang W, Ge L, et al. Effectiveness of SMS for smoking cessation: A meta-analysis. Chin J Evid -Based Med 2017;17(11):1325-31.
57. RefID: 1563. Lichtenschopf A, Osterreichische Gesellschaft fP. [Guidelines for smoking cessation - update 2010]. Wien Klin Wochenschr 2011;123(9-10):299-315.
58. RefID: 1647. Lindstrom D, Tonnesen H, Adami J. [Smoking cessation in surgical interventions. Dramatic drop in the risk of postoperative complications]. Lakartidningen 2010;107(43):2634-5.
59. RefID: 2403. Lisowska A, Makarewicz-Wujec M, Filipiak KJ. Risk factors, prognosis, and secondary prevention of myocardial infarction in young adults in Poland. Kardiol Pol 2016;74(10):1148-53.
60. RefID: 1519. Liu LS, Writing Group. [2010 Chinese guidelines for the management of hypertension]. Chung Hua Hsin Hsueh Kuan Ping Tsa Chih 2011;39(7):579-615.
61. RefID: 1574. Liu M, Huang Xm, Lv J, Li Lm. [Evidence of effectiveness on tobacco control programs: a systematic review of intervention studies in China]. Chung Hua Liu Hsing Ping Hsueh Tsa Chih 2011;32(1):77-80.
62. RefID: 814. Liu Z, Wang Y, Wu Y, Yang J. [Condition and effectiveness evaluation of acupuncture for smoking cessation]. Zhongguo zhenjiu 2015;35(8):851-7.
63. RefID: 2843. Lo RL, Pizzo G. Smoking, alcohol and oral health. Dent Cadmos 2013;81(8):486-503.
64. RefID: 2908. Lukina YV, Martsevich SY. Varenicline in patients with cardiovascular diseases - friend or enemy? Ration Pharmacother Cardiol 2013;9(4):455-8.
65. RefID: 1877. Lungen M, Schroer-Gunther MA, Passon AM, Gerber A, Lauterbach KW. [Does the lack of evidence of effectiveness among international studies on interventions in prevention and health promotion have an impact on a German Act of Prevention?]. Med Klin 2009;104(2):101-7.
66. RefID: 763. Mann K, Batra A. [S3 guidelines on alcohol and tobacco-related disorders]. Nervenarzt 2016;87(1):4-5.
67. RefID: 3517. Mazoni CG, Fernandes S, Pierozan PS, Moreira T, Freese L, Ferigolo M, et al. Efficacy of pharmacological and no-pharmacological treatments for smoking cessation. Estudos de Psicologia 2008;13(2):133-40.
68. RefID: 3269. Mould-Quevedo JF, Contreras-Hernandez I. Cost-effective analysis of varenicline (Champix) versus the nicotine patch in treatment for smoking cessation in Mexico. Pharmacoecon Span Res Artic 2009;6(1):22-32.
69. RefID: 768. Muhlig S, Andreas S, Batra A, Petersen KU, Hoch E, Ruther T. [Psychiatric comorbidities with tobacco-related disorders]. Nervenarzt 2016;87(1):46-52.
70. RefID: 2451. Muhlig S, Paulick J, Lindenmeyer J, Rinck M, Cina R, Wiers RW. Applying the 'cognitive bias modification' concept to smoking cessation - A systematic review. Sucht 2016;62(6):333-54.
71. RefID: 1769. No author listed. [Guideline for diagnosis and management of COPD (chronic obstructive pulmonary disease). The Third Edition: therapy and management]. Nihon Kokyuki Gakkai Zasshi 2009;Suppl Copd:70-144.
72. RefID: 1634. Olano Espinosa E, Minue Lorenzo C, Ayesta Ayesta FJ, Sociedad Espanola de Especialistas en Tabaquismo. [Pharmacotherapies for nicotine dependence: review of scientific evidence. Spanish Society of Smoking Specialists: recommendations about election and individualization]. Med Clin (Barc) 2011;136(2):79-83.
73. RefID: 2729. Oztuna F, Aytemur ZA, Elbek O, Kilinc O, Kucuk CU, Akcay S, et al. Can the use of smokeless tobacco products be accepted as a harm reduction method in tobacco addiction? Turk Toraks Derg 2014;15(4):136-41.
74. RefID: 3116. Park S-W. Evidence-based smoking cessation counseling: Motivational intervention and relapse prevention. J Kor Med Assoc 2011;54(10):1036-46.
75. RefID: 2565. Peters SAE. Smoking cessation is never too late, even in old age. Ned Tijdschr Geneeskd 2015;159(31).
76. RefID: 3850. Pichon-Riviere A, Augustovski F, Garcia Marti S, Bardach A, Lopez A, Regueiro A, et al. Usefulness of varenicline in smoking cessation therapy (Structured abstract). Health Technology Assessment Database 2016;(2016 Issue 4).
77. RefID: 363. Piepoli MF, Hoes AW, Agewall S, Albus C, Brotons C, Catapano AL, et al. 2016 European Guidelines on cardiovascular disease prevention in clinical practice. Eur Heart J 2016;37(29):2315-81.
78. RefID: 3830. Pisinger C, Jorgensen T. Smoking cessation among patients in general practice. Which method is the most cost-effective? (Structured abstract). Health Technology Assessment Database 2016;(2016 Issue 4).
79. RefID: 876. Postolache P, Nemes RM, Serban RI, Rad RM, Stratulat IS. ELECTRONIC CIGARETTE--A WAY OF SMOKING CESSATION? Rev Med Chir Soc Med Nat Iasi 2015;119(2):510-6.
80. RefID: 1843. Rasch A, Greiner W. [Efficacy and cost-effectiveness of smoking cessation courses in the statutory health insurance: a review]. Gesundheitswesen 2009;71(11):732-8.
81. RefID: 1406. Reese C, Spieser A, Mittag O. [Psychological interventions in the rehabilitation of patients with coronary heart disease: summary of evidence and recommendations from systematic reviews and guidelines]. Rehabilitation (Stuttg) 2012;51(6):405-14.
82. RefID: 663. Royo-Bordonada MA, Armario P, Lobos Bejarano JM, Pedro-Botet J, Villar Alvarez F, Elosua R, et al. [Spanish Adaptation of the 2016 European Guidelines on Cardiovascular Disease Prevention in Clinical Practice]. Rev Esp Salud Publica 2016;90:e1-e24.
83. RefID: 2440. Saez De Lafuente MA, Cobos CR, Apinaniz Fernandez De LA, Parraza DN, Aizpuru BF. Spontaneous quitters. Predictors of success in quitting smoking. Systematic review. DOLOR 2016;31(4):135-41.
84. RefID: 1641. Sansores RH, Perez-Bautista O, Ramirez-Venegas A. [Proposal of Latin-American guidelines for smoking cessation]. Salud Publica Mex 2010;52 Suppl 2:S355-S365.
85. RefID: 1401. Sansores RH, Ramirez-Venegas A, Perez-Bautista O, Bustos M. [Vision abnormalities associated to smoking. A systematic review related to a clinical case]. Rev Invest Clin 2012;64(1):89-101.
86. RefID: 1681. Sociedade Brasileira de Pneumologia e Tisiologia, Sociedade Brasileira dC, Associacao Brasileira dP, Federacao Brasileira das Sociedades de Ginecologia e Obstetricia, Sociedade Brasileira dA, Associacao Brasileira de Medicina Intensiva, et al. [Smoking: Part 2]. Rev Assoc Med Bras 2010;56(3):264-7.
87. RefID: 1669. Sociedade Brasileira de Pneumologia e Tisiologia, Sociedade Brasileira dC, Associacao Brasileira dP, Federacao Brasileira das Sociedades de Ginecologia e Obstetricia, Sociedade Brasileira dA, Associacao Brasileira de Medicina Intensiva, et al. [Smoking - part 3]. Rev Assoc Med Bras 2010;56(4):384-8.
88. RefID: 835. Sotoda Y, Hirooka S, Orita H, Wakabayashi I. [Recent Knowledge of Smoking and Peripheral Arterial Disease in Lower Extremities]. Nippon Eiseigaku Zasshi 2015;70(3):211-9.
89. RefID: 1732. Stuck BA, Abrams J, de la Chaux R, Dreher A, Heiser C, Hohenhorst W, et al. [S1 guideline on the "diagnosis and treatment of snoring in adults"]. HNO 2010;58(3):272-8.
90. RefID: 1864. Tang SM, Chen CH, Chen JY. [Applying the transtheoretical model to tobacco prevention education in adolescence: a literature review]. Hu Li Tsa Chih 2009;56(2):81-8.
91. RefID: 892. Taniguchi C, Tanaka H. [Determining an appropriate scheme for "Quitline" in Japan]. Nippon Koshu Eisei Zasshi 2015;62(3):125-32.
92. RefID: 3342. Tonnesen P. Which drug to be used in smoking cessation? Pol Arch Med Wewn 2008;118(6):373-6.
93. RefID: 3450. Topa G, Moriano JA, Morales JF. Giving up smoking: Meta-analysis and structural equation model from the Theory of Planned Behavior. Clinica y Salud 2012;23(1):43-61.
94. RefID: 2311. Tytgat G. Novel developments in pancreatic disorders: Peter Layer Lecture - Gastro Update Europe 2016, Prague. Gastroenterol Hepatol 2017;71(3):261-2.
95. RefID: 895. Vaz-Carneiro A, Costa J. [Analysis of the Cochrane Review: Electronic Cigarettes for Smoking Cessation and Reduction. Cochrane Database Syst Rev. 2014,12: CD010216]. Acta Med Port 2015;28(2):145-7.
96. RefID: 372. Vaz-Carneiro A, Costa J. [Analysis of the Cochrane Review: Incentives for Smoking Cessation. Cochrane Database Syst Rev. 2015;5:CD004307]. Acta Med Port 2016;29(1):12-4.
97. RefID: 1679. von Garnier C. [Preventive relapse treatment for tobacco cessation - is it worthwhile?]. Ther Umsch 2010;67(8):431-3.
98. RefID: 921. Walter M, Dursteler KM, Petitjean SA, Wiesbeck GA, Euler S, Sollberger D, et al. [Psychosocial Treatment of Addictive Disorders--An Overview of Psychotherapeutic Options and their Efficacy]. Fortschr Neurol Psychiatr 2015;83(4):201-10.
99. RefID: 1842. Walter M, Wiesbeck GA. [Pharmacotherapy of substance dependence and withdrawal syndromes]. Ther Umsch 2009;66(6):449-57.
100. RefID: 3217. Wang P, Xie H-X, Qu Y-L, Man Y. Evidence-based treatment for a smoker with periimplantitis. Chin J Evid -Based Med 2010;10(6):759-62.
101. RefID: 676. Watanabe R, Inoue D. [Smoking & Bone.]. Clin calcium 2016;26(10):1445-50.
102. RefID: 1957. Wind LA, Chavannes NH, Kaper J, Frijling BD, van der Laan JR, Wiersma T, et al. [Summary of the practice guideline 'Smoking cessation' from the Dutch College of General Practitioners]. Ned Tijdschr Geneeskd 2008;152(26):1459-64.
103. RefID: 1256. Wirth C, Stanga Z, Birrenbach T, Egermann U, Rodondi N. [Evidence based check-up: the general medical point of view]. Praxis (Bern 1994) 2013;102(11):647-56.
104. RefID: 1786. Worth H. [What is proven in the treatment of COPD?]. Internist (Berl) 2009;50(12):1345-57.
105. RefID: 817. Wu L, Jiang B, Zeng J, He Y. [Minimal-Intervention on smoking cessation: a Meta-analysis]. Chung Hua Liu Hsing Ping Hsueh Tsa Chih 2015;36(6):658-62.
106. Sun AL, Li GJ, Wei T. [Effects of smoking cessation on the risk of hypertension: a meta-analysis]. Chung-Hua i Hsueh Tsa Chih [Chinese Medical Journal]. 2019;99(26):2068–72.
107. Shi FH, He YP, Xu G, Zhu JF. Progress in research and application of new media in tobacco control among adolescents. [Chinese]. Journal of Shanghai Jiaotong University (Medical Science). 2019;39(2):213–6.
108. Petersen KU, Batra A. The standard of smoking cessation interventions. [German]. Atemwegs- und Lungenkrankheiten. 2019;45(7):320–5.
109. Castaneda E, Krikorian A, Alarcon. Current applications of clinical hypnosis in Latin America: A systematic review of the literature. Aplicaciones actuales de la hipnosis clinica en latinoamerica: Una revision sistematica de la literatura. 2018;36(2):269–83.
110. Byaruhanga J, Atorkey P, McLaughlin M, Brown A, Byrnes E, Paul C, et al. Effectiveness of Individual Real-Time Video Counseling on Smoking, Nutrition, Alcohol, Physical Activity, and Obesity Health Risks: Systematic Review. Journal of Medical Internet Research. 2020;22(9):e18621.
111. Bruhn C. Smoking cessation with antidepressants?: Cochrane Review shows opportunities and limits of medicinal smoking cessation. [German]. Deutsche Apotheker Zeitung [Internet]. 2020;160(22). Available from: https://www.deutsche-apotheker-zeitung.de/daz-az/2020/daz-22-2020/rauchstopp-mit-antidepressiva

## Published or produced before 2008 (n=76)

1. RefID: 3697. Andersen S. Smoking cessation: the state of the science. The utility of the transtheoretical model in guiding interventions in smoking cessation (Structured abstract). Database of Abstracts of Reviews of Effects 2015;(2).
2. RefID: 3690. Ashenden R. A systematic review of the effectiveness of promoting lifestyle change in general practice (Structured abstract). Database of Abstracts of Reviews of Effects 2015;(2).
3. RefID: 3749. Ashenden R. A meta-analysis of the effectiveness of acupuncture in smoking cessation (Structured abstract). Database of Abstracts of Reviews of Effects 2015;(2).
4. RefID: 3684. Bains N. The use and impact of incentives in population-based smoking cessation programs: a review (Structured abstract). Database of Abstracts of Reviews of Effects 2015;(2).
5. RefID: 3772. Barth J. Efficacy of psychosocial interventions for smoking cessation in patients with coronary heart disease: a systematic review and meta-analysis (Structured abstract). Database of Abstracts of Reviews of Effects 2015;(2).
6. RefID: 3788. Bize R. Effectiveness of biomedical risk assessment as an aid for smoking cessation: a systematic review (Structured abstract). Database of Abstracts of Reviews of Effects 2015;(2).
7. RefID: 3767. Bradshaw T. Healthy living interventions and schizophrenia: a systematic review (Structured abstract). Database of Abstracts of Reviews of Effects 2015;(2).
8. RefID: 3709. Brothwell D. Should the use of smoking cessation products be promoted by dental offices: an evidence-based report (Structured abstract). Database of Abstracts of Reviews of Effects 2015;(2).
9. RefID: 3845. Canadian Agency for Drugs and Technologies in Health. Nicotine vaccines for smoking cessation (Structured abstract). Health Technology Assessment Database 2016;(2016 Issue 4).
10. RefID: 3731. Coyle S. Outreach-based HIV prevention for injecting drug users: a review of published outcome data (Structured abstract). Database of Abstracts of Reviews of Effects 2015;(2).
11. RefID: 3771. Doolan D. Efficacy of smoking cessation intervention among special populations: review of the literature from 2000 to 2005 (Structured abstract). Database of Abstracts of Reviews of Effects 2015;(2).
12. RefID: 3683. Dunn C. The use of brief interventions adapted from motivational interviewing across behavioral domains: a systematic review (Structured abstract). Database of Abstracts of Reviews of Effects 2015;(2).
13. RefID: 3693. Ebrahim S. Systematic review of randomised controlled trials of multiple risk factor interventions for preventing coronary heart disease (Structured abstract). Database of Abstracts of Reviews of Effects 2015;(2).
14. RefID: 3722. el-Guebaly N. Public health and therapeutic aspects of smoking bans in mental health and addiction settings (Structured abstract). Database of Abstracts of Reviews of Effects 2015;(2).
15. RefID: 3828. Ellis P, Robinson P, Ciliska D. Diffusion and dissemination of evidence-based cancer control interventions (Structured abstract). Health Technology Assessment Database 2016;(2016 Issue 4).
16. RefID: 3717. Eriksen M. A review of the health impact of smoking control at the workplace (Structured abstract). Database of Abstracts of Reviews of Effects 2015;(2).
17. RefID: 3736. Etter J. Nicotine replacement therapy for long-term smoking cessation: a meta-analysis (Provisional abstract). Database of Abstracts of Reviews of Effects 2015;(2).
18. RefID: 3799. Etter J. Cytisine for smoking cessation a literature: review and a meta-analysis (Structured abstract). Database of Abstracts of Reviews of Effects 2015;(2).
19. RefID: 3708. Fang W. Smoking cessation in pregnancy: a review of postpartum relapse prevention strategies (Structured abstract). Database of Abstracts of Reviews of Effects 2015;(2).
20. RefID: 3703. Garrison M. Smoking cessation interventions for adolescents: a systematic review (Structured abstract). Database of Abstracts of Reviews of Effects 2015;(2).
21. RefID: 3686. Girolami B. Treatment of intermittent claudication with physical training, smoking cessation, pentoxifylline, or nafronyl: a meta-analysis (Structured abstract). Database of Abstracts of Reviews of Effects 2015;(2).
22. RefID: 3747. Gorin S. Meta-analysis of the efficacy of tobacco counseling by health care providers (Provisional abstract). Database of Abstracts of Reviews of Effects 2015;(2).
23. RefID: 3745. Greenland S. A meta-analysis to assess the incidence of adverse effects associated with the transdermal nicotine patch (Structured abstract). Database of Abstracts of Reviews of Effects 2015;(2).
24. RefID: 3726. Hawkins R. Preventive health care, 1999 update: prevention of oral cancer mortality (Structured abstract). Database of Abstracts of Reviews of Effects 2015;(2).
25. RefID: 3805. Holmes S. Bupropion as an aid to smoking cessation: a review of real-life effectiveness (Provisional abstract). Database of Abstracts of Reviews of Effects 2015;(2).
26. RefID: 3715. Hopkins D. Reviews of evidence regarding interventions to reduce tobacco use and exposure to environmental tobacco smoke (Structured abstract). Database of Abstracts of Reviews of Effects 2015;(2).
27. RefID: 3759. Hrobjartsson A. Is the placebo powerless: an analysis of clinical trials comparing placebo with no treatment (Structured abstract). Database of Abstracts of Reviews of Effects 2015;(2).
28. RefID: 3769. Hughes J. The feasibility of smoking reduction: an update (Provisional abstract). Database of Abstracts of Reviews of Effects 2015;(2).
29. RefID: 3748. Hughes J. A meta-analysis of the efficacy of over-the-counter nicotine replacement (Structured abstract). Database of Abstracts of Reviews of Effects 2015;(2).
30. RefID: 3727. Kelley M. Prevention of lung cancer: summary of published evidence (Structured abstract). Database of Abstracts of Reviews of Effects 2015;(2).
31. RefID: 3786. Ketola E. Effectiveness of individual lifestyle interventions in reducing cardiovascular disease and risk factors (Structured abstract). Database of Abstracts of Reviews of Effects 2015;(2).
32. RefID: 3790. Khuder S. Effect of smoking cessation on major histologic types of lung cancer (Structured abstract). Database of Abstracts of Reviews of Effects 2015;(2).
33. RefID: 3518. Krebs PM. Computerized, tailored, theory-based interventions for health behavior change: A comprehensive meta-analysis. Dissertation Abstracts International: Section B: The Sciences and Engineering 2008;68(8-B):5552
34. RefID: 3815. Law M. An analysis of the effectiveness of interventions intended to help people stop smoking (Structured abstract). Database of Abstracts of Reviews of Effects 2015;(2).
35. RefID: 3699. Lawrence D. Smoking cessation interventions in U.S. racial/ethnic minority populations: an assessment of the literature (Structured abstract). Database of Abstracts of Reviews of Effects 2015;(2).
36. RefID: 3743. Leistikow B. Might stop smoking reduce injury death risks: a meta-analysis of randomized controlled trials (Structured abstract). Database of Abstracts of Reviews of Effects 2015;(2).
37. RefID: 1922. Lemmens V, Oenema A, Knut IK, Brug J. Effectiveness of smoking cessation interventions among adults: a systematic review of reviews. Eur J Cancer Prev 2008;17(6):535-44.
38. RefID: 3688. Levitt C. Systematic review of the literature on postpartum care: effectiveness of interventions for smoking relapse prevention, cessation, and reduction in postpartum women (Structured abstract). Database of Abstracts of Reviews of Effects 2015;(2).
39. RefID: 3806. Manske S. Best practice in group-based smoking cessation: results of a literature review applying effectiveness, plausibility, and practicality criteria (Provisional abstract). Database of Abstracts of Reviews of Effects 2015;(2).
40. RefID: 3796. May S. Do social support interventions ('buddy systems') aid smoking cessation: a review (Structured abstract). Database of Abstracts of Reviews of Effects 2015;(2).
41. RefID: 3813. McClure J. Are biomarkers a useful aid in smoking cessation: a review and analysis of the literature (Structured abstract). Database of Abstracts of Reviews of Effects 2015;(2).
42. RefID: 3704. Mojica W. Smoking-cessation interventions by type of provider: a meta-analysis (Provisional abstract). Database of Abstracts of Reviews of Effects 2015;(2).
43. RefID: 3843. Moyes L, Mundy L, Sullivan T, Merlin T, Hiller JE. Nicotine metabolite ratio test as a predictor of smoking cessation (Structured abstract). Health Technology Assessment Database 2016;(2016 Issue 4).
44. RefID: 3809. Munafo M. Are there sex differences in transdermal nicotine replacement therapy patch efficacy: a meta-analysis (Structured abstract). Database of Abstracts of Reviews of Effects 2015;(2).
45. RefID: 3842. Mundy L, McCaffrey N, Hiller JE. Program for the distribution of free nicotine patches: a smoking cessation program (Structured abstract). Health Technology Assessment Database 2016;(2016 Issue 4).
46. RefID: 3750. Myung S. Meta-analysis of studies investigating one-year effectiveness of transdermal nicotine patches for smoking cessation (Structured abstract). Database of Abstracts of Reviews of Effects 2015;(2).
47. RefID: 3863. National Institute for Health and Clinical Excellence. Chronic obstructive pulmonary disease: management of chronic obstructive pulmonary disease in adults in primary and secondary care (Structured abstract). Health Technology Assessment Database 2016;(2016 Issue 4).
48. RefID: 3838. National Institute for Health and Clinical Excellence. Brief interventions and referral for smoking cessation in primary care and other settings (Structured abstract). Health Technology Assessment Database 2016;(2016 Issue 4).
49. RefID: 3868. National Institute for Health and Clinical Excellence. Smoking cessation services in primary care, pharmacies, local authorities and workplaces, particularly for manual working groups, pregnant women and hard to reach communities (Structured abstract). Health Technology Assessment Database 2016;(2016 Issue 4).
50. RefID: 3820. NHS Centre for Reviews and Dissemination. Management of lung cancer (Structured abstract). Health Technology Assessment Database 2016;(2016 Issue 4).
51. RefID: 3733. Nice V. Nursing intervention and smoking cessation: a meta-analysis (Structured abstract). Database of Abstracts of Reviews of Effects 2015;(2).
52. RefID: 3744. Nishi N. A meta-analytic review of the effect of exercise on smoking cessation (Structured abstract). Database of Abstracts of Reviews of Effects 2015;(2).
53. RefID: 3725. Pan W. Proactive telephone counseling as an adjunct to minimal intervention for smoking cessation: a meta-analysis (Provisional abstract). Database of Abstracts of Reviews of Effects 2015;(2).
54. RefID: 3751. Prochaska J. A meta-analysis of smoking cessation interventions with individuals in substance abuse treatment or recovery (Structured abstract). Database of Abstracts of Reviews of Effects 2015;(2).
55. RefID: 3840. Ranney L, Melvin C, Lux L, McClain E, Morgan L, Lohr K. Tobacco use: prevention, cessation, and control (Structured abstract). Health Technology Assessment Database 2016;(2016 Issue 4).
56. RefID: 3728. Ray J. Preconception care and the risk of congenital anomalies in the offspring of women with diabetes mellitus: a meta-analysis (Structured abstract). Database of Abstracts of Reviews of Effects 2015;(2).
57. RefID: 3719. Revere D. Review of computer-generated outpatient health behavior interventions: clinical encounters 'in absentia' (Structured abstract). Database of Abstracts of Reviews of Effects 2015;(2).
58. RefID: 3803. Richmond R. A comparison of measures used to assess effectiveness of the transdermal nicotine patch at 1 year (Structured abstract). Database of Abstracts of Reviews of Effects 2015;(2).
59. RefID: 3689. Riemsma R. Systematic review of the effectiveness of stage based interventions to promote smoking cessation (Structured abstract). Database of Abstracts of Reviews of Effects 2015;(2).
60. RefID: 3810. Scharf D. Are there gender differences in smoking cessation, with and without bupropion: pooled- and meta-analyses of clinical trials of Bupropion SR (Structured abstract). Database of Abstracts of Reviews of Effects 2015;(2).
61. RefID: 3780. Silagy C. Effectiveness of training health professionals to provide smoking cessation interventions: systematic review of randomised controlled trials (Structured abstract). Database of Abstracts of Reviews of Effects 2015;(2).
62. RefID: 3802. Sin D. Contemporary management of chronic obstructive pulmonary disease: a scientific review (Structured abstract). Database of Abstracts of Reviews of Effects 2015;(2).
63. RefID: 3696. Sluijs E. Stage-based lifestyle interventions in primary care: are they effective? (Provisional abstract). Database of Abstracts of Reviews of Effects 2015;(2).
64. RefID: 3779. Smedslund G. The effectiveness of workplace smoking cessation programmes: a meta-analysis of recent studies (Structured abstract). Database of Abstracts of Reviews of Effects 2015;(2).
65. RefID: 3766. Tang J. How effective is nicotine replacement therapy in helping people to stop smoking? (Structured abstract). Database of Abstracts of Reviews of Effects 2015;(2).
66. RefID: 3818. Taylor A. The acute effects of exercise on cigarette cravings, withdrawal symptoms, affect and smoking behaviour: a systematic review (Structured abstract). Database of Abstracts of Reviews of Effects 2015;(2).
67. RefID: 3740. Taylor R. Mortality reductions in patients receiving exercise-based cardiac rehabilitation: how much can be attributed to cardiovascular risk factor improvements? (Provisional abstract). Database of Abstracts of Reviews of Effects 2015;(2).
68. RefID: 3710. Wagena E. Should nortriptyline be used as a first-line aid to help smokers quit: results from a systematic review and meta-analysis (Structured abstract). Database of Abstracts of Reviews of Effects 2015;(2).
69. RefID: 3720. Walters S. A review of computer and Internet-based interventions for smoking behavior (Structured abstract). Database of Abstracts of Reviews of Effects 2015;(2).
70. RefID: 3795. West R. Effect of oral nicotine dosing forms on cigarette withdrawal symptoms and craving: a systematic review (Structured abstract). Database of Abstracts of Reviews of Effects 2015;(2).
71. RefID: 3778. White A. The effects of auricular acupuncture on smoking cessation may not depend on the point chosen: an exploratory meta-analysis (Provisional abstract). Database of Abstracts of Reviews of Effects 2015;(2).
72. RefID: 3754. White A. A meta-analysis of acupuncture techniques for smoking cessation (Structured abstract). Database of Abstracts of Reviews of Effects 2015;(2).
73. RefID: 3789. Wilson K. Effect of smoking cessation on mortality after myocardial infarction: meta-analysis of cohort studies (Structured abstract). Database of Abstracts of Reviews of Effects 2015;(2).
74. RefID: 3836. Wilt TJ, Niewoehner D, Kim CB, Kane RL, Linabery A, Tacklind J, et al. Use of spirometry for case finding, diagnosis, and management of chronic obstructive pulmonary disease (COPD) (Structured abstract). Health Technology Assessment Database 2016;(2016 Issue 4).
75. RefID: 3804. Woolacott N. The clinical effectiveness and cost-effectiveness of bupropion and nicotine replacement therapy for smoking cessation: a systematic review and economic evaluation (Structured abstract). Database of Abstracts of Reviews of Effects 2015;(2).
76. RefID: 3782. Wu P. Effectiveness of smoking cessation therapies: a systematic review and meta-analysis (Structured abstract). Database of Abstracts of Reviews of Effects 2015;(2).

## Not a systematic review or an overview of reviews with a network meta-analysis (n=1239)

1. RefID: 1829. Cilostazol: new drug. Intermittent claudication: too little efficacy, too many risks. Prescrire Int 2009;18(100):56-9.
2. RefID: 5306. Combating Tobacco in Military and Veteran Populations. 2009.
3. RefID: 2254. OHTAC recommendation: Population-based smoking cessation strategies. Ont Health Technol Assess Ser 2010;10(1). [PMID: http://www.health.gov.on.ca/english/providers/program/ohtac/tech/recommend/rec_smoking_20100114.pdf]
4. RefID: 3141. Varenicline and cardiovascular events? Drug Ther Bull 2011;49(9):100. [PMID: http://dtb.bmj.com/content/49/9/98.full.pdf]
5. RefID: 3088. More evidence that varenicline harms the heart. Harv Ment Health Lett 2011;28(4):7
6. RefID: 2949. Chronic stable angina guidelines: Is there an emerging international consensus? Br J Cardiol 2012;19(SUPPL.2):S2-S11. [PMID: http://bjcardio.co.uk/files/pdf/supplements/Br-J-Cardiol-2012-19-S2-S2-S11.pdf]
7. RefID: 2998. New Zealand 2012 guidelines for the management of non ST-elevation acute coronary syndromes. New Zealand Med J 2012;125(1357):122-47. [PMID: http://www.nzma.org.nz/journal/index.shtml]
8. RefID: 2709. The cochrane database of systematic reviews - Issue 1 2014. J Evid Based Med 2014;7(1):60-1.
9. RefID: 3402. Editorial: Ceci n'est pas une cigarette. The Lancet Psychiatry 2015;2(12):1043
10. RefID: 2623. Staff gain support on e-cigarette use. Nurs Stand 2015;29(52):10
11. RefID: 2509. Exam 1: Comparative Efficacy of Pharmacological Interventions in Preventing Relapse of Crohn's Disease After Surgery: A Systematic Review and Network Meta-analysis. Gastroenterology 2015;148(1):e14. [PMID: http://www.journals.elsevier.com/gastroenterology/]
12. RefID: 2535. Electronic nicotine delivery systems. Pediatrics 2015;136(5):1018-26. [PMID: http://pediatrics.aappublications.org/content/pediatrics/136/5/1018.full.pdf]
13. RefID: 454. Committee Opinion No. 721 Summary: Smoking Cessation During Pregnancy. Obstet Gynecol 2017;130(4):929-30.
14. RefID: 2272. Erratum: Association between initial use of e-cigarettes and subsequent cigarette smokingAmong adolescents and young adults:A systematic review and Meta-analysis(JAMA Pediatrics (2017) 171:8 (788-797) DOI: 10.1001/jamapediatrics.2017.1488). Jama, Pediatr 2018;172(1):98. [PMID: https://jamanetwork.com/journals/jamapediatrics/articlepdf/2665737/jamapediatrics_2017_cx_170019.pdf]
15. RefID: 1258. Abo-Zaid G, Guo B, Deeks JJ, Debray TPA, Steyerberg EW, Moons KGM, et al. Individual participant data meta-analyses should not ignore clustering. J Clin Epidemiol 2013;66(8):865-73.
16. Aboriginal Health & Medical Research Council of NSW and NSW Ministry of Health 2014. The ATRAC Framework: A strategic framework for Aboriginal tobacco resistance and control in NSW. Sydney: 2019.
17. RefID: 2548. Abrams DB, Niaura R. The importance of science-informed policy and what the data really tell us about e-cigarettes. Isr J Health Policy Res 2015;4(1):22. [PMID: http://www.ijhpr.org/]
18. RefID: 1736. Abrams DB, Graham AL, Levy DT, Mabry PL, Orleans CT. Boosting population quits through evidence-based cessation treatment and policy. Am J Prev Med 2010;38(3 Suppl):S351-S363
19. RefID: 1606. Abroms LC, Padmanabhan N, Thaweethai L, Phillips T. iPhone apps for smoking cessation: a content analysis. Am J Prev Med 2011;40(3):279-85.
20. RefID: 3423. Adams CE, Heppner WL, Houchins S, Stewart DW, Vidrine JI, Wetter DW. Mindfulness meditation and addictive behaviors. 2014;311-43.
21. RefID: 1810. Adams J. The role of time perspective in smoking cessation amongst older English adults. Health Psychol 2009;28(5):529-34.
22. RefID: 433. Afshin A, Babalola D, Mclean M, Yu Z, Ma W, Chen CY, et al. Information Technology and Lifestyle: A Systematic Evaluation of Internet and Mobile Interventions for Improving Diet, Physical Activity, Obesity, Tobacco, and Alcohol Use. J Am Heart Assoc 2016;5(9):
23. RefID: 928. Agboola SA, Coleman T, McNeill A, Leonardi-Bee J. Abstinence and relapse among smokers who use varenicline in a quit attempt-a pooled analysis of randomized controlled trials. Addiction 2015;110(7):1182-93.
24. RefID: 2773. Agnihotri R, Gaur S. Implications of tobacco smoking on the oral health of older adults. Geriatr Gerontol Int 2014;14(3):526-40. [PMID: http://www.blackwellpublishing.com/journals/ggi]
25. RefID: 3132. Agyapong VIO, Farren CK, McLoughlin DM. Mobile Phone Text Message Interventions in Psychiatry - what are the possibilities? Curr Psychiatry Rev 2011;7(1):50-6.
26. RefID: 1643. Ahijevych K, Garrett BE. The role of menthol in cigarettes as a reinforcer of smoking behavior. Nicotine Tob Res 2010;12 Suppl 2:S110-S116
27. RefID: 1605. Ahmad N, Boutron I, Dechartres A, Durieux P, Ravaud P. Geographical representativeness of published and ongoing randomized controlled trials. The example of: Tobacco consumption and HIV infection. PloS one 2011;6(2):e16878
28. RefID: 1306. Ahmed AIA, Ali ANA, Kramers C, Harmark LVD, Burger DM, Verhoeven WMA. Neuropsychiatric adverse events of varenicline: a systematic review of published reports. J Clin Psychopharmacol 2013;33(1):55-62.
29. RefID: 1206. Aiello-Laws LB. Is dyspnea management evidence-based? Oncol Nurs Forum 2013;40(6):533-4.
30. RefID: 966. Akerman SC, Brunette MF, Green AI, Goodman DJ, Blunt HB, Heil SH. Treating tobacco use disorder in pregnant women in medication-assisted treatment for an opioid use disorder: a systematic review. J Subst Abuse Treat 2015;52:40-7.
31. RefID: 3512. Al-Lehiany O, Stanley D. Smoking cessation program targeting adolescents: Saudi Arabia. J Smok Cessat 2009;4(1):3-9.
32. RefID: 3237. Al Bareeq JM, Fedorowicz Z. Best evidence from the Cochrane library. Bahrain Med Bull 2010;32(4). [PMID: http://www.bahrainmedicalbulletin.com/december_2010/Medical_Quiz_Answer.pdf]
33. RefID: 1508. Albrecht S, Kelly-Thomas K, Osborne JW, Ogbagaber S. The SUCCESS program for smoking cessation for pregnant women. J Obstet Gynecol Neonatal Nurs 2011;40(5):520-31.
34. RefID: 1003. Aldrich MC, Hidalgo B, Widome R, Briss P, Brownson RC, Teutsch SM. The role of epidemiology in evidence-based policy making: a case study of tobacco use in youth. Ann Epidemiol 2015;25(5):360-5.
35. RefID: 395. Alghamdi M, Gashgari H, Househ M. A Systematic Review of Mobile Health Technology Use in Developing Countries. Stud Health Technol Inform 2015;213:223-6.
36. RefID: 1434. Ali AY, Safwat T, Onyemelukwe G, Otaibi MAA, Amir AA, Nawas YN, et al. Smoking prevention and cessation in the Africa and Middle East region: a consensus draft guideline for healthcare providers--executive summary. Respiration 2012;83(5):423-32.
37. RefID: 142. Ali A, Kaplan CM, Derefinko KJ, Klesges RC. Smoking Cessation for Smokers Not Ready to Quit: Meta-analysis and Cost-effectiveness Analysis. Am J Prev Med 2018;55(2):253-62.
38. RefID: 3356. Almestahiri R, Rundle-Thiele S, Parkinson J, Arli D. The use of the major components of social marketing: A systematic review of tobacco cessation programs. Social Marketing Quarterly 2017;23(3):232-48.
39. RefID: 3147. Ambrosino N. The case for inspiratory muscle training in COPD. Eur Respir J 2011;37(2):233-7. [PMID: http://erj.ersjournals.com/content/37/2/233.full.pdf+html]
40. RefID: 1442. American College Health Association. Position statement on tobacco on college and university campuses. J Am Coll Health 2012;60(3):266-7.
41. RefID: 1524. American College of Cardiology Foundation/American Heart Association Task Force, American Stroke Association, American Association of Neuroscience Nurses, American Association of Neurological Surgeons, American College of Radiology, American Society of Neuroradiology, et al. 2011 ASA/ACCF/AHA/AANN/AANS/ACR/ASNR/CNS/SAIP/SCAI/SIR/SNIS/SVM/SVS guideline on the management of patients with extracranial carotid and vertebral artery disease: executive summary. J Neurointerv Surg 2011;3(2):100-30.
42. RefID: 974. American Diabetes Association. (4) Foundations of care: education, nutrition, physical activity, smoking cessation, psychosocial care, and immunization. Diabetes care 2015;38 Suppl:S20-S30
43. RefID: 1658. Ameringer KJ, Leventhal AM. Applying the tripartite model of anxiety and depression to cigarette smoking: an integrative review. Nicotine Tob Res 2010;12(12):1183-94.
44. RefID: 1849. Amstadter AB, Broman-Fulks J, Zinzow H, Ruggiero KJ, Cercone J. Internet-based interventions for traumatic stress-related mental health problems: a review and suggestion for future research. Clin Psychol Rev 2009;29(5):410-20.
45. RefID: 3456. Anderson LR. Refining what works in tailoring: Comprehensive meta-analysis of computer-tailored interventions. Dissertation Abstracts International: Section B: The Sciences and Engineering 2012;72(10-B):6366
46. RefID: 1200. Andreas S, Rittmeyer A, Hinterthaner M, Huber RM. Smoking cessation in lung cancer-achievable and effective. Dtsch Arztebl int 2013;110(43):719-24.
47. RefID: 2584. Annamalai A, Singh N, O'Malley SS. Smoking use and cessation among people with serious mental illness. Yale J Biol Med 2015;88(3):271-7. [PMID: http://www.ncbi.nlm.nih.gov/pmc/articles/PMC4553647/pdf/yjbm_88_3_271.pdf]
48. RefID: 2546. Annemans L, Marbaix S, Nackaerts K, Bartsch P. Cost-effectiveness of retreatment with varenicline after failure with or relapse after initial treatment for smoking cessation. Prev Med Rep 2015;2:189-95.
49. RefID: 2877. Ansari S, Ford AC. Initial management of dyspepsia in primary care: An evidence-based approach. Br J Gen Pract 2013;63(614):498-9. [PMID: http://docserver.ingentaconnect.com/deliver/connect/rcgp/09601643/v63n614/s29.pdf?expires=1379740929&id=75556366&titleid=30000031&accname=Elsevier+BV&checksum=30B9037980E7C8B0559EA693DCD72B64]
50. RefID: 704. Anthenelli RM, Benowitz NL, West R, St AL, McRae T, Lawrence D, et al. Neuropsychiatric safety and efficacy of varenicline, bupropion, and nicotine patch in smokers with and without psychiatric disorders (EAGLES): a double-blind, randomised, placebo-controlled clinical trial. Lancet 2016 Jun 18;387(10037):2507-20. [PMID: 27116918]
51. RefID: 1291. Anti-Smoking Promotion Committee of the Japanese Circulation Society. Anti-smoking declaration 2013. Circ J 2013;77(3):559-60.
52. RefID: 3284. Armitage CJ. Is there utility in the transtheoretical model? Br J Health Psychol 2009;14(2):195-210. [PMID: http://docserver.ingentaconnect.com/deliver/connect/bpsoc/1359107x/v14n2/s2.pdf?expires=1245833077&id=50914791&titleid=525&accname=Elsevier+Bibliographic+Databases&checksum=B85AAF229D7EB823E9CAF142391A61C9]
53. RefID: 3131. Aronow WS, Fleg JL, Pepine CJ, Artinian NT, Bakris G, Brown AS, et al. ACCF/AHA 2011 expert consensus document on hypertension in the elderly: A Report of the American College of Cardiology Foundation Task Force on Clinical Expert Consensus Documents Developed in Collaboration with the American Academy of Neurology, American Geriatrics Society, American Society for Preventive Cardiology, American Society of Hypertension, American Society of Nephrology. J Am Soc Hypertens 2011;5(4):259-352.
54. RefID: 1595. Asfar T, Ebbert JO, Klesges RC, Relyea GE. Do smoking reduction interventions promote cessation in smokers not ready to quit? Addict Behav 2011;36(7):764-8.
55. RefID: 3203. Aubin H-J. Management of emergent psychiatric symptoms during smoking cessation. Curr Med Res Opin 2009;25(2):519-25. [PMID: http://www.informapharmascience.com/doi/pdf/10.1185/03007990802707600]
56. RefID: 1192. Aung MN, Yuasa M, Lorga T, Moolphate S, Fukuda H, Kitajima T, et al. Evidence-based new service package vs. routine service package for smoking cessation to prevent high risk patients from cardiovascular diseases (CVD): study protocol for randomized controlled trial. Trials 2013;14:419
57. RefID: 2907. Avery MD. Current resources for evidence-based practice, September/October 2013. J Midwifery Women's Health 2013;58(5):569-73.
58. RefID: 3086. Aveyard P, Bauld L. Incentives for promoting smoking cessation: What we still do not know. Cochrane database of systematic reviews (Online) 2011;(8):ED000027
59. RefID: 1485. Aveyard P, Begh R, Parsons A, West R. Brief opportunistic smoking cessation interventions: a systematic review and meta-analysis to compare advice to quit and offer of assistance. Addiction 2012;107(6):1066-73.
60. RefID: 133. Aveyard P, Lindson N, Tearne S, Adams R, Ahmed K, Alekna R, et al. Nicotine preloading for smoking cessation: the Preloading RCT. Health Technol Assess 2018;22(41):1-84.
61. RefID: 1448. Ayo-Yusuf OA, Burns DM. The complexity of 'harm reduction' with smokeless tobacco as an approach to tobacco control in low-income and middle-income countries. Tob Control 2012;21(2):245-51.
62. RefID: 2599. Ayub H, Obeidat N, Leischow S, Glynn T, Hawari F. Jordan tobacco dependence treatment guidelines: Rationale and development. East Mediterr Health J 2015;21(11):844-50. [PMID: http://applications.emro.who.int/emhj/v21/11/EMHJ_2015_21_11_844_850.pdf?ua=1]
63. RefID: 2576. Aziz UR, Mohamed MHBN, Jamshed S. Safety and effectiveness of electronic cigarettes: A narrative review. Int Med J 2015;22(3):122-31. [PMID: http://uia4.tripod.com/]
64. RefID: 766. Azorin JM, Simon N, Adida M, Belzeaux R. Pharmacological treatment of schizophrenia with comorbid substance use disorder. Expert Opin Pharmacother 2016;17(2):231-53.
65. RefID: 267. Babayan RK. Wound healing and infection in surgery: the pathophysiological impact of smoking, smoking cessation, and nicotine replacement therapy: a systematic review. J Urol 2012;188(6):2243-4.
66. RefID: 1632. Backinger CL, Pilsner AM, Augustson EM, Frydl A, Phillips T, Rowden J. YouTube as a source of quitting smoking information. Tob Control 2011;20(2):119-22.
67. RefID: 1809. Bailey BA, Jones Cole LK. Are obstetricians following best-practice guidelines for addressing pregnancy smoking? Results from northeast Tennessee. South Med J 2009;102(9):894-9.
68. RefID: 3378. Baker AL. Commentary on Mathew et al. (2017): Improving the effectiveness of interventions to reduce smoking among people living with severe mental ill-health. Addiction 2017;112(3):413-4.
69. RefID: 954. Baker TB, Fiore MC. Treating more smokers, more of the time, more successfully. Addiction 2015;110(3):388-9.
70. RefID: 2042. Bakken S, Currie LM, Lee NJ, Roberts WD, Collins SA, Cimino JJ. Integrating evidence into clinical information systems for nursing decision support. Int J Med Inf 2008;77(6):413-20.
71. RefID: 1569. Bakris GL. Recognition, pathogenesis, and treatment of different stages of nephropathy in patients with type 2 diabetes mellitus. Mayo Clin Proc 2011;86(5):444-56.
72. RefID: 2000. Bala MM, Lesniak W, Strzeszynski L. Efficacy of pharmacological methods used for treating tobacco dependence: meta-analysis. Pol Arch Med Wewn 2008;118(1-2):20-8.
73. RefID: 1152. Bam TS, Bellew W, Berezhnova I, Jackson-Morris A, Jones A, Latif E, et al. Position statement on electronic cigarettes or electronic nicotine delivery systems. Int J Tuberc Lung Dis 2014;18(1):5-7.
74. RefID: 2265. Bar-Zeev Y, Lim LL, Bonevski B, Gruppetta M, Gould GS. Nicotine replacement therapy for smoking cessation during pregnancy. Med J Aust 2018;208(1):46-51. [PMID: https://www.mja.com.au/system/files/issues/208_01/10.5694mja17.00446.pdf]
75. RefID: 2449. Barboza JL, Patel R, Patel P, Hudmon KS. An update on the pharmacotherapeutic interventions for smoking cessation. Expert Opin Pharmacother 2016;17(11):1483-96.
76. RefID: 3196. Barros FC, Bhutta ZA, Batra M, Hansen TN, Victora CG, Rubens CE. Global report on preterm birth and stillbirth (3 of 7): Evidence for effectiveness of interventions. BMC Pregnancy Childbirth 2010;10(SUPPL. 1):S3. [PMID: http://www.biomedcentral.com/1471-2393/10?issue=S1]
77. RefID: 2693. Barry AR, Koshman SL, Pearson GJ. Adverse drug reactions: The importance of maintaining pharmacovigilance. Can Pharm J 2014;147(4):233-8. [PMID: http://cph.sagepub.com/content/by/year]
78. RefID: 1533. Barton MK. Smoking cessation telephone quitlines effective regardless of recruitment method. CA Cancer J Clin 2011;61(6):361-2.
79. RefID: 2329. Baskerville NB, Dash D, Shuh A, Wong K, Abramowicz A, Yessis J, et al. Tobacco use cessation interventions for lesbian, gay, bisexual, transgender and queer youth and young adults: A scoping review. Prev Med Rep 2017;6:53-62.
80. RefID: 1530. Batra A. Treatment of tobacco dependence. Dtsch Arztebl int 2011;108(33):555-64.
81. RefID: 3385. Bauld L. Electronic cigarettes and smoking cessation. Nicotine & Tobacco Research 2016;18(10):1925
82. RefID: 437. Bauld L, Graham H, Sinclair L, Flemming K, Naughton F, Ford A, et al. Barriers to and facilitators of smoking cessation in pregnancy and following childbirth: literature review and qualitative study. Health Technol Assess 2017;21(36):1-158.
83. RefID: 1135. Baum HBA, Cagliero E, Berry CA, Mencia WA, Stowell SA, Miller SC. Continuing improvement in type 2 diabetes care through performance-based evaluations. J prim care community health 2014;5(2):107-11.
84. RefID: 2911. Bawagan J. Systematic review wins CMAJ Bruce Squires Award. CMAJ 2013;185(8):643
85. RefID: 3119. Bazzano LA. No effect of folic acid supplementation on cardiovascular events, cancer or mortality after 5 years in people at increased cardiovascular risk, although homocysteine levels are reduced. Evid Based Med 2011;16(4):117-8. [PMID: http://ebm.bmj.com/content/16/4/117.full.pdf]
86. RefID: 1696. Beauchamp A, Peeters A, Tonkin A, Turrell G. Best practice for prevention and treatment of cardiovascular disease through an equity lens: a review. Eur J Cardiovasc Prev Rehabil 2010;17(5):599-606.
87. RefID: 2711. Been JV, Nurmatov UB, Cox B, Nawrot TS, van Schayck CP, Sheikh A. Effect of smoke-free legislation on perinatal and child health: A systematic review and meta-analysis. Obstet Gynecol Surv 2014;69(9):521-3. [PMID: http://journals.lww.com/obgynsurvey]
88. RefID: 2666. Bekki K, Uchiyama S, Ohta K, Inaba Y, Nakagome H, Kunugita N. Carbonyl compounds generated from electronic cigarettes. Int J Environ Res Public Health 2014;11(11):11192-200. [PMID: http://www.mdpi.com/1660-4601/11/11/11192/pdf]
89. RefID: 190. Belita E, Sidani S. Attrition in Smoking Cessation Intervention Studies: A Systematic Review. Can J Nurs Res 2015;47(4):21-40.
90. RefID: 1111. Bellmunt S, Roque M, Osorio D, Pardo H, Escudero JR, Bonfill X. Healthcare quality indicators of peripheral artery disease based on systematic reviews. Eur J Vasc Endovasc Surg 2014;48(1):60-9.
91. RefID: 1272. Belsky DW, Moffitt TE, Baker TB, Biddle AK, Evans JP, Harrington H, et al. Polygenic risk and the developmental progression to heavy, persistent smoking and nicotine dependence: evidence from a 4-decade longitudinal study. JAMA psychiatry 2013;70(5):534-42.
92. RefID: 210. Bennett BL, Deiner M, Pokhrel P. College anti-smoking policies and student smoking behavior: a review of the literature. Tob induc dis 2017;15:11
93. RefID: 1254. Bennett ME, Wilson AL, Genderson M, Saperstein AM. Smoking cessation in people with schizophrenia. Curr Drug Abuse Rev 2013;6(3):180-90.
94. RefID: 2899. Benowitz NL, Prochaska JJ. Smoking cessation after acute myocardial infarction. J Am Coll Cardiol 2013;61(5):533-5.
95. RefID: 2388. Benowitz NL, Burbank AD. Cardiovascular toxicity of nicotine: Implications for electronic cigarette use. Trends Cardiovasc Med 2016;26(6):515-23. [PMID: http://www.elsevier.com/locate/tcm]
96. RefID: 887. Bergen AW, Michel M, Nishita D, Krasnow R, Javitz HS, Conneely KN, et al. Drug Metabolizing Enzyme and Transporter Gene Variation, Nicotine Metabolism, Prospective Abstinence, and Cigarette Consumption. PloS one 2015;10(7):e0126113
97. RefID: 353. Berger I, Mooney-Somers J. Smoking Cessation Programs for Lesbian, Gay, Bisexual, Transgender, and Intersex People: A Content-Based Systematic Review. Nicotine Tob Res 2017;19(12):1408-17.
98. RefID: 3044. Bergman HE, Hunt YM, Augustson E. Smokeless tobacco use in the United States military: A systematic review. Nicotine Tob Res 2012;14(5):507-15.
99. RefID: 1882. Berlin I. Endocrine and metabolic effects of smoking cessation. Curr Med Res Opin 2009;25(2):527-34.
100. RefID: 2407. Bernstein SL. Electronic cigarettes: More light, less heat needed. Lancet Respir Med 2016;4(2):85-7. [PMID: http://www.elsevier.com/journals/the-lancet-respiratory-medicine/2213-2600]
101. RefID: 1629. Berra K. Does nurse case management improve implementation of guidelines for cardiovascular disease risk reduction? J Cardiovasc Nurs 2011;26(2):145-67.
102. RefID: 2523. Bhatia U, Nadkarni A, Murthy P, Rao R, Crome I. Recent advances in treatment for older people with substance use problems: An updated systematic and narrative review. Eur Geriatr Med 2015;6(6):580-6. [PMID: http://www.elsevier.com/wps/find/journaldescription.cws_home/720928/description#description]
103. RefID: 1043. Bhatnagar A, Whitsel LP, Ribisl KM, Bullen C, Chaloupka F, Piano MR, et al. Electronic cigarettes: a policy statement from the American Heart Association. Circulation 2014;130(16):1418-36.
104. RefID: 1507. Bhutani G, Kaushal J, Gupta MC. Critical appraisal of the existing and emerging therapies for smoking cessation. Med J Malaysia 2011;66(5):526-33.
105. RefID: 413. Bialous SA, Sarna L, Wells MJ, Brook JK, Kralikova E, Pankova A, et al. Impact of Online Education on Nurses' Delivery of Smoking Cessation Interventions With Implications for Evidence-Based Practice. Worldviews Evid Based Nurs 2017;14(5):367-76.
106. RefID: 2453. Biddle C. Science and clinical potpourri for your life and your practice. AANA J 2016;84(6):395. [PMID: http://www.aana.com/newsandjournal/20102019/editors-desk-1216-p395.pdf]
107. RefID: 2414. Biddle S. Physical activity and mental health: Evidence is growing. World Psychiatry 2016;15(2):176-7. [PMID: http://onlinelibrary.wiley.com/journal/10.1002/(ISSN)2051-5545]
108. RefID: 1381. Bikdeli B, Barreto-Filho JA. Reducing the cardiovascular disease burden: justified means for getting to the end. Circ Cardiovasc Qual Outcomes 2012;5(4):580-6.
109. RefID: 3271. Bitton A. Web- and computer-based smoking cessation programs are effective for adult smokers. J Clin Outcomes Manage 2009;16(7):301-3. [PMID: http://www.turner-white.com/memberfile.php?PubCode=jcom_jul09_smoking.pdf]
110. RefID: 1728. Black JH. Evidence base and strategies for successful smoking cessation. J Vasc Surg 2010;51(6):1529-37.
111. RefID: 15. Black N, Williams AJ, Javornik N, Scott C, Johnston M, Eisma MC, et al. Enhancing Behavior Change Technique Coding Methods: Identifying Behavioral Targets and Delivery Styles in Smoking Cessation Trials. Ann Behav Med 2018;
112. RefID: 2708. Blake H. Text messaging interventions increase adherence to antiretroviral therapy and smoking cessation. Evid Based Med 2014;19(1):35-6. [PMID: http://ebm.bmj.com/content/19/1/35.full.pdf+html]
113. RefID: 923. Blaser J, Cornuz J. Experts' consensus on use of electronic cigarettes: a Delphi survey from Switzerland. BMJ open 2015;5(4):e007197
114. RefID: 26. Bloom JA, Rashad R, Chatterjee A. The Impact on Mortality and Societal Costs From Smoking Cessation in Aesthetic Plastic Surgery in the United States. Aesthet surg j 2018;
115. RefID: 3871. Blyth A, Maskrey V, Notley C, Barton GR, Brown TJ, Aveyard P, et al. Effectiveness and economic evaluation of self-help educational materials for the prevention of smoking relapse: randomised controlled trial (Structured abstract). Health Technology Assessment Database 2016;(2016 Issue 4):
116. RefID: 875. Blyth A, Maskrey V, Notley C, Barton GR, Brown TJ, Aveyard P, et al. Effectiveness and economic evaluation of self-help educational materials for the prevention of smoking relapse: randomised controlled trial. Health Technol Assess 2015;19(59):1-vi
117. RefID: 1919. Bock BC, Graham AL, Whiteley JA, Stoddard JL. A review of web-assisted tobacco interventions (WATIs). J Med Internet Res 2008;10(5):e39
118. RefID: 1440. Bock C, Diehl K, Schneider S, Diehm C, Litaker D. Behavioral counseling for cardiovascular disease prevention in primary care settings: a systematic review of practice and associated factors. Med Care Res Rev 2012;69(5):495-518.
119. RefID: 811. Bogdanovica I, Agrawal S, Gregory B, Britton J, Leonardi-Bee J, Tobacco Advisory Group of the Royal College of Physicians. What is the quality of smoking cessation advice in guidelines of tobacco-related diseases? Clin Med 2015;15(6):546-9.
120. RefID: 1419. Bolin K. Economic evaluation of smoking-cessation therapies: a critical and systematic review of simulation models. PharmacoEconomics 2012;30(7):551-64.
121. RefID: 2128. Bond CM. Adherence: The Holy Grail? BMJ Qual Saf 2016;25(10):736-8. [PMID: http://qualitysafety.bmj.com/content/by/year]
122. RefID: 1459. Borland R, Partos TR, Cummings KM. Systematic biases in cross-sectional community studies may underestimate the effectiveness of stop-smoking medications. Nicotine Tob Res 2012;14(12):1483-7.
123. RefID: 2580. Born H, Persky M, Kraus DH, Peng R, Amin MR, Branski RC. Electronic cigarettes: A primer for clinicians. Otolaryngol Head Neck Surg 2015;153(1):5-14. [PMID: http://oto.sagepub.com/content/by/year]
124. RefID: 1742. Borrelli B. Smoking cessation: next steps for special populations research and innovative treatments. J Consult Clin Psychol 2010;78(1):1-12.
125. RefID: 1016. Bottorff JL, Haines-Saah R, Kelly MT, Oliffe JL, Torchalla I, Poole N, et al. Gender, smoking and tobacco reduction and cessation: a scoping review. Intern j equity health 2014;13:114
126. RefID: 1146. Bottorff JL, Poole N, Kelly MT, Greaves L, Marcellus L, Jung M. Tobacco and alcohol use in the context of adolescent pregnancy and postpartum: a scoping review of the literature. Health Soc Care Community 2014;22(6):561-74.
127. RefID: 526. Bourke L, Bauld L, Bullen C, Cumberbatch M, Giovannucci E, Islami F, et al. E-cigarettes and Urologic Health: A Collaborative Review of Toxicology, Epidemiology, and Potential Risks. Eur Urol 2017;71(6):915-23.
128. RefID: 320. Boyd M, Wilson N. Just ask Siri? A pilot study comparing smartphone digital assistants and laptop Google searches for smoking cessation advice. PloS one 2018;13(3):e0194811
129. RefID: 2740. Boyle P, Koechlin A, Autier P. Mouthwash and oral malignancy. Oral Dis 2014;20(S1):35-60.
130. RefID: 3515. Brandon TH, Drobes DJ, Ditre JW, Elibero A. Nicotine. 2009;267-93.
131. RefID: 942. Branstetter SA, Lengerich E, Dignan M, Muscat J. Knowledge and perceptions of tobacco-related media in rural Appalachia. RURAL REMOTE HEALTH 2015;15:3136
132. RefID: 95. Breitbarth AK, Morgan J, Jones AL. E-cigarettes-An unintended illicit drug delivery system. Drug Alcohol Depend 2018;192:98-111.
133. RefID: 1911. Brendryen H, Drozd F, Kraft P. A digital smoking cessation program delivered through internet and cell phone without nicotine replacement (happy ending): randomized controlled trial. J Med Internet Res 2008;10(5):e51
134. RefID: 2017. Brendryen H, Kraft P. Happy ending: a randomized controlled trial of a digital multi-media smoking cessation intervention. Addiction 2008;103(3):478-6.
135. RefID: 3498. Brendryen H, Kraft P, Schaalma H. Looking inside the black box: Using intervention mapping to describe the development of the automated smoking cessation intervention 'happy ending.'. J Smok Cessat 2010;5(1):29-56.
136. RefID: 1738. Bright RP. Denial of hepatic transplantation on the basis of smoking: is it ethical? Curr Opin Organ Transplant 2010;15(2):249-53.
137. RefID: 3861. Brinson D. How to increase the delivery of effective smoking cessation treatments in primary care settings: guidance for doctors, nurses, other health professionals and healthcare organisations (Structured abstract). Health Technology Assessment Database 2016;(2016 Issue 4):
138. RefID: 1197. Bristow ST, Wright A, Weaver N, Palmer PR. Does bupropion use lead to better outcome in smoking cessation patients motivated to stop smoking compared to the nicotine patch alone? J Okla State Med Assoc 2013;106(8):321-2.
139. RefID: 2601. Brose LS. E-cigarettes may help smokers stop or reduce smoking. Evid Based Med 2015;20(4):134. [PMID: http://ebm.bmj.com/content/20/4/134.full.pdf+html]
140. RefID: 1546. Brose LS, West R, McDermott MS, Fidler JA, Croghan E, McEwen A. What makes for an effective stop-smoking service? Thorax 2011;66(10):924-6.
141. RefID: 1588. Brott TG, Halperin JL, Abbara S, Bacharach JM, Barr JD, Bush RL, et al. 2011 ASA/ACCF/AHA/AANN/AANS/ACR/ASNR/CNS/SAIP/ SCAI/SIR/SNIS/SVM/SVS guideline on the management of patients with extracranial carotid and vertebral artery disease: executive summary: a report of the American College of Cardiology Foundation/American Heart Association Task Force on Practice Guidelines, and the American Stroke Association, American Association of Neuroscience Nurses, American Association of Neurological Surgeons, American College of Radiology, American Society of Neuroradiology, Congress of Neurological Surgeons, Society of Atherosclerosis Imaging and Prevention, Society for Cardiovascular Angiography and Interventions, Society of Interventional Radiology, Society of NeuroInterventional Surgery, Society for Vascular Medicine, and Society for Vascular Surgery. Vasc Med 2011;16(1):35-77.
142. RefID: 1427. Brown D, Portlock J, Rutter P. Review of services provided by pharmacies that promote healthy living. Int J Clin Pharm 2012;34(3):399-409.
143. RefID: 63. Brown J, Brown B, Schwiebert P, Ramakrisnan K, McCarthy LH. In adult smokers unwilling or unable to quit, does changing from tobacco cigarettes to electronic cigarettes decrease the incidence of negative health effects associated with smoking tobacco? A Clin-IQ. J patient cent res rev 2014;1(2):99-101.
144. RefID: 1717. Bruin JE, Gerstein HC, Holloway AC. Long-term consequences of fetal and neonatal nicotine exposure: a critical review. Toxicol Sci 2010;116(2):364-74.
145. RefID: 1433. Brunzell DH. Preclinical evidence that activation of mesolimbic alpha 6 subunit containing nicotinic acetylcholine receptors supports nicotine addiction phenotype. Nicotine Tob Res 2012;14(11):1258-69.
146. RefID: 1014. Brusse C, Gardner K, McAullay D, Dowden M. Social media and mobile apps for health promotion in Australian Indigenous populations: scoping review. J Med Internet Res 2014;16(12):e280
147. RefID: 1944. Buchanan L, Likness S. Evidence-based practice to assist women in hospital settings to quit smoking and reduce cardiovascular disease risk. J Cardiovasc Nurs 2008;23(5):397-406.
148. RefID: 1212. Buhse S, Heller T, Kasper J, Muhlhauser I, Muller UA, Lehmann T, et al. An evidence-based shared decision making programme on the prevention of myocardial infarction in type 2 diabetes: protocol of a randomised-controlled trial. BMC Fam Pract 2013;14:155
149. RefID: 1973. Bullen C, Walker N, Whittaker R, McRobbie H, Glover M, Fraser T. Smoking cessation competencies for health workers in New Zealand. N Z Med J 2008;121(1276):48-56.
150. RefID: 1701. Bullen C, Howe C, Lin RB, Grigg M, Laugesen M, McRobbie H, et al. Pre-cessation nicotine replacement therapy: pragmatic randomized trial. Addiction 2010;105(8):1474-83.
151. RefID: 3511. Burgess DJ, Fu SS, van Ryn M. Potential unintended consequences of tobacco-control policies on mothers who smoke: A review of the literature. Am J Prev Med 2009;37(2,Suppl):S151-S158
152. RefID: 3028. Burniston J, Eftekhari F, Hrabi S, Worsley R, Dean E. Health behaviour change and lifestyle-related condition prevalence: Comparison of two epochs based on systematic review of the physical therapy literature. Hong Kong Physiother J 2012;30(2):44-56.
153. RefID: 3497. Burrows J, Carlisle J. They don't want it ramming down their throats. Learning from the perspectives of current and ex-smokers with smoking-related illness to improve communication in primary care: A qualitative study. Primary Health Care Research and Development 2010;11(3):206-14.
154. RefID: 1145. Burstyn I. Peering through the mist: systematic review of what the chemistry of contaminants in electronic cigarettes tells us about health risks. BMC public health 2014;14:18
155. RefID: 1436. Burton C. Heavy tailed distributions of effect sizes in systematic reviews of complex interventions. PloS one 2012;7(3):e34222
156. RefID: 3083. Busch AM, Borrelli B, Leventhal AM. The Relationship between Smoking and Depression Post-Acute Coronary Syndrome. Curr Cardiovasc Risk Rep 2011;5(6):510-8.
157. RefID: 490. Bush T, Lovejoy JC, Deprey M, Carpenter KM. The effect of tobacco cessation on weight gain, obesity, and diabetes risk. Obesity (Silver Spring) 2016;24(9):1834-41.
158. RefID: 546. Butler KM, Hedgecock S, Record RA, Derifield S, McGinn C, Murray D, et al. An evidence-based cessation strategy using rural smokers' experiences with tobacco. Nurs Clin North Am 2012;47(1):31-43.
159. RefID: 1464. Butler KM, Fallin A, Ridner SL. Evidence-based smoking cessation for college students. Nurs Clin North Am 2012;47(1):21-30.
160. RefID: 2432. Cadier B, Durand-Zaleski I, Thomas D, Chevreul K. Cost effectiveness of free access to smoking cessation treatment in France considering the economic burden of smoking-related diseases. PloS one 2016;11(2):e0148750. [PMID: http://www.plosone.org/article/fetchObject.action?uri=info:doi/10.1371/journal.pone.0148750&representation=PDF]
161. RefID: 3893. CADTH. Nicotine replacement therapy for smoking cessation or reduction: a review of the clinical evidence (Structured abstract). Health Technology Assessment Database 2016;(2016 Issue 4):
162. RefID: 3895. CADTH. Smoking cessation prior to surgery: clinical evidence (Structured abstract). Health Technology Assessment Database 2016;(2016 Issue 4):
163. RefID: 3890. CADTH. Indigenous knowledge for smoking cessation: benefits and effectiveness (Structured abstract). Health Technology Assessment Database 2016;(2016 Issue 4):
164. RefID: 3884. CADTH. Electronic cigarettes: a review of the clinical evidence and safety (Structured abstract). Health Technology Assessment Database 2016;(2016 Issue 4):
165. RefID: 3883. CADTH. Pharmacologic smoking cessation interventions for patients with cardiovascular conditions: a review of the safety and guidelines (Structured abstract). Health Technology Assessment Database 2016;(2016 Issue 4):
166. RefID: 1626. Cahn Z, Siegel M. Electronic cigarettes as a harm reduction strategy for tobacco control: a step forward or a repeat of past mistakes? J Public Health Policy 2011;32(1):16-31.
167. RefID: 702. Caldwell BO, Crane J. Combination Nicotine Metered Dose Inhaler and Nicotine Patch for Smoking Cessation: A Randomized Controlled Trial. Nicotine Tob Res 2016;18(10):1944-51.
168. RefID: 224. Cambon L, Bergman P, Le Faou A, Vincent I, Le Maitre B, Pasquereau A, et al. Study protocol for a pragmatic randomised controlled trial evaluating efficacy of a smoking cessation e-'Tabac Info Service': ee-TIS trial. BMJ open 2017;7(2):e013604
169. RefID: 727. Campbell-Heider N, Snow D. Teen Use of Electronic Cigarettes: What Does the Research Tell Us? J ADDICT NURS 2016;27(1):56-61.
170. RefID: 2903. Campbell-Scherer D. Multitreatment comparison meta-analysis: Promise and peril. Evid Based Med 2013;18(6):201-3. [PMID: http://ebm.bmj.com/content/18/6/201.full.pdf+html]
171. RefID: 114. Campbell KA, Fergie L, Coleman-Haynes T, Cooper S, Lorencatto F, Ussher M, et al. Improving Behavioral Support for Smoking Cessation in Pregnancy: What Are the Barriers to Stopping and Which Behavior Change Techniques Can Influence These? Application of Theoretical Domains Framework. Int J Environ Res Public Health 2018;15(2):
172. RefID: 3524. Campbell R, Starkey F, Holliday J, Audrey S, Bloor M, Parry-Langdon N, et al. An informal school-based peer-led intervention for smoking prevention in adolescence (ASSIST): A cluster randomised trial. The Lancet 2008;371(9624):1595-602.
173. RefID: 2009. Campion J, Lawn S, Brownlie A, Hunter E, Gynther B, Pols R. Implementing smoke-free policies in mental health inpatient units: learning from unsuccessful experience. Australas psychiatry 2008;16(2):92-7.
174. RefID: 3876. Canadian Agency for Drugs and Technologies in Health. Varenicline for smoking cessation in patients with psychiatric illness: a review of the risks (Structured abstract). Health Technology Assessment Database 2016;(2016 Issue 4):
175. RefID: 2471. Cappella JN. Advancing the evidentiary base for tobacco warning labels: A commentary. Soc Sci Med 2016;164:130-2. [PMID: http://www.elsevier.com/locate/socscimed]
176. RefID: 1609. Carr SM, Lhussier M, Forster N, Geddes L, Deane K, Pennington M, et al. An evidence synthesis of qualitative and quantitative research on component intervention techniques, effectiveness, cost-effectiveness, equity and acceptability of different versions of health-related lifestyle advisor role in improving health. Health Technol Assess 2011;15(9):iii-284
177. RefID: 3426. Cassidy KB. Health system processes, clinician attitudes, and referrals to tobacco treatment programs. Dissertation Abstracts International: Section B: The Sciences and Engineering 2014;75(5-B(E)):No-Specified
178. RefID: 324. Castaldelli-Maia JM, da Silva NR, Campos MR, Moura HF, Zabert G, Champagne BM, et al. Implementing evidence-based smoking cessation treatment in psychosocial care units (CAPS) in Brazil. Int J Soc Psychiatry 2017;63(8):669-73.
179. RefID: 2205. Cather C, Pachas GN, Cieslak KM, Evins AE. Achieving Smoking Cessation in Individuals with Schizophrenia: Special Considerations. CNS drugs 2017;31(6):471-81. [PMID: http://rd.springer.com/journal/40263]
180. RefID: 1396. Catley D, Harris KJ, Goggin K, Richter K, Williams K, Patten C, et al. Motivational Interviewing for encouraging quit attempts among unmotivated smokers: study protocol of a randomized, controlled, efficacy trial. BMC public health 2012;12:456
181. RefID: 1107. Cavichio BV, Pompeo DA, Oller GASAdO, Rossi LA. [Duration of smoking cessation for the prevention of surgical wound healing complications]. Rev Esc Enferm USP 2014;48(1):174-80.
182. RefID: 900. Cawkwell PB, Blaum C, Sherman SE. Pharmacological Smoking Cessation Therapies in Older Adults: A Review of the Evidence. Drugs Aging 2015;32(6):443-51.
183. RefID: 1498. Cengelli S, O'Loughlin J, Lauzon B, Cornuz J. A systematic review of longitudinal population-based studies on the predictors of smoking cessation in adolescent and young adult smokers. Tob Control 2012;21(3):355-62.
184. RefID: 3705. Centre for Reviews and Dissemination. Smoking cessation interventions among individuals in methadone maintenance: a brief review (Structured abstract). Database of Abstracts of Reviews of Effects 2015;(2):
185. RefID: 3758. Centre for Reviews and Dissemination. Low-income groups and behaviour change interventions: a review of intervention content and effectiveness (Structured abstract). Database of Abstracts of Reviews of Effects 2015;(2):
186. RefID: 3677. Centre for Reviews and Dissemination. Anti-tobacco policy in schools: upcoming preventive strategy or prevention myth? A review of 31 studies (Provisional abstract). Database of Abstracts of Reviews of Effects 2015;(2):
187. RefID: 3601. Centre for Reviews and Dissemination. Using mass media campaigns to reduce youth tobacco use: a review (Provisional abstract). Database of Abstracts of Reviews of Effects 2015;(2):
188. RefID: 1365. Cerimele JM, Durango A. Does varenicline worsen psychiatric symptoms in patients with schizophrenia or schizoaffective disorder? A review of published studies. J Clin Psychiatry 2012;73(8):e1039-e1047
189. RefID: 1694. Chadban S, Howell M, Twigg S, Thomas M, Jerums G, Cass A, et al. The CARI guidelines. Prevention and management of chronic kidney disease in type 2 diabetes. Nephrology 2010;15 Suppl 1:S162-S194
190. RefID: 1628. Chaloupka FJ, Straif K, Leon ME, Working Group IAfRoC. Effectiveness of tax and price policies in tobacco control. Tob Control 2011;20(3):235-8.
191. RefID: 1449. Chaloupka FJ, Yurekli A, Fong GT. Tobacco taxes as a tobacco control strategy. Tob Control 2012;21(2):172-80.
192. RefID: 463. Chamberlain C, Perlen S, Brennan S, Rychetnik L, Thomas D, Maddox R, et al. Evidence for a comprehensive approach to Aboriginal tobacco control to maintain the decline in smoking: an overview of reviews among Indigenous peoples. Syst rev 2017;6(1):135
193. RefID: 1230. Chan K, Chandler J, Cheong K, Giam PE, Kanagalingam D, Lee LL, et al. Health promotion board-ministry of health clinical practice guidelines: treating tobacco use and dependence. Singapore Med J 2013;54(7):411-6.
194. RefID: 1444. Chaney SE, Sheriff S. Evidence-based treatments for smoking cessation. Nurse Pract 2012;37(4):24-2.
195. RefID: 215. Chang EHE, Braith A, Hitsman B, Schnoll RA. Treating Nicotine Dependence and Preventing Smoking Relapse in Cancer Patients. Expert Rev Qual Life Cancer Care 2017;2(1):23-39.
196. RefID: 1304. Chang JC, Alexander SC, Holland CL, Arnold RM, Landsittel D, Tulsky JA, et al. Smoking is bad for babies: obstetric care providers' use of best practice smoking cessation counseling techniques. Am J Health Promot 2013;27(3):170-6.
197. RefID: 2139. Chatterjee K, Alzghoul B, Innabi A, Meena N. Is vaping a gateway to smoking: A review of the longitudinal studies. Int J Adolesc Med Health 2018;30(3):20160033. [PMID: http://www.degruyter.com/view/j/ijamh?rskey=qYlGDI&result=1&q=International%20Journal%20of%20Adolescent%20Medicine%20and%20Health]
198. RefID: 1286. Chatziefstratiou A, Giakoumidakis K, Brokalaki H. Cardiac rehabilitation outcomes: modifiable risk factors. Br J Nurs 2013;22(4):200-7.
199. RefID: 299. Chehab OM, Dakik HA. Interventions for smoking cessation in patients admitted with Acute Coronary Syndrome: a review. Postgrad Med J 2018;94(1108):116-20.
200. RefID: 256. Chelladurai Y, Singh S. Varenicline and cardiovascular adverse events: a perspective review. Ther adv drug saf 2014;5(4):167-72.
201. RefID: 596. Chen J, Bullen C, Dirks K. A Comparative Health Risk Assessment of Electronic Cigarettes and Conventional Cigarettes. Int J Environ Res Public Health 2017;14(4):
202. RefID: 184. Chen LS, Bierut LJ. Genomics and personalized medicine: CHRNA5-CHRNA3-CHRNB4 and smoking cessation treatment. J FOOD DRUG ANAL 2013;21(4):S87-S90
203. RefID: 183. Chen LS, Horton A, Bierut L. Pathways to precision medicine in smoking cessation treatments. Neurosci Lett 2018;669:83-92.
204. RefID: 147. Cheung KL, Wijnen BFM, Hiligsmann M, Coyle K, Coyle D, Pokhrel S, et al. Is it cost-effective to provide internet-based interventions to complement the current provision of smoking cessation services in the Netherlands? An analysis based on the EQUIPTMOD. Addiction 2018;113 Suppl 1:87-95.
205. RefID: 819. Choi HD, Shin WG. Association between catechol-O-methyltransferase (COMT) Val/Met genotype and smoking cessation treatment with nicotine: a meta-analysis. Pharmacogenomics 2015;16(16):1879-85.
206. RefID: 3013. Chow WB, Rosenthal RA, Merkow RP, Ko CY, Esnaola NF. Optimal preoperative assessment of the geriatric surgical patient: A best practices guideline from the American college of surgeons national surgical quality improvement program and the American geriatrics society. J Am Coll Surg 2012;215(4):453-66.
207. RefID: 881. Christiansen BA, Reeder KM, TerBeek EG, Fiore MC, Baker TB. Motivating Low Socioeconomic Status Smokers to Accept Evidence-Based Smoking Cessation Treatment: A Brief Intervention for the Community Agency Setting. Nicotine Tob Res 2015;17(8):1002-11.
208. RefID: 1149. Christie J. Interventions for recruiting smokers into cessation programmes: a review summary. Public Health Nurs 2014;31(1):55-7.
209. RefID: 67. Cioe PA. Smoking Cessation Interventions in HIV-Infected Adults in North America: A Literature Review. J addict behav ther rehabil 2013;2(3):1000112
210. RefID: 794. Civljak M, Tudor Car L, Skara S, Oreskovic S. The Gap between the Knowledge and Current Practices--A Case of Tobacco Control Programs in Croatia. Coll Antropol 2015;39(3):803-8.
211. RefID: 3031. Clair C, Rigotti NA. Stopping smoking in the weeks prior to surgery has no effect on the risk of postoperative complications. Evid Based Med 2012;17(3):101-2. [PMID: http://ebm.bmj.com/content/17/3/101.full.pdf+html]
212. RefID: 1697. Clark AM, Haykowsky M, Kryworuchko J, MacClure T, Scott J, DesMeules M, et al. A meta-analysis of randomized control trials of home-based secondary prevention programs for coronary artery disease. Eur J Cardiovasc Prev Rehabil 2010;17(3):261-70.
213. RefID: 1581. Clark BJ, Moss M. Secondary prevention in the intensive care unit: does intensive care unit admission represent a "teachable moment?". Crit Care Med 2011;39(6):1500-6.
214. RefID: 2533. Clark L, Ronaldson S, Dyson L, Hewitt C, Torgerson D, Adamson J. Electronic prompts significantly increase response rates to postal questionnaires: A randomized trial within a randomized trial and meta-analysis. J Clin Epidemiol 2015;68(12):1446-50. [PMID: http://www.elsevier.com/locate/jclinepi]
215. RefID: 3395. Clark MA, Gorelick JJ, Sicks JD, Park ER, Graham AL, Abrams DB, et al. The relations between false positive and negative screens and smoking cessation and relapse in the National Lung Screening Trial: Implications for public health. Nicotine & Tobacco Research 2016;18(1):17-24.
216. RefID: 531. Clinical Practice Guideline Treating Tobacco Use and Dependence. A clinical practice guideline for treating tobacco use and dependence: 2008 update. A U.S. Public Health Service report. Am J Prev Med 2008;35(2):158-76.
217. RefID: 1576. Cluss PA, Levine MD, Landsittel D. The Pittsburgh STOP program: disseminating an evidence-informed intervention for low-income pregnant smokers. Am J Health Promot 2011;25(5 Suppl):S75-S81
218. RefID: 1088. Cobb CO, Graham AL. Use of non-assigned interventions in a randomized trial of internet and telephone treatment for smoking cessation. Nicotine Tob Res 2014;16(10):1289-97.
219. RefID: 2381. Cobb NK, Sonti R. E-cigarettes: The science behind the smoke and mirrors. Respir Care 2016;61(8):1122-8. [PMID: http://rc.rcjournal.com/content/61/8/1122.full.pdf]
220. RefID: 1137. Cobb NK, Jacobs MA, Saul J, Wileyto EP, Graham AL. Diffusion of an evidence-based smoking cessation intervention through Facebook: a randomised controlled trial study protocol. BMJ open 2014;4(1):e004089
221. RefID: 611. Cobb NK, Jacobs MA, Wileyto P, Valente T, Graham AL. Diffusion of an Evidence-Based Smoking Cessation Intervention Through Facebook: A Randomized Controlled Trial. Am J Public Health 2016;106(6):1130-5.
222. RefID: 1022. Codern-Bove N, Pujol-Ribera E, Pla M, Gonzalez-Bonilla J, Granollers S, Ballve JL, et al. Motivational interviewing interactions and the primary health care challenges presented by smokers with low motivation to stop smoking: a conversation analysis. BMC public health 2014;14:1225
223. RefID: 2003. Coleman T. Reducing harm from tobacco smoke exposure during pregnancy. Birth Defects Res Part C Embryo Today 2008;84(1):73-9.
224. RefID: 1446. Coleman T, Cooper S, Thornton JG, Grainge MJ, Watts K, Britton J, et al. A randomized trial of nicotine-replacement therapy patches in pregnancy. N Engl J Med 2012;366(9):808-18.
225. RefID: 1349. Colivicchi F, Mocini D, Uguccioni M, Strano S, Imperoli G, Santini M, et al. Smoking cessation interventions after acute coronary syndromes. Results of a cross-sectional survey in the Lazio Region of Italy. Monaldi Arch Chest Dis 2012;78(2):85-8.
226. RefID: 45. Collins L, Glasser AM, Abudayyeh H, Pearson JL, Villanti AC. E-Cigarette Marketing and Communication: How E-Cigarette Companies Market E-Cigarettes and the Public Engages with E-cigarette Information. Nicotine Tob Res 2018;
227. RefID: 74. Collins SE, Witkiewitz K, Kirouac M, Marlatt GA. Preventing Relapse Following Smoking Cessation. Curr Cardiovasc Risk Rep 2010;4(6):421-8.
228. RefID: 598. Colomar M, Tong VT, Morello P, Farr SL, Lawsin C, Dietz PM, et al. Barriers and promoters of an evidenced-based smoking cessation counseling during prenatal care in Argentina and Uruguay. Matern Child Health J 2015;19(7):1481-9.
229. RefID: 2199. Cook JL, Green CR, de la Ronde S, Dell CA, Graves L, Morgan L, et al. Screening and Management of Substance Use in Pregnancy: A Review. J Obstet Gynaecol Can 2017;39(10):897-905. [PMID: http://www.journals.elsevier.com/journal-of-obstetrics-and-gynaecology-canada/]
230. RefID: 2703. Cooper S, Lewis S, Thornton JG, Marlow N, Watts K, Britton J, et al. The SNAP trial: A randomised placebo-controlled trial of nicotine replacement therapy in pregnancy - Clinical effectiveness and safety until 2 years after delivery, with economic evaluation. Health Technol Assess 2014;18(54):1-128. [PMID: http://www.journalslibrary.nihr.ac.uk/__data/assets/pdf_file/0009/124569/FullReport-hta18540.pdf]
231. RefID: 400. Cooper SJ, Reynolds GP, With expert co-authors (in alphabetical order), Barnes T, England E, Haddad PM, et al. BAP guidelines on the management of weight gain, metabolic disturbances and cardiovascular risk associated with psychosis and antipsychotic drug treatment. J Psychopharmacol 2016;30(8):717-48.
232. RefID: 1052. Cooper S, Taggar J, Lewis S, Marlow N, Dickinson A, Whitemore R, et al. Effect of nicotine patches in pregnancy on infant and maternal outcomes at 2 years: follow-up from the randomised, double-blind, placebo-controlled SNAP trial. Lancet Respir Med 2014;2(9):728-37.
233. RefID: 1348. Corbett C, Armstrong MJ, Neuberger J. Tobacco smoking and solid organ transplantation. Transplantation 2012;94(10):979-87.
234. RefID: 2314. Corcos J, Przydacz M, Campeau L, Gray G, Hickling D, Honeine C, et al. CUA guideline on adult overactive bladder. Can Urol Assoc J 2017;11(5):E142-E173. [PMID: http://www.cuaj.ca/index.php/journal/article/view/4586/3143]
235. RefID: 2865. Corlateanu A, Montanari G, Mathioudakis AG, Botnaru V, Siafakas N. Management of stable COPD: An update. Curr Respir Med Rev 2013;9(6):352-9. [PMID: http://www.benthamdirect.org/pages/all_b_bypublication.php]
236. RefID: 31. Cornacchione Ross J, Noar SM, Sutfin EL. Systematic Review of Health Communication for Non-Cigarette Tobacco Products. Health Commun 2017;1-9.
237. RefID: 392. Correa JB, Ariel I, Menzie NS, Brandon TH. Documenting the emergence of electronic nicotine delivery systems as a disruptive technology in nicotine and tobacco science. Addict Behav 2017;65:179-84.
238. RefID: 3090. Cosgrove KP, Esterlis I, Mason GF, Bois F, O'Malley SS, Krystal JH. Invited review: Neuroimaging insights into the role of cortical GABA systems and the influence of nicotine on the recovery from alcohol dependence. Neuropharmacology 2011;60(7-8):1318-25.
239. RefID: 234. Coughlin S, Thind H, Liu B, Champagne N, Jacobs M, Massey RI. Mobile Phone Apps for Preventing Cancer Through Educational and Behavioral Interventions: State of the Art and Remaining Challenges. JMIR Mhealth Uhealth 2016;4(2):e69
240. RefID: 1800. Coups EJ, Dhingra LK, Heckman CJ, Manne SL. Receipt of provider advice for smoking cessation and use of smoking cessation treatments among cancer survivors. J Gen Intern Med 2009;24 Suppl 2:S480-S486
241. RefID: 894. Courtney RJ, Naicker S, Shakeshaft A, Clare P, Martire KA, Mattick RP. Smoking Cessation among Low-Socioeconomic Status and Disadvantaged Population Groups: A Systematic Review of Research Output. Int J Environ Res Public Health 2015;12(6):6403-22.
242. RefID: 1578. Cox LS, Okuyemi K, Choi WS, Ahluwalia JS. A review of tobacco use treatments in U.S. ethnic minority populations. Am J Health Promot 2011;25(5 Suppl):S11-S30
243. RefID: 746. Cross E, Garrison S, Kolber MR. Electronic cigarettes: help, hurt, or hype? Can Fam Physician 2016;62(1):51
244. RefID: 3418. Crossland N, Thomson G, Morgan H, Dombrowski SU, Hoddinott P. Incentives for breastfeeding and for smoking cessation in pregnancy: An exploration of types and meanings. Social Science & Medicine 2015;128:10-7.
245. RefID: 2456. Crossman JM. Planning, practising and prioritising wellness through an integrative behaviour change plan. Health Educ J 2016;75(7):823-32. [PMID: http://www.sagepub.co.uk/journal.aspx?pid=107639]
246. RefID: 561. Crowe SD, Gregg LC, DeFrancesco MS. Obesity and Tobacco Cessation Toolkits: Practical Tips and Tools to Save Lives. Obstet Gynecol 2016;128(6):1314-9.
247. RefID: 1123. Csikesz NG, Gartman EJ. New developments in the assessment of COPD: early diagnosis is key. Int J Chron Obstruct Pulmon Dis 2014;9:277-86.
248. RefID: 537. Cumberbatch MG, Rota M, Catto JWF, La Vecchia C. The Role of Tobacco Smoke in Bladder and Kidney Carcinogenesis: A Comparison of Exposures and Meta-analysis of Incidence and Mortality Risks. Eur Urol 2016;70(3):458-66.
249. RefID: 1279. Cupertino AP, Hunt JJ, Gajewski BJ, Jiang Y, Marquis J, Friedmann PD, et al. The index of tobacco treatment quality: development of a tool to assess evidence-based treatment in a national sample of drug treatment facilities. Subst Abuse Treat Prev Policy 2013;8:13
250. RefID: 2034. Curry SJ, Keller PA, Orleans CT, Fiore MC. The role of health care systems in increased tobacco cessation. Annu Rev Public Health 2008;29:411-28.
251. RefID: 579. Czoli CD, Fong GT, Mays D, Hammond D. How do consumers perceive differences in risk across nicotine products? A review of relative risk perceptions across smokeless tobacco, e-cigarettes, nicotine replacement therapy and combustible cigarettes. Tob Control 2017;26(e1):e49-e58
252. RefID: 1577. D'Silva J, Schillo BA, Sandman NR, Leonard TL, Boyle RG. Evaluation of a tailored approach for tobacco dependence treatment for American Indians. Am J Health Promot 2011;25(5 Suppl):S66-S69
253. RefID: 1504. D'Souza MS, Markou A. Schizophrenia and tobacco smoking comorbidity: nAChR agonists in the treatment of schizophrenia-associated cognitive deficits. Neuropharmacology 2012;62(3):1564-73.
254. RefID: 1112. Dai CL, Sharma M. Between inhale and exhale: yoga as an intervention in smoking cessation. J Evid Based Complementary Altern Med 2014;19(2):144-9.
255. RefID: 2290. Dalal F, Dalal HM, Voukalis C, Gandhi MM. Management of patients after primary percutaneous coronary intervention for myocardial infarction. BMJ (Online) 2017;358:j3237. [PMID: http://www.bmj.com/]
256. RefID: 2556. Damluji AA, Ramireddy A, Otalvaro L, Forman DE. Secondary cardiovascular prevention in older adults: An evidence based review. J Geriatr Cardiol 2015;12(5):459-64. [PMID: http://www.jgc301.com/ch/reader/create_pdf.aspx?file_no=20150917002&year_id=2015&quarter_id=5&falg=1]
257. RefID: 430. Danan ER, Joseph AM, Sherman SE, Burgess DJ, Noorbaloochi S, Clothier B, et al. Does Motivation Matter? Analysis of a Randomized Trial of Proactive Outreach to VA Smokers. J Gen Intern Med 2016;31(8):878-87.
258. RefID: 1051. Danielsson AK, Eriksson AK, Allebeck P. Technology-based support via telephone or web: a systematic review of the effects on smoking, alcohol use and gambling. Addict Behav 2014;39(12):1846-68.
259. RefID: 1055. Darville A, Hahn EJ. Hardcore smokers: what do we know? Addict Behav 2014;39(12):1706-12.
260. RefID: 3427. Darville A. Medically ill smokers and planning to quit. Dissertation Abstracts International: Section B: The Sciences and Engineering 2014;75(5-B(E)):No-Specified
261. RefID: 1490. David EA, Marshall MB. Physiologic evaluation of lung resection candidates. Thorac Surg Clin 2012;22(1):47-vi
262. RefID: 3140. David HL, Smith PK, Bittl JA, Bridges CR, Byrne JG, Cigarroa JE, et al. 2011 ACCF/AHA guideline for coronary artery bypass graft surgery a report of the American College of Cardiology Foundation/American Heart Association Task Force on Practice Guidelines. Circulation 2011;124(23):e652-e735
263. RefID: 3107. David SP, Johnstone EC, Churchman M, Aveyard P, Murphy MFG, Munafo MR. Pharmacogenetics of smoking cessation in general practice: Results from the Patch II and Patch in Practice trials. Nicotine Tob Res 2011;13(3):157-67.
264. RefID: 1979. David SP, Johnstone EC, Murphy MFG, Aveyard P, Guo B, Lerman C, et al. Genetic variation in the serotonin pathway and smoking cessation with nicotine replacement therapy: new data from the Patch in Practice trial and pooled analyses. Drug Alcohol Depend 2008;98(1-2):77-85.
265. RefID: 2458. David T, Tharyan P. Electronic cigarettes for smoking cessation and reduction. Summary of the evidence and implications for public health programmes. Clin Epidemiol Global Health 2016;4(3):146-50. [PMID: http://www.elsevier.com/journals/clinical-epidemiology-and-global-health/2213-3984]
266. RefID: 1190. Davidson EM, Liu JJ, Bhopal R, White M, Johnson MRD, Netto G, et al. Behavior change interventions to improve the health of racial and ethnic minority populations: a tool kit of adaptation approaches. Milbank Q 2013;91(4):811-51.
267. RefID: 1173. Davidson EM, Liu JJ, Bhopal RS, White M, Johnson MRD, Netto G, et al. Consideration of ethnicity in guidelines and systematic reviews promoting lifestyle interventions: a thematic analysis. Eur J Public Health 2014;24(3):508-13.
268. RefID: 2375. Davis DR, Kurti AN, Skelly JM, Redner R, White TJ, Higgins ST. A review of the literature on contingency management in the treatment of substance use disorders, 2009-2014. Prev Med 2016;92:36-46. [PMID: http://www.elsevier.com/inca/publications/store/6/2/2/9/3/4/index.htt]
269. RefID: 1873. Davis D, Galbraith R, American College of Chest Physicians Health and Science Policy Committee. Continuing medical education effect on practice performance: effectiveness of continuing medical education: American College of Chest Physicians Evidence-Based Educational Guidelines. Chest 2009;135(3 Suppl):42S-8S.
270. RefID: 447. De Hert M, Vancampfort D, Correll CU, Mercken V, Peuskens J, Sweers K, et al. Guidelines for screening and monitoring of cardiometabolic risk in schizophrenia: systematic evaluation. Br J Psychiatry 2011;199(2):99-105.
271. RefID: 1029. De Long NE, Barra NG, Hardy DB, Holloway AC. Is it safe to use smoking cessation therapeutics during pregnancy? Expert Opin Drug Saf 2014;13(12):1721-31.
272. RefID: 1954. de Moor JS, Elder K, Emmons KM. Smoking prevention and cessation interventions for cancer survivors. Semin Oncol Nurs 2008;24(3):180-92.
273. RefID: 242. De Nunzio C, Andriole GL, Thompson IMJ, Freedland SJ. Smoking and Prostate Cancer: A Systematic Review. Eur Urol Focus 2015;1(1):28-38.
274. RefID: 3358. de Ridder D, Adriaanse M, Fujita K. The Routledge international handbook of self-control in health and well-being. 2018;
275. RefID: 390. de Ruijter D, Smit ES, de Vries H, Goossens L, Hoving C. Understanding Dutch practice nurses' adherence to evidence-based smoking cessation guidelines and their needs for web-based adherence support: results from semistructured interviews. BMJ open 2017;7(3):e014154
276. RefID: 436. de Ruijter D, Smit ES, de Vries H, Hoving C. Dutch practice nurses' adherence to evidence-based smoking cessation treatment guidelines. Fam Pract 2017;34(6):685-91.
277. RefID: 1241. de Viron S, Malats N, Van der Heyden J, Van Oyen H, Brand A. Environmental and genomic factors as well as interventions influencing smoking cessation: a systematic review of reviews and a proposed working model. Public health genomics 2013;16(4):159-73.
278. RefID: 2730. Dean SV, Lassi ZS, Imam AM, Bhutta ZA. Preconception care: Closing the gap in the continuum of care to accelerate improvements in maternal, newborn and child health. Reprod Health 2014;11(Supplement 3):S1. [PMID: http://www.reproductive-health-journal.com/home/]
279. RefID: 1970. Decramer M, Nici L, Nardini S, Reardon J, Rochester CL, Sanguinetti CM, et al. Targeting the COPD exacerbation. Respir Med 2008;102 Suppl 1:S3-15.
280. RefID: 699. Deleanu OC, Pocora D, Mihalcuta S, Ulmeanu R, Zaharie AM, Mihaltan FD. Influence of smoking on sleep and obstructive sleep apnea syndrome. Pneumologia 2016;65(1):28-35.
281. RefID: 1295. Derlon V, Wirth N, Martinet PY, McNelis U, Minary L, Boileau S, et al. [Management of smoking in the perioperative period: survey of practices amongst anaesthetists in Lorraine]. Ann Fr Anesth Reanim 2013;32(2):89-93.
282. RefID: 2061. Devine KA, Viola AS, Coups EJ, Wu YP. Digital health interventions for adolescent and young adult cancer survivors. JCO Clin Cancer Inform 2018;2018(2):1-15. [PMID: http://ascopubs.org/doi/pdfdirect/10.1200/CCI.17.00138]
283. RefID: 1039. Dhurandhar EJ, Keith SW. The aetiology of obesity beyond eating more and exercising less. Baillieres Best Pract Res Clin Gastroenterol 2014;28(4):533-44.
284. RefID: 1879. Dickerson DL, Leeman RF, Mazure CM, O'Malley SS. The inclusion of women and minorities in smoking cessation clinical trials: a systematic review. Am J Addict 2009;18(1):21-8.
285. RefID: 3390. Diclemente CC. Failure to change or failure to sustain: Pregnancy smoking and postpartum abstinence. Addiction 2016;111(6):992-3.
286. RefID: 3226. DiFranza J, Ursprung WWS, Lauzon B, Bancej C, Wellman RJ, Ziedonis D, et al. A systematic review of the Diagnostic and Statistical Manual diagnostic criteria for nicotine dependence. Addict Behav 2010;35(5):373-82.
287. RefID: 3451. DiFranza JR. Which interventions against the sale of tobacco to minors can be expected to reduce smoking? Tobacco Control: An International Journal 2012;21(4):436-42.
288. RefID: 1567. DiGiacomo M, Davidson PM, Abbott PA, Davison J, Moore L, Thompson SC. Smoking cessation in indigenous populations of Australia, New Zealand, Canada, and the United States: elements of effective interventions. Int J Environ Res Public Health 2011;8(2):388-410.
289. RefID: 1505. Dino GA, Pignataro R, Breland A, Murray PJ, Horn K. Adolescent smoking cessation: promising strategies and evidence-based recommendations. Adolesc Med State Art Rev 2011;22(3):614-xiv
290. RefID: 944. Djachenko A, St John W, Mitchell C. Smoking cessation in male prisoners: a literature review. Int J Prison Health 2015;11(1):39-48.
291. RefID: 3878. Dobbie F, Hiscock R, Leonardi-Bee J, Murray S, Shahab L, Aveyard P, et al. Evaluating Long-term Outcomes of NHS Stop Smoking Services (ELONS): a prospective cohort study (Structured abstract). Health Technology Assessment Database 2016;(2016 Issue 4):
292. RefID: 816. Dobbie F, Hiscock R, Leonardi-Bee J, Murray S, Shahab L, Aveyard P, et al. Evaluating Long-term Outcomes of NHS Stop Smoking Services (ELONS): a prospective cohort study. Health Technol Assess 2015;19(95):1-156.
293. RefID: 3336. Dobbins M, DeCorby K, Manske S, Goldblatt E. Effective practices for school-based tobacco use prevention. Prev Med 2008;46(4):289-97.
294. RefID: 1069. Donny EC, Hatsukami DK, Benowitz NL, Sved AF, Tidey JW, Cassidy RN. Reduced nicotine product standards for combustible tobacco: building an empirical basis for effective regulation. Prev Med 2014;68:17-22.
295. RefID: 1912. Doolan DM, Froelicher ES. Smoking cessation interventions and older adults. Prog Cardiovasc Nurs 2008;23(3):119-27.
296. RefID: 1716. Doonan RJ, Hausvater A, Scallan C, Mikhailidis DP, Pilote L, Daskalopoulou SS. The effect of smoking on arterial stiffness. Hypertens Res 2010;33(5):398-410.
297. RefID: 2413. dos Santos RG, Osorio FL, Crippa JAS, Riba J, Zuardi AW, Hallak JEC. Antidepressive, anxiolytic, and antiaddictive effects of ayahuasca, psilocybin and lysergic acid diethylamide (LSD): a systematic review of clinical trials published in the last 25 years. Ther Adv Psychopharmacol 2016;6(3):193-213. [PMID: http://www.uk.sagepub.com/journals/Journal201949#tabview=aimsAndScope]
298. RefID: 630. Dotson JAW, Pineda R, Cylkowski H, Amiri S. Development and Evaluation of an iPad Application to Promote Knowledge of Tobacco Use and Cessation by Pregnant Women. Nurs Womens Health 2017;21(3):174-85.
299. RefID: 1550. Drozda JJ, Messer JV, Spertus J, Abramowitz B, Alexander K, Beam CT, et al. ACCF/AHA/AMA-PCPI 2011 performance measures for adults with coronary artery disease and hypertension: a report of the American College of Cardiology Foundation/American Heart Association Task Force on Performance Measures and the American Medical Association-Physician Consortium for Performance Improvement. J Am Coll Cardiol 2011;58(3):316-36.
300. RefID: 1380. Drozda JPJ, Holmes DPJ. Performance measures in million hearts: 2 partners' perspective. Circ Cardiovasc Qual Outcomes 2012;5(4):587-8.
301. RefID: 2175. Duailibi MS, Cordeiro Q, Brietzke E, Ribeiro M, LaRowe S, Berk M, et al. N-acetylcysteine in the treatment of craving in substance use disorders: Systematic review and meta-analysis. Am J Addict 2017;26(7):660-6.
302. RefID: 1376. Duaso M, Duncan D. Health impact of smoking and smoking cessation strategies: current evidence. Br J Community Nurs 2012;17(8):356-63.
303. RefID: 1005. Ducasse D, Fond G. [Acceptance and commitment therapy]. Encephale 2015;41(1):1-9.
304. RefID: 2987. Duerksen A, Dubey V, Iglar K. Annual adult health checkup: Update on the preventive care checklist form-¬. Can Fam Phys 2012;58(1):43-e20. [PMID: http://www.cfp.ca/content/58/1/43.full.pdf+html]
305. RefID: 211. Duffy SA, Cummins SE, Fellows JL, Harrington KF, Kirby C, Rogers E, et al. Fidelity monitoring across the seven studies in the Consortium of Hospitals Advancing Research on Tobacco (CHART). Tob induc dis 2015;13(1):29
306. RefID: 3502. Dupont P. Smoking and varenicline. Alcoologie et Addictologie 2008;30(1):29-35.
307. RefID: 1451. Durkin S, Brennan E, Wakefield M. Mass media campaigns to promote smoking cessation among adults: an integrative review. Tob Control 2012;21(2):127-38.
308. RefID: 3520. Ebbert JO, Hays JT. The missing link in tobacco control. Canadian Medical Association Journal 2008;179(2):123-4.
309. RefID: 1486. Ebbert JO, Fagerstrom K. Pharmacological interventions for the treatment of smokeless tobacco use. CNS drugs 2012;26(1):1-10.
310. RefID: 580. Echevarria C, Sinha IP. Heterogeneity in the measurement and reporting of outcomes in studies of electronic cigarette use in adolescents: a systematic analysis of observational studies. Tob Control 2017;26(3):247-53.
311. RefID: 2073. Edmiston EK, Donald CA, Sattler AR, Peebles JK, Ehrenfeld JM, Eckstrand KL. Opportunities and Gaps in Primary Care Preventative Health Services for Transgender Patients: A Systematic Review. Transgender Health 2016;1(1):216-30. [PMID: http://www.liebertpub.com/overview/transgender-health/634/]
312. RefID: 1765. Edrich T, Sadovnikoff N. Anesthesia for patients with severe chronic obstructive pulmonary disease. Curr Opin Anaesthesiol 2010;23(1):18-24.
313. RefID: 3469. Efroymson D, Pham HA, Jones L, FitzGerald S, Thu LT, Hien LTT. Tobacco and poverty: Evidence from Vietnam. Tobacco Control: An International Journal 2011;20(4):296-301.
314. RefID: 3332. Eisenberg MJ, Filion KB, Yavin D. Pharmacotherapies for smoking cessation: A meta-analysis of randomized controlled trials (Canadian Medical Association Journal (2008) 179 (135-144)). CMAJ 2008;179(8):802. [PMID: http://www.cmaj.ca/cgi/reprint/179/8/802]
315. RefID: 1965. Eisenberg MJ, Filion KB, Yavin D, Belisle P, Mottillo S, Joseph L, et al. Pharmacotherapies for smoking cessation: a meta-analysis of randomized controlled trials. CMAJ 2008;179(2):135-44.
316. RefID: 625. Eisenberg MJ, Blum LM, Filion KB, Rinfret S, Pilote L, Paradis G, et al. The efficacy of smoking cessation therapies in cardiac patients: a meta-analysis of randomized controlled trials. Can J Cardiol 2010;26(2):73-9.
317. RefID: 1753. Eisner MD. Secondhand smoke at work. Curr Opin Allergy Clin Immunol 2010;10(2):121-6.
318. RefID: 753. Ellegaard PK, Poulsen HE. Tobacco smoking and oxidative stress to DNA: a meta-analysis of studies using chromatographic and immunological methods. Scand J Clin Lab Invest 2016;76(2):151-8.
319. RefID: 3869. Ellery B, Hiller JE. Quit onQ SMS for smoking cessation support for individuals (Structured abstract). Health Technology Assessment Database 2016;(2016 Issue 4):
320. RefID: 2147. Elshatarat RA, Yacoub MI, Khraim FM, Saleh ZT, Afaneh TR. Self-efficacy in treating tobacco use: A review article. Proc Singapore Healthcare 2016;25(4):243-8. [PMID: http://psh.sagepub.com/content/by/year]
321. RefID: 1201. Englisch S, Morgen K, Meyer-Lindenberg A, Zink M. Risks and benefits of bupropion treatment in schizophrenia: a systematic review of the current literature. Clin Neuropharmacol 2013;36(6):203-15.
322. RefID: 1910. Erguder T, Cakir B, Aslan D, Warren CW, Jones NR, Asma S. Evaluation of the use of Global Youth Tobacco Survey (GYTS) data for developing evidence-based tobacco control policies in Turkey. BMC public health 2008;8 Suppl 1:S4
323. RefID: 3236. Ernst E, Lee MS, Choi T-Y. Acupuncture for addictions: A systematic review of systematic reviews. Focus Altern Complement Ther 2010;15(2):97-100.
324. RefID: 1795. Etter JF. Comparing computer-tailored, internet-based smoking cessation counseling reports with generic, untailored reports: a randomized trial. J Health Commun 2009;14(7):646-57.
325. RefID: 1721. Everett-Murphy K, Steyn K, Mathews C, Petersen Z, Odendaal H, Gwebushe N, et al. The effectiveness of adapted, best practice guidelines for smoking cessation counseling with disadvantaged, pregnant smokers attending public sector antenatal clinics in Cape Town, South Africa. Acta Obstet Gynecol Scand 2010;89(4):478-89.
326. RefID: 3440. Evins AE. Reassessing the safety of varencline. The American Journal of Psychiatry 2013;170(12):1385-7.
327. RefID: 3415. Evins AE, Hong LE, Kelly DL. T. Kishi and N. Iwata: Varenicline for smoking cessation in people with schizophrenia: Systematic review meta-analysis. Eur Arch Psychiatry Clin Neurosci 2015;265(3):269-70.
328. RefID: 945. Evins AE, Cather C, Laffer A. Treatment of tobacco use disorders in smokers with serious mental illness: toward clinical best practices. Harv Rev Psychiatry 2015;23(2):90-8.
329. RefID: 3521. Fagan P. Examining the evidence base of mass media campaigns for socially disadvantaged populations: What do we know, what do we need to learn, and what should we do now? A commentary on Niederdeppe's article. Social Science & Medicine 2008;67(9):1356-8.
330. RefID: 3204. Fagerstrom K, Aubin H-J. Management of smoking cessation in patients with psychiatric disorders. Curr Med Res Opin 2009;25(2):511-8. [PMID: http://www.informapharmascience.com/doi/pdf/10.1185/03007990802707568]
331. RefID: 3343. Fagerstrom KO, Jimenez-Ruiz CA. Pharmacological treatments for tobacco dependence. European Respiratory Review 2008;17(110):192-8. [PMID: http://err.ersjournals.com/cgi/reprint/17/110/192]
332. RefID: 1804. Fagerstrom K. Nicotine replacement: an update. Pneumologia 2009;58(3):176
333. RefID: 1684. Fagerstrom K, Nakamura M, Cho HJ, Tsai ST, Wang C, Davies S, et al. Varenicline treatment for smoking cessation in Asian populations: a pooled analysis of placebo-controlled trials conducted in six Asian countries. Curr Med Res Opin 2010;26(9):2165-73.
334. RefID: 393. Fan H, Song F, Gu H, Wang J, Jia G, Lu M, et al. An assessment of factors associated with quality of randomized controlled trials for smoking cessation. Oncotarget 2016;7(33):53762-71.
335. RefID: 2335. Farham B. 30 days in medicine. S Afr Med J 2017;107(1):15. [PMID: http://www.samj.org.za/index.php/samj/article/download/11790/7940]
336. RefID: 488. Farraye FA, Melmed GY, Lichtenstein GR, Kane SV. ACG Clinical Guideline: Preventive Care in Inflammatory Bowel Disease. Am J Gastroenterol 2017;112(2):241-58.
337. RefID: 2515. Farsalinos KE, Voudris V, Poulas K. Are Metals Emitted from Electronic Cigarettes a Reason for Health Concern? A Risk-Assessment Analysis of Currently Available Literature. Int J Environ Res Public Health 2015;12(5):5215-32. [PMID: http://www.mdpi.com/1660-4601/12/5/5215/pdf]
338. RefID: 257. Farsalinos KE, Polosa R. Safety evaluation and risk assessment of electronic cigarettes as tobacco cigarette substitutes: a systematic review. Ther adv drug saf 2014;5(2):67-86.
339. RefID: 2684. Favaloro EJ. Hot topics v. Semin Thromb Hemost 2014;40(1):5-10.
340. RefID: 3. Fearon IM, Eldridge AC, Gale N, McEwan M, Stiles MF, Round EK. Nicotine pharmacokinetics of electronic cigarettes: A review of the literature. Regul Toxicol Pharmacol 2018;100:25-34.
341. RefID: 567. Feirman SP, Glasser AM, Rose S, Niaura R, Abrams DB, Teplitskaya L, et al. Computational Models Used to Assess US Tobacco Control Policies. Nicotine Tob Res 2017;19(11):1257-67.
342. RefID: 1792. Fernander A, Bush H, Goldsmith-Mason S, White P, Obi B. End-of-treatment smoking cessation among African American female participants in the Breathe Free for Women smoking cessation program: results of a pilot study. J Natl Med Assoc 2009;101(10):1034-40.
343. RefID: 1347. Fihn SD, Gardin JM, Abrams J, Berra K, Blankenship JC, Dallas AP, et al. 2012 ACCF/AHA/ACP/AATS/PCNA/SCAI/STS Guideline for the diagnosis and management of patients with stable ischemic heart disease: a report of the American College of Cardiology Foundation/American Heart Association Task Force on Practice Guidelines, and the American College of Physicians, American Association for Thoracic Surgery, Preventive Cardiovascular Nurses Association, Society for Cardiovascular Angiography and Interventions, and Society of Thoracic Surgeons. J Am Coll Cardiol 2012;60(24):e44-e164
344. RefID: 1537. Filion KB, Abenhaim HA, Mottillo S, Joseph L, Gervais A, O'Loughlin J, et al. The effect of smoking cessation counselling in pregnant women: a meta-analysis of randomised controlled trials. BJOG 2011;118(12):1422-8.
345. RefID: 1523. Fiore MC, Baker TB. Clinical practice. Treating smokers in the health care setting. N Engl J Med 2011;365(13):1222-31.
346. RefID: 706. Fiore MC, Adsit R. Will Hospitals Finally "Do the Right Thing"? Providing Evidence-Based Tobacco Dependence Treatments to Hospitalized Patients Who Smoke. Jt Comm J Qual Patient Saf 2016;42(5):207-8.
347. RefID: 1196. Fishwick D, Carroll C, McGregor M, Drury M, Webster J, Bradshaw L, et al. Smoking cessation in the workplace. Occup Med (Oxf) 2013;63(8):526-36.
348. RefID: 1463. Fitzgerald EM. Evidence-based tobacco cessation strategies with pregnant Latina women. Nurs Clin North Am 2012;47(1):45-54.
349. RefID: 1886. Fjeldsoe BS, Marshall AL, Miller YD. Behavior change interventions delivered by mobile telephone short-message service. Am J Prev Med 2009;36(2):165-73.
350. RefID: 3272. Flay BR. The promise of long-term effectiveness of school-based smoking prevention programs: A critical review of reviews. Tob induc dis 2009;5(1):7
351. RefID: 1903. Flenady V, Macphail J, New K, Devenish-Meares P, Smith J. Implementation of a clinical practice guideline for smoking cessation in a public antenatal care setting. Aust N Z J Obstet Gynaecol 2008;48(6):552-8.
352. RefID: 1699. Flynn BS, Worden JK, Bunn JY, Solomon LJ, Ashikaga T, Connolly SW, et al. Mass media interventions to reduce youth smoking prevalence. Am J Prev Med 2010;39(1):53-62.
353. RefID: 2770. Fogleman CD. Prophylactic antibiotics for the prevention of COPD exacerbation. Am Fam Physician 2014;89(11):870. [PMID: http://www.aafp.org/afp/2014/0601/p870.pdf]
354. RefID: 1438. Foley KL, Pockey JR, Helme DW, Song EY, Stewart K, Jones C, et al. Integrating evidence-based tobacco cessation interventions in free medical clinics: opportunities and challenges. HEALTH PROMOT PRACT 2012;13(5):687-95.
355. RefID: 1644. Foulds J, Hooper MW, Pletcher MJ, Okuyemi KS. Do smokers of menthol cigarettes find it harder to quit smoking? Nicotine Tob Res 2010;12 Suppl 2:S102-S109
356. RefID: 654. Fowkes FG, Forster RB, Levin CE, Naidoo NG, Roy A, Shu C, et al. Prioritization of treatments for lower extremity peripheral artery disease in low- and middle-income countries. Int Angiol 2017;36(3):203-15.
357. RefID: 2807. Fox B, Felkey B. An optimistic but cautionary outlook on mobile health. Hosp Pharm 2013;48(5):438-9.
358. RefID: 2162. Franck C, Filion KB, Eisenberg MJ. Smoking Cessation in Patients With Acute Coronary Syndrome. Am J Cardiol 2018;121(9):1105-11. [PMID: http://www.elsevier.com/locate/amjcard]
359. RefID: 458. French DP, Cameron E, Benton JS, Deaton C, Harvie M. Can Communicating Personalised Disease Risk Promote Healthy Behaviour Change? A Systematic Review of Systematic Reviews. Ann Behav Med 2017;51(5):718-29.
360. RefID: 1101. Fu SS, van Ryn M, Burgess DJ, Nelson D, Clothier B, Thomas JL, et al. Proactive tobacco treatment for low income smokers: study protocol of a randomized controlled trial. BMC public health 2014;14:337
361. RefID: 1073. Fucito LM, Bars MP, Forray A, Rojewski AM, Shiffman S, Selby P, et al. Addressing the evidence for FDA nicotine replacement therapy label changes: a policy statement of the Association for the Treatment of Tobacco use and Dependence and the Society for Research on Nicotine and Tobacco. Nicotine Tob Res 2014;16(7):909-14.
362. RefID: 731. Fucito LM, Czabafy S, Hendricks PS, Kotsen C, Richardson D, Toll BA, et al. Pairing smoking-cessation services with lung cancer screening: A clinical guideline from the Association for the Treatment of Tobacco Use and Dependence and the Society for Research on Nicotine and Tobacco. Cancer 2016;122(8):1150-9.
363. RefID: 2867. Fukuoka Y, Beatty AL, Whooley MA. Using mobile technology for cardiac rehabilitation: A review and framework for development and evaluation. J Am Heart Assoc 2013;2(6):e000568. [PMID: http://jaha.ahajournals.org/content/2/6/e000568.full.pdf]
364. RefID: 977. Furukawa TA. Helping people with schizophrenia to quit smoking. Evid Based Ment Health 2015;18(1):14-5.
365. RefID: 1775. Gaffney KF, Baghi H, Sheehan SE. Two decades of nurse-led research on smoking during pregnancy and postpartum: concept development to intervention trials. Annu Rev Nurs Res 2009;27:195-219.
366. RefID: 3472. Galea S. Tobacco abuse: Treatment and management. 2011;251-73.
367. RefID: 572. Gallagher KM, Updegraff JA. Health message framing effects on attitudes, intentions, and behavior: a meta-analytic review. Ann Behav Med 2012;43(1):101-16.
368. RefID: 317. Garber AJ, Abrahamson MJ, Barzilay JI, Blonde L, Bloomgarden ZT, Bush MA, et al. CONSENSUS STATEMENT BY THE AMERICAN ASSOCIATION OF CLINICAL ENDOCRINOLOGISTS AND AMERICAN COLLEGE OF ENDOCRINOLOGY ON THE COMPREHENSIVE TYPE 2 DIABETES MANAGEMENT ALGORITHM - 2018 EXECUTIVE SUMMARY. Endocr Pract 2018;24(1):91-120.
369. RefID: 1850. Garrison GD, Dugan SE. Varenicline: a first-line treatment option for smoking cessation. Clin Ther 2009;31(3):463-91.
370. RefID: 2864. Garvey C. Identification and treatment of patients with early COPD. Curr Respir Med Rev 2013;9(6):407-17. [PMID: http://www.benthamdirect.org/pages/all_b_bypublication.php]
371. RefID: 28. Gaur S, Agnihotri R. Health Effects of Trace Metals in Electronic Cigarette Aerosols-a Systematic Review. Biol Trace Elem Res 2018;
372. RefID: 1072. Genco RJ, Genco FD. Common risk factors in the management of periodontal and associated systemic diseases: the dental setting and interprofessional collaboration. J evid -based dent pract 2014;14 Suppl:4-16.
373. RefID: 2220. Geng EH, Petersen ML. Network meta-analyses: Powerful but not without perils. Lancet HIV 2014;1(3):e95-e96. [PMID: http://www.journals.elsevier.com/the-lancet-hiv/]
374. RefID: 2638. Gershon AS, Campitelli MA, Hawken S, Victor JC, Sproule B, Kurdyak P, et al. The risk of cardiovascular and neuropsychiatric adverse events following varenicline use for tobacco cessation in Ontario, Canada: A self-controlled study. Am J Respir Crit Care Med 2015;191(MeetingAbstracts). [PMID: http://www.atsjournals.org/doi/pdf/10.1164/ajrccm-conference.2015.191.1_MeetingAbstracts.A5388]
375. RefID: 2091. Ghamri RA. Identification of the most effective pharmaceutical products for smoking cessation: A literature review. J Subst Use 2018;23(6):670-4.
376. RefID: 253. Ghorai K, Akter S, Khatun F, Ray P. mHealth for Smoking Cessation Programs: A Systematic Review. J pers med 2014;4(3):412-23.
377. RefID: 2248. Giacomini M, DeJean D, Simeonov D, Smith A. Experiences of living and dying with COPD: A systematic review and synthesis of the qualitative empirical literature. Ont Health Technol Assess Ser 2012;12(13):1-47. [PMID: http://www.hqontario.ca/en/mas/tech/pdfs/2012/rev_COPD_Qualitative_March.pdf]
378. RefID: 1955. Gianakos D, Kaczynski R. Are your COPD patients benefiting from best practices? J FAM PRACT 2008;57(8):532-6.
379. RefID: 3017. Giannuzzi P. Getting patients to target- implementing the guidelines. Curr Vasc Pharmacol 2012;10(6):715-7.
380. RefID: 1174. Gibson I, Flaherty G, Cormican S, Jones J, Kerins C, Walsh AM, et al. Translating guidelines to practice: findings from a multidisciplinary preventive cardiology programme in the west of Ireland. Eur J Prev Cardiolog 2014;21(3):366-76.
381. RefID: 364. Gilbert H, Sutton S, Morris R, Petersen I, Galton S, Wu Q, et al. Effectiveness of personalised risk information and taster sessions to increase the uptake of smoking cessation services (Start2quit): a randomised controlled trial. Lancet 2017;389(10071):823-33.
382. RefID: 438. Gilbert H, Sutton S, Morris R, Petersen I, Wu Q, Parrott S, et al. Start2quit: a randomised clinical controlled trial to evaluate the effectiveness and cost-effectiveness of using personal tailored risk information and taster sessions to increase the uptake of the NHS Stop Smoking Services. Health Technol Assess 2017;21(3):1-206.
383. RefID: 2819. Gilbey A, Ernst E, Tani K. A systematic review of reviews of systematic reviews of acupuncture. Focus Altern Complement Ther 2013;18(1):8-18.
384. RefID: 847. Gilbody S, Peckham E, Man MS, Mitchell N, Li J, Becque T, et al. Bespoke smoking cessation for people with severe mental ill health (SCIMITAR): a pilot randomised controlled trial. Lancet Psychiatry 2015;2(5):395-402.
385. RefID: 694. Giles EL, Becker F, Ternent L, Sniehotta FF, McColl E, Adams J. Acceptability of Financial Incentives for Health Behaviours: A Discrete Choice Experiment. PloS one 2016;11(6):e0157403
386. RefID: 1956. Ginn MB, Cox G, Heath J. Evidence-based approach to an inpatient tobacco cessation protocol. AACN Adv Crit Care 2008;19(3):268-80.
387. RefID: 2882. Giraldi G, de Ruggiero GF, Marsella LT, d'Alessandro EDL. Environmental tobacco smoke: Health policy and focus on Italian legislation. Clin Ter 2013;164(5 SUPPL.):e429-e435. [PMID: http://www.seuroma.com/clinica_terapeutica/files/2013/05/24_e_pub_Girardi_de_Luca.x_fascicolo.pdf]
388. RefID: 218. Girard N. Evidence appraisal of Prestwich A, Moore S, Kotze A, Budworth L, Lawton R, Kellar I. How can smoking cessation be induced before surgery? A systematic review and meta-analysis of behavior change techniques and other intervention characteristics.: Front Psychol. 2017;8:915. doi:10.3389/fpsyg.2017.00915. AORN J 2017;106(4):346-51.
389. RefID: 1180. Glass TW, Maher CG. Physical activity reduces cigarette cravings. BJSM online 2014;48(16):1263-4.
390. RefID: 391. Glasser AM, Collins L, Pearson JL, Abudayyeh H, Niaura RS, Abrams DB, et al. Overview of Electronic Nicotine Delivery Systems: A Systematic Review. Am J Prev Med 2017;52(2):e33-e66
391. RefID: 728. Gogliettino AR, Potenza MN, Yip SW. White matter development and tobacco smoking in young adults: A systematic review with recommendations for future research. Drug Alcohol Depend 2016;162:26-33.
392. RefID: 3462. Goldade K, Burgess D, Olayinka A, Whembolua GL, Okuyemi KS. Applying anthropology to eliminate tobacco-related health disparities. Nicotine & Tobacco Research 2012;14(6):631-8.
393. RefID: 1033. Goldenberg M, Danovitch I, IsHak WW. Quality of life and smoking. Am J Addict 2014;23(6):540-62.
394. RefID: 238. Golechha M. Health Promotion Methods for Smoking Prevention and Cessation: A Comprehensive Review of Effectiveness and the Way Forward. Int J Prev Med 2016;7:7
395. RefID: 201. Gomez-Coronado N, Walker AJ, Berk M, Dodd S. Current and Emerging Pharmacotherapies for Cessation of Tobacco Smoking. Pharmacotherapy 2018;38(2):235-58.
396. RefID: 1510. Goodman NF, Cobin RH, Ginzburg SB, Katz IA, Woode DE, American Association of Clinical Endocrinologists. American Association of Clinical Endocrinologists Medical Guidelines for Clinical Practice for the diagnosis and treatment of menopause: executive summary of recommendations. Endocr Pract 2011;17(6):949-54.
397. RefID: 277. Gordon LG, Hirst NG, Young RP, Brown PM. Within a smoking-cessation program, what impact does genetic information on lung cancer need to have to demonstrate cost-effectiveness? Cost Eff Resour Alloc 2010;8:18
398. RefID: 3412. Gorelick DA. Sex difference in response to varenicline for smoking cessation. The American Journal of Psychiatry 2015;172(4):394-5.
399. RefID: 227. Gottlieb J, Higley C, Sosnowski R, Bjurlin MA. Smoking-related genitourinary cancers: A global call to action in smoking cessation. Rev urol 2016;18(4):194-204.
400. RefID: 346. Gould GS, Bar-Zeev Y, Bovill M, Atkins L, Gruppetta M, Clarke MJ, et al. Designing an implementation intervention with the Behaviour Change Wheel for health provider smoking cessation care for Australian Indigenous pregnant women. Implement Sci 2017 Sep 15;12(1):114. [PMID: 28915815]
401. RefID: 569. Gould GS, Lim LL, Mattes J. Prevention and Treatment of Smoking and Tobacco Use During Pregnancy in Selected Indigenous Communities in High-Income Countries of the United States, Canada, Australia, and New Zealand: An Evidence-Based Review. Chest 2017;152(4):853-66.
402. RefID: 1368. Graham AL, Fang Y, Moreno JL, Streiff SL, Villegas J, Munoz RF, et al. Online advertising to reach and recruit Latino smokers to an internet cessation program: impact and costs. J Med Internet Res 2012;14(4):e116
403. RefID: 325. Graham AL, Burke MV, Jacobs MA, Cha S, Croghan IT, Schroeder DR, et al. An integrated digital/clinical approach to smoking cessation in lung cancer screening: study protocol for a randomized controlled trial. Trials 2017;18(1):568
404. RefID: 1883. Gratziou C. Respiratory, cardiovascular and other physiological consequences of smoking cessation. Curr Med Res Opin 2009;25(2):535-45.
405. RefID: 1904. Gray BH. Milbank quarterly. Milbank Q 2008;86(4):529-32.
406. RefID: 946. Green AC, Hayman LL, Cooley ME. Multiple health behavior change in adults with or at risk for cancer: a systematic review. Am J Health Behav 2015;39(3):380-94.
407. RefID: 2025. Green JP, Lynn SJ, Montgomery GH. Gender-related differences in hypnosis-based treatments for smoking: a follow-up meta-analysis. Am J Clin Hypn 2008;50(3):259-71.
408. RefID: 347. Greenberg BL, Glick M, Tavares M. Addressing obesity in the dental setting: What can be learned from oral health care professionals' efforts to screen for medical conditions. J Public Health Dent 2017;77 Suppl 1:S67-S78
409. RefID: 2179. Greener M. E-cigarettes: making healthcare professionals myth busters. Prescriber 2018;29(4):20-4. [PMID: http://onlinelibrary.wiley.com/journal/10.1002/(ISSN)1931-2253]
410. RefID: 2262. Greenhill R, Dawkins L, Notley C, Finn MD, Turner JJD. Adolescent Awareness and Use of Electronic Cigarettes: A Review of Emerging Trends and Findings. J Adolesc Health 2016;59(6):612-9. [PMID: http://www.elsevier.com/locate/jadohea]
411. RefID: 2966. Gross N. The COPD pipeline XV. COPD J Chronic Obstructive Pulm Dis 2012;9(2):203-4.
412. RefID: 284. Grossman E, Sherman S. Telling smokers their "lung age" promoted successful smoking cessation. ACP J Club 2008;149(1):5
413. RefID: 1661. Guglielmo BJ. A prescription for improved chronic disease management: have community pharmacists function at the top of their training: comment on "Engaging physicians and pharmacists in providing smoking cessation counseling". Arch Intern Med 2010;170(18):1646-7.
414. RefID: 356. Guignard R, Nguyen-Thanh V, Delmer O, Lenormand MC, Blanchoz JM, Arwidson P. [Interventions for smoking cessation among low socioeconomic status smokers: a literature review]. Sante Publique 2018;30(1):45-60.
415. RefID: 678. Gulland A. E-cigarettes help smokers quit, Cochrane review confirms. BMJ 2016;354:i4993
416. RefID: 2735. Haasova M, Warren FC, Ussher M, Janse Van RK, Faulkner G, Cropley M, et al. The acute effects of physical activity on cigarette cravings: Exploration of potential moderators, mediators and physical activity attributes using individual participant data (IPD) meta-analyses. Psychopharmacology (Berl) 2014;231(7):1267-75. [PMID: http://link.springer.de/link/service/journals/00213/index.htm]
417. RefID: 544. Haasova M, Warren FC, Thompson T, Ussher M, Taylor AH. The association between habitual physical activity and cigarette cravings, and influence of smokers' characteristics in disadvantaged smokers not ready to quit. Psychopharmacology (Berl) 2016;233(14):2765-74.
418. RefID: 1165. Haber SL, Boomershine V, Raney E. Safety of varenicline in patients with cardiovascular disease. J Pharm Pract 2014;27(1):65-70.
419. RefID: 1995. Hackbarth D. Research reporting and evidence of effectiveness: why "no difference" matters. Am J Crit Care 2008;17(3):218-20.
420. RefID: 1461. Hackbarth DP. Preventing adolescent tobacco use and assisting young people to quit: population-, community-, and individually focused evidence-based interventions. Nurs Clin North Am 2012;47(1):119-40.
421. RefID: 1651. Hahn EJ. Smokefree legislation: a review of health and economic outcomes research. Am J Prev Med 2010;39(6 Suppl 1):S66-S76
422. RefID: 2801. Hajek P, McRobbie H, Myers K. Review: Cytisine increases smoking abstinence. Ann Intern Med 2013;159(4):JC11. [PMID: http://annals.org/data/Journals/AIM/927426/0000605-201308200-02011.pdf]
423. RefID: 1059. Hajek P, Etter JF, Benowitz N, Eissenberg T, McRobbie H. Electronic cigarettes: review of use, content, safety, effects on smokers and potential for harm and benefit. Addiction 2014;109(11):1801-10.
424. RefID: 937. Hall AK, Cole-Lewis H, Bernhardt JM. Mobile text messaging for health: a systematic review of reviews. Annu Rev Public Health 2015;36:393-415.
425. RefID: 2450. Hall FS. Genetic Risk for Lung Cancer and the Benefits of Quitting Smoking. EBioMedicine 2016;11:19-20. [PMID: http://www.journals.elsevier.com/ebiomedicine/]
426. RefID: 1366. Hall K, Gibbie T, Lubman DI. Motivational interviewing techniques - facilitating behaviour change in the general practice setting. Aust Fam Physician 2012;41(9):660-7.
427. RefID: 2031. Hall WD, Gartner CE, Carter A. The genetics of nicotine addiction liability: ethical and social policy implications. Addiction 2008;103(3):350-9.
428. RefID: 2639. Halpern SD, French B, Small D, Saulsgiver KA, Harhay MO, Audrain-McGovern JE, et al. A randomized trial of four financial incentive programs for smoking cessation. Am J Respir Crit Care Med 2015;191(MeetingAbstracts). [PMID: http://www.atsjournals.org/doi/pdf/10.1164/ajrccm-conference.2015.191.1_MeetingAbstracts.A5385]
429. RefID: 619. Hamlett-Berry K, Davison J, Kivlahan DR, Matthews MH, Hendrickson JE, Almenoff PL. Evidence-based national initiatives to address tobacco use as a public health priority in the Veterans Health Administration. Mil Med 2009;174(1):29-34.
430. RefID: 1560. Hammond D. Health warning messages on tobacco products: a review. Tob Control 2011;20(5):327-37.
431. RefID: 2968. Hamzaoui A, Ottmani S. Practical approach to lung health: Lung health for everyone? European Respiratory Review 2012;21(125):186-95. [PMID: http://err.ersjournals.com/content/21/125/186.full.pdf+html]
432. RefID: 2015. Hanania NA. The impact of inhaled corticosteroid and long-acting beta-agonist combination therapy on outcomes in COPD. Pulm Pharmacol Ther 2008;21(3):540-50.
433. RefID: 1119. Hand DJ, Heil SH, Sigmon SC, Higgins ST. Improving medicaid health incentives programs: lessons from substance abuse treatment research. Prev Med 2014;63:87-9.
434. RefID: 2406. Harris KK, Zopey M, Friedman TC. Metabolic effects of smoking cessation. Nat Rev Endocrionol 2016;12(5):299-308. [PMID: http://www.nature.com/nrendo/index.html]
435. RefID: 3032. Harris MJ, Haine A. The potential contribution of community health workers to improving health outcomes in UK primary care. J R Soc Med Suppl 2012;105(8):330-5. [PMID: http://jrsm.rsmjournals.com/content/105/8/324.2.full.pdf+html]
436. RefID: 1067. Hartmann-Boyce J, Stead LF, Cahill K, Lancaster T. Efficacy of interventions to combat tobacco addiction: Cochrane update of 2013 reviews. Addiction 2014;109(9):1414-25.
437. RefID: 1633. Hauri DD, Lieb CM, Rajkumar S, Kooijman C, Sommer HL, Roosli M. Direct health costs of environmental tobacco smoke exposure and indirect health benefits due to smoking ban introduction. Eur J Public Health 2011;21(3):316-22.
438. RefID: 1015. Haut Conseil de la Sante publique (HCSP). [Haut Conseil de la Sante publique (HCSP). Guidance on the benefits and risks of the electronic cigarette or e-cigarette in the general population (25 April 2014)]. Rev Mal Respir 2014;31(10):1013-20.
439. RefID: 2695. Hays JT. Varenicline may reduce negative effect while aiding smoking cessation. Evid Based Med 2014;19(1):23. [PMID: http://ebm.bmj.com/content/19/1/23.full.pdf+html]
440. RefID: 3483. Hays JT. Varenicline for smoking cessation: Is it a heartbreaker? Canadian Medical Association Journal 2011;183(12):1346-7.
441. RefID: 1207. Head KJ, Noar SM, Iannarino NT, Grant Harrington N. Efficacy of text messaging-based interventions for health promotion: a meta-analysis. Soc Sci Med 2013;97:41-8.
442. RefID: 275. Health QO. Population-based smoking cessation strategies: a summary of a select group of evidence-based reviews. Ont Health Technol Assess Ser 2010;10(1):1-44.
443. RefID: 3879. Health TA. A randomised trial of nicotine patch preloading for smoking cessation (Project record). Health Technology Assessment Database 2016;(2016 Issue 4):
444. RefID: 3857. Health TA. A randomised trial to increase the uptake of smoking cessation services using personal targeted risk information and taster sessions (Project record). Health Technology Assessment Database 2016;(2016 Issue 4):
445. RefID: 3856. Health TA. A pragmatic randomized controlled trial of physical activity as an aid to smoking cessation during pregnancy (Project record). Health Technology Assessment Database 2016;(2016 Issue 4):
446. RefID: 1774. Heath J, Young S, Bennett S, Ginn MB, Cox G. Evidence-based smoking cessation interventions for patients with acute respiratory disorders. Annu Rev Nurs Res 2009;27:273-96.
447. RefID: 1682. Heckman CJ, Egleston BL, Hofmann MT. Efficacy of motivational interviewing for smoking cessation: a systematic review and meta-analysis. Tob Control 2010;19(5):410-6.
448. RefID: 3117. Heffner JL, Strawn JR, Delbello MP, Strakowski SM, Anthenelli RM. The co-occurrence of cigarette smoking and bipolar disorder: Phenomenology and treatment considerations. Bipolar Disord 2011;13(5-6):439-53.
449. RefID: 1321. Heffner JL, Lewis DF, Winhusen TM. Preliminary evidence that adherence to counseling mediates the effects of pretreatment self-efficacy and motivation on outcome of a cessation attempt in smokers with ADHD. Nicotine Tob Res 2013;15(2):393-400.
450. RefID: 403. Hefner K, Valentine G, Sofuoglu M. Electronic cigarettes and mental illness: Reviewing the evidence for help and harm among those with psychiatric and substance use disorders. Am J Addict 2017;26(4):306-15.
451. RefID: 236. Heminger CL, Schindler-Ruwisch JM, Abroms LC. Smoking cessation support for pregnant women: role of mobile technology. Subst abuse rehabil 2016;7:15-26.
452. RefID: 313. Hendrick V, Suri R, Gitlin MJ, Ortiz-Portillo E. Bupropion Use During Pregnancy: A Systematic Review. Prim Care Companion CNS Disord 2017;19(5):
453. RefID: 1158. Heo HH, Braun KL. Culturally tailored interventions of chronic disease targeting Korean Americans: a systematic review. Ethn Health 2014;19(1):64-85.
454. RefID: 1501. Herie M, Connolly H, Voci S, Dragonetti R, Selby P. Changing practitioner behavior and building capacity in tobacco cessation treatment: the TEACH project. Patient Educ Couns 2012;86(1):49-56.
455. RefID: 1934. Herzog TA. Analyzing the transtheoretical model using the framework of Weinstein, Rothman, and Sutton (1998): the example of smoking cessation. Health Psychol 2008;27(5):548-56.
456. RefID: 672. Hess IM, Lachireddy K, Capon A. A systematic review of the health risks from passive exposure to electronic cigarette vapour. Public health res pract 2016;26(2):
457. RefID: 258. Heydari G, Masjedi M, Ahmady AE, Leischow SJ, Lando HA, Shadmehr MB, et al. A comparative study on tobacco cessation methods: a quantitative systematic review. Int J Prev Med 2014;5(6):673-8.
458. RefID: 226. Heydari G, Ahmady AE, Chamyani F, Masjedi M, Fadaizadeh L. Electronic cigarette, effective or harmful for quitting smoking and respiratory health: A quantitative review papers. Lung India 2017;34(1):25-8.
459. RefID: 1371. Higashi H, Ngo AD, Hill PS. Translating knowledge into policy: provision and use of evidence in the Tobacco Harm Prevention Law in Vietnam. J Public Health Policy 2012;33(4):454-61.
460. RefID: 1476. Higgins ST, Washio Y, Heil SH, Solomon LJ, Gaalema DE, Higgins TM, et al. Financial incentives for smoking cessation among pregnant and newly postpartum women. Prev Med 2012;55 Suppl:S33-S40
461. RefID: 58. Higgins ST, Solomon LJ. Some Recent Developments on Financial Incentives for Smoking Cessation Among Pregnant and Newly Postpartum Women. Curr Addict Rep 2016;3(1):9-18.
462. RefID: 1168. Hill S, Amos A, Clifford D, Platt S. Impact of tobacco control interventions on socioeconomic inequalities in smoking: review of the evidence. Tob Control 2014;23(e2):e89-e97
463. RefID: 1170. Himelhoch S, Riddle J, Goldman HH. Barriers to implementing evidence-based smoking cessation practices in nine community mental health sites. Psychiatr Serv 2014;65(1):75-80.
464. RefID: 1805. Hind D, Tappenden P, Peters J, Kenjegalieva K. Varenicline in the management of smoking cessation: a single technology appraisal. Health Technol Assess 2009;13 Suppl 2:9-13.
465. RefID: 3886. Hind D, Tappenden P, Peters J, Kenjegalieva K. Varenicline in the management of smoking cessation: a single technology appraisal (Structured abstract). Health Technology Assessment Database 2016;(2016 Issue 4):
466. RefID: 638. Hines KL, Peebles RSJ. Management of the Asthma-COPD Overlap Syndrome (ACOS): a Review of the Evidence. Curr Allergy Asthma Rep 2017;17(3):15
467. RefID: 863. Hiscock R, Dobbie F, Bauld L. Smoking Cessation and Socioeconomic Status: An Update of Existing Evidence from a National Evaluation of English Stop Smoking Services. Biomed Res Int 2015;2015:274056
468. RefID: 1314. Hitsman B, Papandonatos GD, McChargue DE, DeMott A, Herrera MJ, Spring B, et al. Past major depression and smoking cessation outcome: a systematic review and meta-analysis update. Addiction 2013;108(2):294-306.
469. RefID: 1642. Hoedjes M, Berks D, Vogel I, Franx A, Visser W, Duvekot JJ, et al. Effect of postpartum lifestyle interventions on weight loss, smoking cessation, and prevention of smoking relapse: a systematic review. Obstet Gynecol Surv 2010;65(10):631-52.
470. RefID: 869. Hoffman SJ, Tan C. Overview of systematic reviews on the health-related effects of government tobacco control policies. BMC public health 2015;15:744
471. RefID: 1568. Hoffmann R, Little V. Trans-disciplinary care: a new approach to improving the effectiveness of tobacco use interventions. J Health Care Poor Underserved 2011;22(2):409-14.
472. RefID: 3160. Hoogendoorn M, Rutten-van MM, Hoogenveen R, Al M, Feenstra T. Comparing the cost-effectiveness of a wide range of COPD interventions using a stochastic population model for COPD. Eur Respir J 2011;38(SUPPL. 55). [PMID: http://erj.ersjournals.com/content/38/Suppl_55/p4126]
473. RefID: 1517. Hoogendoorn M, Rutten-van Molken MPMH, Hoogenveen RT, Al MJ, Feenstra TL. Developing and applying a stochastic dynamic population model for chronic obstructive pulmonary disease. Value Health 2011;14(8):1039-47.
474. RefID: 412. Hsia SL, Myers MG, Chen TC. Combination nicotine replacement therapy: strategies for initiation and tapering. Prev Med 2017;97:45-9.
475. RefID: 1131. Hsueh KC, Hsueh SC, Chou MY, Pan LF, Tu MS, McEwen A, et al. Varenicline versus transdermal nicotine patch: a 3-year follow-up in a smoking cessation clinic in Taiwan. Psychopharmacology (Berl) 2014;231(14):2819-23.
476. RefID: 1248. Huang J, Zheng R, Emery S. Assessing the impact of the national smoking ban in indoor public places in china: evidence from quit smoking related online searches. PloS one 2013;8(6):e65577
477. RefID: 1337. Huang Y, Britton J, Hubbard R, Lewis S. Who receives prescriptions for smoking cessation medications? An association rule mining analysis using a large primary care database. Tob Control 2013;22(4):274-9.
478. RefID: 251. Hudson SV, O'Malley DM, Miller SM. Achieving optimal delivery of follow-up care for prostate cancer survivors: improving patient outcomes. Patient relat outcome meas 2015;6:75-90.
479. RefID: 3003. Huffman MD, Bhatnagar D. Novel treatments for cardiovascular disease prevention. Cardiovasc Ther 2012;30(5):257-63.
480. RefID: 2427. Hughes JR. Varenicline as a cause of suicidal outcomes. Nicotine Tob Res 2016;18(1):2-9. [PMID: http://ntr.oxfordjournals.org/]
481. RefID: 1949. Hughes JR, Peters EN, Naud S. Relapse to smoking after 1 year of abstinence: a meta-analysis. Addict Behav 2008;33(12):1516-20.
482. RefID: 1816. Hughes JR. How confident should we be that smoking cessation treatments work? Addiction 2009;104(10):1637-40.
483. RefID: 1704. Hughes JR, Carpenter MJ, Naud S. Do point prevalence and prolonged abstinence measures produce similar results in smoking cessation studies? A systematic review. Nicotine Tob Res 2010;12(7):756-62.
484. RefID: 1587. Hughes JR, Peters EN, Naud S. Effectiveness of over-the-counter nicotine replacement therapy: a qualitative review of nonrandomized trials. Nicotine Tob Res 2011;13(7):512-22.
485. RefID: 1277. Hughes JR. An updated algorithm for choosing among smoking cessation treatments. J Subst Abuse Treat 2013;45(2):215-21.
486. RefID: 3389. Hughes JR. Editor's choice: Varenicline as a cause of suicidal outcomes. Nicotine & Tobacco Research 2016;18(1):2-9.
487. RefID: 1602. Hughes MC, Yette EM, Hannon PA, Harris JR, Tran NM, Reid TR. Promoting tobacco cessation via the workplace: opportunities for improvement. Tob Control 2011;20(4):305-8.
488. RefID: 1891. Hung DY, Shelley DR. Multilevel analysis of the chronic care model and 5A services for treating tobacco use in urban primary care clinics. Health Serv Res 2009;44(1):103-27.
489. RefID: 1499. Hunt JJ, Cupertino AP, Garrett S, Friedmann PD, Richter KP. How is tobacco treatment provided during drug treatment? J Subst Abuse Treat 2012;42(1):4-15.
490. RefID: 806. Hurst D. Nicotine lozenges and behavioural interventions may help smokeless tobacco users to quit. Evid - based dent 2015;16(4):104-5.
491. RefID: 113. Hurt RT, Mundi MS, Ebbert JO. Challenging obesity, diabetes, and addiction: the potential of lorcaserin extended release. Diabetes Metab Syndr Obes 2018;11:469-78.
492. RefID: 14. Hussenoeder FS, Riedel-Heller SG. Primary prevention of dementia: from modifiable risk factors to a public brain health agenda? Soc Psychiatry Psychiatr Epidemiol 2018;
493. RefID: 2436. Ioakeimidis N, Vlachopoulos C, Tousoulis D. Efficacy and safety of electronic cigarettes for smoking cessation: A critical approach. Hell J Cardiol 2016;57(JANUARY-FEBRUARY):1-6. [PMID: http://hellenicjcardiol.com/archive/full_text/2016/1/2016_1_1.pdf]
494. RefID: 12. Ioakeimidis N, Vlachopoulos C, Katsi V, Tousoulis D. Smoking Cessation Strategies in Pregnancy: Current Concepts And Controversies. HJC Hell J Cardiol 2018;
495. RefID: 2029. Iodice S, Gandini S, Maisonneuve P, Lowenfels AB. Tobacco and the risk of pancreatic cancer: a review and meta-analysis. Langenbecks Arch Surg 2008;393(4):535-45.
496. RefID: 3885. IQWiG. Cessation of smoking in essential hypertension - Rapid report (Structured abstract). Health Technology Assessment Database 2016;(2016 Issue 4):
497. RefID: 1465. Isensee B, Hanewinkel R. Meta-analysis on the effects of the smoke-free class competition on smoking prevention in adolescents. Eur Addict Res 2012;18(3):110-5.
498. RefID: 241. Islami F, Stoklosa M, Drope J, Jemal A. Global and Regional Patterns of Tobacco Smoking and Tobacco Control Policies. Eur Urol Focus 2015;1(1):3-16.
499. Ivers R. Anti-tobacco programs for Aboriginal and Torres Strait Islander people. Cat. No. IHW 37. 2011.
500. RefID: 557. Jaber R, Taleb ZB, Bahelah R, Madhivanan P, Maziak W. Perception, intention and attempts to quit smoking among Jordanian adolescents from the Irbid Longitudinal Study. Int J Tuberc Lung Dis 2016;20(12):1689-94.
501. RefID: 1037. Jacobs MA, Cobb CO, Abroms L, Graham AL. Facebook apps for smoking cessation: a review of content and adherence to evidence-based guidelines. J Med Internet Res 2014;16(9):e205
502. RefID: 1466. Jaehne A, Loessl B, Frick K, Berner M, Hulse G, Balmford J. The efficacy of stepped care models involving psychosocial treatment of alcohol use disorders and nicotine dependence: a systematic review of the literature. Curr Drug Abuse Rev 2012;5(1):41-51.
503. RefID: 3009. Jain M, Rosenberg M. Commentary. Ann Intern Med 2012;157(8):JC4-JC2. [PMID: http://annals.org/data/Journals/AIM/25337/0000605-201210160-02002.pdf]
504. RefID: 2821. Jain R, Majumder P, Gupta T. Pharmacological intervention of nicotine dependence. Biomed Res Int 2013;2013:278392
505. RefID: 149. Jancey J, Maycock B, McCausland K, Howat P. E-Cigarettes: Implications for Health Promotion in the Asian Pacific Region. Asia Pac J Public Health 2018;30(4):321-7.
506. RefID: 634. Jankowski P, Kawecka-Jaszcz K, Kopec G, Podolec J, Pajak A, Sarnecka A, et al. Polish Forum for Prevention Guidelines on Smoking: update 2017. Kardiol Pol 2017;75(4):409-11.
507. RefID: 1457. Javitz HS, Lerman C, Swan GE. Comparative dynamics of four smoking withdrawal symptom scales. Addiction 2012;107(8):1501-11.
508. RefID: 1474. Jay SJ, Torabi MR, Spitznagle MH. A decade of sustaining best practices for tobacco control: Indiana's story. Prev Chronic Dis 2012;9:E37
509. RefID: 2059. Jazieh A, Alghamdi M, Alghanem S, Algarni M, Alkattan K, Alrujaib M, et al. Saudi lung cancer prevention and screening guidelines. Ann Thorac Med 2018;13(4):198-204. [PMID: http://www.thoracicmedicine.org]
510. RefID: 1455. JCS Joint Working Group, Japanese Society for Oral Health, Japanese Society of Oral and Maxillofacial Surgeons, Japanese Society of Public Health, Japanese Respiratory Society, Japan Society of Obstetrics and Gynecology, et al. Guidelines for Smoking Cessation (JCS 2010)--digest version. Circ J 2012;76(4):1024-43.
511. RefID: 1428. Jellinger PS, Smith DA, Mehta AE, Ganda O, Handelsman Y, Rodbard HW, et al. American Association of Clinical Endocrinologists' Guidelines for Management of Dyslipidemia and Prevention of Atherosclerosis. Endocr Pract 2012;18 Suppl 1:1-78.
512. RefID: 1430. Jellinger PS, Smith DA, Mehta AE, Ganda O, Handelsman Y, Rodbard HW, et al. American Association of Clinical Endocrinologists' Guidelines for Management of Dyslipidemia and Prevention of Atherosclerosis: executive summary. Endocr Pract 2012;18(2):269-93.
513. RefID: 1670. Jepson RG, Harris FM, Platt S, Tannahill C. The effectiveness of interventions to change six health behaviours: a review of reviews. BMC public health 2010;10:538
514. RefID: 3335. Jimenez-Ruiz CA, Fagerstrom KO, Miranda JAR. Pharmacologic treatments for smoking cessation. Clin Pulm Med 2008;15(2):106-12.
515. RefID: 2909. Jimenez-Ruiz CA, Fagerstrom KO. Smoking cessation treatment for COPD smokers: The role of pharmacological interventions. Monaldi Arch Chest Dis Card Ser 2013;79(1):27-32. [PMID: http://archest.fsm.it/car/index.html]
516. RefID: 2526. Jimenez-Ruiz CA, Andreas S, Lewis KE, Tonnesen P, van Schayck CP, Hajek P, et al. Statement on smoking cessation in COPD and other pulmonary diseases and in smokers with comorbidities who find it difficult to quit. Eur Respir J 2015;46(1):61-79. [PMID: http://erj.ersjournals.com/content/46/1/61.full.pdf+html]
517. RefID: 1260. Jimenez-Ruiz CA, Riesco Miranda JA, Altet Gomez N, Lorza Blasco JJ, Signes-Costa Minana J, Solano Reina S, et al. Treatment of smoking in smokers with chronic obstructive pulmonary disease. Sociedad Espanola de Neumologia y Cirugia Toracica (SEPAR). Arch Bronconeumol 2013;49(8):354-63.
518. RefID: 3010. Jimenez Ruiz CA, Pinedo AR, Guerrero AC, Ulibarri MM, Fernandez MC, Gonzalez GL. Characteristics of COPD smokers and effectiveness and safety of smoking cessation medications. Nicotine Tob Res 2012;14(9):1035-9.
519. RefID: 1105. Jimenez Ruiz CA, Solano Reina S, de Granda Orive JI, Signes-Costa Minaya J, de Higes Martinez E, Riesco Miranda JA, et al. The electronic cigarette. Official statement of the Spanish Society of Pneumology and Thoracic Surgery (SEPAR) on the efficacy, safety and regulation of electronic cigarettes. Arch Bronconeumol 2014;50(8):362-7.
520. RefID: 327. Jimenez Ruiz CA, Buljubasich D, Riesco Miranda JA, Acuna Izcaray A, de Granda Orive JI, Chatkin JM, et al. Using PICO Methodology to Answer Questions About Smoking in COPD Patients. Arch Bronconeumol 2017;53(11):622-8.
521. RefID: 354. Jimenez Ruiz CA, de Granda Orive JI, Solano Reina S, Riesco Miranda JA, de Higes Martinez E, Pascual Lledo JF, et al. Guidelines for the Treatment of Smoking in Hospitalized Patients. Arch Bronconeumol 2017;53(7):387-94.
522. RefID: 1794. Johnson KS, Tankersley MS. Smoking cessation toolbox for allergists. Ann Allergy Asthma Immunol 2009;103(4):271-336.
523. RefID: 1509. Johnston KN, Young M, Grimmer-Somers KA, Antic R, Frith PA. Why are some evidence-based care recommendations in chronic obstructive pulmonary disease better implemented than others? Perspectives of medical practitioners. Int J Chron Obstruct Pulmon Dis 2011;6:659-67.
524. RefID: 2830. Johnston W, Buscemi J, Coons MJ. Multiple health behavior change: A synopsis and comment on "A review of multiple health behavior change interventions for primary prevention". Transl Behav Med 2013;3(1):6-7.
525. RefID: 1124. Jones KR, Lekhak N, Kaewluang N. Using mobile phones and short message service to deliver self-management interventions for chronic conditions: a meta-review. Worldviews Evid Based Nurs 2014;11(2):81-8.
526. RefID: 2869. Juonala M, Magnussen CG, Raitakari OT. Parental smoking produces long-term damage to vascular function in their children. Curr Opin Cardiol 2013;28(5):569-74.
527. RefID: 2412. Kaczorowski J, Campbell NRC, Duhaney T, Mang E, Gelfer M. Reducing deaths by diet: Call to action for a public policy agenda for chronic disease prevention. Can Fam Phys 2016;62(6). [PMID: http://www.cfp.ca/content/62/6/469.full.pdf+html]
528. RefID: 983. Kadowaki J, Vuolo M, Kelly BC. A review of the current geographic distribution of and debate surrounding electronic cigarette clean air regulations in the United States. Health Place 2015 Jan;31:75-82. [PMID: 25463920]
529. RefID: 303. Kale D, Stautz K, Cooper A. Impulsivity related personality traits and cigarette smoking in adults: A meta-analysis using the UPPS-P model of impulsivity and reward sensitivity. Drug Alcohol Depend 2018;185:149-67.
530. RefID: 3431. Kalkhoran S, Glantz SA. Smoke-free policies: Cleaning the air with money to spare. The Lancet 2014;383(9928):1526-8.
531. RefID: 1470. Kamerow D. Does nicotine replacement really help smokers quit? BMJ 2012;344:e450
532. RefID: 2400. Kaplan NM. Poor choices of antihypertensive drugs. J Am Soc Hypertens 2016;10(1):3-4. [PMID: http://www.elsevier.com]
533. RefID: 2374. Karam-Hage M, Oughli HA, Rabius V, Beneventi D, Wippold RC, Blalock JA, et al. Tobacco cessation treatment pathways for patients with cancer: 10 years in the making. JNCCN J Nat Compr Cancer Netw 2016;14(11):1469-77. [PMID: http://www.jnccn.org/content/14/11/1469.full.pdf]
534. RefID: 340. Karmali KN, Lloyd-Jones DM, Berendsen MA, Goff DCJ, Sanghavi DM, Brown NC, et al. Drugs for Primary Prevention of Atherosclerotic Cardiovascular Disease: An Overview of Systematic Reviews. JAMA Cardiol 2016;1(3):341-9.
535. RefID: 443. Kathuria H, Detterbeck FC, Fathi JT, Fennig K, Gould MK, Jolicoeur DG, et al. Stakeholder Research Priorities for Smoking Cessation Interventions within Lung Cancer Screening Programs. An Official American Thoracic Society Research Statement. Am J Respir Crit Care Med 2017;196(9):1202-12.
536. RefID: 1113. Kato T, Umeda A, Miyagawa K, Takeda H, Adachi T, Toyoda S, et al. Varenicline-assisted smoking cessation decreases oxidative stress and restores endothelial function. Hypertens Res 2014;37(7):655-8.
537. RefID: 2894. Katsiki N, Papadopoulou SK, Fachantidou AI, Mikhailidis DP. Smoking and vascular risk: Are all forms of smoking harmful to all types of vascular disease? Public health 2013;127(5):435-41.
538. RefID: 2287. Katsiki N, Purrello F, Tsioufis C, Mikhailidis DP. Cardiovascular disease prevention strategies for type 2 diabetes mellitus. Expert Opin Pharmacother 2017;18(12):1243-60.
539. RefID: 911. Kauczor HU, Bonomo L, Gaga M, Nackaerts K, Peled N, Prokop M, et al. ESR/ERS white paper on lung cancer screening. Eur Radiol 2015;25(9):2519-31.
540. RefID: 3437. Kaufman A, Hunt Y. Impact of tobacco control policies on youth smoking rates. 2013;933-44.
541. RefID: 307. Kaur G, Pinkston R, Mclemore B, Dorsey WC, Batra S. Immunological and toxicological risk assessment of e-cigarettes. EUR RESPIR REV 2018;27(147):
542. RefID: 278. Kaur K, Kaushal S, Chopra SC. Varenicline for smoking cessation: A review of the literature. Curr Ther Res Clin Exp 2009;70(1):35-54.
543. RefID: 368. Kazemzadeh Z, Manzari ZS, Pouresmail Z. Nursing interventions for smoking cessation in hospitalized patients: a systematic review. Int Nurs Rev 2017;64(2):263-75.
544. RefID: 87. Kearns NT, Carl E, Stein AT, Vujanovic AA, Zvolensky MJ, Smits JAJ, et al. Posttraumatic stress disorder and cigarette smoking: A systematic review. Depress Anxiety 2018;35(11):1056-72.
545. RefID: 3039. Kelly MM, Latta RE, Gimmestad K. Acceptance and mindfulness-based tobacco cessation interventions for individuals with mental health disorders. J Dual Diagn 2012;8(2):89-98.
546. RefID: 2510. Kelly MM, Jensen KP, Sofuoglu M. Co-occurring tobacco use and posttraumatic stress disorder: Smoking cessation treatment implications. Am J Addict 2015;24(8):695-704.
547. RefID: 1770. Kengne AP, Nakamura K, Barzi F, Lam TH, Huxley R, Gu D, et al. Smoking, diabetes and cardiovascular diseases in men in the Asia Pacific region. J Diabetes 2009;1(3):173-81.
548. RefID: 2586. Kennedy SM, Davis SP, Thorne SL. Smoke-free policies in U.S. prisons and jails: A review of the literature. Nicotine Tob Res 2015;17(6):629-35. [PMID: http://ntr.oxfordjournals.org/content/17/6/629.full.pdf]
549. RefID: 2945. Ker JA. The influence of common simple investigations on treatment and outcome in hypertensive patients. S Afr Fam Pract 2012;54(1):46-7. [PMID: http://www.journals.co.za/WebZ/images/ejour/mp_safp/mp_safp_v54_n1_a10.pdf?sessionid=01-34469-528247472&format=F]
550. RefID: 1280. Kerr S, Woods C, Knussen C, Watson H, Hunter R. Breaking the habit: a qualitative exploration of barriers and facilitators to smoking cessation in people with enduring mental health problems. BMC public health 2013;13:221
551. RefID: 2214. Keyser-Marcus L, Vassileva J, Stewart K, Johns S. Impulsivity and cue reactivity in smokers with comorbid depression and anxiety: Possible implications for smoking cessation treatment strategies. Am J Drug Alcohol Abuse 2017;43(4):432-41.
552. RefID: 2313. Khan N, Marvel FA, Wang J, Martin SS. Digital Health Technologies to Promote Lifestyle Change and Adherence. Curr Treat Options Cardiovasc Med 2017;19(8):60. [PMID: http://www.springer.com/medicine/cardiology/journal/11936]
553. RefID: 1042. Kim DJ, Choo EK, Ranney ML. Impact of gender on patient preferences for technology-based behavioral interventions. West J Emerg Med 2014;15(5):593-9.
554. RefID: 3114. Kimokoti RW, Brown LS. Dietary management of the metabolic syndrome. Clin Pharmacol Ther 2011;90(1):184-7.
555. RefID: 3286. Kimura K, Sairenchi T, Muto T. Meta-analysis Study for One Year Effects of a Nicotine Patch. J Health Sci 2009;55(2):233-41. [PMID: http://jhs.pharm.or.jp/data/55(2)/55-233.pdf]
556. RefID: 996. Kinahan CE, Mazloom S, Fernandez AP. Impact of smoking on response to systemic treatment in patients with psoriasis: a retrospective case-control study. Br J Dermatol 2015;172(2):428-36.
557. RefID: 3115. King A. Risk factors: Cigarette smoking increases the risk of coronary heart disease in women more than in men. Nat Rev Cardiol 2011;8(11):612
558. RefID: 3231. Kishore SP, Bitton A, Cravioto A, Yach D. Enabling access to new WHO essential medicines: The case for nicotine replacement therapies. Globalization Health 2010;6:22. [PMID: http://www.globalizationandhealth.com/content/6/1/22]
559. RefID: 2954. Kistler CE, Goldstein AO. The risk of adverse cardiovascular events from varenicline balanced against the benefits in mortality from smoking cessation. Nicotine Tob Res 2012;14(12):1391-3.
560. RefID: 338. Kittle J, Lopes RD, Huang M, Marquess ML, Wilson MD, Ascher J, et al. Cardiovascular adverse events in the drug-development program of bupropion for smoking cessation: A systematic retrospective adjudication effort. Clin Cardiol 2017;40(10):899-906.
561. RefID: 2426. Klemperer EM, Hughes JR. Does the magnitude of reduction in cigarettes per day predict smoking cessation? A qualitative review. Nicotine Tob Res 2016;18(1):88-92. [PMID: http://ntr.oxfordjournals.org/]
562. RefID: 6. Klemperer EM, Hughes JR, Naud S. Study characteristics influence the efficacy of substance abuse treatments: A meta-analysis of medications for smoking cessation. Nicotine Tob Res 2018;
563. RefID: 230. Knight-West O, Bullen C. E-cigarettes for the management of nicotine addiction. Subst abuse rehabil 2016;7:111-8.
564. RefID: 1759. Knight C, Howard P, Baker CL, Marton JP. The cost-effectiveness of an extended course (12+12 weeks) of varenicline compared with other available smoking cessation strategies in the United States: an extension and update to the BENESCO model. Value Health 2010;13(2):209-14.
565. RefID: 2755. Knowlden AP, Ickes MJ, Sharma M. Systematic analysis of tobacco treatment interventions implemented in worksite settings. J Subst Use 2014;19(4):283-94. [PMID: http://informahealthcare.com/jsu]
566. RefID: 2752. Kokubo Y. Prevention of hypertension and cardiovascular diseases: A comparison of lifestyle factors in westerners and east asians. Hypertension 2014;63(4):655-60.
567. RefID: 2992. Kong G, Singh N, Krishnan-Sarin S. A review of culturally targeted/tailored tobacco prevention and cessation interventions for minority adolescents. Nicotine Tob Res 2012;14(12):1394-406.
568. RefID: 2692. Kong G, Ells DM, Camenga DR, Krishnan-Sarin S. Text messaging-based smoking cessation intervention: A narrative review. Addict Behav 2014;39(5):907-17. [PMID: http://www.elsevier.com/locate/addictbeh]
569. RefID: 1915. Korenstein D, McGinn T. The impact of an intensive smoking cessation intervention. Mt Sinai J Med 2008;75(6):552-5.
570. RefID: 1409. Kotani K, Hazama A, Hagimoto A, Saika K, Shigeta M, Katanoda K, et al. Adiponectin and smoking status: a systematic review. J Atheroscler Thromb 2012;19(9):787-94.
571. RefID: 23. Kotsen C, Santorelli ML, Bloom EL, Goldstein AO, Ripley-Moffitt C, Steinberg MB, et al. A Narrative Review of Intensive Group Tobacco Treatment: Clinical, Research, and U.S. Policy Recommendations. Nicotine Tob Res 2018;
572. RefID: 955. Kotz D. Implementation of a new 'opt-out' default for tobacco treatment is urgently needed, but requires free access to evidence-based treatments. Addiction 2015;110(3):387-8.
573. RefID: 228. Kowalski M, Udy AA, McRobbie HJ, Dooley MJ. Nicotine replacement therapy for agitation and delirium management in the intensive care unit: a systematic review of the literature. J Intensive Care 2016;4:69
574. RefID: 2047. Kozlowski LT. Origins in the USA in the 1980s of the warning that smokeless tobacco is not a safe alternative to cigarettes: A historical, documents-based assessment with implications for comparative warnings on less harmful tobacco/nicotine products. Harm Reduct J 2018;15(1):21. [PMID: http://www.harmreductionjournal.com/home/]
575. RefID: 1787. Kreshak AA, Clark AK, Clark RF, Ly BT, Cantrell FL. A retrospective poison center review of varenicline-exposed patients. Ann Pharmacother 2009;43(12):1986-91.
576. RefID: 1852. Krishna S, Boren SA, Balas EA. Healthcare via cell phones: a systematic review. Telemed J E Health 2009;15(3):231-40.
577. RefID: 1020. Krishnan V, Dixon-Williams S, Thornton JD. Where there is smoke...there is sleep apnea: exploring the relationship between smoking and sleep apnea. Chest 2014;146(6):1673-80.
578. RefID: 738. Kruger J, O'Halloran A, Rosenthal AC, Babb SD, Fiore MC. Receipt of evidence-based brief cessation interventions by health professionals and use of cessation assisted treatments among current adult cigarette-only smokers: National Adult Tobacco Survey, 2009-2010. BMC public health 2016;16:141
579. RefID: 3020. Kruse GR, Kelley JHK, Linder JA, Park ER, Rigotti NA. Implementation of an electronic health record-based care management system to improve tobacco treatment. J Gen Intern Med 2012;27(12):1690-6. [PMID: http://www.springerlink.com/content/120414/]
580. RefID: 1130. Kuipers E, Yesufu-Udechuku A, Taylor C, Kendall T. Management of psychosis and schizophrenia in adults: summary of updated NICE guidance. BMJ 2014;348:g1173
581. RefID: 2420. Kulak JA, Cornelius ME, Fong GT, Giovino GA. Differences in Quit Attempts and Cigarette Smoking Abstinence Between Whites and African Americans in the United States: Literature Review and Results From the International Tobacco Control US Survey. Nicotine Tob Res 2016;18(Supplement 1):S79-S87. [PMID: http://ntr.oxfordjournals.org/]
582. RefID: 1539. Kumar S, Kodela S, Detweiler JG, Kim KY, Detweiler MB. Bupropion-induced psychosis: folklore or a fact? A systematic review of the literature. Gen Hosp Psychiatry 2011;33(6):612-7.
583. RefID: 2112. Kwan ML, Garren B, Nielsen ME, Tang L. Lifestyle and nutritional modifiable factors in the prevention and treatment of bladder cancer. Urol Oncol Semin Orig Invest 2018. [PMID: http://www.elsevier.com/locate/issn/10781439]
584. RefID: 1621. Ladapo JA, Jaffer FA, Weinstein MC, Froelicher ES. Projected cost-effectiveness of smoking cessation interventions in patients hospitalized with myocardial infarction. Arch Intern Med 2011;171(1):39-45.
585. RefID: 1435. Laing SS, Hannon PA, Talburt A, Kimpe S, Williams B, Harris JR. Increasing evidence-based workplace health promotion best practices in small and low-wage companies, Mason County, Washington, 2009. Prev Chronic Dis 2012;9:E83
586. RefID: 3320. Lancaster T, Stead L, Cahill K. An update on therapeutics for tobacco dependence. Expert Opin Pharmacother 2008;9(1):15-22.
587. RefID: 2525. Lancaster T, Cahill K. Review: Varenicline does not differ from placebo for adverse neuropsychiatric events. Ann Intern Med 2015;163(2):JC6. [PMID: http://annals.org/data/Journals/AIM/934240/ACPJC-2015-163-2-006.pdf]
588. RefID: 994. Lando HA, Michaud ME, Poston WSC, Jahnke SA, Williams L, Haddock CK. Banning cigarette smoking on US Navy submarines: a case study. Tob Control 2015;24(e3):e188-e192
589. RefID: 1733. Laniado-Laborin R. Smoking cessation intervention: an evidence-based approach. Postgrad Med 2010;122(2):74-82.
590. RefID: 1826. Lawhon D, Humfleet GL, Hall SM, Munoz RF, Reus VI. Longitudinal analysis of abstinence-specific social support and smoking cessation. Health Psychol 2009;28(4):465-72.
591. RefID: 2814. Lawn S, Campion J. Achieving smoke-free mental health services: Lessons from the past decade of implementation research. Int J Environ Res Public Health 2013;10(9):4224-44. [PMID: http://www.mdpi.com/1660-4601/10/9/4224/pdf]
592. RefID: 1558. Lawrence D, Mitrou F, Zubrick SR. Global research neglect of population-based approaches to smoking cessation: time for a more rigorous science of population health interventions. Addiction 2011;106(9):1549-54.
593. RefID: 1354. Le Faou AL, Baha M. [Policy and routine practice for smoking cessation in France]. Presse Med 2012;41(12 Pt 1):1279-85.
594. RefID: 532. Le Foll B, Forget B, Aubin HJ, Goldberg SR. Blocking cannabinoid CB1 receptors for the treatment of nicotine dependence: insights from pre-clinical and clinical studies. Addict Biol 2008;13(2):239-52.
595. RefID: 502. Le Foll B, Justinova Z, Tanda G, Goldberg SR. [Future medications for tobacco and cannabis dependence]. Bull Acad Natl Med 2008;192(1):45-7.
596. RefID: 850. Leach PT, Gould TJ. Thyroid hormone signaling: Contribution to neural function, cognition, and relationship to nicotine. Neurosci Biobehav Rev 2015;57:252-63.
597. RefID: 495. Leduc C, Quoix E. Is there a role for e-cigarettes in smoking cessation? Therap adv respir dis 2016;10(2):130-5.
598. RefID: 986. Lee AHY, Stater BJ, Close L, Rahmati R. Are e-cigarettes effective in smoking cessation? Laryngoscope 2015;125(4):785-7.
599. RefID: 856. Lee EB, An W, Levin ME, Twohig MP. An initial meta-analysis of Acceptance and Commitment Therapy for treating substance use disorders. Drug Alcohol Depend 2015;155:1-7.
600. RefID: 1895. Lee JH, Jones PG, Bybee K, O'Keefe JH. A longer course of varenicline therapy improves smoking cessation rates. Prev Cardiol 2008;11(4):210-4.
601. RefID: 886. Lee JY, Park HA, Min YH. Transtheoretical Model-based Nursing Intervention on Lifestyle Change: A Review Focused on Intervention Delivery Methods. Asian nurs res 2015;9(2):158-67.
602. RefID: 1019. Lee JGL, Matthews AK, McCullen CA, Melvin CL. Promotion of tobacco use cessation for lesbian, gay, bisexual, and transgender people: a systematic review. Am J Prev Med 2014;47(6):823-31.
603. RefID: 2661. Lee PN, Fry JS, Forey BA. A review of the evidence on smoking bans and incidence of heart disease. Regul Toxicol Pharmacol 2014;70(1):7-23. [PMID: http://www.elsevier.com/inca/publications/store/6/2/2/9/3/9/index.htt]
604. RefID: 1645. Lee PN, Fry JS. Systematic review of the evidence relating FEV1 decline to giving up smoking. BMC Med 2010;8:84
605. RefID: 1624. Lee PN. Summary of the epidemiological evidence relating snus to health. Regul Toxicol Pharmacol 2011;59(2):197-214.
606. RefID: 1191. Lee PN. Epidemiological evidence relating snus to health--an updated review based on recent publications. Harm Reduct J 2013;10:36
607. RefID: 1285. Lee PN. The effect on health of switching from cigarettes to snus - a review. Regul Toxicol Pharmacol 2013;66(1):1-5.
608. RefID: 1162. Lee PN. Health risks related to dual use of cigarettes and snus - a systematic review. Regul Toxicol Pharmacol 2014;69(1):125-34.
609. RefID: 2027. Leonard EE, Wynd CA. Meta-analysis as a tool for evidence-based practice: an example using the Rice meta-analysis of smoking cessation interventions. Appl Nurs Res 2008;21(1):40-4.
610. RefID: 1259. Leone FT, Evers-Casey S, Toll BA, Vachani A. Treatment of tobacco use in lung cancer: Diagnosis and management of lung cancer, 3rd ed: American College of Chest Physicians evidence-based clinical practice guidelines. Chest 2013;143(5 Suppl):e61S-e77S
611. RefID: 872. Leone FT, Carlsen KH, Folan P, Latzka K, Munzer A, Neptune E, et al. An Official American Thoracic Society Research Statement: Current Understanding and Future Research Needs in Tobacco Control and Treatment. Am J Respir Crit Care Med 2015;192(3):e22-e41
612. RefID: 813. Leung LWS, Davies GA. Smoking Cessation Strategies in Pregnancy. J Obstet Gynaecol Can 2015;37(9):791-7.
613. RefID: 908. Leung T, Bergen A, Munafo MR, De Ruyck K, Selby P, De Luca V. Effect of the rs1051730-rs16969968 variant and smoking cessation treatment: a meta-analysis. Pharmacogenomics 2015;16(7):713-20.
614. RefID: 3398. Leventhal AM, Zvolensky MJ. Anxiety, depression, and cigarette smoking: A transdiagnostic vulnerability framework to understanding emotion-smoking comorbidity. Psychol Bull 2015;141(1):176-212.
615. RefID: 345. Levine GN, Lange RA, Bairey-Merz CN, Davidson RJ, Jamerson K, Mehta PK, et al. Meditation and Cardiovascular Risk Reduction: A Scientific Statement From the American Heart Association. J Am Heart Assoc 2017;6(10):
616. RefID: 1467. Levinson AH, Hood N, Mahajan R, Russ R. Smoking cessation treatment preferences, intentions, and behaviors among a large sample of Colorado gay, lesbian, bisexual, and transgendered smokers. Nicotine Tob Res 2012;14(8):910-8.
617. RefID: 845. Li H, Li S, Wang Q, Pan L, Jiang F, Yang X, et al. Association of 5-HTTLPR polymorphism with smoking behaviors: A meta-analysis. Physiol Behav 2015;152(Pt A):32-40.
618. RefID: 1543. Li WJ, Zhang HY, Miao CL, Tang RB, DU X, Shi JH, et al. Cigarette smoking inhibits the anti-platelet activity of aspirin in patients with coronary heart disease. Chin Med J 2011;124(10):1569-72.
619. RefID: 3374. Li W. Mindfulness-Oriented Recovery Enhancement for video game addiction in U.S. emerging adults. Dissertation Abstracts International Section A: Humanities and Social Sciences 2017;77(11-A(E)):No-Specified
620. RefID: 2012. Lin K, Watkins B, Johnson T, Rodriguez JA, Barton MB, U.S.Preventive Services Task Force. Screening for chronic obstructive pulmonary disease using spirometry: summary of the evidence for the U.S. Preventive Services Task Force. Ann Intern Med 2008;148(7):535-43.
621. RefID: 2685. Lin S-K. Pharmacological means of reducing human drug dependence: A selective and narrative review of the clinical literature. Br J Clin Pharmacol 2014;77(2):242-52.
622. RefID: 1761. Lindahl AK. Comments regarding "a systematic review of implementation of established recommended secondary prevention measures in PAOD". Eur J Vasc Endovasc Surg 2010;39(1):87-8.
623. RefID: 1062. Lindson-Hawley N, Coleman T, Docherty G, Hajek P, Lewis S, Lycett D, et al. Nicotine patch preloading for smoking cessation (the preloading trial): study protocol for a randomized controlled trial. Trials 2014;15:296
624. RefID: 326. Lindson N, Richards-Doran D, Heath L, Hartmann-Boyce J, CTAG taps team. Setting research priorities in tobacco control: a stakeholder engagement project. Addiction 2017;112(12):2257-71.
625. RefID: 2974. Lingford-Hughes AR, Welch S, Peters L, Nutt DJ. BAP updated guidelines: Evidence-based guidelines for the pharmacological management of substance abuse, harmful use, addiction and comorbidity: Recommendations from BAP. J Psychopharmacol 2012;26(7):899-952.
626. RefID: 757. Little MA, Ebbert JO. The safety of treatments for tobacco use disorder. Expert Opin Drug Saf 2016;15(3):333-41.
627. RefID: 2080. Livingston G, Sommerlad A, Orgeta V, Costafreda SG, Huntley J, Ames D, et al. Dementia prevention, intervention, and care. The Lancet 2017;390(10113):2673-734. [PMID: http://www.journals.elsevier.com/the-lancet/]
628. RefID: 2718. Lo L, Wong T-K, Chao DVK. Review on pharmacological treatments for smoking cessation. Hong Kong Pract 2014;36(2):52-60.
629. RefID: 1484. Loh WY, Piper ME, Schlam TR, Fiore MC, Smith SS, Jorenby DE, et al. Should all smokers use combination smoking cessation pharmacotherapy? Using novel analytic methods to detect differential treatment effects over 8 weeks of pharmacotherapy. Nicotine Tob Res 2012;14(2):131-41.
630. RefID: 1458. Lorencatto F, West R, Michie S. Specifying evidence-based behavior change techniques to aid smoking cessation in pregnancy. Nicotine Tob Res 2012;14(9):1019-26.
631. RefID: 1269. Lorencatto F, West R, Christopherson C, Michie S. Assessing fidelity of delivery of smoking cessation behavioural support in practice. Implement Sci 2013;8:40
632. RefID: 209. Losby JL, House MJ, Osuji T, O'Dell SA, Mirambeau AM, Elmi J, et al. Initiatives to Enhance Primary Care Delivery: Two Examples from the Field. Health serv res manag epidemiol 2015;2:
633. RefID: 1727. Lowe EJ, Ackman ML. Impact of tobacco smoking cessation on stable clozapine or olanzapine treatment. Ann Pharmacother 2010;44(4):727-32.
634. RefID: 1830. Lozier EB, Gonzalez YM. Smoking cessation practices in the dental profession. J Contemp Dent Pract 2009;10(4):97-103.
635. RefID: 1323. Luca NR, Suggs LS. Theory and model use in social marketing health interventions. J Health Commun 2013;18(1):20-40.
636. RefID: 231. Lucchiari C, Masiero M, Botturi A, Pravettoni G. Helping patients to reduce tobacco consumption in oncology: a narrative review. SpringerPlus 2016;5(1):1136
637. RefID: 3447. Lunde S. Youth tobacco use: Who has a say? The Lancet 2013;381(9864):357
638. RefID: 2018. Lundgren JD, Battegay M, Behrens G, De Wit S, Guaraldi G, Katlama C, et al. European AIDS Clinical Society (EACS) guidelines on the prevention and management of metabolic diseases in HIV. HIV Med 2008;9(2):72-81.
639. RefID: 3468. Lv J, Su M, Hong Z, Zhang T, Huang X, Wang B, et al. Implementation of the WHO Framework Convention on Tobacco Control in mainland China. Tobacco Control: An International Journal 2011;20(4):309-14.
640. RefID: 3386. Ly C. The Texas Tobacco Prevention Program (T2P 2): An update of the Minnesota Smoking Prevention Program (MSPP). Dissertation Abstracts International: Section B: The Sciences and Engineering 2016;77(1-B(E)):No-Specified
641. RefID: 1246. Lycett D, Aveyard P, Farmer A, Lewis A, Munafo M. Slimming World in Stop Smoking Services (SWISSS): study protocol for a randomized controlled trial. Trials 2013;14:182
642. RefID: 3329. Lynn SJ, Boycheva E, Barnes S. To assess or not assess hypnotic suggestibility? That is the question. Am J Clin Hypn 2008;51(2):161-5.
643. RefID: 831. Mackey TK, Miner A, Cuomo RE. Exploring the e-cigarette e-commerce marketplace: Identifying Internet e-cigarette marketing characteristics and regulatory gaps. Drug Alcohol Depend 2015;156:97-103.
644. RefID: 173. Macready AL, Fallaize R, Butler LT, Ellis JA, Kuznesof S, Frewer LJ, et al. Application of Behavior Change Techniques in a Personalized Nutrition Electronic Health Intervention Study: Protocol for the Web-Based Food4Me Randomized Controlled Trial. JMIR Res Protoc 2018;7(4):e87
645. RefID: 3432. Madan J, Chen YF, Aveyard P, Wang D, Yahaya I, Munafo M, et al. Synthesis of evidence on heterogeneous interventions with multiple outcomes recorded over multiple follow-up times reported inconsistently: A smoking cessation case-study. Journal of the Royal Statistical Society: Series A (Statistics in Society) 2014;177(1):295-314.
646. RefID: 2690. Magee LA, Pels A, Helewa M, Rey E, Von DP. Diagnosis, evaluation, and management of the hypertensive disorders of pregnancy. Pregnancy Hypertens 2014;4(2):105-45. [PMID: http://www.elsevier.com/wps/find/journaldescription.cws_home/725004/description#description]
647. RefID: 2289. Maggi P, Di BA, Rusconi S, Cicalini S, D'Abbraccio M, d'Ettorre G, et al. Cardiovascular risk and dyslipidemia among persons living with HIV: A review. BMC Infect Dis 2017;17(1):551. [PMID: http://www.biomedcentral.com/bmcinfectdis/]
648. RefID: 3441. Mahabee-Gittens EM, Stone L, Gordon JS. Pediatric emergency department is a promising venue for adult tobacco cessation interventions. Nicotine & Tobacco Research 2013;15(10):1792-3.
649. RefID: 1987. Main C, Thomas S, Ogilvie D, Stirk L, Petticrew M, Whitehead M, et al. Population tobacco control interventions and their effects on social inequalities in smoking: placing an equity lens on existing systematic reviews. BMC public health 2008;8:178
650. RefID: 656. Maisonneuve E. [Lifestyle recommendations for prevention of spontaneous preterm birth in asymptomatic pregnant women]. J Gynecol Obstet Biol Reprod (Paris) 2016;45(10):1231-46.
651. RefID: 2747. Maity N, Chand P, Murthy P. Role of nicotine receptor partial agonists in tobacco cessation. Indian J Psychiatry 2014;56(1):17-23.
652. RefID: 3068. Majer IM, Rozenbaum MH, Verheggen B. Cost-effectiveness of smoking cessation interventions in smokers with cardiovascular disease in the netherlands. Value in Health 2012;15(7):A374-A375
653. RefID: 1183. Mak JCS, Fransen M, Jennings M, March L, Mittal R, Harris IA, et al. Evidence-based review for patients undergoing elective hip and knee replacement. ANZ J Surg 2014;84(1-2):17-24.
654. RefID: 2064. Makani R, Pradhan B, Shah U, Parikh T. Role of repetitive transcranial magnetic stimulation (rTMS) in treatment of addiction and related disorders: A systematic review. Curr Drug Abuse Rev 2017;10(1):31-43. [PMID: http://www.eurekaselect.com/671/journal/current-drug-abuse-reviews]
655. Malaysian Academy of Pharmacy. CLINICAL PRACTICE GUIDELINES ON TREATMENT OF TOBACCO USE DISORDER. Tobacco Control Unit & FCTC Secretariat; 2016.
656. RefID: 978. Malinowski B, Minkler M, Stock L. Labor unions: a public health institution. Am J Public Health 2015;105(2):261-71.
657. RefID: 1450. Malone RE, Grundy Q, Bero LA. Tobacco industry denormalisation as a tobacco control intervention: a review. Tob Control 2012;21(2):162-70.
658. RefID: 1307. Maltais F, Dennis N, Chan CKN. Rationale for earlier treatment in COPD: a systematic review of published literature in mild-to-moderate COPD. COPD 2013;10(1):79-103.
659. RefID: 2859. Mancia G, Fagard R, Narkiewicz K, Redon J, Zanchetti A, Bohm M, et al. 2013 ESH/ESC guidelines for the management of arterial hypertension. Blood Press 2013;22(4):193-278.
660. RefID: 1249. Mancia G, Fagard R, Narkiewicz K, Redon J, Zanchetti A, Bohm M, et al. 2013 ESH/ESC guidelines for the management of arterial hypertension: the Task Force for the Management of Arterial Hypertension of the European Society of Hypertension (ESH) and of the European Society of Cardiology (ESC). Eur Heart J 2013;34(28):2159-219.
661. RefID: 187. Marcolino MS, Oliveira JAQ, D'Agostino M, Ribeiro AL, Alkmim MBM, Novillo-Ortiz D. The Impact of mHealth Interventions: Systematic Review of Systematic Reviews. JMIR Mhealth Uhealth 2018;6(1):e23
662. RefID: 2373. Maret-Ouda J, El-Serag HB, Lagergren J. Opportunities for preventing esophageal adenocarcinoma. Cancer Prev Res 2016;9(11):828-34. [PMID: http://cancerpreventionresearch.aacrjournals.org/content/9/11/828.full-text.pdf]
663. RefID: 1142. Marley JV, Atkinson D, Kitaura T, Nelson C, Gray D, Metcalf S, et al. The Be Our Ally Beat Smoking (BOABS) study, a randomised controlled trial of an intensive smoking cessation intervention in a remote aboriginal Australian health care setting. BMC public health 2014;14:32
664. RefID: 1607. Marques-Vidal P, Melich-Cerveira J, Paccaud F, Waeber G, Vollenweider P, Cornuz J. High expectation in non-evidence-based smoking cessation interventions among smokers--the CoLaus study. Prev Med 2011;52(3-4):258-61.
665. RefID: 2502. Marshall N, Taylor S, Shekelle P, Hempel S, Miake-Lye I, Beroes J. An evidence map of the effect of mindfulness on health outcomes. J Altern Complement Med 2016;22(6):A113
666. RefID: 2117. Martinez C, Fu M, Galan I, Perez-Rios M, Martinez-Sanchez JM, Lopez MJ, et al. Conflicts of interest in research on electronic cigarettes. Tob induc dis 2018;16(June):28. [PMID: http://www.tobaccoinduceddiseases.org/pdf-90668-26171?filename=Conflicts%20of%20interest%20in.pdf]
667. RefID: 1002. Mason M, Ola B, Zaharakis N, Zhang J. Text messaging interventions for adolescent and young adult substance use: a meta-analysis. Prev Sci 2015;16(2):181-8.
668. RefID: 380. Mathew AR, Hogarth L, Leventhal AM, Cook JW, Hitsman B. Cigarette smoking and depression comorbidity: systematic review and proposed theoretical model. Addiction 2017;112(3):401-12.
669. RefID: 309. Mavrot C, Stucki I, Sager F, Etter JF. Efficacy of an Internet-based, individually tailored smoking cessation program: A randomized-controlled trial. J Telemed Telecare 2017;23(5):521-8.
670. RefID: 2051. McArdle MJ, Abbott AL, Krajcer Z. Carotid artery stenosis in women. Tex Heart Inst J 2018;45(4):243-5. [PMID: http://www.thij.org/doi/pdf/10.14503/THIJ-18-6711]
671. RefID: 1993. McCambridge J, Jenkins RJ. Do brief interventions which target alcohol consumption also reduce cigarette smoking? Systematic review and meta-analysis. Drug Alcohol Depend 2008;96(3):263-70.
672. RefID: 957. McClernon FJ, Addicott MA, Sweitzer MM. Smoking abstinence and neurocognition: implications for cessation and relapse. Curr Top Behav Neurosci 2015;23:193-227.
673. RefID: 200. McCowan LM, Figueras F, Anderson NH. Evidence-based national guidelines for the management of suspected fetal growth restriction: comparison, consensus, and controversy. Am J Obstet Gynecol 2018;218(2S):S855-S868
674. RefID: 3367. McCubbin A, Fallin-Bennett A, Barnett J, Ashford K. Perceptions and use of electronic cigarettes in pregnancy. Health Educ Res 2017;32(1):22-32.
675. RefID: 1412. McDermott MS, Thomson H, West R, Kenyon JAM, McEwen A. Translating evidence-based guidelines into practice: a survey of practices of commissioners and managers of the English stop smoking services. BMC Health Serv Res 2012;12:121
676. RefID: 3421 McGrath DS, Stewart SH. Tobacco dependence. In: Agboola BBBBBBBBBBCCDDEFFFGG-VGHHHHHHHH, editors. The Wiley handbook of cognitive behavioral therapy., Vols. 1-3, Wiley-Blackwell; 2014. p. 1299-314.
677. RefID: 485. McIvor A, Kayser J, Assaad JM, Brosky G, Demarest P, Desmarais P, et al. Best practices for smoking cessation interventions in primary care. Can Respir J 2009;16(4):129-34.
678. RefID: 3406. McKee M, Capewell S. Electronic cigarettes: We need evidence, not opinions. The Lancet 2015;386(10000):1239
679. RefID: 898. McKeganey N, Russell C. Tobacco plain packaging: Evidence based policy or public health advocacy? Int J Drug Policy 2015;26(6):560-8.
680. RefID: 3393. McKeganey N, Russell C. "Tobacco plain packaging: Evidence based policy or public health advocacy": Corrigendum. International Journal of Drug Policy 2016;27:186
681. RefID: 1703. McLeish AC, Zvolensky MJ. Asthma and cigarette smoking: a review of the empirical literature. J Asthma 2010;47(4):345-61.
682. RefID: 362. McMenamin UC, McCain S, Kunzmann AT. Do smoking and alcohol behaviours influence GI cancer survival? Baillieres Best Pract Res Clin Gastroenterol 2017;31(5):569-77.
683. RefID: 1692. McNeil JJ, Piccenna L, Ioannides-Demos LL. Smoking cessation-recent advances. Cardiovasc Drugs Ther 2010;24(4):359-67.
684. RefID: 1352. McNeill A, Amos A, McEwen A, Ferguson J, Croghan E. Developing the evidence base for addressing inequalities and smoking in the United Kingdom. Addiction 2012;107 Suppl 2:1-7.
685. RefID: 1038. McNeill A, Etter JF, Farsalinos K, Hajek P, le Houezec J, McRobbie H. A critique of a World Health Organization-commissioned report and associated paper on electronic cigarettes. Addiction 2014;109(12):2128-34.
686. RefID: 1972. McRobbie H, Bullen C, Glover M, Whittaker R, Wallace-Bell M, Fraser T, et al. New Zealand smoking cessation guidelines. N Z Med J 2008;121(1276):57-70.
687. RefID: 1310. McRobbie H, Raw M, Chan S. Research priorities for Article 14--demand reduction measures concerning tobacco dependence and cessation. Nicotine Tob Res 2013;15(4):805-16.
688. RefID: 913. Meernik C, Goldstein AO. A critical review of smoking, cessation, relapse and emerging research in pregnancy and post-partum. Br Med Bull 2015;114(1):135-46.
689. RefID: 2394. Mehta GR, Sarfraz S, Khan T, Ahmed K, Villareal M, Martinez D, et al. Chronic obstructive pulmonary disease: A guide for the primary care physician. Dis Mon 2016;62(6):164-87. [PMID: http://www.elsevier.com/inca/publications/store/6/2/3/2/9/2/index.htt]
690. RefID: 1189. Mehta M. The Cochrane tobacco addiction group. J Evid Based Med 2013;6(4):213-5.
691. RefID: 2391. Mena A, Meijide H, Marcos PJ. Lung cancer in HIV-infected patients. AIDS Rev 2016;18(3):138-44. [PMID: http://www.aidsreviews.com/files/2016_18_3_138-144.pdf]
692. RefID: 2570. Mendelsohn CP, Kirby DP, Castle DJ. Smoking and mental illness. An update for psychiatrists. Australasian Psychiatry 2015;23(1):37-43.
693. RefID: 1554. Mendelsohn C. Women who smoke - A review of the evidence. Aust Fam Physician 2011;40(6):403-7.
694. RefID: 1426. Mendelsohn C. Smoking and depression--a review. Aust Fam Physician 2012;41(5):304-7.
695. RefID: 1247. Mendelsohn C. Optimising nicotine replacement therapy in clinical practice. Aust Fam Physician 2013;42(5):305-9.
696. RefID: 1125. Mendelsohn C, Gould GS, Oncken C. Management of smoking in pregnant women. Aust Fam Physician 2014;43(1):46-51.
697. RefID: 867. Mendelsohn C. Managing nicotine dependence in NSW hospitals under the Smoke-free Health Care Policy. Public health res pract 2015;25(3):e2531533
698. RefID: 958. Menezes AM, Landis SH, Han MK, Muellerova H, Aisanov Z, van der Molen T, et al. Continuing to confront COPD International Surveys: comparison of patient and physician perceptions about COPD risk and management. Int J Chron Obstruct Pulmon Dis 2015;10:159-72.
699. RefID: 1235. Menossi HS, Goudriaan AE, de Azevedo-Marques Perico C, Nicastri S, de Andrade AG, D'Elia G, et al. Neural bases of pharmacological treatment of nicotine dependence - insights from functional brain imaging: a systematic review. CNS drugs 2013;27(11):921-41.
700. RefID: 2710. Meo SA, Al Asiri SA. Effects of electronic cigarette smoking on human health. Eur Rev Med Pharmacol Sci 2014;18(21):3315-9. [PMID: http://www.europeanreview.org/]
701. RefID: 879. Mercken L, Saul JE, Lemaire RH, Valente TW, Leischow SJ. Coevolution of Information Sharing and Implementation of Evidence-Based Practices Among North American Tobacco Cessation Quitlines. Am J Public Health 2015;105(9):1814-22.
702. RefID: 2294. Metse AP, Wiggers JH, Wye PM, Wolfenden L, Prochaska JJ, Stockings EA, et al. Smoking and mental illness: A bibliometric analysis of research output over time. Nicotine Tob Res 2017;19(1):24-31. [PMID: http://ntr.oxfordjournals.org/]
703. RefID: 3258. Meyers DG, Neuberger JS, He J. Cardiovascular Effect of Bans on Smoking in Public Places. A Systematic Review and Meta-Analysis. J Am Coll Cardiol 2009;54(14):1249-55.
704. RefID: 268. Meysamie A, Ghaletaki R, Zhand N, Abbasi M. Cigarette smoking in iran. Iran J Public Health 2012;41(2):1-14.
705. RefID: 1851. Michie S, Jochelson K, Markham WA, Bridle C. Low-income groups and behaviour change interventions: a review of intervention content, effectiveness and theoretical frameworks. J Epidemiol Community Health 2009;63(8):610-22.
706. RefID: 141. Michie S, Brown J, Geraghty AWA, Miller S, Yardley L, Gardner B, et al. Development of StopAdvisor: A theory-based interactive internet-based smoking cessation intervention. Transl Behav Med 2012;2(3):263-75.
707. RefID: 812. Michie S, Wood CE, Johnston M, Abraham C, Francis JJ, Hardeman W. Behaviour change techniques: the development and evaluation of a taxonomic method for reporting and describing behaviour change interventions (a suite of five studies involving consensus methods, randomised controlled trials and analysis of qualitative data). Health Technol Assess 2015;19(99):1-188.
708. RefID: 2231. Miedema MD, Virani SS. Aspirin reduces cardiovascular events in primary prevention of cardiovascular disease but at a near equivalent risk of increased bleeding. Evid Based Med 2016;21(6):225. [PMID: http://ebm.bmj.com/]
709. RefID: 1573. Mills SC, von Roon AC, Tekkis PP, Orchard TR. Crohn's disease. Clin Evid (Online) 2011;2011:
710. RefID: 118. Minguez-Alarcon L, Chavarro JE, Gaskins AJ. Caffeine, alcohol, smoking, and reproductive outcomes among couples undergoing assisted reproductive technology treatments. Fertil Steril 2018;110(4):587-92.
711. RefID: 2876. Minichino A, Bersani FS, Calo WK, Spagnoli F, Francesconi M, Vicinanza R, et al. Smoking behaviour and mental health disorders-mutual influences and implications for therapy. Int J Environ Res Public Health 2013;10(10):4790-811. [PMID: http://www.mdpi.com/1660-4601/10/10/4790/pdf]
712. RefID: 85. Minnix JA, Karam-Hage M, Blalock JA, Cinciripini PM. The importance of incorporating smoking cessation into lung cancer screening. Transl lung cancer res 2018;7(3):272-80.
713. RefID: 2861. Miranda JAR, Ruiz CAJ, Rebollo JCS. Smoking cessation: Update. Clin Pulm Med 2013;20(3):129-36.
714. RefID: 1986. Mitchell EN, Hawkshaw BN, Naylor CJ, Soewido D, Sanders JM. Enabling the NSW health workforce to provide evidence-based smoking-cessation advice through competency-based training delivered via video conferencing. N S W Public Health Bull 2008;19(3-4):56-9.
715. RefID: 2298. Miyazaki T, Kitagawa Y, Kuwano H, Kusano M, Oyama T, Muto M, et al. Decreased risk of esophageal cancer owing to cigarette and alcohol cessation in smokers and drinkers: a systematic review and meta-analysis. Esophagus 2017;14(4):290-302. [PMID: http://www.springeronline.com/sgw/cda/frontpage/0,11855,5-0-70-19483014-0,00.html?referer=www.springeronline.com/east/journal/10388]
716. RefID: 248. Miyazaki Y, Hayashi K, Imazeki S. Smoking cessation in pregnancy: psychosocial interventions and patient-focused perspectives. Int J Women Health 2015;7:415-27.
717. RefID: 3488. Mohamed MHN. Group-based behavioural intervention for smoking cessation: Is it all in their heads? J Smok Cessat 2011;6(1):1-2.
718. RefID: 2211. Mohan P, Lando HA. Oral Tobaccco and Mortality in India. Integr Med Insights 2016;7. [PMID: http://www.la-press.com/journal.php?journal_id=21&issue_id=458&tab=volume&order_by=volume&max_results=50]
719. RefID: 1597. Molina-Linde JM. Effectiveness of smoking cessation programs for seriously mentally ill. Actas Esp Psiquiatr 2011;39(2):106-14.
720. RefID: 474. Moll FL, Powell JT, Fraedrich G, Verzini F, Haulon S, Waltham M, et al. Management of abdominal aortic aneurysms clinical practice guidelines of the European society for vascular surgery. Eur J Vasc Endovasc Surg 2011;41 Suppl 1:S1-S58
721. RefID: 920. Mons U, Muezzinler A, Gellert C, Schottker B, Abnet CC, Bobak M, et al. Impact of smoking and smoking cessation on cardiovascular events and mortality among older adults: meta-analysis of individual participant data from prospective cohort studies of the CHANCES consortium. BMJ 2015;350:h1551
722. RefID: 1437. Moore RA, Aubin HJ. Do placebo response rates from cessation trials inform on strength of addictions? Int J Environ Res Public Health 2012;9(1):192-211.
723. RefID: 804. Morales-Rosado JA, Cousin MA, Ebbert JO, Klee EW. A Critical Review of Repurposing Apomorphine for Smoking Cessation. Assay Drug Dev Technol 2015;13(10):612-22.
724. RefID: 722. Moreira-Santos TM, Godoy I, de Godoy I. Psychological distress related to smoking cessation in patients with acute myocardial infarction. J Bras Pneumol 2016;42(1):61-7.
725. RefID: 1754. Morgan G, Backinger C, Lerman C, Vocci F. Translational medication development for nicotine addiction. Am J Health Behav 2010;34(3):267-74.
726. RefID: 1299. Morley CP, Pratte MA. State-level tobacco control and adult smoking rate in the United States: an ecological analysis of structural factors. J Public Health Manag Pract 2013;19(6):E20-E27
727. RefID: 849. Morozova M, Rabin RA, George TP. Co-morbid tobacco use disorder and depression: A re-evaluation of smoking cessation therapy in depressed smokers. Am J Addict 2015;24(8):687-94.
728. RefID: 1579. Morris CD, May MG, Devine K, Smith S, DeHay T, Mahalik J. Multiple perspectives on tobacco use among youth with mental health disorders and addictions. Am J Health Promot 2011;25(5 Suppl):S31-S37
729. RefID: 1175. Moscou-Jackson G, Commodore-Mensah Y, Farley J, DiGiacomo M. Smoking-cessation interventions in people living with HIV infection: a systematic review. J Assoc Nurses AIDS Care 2014;25(1):32-45.
730. RefID: 3126. Moy I, Crome P, Crome I, Fisher M. Systematic and narrative review of treatment for older people with substance problems. Eur Geriatr Med 2011;2(4):212-36.
731. RefID: 878. Muezzinler A, Mons U, Gellert C, Schottker B, Jansen E, Kee F, et al. Smoking and All-cause Mortality in Older Adults: Results From the CHANCES Consortium. Am J Prev Med 2015;49(5):e53-e63
732. RefID: 1548. Munafo MR, Johnstone EC, Walther D, Uhl GR, Murphy MFG, Aveyard P. CHRNA3 rs1051730 genotype and short-term smoking cessation. Nicotine Tob Res 2011;13(10):982-8.
733. RefID: 1836. Mundey K. An appraisal of smoking cessation aids. Curr Opin Pulm Med 2009;15(2):105-12.
734. RefID: 2895. Muniraj T, Jamidar PA, Aslanian HR. Pancreatic cancer: A comprehensive review and update. Dis Mon 2013;59(11):368-402.
735. RefID: 3324. Murray E. Internet-delivered treatments for long-term conditions: Strategies, efficiency and cost-effectiveness. Expert Review of Pharmacoeconomics and Outcomes Research 2008;8(3):261-72. [PMID: http://www.expert-reviews.com/doi/pdf/10.1586/14737167.8.3.261]
736. RefID: 2030. Murray RL, Coleman T, Antoniak M, Fergus A, Britton J, Lewis SA. The potential to improve ascertainment and intervention to reduce smoking in primary care: a cross sectional survey. BMC Health Serv Res 2008;8:6
737. RefID: 1811. Musich S, Chapman LS, Ozminkowski R. Best practices for smoking cessation: implications for employer-based programs. Am J Health Promot 2009;24(1):TAHP-10
738. RefID: 1823. Nakamura K, Huxley R, Ansary-Moghaddam A, Woodward M. The hazards and benefits associated with smoking and smoking cessation in Asia: a meta-analysis of prospective studies. Tob Control 2009;18(5):345-53.
739. RefID: 1815. Narayanan S, Ebbert JO, Sood A. Gender differences in self-reported use, perceived efficacy, and interest in future use of nicotine-dependence treatments: A cross-sectional survey in adults at a tertiary care center for nicotine dependence. Gend Med 2009;6(2):362-8.
740. RefID: 1603. Nasser M. Evidence summary: is smoking cessation an effective and cost-effective service to be introduced in NHS dentistry? Br Dent J 2011;210(4):169-77.
741. RefID: 5201. National Institute for Health and Care Excellence. Stop smoking interventions and services. [A] Evidence reviews for stop smoking services. [Internet]. Available at: https://www.nice.org.uk/guidance/ng92. Last Accessed: 3-3-2020.
742. RefID: 5203. National Institute for Health and Care Excellence. Smoking cessation interventions and services [C] Evidence reviews for advice on e-cigarettes. [Internet]. Available at: https://www.nice.org.uk/guidance/ng92/evidence/c-advice-on-ecigarettes-on-general-sale-pdf-4788920848. Last Accessed: 3-3-2020.
743. RefID: 5202. National Institute for Health and Care Excellence. Smoking cessation interventions and services. [B] Evidence reviews for interventions to aid. [Internet]. Available at: https://www.nice.org.uk/guidance/ng92/documents/evidence-review. Last Accessed: 3-3-2020.
744. RefID: 3866. National Institute for Health and Clinical Excellence. School-based interventions to prevent the uptake of smoking among children (Structured abstract). Health Technology Assessment Database 2016;(2016 Issue 4):
745. RefID: 1545. Nau JY. [Gums and aorta; tobacco and the brain]. Rev Med Suisse 2011;7(296):1172-3.
746. RefID: 272. Nayan S, Gupta MK, Sommer DD. Evaluating smoking cessation interventions and cessation rates in cancer patients: a systematic review and meta-analysis. ISRN oncol 2011;2011:849023
747. RefID: 1975. Nelson JP, Pederson LL. Military tobacco use: a synthesis of the literature on prevalence, factors related to use, and cessation interventions. Nicotine Tob Res 2008;10(5):775-90.
748. RefID: 786. Ness-Jensen E, Hveem K, El-Serag H, Lagergren J. Lifestyle Intervention in Gastroesophageal Reflux Disease. Clin Gastroenterol Hepatol 2016;14(2):175-3.
749. RefID: 1266. Newhouse RP, Dennison Himmelfarb C, Morlock L, Frick KD, Pronovost P, Liang Y. A phased cluster-randomized trial of rural hospitals testing a quality collaborative to improve heart failure care: organizational context matters. Med Care 2013;51(5):396-403.
750. RefID: 426. Newton JN, Dockrell M, Marczylo T. Making sense of the latest evidence on electronic cigarettes. Lancet 2018;391(10121):639-42.
751. RefID: 3012. Niaura R, Chander G, Hutton H, Stanton C. Interventions to address chronic disease and HIV: Strategies to promote smoking cessation among HIV-infected individuals. Curr HIV/AIDS Rep 2012;9(4):375-84.
752. RefID: 2006. Niaura R. Nonpharmacologic therapy for smoking cessation: characteristics and efficacy of current approaches. Am J Med 2008;121(4 Suppl 1):S11-S19
753. RefID: 1953. Niederdeppe J, Kuang X, Crock B, Skelton A. Media campaigns to promote smoking cessation among socioeconomically disadvantaged populations: what do we know, what do we need to learn, and what should we do now? Soc Sci Med 2008;67(9):1343-55.
754. RefID: 1952. Niederdeppe J, Kuang X, Crock B, Skelton A. Critical steps in building the evidence base regarding media campaign effects on disadvantaged populations: a response to Fagan. Soc Sci Med 2008;67(9):1359-60.
755. RefID: 3902. NIHR HSRI. Voke 0.45mg inhaler for smoking cessation - first line (Structured abstract). Health Technology Assessment Database 2016;(2016 Issue 4):
756. RefID: 3016. Nikfardjam M. Omega-3 fatty acids and cardioprotection-IIb or not IIb? The Lancet 2012;380(9858):1976-7.
757. RefID: 286. Niznik JD, He H, Kane-Gill SL. Impact of clinical pharmacist services delivered via telemedicine in the outpatient or ambulatory care setting: A systematic review. Res Social Adm Pharm 2018;14(8):707-17.
758. RefID: 3882. No author listed. Smoking Cessation Interventions for Pregnant Women and Mothers of Infants: A Review of the Clinical Effectiveness, Safety, and Guidelines. [Internet]. Available at: CADTH Rapid Response. Last Accessed: 3-3-2020. [PMID:https://cadth.ca/smoking-cessation-interventions-pregnant-women-and-mothers-infants-review-clinical-effectiveness]
759. RefID: 3417. Noar SM, Hall MG, Brewer NT. Pictorial cigarette pack warnings have important effects. Am J Public Health 2015;105(3):e1
760. RefID: 556. Noar SM, Francis DB, Bridges C, Sontag JM, Ribisl KM, Brewer NT. The impact of strengthening cigarette pack warnings: Systematic review of longitudinal observational studies. Soc Sci Med 2016;164:118-29.
761. RefID: 787. Noar SM, Hall MG, Francis DB, Ribisl KM, Pepper JK, Brewer NT. Pictorial cigarette pack warnings: a meta-analysis of experimental studies. Tob Control 2016;25(3):341-54.
762. RefID: 144. Noar SM, Francis DB, Bridges C, Sontag JM, Brewer NT, Ribisl KM. Effects of Strengthening Cigarette Pack Warnings on Attention and Message Processing: A Systematic Review. JMCQ 2017;94(2):416-42.
763. RefID: 1225. Nocente R, Vitali M, Balducci G, Enea D, Kranzler HR, Ceccanti M. Varenicline and neuronal nicotinic acetylcholine receptors: a new approach to the treatment of co-occurring alcohol and nicotine addiction? Am J Addict 2013;22(5):453-9.
764. RefID: 626. Nolan MB, Warner DO. Perioperative tobacco use treatments: putting them into practice. BMJ 2017;358:j3340
765. RefID: 2283. Nordin N, Hassali MAA, Sarriff A. Actual or potential extended services performed by Malaysian community pharmacists, perceptions and barriers towards it's performance: A systematic review. Int J Pharmcy Pharm Sci 2017;9(10):13-20. [PMID: https://innovareacademics.in/journals/index.php/ijpps/article/download/20694/12520]
766. RefID: 385. Norris AR, Estes Miller J. Motivational interviewing or counseling, medical therapies or no intervention to improve tobacco cessation in adults and adolescents. J Okla State Med Assoc 2017;110(3):142-3.
767. RefID: 1078. Nowak D, Jorres RA, Ruther T. E-cigarettes--prevention, pulmonary health, and addiction. Dtsch Arztebl int 2014;111(20):349-55.
768. RefID: 3265. Nturibi EM, Kolawole AA, McCurdy SA. Smoking prevalence and tobacco control measures in Kenya, Uganda, the Gambia and Liberia: A review. Int J Tuberc Lung Dis 2009;13(2):165-70.
769. O'Leary R, MacDonald M, Stockwell T et al. Clearing the Air: A systematic review on the harms and benefits of e-cigarettes and vapour devices. CRD42015025267. Victoria, BC: Centre for Addictions Research of BC. CIHR; 2019.
770. RefID: 2014. Ogilvie D, Fayter D, Petticrew M, Sowden A, Thomas S, Whitehead M, et al. The harvest plot: a method for synthesising evidence about the differential effects of interventions. BMC Med Res Methodol 2008;8:8
771. RefID: 677. Ogundeji YK, Bland JM, Sheldon TA. The effectiveness of payment for performance in health care: A meta-analysis and exploration of variation in outcomes. Health Policy 2016;120(10):1141-50.
772. RefID: 482. Okoli CTC, Greaves L, Bottorff JL, Marcellus LM. Health care providers' engagement in smoking cessation with pregnant smokers. J Obstet Gynecol Neonatal Nurs 2010;39(1):64-77.
773. RefID: 2751. Olin JW, Gornik HL, Bacharach JM, Biller J, Fine LJ, Gray BH, et al. Fibromuscular dysplasia: State of the science and critical unanswered questions: A scientific statement from the american heart association. Circulation 2014;129(9):1048-78.
774. RefID: 3021. Olive MF, Cleva RM, Kalivas PW, Malcolm RJ. Glutamatergic medications for the treatment of drug and behavioral addictions. Pharmacol Biochem Behav 2012;100(4):801-10. [PMID: http://www.elsevier.com/locate/pharmbiochembeh]
775. RefID: 239. Omana-Cepeda C, Jane-Salas E, Estrugo-Devesa A, Chimenos-Kustner E, Lopez-Lopez J. Effectiveness of dentist's intervention in smoking cessation: A review. J clin exp dent 2016;8(1):e78-e83
776. RefID: 1726. Oncken CA, Dietz PM, Tong VT, Belizan JM, Tolosa JE, Berghella V, et al. Prenatal tobacco prevention and cessation interventions for women in low- and middle-income countries. Acta Obstet Gynecol Scand 2010;89(4):442-53. [PMID: 20235895]
777. RefID: 2194. Onor IO, Stirling DL, Williams SR, Bediako D, Borghol A, Harris MB, et al. Clinical effects of cigarette smoking: Epidemiologic impact and review of pharmacotherapy options. Int J Environ Res Public Health 2017;14(10):1147. [PMID: http://www.mdpi.com/1660-4601/14/10/1147/pdf]
778. RefID: 370. Ordean A, Wong S, Graves L. No. 349-Substance Use in Pregnancy. J Obstet Gynaecol Can 2017;39(10):922-37.
779. RefID: 711. Ordonez-Mena JM, Schottker B, Mons U, Jenab M, Freisling H, Bueno-de-Mesquita B, et al. Quantification of the smoking-associated cancer risk with rate advancement periods: meta-analysis of individual participant data from cohorts of the CHANCES consortium. BMC Med 2016;14:62
780. RefID: 1047. Orr KK, Asal NJ. Efficacy of electronic cigarettes for smoking cessation. Ann Pharmacother 2014;48(11):1502-6.
781. RefID: 1098. Orr MS. Electronic cigarettes in the USA: a summary of available toxicology data and suggestions for the future. Tob Control 2014;23 Suppl 2:ii18-ii22
782. RefID: 3507. Oshima A, Ito Y, Nomura H. Sensitivity analysis of the efficacy of varenicline in smoking cessation with a special reference to study dropouts. J Smok Cessat 2009;4(2):86-91.
783. RefID: 444. Osthoff M, Jenkins C, Leuppi JD. Chronic obstructive pulmonary disease--a treatable disease. Swiss Med Wkly 2013;143:w13777
784. RefID: 1755. Ovbiagele B. Optimizing vascular risk reduction in the stroke patient with atherothrombotic disease. Med Princ Pract 2010;19(1):1-12.
785. RefID: 3383. Owotomo O. Current trends and impact of smoking cessation interventions for adult smokers in low and middle income countries: A systematic literature review. J Smok Cessat 2016;11(1):37-49.
786. RefID: 2592. Oza R, Garcellano M. Nonpharmacologic management of hypertension: What works? Am Fam Physician 2015;91(11):772-6. [PMID: http://www.aafp.org/afp/2015/0601/p772.pdf]
787. RefID: 2143. Ozemek C, Phillips SA, Popovic D, Laddu-Patel D, Fancher IS, Arena R, et al. Nonpharmacologic management of hypertension: A multidisciplinary approach. Curr Opin Cardiol 2017;32(4):381-8. [PMID: http://journals.lww.com/co-cardiology/pages/default.aspx]
788. RefID: 244. Pacek LR, Crum RM. A Review of the Literature Concerning HIV and Cigarette Smoking: Morbidity and Mortality, Associations with Individual- and Social-Level Characteristics, and Smoking Cessation Efforts. Addict Res Theory 2015;23(1):10-23.
789. RefID: 120. Pacek LR, McClernon FJ, Bosworth HB. Adherence to Pharmacological Smoking Cessation Interventions: A Literature Review and Synthesis of Correlates and Barriers. Nicotine Tob Res 2018;20(10):1163-72.
790. RefID: 2393. Palmer BW, Raskind MA. Posttraumatic Stress Disorder and Aging. Am J Geriatr Psychiatry 2016;24(3):177-80. [PMID: http://www.sciencedirect.com/science/journal/10647481]
791. RefID: 2185. Panagiotakos DB, Notara V, Kouvari M, Pitsavos C. The mediterranean and other dietary patterns in secondary cardiovascular disease prevention: A review. Curr Vasc Pharmacol 2016;14(5):442-51. [PMID: http://www.benthamdirect.org/pages/all_b_bypublication.php]
792. RefID: 1065. Papadakis S, Gharib M, Hambleton J, Reid RD, Assi R, Pipe AL. Delivering evidence-based smoking cessation treatment in primary care practice: experience of Ontario family health teams. Can Fam Physician 2014;60(7):e362-e371
793. RefID: 3035. Papanastasiou E. Interventions for the metabolic syndrome in schizophrenia: A review. Ther Adv Endocrinol Metab 2012;3(5):141-62.
794. RefID: 2102. Paraskevas KI, Veith FJ, Ricco J-B. Best medical treatment alone may not be adequate for all patients with asymptomatic carotid artery stenosis. J Vasc Surg 2018;68(2):572-5. [PMID: http://www.elsevier.com/inca/publications/store/6/2/3/1/5/2/index.htt]
795. RefID: 745. Parikh V, Kutlu MG, Gould TJ. nAChR dysfunction as a common substrate for schizophrenia and comorbid nicotine addiction: Current trends and perspectives. Schizophr Res 2016;171(1-3):1-15.
796. RefID: 1221. Park AL, McDaid D, Weiser P, Von Gottberg C, Becker T, Kilian R, et al. Examining the cost effectiveness of interventions to promote the physical health of people with mental health problems: a systematic review. BMC public health 2013;13:787
797. RefID: 764. Park SJ, Yi B, Lee HS, Oh WY, Na HK, Lee M, et al. To quit or not: Vulnerability of women to smoking tobacco. J ENVIRON SCI HEALTH PART C ENVIRON CARCINOG ECOTOXICOL REV 2016;34(1):33-56.
798. RefID: 3491. Parsons A, Lycett D, Aveyard P. Response to Spring et al.: What is the best method to assess the effect of combined interventions for smoking cessation and post-cessation weight gain? Addiction 2011;106(3):675-6.
799. RefID: 91. Parvar SL, Fitridge R, Dawson J, Nicholls SJ. Medical and lifestyle management of peripheral arterial disease. J Vasc Surg 2018;68(5):1595-606.
800. RefID: 536. Patten SB, Williams JVA, Lavorato DH, Wang JL, Sajobi TT, Bulloch AGM. Major depression and non-specific distress following smoking cessation in the Canadian general population. J Affect Disord 2017;218:182-7.
801. RefID: 43. Patterson F, Grandner MA, Malone SK, Rizzo A, Davey A, Edwards DG. Sleep as a Target for Optimized Response to Smoking Cessation Treatment. Nicotine Tob Res 2017;
802. RefID: 1166. Patwardhan PD, Amin ME, Chewning BA. Intervention research to enhance community pharmacists' cognitive services: a systematic review. Res Social Adm Pharm 2014;10(3):475-93.
803. RefID: 29. Pauly N, Talbert J, Parsley S, Gray B, Hahn EJ. Kentucky's Smoking Cessation Quitline: Annual Estimated Return on Investment to Employers. Am J Health Promot 2018;890117118784875
804. RefID: 1198. Pavy B, Barbet R, Carre F, Champion C, Iliou MC, Jourdain P, et al. Therapeutic education in coronary heart disease: position paper from the Working Group of Exercise Rehabilitation and Sport (GERS) and the Therapeutic Education Commission of the French Society of Cardiology. Arch Cardiovasc Dis 2013;106(12):680-9.
805. RefID: 1875. Paynter J, Edwards R. The impact of tobacco promotion at the point of sale: a systematic review. Nicotine Tob Res 2009;11(1):25-35.
806. RefID: 858. Pbert L, Druker S, Flint AJ, Young MH, DiFranza JR. Perspectives in Implementing a Pragmatic Pediatric Primary Care-Based Intervention Trial. Am J Prev Med 2015;49(3 Suppl 2):S200-S207
807. RefID: 931. Peckham E, Man MS, Mitchell N, Li J, Becque T, Knowles S, et al. Smoking Cessation Intervention for severe Mental Ill Health Trial (SCIMITAR): a pilot randomised control trial of the clinical effectiveness and cost-effectiveness of a bespoke smoking cessation service. Health Technol Assess 2015;19(25):1-vi
808. RefID: 1793. Percival J. Smoking cessation 1: best practice and treatment and support options for patients. Nurs Times 2009;105(38):21-3.
809. RefID: 2293. Perez-Lopez FR, Ceausu I, Depypere H, Kehoe S, Lambrinoudaki I, Mueck A, et al. Interventions to reduce the risk of ovarian and fallopian tube cancer: A European Menopause and Andropause Society Postition Statement. Maturitas 2017;100:86-91. [PMID: http://www.elsevier.com/locate/maturitas]
810. RefID: 773. Pericot-Valverde I, Germeroth LJ, Tiffany ST. The Use of Virtual Reality in the Production of Cue-Specific Craving for Cigarettes: A Meta-Analysis. Nicotine Tob Res 2016;18(5):538-46.
811. RefID: 523. Perk J, De Backer G, Gohlke H, Graham I, Reiner Z, Verschuren M, et al. European Guidelines on cardiovascular disease prevention in clinical practice (version 2012). The Fifth Joint Task Force of the European Society of Cardiology and Other Societies on Cardiovascular Disease Prevention in Clinical Practice (constituted by representatives of nine societies and by invited experts). Eur Heart J 2012;33(13):1635-701.
812. RefID: 2967. Perkins KA. Subjective reactivity to smoking cues as a predictor of quitting success. Nicotine Tob Res 2012;14(4):383-7.
813. RefID: 1963. Perkins KA, Scott J. Sex differences in long-term smoking cessation rates due to nicotine patch. Nicotine Tob Res 2008;10(7):1245-50.
814. RefID: 655. Perraudin C, Bugnon O, Pelletier-Fleury N. Expanding professional pharmacy services in European community setting: Is it cost-effective? A systematic review for health policy considerations. Health Policy 2016;120(12):1350-62.
815. RefID: 1452. Peters EN, Budney AJ, Carroll KM. Clinical correlates of co-occurring cannabis and tobacco use: a systematic review. Addiction 2012;107(8):1404-17.
816. RefID: 3351. Peters MJ. Electronic cigarettes: Adolescent health and wellbeing. The Lancet 2018;392(10146):473-4.
817. RefID: 3452. Phillips JC, Oliffe JL, Ensom MHH, Bottorff JL, Bissell LJL, Boomer J, et al. An overlooked majority: HIV-positive gay men who smoke. Journal of Men's Health 2012;9(1):17-24.
818. RefID: 1935. PHS Guideline Update Panel LaS. Treating tobacco use and dependence: 2008 update U.S. Public Health Service Clinical Practice Guideline executive summary. Respir Care 2008;53(9):1217-22.
819. RefID: 968. Picciotto MR, Lewis AS, van Schalkwyk GI, Mineur YS. Mood and anxiety regulation by nicotinic acetylcholine receptors: A potential pathway to modulate aggression and related behavioral states. Neuropharmacology 2015;96(Pt B):235-43.
820. RefID: 2582. Pineda JRET, Kim ESH, Osinbowale OO. Impact of pharmacologic interventions on peripheral artery disease. Prog Cardiovasc Dis 2015;57(5):510-20. [PMID: http://www.elsevier.com/inca/publications/store/6/2/3/3/2/9/index.htt]
821. RefID: 603. Pineiro B, Simmons VN, Palmer AM, Correa JB, Brandon TH. Smoking cessation interventions within the context of Low-Dose Computed Tomography lung cancer screening: A systematic review. Lung Cancer 2016;98:91-8.
822. RefID: 1074. Pipe AL. Network meta-analysis demonstrates the safety of pharmacotherapy for smoking cessation in cardiovascular patients. Evid Based Med 2014;19(5):193
823. RefID: 1018. Pisinger C, Dossing M. A systematic review of health effects of electronic cigarettes. Prev Med 2014;69:248-60.
824. RefID: 712. Pita-Fernandez S, Seijo-Bestilleiro R, Pertega-Diaz S, Alonso-Hernandez A, Fernandez-Rivera C, Cao-Lopez M, et al. A randomized clinical trial to determine the effectiveness of CO-oximetry and anti-smoking brief advice in a cohort of kidney transplant patients who smoke: study protocol for a randomized controlled trial. Trials 2016 Apr 1;17:174. [PMID: 27036112]
825. RefID: 3159. Plattner A, Dantz B. Tricyclic antidepressants: An underutilized treatment? Part II. Psychopharm Rev 2011;46(3):17-23.
826. RefID: 1478. Poghosyan H, Kennedy Sheldon L, Cooley ME. The impact of computed tomography screening for lung cancer on smoking behaviors: a teachable moment? Cancer Nurs 2012;35(6):446-75.
827. RefID: 3276. Pollard A, Eakin E, Vardy J, Hawkes A. Health behaviour interventions for cancer survivors: An overview of the evidence and contemporary Australian trials. Cancer Forum 2009;33(3):182-6.
828. RefID: 828. Polosa R, Campagna D, Caponnetto P. What to advise to respiratory patients intending to use electronic cigarettes. Discov medicin 2015;20(109):155-61.
829. RefID: 772. Polosa R, Campagna D, Sands MF. Counseling patients with asthma and allergy about electronic cigarettes: an evidence-based approach. Ann Allergy Asthma Immunol 2016;116(2):106-11.
830. RefID: 1000. Poorman E, Gazmararian J, Parker RM, Yang B, Elon L. Use of text messaging for maternal and infant health: a systematic review of the literature. Matern Child Health J 2015;19(5):969-89.
831. RefID: 3260. Popova S, Patra J, Rehm J. Avoidable portion of tobacco-attributable acute care hospital days and its cost due to implementation of different intervention strategies in Canada. Int J Environ Res Public Health 2009;6(8):2179-92. [PMID: http://www.mdpi.com/1660-4601/6/8/2179/pdf]
832. RefID: 2106. Porter BL, Adkins S, Shubrook JH. Outpatient interventions for smoking cessation: The pharmacist's role as an extender. Osteopath Fam Phys 2016;8(1):34-40. [PMID: https://imis.acofp.org/ACOFPIMIS/Acofporg/PDFs/open/Jan_Feb2016.pdf]
833. RefID: 1398. Porterfield DS, Hinnant LW, Kane H, Horne J, McAleer K, Roussel A. Linkages between clinical practices and community organizations for prevention: a literature review and environmental scan. Am J Prev Med 2012;42(6 Suppl 2):S163-S171
834. RefID: 159. Potvin S, Lalonde M. [Psychosis and addiction: The evidence cemetery]. Sante Ment Que 2014;39(2):75-98.
835. RefID: 1723. Poulsen PB, Dollerup J, Moller AM. Is a percentage a percentage? Systematic review of the effectiveness of Scandinavian behavioural modification smoking cessation programmes. Clin Respir J 2010;4(1):3-12.
836. RefID: 1783. Power J, Grealy C, Rintoul D. Tobacco interventions for Indigenous Australians: a review of current evidence. HEALTH PROMOT J AUST 2009;20(3):186-94.
837. RefID: 3506. Powers MB, Emmelkamp PMG. Response to 'Is Acceptance and Commitment Therapy superior to established treatment comparisons?'. Psychotherapy and Psychosomatics 2009;78(6):380-1.
838. RefID: 1367. Practice Committee of the American Society for Reproductive Medicine. Smoking and infertility: a committee opinion. Fertil Steril 2012;98(6):1400-6.
839. RefID: 2417. Printz C. UCSF study: E-cigarettes are not helping smokers quit. Cancer 2016;122(11):1636-7. [PMID: http://onlinelibrary.wiley.com/journal/10.1002/(ISSN)1097-0142]
840. RefID: 2513. Prochaska JJ, Benowitz NL. Smoking cessation and the cardiovascular patient. Curr Opin Cardiol 2015;30(5):506-11. [PMID: http://journals.lww.com/co-cardiology/pages/default.aspx]
841. RefID: 2522. Prochaska JJ. Nicotine replacement therapy as a maintenance treatment. J Am Med Assoc 2015;314(7):718-9. [PMID: http://jama.jamanetwork.com/article.aspx?articleid=2428943]
842. RefID: 496. Prochaska JJ, Fromont SC, Leek D, Hudmon KS, Louie AK, Jacobs MH, et al. Evaluation of an evidence-based tobacco treatment curriculum for psychiatry residency training programs. Acad Psychiatry 2008;32(6):484-92.
843. RefID: 1525. Prochaska JJ, Benowitz NL, Glantz SA, Hudmon KS, Grossman W. Cardiology Rx for Change: improving clinical attention to tobacco use and secondhand smoke exposure in cardiology. Clin Cardiol 2011;34(12):738-43.
844. RefID: 780. Prochaska JJ, Benowitz NL. The Past, Present, and Future of Nicotine Addiction Therapy. Annu Rev Med 2016;67:467-86.
845. RefID: 3387. Prochaska JJ. Commentary on Roberts et al. (2016): Bupropion and varenicline are efficacious and well-tolerated cessation medications for smokers with serious mental illness. Addiction 2016;111(4):613-4.
846. RefID: 578. Prokhorov AV, Calabro KS, Tami-Maury I. Nicotine and Tobacco Use Prevention among Youth and Families. Semin Oncol Nurs 2016;32(3):197-205.
847. RefID: 2558. Prud'Homme M, Cata R, Jutras-Aswad D. Cannabidiol as an Intervention for Addictive Behaviors: A Systematic Review of the Evidence. Subst Abuse 2015;9:33-8. [PMID: 26056464]
848. RefID: 335. Pujol CN, Paasche C, Laprevote V, Trojak B, Vidailhet P, Bacon E, et al. Cognitive effects of labeled addictolytic medications. Prog Neuropsychopharmacol Biol Psychiatry 2018;81:306-32.
849. RefID: 441. Pulakka A, Halonen JI, Kawachi I, Pentti J, Stenholm S, Jokela M, et al. Association Between Distance From Home to Tobacco Outlet and Smoking Cessation and Relapse. JAMA Intern Med 2016;176(10):1512-9.
850. RefID: 1737. Purvis TL, Nelson LA, Mambourg SE. Varenicline use in patients with mental illness: an update of the evidence. Expert Opin Drug Saf 2010;9(3):471-82.
851. RefID: 1413. Quintiliani L, Stoddard A, Lederman R, Harden E, Wallace L, Sorensen G. Dissemination of a tobacco cessation program for unionized workers. Fam Community Health 2012;35(3):246-55.
852. RefID: 3184. Rabasseda X. A report from the 106th International Conference of the American Thoracic Society (May 14-19, 2010 - New Orleans, Louisiana, USA). Drugs Today 2010;46(8):609-29.
853. RefID: 2748. Rahman MA, Hann N, Wilson A, Worrall-Carter L. Electronic cigarettes: Patterns of use, health effects, use in smoking cessation and regulatory issues. Tob induc dis 2014;12(1):21. [PMID: http://www.tobaccoinduceddiseases.com/]
854. RefID: 1473. Ramo DE, Liu H, Prochaska JJ. Tobacco and marijuana use among adolescents and young adults: a systematic review of their co-use. Clin Psychol Rev 2012;32(2):105-21.
855. RefID: 1880. Ramseier CA, Fundak A. Tobacco use cessation provided by dental hygienists. Int j dent hyg 2009;7(1):39-48.
856. RefID: 980. Ramseier CA, Suvan JE. Behaviour change counselling for tobacco use cessation and promotion of healthy lifestyles: a systematic review. J Clin Periodontol 2015;42 Suppl 16:S47-S58
857. RefID: 3001. Raupach T, Brown J. Treatment of tobacco addiction and the cardiovascular specialist. Curr Opin Cardiol 2012;27(5):525-32.
858. RefID: 1172. Raupach T, Brown J, Herbec A, Brose L, West R. A systematic review of studies assessing the association between adherence to smoking cessation medication and treatment success. Addiction 2014;109(1):35-43.
859. RefID: 1831. Raw M, Regan S, Rigotti NA, McNeill A. A survey of tobacco dependence treatment guidelines in 31 countries. Addiction 2009;104(7):1243-50.
860. RefID: 1677. Raw M, McNeill A, Murray R. Case studies of tobacco dependence treatment in Brazil, England, India, South Africa and Uruguay. Addiction 2010;105(10):1721-8.
861. RefID: 2004. Rees M, Stevenson J, British Menopause Society. Primary prevention of coronary heart disease in women. Menopause Int 2008;14(1):40-5.
862. RefID: 77. Reeves S, Bernstein I. Effects of maternal tobacco-smoke exposure on fetal growth and neonatal size. Expert rev obstet gynecol 2008;3(6):719-30.
863. RefID: 617. Register SJ, Harrington KF, Agne AA, Cherrington AL. Effectiveness of Non-Primary Care-Based Smoking Cessation Interventions for Adults with Diabetes: A Systematic Literature Review. Curr Diab Rep 2016;16(9):81
864. RefID: 292. Rehan HS, Maini J, Hungin APS. Vaping versus Smoking: A Quest for Efficacy and Safety of E-cigarette. Curr Drug Saf 2018;13(2):92-101.
865. RefID: 2005. Rehring TF, Stolcpart RS, Hollis HWJ, Society for Vascular Surgery. Pharmacologic risk factor management in peripheral arterial disease: a vade mecum for vascular surgeons. J Vasc Surg 2008;47(5):1108-15.
866. RefID: 3416. Reich-Erkelenz D, Schmitt A, Falkai P. Psychiatrists' self-stigma, the DGPPN guideline for psychosocial interventions, and contemporary treatment strategies. Eur Arch Psychiatry Clin Neurosci 2015;265(3):171-2.
867. RefID: 3144. Reid RD, Mullen KA, Pipe AL. Systematic approaches to smoking cessation in the cardiac setting. Curr Opin Cardiol 2011;26(5):443-8.
868. RefID: 1303. Reitzel LR, Li Y, Stewart DW, Cao Y, Wetter DW, Waters AJ, et al. Race moderates the effect of menthol cigarette use on short-term smoking abstinence. Nicotine Tob Res 2013;15(5):883-9.
869. RefID: 3314. Reus VI, Smith BJ. Multimodal techniques for smoking cessation: A review of their efficacy and utilisation and clinical practice guidelines. Int J Clin Pract 2008;62(11):1753-68.
870. RefID: 1296. Richardson A, Graham AL, Cobb N, Xiao H, Mushro A, Abrams D, et al. Engagement promotes abstinence in a web-based cessation intervention: cohort study. J Med Internet Res 2013;15(1):e14
871. RefID: 3307. Richter D. Smoking: A deadly pleasant habit - Is there a way out? Angiology 2008;59(2_suppl):49S-53S. [PMID: http://70.107.241.254/index.htm]
872. RefID: 989. Richter KP, Ellerbeck EF. It's time to change the default for tobacco treatment. Addiction 2015;110(3):381-6.
873. RefID: 541. Richter KP, Hunt JJ, Cupertino AP, Gajewski BJ, Jiang Y, Marquis J, et al. Commitment and capacity for providing evidence-based tobacco treatment in US drug treatment facilities. Subst Abus 2017;38(1):35-9.
874. RefID: 2866. Rigotti NA, Clair C. Managing tobacco use: The neglected cardiovascular disease risk factor. Eur Heart J 2013;34(42):3259-67.
875. RefID: 243. Rink M, Crivelli JJ, Shariat SF, Chun FK, Messing EM, Soloway MS. Smoking and Bladder Cancer: A Systematic Review of Risk and Outcomes. Eur Urol Focus 2015;1(1):17-27.
876. RefID: 1271. Rinker B. The evils of nicotine: an evidence-based guide to smoking and plastic surgery. Ann Plast Surg 2013;70(5):599-605.
877. RefID: 1625. Ritter C, Stover H, Levy M, Etter JF, Elger B. Smoking in prisons: the need for effective and acceptable interventions. J Public Health Policy 2011;32(1):32-45.
878. RefID: 797. Rizer AM, Mauery DR, Haynes SG, Couser B, Gruman C. Challenges in Intervention Research for Lesbian and Bisexual Women. LGBT health 2015;2(2):105-12.
879. RefID: 3142. Robinson JG, Goldberg AC. Treatment of adults with familial hypercholesterolemia and evidence for treatment: Recommendations from the National Lipid Association Expert Panel on Familial Hypercholesterolemia. J Clin Lipidology 2011;5(3 SUPPL.):S18-S29
880. RefID: 2040. Robinson LA, Emmons KM, Moolchan ET, Ostroff JS. Developing smoking cessation programs for chronically ill teens: lessons learned from research with healthy adolescent smokers. J Pediatr Psychol 2008;33(2):133-44.
881. RefID: 1372. Robinson LM, Vail SR. An integrative review of adolescent smoking cessation using the Transtheoretical Model of Change. J Pediatr Health Care 2012;26(5):336-45.
882. RefID: 1292. Robson AK, Paleri V. Role of dietetics, psychology, dentistry and physiotherapy in head and neck cancer treatment: the evidence. Clin Otolaryngol 2013;38(1):66-8.
883. RefID: 3353. Robson D, McNeill A. Answering the question or questioning the answer? Addiction 2018;113(3):407-9.
884. RefID: 3101. Robson N. Varenicline: A new pharmacotherapy for smoking cessation in primary care practice. S Afr Fam Pract 2011;53(3):217-22. [PMID: http://www.safpj.co.za/index.php/safpj/article/view/1421/2336]
885. RefID: 93. Romijnders KAGJ, van Osch L, de Vries H, Talhout R. Perceptions and Reasons Regarding E-Cigarette Use among Users and Non-Users: A Narrative Literature Review. Int J Environ Res Public Health 2018;15(6):
886. RefID: 1527. Rooke TW, Hirsch AT, Misra S, Sidawy AN, Beckman JA, Findeiss LK, et al. 2011 ACCF/AHA focused update of the guideline for the management of patients with peripheral artery disease (updating the 2005 guideline): a report of the American College of Cardiology Foundation/American Heart Association Task Force on Practice Guidelines: developed in collaboration with the Society for Cardiovascular Angiography and Interventions, Society of Interventional Radiology, Society for Vascular Medicine, and Society for Vascular Surgery. J Vasc Surg 2011;54(5):e32-e58
887. RefID: 1713. Rosen LJ, Ben Noach M, Rosenberg E. Missing the forest (plot) for the trees? A critique of the systematic review in tobacco control. BMC Med Res Methodol 2010;10:34
888. RefID: 1751. Rosen LJ, Ben Noach M. Systematic reviews on tobacco control from Cochrane and the Community Guide: different methods, similar findings. J Clin Epidemiol 2010;63(6):596-606.
889. RefID: 249. Rosen LJ, Peled-Raz M. Tobacco policy in Israel: 1948-2014 and beyond. Isr J Health Policy Res 2015;4:12
890. RefID: 167. Rosen LJ, Galili T, Kott J, Goodman M, Freedman LS. Diminishing benefit of smoking cessation medications during the first year: a meta-analysis of randomized controlled trials. Addiction 2018;113(5):805-16.
891. RefID: 2518. Rosendorff C. Treatment: special conditions: Co-existing heart disease: coronary artery disease, myocardial infarction, heart failure. J Am Soc Hypertens 2015;9(8):651-4. [PMID: http://www.elsevier.com]
892. RefID: 1623. Rothrauff TC, Eby LT. Counselors' knowledge of the adoption of tobacco cessation medications in substance abuse treatment programs. Am J Addict 2011;20(1):56-62.
893. RefID: 3151. Rowe BH, Bhutani M, Stickland MK, Cydulka R. Assessment and management of chronic obstructive pulmonary disease in the emergency department and beyond. Expert Rev Respir Med 2011;5(4):549-59.
894. RefID: 2737. Rowland K. Review: Nicotine replacement therapy increases CVD events; Bupropion and varenicline do not. Ann Intern Med 2014;160(8):JC2. [PMID: http://annals.org/data/Journals/AIM/929992/0000605-201404150-02002.pdf]
895. RefID: 2746. Ruiz-Canela M, Martinez-Gonzalez MA. Lifestyle and dietary risk factors for peripheral artery disease. Circ J 2014;78(3):553-9. [PMID: https://www.jstage.jst.go.jp/article/circj/78/3/78_CJ-14-0062/_pdf]
896. RefID: 1133. Ruther T, Bobes J, De HM, Svensson TH, Mann K, Batra A, et al. EPA guidance on tobacco dependence and strategies for smoking cessation in people with mental illness. Eur Psychiatry 2014 Feb;29(2):65-82. [PMID: 24485753]
897. RefID: 3445. Rutqvist LE, Fry JS, Lee PN. Systematic review of Swedish snus for smoking cessation based on primary subject data from randomised clinical trials. J Smok Cessat 2013;8(1):33-44.
898. RefID: 2799. Ryden L, Grant PJ, Anker SD, Berne C, Cosentino F, Danchin N, et al. ESC guidelines on diabetes, pre-diabetes, and cardiovascular diseases developed in collaboration with the EASD. Eur Heart J 2013;34(39):3035-87.
899. RefID: 2765. Ryden L, Grant PJ, Anker SD, Berne C, Cosentino F, Danchin N, et al. ESC Guidelines on diabetes, pre-diabetes, and cardiovascular diseases developed in collaboration with the EASD - Summary The Task Force on diabetes, pre-diabetes, and cardiovascular diseases of the European Society of Cardiology (ESC) and developed in collaboration with the European Association for the Study of Diabetes (EASD). Diabets Vasc Dis Res 2014;11(3):133-73.
900. RefID: 3862. Saeterdal I, Ringerike T, Odgaard-Jensen J, Harboe I, Hagen G, Reikvam A, et al. Drugs for smoking cessation (Structured abstract). Health Technology Assessment Database 2016;(2016 Issue 4):
901. RefID: 82. Salloum NC, Buchalter EL, Chanani S, Espejo G, Ismail MS, Laine RO, et al. From genes to treatments: a systematic review of the pharmacogenetics in smoking cessation. Pharmacogenomics 2018;19(10):861-71.
902. RefID: 2750. Samet JM. Cytisine is effective for smoking cessation: Should clinicians use it? Evid Based Med 2014;19(4):134. [PMID: http://ebm.bmj.com/content/19/4/134.full.pdf+html]
903. RefID: 3476. Samuels L. Varenicline: Cardiovascular safety. Canadian Medical Association Journal 2011;183(12):1407-8.
904. RefID: 117. San-Juan-Rodriguez A, Newman TV, Hernandez I, Swart ECS, Klein-Fedyshin M, Shrank WH, et al. Impact of community pharmacist-provided preventive services on clinical, utilization, and economic outcomes: An umbrella review. Prev Med 2018;115:145-55.
905. RefID: 1500. Sangthong R, Wichaidit W, Ketchoo C. Current situation and future challenges of tobacco control policy in Thailand. Tob Control 2012;21(1):49-54.
906. RefID: 2120. Santus P, Bassi L, Airoldi A, Giovannelli F, Radovanovic D. Varenicline and evaluated outcomes in smoking cessation programmes. Monaldi Arch Chest Dis Pulm Ser 2013;79(1):20-6. [PMID: http://www.archest.fsm.it/pne/pdf/79/01/pne79-1-05-santus.pdf]
907. RefID: 868. Sarin J, Hunt J, Ivers R, Smyth C. Lifting the burden: a coordinated approach to action on Aboriginal tobacco resistance and control in NSW. Public health res pract 2015;25(3):e2531528
908. RefID: 618. Sarna L, Bialous SA, Tobacco Control Sub-group and Health Behavior Expert Panel AAoN. Nursing scholarship and leadership in tobacco control. Nurs Outlook 2013;61(3):181-2.
909. RefID: 3216. Satur JG, Gussy MG, Morgan MV, Calache H, Wright C. Review of the evidence for oral health promotion effectiveness. Health Educ J 2010;69(3):257-66.
910. RefID: 1827. Schane RE, Glantz SA, Ling PM. Social smoking implications for public health, clinical practice, and intervention research. Am J Prev Med 2009;37(2):124-31.
911. RefID: 1894. Schepis TS, Rao U. Smoking cessation for adolescents: a review of pharmacological and psychosocial treatments. Curr Drug Abuse Rev 2008;1(2):142-55.
912. RefID: 501. Scherma M, Fadda P, Le Foll B, Forget B, Fratta W, Goldberg SR, et al. The endocannabinoid system: a new molecular target for the treatment of tobacco addiction. CNS Neurol Disord Drug Targets 2008;7(5):468-81.
913. RefID: 446. Schindler-Ruwisch JM, Abroms LC, Bernstein SL, Heminger CL. A content analysis of electronic health record (EHR) functionality to support tobacco treatment. Transl Behav Med 2017;7(2):148-56.
914. RefID: 1966. Schmelzle J, Rosser WW, Birtwhistle R. Update on pharmacologic and nonpharmacologic therapies for smoking cessation. Can Fam Physician 2008;54(7):994-9.
915. RefID: 1017. Schmidt AM, Ranney LM, Goldstein AO. Communicating program outcomes to encourage policymaker support for evidence-based state tobacco control. Int J Environ Res Public Health 2014;11(12):12562-74.
916. RefID: 2720. Schneider KI, Schmidtke J. Patient compliance based on genetic medicine: A literature review. J community genet 2014;5(1):31-48. [PMID: http://www.springer.com/biomed/human+genetics/journal/12687]
917. RefID: 1747. Schneider S, Huy C, Schutz J, Diehl K. Smoking cessation during pregnancy: a systematic literature review. Drug Alcohol Rev 2010;29(1):81-90.
918. RefID: 3404. Schneider T, De Jesus S, Prapavessis H. The effect of acute exercise on smoking topography: No evidence for cutting down one puff at a time. J Smok Cessat 2015;10(2):146-53.
919. RefID: 165. Schrader C, Lenton A, Gertonson P, Rahimi A. Redeveloping Substance Abuse Treatment for Military Personnel. Curr Psychiatry Rep 2018;20(6):45
920. RefID: 1066. Schraufnagel DE, Blasi F, Drummond MB, Lam DCL, Latif E, Rosen MJ, et al. Electronic cigarettes. A position statement of the forum of international respiratory societies. Am J Respir Crit Care Med 2014;190(6):611-8.
921. RefID: 1776. Schultz ASH, Bottorff JL, McKeown SB. Nurses' use of qualitative research approaches to investigate tobacco use and control. Annu Rev Nurs Res 2009;27:115-44.
922. RefID: 179. Schwindt R, Hudmon KS, Knisely M, Davis L, Pike C. Impact of Tobacco Quitlines on Smoking Cessation in Persons With Mental Illness: A Systematic Review. J Drug Educ 2017;47(1-2):68-81.
923. RefID: 1229. Schwindt RG, Sharp D. Making a case for systematic integration of theory-based tobacco education into graduate psychiatric/mental health nursing curriculum. Arch Psychiatr Nurs 2013;27(4):166-70.
924. RefID: 2719. Seah J-M, Yao H, MacIsaac RJ, Ekinci EI, Jerums G. Reducing the complications of type 2 diabetes: Challenges in individualising care. Med Today 2014;15(3):37-47. [PMID: http://www.medicinetoday.com.au/]
925. RefID: 2544. Selby P, deRuiter WK. Call to action: Training in tobacco addiction in Canada. Can J Resp Ther 2015;51(4):83-4. [PMID: http://www.pulsus.com/pdfs/open/cjrt/17162_selb.pdf]
926. RefID: 1676. Selby P, van Mierlo T, Voci SC, Parent D, Cunningham JA. Online social and professional support for smokers trying to quit: an exploration of first time posts from 2562 members. J Med Internet Res 2010;12(3):e34
927. RefID: 269. Senore C, Giordano L, Bellisario C, Di Stefano F, Segnan N. Population based cancer screening programmes as a teachable moment for primary prevention interventions. A review of the literature. Front oncol 2012;2:45
928. RefID: 1390. Sepehripour AH, Lo TT, McCormack DJ, Shipolini AR. Is there benefit in smoking cessation prior to cardiac surgery? Interact Cardiovasc Thorac Surg 2012;15(4):726-32.
929. RefID: 716. Shackleton N, Jamal F, Viner RM, Dickson K, Patton G, Bonell C. School-Based Interventions Going Beyond Health Education to Promote Adolescent Health: Systematic Review of Reviews. J Adolesc Health 2016;58(4):382-96.
930. RefID: 1933. Shah SD, Wilken LA, Winkler SR, Lin SJ. Systematic review and meta-analysis of combination therapy for smoking cessation. J Am Pharm Assoc (2003) 2008;48(5):659-65.
931. RefID: 584. Shahwan S, Fauziana R, Satghare P, Vaingankar J, Picco L, Chong SA, et al. Qualitative study of Singaporean youths' perception of antismoking campaigns: what works and what does not. Tob Control 2016;25(e2):e101-e106
932. RefID: 3225. Shannon R, Davies E. How motivational interviewing can help patients change their lifestyles. Clin Pharm 2010;2(1):28-30. [PMID: http://www.pjonline.com/fileproxy/10216]
933. RefID: 2221. Sharma A, Thakar S, Lavie CJ, Garg J, Krishnamoorthy P, Sochor O, et al. Cardiovascular Adverse Events Associated with Smoking-Cessation Pharmacotherapies. Curr Cardiol Rep 2014;17(1). [PMID: http://link.springer.com/journal/volumesAndIssues/11886]
934. RefID: 2552. Sharma D, Kar SK. Recent advances in pharmacological management of substance use disorders. Int J Pharm Invest 2015;5(4):118-27. [PMID: http://www.jpionline.org/]
935. RefID: 2441. Sharma N, Ho KY. The medical management of gastro-oesophageal reflux disease. Inflamm Intest Dis 2016;1(2):96-9. [PMID: http://www.karger.com/Journal/Home/261895]
936. RefID: 2137. Sharma R, Alla K, Pfeffer D, Meurk C, Ford P, Kisely S, et al. An appraisal of practice guidelines for smoking cessation in people with severe mental illness. Aust New Zealand J Psychiatry 2017;51(11):1106-20. [PMID: http://anp.sagepub.com/content/by/year]
937. RefID: 668. Shaw R, Beasley N. Aetiology and risk factors for head and neck cancer: United Kingdom National Multidisciplinary Guidelines. J Laryngol Otol 2016;130(S2):S9-S12
938. RefID: 1817. Sheffer CE, Barone CP, Anders ME. Training health care providers in the treatment of tobacco use and dependence: pre- and post-training results. J Eval Clin Pract 2009;15(4):607-13.
939. RefID: 1768. Shelley D, Nguyen N, Peng CH, Chin M, Chang Md, Fahs M. Increasing access to evidence-based smoking cessation treatment: effectiveness of a free nicotine patch program among Chinese immigrants. J Immigr Minor Health 2010;12(2):198-205.
940. RefID: 1526. Shi Y, Warner DO. Brief preoperative smoking abstinence: is there a dilemma? Anesth Analg 2011;113(6):1348-51.
941. RefID: 1969. Shields AE, Levy DE, Blumenthal D, Currivan D, McGinn-Shapiro M, Weiss KB, et al. Primary care physicians' willingness to offer a new genetic test to tailor smoking treatment, according to test characteristics. Nicotine Tob Res 2008;10(6):1037-45.
942. RefID: 3085. Shields PG. Long-term nicotine replacement therapy: Cancer risk in context. Cancer Prev Res 2011;4(11):1719-23. [PMID: http://cancerpreventionresearch.aacrjournals.org/content/4/11/1719.full.pdf+html]
943. RefID: 1945. Shiffman S, Ferguson SG. The effect of a nicotine patch on cigarette craving over the course of the day: results from two randomized clinical trials. Curr Med Res Opin 2008;24(10):2795-804.
944. RefID: 2008. Shiffman S, Ferguson SG. Nicotine patch therapy prior to quitting smoking: a meta-analysis. Addiction 2008;103(4):557-63.
945. RefID: 1138. Shimony A, Grandi SM, Pilote L, Joseph L, O'Loughlin J, Paradis G, et al. Utilization of evidence-based therapy for acute coronary syndrome in high-income and low/middle-income countries. Am J Cardiol 2014;113(5):793-7.
946. RefID: 1718. Shinn AK, Greenfield SF. Topiramate in the treatment of substance-related disorders: a critical review of the literature. J Clin Psychiatry 2010;71(5):634-48.
947. RefID: 708. Shoaib M, Buhidma Y. How can we Improve on Modeling Nicotine Addiction to Develop Better Smoking Cessation Treatments? Int Rev Neurobiol 2016;126:121-56.
948. RefID: 2963. Shrivastav R, Nazar GP, Stigler MH, Arora M. Health promotion for primordial prevention of tobacco use. Glo Heart 2012;7(2):143-50. [PMID: http://www.elsevier.com/wps/find/journaldescription.cws_home/726194/description#description]
949. RefID: 2079. Signorelli SS, Katsiki N. Oxidative stress and inflammation: Their role in the pathogenesis of peripheral artery disease with or without type 2 diabetes mellitus. Curr Vasc Pharmacol 2018;16(6):547-54. [PMID: http://www.eurekaselect.com/154598]
950. RefID: 1335. Sin HPY, Liu DTL, Lam DSC. Lifestyle modification, nutritional and vitamins supplements for age-related macular degeneration. Acta Ophthalmol (Oxf) 2013;91(1):6-11.
951. RefID: 283. Singh J, Budhiraja S. Partial nicotinic acetylcholine (alpha4beta2) agonists as promising new medications for smoking cessation. Indian J Pharmacol 2008;40(5):191-6.
952. RefID: 491. Singh T, Agaku IT, Arrazola RA, Marynak KL, Neff LJ, Rolle IT, et al. Exposure to Advertisements and Electronic Cigarette Use Among US Middle and High School Students. Pediatrics 2016;137(5):
953. RefID: 1978. Skara S, Kovacic L, Civljak M, Voncina L. Translation of evidence-based tobacco use prevention programming in Croatia. Eval Health Prof 2008;31(3):297-305.
954. RefID: 3270. Smetana GW, Sillman JS. Update in new medications for primary care. J Gen Intern Med 2009;24(1):111-7.
955. RefID: 1132. Smith AA, Kepka D, Yabroff KR. Advanced practice registered nurses, physician assistants and cancer prevention and screening: a systematic review. BMC Health Serv Res 2014;14:68
956. RefID: 1006. Smith AL, Chapman S, Dunlop SM. What do we know about unassisted smoking cessation in Australia? A systematic review, 2005-2012. Tob Control 2015;24(1):18-27.
957. RefID: 1282. Smith AJB, Tennison I, Roberts I, Cairns J, Free C. The carbon footprint of behavioural support services for smoking cessation. Tob Control 2013;22(5):302-7.
958. RefID: 250. Smith CN, Kraemer JD, Johnson AC, Mays D. Plain packaging of cigarettes: do we have sufficient evidence? Risk manag healthc policy 2015;8:21-30.
959. RefID: 542. Smith PH, Zhang J, Weinberger AH, Mazure CM, McKee SA. Gender differences in the real-world effectiveness of smoking cessation medications: Findings from the 2010-2011 Tobacco Use Supplement to the Current Population Survey. Drug Alcohol Depend 2017;178:485-91.
960. RefID: 96. Smith TT, Hatsukami DK, Benowitz NL, Colby SM, McClernon FJ, Strasser AA, et al. Whether to push or pull? Nicotine reduction and non-combusted alternatives - Two strategies for reducing smoking and improving public health. Prev Med 2018;117:8-14.
961. RefID: 3218. Sofuoglu M. Cognitive enhancement as a pharmacotherapy target for stimulant addiction. Addiction 2010;105(1):38-48.
962. RefID: 3120. Sofuoglu M, Duffey D, Mooney ME. Varenicline increases smoking abstinence at 6 months to a year compared with placebo or bupropion; nausea is the most commonly reported adverse effect. Evid Based Med 2011;16(4):113-4. [PMID: http://ebm.bmj.com/content/16/4/113.full.pdf]
963. RefID: 208. Soneji S. Errors in Data Input in Meta-analysis on Association Between Initial Use of e-Cigarettes and Subsequent Cigarette Smoking Among Adolescents and Young Adults. Jama, Pediatr 2018;172(1):92-3.
964. RefID: 2001. Song F, Harvey I, Lilford R. Adjusted indirect comparison may be less biased than direct comparison for evaluating new pharmaceutical interventions. J Clin Epidemiol 2008;61(5):455-63.
965. RefID: 1410. Song F, Holland R, Barton GR, Bachmann M, Blyth A, Maskrey V, et al. Self-help materials for the prevention of smoking relapse: study protocol for a randomized controlled trial. Trials 2012;13:69
966. RefID: 995. Sosnowski R, Przewozniak K. The role of the urologist in smoking cessation: why is it important? UROL ONCOL 2015;33(1):30-9.
967. RefID: 2288. Soyka M, Muller CA. Pharmacotherapy of alcoholism-an update on approved and off-label medications. Expert Opin Pharmacother 2017;18(12):1187-99.
968. RefID: 1664. Spata J, Kelsberg G, Safranek S. Clinical inquiries. Does office spirometry improve quit rates in smokers? J FAM PRACT 2010;59(10):593-4.
969. RefID: 132. Spaulding AC, Eldridge GD, Chico CE, Morisseau N, Drobeniuc A, Fils-Aime R, et al. Smoking in Correctional Settings Worldwide: Prevalence, Bans, and Interventions. Epidemiol Rev 2018;40(1):82-95.
970. RefID: 735. Spencer JC, Wheeler SB. A systematic review of Motivational Interviewing interventions in cancer patients and survivors. Patient Educ Couns 2016;99(7):1099-105.
971. RefID: 2448. Spindel ER, McEvoy CT. The Role of Nicotine in the Effects of Maternal Smoking during Pregnancy on Lung Development and Childhood Respiratory Disease. Implications for Dangers of E-Cigarettes. Am J Respir Crit Care Med 2016 Mar 1;193(5):486-94. [PMID: 26756937]
972. RefID: 950. Spohr SA, Nandy R, Gandhiraj D, Vemulapalli A, Anne S, Walters ST. Efficacy of SMS Text Message Interventions for Smoking Cessation: A Meta-Analysis. J Subst Abuse Treat 2015;56:1-10.
973. RefID: 3492. Spring B, McFadden HG, Rademaker AW, Hitsman B. Behavioral interventions to promote smoking cessation and prevent weight gain: A reply. Addiction 2011;106(3):674-5.
974. RefID: 859. Staccini P, Fernandez-Luque L. Health Social Media and Patient-Centered Care: Buzz or Evidence? Findings from the Section "Education and Consumer Health Informatics" of the 2015 Edition of the IMIA Yearbook. Yearb med inform 2015;10(1):160-3.
975. RefID: 1653. Stahre M, Okuyemi KS, Joseph AM, Fu SS. Racial/ethnic differences in menthol cigarette smoking, population quit ratios and utilization of evidence-based tobacco cessation treatments. Addiction 2010;105 Suppl 1:75-83.
976. RefID: 107. Stanisce L, Levin K, Ahmad N, Koshkareva Y. Reviewing smokeless tobacco epidemiology, carcinogenesis, and cessation strategy for otolaryngologists. Laryngoscope 2018;128(9):2067-71.
977. RefID: 416. Stanley TD, Massey S. Evidence of nicotine replacement's effectiveness dissolves when meta-regression accommodates multiple sources of bias. J Clin Epidemiol 2016;79:41-5.
978. RefID: 3525. Stapleton JA. Breaking away from a narrow prescribing protocol for medicinal nicotine. Addiction 2008;103(4):564-5.
979. RefID: 3499. Stapleton JA. Commentary on Banham & Gilbody (2010): The scandal of smoking and mental illness. Addiction 2010;105(7):1190-1.
980. RefID: 2115. Stearns M, Nambiar S, Nikolaev A, Semenov A, McIntos S. Towards evaluating and enhancing the reach of online health forums for smoking cessation. Netw Model Anal Health Informatics Bioinformatics 2014;3(1). [PMID: http://www.springer.com/new+%26+forthcoming+titles+%28default%29/journal/13721]
981. RefID: 352. Steed L, Sohanpal R, James WY, Rivas C, Jumbe S, Chater A, et al. Equipping community pharmacy workers as agents for health behaviour change: developing and testing a theory-based smoking cessation intervention. BMJ open 2017;7(8):e015637
982. RefID: 3005. Steg PG, James SK, Atar D, Badano LP, Lundqvist CB, Borger MA, et al. ESC Guidelines for the management of acute myocardial infarction in patients presenting with ST-segment elevation. Eur Heart J 2012;33(20):2569-619.
983. RefID: 2971. Stephens LA, Stevermer JJ. Counseling is a must with this smoking cessation aid. Journal of Family Practice 2012;61(3):156
984. RefID: 506. Stevenson L, Campbell S, Bohanna I, Gould GS, Robertson J, Clough AR. Establishing Smoke-Free Homes in the Indigenous Populations of Australia, New Zealand, Canada and the United States: A Systematic Literature Review. Int J Environ Res Public Health 2017;14(11):
985. RefID: 3233. Stramba-Badiale M. Women and research on cardiovascular diseases in Europe: A report from the European Heart Health Strategy (EuroHeart) project. Eur Heart J 2010;31(14):
986. RefID: 1126. Strong DR, Uebelacker L, Fokas K, Saritelli J, Matsko S, Abrantes AM, et al. Utilization of evidence-based smoking cessation treatments by psychiatric inpatient smokers with depression. J Addict Med 2014;8(2):77-83.
987. RefID: 943. Stubbs B, Vancampfort D, Bobes J, De Hert M, Mitchell AJ. How can we promote smoking cessation in people with schizophrenia in practice? A clinical overview. Acta Psychiatr Scand 2015;132(2):122-30.
988. RefID: 1813. Studts JL, Burris JL, Kearns DK, Worth CT, Sorrell CL. "Providers practice prevention": promoting dental hygienists' use of evidence-based treatment of tobacco use and dependence. J Dent Educ 2009;73(9):1069-82.
989. RefID: 1601. Studts JL, Burris JL, Kearns DK, Worth CT, Sorrell CL. Evidence-based tobacco cessation treatment by dental hygienists. J Dent Hyg 2011;85(1):13-21.
990. RefID: 2800. Sucala M, Schnur JB, Glazier K, Miller SJ, Green JP, Montgomery GH. Hypnosis-there's an app for that: A systematic review of hypnosis apps. Int J Clin Exp Hypn 2013;61(4):463-74.
991. RefID: 807. Suckow BD, Stone DH. Vascular surgery institutional-based quality and performance measures for the care of patients with critical limb ischemia. Semin Vasc Surg 2015;28(2):92-6.
992. RefID: 1416. Suls JM, Luger TM, Curry SJ, Mermelstein RJ, Sporer AK, An LC. Efficacy of smoking-cessation interventions for young adults: a meta-analysis. Am J Prev Med 2012;42(6):655-62.
993. RefID: 1032. Sussman S, Arriaza B, Grigsby TJ. Alcohol, tobacco, and other drug misuse prevention and cessation programming for alternative high school youth: a review. J Sch Health 2014;84(11):748-58.
994. RefID: 1702. Tait RJ, Christensen H. Internet-based interventions for young people with problematic substance use: a systematic review. Med J Aust 2010;192(11 Suppl):S15-S21
995. RefID: 927. Tam J, Day HR, Rostron BL, Apelberg BJ. A systematic review of transitions between cigarette and smokeless tobacco product use in the United States. BMC public health 2015;15:258
996. RefID: 679. Tambor M, Pavlova M, Golinowska S, Arsenijevic J, Groot W. Financial incentives for a healthy life style and disease prevention among older people: a systematic literature review. BMC Health Serv Res 2016;16 Suppl 5:426
997. RefID: 3464. Tanski SE, Wilson KM. Children and secondhand smoke: Clear evidence for action. Pediatrics 2012;129(1):170-1.
998. RefID: 3391. Tappin D. The importance of permanent cessation for pregnant smokers. Addiction 2016;111(6):991-2.
999. RefID: 2890. Tattersall MC, Johnson HM, Mason PJ. Contemporary and optimal medical management of peripheral arterial disease. Surg Clin North Am 2013;93(4):761-78.
1000. RefID: 1140. Taylor AH, Thompson TP, Greaves CJ, Taylor RS, Green C, Warren FC, et al. A pilot randomised trial to assess the methods and procedures for evaluating the clinical effectiveness and cost-effectiveness of Exercise Assisted Reduction then Stop (EARS) among disadvantaged smokers. Health Technol Assess 2014;18(4):1-324.
1001. RefID: 1943. Taylor CB, Chang VY. Issues in the dissemination of cognitive-behavior therapy. Nord J Psychiatry 2008;62 Suppl 47:37-44.
1002. RefID: 1892. Taylor DCA, Chu P, Rosen VM, Baker CL, Thompson D. Budgetary impact of varenicline in smoking cessation in the United Kingdom. Value Health 2009;12(1):28-33.
1003. RefID: 2201. Taylor GMJ, Taylor AE, Thomas KH, Jones T, Martin RM, Munafo MR, et al. Effectiveness of varenicline versus nicotine replacement therapy on long-term smoking cessation in primary care: A prospective, cohort study of electronic medical records. The Lancet 2016;388(SPEC.ISS 1):107
1004. RefID: 824. Taylor G, Girling A, McNeill A, Aveyard P. Does smoking cessation result in improved mental health? A comparison of regression modelling and propensity score matching. BMJ open 2015;5(10):e008774
1005. RefID: 1566. Taylor M, Leonardi-Bee J, Agboola S, McNeill A, Coleman T. Cost effectiveness of interventions to reduce relapse to smoking following smoking cessation. Addiction 2011;106(10):1819-26.
1006. RefID: 2790. Taylor S, Hempel S, Solloway M, Miake-Lye I, Beroes J, Shekelle P. An evidence map of the effects of acupuncture. J Altern Complement Med 2014;20(5):A91-A92
1007. RefID: 2849. Teramoto T, Sasaki J, Ishibashi S, Birou S, Daida H, Dohi S, et al. Treatment A) lifestyle modification: Executive summary of the Japan Atherosclerosis Society (JAS) guidelines for the diagnosis and prevention of atherosclerotic cardiovascular diseases in Japan - 2012 version. J Atheroscler Thromb 2013;20(12):835-49. [PMID: https://www.jstage.jst.go.jp/article/jat/20/12/20_18820/_pdf]
1008. RefID: 3501. Tessier SF, Bissette ME, Bantegnie MD, Lebeau MC. Evaluation of a tobacco prevention program in secondary schools of the fle-de-France region. Alcoologie et Addictologie 2008;30(2):173-80.
1009. RefID: 1841. Thomas PD, Mi H, Swan GE, Lerman C, Benowitz N, Tyndale RF, et al. A systems biology network model for genetic association studies of nicotine addiction and treatment. Pharmacogenet Genomics 2009;19(7):538-51.
1010. RefID: 2813. Thomas RE, Mclennan J, Perera R. Cochrane in context: School-based programmes for preventing smoking. Evid Based Child Health 2013;8(5):2041-3.
1011. RefID: 2642. Thompson E, Jennings C, Kotseva K, De BD, Hoes A, De VJ, et al. Effectiveness of the EUROACTION PLUS (EA+) preventive cardiology programme for high CVD risk smokers in modifying dietary habits and anthropometric indices. Eur Heart J 2015;36(SUPPL. 1):475
1012. RefID: 1857. Thomsen T, Tonnesen H, Moller AM. Effect of preoperative smoking cessation interventions on postoperative complications and smoking cessation. Br J Surg 2009;96(5):451-61.
1013. RefID: 553. Thomson G, Morgan H, Crossland N, Bauld L, Dykes F, Hoddinott P, et al. Unintended consequences of incentive provision for behaviour change and maintenance around childbirth. PloS one 2014;9(10):e111322
1014. RefID: 97. Thomson NC. Challenges in the management of asthma associated with smoking-induced airway diseases. Expert Opin Pharmacother 2018;19(14):1565-79.
1015. RefID: 2250. Thorkelson G, Bielefeldt K, Szigethy E. Empirically supported use of psychiatric medications in adolescents and adults with IBD. Inflammatory Bowel Dis 2016;22(6):1509-22. [PMID: http://journals.lww.com/ibdjournal/pages/default.aspx]
1016. RefID: 1832. Thorndike AN, Rigotti NA. A tragic triad: coronary artery disease, nicotine addiction, and depression. Curr Opin Cardiol 2009;24(5):447-53.
1017. RefID: 3181. Thornton JG, Coleman T, Britton J, Cooper S, Watts K, Lewis S, et al. The smoking, nicotine and pregnancy (SNAP) trial: Main results. Arch Dis Child Fetal Neonatal Ed 2011;96(SUPPL. 1). [PMID: http://fn.bmj.com/content/96/Suppl_1/Fa109.1.full.pdf]
1018. RefID: 473. Toll BA, Brandon TH, Gritz ER, Warren GW, Herbst RS, AACR Subcommittee on Tobacco and Cancer. Assessing tobacco use by cancer patients and facilitating cessation: an American Association for Cancer Research policy statement. Clin Cancer Res 2013;19(8):1941-8.
1019. RefID: 1139. Toll BA, Rojewski AM, Duncan LR, Latimer-Cheung AE, Fucito LM, Boyer JL, et al. "Quitting smoking will benefit your health": the evolution of clinician messaging to encourage tobacco cessation. Clin Cancer Res 2014;20(2):301-9.
1020. RefID: 721. Tomashefski A. The perceived effects of electronic cigarettes on health by adult users: A state of the science systematic literature review. J Am Assoc Nurse Pract 2016;28(9):510-5.
1021. RefID: 2372. Tomko RL, Bountress KE, Gray KM. Personalizing substance use treatment based on pre-treatment impulsivity and sensation seeking: A review. Drug Alcohol Depend 2016;167:1-7. [PMID: http://www.elsevier.com/locate/drugalcdep]
1022. RefID: 2113. Tomko RL, Gray KM, Oppenheimer SR, Wahlquist AE, McClure EA. Using REDCap for ambulatory assessment: Implementation in a clinical trial for smoking cessation to augment in-person data collection. Am J Drug Alcohol Abuse 2018;1-16.
1023. RefID: 960. Tonetti MS, Eickholz P, Loos BG, Papapanou P, van der Velden U, Armitage G, et al. Principles in prevention of periodontal diseases: Consensus report of group 1 of the 11th European Workshop on Periodontology on effective prevention of periodontal and peri-implant diseases. J Clin Periodontol 2015;42 Suppl 16:S5-11.
1024. RefID: 595. Tonnesen H, Nielsen PR, Lauritzen JB, Moller AM. Smoking and alcohol intervention before surgery: evidence for best practice. Br J Anaesth 2009;102(3):297-306.
1025. RefID: 1812. Tonnesen P. Smoking cessation: How compelling is the evidence? A review. Health Policy 2009;91 Suppl 1:S15-S25
1026. RefID: 1724. Tonstad S, Davies S, Flammer M, Russ C, Hughes J. Psychiatric adverse events in randomized, double-blind, placebo-controlled clinical trials of varenicline: a pooled analysis. Drug Saf 2010;33(4):289-301.
1027. RefID: 1080. Tonstad S, Gustavsson G, Kruse E, Walmsley JM, Westin A. Symptoms of nicotine toxicity in subjects achieving high cotinine levels during nicotine replacement therapy. Nicotine Tob Res 2014;16(9):1266-71.
1028. RefID: 1456. Torchalla I, Okoli CTC, Bottorff JL, Qu A, Poole N, Greaves L. Smoking cessation programs targeted to women: a systematic review. Women Health 2012;52(1):32-54.
1029. RefID: 899. Tremblay MC, Pluye P, Gore G, Granikov V, Filion KB, Eisenberg MJ. Regulation profiles of e-cigarettes in the United States: a critical review with qualitative synthesis. BMC Med 2015;13:130
1030. RefID: 3463. Troxel AB, Volpp KG. Effectiveness of financial incentives for longer term smoking cessation: Evidence of absence or absence of evidence? American Journal of Health Promotion 2012;26(4):204-7.
1031. RefID: 1289. Tsiapa G, Gkiozos I, Souliotis K, Syrigos K. Review: smoking cessation strategies in patients with lung disease. In Vivo 2013;27(2):171-6.
1032. RefID: 61. Twarozek AM, Eggert T, Puca ZG, DuPont N, Erwin DO, Fox CH, et al. Promoting Tobacco Cessation in a Community-Based Women's Health Centre. J women's health care 2015;4(2):
1033. RefID: 2321. Tzelepis F, Paul CL, Williams CM, Gilligan C, Regan T, Daly J, et al. Real-time video counselling for smoking cessation. Cochrane Database Syst Rev 2017;2017(5):CD012659. [PMID: http://as.wiley.com/WileyCDA/Brand/id-6.html]
1034. RefID: 2594. Ubben JFH, Lance MD, Buhre WF, Schreiber JU. Clinical strategies to prevent pulmonary complications in cardiac surgery: An overview. J Cardiothorac Vasc Anesth 2015;29(2):481-90. [PMID: http://www.journals.elsevier.com/journal-of-cardiothoracic-and-vascular-anesthesia/]
1035. RefID: 1556. Ubina EC, Van Sell SL, Arnold C, Woods S. Best practices guidelines for nurse practitioners regarding smoking cessation in American Indian and Alaskan Native youth. Fam Community Health 2011;34(3):266-74.
1036. RefID: 264. Umeda A, Kato T, Yamane T, Yano H, Ieiri T, Miyagawa K, et al. Does smoking cessation with varenicline worsen vascular endothelial function? BMJ open 2013;3(6):
1037. RefID: 158. Underner M, Peiffer G, Perriot J, Harika-Germaneau G, Jaafari N. [Is reduction of tobacco consumption associated with reduced risk of cardiovascular and pulmonary mortality and morbidity?]. Rev Pneumol Clin 2018;74(3):188-95.
1038. RefID: 126. Underner M, Perriot J, Peiffer G, Harika-Germaneau G, Jaafari N. [Smoking cessation: Pharmacological strategies different from standard treatments]. Rev Pneumol Clin 2018;74(4):205-14.
1039. RefID: 715. Underner M, Perriot J, Cosnes J, Beau P, Peiffer G, Meurice JC. [Smoking, smoking cessation and Crohn's disease]. Presse Med 2016;45(4 Pt 1):390-402.
1040. RefID: 5304. Upton P, Davey R, Evans M et al. Tackling Indigenous Smoking and Healthy Lifestyle programme review: a rapid review of the literature. Canberra: Australian Department of Health; 2014.
1041. RefID: 823. Ussher M. The London Exercise And Pregnant smokers (LEAP) trial: a randomised controlled trial of physical activity for smoking cessation in pregnancy with an economic evaluation. Health Technol Assess 2014;19(84):
1042. RefID: 1635. van Achterberg T, Huisman-de Waal GGJ, Ketelaar NABM, Oostendorp RA, Jacobs JE, Wollersheim HCH. How to promote healthy behaviours in patients? An overview of evidence for behaviour change techniques. Health Promot Internation 2011;26(2):148-62.
1043. RefID: 287. van Amsterdam J, van der Velde B, Schulte M, van den Brink W. Causal Factors of Increased Smoking in ADHD: A Systematic Review. Subst Use Misuse 2018;53(3):432-45.
1044. RefID: 1488. van den Brink W. Evidence-based pharmacological treatment of substance use disorders and pathological gambling. Curr Drug Abuse Rev 2012;5(1):3-31.
1045. RefID: 153. van den Heuvel JF, Groenhof TK, Veerbeek JH, van Solinge WW, Lely AT, Franx A, et al. eHealth as the Next-Generation Perinatal Care: An Overview of the Literature. J Med Internet Res 2018;20(6):e202
1046. RefID: 161. van der Plas A, Pouly S, de La Bourdonnaye G, Ng WT, Baker G, Ludicke F. Influence of smoking and smoking cessation on levels of urinary 11-dehydro thromboxane B2. Toxicol Rep 2018;5:561-7.
1047. RefID: 349. van Eerd EAM, Bech Risor M, Spigt M, Godycki-Cwirko M, Andreeva E, Francis N, et al. Why do physicians lack engagement with smoking cessation treatment in their COPD patients? A multinational qualitative study. NPJ Prim Care Respir Med 2017;27(1):41
1048. RefID: 1287. van Hasselt FM, Krabbe PFM, van Ittersum DG, Postma MJ, Loonen AJM. Evaluating interventions to improve somatic health in severe mental illness: a systematic review. Acta Psychiatr Scand 2013;128(4):251-60.
1049. RefID: 1941. van Schayck OCP, Pinnock H, Ostrem A, Litt J, Tomlins R, Williams S, et al. IPCRG Consensus statement: tackling the smoking epidemic - practical guidance for primary care. Prim care respir j 2008;17(3):185-93.
1050. RefID: 1209. van Zyl-Smit RN, Allwood B, Stickells D, Symons G, Abdool-Gaffar S, Murphy K, et al. South African tobacco smoking cessation clinical practice guideline. SAMJ, S Afr med j 2013;103(11):869-76.
1051. RefID: 2411. van AT, Hernaus D. Effect of pharmacological interventions on the fronto-cingulo-parietal cognitive control network in psychiatric disorders: A transdiagnostic systematic review of fMRI studies. Front Psychiatr 2016;7(MAY):82. [PMID: http://journal.frontiersin.org/article/10.3389/fpsyt.2016.00082/full]
1052. RefID: 1542. Vangeli E, Stapleton J, Smit ES, Borland R, West R. Predictors of attempts to stop smoking and their success in adult general population samples: a systematic review. Addiction 2011;106(12):2110-21.
1053. RefID: 1234. Vansteenkiste J, De Ruysscher D, Eberhardt WEE, Lim E, Senan S, Felip E, et al. Early and locally advanced non-small-cell lung cancer (NSCLC): ESMO Clinical Practice Guidelines for diagnosis, treatment and follow-up. Ann Oncol 2013;24 Suppl 6:vi89-vi98
1054. RefID: 781. Vasiljevic M, Ng YL, Griffin SJ, Sutton S, Marteau TM. Is the intention-behaviour gap greater amongst the more deprived? A meta-analysis of five studies on physical activity, diet, and medication adherence in smoking cessation. Br J Health Psychol 2016;21(1):11-30.
1055. RefID: 137. Vasiljevic M, St John Wallis A, Codling S, Couturier DL, Sutton S, Marteau TM. E-cigarette adverts and children's perceptions of tobacco smoking harms: an experimental study and meta-analysis. BMJ open 2018;8(7):e020247
1056. RefID: 1685. Vemer P, Rutten-van Molken MPMH, Kaper J, Hoogenveen RT, van Schayck CP, Feenstra TL. If you try to stop smoking, should we pay for it? The cost-utility of reimbursing smoking cessation support in the Netherlands. Addiction 2010;105(6):1088-97.
1057. RefID: 3007. Venkatesh S, Sinha DN. Involvement of health professionals in tobacco control in the South-East Asia Region. Indian J Cancer 2012;49(4):327-35.
1058. RefID: 606. Verbiest M, Brakema E, van der Kleij R, Sheals K, Allistone G, Williams S, et al. National guidelines for smoking cessation in primary care: a literature review and evidence analysis. NPJ Prim Care Respir Med 2017;27(1):2
1059. RefID: 940. Verplaetse TL, Weinberger AH, Smith PH, Cosgrove KP, Mineur YS, Picciotto MR, et al. Targeting the noradrenergic system for gender-sensitive medication development for tobacco dependence. Nicotine Tob Res 2015;17(4):486-95.
1060. RefID: 84. Vieira A, Reis AM, Matos LC, Machado J, Moreira A. Does auriculotherapy have therapeutic effectiveness? An overview of systematic reviews. Complement Ther Clin Pract 2018;33:61-70.
1061. RefID: 2192. Vijayvergia N, Denlinger CS. Lifestyle factors in cancer survivorship: Where we are and where we are headed. J pers med 2015;5(3):243-63. [PMID: http://www.mdpi.com/2075-4426/5/3/243/pdf]
1062. RefID: 361. Villanti AC, Collins LK, Niaura RS, Gagosian SY, Abrams DB. Menthol cigarettes and the public health standard: a systematic review. BMC public health 2017;17(1):983
1063. RefID: 3365. Villanti AC, Feirman SP, Niaura RS, Pearson JL, Glasser AM, Collins LK, et al. How do we determine the impact of e-cigarettes on cigarette smoking cessation or reduction? Review and recommendations for answering the research question with scientific rigor. Addiction 2018;113(3):391-404.
1064. RefID: 1154. Vlaanderen J, Portengen L, Schuz J, Olsson A, Pesch B, Kendzia B, et al. Effect modification of the association of cumulative exposure and cancer risk by intensity of exposure and time since exposure cessation: a flexible method applied to cigarette smoking and lung cancer in the SYNERGY Study. Am J Epidemiol 2014;179(3):290-8.
1065. RefID: 744. Vogeler T, McClain C, Evoy KE. Combination bupropion SR and varenicline for smoking cessation: a systematic review. Am J Drug Alcohol Abuse 2016;42(2):129-39.
1066. RefID: 1134. von Wartburg M, Raymond V, Paradis PE. The long-term cost-effectiveness of varenicline (12-week standard course and 12 + 12-week extended course) vs. other smoking cessation strategies in Canada. Int J Clin Pract 2014;68(5):639-46.
1067. RefID: 2550. Waghel RC, Battise DM, Ducker ML. Effectiveness of electronic cigarettes as a tool for smoking cessation or reduction. J Pharm Technol 2015;31(1):8-12. [PMID: http://pmt.sagepub.com/]
1068. RefID: 1518. Walker N, Howe C, Bullen C, McRobbie H, Glover M, Parag V, et al. Study protocol for a non-inferiority trial of cytisine versus nicotine replacement therapy in people motivated to stop smoking. BMC public health 2011;11:880
1069. RefID: 1951. Walsh RA. Over-the-counter nicotine replacement therapy: a methodological review of the evidence supporting its effectiveness. Drug Alcohol Rev 2008;27(5):529-47.
1070. RefID: 2910. Walter C, Friedmann A. Evidence supports the impact of smoking cessation protocols in periodontal therapy. Journal of Evidence-Based Dental Practice 2013;13(4):142-4.
1071. RefID: 2116. Wang J-H, Wang M, Liu S-C, Du X-F, Han M, Liu J-F, et al. A bibliometric analysis of clinical study literature of traditional Chinese medicine therapies for smoking cessation. Tob induc dis 2018;16(April):15. [PMID: http://www.tobaccoinduceddiseases.org/pdf-86330-24948?filename=A%20bibliometric%20analysis.pdf]
1072. RefID: 896. Ward KD, Siddiqi K, Ahluwalia JS, Alexander AC, Asfar T. Waterpipe tobacco smoking: The critical need for cessation treatment. Drug Alcohol Depend 2015;153:14-21.
1073. RefID: 1263. Ware JH, Vetrovec GW, Miller AB, Van Tosh A, Gaffney M, Yunis C, et al. Cardiovascular safety of varenicline: patient-level meta-analysis of randomized, blinded, placebo-controlled trials. Am J Ther 2013;20(3):235-46.
1074. RefID: 3322. Wark DM. What we can do with hypnosis: A brief note. Am J Clin Hypn 2008;51(1):29-36.
1075. RefID: 1720. Warnakulasuriya S, Dietrich T, Bornstein MM, Casals Peidro E, Preshaw PM, Walter C, et al. Oral health risks of tobacco use and effects of cessation. Int Dent J 2010;60(1):7-30.
1076. RefID: 37. Warner KE. How to Think - Not Feel - about Tobacco Harm Reduction. Nicotine Tob Res 2018;
1077. RefID: 481. Warren GW, Sobus S, Gritz ER. The biological and clinical effects of smoking by patients with cancer and strategies to implement evidence-based tobacco cessation support. Lancet Oncol 2014;15(12):e568-e580
1078. RefID: 59. Washio Y, Cassey H. Systematic Review of Interventions for Racial/Ethnic-Minority Pregnant Smokers. J Smok Cessat 2016;11(1):12-27.
1079. RefID: 1570. Weaver FM, Smith B, LaVela SL, Evans CT, Ullrich P, Miskevics S, et al. Smoking behavior and delivery of evidence-based care for veterans with spinal cord injuries and disorders. J Spinal Cord Med 2011;34(1):35-45.
1080. RefID: 1657. Webb MS, Rodriguez-Esquivel D, Baker EA. Smoking cessation interventions among Hispanics in the United States: A systematic review and mini meta-analysis. Am J Health Promot 2010;25(2):109-18.
1081. RefID: 1208. Wei X, Zou G, Gong W, Yin J, Yu Y, Walley J, et al. Cardiovascular disease risk reduction in rural China: a clustered randomized controlled trial in Zhejiang. Trials 2013;14:354
1082. RefID: 1311. Weinberger AH, Mazure CM, Morlett A, McKee SA. Two decades of smoking cessation treatment research on smokers with depression: 1990-2010. Nicotine Tob Res 2013;15(6):1014-31.
1083. RefID: 1049. Weinberger AH, Smith PH, Kaufman M, McKee SA. Consideration of sex in clinical trials of transdermal nicotine patch: a systematic review. Exp Clin Psychopharmacol 2014;22(5):373-83.
1084. RefID: 941. Weinberger AH, Smith PH, Allen SS, Cosgrove KP, Saladin ME, Gray KM, et al. Systematic and meta-analytic review of research examining the impact of menstrual cycle phase and ovarian hormones on smoking and cessation. Nicotine Tob Res 2015;17(4):407-21.
1085. RefID: 459. Weinberger AH, Funk AP, Goodwin RD. A review of epidemiologic research on smoking behavior among persons with alcohol and illicit substance use disorders. Prev Med 2016;92:148-59.
1086. RefID: 1502. Weiner E, Ball MP, Buchholz AS, Gold JM, Evins AE, McMahon RP, et al. Bupropion sustained release added to group support for smoking cessation in schizophrenia: a new randomized trial and a meta-analysis. J Clin Psychiatry 2012;73(1):95-102.
1087. RefID: 2435. Weinstein LC, Stefancic A, Cunningham AT, Hurley KE, Cabassa LJ, Wender RC. Cancer screening, prevention, and treatment in people with mental illness. CA Cancer J Clin 2016;66(2):133-51. [PMID: http://onlinelibrary.wiley.com/journal/10.3322/(ISSN)1542-4863]
1088. RefID: 1985. Weiss PA. Does smoking marijuana contribute to the risk of developing lung cancer? Clin J Oncol Nurs 2008;12(3):517-9.
1089. RefID: 2608. Welte T, Vogelmeier C, Papi A. COPD: Early diagnosis and treatment to slow disease progression. Int J Clin Pract 2015;69(3):336-49. [PMID: http://onlinelibrary.wiley.com/journal/10.1111/(ISSN)1742-1241]
1090. RefID: 2032. Welton NJ, Johnstone EC, David SP, Munafo MR. A cost-effectiveness analysis of genetic testing of the DRD2 Taq1A polymorphism to aid treatment choice for smoking cessation. Nicotine Tob Res 2008;10(1):231-40.
1091. RefID: 2465. Wen J, Hou X, Wang R, Chang Y, Wang K, Ma X, et al. Analyzing the surgical resection of lung cancer. Int J Clin Exp Med 2016;9(7):14576-85. [PMID: http://www.ijcem.com/files/ijcem0023698.pdf]
1092. RefID: 259. Wen KY, Miller SM, Kilby L, Fleisher L, Belton TD, Roy G, et al. Preventing postpartum smoking relapse among inner city women: development of a theory-based and evidence-guided text messaging intervention. JMIR Res Protoc 2014;3(2):e20
1093. RefID: 2002. Wensel TM, Stump AL. Helping patients kick the "other" habit. J FAM PRACT 2008;57(4):238-45.
1094. RefID: 2065. Werner F-M, Covenas R. Long-term administration of antipsychotic drugs in schizophrenia and influence of substance and drug abuse on the disease outcome. Curr Drug Abuse Rev 2017;10(1):19-24. [PMID: http://www.eurekaselect.com/671/journal/current-drug-abuse-reviews]
1095. RefID: 1561. West O, Hajek P, McRobbie H. Systematic review of the relationship between the 3-hydroxycotinine/cotinine ratio and cigarette dependence. Psychopharmacology (Berl) 2011;218(2):313-22.
1096. RefID: 2039. West R, Baker CL, Cappelleri JC, Bushmakin AG. Effect of varenicline and bupropion SR on craving, nicotine withdrawal symptoms, and rewarding effects of smoking during a quit attempt. Psychopharmacology (Berl) 2008;197(3):371-7.
1097. RefID: 1224. West R, May S, West M, Croghan E, McEwen A. Performance of English stop smoking services in first 10 years: analysis of service monitoring data. BMJ 2013;347:f4921
1098. RefID: 148. West R, Coyle K, Owen L, Coyle D, Pokhrel S, EQUIPT Study Group. Estimates of effectiveness and reach for 'return on investment' modelling of smoking cessation interventions using data from England. Addiction 2018;113 Suppl 1:19-31.
1099. RefID: 1869. Wexler R, Elton T, Pleister A, Feldman D. You can do more to slow the progression of heart failure. J FAM PRACT 2009;58(3):122-8.
1100. RefID: 2969. Whelton SP, Nasir K, Blaha MJ, Gransar H, Metkus TS, Coresh J, et al. Coronary artery calcium and primary prevention risk assessment: What is the evidence? An updated meta-analysis on patient and physician behavior. Circ Cardiovasc Qual Outcomes 2012;5(4):601-7.
1101. RefID: 49. White P, Skirrow H, George A, Memon A. A systematic review of economic evaluations of local authority commissioned preventative public health interventions in overweight and obesity, physical inactivity, alcohol and illicit drugs use and smoking cessation in the United Kingdom. J Public Health (Oxf) 2018;
1102. RefID: 1914. Wiehe SE. Pediatric office-based tobacco screening and prevention. Pediatr Ann 2008;37(11):733-9.
1103. RefID: 3496. Wildgust HJ, Beary M. Review: Are there modifiable risk factors which will reduce the excess mortality in schizophrenia? J Psychopharmacol 2010;24(11, Suppl 4):37-50.
1104. RefID: 2564. Wilkin T. Primary care for men who have sex with men. New Engl J Med 2015;373(9):854-62. [PMID: http://www.nejm.org/doi/pdf/10.1056/NEJMcp1401303]
1105. RefID: 1331. Willems RA, Willemsen MC, Nagelhout GE, de Vries H. Understanding smokers' motivations to use evidence-based smoking cessation aids. Nicotine Tob Res 2013;15(1):167-76.
1106. RefID: 260. Wilson DE, Van Vlack T, Schievink BP, Doak EB, Shane JS, Dean E. Lifestyle factors in hypertension drug research: systematic analysis of articles in a leading cochrane report. Int J Hypertens 2014;2014:835716
1107. RefID: 1971. Wilson N, Thomson G, Edwards R. Use of four major tobacco control interventions in New Zealand: a review. N Z Med J 2008;121(1276):71-86.
1108. RefID: 1242. Winickoff JP, Nabi-Burza E, Chang Y, Finch S, Regan S, Wasserman R, et al. Implementation of a parental tobacco control intervention in pediatric practice. Pediatrics 2013;132(1):109-17.
1109. RefID: 3185. Winstock AR, Ford C, Witton J. Assessment and management of cannabis use disorders in primary care. BMJ (Online) 2010;340(7750):800-4.
1110. RefID: 2425. Winter SJ, Sheats JL, King AC. The Use of Behavior Change Techniques and Theory in Technologies for Cardiovascular Disease Prevention and Treatment in Adults: A Comprehensive Review. Prog Cardiovasc Dis 2016;58(6):605-12.
1111. RefID: 1668. Wolfe CDA, Redfern J, Rudd AG, Grieve AP, Heuschmann PU, McKevitt C. Cluster randomized controlled trial of a patient and general practitioner intervention to improve the management of multiple risk factors after stroke: stop stroke. Stroke 2010;41(11):2470-6.
1112. RefID: 2028. Wolfenden L, Campbell E, Wiggers J, Walsh RA, Bailey LJ. Helping hospital patients quit: what the evidence supports and what guidelines recommend. Prev Med 2008;46(4):346-57.
1113. RefID: 1863. Wolfenden L, Wiggers J, Campbell E, Knight J, Kerridge R, Spigelman A. Providing comprehensive smoking cessation care to surgical patients: the case for computers. Drug Alcohol Rev 2009;28(1):60-5.
1114. RefID: 3102. Wong S, Ordean A, Kahan M, Gagnon R, Hudon L, Basso M, et al. Substance Use in Pregnancy. J Obstet Gynaecol Can 2011;33(4):367-84.
1115. RefID: 1302. Wray JM, Gass JC, Tiffany ST. A systematic review of the relationships between craving and smoking cessation. Nicotine Tob Res 2013;15(7):1167-82.
1116. RefID: 601. Wright JS, Wall HK, Briss PA, Schooley M. Million hearts--where population health and clinical practice intersect. Circ Cardiovasc Qual Outcomes 2012;5(4):589-91.
1117. RefID: 304. Writing Committee, Gerhard-Herman MD, Gornik HL, Barrett C, Barshes NR, Corriere MA, et al. 2016 AHA/ACC Guideline on the Management of Patients with Lower Extremity Peripheral Artery Disease: Executive Summary. Vasc Med 2017;22(3):NP1-NP43
1118. RefID: 2537. Xiao D, Bai C-X, Chen Z-M, Wang C. Implementation of the World Health Organization Framework Convention on Tobacco Control in China: An arduous and long-term task. Cancer 2015;121(S17):3061-8.
1119. RefID: 1100. Xu X, Aron A, Westmaas JL, Wang J, Sweet LH. An fMRI study of nicotine-deprived smokers' reactivity to smoking cues during novel/exciting activity. PloS one 2014;9(4):e94598
1120. RefID: 1984. Yano EM, Rubenstein LV, Farmer MM, Chernof BA, Mittman BS, Lanto AB, et al. Targeting primary care referrals to smoking cessation clinics does not improve quit rates: implementing evidence-based interventions into practice. Health Serv Res 2008;43(5 Pt 1):1637-61.
1121. RefID: 2511. Yardley MM, Mirbaba MM, Ray LA. Pharmacological Options for Smoking Cessation in Heavy-Drinking Smokers. CNS drugs 2015;29(10):833-45. [PMID: http://rd.springer.com/journal/40263]
1122. RefID: 535. Ybarra ML, Jiang Y, Free C, Abroms LC, Whittaker R. Participant-level meta-analysis of mobile phone-based interventions for smoking cessation across different countries. Prev Med 2016;89:90-7.
1123. RefID: 1749. Young RP, Hopkins RJ, Smith M, Hogarth DK. Smoking cessation: the potential role of risk assessment tools as motivational triggers. Postgrad Med J 2010;86(1011):26-2.
1124. RefID: 2485. Yousefzadeh A, Chung F, Wong DT, Warner D, Wong J. Smoking cessation: The role of the anesthesiologist. Anesth Analg 2016;122(5 Supplement 3):S277
1125. RefID: 351. Zainol Abidin N, Zainal Abidin E, Zulkifli A, Karuppiah K, Syed Ismail SN, Amer Nordin AS. Electronic cigarettes and indoor air quality: a review of studies using human volunteers. Rev Environ Health 2017;32(3):235-44.
1126. RefID: 3051. Zaman M, Bilal H, Mahmood S, Tang A. Does getting smokers to stop smoking before lung resections reduce their risk? Interact Cardiovasc Thorac Surg 2012;14(3):320-3.
1127. RefID: 337. Zare S, Nemati M, Zheng Y. A systematic review of consumer preference for e-cigarette attributes: Flavor, nicotine strength, and type. PloS one 2018;13(3):e0194145
1128. RefID: 684. Zarobkiewicz MK, Wozniakowski MM, Slawinski MA, Samborski PM, Wawryk-Gawda E, Jodlowska-Jedrych B. Analysis of Polish internet retail sites offering electronic cigarettes. Rocz Panstw Zakl Hig 2016;67(3):287-90.
1129. RefID: 646. Zborovskaya Y. E-Cigarettes and Smoking Cessation: A Primer for Oncology Clinicians. Clin J Oncol Nurs 2017;21(1):54-63.
1130. RefID: 1878. Zeller M, Hatsukami D, Strategic Dialogue on Tobacco Harm Reduction Group. The Strategic Dialogue on Tobacco Harm Reduction: a vision and blueprint for action in the US. Tob Control 2009;18(4):324-32.
1131. RefID: 568. Zhan Y, Liu R, Li Q, Leischow SJ, Zeng DD. Identifying Topics for E-Cigarette User-Generated Contents: A Case Study From Multiple Social Media Platforms. J Med Internet Res 2017;19(1):e24
1132. RefID: 131. Zhang G, Wang Z, Zhang K, Hou R, Xing C, Yu Q, et al. Safety Assessment of Electronic Cigarettes and Their Relationship with Cardiovascular Disease. Int J Environ Res Public Health 2018;15(1):
1133. RefID: 3413. Zhang L, Hsia J, Tu X, Xia Y, Zhang L, Bi Z, et al. Exposure to secondhand tobacco smoke and interventions among pregnant women in China: A systematic review. Preventing Chronic Disease: Public Health Research, Practice, and Policy 2015;12:
1134. RefID: 624. Zhong J, Cao S, Gong W, Fei F, Wang M. Electronic Cigarettes Use and Intention to Cigarette Smoking among Never-Smoking Adolescents and Young Adults: A Meta-Analysis. Int J Environ Res Public Health 2016;13(5):
1135. RefID: 1520. Zoorob RJ, Kihlberg C, Taylor SE. Aging and disease prevention. Clin Geriatr Med 2011;27(4):523-39.
1136. RefID: 1411. Zschucke E, Heinz A, Strohle A. Exercise and physical activity in the therapy of substance use disorders. ScientificWorldJournal 2012;2012:901741
1137. RefID: 312. Zulkifli A, Abidin EZ, Abidin NZ, Amer Nordin AS, Praveena SM, Syed Ismail SN, et al. Electronic cigarettes: a systematic review of available studies on health risk assessment. Rev Environ Health 2018;33(1):43-52.
1138. RefID: 2744. Zuo L, He F, Sergakis GG, Koozehchian MS, Stimpfl JN, Rong Y, et al. Interrelated role of cigarette smoking, oxidative stress, and immune response in COPD and corresponding treatments. Am J Physiol Lung Cell Mol Physiol 2014;307(3):L205-L218. [PMID: http://ajplung.physiology.org/content/307/3/L205.full.pdf]
1139. RefID: 2026. Zwar N. Smoking cessation--what works? Aust Fam Physician 2008;37(1-2):10-4.
1140. RefID: 1141. Zwar NA, Mendelsohn CP, Richmond RL. Supporting smoking cessation. BMJ 2014;348:f7535
1141. RefID: 2264. Zyoud SH. Estimates of global research productivity in using nicotine replacement therapy for tobacco cessation: A bibliometric study. Globalization Health 2018;14(1):14. [PMID: http://www.globalizationandhealth.com/]
1142. RefID: 1353. Adams J, Giles EL, Robalino S, McColl E, Sniehotta FF. A systematic review of the use of financial incentives and penalties to encourage uptake of healthy behaviors: protocol. Syst rev 2012;1:51
1143. RefID: 2870. Buhi ER, Trudnak TE, Martinasek MP, Oberne AB, Fuhrmann HJ, McDermott RJ. Mobile phone-based behavioural interventions for health: A systematic review. Health Educ J 2013;72(5):564-83.
1144. RefID: 328. Canuto K, Aromataris E, Lockwood C, Tufanaru C, Brown A. Aboriginal and Torres Strait Islander health promotion programs for the prevention and management of chronic diseases: a scoping review protocol. JBI Database System Rev Implement Rep 2017;15(1):10-4.
1145. RefID: 2816. Casimir YE, Williams MM, Liang MY, Pitakmongkolkul S, Slyer JT. Effectiveness of patient-centered self-care education for adults with heart failure on knowledge, self-care behaviors, quality of life, and readmissions: A systematic review protocol. JBI Database System Rev Implement Rep 2013;11(8):107-28.
1146. RefID: 3565. Chi C, Ko S, Yeh M, Wang S, Tsai Y, Hsu M. Lifestyle changes for treating psoriasis. (11):
1147. RefID: 2228. Civljak M, Bilic P, Milosevic M. Interventions for smoking cessation in psychiatric settings. Cochrane Database Syst Rev 2015;2015(6):CD011722.
1148. RefID: 2240. David SP, Bergen AW, Munafo MR, Schuit E, Bennett DA, Panagiotou OA. Genomic analysis to guide choice of treatment for smoking cessation. Cochrane Database Syst Rev 2015;2015(8):CD011823.
1149. RefID: 703. de Bruin M, Viechtbauer W, Eisma MC, Hartmann-Boyce J, West R, Bull E, et al. Identifying effective behavioural components of Intervention and Comparison group support provided in SMOKing cEssation (IC-SMOKE) interventions: a systematic review protocol. Syst rev 2016;5:77
1150. RefID: 387. Dinsdale S, Azevedo LB, Shucksmith J, Newham J, Ells LJ, Jones D, et al. Effectiveness of weight management, smoking cessation and alcohol reduction interventions in changing behaviors during pregnancy: an umbrella review protocol. JBI Database System Rev Implement Rep 2016;14(10):29-47.
1151. RefID: 417. Hyndman K, Thomas R, Patterson S, Compton S, Schira R, Godfrey C, et al. Effectiveness of tobacco intervention education in health professional students' practice: a systematic review protocol. JBI Database System Rev Implement Rep 2016;14(6):78-90.
1152. RefID: 469. Kay-Lambkin FJ, Thornton L, Lappin JM, Hanstock T, Sylvia L, Jacka F, et al. Study protocol for a systematic review of evidence for lifestyle interventions targeting smoking, sleep, alcohol/other drug use, physical activity, and healthy diet in people with bipolar disorder. Syst rev 2016;5(1):106
1153. RefID: 440. Kingkaew P, Glidewell L, Walwyn R, Fraser H, Wyatt JC. Identifying effective components for mobile health behaviour change interventions for smoking cessation and service uptake: protocol of a systematic review and planned meta-analysis. Syst rev 2017;6(1):193
1154. RefID: 2159. Kourbelis C, Franzon J, Foote J, Brown A, Daniel M, Coffee NT, et al. Effectiveness of discharge education on outcomes in acute coronary syndrome patients: A systematic review protocol. JBI Database System Rev Implement Rep 2018;16(4):817-24. [PMID: https://journals.lww.com/jbisrir/Pages/default.aspx]
1155. RefID: 2326. Lee JA, Choi J, Jun JH, Choi T-Y, Lee MS, Jang S, et al. Acupuncture for smoking cessation: A protocol for a systematic review of randomized controlled trials. Eur J Integr Med 2017;12:35-7. [PMID: http://www.elsevier.com/journals/european-journal-of-integrative-medicine/1876-3820]
1156. RefID: 701. MacDonald M, O'Leary R, Stockwell T, Reist D, Clearing the Air project team. Clearing the air: protocol for a systematic meta-narrative review on the harms and benefits of e-cigarettes and vapour devices. Syst rev 2016;5:85
1157. RefID: 329. McCausland K, Maycock B, Jancey J. The messages presented in online electronic cigarette promotions and discussions: a scoping review protocol. BMJ open 2017;7(11):e018633
1158. RefID: 191. Minian N, deRuiter WK, Lingam M, Corrin T, Dragonetti R, Manson H, et al. The effects of interventions targeting multiple health behaviors on smoking cessation outcomes: a rapid realist review protocol. Syst rev 2018;7(1):38
1159. RefID: 2255. Moi JHY, Sriranganathan MK, Edwards CJ, Buchbinder R. Lifestyle interventions for acute gout. Cochrane Database Syst Rev 2013;2013(5):CD010519. [PMID: http://as.wiley.com/WileyCDA/Brand/id-6.html]
1160. RefID: 321. Moyo F, Archibald E, Slyer JT. Effectiveness of decision aids on smoking cessation in adult patients: a systematic review protocol. JBI Database System Rev Implement Rep 2017;15(12):2881-9.
1161. RefID: 2225. Papadakis S, Pipe A, Kelly S, Pritchard G, Wells GA. Strategies to improve the delivery of tobacco use treatment in primary care practice. Cochrane Database Syst Rev 2015;2015(3):CD011556. [PMID: http://as.wiley.com/WileyCDA/Brand/id-6.html]
1162. RefID: 2226. Roelsgaard IK, Esbensen BA, Ostergaard M, Rollefstad S, Semb AG, Christensen R, et al. Smoking cessation intervention for reducing disease activity in chronic autoimmune inflammatory joint diseases. Cochrane Database Syst Rev 2018;2018(2):CD012958. [PMID: http://as.wiley.com/WileyCDA/Brand/id-6.html]
1163. RefID: 2573. Rushton M, Clark R, Brideson G, Damarell R. The effectiveness of non-pharmacological interventions for the management of cancer treatment-induced cardiotoxicity: A systematic review protocol. JBI Libr Syst Rev 2015;13(5):53-73. [PMID: http://www.joannabriggslibrary.org/jbilibrary/index.php/jbisrir/article/download/2011/2484]
1164. RefID: 462. Shingler E, Robles LA, Perry R, Penfold C, Ness A, Thomas S, et al. Tobacco and alcohol cessation or reduction interventions in people with oral dysplasia and head and neck cancer: systematic review protocol. Syst rev 2017;6(1):161
1165. RefID: 2237. Singh JA, Kalore NV, Bharat A. Perioperative interventions for smoking cessation in hip and knee arthroplasty for osteoarthritis and other non-traumatic diseases. Cochrane Database Syst Rev 2013;2013(7):CD010674. [PMID: http://as.wiley.com/WileyCDA/Brand/id-6.html]
1166. RefID: 2460. Stevenson JK, Campbell ZC, Webster AC, Chow CK, Campbell KL, Lee VWS. eHealth interventions for people with chronic kidney disease. Cochrane Database Syst Rev 2016;2016(10):CD012379. [PMID: http://as.wiley.com/WileyCDA/Brand/id-6.html]
1167. RefID: 334. Taylor G, Aveyard P, Meer RVd, Toze D, Stuijfzand B, Kessler D, et al. Impact of variation in functions and delivery on the effectiveness of behavioural and mood management interventions for smoking cessation in people with depression: protocol for a systematic review and meta-analysis. BMJ open 2017;7(11):e018617
1168. RefID: 507. Thomas KH, Caldwell D, Dalili MN, Gunnell D, Munafo MR, Stevenson M, et al. How do smoking cessation medicines compare with respect to their neuropsychiatric safety? A protocol for a systematic review, network meta-analysis and cost-effectiveness analysis. BMJ open 2017;7(6):e015414
1169. Yammine L, Kosten TR, Pimenova M, Schmitz JM. Cigarette smoking, type 2 diabetes mellitus, and glucagon-like peptide-1 receptor agonists as a potential treatment for smokers with diabetes: An integrative review. Diabetes Research and Clinical Practice. 2019;149:78–88.
1170. Worku D, Worku E. A narrative review evaluating the safety and efficacy of e-cigarettes as a newly marketed smoking cessation tool. SAGE Open Medicine [Internet]. 2019;7(no pagination). Available from: http://smo.sagepub.com/
1171. Wolf S, O’Sullivan S, Dean R, Owens T. Does utilization of electronic cigarettes facilitate smoking cessation compared to other interventions? Journal - Oklahoma State Medical Association. 2019;112(5):34–5.
1172. Willcox JC, Dobson R, Whittaker R. Old-Fashioned Technology in the Era of “Bling”: Is There a Future for Text Messaging in Health Care? Journal of Medical Internet Research. 2019;21(12):e16630.
1173. Whitemore R, Leonardi-Bee J, Naughton F, Sutton S, Cooper S, Parrott S, et al. Effectiveness and cost-effectiveness of a tailored text-message programme (MiQuit) for smoking cessation in pregnancy: study protocol for a randomised controlled trial (RCT) and meta-analysis. Trials [Electronic Resource]. 2019;20(1):280.
1174. Warren GW, Alberg A. Smoking cessation after a cancer diagnosis and survival in cancer patients. Journal of Clinical Oncology Conference [Internet]. 2018;36(15 Supplement 1). Available from: http://ascopubs.org/doi/abs/10.1200/JCO.2018.36.15_suppl.1561
1175. Warner KE, Mendez D. E-cigarettes: comparing the possible risks of increasing smoking initiation with the potential benefits of increasing smoking cessation. Nicotine and Tobacco Research. 2019;21(1):41–7.
1176. Wang JH, Wang M, Liu SC, Du XF, Han M, Liu JF, et al. A bibliometric analysis of clinical study literatures of traditional Chinese medicine therapies for smoking cessation. Global Advances in Health and Medicine. 2018;7:114.
1177. Wallace AM, Foronjy RE. Electronic cigarettes: Not evidence-based cessation. Translational lung cancer research. 2019;8(Supplement1):S7–10.
1178. Vlad C, Arnsten JH, Nahvi S. Achieving Smoking Cessation Among Persons with Opioid Use Disorder. CNS Drugs. 2020;34(4):367–87.
1179. Vinnikov D, Tutka P, Brimkulov N, Kolodziejczyk P, Courtney R. Cytisine is an effective smoking cessation medication: More evidence now than ever before. European Respiratory Journal Conference: European Respiratory Society International Congress, ERS [Internet]. 2017;50(Supplement 61). Available from: http://erj.ersjournals.com/content/50/suppl_61/PA4482
1180. Villalobos RE, Ambrocio GPL, Fernandez L. Electronic cigarettes for smoking cessation: An individual patient meta-analysis of randomized controlled trials. European Respiratory Journal Conference: 29th International Congress of the European Respiratory Society, ERS Spain [Internet]. 2019;54(Supplement 63). Available from: https://erj.ersjournals.com/content/54/suppl_63/OA5135
1181. Vijayaraghavan M, Elser H, Apollonio D. Interventions to reduce tobacco use in people experiencing homelessness. Cochrane Database of Systematic Reviews [Internet]. 2019;(9). Available from: https://ovidsp.ovid.com/ovidweb.cgi?T=JS&CSC=Y&NEWS=N&PAGE=fulltext&D=coch&AN=00075320-100000000-11813
1182. Vazquez-Beceiro P, Bort M, Marron EM, Sobera RV. Transcranial magnetic stimulation for the treatment of nicotine addiction: A systematic review. Brain Stimulation. 2019;12 (2):469.
1183. U.S. Department of Health and Human Services. Smoking Cessation: A Report of the Surgeon General [Internet]. Atlanta, GA: U.S: Department of Health and Human Services, Centers for Disease Control and Prevention, National Center for Chronic Disease Prevention and Health Promotion, Office on Smoking and Health; 2020 p. 700. Available from: www.surgeongeneral.gov
1184. Tutka P, Vinnikov D, Courtney RJ, Benowitz NL. Cytisine for nicotine addiction treatment: a review of pharmacology, therapeutics and an update of clinical trial evidence for smoking cessation. Addiction. 2019;114(11):1951–69.
1185. Tonstad S, Arons C, Rollema H, Berlin I, Hajek P, Fagerstrom K, et al. Varenicline: mode of action, efficacy, safety and accumulated experience salient for clinical populations. Current Medical Research and Opinion. 2020;36(5):713–30.
1186. Tofield A. Cardiology papers in Top 100 of Altmetric 2019. European Heart Journal. 2020;41(8):903.
1187. Taylor GMJ, McNeill A, Farley A, Lindson N, Aveyard P. Smoking cessation for improving mental health. Cochrane Database of Systematic Reviews [Internet]. 2020;2020(1). Available from: https://www.cochranelibrary.com/cdsr/table-of-contents
1188. Tammemagi M. MS18.06 Captive Audience, Teachable Moment - Integrating Tobacco Cessation in Lung Cancer Screening. Journal of Thoracic Oncology. 2019;14 (10 Supplement):S195.
1189. Stockings E, Black N, Bartlem KM, Metse AP, Regan T, Bailey JM, et al. Outpatient interventions for smoking cessation and reduction for adults with a mental disorder. Cochrane Database of Systematic Reviews [Internet]. 2019;2019(3). Available from: http://as.wiley.com/WileyCDA/Brand/id-6.html
1190. Siskind D, Wu B, Wong T, Kisely S. Pharmacological interventions for smoking cessation among people with schizophrenia: A systematic review and meta-analysis. Schizophrenia Bulletin. 2020;46 (Supplement 1):S168.
1191. Roche DJO, Bennett M, Weiner E. Pharmacological interventions to address cigarette smoking in people with schizophrenia spectrum disorders. The Lancet Psychiatry. 2020;7(9):723–4.
1192. Robinson R. What’s hot that the other lot got. Thorax. 2018;73(12):1194.
1193. Ridker PM, Koenig W, Kastelein JJ, Mac HF, Luscher TF. Has the time finally come to measure hsCRP universally in primary and secondary cardiovascular prevention? European Heart Journal. 2018;39(46):4109–11.
1194. Parikh NS, Omran SS, Elkind MS, Willey J. Pharmacological smoking cessation therapies for patients with cerebrovascular disease: A systematic review. Stroke Conference: American Heart Association/American Stroke Association [Internet]. 2019;50(Supplement 1). Available from: https://ovidsp.ovid.com/ovidweb.cgi?T=JS&CSC=Y&NEWS=N&PAGE=fulltext&D=emexa&AN=628148515
1195. Pandria N, Athanasiou A, Konstantara L, Karagianni M, Bamidis PD. Advances in biofeedback and neurofeedback studies on smoking. NeuroImage Clinical. 2020;28:102397.
1196. Ortis A, Caponnetto P, Polosa R, Urso S, Battiato S. A report on smoking detection and quitting technologies. International Journal of Environmental Research and Public Health [Internet]. 2020;17(7). Available from: https://www.mdpi.com/1660-4601/17/7/2614/pdf
1197. O’Sullivan C, Bermingham M. A systematic review of pharmacist interventions using motivational interviewing methodology. Pharmacoepidemiology and Drug Safety. 2020;29 (Supplement 2):6.
1198. Notley C, Gentry S, Livingstone-Banks J, Bauld L, Perera R, Hartman-Boyce J. Smoking cessation for cancer prevention: Can incentives play a role? Evidence from a Cochrane review. British Journal of Cancer. 2019;121 (1):9.
1199. NICE. Smoking overview. 2020.
1200. NICE. Smoking cessation in secondary care overview. 2020.
1201. Nethan S, Sinha D, Chandan K, Mehrotra R. Article 14 of WHO FCTC: Gaps in implementation & recommendations. Tobacco Induced Diseases. 2018;16 (Supplement 1):165.
1202. Minian N, Corrin T, Lingam M, deRuiter WK, Rodak T, Taylor VH, et al. Identifying contexts and mechanisms in multiple behavior change interventions affecting smoking cessation success: a rapid realist review. BMC Public Health. 2020;20(1):918.
1203. Miguel RTD, Steffensen I. E-cigarette use and cigarette smoking initiation studies: A word of caution. Tobacco Prevention & Cessation. 2019;5:37.
1204. Mersha AG, Eftekhari P, Bovill M, Tollosa DN, Gould GS. Evaluating level of adherence to nicotine replacement therapy and its impact on smoking cessation: a protocol for systematic review and meta-analysis. BMJ open. 2020;10(9):e039775.
1205. Melka AS, Chojenta CL, Holliday EG, Loxton DJ. Effectiveness of pharmacotherapy for smoking cessation: protocol for umbrella review and quality assessment of systematic reviews. Systematic reviews. 2018;7(1):210.
1206. McGregor G, Brown R. Physiotherapists using very brief interventions to make every contact count in multiple long-term conditions: a scoping review. Physiotherapy (United Kingdom). 2020;107 (Supplement 1):e180–1.
1207. McDonnell BP, Regan C. Smoking in pregnancy: pathophysiology of harm and current evidence for monitoring and cessation. Obstetrician and Gynaecologist. 2019;21(3):169–75.
1208. McDonald CF, Jones S, Beckert L, Bonevski B, Buchanan T, Bozier J, et al. Electronic cigarettes: A position statement from the Thoracic Society of Australia and New Zealand*. Respirology [Internet]. 2020; Available from: http://onlinelibrary.wiley.com/journal/10.1111/(ISSN)1440-1843
1209. Madara J, West FM, Zappetti D. What’s the Risk? Varenicline May Increase Cardiovascular Complications in Users. Clinical Pulmonary Medicine. 2018;25(6):233.
1210. Livingston CJ, Freeman RJ, Costales VC, Westhoff JL, Caplan LS, Sherin KM, et al. Electronic Nicotine Delivery Systems or E-cigarettes: American College of Preventive Medicine’s Practice Statement. American Journal of Preventive Medicine. 2019;56(1):167–78.
1211. Lin L. Use of prediction intervals in network meta-analysis. JAMA Network Open [Internet]. 2019;2(8). Available from: https://jamanetwork.com/journals/jamanetworkopen
1212. Lhommeau N, Huchet A, Castera P. [Acupuncture and smoking cessation, a review of the literature]. Revue des Maladies Respiratoires. 2020;37(6):474–8.
1213. Kwon S. Financial incentives encourage pregnant women to quit smoking. Managed Care. 2018;27(11):32–4.
1214. Kopsaftis Z, Van Agteren J, Carson K, O’Loughlin T, Smith B. Smoking cessation in the hospital setting: A systematic review and meta-analysis. European Respiratory Journal Conference: European Respiratory Society International Congress, ERS [Internet]. 2017;50(Supplement 61). Available from: http://erj.ersjournals.com/content/50/suppl_61/PA1271
1215. Klemperer EM, Lindson N. Neither NRT aided gradual cessation nor abrupt cessation is superior in producing long-term abstinence: Reconciling conflicting results from two recent meta-analyses. Tobacco Induced Diseases. 2019;17:81.
1216. Kisely S, Arroyo D, Siskind D. Safety and use of e-cigarettes as a harm minimization measure-what’s the evidence? Australian and New Zealand Journal of Psychiatry. 2019;53 (Supplement 1):101–2.
1217. King JL. Factors related to nicotine replacement therapy for adolescent smoking cessation. Dissertation Abstracts International: Section B: The Sciences and Engineering. 2019;80(7-B(E)):No-Specified.
1218. Keller KG, Lach HW. Nurse Counseling as Part of a Multicomponent Tobacco Treatment Intervention: An Integrative Review. Journal of Addictions Nursing. 2020;31(3):161–79.
1219. Kagabo R, Gordon AJ, Okuyemi K. Smoking cessation in inpatient psychiatry treatment facilities: A review. Addictive behaviors reports [Internet]. 2020;11 (no pagination). Available from: http://www.journals.elsevier.com/addictive-behaviors-reports/
1220. Jackson SE, McGowan JA, Ubhi HK, Proudfoot H, Shahab L, Brown J, et al. Modelling continuous abstinence rates over time from clinical trials of pharmacological interventions for smoking cessation. Addiction. 2019;114(5):787–97.
1221. Jackson S, Brown J, West R, Norris E, Livingstone-Banks J, Lindson N. Mindfulness for smoking cessation. Cochrane Database of Systematic Reviews [Internet]. 2020;2020(7). Available from: https://www.cochranelibrary.com/cdsr/table-of-contents
1222. Jackson MA, Baker AL, McCarter KL, Brown AL, Gould GS, Dunlop AJ. Interventions for pregnant women who use tobacco and other substances: a systematic review protocol. BMJ open. 2019;9(11):e032449.
1223. Huang J. MS15.04 Approaching Cessation in the Patient Using Electronic Cigarettes. Journal of Thoracic Oncology. 2019;14 (10 Supplement):S186.
1224. Hillson R. Smoking and diabetes. Practical Diabetes. 2019;36(2):43–4.
1225. Hansen D, Piepoli MF, Doehner W. The importance of rehabilitation in the secondary prevention of cardiovascular disease. European journal of preventive cardiology. 2019;26(3):273–6.
1226. Gotts JE, Jordt SE, McConnell R, Tarran R. What are the respiratory effects of e-cigarettes? BMJ. 2019;366:l5275.
1227. Garcia-Pazo P, Fornes-Vives J, Sese A, Perez-Pareja FJ. Apps for smoking cessation through Cognitive Behavioural Therapy. A review. Adicciones. 2020;0(0):1431.
1228. Garcia-Gomez L, Hernandez-Perez A, Noe-Diaz V, Riesco-Miranda JA, Jimenez-Ruiz C. Smoking Cessation Treatments: Current Psychological and Pharmacological Options. Revista de Investigación Clínica. 2019;71(1):7–16.
1229. Evans W, Truscott R, Cameron E, Timmings C, Haque M, Halligan M, et al. ES20.03 Tobacco Control Integration in Cancer Care: The Canadian Experience. Journal of Thoracic Oncology. 2019;14 (10 Supplement):S62.
1230. Diamanti A, Papadakis S, Schoretsaniti S, Rovina N, Vivilaki V, Gratziou C, et al. Smoking cessation in pregnancy: An update for maternity care practitioners. Tobacco Induced Diseases [Internet]. 2019;17(August). Available from: http://www.tobaccoinduceddiseases.org/Smoking-cessation-in-pregnancy-An-update-for-maternity-ncare-practitioners,109906,0,2.html
1231. Canadian Partnership Against Cancer. Implementing Smoking Cessation in Cancer Care Across Canada: A Framework for Action. Toronto, Canada: Canadian Partnership Against Cancer; 2019.
1232. Barnett A, Yang I, Hay K, Ding H, Bowman R, Fong K, et al. A meta-analysis of the effectiveness of smart phone applications to aid smoking cessation. European Respiratory Journal Conference: 29th International Congress of the European Respiratory Society, ERS Spain [Internet]. 2019;54(Supplement 63). Available from: https://erj.ersjournals.com/content/54/suppl_63/OA5134
1233. Arnett DK, Khera A, Blumenthal RS. 2019 ACC/AHA Guideline on the Primary Prevention of Cardiovascular Disease: Part 1, Lifestyle and Behavioral Factors. JAMA cardiology. 2019;4(10):1043–4.
1234. Anonymous. Erratum: Electronic nicotine delivery systems and/or electronic non-nicotine delivery systems for tobacco smoking cessation or reduction: A systematic review and meta-analysis (BMJ Open (2017) 7 (e012680) DOI: 10.1136/bmjopen-2016-012680). BMJ open [Internet]. 2020;10(1). Available from: http://bmjopen.bmj.com/content/early/by/section
1235. Anonymous. Correction: electronic nicotine delivery systems and/or electronic non-nicotine delivery systems for tobacco smoking cessation or reduction: a systematic review and meta-analysis. BMJ open. 2020;10(1):e012680corr1.
1236. Anonymous. Benefits of smoking cessation medicines decline over first year. Clinical Pharmacist [Internet]. 2018;10(4). Available from: https://www.pharmaceutical-journal.com/news-and-analysis/research-briefing/ibuprofen-use-in-early-pregnancy-might-impact-female-offsprings-fertility/20204466.article
1237. Altman D, Clement FM, Barnieh L, Manns B, Penz E. Cost-effectiveness of universally funding smoking cessation pharmacotherapy. Canadian Journal of Respiratory, Critical Care, and Sleep Medicine. 2019;3(2):67–75.
1238. Alshahrani A. The Effects of Smoking Cessation on Diabetes Mellitus Patients. Current Diabetes Reviews. 2020;16(2):137–42.
1239. Adinoff B, Griffiths R, Hendricks P, Garcia-Romeu A, Bogenschutz M, Ferguson SS, et al. A new era of treating substance use disorders with psychedelics. American Journal on Addictions. 2019;28 (3):162–4.

## Does not examine a relevant intervention (n=164)

1. RefID: 1734. Ahmad N, Boutron I, Dechartres A, Durieux P, Ravaud P. Applicability and generalisability of the results of systematic reviews to public health practice and policy: a systematic review. Trials 2010;11:20.
2. RefID: 5003. Ali I, Patthi B, Singla A, Malhi R, Niraj L, Dhama K. Role of E-Cigarettes in smoking cessation: A systematic review. Journal of Indian Association of Public Health Dentistry 2018 Apr 1;16(2):94-102.
3. RefID: 3348. Amin F, Al HA, Civelek B, Fedorowicz Z, Manzer BM. Enhanced external counterpulsation for chronic angina pectoris. Cochrane Database Syst Rev 2008;(3):CD007219.
4. RefID: 5206. Angus K. A review of the effectiveness of smokefree strategies and interventions in secondary care settings. National Institute of Health and Care Excellence; 2013.
5. RefID: 1001. Atusingwize E, Lewis S, Langley T. Economic evaluations of tobacco control mass media campaigns: a systematic review. Tob Control 2015;24(4):320-7.
6. RefID: 3250. Auer R, Ghali W, Rodondi N, De Bosset-Sulzer V, Cornuz J. Is a the rosclerosis screening by noninvasive imaging effective in cardiovascular prevention? A systematic review. J Gen Intern Med 2010;25(SUPPL. 3):S317-S318.
7. RefID: 106. Aune D, Schlesinger S, Norat T, Riboli E. Tobacco smoking and the risk of abdominal aortic aneurysm: a systematic review and meta-analysis of prospective studies. Sci rep 2018;8(1):14786.
8. RefID: 48. Ayerbe L, Forgnone I, Foguet-Boreu Q, Gonzalez E, Addo J, Ayis S. Disparities in the management of cardiovascular risk factors in patients with psychiatric disorders: a systematic review and meta-analysis. Psychol Med 2018;1-9.
9. RefID: 1514. Bader P, Boisclair D, Ferrence R. Effects of tobacco taxation and pricing on smoking behavior in high risk populations: a knowledge synthesis. Int J Environ Res Public Health 2011;8(11):4118-39.
10. RefID: 2021. Bala M, Strzeszynski L, Cahill K. Mass media interventions for smoking cessation in adults. Cochrane Database Syst Rev 2008;(1):CD004704.
11. RefID: 657. Bartsch AL, Harter M, Niedrich J, Brutt AL, Buchholz A. A Systematic Literature Review of Self-Reported Smoking Cessation Counseling by Primary Care Physicians. PloS one 2016;11(12):e0168482.
12. RefID: 3444. Beard E, Aveyard P, Michie S, McNeill A, West R. Does use of nicotine replacement therapy while continuing to smoke undermine cessation?: A systematic review. J Smok Cessat 2013;8(1):45-56.
13. RefID: 216. Bello AK, Qarni B, Samimi A, Okel J, Chatterley T, Okpechi IG, et al. Effectiveness of Multifaceted Care Approach on Adverse Clinical Outcomes in Nondiabetic CKD: A Systematic Review and Meta-analysis. KI Rep 2017;2(4):617-25.
14. RefID: 1854. Bize R, Burnand B, Mueller Y, Rege Walther M, Cornuz J. Biomedical risk assessment as an aid for smoking cessation. Cochrane Database Syst Rev 2009;(2):CD004705.
15. RefID: 407. Bolshinsky V, Li MHG, Ismail H, Burbury K, Riedel B, Heriot A. Multimodal Prehabilitation Programs as a Bundle of Care in Gastrointestinal Cancer Surgery: A Systematic Review. Dis Colon Rectum 2018;61(1):124-38.
16. RefID: 2878. Bolton CE, Bevan-Smith EF, Blakey JD, Crowe P, Elkin SL, Garrod R, et al. British Thoracic Society guideline on pulmonary rehabilitation in adults. Thorax 2013;68(SUPPL. 2).
17. RefID: 893. Borrelli B, Tooley EM, Scott-Sheldon LAJ. Motivational Interviewing for Parent-child Health Interventions: A Systematic Review and Meta-Analysis. Pediatr Dent 2015;37(3):254-65.
18. RefID: 1108. Brown T, Platt S, Amos A. Equity impact of population-level interventions and policies to reduce smoking in adults: a systematic review. Drug Alcohol Depend 2014;138:7-16.
19. RefID: 643. Buoli M, Grassi S, Ciappolino V, Serati M, Altamura AC. The Use of Zonisamide for the Treatment of Psychiatric Disorders: A Systematic Review. Clin Neuropharmacol 2017;40(2):85-92.
20. RefID: 1962. Cahill K, Perera R. Competitions and incentives for smoking cessation. Cochrane Database Syst Rev 2008;(3):CD004307.
21. RefID: 1926. Cahill K, Perera R. Quit and Win contests for smoking cessation. Cochrane Database Syst Rev 2008;(4):CD004986.
22. RefID: 1596. Cahill K, Ussher MH. Cannabinoid type 1 receptor antagonists for smoking cessation. Cochrane Database Syst Rev 2011;(3):CD005353.
23. RefID: 904. Cahill K, Hartmann-Boyce J, Perera R. Incentives for smoking cessation. Cochrane Database Syst Rev 2015;(5):CD004307.
24. RefID: 2292. Carson KV, Ameer F, Sayehmiri K, Hnin K, van Agteren JE, Sayehmiri F, et al. Mass media interventions for preventing smoking in young people. Cochrane Database Syst Rev 2017;2017(6):CD001006.
25. RefID: 3645. Centre for Reviews and Dissemination. Glutathione S-transferase polymorphism interactions with smoking status and HPV infection in cervical cancer risk: an evidence-based meta-analysis (Provisional abstract). Database of Abstracts of Reviews of Effects 2015;(2).
26. RefID: 2247. Chandra K, Blackhouse G, McCurdy BR, Bornstein M, Campbell K, Costa V, et al. Cost-effectiveness of interventions for chronic obstructive pulmonary disease (COPD) using an ontario policy model. Ont Health Technol Assess Ser 2012;12(12):1-61.
27. RefID: 2675. Chang SJ, Choi S, Kim S-A, Song M. Intervention strategies based on information-motivation-behavioral skills model for health behavior change: A systematic review. Asian nurs res 2014;8(3):172-81.
28. RefID: 734. Choi HD, Shin WG. Meta-analysis of the association between a serotonin transporter 5-HTTLPR polymorphism and smoking cessation. Psychiatr Genet 2016;26(2):87-91.
29. RefID: 3422. Christie J. Legislative smoking bans for reducing secondhand smoke exposure, smoking prevalence and tobacco consumption. 2014;195-9.
30. RefID: 5305. Clifford A, Pulver LJ, Richmond R, Shakeshaft A, Ivers R. Smoking, nutrition, alcohol and physical activity interventions targeting Indigenous Australians: rigorous evaluations and new directions needed. Aust N Z J Public Health 2011 Feb;35(1):38-46.
31. RefID: 3134. Cole JA, Smith SM, Hart N, Cupples ME. Systematic review of the effect of diet and exercise lifestyle interventions in the secondary prevention of coronary heart disease. Cardiol Res Pract 2011;1(1):232351.
32. RefID: 285. Conlon K, Pattinson L, Hutton D. Attitudes of oncology healthcare practitioners towards smoking cessation: A systematic review of the facilitators, barriers and recommendations for delivery of advice and support to cancer patients. RADIOGRAPHY 2017;23(3):256-63.
33. RefID: 1025. Coppo A, Galanti MR, Giordano L, Buscemi D, Bremberg S, Faggiano F. School policies for preventing smoking among young people. Cochrane Database Syst Rev 2014;(10):CD009990.
34. RefID: 174. Corepal R, Tully MA, Kee F, Miller SJ, Hunter RF. Behavioural incentive interventions for health behaviour change in young people (5-18years old): A systematic review and meta-analysis. Prev Med 2018;110:55-66.
35. RefID: 3214. Crawford-Faucher A. Home- and center-based cardiac rehabilitation equally effective. Am Fam Physician 2010;82(8):994-5.
36. RefID: 388. Daly B, Tian CJL, Scragg RKR. Effect of nurse-led randomised control trials on cardiovascular risk factors and HbA1c in diabetes patients: A meta-analysis. Diabetes Res Clin Pract 2017;131:187-99.
37. RefID: 1251. David SP, Lancaster T, Stead LF, Evins AE, Prochaska JJ. Opioid antagonists for smoking cessation. Cochrane Database Syst Rev 2013;(6):CD003086.
38. RefID: 1116. David SP, Chu IM, Lancaster T, Stead LF, Evins AE, Prochaska JJ. Systematic review and meta-analysis of opioid antagonists for smoking cessation. BMJ open 2014;4(3):e004393.
39. RefID: 865. de Kleijn MJJ, Farmer MM, Booth M, Motala A, Smith A, Sherman S, et al. Systematic review of school-based interventions to prevent smoking for girls. Syst rev 2015;4:109.
40. RefID: 1382. de Viron S, Van der Heyden J, Ambrosino E, Arbyn M, Brand A, Van Oyen H. Impact of genetic notification on smoking cessation: systematic review and pooled-analysis. PloS one 2012;7(7):e40230.
41. RefID: 1608. Dhippayom T, Chaiyakunapruk N, Jongchansittho T. Safety of nortriptyline at equivalent therapeutic doses for smoking cessation: a systematic review and meta-analysis. Drug Saf 2011;34(3):199-210.
42. RefID: 2135. Drovandi A, Teague P-A, Glass B, Malau-Aduli B. A systematic review of smoker and non-smoker perceptions of visually unappealing cigarette sticks. Tob induc dis 2018;16(January).
43. Eadie D. A review of the barriers and facilitators to implementing smokefree strategies and interventions in secondary care settings. National Institute of Health Care Excellence; 2012.
44. RefID: 1155. Edwards SA, Bondy SJ, Callaghan RC, Mann RE. Prevalence of unassisted quit attempts in population-based studies: a systematic review of the literature. Addict Behav 2014;39(3):512-9.
45. RefID: 759. Ekong G, Kavookjian J. Motivational interviewing and outcomes in adults with type 2 diabetes: A systematic review. Patient Educ Couns 2016;99(6):944-52.
46. RefID: 247. Ekpu VU, Brown AK. The Economic Impact of Smoking and of Reducing Smoking Prevalence: Review of Evidence. Tob use insights 2015;8:1-35.
47. RefID: 1097. Evans SE, Hoffman AC. Electronic cigarettes: abuse liability, topography and subjective effects. Tob Control 2014;23 Suppl 2:ii23-ii29.
48. RefID: 853. Evans WD, Horn KA, Gray T. Systematic Review to Inform Dual Tobacco Use Prevention. Pediatr Clin North Am 2015;62(5):1159-72.
49. RefID: 150. Faber T, Kumar A, Mackenbach JP, Millett C, Basu S, Sheikh A, et al. Effect of tobacco control policies on perinatal and child health: a systematic review and meta-analysis. Lancet Public Health 2017;2(9):e420-e437.
50. RefID: 2419. Feirman SP, Lock D, Cohen JE, Holtgrave DR, Li T. Flavored Tobacco Products in the United States: A Systematic Review Assessing Use and Attitudes. Nicotine Tob Res 2016;18(5):739-49.
51. RefID: 713. Flemming K, Graham H, McCaughan D, Angus K, Sinclair L, Bauld L. Health professionals' perceptions of the barriers and facilitators to providing smoking cessation advice to women in pregnancy and during the post-partum period: a systematic review of qualitative research. BMC public health 2016;16:290.
52. RefID: 1760. Flu HC, Tamsma JT, Lindeman JH, Hamming JF, Lardenoye JH. A systematic review of implementation of established recommended secondary prevention measures in patients with PAOD. Eur J Vasc Endovasc Surg 2010 Jan;39(1):70-86.
53. RefID: 2246. Franek J. Home telehealth for patients with chronic obstructive pulmonary disease (COPD): An evidence-based analysis. Ont Health Technol Assess Ser 2012;12(11):1-58.
54. RefID: 2236. Frazer K, Mchugh J, Callinan JE, Kelleher C. Impact of institutional smoking bans on reducing harms and secondhand smoke exposure. Cochrane Database Syst Rev 2015;2015(9):CD011856.
55. RefID: 2178. Galling B, Correll C. Efficacy and safety of antidepressant augmentation of antipsychotics in schizophrenia. Schizophr Bull 2018;44(Supplement 1):S206-S207.
56. RefID: 1178. Gao X, Lo ECM, Kot SCC, Chan KCW. Motivational interviewing in improving oral health: a systematic review of randomized controlled trials. J Periodontol 2014;85(3):426-37.
57. RefID: 1402. Gellert C, Schottker B, Brenner H. Smoking and all-cause mortality in older people: systematic review and meta-analysis. Arch Intern Med 2012;172(11):837-44.
58. RefID: 1118. Giles EL, Robalino S, McColl E, Sniehotta FF, Adams J. The effectiveness of financial incentives for health behaviour change: systematic review and meta-analysis. PloS one 2014;9(3):e90347.
59. RefID: 1316. Gould GS, Munn J, Watters T, McEwen A, Clough AR. Knowledge and views about maternal tobacco smoking and barriers for cessation in Aboriginal and Torres Strait Islanders: A systematic review and meta-ethnography. Nicotine Tob Res 2013;15(5):863-74.
60. RefID: 1325. Gould GS, McEwen A, Watters T, Clough AR, van der Zwan R. Should anti-tobacco media messages be culturally targeted for Indigenous populations? A systematic review and narrative synthesis. Tob Control 2013;22(4):e7.
61. RefID: 419. Grabski M, Curran HV, Nutt DJ, Husbands SM, Freeman TP, Fluharty M, et al. Behavioural tasks sensitive to acute abstinence and predictive of smoking cessation success: a systematic review and meta-analysis. Addiction 2016;111(12):2134-44.
62. RefID: 1161. Graham H, Flemming K, Fox D, Heirs M, Sowden A. Cutting down: insights from qualitative studies of smoking in pregnancy. Health Soc Care Community 2014;22(3):259-67.
63. RefID: 2490. Guillaumier A, Bonevski B, Twyman L, Paul C, Baker A. Enforcement strategies for effective smoke-free policy implementation: A systematic review. Asia-Pac J Clin Oncol 2016;12(Supplement 6):10-1.
64. RefID: 1423. Guillaumier A, Bonevski B, Paul C. Anti-tobacco mass media and socially disadvantaged groups: a systematic and methodological review. Drug Alcohol Rev 2012;31(5):698-708.
65. RefID: 1598. Hackam DG, Shojania KG, Spence JD, Alter DA, Beanlands RS, Dresser GK, et al. Influence of noninvasive cardiovascular imaging in primary prevention: systematic review and meta-analysis of randomized trials. Arch Intern Med 2011;171(11):977-82.
66. RefID: 315. Hall K, Kisely S, Urrego F. The Use of Pediatrician Interventions to Increase Smoking Cessation Counseling Among Smoking Caregivers: A Systematic Review. Clin Pediatr (Phila) 2016;55(7):583-92.
67. RefID: 1338. Hamilton FL, Greaves F, Majeed A, Millett C. Effectiveness of providing financial incentives to healthcare professionals for smoking cessation activities: systematic review. Tob Control 2013;22(1):3-8.
68. RefID: 1375. Hartmann-Boyce J, Cahill K, Hatsukami D, Cornuz J. Nicotine vaccines for smoking cessation. Cochrane Database Syst Rev 2012;(8):CD007072.
69. RefID: 3888. Health TA. Barriers and facilitators to smoking cessation in pregnancy and following childbirth (Project record). Health Technology Assessment Database 2016;(2016 Issue 4).
70. RefID: 2304. Hefler M, Liberato SC, Thomas DP. Incentives for preventing smoking in children and adolescents. Cochrane Database Syst Rev 2017;2017(6):CD008645.
71. RefID: 381. Hempel S, Graham GD, Fu N, Estrada E, Chen AY, Miake-Lye I, et al. A systematic review of the effects of modifiable risk factor interventions on the progression of multiple sclerosis. Mult Scler 2017;23(4):513-24.
72. RefID: 647. Heron N, Kee F, Cardwell C, Tully MA, Donnelly M, Cupples ME. Secondary prevention lifestyle interventions initiated within 90 days after TIA or 'minor' stroke: a systematic review and meta-analysis of rehabilitation programmes. Br J Gen Pract 2017;67(654):e57-e66.
73. RefID: 1648. Hettema JE, Hendricks PS. Motivational interviewing for smoking cessation: a meta-analytic review. J Consult Clin Psychol 2010;78(6):868-84.
74. RefID: 901. Ho SY, Alnashri N, Rohde D, Murphy P, Doyle F. Systematic review and meta-analysis of the impact of depression on subsequent smoking cessation in patients with chronic respiratory conditions. Gen Hosp Psychiatry 2015;37(5):399-407.
75. RefID: 123. Hofmann P, Benden C, Kohler M, Schuurmans MM. Smoking resumption after heart or lung transplantation: a systematic review and suggestions for screening and management. J thorac dis 2018;10(7):4609-18.
76. RefID: 621. Hollands GJ, French DP, Griffin SJ, Prevost AT, Sutton S, King S, et al. The impact of communicating genetic risks of disease on risk-reducing health behaviour: systematic review with meta-analysis. BMJ 2016;352:i1102.
77. RefID: 1740. Hopkins DP, Razi S, Leeks KD, Priya Kalra G, Chattopadhyay SK, Soler RE, et al. Smokefree policies to reduce tobacco use. A systematic review. Am J Prev Med 2010;38(2 Suppl):S275-S289.
78. RefID: 3588. Hughes JR, Stead LF, Lancaster T. Anxiolytics for smoking cessation. (8).
79. RefID: 987. Hughes JR, Dash M, Callas PW. Is impulsivity a symptom of initial tobacco withdrawal? A meta-analysis and qualitative systematic review. Nicotine Tob Res 2015;17(5):503-9.
80. RefID: 719. Hughes N, Arora M, Grills N. Perceptions and impact of plain packaging of tobacco products in low and middle income countries, middle to upper income countries and low-income settings in high-income countries: a systematic review of the literature. BMJ open 2016 Mar 21;6(3):e010391.
81. RefID: 809. Imamura M, Williams K, Wells M, McGrother C. Lifestyle interventions for the treatment of urinary incontinence in adults. Cochrane Database Syst Rev 2015;(12):CD003505.
82. RefID: 371. Jawad M, Jawad S, Waziry RK, Ballout RA, Akl EA. Interventions for waterpipe tobacco smoking prevention and cessation: a systematic review. Sci rep 2016;6:25872.
83. RefID: 366. Jiang S, Wu L, Gao X. Beyond face-to-face individual counseling: A systematic review on alternative modes of motivational interviewing in substance abuse treatment and prevention. Addict Behav 2017 Oct;73:216-35.
84. RefID: 318. Keith DR, Kurti AN, Davis DR, Zvorsky IA, Higgins ST. A review of the effects of very low nicotine content cigarettes on behavioral and cognitive performance. Prev Med 2017;104:100-16.
85. RefID: 255. Khan Z, Tonnies J, Muller S. Smokeless tobacco and oral cancer in South Asia: a systematic review with meta-analysis. J Cancer Epidemiol 2014;2014:394696.
86. RefID: 935. Krauth D, Apollonio D. Accuracy of popular media reporting on tobacco cessation therapy in substance abuse and mental health populations. BMJ open 2015;5(3):e007169.
87. RefID: 1363. Lancaster T, Stead LF. Silver acetate for smoking cessation. Cochrane Database Syst Rev 2012;(9):CD000191.
88. RefID: 1443. Lawrence M, Kerr S, McVey C, Godwin J. The effectiveness of secondary prevention lifestyle interventions designed to change lifestyle behavior following stroke: summary of a systematic review. Int j stroke 2012;7(3):243-7.
89. RefID: 1741. Leeks KD, Hopkins DP, Soler RE, Aten A, Chattopadhyay SK, Task Force on Community Preventive Services. Worksite-based incentives and competitions to reduce tobacco use. A systematic review. Am J Prev Med 2010;38(2 Suppl):S263-S274.
90. RefID: 25. Leite FRM, Nascimento GG, Baake S, Pedersen LD, Scheutz F, Lopez R. Impact of smoking cessation on periodontitis: A systematic review and meta-analysis of prospective longitudinal observational and interventional studies. Nicotine Tob Res 2018.
91. RefID: 627. Lenferink A, Brusse-Keizer M, van der Valk PD, Frith PA, Zwerink M, Monninkhof EM, et al. Self-management interventions including action plans for exacerbations versus usual care in patients with chronic obstructive pulmonary disease. Cochrane Database Syst Rev 2017;8:CD011682.
92. Leonardi-Bee J. Barriers and Facilitators for Smoking cessation interventions in Mental Health. UK Centre for Tobacco Control Studies; 2012.
93. RefID: 125. Lilic N, Stretton M, Prakash M. How effective is the plain packaging of tobacco policy on rates of intention to quit smoking and changing attitudes to smoking? ANZ J Surg 2018;88(9):825-30.
94. RefID: 948. Lindson-Hawley N, Thompson TP, Begh R. Motivational interviewing for smoking cessation. Cochrane Database Syst Rev 2015;(3):CD006936.
95. RefID: 3593. LopezArrieta J, Sanz JF. Nicotine for Alzheimer's disease. (7).
96. RefID: 300. Malone V, Harrison R, Daker-White G. Mental health service user and staff perspectives on tobacco addiction and smoking cessation: A meta-synthesis of published qualitative studies. J Psychiatr Ment Health Nurs 2018;25(4):270-82.
97. RefID: 3446. Mantler T. A systematic review of smoking Youths' perceptions of addiction and health risks associated with smoking: Utilizing the framework of the health belief model. Addict Res Theory 2013;21(4):306-17.
98. RefID: 1342. Marcano Belisario JS, Bruggeling MN, Gunn LH, Brusamento S, Car J. Interventions for recruiting smokers into cessation programmes. Cochrane Database Syst Rev 2012;12:CD009187.
99. RefID: 1662. Marteau TM, French DP, Griffin SJ, Prevost AT, Sutton S, Watkinson C, et al. Effects of communicating DNA-based disease risk estimates on risk-reducing behaviours. Cochrane Database Syst Rev 2010;(10):CD007275.
100. RefID: 2245. McCurdy BR. hospital-at-home programs for patients with acute exacerbations of chronic obstructive pulmonary disease (COPD): An evidence-based analysis. Ont Health Technol Assess Ser 2012;12(10):1-65.
101. RefID: 3595. McGrath J, McDonald WJ, MacDonald JK. Transdermal nicotine for induction of remission in ulcerative colitis. (4).
102. RefID: 1555. McIvor RA, Tunks M, Todd DC. Copd. Clin Evid (Online) 2011;2011.
103. RefID: 930. McLean S, Barbour V, Wild S, Simpson C, Sheikh A. Models for estimating projections for disease prevalence and burden: a systematic review focusing on chronic obstructive pulmonary disease. J Health Serv Res Policy 2015;20(4):246-53.
104. RefID: 2235. McNeill A, Bauld L, Birken M, Hammond D, Moodie C, Stead M, et al. Tobacco packaging design for tobacco use cessation and reduction. Cochrane Database Syst Rev 2014;2014(8):CD011244.
105. RefID: 508. McNeill A, Gravely S, Hitchman SC, Bauld L, Hammond D, Hartmann-Boyce J. Tobacco packaging design for reducing tobacco use. Cochrane Database Syst Rev 2017;4:CD011244.
106. RefID: 373. Mola A, Lloyd MM, Villegas-Pantoja MA. A Mixed Method Review of Tobacco Cessation for the Cardiopulmonary Rehabilitation Clinician. J Mol Signal 2017;37(3):160-74.
107. RefID: 793. Monarrez-Espino J, Liu B, Greiner F, Bremberg S, Galanti R. [Systematic review of the effect of pictorial warnings on cigarette packages in smoking behavior]. Rev Panam Salud Publica 2015;38(6):515-32.
108. RefID: 3370. Monson E, Arsenault N. Effects of enactment of legislative (public) smoking bans on voluntary home smoking restrictions: A review. Nicotine & Tobacco Research 2017;19(2):141-8.
109. RefID: 3880. Morgan H, Hoddinott P, Thomson G, Crossland N, Farrar S, Yi D, et al. Benefits of Incentives for Breastfeeding and Smoking cessation in pregnancy (BIBS): a mixed-methods study to inform trial design (Structured abstract). Health Technology Assessment Database 2016;(2016 Issue 4).
110. RefID: 919. Morgan H, Hoddinott P, Thomson G, Crossland N, Farrar S, Yi D, et al. Benefits of Incentives for Breastfeeding and Smoking cessation in pregnancy (BIBS): a mixed-methods study to inform trial design. Health Technol Assess 2015;19(30):1-viii.
111. RefID: 641. Mosdol A, Lidal IB, Straumann GH, Vist GE. Targeted mass media interventions promoting healthy behaviours to reduce risk of non-communicable diseases in adult, ethnic minorities. Cochrane Database Syst Rev 2017;2:CD011683.
112. RefID: 116. Moyo F, Archibald E, Slyer JT. Effectiveness of decision aids for smoking cessation in adults: a quantitative systematic review. JBI Database System Rev Implement Rep 2018;16(9):1791-822.
113. Myers K. Smoking cessation interventions in acute and maternity services: Review of Barriers and Facilitators. National Institute for Health and Clinical Excellence; 2012.
114. RefID: 1599. Myers K, Hajek P, Hinds C, McRobbie H. Stopping smoking shortly before surgery and postoperative complications: a systematic review and meta-analysis. Arch Intern Med 2011;171(11):983-9.
115. RefID: 5015. National Institute for Health and Care Excellence. Smoking: preventing uptake in children and young people [Internet]. Available at: https://www.nice.org.uk/guidance/ph14. Last Accessed: 3-3-2020. [PMID: ]
116. RefID: 2244. No author listed. Long-term oxygen therapy for patients with chronic obstructive pulmonary disease (COPD): An evidence-based analysis. Ont Health Technol Assess Ser 2012;12(7):1-64.
117. RefID: 848. Notley C, Blyth A, Craig J, Edwards A, Holland R. Postpartum smoking relapse--a thematic synthesis of qualitative studies. Addiction 2015;110(11):1712-23.
118. RefID: 1487. Oosterom-Calo R, van Ballegooijen AJ, Terwee CB, te Velde SJ, Brouwer IA, Jaarsma T, et al. Determinants of heart failure self-care: a systematic literature review. Heart Fail Rev 2012;17(3):367-85.
119. RefID: 172. Orton S, Coleman T, Coleman-Haynes T, Ussher M. Predictors of Postpartum Return to Smoking: A Systematic Review. Nicotine Tob Res 2018;20(6):665-73.
120. RefID: 316. Oyewole BK, Animasahun VJ, Chapman HJ. Tobacco use in Nigerian youth: A systematic review. PloS one 2018;13(5):e0196362.
121. RefID: 1743. Parsons A, Daley A, Begh R, Aveyard P. Influence of smoking cessation after diagnosis of early stage lung cancer on prognosis: systematic review of observational studies with meta-analysis. BMJ 2010;340:b5569.
122. RefID: 3428. Pineles BL. Smoking in pregnancy: From effects to solutions. Dissertation Abstracts International: Section B: The Sciences and Engineering 2014;75(2-B(E)):No-Specified.
123. RefID: 2633. Rahman M, Edward K, Montgomery L, McEvedy S, Wilson A, Worrall-Carter L. A systematic review investigating smoking relapse in women hospitalised due to coronary heart disease. Heart Lung Circul 2015;24(SUPPL. 3):S448.
124. RefID: 182. Regmi K, Kaphle D, Timilsina S, Tuha NAA. Application of Discrete-Choice Experiment Methods in Tobacco Control: A Systematic Review. Pharmacoeconom Open 2018;2(1):5-17.
125. RefID: 2629. Reynolds JA, Peng Y, Vaidya N, Dumitru CG, Hopkins DP. Comprehensive tobacco control programs reduce tobacco use and secondhand smoke exposure: A systematic review. J Thorac Oncol 2015;10(9 SUPPL. 2):S302.
126. RefID: 175. Riaz M, Lewis S, Naughton F, Ussher M. Predictors of smoking cessation during pregnancy: a systematic review and meta-analysis. Addiction 2018;113(4):610-22.
127. RefID: 993. Robertson L, McGee R, Marsh L, Hoek J. A systematic review on the impact of point-of-sale tobacco promotion on smoking. Nicotine Tob Res 2015;17(1):2-17.
128. RefID: 1496. Rodondi N, Auer R, de Bosset Sulzer V, Ghali WA, Cornuz J. Atherosclerosis screening by noninvasive imaging for cardiovascular prevention: a systematic review. J Gen Intern Med 2012;27(2):220-31.
129. RefID: 169. Schadewaldt V, Schultz T. A systematic review on the effectiveness of nurse-led cardiac clinics for adult patients with coronary heart disease. JBI Libr Syst Rev 2010;8(2):53-89.
130. RefID: 1536. Schadewaldt V, Schultz T. Nurse-led clinics as an effective service for cardiac patients: results from a systematic review. Int j evid -based healthc 2011;9(3):199-214.
131. RefID: 1318. Schmidt-Hansen M, Page R, Hasler E. The effect of preoperative smoking cessation or preoperative pulmonary rehabilitation on outcomes after lung cancer surgery: a systematic review. CLIN LUNG CANCER 2013;14(2):96-102.
132. RefID: 1532. Scott A, Sivey P, Ait Ouakrim D, Willenberg L, Naccarella L, Furler J, et al. The effect of financial incentives on the quality of health care provided by primary care physicians. Cochrane Database Syst Rev 2011;(9):CD008451.
133. RefID: 2243. Sikich N. Community-based multidisciplinary care for patients with stable chronic obstructive pulmonary disease (COPD): An evidence-based analysis. Ont Health Technol Assess Ser 2012;12(5):1-51.
134. RefID: 20. Simonavicius E, McNeill A, Shahab L, Brose LS. Heat-not-burn tobacco products: a systematic literature review. Tob Control 2018.
135. RefID: 202. Small S, Porr C, Swab M, Murray C. Experiences and cessation needs of Indigenous women who smoke during pregnancy: a systematic review of qualitative evidence. JBI Database System Rev Implement Rep 2018;16(2):385-452.
136. RefID: 1495. Smerecnik C, Grispen JEJ, Quaak M. Effectiveness of testing for genetic susceptibility to smoking-related diseases on smoking cessation outcomes: a systematic review and meta-analysis. Tob Control 2012;21(3):347-54.
137. RefID: 770. Snaterse M, Dobber J, Jepma P, Peters RJG, Ter Riet G, Boekholdt SM, et al. Effective components of nurse-coordinated care to prevent recurrent coronary events: a systematic review and meta-analysis. Heart 2016;102(1):50-6.
138. RefID: 1454. Stead LF, Hughes JR. Lobeline for smoking cessation. Cochrane Database Syst Rev 2012;(2):CD000124.
139. RefID: 1210. Stead M, Moodie C, Angus K, Bauld L, McNeill A, Thomas J, et al. Is consumer response to plain/standardised tobacco packaging consistent with framework convention on tobacco control guidelines? A systematic review of quantitative studies. PloS one 2013;8(10):e75919.
140. RefID: 1129. Taylor G, McNeill A, Girling A, Farley A, Lindson-Hawley N, Aveyard P. Change in mental health after smoking cessation: systematic review and meta-analysis. BMJ 2014;348:g1151.
141. RefID: 5100. Taylor RS, Dalal H, Jolly K, Zawada A, Dean SG, Cowie A, et al. Home-based versus centre-based cardiac rehabilitation. Cochrane Database Syst Rev 2015 Aug 18;(8):CD007130.
142. RefID: 903. Tee GH, Hairi NN, Nordin F, Choo WY, Chan YY, Kaur G, et al. Systematic review on international practices in controlling waterpipe tobacco smoking. Asian Pac J Cancer Prev 2015;16(9):3659-65.
143. RefID: 3162. Thornton M, Mackay-Lyons M, Ruggles T. Systematic review of non-pharmacological interventions for transient ischemic attack (TIA) and non-disabling stroke (NDS) as secondary stroke prevention, preliminary results. Physiotherapy 2011;97(SUPPL. 1):eS1232-eS1233.
144. RefID: 605. Tian J, Venn A, Otahal P, Gall S. The association between quitting smoking and weight gain: a systematic review and meta-analysis of prospective cohort studies. Obes Rev 2015;16(10):883-901.
145. RefID: 3154. Ton TGN, Fogg TT, Fong C-T, John C, Li SXL, Marshall JA, et al. Knowledge, perception, and behaviors of relatives of people with premature heart disease: A systematic literature review. Circulation 2011;124(8):958-64.
146. RefID: 311. Usher-Smith JA, Silarova B, Sharp SJ, Mills K, Griffin SJ. Effect of interventions incorporating personalised cancer risk information on intentions and behaviour: a systematic review and meta-analysis of randomised controlled trials. BMJ open 2018;8(1):e017717.
147. RefID: 410. van den Brand FA, Nagelhout GE, Reda AA, Winkens B, Evers SMAA, Kotz D, et al. Healthcare financing systems for increasing the use of tobacco dependence treatment. Cochrane Database Syst Rev 2017;9:CD004305.
148. RefID: 401. van Osch FH, Jochems SH, van Schooten FJ, Bryan RT, Zeegers MP. Quantified relations between exposure to tobacco smoking and bladder cancer risk: a meta-analysis of 89 observational studies. Int J Epidemiol 2016;45(3):857-70.
149. RefID: 89. Veisani Y, Jenabi E, Delpisheh A, Khazaei S. Effect of prenatal smoking cessation interventions on birth weight: meta-analysis. J Matern Fetal Neonatal Med 2019;32(2):332-8.
150. RefID: 240. Verze P, Margreiter M, Esposito K, Montorsi P, Mulhall J. The Link Between Cigarette Smoking and Erectile Dysfunction: A Systematic Review. Eur Urol Focus 2015;1(1):39-46.
151. RefID: 633. Virtue SM, Waldron EM, Darabos K, DeAngelis C, Moore DA, Fornatora M, et al. Dental Students' Attitudes Toward Tobacco Cessation in the Dental Setting: A Systematic Review. J Dent Educ 2017;81(5):500-16.
152. RefID: 155. Wang QL, Xie SH, Li WT, Lagergren J. Smoking Cessation and Risk of Esophageal Cancer by Histological Type: Systematic Review and Meta-analysis. J Natl Cancer Inst 2017;109(12).
153. RefID: 609. Webb MJ, Kauer SD, Ozer EM, Haller DM, Sanci LA. Does screening for and intervening with multiple health compromising behaviours and mental health disorders amongst young people attending primary care improve health outcomes? A systematic review. BMC Fam Pract 2016;17:104.
154. RefID: 1395. Wilson LM, Avila Tang E, Chander G, Hutton HE, Odelola OA, Elf JL, et al. Impact of tobacco control interventions on smoking initiation, cessation, and prevalence: a systematic review. J Environ Public Health 2012;2012:961724.
155. RefID: 1482. Wong J, Lam DP, Abrishami A, Chan MTV, Chung F. Short-term preoperative smoking cessation and postoperative complications: a systematic review and meta-analysis. Can J Anaesth 2012;59(3):268-79.
156. RefID: 86. Wynne O, Guillaumier A, Twyman L, McCrabb S, Denham AMJ, Paul C, et al. Signs, Fines and Compliance Officers: A Systematic Review of Strategies for Enforcing Smoke-Free Policy. Int J Environ Res Public Health 2018;15(7).
157. RefID: 1095. Yang L, Rudy SF, Cheng JM, Durmowicz EL. Electronic cigarettes: incorporating human factors engineering into risk assessments. Tob Control 2014;23 Suppl 2:ii47-ii53.
158. RefID: 2461. Yi L-J, Tian X, Shuai T, Zeng Z, Ma L, Wang Y, et al. Case management does not decrease mortality of patients with myocardial infarction or unstable angina: Evidence from a systematic review. Int J Nurs Sci 2016;3(2):190-7.
159. RefID: 3443. Young CF, Skorga P. Interventions for tobacco use prevention in Indigenous youth. Public Health Nursing 2013;30(5):448-50.
160. RefID: 2430. Yu CH, Guarna G, Tsao P, Jesuthasan JR, Lau ANC, Siddiqi FS, et al. Incentivizing health care behaviors in emerging adults: A systematic review. Patient Prefer Adherence 2016;10:371-81.
161. RefID: 1974. Zhang J, Wang Z. Factors associated with smoking in Asian American adults: a systematic review. Nicotine Tob Res 2008;10(5):791-801.
162. RefID: 648. Zuo JJ, Tao ZZ, Chen C, Hu ZW, Xu YX, Zheng AY, et al. Characteristics of cigarette smoking without alcohol consumption and laryngeal cancer: overall and time-risk relation. A meta-analysis of observational studies. Eur Arch Otorhinolaryngol 2017;274(3):1617-31.
163. RefID: 2227. Zwerink M, Brusse-Keizer M, van der Valk PDLP, Zielhuis GA, Monninkhof EM, van der Palen J, et al. Self management for patients with chronic obstructive pulmonary disease. Cochrane Database Syst Rev 2014;2014(3):CD002990.
164. Campbell K, Coleman-Haynes T, Bowker K, Cooper SE, Connelly S, Coleman T. Factors influencing the uptake and use of nicotine replacement therapy and e-cigarettes in pregnant women who smoke: a qualitative evidence synthesis. Cochrane Database of Systematic Reviews. 2020;5:CD013629.

## Does not report relevant outcomes (n=59)

1. RefID: 882. Barber CEH, Marshall DA, Alvarez N, Mancini GBJ, Lacaille D, Keeling S, et al. Development of Cardiovascular Quality Indicators for Rheumatoid Arthritis: Results from an International Expert Panel Using a Novel Online Process. J Rheumatol 2015;42(9):1548-55.
2. RefID: 1708. Baxter S, Everson-Hock E, Messina J, Guillaume L, Burrows J, Goyder E. Factors relating to the uptake of interventions for smoking cessation among pregnant women: a systematic review and qualitative synthesis. Nicotine Tob Res 2010;12(7):685-94.
3. RefID: 374. Berg ML, Cheung KL, Hiligsmann M, Evers S, de Kinderen RJA, Kulchaitanaroaj P, et al. Model-based economic evaluations in smoking cessation and their transferability to new contexts: a systematic review. Addiction 2017;112(6):946-67.
4. RefID: 1046. Bryant J, Passey ME, Hall AE, Sanson-Fisher RW. A systematic review of the quality of reporting in published smoking cessation trials for pregnant women: an explanation for the evidence-practice gap? Implement Sci 2014;9:94
5. RefID: 112. Cann KF, Heneghan KD, Knight T. The impact of restricting the use of e-cigarettes in public places: a systematic review. J Public Health (Oxf) 2018;40(3):533-9.
6. RefID: 1281. Carroll C, Booth A, Leaviss J, Rick J. "Best fit" framework synthesis: refining the method. BMC Med Res Methodol 2013;13:37
7. RefID: 1194. Carroll C, Rick J, Leaviss J, Fishwick D, Booth A. A qualitative evidence synthesis of employees' views of workplace smoking reduction or cessation interventions. BMC public health 2013;13:1095
8. RefID: 3078. Carson KV, Brinn MP, Labiszewski NA, Esterman AJ, Chang AB, Smith BJ. Community interventions for preventing smoking in young people. Cochrane Database Syst Rev 2011;2017(12):CD001291. [PMID: http://as.wiley.com/WileyCDA/Brand/id-6.html]
9. RefID: 1586. Cassar K. Peripheral arterial disease. Clin Evid (Online) 2011;2011:
10. RefID: 1265. Chambrone L, Preshaw PM, Rosa EF, Heasman PA, Romito GA, Pannuti CM, et al. Effects of smoking cessation on the outcomes of non-surgical periodontal therapy: a systematic review and individual patient data meta-analysis. J Clin Periodontol 2013;40(6):607-15.
11. RefID: 922. Choi HD, Shin WG. Lack of association between DRD2 Taq1A gene polymorphism and smoking cessation therapy: a meta-analysis. Int J Clin Pharmacol Ther 2015;53(6):415-21.
12. RefID: 2463. Cristea IA, Kok RN, Cuijpers P. The effectiveness of cognitive bias modification interventions for substance addictions: A meta-analysis. PloS one 2016;11(9):e0162226. [PMID: http://journals.plos.org/plosone/article/asset?id=10.1371/journal.pone.0162226.PDF]
13. RefID: 1109. De Leon E, Fuentes LW, Cohen JE. Characterizing periodic messaging interventions across health behaviors and media: systematic review. J Med Internet Res 2014;16(3):e93
14. RefID: 871. Donker T, Blankers M, Hedman E, Ljotsson B, Petrie K, Christensen H. Economic evaluations of Internet interventions for mental health: a systematic review. Psychol Med 2015;45(16):3357-76.
15. RefID: 1064. Duaso MJ, McDermott MS, Mujika A, Purssell E, While A. Do doctors' smoking habits influence their smoking cessation practices? A systematic review and meta-analysis. Addiction 2014;109(11):1811-23.
16. RefID: 405. Duaso MJ, Bakhshi S, Mujika A, Purssell E, While AE. Nurses' smoking habits and their professional smoking cessation practices. A systematic review and meta-analysis. Int J Nurs Stud 2017;67:3-11.
17. RefID: 1181. Fiorini T, Musskopf ML, Oppermann RV, Susin C. Is there a positive effect of smoking cessation on periodontal health? A systematic review. J Periodontol 2014;85(1):83-91.
18. RefID: 821. Fischer C, Steyerberg EW, Fonarow GC, Ganiats TG, Lingsma HF. A systematic review and meta-analysis on the association between quality of hospital care and readmission rates in patients with heart failure. Am Heart J 2015;170(5):1005-17.
19. RefID: 5101. Free C, Phillips G, Watson L, Galli L, Felix L, Edwards P, et al. The effectiveness of mobile-health technologies to improve health care service delivery processes: a systematic review and meta-analysis. PLoS Med 2013;10(1):e1001363. [PMID: PM:23458994]
20. RefID: 1976. Freund M, Campbell E, Paul C, McElduff P, Walsh RA, Sakrouge R, et al. Smoking care provision in hospitals: a review of prevalence. Nicotine Tob Res 2008;10(5):757-74.
21. RefID: 1848. Freund M, Campbell E, Paul C, Sakrouge R, McElduff P, Walsh RA, et al. Increasing smoking cessation care provision in hospitals: a meta-analysis of intervention effect. Nicotine Tob Res 2009;11(6):650-62.
22. RefID: 487. Gentry S, Craig J, Holland R, Notley C. Smoking cessation for substance misusers: A systematic review of qualitative studies on participant and provider beliefs and perceptions. Drug Alcohol Depend 2017;180:178-92.
23. RefID: 658. Greenhalgh T, Macfarlane F, Steed L, Walton R. What works for whom in pharmacist-led smoking cessation support: realist review. BMC Med 2016;14(1):209
24. RefID: 1328. Haasova M, Warren FC, Ussher M, Janse Van Rensburg K, Faulkner G, Cropley M, et al. The acute effects of physical activity on cigarette cravings: systematic review and meta-analysis with individual participant data. Addiction 2013;108(1):26-37.
25. RefID: 2567. Harrison SL, Janaudis-Ferreira T, Brooks D, Desveaux L, Goldstein RS. Self-management following an acute exacerbation of COPD: A systematic review. Chest 2015;147(3):646-61. [PMID: http://journal.publications.chestnet.org/data/Journals/CHEST/933270/chest_147_3_646.pdf]
26. RefID: 323. Hartwell G, Thomas S, Egan M, Gilmore A, Petticrew M. E-cigarettes and equity: a systematic review of differences in awareness and use between sociodemographic groups. Tob Control 2017;26(e2):e85-e91
27. RefID: 445. Haskins BL, Lesperance D, Gibbons P, Boudreaux ED. A systematic review of smartphone applications for smoking cessation. Transl Behav Med 2017;7(2):292-9.
28. RefID: 10. Hickson C, Lewis S, Campbell KA, Cooper S, Berlin I, Claire R, et al. Comparison of nicotine exposure during pregnancy when smoking and abstinent with nicotine replacement therapy: systematic review and meta-analysis. Addiction 2018;
29. RefID: 815. Jones M, Lewis S, Parrott S, Coleman T. Systematic critical review of previous economic evaluations of smoking cessation during pregnancy. BMJ open 2015;5(11):e008998
30. RefID: 140. Lee JGL, Orlan EN, Sewell KB, Ribisl KM. A new form of nicotine retailers: a systematic review of the sales and marketing practices of vape shops. Tob Control 2018;27(e1):e70-e75
31. RefID: 1683. Li R, Zhang P, Barker LE, Chowdhury FM, Zhang X. Cost-effectiveness of interventions to prevent and control diabetes mellitus: a systematic review. Diabetes care 2010;33(8):1872-94.
32. RefID: 136. Luther A, Gabriel J, Watson RP, Francis NK. The Impact of Total Body Prehabilitation on Post-Operative Outcomes After Major Abdominal Surgery: A Systematic Review. World J Surg 2018;42(9):2781-91.
33. RefID: 1613. Mills E, Eyawo O, Lockhart I, Kelly S, Wu P, Ebbert JO. Smoking cessation reduces postoperative complications: a systematic review and meta-analysis. Am J Med 2011;124(2):144-54.
34. RefID: 2330. Min W, An R, Li S, Feng J, Yang J, Huang Z. The effects of preoperative smoking cessation on the healing of fractures and postoperative complications: A systematic review and meta-analysis. Biomed Res 2017;28(4):1883-9.
35. RefID: 979. Mitchell AJ, Vancampfort D, De Hert M, Stubbs B. Do people with mental illness receive adequate smoking cessation advice? A systematic review and meta-analysis. Gen Hosp Psychiatry 2015;37(1):14-23.
36. RefID: 377. Moug SJ, Bryce A, Mutrie N, Anderson AS. Lifestyle interventions are feasible in patients with colorectal cancer with potential short-term health benefits: a systematic review. Int J Colorectal Dis 2017;32(6):765-75.
37. RefID: 281. Muller-Riemenschneider F, Damm K, Meinhard C, Bockelbrink A, Vauth C, Willich SN, et al. Evaluation of medical and health economic effectiveness of non-pharmacological secondary prevention of coronary heart disease. GMS Health Technol Assess 2009;5:Doc16
38. RefID: 1722. Netto G, Bhopal R, Lederle N, Khatoon J, Jackson A. How can health promotion interventions be adapted for minority ethnic communities? Five principles for guiding the development of behavioural interventions. Health Promot Internation 2010;25(2):248-57.
39. RefID: 947. Orr JA, King RJ. Mobile phone SMS messages can enhance healthy behaviour: a meta-analysis of randomised controlled trials. Health psychol rev 2015;9(4):397-416.
40. RefID: 3859. Paech D, Mernagh P, Weston A. A systematic review of economic evaluations for tobacco control programs (Structured abstract). Health Technology Assessment Database 2016;(2016 Issue 4):
41. RefID: 2402. Peirson L, Ali MU, Kenny M, Raina P, Sherifali D. Interventions for prevention and treatment of tobacco smoking in school-aged children and adolescents: A systematic review and meta-analysis. Prev Med 2016;85:20-31.
42. RefID: 262. Pennington M, Visram S, Donaldson C, White M, Lhussier M, Deane K, et al. Cost-effectiveness of health-related lifestyle advice delivered by peer or lay advisors: synthesis of evidence from a systematic review. Cost Eff Resour Alloc 2013;11(1):30
43. RefID: 866. Pereira CF, de Vargas D. Profile of women who carried out smoking cessation treatment: a systematic review. Rev Saude Publica 2015;49:40
44. RefID: 771. Riley HEM, Berry-Bibee E, England LJ, Jamieson DJ, Marchbanks PA, Curtis KM. Hormonal contraception among electronic cigarette users and cardiovascular risk: a systematic review. Contraception 2016;93(3):190-208.
45. RefID: 3489. Roberts SH, Bailey JE. Incentives and barriers to lifestyle interventions for people with severe mental illness: A narrative synthesis of quantitative, qualitative and mixed methods studies. J Adv Nurs 2011;67(4):690-708.
46. RefID: 1278. Rongen A, Robroek SJW, van Lenthe FJ, Burdorf A. Workplace health promotion: a meta-analysis of effectiveness. Am J Prev Med 2013;44(4):406-15.
47. RefID: 2044. Ruger JP, Emmons KM. Economic evaluations of smoking cessation and relapse prevention programs for pregnant women: a systematic review. Value Health 2008;11(2):180-90.
48. RefID: 1477. Ruger JP, Lazar CM. Economic evaluation of pharmaco- and behavioral therapies for smoking cessation: a critical and systematic review of empirical research. Annu Rev Public Health 2012;33:279-305.
49. RefID: 1538. Skinner JS, Cooper A. Secondary prevention of ischaemic cardiac events. Clin Evid (Online) 2011;2011:
50. RefID: 421. Soneji S, Barrington-Trimis JL, Wills TA, Leventhal AM, Unger JB, Gibson LA, et al. Association Between Initial Use of e-Cigarettes and Subsequent Cigarette Smoking Among Adolescents and Young Adults: A Systematic Review and Meta-analysis. Jama, Pediatr 2017;171(8):788-97.
51. RefID: 1220. Stoilkova A, Janssen DJA, Wouters EFM. Educational programmes in COPD management interventions: a systematic review. Respir Med 2013;107(11):1637-50.
52. RefID: 2774. Sutton L, Karan A, Mahal A. Evidence for cost-effectiveness of lifestyle primary preventions for cardiovascular disease in the Asia-Pacific Region: A systematic review. Globalization Health 2014;10(1):79. [PMID: http://www.globalizationandhealth.com/]
53. RefID: 428. Trainor K, Leavey G. Barriers and Facilitators to Smoking Cessation Among People With Severe Mental Illness: A Critical Appraisal of Qualitative Studies. Nicotine Tob Res 2017;19(1):14-23.
54. RefID: 2108. Vilhelmsson A, Ostergren P-O. Reducing health inequalities with interventions targeting behavioral factors among individuals with low levels of education - A rapid review. PloS one 2018;13(4):e0195774.
55. RefID: 2118. Wang J-W, Cao S-S, Hu R-Y. Smoking by family members and friends and electronic-cigarette use in adolescence: A systematic review and meta-analysis. Tob induc dis 2018;16(February):5.
56. RefID: 750. Wang M, Wang JW, Cao SS, Wang HQ, Hu RY. Cigarette Smoking and Electronic Cigarettes Use: A Meta-Analysis. Int J Environ Res Public Health 2016;13(1):
57. RefID: 1184. Wilby KJ, Harder CK. Nicotine replacement therapy in the intensive care unit: a systematic review. J Intensive Care Med 2014;29(1):22-30.
58. RefID: 665. Xu Y, Guo Y, Liu K, Liu Z, Wang X. E-Cigarette Awareness, Use, and Harm Perception among Adults: A Meta-Analysis of Observational Studies. PloS one 2016;11(11):e0165938
59. Ebbert JO, Elrashidi MY, Stead LF. Interventions for smokeless tobacco use cessation. Cochrane Database of Systematic Reviews [Internet]. 2019;(10). Available from: https://ovidsp.ovid.com/ovidweb.cgi?T=JS&CSC=Y&NEWS=N&PAGE=fulltext&D=coch&AN=00075320-100000000-03294

## Exclusively in children/adolescents (n=3)

1. RefID: 2307. Kew KM, Carr R, Crossingham I. Lay-led and peer support interventions for adolescents with asthma. Cochrane Database Syst Rev 2017;2017(4):CD012331.
2. RefID: 3841. Mueller-Riemenschneider F, Rasch A, Bockelbrink A, Vauth C, Willich SN, Greiner W. Effectiveness and cost-effectiveness of behavioural strategies in the prevention of cigarette smoking (Structured abstract). Health Technology Assessment Database 2016;(2016 Issue 4):
3. RefID: 5001. Patnode CD, O'Connor E, Whitlock EP, Perdue LA, Soh C. Primary Care Relevant Interventions for Tobacco Use Prevention and Cessation in Children and Adolescents: A Systematic Evidence Review for the U.S. Preventive Services Task Force [Internet]. Agency for Healthcare Research and Quality 2012 Dec. [PMID: 23270006]

## Examines intervention(s) targeted to adults other than the adult smoker (e.g., partners, healthcare providers) (n=9)

1. RefID: 1515. Boyle R, Solberg L, Fiore M. Use of electronic health records to support smoking cessation. Cochrane Database Syst Rev 2011;(12):CD008743
2. RefID: 1650. Boyle RG, Solberg LI, Fiore MC. Electronic medical records to increase the clinical treatment of tobacco dependence: a systematic review. Am J Prev Med 2010;39(6 Suppl 1):S77-S82
3. RefID: 3860. Brinson D, Ali W. The effectiveness of interventions to increase the delivery of effective smoking cessation treatments in primary care settings - the ABCs (Structured abstract). Health Technology Assessment Database 2016;(2016 Issue 4):
4. RefID: 1418. Carson KV, Verbiest MEA, Crone MR, Brinn MP, Esterman AJ, Assendelft WJJ, et al. Training health professionals in smoking cessation. Cochrane Database Syst Rev 2012;(5):CD000214
5. RefID: 2013. Dexheimer JW, Talbot TR, Sanders DL, Rosenbloom ST, Aronsky D. Prompting clinicians about preventive care measures: a systematic review of randomized controlled trials. J Am Med Inform Assoc 2008;15(3):311-20.
6. RefID: 331. Dherani M, Zehra SN, Jackson C, Satyanaryana V, Huque R, Chandra P, et al. Behaviour change interventions to reduce second-hand smoke exposure at home in pregnant women - a systematic review and intervention appraisal. BMC Pregnancy Childbirth 2017;17(1):378
7. RefID: 1961. Farmer AP, Legare F, Turcot L, Grimshaw J, Harvey E, McGowan JL, et al. Printed educational materials: effects on professional practice and health care outcomes. Cochrane Database Syst Rev 2008;(3):CD004398
8. RefID: 79. Skelton E, Tzelepis F, Shakeshaft A, Guillaumier A, McCrabb S, Bonevski B. Integrating smoking cessation care in alcohol and other drug treatment settings using an organizational change intervention: a systematic review. Addiction 2018;113(12):2158-72.
9. Papadakis S, Pipe A, Kelly S, Pritchard G, Wells GA. Strategies to improve the delivery of tobacco use treatment in primary care practice. Cochrane Database of Systematic Reviews [Internet]. 2020;(2). Available from: https://ovidsp.ovid.com/ovidweb.cgi?T=JS&CSC=Y&NEWS=N&PAGE=fulltext&D=coch&AN=00075320-100000000-09993

## Exclusively in settings not relevant to primary care (n=28)

1. RefID: 3285. Aziz O, Skapinakis P, Rahman S, Rao C, Ashrafian H, Panesar SS, et al. Behavioural interventions for smoking cessation in patients hospitalised for a major cardiovascular event. Int J Cardiol 2009;137(2):171-4.
2. RefID: 188. Badawy SM, Kuhns LM. Texting and Mobile Phone App Interventions for Improving Adherence to Preventive Behavior in Adolescents: A Systematic Review. JMIR Mhealth Uhealth 2017;5(4):e50
3. RefID: 800. Berlin NL, Cutter C, Battaglia C. Will preoperative smoking cessation programs generate long-term cessation? A systematic review and meta-analysis. Am J Manag Care 2015;21(11):e623-e631
4. RefID: 122. Brose LS, Simonavicius E, McNeill A. Maintaining abstinence from smoking after a period of enforced abstinence - systematic review, meta-analysis and analysis of behaviour change techniques with a focus on mental health. Psychol Med 2018;48(4):669-78.
5. RefID: 1927. Cahill K, Moher M, Lancaster T. Workplace interventions for smoking cessation. Cochrane Database Syst Rev 2008;(4):CD003440
6. RefID: 2010. Cropley M, Theadom A, Pravettoni G, Webb G. The effectiveness of smoking cessation interventions prior to surgery: a systematic review. Nicotine Tob Res 2008;10(3):407-12.
7. RefID: 470. de Andrade D, Kinner SA. Systematic review of health and behavioural outcomes of smoking cessation interventions in prisons. Tob Control 2016;26(5):495-501.
8. RefID: 1151. Doyle F, Rohde D, Rutkowska A, Morgan K, Cousins G, McGee H. Systematic review and meta-analysis of the impact of depression on subsequent smoking cessation in patients with coronary heart disease: 1990 to 2013. Psychosom Med 2014;76(1):44-57.
9. RefID: 3900. Feltner C, Peterson K, Palmieri Weber R, Cluff L, Coker-Schwimmer E, Viswanathan M, et al. Total Worker HealthReg. (Structured abstract). Health Technology Assessment Database 2016;(2016 Issue 4):
10. RefID: 620. Feltner C, Peterson K, Palmieri Weber R, Cluff L, Coker-Schwimmer E, Viswanathan M, et al. The Effectiveness of Total Worker Health Interventions: A Systematic Review for a National Institutes of Health Pathways to Prevention Workshop. Ann Intern Med 2016;165(4):262-9.
11. RefID: 1195. Grandi SM, Shimony A, Eisenberg MJ. Bupropion for smoking cessation in patients hospitalized with cardiovascular disease: a systematic review and meta-analysis of randomized controlled trials. Can J Cardiol 2013;29(12):1704-11.
12. RefID: 912. Gulliver A, Farrer L, Chan JKY, Tait RJ, Bennett K, Calear AL, et al. Technology-based interventions for tobacco and other drug use in university and college students: a systematic review and meta-analysis. Addict Sci Clin Pract 2015;10:5
13. RefID: 1746. Huttunen-Lenz M, Song F, Poland F. Are psychoeducational smoking cessation interventions for coronary heart disease patients effective? Meta-analysis of interventions. Br J Health Psychol 2010;15(Pt 4):749-77.
14. RefID: 1089. Kotb A, Hsieh S, Wells GA. The effect of telephone support interventions on coronary artery disease (CAD) patient outcomes during cardiac rehabilitation: a systematic review and meta-analysis. PloS one 2014;9(5):e96581
15. RefID: 314. Mohan ARM, Thomson P, Leslie SJ, Dimova E, Haw S, McKay JA. A Systematic Review of Interventions to Improve Health Factors or Behaviors of the Cardiovascular Health of Prisoners During Incarceration. J Cardiovasc Nurs 2018;33(1):72-81.
16. RefID: 3844. National Institute for Health and Clinical Excellence. Workplace health promotion: how to help employees to stop smoking (Structured abstract). Health Technology Assessment Database 2016;(2016 Issue 4):
17. RefID: 409. Ng KT, Gillies M, Griffith DM. Effect of nicotine replacement therapy on mortality, delirium, and duration of therapy in critically ill smokers: a systematic review and meta-analysis. Anaesth Intensive Care 2017;45(5):556-61.
18. RefID: 32. Parappilly BP, Field TS, Mortenson WB, Sakakibara BM, Eng JJ. Effectiveness of interventions involving nurses in secondary stroke prevention: A systematic review and meta-analysis. EUR J CARDIOVASC NURS 2018;1474515118779732
19. RefID: 1288. Pires-Yfantouda R, Absalom G, Clemens F. Smoking cessation interventions for COPD: a review of the literature. Respir Care 2013;58(11):1955-62.
20. RefID: 221. Prestwich A, Moore S, Kotze A, Budworth L, Lawton R, Kellar I. How Can Smoking Cessation Be Induced Before Surgery? A Systematic Review and Meta-Analysis of Behavior Change Techniques and Other Intervention Characteristics. Front Psychol 2017;8:915
21. RefID: 1925. Rigotti NA, Munafo MR, Stead LF. Smoking cessation interventions for hospitalized smokers: a systematic review. Arch Intern Med 2008;168(18):1950-60.
22. RefID: 1429. Sorensen LT. Wound healing and infection in surgery. The clinical impact of smoking and smoking cessation: a systematic review and meta-analysis. Arch Surg 2012;147(4):373-83.
23. RefID: 1087. Stockings EA, Bowman JA, Prochaska JJ, Baker AL, Clancy R, Knight J, et al. The impact of a smoke-free psychiatric hospitalization on patient smoking outcomes: a systematic review. Aust N Z J Psychiatry 2014;48(7):617-33.
24. RefID: 2795. Thomas RE, Mclellan J, Perera R. School-based programmes for preventing smoking. Cochrane Database Syst Rev 2013;2017(12):CD001293. [PMID: http://as.wiley.com/WileyCDA/Brand/id-6.html]
25. RefID: 5300. Thomsen T, Villebro N, Moller AM. Interventions for preoperative smoking cessation. Cochrane Database Syst Rev 2014 Mar 27;(3):CD002294. [PMID: PM:24671929]
26. RefID: 383. Turgeon RD, Chang SJ, Dandurand C, Gooderham PA, Hunt C. Nicotine replacement therapy in patients with aneurysmal subarachnoid hemorrhage: Systematic review of the literature, and survey of Canadian practice. J Clin Neurosci 2017;42:48-53.
27. RefID: 1186. Wierenga D, Engbers LH, Van Empelen P, Duijts S, Hildebrandt VH, Van Mechelen W. What is actually measured in process evaluations for worksite health promotion programs: a systematic review. BMC public health 2013;13:1190
28. RefID: 2035. Zaki A, Abrishami A, Wong J, Chung FF. Interventions in the preoperative clinic for long term smoking cessation: a quantitative systematic review. Can J Anaesth 2008;55(1):11-21.

## >50% of included studies conducted in countries “high”, “medium”, or “low” on the Human Development Index (n=9)

1. RefID: 101. Alzahrane A, West R, Ubhi HK, Brown J, Abdulqader N, Samarkandi O. Evaluations of clinical tobacco cessation interventions in Arab populations: A systematic review. Addict Behav 2019;88:169-74.
2. RefID: 1472. Kim SS, Chen W, Kolodziej M, Wang X, Wang VJ, Ziedonis D. A systematic review of smoking cessation intervention studies in China. Nicotine Tob Res 2012;14(8):891-9.
3. RefID: 873. Maziak W, Jawad M, Jawad S, Ward KD, Eissenberg T, Asfar T. Interventions for waterpipe smoking cessation. Cochrane Database Syst Rev 2015;(7):CD005549
4. RefID: 517. McKay AJ, Patel RKK, Majeed A. Strategies for tobacco control in India: a systematic review. PloS one 2015;10(4):e0122610
5. RefID: 305. Puttarak P, Pornpanyanukul P, Meetam T, Bunditanukul K, Chaiyakunapruk N. Efficacy and safety of Vernonia cinerea (L.) Less. for smoking cessation: A systematic review and meta-analysis of randomized controlled trials. Complement Ther Med 2018;37:37-42.
6. RefID: 1506. Schroer-Gunther MA, Zhou M, Gerber A, Passon AM. Primary tobacco prevention in China--a systematic review. Asian Pac J Cancer Prev 2011;12(11):2973-80.
7. RefID: 2141. Uthman OA, Hartley L, Rees K, Taylor F, Ebrahim S, Clarke A. Multiple Risk Factor Interventions for Primary Prevention of CVD in LMIC: A Cochrane Review. Glo Heart 2017;12(3):199. [PMID: http://www.elsevier.com/wps/find/journaldescription.cws_home/726194/description#description]
8. RefID: 864. Uthman OA, Hartley L, Rees K, Taylor F, Ebrahim S, Clarke A. Multiple risk factor interventions for primary prevention of cardiovascular disease in low- and middle-income countries. Cochrane Database Syst Rev 2015;(8):CD011163
9. RefID: 151. Whitehouse E, Lai J, Golub JE, Farley JE. A systematic review of the effectiveness of smoking cessation interventions among patients with tuberculosis. Public health action 2018;8(2):37-49.

## Inadequate timepoints of follow-up (n=6)

1. RefID: 108. Cooper V, Clatworthy J, Whetham J, Consortium E. mHealth Interventions To Support Self-Management In HIV: A Systematic Review. Open AIDS J 2017;11:119-32.
2. RefID: 342. Covolo L, Ceretti E, Moneda M, Castaldi S, Gelatti U. Does evidence support the use of mobile phone apps as a driver for promoting healthy lifestyles from a public health perspective? A systematic review of Randomized Control Trials. Patient Educ Couns 2017;100(12):2231-43.
3. RefID: 2196. Gould GS, Bar-Zeev Y, Twyman T, Stevenson L, Palazzi K, Bonevski B. Health providers' performance of the 5as for smoking cessation care during pregnancy: A systematic review and meta-analysis. J Paediatr Child Health 2018;54(Supplement 1):78
4. RefID: 3396. King JL, Pomeranz JL, Merten JW. A systematic review and meta-evaluation of adolescent smoking cessation interventions that utilized nicotine replacement therapy. Addict Behav 2016;52:39-45.
5. RefID: 343. Oikonomou MT, Arvanitis M, Sokolove RL. Mindfulness training for smoking cessation: A meta-analysis of randomized-controlled trials. J HEALTH PSYCHOL 2017;22(14):1841-50.
6. RefID: 290. Yee A, Hoong MC, Joyce YC, Loh HS. Smoking Cessation Among Methadone-Maintained Patients: A Meta-Analysis. Subst Use Misuse 2018;53(2):276-85.

## Other reasons for exclusion - no or inadequate synthesis across studies for relevant outcomes (n=22)

1. RefID: 135. Al-Bashaireh AM, Haddad LG, Weaver M, Kelly DL, Chengguo X, Yoon S. The Effect of Tobacco Smoking on Musculoskeletal Health: A Systematic Review. J Environ Public Health 2018;2018:4184190
2. RefID: 1616. Baxter S, Blank L, Everson-Hock ES, Burrows J, Messina J, Guillaume L, et al. The effectiveness of interventions to establish smoke-free homes in pregnancy and in the neonatal period: a systematic review. Health Educ Res 2011;26(2):265-82.
3. RefID: 1082. Brown T, Platt S, Amos A. Equity impact of interventions and policies to reduce smoking in youth: systematic review. Tob Control 2014;23(e2):e98-105.
4. RefID: 616. Cass SJ, Ball LE, Leveritt MD. Passive interventions in primary healthcare waiting rooms are effective in promoting healthy lifestyle behaviours: an integrative review. Aust J Prim Health 2016;22(3):198-210.
5. RefID: 273. Coronini-Cronberg S, Heffernan C, Robinson M. Effective smoking cessation interventions for COPD patients: a review of the evidence. JRSM Short Rep 2011;2(10):78
6. RefID: 1610. Ebbert J, Montori VM, Erwin PJ, Stead LF. Interventions for smokeless tobacco use cessation. Cochrane Database Syst Rev 2011;(2):CD004306
7. RefID: 1330. Flett K, Clark-Carter D, Grogan S, Davey R. How effective are physical appearance interventions in changing smoking perceptions, attitudes and behaviours? A systematic review. Tob Control 2013;22(2):74-9.
8. RefID: 2066. Heitmann J, Bennik EC, Van Hemel-Ruiter ME, De Jong PJ. The effectiveness of attentional bias modification for substance use disorder symptoms in adults: A systematic review. Syst rev 2018;7(1):160. [PMID: http://www.systematicreviewsjournal.com/]
9. RefID: 3522. Hyde J, Hankins M, Deale A, Marteau TM. Interventions to increase self-efficacy in the context of addiction behaviours: A systematic literature review. J HEALTH PSYCHOL 2008;13(5):607-23.
10. RefID: 965. Kirsch F. A systematic review of quality and cost-effectiveness derived from Markov models evaluating smoking cessation interventions in patients with chronic obstructive pulmonary disease. Expert rev pharmacoecon outcomes res 2015;15(2):301-16.
11. RefID: 1541. Lehto T, Oinas-Kukkonen H. Persuasive features in web-based alcohol and smoking interventions: a systematic review of the literature. J Med Internet Res 2011;13(3):e46
12. RefID: 1350. Liu J, Davidson E, Bhopal R, White M, Johnson M, Netto G, et al. Adapting health promotion interventions to meet the needs of ethnic minority groups: mixed-methods evidence synthesis. Health Technol Assess 2012;16(44):1-469.
13. RefID: 1471. Mahmoudi M, Coleman CI, Sobieraj DM. Systematic review of the cost-effectiveness of varenicline vs. bupropion for smoking cessation. Int J Clin Pract 2012;66(2):171-82.
14. RefID: 1407. Mantler T, Irwin JD, Morrow D. Motivational interviewing and smoking behaviors: a critical appraisal and literature review of selected cessation initiatives. Psychol Rep 2012;110(2):445-60.
15. RefID: 1881. Murray RL, Bauld L, Hackshaw LE, McNeill A. Improving access to smoking cessation services for disadvantaged groups: a systematic review. J Public Health (Oxf) 2009;31(2):258-77.
16. RefID: 3495. No ai. Systematic review of how to stop smoking in pregnancy and following childbirth. J Adv Nurs 2010;66(12):2620-6.
17. RefID: 170. Overdijkink SB, Velu AV, Rosman AN, van Beukering MD, Kok M, Steegers-Theunissen RP. The Usability and Effectiveness of Mobile Health Technology-Based Lifestyle and Medical Intervention Apps Supporting Health Care During Pregnancy: Systematic Review. JMIR Mhealth Uhealth 2018;6(4):e109
18. RefID: 439. Peletidi A, Nabhani-Gebara S, Kayyali R. Smoking Cessation Support Services at Community Pharmacies in the UK: A Systematic Review. HJC Hell J Cardiol 2016;57(1):7-15.
19. RefID: 435. Pugh G, Gravestock HL, Hough RE, King WM, Wardle J, Fisher A. Health Behavior Change Interventions for Teenage and Young Adult Cancer Survivors: A Systematic Review. J Adolesc Young Adult Oncol 2016;5(2):91-105.
20. RefID: 565. Rathbone AL, Prescott J. The Use of Mobile Apps and SMS Messaging as Physical and Mental Health Interventions: Systematic Review. J Med Internet Res 2017;19(8):e295
21. RefID: 505. Schuit E, Panagiotou OA, Munafo MR, Bennett DA, Bergen AW, David SP. Pharmacotherapy for smoking cessation: effects by subgroup defined by genetically informed biomarkers. Cochrane Database Syst Rev 2017;9:CD011823
22. RefID: 790. Schwartz J, Fadahunsi O, Hingorani R, Mainali NR, Oluwasanjo A, Aryal MR, et al. Use of Varenicline in Smokeless Tobacco Cessation: A Systematic Review and Meta-Analysis. Nicotine Tob Res 2016;18(1):10-6.

# Additional exclusions

## Irrelevant intervention (e.g., relapse prevention, prenatal ultrasonography) (n=10)

1. RefID: 644. Thomas D, Abramson MJ, Bonevski B, George J. System change interventions for smoking cessation. Cochrane Database Syst Rev 2017;2:CD010742
2. RefID: 1885. Hajek P, Stead LF, West R, Jarvis M, Lancaster T. Relapse prevention interventions for smoking cessation. Cochrane Database Syst Rev 2009;(1):CD003999
3. RefID: 1744. Hollands GJ, Hankins M, Marteau TM. Visual feedback of individuals' medical imaging results for changing health behaviour. Cochrane Database Syst Rev 2010;(1):CD007434
4. RefID: 632. McCabe C, McCann M, Brady AM. Computer and mobile technology interventions for self-management in chronic obstructive pulmonary disease. Cochrane Database Syst Rev 2017;5:CD011425
5. RefID: 3347. Nabhan AF, Faris MA. High feedback versus low feedback of routine prenatal ultrasound for improving outcomes. Cochrane Database Syst Rev 2008;(3):CD007208. [PMID: <http://www.mrw.interscience.wiley.com/cochrane/clsysrev/articles/CD007208/pdf_fs.html>]
6. Notley C, Gentry S, Livingstone-Banks J, Bauld L, Perera R, Hartmann-Boyce J. Incentives for smoking cessation. Cochrane Database of Systematic Reviews. 2019;7:CD004307.
7. Livingstone-Banks J, Norris E, Hartmann-Boyce J, West R, Jarvis M, Chubb E, et al. Relapse prevention interventions for smoking cessation. Cochrane Database of Systematic Reviews. 2019;10(10):28.
8. Lindson N, Thompson TP, Ferrey A, Lambert JD, Aveyard P. Motivational interviewing for smoking cessation. Cochrane Database of Systematic Reviews [Internet]. 2019;(7). Available from: https://ovidsp.ovid.com/ovidweb.cgi?T=JS&CSC=Y&NEWS=N&PAGE=fulltext&D=coch&AN=00075320-100000000-05617
9. Fanshawe TR, Hartmann-Boyce J, Perera R, Lindson N. Competitions for smoking cessation. Cochrane Database of Systematic Reviews. 2019;2:CD013272.
10. Clair C, Mueller Y, Livingstone-Banks J, Burnand B, Camain JY, Cornuz J, et al. Biomedical risk assessment as an aid for smoking cessation. Cochrane Database of Systematic Reviews. 2019;3:CD004705.

## Reported analyses combine trials of various smoking cessation interventions such that the effect of each cannot be isolated (n=4)

1. RefID: 1400. Carr AB, Ebbert J. Interventions for tobacco cessation in the dental setting. Cochrane Database Syst Rev 2012;(6):CD005084
2. RefID: 2023. Rice VH, Stead LF. Nursing interventions for smoking cessation. Cochrane Database Syst Rev 2008;(1):CD001188
3. Lindson N, Klemperer E, Hong B, Ordonez-Mena JM, Aveyard P. Smoking reduction interventions for smoking cessation. Cochrane Database of Systematic Reviews. 2019;9:CD013183.
4. Carson-Chahhoud KV, Livingstone-Banks J, Sharrad KJ, Kopsaftis Z, Brinn MP, To ANR, et al. Community pharmacy personnel interventions for smoking cessation. Cochrane Database of Systematic Reviews. 2019;10(10):31.

## Inadequate syntheses (data cannot be collected at face-value) (n=1)

1. RefID: 2659. Baxi R, Sharma M, Roseby R, Polnay A, Priest N, Waters E, et al. Family and carer smoking control programmes for reducing children's exposure to environmental tobacco smoke. Cochrane Database Syst Rev 2014;2017(12):CD001746.

## Largely indirect (majority of studies in smokers <18 years; some trials include adults but only up to 20 years of age) (n=1)

1. RefID: 503. Fanshawe TR, Halliwell W, Lindson N, Aveyard P, Livingstone-Banks J, Hartmann-Boyce J. Tobacco cessation interventions for young people. Cochrane Database Syst Rev 2017;11:CD003289

## Candidate review on e-cigarettes selected for updating (n=2)

1. RefID: 177. Hartmann-Boyce J, McRobbie H, Bullen C, Begh R, Stead LF, Hajek P. Electronic cigarettes for smoking cessation. Cochrane Database Syst Rev 2016;9:CD010216
2. Hartmann-Boyce J, McRobbie H, Lindson N, Bullen C, Begh R, Theodoulou A, et al. Electronic cigarettes for smoking cessation. Cochrane Database of Systematic Reviews [Internet]. 2020; Available from: <http://doi.wiley.com/10.1002/14651858.CD010216.pub4>

## Cochrane overview superseded by updated versions of included reviews (n=2)

1. RefID: 1253. Cahill K, Stevens S, Perera R, Lancaster T. Pharmacological interventions for smoking cessation: an overview and network meta-analysis. Cochrane Database Syst Rev 2013;(5):CD009329
2. Lindson N, Aveyard P, Hughes JR. WITHDRAWN: Reduction versus abrupt cessation in smokers who want to quit. Cochrane Database of Systematic Reviews. 2019;10:CD008033.

## Cochrane review - Subpopulations not of interest and/or comparative effectiveness only (n=23)

1. RefID: 1468. Carson KV, Brinn MP, Peters M, Veale A, Esterman AJ, Smith BJ. Interventions for smoking cessation in Indigenous populations. Cochrane Database Syst Rev 2012;1:CD009046
[truncated: 69,872 more chars]
