# Supplementary material for: Effectiveness of smoking cessation interventions among adults: an overview of systematic reviews
Source: Syst Rev. 2024 Jul 12;13:179. doi: 10.1186/s13643-024-02570-9 (PMC11242003; doi:10.1186/s13643-024-02570-9)
Supplement: Supplementary file 8 — Additional file 8. Overview of reviews data extraction items. [file 13643_2024_2570_MOESM8_ESM.docx]

**Additional file 8. Overview of reviews: Data extraction items**

**Population**

- Population recruited in each trial included in the analysis *

**Intervention comparison**

- Intervention and comparator including specific characteristics (e.g., variation in control conditions across trials)
- Co-interventions provided *

**Outcome**

- Outcome for which data is reported
- Outcome measure (e.g., % studies reporting each of point prevalence, sustained abstinence, prolonged abstinence; tools for measuring QoL or change in emotional state outcomes, etc.) *
- Outcome ascertainment method (e.g., % studies biochemically validated) *

**Timepoint of follow-up**

**Setting restrictions (review-level)**

**Results**

- Number of studies and total number of participants in analysis
- Study designs
- Type of synthesis (e.g., meta-analysis, narrative, vote count)
- Number of events and denominator (intervention, comparator)
- Type of model (fixed or random effects)
- Pooled effect estimate, corresponding 95% CI, heterogeneity statistics (e.g., I2 and p-value)
- Narrative synthesis as reported by review authors
- Results of review authors’ GRADE assessment, where applicable
- Subgroup data (effect estimate and corresponding 95% CI + heterogeneity statistic for each subgroup, test for subgroup differences)

**Indirectness**

- Proportion of indirect evidence for each review exclusion criterion excluding specialized behavioural counselling *

**Risk of bias**

- For each trial included in the analysis, risk of bias rating for each domain (used authors’ ratings for individual domains at face value)

** Using data reported in review evidence tables, individual study information was extracted and collated*
