# Supplementary material for: Effectiveness of smoking cessation interventions among adults: an overview of systematic reviews
Source: Syst Rev. 2024 Jul 12;13:179. doi: 10.1186/s13643-024-02570-9 (PMC11242003; doi:10.1186/s13643-024-02570-9)

**Additional file 9. Forest plots for included analyses**

Table of Contents

[Barnes 2019 {3836} 5](#_Toc34435894)

[Hypnotherapy alone versus Placebo- Abstinence/cessation, 12 months 5](#_Toc34435895)

[Cahill 2010 {1652} 6](#_Toc34435896)

[Stage-based expert systems or tailored self-help materials versus Assessment only- Abstinence/cessation, 6+ months 6](#_Toc34435897)

[Stage-based interactive computer programmes versus Usual care- Abstinence/cessation, 12+ month 6](#_Toc34435898)

[Stage-based telephone counselling versus Usual Care- Abstinence/cessation, 12 months 7](#_Toc34435899)

[Stage-based individual counselling and/or advice versus Usual care- Abstinence/cessation, 6+ months 7](#_Toc34435900)

[Stage-based individual counselling or advice versus Assessment only- Abstinence/cessation, 6+ months 7](#_Toc34435901)

[Cahill 2016 {1960} 8](#_Toc34435902)

[Cystisine versus Placebo- Abstinence/cessation (continuous), 6+ months 8](#_Toc34435903)

[Cytisine versus Placebo- Abstinence/cessation (point prevalence), 2 years 8](#_Toc34435904)

[Varenicline versus Placebo- Abstinence/cessation, longest follow-up (6+ months) 9](#_Toc34435905)

[Varenicline versus Placebo- Abstinence/cessation, 6 months 10](#_Toc34435906)

[Long term varenicline versus Placebo- Abstinence/cessation, 6-12 months 10](#_Toc34435907)

[Low dose varenicline versus Placebo- Abstinence/cessation- 12 months 11](#_Toc34435908)

[Variable dosing of varenicline versus Placebo- Abstinence/cessation, 12 months 11](#_Toc34435909)

[Varenicline versus Placebo- Abstinence/cessation in smokers reducing to quit, 12 months 11](#_Toc34435910)

[Varenicline versus Placebo- Abstinence/cessation in smokers with schizophrenia, bipolar, or other psychiatric disorder, 6 months 12](#_Toc34435911)

[Varenicline versus Placebo- Abstinence/cessation in smokers with depression and motivated/wishing to quit, 12 months 12](#_Toc34435912)

[Varenicline versus Placebo- Abstinence/cessation in smokers who previously failed to quit on varenicline but are motivated/wishing to try quitting again, 12 months 12](#_Toc34435913)

[Varenicline versus Placebo- Adverse events (nausea), Range of follow-up times 13](#_Toc34435914)

[Varenicline versus Placebo- Adverse events (insomnia), Range of follow-up times 14](#_Toc34435915)

[Varenicline versus Placebo- Adverse events (abnormal dreams), Range of follow-up times 15](#_Toc34435916)

[Varenicline versus Placebo- Adverse events (headache), Range of follow-up times 16](#_Toc34435917)

[Varenicline versus Placebo- Adverse events (depression), Range of follow-up times 17](#_Toc34435918)

[Varenicline versus Placebo- Adverse events (suicidal ideation), Range of follow-up times 18](#_Toc34435919)

[Varenicline versus Placebo- Serious adverse event (at least one), Range of follow-up times 19](#_Toc34435920)

[Varenicline versus Placebo- Serious adverse event (at least one during or immediately after treatment), Range of follow-up times 20](#_Toc34435921)

[Varenicline versus Placebo- Neuropsychiatric events (depression, suicidal ideation), not deaths, Range of follow-up times 21](#_Toc34435922)

[Varenicline versus Placebo- Serious adverse events (cardiac, including deaths), Range of follow-up times 22](#_Toc34435923)

[Farley 2012 {1469} 23](#_Toc34435924)

[Buproprion versus Placebo- Weight gain, End of treatment 23](#_Toc34435925)

[Buproprion versus Placebo- Weight gain, 6 months 23](#_Toc34435926)

[Buproprion versus Placebo- Weight gain, 12 months 23](#_Toc34435927)

[NRT versus Placebo- Weight gain, End of treatment 24](#_Toc34435928)

[NRT versus Placebo- Weight gain, 6 months 25](#_Toc34435929)

[NRT versus Placebo- Weight gain, 12 months 26](#_Toc34435930)

[Varenicline 2mg/day versus Placebo- Weight gain, End of treatment 27](#_Toc34435931)

[Varenicline 2mg/day versus Placebo- Weight gain, 6 months 27](#_Toc34435932)

[Varenicline 2mg/day versus Placebo- Weight gain, 12 months 27](#_Toc34435933)

[Varenicline 1mg/day versus Placebo- Weight gain, End of treatment 27](#_Toc34435934)

[Hartmann-Boyce 2018 {332} 28](#_Toc34435935)

[NRT patch versus Placebo- Adverse events (Palpitations/chest pains), Range of follow-up times 28](#_Toc34435936)

[Hollands 2015 {916} 29](#_Toc34435937)

[Interventions to increase adherence for tobacco dependence versus Usual or standard care- Abstinence/cessation, 6 months 29](#_Toc34435938)

[Hughes 2014 {1147} 30](#_Toc34435939)

[Bupropion versus Placebo- Abstinence/cessation, 6 months 30](#_Toc34435940)

[Bupropion versus Placebo- Reduction in cotinine >50%, 12 months 30](#_Toc34435941)

[St John’s wort versus Placebo- Abstinence/cessation, 6 months 30](#_Toc34435942)

[S-Adenosyl-L-Methionine (SAMe) versus Placebo- Abstinence/cessation, 6 months 30](#_Toc34435943)

[Lancaster 2017 {539} 31](#_Toc34435944)

[Individual counselling versus Minimal contact control- Abstinence/cessation, 6+ months 31](#_Toc34435945)

[Lindson-Hawley 2016 {671} 32](#_Toc34435946)

[NRT versus Placebo- Abstinence/cessation, 12 months to 24 months 32](#_Toc34435947)

[NRT versus Placebo- Reduction in cigarettes/day of >50% of baseline or cessation, 12+ months 33](#_Toc34435948)

[Bupropion versus Placebo- Abstinence/cessation, 6 months 33](#_Toc34435949)

[Bupropion versus Placebo- Reduction in cigarettes/day of >50% of baseline or cessation, 12 months 34](#_Toc34435950)

[Varenicline versus Placebo- Abstinence/cessation, 6 months 34](#_Toc34435951)

[Telephone counselling plus self-help materials versus Usual care- Abstinence/cessation, 12 months & 34](#_Toc34435952)

[Telephone counselling plus self-help materials versus Usual care- Reduction in cigarettes/day of >50% of baseline or cessation, 12 months 34](#_Toc34435953)

[E-cigarettes versus Placebo- Abstinence/cessation, 12 months 35](#_Toc34435954)

[E-cigarettes versus Placebo- Reduction in cigarettes/day of >50% of baseline or cessation, 12months 35](#_Toc34435955)

[Livingstone-Banks 2019 {1077} 36](#_Toc34435956)

[Non-tailored print-based self-help materials (no face-to-face contact) versus No materials/no intervention- Abstinence/cessation, 6+ months 36](#_Toc34435957)

[Non-tailored print-based self-help (no face-to-face contact) versus No materials/no interventions- Abstinence/cessation, 6 months 36](#_Toc34435958)

[Non-tailored print-based self-help materials (no face-to-face contact) versus Brief leaflet- Abstinence/cessation, 6+ months 37](#_Toc34435959)

[Non-tailored print-based self-help materials (with face-to-face contact) versus No treatment or leaflet only- Abstinence/cessation, 6+ months 37](#_Toc34435960)

[Individually tailored print-based self-help materials (no face-to-face contact) versus No materials/ no interventions- Abstinence/cessation- 6+ months 38](#_Toc34435961)

[Matkin 2019 {1228} 39](#_Toc34435962)

[Hotline and self-help materials versus Minimal intervention- Abstinence/cessation, 12-18 months 39](#_Toc34435963)

[Intense telephone counselling versus Minimal intervention- Abstinence/cessation, 6+ months 39](#_Toc34435964)

[Brief motivational telephone counselling versus Usual care telephone call- Abstinence/cessation- 12 months 39](#_Toc34435965)

[Telephone counselling for smoking reduction versus Usual care telephone call- Abstinence/cessation, 12 months 40](#_Toc34435966)

[Stead 2013 {1998} 41](#_Toc34435967)

[Physician advice (minimal or intensive interventions) versus No advice (or usual care)- Abstinence/cessation, 6+ months 41](#_Toc34435968)

[Physician advice with follow-up versus Minimal intervention /advice with single visit- Abstinence/cessation, 6+ months 42](#_Toc34435969)

[Intensive advice versus Minimal advice- Abstinence/cessation, 6+ months 43](#_Toc34435970)

[Stead 2016 {1356} 44](#_Toc34435971)

[Combined pharmacotherapy and behavioural interventions versus Usual care or minimal intervention- Abstinence/cessation, 6+ months 44](#_Toc34435972)

[Combined pharmacotherapy and behavioural interventions versus Usual care or no intervention- Abstinence/cessation, 12 months 45](#_Toc34435973)

[Stead 2017 {538} 46](#_Toc34435974)

[Group therapy versus No intervention- Abstinence/cessation, 6+ months 46](#_Toc34435975)

[Taylor 2017 {411} 47](#_Toc34435976)

[Interactive and tailored internet intervention versus Non-active control- Abstinence/cessation, 6-12 months 47](#_Toc34435977)

[Internet plus behavioural support versus Non-internet-based non-active control- Abstinence/cessation, 6-12 months 47](#_Toc34435978)

[Tsoi 2013 {1698} 48](#_Toc34435979)

[Bupropion versus Placebo- Abstinence/cessation, 6 months 48](#_Toc34435980)

[Bupropion versus Placebo- Reduction in number of cigarettes per day from baseline, 6 months 48](#_Toc34435981)

[Bupropion versus Placebo- Tobacco smoking reduction- Expired CO level, 6 months 49](#_Toc34435982)

[Bupropion versus Placebo- Change in emotional state End of treatment & 49](#_Toc34435983)

[Varenicline versus Placebo- Abstinence/cessation, 6 months 50](#_Toc34435984)

[Van der Meer 2013 {1223} 51](#_Toc34435985)

[Bupropion versus Placebo (current depression)- Abstinence/cessation, 6-12 months 51](#_Toc34435986)

[Bupropion versus Placebo (past depression)- Abstinence/cessation, 6-12 months 51](#_Toc34435987)

[NRT versus Placebo (past depression)- Abstinence/cessation, 6+ months 51](#_Toc34435988)

[Vodoplivec-Jamsek 2012 {1343} 53](#_Toc34435989)

[Mobile phone short message service versus Control- Abstinence/cessation, 6 months 53](#_Toc34435990)

[Mobile phone short message service versus Control - Adverse events (rates of car crash), 6 months 53](#_Toc34435991)

[Mobile phone short message service versus Control - Adverse events (pain in thumb/finger joint), 6 months 54](#_Toc34435992)

[White 2014 {1618} 55](#_Toc34435993)

[Acupuncture versus Sham acupuncture- Abstinence/cessation, 6-12 months 55](#_Toc34435994)

[Acupuncture versus Waiting list/no intervention- Abstinence/cessation, 6-12 months 55](#_Toc34435995)

[Continuous auricular stimulation versus Sham stimulation- Abstinence/cessation, 6-12 months 56](#_Toc34435996)

[Laser therapy versus Sham laser- Abstinence/cessation, 6-12 months 56](#_Toc34435997)

[Electrostimulation versus Sham electrostimulation- Abstinence/cessation, 6-12 months 57](#_Toc34435998)

[Whittaker 2016 {1803} 58](#_Toc34435999)

[Mobile-phone based intervention versus Usual care Abstinence/cessation, 6+ months 58](#_Toc34436000)

#

# Barnes 2019 {3836}

## Hypnotherapy alone versus Placebo- Abstinence/cessation, 12 months


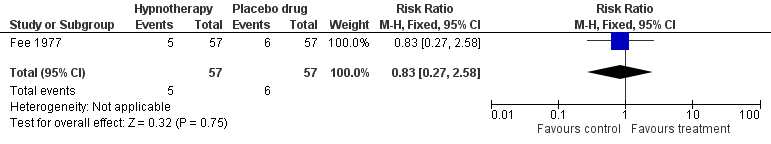


# Cahill 2010 {1652}

## Stage-based expert systems or tailored self-help materials versus Assessment only- Abstinence/cessation, 6+ months


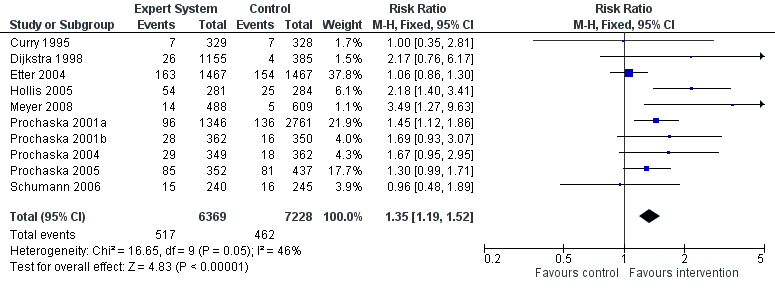


## Stage-based interactive computer programmes versus Usual care- Abstinence/cessation, 12+ month


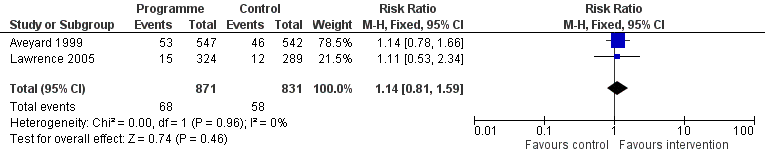


## Stage-based telephone counselling versus Usual Care- Abstinence/cessation, 12 months


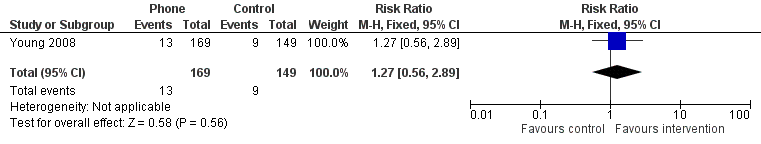


## Stage-based individual counselling and/or advice versus Usual care- Abstinence/cessation, 6+ months


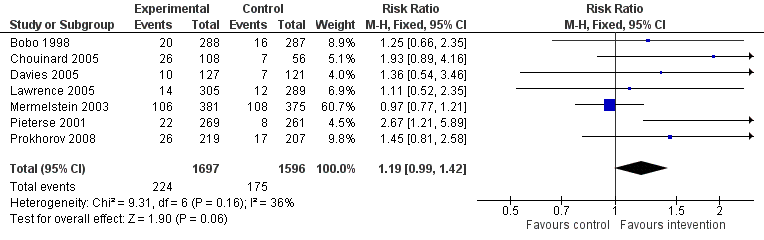


## Stage-based individual counselling or advice versus Assessment only- Abstinence/cessation, 6+ months


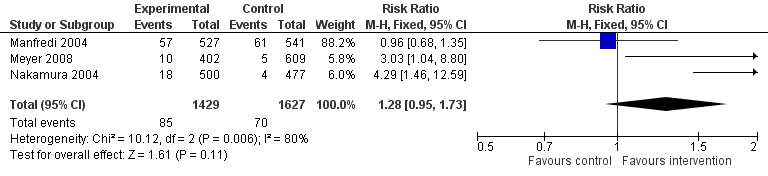


# Cahill 2016 {1960}

## Cystisine versus Placebo- Abstinence/cessation (continuous), 6+ months


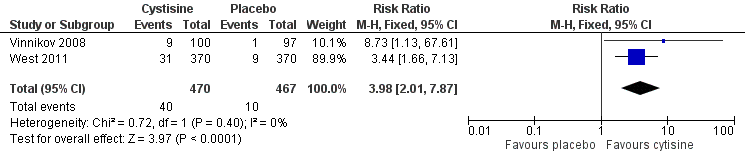


## Cytisine versus Placebo- Abstinence/cessation (point prevalence), 2 years


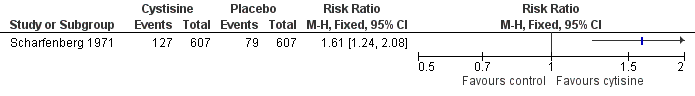


## Varenicline versus Placebo- Abstinence/cessation, longest follow-up (6+ months)


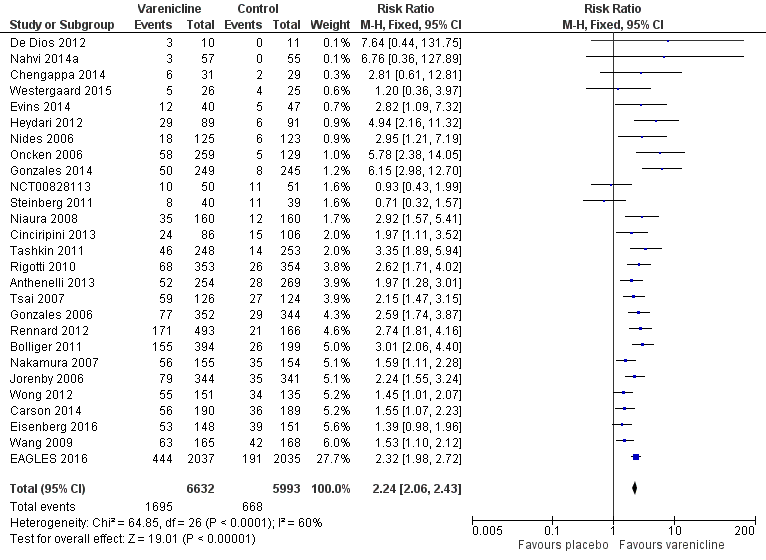


## Varenicline versus Placebo- Abstinence/cessation, 6 months


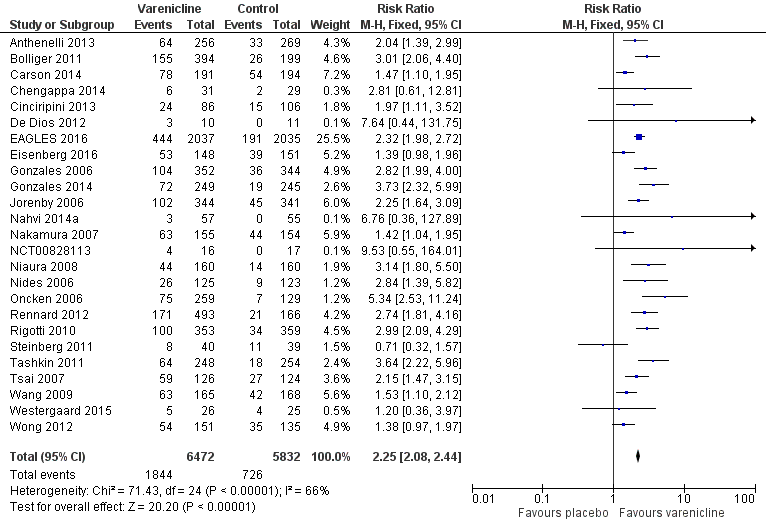


## Long term varenicline versus Placebo- Abstinence/cessation, 6-12 months


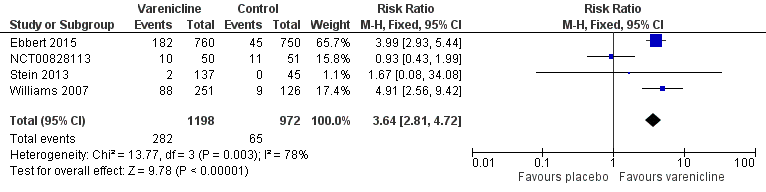


## Low dose varenicline versus Placebo- Abstinence/cessation- 12 months


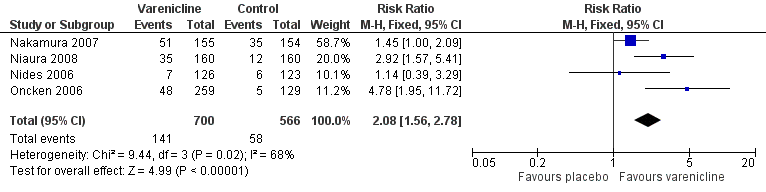


## Variable dosing of varenicline versus Placebo- Abstinence/cessation, 12 months


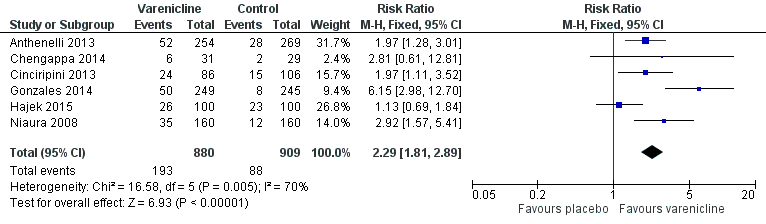


## Varenicline versus Placebo- Abstinence/cessation in smokers reducing to quit, 12 months


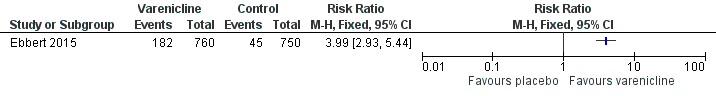


## Varenicline versus Placebo- Abstinence/cessation in smokers with schizophrenia, bipolar, or other psychiatric disorder, 6 months


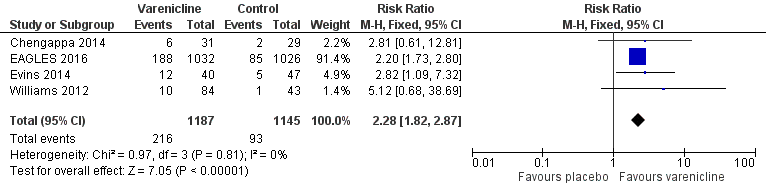


## Varenicline versus Placebo- Abstinence/cessation in smokers with depression and motivated/wishing to quit, 12 months


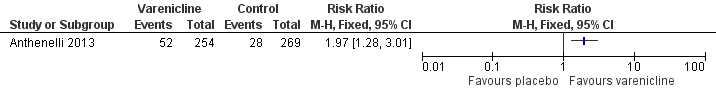


## Varenicline versus Placebo- Abstinence/cessation in smokers who previously failed to quit on varenicline but are motivated/wishing to try quitting again, 12 months


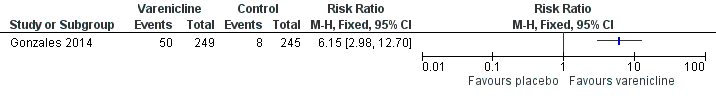


## Varenicline versus Placebo- Adverse events (nausea), Range of follow-up times


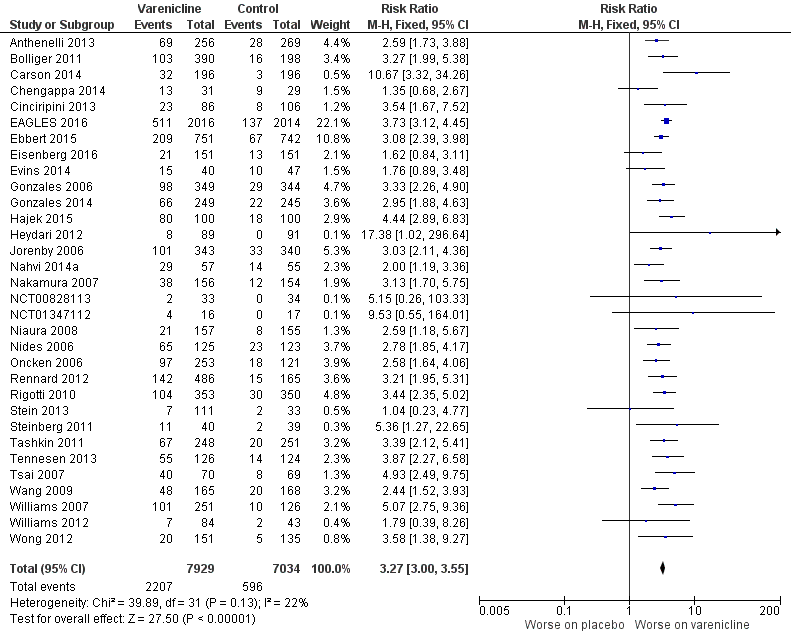


## Varenicline versus Placebo- Adverse events (insomnia), Range of follow-up times


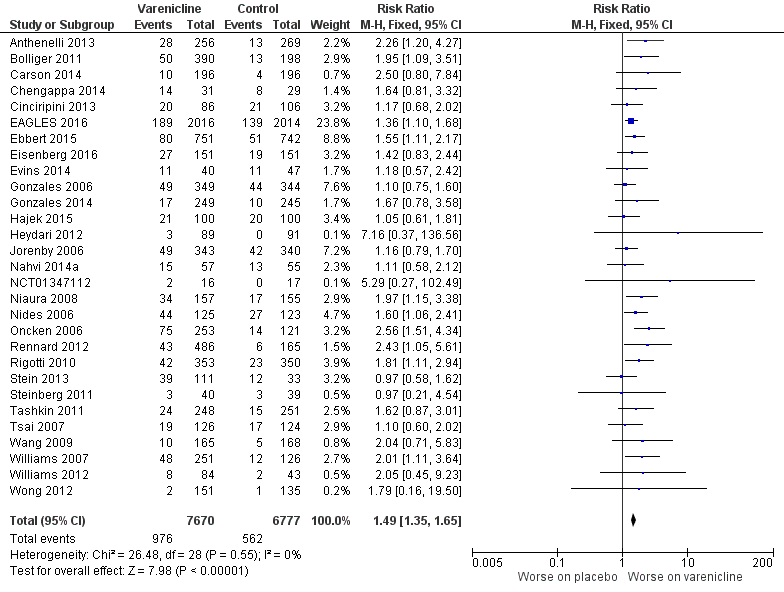


## Varenicline versus Placebo- Adverse events (abnormal dreams), Range of follow-up times


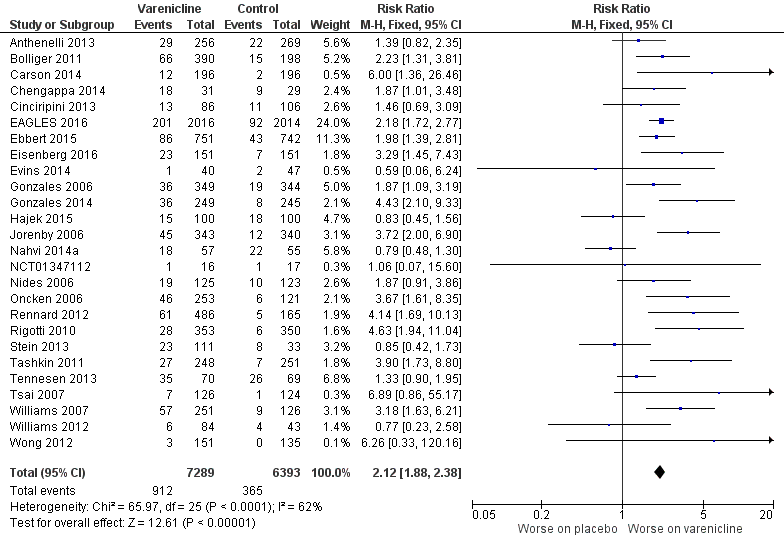


## Varenicline versus Placebo- Adverse events (headache), Range of follow-up times


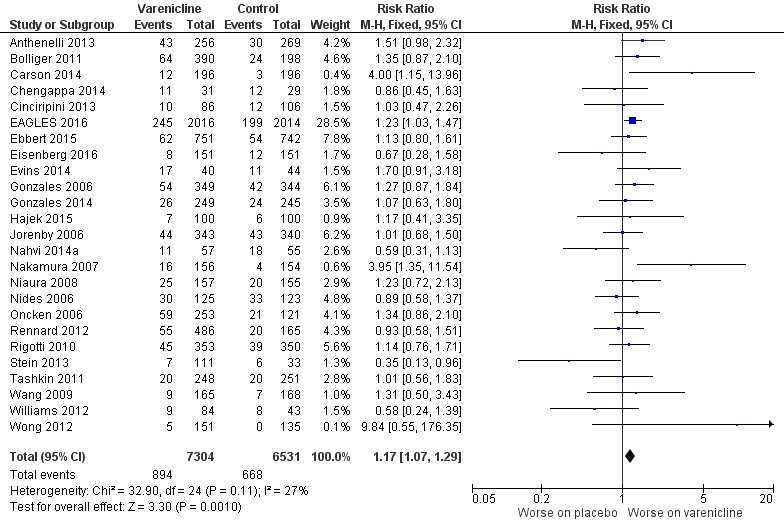


## Varenicline versus Placebo- Adverse events (depression), Range of follow-up times


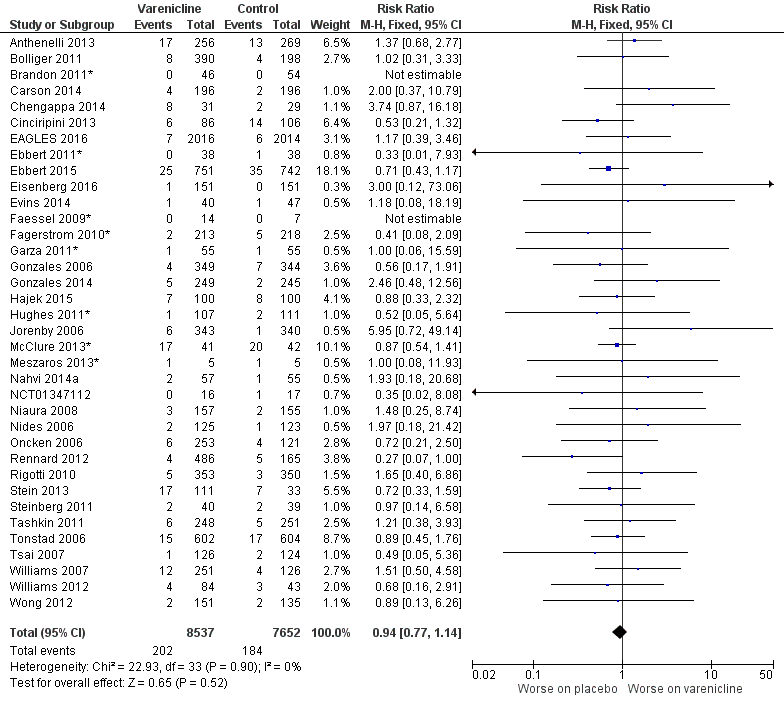


## Varenicline versus Placebo- Adverse events (suicidal ideation), Range of follow-up times


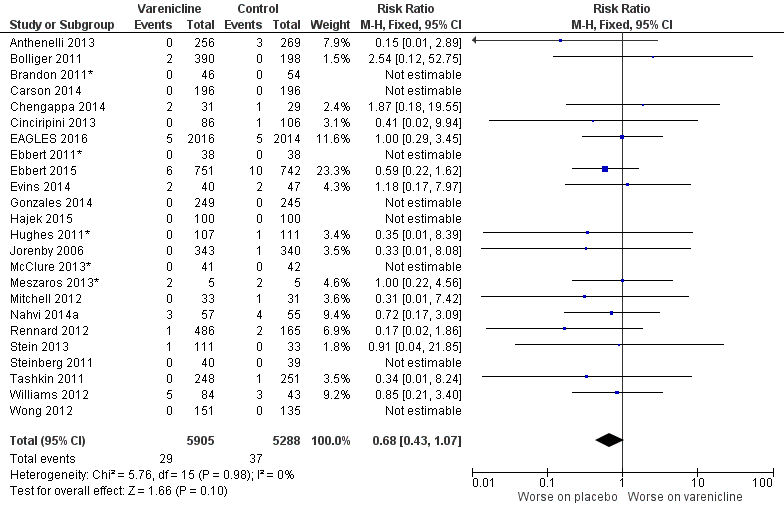


## Varenicline versus Placebo- Serious adverse event (at least one), Range of follow-up times


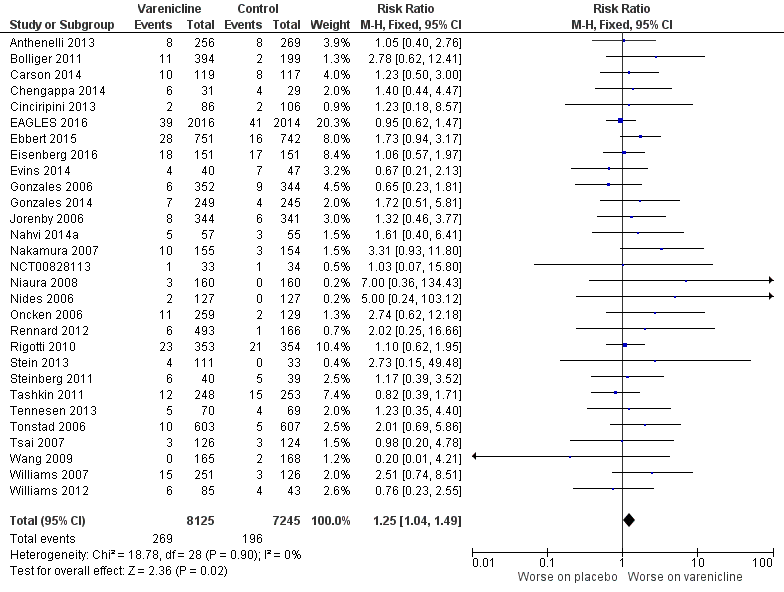


## Varenicline versus Placebo- Serious adverse event (at least one during or immediately after treatment), Range of follow-up times


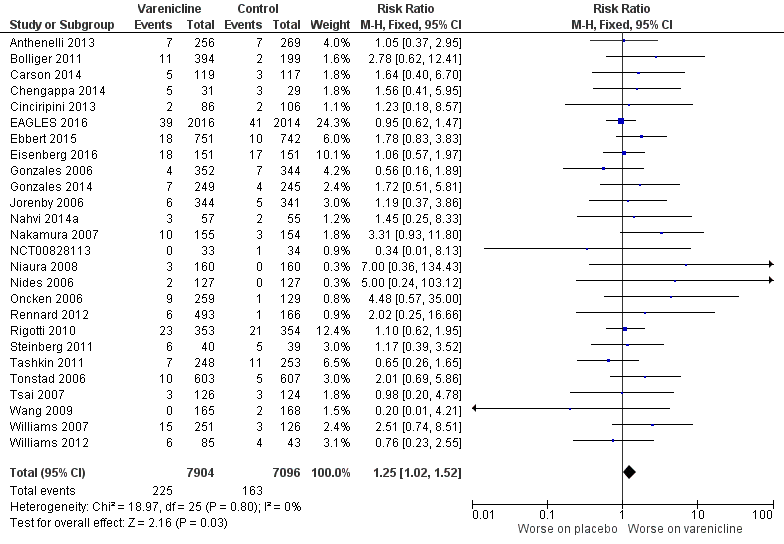


## Varenicline versus Placebo- Neuropsychiatric events (depression, suicidal ideation), not deaths, Range of follow-up times


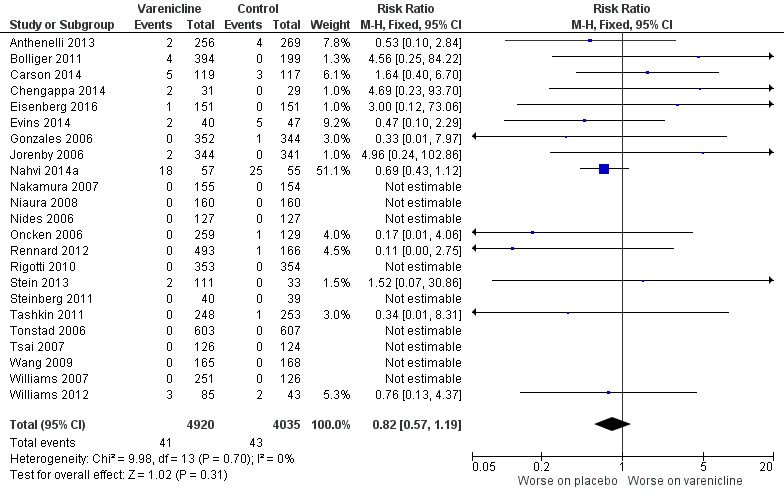


## Varenicline versus Placebo- Serious adverse events (cardiac, including deaths), Range of follow-up times


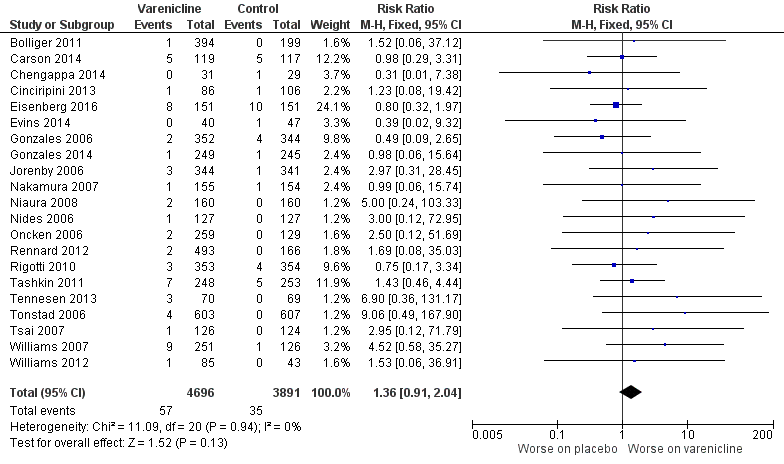


# Farley 2012 {1469}

## Buproprion versus Placebo- Weight gain, End of treatment


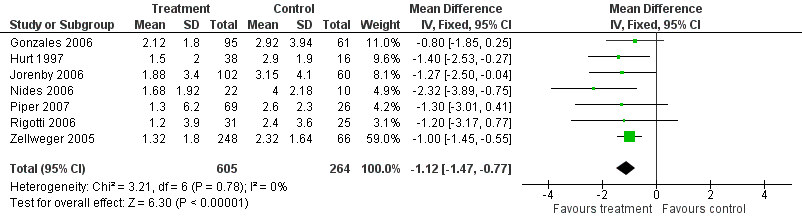


## Buproprion versus Placebo- Weight gain, 6 months


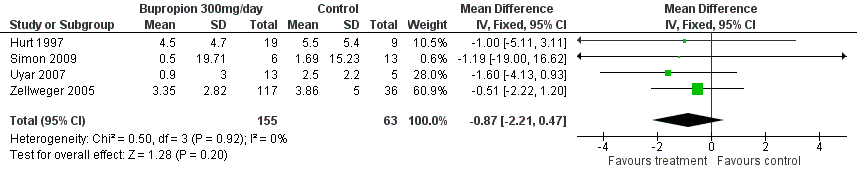


## Buproprion versus Placebo- Weight gain, 12 months


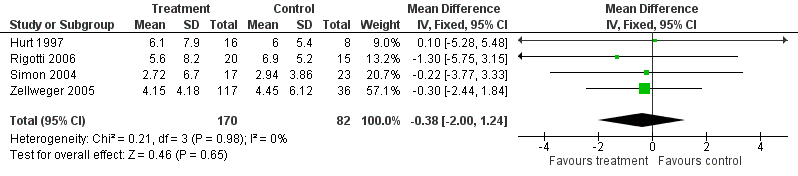


## NRT versus Placebo- Weight gain, End of treatment


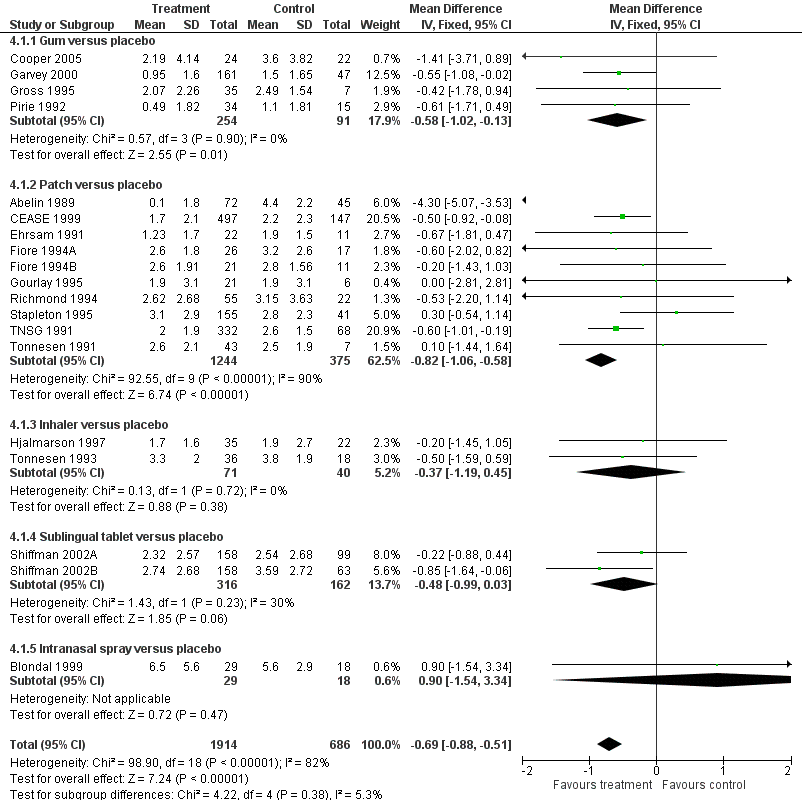


## NRT versus Placebo- Weight gain, 6 months


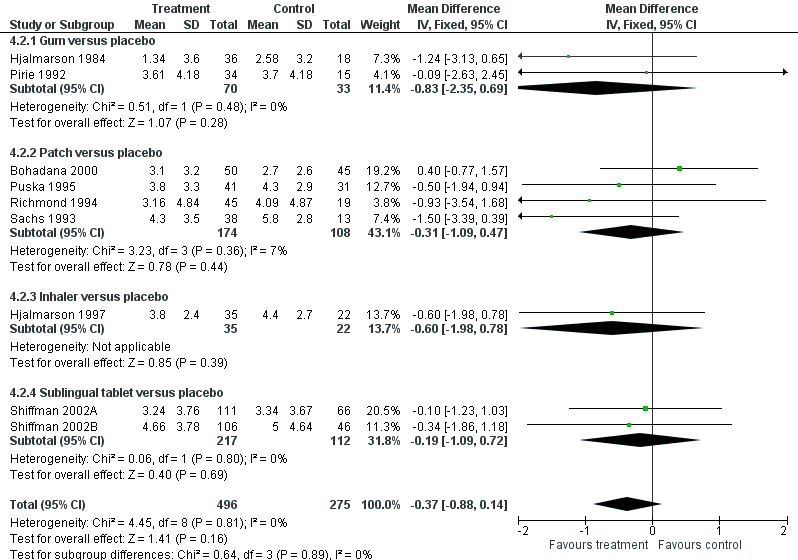


## NRT versus Placebo- Weight gain, 12 months


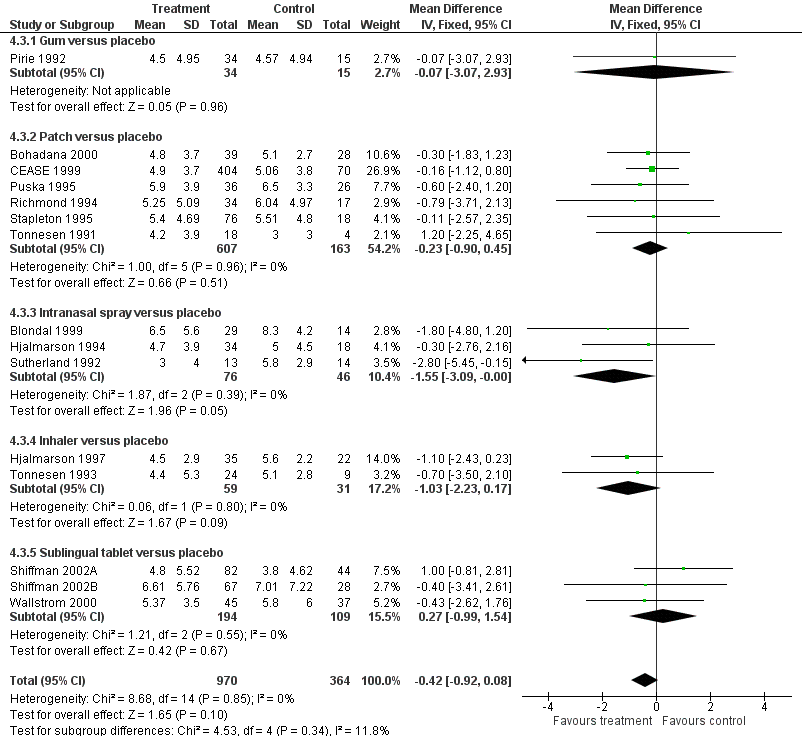


## Varenicline 2mg/day versus Placebo- Weight gain, End of treatment


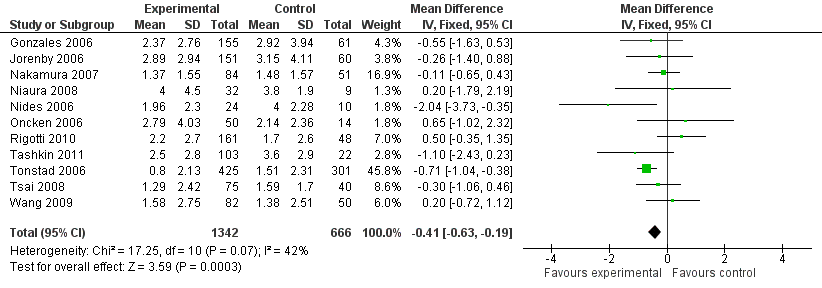


## Varenicline 2mg/day versus Placebo- Weight gain, 6 months


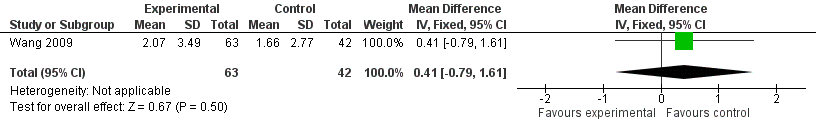


## Varenicline 2mg/day versus Placebo- Weight gain, 12 months


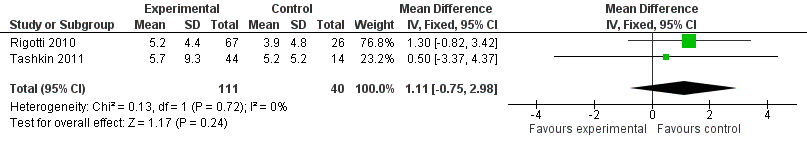


## Varenicline 1mg/day versus Placebo- Weight gain, End of treatment


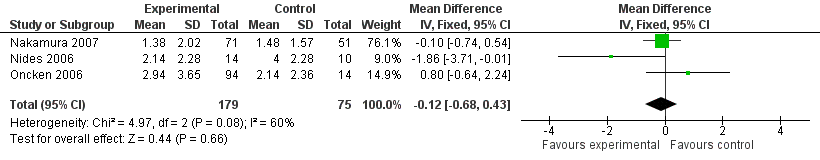


# Hartmann-Boyce 2018 {332}

## NRT patch versus Placebo- Adverse events (Palpitations/chest pains), Range of follow-up times


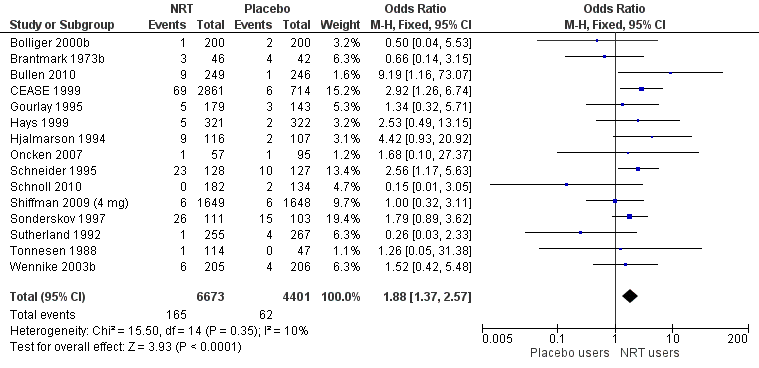


# Hollands 2019 {3841}

## Interventions to increase adherence for tobacco dependence versus Usual or standard care- Abstinence/cessation, 6 months


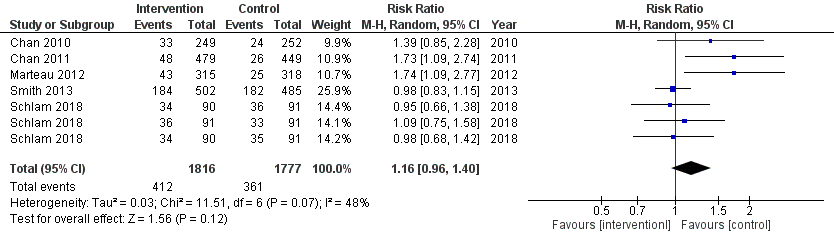


# Howes 2020 {96}

## Bupropion versus Placebo- Abstinence/cessation, 6 months


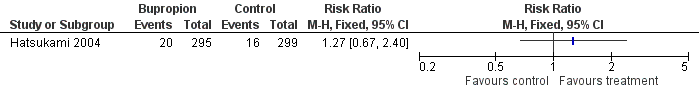


## Bupropion versus Placebo- Reduction in cotinine >50%, 12 months


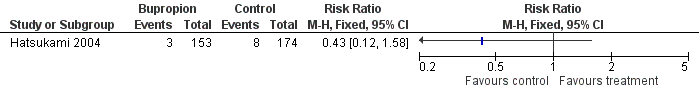


## St John’s wort versus Placebo- Abstinence/cessation, 6 months


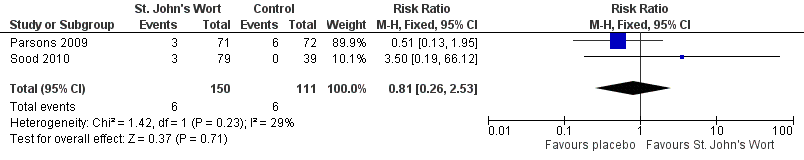


## S-Adenosyl-L-Methionine (SAMe) versus Placebo- Abstinence/cessation, 6 months


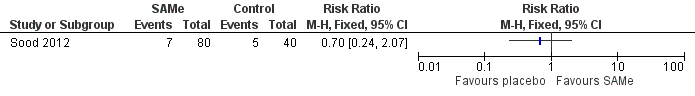


# Lancaster 2017 {539}

## Individual counselling versus Minimal contact control- Abstinence/cessation, 6+ months


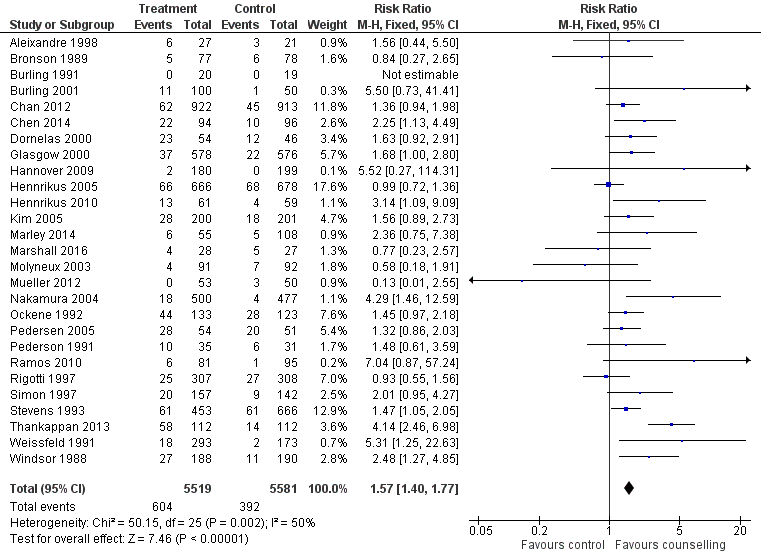


# Lindson-Hawley 2016 {671}

## NRT versus Placebo- Abstinence/cessation, 12 months to 24 months


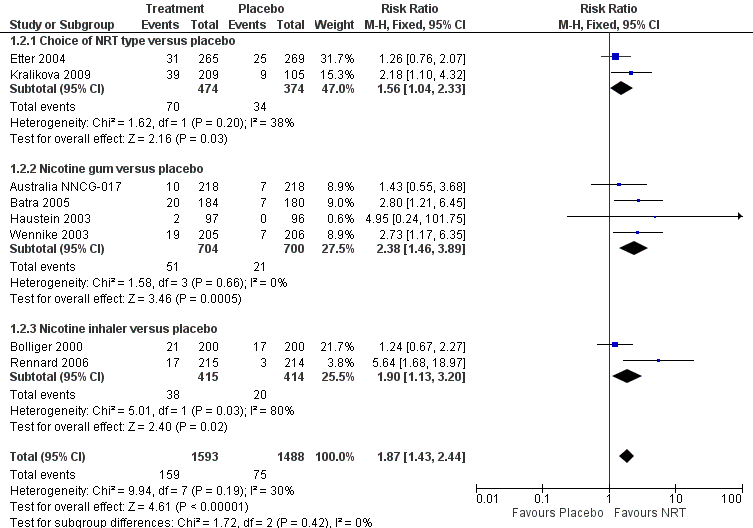


## NRT versus Placebo- Reduction in cigarettes/day of >50% of baseline or cessation, 12+ months


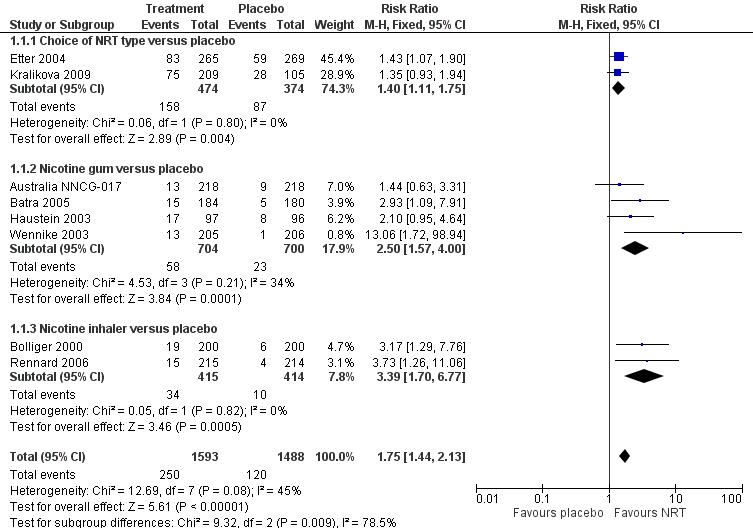


## Bupropion versus Placebo- Abstinence/cessation, 6 months


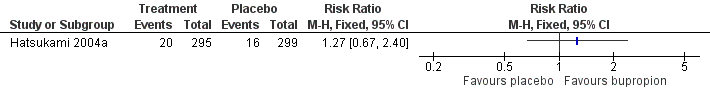


## Bupropion versus Placebo- Reduction in cigarettes/day of >50% of baseline or cessation, 12 months


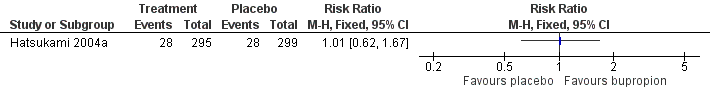


## Varenicline versus Placebo- Abstinence/cessation, 6 months


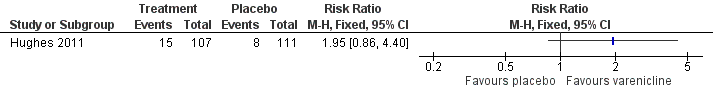


## Telephone counselling plus self-help materials versus Usual care- Abstinence/cessation, 12 months &

## Telephone counselling plus self-help materials versus Usual care- Reduction in cigarettes/day of >50% of baseline or cessation, 12 months


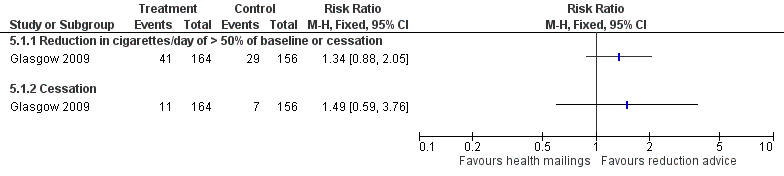


## E-cigarettes versus Placebo- Abstinence/cessation, 12 months


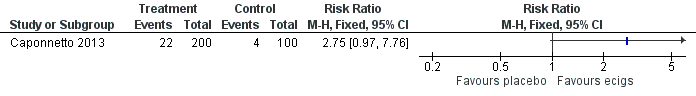


## E-cigarettes versus Placebo- Reduction in cigarettes/day of >50% of baseline or cessation, 12months


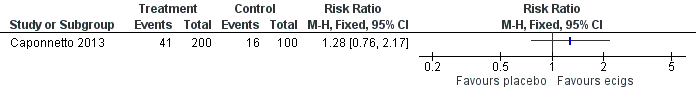


# Livingstone-Banks 2019 {1077}

## Non-tailored print-based self-help materials (no face-to-face contact) versus No materials/no intervention- Abstinence/cessation, 6+ months


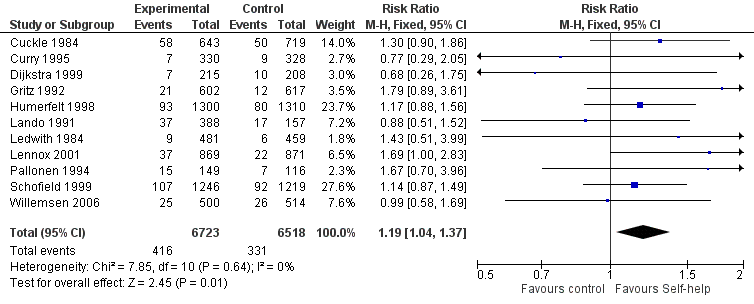


## Non-tailored print-based self-help (no face-to-face contact) versus No materials/no interventions- Abstinence/cessation, 6 months


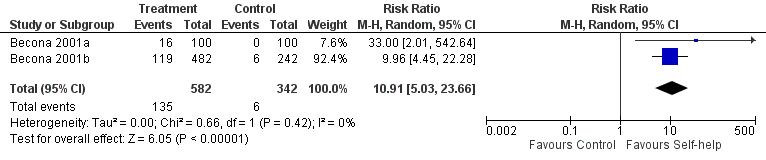


## Non-tailored print-based self-help materials (no face-to-face contact) versus Brief leaflet- Abstinence/cessation, 6+ months


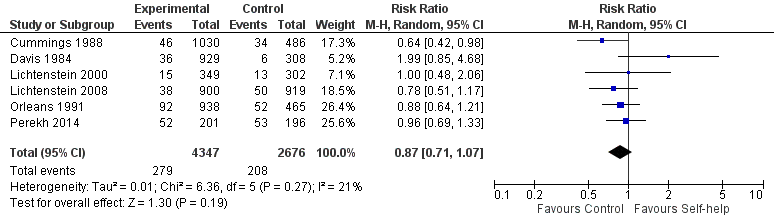


## Non-tailored print-based self-help materials (with face-to-face contact) versus No treatment or leaflet only- Abstinence/cessation, 6+ months


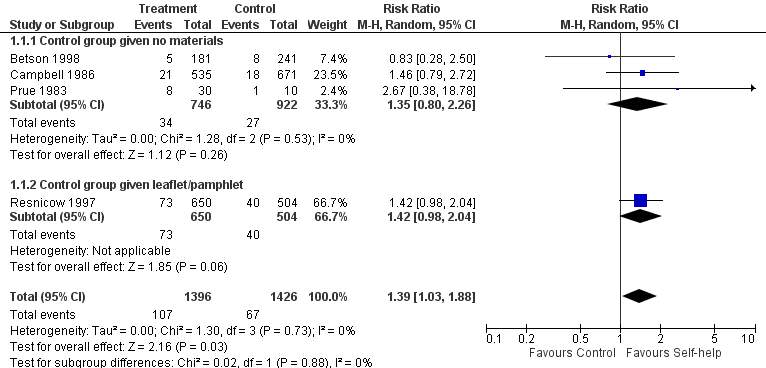


## Individually tailored print-based self-help materials (no face-to-face contact) versus No materials/ no interventions- Abstinence/cessation- 6+ months


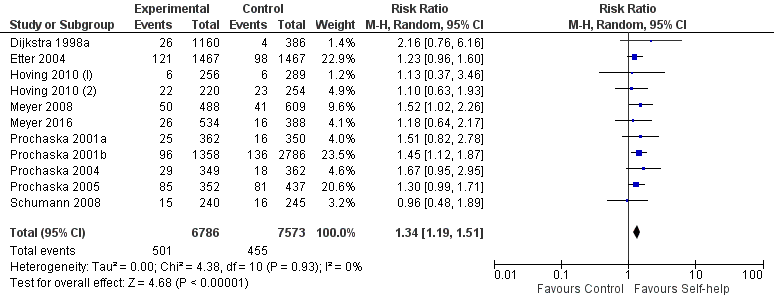


# Matkin 2019 {1228}

## Hotline and self-help materials versus Minimal intervention- Abstinence/cessation, 12-18 months


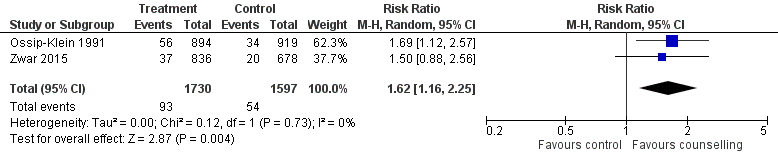


## Intense telephone counselling versus Minimal intervention- Abstinence/cessation, 6+ months


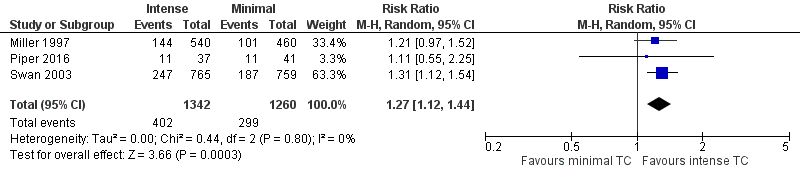


## Brief motivational telephone counselling versus Usual care telephone call- Abstinence/cessation- 12 months


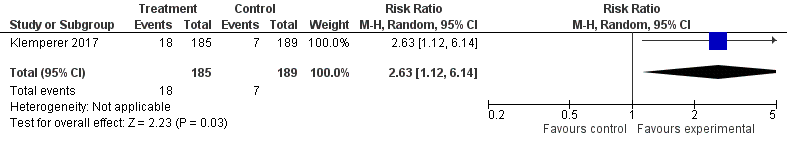


## Telephone counselling for smoking reduction versus Usual care telephone call- Abstinence/cessation, 12 months


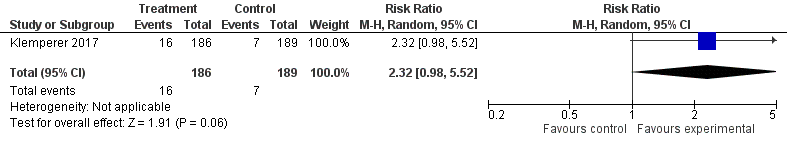


# Stead 2013 {1998}

## Physician advice (minimal or intensive interventions) versus No advice (or usual care)- Abstinence/cessation, 6+ months


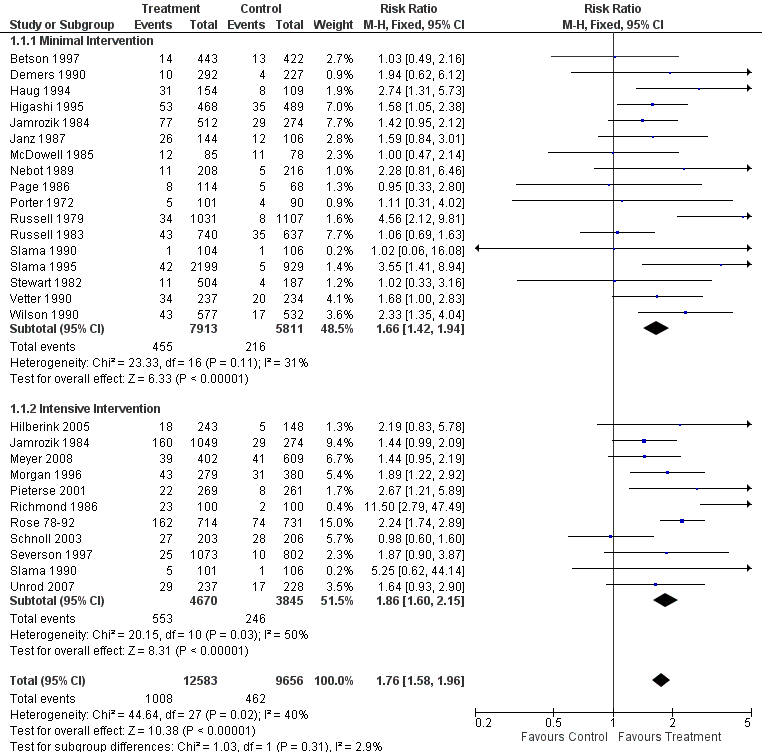


## Physician advice with follow-up versus Minimal intervention /advice with single visit- Abstinence/cessation, 6+ months


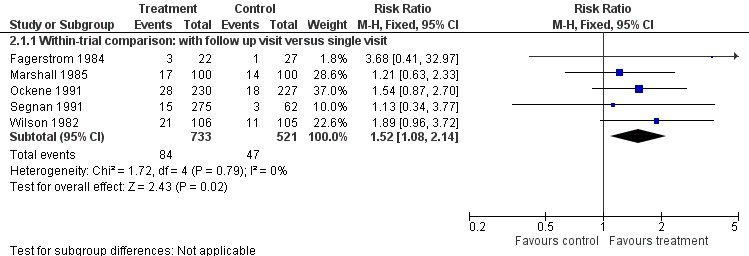


## Intensive advice versus Minimal advice- Abstinence/cessation, 6+ months


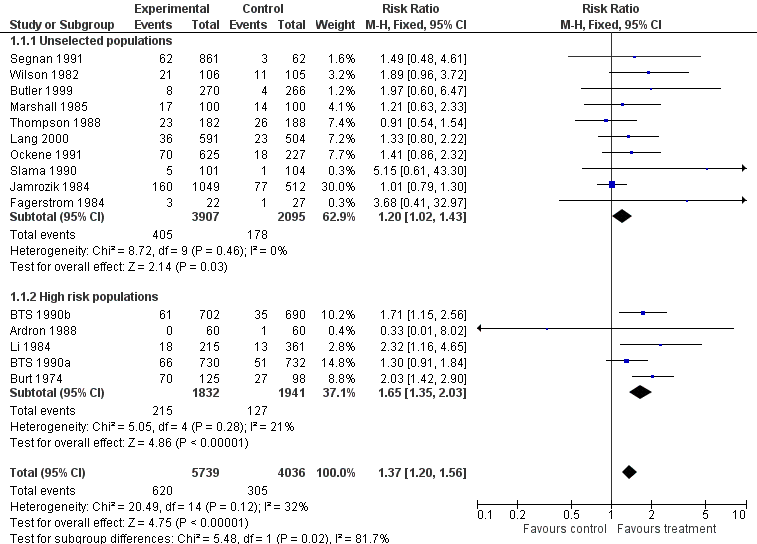


# Stead 2016 {1356}

## Combined pharmacotherapy and behavioural interventions versus Usual care or minimal intervention- Abstinence/cessation, 6+ months


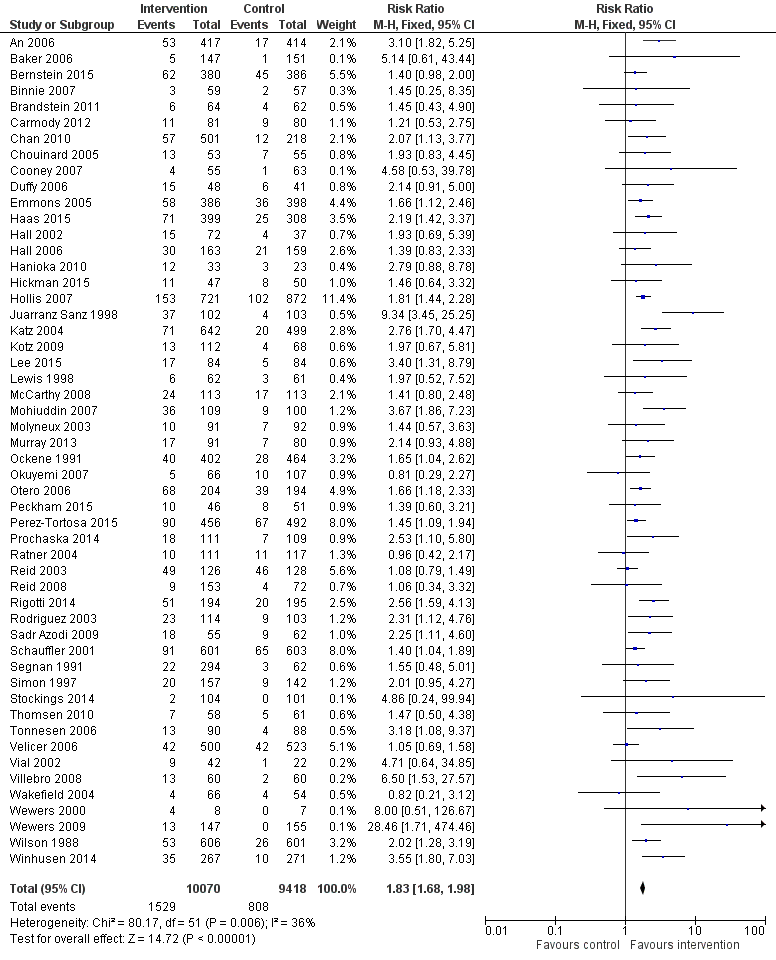


## Combined pharmacotherapy and behavioural interventions versus Usual care or no intervention- Abstinence/cessation, 12 months


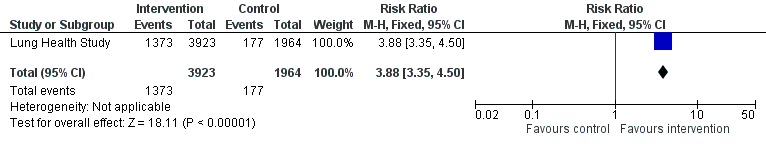


# Stead 2017 {538}

## Group therapy versus No intervention- Abstinence/cessation, 6+ months


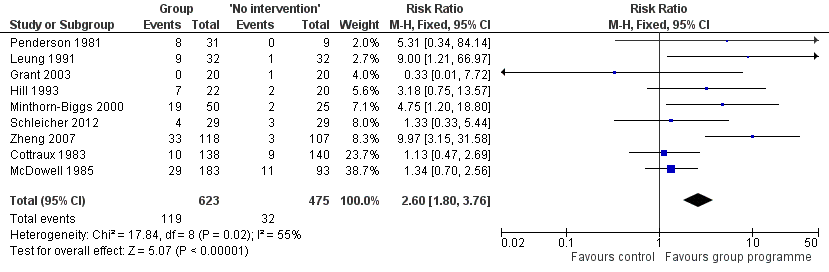


# Taylor 2017 {411}

## Interactive and tailored internet intervention versus Non-active control- Abstinence/cessation, 6-12 months


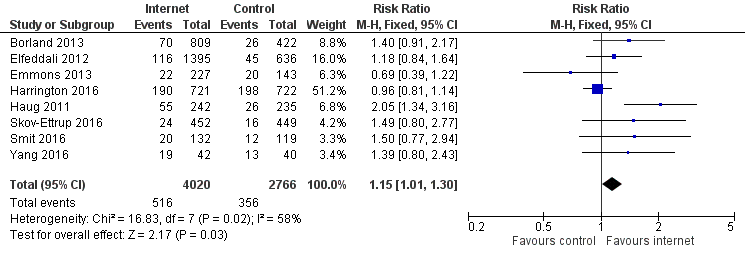


## Internet plus behavioural support versus Non-internet-based non-active control- Abstinence/cessation, 6-12 months


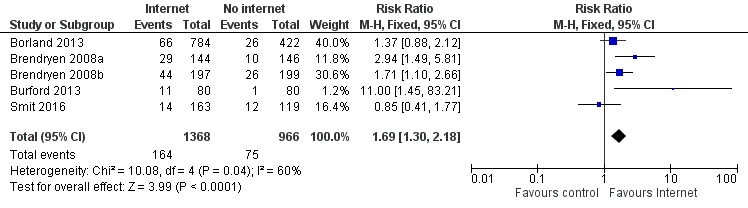


# Tsoi 2013 {1698}

## Bupropion versus Placebo- Abstinence/cessation, 6 months


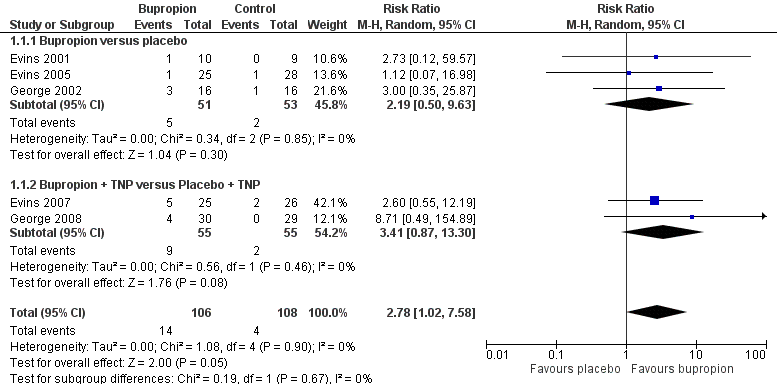


## Bupropion versus Placebo- Reduction in number of cigarettes per day from baseline, 6 months


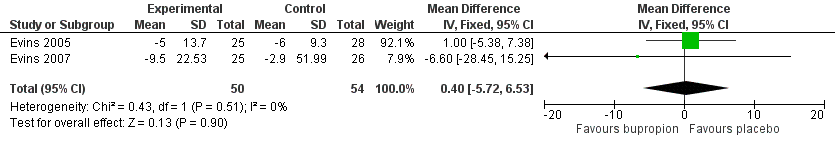


## Bupropion versus Placebo- Tobacco smoking reduction- Expired CO level, 6 months


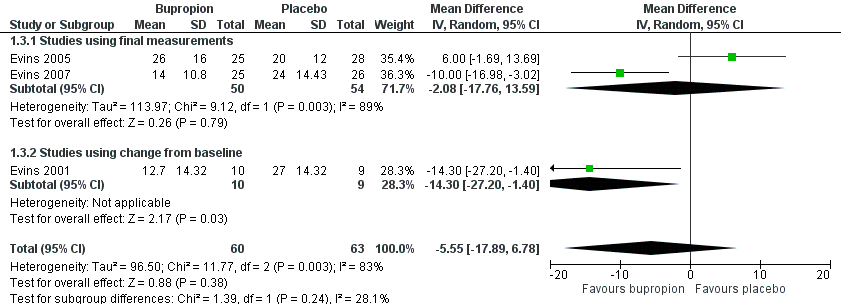


## Bupropion versus Placebo- Change in emotional state (positive symptoms, negative symptoms, depressive symptoms), End of treatment


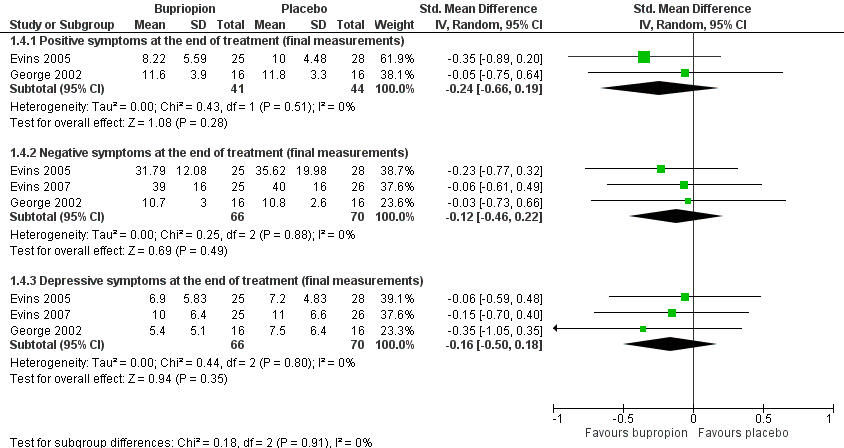


##

## Varenicline versus Placebo- Abstinence/cessation, 6 months


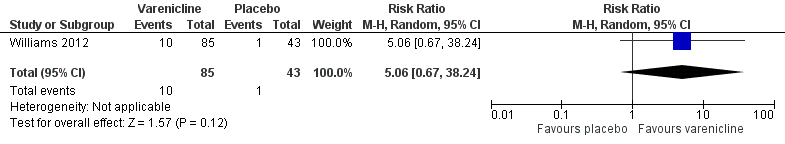


# Van der Meer 2013 {1223}

## Bupropion versus Placebo (current depression)- Abstinence/cessation, 6-12 months


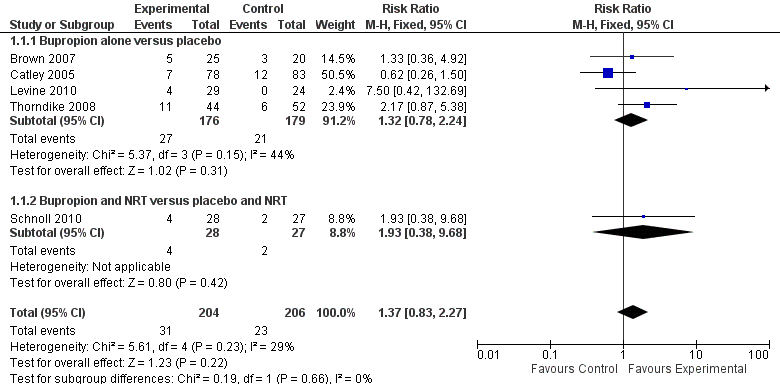


## Bupropion versus Placebo (past depression)- Abstinence/cessation, 6-12 months


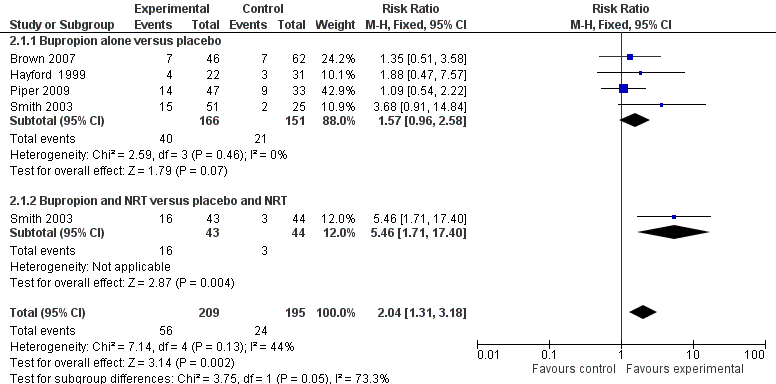


## NRT versus Placebo (past depression)- Abstinence/cessation, 6+ months


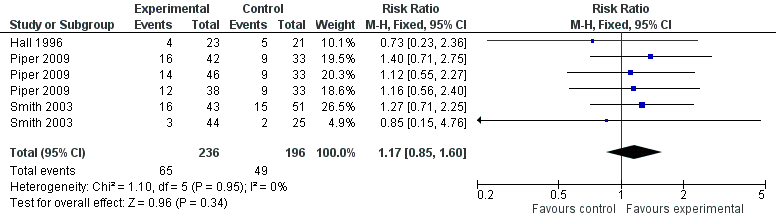


# Vodoplivec-Jamsek 2012 {1343}

## Mobile phone short message service versus Control- Abstinence/cessation, 6 months


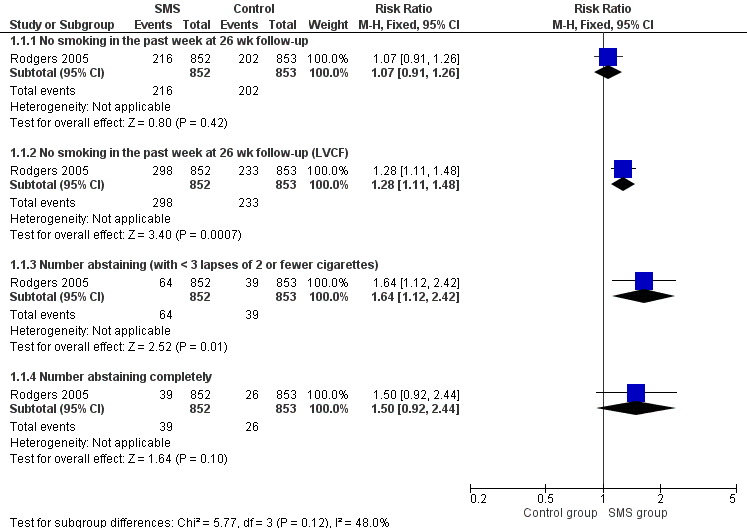


## Mobile phone short message service versus Control - Adverse events (rates of car crash), 6 months


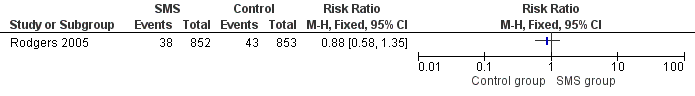


## Mobile phone short message service versus Control - Adverse events (pain in thumb/finger joint), 6 months


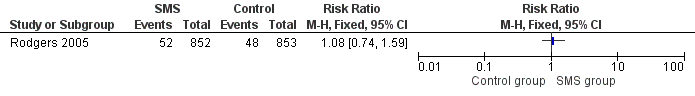


# White 2014 {1618}

## Acupuncture versus Sham acupuncture- Abstinence/cessation, 6-12 months


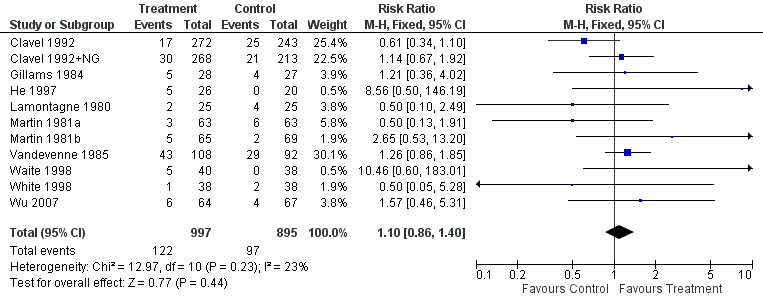


## Acupuncture versus Waiting list/no intervention- Abstinence/cessation, 6-12 months


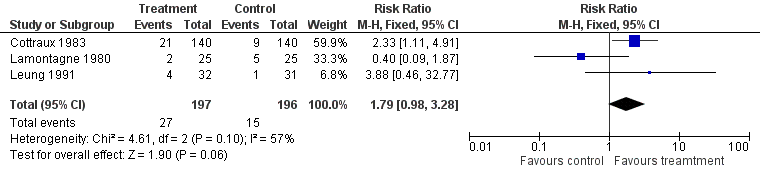


## Continuous auricular stimulation versus Sham stimulation- Abstinence/cessation, 6-12 months


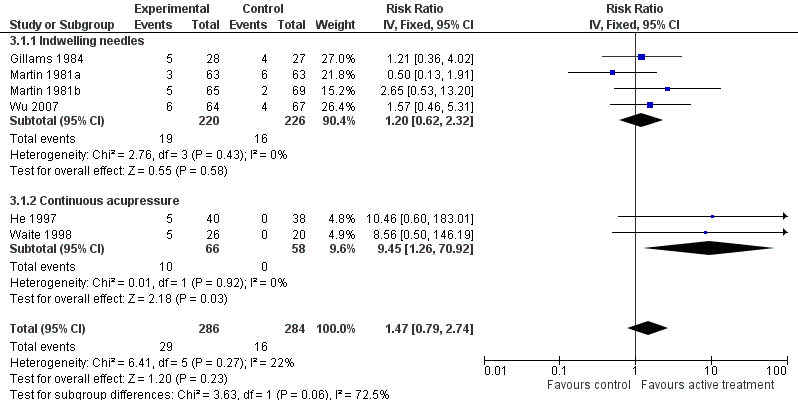


## Laser therapy versus Sham laser- Abstinence/cessation, 6-12 months


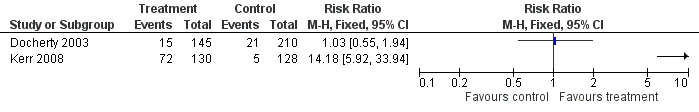


## Electrostimulation versus Sham electrostimulation- Abstinence/cessation, 6-12 months


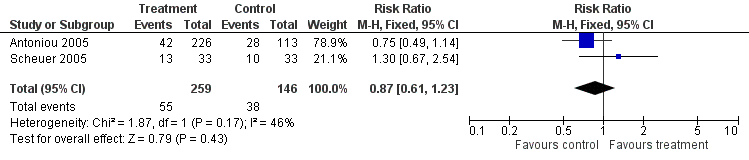


# Whittaker 2019 {1803}

## Mobile-phone based intervention versus Usual care Abstinence/cessation, 6+ months


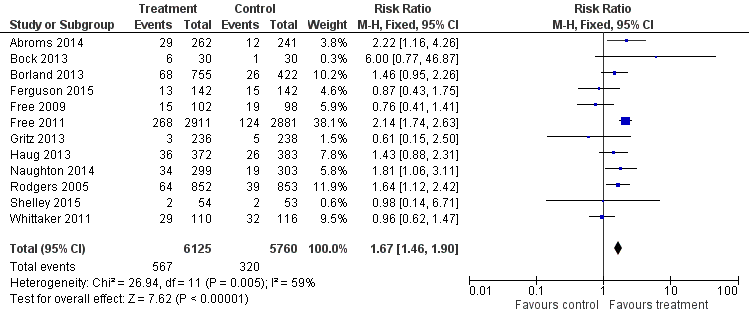

Supplement: Supplementary file 9 — Additional file 9. Forest plots for included analyses. [file 13643_2024_2570_MOESM9_ESM.docx]
