# Supplementary material for: Effectiveness of smoking cessation interventions among adults: an overview of systematic reviews
Source: Syst Rev. 2024 Jul 12;13:179. doi: 10.1186/s13643-024-02570-9 (PMC11242003; doi:10.1186/s13643-024-02570-9)
Supplement: Supplementary file 10 — Additional file 10. Risk of bias figures for included analyses. [file 13643_2024_2570_MOESM10_ESM.docx]

**Additional file 10. Risk of bias figures for included analyses**

Table of Contents

[Barnes 2019 {3836} 6](#_Toc34436001)

[Hypnotherapy alone versus Placebo- Abstinence/cessation, 12 months 6](#_Toc34436002)

[Cahill 2010 {1652} 7](#_Toc34436003)

[Stage-based expert systems or tailored self-help materials versus Assessment only- Abstinence/cessation, 6+ months 7](#_Toc34436004)

[Stage-based expert systems or tailored self-help materials versus Assessment only- Abstinence/cessation, 14 months 7](#_Toc34436005)

[Stage-based interactive computer programmes versus Usual care- Abstinence/cessation, 12+ months 8](#_Toc34436006)

[Stage-based telephone counselling versus Usual Care- Abstinence/cessation, 12 months 8](#_Toc34436007)

[Stage-based individual counselling and/or advice versus Usual care- Abstinence/cessation, 6+ months 9](#_Toc34436008)

[Stage-based individual counselling or advice versus Assessment only- Abstinence/cessation, 6+ months 9](#_Toc34436009)

[Cahill 2016 {1960} 10](#_Toc34436010)

[Cystisine versus Placebo- Abstinence/cessation (continuous), 6+ months 10](#_Toc34436011)

[Cytisine versus Placebo- Abstinence/cessation (point prevalence), 2 years 10](#_Toc34436012)

[Cytisine versus Placebo- Adverse events, Follow-up unclear/NR 10](#_Toc34436013)

[Varenicline versus Placebo- Abstinence/cessation, longest follow-up (6+ months) 11](#_Toc34436014)

[Varenicline versus Placebo- Abstinence/cessation, 6 months 12](#_Toc34436015)

[Long term varenicline versus Placebo- Abstinence/cessation, 6-12 months 13](#_Toc34436016)

[Low dose varenicline versus Placebo- Abstinence/cessation- 12 months 13](#_Toc34436017)

[Variable dosing of varenicline versus Placebo- Abstinence/cessation, 12 months 14](#_Toc34436018)

[Varenicline versus Placebo- Abstinence/cessation in smokers reducing to quit, 12 months 14](#_Toc34436019)

[Varenicline versus Placebo- Abstinence/cessation in smokers with schizophrenia, bipolar, or other psychiatric disorder, 6 months 15](#_Toc34436020)

[Varenicline versus Placebo- Abstinence/cessation in smokers with depression and motivated/wishing to quit, 12 months 15](#_Toc34436021)

[Varenicline versus Placebo- Abstinence/cessation in smokers who previously failed to quit on varenicline but are motivated/wishing to try quitting again, 12 months 15](#_Toc34436022)

[Varenicline versus Placebo- Adverse events (nausea), Range of follow-up times 16](#_Toc34436023)

[Varenicline versus Placebo- Adverse events (insomnia), Range of follow-up times 18](#_Toc34436024)

[Varenicline versus Placebo- Adverse events (abnormal dreams), Range of follow-up times 19](#_Toc34436025)

[Varenicline versus Placebo- Adverse events (headache), Range of follow-up times 20](#_Toc34436026)

[Varenicline versus Placebo- Adverse events (depression), Range of follow-up times 21](#_Toc34436027)

[Varenicline versus Placebo- Adverse events (suicidal ideation), Range of follow-up times 23](#_Toc34436028)

[Varenicline versus Placebo- Serious adverse event (at least one), Range of follow-up times 24](#_Toc34436029)

[Varenicline versus Placebo- Serious adverse event (at least one during or immediately after treatment), Range of follow-up times 25](#_Toc34436030)

[Varenicline versus Placebo- Neuropsychiatric events (depression, suicidal ideation), not deaths, Range of follow-up times 26](#_Toc34436031)

[Varenicline versus Placebo- Serious adverse events (cardiac, including deaths), Range of follow-up times 27](#_Toc34436032)

[Varenicline versus Placebo- Adverse events (discontinuation), Range of follow-up times 28](#_Toc34436033)

[Farley 2012 {1469} 29](#_Toc34436034)

[Buproprion versus Placebo- Weight gain, End of treatment 29](#_Toc34436035)

[Buproprion versus Placebo- Weight gain, 6 months 29](#_Toc34436036)

[Bupropion versus Placebo- Weight gain, 12 months 30](#_Toc34436037)

[NRT versus Placebo- Weight gain, End of treatment 31](#_Toc34436038)

[NRT versus Placebo- Weight gain, 6 months 32](#_Toc34436039)

[NRT versus Placebo- Weight gain, 12 months 33](#_Toc34436040)

[Varenicline 2mg/day versus Placebo- Weight gain, End of treatment 34](#_Toc34436041)

[Varenicline 2mg/day versus Placebo- Weight gain, 6 months 34](#_Toc34436042)

[Varenicline 2mg/day versus Placebo- Weight gain, 12 months 35](#_Toc34436043)

[Varenicline 1mg/day versus Placebo- Weight gain, End of treatment 35](#_Toc34436044)

[Hartmann-Boyce 2018 {332} 36](#_Toc34436045)

[NRT patch versus Placebo- Abstinence/cessation, 6 months 36](#_Toc34436046)

[NRT patch versus Placebo- Adverse events (Palpitations/chest pains), Range of follow-up times 36](#_Toc34436047)

[NRT patch versus Placebo- Adverse events (Attrition), Follow-up unclear/NR 37](#_Toc34436048)

[NRT patch versus Placebo- Adverse events (All), Follow-up unclear/NR 37](#_Toc34436049)

[Hollands 2015 {916} 38](#_Toc34436050)

[Interventions to increase adherence for tobacco dependence versus Usual or standard care- Abstinence/cessation, 6 months 38](#_Toc34436051)

[Interventions to increase adherence to medications for tobacco dependence versus Usual or standard care- Adverse events, Follow-up unclear/NR 39](#_Toc34436052)

[Interventions to increase adherence to medications for tobacco dependence versus Usual or standard care- Change in emotional state (Anxiety), 1-week and 6 months 39](#_Toc34436053)

[Hughes 2014 {1147} 40](#_Toc34436054)

[Bupropion versus Placebo- Abstinence/cessation, 6 months 40](#_Toc34436055)

[Bupropion versus Placebo- Reduction in cigarettes/day, 12 months 40](#_Toc34436056)

[Bupropion versus Placebo- Reduction in cotinine >50%, 12 months 40](#_Toc34436057)

[Bupropion versus Placebo- Change in emotional state (depressive symptoms), Specific timepoints NR 41](#_Toc34436058)

[St John’s wort versus Placebo- Abstinence/cessation, 6 months 41](#_Toc34436059)

[S-Adenosyl-L-Methionine (SAMe) versus Placebo- Abstinence/cessation, 6 months 41](#_Toc34436060)

[Lancaster 2017 {539} 42](#_Toc34436061)

[Individual counselling versus Minimal contact control- Abstinence/cessation, 6+ months 42](#_Toc34436062)

[Lindson-Hawley 2016 {671} 43](#_Toc34436063)

[NRT versus Placebo- Abstinence/cessation, 12 months to 24 months 43](#_Toc34436064)

[NRT versus Placebo- Reduction in cigarettes/day of >50% of baseline or cessation, 12+ months 44](#_Toc34436065)

[Bupropion versus Placebo- Abstinence/cessation, 6 months 44](#_Toc34436066)

[Bupropion versus Placebo- Reduction in cigarettes/day of >50% of baseline or cessation, 12 months 44](#_Toc34436067)

[Bupropion versus Placebo- Reduction in cotinine >50%, 12 months 45](#_Toc34436068)

[Bupropion versus Placebo- Reduction in cotinine, 12 months 45](#_Toc34436069)

[Bupropion versus Placebo- Serious adverse events, Follow up unclear/NR 45](#_Toc34436070)

[Varenicline versus Placebo- Abstinence/cessation, 6 months 45](#_Toc34436071)

[Varenicline versus Placebo- Adverse events (stopping medication due to adverse event), Follow-up unclear/NR 46](#_Toc34436072)

[Telephone counselling plus self-help materials versus Usual care- Abstinence/cessation, 12 months 46](#_Toc34436073)

[Telephone counselling plus self-help materials versus Usual care- Reduction in cigarettes/day of >50% of baseline or cessation, 12 months 46](#_Toc34436074)

[Telephone counselling plus self-help materials versus Usual care- Reduction in CO >50%, 12 months 47](#_Toc34436075)

[Telephone counselling plus self-help materials versus Usual care- Reduction in cigarettes/day from baseline, 12 months 47](#_Toc34436076)

[Telephone counselling plus self-help materials versus Usual care- Reduction in CO from baseline, 12 months 47](#_Toc34436077)

[Behavioural support (advice) plus NRT plus phone calls versus No intervention- Abstinence/cessation, 6 months 48](#_Toc34436078)

[Behavioural support (advice) plus NRT plus phone calls versus No intervention- Reduction in number of cigarettes/day, 6 months 48](#_Toc34436079)

[E-cigarettes versus Placebo- Abstinence/cessation, 12 months 48](#_Toc34436080)

[E-cigarettes versus Placebo- Reduction in cigarettes/day of >50% of baseline or cessation, 12months 49](#_Toc34436081)

[E-cigarettes versus Placebo- Reduction in number of cigarettes/day, 12 months 49](#_Toc34436082)

[E-cigarettes versus Placebo- Reduction in CO, 12 months 49](#_Toc34436083)

[E-cigarettes versus Placebo- Adverse events, Baseline, 3 months, 12 months 49](#_Toc34436084)

[E-cigarettes versus Placebo- Serious adverse events, 12 months 50](#_Toc34436085)

[E-cigarettes versus Placebo- Weight gain, Follow-up unclear/NR 50](#_Toc34436086)

[Livingstone-Banks 2019 {1077} 51](#_Toc34436087)

[Non-tailored print-based self-help materials (no face-to-face contact) versus No materials/no intervention- Abstinence/cessation, 6+ months 51](#_Toc34436088)

[Non-tailored print-based self-help (no face-to-face contact) versus No materials/no interventions- Abstinence/cessation, 6 months 52](#_Toc34436089)

[Non-tailored print-based self-help materials (no face-to-face contact) versus Brief leaflet- Abstinence/cessation, 6+ months 52](#_Toc34436090)

[Non-tailored print-based self-help materials (with face-to-face contact) versus No treatment or leaflet only- Abstinence/cessation, 6+ months 53](#_Toc34436091)

[Individually tailored print-based self-help materials (no face-to-face contact) versus No materials/ no interventions- Abstinence/cessation- 6+ months 53](#_Toc34436092)

[Matkin 2019 {1228} 54](#_Toc34436093)

[Hotline and self-help materials versus Minimal intervention- Abstinence/cessation, 12-18 months 54](#_Toc34436094)

[Intense telephone counselling versus Minimal intervention- Abstinence/cessation, 6+ months 54](#_Toc34436095)

[Brief motivational telephone counselling versus Usual care telephone call- Abstinence/cessation- 12 months 54](#_Toc34436096)

[Telephone counselling for smoking reduction versus Usual care telephone call- Abstinence/cessation, 12 months 55](#_Toc34436097)

[Posadzki 2016 {659} 56](#_Toc34436098)

[Interactive voice response (IVR) systems versus No intervention- Abstinence/cessation, 24 months 56](#_Toc34436099)

[Stead 2013 {1998} 57](#_Toc34436100)

[Physician advice (minimal or intensive interventions) versus No advice (or usual care)- Abstinence/cessation, 6+ months 57](#_Toc34436101)

[Physician advice with follow-up versus Minimal intervention /advice with single visit- Abstinence/cessation, 6+ months 58](#_Toc34436102)

[Intensive advice versus Minimal advice- Abstinence/cessation, 6+ months 59](#_Toc34436103)

[Stead 2016 {1356} 60](#_Toc34436104)

[Combined pharmacotherapy and behavioural interventions versus Usual care or minimal intervention- Abstinence/cessation, 6+ months 60](#_Toc34436105)

[Combined pharmacotherapy and behavioural interventions versus Usual care or no intervention- Abstinence/cessation, 12 months 62](#_Toc34436106)

[Stead 2017 {538} 63](#_Toc34436107)

[Group therapy versus No intervention- Abstinence/cessation, 6+ months 63](#_Toc34436108)

[Taylor 2017 {411} 64](#_Toc34436109)

[Interactive and tailored internet intervention versus Non-active control- Abstinence/cessation, 6-12 months 64](#_Toc34436110)

[Internet plus behavioural support versus Non-internet-based non-active control- Abstinence/cessation, 6-12 months 64](#_Toc34436111)

[Tsoi 2013 {1698} 65](#_Toc34436112)

[Bupropion versus Placebo- Abstinence/cessation, 6 months 65](#_Toc34436113)

[Bupropion versus Placebo- Reduction in number of cigarettes per day from baseline, 6 months 65](#_Toc34436114)

[Bupropion versus Placebo- Tobacco smoking reduction- Expired CO level, 6 months 65](#_Toc34436115)

[Bupropion versus Placebo- Change in emotional state (positive symptoms), End of treatment 66](#_Toc34436116)

[Bupropion versus Placebo- Change in emotional state (negative symptoms), End of treatmen 66](#_Toc34436117)

[Bupropion versus Placebo- Changes in emotional state (depressive symptoms), End of treatment 66](#_Toc34436118)

[Bupropion versus Placebo- Adverse events, Follow-up unclear/NR 67](#_Toc34436119)

[Bupropion (reduction trials only) versus Placebo- Change in emotional state (positive, negative, and psychiatric symptoms), End of treatment 67](#_Toc34436120)

[Bupropion (reduction trials only) versus Placebo- Adverse events, Follow-up unclear/NR 68](#_Toc34436121)

[Varenicline versus Placebo- Abstinence/cessation, 6 months 68](#_Toc34436122)

[Varenicline versus Placebo- Reduction in number of cigarettes per day from baseline, 6 months 68](#_Toc34436123)

[Varenicline versus Placebo- Changes in emotional state (positive, negative and depressive symptoms), End of treatment 69](#_Toc34436124)

[Varenicline versus Placebo- Adverse events, Follow-up unclear/NR 69](#_Toc34436125)

[Varenicline versus Placebo (trials with primary aim other than smoking cessation, reduction, and relapse)- Adverse events (including serious adverse events), Follow-up unclear/NR 69](#_Toc34436126)

[Varenicline versus Placebo (trials with primary aim other than smoking cessation, reduction and relapse)- Mental state, Follow-up unclear/NR 70](#_Toc34436127)

[NRT patch versus Placebo- Change in mental state and adverse events, Follow-up unclear/NR 70](#_Toc34436128)

[NRT patch versus Placebo- Adverse events, Follow-up unclear/NR 70](#_Toc34436129)

[Individual smoking cessation intervention (cognitive behavioural therapy and motivational interviewing) plus NRT patch versus Routine care- Abstinence/cessation, 6 months, 12 months, 4 years 71](#_Toc34436130)

[Individual smoking cessation intervention (cognitive behavioural therapy and motivational interviewing) plus NRT patch versus Routine care- Reduction in cigarettes/day of >50% of baseline, 6 months, 12 months and 4 year follow-up 71](#_Toc34436131)

[Van der Meer 2013 {1223} 72](#_Toc34436132)

[Bupropion versus Placebo (current depression)- Abstinence/cessation, 6-12 months 72](#_Toc34436133)

[Bupropion versus Placebo (past depression)- Abstinence/cessation, 6-12 months 72](#_Toc34436134)

[NRT gum versus Placebo (current depression)- Abstinence/cessation, 12 months 73](#_Toc34436135)

[NRT versus Placebo (past depression)- Abstinence/cessation, 6+ months 73](#_Toc34436136)

[Standard treatment plus extended NRT and extended CBT versus Standard treatment (past depression)- Abstinence/cessation, Follow-up unclear/NR 73](#_Toc34436137)

[Vodoplivec-Jamsek 2012 {1343} 74](#_Toc34436138)

[Mobile phone short message service versus Control- Abstinence/cessation, 6 months 74](#_Toc34436139)

[Mobile phone short message service versus Control - Adverse events (rates of car crash), 6 months 74](#_Toc34436140)

[Mobile phone short message service versus Control - Adverse events (pain in thumb/finger joint), 6 months 75](#_Toc34436141)

[White 2014 {1618} 76](#_Toc34436142)

[Acupuncture versus Sham acupuncture- Abstinence/cessation, 6-12 months 76](#_Toc34436143)

[Acupuncture versus Waiting list/no intervention- Abstinence/cessation, 6-12 months 76](#_Toc34436144)

[Continuous auricular stimulation versus Sham stimulation- Abstinence/cessation, 6-12 months 77](#_Toc34436145)

[Laser therapy versus Sham laser- Abstinence/cessation, 6-12 months 77](#_Toc34436146)

[Electrostimulation versus Sham electrostimulation- Abstinence/cessation, 6-12 months 77](#_Toc34436147)

[Whittaker 2016 {1803} 78](#_Toc34436148)

[Mobile-phone based intervention versus Usual care- Abstinence/cessation, 6+ months 78](#_Toc34436149)

#

# Barnes 2019 {3836}

## Hypnotherapy alone versus Placebo- Abstinence/cessation, 12 months

|  | **Selection bias - randomization** | **Selection bias - allocation** | **Blinding** | **Attrition bias** | **Selective reporting** | **Verification of smoking cessation** | **Baseline imbalance** | **Weight** |
| --- | --- | --- | --- | --- | --- | --- | --- | --- |
| **Fee 1977** |  |  |  |  |  |  |  | 100% |

Colour coding identification:

|  | Some concerns |
| --- | --- |
|  | Low risk of bias |
|  | High risk of bias |

# Cahill 2010 {1652}

## Stage-based expert systems or tailored self-help materials versus Assessment only- Abstinence/cessation, 6+ months

|  | **Selection bias - randomization** | **Selection bias - allocation** | **Blinding** | **Attrition** | **Other bias** | **Weight (%)** |
| --- | --- | --- | --- | --- | --- | --- |
| **Curry 1995** |  |  |  |  |  | 1.7 |
| **Dijkstra 1998** |  |  |  |  |  | 1.5 |
| **Etter 2004** |  |  |  |  |  | 37.8 |
| **Hollis 2005** |  |  |  |  |  | 6.1 |
| **Meyer 2008** |  |  |  |  |  | 1.1 |
| **Prochaska 2001a** |  |  |  |  |  | 21.9 |
| **Prochaska 2001b** |  |  |  |  |  | 4 |
| **Prochaska 2004** |  |  |  |  |  | 4.3 |
| **Prochaska 2005** |  |  |  |  |  | 17.7 |
| **Schumann 2006** |  |  |  |  |  | 3.9 |

## Stage-based expert systems or tailored self-help materials versus Assessment only- Abstinence/cessation, 14 months

|  | **Selection bias - randomization** | **Selection bias - allocation** | **Blinding** | **Attrition** | **Other bias** |
| --- | --- | --- | --- | --- | --- |
| **Dijkstra 1998** |  |  |  |  |  |

## Stage-based interactive computer programmes versus Usual care- Abstinence/cessation, 12+ months

|  | **Selection bias - randomization** | **Selection bias - allocation** | **Blinding** | **Attrition** | **Other bias** | **Weight (%)** |
| --- | --- | --- | --- | --- | --- | --- |
| **Aveyard 1999** |  |  |  |  |  | 78.5 |
| **Lawrence 2005** |  |  |  |  |  | 21.5 |

## Stage-based telephone counselling versus Usual Care- Abstinence/cessation, 12 months

|  | **Selection bias - randomization** | **Selection bias - allocation** | **Blinding** | **Attrition** | **Other bias** | **Weight (%)** |
| --- | --- | --- | --- | --- | --- | --- |
| **Young 2008** |  |  |  |  |  | 100 |

## Stage-based individual counselling and/or advice versus Usual care- Abstinence/cessation, 6+ months

|  | **Selection bias - randomization** | **Selection bias - allocation** | **Blinding** | **Attrition** | **Other bias** | **Weight (%)** |
| --- | --- | --- | --- | --- | --- | --- |
| **Bobo 1998** |  |  |  |  |  | 8.9 |
| **Chouinard 2005** |  |  |  |  |  | 5.1 |
| **Davies 2005** |  |  |  |  |  | 4 |
| **Lawrence 2005** |  |  |  |  |  | 6.9 |
| **Mermelstein 2003** |  |  |  |  |  | 60.7 |
| **Pieterse 2001** |  |  |  | NA |  | 4.5 |
| **Prokhorov 2008** |  |  |  |  |  | 9.8 |

NA= Not assessed

## Stage-based individual counselling or advice versus Assessment only- Abstinence/cessation, 6+ months

|  | **Selection bias - randomization** | **Selection bias - allocation** | **Blinding** | **Attrition** | **Other bias** | **Weight (%)** |
| --- | --- | --- | --- | --- | --- | --- |
| **Manfredi 2004** |  |  |  |  |  | 88.2 |
| **Meyer 2008** |  |  |  |  |  | 5.8 |
| **Nakamura 2004** |  |  |  |  |  | 6 |

# Cahill 2016 {1960}

## Cystisine versus Placebo- Abstinence/cessation (continuous), 6+ months

|  | **Selection bias - randomization** | **Selection bias - allocation** | **Blinding** | **Attrition** | **Selective reporting** | **Other bias** | **Weight (%)** |
| --- | --- | --- | --- | --- | --- | --- | --- |
| **Vinnikov 2008** |  |  |  |  |  | NA | 10.1 |
| **West 2011** |  |  |  |  |  | NA | 89.9 |

NA= Not assessed

## Cytisine versus Placebo- Abstinence/cessation (point prevalence), 2 years

|  | **Selection bias - randomization** | **Selection bias - allocation** | **Blinding** | **Attrition** | **Selective reporting** | **Other bias** | **Weight (%)** |
| --- | --- | --- | --- | --- | --- | --- | --- |
| **Scharfenberg 1971** |  |  |  |  |  | NA | 100 |

NA= Not assessed

## Cytisine versus Placebo- Adverse events, Follow-up unclear/NR

|  | **Selection bias - randomization** | **Selection bias - allocation** | **Blinding** | **Attrition** | **Selective reporting** | **Other bias** |
| --- | --- | --- | --- | --- | --- | --- |
| **Scharfenberg 1971** |  |  |  |  |  | NA |
| **Vinnikov 2008** |  |  |  |  |  | NA |
| **West 2011** |  |  |  |  |  | NA |

NA= Not assessed

## Varenicline versus Placebo- Abstinence/cessation, longest follow-up (6+ months)

|  | **Selection bias - randomization** | **Selection bias - allocation** | **Blinding** | **Attrition** | **Selective reporting** | **Other bias** | **Weight (%)** |
| --- | --- | --- | --- | --- | --- | --- | --- |
| **De Dios 2012** |  |  |  |  |  |  | 0.1 |
| **Nahvi 2014a** |  |  |  |  |  |  | 0.1 |
| **Chengappa 2014** |  |  |  |  |  |  | 0.3 |
| **Westergaard 2015** |  |  |  |  |  |  | 0.6 |
| **Evins 2014** |  |  |  |  |  |  | 0.7 |
| **Heydari 2012** |  |  |  |  |  |  | 0.9 |
| **Nides 2006** |  |  |  |  |  |  | 0.9 |
| **Oncken 2006** |  |  |  |  |  |  | 1 |
| **Gonzales 2014** |  |  |  |  |  |  | 1.2 |
| **NCT00828113** |  |  |  |  |  | NA | 1.6 |
| **Steinberg 2011** |  |  |  |  |  |  | 1.6 |
| **Niaura 2008** |  |  |  |  |  |  | 1.7 |
| **Cinciripini 2013** |  |  |  |  |  |  | 2 |
| **Tashkin 2011** |  |  |  |  |  |  | 2 |
| **Rigotti 2010** |  |  |  |  |  |  | 3.8 |
| **Anthenelli 2013** |  |  |  |  |  |  | 3.9 |
| **Tsai 2007** |  |  |  |  |  |  | 3.9 |
| **Gonzales 2006** |  |  |  |  |  |  | 4.3 |
| **Rennard 2012** |  |  |  |  |  |  | 4.6 |
| **Bolliger 2011** |  |  |  |  |  | NA | 5 |
| **Nakamura 2007** |  |  |  |  |  |  | 5.1 |
| **Jorenby 2006** |  |  |  |  |  |  | 5.1 |
| **Wong 2012** |  |  |  |  |  |  | 5.2 |
| **Carson 2014** |  |  |  |  |  |  | 5.2 |
| **Eisenberg 2016** |  |  |  |  |  |  | 5.6 |
| **Wang 2009** |  |  |  |  |  |  | 6 |
| **EAGLES 2016** |  |  |  |  |  |  | 27.7 |

NA= Not assessed

## Varenicline versus Placebo- Abstinence/cessation, 6 months

|  | **Selection bias - randomization** | **Selection bias - allocation** | **Blinding** | **Attrition** | **Selective reporting** | **Other bias** | **Weight (%)** |
| --- | --- | --- | --- | --- | --- | --- | --- |
| **Anthenelli 2013** |  |  |  |  |  |  | 4.3 |
| **Bolliger 2011** |  |  |  |  |  | NA | 4.6 |
| **Carson 2014** |  |  |  |  |  |  | 7.2 |
| **Chengappa 2014** |  |  |  |  |  |  | 0.3 |
| **Cinciripini 2013** |  |  |  |  |  |  | 1.8 |
| **De Dios 2012** |  |  |  |  |  |  | 0.1 |
| **EAGLES 2016** |  |  |  |  |  |  | 25.5 |
| **Eisenberg 2016** |  |  |  |  |  |  | 5.2 |
| **Gonzales 2006** |  |  |  |  |  |  | 4.9 |
| **Gonzales 2014** |  |  |  |  |  |  | 2.6 |
| **Jorenby 2006** |  |  |  |  |  |  | 6 |
| **Nahvi 2014a** |  |  |  |  |  |  | 0.1 |
| **Nakamura 2007** |  |  |  |  |  |  | 5.9 |
| **NCT01347112** |  |  |  |  |  |  | 0.1 |
| **Niaura 2008** |  |  |  |  |  |  | 1.9 |
| **Nides 2006** |  |  |  |  |  |  | 1.2 |
| **Oncken 2006** |  |  |  |  |  |  | 1.2 |
| **Rennard 2012** |  |  |  |  |  |  | 4.2 |
| **Rigotti 2010** |  |  |  |  |  |  | 4.5 |
| **Steinberg 2011** |  |  |  |  |  |  | 1.5 |
| **Tashkin 2011** |  |  |  |  |  |  | 2.4 |
| **Tsai 2007** |  |  |  |  |  |  | 3.6 |
| **Wang 2009** |  |  |  |  |  |  | 5.6 |
| **Westergaard 2015** |  |  |  |  |  |  | 0.5 |
| **Wong 2012** |  |  |  |  |  |  | 4.9 |

NA= Not assessed

## Long term varenicline versus Placebo- Abstinence/cessation, 6-12 months

|  | **Selection bias - randomization** | **Selection bias - allocation** | **Blinding** | **Attrition** | **Selective reporting** | **Other bias** | **Weight (%)** |
| --- | --- | --- | --- | --- | --- | --- | --- |
| **Ebbert 2015** |  |  |  |  |  |  | 65.7 |
| **NCT00828113** |  |  |  |  |  | NA | 15.8 |
| **Stein 2013** |  |  |  |  |  |  | 1.1 |
| **Williams 2007** |  |  |  |  |  |  | 17.4 |

NA= Not assessed

## Low dose varenicline versus Placebo- Abstinence/cessation- 12 months

|  | **Selection bias - randomization** | **Selection bias - allocation** | **Blinding** | **Attrition** | **Selective reporting** | **Other bias** | **Weight (%)** |
| --- | --- | --- | --- | --- | --- | --- | --- |
| **Nakamura 2007** |  |  |  |  |  |  | 58.7 |
| **Niaura 2008** |  |  |  |  |  |  | 20 |
| **Nides 2006** |  |  |  |  |  |  | 10.1 |
| **Oncken 2006** |  |  |  |  |  |  | 11.2 |

## Variable dosing of varenicline versus Placebo- Abstinence/cessation, 12 months

|  | **Selection bias - randomization** | **Selection bias - allocation** | **Blinding** | **Attrition** | **Selective reporting** | **Other bias** | **Weight (%)** |
| --- | --- | --- | --- | --- | --- | --- | --- |
| **Anthenelli 2013** |  |  |  |  |  |  | 31.7 |
| **Chengappa 2014** |  |  |  |  |  |  | 2.4 |
| **Cinciripini 2013** |  |  |  |  |  |  | 15.7 |
| **Gonzales 2014** |  |  |  |  |  |  | 9.4 |
| **Hajek 2015** |  |  |  |  |  |  | 26.8 |
| **Niaura 2008** |  |  |  |  |  |  | 14 |

## Varenicline versus Placebo- Abstinence/cessation in smokers reducing to quit, 12 months

|  | **Selection bias - randomization** | **Selection bias - allocation** | **Blinding** | **Attrition** | **Selective reporting** | **Other bias** | **Weight (%)** |
| --- | --- | --- | --- | --- | --- | --- | --- |
| **Ebbert 2015** |  |  |  |  |  |  | 100 |

## Varenicline versus Placebo- Abstinence/cessation in smokers with schizophrenia, bipolar, or other psychiatric disorder, 6 months

|  | **Selection bias - randomization** | **Selection bias - allocation** | **Blinding** | **Attrition** | **Selective reporting** | **Other bias** | **Weight (%)** |
| --- | --- | --- | --- | --- | --- | --- | --- |
| **Chengappa 2014** |  |  |  |  |  |  | 2.2 |
| **EAGLES 2016** |  |  |  |  |  |  | 91.4 |
| **Evins 2014** |  |  |  |  |  |  | 4.9 |
| **Williams 2012** |  |  | NA |  | NA | NA | 1.4 |

NA= Not assessed

## Varenicline versus Placebo- Abstinence/cessation in smokers with depression and motivated/wishing to quit, 12 months

|  | **Selection bias - randomization** | **Selection bias - allocation** | **Blinding** | **Attrition** | **Selective reporting** | **Other bias** | **Weight (%)** |
| --- | --- | --- | --- | --- | --- | --- | --- |
| **Anthenelli 2013** |  |  |  |  |  |  | 100 |

## Varenicline versus Placebo- Abstinence/cessation in smokers who previously failed to quit on varenicline but are motivated/wishing to try quitting again, 12 months

|  | **Selection bias - randomization** | **Selection bias - allocation** | **Blinding** | **Attrition** | **Selective reporting** | **Other bias** | **Weight (%)** |
| --- | --- | --- | --- | --- | --- | --- | --- |
| **Gonzales 2014** |  |  |  |  |  |  | 100 |

## Varenicline versus Placebo- Adverse events (nausea), Range of follow-up times

|  | **Selection bias - randomization** | **Selection bias - allocation** | **Blinding** | **Attrition** | **Selective reporting** | **Other bias** | **Weight (%)** |
| --- | --- | --- | --- | --- | --- | --- | --- |
| **Anthenelli 2013** |  |  |  |  |  |  | 4.4 |
| **Bolliger 2011** |  |  |  |  |  | NA | 3.4 |
| **Carson 2014** |  |  |  |  |  |  | 0.5 |
| **Chengappa 2014** |  |  |  |  |  |  | 1.5 |
| **Cinciripini 2013** |  |  |  |  |  |  | 1.2 |
| **EAGLES 2016** |  |  |  |  |  |  | 22.1 |
| **Ebbert 2015** |  |  |  |  |  |  | 10.8 |
| **Eisenberg 2016** |  |  |  |  |  |  | 2.1 |
| **Evins 2014** |  |  |  |  |  |  | 1.5 |
| **Gonzales 2006** |  |  |  |  |  |  | 4.7 |
| **Gonzales 2014** |  |  |  |  |  |  | 3.6 |
| **Hajek 2015** |  |  |  |  |  |  | 2.9 |
| **Heydari 2012** |  |  |  |  |  |  | 0.1 |
| **Jorenby 2006** |  |  |  |  |  |  | 5.3 |
| **Nahvi 2014a** |  |  |  |  |  |  | 2.3 |
| **Nakamura 2007** |  |  |  |  |  |  | 1.9 |
| **NCT00828113** |  |  |  |  |  | NA | 0.1 |
| **NCT01347112** |  |  |  |  |  |  | 0.1 |
| **Niaura 2008** |  |  |  |  |  |  | 1.3 |
| **Nides 2006** |  |  |  |  |  |  | 3.7 |
| **Oncken 2006** |  |  |  |  |  |  | 3.9 |
| **Rennard 2012** |  |  |  |  |  |  | 3.6 |
| **Rigotti 2010** |  |  |  |  |  |  | 4.8 |
| **Stein 2013** |  |  |  |  |  |  | 0.5 |
| **Steinberg 2011** |  |  |  |  |  |  | 0.3 |
| **Tashkin 2011** |  |  |  |  |  |  | 3.2 |
| **Tasi 2007** |  |  |  |  |  |  | 2.3 |
| **Tonnensen 2013** |  |  |  |  |  |  | 1.3 |
| **Wang 2009** |  |  |  |  |  |  | 3.2 |
| **Williams 2007** |  |  |  |  |  |  | 2.1 |
| **Williams 2012** |  |  | NA |  | NA | NA | 0.4 |
| **Wong 2012** |  |  |  |  |  |  | 0.8 |

NA= Not assessed

## Varenicline versus Placebo- Adverse events (insomnia), Range of follow-up times

|  | **Selection bias - randomization** | **Selection bias - allocation** | **Blinding** | **Attrition** | **Selective reporting** | **Other bias** | **Weight (%)** |
| --- | --- | --- | --- | --- | --- | --- | --- |
| **Anthenelli 2013** |  |  |  |  |  |  | 2.2 |
| **Bolliger 2011** |  |  |  |  |  | NA | 2.9 |
| **Carson 2014** |  |  |  |  |  |  | 0.7 |
| **Chengappa 2014** |  |  |  |  |  |  | 1.4 |
| **Cinciripini 2013** |  |  |  |  |  |  | 3.2 |
| **EAGLES 2016** |  |  |  |  |  |  | 23.8 |
| **Ebbert 2015** |  |  |  |  |  |  | 8.8 |
| **Eisenberg 2016** |  |  |  |  |  |  | 3.2 |
| **Evins 2014** |  |  |  |  |  |  | 1.7 |
| **Gonzales 2006** |  |  |  |  |  |  | 7.6 |
| **Gonzales 2014** |  |  |  |  |  |  | 1.7 |
| **Hajek 2015** |  |  |  |  |  |  | 3.4 |
| **Heydari 2012** |  |  |  |  |  |  | 0.1 |
| **Jorenby 2006** |  |  |  |  |  |  | 7.2 |
| **Nahvi2014a** |  |  |  |  |  |  | 2.3 |
| **NCT01347112** |  |  |  |  |  |  | 0.1 |
| **Niaura 2008** |  |  |  |  |  |  | 2.9 |
| **Nides 2006** |  |  |  |  |  |  | 4.7 |
| **Oncken 2006** |  |  |  |  |  |  | 3.2 |
| **Rennard 2012** |  |  |  |  |  |  | 1.5 |
| **Rigotti 2010** |  |  |  |  |  |  | 3.9 |
| **Stein 2013** |  |  |  |  |  |  | 3.2 |
| **Steinberg 2011** |  |  |  |  |  |  | 0.5 |
| **Tashkin 2011** |  |  |  |  |  |  | 2.5 |
| **Tasi 2007** |  |  |  |  |  |  | 2.9 |
| **Wang 2009** |  |  |  |  |  |  | 0.8 |
| **Williams 2007** |  |  |  |  |  |  | 2.7 |
| **Williams 2012** |  |  | NA |  | NA | NA | 0.5 |
| **Wong 2012** |  |  |  |  |  |  | 0.2 |

NA= Not assessed

## Varenicline versus Placebo- Adverse events (abnormal dreams), Range of follow-up times

|  | **Selection bias - randomization** | **Selection bias - allocation** | **Blinding** | **Attrition** | **Selective reporting** | **Other bias** | **Weight (%)** |
| --- | --- | --- | --- | --- | --- | --- | --- |
| **Anthenelli 2013** |  |  |  |  |  |  | 2.2 |
| **Bolliger 2011** |  |  |  |  |  | NA | 2.9 |
| **Carson 2014** |  |  |  |  |  |  | 0.7 |
| **Chengappa 2014** |  |  |  |  |  |  | 1.4 |
| **Cinciripini 2013** |  |  |  |  |  |  | 3.2 |
| **EAGLES 2016** |  |  |  |  |  |  | 23.8 |
| **Ebbert 2015** |  |  |  |  |  |  | 8.8 |
| **Eisenberg 2016** |  |  |  |  |  |  | 3.2 |
| **Evins 2014** |  |  |  |  |  |  | 1.7 |
| **Gonzales 2006** |  |  |  |  |  |  | 7.6 |
| **Gonzales 2014** |  |  |  |  |  |  | 1.7 |
| **Hajek 2015** |  |  |  |  |  |  | 3.4 |
| **Jorenby 2006** |  |  |  |  |  |  | 7.2 |
| **Nahvi2014a** |  |  |  |  |  |  | 2.3 |
| **NCT01347112** |  |  |  |  |  |  | 0.1 |
| **Nides 2006** |  |  |  |  |  |  | 4.7 |
| **Oncken 2006** |  |  |  |  |  |  | 3.2 |
| **Rennard 2012** |  |  |  |  |  |  | 1.5 |
| **Rigotti 2010** |  |  |  |  |  |  | 3.9 |
| **Stein 2013** |  |  |  |  |  |  | 3.2 |
| **Tashkin 2011** |  |  |  |  |  |  | 2.5 |
| **Tsai 2007** |  |  |  |  |  |  | 2.9 |
| **Tonnensen 2013** |  |  |  |  |  |  | 6.8 |
| **Williams 2007** |  |  |  |  |  |  | 2.7 |
| **Williams 2012** |  |  | NA |  | NA | NA | 0.5 |
| **Wong 2012** |  |  |  |  |  |  | 0.2 |

NA= Not assessed

## Varenicline versus Placebo- Adverse events (headache), Range of follow-up times

|  | **Selection bias - randomization** | **Selection bias - allocation** | **Blinding** | **Attrition** | **Selective reporting** | **Other bias** | **Weight (%)** |
| --- | --- | --- | --- | --- | --- | --- | --- |
| **Anthenelli 2013** |  |  |  |  |  |  | 4.2 |
| **Bolliger 2011** |  |  |  |  |  | NA | 4.6 |
| **Carson 2014** |  |  |  |  |  |  | 0.4 |
| **Chengappa 2014** |  |  |  |  |  |  | 1.8 |
| **Cinciripini 2013** |  |  |  |  |  |  | 1.5 |
| **EAGLES 2016** |  |  |  |  |  |  | 28.5 |
| **Ebbert 2015** |  |  |  |  |  |  | 7.8 |
| **Eisenberg 2016** |  |  |  |  |  |  | 1.7 |
| **Evins 2014** |  |  |  |  |  |  | 1.5 |
| **Gonzales 2006** |  |  |  |  |  |  | 6.1 |
| **Gonzales 2014** |  |  |  |  |  |  | 3.5 |
| **Hajek 2015** |  |  |  |  |  |  | 0.9 |
| **Jorenby 2006** |  |  |  |  |  |  | 6.2 |
| **Nahvi2014a** |  |  |  |  |  |  | 2.6 |
| **Nakamura 2007** |  |  |  |  |  |  | 0.6 |
| **Niaura 2008** |  |  |  |  |  |  | 2.9 |
| **Nides 2006** |  |  |  |  |  |  | 4.8 |
| **Oncken 2006** |  |  |  |  |  |  | 4.1 |
| **Rennard 2012** |  |  |  |  |  |  | 4.3 |
| **Rigotti 2010** |  |  |  |  |  |  | 5.6 |
| **Stein 2013** |  |  |  |  |  |  | 1.3 |
| **Tashkin 2011** |  |  |  |  |  |  | 2.8 |
| **Wang 2009** |  |  |  |  |  |  | 1 |
| **Williams 2012** |  |  | NA |  | NA | NA | 1.5 |
| **Wong 2012** |  |  |  |  |  |  | 0.1 |

NA= Not assessed

## Varenicline versus Placebo- Adverse events (depression), Range of follow-up times

|  | **Selection bias - randomization** | **Selection bias - allocation** | **Blinding** | **Attrition** | **Selective reporting** | **Other bias** | **Weight (%)** |
| --- | --- | --- | --- | --- | --- | --- | --- |
| **Anthenelli 2013** |  |  |  |  |  |  | 6.5 |
| **Bolliger 2011** |  |  |  |  |  | NA | 2.7 |
| ***Brandon 2011**** |  |  |  |  |  |  | not estimable |
| **Carson 2014** |  |  |  |  |  |  | 1 |
| **Chengappa 2014** |  |  |  |  |  |  | 1.1 |
| **Cinciripini 2013** |  |  |  |  |  |  | 6.4 |
| **EAGLES 2016** |  |  |  |  |  |  | 3.1 |
| ***Ebbert 2011**** |  |  |  |  |  |  | 0.8 |
| **Ebbert 2015** |  |  |  |  |  |  | 18.1 |
| **Eisenberg 2016** |  |  |  |  |  |  | 0.3 |
| **Evins 2014** |  |  |  |  |  |  | 0.5 |
| ***Faessel 2009**** |  |  |  |  |  |  | not estimable |
| ***Fagerstrom 2010**** |  |  |  |  |  |  | 2.5 |
| ***Garza 2011**** |  |  |  |  |  |  | 0.5 |
| **Gonzales 2006** |  |  |  |  |  |  | 3.6 |
| **Gonzales 2014** |  |  |  |  |  |  | 1 |
| **Hajek 2015** |  |  |  |  |  |  | 4.1 |
| ***Hughes 2011**** |  |  |  |  |  |  | 1 |
| **Jorenby 2006** |  |  |  |  |  |  | 0.5 |
| ***McClure 2013**** |  |  |  |  |  |  | 10.1 |
| ***Meszaros 2013**** |  |  |  |  |  |  | 0.5 |
| **Nahvi2014a** |  |  |  |  |  |  | 0.5 |
| **NCT01347112** |  |  |  |  |  |  | 0.7 |
| **Niaura 2008** |  |  |  |  |  |  | 1 |
| **Nides 2006** |  |  |  |  |  |  | 0.5 |
| **Oncken 2006** |  |  |  |  |  |  | 2.8 |
| **Rennard 2012** |  |  |  |  |  |  | 3.8 |
| **Rigotti 2010** |  |  |  |  |  |  | 1.5 |
| **Stein 2013** |  |  |  |  |  |  | 5.5 |
| **Steinberg 2011** |  |  |  |  |  |  | 1 |
| **Tashkin 2011** |  |  |  |  |  |  | 2.5 |
| **Tonstad 2006** |  |  |  |  |  |  | 8.7 |
| **Tsai 2007** |  |  |  |  |  |  | 1 |
| **Williams 2007** |  |  |  |  |  |  | 2.7 |
| **Williams 2012** |  |  | NA |  | NA | NA | 2 |
| **Wong 2012** |  |  |  |  |  |  | 1.1 |

NA= Not assessed

Italicized* and greyed out= Studies which were originally excluded from the original review but had since been included by review authors in order to utilize their neuropsychiatric adverse events data. These studies do not contribute to efficacy data; and risk of bias assessments were not performed.

## Varenicline versus Placebo- Adverse events (suicidal ideation), Range of follow-up times

|  | **Selection bias - randomization** | **Selection bias - allocation** | **Blinding** | **Attrition** | **Selective reporting** | **Other bias** | **Weight (%)** |
| --- | --- | --- | --- | --- | --- | --- | --- |
| **Anthenelli 2013** |  |  |  |  |  |  | 7.9 |
| **Bolliger 2011** |  |  |  |  |  | NA | 1.5 |
| ***Brandon 2011**** |  |  |  |  |  |  | not estimable |
| **Carson 2014** |  |  |  |  |  |  | not estimable |
| **Chengappa 2014** |  |  |  |  |  |  | 2.4 |
| **Cinciripini 2013** |  |  |  |  |  |  | 3.1 |
| **EAGLES 2016** |  |  |  |  |  |  | 11.6 |
| ***Ebbert 2011**** |  |  |  |  |  |  | 0.8 |
| **Ebbert 2015** |  |  |  |  |  |  | 23.3 |
| **Evins 2014** |  |  |  |  |  |  | 4.3 |
| **Gonzales 2014** |  |  |  |  |  |  | not estimable |
| **Hajek 2015** |  |  |  |  |  |  | not estimable |
| ***Hughes 2011**** |  |  |  |  |  |  | 1 |
| **Jorenby 2006** |  |  |  |  |  |  | 3.5 |
| ***McClure 2013**** |  |  |  |  |  |  | 10.1 |
| ***Meszaros 2013**** |  |  |  |  |  |  | 0.5 |
| ***Mitchell 2012**** |  |  |  |  |  |  | 3.6 |
| **Nahvi 2014a** |  |  |  |  |  |  | 9.4 |
| **Rennard 2012** |  |  |  |  |  |  | 6.9 |
| **Stein 2013** |  |  |  |  |  |  | 1.8 |
| **Steinberg 2011** |  |  |  |  |  |  | not estimable |
| **Tashkin 2011** |  |  |  |  |  |  | 3.5 |
| **Williams 2012** |  |  | NA |  | NA | NA | 9.2 |
| **Wong 2012** |  |  |  |  |  |  | not estimable |

NA= Not assessed

Italicized* and greyed out= Studies which were originally excluded from the original review but had since been included by review authors in order to utilize their neuropsychiatric adverse events data. These studies do not contribute to efficacy data; and risk of bias assessments were not performed.

## Varenicline versus Placebo- Serious adverse event (at least one), Range of follow-up times

|  | **Selection bias - randomization** | **Selection bias - allocation** | **Blinding** | **Attrition** | **Selective reporting** | **Other bias** | **Weight (%)** |
| --- | --- | --- | --- | --- | --- | --- | --- |
| **Anthenelli 2013** |  |  |  |  |  |  | 3.9 |
| **Bolliger 2011** |  |  |  |  |  | NA | 1.3 |
| **Carson 2014** |  |  |  |  |  |  | 4 |
| **Chengappa 2014** |  |  |  |  |  |  | 2 |
| **Cinciripini 2013** |  |  |  |  |  |  | 0.9 |
| **EAGLES 2016** |  |  |  |  |  |  | 20.3 |
| **Ebbert 2015** |  |  |  |  |  |  | 8 |
| **Eisenberg 2016** |  |  |  |  |  |  | 8.4 |
| **Evins 2014** |  |  |  |  |  |  | 3.2 |
| **Gonzales 2006** |  |  |  |  |  |  | 4.5 |
| **Gonzales 2014** |  |  |  |  |  |  | 2 |
| **Jorenby 2006** |  |  |  |  |  |  | 3 |
| **Nahvi 2014a** |  |  |  |  |  |  | 1.5 |
| **Nakamura 2007** |  |  |  |  |  |  | 1.5 |
| **NCT00828113** |  |  |  |  |  | NA | 0.5 |
| **Niaura 2008** |  |  |  |  |  |  | 0.2 |
| **Nides 2006** |  |  |  |  |  |  | 0.2 |
| **Oncken 2006** |  |  |  |  |  |  | 1.3 |
| **Rennard 2012** |  |  |  |  |  |  | 0.7 |
| **Rigotti 2010** |  |  |  |  |  |  | 10.4 |
| **Stein 2013** |  |  |  |  |  |  | 0.4 |
| **Steinberg 2011** |  |  |  |  |  |  | 2.5 |
| **Tashkin 2011** |  |  |  |  |  |  | 7.4 |
| **Tonstad 2006** |  |  |  |  |  |  | 2.5 |
| **Tsai 2007** |  |  |  |  |  |  | 1.5 |
| **Tonnensen 2013** |  |  |  |  |  |  | 2 |
| **Wang 2009** |  |  |  |  |  |  | 1.2 |
| **Williams 2007** |  |  |  |  |  |  | 2 |
| **Williams 2012** |  |  | NA |  | NA | NA | 2.6 |

## Varenicline versus Placebo- Serious adverse event (at least one during or immediately after treatment), Range of follow-up times

|  | **Selection bias - randomization** | **Selection bias - allocation** | **Blinding** | **Attrition** | **Selective reporting** | **Other bias** | **Weight (%)** |
| --- | --- | --- | --- | --- | --- | --- | --- |
| **Anthenelli 2013** |  |  |  |  |  |  | 4 |
| **Bolliger 2011** |  |  |  |  |  | NA | 1.6 |
| **Carson 2014** |  |  |  |  |  |  | 1.8 |
| **Chengappa 2014** |  |  |  |  |  |  | 1.8 |
| **Cinciripini 2013** |  |  |  |  |  |  | 1.1 |
| **EAGLES 2016** |  |  |  |  |  |  | 24.3 |
| **Ebbert 2015** |  |  |  |  |  |  | 6 |
| **Eisenberg 2016** |  |  |  |  |  |  | 10.1 |
| **Gonzales 2006** |  |  |  |  |  |  | 4.2 |
| **Gonzales 2014** |  |  |  |  |  |  | 2.4 |
| **Jorenby 2006** |  |  |  |  |  |  | 3 |
| **Nahvi 2014a** |  |  |  |  |  |  | 1.2 |
| **Nakamura 2007** |  |  |  |  |  |  | 1.8 |
| **NCT00828113** |  |  |  |  |  | NA | 0.9 |
| **Niaura 2008** |  |  |  |  |  |  | 0.3 |
| **Nides 2006** |  |  |  |  |  |  | 0.3 |
| **Oncken 2006** |  |  |  |  |  |  | 0.8 |
| **Rennard 2012** |  |  |  |  |  |  | 0.9 |
| **Rigotti 2010** |  |  |  |  |  |  | 12.4 |
| **Steinberg 2011** |  |  |  |  |  |  | 3 |
| **Tashkin 2011** |  |  |  |  |  |  | 6.5 |
| **Tonstad 2006** |  |  |  |  |  |  | 3 |
| **Tsai 2007** |  |  |  |  |  |  | 1.8 |
| **Wang 2009** |  |  |  |  |  |  | 1.5 |
| **Williams 2007** |  |  |  |  |  |  | 2.4 |
| **Williams 2012** |  |  | NA |  | NA | NA | 3.1 |

NA= Not assessed

## Varenicline versus Placebo- Neuropsychiatric events (depression, suicidal ideation), not deaths, Range of follow-up times

|  | **Selection bias - randomization** | **Selection bias - allocation** | **Blinding** | **Attrition** | **Selective reporting** | **Other bias** | **Weight (%)** |
| --- | --- | --- | --- | --- | --- | --- | --- |
| **Anthenelli 2013** |  |  |  |  |  |  | 7.8 |
| **Bolliger 2011** |  |  |  |  |  | NA | 1.3 |
| **Carson 2014** |  |  |  |  |  |  | 6.1 |
| **Chengappa 2014** |  |  |  |  |  |  | 1 |
| **Eisenberg 2016** |  |  |  |  |  |  | 1 |
| **Evins 2014** |  |  |  |  |  |  | 9.2 |
| **Gonzales 2006** |  |  |  |  |  |  | 3 |
| **Jorenby 2006** |  |  |  |  |  |  | 1 |
| **Nahvi2014a** |  |  |  |  |  |  | 51.1 |
| **Nakamura 2007** |  |  |  |  |  |  | not estimable |
| **Niaura 2008** |  |  |  |  |  |  | not estimable |
| **Nides 2006** |  |  |  |  |  |  | not estimable |
| **Oncken 2006** |  |  |  |  |  |  | 4 |
| **Rennard 2012** |  |  |  |  |  |  | 4.5 |
| **Rigotti 2010** |  |  |  |  |  |  | not estimable |
| **Stein 2013** |  |  |  |  |  |  | 1.5 |
| **Steinberg 2011** |  |  |  |  |  |  | not estimable |
| **Tashkin 2011** |  |  |  |  |  |  | 3 |
| **Tonstad 2011** |  |  |  |  |  | NA | not estimable |
| **Tsai 2007** |  |  |  |  |  |  | not estimable |
| **Wang 2009** |  |  |  |  |  |  | not estimable |
| **Williams 2007** |  |  |  |  |  |  | not estimable |
| **Williams 2012** |  |  | NA |  | NA | NA | 5.3 |

NA= Not assessed

## Varenicline versus Placebo- Serious adverse events (cardiac, including deaths), Range of follow-up times

|  | **Selection bias - randomization** | **Selection bias - allocation** | **Blinding** | **Attrition** | **Selective reporting** | **Other bias** | **Weight (%)** |
| --- | --- | --- | --- | --- | --- | --- | --- |
| **Bolliger 2011** |  |  |  |  |  | NA | 1.6 |
| **Carson 2014** |  |  |  |  |  |  | 12.2 |
| **Chengappa 2014** |  |  |  |  |  |  | 3.7 |
| **Cinciripini 2013** |  |  |  |  |  |  | 2.2 |
| **Eisenberg 2016** |  |  |  |  |  |  | 24.1 |
| **Evins 2014** |  |  |  |  |  |  | 3.3 |
| **Gonzales 2006** |  |  |  |  |  |  | 9.8 |
| **Gonzales 2014** |  |  |  |  |  |  | 2.4 |
| **Jorenby 2006** |  |  |  |  |  |  | 2.4 |
| **Nakamura 2007** |  |  |  |  |  |  | 2.4 |
| **Niaura 2008** |  |  |  |  |  |  | 1.2 |
| **Nides 2006** |  |  |  |  |  |  | 1.2 |
| **Oncken 2006** |  |  |  |  |  |  | 1.6 |
| **Rennard 2012** |  |  |  |  |  |  | 1.8 |
| **Rigotti 2010** |  |  |  |  |  |  | 9.6 |
| **Tashkin 2011** |  |  |  |  |  |  | 11.9 |
| **Tonstad 2011** |  |  |  |  |  | NA | 1.2 |
| **Tasi 2007** |  |  |  |  |  |  | 1.2 |
| **Tonnensen 2013** |  |  |  |  |  |  | 1.2 |
| **Williams 2007** |  |  |  |  |  |  | 3.2 |
| **Williams 2012** |  |  | NA |  | NA | NA | 1.6 |

NA= Not assessed

## Varenicline versus Placebo- Adverse events (discontinuation), Range of follow-up times

|  | **Selection bias - randomization** | **Selection bias - allocation** | **Blinding** | **Attrition** | **Selective reporting** | **Other bias** | **Weight (%)** |
| --- | --- | --- | --- | --- | --- | --- | --- |
| **Evins 2014** |  |  |  |  |  |  | unclear data |
| **Gonzales 2006** |  |  |  |  |  |  | NR |
| **Jorenby 2006** |  |  |  |  |  |  | NR |
| **Williams 2007** |  |  |  |  |  |  | NR |

NR= Not reported

# Farley 2012 {1469}

## Buproprion versus Placebo- Weight gain, End of treatment

|  | **Selection bias - randomization** | **Selection bias - allocation** | **Performance and detection bias** | **Definition of abstinence** | **Weight (%)** |
| --- | --- | --- | --- | --- | --- |
| **Nides 2006** |  |  |  |  | 4.9 |
| **Hurt 1997** |  |  |  |  | 9.6 |
| **Piper 2007** |  |  |  |  | 4.2 |
| **Jorenby 2006** |  |  |  |  | 8.1 |
| **Rigotti 2006** |  |  |  |  | 3.1 |
| **Zellweger 2005** |  |  |  |  | 59 |
| **Gonzales 2006** |  |  |  |  | 11 |

## Buproprion versus Placebo- Weight gain, 6 months

|  | **Selection bias - randomization** | **Selection bias - allocation** | **Performance and detection bias** | **Definition of abstinence** | **Weight (%)** |
| --- | --- | --- | --- | --- | --- |
| **Uyar 2007** |  |  |  |  | 28 |
| **Simon 2009** |  |  |  |  | 0.6 |
| **Hurt 1997** |  |  |  |  | 10.5 |
| **Zellweger 2005** |  |  |  |  | 60.9 |

## Bupropion versus Placebo- Weight gain, 12 months

|  | **Selection bias - randomization** | **Selection bias - allocation** | **Performance and detection bias** | **Definition of abstinence** | **Weight (%)** |
| --- | --- | --- | --- | --- | --- |
| **Rigotti 2006** |  |  |  |  | 13.2 |
| **Zellweger 2005** |  |  |  |  | 57.1 |
| **Simon 2004** |  |  |  |  | 20.7 |
| **Hurt 1997** |  |  |  |  | 9 |

## NRT versus Placebo- Weight gain, End of treatment

|  | **Selection bias - randomization** | **Selection bias - allocation** | **Performance and detection bias** | **Definition of abstinence** | **Weight (%)** |
| --- | --- | --- | --- | --- | --- |
| **Cooper 2005** |  |  |  |  | 0.7 |
| **Pirie 1992** |  |  |  |  | 2.9 |
| **Garvey 2000** |  |  |  |  | 12.5 |
| **Gross 1995** |  |  |  |  | 1.9 |
| **Abelin 1989** |  |  |  |  | 6 |
| **Ehrsam 1991** |  |  |  |  | 2.7 |
| **TNSG 1991** |  |  |  |  | 20.9 |
| **Fiore 1994A** |  |  |  |  | 1.8 |
| **Richmond 1994** |  |  |  |  | 1.3 |
| **CEASE 1999** |  |  |  |  | 20.5 |
| **Fiore 1994B** |  |  |  |  | 2.3 |
| **Gourlay 1995** |  |  |  |  | 0.4 |
| **Tonnesen 1991** |  |  |  |  | 1.5 |
| **Stapleton 1995** |  |  |  |  | 5 |
| **Tonnesen 1993** |  |  |  |  | 3 |
| **Hjalmarson 1997** |  |  |  |  | 2.3 |
| **Shiffman 2002B** |  |  |  |  | 5.6 |
| **Shiffman 2002A** |  |  |  |  | 8 |
| **Blondal 1999** |  |  |  |  | 0.6 |

## NRT versus Placebo- Weight gain, 6 months

|  | **Selection bias - randomization** | **Selection bias - allocation** | **Performance and detection bias** | **Definition of abstinence** | **Weight (%)** |
| --- | --- | --- | --- | --- | --- |
| **Hjalmarson 1984** |  |  |  |  | 7.3 |
| **Pirie 1992** |  |  |  |  | 4.1 |
| **Sachs 1993** |  |  |  |  | 7.4 |
| **Richmond 1994** |  |  |  |  | 3.8 |
| **Puska 1995** |  |  |  |  | 12.7 |
| **Bohadana 2000** |  |  |  |  | 19.2 |
| **Hjalmarson 1997** |  |  |  |  | 13.7 |
| **Shiffman 2002B** |  |  |  |  | 11.3 |
| **Shiffman 2002A** |  |  |  |  | 20.5 |

## NRT versus Placebo- Weight gain, 12 months

|  | **Selection bias - randomization** | **Selection bias - allocation** | **Performance and detection bias** | **Definition of abstinence** | **Weight (%)** |
| --- | --- | --- | --- | --- | --- |
| **Pirie 1992** |  |  |  |  | 2.7 |
| **Richmond 1994** |  |  |  |  | 2.9 |
| **Puska 1995** |  |  |  |  | 7.7 |
| **Bohadana 2000** |  |  |  |  | 10.6 |
| **CEASE 1999** |  |  |  |  | 26.9 |
| **Stapleton 1995** |  |  |  |  | 4.1 |
| **Tonnesen 1991** |  |  |  |  | 2.1 |
| **Sutherland 1992** |  |  |  |  | 3.5 |
| **Blondal 1999** |  |  |  |  | 2.8 |
| **Hjalmarson 1994** |  |  |  |  | 4.1 |
| **Hjalmarson 1997** |  |  |  |  | 14 |
| **Tonnesen 1993** |  |  |  |  | 3.2 |
| **Wallstrom 2000** |  |  |  |  | 5.2 |
| **Shiffman 2002B** |  |  |  |  | 2.7 |
| **Shiffman 2002A** |  |  |  |  | 7.5 |

## Varenicline 2mg/day versus Placebo- Weight gain, End of treatment

|  | **Selection bias - randomization** | **Selection bias - allocation** | **Performance and detection bias** | **Definition of abstinence** | **Weight (%)** |
| --- | --- | --- | --- | --- | --- |
| **Gonzales 2006** |  |  |  |  | 4.3 |
| **Jorenby 2006** |  |  |  |  | 3.8 |
| **Nakamura 2007** |  |  |  |  | 16.9 |
| **Niaura 2008** |  |  |  |  | 1.3 |
| **Nides 2006** |  |  |  |  | 1.8 |
| **Oncken 2006** |  |  |  |  | 1.8 |
| **Rigotti 2010** |  |  |  |  | 7 |
| **Tashkin 2011** |  |  |  |  | 2.8 |
| **Tonstad 2006** |  |  |  |  | 45.8 |
| **Tsai 2008** |  |  |  |  | 8.6 |
| **Wang 2009** |  |  |  |  | 6 |

## Varenicline 2mg/day versus Placebo- Weight gain, 6 months

|  | **Selection bias - randomization** | **Selection bias - allocation** | **Performance and detection bias** | **Definition of abstinence** | **Weight (%)** |
| --- | --- | --- | --- | --- | --- |
| **Wang 2009** |  |  |  |  | 100 |

## Varenicline 2mg/day versus Placebo- Weight gain, 12 months

|  | **Selection bias - randomization** | **Selection bias - allocation** | **Performance and detection bias** | **Definition of abstinence** | **Weight (%)** |
| --- | --- | --- | --- | --- | --- |
| **Rigotti 2010** |  |  |  |  | 76.8 |
| **Tashkin 2011** |  |  |  |  | 23.2 |

## Varenicline 1mg/day versus Placebo- Weight gain, End of treatment

|  | **Selection bias - randomization** | **Selection bias - allocation** | **Performance and detection bias** | **Definition of abstinence** | **Weight (%)** |
| --- | --- | --- | --- | --- | --- |
| **Nakamura 2007** |  |  |  |  | 76.1 |
| **Nides 2006** |  |  |  |  | 9 |
| **Oncken 2006** |  |  |  |  | 14.9 |

# Hartmann-Boyce 2018 {332}

## NRT patch versus Placebo- Abstinence/cessation, 6 months

|  | **Selection bias - randomization** | **Selection bias - allocation** | **Blinding** | **Attrition bias** |
| --- | --- | --- | --- | --- |
| **Gourlay 1995** |  |  |  |  |

## NRT patch versus Placebo- Adverse events (Palpitations/chest pains), Range of follow-up times

|  | **Selection bias - randomization** | **Selection bias - allocation** | **Blinding** | **Attrition bias** | **Weight (%)** |
| --- | --- | --- | --- | --- | --- |
| **Bolliger 2000b** |  |  |  |  | 3.2 |
| **Brantmark 1973b** |  |  |  |  | 6.3 |
| **Bullen 2010** |  |  |  |  | 1.6 |
| **CEASE 1999** |  |  |  |  | 15.2 |
| **Gourlay 1995** |  |  |  |  | 5.3 |
| **Hays 1999** |  |  |  |  | 3.2 |
| **Hjalmarson 1994** |  |  |  |  | 3.1 |
| **Oncken 2007** |  |  |  |  | 1.2 |
| **Schneider 1995** |  |  |  |  | 13.4 |
| **Schnoll 2010** |  |  |  |  | 4.7 |
| **Shiffman 2009**  **(4 mg)** |  |  |  |  | 9.7 |
| **Sutherland 1992** |  |  |  |  | 19.4 |
| **Sonderskov 1997** |  |  |  |  | 6.3 |
| **Tonnesen 1988** |  |  |  |  | 1.1 |
| **Wennike 2003b** |  |  |  |  | 6.3 |

Greyed out= Studies which were originally excluded from the original review but had since been included by review authors in order to utilize their adverse events data. These studies do not contribute to efficacy data; and risk of bias assessments were not performed.

## NRT patch versus Placebo- Adverse events (Attrition), Follow-up unclear/NR

Trials included in this analysis are not clearly stated.

## NRT patch versus Placebo- Adverse events (All), Follow-up unclear/NR

|  | **Selection bias - randomization** | **Selection bias - allocation** | **Blinding** | **Attrition bias** |
| --- | --- | --- | --- | --- |
| **Schneider 1996** |  |  |  |  |
| **Tonnesen 2012** |  |  |  |  |
| **Fiore 1992** |  |  |  |  |
| **Palmer 1992** |  |  |  |  |
| **Wallstrom 1999** |  |  |  |  |
| **Bailey 2012** |  |  |  |  |

Greyed out= Studies which were originally excluded from the original review but had since been included by review authors in order to utilize their adverse events data. These studies do not contribute to efficacy data; and risk of bias assessments were not performed.

# Hollands 2019 {3841}

## Interventions to increase adherence for tobacco dependence versus Usual or standard care- Abstinence/cessation, 6 months

|  | **Selection bias - randomization** | **Selection bias - allocation** | **Blinding of participants and personnel (performance)** | **Blinding of outcome assessment (detection)** | **Attrition** | **Selective reporting** | **Validity and reliability of outcome measures** | **Baseline comparability** | **Consistency in intervention delivery** | **Summary ROB** | **Weight (%)** |
| --- | --- | --- | --- | --- | --- | --- | --- | --- | --- | --- | --- |
| **Chan 2010** |  |  |  |  |  |  |  |  |  |  | 9.2 |
| **Chan 2011** |  |  |  |  |  |  |  |  |  |  | 10.3 |
| **Marteau 2012** |  |  |  |  |  |  |  |  |  |  | 9.5 |
| **Smith 2013** |  |  |  |  |  |  |  |  |  |  | 71 |
| **Schlam 2018** |  |  |  |  |  |  |  |  |  |  |  |

## Interventions to increase adherence to medications for tobacco dependence versus Usual or standard care- Adverse events, Follow-up unclear/NR

|  | **Selection bias - randomization** | **Selection bias - allocation** | **Blinding of participants and personnel (performance)** | **Blinding of outcome assessment (detection)** | **Attrition** | **Selective reporting** | **Validity and reliability of outcome measures** | **Baseline comparability** | **Consistency in intervention delivery** | **Summary ROB** |
| --- | --- | --- | --- | --- | --- | --- | --- | --- | --- | --- |
| **Marteau 2012** |  |  |  |  |  |  |  |  |  |  |
| **Smith 2013** |  |  |  |  |  |  |  |  |  |  |
| **Mooney 2005** |  |  |  |  |  |  |  |  |  |  |

## Interventions to increase adherence to medications for tobacco dependence versus Usual or standard care- Change in emotional state (Anxiety), 1-week and 6 months

|  | **Selection bias - randomization** | **Selection bias - allocation** | **Blinding of participants and personnel (performance)** | **Blinding of outcome assessment (detection)** | **Attrition** | **Selective reporting** | **Validity and reliability of outcome measures** | **Baseline comparability** | **Consistency in intervention delivery** | **Summary ROB** |
| --- | --- | --- | --- | --- | --- | --- | --- | --- | --- | --- |
| **Marteau 2012** |  |  |  |  |  |  |  |  |  |  |

# Howes 2020 {96}

## Bupropion versus Placebo- Abstinence/cessation, 6 months

|  | **Selection bias - randomization** | **Selection bias - allocation** | **Blinding** | **Attrition** | **Other bias** | **Weight (%)** |
| --- | --- | --- | --- | --- | --- | --- |
| **Hatsukami 2004** |  |  |  |  | NA | 100 |

NA= Not assessed

## Bupropion versus Placebo- Reduction in cigarettes/day, 12 months

|  | **Selection bias - randomization** | **Selection bias - allocation** | **Blinding** | **Attrition** | **Other bias** |
| --- | --- | --- | --- | --- | --- |
| **Hatsukami 2004** |  |  |  |  | NA |

NA= Not assessed

## Bupropion versus Placebo- Reduction in cotinine >50%, 12 months

|  | **Selection bias - randomization** | **Selection bias - allocation** | **Blinding** | **Attrition** | **Other bias** | **Weight (%)** |
| --- | --- | --- | --- | --- | --- | --- |
| **Hatsukami 2004** |  |  |  |  | NA | 100 |

NA= Not assessed

## Bupropion versus Placebo- Change in emotional state (depressive symptoms), Specific timepoints NR

|  | **Selection bias - randomization** | **Selection bias - allocation** | **Blinding** | **Attrition** | **Other bias** |
| --- | --- | --- | --- | --- | --- |
| **Collins 2004** |  |  |  |  | NA |

NA= Not assessed

## St John’s wort versus Placebo- Abstinence/cessation, 6 months

|  | **Selection bias - randomization** | **Selection bias - allocation** | **Blinding** | **Attrition** | **Other bias** | **Weight (%)** |
| --- | --- | --- | --- | --- | --- | --- |
| **Parsons 2009** |  |  |  |  | NA | 89.9 |
| **Sood 2010** |  |  |  |  | NA | 10.1 |

NA= Not assessed

## S-Adenosyl-L-Methionine (SAMe) versus Placebo- Abstinence/cessation, 6 months

|  | **Selection bias - randomization** | **Selection bias - allocation** | **Blinding** | **Attrition** | **Other bias** | **Weight (%)** |
| --- | --- | --- | --- | --- | --- | --- |
| Sood 2012 |  |  |  |  | NA | 100 |

NA= Not assessed

# Lancaster 2017 {539}

## Individual counselling versus Minimal contact control- Abstinence/cessation, 6+ months

|  | **Selection bias - randomization** | **Selection bias - allocation** | **Detection bias** | **Attrition bias** | **Weight (%)** |
| --- | --- | --- | --- | --- | --- |
| **Aleixandre 1998** |  |  |  |  | 0.9 |
| **Bronson 1989** |  |  |  |  | 1.6 |
| **Burling 1991** |  |  |  |  | Not estimable |
| **Burling 2001** |  |  |  |  | 0.3 |
| **Chan 2012** |  |  |  |  | 11.8 |
| **Chen 2014** |  |  |  |  | 2.6 |
| **Dornelas 2000** |  |  |  |  | 3.4 |
| **Glasgow 2000** |  |  |  |  | 5.7 |
| **Hannover 2009** |  |  |  |  | 0.1 |
| **Hennrikus 2005** |  |  |  |  | 17.6 |
| **Hennrikus 2010** |  |  |  |  | 1.1 |
| **Kim 2005** |  |  |  |  | 4.7 |
| **Marley 2014** |  |  |  |  | 0.9 |
| **Marshall 2016** |  |  |  |  | 1.3 |
| **Molyneux 2003** |  |  |  |  | 1.8 |
| **Mueller 2012** |  |  |  |  | 0.9 |
| **Nakamura 2004** |  |  |  |  | 1.1 |
| **Ockene 1992** |  |  |  |  | 7.6 |
| **Pedersen 2005** |  |  |  |  | 5.4 |
| **Pederson 1991** |  |  |  |  | 1.7 |
| **Ramos 2010** |  |  |  |  | 0.2 |
| **Rigotti 1997** |  |  |  |  | 7 |
| **Simon 1997** |  |  |  |  | 2.5 |
| **Stevens 1993** |  |  |  |  | 12.9 |
| **Thankappan 2013** |  |  |  |  | 3.6 |
| **Weissfeld 1991** |  |  |  |  | 0.7 |
| **Windsor 1988** |  |  |  |  | 2.8 |

# Lindson-Hawley 2016 {671}

## NRT versus Placebo- Abstinence/cessation, 12 months to 24 months

|  | **Selection bias - randomization** | **Selection bias - allocation** | **Performance and detection bias** | **Attrition bias** | **Biochemical validation** | **Weight (%)** |
| --- | --- | --- | --- | --- | --- | --- |
| **Kralikova 2009** |  |  |  |  |  | 15.3 |
| **Etter 2004** |  |  |  |  |  | 31.7 |
| **Australia NNCG-017** |  |  |  |  |  | 8.9 |
| **Haustein 2003** |  |  |  |  |  | 0.6 |
| **Batra 2005** |  |  |  |  |  | 9 |
| **Wennike 2003** |  |  |  |  |  | 8.9 |
| **Bolliger 2000** |  |  |  |  |  | 21.7 |
| **Rennard 2006** |  |  |  |  |  | 3.8 |

## NRT versus Placebo- Reduction in cigarettes/day of >50% of baseline or cessation, 12+ months

|  | **Selection bias - randomization** | **Selection bias - allocation** | **Performance and detection bias** | **Attrition bias** | **Biochemical validation** | **Weight (%)** |
| --- | --- | --- | --- | --- | --- | --- |
| **Kralikova 2009** |  |  |  |  |  | 28.9 |
| **Etter 2004** |  |  |  |  |  | 45.4 |
| **Australia NNCG-017** |  |  |  |  |  | 7 |
| **Haustein 2003** |  |  |  |  |  | 6.2 |
| **Batra 2005** |  |  |  |  |  | 3.9 |
| **Wennike 2003** |  |  |  |  |  | 0.8 |
| **Bolliger 2000** |  |  |  |  |  | 4.7 |
| **Rennard 2006** |  |  |  |  |  | 3.1 |

## Bupropion versus Placebo- Abstinence/cessation, 6 months

|  | **Selection bias - randomization** | **Selection bias - allocation** | **Performance and detection bias** | **Attrition bias** | **Weight (%)** |
| --- | --- | --- | --- | --- | --- |
| **Hatsukami 2004a** |  |  |  |  | 100 |

## Bupropion versus Placebo- Reduction in cigarettes/day of >50% of baseline or cessation, 12 months

|  | **Selection bias - randomization** | **Selection bias - allocation** | **Performance and detection bias** | **Attrition bias** | **Weight (%)** |
| --- | --- | --- | --- | --- | --- |
| **Hatsukami 2004a** |  |  |  |  | 100 |

## Bupropion versus Placebo- Reduction in cotinine >50%, 12 months

|  | **Selection bias - randomization** | **Selection bias - allocation** | **Performance and detection bias** | **Attrition bias** |
| --- | --- | --- | --- | --- |
| **Hatsukami 2004a** |  |  |  |  |

## Bupropion versus Placebo- Reduction in cotinine, 12 months

|  | **Selection bias - randomization** | **Selection bias - allocation** | **Performance and detection bias** | **Attrition bias** |
| --- | --- | --- | --- | --- |
| **Hatsukami 2004a** |  |  |  |  |

## Bupropion versus Placebo- Serious adverse events, Follow up unclear/NR

|  | **Selection bias - randomization** | **Selection bias - allocation** | **Performance and detection bias** | **Attrition bias** |
| --- | --- | --- | --- | --- |
| **Hatsukami 2004a** |  |  |  |  |

## Varenicline versus Placebo- Abstinence/cessation, 6 months

|  | **Selection bias - randomization** | **Selection bias - allocation** | **Performance and detection bias** | **Attrition bias** | **Weight (%)** |
| --- | --- | --- | --- | --- | --- |
| **Hughes 2011** |  |  |  |  | 100 |

## Varenicline versus Placebo- Adverse events (stopping medication due to adverse event), Follow-up unclear/NR

|  | **Selection bias - randomization** | **Selection bias - allocation** | **Performance and detection bias** | **Attrition bias** |
| --- | --- | --- | --- | --- |
| **Hughes 2011** |  |  |  |  |

## Telephone counselling plus self-help materials versus Usual care- Abstinence/cessation, 12 months

|  | **Selection bias - randomization** | **Selection bias - allocation** | **Performance and detection bias** | **Attrition bias** | **Weight (%)** |
| --- | --- | --- | --- | --- | --- |
| **Glasgow 2009** |  |  |  |  | 100 |

## Telephone counselling plus self-help materials versus Usual care- Reduction in cigarettes/day of >50% of baseline or cessation, 12 months

|  | **Selection bias - randomization** | **Selection bias - allocation** | **Performance and detection bias** | **Attrition bias** | **Weight (%)** |
| --- | --- | --- | --- | --- | --- |
| **Glasgow 2009** |  |  |  |  | 100 |

## Telephone counselling plus self-help materials versus Usual care- Reduction in CO >50%, 12 months

|  | **Selection bias - randomization** | **Selection bias - allocation** | **Performance and detection bias** | **Attrition bias** |
| --- | --- | --- | --- | --- |
| **Glasgow 2009** |  |  |  |  |

## Telephone counselling plus self-help materials versus Usual care- Reduction in cigarettes/day from baseline, 12 months

|  | **Selection bias - randomization** | **Selection bias - allocation** | **Performance and detection bias** | **Attrition bias** |
| --- | --- | --- | --- | --- |
| **Glasgow 2009** |  |  |  |  |

## Telephone counselling plus self-help materials versus Usual care- Reduction in CO from baseline, 12 months

|  | **Selection bias - randomization** | **Selection bias - allocation** | **Performance and detection bias** | **Attrition bias** |
| --- | --- | --- | --- | --- |
| **Glasgow 2009** |  |  |  |  |

## Behavioural support (advice) plus NRT plus phone calls versus No intervention- Abstinence/cessation, 6 months

|  | **Selection bias - randomization** | **Selection bias - allocation** | **Performance and detection bias** | **Attrition bias** |
| --- | --- | --- | --- | --- |
| **Carpenter 2004** |  |  |  |  |

## Behavioural support (advice) plus NRT plus phone calls versus No intervention- Reduction in number of cigarettes/day, 6 months

|  | **Selection bias - randomization** | **Selection bias - allocation** | **Performance and detection bias** | **Attrition bias** |
| --- | --- | --- | --- | --- |
| **Carpenter 2004** |  |  |  |  |

## E-cigarettes versus Placebo- Abstinence/cessation, 12 months

|  | **Selection bias - randomization** | **Selection bias - allocation** | **Performance and detection bias** | **Attrition bias** | **Weight (%)** |
| --- | --- | --- | --- | --- | --- |
| **Caponnetto 2013** |  |  |  |  | 100 |

## E-cigarettes versus Placebo- Reduction in cigarettes/day of >50% of baseline or cessation, 12months

|  | **Selection bias - randomization** | **Selection bias - allocation** | **Performance and detection bias** | **Attrition bias** | **Weight (%)** |
| --- | --- | --- | --- | --- | --- |
| **Caponnetto 2013** |  |  |  |  | 100 |

## E-cigarettes versus Placebo- Reduction in number of cigarettes/day, 12 months

|  | **Selection bias - randomization** | **Selection bias - allocation** | **Performance and detection bias** | **Attrition bias** |
| --- | --- | --- | --- | --- |
| **Caponnetto 2013** |  |  |  |  |

## E-cigarettes versus Placebo- Reduction in CO, 12 months

|  | **Selection bias - randomization** | **Selection bias - allocation** | **Performance and detection bias** | **Attrition bias** |
| --- | --- | --- | --- | --- |
| **Caponnetto 2013** |  |  |  |  |

## E-cigarettes versus Placebo- Adverse events, Baseline, 3 months, 12 months

|  | **Selection bias - randomization** | **Selection bias - allocation** | **Performance and detection bias** | **Attrition bias** |
| --- | --- | --- | --- | --- |
| **Caponnetto 2013** |  |  |  |  |

## E-cigarettes versus Placebo- Serious adverse events, 12 months

|  | **Selection bias - randomization** | **Selection bias - allocation** | **Performance and detection bias** | **Attrition bias** |
| --- | --- | --- | --- | --- |
| **Caponnetto 2013** |  |  |  |  |

## E-cigarettes versus Placebo- Weight gain, Follow-up unclear/NR

|  | **Selection bias - randomization** | **Selection bias - allocation** | **Performance and detection bias** | **Attrition bias** |
| --- | --- | --- | --- | --- |
| **Caponnetto 2013** |  |  |  |  |

# Livingstone-Banks 2019 {1077}

## Non-tailored print-based self-help materials (no face-to-face contact) versus No materials/no intervention- Abstinence/cessation, 6+ months

|  | **Selection bias - randomization** | **Selection bias - allocation** | **Blinding** | **Attrition** | **Other bias** | **Weight (%)** |
| --- | --- | --- | --- | --- | --- | --- |
| **Cuckle 1984** |  |  |  |  | NA | 14 |
| **Curry 1995** |  |  |  |  | NA | 2.7 |
| **Dijkstra 1999** |  |  |  |  | NA | 3 |
| **Gritz 1992** |  |  |  |  | NA | 3.5 |
| **Humerfelt 1998** |  |  |  |  | NA | 23.7 |
| **Lando 1991** |  |  |  |  | NA | 7.2 |
| **Ledwith 1984** |  |  |  |  | NA | 1.8 |
| **Lennox 2001** |  |  |  |  | NA | 6.5 |
| **Pallonen 1994** |  |  |  |  | NA | 2.3 |
| **Schofield 1999** |  |  |  |  | NA | 27.6 |
| **Willemsen 2006** |  |  |  |  | NA | 7.6 |

NA= Not assessed

## Non-tailored print-based self-help (no face-to-face contact) versus No materials/no interventions- Abstinence/cessation, 6 months

|  | **Selection bias - randomization** | **Selection bias - allocation** | **Blinding** | **Attrition** | **Other bias** | **Weight (%)** |
| --- | --- | --- | --- | --- | --- | --- |
| **Becona 2001a** |  |  |  |  | NA | 7.6 |
| **Becona 2001b** |  |  |  |  | NA | 92.4 |

NA= Not assessed

## Non-tailored print-based self-help materials (no face-to-face contact) versus Brief leaflet- Abstinence/cessation, 6+ months

|  | **Selection bias - randomization** | **Selection bias - allocation** | **Blinding** | **Attrition** | **Other bias** | **Weight (%)** |
| --- | --- | --- | --- | --- | --- | --- |
| **Cummings 1988** |  |  |  |  | NA | 17.3 |
| **Davis 1984** |  |  |  |  | NA | 5.2 |
| **Lichtenstein 2000** |  |  |  |  | NA | 7.1 |
| **Lichtenstein 2008** |  |  |  |  | NA | 18.5 |
| **Orleans 1991** |  |  |  |  | NA | 26.4 |
| **Parekh 2014** |  |  |  |  | NA | 25.6 |

NA= Not assessed

## Non-tailored print-based self-help materials (with face-to-face contact) versus No treatment or leaflet only- Abstinence/cessation, 6+ months

|  | **Selection bias - randomization** | **Selection bias - allocation** | **Blinding** | **Attrition** | **Other bias** | **Weight (%)** |
| --- | --- | --- | --- | --- | --- | --- |
| **Betson 1998** |  |  |  |  | NA | 7.4 |
| **Campbell 1986** |  |  |  |  | NA | 23.5 |
| **Prue 1983** |  |  |  |  | NA | 2.4 |
| **Resnicow 1997** |  |  |  |  | NA | 66.7 |

NA= Not assessed

## Individually tailored print-based self-help materials (no face-to-face contact) versus No materials/ no interventions- Abstinence/cessation- 6+ months

|  | **Selection bias - randomization** | **Selection bias - allocation** | **Blinding** | **Attrition** | **Other bias** | **Weight (%)** |
| --- | --- | --- | --- | --- | --- | --- |
| **Dijkstra 1998a** |  |  |  |  | NA | 1.4 |
| **Etter 2004** |  |  |  |  | NA | 22.9 |
| **Hoving 2010 (I)** |  |  |  |  | NA | 1.2 |
| **Hoving 2010 (2)** |  |  |  |  | NA | 4.9 |
| **Meyer 2008** |  |  |  |  | NA | 9.6 |
| **Meyer 2016** |  |  |  |  | NA | 4.1 |
| **Prochaska 2001a** |  |  |  |  | NA | 4 |
| **Prochaska 2001b** |  |  |  |  | NA | 23.5 |
| **Prochaska 2004** |  |  |  |  | NA | 4.6 |
| **Prochaska 2005** |  |  |  |  | NA | 20.6 |
| **Schumann 2008** |  |  |  |  | NA | 3.2 |

NA= Not assessed

1. Pharmacy arms
2. GP arms

# Matkin 2019 {1228}

## Hotline and self-help materials versus Minimal intervention- Abstinence/cessation, 12-18 months

|  | **Selection bias - randomization** | **Selection bias - allocation** | **Detection bias** | **Attrition** | **Weight (%)** |
| --- | --- | --- | --- | --- | --- |
| **Ossip-Klein 1991** |  |  |  |  | 62.3 |
| **Zwar 2015** |  |  |  |  | 37.7 |

## Intense telephone counselling versus Minimal intervention- Abstinence/cessation, 6+ months

|  | **Selection bias - randomization** | **Selection bias - allocation** | **Detection bias** | **Attrition** | **Weight (%)** |
| --- | --- | --- | --- | --- | --- |
| **Miller 1997** |  |  |  |  | 33.4 |
| **Piper 2016** |  |  |  |  | 3.3 |
| **Swan 2003** |  |  |  |  | 63.3 |

## Brief motivational telephone counselling versus Usual care telephone call- Abstinence/cessation- 12 months

|  | **Selection bias - randomization** | **Selection bias - allocation** | **Detection bias** | **Attrition** | **Weight (%)** |
| --- | --- | --- | --- | --- | --- |
| **Klemperer 2017** |  |  |  |  | 100 |

## Telephone counselling for smoking reduction versus Usual care telephone call- Abstinence/cessation, 12 months

|  | **Selection bias - randomization** | **Selection bias - allocation** | **Detection bias** | **Attrition** | **Weight (%)** |
| --- | --- | --- | --- | --- | --- |
| **Klemperer 2017** |  |  |  |  | 100 |

# Posadzki 2016 {659}

## Interactive voice response (IVR) systems versus No intervention- Abstinence/cessation, 24 months

|  | **Selection bias - randomization** | **Selection bias - allocation** | **Performance bias** | **Detection bias** | **Attrition** | **Selective reporting** | **Other bias** |
| --- | --- | --- | --- | --- | --- | --- | --- |
| **McNaughton 2013** |  |  |  |  |  |  |  |

# Stead 2013 {1998}

## Physician advice (minimal or intensive interventions) versus No advice (or usual care)- Abstinence/cessation, 6+ months

|  | **Selection bias - randomization** | **Selection bias - allocation** | **Attrition** | **Weight (%)** |
| --- | --- | --- | --- | --- |
| **Slama 1990** |  |  |  | 0.4 |
| **Porter 1972** |  |  |  | 0.9 |
| **Demers 1990** |  |  |  | 0.9 |
| **Nebot 1989** |  |  |  | 1 |
| **Stewart 1982** |  |  |  | 1.2 |
| **Page 1986** |  |  |  | 1.3 |
| **Slama 1995** |  |  |  | 1.4 |
| **Russell 1979** |  |  |  | 1.6 |
| **Haug 1994** |  |  |  | 1.9 |
| **McDowell 1985** |  |  |  | 2.3 |
| **Betson 1997** |  |  |  | 2.7 |
| **Janz 1987** |  |  |  | 2.8 |
| **Wilson 1990** |  |  |  | 3.6 |
| **Vetter 1990** |  |  |  | 4.1 |
| **Higashi 1995** |  |  |  | 7 |
| **Russell 1983** |  |  |  | 7.7 |
| **Jamrozik 1984** |  |  |  | 17.1 |
| **Richmond 1986** |  |  |  | 0.4 |
| **Hilberink 2005** |  |  |  | 1.3 |
| **Pieterse 2001** |  |  |  | 1.7 |
| **Severson 1997** |  |  |  | 2.3 |
| **Unrod 2007** |  |  |  | 3.5 |
| **Morgan 1996** |  |  |  | 5.4 |
| **Schnoll 2003** |  |  |  | 5.7 |
| **Meyer 2008** |  |  |  | 6.7 |
| **Rose 78-92** |  |  |  | 15 |

## Physician advice with follow-up versus Minimal intervention /advice with single visit- Abstinence/cessation, 6+ months

|  | **Selection bias - randomization** | **Selection bias - allocation** | **Attrition** | **Weight (%)** |
| --- | --- | --- | --- | --- |
| **Fagerstrom 1984** |  |  |  | 1.8 |
| **Marshall 1985** |  |  |  | 28.6 |
| **Ockene 1991** |  |  |  | 37 |
| **Segnan 1991** |  |  |  | 10 |
| **Wilson 1982** |  |  |  | 22.6 |

## Intensive advice versus Minimal advice- Abstinence/cessation, 6+ months

|  | **Selection bias - randomization** | **Selection bias - allocation** | **Attrition** | **Weight (%)** |
| --- | --- | --- | --- | --- |
| **Segnan 1991** |  |  |  | 1.6 |
| **Wilson 1982** |  |  |  | 3.2 |
| **Butler 1999** |  |  |  | 1.2 |
| **Marshall 1985** |  |  |  | 4.1 |
| **Thompson 1988** |  |  |  | 7.4 |
| **Lang 2000** |  |  |  | 7.2 |
| **Ockene 1991** |  |  |  | 7.7 |
| **Slama 1990** |  |  |  | 0.3 |
| **Jamrozik 1984** |  |  |  | 30 |
| **Fagerstrom 1984** |  |  |  | 0.3 |
| **BTS 1990b** |  |  |  | 10.2 |
| **Ardron 1988** |  |  |  | 0.4 |
| **Li 1984** |  |  |  | 2.8 |
| **BTS 1990a** |  |  |  | 14.8 |
| **Burt 1974** |  |  |  | 8.8 |

# Stead 2016 {1356}

## Combined pharmacotherapy and behavioural interventions versus Usual care or minimal intervention- Abstinence/cessation, 6+ months

|  | **Selection bias - randomization** | **Selection bias - allocation** | **Attrition bias** | **Weight (%)** |
| --- | --- | --- | --- | --- |
| **An 2006** |  |  |  | 2.1 |
| **Baker 2006** |  |  |  | 0.1 |
| **Bernstein 2015** |  |  |  | 5.5 |
| **Binnie 2007** |  |  |  | 0.3 |
| **Brandstein 2011** |  |  |  | 0.5 |
| **Carmody 2012** |  |  |  | 1.1 |
| **Chan 2010** |  |  |  | 2.1 |
| **Chouinard 2005** |  |  |  | 0.8 |
| **Cooney 2007** |  |  |  | 0.1 |
| **Duffy 2006** |  |  |  | 0.8 |
| **Emmons 2005** |  |  |  | 4.4 |
| **Haas 2015** |  |  |  | 3.5 |
| **Hall 2002** |  |  |  | 0.7 |
| **Hall 2006** |  |  |  | 2.6 |
| **Hanioka 2010** |  |  |  | 0.4 |
| **Hickman 2015** |  |  |  | 1 |
| **Hollis 2007** |  |  |  | 11.4 |
| **Juarranz Sanz 1998** |  |  |  | 0.5 |
| **Katz 2004** |  |  |  | 2.8 |
| **Kotz 2009** |  |  |  | 0.6 |
| **Lee 2015** |  |  |  | 0.6 |
| **Lewis 1998** |  |  |  | 0.4 |
| **McCarthy 2008** |  |  |  | 2.1 |
| **Mohiuddin 2007** |  |  |  | 1.2 |
| **Molyneux 2003** |  |  |  | 0.9 |
| **Murray 2013** |  |  |  | 0.9 |
| **Ockene 1991** |  |  |  | 3.2 |
| **Okuyemi 2007** |  |  |  | 0.9 |
| **Otero 2006** |  |  |  | 4.9 |
| **Peckham 2015** |  |  |  | 0.9 |
| **Perez-Tortosa 2015** |  |  |  | 8 |
| **Prochaska 2014** |  |  |  | 0.9 |
| **Ratner 2004** |  |  |  | 1.3 |
| **Reid 2003** |  |  |  | 5.6 |
| **Reid 2008** |  |  |  | 0.8 |
| **Rigotti 2014** |  |  |  | 2.5 |
| **Rodriguez 2003** |  |  |  | 1.2 |
| **Sadr Azodi 2009** |  |  |  | 1 |
| **Schauffler 2001** |  |  |  | 8 |
| **Segnan 1991** |  |  |  | 0.6 |
| **Simon 1997** |  |  |  | 1.2 |
| **Stockings 2014** |  |  |  | 0.1 |
| **Thomsen 2010** |  |  |  | 0.6 |
| **Tonnesen 2006** |  |  |  | 0.5 |
| **Velicer 2006** |  |  |  | 5.1 |
| **Vial 2002** |  |  |  | 0.2 |
| **Villebro 2008** |  |  |  | 0.2 |
| **Wakefield 2004** |  |  |  | 0.5 |
| **Wewers 2000** |  |  |  | 0.1 |
| **Wewers 2009** |  |  |  | 0.1 |
| **Wilson 1988** |  |  |  | 3.2 |
| **Winhusen 2014** |  |  |  | 1.2 |

## Combined pharmacotherapy and behavioural interventions versus Usual care or no intervention- Abstinence/cessation, 12 months

|  | **Selection bias - randomization** | **Selection bias - allocation** | **Attrition bias** | **Weight (%)** |
| --- | --- | --- | --- | --- |
| **Lung Health Study** |  |  |  | 100 |

# Stead 2017 {538}

## Group therapy versus No intervention- Abstinence/cessation, 6+ months

|  | **Selection bias - randomization** | **Selection bias - allocation** | **Blinding (Detection bias only*)** | **Attrition** | **Weight (%)** |
| --- | --- | --- | --- | --- | --- |
| **Pederson 1981** |  |  |  |  | 2 |
| **Leung 1991** |  |  |  |  | 2.7 |
| **Grant 2003** |  |  |  |  | 4 |
| **Hill 1993** |  |  |  |  | 5.6 |
| **Minthorn-Biggs 2000** |  |  |  |  | 7.1 |
| **Schleicher 2012** |  |  |  |  | 8 |
| **Zheng 2007** |  |  |  |  | 8.3 |
| **Cottraux 1983** |  |  |  |  | 23.7 |
| **McDowell 1985** |  |  |  |  | 38.7 |

* Biochemical validation accounted for the detection bias assessment

# Taylor 2017 {411}

## Interactive and tailored internet intervention versus Non-active control- Abstinence/cessation, 6-12 months

|  | **Selection bias - randomization** | **Selection bias - allocation** | **Attrition** | **Weight (%)** |
| --- | --- | --- | --- | --- |
| **Haug 2011** |  |  |  | 6.8 |
| **Elfeddali 2012** |  |  |  | 16 |
| **Harrington 2016** |  |  |  | 51.2 |
| **Borland 2013** |  |  |  | 8.8 |
| **Emmon 2013** |  |  |  | 6.3 |
| **Smit 2016** |  |  |  | 3.3 |
| **Skov-Ettrup 2016** |  |  |  | 4.2 |
| **Yang 2016** |  |  |  | 3.4 |

## Internet plus behavioural support versus Non-internet-based non-active control- Abstinence/cessation, 6-12 months

|  | **Selection bias - randomization** | **Selection bias - allocation** | **Performance bias - blinding of participants** | **Weight (%)** |
| --- | --- | --- | --- | --- |
| **Brendryen 2008a** |  |  |  | 11.8 |
| **Brendryen 2008b** |  |  |  | 30.6 |
| **Borland 2013** |  |  |  | 40 |
| **Burford 2013** |  |  |  | 1.2 |
| **Smit 2016** |  |  |  | 16.4 |

# Tsoi 2013 {1698}

## Bupropion versus Placebo- Abstinence/cessation, 6 months

|  | **Selection bias - randomization** | **Selection bias - allocation** | **Blinding** | **Attrition** | **Selective reporting** | **Other** | **Weight** |
| --- | --- | --- | --- | --- | --- | --- | --- |
| **Evins 2001** |  |  |  |  |  |  | 10.6 |
| **Evins 2005** |  |  |  |  |  |  | 13.6 |
| **George 2002** |  |  |  |  |  |  | 21.6 |
| **George 2008** |  |  |  |  |  |  | 12.1 |
| **Evins 2007** |  |  |  |  |  |  | 42.1 |

## Bupropion versus Placebo- Reduction in number of cigarettes per day from baseline, 6 months

|  | **Selection bias** |  | **Blinding** | **Attrition** | **Selective reporting** | **Other** | **Weight** |
| --- | --- | --- | --- | --- | --- | --- | --- |
| **Evins 2005** |  |  |  |  |  |  | 92.1 |
| **Evins 2007** |  |  |  |  |  |  | 7.9 |

## Bupropion versus Placebo- Tobacco smoking reduction- Expired CO level, 6 months

|  | **Selection bias** |  | **Blinding** | **Attrition** | **Selective reporting** | **Other** | **Weight** |
| --- | --- | --- | --- | --- | --- | --- | --- |
| **Evins 2007** |  |  |  |  |  |  | 36.3 |
| **Evins 2005** |  |  |  |  |  |  | 35.4 |
| **Evins 2001** |  |  |  |  |  |  | 28.3 |

## Bupropion versus Placebo- Change in emotional state (positive symptoms), End of treatment

|  | **Selection bias** |  | **Blinding** | **Attrition** | **Selective reporting** | **Other** | **Weight** |
| --- | --- | --- | --- | --- | --- | --- | --- |
| **Evins 2005** |  |  |  |  |  |  | 61.9 |
| **George 2002** |  |  |  |  |  |  | 38.1 |

## Bupropion versus Placebo- Change in emotional state (negative symptoms), End of treatmen

|  | **Selection bias** |  | **Blinding** | **Attrition** | **Selective reporting** | **Other** | **Weight** |
| --- | --- | --- | --- | --- | --- | --- | --- |
| **Evins 2005** |  |  |  |  |  |  | 38.7 |
| **Evins 2007** |  |  |  |  |  |  | 37.6 |
| **George 2002** |  |  |  |  |  |  | 23.6 |

## Bupropion versus Placebo- Changes in emotional state (depressive symptoms), End of treatment

|  | **Selection bias** |  | **Blinding** | **Attrition** | **Selective reporting** | **Other** | **Weight** |
| --- | --- | --- | --- | --- | --- | --- | --- |
| **Evins 2005** |  |  |  |  |  |  | 39.1 |
| **Evins 2007** |  |  |  |  |  |  | 37.6 |
| **George 2002** |  |  |  |  |  |  | 23.3 |

## Bupropion versus Placebo- Adverse events, Follow-up unclear/NR

|  | **Selection bias** |  | **Blinding** | **Attrition** | **Selective reporting** | **Other** |
| --- | --- | --- | --- | --- | --- | --- |
| **Weiner 2012** |  |  |  |  |  |  |
| **George 2002** |  |  |  |  |  |  |
| **George 2008** |  |  |  |  |  |  |
| **Li 2009** |  |  |  |  |  |  |
| **Evins 2005** |  |  |  |  |  |  |
| **Evins 2007** |  |  |  |  |  |  |
| **Evins 2001** |  |  |  |  |  |  |

## Bupropion (reduction trials only) versus Placebo- Change in emotional state (positive, negative, and psychiatric symptoms), End of treatment

|  | **Selection bias** |  | **Blinding** | **Attrition** | **Selective reporting** | **Other** |
| --- | --- | --- | --- | --- | --- | --- |
| **Bloch 2010** |  |  |  |  |  |  |
| **Fatemi 2005** |  |  |  |  |  |  |
| **Tidey 2011** |  |  |  |  |  |  |

## Bupropion (reduction trials only) versus Placebo- Adverse events, Follow-up unclear/NR

|  | **Selection bias** |  | **Blinding** | **Attrition** | **Selective reporting** | **Other** |
| --- | --- | --- | --- | --- | --- | --- |
| **Bloch 2010** |  |  |  |  |  |  |
| **Akbarpour 2010** |  |  |  |  |  |  |
| **Tidey 2011** |  |  |  |  |  |  |

## Varenicline versus Placebo- Abstinence/cessation, 6 months

|  | **Selection bias** |  | **Blinding** | **Attrition** | **Selective reporting** | **Other** | **Weight (%)** |
| --- | --- | --- | --- | --- | --- | --- | --- |
| **Williams 2012** |  |  |  |  |  |  | 100 |

## Varenicline versus Placebo- Reduction in number of cigarettes per day from baseline, 6 months

|  | **Selection bias** |  | **Blinding** | **Attrition** | **Selective reporting** | **Other** |
| --- | --- | --- | --- | --- | --- | --- |
| **Williams 2012** |  |  |  |  |  |  |

## Varenicline versus Placebo- Changes in emotional state (positive, negative and depressive symptoms), End of treatment

|  | **Selection bias** |  | **Blinding** | **Attrition** | **Selective reporting** | **Other** |
| --- | --- | --- | --- | --- | --- | --- |
| **Williams 2012** |  |  |  |  |  |  |
| **Weiner 2011** |  |  |  |  |  |  |

## Varenicline versus Placebo- Adverse events, Follow-up unclear/NR

|  | **Selection bias - randomization** | **Selection bias - allocation** | **Blinding** | **Attrition** | **Selective reporting** | **Other** |
| --- | --- | --- | --- | --- | --- | --- |
| **Williams 2012** |  |  |  |  |  |  |
| **Weiner 2011** |  |  |  |  |  |  |

## Varenicline versus Placebo (trials with primary aim other than smoking cessation, reduction, and relapse)- Adverse events (including serious adverse events), Follow-up unclear/NR

|  | **Selection bias - randomization** | **Selection bias - allocation** | **Blinding** | **Attrition** | **Selective reporting** | **Other** |
| --- | --- | --- | --- | --- | --- | --- |
| **Hong 2011** |  |  |  |  |  |  |
| **Shim 2012** |  |  |  |  |  |  |
| **Meszaros 2012** |  |  |  |  |  |  |

## Varenicline versus Placebo (trials with primary aim other than smoking cessation, reduction and relapse)- Mental state, Follow-up unclear/NR

|  | **Selection bias - randomization** | **Selection bias - allocation** | **Blinding** | **Attrition** | **Selective reporting** | **Other** |
| --- | --- | --- | --- | --- | --- | --- |
| **Hong 2011** |  |  |  |  |  |  |
| **Shim 2012** |  |  |  |  |  |  |
| **Meszaros 2012** |  |  |  |  |  |  |

## NRT patch versus Placebo- Change in mental state and adverse events, Follow-up unclear/NR

|  | **Selection bias - randomization** | **Selection bias - allocation** | **Blinding** | **Attrition** | **Selective reporting** | **Other** |
| --- | --- | --- | --- | --- | --- | --- |
| **Dalack 1999** |  |  |  |  |  |  |
| **Hartman 1991** |  |  |  |  |  |  |

## NRT patch versus Placebo- Adverse events, Follow-up unclear/NR

|  | **Selection bias** |  | **Blinding** | **Attrition** | **Selective reporting** | **Other** |
| --- | --- | --- | --- | --- | --- | --- |
| **Dalack 1999** |  |  |  |  |  |  |

## Individual smoking cessation intervention (cognitive behavioural therapy and motivational interviewing) plus NRT patch versus Routine care- Abstinence/cessation, 6 months, 12 months, 4 years

|  | **Selection bias - randomization** | **Selection bias - allocation** | **Blinding** | **Attrition** | **Selective reporting** | **Other** |
| --- | --- | --- | --- | --- | --- | --- |
| **Baker 2006** |  |  |  |  |  |  |

## Individual smoking cessation intervention (cognitive behavioural therapy and motivational interviewing) plus NRT patch versus Routine care- Reduction in cigarettes/day of >50% of baseline, 6 months, 12 months and 4 year follow-up

|  | **Selection bias - randomization** | **Selection bias - allocation** | **Blinding** | **Attrition** | **Selective reporting** | **Other** |
| --- | --- | --- | --- | --- | --- | --- |
| **Baker 2006** |  |  |  |  |  |  |

# Van der Meer 2013 {1223}

## Bupropion versus Placebo (current depression)- Abstinence/cessation, 6-12 months

|  | **Selection bias - randomization** | **Selection bias - allocation** | **Blinding** | **Attrition bias** | **Weight (%)** |
| --- | --- | --- | --- | --- | --- |
| **Brown 2007** |  |  |  |  | 14.5 |
| **Catley 2005** |  |  |  |  | 50.5 |
| **Levine 2010** |  |  |  |  | 2.4 |
| **Thorndike 2008** |  |  |  |  | 23.9 |
| **Schnoll 2010** |  |  |  |  | 8.8 |

## Bupropion versus Placebo (past depression)- Abstinence/cessation, 6-12 months

|  | **Selection bias - randomization** | **Selection bias - allocation** | **Blinding** | **Attrition bias** | **Weight (%)** |
| --- | --- | --- | --- | --- | --- |
| **Brown 2007** |  |  |  |  | 24.2 |
| **Hayford 1999** |  |  |  |  | 10.1 |
| **Piper 2009** |  |  |  |  | 42.9 |
| **Smith 2003** |  |  |  |  | 22.9 |

## NRT gum versus Placebo (current depression)- Abstinence/cessation, 12 months

|  | **Selection bias - randomization** | **Selection bias - allocation** | **Blinding** | **Attrition** | **Weight (%)** |
| --- | --- | --- | --- | --- | --- |
| **Kinnunen 2008** |  |  |  |  | 100 |

## NRT versus Placebo (past depression)- Abstinence/cessation, 6+ months

|  | **Selection bias - randomization** | **Selection bias - allocation** | **Blinding** | **Attrition** | **Weight** |
| --- | --- | --- | --- | --- | --- |
| **Hall 1996** |  |  |  |  | 10.1 |
| **Piper 2009** |  |  |  |  | 58.4 |
| **Smith 2003** |  |  |  |  | 31.4 |

## Standard treatment plus extended NRT and extended CBT versus Standard treatment (past depression)- Abstinence/cessation, Follow-up unclear/NR

|  | **Selection bias - randomization** | **Selection bias - allocation** | **Blinding** | **Attrition** | **Weight (%)** |
| --- | --- | --- | --- | --- | --- |
| **Hall 2009** |  |  |  |  | 100 |

# Vodoplivec-Jamsek 2012 {1343}

## Mobile phone short message service versus Control- Abstinence/cessation, 6 months

|  | **Selection bias - randomization** | **Selection bias - allocation** | **Blinding** | **Attrition bias - short-term outcomes** | **Attrition bias - longer-term outcomes** | **Selective reporting** | **Other bias** | **Weight (%)** |
| --- | --- | --- | --- | --- | --- | --- | --- | --- |
| **Rodgers 2005** |  |  |  |  |  |  |  | 100 |

## Mobile phone short message service versus Control - Adverse events (rates of car crash), 6 months

|  | **Selection bias - randomization** | **Selection bias - allocation** | **Blinding** | **Attrition bias - short-term outcomes** | **Attrition bias - longer-term outcomes** | **Selective reporting** | **Other bias** | **Weight (%)** |
| --- | --- | --- | --- | --- | --- | --- | --- | --- |
| **Rodgers 2005** |  |  |  |  |  |  |  | 100 |

## Mobile phone short message service versus Control - Adverse events (pain in thumb/finger joint), 6 months

|  | **Selection bias - randomization** | **Selection bias - allocation** | **Blinding** | **Attrition bias - short-term outcomes** | **Attrition bias - longer-term outcomes** | **Selective reporting** | **Other bias** | **Weight (%)** |
| --- | --- | --- | --- | --- | --- | --- | --- | --- |
| **Rodgers 2005** |  |  |  |  |  |  |  | 100 |

# White 2014 {1618}

## Acupuncture versus Sham acupuncture- Abstinence/cessation, 6-12 months

|  | **Selection bias - randomization** | **Selection bias - allocation** | **Performance and detection bias** | **Attrition bias** | **Other** | **Weight (%)** |
| --- | --- | --- | --- | --- | --- | --- |
| **Waite 1998** |  |  |  |  |  | 0.5 |
| **He 1997** |  |  |  |  |  | 0.5 |
| **Martin 1981b** |  |  |  |  |  | 1.9 |
| **White 1998** |  |  |  |  |  | 1.9 |
| **Wu 2007** |  |  |  |  |  | 3.8 |
| **Lamontagne 1980** |  |  |  |  |  | 3.8 |
| **Gillams 1984** |  |  |  |  |  | 3.9 |
| **Martin 1981a** |  |  |  |  |  | 5.8 |
| **Clavel 1992+NG** |  |  |  |  |  | 22.5 |
| **Clavel 1992** |  |  |  |  |  | 25.4 |
| **Vandevenne 1985** |  |  |  |  |  | 30.1 |

## Acupuncture versus Waiting list/no intervention- Abstinence/cessation, 6-12 months

|  | **Selection bias - randomization** | **Selection bias - allocation** | **Performance and detection bias** | **Attrition bias** | **Other** | **Weight (%)** |
| --- | --- | --- | --- | --- | --- | --- |
| **Cottraux 1983** |  |  |  |  |  | 59.9 |
| **Lamontagne 1980** |  |  |  |  |  | 33.3 |
| **Leung 1991** |  |  |  |  |  | 6.8 |

## Continuous auricular stimulation versus Sham stimulation- Abstinence/cessation, 6-12 months

|  | **Selection bias - randomization** | **Selection bias - allocation** | **Performance and detection bias** | **Attrition bias** | **Other** | **Weight (%)** |
| --- | --- | --- | --- | --- | --- | --- |
| **Martin 1981b** |  |  |  |  |  | 15.2 |
| **Martin 1981a** |  |  |  |  |  | 21.8 |
| **Wu 2007** |  |  |  |  |  | 26.4 |
| **Gillams 1984** |  |  |  |  |  | 27 |
| **Waite 1998** |  |  |  |  |  | 4.8 |
| **He 1997** |  |  |  |  |  | 4.9 |

## Laser therapy versus Sham laser- Abstinence/cessation, 6-12 months

|  | **Selection bias - randomization** | **Selection bias - allocation** | **Performance and detection bias** | **Attrition bias** | **Other** |
| --- | --- | --- | --- | --- | --- |
| **Docherty 2003** |  |  |  |  |  |
| **Kerr 2008** |  |  |  |  |  |

## Electrostimulation versus Sham electrostimulation- Abstinence/cessation, 6-12 months

|  | **Selection bias - randomization** | **Selection bias - allocation** | **Performance and detection bias** | **Attrition bias** | **Other** | **Weight (%)** |
| --- | --- | --- | --- | --- | --- | --- |
| **Scheuer 2005** |  |  |  |  |  | 21.1 |
| **Antoniou 2005** |  |  |  |  |  | 78.9 |

# Whittaker 2019 {1803}

## Mobile-phone based intervention versus Usual care- Abstinence/cessation, 6+ months

|  | **Selection bias - randomization** | **Selection bias - allocation** | **Blinding** | **Attrition** | **Other bias** | **Weight (%)** |
| --- | --- | --- | --- | --- | --- | --- |
| **Bock 2013** |  |  |  |  |  | 0.3 |
| **Shelley 2015** |  |  |  |  |  | 0.6 |
| **Gritz 2013** |  |  |  |  |  | 1.5 |
| **Abroms 2014** |  |  |  |  |  | 3.8 |
| **Ferguson 2015** |  |  |  |  |  | 4.6 |
| **Naughton 2014** |  |  |  |  |  | 5.8 |
| **Free 2009** |  |  |  |  |  | 5.9 |
| **Haug 2013** |  |  |  |  |  | 7.8 |
| **Whittaker 2011** |  |  |  |  |  | 9.5 |
| **Borland 2013** |  |  |  |  |  | 10.2 |
| **Rodgers 2005** |  |  |  |  |  | 11.9 |
| **Free 2011** |  |  |  |  |  | 38.1 |
