# Supplementary material for: Effectiveness of smoking cessation interventions among adults: an overview of systematic reviews
Source: Syst Rev. 2024 Jul 12;13:179. doi: 10.1186/s13643-024-02570-9 (PMC11242003; doi:10.1186/s13643-024-02570-9)
Supplement: Supplementary file 11 — Additional file 11. Grading of Recommendations, Assessment, Development and Evaluation (GRADE) Evidence Profile and Summary of Findings (SoF) tables. [file 13643_2024_2570_MOESM11_ESM.docx]

**Additional file 11. GRADE Evidence Profile & Summary of Findings Tables**

Table of Contents

[Barnes 2019 {3836} 6](#_Toc143613673)

[Appendix K Table 1. Hypnotherapy versus Placebo drug: Smoking cessation in smokers motivated/wishing to quit 6](#_Toc143613674)

[Summary of findings: 7](#_Toc143613675)

[Cahill 2010 {1652} 9](#_Toc143613676)

[Appendix K Table 2. Stage-based expert systems or tailored self-help materials versus Assessment only: Smoking cessation in general/mixed population of smokers 9](#_Toc143613677)

[Summary of findings: 11](#_Toc143613678)

[Appendix K Table 3. Stage-based interactive computer programmes versus Usual care: Smoking cessation in general/mixed population of smokers 13](#_Toc143613679)

[Summary of findings: 14](#_Toc143613680)

[Appendix K Table 4. Stage-based telephone counselling versus Usual care: Smoking cessation in general/mixed population of smokers 16](#_Toc143613681)

[Summary of findings: 17](#_Toc143613682)

[Appendix K Table 5. Stage-based individual counselling and/or advice versus Usual care: Smoking cessation in general/mixed population of smokers 18](#_Toc143613683)

[Summary of findings: 19](#_Toc143613684)

[Appendix K Table 6. Stage-based individual counselling or advice versus Assessment only: Smoking cessation in general/mixed population of smokers 21](#_Toc143613685)

[Summary of findings: 22](#_Toc143613686)

[Cahill 2016 {1960} 24](#_Toc143613687)

[Appendix K Table 7. Cytisine versus Placebo: Smoking cessation in smokers motivated/wishing to quit 24](#_Toc143613688)

[Summary of findings: 25](#_Toc143613689)

[Appendix K Table 8. Cytisine versus Placebo: Smoking cessation and adverse events in general/mixed population of smokers 26](#_Toc143613690)

[Summary of findings: 28](#_Toc143613691)

[Appendix K Table 9. Varenicline versus Placebo: Smoking cessation in general/mixed population of smokers 30](#_Toc143613692)

[Summary of findings: 32](#_Toc143613693)

[Appendix K Table 10. Long-term varenicline use versus Placebo: Smoking cessation in general/mixed population of smokers 34](#_Toc143613694)

[Summary of findings: 35](#_Toc143613695)

[Appendix K Table 11. Low-dose varenicline use versus Placebo: Smoking cessation in general/mixed population of smokers 37](#_Toc143613696)

[Summary of findings: 38](#_Toc143613697)

[Appendix K Table 12. Variable dosing of varenicline versus Placebo: Smoking cessation in general/mixed population of smokers 39](#_Toc143613698)

[Summary of findings: 40](#_Toc143613699)

[Appendix K Table 13. Varenicline versus Placebo: Smoking cessation in smokers reducing to quit 42](#_Toc143613700)

[Summary of findings: 43](#_Toc143613701)

[Appendix K Table 14. Varenicline versus Placebo: Smoking cessation in smokers with schizophrenia, bipolar, or other psychiatric disorder 44](#_Toc143613702)

[Summary of findings: 45](#_Toc143613703)

[Appendix K Table 15. Varenicline versus Placebo: Smoking cessation in smokers with depression and motivated/wishing to quit 47](#_Toc143613704)

[Summary of findings: 48](#_Toc143613705)

[Appendix K Table 16. Varenicline versus Placebo: Smoking cessation in smokers who previously failed to quit on varenicline but are motivated/wishing to try quitting again 49](#_Toc143613706)

[Summary of findings: 50](#_Toc143613707)

[Appendix K Table 17. Varenicline versus Placebo: Adverse effects in general/mixed population of smokers 51](#_Toc143613708)

[Summary of findings: 56](#_Toc143613709)

[Farley 2012 {1469} 61](#_Toc143613710)

[Appendix K Table 18. Bupropion versus Placebo: Weight gain in smokers motivated to quit at baseline and abstinent at follow-up 61](#_Toc143613711)

[Summary of findings: 63](#_Toc143613712)

[Appendix K Table 19. NRT versus Placebo: Weight gain in smokers motivated to quit at baseline and abstinent at follow-up 66](#_Toc143613713)

[Summary of findings: 68](#_Toc143613714)

[Appendix K Table 20. Varenicline (2mg/day) versus Placebo: Weight gain in smokers motivated to quit at baseline and abstinent at follow-up 71](#_Toc143613715)

[Summary of findings: 73](#_Toc143613716)

[Appendix K Table 21. Varenicline (1 mg/day) versus Placebo: Weight gain in smokers motivated to quit at baseline and abstinent at follow-up 76](#_Toc143613717)

[Summary of findings: 77](#_Toc143613718)

[Hartmann-Boyce 2018 {332} 79](#_Toc143613719)

[Appendix K Table 22. NRT patch vs Placebo: Smoking cessation in relapsed smokers who are motivated to quit 79](#_Toc143613720)

[Summary of findings: 80](#_Toc143613721)

[Appendix K Table 23. NRT vs Placebo: Adverse events in smokers motivated/wishing to quit 81](#_Toc143613722)

[Summary of findings: 83](#_Toc143613723)

[Hollands 2019 {3841} 85](#_Toc143613724)

[Appendix K Table 24. Interventions to increase adherence to medications for tobacco dependence versus Usual or standard care: Smoking cessation, adverse events, and change in emotional state (anxiety) in smokers motivated/wishing to quit or reduce smoking 85](#_Toc143613725)

[Summary of findings: 87](#_Toc143613726)

[Howes 2020 {96} 90](#_Toc143613727)

[Appendix K Table 25. Bupropion versus Placebo: Smoking cessation and reduction in smokers not motivated/wishing to quit 90](#_Toc143613728)

[Summary of findings: 92](#_Toc143613729)

[Appendix K Table 26. Bupropion versus Placebo: Change in emotional state (depressive symptoms) in general/mixed population of smokers 94](#_Toc143613730)

[Summary of findings: 95](#_Toc143613731)

[Appendix K Table 27. St John’s wort versus Placebo: Smoking cessation in smokers motivated/wishing to quit 96](#_Toc143613732)

[Summary of findings: 97](#_Toc143613733)

[Appendix K Table 28. S-Adenosyl-L-Methionine (SAMe) versus Placebo: Smoking cessation in smokers motivated/wishing to quit 98](#_Toc143613734)

[Summary of findings: 99](#_Toc143613735)

[Lancaster 2017 {539} 100](#_Toc143613736)

[Appendix K Table 29. Individual counselling versus Minimal contact control: Smoking cessation in general/mixed population of smokers 100](#_Toc143613737)

[Summary of findings: 102](#_Toc143613738)

[Lindson-Hawley 2016 {671} 104](#_Toc143613739)

[Appendix K Table 30. NRT versus Placebo: Smoking cessation and reduction in smokers not motivated/wishing to quit 104](#_Toc143613740)

[Summary of findings: 106](#_Toc143613741)

[Appendix K Table 31. Bupropion versus Placebo: Smoking cessation, reduction, and serious adverse events in smokers not motivated/wishing to quit 108](#_Toc143613742)

[Summary of findings: 110](#_Toc143613743)

[Appendix K Table 32. Varenicline versus Placebo: Smoking cessation and adverse events in smokers not motivated/wishing to quit 113](#_Toc143613744)

[Summary of findings: 114](#_Toc143613745)

[Appendix K Table 33. Telephone counselling plus self-help materials versus Usual care: Smoking cessation and reduction in smokers not motivated/wishing to quit 116](#_Toc143613746)

[Summary of findings: 118](#_Toc143613747)

[Appendix K Table 34. Behavioural support (advice) plus NRT plus phone calls versus No Intervention: Smoking cessation and reduction in smokers not motivated/wishing to quit 121](#_Toc143613748)

[Summary of findings: 123](#_Toc143613749)

[Appendix K Table 35. E-cigarette versus Placebo: Smoking cessation, reduction, adverse events, and weight gain in smokers not motivated/wishing to quit 125](#_Toc143613750)

[Summary of findings: 127](#_Toc143613751)

[Livingstone-Banks 2019 {1077} 130](#_Toc143613752)

[Appendix K Table 36. Non-tailored print-based self-help materials (no face-to-face contact) versus No materials/no intervention: Smoking cessation in general/mixed population of smokers 130](#_Toc143613753)

[Summary of findings: 132](#_Toc143613754)

[Appendix K Table 37. Non-tailored print-based self-help materials (no face-to-face contact) versus No materials/no intervention: Smoking cessation in smokers motivated/wishing to quit 134](#_Toc143613755)

[Summary of findings: 135](#_Toc143613756)

[Appendix K Table 38. Non-tailored print-based self-help materials (no face-to-face contact) versus Brief leaflet: Smoking cessation in general/mixed population of smokers 137](#_Toc143613757)

[Summary of findings: 138](#_Toc143613758)

[Appendix K Table 39. Non-tailored print-based self-help materials (with face-to-face contact) versus No intervention or leaflet only: Smoking cessation in general/mixed population of smokers 140](#_Toc143613759)

[Summary of findings: 141](#_Toc143613760)

[Appendix K Table 40. Individually tailored print-based self-help materials (no face-to-face) versus No materials/no intervention: Smoking cessation in general/mixed population of smokers 143](#_Toc143613761)

[Summary of findings: 144](#_Toc143613762)

[Matkin 2019 {1228} 146](#_Toc143613763)

[Appendix K Table 41. Hotline and self-help materials versus Minimal intervention in general/mixed population of smokers 146](#_Toc143613764)

[Summary of findings: 147](#_Toc143613765)

[Appendix K Table 42. Intensive telephone counselling versus Minimal telephone counselling in general/mixed population of smokers 149](#_Toc143613766)

[Summary of findings: 150](#_Toc143613767)

[Appendix K Table 43. Brief motivational telephone counselling versus Usual care telephone call in general/mixed population of smokers 151](#_Toc143613768)

[Summary of findings: 152](#_Toc143613769)

[Appendix K Table 44. Telephone counselling for smoking reduction versus Usual care telephone call in general/mixed population of smokers 153](#_Toc143613770)

[Summary of findings: 154](#_Toc143613771)

[Posadzki 2016 {659} 155](#_Toc143613772)

[Appendix K Table 45. Interactive voice response (IVR) systems versus No intervention: Smoking cessation in general/mixed population of smokers 155](#_Toc143613773)

[Summary of findings: 156](#_Toc143613774)

[Stead 2013 {1998} 158](#_Toc143613775)

[Appendix K Table 46. Physician advice (minimal or intensive interventions) versus No advice (or usual care): Smoking cessation in general/mixed population of smokers 158](#_Toc143613776)

[Summary of findings: 160](#_Toc143613777)

[Appendix K Table 47. Physician advice with follow-up versus Minimal intervention/advice with single visit: Smoking cessation in general/mixed population of smokers 162](#_Toc143613778)

[Summary of findings: 163](#_Toc143613779)

[Appendix K Table 48. Intensive advice versus Minimal advice: Smoking cessation in general/mixed population of smokers 165](#_Toc143613780)

[Summary of findings: 166](#_Toc143613781)

[Stead 2016 {1356} 167](#_Toc143613782)

[Appendix K Table 49. Combined pharmacotherapy and behavioural interventions versus Usual care or minimal intervention: Smoking cessation in the general/mixed population 167](#_Toc143613783)

[Summary of findings: 169](#_Toc143613784)

[Appendix K Table 50. Combined pharmacotherapy and behavioural interventions versus Usual care or no intervention: Smoking cessation in general/mixed population of smokers 171](#_Toc143613785)

[Summary of findings: 172](#_Toc143613786)

[Stead 2017 {538} 174](#_Toc143613787)

[Appendix K Table 51. Group therapy vs No intervention: Smoking cessation in general/mixed population of smokers 174](#_Toc143613788)

[Summary of findings: 175](#_Toc143613789)

[Taylor 2017 {411} 177](#_Toc143613790)

[Appendix K Table 52. Interactive and tailored internet interventions versus Non-active controls: Smoking cessation in general/mixed population of smokers 177](#_Toc143613791)

[Summary of findings: 179](#_Toc143613792)

[Appendix K Table 53. Internet plus behavioural support versus Non-internet-based non-active controls: Smoking cessation in general/mixed population of smokers 181](#_Toc143613793)

[Summary of findings: 182](#_Toc143613794)

[Tsoi 2013 {1698} 184](#_Toc143613795)

[Appendix K Table 54. Bupropion vs Placebo: Smoking cessation, reduction, change in mental state, and adverse events in smokers with schizophrenia or schizoaffective disorder 184](#_Toc143613796)

[Summary of findings: 189](#_Toc143613797)

[Appendix K Table 55. Varenicline vs Placebo: Smoking cessation, reduction, change in mental state, and adverse events in smokers with schizophrenia or schizoaffective disorder 195](#_Toc143613798)

[Summary of findings: 199](#_Toc143613799)

[Appendix K Table 56. NRT patch vs Placebo patch: Change in mental state and adverse events in smokers with schizophrenia or schizoaffective disorder 204](#_Toc143613800)

[Summary of findings: 206](#_Toc143613801)

[Appendix K Table 57. Individual smoking cessation intervention (cognitive behavioural therapy and motivational interviewing) plus NRT patch versus Routine care: Smoking cessation and reduction in smokers with schizophrenia or schizoaffective disorder 208](#_Toc143613802)

[Summary of findings: 210](#_Toc143613803)

[Van der Meer 2013 {1223} 212](#_Toc143613804)

[Appendix K Table 58. Bupropion versus Placebo: Smoking cessation in smokers with current depression 212](#_Toc143613805)

[Summary of findings: 213](#_Toc143613806)

[Appendix K Table 59. Bupropion versus Placebo: Smoking cessation in smokers with past depression 215](#_Toc143613807)

[Summary of findings: 216](#_Toc143613808)

[Appendix K Table 60. NRT gum versus Placebo: Smoking cessation in smokers with current depression 218](#_Toc143613809)

[Summary of findings: 219](#_Toc143613810)

[Appendix K Table 61. NRT versus Placebo: Smoking cessation in smokers with past depression 220](#_Toc143613811)

[Summary of findings: 221](#_Toc143613812)

[Appendix K Table 62. Standard treatment plus extended NRT and extended CBT versus Standard treatment: Smoking cessation in smokers with past depression 223](#_Toc143613813)

[Summary of findings: 224](#_Toc143613814)

[Vodoplivec-Jamsek 2012 {1343} 226](#_Toc143613815)

[Appendix K Table 63. Mobile phone short message service vs Control: Smoking cessation in smokers motivated/wishing to quit 226](#_Toc143613816)

[Summary of findings: 228](#_Toc143613817)

[White 2014 {1618} 230](#_Toc143613818)

[Appendix K Table 64. Acupuncture versus Sham: Smoking cessation in smokers motivated/wishing to quit 230](#_Toc143613819)

[Summary of findings: 232](#_Toc143613820)

[Appendix K Table 65. Acupuncture versus Waiting list/no intervention: Smoking cessation in smokers motivated/wishing to quit 234](#_Toc143613821)

[Summary of findings: 235](#_Toc143613822)

[Appendix K Table 66. Continuous auricular stimulation versus Sham: Smoking cessation in smokers motivated/wishing to quit 237](#_Toc143613823)

[Summary of findings: 238](#_Toc143613824)

[Appendix K Table 67. Laser therapy versus Sham: Smoking cessation in smokers motivated/wishing to quit 240](#_Toc143613825)

[Summary of findings: 241](#_Toc143613826)

[Appendix K Table 68. Electrostimulation versus Sham: Smoking cessation in smokers motivated/wishing to quit 243](#_Toc143613827)

[Summary of findings: 244](#_Toc143613828)

[Whittaker 2019 {1803} 245](#_Toc143613829)

[Appendix K Table 69. Mobile phone-based interventions versus Usual care: Smoking cessation in smokers motivated/wishing to quit 245](#_Toc143613830)

[Summary of findings: 247](#_Toc143613831)

Part I: Overview of reviews

# Barnes 2019 {3836}

## Appendix K Table 1. Hypnotherapy versus Placebo drug: Smoking cessation in smokers motivated/wishing to quit

| Hypnotherapy compared to placebo drug alone in smokers motivated/wishing to quit  **Bibliography:** Barnes 2019; Date of last search: July 2018 | | | | | | | | | | | |
| --- | --- | --- | --- | --- | --- | --- | --- | --- | --- | --- | --- |
| **Certainty assessment** | | | | | | | **Summary of findings** | | | | |
| **№ of participants (studies) Follow-up** | **Risk of bias** | **Inconsistency** | **Indirectness** | **Imprecision** | **Publication bias** | **Overall certainty of evidence** | **Study event rates (%)** | | **Relative effect (95% CI)** | **Anticipated absolute effects** | |
|  |  |  |  |  |  |  | **With placebo drug alone** | **With Hypnotherapy** |  | **Risk with placebo drug alone** | **Risk difference with Hypnotherapy** |
| **Abstinence/Cessation (follow up: 12 months)** ^a,b,c^  Outcome measurement: point prevalence 100% studies  Biochemical validation: 0% studies | | | | | | | | | | | |
| 114 (1 RCT) | very serious ^d^ | not serious ^e^ | not serious ^f^ | very serious ^g^ | none ^h^ | ⨁◯◯◯ VERY LOW | 6/57 (10.5%) | 5/57 (8.8%) | RR 0.83 (0.27 to 2.58) | 105 per 1,000 | **18 fewer per 1,000** (from 77 fewer to 166 more) |

**CI:** Confidence interval; **RR:** Risk ratio

#### Explanations

a. Population eligibility criterion is people wishing to quit. However, motivation or interest in quitting is not explicitly stated in the review evidence table for the only trial included in this analysis. Study characteristics are not adequately reported for this trial but appears to have recruited general smokers.

b. Hypnotherapy: Number of sessions and duration not reported. No co-interventions provided.

c. Placebo drug: No co-interventions provided.

d. The only study in this analysis is at high risk of bias due to lack of biochemical validation. The trial is also at unclear risk for selection bias, performance/detection bias, and baseline imbalance. We downrate this domain by -2.0.

e. Not applicable - single study.

f. No indirectness.

g. Confidence interval encompasses harm (moderate) to benefit (large). The optimal information size is not met (total of 11 events) and inadequate sample size (<2000 participants). We downrate this domain by -2.0.

h. Search was not completely comprehensive but the only trial in this analysis reports negative findings. We do not downrate this domain.

| Summary of findings: | | | | | | |
| --- | --- | --- | --- | --- | --- | --- |
| **Hypnotherapy compared to placebo drug alone in smokers motivated to quit/wishing to quit** | | | | | | |
| **Patient or population**: Smokers motivated/wishing to quit  **Setting**: No restriction  **Intervention**: Hypnotherapy  **Comparison**: Placebo drug alone | | | | | | |
| Outcomes | **Anticipated absolute effects^*^** (95% CI) | | Relative effect (95% CI) | № of participants  (studies) | Certainty of the evidence (GRADE) | Comments |
|  | **Risk with placebo drug alone** | **Risk with Hypnotherapy** |  |  |  |  |
| Abstinence/Cessation ^a,b,c^  Outcome measurement: point prevalence 100% studies  Biochemical validation: 0% studies  Follow up: 12 months | 105 per 1,000 | **87 per 1,000** (28 to 272) | RR 0.83 (0.27 to 2.58) | 114 (1 RCT) | ⨁◯◯◯ VERY LOW ^d,e,f,g,h^ | AMSTAR-2: Critically low.  Date of last search: July 2010.  Not GRADEd by review authors. |
| ***The risk in the intervention group** (and its 95% confidence interval) is based on the assumed risk in the comparison group and the **relative effect** of the intervention (and its 95% CI).   **CI:** Confidence interval; **RR:** Risk ratio | | | | | | |
| **GRADE Working Group grades of evidence** **High certainty:** We are very confident that the true effect lies close to that of the estimate of the effect **Moderate certainty:** We are moderately confident in the effect estimate: The true effect is likely to be close to the estimate of the effect, but there is a possibility that it is substantially different **Low certainty:** Our confidence in the effect estimate is limited: The true effect may be substantially different from the estimate of the effect **Very low certainty:** We have very little confidence in the effect estimate: The true effect is likely to be substantially different from the estimate of effect | | | | | | |

#### Explanations

a. Population eligibility criterion is people wishing to quit. However, motivation or interest in quitting is not explicitly stated in the review evidence table for the only trial included in this analysis. Study characteristics are not adequately reported for this trial but appears to have recruited general smokers.

b. Hypnotherapy: Number of sessions and duration not reported. No co-interventions provided.

c. Placebo drug: No co-interventions provided.

d. The only study in this analysis is at high risk of bias due to lack of biochemical validation. The trial is also at unclear risk for selection bias, performance/detection bias, and baseline imbalance. We downrate this domain by -2.0.

e. Not applicable - single study.

f. No indirectness.

g. Confidence interval encompasses harm (moderate) to benefit (large). The optimal information size is not met (total of 11 events) and inadequate sample size (<2000 participants). We downrate this domain by -2.0.

h. Search was not completely comprehensive but the only trial in this analysis reports negative findings. We do not downrate this domain.

# Cahill 2010 {1652}

## Appendix K Table 2. Stage-based expert systems or tailored self-help materials versus Assessment only: Smoking cessation in general/mixed population of smokers

| Stage-based expert systems or tailored self-help materials compared to assessment only in general/mixed population of smokers  **Bibliography:** Cahill 2010; Date of last search: August 2010 | | | | | | | | | | | |
| --- | --- | --- | --- | --- | --- | --- | --- | --- | --- | --- | --- |
| **Certainty assessment** | | | | | | | **Summary of findings** | | | | |
| **№ of participants (studies) Follow-up** | **Risk of bias** | **Inconsistency** | **Indirectness** | **Imprecision** | **Publication bias** | **Overall certainty of evidence** | **Study event rates (%)** | | **Relative effect (95% CI)** | **Anticipated absolute effects** | |
|  |  |  |  |  |  |  | **With assessment only** | **With Stage-based expert systems or tailored self-help materials** |  | **Risk with assessment only** | **Risk difference with Stage-based expert systems or tailored self-help materials** |
| **Abstinence/Cessation (follow up: 6+ months)** ^a,b,c^  Outcome measurement: point prevalence 40%, continuous/sustained abstinence 10%, prolonged abstinence 50% studies  Biochemical validation: 0% studies | | | | | | | | | | | |
| 13597 (10 RCTs) | very serious ^d^ | serious ^e^ | not serious ^f^ | not serious ^g^ | none ^h^ | ⨁◯◯◯ VERY LOW | 462/7228 (6.4%) | 517/6369 (8.1%) | **RR 1.35** (1.19 to 1.52) | 64 per 1,000 | **22 more per 1,000** (from 12 more to 33 more) |
| **Abstinence/Cessation (follow up: 14 months)** ^i,j,k^  Outcome measurement: continuous/sustained abstinence 100% studies  Biochemical validation: 0% studies | | | | | | | | | | | |
| Unclear/NR (1 RCT) ^l^ | very serious ^m^ | not serious ^n^ | not serious ^o^ | serious to very serious ^p^ | none ^q^ | ⨁◯◯◯ VERY LOW | Significant difference between groups with more quitters in the intervention arm (OR 3.74, 95% CI: NR). | | | | |

**CI:** Confidence interval; **NR:** Not reported; **RR:** Risk ratio

#### Explanations

a. One study recruited smokers and ex-smokers 14-17 years of age. Remaining studies recruited general smokers. Motivation to quit was not required in majority of studies.

b. Intervention: The intervention involved personalised reports or letters matched to stage of change. Letters/reports often produced electronically according to questionnaires or interviews. The intervention may include self-help materials (tailored or standard) and/or follow-up phone calls. Behavioural co-intervention provided in one trial. In another trial, 25% of the intervention arm used NRT by 2-year follow-up. 4% of participants in the intervention arm received specialized behavioural counselling (i.e., motivational interviewing).

c. Control: Review authors consider 'assessment only' as a no intervention control. Across trials, control conditions included 'assessment only', no intervention, a letter confirming no self-help information will be sent, and non-smoking related healthcare intervention including 3-5 minute dietary advice. In one trial, control participants did not receive smoking cessation advice but 21% had used NRT by 2-year follow-up. No control participants in this analysis received specialized behavioural counselling.

d. 90% of the evidence is at high risk of bias for one or more domains (mainly biochemical validation but also selection bias) and at unclear risk of bias for two or more domains (i.e., selection, performance/detection, and attrition bias). We downrate this domain by -2.0.

e. Variation in point estimates but confidence intervals mostly overlap (I2=46%, p=0.05). We downrate this domain by -1.0.

f. 6% of the evidence is indirect (study recruiting smokers and ex-smokers <18 years old). We do not downrate this domain.

g. Confidence interval encompasses small but important benefit. The optimal information size is met (total of 979 events) and adequate sample size. We do not downrate this domain.

h. Funnel plot including all studies in the review is not suggestive of publication bias. Search is not completely comprehensive but mix of results across trials. We do not downrate this domain.

i. The only study in this analysis recruits general smokers. Motivation to quit was not required.

j. Intervention: Computer-generated tailored letter addressing (a) outcomes of smoking and quitting and (b) self-efficacy, active skills to quit, boosting confidence, coping skills. No co-interventions provided.

k. Control: Participants received letter confirming no self-help information would be sent. No co-intervention provided.

l. Number of participants included in this analysis unclear/NR. 386 participants randomized to intervention arm and 385 to control.

m. Study is at high risk of bias due to lack of biochemical validation and unclear risk of selection and performance/detection bias. We downrate this domain by -2.0.

n. Not applicable - single study.

o. No indirectness.

p. Unable to assess confidence intervals. The number of participants included in this analysis unclear, but 771 participants randomized and the optimal information size cannot be met. We cannot rate this domain.

q. Search is not completely comprehensive, and the study reports positive findings. However, lack of negative trials may be reflective of an under-researched area as opposed to suppression of findings. Given not enough information exists to strongly suspect publication bias, we do not downrate this domain.

| Summary of findings: | | | | | | |
| --- | --- | --- | --- | --- | --- | --- |
| **Stage-based expert systems or tailored self-help materials compared to assessment only in general/mixed population of smokers** | | | | | | |
| **Patient or population**: General/Mixed population of smokers  **Setting**: No restriction  **Intervention**: Stage-based expert systems or tailored self-help materials  **Comparison**: Assessment only | | | | | | |
| Outcomes | **Anticipated absolute effects^*^** (95% CI) | | Relative effect (95% CI) | № of participants  (studies) | Certainty of the evidence (GRADE) | Comments |
|  | **Risk with assessment only** | **Risk with Stage-based expert systems or tailored self-help materials** |  |  |  |  |
| Abstinence/Cessation ^a,b,c^  Outcome measurement: point prevalence 40%, continuous/sustained abstinence 10%, prolonged abstinence 50% studies  Biochemical validation: 0% studies  Follow up: 6+ months | 64 per 1,000 | **86 per 1,000** (76 to 97) | **RR 1.35** (1.19 to 1.52) | 13597 (10 RCTs) | ⨁◯◯◯ VERY LOW ^d,e,f,g,h^ | Abstinence defined as point prevalence at 6 or 12 months: RR 1.20 (95% CI: 1.09, 1.33), 10 studies, n=13676; I2=50%, p=0.03.  Per protocol analysis: RR 1.39 (95% CI: 1.24, 1.57), 10 studies, n=9833; I2=50%, p=0.03.  Fixed effects analysis.  AMSTAR-2: Critically low.  Date of last search: August 2010.  Not GRADEd by review authors. |
| Abstinence/Cessation ^i,j,k^  Outcome measurement: Continuous/sustained abstinence 100% studies  Biochemical validation: 0% studies  Follow up: 14 months | Significant difference between groups with more quitters in the intervention arm (OR 3.74, 95% CI: NR). | |  | Unclear/NR  (1 RCT) ^l^ | ⨁◯◯◯ VERY LOW ^m,n,o,p,q^ | AMSTAR-2: Critically low.  Date of last search: August 2010.  Not GRADEd by review authors. |
| ***The risk in the intervention group** (and its 95% confidence interval) is based on the assumed risk in the comparison group and the **relative effect** of the intervention (and its 95% CI).   **CI:** Confidence interval; **RR:** Risk ratio | | | | | | |
| **GRADE Working Group grades of evidence** **High certainty:** We are very confident that the true effect lies close to that of the estimate of the effect **Moderate certainty:** We are moderately confident in the effect estimate: The true effect is likely to be close to the estimate of the effect, but there is a possibility that it is substantially different **Low certainty:** Our confidence in the effect estimate is limited: The true effect may be substantially different from the estimate of the effect **Very low certainty:** We have very little confidence in the effect estimate: The true effect is likely to be substantially different from the estimate of effect | | | | | | |

#### Explanations

a. One study recruited smokers and ex-smokers 14-17 years of age. Remaining studies recruited general smokers. Motivation to quit was not required in majority of studies.

b. Intervention: The intervention involved personalised reports or letters matched to stage of change. Letters/reports often produced electronically according to questionnaires or interviews. The intervention may include self-help materials (tailored or standard) and/or follow-up phone calls. Behavioural co-intervention provided in one trial. In another trial, 25% of the intervention arm used NRT by 2-year follow-up. 4% of participants received specialized behavioural counselling (i.e., motivational interviewing).

c. Control: Review authors consider 'assessment only' as a no intervention control. Across trials, control conditions included 'assessment only', no intervention, a letter confirming no self-help information will be sent, and non-smoking related healthcare intervention including 3-5 minute dietary advice. In one trial, control participants did not receive smoking cessation advice but 21% had used NRT by 2-year follow-up. No control participants in this analysis received specialized behavioural counselling.

d. 90% of the evidence is at high risk of bias for one or more domains (mainly biochemical validation but also selection bias) and at unclear risk of bias for two or more domains (i.e., selection, performance/detection, and attrition bias). We downrate this domain by -2.0.

e. Variation in point estimates but confidence intervals mostly overlap (I2=46%, p=0.05). We downrate this domain by -1.0.

f. 6% of the evidence is indirect (study recruiting smokers and ex-smokers <18 years old). We do not downrate this domain.

h. Funnel plot including all studies in the review is not suggestive of publication bias. Search is not completely comprehensive but mix of results across trials. We do not downrate this domain.

i. The only study in this analysis recruits general smokers. Motivation to quit was not required.

j. Intervention: Computer-generated tailored letter addressing (a) outcomes of smoking and quitting and (b) self-efficacy, active skills to quit, boosting confidence, coping skills. No co-interventions provided.

k. Control: Participants received letter confirming no self-help information would be sent. No co-intervention provided.

l. Number of participants included in this analysis unclear/NR. 386 participants randomized to intervention arm and 385 to control.

m. Study is at high risk of bias due to lack of biochemical validation and unclear risk of selection and performance/detection bias. We downrate this domain by -2.0.

n. Not applicable - single study.

o. No indirectness.

p. Unable to assess confidence intervals. The number of participants included in this analysis unclear, but 771 participants randomized and the optimal information size cannot be met. We cannot rate this domain.

q. Search is not completely comprehensive, and the study reports positive findings. However, lack of negative trials may be reflective of an under-researched area as opposed to suppression of findings. Given not enough information exists to strongly suspect publication bias, we do not downrate this domain.

## Appendix K Table 3. Stage-based interactive computer programmes versus Usual care: Smoking cessation in general/mixed population of smokers

| Stage-based interactive computer programmes compared to usual care in general/mixed population of smokers  **Bibliography:** Cahill 2010; Date of last search: August 2010 | | | | | | | | | | | |
| --- | --- | --- | --- | --- | --- | --- | --- | --- | --- | --- | --- |
| **Certainty assessment** | | | | | | | **Summary of findings** | | | | |
| **№ of participants (studies) Follow-up** | **Risk of bias** | **Inconsistency** | **Indirectness** | **Imprecision** | **Publication bias** | **Overall certainty of evidence** | **Study event rates (%)** | | **Relative effect (95% CI)** | **Anticipated absolute effects** | |
|  |  |  |  |  |  |  | **With usual care** | **With Stage-based interactive computer programmes** |  | **Risk with usual care** | **Risk difference with Stage-based interactive computer programmes** |
| **Abstinence/Cessation (follow up: 12+ months)** ^a,b,c^  Outcome measurement: point prevalence 50%, continuous/sustained abstinence 50% studies  Biochemical validation: 0% studies | | | | | | | | | | | |
| 1702 (2 RCTs) ^d^ | very serious ^e^ | not serious ^f^ | serious ^g^ | very serious ^h^ | none ^i^ | ⨁◯◯◯  VERY LOW | 58/831 (7.0%) | 68/871 (7.8%) | **RR 1.14** (0.81 to 1.59) | 70 per 1,000 | **10 more per 1,000** (from 13 fewer to 41 more) |

**CI:** Confidence interval; **RR:** Risk ratio

#### Explanations

a. One study recruited pregnant women and the other enrolled students 13-14 years old. Motivation to quit not required in one of the studies; unclear for other.

b. Intervention typically delivered within fixed time frame and sessions may include supervision with oral and written feedback provided. No co-interventions provided in either trial.

c. Comparator described as usual care by review authors. In one study, usual care group received advice plus self-help materials. In the second school-based study, usual care was 'standard English curriculum on smoking i.e. smoking quizzes and advice on persuading people to quit'. No co-interventions provided in either study.

d. Both studies are cluster randomized trials.

e. Both trials are at high risk due to lack of biochemical validation and one also at high risk for selection bias. Both at unclear for performance/detection bias. We downrate this domain by -1.5 but also reflect partial downrating (-0.5) from the indirectness domain here.

f. Little variation in point estimates and confidence intervals overlap (I2=0%, p=0.96). We do not downrate this domain.

g. 78.5% of the evidence is indirect (students <18 years old). We downrate this domain by -1.5 but reflect part of this downrating in the risk of bias domain.

h. Confidence interval encompasses both harm (small but important) and benefit (small but important). The optimal information size not met (total of 126 events) and inadequate sample size (<2000 participants). We downrate this domain by -2.0.

i. Funnel plot including all studies in the review is not suggestive of publication bias. Search is not completely comprehensive but both trials report negative findings. We do not downrate this domain.

| Summary of findings: | | | | | | |
| --- | --- | --- | --- | --- | --- | --- |
| **Stage-based interactive computer programmes compared to usual care in general/mixed population of smokers** | | | | | | |
| **Patient or population**: General/mixed population of smokers  **Setting**: No restriction  **Intervention**: Stage-based interactive computer programmes  **Comparison**: Usual care | | | | | | |
| Outcomes | **Anticipated absolute effects^*^** (95% CI) | | Relative effect (95% CI) | № of participants  (studies) | Certainty of the evidence (GRADE) | Comments |
|  | **Risk with usual care** | **Risk with Stage-based interactive computer programmes** |  |  |  |  |
| Abstinence/Cessation ^a,b,c^  Outcome measurement: point prevalence 50%, continuous/sustained abstinence 50% studies  Biochemical validation: 0% studies  Follow up: 12+ months | 70 per 1,000 | **80 per 1,000** (57 to 111) | **RR 1.14** (0.81 to 1.59) | 1702 (2 RCTs) ^d^ | ⨁◯◯◯  VERY LOW ^e,f,g,h,i^ | Evidence is applicable to a mixed population of adolescents and pregnant women. Accumulation of more studies required to consider applicability to a general adult population.  Adjustment for clustering: RR 1.14 (95% CI: 0.79, 1.63); I2=0%, p=0.96.  Abstinence defined as point prevalence at 6 or 12 months: RR 1.25 (95% CI: 0.93, 1.67), 2 trials, n=1702; I2=0%, p=0.74.  Per protocol analysis: RR 1.16 (95% CI: 0.83, 1.61), 2 trials, n=1347; I2=0%, p=0.89.  Fixed effects analysis.  AMSTAR-2: Critically low.  Date of last search: August 2010.  Not GRADEd by review authors. |
| ***The risk in the intervention group** (and its 95% confidence interval) is based on the assumed risk in the comparison group and the **relative effect** of the intervention (and its 95% CI).   **CI:** Confidence interval; **RR:** Risk ratio | | | | | | |
| **GRADE Working Group grades of evidence** **High certainty:** We are very confident that the true effect lies close to that of the estimate of the effect **Moderate certainty:** We are moderately confident in the effect estimate: The true effect is likely to be close to the estimate of the effect, but there is a possibility that it is substantially different **Low certainty:** Our confidence in the effect estimate is limited: The true effect may be substantially different from the estimate of the effect **Very low certainty:** We have very little confidence in the effect estimate: The true effect is likely to be substantially different from the estimate of effect | | | | | | |

#### Explanations

a. One study recruited pregnant women and the other enrolled students 13-14 years old. Motivation to quit not required in one of the studies; unclear for other.

b. Intervention typically delivered within fixed time frame and sessions may include supervision with oral and written feedback provided. No co-interventions provided in either trial.

c. Comparator described as usual care by review authors. In one study, usual care group received advice plus self-help materials. In the second school-based study, usual care was 'standard English curriculum on smoking i.e. smoking quizzes and advice on persuading people to quit'. No co-interventions provided in either study.

d. Both studies are cluster randomized trials.

e. Both trials are at high risk due to lack of biochemical validation and one also at high risk for selection bias. Both at unclear for performance/detection bias. We downrate this domain by -1.5 but also reflect partial downrating (-0.5) from the indirectness domain here.

f. Little variation in point estimates and confidence intervals overlap (I2=0%, p=0.96). We do not downrate this domain.

g. 78.5% of the evidence is indirect (students <18 years old). We downrate this domain by -1.5 but reflect part of this downrating in the risk of bias domain.

h. Confidence interval encompasses both harm (small but important) and benefit (small but important). The optimal information size not met (total of 126 events) and inadequate sample size (<2000 participants). We downrate this domain by -2.0.

i. Funnel plot including all studies in the review is not suggestive of publication bias. Search is not completely comprehensive but both trials report negative findings. We do not downrate this domain.

## Appendix K Table 4. Stage-based telephone counselling versus Usual care: Smoking cessation in general/mixed population of smokers

| Stage-based telephone counselling compared to usual care in general/mixed population of smokers  **Bibliography:** Cahill 2010; Date of last search: August 2010 | | | | | | | | | | | |
| --- | --- | --- | --- | --- | --- | --- | --- | --- | --- | --- | --- |
| **Certainty assessment** | | | | | | | **Summary of findings** | | | | |
| **№ of participants (studies) Follow-up** | **Risk of bias** | **Inconsistency** | **Indirectness** | **Imprecision** | **Publication bias** | **Overall certainty of evidence** | **Study event rates (%)** | | **Relative effect (95% CI)** | **Anticipated absolute effects** | |
|  |  |  |  |  |  |  | **With usual care** | **With Stage-based telephone counselling** |  | **Risk with usual care** | **Risk difference with Stage-based telephone counselling** |
| **Abstinence/Cessation (follow up: 12 months)** ^a,b,c^  Outcome measurement: point prevalence 100% studies  Biochemical validation: 0% studies | | | | | | | | | | | |
| 318 (1 RCT) | serious ^d^ | not serious ^e^ | not serious ^f^ | very serious ^g^ | none ^h^ | ⨁◯◯◯  VERY LOW | 9/149 (6.0%) | 13/169 (7.7%) | **RR 1.27** (0.56 to 2.89) | 60 per 1,000 | **16 more per 1,000** (from 27 fewer to 114 more) |

**CI:** Confidence interval; **RR:** Risk ratio

#### Explanations

a. The only study in this analysis recruited general smokers. Unclear whether motivation to quit was required.

b. Intervention: Assessments and counselling delivered over the phone with advice tailored to stage of change. Counselling was based on specialized approaches (5As, motivational interviewing, 5Rs). Those attempting to quit received quit pack and encouraged to use NRT. Those not ready to quit received motivational intervention to advance stage of change.

c. Control: Participants received usual care including free quit kits. No co-interventions provided.

d. Study is at high risk of bias due to lack of biochemical validation and unclear risk of bias for performance/detection bias. We downrate this domain by -1.5.

e. Not applicable - single study

f. No indirectness.

g. Confidence interval encompasses both harm (moderate) and benefit (moderate). The optimal information size not met (total of 22 events), inadequate sample size (<2000 participants). We downrate this domain by -2.0.

h. Funnel plot including all studies in the review not suggestive of publication bias. Search is not completely comprehensive but the only study in this analysis reports negative findings. We do not downrate this domain.

| Summary of findings: | | | | | | |
| --- | --- | --- | --- | --- | --- | --- |
| **Stage-based telephone counselling compared to usual care in general/mixed population of smokers** | | | | | | |
| **Patient or population**: General/mixed population of smokers  **Setting**: No restriction  **Intervention**: Stage-based telephone counselling  **Comparison**: Usual care | | | | | | |
| Outcomes | **Anticipated absolute effects^*^** (95% CI) | | Relative effect (95% CI) | № of participants  (studies) | Certainty of the evidence (GRADE) | Comments |
|  | **Risk with usual care** | **Risk with Stage-based telephone counselling** |  |  |  |  |
| Abstinence/Cessation ^a,b,c^  Outcome measurement: point prevalence 100% studies  Biochemical validation: 0% studies  Follow up: 12 months | 60 per 1,000 | **77 per 1,000** (34 to 175) | **RR 1.27** (0.56 to 2.89) | 318 (1 RCT) | ⨁◯◯◯  VERY LOW ^d,e,f,g,h^ | Per protocol analysis: RR 1.09 (95% CI: 0.49, 2.43), 1 trial, n=205.  AMSTAR-2: Critically low.  Date of last search: August 2010.  Not GRADEd by review authors. |
| ***The risk in the intervention group** (and its 95% confidence interval) is based on the assumed risk in the comparison group and the **relative effect** of the intervention (and its 95% CI).   **CI:** Confidence interval; **RR:** Risk ratio | | | | | | |
| **GRADE Working Group grades of evidence** **High certainty:** We are very confident that the true effect lies close to that of the estimate of the effect **Moderate certainty:** We are moderately confident in the effect estimate: The true effect is likely to be close to the estimate of the effect, but there is a possibility that it is substantially different **Low certainty:** Our confidence in the effect estimate is limited: The true effect may be substantially different from the estimate of the effect **Very low certainty:** We have very little confidence in the effect estimate: The true effect is likely to be substantially different from the estimate of effect | | | | | | |

#### Explanations

a. The only study in this analysis recruited general smokers. Unclear whether motivation to quit was required.

b. Intervention: Assessments and counselling delivered over the phone with advice tailored to stage of change. Counselling was based on specialized approaches (5As, motivational interviewing, 5Rs). Those attempting to quit received quit pack and encouraged to use NRT. Those not ready to quit received motivational intervention to advance stage of change.

c. Control: Participants received usual care including free quit kits. No co-interventions provided.

d. Study is at high risk of bias due to lack of biochemical validation and unclear risk of bias for performance/detection bias. We downrate this domain by -1.5.

e. Not applicable - single study

f. No indirectness.

g. Confidence interval encompasses both harm (moderate) and benefit (moderate). The optimal information size not met (total of 22 events), inadequate sample size (<2000 participants). We downrate this domain by -2.0.

h. Funnel plot including all studies in the review not suggestive of publication bias. Search is not completely comprehensive but the only study in this analysis reports negative findings. We do not downrate this domain.

## Appendix K Table 5. Stage-based individual counselling and/or advice versus Usual care: Smoking cessation in general/mixed population of smokers

| Stage-based individual counselling and/or advice compared to usual care in general/mixed population of smokers  **Bibliography:** Cahill 2010; Date of last search: August 2010 | | | | | | | | | | | |
| --- | --- | --- | --- | --- | --- | --- | --- | --- | --- | --- | --- |
| **Certainty assessment** | | | | | | | **Summary of findings** | | | | |
| **№ of participants (studies) Follow-up** | **Risk of bias** | **Inconsistency** | **Indirectness** | **Imprecision** | **Publication bias** | **Overall certainty of evidence** | **Study event rates (%)** | | **Relative effect (95% CI)** | **Anticipated absolute effects** | |
|  |  |  |  |  |  |  | **With usual care** | **With Stage-based individual counselling and/or advice** |  | **Risk with usual care** | **Risk difference with Stage-based individual counselling and/or advice** |
| **Abstinence/Cessation (follow up: 6+ months)** ^a,b,c^  Outcome measurement: point prevalence 57%, continuous/sustained abstinence 43% studies;  Biochemical validation: 43% | | | | | | | | | | | |
| 3293 (7 RCTs) ^d^ | serious ^e^ | not serious ^f^ | not serious ^g^ | not serious ^h^ | none ^i^ | ⨁⨁⨁◯  MODERATE | 175/1596 (11.0%) | 224/1697 (13.2%) | **RR 1.19** (0.99 to 1.42) | 110 per 1,000 | **21 more per 1,000** (from 1 fewer to 46 more) |

**CI:** Confidence interval; **RR:** Risk ratio

#### Explanations

a. Three studies recruited general smokers. One trial each recruited smokers with substance use disorder, cardiovascular inpatients, pregnant women, and low-income Africa-American inpatient smokers. Motivation to quit not required in all trials.

b. Intervention: One trial provided both stage-based counselling and advice; other trials offered either counselling or advice tailored to stage of change. Intervention usually includes follow-up phone call(s) and typically delivered by a physician, healthcare worker, or trained counsellor. Behavioural co-intervention provided in half of the studies; of these, three studies also recommended pharmacotherapy (NRT). One study encouraged pharmacotherapy (NRT or bupropion) without a behavioural co-intervention and another provided a lung function test and CO test feedback with individualized newsletter of the data. 13% of intervention participants in this analysis received specialized behavioural counselling (motivational interviewing).

c. Control: Usual care varies across trial. In some trials, active smoking cessation interventions provided as part of or in conjunction with usual care. For example, one study provided controls with general smoking cessation advice and all study participants were encouraged to use NRT or bupropion. In another study, all participants (including controls), completed group smoking cessation programme, received telephone calls from a counsellor, and possibly recommended pharmacotherapy. In a third study, usual care consisted of brief motivational interviewing and a self-help manual. 13% of controls in this analysis received specialized behavioural counselling (motivational interviewing).

d. Five of the studies were cluster randomized trials.

e. All evidence at risk for selection bias (minority of which is at high risk) with some evidence also at unclear risk for other domains (performance/detection, attrition). Minority of evidence at high risk for lack of biochemical validation. We downrate this domain by -1.0.

f. Variation in point estimates but confidence intervals largely overlap (I2=36%, p=0.16). We downrate this domain by -0.5.

g. 19% of the evidence is indirect (in-patient and college setting). We do not downrate this domain.

h. Confidence interval encompasses one range of effect (little to no difference to small but important benefit). The optimal information size was not quite met (399 events), but we did not think the concerns were serious and adequate sample size. We did not downrate this domain.

i. Funnel plot including all studies in the review not suggestive of publication bias. Search not completely comprehensive but mix of findings across studies. We do not downrate this domain.

| Summary of findings: | | | | | | |
| --- | --- | --- | --- | --- | --- | --- |
| **Stage-based individual counselling and/or advice compared to usual care in general/mixed population of smokers** | | | | | | |
| **Patient or population**: General/mixed population of smokers  **Setting**: No restriction  **Intervention**: Stage-based individual counselling and/or advice  **Comparison**: Usual care | | | | | | |
| Outcomes | **Anticipated absolute effects^*^** (95% CI) | | Relative effect (95% CI) | № of participants  (studies) | Certainty of the evidence (GRADE) | Comments |
|  | **Risk with usual care** | **Risk with Stage-based individual counselling and/or advice** |  |  |  |  |
| Abstinence/Cessation ^a,b,c^  Outcome measurement: point prevalence 57%, continuous/sustained abstinence 43% studies  Biochemical validation 43%, partial biochemical validation 14% studies  Follow up: 6+ months | 110 per 1,000 | **130 per 1,000** (109 to 156) | **RR 1.19** (0.99 to 1.42) | 3293 (7 RCTs) ^d^ | ⨁⨁⨁◯  MODERATE ^e,f,g,h,i^ | Adjustment for clustering: RR 1.24 (0.99, 1.56); I2=16%, p=0.31;  Abstinence defined as point prevalence at 6 or 12 months: RR 1.19 (1.01, 1.41), 7 trials, n=3293; I2=37%, p=0.15%;  Per protocol analysis: RR 1.19 (1.00, 1.42), 7 trials, n=2314; I2=51%, p=0.06;  Fixed effects analysis.  AMSTAR-2: Critically low.  Date of last search: August 2010.  Not GRADEd by review authors. |
| ***The risk in the intervention group** (and its 95% confidence interval) is based on the assumed risk in the comparison group and the **relative effect** of the intervention (and its 95% CI).   **CI:** Confidence interval; **RR:** Risk ratio | | | | | | |
| **GRADE Working Group grades of evidence** **High certainty:** We are very confident that the true effect lies close to that of the estimate of the effect **Moderate certainty:** We are moderately confident in the effect estimate: The true effect is likely to be close to the estimate of the effect, but there is a possibility that it is substantially different **Low certainty:** Our confidence in the effect estimate is limited: The true effect may be substantially different from the estimate of the effect **Very low certainty:** We have very little confidence in the effect estimate: The true effect is likely to be substantially different from the estimate of effect | | | | | | |

#### Explanations

a. Three studies recruited general smokers. One trial each recruited smokers with substance use disorder, cardiovascular inpatients, pregnant women, and low-income Africa-American inpatient smokers. Motivation to quit not required in all trials.

b. Intervention: One trial provided both stage-based counselling and advice; other trials offered either counselling or advice tailored to stage of change. Intervention usually includes follow-up phone call(s) and typically delivered by a physician, healthcare worker, or trained counsellor. Behavioural co-intervention provided in half of the studies; of these, three studies also recommended pharmacotherapy (NRT). One study encouraged pharmacotherapy (NRT or bupropion) without a behavioural co-intervention and another provided a lung function test and CO test feedback with individualized newsletter of the data. 13% of intervention participants in this analysis received specialized behavioural counselling (motivational interviewing).

c. Control: Usual care varies across trial. In some trials, active smoking cessation interventions provided as part of or in conjunction with usual care. For example, one study provided controls with general smoking cessation advice and all study participants were encouraged to use NRT or bupropion. In another study, all participants (including controls) completed group smoking cessation programme, received telephone calls from a counsellor, and possibly recommended pharmacotherapy. In a third study, usual care consisted of brief motivational interviewing and a self-help manual. 13% of controls in this analysis received specialized behavioural counselling (motivational interviewing).

d. Five of the studies were cluster randomized trials.

e. All evidence at risk for selection bias (minority of which is at high risk) with some evidence also at unclear risk for other domains (performance/detection, attrition). Minority of evidence at high risk for lack of biochemical validation. We downrate this domain by -1.0.

f. Variation in point estimates but confidence intervals largely overlap (I2=36%, p=0.16). We downrate this domain by -0.5.

g. 19% of the evidence is indirect (in-patient and college setting). We do not downrate this domain.

h. Confidence interval encompasses one range of effect (little to no difference to small but important benefit). The optimal information size was not quite met (399 events), but we did not think the concerns were serious and adequate sample size. We did not downrate this domain.

i. Funnel plot including all studies in the review not suggestive of publication bias. Search not completely comprehensive but mix of findings across studies. We do not downrate this domain.

## Appendix K Table 6. Stage-based individual counselling or advice versus Assessment only: Smoking cessation in general/mixed population of smokers

| Stage-based individual counselling or advice compared to assessment only in general/mixed population of smokers  **Bibliography:** Cahill 2010; Date of last search: August 2010 | | | | | | | | | | | |
| --- | --- | --- | --- | --- | --- | --- | --- | --- | --- | --- | --- |
| **Certainty assessment** | | | | | | | **Summary of findings** | | | | |
| **№ of participants (studies) Follow-up** | **Risk of bias** | **Inconsistency** | **Indirectness** | **Imprecision** | **Publication bias** | **Overall certainty of evidence** | **Study event rates (%)** | | **Relative effect (95% CI)** | **Anticipated absolute effects** | |
|  |  |  |  |  |  |  | **With assessment only** | **With Stage-based individual counselling or advice** |  | **Risk with assessment only** | **Risk difference with Stage-based individual counselling or advice** |
| **Abstinence/Cessation (follow up: 6+ months)** ^a,b,c^  Outcome measurement: point prevalence 67%, prolonged abstinence 33% studies  Biochemical validation 33% studies | | | | | | | | | | | |
| 3056 (3 RCTs) ^d^ | serious ^e^ | very serious ^f^ | not serious ^g^ | not serious ^h^ | none ^i^ | ⨁◯◯◯  VERY LOW | 70/1627 (4.3%) | 85/1429 (5.9%) | **RR 1.28** (0.95 to 1.73) | 43 per 1,000 | **12 more per 1,000** (from 2 fewer to 31 more) |

**CI:** Confidence interval; **RR:** Risk ratio

#### Explanations

a. One trial each recruited females only, smokers with hypertension or hypercholesterolemia, and general smokers. Motivation to quit not required in all trials.

b. Intervention: Two trials provided stage-based brief advice and one provided stage-based counselling. Intervention usually includes follow-up phone call(s) and typically delivered by a physician, healthcare worker, or trained counsellor. All studies provided a behavioural co-intervention and one study also allowed physicians to prescribe NRT (<1% of participants took NRT). In one trial, an optional motivational interviewing phone call was offered but unclear how many participants received it.

c. Control: Review authors consider 'assessment only' as a no intervention control. Across trials, control conditions include measurement only (n=2) and interventions for hypertension or hypercholesterolaemia (n=1). No co-interventions provided.

d. One study is a cluster randomized trial

e. All evidence at risk of selection bias (minority at high risk) and 94% at high risk for lack of biochemical validation. We downrate this domain by -1.0.

f. Effect estimates vary and limited overlap of confidence intervals (I2=80%, p=0.01). We downrate this domain by -2.0.

g. No indirectness.

h. Confidence interval encompasses one range of effect (little to no difference to small but important benefit). The optimal information size not met (total of 155 events), but adequate sample size. We did not downrate this domain.

i. Funnel plot including all studies in the review not suggestive of publication bias. Search not completely comprehensive but mix of findings across studies. We do not downrate this domain.

| Summary of findings: | | | | | | |
| --- | --- | --- | --- | --- | --- | --- |
| **Stage-based individual counselling or advice compared to assessment only in general/mixed population of smokers** | | | | | | |
| **Patient or population**: General/mixed population of smokers  **Setting**: No restriction  **Intervention**: Stage-based individual counselling or advice  **Comparison**: Assessment only | | | | | | |
| Outcomes | **Anticipated absolute effects^*^** (95% CI) | | Relative effect (95% CI) | № of participants  (studies) | Certainty of the evidence (GRADE) | Comments |
|  | **Risk with assessment only** | **Risk with Stage-based individual counselling or advice** |  |  |  |  |
| Abstinence/Cessation ^a,b,c^  Outcome measurement: point prevalence 67%, prolonged abstinence 33% studies  Biochemical validation 33% studies  Follow up: 6+ months | 43 per 1,000 | **55 per 1,000** (41 to 74) | **RR 1.28** (0.95 to 1.73) | 3056 (3 RCTs) ^d^ | ⨁◯◯◯  VERY LOW ^e,f,g,h,i^ | Adjustment for clustering: RR 1.43 (0.94, 2.17); I2=76%, p=0.02;  Abstinence defined as point prevalence at 6 or 12 months: RR 1.70 (1.34, 2.14), 3 trials, n=3056; I2=91%, p<0.00001;  Per protocol analysis: 3 trials; RR 1.44 (1.10, 1.89), 3 trials, n=2267; I2=85%, p=0.001;  Fixed effects analysis.  AMSTAR-2: Critically low.  Date of last search: August 2010.  Not GRADEd by review authors. |
| ***The risk in the intervention group** (and its 95% confidence interval) is based on the assumed risk in the comparison group and the **relative effect** of the intervention (and its 95% CI).   **CI:** Confidence interval; **RR:** Risk ratio | | | | | | |
| **GRADE Working Group grades of evidence** **High certainty:** We are very confident that the true effect lies close to that of the estimate of the effect **Moderate certainty:** We are moderately confident in the effect estimate: The true effect is likely to be close to the estimate of the effect, but there is a possibility that it is substantially different **Low certainty:** Our confidence in the effect estimate is limited: The true effect may be substantially different from the estimate of the effect **Very low certainty:** We have very little confidence in the effect estimate: The true effect is likely to be substantially different from the estimate of effect | | | | | | |

#### Explanations

a. One trial each recruited females only, smokers with hypertension or hypercholesterolemia, and general smokers. Motivation to quit not required in all trials.

b. Intervention: Two trials provided stage-based brief advice and one provided stage-based counselling. Intervention usually includes follow-up phone call(s) and typically delivered by a physician, healthcare worker, or trained counsellor. All studies provided a behavioural co-intervention and one study also allowed physicians to prescribe NRT (<1% of participants took NRT). In one trial, an optional motivational interviewing phone call was offered but unclear how many participants received it.

c. Control: Review authors consider 'assessment only' as a no intervention control. Across trials, control conditions include measurement only (n=2) and interventions for hypertension or hypercholesterolaemia (n=1). No co-interventions provided.

d. One study is a cluster randomized trial

e. All evidence at risk of selection bias (minority at high risk) and 94% at high risk for lack of biochemical validation. We downrate this domain by -1.0.

f. Effect estimates vary and limited overlap of confidence intervals (I2=80%, p=0.01). We downrate this domain by -2.0.

g. No indirectness.

h. Confidence interval encompasses one range of effect (little to no difference to small but important benefit). The optimal information size not met (total of 155 events), but adequate sample size. We did not downrate this domain.

i. Funnel plot including all studies in the review not suggestive of publication bias. Search not completely comprehensive but mix of findings across studies. We do not downrate this domain.

# Cahill 2016 {1960}

## Appendix K Table 7. Cytisine versus Placebo: Smoking cessation in smokers motivated/wishing to quit

| Cytisine compared to placebo in smokers motivated/wishing to quit  **Bibliography:** Cahill 2016; Last date of search: May 2015 | | | | | | | | | | | |
| --- | --- | --- | --- | --- | --- | --- | --- | --- | --- | --- | --- |
| **Certainty assessment** | | | | | | | **Summary of findings** | | | | |
| **№ of participants (studies) Follow-up** | **Risk of bias** | **Inconsistency** | **Indirectness** | **Imprecision** | **Publication bias** | **Overall certainty of evidence** | **Study event rates (%)** | | **Relative effect (95% CI)** | **Anticipated absolute effects** | |
|  |  |  |  |  |  |  | **With placebo** | **With Cytisine** |  | **Risk with placebo** | **Risk difference with Cytisine** |
| **Abstinence/cessation (follow up: 6+ months)** ^a,b,c^  Outcome measurement: continuous/sustained abstinence 100% studies  Biochemical validation: 100% studies | | | | | | | | | | | |
| 937 (2 RCTs) | not serious ^d^ | not serious ^e^ | not serious ^f^ | serious ^g^ | none ^h^ | ⨁⨁⨁◯ MODERATE | 10/467 (2.1%) | 40/470 (8.5%) | **RR 3.98** (2.01 to 7.87) | 21 per 1,000 | **64 more per 1,000** (from 22 more to 147 more) |

**CI:** Confidence interval; **RR:** Risk ratio

#### Explanations

a. 1.5 mg tablets (variable per day doses) for 25-day period with behavioural support kept to a minimum.

b. Review authors broadly included studies of adult smokers but an examination of study-level information in the review showed that all participants in the analysis were smokers motivated to quit, while one study (21% of participants in analysis) was in men.

c. Co-interventions (both groups): Authors state that behavioural support kept to a minimum, but counselling/support provided in both studies.

d. No substantial risk of bias in any of the ROB domains. We do not downrate this domain.

e. Little variation in point estimates and confidence intervals overlap. I2=0%, p=0.40. We do not downrate this domain.

f. One study contributing 10% of the weight in the meta-analysis was indirect for setting and country. We do not downrate this domain.

g. Confidence interval encompasses two ranges of effect (small but important benefit to large benefit). The optimal information size not met (total of 50 events) and inadequate sample size (<2000 participants). We downrate this domain by -1.5.

h. Although few studies located, search was comprehensive. Unlikely that unidentified results exist. We do not downrate this domain.

| Summary of findings: | | | | | | |
| --- | --- | --- | --- | --- | --- | --- |
| **Cytisine compared to placebo in smokers motivated/wishing to quit** | | | | | | |
| **Patient or population**: Smokers motivated/wishing to quit  **Setting**: Not restricted  **Intervention**: Cytisine  **Comparison**: Placebo | | | | | | |
| Outcomes | **Anticipated absolute effects^*^** (95% CI) | | Relative effect (95% CI) | № of participants  (studies) | Certainty of the evidence (GRADE) | Comments |
|  | **Risk with placebo** | **Risk with Cytisine** |  |  |  |  |
| Abstinence/cessation ^a,b,c^  Outcome measurement: continuous/sustained abstinence 100% studies  Biochemical validation: 100% studies  Follow up: 6+ months | 21 per 1,000 | **85 per 1,000** (43 to 169) | **RR 3.98** (2.01 to 7.87) | 937 (2 RCTs) | ⨁⨁⨁◯ MODERATE ^d,e,f,g,h^ | Sensitivity analysis including a third study (Scharfenberg 1971) increased heterogeneity to I2=68%.  Fixed effects meta-analysis.  AMSTAR-2: Low.  Date of last search: May 2015.  Authors rated certainty as low. ^i,j^ |
| ***The risk in the intervention group** (and its 95% confidence interval) is based on the assumed risk in the comparison group and the **relative effect** of the intervention (and its 95% CI).   **CI:** Confidence interval; **RR:** Risk ratio | | | | | | |
| **GRADE Working Group grades of evidence** **High certainty:** We are very confident that the true effect lies close to that of the estimate of the effect **Moderate certainty:** We are moderately confident in the effect estimate: The true effect is likely to be close to the estimate of the effect, but there is a possibility that it is substantially different **Low certainty:** Our confidence in the effect estimate is limited: The true effect may be substantially different from the estimate of the effect **Very low certainty:** We have very little confidence in the effect estimate: The true effect is likely to be substantially different from the estimate of effect | | | | | | |

#### Explanations

a. 1.5 mg tablets (variable per day doses) for 25-day period with behavioural support kept to a minimum.

b. Review authors broadly included studies of adult smokers but an examination of study-level information in the review showed that all participants in the analysis were smokers motivated to quit, while one study (21% of participants in analysis) was in men.

c. Co-interventions (both groups): Authors state that behavioural support kept to a minimum, but counselling/support provided in both studies.

d. No substantial risk of bias in any of the ROB domains. We do not downrate this domain.

e. Little variation in point estimates and confidence intervals overlap. I2=0%, p=0.40. We do not downrate this domain.

f. One study contributing 10% of the weight in the meta-analysis was indirect for setting and country. We do not downrate this domain.

g. Confidence interval encompasses two ranges of effect (small but important benefit to large benefit). The optimal information size not met (total of 50 events) and inadequate sample size (<2000 participants). We downrate this domain by -1.5.

h. Although few studies located, search was comprehensive. Unlikely that unidentified results exist. We do not downrate this domain.

i. Authors rated certainty as low (imprecision downrated twice for number of events and few studies).

j. Refer to companion analysis: cytisine vs placebo, point prevalence at 2 years in general/mixed smokers.

## Appendix K Table 8. Cytisine versus Placebo: Smoking cessation and adverse events in general/mixed population of smokers

| Cytisine compared to placebo in a general/mixed population of smokers **Bibliography:** Cahill 2016; Last date of search: May 2015 | | | | | | | | | | | |
| --- | --- | --- | --- | --- | --- | --- | --- | --- | --- | --- | --- |
| **Certainty assessment** | | | | | | | **Summary of findings** | | | | |
| **№ of participants (studies) Follow-up** | **Risk of bias** | **Inconsistency** | **Indirectness** | **Imprecision** | **Publication bias** | **Overall certainty of evidence** | **Study event rates (%)** | | **Relative effect (95% CI)** | **Anticipated absolute effects** | |
|  |  |  |  |  |  |  | **With placebo** | **With Cytisine** |  | **Risk with placebo** | **Risk difference with Cytisine** |
| **Abstinence/cessation (follow up: 2 years)** ^a,b,c^  Outcome measurement: point prevalence 100% studies  Biochemical validation: 0% studies | | | | | | | | | | | |
| 1214 (1 RCT) | very serious ^d^ | not serious ^e^ | not serious ^f^ | serious ^g^ | none ^h^ | ⨁◯◯◯  VERY LOW | 79/607 (13.0%) | 127/607 (20.9%) | **RR 1.61** (1.24 to 2.08) | 130 per 1,000 | **79 more per 1,000** (from 31 more to 141 more) |
| **Adverse events (follow up: Not reported)** ^i,j,k^  Outcome measurement: Not reported | | | | | | | | | | | |
| NR (3 RCTs) | not serious ^l^ | not serious ^m^ | not serious ^n^ | not serious to very serious ^o^ | none ^p^ | unable to assess | Adverse events largely similar between groups. Similar rates of mild adverse events (nausea, restlessness, insomnia, irritability) in abstinent smokers at 4 weeks in one study between groups (23.4% vs 20%); longer term information not reported. A total of 10 events (e.g., dyspepsia, nausea, and headache) from four people in each group from a second study. The third study reported higher rates of gastrointestinal disorders with cytisine (13.8% vs 8.1%, p=0.02). | | | | |

**CI:** Confidence interval; **RR:** Risk ratio; **NR**: Not reported

#### Explanations

a. 1.5 mg tablets (variable per day doses) for 25-day period with behavioural support kept to a minimum.

b. Study-level description did not report co-interventions.

c. Review authors broadly included studies of adult smokers. This study was a general/mixed population of smokers.

d. High risk for detection bias (self-reported smoking) and unclear risk for remaining domains. We downrate this domain by -2.0.

e. One study only. We do not downrate this domain.

f. An evaluation of the study-level information provided in the review signaled no issues of indirectness to the question of interest. We do not downrate this domain.

g. Confidence interval encompasses two ranges of effect (small but important benefit to large benefit). The optimal information size not met (total of 206 events) and inadequate sample size (<2000 participants). We downrate this domain by -1.5.

h. Although few studies located in this review for cytisine, search was comprehensive. Unlikely that unidentified results exist. We do not downrate this domain.

i. 1.5 mg tables (varied per-day doses across studies) for 20- or 25-day period, with or without minimal behavioural support.

j. Co-interventions (both groups): Behavioural provided in most studies (total n=3 trials), inclusive of counselling and support.

k. Review authors broadly included studies of adult smokers but an examination of study-level information in the review showed two studies in smokers motivated to quit (one in males only) and one in an unspecified (general/mixed) population.

l. Two studies at unclear risk of bias for incomplete outcome reporting. We downrate by -0.5, but this is not substantial enough to assign a ‘serious’ rating.

m. According to authors’ synthesis, results are largely congruent. We do not downrate this domain.

n. One of three studies indirect for setting and country. Authors did not report how studies were weighted in narrative analysis. We do not downrate for this domain.

o. Sample size not reported and unable to assess optimal information size and confidence intervals. We cannot rate this domain.

p. Although few studies located in this review for cytisine, search was comprehensive. Unlikely that unidentified results exist. We do not downrate this domain.

| Summary of findings: | | | | | | |
| --- | --- | --- | --- | --- | --- | --- |
| **Cytisine compared to placebo in smokers motivated to quit** | | | | | | |
| **Patient or population**: Smokers motivated to quit  **Setting**: Not restricted  **Intervention**: Cytisine  **Comparison**: Placebo | | | | | | |
| Outcomes | **Anticipated absolute effects^*^** (95% CI) | | Relative effect (95% CI) | № of participants  (studies) | Certainty of the evidence (GRADE) | Comments |
|  | **Risk with placebo** | **Risk with Cytisine** |  |  |  |  |
| Abstinence/cessation ^a,b,c^  Outcome measurement: point prevalence 100% studies  Biochemical validation: 0% studies  Follow up: 2 years | 130 per 1,000 | **210 per 1,000** (161 to 271) | **RR 1.61** (1.24 to 2.08) | 1214 (1 RCT) | ⨁◯◯◯  VERY LOW ^d,e,f,g,h^ | At 6 months, RR 1.91 (95% CI, 1.53 to 2.37); by-group data not provided for the six month analysis.  Refer to companion analysis comparing cytisine vs placebo in smokers motivated to quit, continuous abstinence at six months or longer; a combined analysis (sensitivity) showed a high amount of statistical heterogeneity.  Date of last search: May 2015.  AMSTAR-2: Low.  Review authors did not GRADE this analysis. |
| Adverse events ^i,j,k^  Outcome measurement: Not reported  Follow up: Not reported | Adverse events largely similar between groups. Similar rates of mild adverse events (nausea, restlessness, insomnia, irritability) in abstinent smokers at 4 weeks in one study between groups (23.4% vs 20%); longer term information not reported. A total of 10 events (e.g., dyspepsia, nausea, and headache) from four people in each group from a second study. The third study reported higher rates of gastrointestinal disorders with cytisine (13.8% vs 8.1%, p=0.02). | |  | (3 RCTs) | unable to assess ^l,m,n,o,p^ | Incomplete quantitative information reported.  Date of last search: May 2015.  AMSTAR-2: Low.  Review authors did not GRADE this analysis. |
| ***The risk in the intervention group** (and its 95% confidence interval) is based on the assumed risk in the comparison group and the **relative effect** of the intervention (and its 95% CI).   **CI:** Confidence interval; **RR:** Risk ratio | | | | | | |
| **GRADE Working Group grades of evidence** **High certainty:** We are very confident that the true effect lies close to that of the estimate of the effect **Moderate certainty:** We are moderately confident in the effect estimate: The true effect is likely to be close to the estimate of the effect, but there is a possibility that it is substantially different **Low certainty:** Our confidence in the effect estimate is limited: The true effect may be substantially different from the estimate of the effect **Very low certainty:** We have very little confidence in the effect estimate: The true effect is likely to be substantially different from the estimate of effect | | | | | | |

#### Explanations

a. 1.5 mg tablets (variable per day doses) for 25-day period with behavioural support kept to a minimum.

b. Study-level description did not report co-interventions.

c. Review authors broadly included studies of adult smokers. This study was a general/mixed population of smokers.

d. High risk for detection bias (self-reported smoking) and unclear risk for remaining domains. We downrate this domain by -2.0.

e. One study only. We do not downrate this domain.

f. An evaluation of the study-level information provided in the review signalled no issues of indirectness to the question of interest. We do not downrate this domain.

g. Confidence interval encompasses two ranges of effect (small but important benefit to large benefit). The optimal information size not met (total of 206 events) and inadequate sample size (<2000 participants). We downrate this domain by -1.5.

h. Although few studies located in this review for cytisine, search was comprehensive. Unlikely that unidentified results exist. We do not downrate this domain.

i. 1.5 mg tables (varied per-day doses across studies) for 20- or 25-day period, with or without minimal behavioural support.

j. Co-interventions (both groups): Behavioural provided in most studies (total n=3 trials), inclusive of counselling and support.

k. Review authors broadly included studies of adult smokers but an examination of study-level information in the review showed studies in smokers motivated to quit (one in males only) and one in an unspecified (general/mixed) population.

l. Two studies at unclear risk of bias for incomplete outcome reporting. We downrate by -0.5, but this is not substantial enough to assign a ‘serious’ rating.

m. According to authors’ synthesis, results are largely congruent. We do not downrate this domain.

n. One of three studies indirect for setting and country. Authors did not report how studies were weighted in narrative analysis. We do not downrate for this domain.

o. Sample size not reported and unable to assess optimal information size and confidence intervals. We cannot rate this domain.

p. Although few studies located in this review for cytisine, search was comprehensive. Unlikely that unidentified results exist. We do not downrate this domain.

## Appendix K Table 9. Varenicline versus Placebo: Smoking cessation in general/mixed population of smokers

| Varenicline compared to placebo in general/mixed population of smokers  **Bibliography:** Cahill 2016; Date last searched: May 2015 | | | | | | | | | | | |
| --- | --- | --- | --- | --- | --- | --- | --- | --- | --- | --- | --- |
| **Certainty assessment** | | | | | | | **Summary of findings** | | | | |
| **№ of participants (studies) Follow-up** | **Risk of bias** | **Inconsistency** | **Indirectness** | **Imprecision** | **Publication bias** | **Overall certainty of evidence** | **Study event rates (%)** | | **Relative effect (95% CI)** | **Anticipated absolute effects** | |
|  |  |  |  |  |  |  | **With placebo** | **With Varenicline** |  | **Risk with placebo** | **Risk difference with Varenicline** |
| **Abstinence/cessation (follow up: 6+ months)** ^a,b,c,d,e^  Outcome measurement: continuous/sustained abstinence 74%, point prevalence 22%, unclear 4% studies  Biochemical validation: 100% studies | | | | | | | | | | | |
| 12625 (27 RCTs) ^e^ | not serious ^f^ | serious ^g^ | not serious ^h^ | not serious ^i^ | none ^j^ | ⨁⨁⨁◯  MODERATE | 668/5993 (11.1%) | 1695/6632 (25.6%) | **RR 2.24** (2.06 to 2.43) | 111 per 1,000 | **138 more per 1,000** (from 118 more to 159 more) |
| **Abstinence/cessation (follow up: 6 months)** ^a,c,d,e,k^  Outcome measurement: continuous/sustained abstinence 76%, prolonged abstinence 4%, point prevalence 20% studies  Biochemical validation: 96% studies | | | | | | | | | | | |
| 12304 (25 RCTs) ^e^ | not serious ^l^ | serious ^m^ | not serious ^n^ | not serious ^o^ | none ^p^ | ⨁⨁⨁◯  MODERATE | 726/5832 (12.4%) | 1844/6472 (28.5%) | **RR 2.25** (2.08 to 2.44) | 124 per 1,000 | **156 more per 1,000** (from 134 more to 179 more) |

**CI:** Confidence interval; **RR:** Risk ratio

#### Explanations

a. Varenicline 1 mg twice daily for 12 weeks except two studies (8 week).

b. Review authors broadly included studies of adult smokers but an examination of study-level information in the review showed a mixed patient population composition for this analysis: 52% of trials motivated to quit (including one-to-two trials each in various specified subgroups such as substance use, COPD, CVD, and mental health), 33% of unspecified (general/mixed) populations, and a small number of other studies (mental health, cancer, asthma, surgical patients).

c. Co-interventions (both groups): Behavioural provided in most studies, inclusive of counselling, (telephone) support, self-help, advice. For the longer analysis of 6+ months follow-up, 2.7% of participants across studies allocated to varenicline and 3.4% in the placebo group received specialized counselling (CBT, relapse prevention, MI). For the analysis at 6 months, 1.3% and 1.8% in the varenicline and placebo groups, respectively, received specialized counselling (MI). For both analyses, few studies were unclear/not reported for co-interventions.

d. One of the studies used biochemical validation in a subset of patients and therefore not counted as biochemically validated.

e. Nine studies with 24-week follow-up.

f. Some risk of bias issues across evidence base but not substantive enough to warrant downrating.

g. Some variation in magnitude of point estimates but largely not in direction of effect. I2=60%, small p value (0.00004) may be due to sensitivity with a large dataset. We downrate by -1.0.

h. 19% of evidence was indirect (inpatient and specialized medical settings). We do not downrate. Not substantive enough for downrating was 1% studies indirect for country. An additional 11% studies with subset of countries that are indirect; these studies also do not contribute to indirectness estimation.

i. Confidence interval encompasses large benefit. The optimal information size is met (total of 2363 events) and large sample size. We did not downrate this domain.

j. Authors state that a funnel plot assessment appears to show a lack of smaller studies with negative findings but attributes this to effectiveness estimates being reflective of a true finding than of suppression or selective management of data. We do not downrate this domain.

k. Review authors broadly included studies of adult smokers but an examination of study-level information in the review showed a mixed patient population composition for this analysis: 48% of trials motivated to quit (including one-to-two trials each in various specified subgroups such as substance use, COPD, CVD, and mental health), 32% of unspecified (general/mixed) populations, and various other single studies (mental health, cancer, asthma, surgical patients, substance use).

l. Some risk of bias issues across evidence base but not substantive enough to warrant downrating. We do not downrate this domain.

m. Variability in magnitude of point estimates evidence. I2=66%, and very small p value (<0.00001) may be due to sensitivity with a large dataset. We downrate by -1.5.

n. 21% of evidence was indirect (inpatient and specialized medical settings). We downrate by -0.5. An additional 49% of evidence with subset of countries that are indirect (proportion unknown); these studies do not contribute to indirectness estimation.

o. Confidence interval encompasses large benefit. The optimal information size is met (total of 2570 events) and large sample size. We did not downrate this domain.

p. Given assessment for small study effects in the main analysis, a lack of smaller studies with negative findings not likely due to suppression or selective management of data. We do not downrate this domain.

| Summary of findings: | | | | | | |
| --- | --- | --- | --- | --- | --- | --- |
| **Varenicline compared to placebo in general/mixed population of smokers** | | | | | | |
| **Patient or population**: General/mixed population of smokers  **Setting**: Not restricted  **Intervention**: Varenicline  **Comparison**: Placebo | | | | | | |
| Outcomes | **Anticipated absolute effects^*^** (95% CI) | | Relative effect (95% CI) | № of participants  (studies) | Certainty of the evidence (GRADE) | Comments |
|  | **Risk with placebo** | **Risk with Varenicline** |  |  |  |  |
| Abstinence/cessation ^a,b,c,d,e^  Outcome measurement: continuous/sustained abstinence 74%, point prevalence 22%, unclear 4% studies  Biochemical validation:100% studies  Follow up: 6+ months | 111 per 1,000 | **250 per 1,000** (230 to 271) | **RR 2.24** (2.06 to 2.43) | 12625 (27 RCTs) | ⨁⨁⨁◯  MODERATE ^f,g,h,i,j^ | Fixed effects meta-analysis.  Authors state a sensitivity excluding one of the trials for providing counselling in lieu of placebo made no appreciable difference. Revised analysis restricting to 15 studies (n=5904) with 12-month follow-up (no forest plot) showed little difference in results (RR 2.29, 95% CI 2.02 to 2.60).  Companion analysis below.  Date of last search: May 2015.  AMSTAR-2: Low.  High GRADE certainty by review authors. ^k^ |
| Abstinence/cessation ^a,c,d,l^  Outcome measurement: continuous/sustained abstinence 76%, prolonged abstinence 4%, point prevalence 20% studies  Biochemical validation: 96% studies  Follow up: 6 months | 124 per 1,000 | **280 per 1,000** (259 to 304) | **RR 2.25** (2.08 to 2.44) | 12304 (25 RCTs) | ⨁⨁⨁◯  MODERATE ^m,n,o,p,q^ | Fixed effects meta-analysis.  Companion analysis above.  Date of last search: May 2015.  AMSTAR-2: Low.  Authors did not GRADE this analysis. |
| ***The risk in the intervention group** (and its 95% confidence interval) is based on the assumed risk in the comparison group and the **relative effect** of the intervention (and its 95% CI).   **CI:** Confidence interval; **RR:** Risk ratio | | | | | | |
| **GRADE Working Group grades of evidence** **High certainty:** We are very confident that the true effect lies close to that of the estimate of the effect **Moderate certainty:** We are moderately confident in the effect estimate: The true effect is likely to be close to the estimate of the effect, but there is a possibility that it is substantially different **Low certainty:** Our confidence in the effect estimate is limited: The true effect may be substantially different from the estimate of the effect **Very low certainty:** We have very little confidence in the effect estimate: The true effect is likely to be substantially different from the estimate of effect | | | | | | |

**Explanations**

a. Varenicline 1 mg twice daily for 12 weeks except two studies (8 week).

b. Review authors broadly included studies of adult smokers but an examination of study-level information in the review showed a mixed patient population composition for this analysis: 52% of trials motivated to quit (including one-to-two trials each in various specified subgroups such as substance use, COPD, CVD, and mental health), 33% of unspecified (general/mixed) populations, and a small number of other studies (mental health, cancer, asthma, surgical patients).

c. Co-interventions (both groups): Behavioural provided in most studies, inclusive of counselling, (telephone) support, self-help, advice. For the longer analysis of 6+ months follow-up, 2.7% of participants across studies allocated to varenicline and 3.4% in the placebo group received specialized counselling (CBT, relapse prevention, MI). For the analysis at 6 months, 1.3% and 1.8% in the varenicline and placebo groups, respectively, received specialized counselling (MI). For both analyses, few studies were unclear/not reported for co-interventions.

d. One of the studies used biochemical validation in a subset of patients and therefore not counted as biochemically validated.

e. Nine studies with 24-week follow-up.

f. Some risk of bias issues across evidence base but not substantive enough to warrant downrating.

g. Some variation in magnitude of point estimates but largely not in direction of effect. I2=60%, small p value (0.00004) may be due to sensitivity with a large dataset. We downrate by -1.0.

h. 19% of evidence was indirect (inpatient and specialized medical settings). We do not downrate. Not substantive enough for downrating was 1% studies indirect for country. An additional 11% studies with subset of countries that are indirect; these studies also do not contribute to indirectness estimation.

i. Confidence interval encompasses large benefit. The optimal information size is met (total of 2363 events) and large sample size. We did not downrate this domain.

j. Authors state that a funnel plot assessment appears to show a lack of smaller studies with negative findings but attributes this to effectiveness estimates being reflective of a true finding than of suppression or selective management of data. We do not downrate this domain.

k. Despite moderate heterogeneity, authors did not downrate as all but two studies showed results favouring varenicline. Authors do not feel as though any results were suppressed or selectively reported.

l. Review authors broadly included studies of adult smokers but an examination of study-level information in the review showed a mixed patient population composition for this analysis: 48% of trials motivated to quit (including one-to-two trials each in various specified subgroups such as substance use, COPD, CVD, and mental health), 32% of unspecified (general/mixed) populations, and various other single studies (mental health, cancer, asthma, surgical patients, substance use).

m. Some risk of bias issues across evidence base but not substantive enough to warrant downrating. We do not downrate this domain.

n. Variability in magnitude of point estimates evidence. I2=66%, and very small p value (<0.00001) may be due to sensitivity with a large dataset. We downrate by -1.5 but keep rating at serious.

o. 21% of evidence was indirect (inpatient and specialized medical settings). We downrate by -0.5. An additional 49% of evidence with subset of countries that are indirect (proportion unknown); these studies do not contribute to indirectness estimation.

p. Confidence interval encompasses large benefit. The optimal information size is met (total of 2363 events) and large sample size. We did not downrate this domain.

q. Given assessment for small study effects in the main analysis, a lack of smaller studies with negative findings not likely due to suppression or selective management of data. We do not downrate this domain.

## Appendix K Table 10. Long-term varenicline use versus Placebo: Smoking cessation in general/mixed population of smokers

| Varenicline (long-term use) compared to placebo in general/mixed population of smokers  **Bibliography:** Cahill 2016; Date last searched: May 2015 | | | | | | | | | | | |
| --- | --- | --- | --- | --- | --- | --- | --- | --- | --- | --- | --- |
| **Certainty assessment** | | | | | | | **Summary of findings** | | | | |
| **№ of participants (studies) Follow-up** | **Risk of bias** | **Inconsistency** | **Indirectness** | **Imprecision** | **Publication bias** | **Overall certainty of evidence** | **Study event rates (%)** | | **Relative effect (95% CI)** | **Anticipated absolute effects** | |
|  |  |  |  |  |  |  | **With placebo** | **With Varenicline (long-term use)** |  | **Risk with placebo** | **Risk difference with Varenicline (long-term use)** |
| **Abstinence/cessation (follow up: 6-12 months)** ^a,b,c,d,e^  Outcome measurement: point prevalence 50%, continuous/sustained abstinence 25%, mixed 25% studies  Biochemical validation: 100% studies | | | | | | | | | | | |
| 2170 (4 studies) ^e^ | not serious ^f^ | very serious ^g^ | serious ^h^ | not serious ^i^ | none ^j^ | ⨁◯◯◯  VERY LOW | 65/972 (6.7%) | 282/1198 (23.5%) | **RR 3.64** (2.81 to 4.72) | 67 per 1,000 | **177 more per 1,000** (from 121 more to 249 more) |

**CI:** Confidence interval; **RR:** Risk ratio

#### Explanations

a. Extended varenicline treatment at 1 mg twice daily for 6-12 months.

b. Review authors broadly included studies of adult smokers but an examination of study-level information in the review showed a mixed patient population composition for this analysis: two trials of unspecified (general/mixed) population and two motivated to quit (one study in people with substance use disorder).

c. Co-interventions (both groups): Behavioural provided in most studies, inclusive of counselling, self-help, advice, and support. One study unclear/not reported.

d. Mixed outcome assessment (cessation): continuous abstinence with the exception of smokers deemed as non-quitters, whereby a reduction in cigarettes per day one month before follow-up was used.

e. Presumed based on planned treatment course in authors’ study tables.

f. Some risk of bias issues across evidence base but not substantive enough to warrant downrating.

g. Variability in magnitude of point estimates evidence and confidence intervals do not all overlap. I2=78%, p=0.003. We downrate by -2.0.

h. 66% of evidence indirect for specialized medical setting. We downrate by -1.0.

i. Confidence interval encompasses large benefit. The optimal information size was not quite met (347 events), but adequate sample size (>2000). We did not downrate this domain.

j. Mix of results among studies, comprehensive search. We do not downrate this domain.

| Summary of findings: | | | | | | |
| --- | --- | --- | --- | --- | --- | --- |
| **Varenicline (long-term use) compared to placebo in general/mixed population of smokers** | | | | | | |
| **Patient or population**: General/mixed population of smokers  **Setting**: Not restricted  **Intervention**: Varenicline (long-term use)  **Comparison**: Placebo | | | | | | |
| Outcomes | **Anticipated absolute effects^*^** (95% CI) | | Relative effect (95% CI) | № of participants  (studies) | Certainty of the evidence (GRADE) | Comments |
|  | **Risk with placebo** | **Risk with Varenicline (long-term use)** |  |  |  |  |
| Abstinence/cessation ^a,b,c,d^  Outcome measurement: point prevalence 50%, continuous/sustained abstinence 25%, mixed 25% studies  Biochemical validation: 100% studies  Follow up: 6-12 months | 67 per 1,000 | **243 per 1,000** (188 to 316) | **RR 3.64** (2.81 to 4.72) | 2170 (4 studies) ^e^ | ⨁◯◯◯  VERY LOW ^f,g,h,i,j^ | Fixed effects meta-analysis.  Date of last search: May 2015.  AMSTAR-2: Low.  Authors did not GRADE this analysis. |
| ***The risk in the intervention group** (and its 95% confidence interval) is based on the assumed risk in the comparison group and the **relative effect** of the intervention (and its 95% CI).   **CI:** Confidence interval; **RR:** Risk ratio | | | | | | |
| **GRADE Working Group grades of evidence** **High certainty:** We are very confident that the true effect lies close to that of the estimate of the effect **Moderate certainty:** We are moderately confident in the effect estimate: The true effect is likely to be close to the estimate of the effect, but there is a possibility that it is substantially different **Low certainty:** Our confidence in the effect estimate is limited: The true effect may be substantially different from the estimate of the effect **Very low certainty:** We have very little confidence in the effect estimate: The true effect is likely to be substantially different from the estimate of effect | | | | | | |

**Explanations**

a. Extended varenicline treatment at 1 mg twice daily for 6-12 months.

b. Review authors broadly included studies of adult smokers but an examination of study-level information in the review showed a mixed patient population composition for this analysis: two trials of unspecified (general/mixed) population and two motivated to quit (one study in people with substance use disorder).

c. Co-interventions (both groups): Behavioural provided in most studies, inclusive of counselling, self-help, advice, and support. One study unclear/not reported.

d. Mixed (cessation): continuous abstinence with the exception of smokers deemed as non-quitters, whereby a reduction in cigarettes per day one month before follow-up was used.

e. Presumed based on planned treatment course in authors’ study tables.

f. Some risk of bias issues across evidence base but not substantive enough to warrant downrating.

g. Variability in magnitude of point estimates evidence and confidence intervals don’t all overlap. I2=78%, p=0.003. We downrate by -2.0.

h. 66% of evidence indirect for specialized medical setting. We downrate by -1.0.

i. Confidence interval encompasses large benefit. The optimal information size was not quite met (347 events), but adequate sample size (>2000). We did not downrate this domain.

j. Mix of results among studies, comprehensive search. We do not downrate this domain.

## Appendix K Table 11. Low-dose varenicline use versus Placebo: Smoking cessation in general/mixed population of smokers

| Varenicline (low dose) compared to placebo in general/mixed population of smokers  **Bibliography:** Cahill 2016; Date last searched: May 2015 | | | | | | | | | | | |
| --- | --- | --- | --- | --- | --- | --- | --- | --- | --- | --- | --- |
| **Certainty assessment** | | | | | | | **Summary of findings** | | | | |
| **№ of participants (studies) Follow-up** | **Risk of bias** | **Inconsistency** | **Indirectness** | **Imprecision** | **Publication bias** | **Overall certainty of evidence** | **Study event rates (%)** | | **Relative effect (95% CI)** | **Anticipated absolute effects** | |
|  |  |  |  |  |  |  | **With placebo** | **With Varenicline (low dose)** |  | **Risk with placebo** | **Risk difference with Varenicline (low dose)** |
| **Abstinence/cessation (follow up: 12 months)** ^a,b,c,d^  Outcome measurement: continuous/sustained abstinence 100% studies  Biochemical validation: 100% studies | | | | | | | | | | | |
| 1266 (4 RCTs) | serious ^e^ | very serious ^f^ | not serious ^g^ | serious ^h^ | none ^i^ | ⨁◯◯◯  VERY LOW | 58/566 (10.2%) | 141/700 (20.1%) | **RR 2.08** (1.56 to 2.78) | 102 per 1,000 | **111 more per 1,000** (from 57 more to 182 more) |

**CI:** Confidence interval; **RR:** Risk ratio

#### Explanations

a. Half dosage (1mg/d) in three studies; one at participants’ discretion (0.5 to 2.0 mg/d) but mean modal varenicline dose 1.35 mg/d and placebo 1.63 mg/d.

b. All trials of a general/mixed population.

c. Co-interventions (both groups): Behavioural for all studies, inclusive of counselling and self-help.

d. Note, one study is unclear due to follow-up of 4-wk continuous abstinence as noted by authors.

e. 70% of evidence with substantive bias issues: 60% at high risk for selective reporting and unclear for attrition, 10% at unclear risk for blinding and attrition. We downrate by -1.5.

f. Variability in magnitude of point estimates and some overlap in confidence intervals. I2=68% and p=0.02. We downrate by -1.5.

g. No indirectness detected with perusal of review's study-level information.

h. Confidence interval encompasses one range of effect (moderate benefit to large benefit). The optimal information size not met (total of 199 events) and inadequate sample size (<2000 participants). We downrate this domain by -1.0.

i. Mix of results among studies, comprehensive search. We do not downrate this domain.

| Summary of findings: | | | | | | |
| --- | --- | --- | --- | --- | --- | --- |
| **Varenicline (low dose) compared to placebo in general/mixed population of smokers** | | | | | | |
| **Patient or population**: General/mixed population of smokers  **Setting**: Not restricted  **Intervention**: Varenicline (low dose)  **Comparison**: Placebo | | | | | | |
| Outcomes | **Anticipated absolute effects^*^** (95% CI) | | Relative effect (95% CI) | № of participants  (studies) | Certainty of the evidence (GRADE) | Comments |
|  | **Risk with placebo** | **Risk with Varenicline (low dose)** |  |  |  |  |
| Abstinence/cessation ^a,b,c,d^  Outcome measurement: continuous/sustained abstinence 100% studies  Biochemical validation: 100% studies  Follow up: 12 months | 102 per 1,000 | **213 per 1,000** (160 to 285) | **RR 2.08** (1.56 to 2.78) | 1266 (4 RCTs) | ⨁◯◯◯  VERY LOW ^e,f,g,h,i^ | Fixed effects meta-analysis.  Date of last search: May 2015.  AMSTAR-2: Low.  Authors did not GRADE this analysis. |
| ***The risk in the intervention group** (and its 95% confidence interval) is based on the assumed risk in the comparison group and the **relative effect** of the intervention (and its 95% CI).   **CI:** Confidence interval; **RR:** Risk ratio | | | | | | |
| **GRADE Working Group grades of evidence** **High certainty:** We are very confident that the true effect lies close to that of the estimate of the effect **Moderate certainty:** We are moderately confident in the effect estimate: The true effect is likely to be close to the estimate of the effect, but there is a possibility that it is substantially different **Low certainty:** Our confidence in the effect estimate is limited: The true effect may be substantially different from the estimate of the effect **Very low certainty:** We have very little confidence in the effect estimate: The true effect is likely to be substantially different from the estimate of effect | | | | | | |

**Explanations**

a. Half dosage (1mg/d) in three studies; one at participants’ discretion (0.5 to 2.0 mg/d) but mean modal varenicline dose 1.35 mg/d and placebo 1.63 mg/d.

b. All trials of a general/mixed population.

c. Co-interventions (both groups): Behavioural for all studies, inclusive of counselling and self-help.

d. One study is unclear due to 4-wk continuous abstinence as noted by authors.

e. 70% of evidence with substantive bias issues: 60% at high risk for selective reporting and unclear for attrition, 10% at unclear risk for blinding and attrition. We downrate by -1.5.

f. Variability in magnitude of point estimates and some overlap in confidence intervals. I2=68% and p=0.02. We downrate by -1.5 and reflect residual risk of bias downrating.

g. No indirectness detected with perusal of review's study-level information.

h. Confidence interval encompasses one range of effect (moderate benefit to large benefit). The optimal information size not met (total of 199 events) and inadequate sample size (<2000 participants). We downrate this domain by -1.0.

i. Mix of results among studies, comprehensive search. We do not downrate this domain.

## Appendix K Table 12. Variable dosing of varenicline versus Placebo: Smoking cessation in general/mixed population of smokers

| Varenicline (variable dosing) compared to placebo in general/mixed population of smokers  **Bibliography:** Cahill 2016; Date last searched: May 2015 | | | | | | | | | | | |
| --- | --- | --- | --- | --- | --- | --- | --- | --- | --- | --- | --- |
| **Certainty assessment** | | | | | | | **Summary of findings** | | | | |
| **№ of participants (studies) Follow-up** | **Risk of bias** | **Inconsistency** | **Indirectness** | **Imprecision** | **Publication bias** | **Overall certainty of evidence** | **Study event rates (%)** | | **Relative effect (95% CI)** | **Anticipated absolute effects** | |
|  |  |  |  |  |  |  | **With placebo** | **With Varenicline (variable dosing)** |  | **Risk with placebo** | **Risk difference with Varenicline (variable dosing)** |
| **Abstinence/cessation (follow up: 12 months)** ^a,b,c,d^  Outcome measurement: continuous/sustained abstinence 100% studies  Biochemical validation: 100% studies | | | | | | | | | | | |
| 1789 (6 RCTs) ^d^ | not serious ^e^ | serious ^f^ | not serious ^g^ | serious ^h^ | none ^i^ | ⨁⨁◯◯  LOW | 88/909 (9.7%) | 193/880 (21.9%) | **RR 2.29** (1.81 to 2.89) | 97 per 1,000 | **125 more per 1,000** (from 78 more to 183 more) |

**CI:** Confidence interval; **RR:** Risk ratio

#### Explanations

a. Option to reduce varenicline dosage to moderate side effects, at discretion.

b. Review authors broadly included studies of adult smokers but an examination of study-level information in the review showed a mixed patient population composition for this analysis: two trials of an unrestricted (general/mixed) population, three studies motivated to quit (two studies in people with mental health), and one study in cancer.

c. Co-interventions (both groups): Behavioural for all studies, inclusive of counselling, (telephone) support, and self-help. 9.8% participants in the varenicline group and 11.7% in the placebo group received specialized counselling (MI).

d. Although authors banner analysis as 12 months, information in study tables suggests about half of studies had 6-month follow-up.

e. Some risk of bias issues across evidence base but not substantive enough to warrant downrating.

f. Variability in magnitude of point estimates and some overlap in confidence intervals. I2=70% and p=0.01. We downrate by -1.5.

g. No substantive indirectness. 32% of evidence with mix of direct and indirect country (proportion unknown) and this evidence does not contribute to indirectness estimation. We do not downrate this domain.

h. Confidence interval encompasses one range of effect (moderate benefit to large benefit). The optimal information size not met (total of 281 events) and inadequate sample size (<2000 participants). We downrate this domain by -1.0.

i. Mix of results among studies, comprehensive search. We do not downrate this domain.

| Summary of findings: | | | | | | |
| --- | --- | --- | --- | --- | --- | --- |
| **Varenicline (variable dosing) compared to placebo in general/mixed population of smokers** | | | | | | |
| **Patient or population**: General/mixed population of smokers  **Setting**: Not restricted  **Intervention**: Varenicline (variable dosing)  **Comparison**: Placebo | | | | | | |
| Outcomes | **Anticipated absolute effects^*^** (95% CI) | | Relative effect (95% CI) | № of participants  (studies) | Certainty of the evidence (GRADE) | Comments |
|  | **Risk with placebo** | **Risk with Varenicline (variable dosing)** |  |  |  |  |
| Abstinence/cessation ^a,b,c^  Outcome measurement: continuous/sustained abstinence 100% studies  Biochemical validation: 100% studies  Follow up: 12 months | 97 per 1,000 | **222 per 1,000** (175 to 280) | **RR 2.29** (1.81 to 2.89) | 1789 (6 RCTs) ^d^ | ⨁⨁◯◯  LOW ^e,f,g,h,i^ | Fixed effects meta-analysis.  Date of last search: May 2015.  AMSTAR-2: Low.  Review authors did not GRADE this analysis. |
| ***The risk in the intervention group** (and its 95% confidence interval) is based on the assumed risk in the comparison group and the **relative effect** of the intervention (and its 95% CI).   **CI:** Confidence interval; **RR:** Risk ratio | | | | | | |
| **GRADE Working Group grades of evidence** **High certainty:** We are very confident that the true effect lies close to that of the estimate of the effect **Moderate certainty:** We are moderately confident in the effect estimate: The true effect is likely to be close to the estimate of the effect, but there is a possibility that it is substantially different **Low certainty:** Our confidence in the effect estimate is limited: The true effect may be substantially different from the estimate of the effect **Very low certainty:** We have very little confidence in the effect estimate: The true effect is likely to be substantially different from the estimate of effect | | | | | | |

**Explanations**

a. Option to reduce varenicline dosage to moderate side effects, at discretion

b. Review authors broadly included studies of adult smokers but an examination of study-level information in the review showed a mixed patient population composition for this analysis: two trials of an unrestricted (general/mixed) population, three studies motivated to quit (two studies in people with mental health), and one study in cancer.

c. Co-interventions (both groups): Behavioural for all studies, inclusive of counselling, (telephone) support, and self-help. 9.8% participants in the varenicline group and 11.7% in the placebo group received specialized counselling (MI).

d. Although authors banner analysis as 12 months, information in study tables lends to about half with 24-week follow-up.

e. Some risk of bias issues across evidence base but not substantive enough to warrant downrating.

f. Variability in magnitude of point estimates and some overlap in confidence intervals. I2=70% and p=0.01. We downrate by -1.5.

g. No substantive indirectness. 32% of evidence with mix of direct and indirect country (proportion unknown); this evidence does not contribute to indirectness estimation.

h. Confidence interval encompasses one range of effect (moderate benefit to large benefit). The optimal information size not met (total of 281 events) and inadequate sample size (<2000 participants). We downrate this domain by -1.0.

i. Mix of results among studies, comprehensive search. We do not downrate this domain.

## Appendix K Table 13. Varenicline versus Placebo: Smoking cessation in smokers reducing to quit

| Varenicline compared to placebo in smokers reducing to quit  **Bibliography:** Cahill 2016; Date last searched: May 2015 | | | | | | | | | | | |
| --- | --- | --- | --- | --- | --- | --- | --- | --- | --- | --- | --- |
| **Certainty assessment** | | | | | | | **Summary of findings** | | | | |
| **№ of participants (studies) Follow-up** | **Risk of bias** | **Inconsistency** | **Indirectness** | **Imprecision** | **Publication bias** | **Overall certainty of evidence** | **Study event rates (%)** | | **Relative effect (95% CI)** | **Anticipated absolute effects** | |
|  |  |  |  |  |  |  | **With placebo** | **With Varenicline** |  | **Risk with placebo** | **Risk difference with Varenicline** |
| **Abstinence/cessation (follow up: 12 months)** ^a,b,c^  Outcome measurement: continuous/sustained cessation 100% studies  Biochemical validation: 100% studies | | | | | | | | | | | |
| 1510 (1 RCT) | not serious ^d^ | not serious | not serious ^e^ | serious ^f^ | none ^g^ | ⨁⨁⨁◯  MODERATE | 45/750 (6.0%) | 182/760 (23.9%) | **RR 3.99** (2.93 to 5.44) | 60 per 1,000 | **179 more per 1,000** (from 116 more to 266 more) |

**CI:** Confidence interval; **RR:** Risk ratio

#### Explanations

a. Treatment course 6 months. Participant not willing to quit abruptly but were interested in quitting in the following three months.

b. Review authors broadly included studies of adult smokers but this study assessed participants who were interested in reducing smoking to quit.

c. Co-interventions (both groups): Behavioural (counselling and self-help).

d. No issues of bias were raised.

e. No substantive issues of indirectness. Unclear are how many participants from specialized medical setting and from indirect countries.

f. Confidence interval encompasses large benefit. The optimal information size not met (total of 227 events) and inadequate sample size (<2000 participants). We downrate this domain by -1.0.

g. Few studies despite comprehensive search. Unlikely due to suppression of results. We do not downrate this domain.

| Summary of findings: | | | | | | |
| --- | --- | --- | --- | --- | --- | --- |
| **Varenicline compared to placebo in smokers reducing to quit** | | | | | | |
| **Patient or population**: Smokers reducing to quit  **Setting**: Not restricted  **Intervention**: Varenicline  **Comparison**: Placebo | | | | | | |
| Outcomes | **Anticipated absolute effects^*^** (95% CI) | | Relative effect (95% CI) | № of participants  (studies) | Certainty of the evidence (GRADE) | Comments |
|  | **Risk with placebo** | **Risk with Varenicline** |  |  |  |  |
| Abstinence/cessation ^a,b,c^  Outcome measurement: continuous/sustained cessation 100% studies  Biochemical validation: 100% studies  Follow up: 12 months | 60 per 1,000 | **239 per 1,000** (176 to 326) | **RR 3.99** (2.93 to 5.44) | 1510 (1 RCT) | ⨁⨁⨁◯  MODERATE ^d,e,f,g^ | Date of last search: May 2015.  AMSTAR-2: Low.  Authors do not GRADE this analysis. |
| ***The risk in the intervention group** (and its 95% confidence interval) is based on the assumed risk in the comparison group and the **relative effect** of the intervention (and its 95% CI).   **CI:** Confidence interval; **RR:** Risk ratio | | | | | | |
| **GRADE Working Group grades of evidence** **High certainty:** We are very confident that the true effect lies close to that of the estimate of the effect **Moderate certainty:** We are moderately confident in the effect estimate: The true effect is likely to be close to the estimate of the effect, but there is a possibility that it is substantially different **Low certainty:** Our confidence in the effect estimate is limited: The true effect may be substantially different from the estimate of the effect **Very low certainty:** We have very little confidence in the effect estimate: The true effect is likely to be substantially different from the estimate of effect | | | | | | |

**Explanations**

a. Treatment course 6 months. Participant not willing to quit abruptly but were interested in quitting in the following three months.

b. Review authors broadly included studies of adult smokers but this study assessed participants who were interested in reducing smoking to quit.

c. Co-interventions (both groups): Behavioural (counselling and self-help).

d. No issues of bias were raised.

e. No substantive issues of indirectness. Unclear are how many participants from specialized medical setting and from indirect countries.

f. Confidence interval encompasses large benefit. The optimal information size not met (total of 227 events) and inadequate sample size (<2000 participants). We downrate this domain by -1.0.

g. Few studies despite comprehensive search. Unlikely due to suppression of results. We do not downrate this domain.

## Appendix K Table 14. Varenicline versus Placebo: Smoking cessation in smokers with schizophrenia, bipolar, or other psychiatric disorder

| Varenicline compared to placebo in smokers with schizophrenia, bipolar, or other psychiatric disorder  **Bibliography:** Cahill 2016; Date last searched: May 2015 | | | | | | | | | | | |
| --- | --- | --- | --- | --- | --- | --- | --- | --- | --- | --- | --- |
| **Certainty assessment** | | | | | | | **Summary of findings** | | | | |
| **№ of participants (studies) Follow-up** | **Risk of bias** | **Inconsistency** | **Indirectness** | **Imprecision** | **Publication bias** | **Overall certainty of evidence** | **Study event rates (%)** | | **Relative effect (95% CI)** | **Anticipated absolute effects** | |
|  |  |  |  |  |  |  | **With placebo** | **With Varenicline** |  | **Risk with placebo** | **Risk difference with Varenicline** |
| **Abstinence/cessation (follow up: 6 months)** ^a,b^  Outcome measurement: continuous/sustained abstinence 75%, point prevalence 25% studies  Biochemical validation: 100% studies | | | | | | | | | | | |
| 2332 (4 RCTs) | not serious ^c^ | not serious ^d^ | not serious ^e^ | not serious ^f^ | none ^g^ | ⨁⨁⨁⨁  HIGH | 93/1145 (8.1%) | 216/1187 (18.2%) | **RR 2.28** (1.82 to 2.87) | 81 per 1,000 | **104 more per 1,000** (from 67 more to 152 more) |

**CI:** Confidence interval; **RR:** Risk ratio

#### Explanations

a. 1 mg twice daily for 12 weeks except one study (5% evidence) with open label phase varenicline (12 wks) and varenicline vs placebo (40 wks) provided following abstinence.

b. Co-interventions (both groups): Behavioural for all studies, inclusive of counselling, telephone support, and specialized behavioural therapy (CBT and relapse prevention, 3.4% participants in varenicline and 4.1% in placebo groups).

c. Little risk of bias issues across evidence base to warrant downrating.

d. Point estimates similar and confidence intervals overlap. I2=0%, p=0.81. We do not downrate.

e. 2% of evidence in indirect setting (specialized medical) not substantive for downrating. 91% of evidence took place partially in indirect countries (proportion unknown); not factored into assessment.

f. Confidence interval encompasses one range of effect (moderate benefit to large benefit). The optimal information size not met (total of 309 events), but adequate sample size (>2000 participants). We did not downrate this domain.

g. Comprehensive search, some studies with null-inclusive results. We do not downrate.

| Summary of findings: | | | | | | |
| --- | --- | --- | --- | --- | --- | --- |
| **Varenicline compared to placebo in smokers with schizophrenia, bipolar, or other psychiatric disorder** | | | | | | |
| **Patient or population**: Smokers with schizophrenia, bipolar, or other psychiatric disorder  **Setting**: Not restricted  **Intervention**: Varenicline  **Comparison**: Placebo | | | | | | |
| Outcomes | **Anticipated absolute effects^*^** (95% CI) | | Relative effect (95% CI) | № of participants  (studies) | Certainty of the evidence (GRADE) | Comments |
|  | **Risk with placebo** | **Risk with Varenicline** |  |  |  |  |
| Abstinence/cessation ^a,b^  Outcome measurement: continuous/sustained abstinence 75%, point prevalence 25% studies  Biochemical validation: 100% studies  Follow up: 6 months | 81 per 1,000 | **185 per 1,000** (148 to 233) | **RR 2.28** (1.82 to 2.87) | 2332 (4 RCTs) | ⨁⨁⨁⨁  HIGH ^c,d,e,f,g^ | Fixed effects meta-analysis.  Date of last search: May 2015.  AMSTAR-2: Low.  Authors did not GRADE this analysis. |
| ***The risk in the intervention group** (and its 95% confidence interval) is based on the assumed risk in the comparison group and the **relative effect** of the intervention (and its 95% CI).   **CI:** Confidence interval; **RR:** Risk ratio | | | | | | |
| **GRADE Working Group grades of evidence** **High certainty:** We are very confident that the true effect lies close to that of the estimate of the effect **Moderate certainty:** We are moderately confident in the effect estimate: The true effect is likely to be close to the estimate of the effect, but there is a possibility that it is substantially different **Low certainty:** Our confidence in the effect estimate is limited: The true effect may be substantially different from the estimate of the effect **Very low certainty:** We have very little confidence in the effect estimate: The true effect is likely to be substantially different from the estimate of effect | | | | | | |

**Explanations**

a. 1 mg twice daily for 12 weeks except one study (5% evidence) with open label phase varenicline (12 wks) and varenicline vs placebo (40 wks) provided following abstinence.

b. Co-interventions (both groups): Behavioural for all studies, inclusive of counselling, telephone support, and specialized behavioural therapy (CBT and relapse prevention, 3.4% participants in varenicline and 4.1% in placebo groups).

c. Little risk of bias issues across evidence base to warrant downrating.

d. Point estimates similar and confidence intervals overlap. I2=0%, p=0.81. We do not downrate.

e. 2% of evidence in indirect setting (specialized medical) not substantive for downrating. 91% of evidence took place partially in indirect countries (proportion unknown); not factored into assessment.

f. Confidence interval encompasses one range of effect (moderate benefit to large benefit). The optimal information size not met (total of 309 events), but adequate sample size (>2000 participants). We did not downrate this domain.

g. Comprehensive search, some studies with null-inclusive results. We do not downrate.

## Appendix K Table 15. Varenicline versus Placebo: Smoking cessation in smokers with depression and motivated/wishing to quit

| Varenicline compared to placebo in smokers with depression and motivated/wishing to quit  **Bibliography:** Cahill 2016; Date of last search: May 2015 | | | | | | | | | | | |
| --- | --- | --- | --- | --- | --- | --- | --- | --- | --- | --- | --- |
| **Certainty assessment** | | | | | | | **Summary of findings** | | | | |
| **№ of participants (studies) Follow-up** | **Risk of bias** | **Inconsistency** | **Indirectness** | **Imprecision** | **Publication bias** | **Overall certainty of evidence** | **Study event rates (%)** | | **Relative effect (95% CI)** | **Anticipated absolute effects** | |
|  |  |  |  |  |  |  | **With placebo** | **With Varenicline** |  | **Risk with placebo** | **Risk difference with Varenicline** |
| **Abstinence/cessation (follow up: 12 months)** ^a,b^  Outcome measurement: continuous/sustained abstinence 100% studies  Biochemical validation: 100% studies | | | | | | | | | | | |
| 523 (1 RCT) | not serious ^c^ | not serious | not serious ^d^ | serious ^e^ | none ^f^ | ⨁⨁⨁◯ MODERATE | 28/269 (10.4%) | 52/254 (20.5%) | **RR 1.97** (1.28 to 3.01) | 104 per 1,000 | **101 more per 1,000** (from 29 more to 209 more) |

**CI:** Confidence interval; **RR:** Risk ratio

#### Explanations

a. 1 mg twice daily for 12 weeks.

b. Co-interventions (both groups): Behavioural (counselling, (telephone) support).

c. No issues of bias were raised

d. No substantive information to warrant downrating. This study was conducted partially in indirect countries (proportion unknown); not factored into assessment.

e. Confidence interval encompasses two ranges of effect (small but important benefit to large benefit). The optimal information size not met (total of 80 events) and inadequate sample size (<2000 participants). We downrate this domain by -1.5.

f. Few studies, comprehensive search. We do not downrate.

| Summary of findings: | | | | | | |
| --- | --- | --- | --- | --- | --- | --- |
| **Varenicline compared to placebo in smokers with depression and motivated/wishing to quit** | | | | | | |
| **Patient or population**: Smokers with depression and motivated/wishing to quit  **Setting**: Not restricted  **Intervention**: Varenicline  **Comparison**: Placebo | | | | | | |
| Outcomes | **Anticipated absolute effects^*^** (95% CI) | | Relative effect (95% CI) | № of participants  (studies) | Certainty of the evidence (GRADE) | Comments |
|  | **Risk with placebo** | **Risk with Varenicline** |  |  |  |  |
| Abstinence/cessation ^a,b^  Outcome measurement: continuous/sustained abstinence 100% studies  Biochemical validation: 100% studies  Follow up: 12 months | 104 per 1,000 | **205 per 1,000** (133 to 313) | **RR 1.97** (1.28 to 3.01) | 523 (1 RCT) | ⨁⨁⨁◯ MODERATE ^c,d,e,f^ | Date of last search: May 2015.  AMSTAR-2: Low.  Authors did not GRADE this analysis. |
| ***The risk in the intervention group** (and its 95% confidence interval) is based on the assumed risk in the comparison group and the **relative effect** of the intervention (and its 95% CI).   **CI:** Confidence interval; **RR:** Risk ratio | | | | | | |
| **GRADE Working Group grades of evidence** **High certainty:** We are very confident that the true effect lies close to that of the estimate of the effect **Moderate certainty:** We are moderately confident in the effect estimate: The true effect is likely to be close to the estimate of the effect, but there is a possibility that it is substantially different **Low certainty:** Our confidence in the effect estimate is limited: The true effect may be substantially different from the estimate of the effect **Very low certainty:** We have very little confidence in the effect estimate: The true effect is likely to be substantially different from the estimate of effect | | | | | | |

**Explanations**

a. 1 mg twice daily for 12 weeks.

b. Co-interventions (both groups): Behavioural (counselling, (telephone) support).

c. No issues of bias were raised

d. No substantive information to warrant downrating. This study was conducted partially in indirect countries (proportion unknown); not factored into assessment.

e. Confidence interval encompasses two ranges of effect (small but important benefit to large benefit). The optimal information size not met (total of 80 events) and inadequate sample size (<2000 participants). We downrate this domain by -1.5.

f. Few studies, comprehensive search. We do not downrate.

## Appendix K Table 16. Varenicline versus Placebo: Smoking cessation in smokers who previously failed to quit on varenicline but are motivated/wishing to try quitting again

| Varenicline compared to placebo in smokers who previously failed to quit on varenicline but are motivated/wishing to try quitting again  **Bibliography:** Cahill 2016; Date of last search: May 2015 | | | | | | | | | | | |
| --- | --- | --- | --- | --- | --- | --- | --- | --- | --- | --- | --- |
| **Certainty assessment** | | | | | | | **Summary of findings** | | | | |
| **№ of participants (studies) Follow-up** | **Risk of bias** | **Inconsistency** | **Indirectness** | **Imprecision** | **Publication bias** | **Overall certainty of evidence** | **Study event rates (%)** | | **Relative effect (95% CI)** | **Anticipated absolute effects** | |
|  |  |  |  |  |  |  | **With placebo** | **With Varenicline** |  | **Risk with placebo** | **Risk difference with Varenicline** |
| **Abstinence/cessation (follow up: 12 months)** ^a,b^  Outcome measurement: continuous/sustained abstinence 100% studies  Biochemical validation 100% studies | | | | | | | | | | | |
| 494 (1 RCT) | serious ^c^ | not serious | not serious ^d^ | serious ^e^ | none ^f^ | ⨁⨁◯◯  LOW | 8/245 (3.3%) | 50/249 (20.1%) | **RR 6.15** (2.98 to 12.70) | 33 per 1,000 | **168 more per 1,000** (from 65 more to 382 more) |

**CI:** Confidence interval; **RR:** Risk ratio

#### Explanations

a. 1 mg twice daily for 12 weeks.

b. Co-interventions (both groups): Behavioural (counselling).

c. Unclear risk of bias for selection bias. We downrate by -1.0.

d. No substantive issues of indirectness. Unclear are how many participants from hospital setting.

e. Confidence interval encompasses one range of effect (moderate benefit to large benefit). The optimal information size not met (total of 58 events) and inadequate sample size (<2000 participants). We downrate this domain by -1.0.

f. Few studies despite comprehensive search. Unlikely due to suppression of results. We do not downrate this domain.

| Summary of findings: | | | | | | |
| --- | --- | --- | --- | --- | --- | --- |
| **Varenicline compared to placebo in smokers who previously failed to quit on varenicline but are motivated/wishing to try quitting again** | | | | | | |
| **Patient or population**: Smokers who previously failed to quit on varenicline but are motivated/wishing to try quitting again  **Setting**: Not restricted  **Intervention**: Varenicline  **Comparison**: Placebo | | | | | | |
| Outcomes | **Anticipated absolute effects^*^** (95% CI) | | Relative effect (95% CI) | № of participants  (studies) | Certainty of the evidence (GRADE) | Comments |
|  | **Risk with placebo** | **Risk with Varenicline** |  |  |  |  |
| Abstinence/cessation ^a,b^  Outcome measurement: continuous/sustained abstinence 100% studies  Biochemical validation: 100% studies  Follow up: 12 months | 33 per 1,000 | **201 per 1,000** (97 to 415) | **RR 6.15** (2.98 to 12.70) | 494 (1 RCT) | ⨁⨁◯◯  LOW ^c,d,e,f^ | Date of last search: May 2015.  AMSTAR-2: Low.  Authors did not GRADE this analysis. |
| ***The risk in the intervention group** (and its 95% confidence interval) is based on the assumed risk in the comparison group and the **relative effect** of the intervention (and its 95% CI).   **CI:** Confidence interval; **RR:** Risk ratio | | | | | | |
| **GRADE Working Group grades of evidence** **High certainty:** We are very confident that the true effect lies close to that of the estimate of the effect **Moderate certainty:** We are moderately confident in the effect estimate: The true effect is likely to be close to the estimate of the effect, but there is a possibility that it is substantially different **Low certainty:** Our confidence in the effect estimate is limited: The true effect may be substantially different from the estimate of the effect **Very low certainty:** We have very little confidence in the effect estimate: The true effect is likely to be substantially different from the estimate of effect | | | | | | |

**Explanations**

a. 1 mg twice daily for 12 weeks.

b. Co-interventions (both groups): Behavioural (counselling).

c. Unclear risk of bias for selection bias. We downrate by -1.0.

d. No substantive issues of indirectness. Unclear are how many participants from hospital setting.

e. Confidence interval encompasses one range of effect (moderate benefit to large benefit). The optimal information size not met (total of 58 events) and inadequate sample size (<2000 participants). We downrate this domain by -1.0.

f. Few studies despite comprehensive search. Unlikely due to suppression of results. We do not downrate this domain.

## Appendix K Table 17. Varenicline versus Placebo: Adverse effects in general/mixed population of smokers

| Varenicline compared to placebo in general/mixed population of smokers  **Bibliography:** Cahill 2016; Date of last search: May 2015 | | | | | | | | | | | |
| --- | --- | --- | --- | --- | --- | --- | --- | --- | --- | --- | --- |
| **Certainty assessment** | | | | | | | **Summary of findings** | | | | |
| **№ of participants (studies) Follow-up** | **Risk of bias** | **Inconsistency** | **Indirectness** | **Imprecision** | **Publication bias** | **Overall certainty of evidence** | **Study event rates (%)** | | **Relative effect (95% CI)** | **Anticipated absolute effects** | |
|  |  |  |  |  |  |  | **With placebo** | **With Varenicline** |  | **Risk with placebo** | **Risk difference with Varenicline** |
| **Nausea (follow up: Range of follow-up times)** ^a,b,c^  Outcome measurement: NR | | | | | | | | | | | |
| 14963 (32 RCTs) | not serious ^i^ | not serious ^d^ | not serious ^e^ | not serious ^f^ | none ^g^ | ⨁⨁⨁⨁  HIGH | 596/7034 (8.5%) | 2207/7929 (27.8%) | **RR 3.27** (3.00 to 3.55) | 85 per 1,000 | **192 more per 1,000** (from 169 more to 216 more) |
| **Insomnia (follow up: Range of follow-up times)** ^a,c,h^  Outcome measurement: NR | | | | | | | | | | | |
| 14447 (29 RCTs) | not serious ^i^ | not serious ^j^ | not serious ^k^ | not serious ^l^ | none ^g^ | ⨁⨁⨁⨁  HIGH | 562/6777 (8.3%) | 976/7670 (12.7%) | **RR 1.49** (1.35 to 1.65) | 83 per 1,000 | **41 more per 1,000** (from 29 more to 54 more) |
| **Abnormal dreams (follow up: Range of follow-up times)** ^a,m,n^  Outcome measurement: NR | | | | | | | | | | | |
| 13682 (26 RCTs) | not serious ^i^ | serious ^o^ | not serious ^p^ | not serious ^q^ | none ^g^ | ⨁⨁⨁◯  MODERATE | 365/6393 (5.7%) | 912/7289 (12.5%) | **RR 2.12** (1.88 to 2.38) | 57 per 1,000 | **64 more per 1,000** (from 50 more to 79 more) |
| **Headache (follow up: Range of follow-up times)** ^a,h,r^  Outcome measurement: NR | | | | | | | | | | | |
| 13835 (25 studies) | not serious ^i^ | not serious ^s^ | not serious ^t^ | not serious ^u^ | none ^g^ | ⨁⨁⨁⨁  HIGH | 668/6531 (10.2%) | 894/7304 (12.2%) | **RR 1.17** (1.07 to 1.29) | 102 per 1,000 | **17 more per 1,000** (from 7 more to 30 more) |
| **Depression (follow up: Range of follow-up times)** ^v,w,x^  Outcome measurement: NR | | | | | | | | | | | |
| 16189 (36 studies) | not serious ^i^ | not serious ^y^ | not serious ^z^ | not serious ^aa^ | none ^g^ | ⨁⨁⨁⨁  HIGH | 184/7652 (2.4%) | 202/8537 (2.4%) | **RR 0.94** (0.77 to 1.14) | 24 per 1,000 | **1 fewer per 1,000** (from 6 fewer to 3 more) |
| **Suicidal ideation (follow up: Range of follow-up times)** ^v,ab,ac^  Outcome measurement: NR | | | | | | | | | | | |
| 11193 (24 studies) | not serious ^i^ | not serious ^ad^ | not serious ^ae^ | not serious ^af^ | none ^g^ | ⨁⨁⨁⨁  HIGH | 37/5288 (0.7%) | 29/5905 (0.5%) | **RR 0.68** (0.43 to 1.07) | 7 per 1,000 | **2 fewer per 1,000** (from 4 fewer to 0 fewer) |
| **At least one serious adverse event (follow up: Range of follow-up times)** ^a,ag,m^  Outcome measurement: NR | | | | | | | | | | | |
| 15370 (29 RCTs) | not serious ^i^ | not serious ^ah^ | not serious ^ai^ | not serious ^aj^ | none ^g^ | ⨁⨁⨁⨁  HIGH | 196/7245 (2.7%) | 269/8125 (3.3%) | **RR 1.25** (1.04 to 1.49) | 27 per 1,000 | **7 more per 1,000** (from 1 more to 13 more) |
| **At least one serious adverse event during or immediately after treatment (follow up: Range of follow-up times)** ^a,ak,al^  Outcome measurement: NR | | | | | | | | | | | |
| 15000 (26 RCTs) | not serious ^i^ | not serious ^am^ | not serious ^an^ | not serious ^ao^ | none ^g^ | ⨁⨁⨁⨁  HIGH | 163/7096 (2.3%) | 225/7904 (2.8%) | **RR 1.25** (1.02 to 1.52) | 23 per 1,000 | **6 more per 1,000** (from 0 fewer to 12 more) |
| **Neuropsychiatric events (depression, suicidal ideation), not deaths (follow up: Range of follow-up times)** ^a,ap,m^  Outcome measurement: NR | | | | | | | | | | | |
| 8955 (23 RCTs) | not serious ^i^ | not serious ^aq^ | not serious ^ar^ | not serious ^as^ | none ^g^ | ⨁⨁⨁⨁  HIGH | 43/4035 (1.1%) | 41/4920 (0.8%) | **RR 0.82** (0.57 to 1.19) | 11 per 1,000 | **2 fewer per 1,000** (from 5 fewer to 2 more) |
| **Cardiac serious adverse events, including deaths (follow up: Range of follow-up times)** ^a,at,m^  Outcome measurement: NR | | | | | | | | | | | |
| 8587 (21 RCTs) | serious ^au^ | not serious ^av^ | serious ^aw^ | not serious ^ax^ | none ^g^ | ⨁⨁◯◯  LOW | 35/3891 (0.9%) | 57/4696 (1.2%) | **RR 1.36** (0.91 to 2.04) | 9 per 1,000 | **3 more per 1,000** (from 1 fewer to 9 more) |
| **Discontinuation (follow up: Range of follow-up times)** ^ay,az,aaa^  Outcome measurement: NR | | | | | | | | | | | |
| NR (4 RCTs) | not serious ^i^ | not serious ^aab^ | not serious ^aac^ | not serious to very serious ^aad^ | none ^aae^ | unable to assess | Across three studies, treatment discontinuation ranged from 9.5% to 28% with varenicline and from 8% to 10% in the placebo group.  In the fourth study, where study discontinuation was assessed during the 12-week varenicline open label phase, 32% exited the study because of discontinuation, non-adherence to protocol, and relapse. | | | | |

**CI:** Confidence interval; **RR:** Risk ratio; **NR**: Not reported

#### Explanations

a. Most studies 1 mg twice daily for 12 weeks.

b. Review authors broadly included studies of adult smokers but a mix of trial populations in this analysis: 50% motivated to quit (less than half are co-morbid populations: COPD, mental health, CVD, substance use), 31% unrestricted (general/mixed) populations, and the remainder were small frequencies of other populations (co-morbid, quitters, reduction in use).

c. Co-interventions (both groups): Most behavioural, inclusive of counselling, (telephone) support, self-help, advice. The following provides proportion of participants in each group (varenicline vs placebo), for each analysis, receiving specialized counselling. Nausea (CBT, relapse prevention, MI), 2.5% vs 1%. Insomnia (CBT, relapse prevention, MI), 1.6% vs 2.2%. Abnormal dreams (CBT, relapse prevention, MI), 1.7% vs 2.4%. Headache (CBT, relapse prevention, MI), 1.7% vs 2.3%. Depression (CBT, relapse prevention, MI), 1.5% vs 2%. Suicidal ideation (CBT, relapse prevention, MI), 2.1% vs 2.9%. One or more serious adverse events (CBT, relapse, MI), 2.4% vs 2.1%. Serious adverse events during or immediately after treatment (MI), 1.1% vs 1.5%. Neuropsychiatric events (CBT, relapse prevention), 0.8% vs 1.2%. Cardiac serious adverse events (CBT, relapse prevention, MI), 2.7% vs 3.9%. Discontinuation (CBT, relapse prevention), subset not quantifiable due to lack of denominator for the analysis.

d. Some variation in point estimates across evidence base but the outliers not weighted heavily in the analysis. Confidence intervals overlap across majority. I2=22%, p=0.13. We do not downrate this domain.

e. Very little of the evidence (8%) is indirect. We do not downrate this domain. Not included in the assessment were studies with small or unclear indirectness: 0.1% for indirect country, 41.5% evidence with unknown proportion indirect country, and 14% unknown proportion in specialized medical settings.

f. Confidence interval encompasses small but important harm. The optimal information size is met (2803 events) and large sample size (>2000). We did not downrate this domain.

g. Mix of trial results and comprehensive literature search. We do not downrate.

h. Review authors broadly included studies of adult smokers but a mix of trial populations in this analysis: 56% motivated to quit (more than half are co-morbid populations: COPD, mental health, CVD, substance use), 28% unrestricted (general/mixed) populations, and the remainder were small frequencies of other populations (co-morbid, reduction in use).

i. Issues of bias across evidence base not substantive enough to warrant downrating.

j. Little variation in point estimates across evidence base but the outliers not weighted heavily in the analysis. Confidence intervals overlap across majority. I2=0%, p=0.55. We do not downrate this domain.

k. Very little of the evidence (6%) is indirect. We do not downrate this domain. Not included in the assessment were studies with small or unclear indirectness: 0.1% for indirect country, 52% evidence with unknown proportion indirect country or in specialized medical settings.

l. Confidence interval encompasses small but important harm. The optimal information size is met (1538 events) and large sample size (>2000). We did not downrate this domain.

m. Co-interventions (both groups): Most behavioural, inclusive of counselling, (telephone) support, self-help, advice; 4% studies with specialized behavioural counselling.

n. Review authors broadly included studies of adult smokers but a mix of trial populations in this analysis: 58% motivated to quit (more than half are co-morbid populations: COPD, mental health, CVD, substance use), 19% unrestricted (general/mixed) populations, and the remainder were small frequencies of other populations (co-morbid, quitters, reduction in use).

o. Variation in point estimates across evidence base, not all confidence intervals overlap. I2=62%, p<0.0001. We downrate this domain by -1.0.

p. 13% indirectness across the evidence is not substantive. We do not downrate this domain. Not included in the assessment were 53% evidence with unclear proportion of indirectness (country, specialized medical settings).

q. Confidence interval encompasses small but important harm. The optimal information size is met (1277 events) and large sample size (>2000). We did not downrate this domain.

r. Co-interventions (both groups): All behavioural, inclusive of counselling, (telephone) support, self-help, advice; 4% studies with specialized behavioural counselling.

s. Variation in point estimates across evidence base, confidence intervals largely overlap. Outlier estimates contributed little weight in the meta-analysis. I2=27%, p=0.11. We do not downrate this domain.

t. 21% indirectness across the evidence (inpatient and specialized medical settings). We downrate this domain by -0.5. Not included in the assessment were 53% evidence with unclear proportion of indirectness (country, specialized medical settings).

u. Confidence interval encompasses little to no difference. The optimal information size is met (1562 events) and large sample size (>2000). We did not downrate this domain.

v. Many studies 1 mg twice daily for 12 weeks. Not reported for studies not included for efficacy.

w. Review authors broadly included studies of adult smokers but a mix of trial populations in this analysis: 42% motivated to quit (more than half are co-morbid populations: COPD, mental health, CVD, substance use), 22% were not reported, 19% unrestricted (general/mixed) populations, and the remainder were small frequencies of other populations (co-morbid, reduction in use, quitters).

x. Co-interventions (both groups): Behavioural in most, inclusive of counselling, (telephone) support, self-help, advice; 3% of studies with specialized behavioural counselling. Not reported in 22%.

y. Variation in point estimates across evidence base, but confidence intervals overlap. Outlier estimates contributed little weight in the meta-analysis. I2=0%, p=0.90. We do not downrate this domain.

z. 23% indirectness across the evidence (mainly inpatient and specialized medical settings). We downrate by -0.5. Not included in the assessment was 40% evidence with unclear proportion of indirectness (country, specialized medical settings).

aa. Confidence interval encompasses little to no difference. The optimal information size was not quite met (386 events) but we did not think the concerns were serious and large sample size (>2000). We did not downrate this domain.

ab. Review authors broadly included studies of adult smokers but a mix of trial populations in this analysis: 42% motivated to quit (less than half are co-morbid populations: COPD, mental health, CVD, substance use), 21% were not reported, 8% unrestricted (general/mixed) populations, and the remainder were small frequencies of other populations (co-morbid, reduction in use, quitters).

ac. Co-interventions (both groups): Behavioural: most. Inclusive of counselling, (telephone) support, self-help, advice; 4% with specialized behavioural counselling. 25% NR.

ad. Variation in point estimates across evidence base, but confidence intervals overlap. Outlier estimates contributed little weight in the meta-analysis. I2=0%, p=0.98. We do not downrate this domain.

ae. 5.5% indirectness across the evidence (specialized medical settings). We do not downrate. Not included in the assessment was 51% evidence with unclear proportion of indirectness (country, specialized medical settings).

af. Confidence interval encompasses little to no difference. The optimal information size is not met (66 events), but large sample size (>2000). We did not downrate this domain.

ag. Review authors broadly included studies of adult smokers but a mix of trial populations in this analysis: 50% motivated to quit (half are co-morbid populations: COPD, mental health, CVD, substance use), 42% unrestricted (general/mixed) populations, and the remainder were small frequencies of other populations (co-morbid, reduction in use, quitters).

ah. Some variation in point estimates across evidence base, but confidence intervals overlap. Outlier estimates contributed little weight in the meta-analysis. I2=0%, p=0.90. We do not downrate this domain.

ai. 22% indirectness across the evidence (specialized medical settings). We downrate by -0.5. Not included in the assessment was 48% evidence with unclear proportion of indirectness (country, specialized medical settings).

aj. Confidence interval encompasses one range of effect (little to no difference to small but important harm). The optimal information size is met (465 events) and large sample size (>2000). We did not downrate this domain.

ak. Review authors broadly included studies of adult smokers but a mix of trial populations in this analysis: 50% motivated to quit (more than half are co-morbid populations: COPD, mental health, CVD, substance use), 38% unrestricted (general/mixed) populations, and the remainder were small frequencies of other populations (co-morbid, reduction in use, quitters).

al. Co-interventions (both groups): Behavioural: most. Inclusive of counselling, (telephone) support, self-help, advice; none with specialized behavioural counselling.

am. Some variation in point estimates across evidence base, but confidence intervals overlap. Outlier estimates contributed little weight in the meta-analysis. I2=0%, p=0.80. We do not downrate this domain.

an. 21% indirectness across the evidence (specialized medical settings). We downrate by -0.5. Not included in the assessment was 53% evidence with unclear proportion of indirectness (country, specialized medical settings).

ao. Confidence interval encompasses one range of effect (little to no difference to small but important harm). The optimal information size was not quite met (388 events), but large sample size (>2000). We did not downrate this domain.

ap. Review authors broadly included studies of adult smokers but a mix of trial populations in this analysis: 52% motivated to quit (more than half are co-morbid populations: COPD, mental health, CVD, substance use), 39% unrestricted (general/mixed) populations, and the remainder were small frequencies of other populations (mental health, quitters).

aq. Variation in point estimates across evidence base, but confidence intervals overlap. Outlier estimates contributed little weight in the meta-analysis. I2=0%, p=0.70. We do not downrate this domain.

ar. 8.1% indirectness across the evidence (specialized medical settings, inpatient). We do not downrate. Not included in the assessment was 14% evidence with unclear proportion of indirectness (country, specialized medical settings).

as. Confidence interval encompasses little to no difference. The optimal information size is not met (84 events), but large sample size (>2000). We did not downrate this domain

at. Review authors broadly included studies of adult smokers but a mix of trial populations in this analysis: 48% motivated to quit (half are co-morbid populations: COPD, CVD, mental health), 33% unrestricted (general/mixed) populations, and the remainder were small frequencies of other populations (co-morbid, quitters).

au. 67% of evidence at unclear risk of bias, mainly due to issues of selection bias and blinding. We downrate by -1.0.

av. Variation in point estimates across evidence base, but confidence intervals overlap. Outlier estimates contributed little weight in the meta-analysis. I2=0%, p=0.94. We do not downrate this domain.

aw. 43% indirectness across the evidence (specialized medical settings, inpatient, quitters). We downrate by -1.0. Not included in the assessment was 24% evidence with unclear proportion of indirectness (country, specialized medical settings).

ax. Confidence interval encompasses one range of effect (little to no difference to small but important harm). The optimal information size is not met (92 events), but large sample size (>2000). We did not downrate this domain.

ay. Half of studies 1 mg twice daily for 40 wk.

az. Review authors broadly included studies of adult smokers. 75% unrestricted (general/mixed) populations, and one study in people with mental health diagnoses.

aaa. Co-interventions (both groups): All behavioural, inclusive of counselling and self-help; 25% specialized behavioural counselling.

aab. Given information, variation exists in the data and lends to some inconsistency. We downrate by -0.5.

aac. No issues of indirectness identified.

aad. Unable to assess confidence intervals. Unable to assess optimal information size and sample size not reported. We cannot rate this domain.

aae. Difficult to assess due to nature of reported data. Comprehensive literature search. We do not downrate this domain.

| Summary of findings: | | | | | | |
| --- | --- | --- | --- | --- | --- | --- |
| **Varenicline compared to placebo in general/mixed population of smokers** | | | | | | |
| **Patient or population**: General/mixed population of smokers  **Setting**: Not restricted  **Intervention**: Varenicline  **Comparison**: Placebo | | | | | | |
| Outcomes | **Anticipated absolute effects^*^** (95% CI) | | Relative effect (95% CI) | № of participants  (studies) | Certainty of the evidence (GRADE) | Comments |
|  | **Risk with placebo** | **Risk with Varenicline** |  |  |  |  |
| Nausea ^a,b,c^  Outcome measurement: NR  Follow up: Range of follow-up times | 85 per 1,000 | **277 per 1,000** (254 to 301) | **RR 3.27** (3.00 to 3.55) | 14963 (32 RCTs) | ⨁⨁⨁⨁  HIGH ^d,e,f,g,i^ | Fixed effects meta-analysis.  Date of last search: May 2015.  AMSTAR-2: Low.  Review authors rated as high certainty. |
| Insomnia ^a,c,h^  Outcome measurement: NR  Follow up: Range of follow-up times | 83 per 1,000 | **124 per 1,000** (112 to 137) | **RR 1.49** (1.35 to 1.65) | 14447 (29 RCTs) | ⨁⨁⨁⨁  HIGH ^g,i,j,k,l^ | Fixed effects meta-analysis.  Date of last search: May 2015.  AMSTAR-2: Low.  Review authors did not GRADE this analysis. |
| Abnormal dreams ^a,m,n^  Outcome measurement: NR  Follow up: Range of follow-up times | 57 per 1,000 | **121 per 1,000** (107 to 136) | **RR 2.12** (1.88 to 2.38) | 13682 (26 RCTs) | ⨁⨁⨁◯  MODERATE ^g,i,o,p,q^ | Fixed effects meta-analysis.  Date of last search: May 2015.  AMSTAR-2: Low.  Review authors did not GRADE this analysis. |
| Headache ^a,h,r^  Outcome measurement: NR  Follow up: Range of follow-up times | 102 per 1,000 | **120 per 1,000** (109 to 132) | **RR 1.17** (1.07 to 1.29) | 13835 (25 studies) | ⨁⨁⨁⨁  HIGH ^g,i,s,t,u^ | Fixed effects meta-analysis.  Date of last search: May 2015.  AMSTAR-2: Low.  Review authors did not GRADE this analysis. |
| Depression ^v,w,x^  Outcome measurement: NR  Follow up: Range of follow-up times | 24 per 1,000 | **23 per 1,000** (19 to 27) | **RR 0.94** (0.77 to 1.14) | 16189 (36 studies) | ⨁⨁⨁⨁  HIGH ^g,i,y,z,aa^ | Fixed effects meta-analysis.  Date of last search: May 2015.  AMSTAR-2: Low.  Review authors did not GRADE this analysis. |
| Suicidal ideation ^v,ab,ac^  Outcome measurement: NR  Follow up: Range of follow-up times | 7 per 1,000 | **5 per 1,000** (3 to 7) | **RR 0.68** (0.43 to 1.07) | 11193 (24 studies) | ⨁⨁⨁⨁  HIGH ^g,i,ad,ae,af^ | Fixed effects meta-analysis.  Date of last search: May 2015.  AMSTAR-2: Low.  Review authors did not GRADE this analysis. |
| At least one serious adverse event ^a,m,ag^  Outcome measurement: NR  Follow up: Range of follow-up times | 27 per 1,000 | **34 per 1,000** (28 to 40) | **RR 1.25** (1.04 to 1.49) | 15370 (29 RCTs) | ⨁⨁⨁⨁  HIGH ^g,i,ah,ai,aj^ | Fixed effects meta-analysis.  Companion secondary analysis in next row.  Authors state Peto OR analysis for this analysis of rare events did not change the findings.  Date of last search: May 2015.  AMSTAR-2: Low.  Review authors rate as high certainty. |
| At least one serious adverse event during or immediately after treatment ^a,ak,al^  Outcome measurement: NR  Follow up: Range of follow-up times | 23 per 1,000 | **29 per 1,000** (23 to 35) | **RR 1.25** (1.02 to 1.52) | 15000 (26 RCTs) | ⨁⨁⨁⨁  HIGH ^g,i,am,an,ao^ | Refer to companion analysis above.  Fixed effects meta-analysis.  Date of last search: May 2015.  AMSTAR-2: Low.  Review authors did not GRADE this analysis. |
| Neuropsychiatric events (depression, suicidal ideation), not deaths ^a,m,ap^  Outcome measurement: NR  Follow up: Range of follow-up times | 11 per 1,000 | **9 per 1,000** (6 to 13) | **RR 0.82** (0.57 to 1.19) | 8955 (23 RCTs) | ⨁⨁⨁⨁  HIGH ^g,i,aq,ar,as^ | Fixed effects meta-analysis.  Date of last search: May 2015.  AMSTAR-2: Low.  Review authors did not GRADE this analysis. |
| Cardiac serious adverse events, including deaths ^a,m,at^  Outcome measurement: NR  Follow up: Range of follow-up times | 9 per 1,000 | **12 per 1,000** (8 to 18) | **RR 1.36** (0.91 to 2.04) | 8587 (21 RCTs) | ⨁⨁◯◯  LOW ^g,au,av,aw,ax^ | Fixed effects meta-analysis.  Date of last search: May 2015.  AMSTAR-2: Low.  Review authors rate as moderate certainty. ^ay^ |
| Discontinuation ^az,aaa,aab^  Outcome measurement: NR  Follow up: Range of follow-up times | Across three studies, treatment discontinuation ranged from 9.5% to 28% with varenicline and from 8% to 10% in the placebo group.  In the fourth study, where study discontinuation was assessed during the 12-week varenicline open label phase, 32% exited the study because of discontinuation, non-adherence to protocol, and relapse. | |  | NR  (4 RCTs) | unable to assess ^i,aac,aad,aae,aaf^ | Date of last search: May 2015.  AMSTAR-2: Low.  Review authors did not GRADE this analysis. |
| ***The risk in the intervention group** (and its 95% confidence interval) is based on the assumed risk in the comparison group and the **relative effect** of the intervention (and its 95% CI).   **CI:** Confidence interval; **RR:** Risk ratio | | | | | | |
| **GRADE Working Group grades of evidence** **High certainty:** We are very confident that the true effect lies close to that of the estimate of the effect **Moderate certainty:** We are moderately confident in the effect estimate: The true effect is likely to be close to the estimate of the effect, but there is a possibility that it is substantially different **Low certainty:** Our confidence in the effect estimate is limited: The true effect may be substantially different from the estimate of the effect **Very low certainty:** We have very little confidence in the effect estimate: The true effect is likely to be substantially different from the estimate of effect | | | | | | |

**Explanations**

a. Most studies 1 mg twice daily for 12 weeks.

b. Review authors broadly included studies of adult smokers but a mix of trial populations in this analysis: 50% motivated to quit (less than half are co-morbid populations: COPD, mental health, CVD, substance use), 31% unrestricted (general/mixed) populations, and the remainder were small frequencies of other populations (co-morbid, quitters, reduction in use).

c. Co-interventions (both groups): Most behavioural, inclusive of counselling, (telephone) support, self-help, advice. The following provides proportion of participants in each group (varenicline vs placebo), for each analysis, receiving specialized counselling. Nausea (CBT, relapse prevention, MI), 2.5% vs 1%. Insomnia (CBT, relapse prevention, MI), 1.6% vs 2.2%. Abnormal dreams (CBT, relapse prevention, MI), 1.7% vs 2.4%. Headache (CBT, relapse prevention, MI), 1.7% vs 2.3%. Depression (CBT, relapse prevention, MI), 1.5% vs 2%. Suicidal ideation (CBT, relapse prevention, MI), 2.1% vs 2.9%. One or more serious adverse events (CBT, relapse, MI), 2.4% vs 2.1%. Serious adverse events during or immediately after treatment (MI), 1.1% vs 1.5%. Neuropsychiatric events (CBT, relapse prevention), 0.8% vs 1.2%. Cardiac serious adverse events (CBT, relapse prevention, MI), 2.7% vs 3.9%. Discontinuation (CBT, relapse prevention), subset not quantifiable due to lack of denominator for the analysis.

d. Some variation in point estimates across evidence base but the outliers not weighted heavily in the analysis. Confidence intervals overlap across majority. I2=22%, p=0.13. We do not downrate this domain.

e. Very little of the evidence (8%) is indirect. We do not downrate this domain. Not included in the assessment were studies with small or unclear indirectness: 0.1% for indirect country, 41.5% evidence with unknown proportion indirect country, and 14% unknown proportion in specialized medical settings.

f. Confidence interval encompasses small but important harm. The optimal information size is met (2803 events) and large sample size (>2000). We did not downrate this domain.

g. Mix of trial results and comprehensive literature search. We do not downrate.

h. Review authors broadly included studies of adult smokers but a mix of trial populations in this analysis: 56% motivated to quit (more than half are co-morbid populations: COPD, mental health, CVD, substance use), 28% unrestricted (general/mixed) populations, and the remainder were small frequencies of other populations (co-morbid, reduction in use).

i. Issues of bias across evidence base not substantive enough to warrant downrating.

j. Little variation in point estimates across evidence base but the outliers not weighted heavily in the analysis. Confidence intervals overlap across majority. I2=0%, p=0.55. We do not downrate this domain.

k. Very little of the evidence (6%) is indirect. We do not downrate this domain. Not included in the assessment were studies with small or unclear indirectness: 0.1% for indirect country, 52% evidence with unknown proportion indirect country or in specialized medical settings.

l. Confidence interval encompasses small but important harm. The optimal information size is met (1538 events) and large sample size (>2000). We did not downrate this domain.

m. Co-interventions (both groups): Most behavioural, inclusive of counselling, (telephone) support, self-help, advice; 4% studies with specialized behavioural counselling.

n. Review authors broadly included studies of adult smokers but a mix of trial populations in this analysis: 58% motivated to quit (more than half are co-morbid populations: COPD, mental health, CVD, substance use), 19% unrestricted (general/mixed) populations, and the remainder were small frequencies of other populations (co-morbid, quitters, reduction in use).

o. Variation in point estimates across evidence base, not all confidence intervals overlap. I2=62%, p<0.0001. We downrate this domain by -1.0.

p. 13% indirectness across the evidence is not substantive. We do not downrate this domain. Not included in the assessment were 53% evidence with unclear proportion of indirectness (country, specialized medical settings).

q. Confidence interval encompasses small but important harm. The optimal information size is met (1277 events) and large sample size (>2000). We did not downrate this domain.

r. Co-interventions (both groups): All behavioural, inclusive of counselling, (telephone) support, self-help, advice; 4% studies with specialized behavioural counselling.

s. Variation in point estimates across evidence base, confidence intervals largely overlap. Outlier estimates contributed little weight in the meta-analysis. I2=27%, p=0.11. We do not downrate this domain.

t. 21% indirectness across the evidence (inpatient and specialized medical settings). We downrate this domain by -0.5. Not included in the assessment were 53% evidence with unclear proportion of indirectness (country, specialized medical settings).

u. Confidence interval encompasses little to no difference. The optimal information size is met (1562 events) and large sample size (>2000). We did not downrate this domain.

v. Many studies 1 mg twice daily for 12 weeks. Not reported for studies not included for efficacy.

w. Review authors broadly included studies of adult smokers but a mix of trial populations in this analysis: 42% motivated to quit (more than half are co-morbid populations: COPD, mental health, CVD, substance use), 22% were not reported, 19% unrestricted (general/mixed) populations, and the remainder were small frequencies of other populations (co-morbid, reduction in use, quitters).

x. Co-interventions (both groups): Behavioural in most, inclusive of counselling, (telephone) support, self-help, advice; 3% of studies with specialized behavioural counselling. Not reported in 22%.

y. Variation in point estimates across evidence base, but confidence intervals overlap. Outlier estimates contributed little weight in the meta-analysis. I2=0%, p=0.90. We do not downrate this domain.

z. 23% indirectness across the evidence (mainly inpatient and specialized medical settings). We downrate by -0.5. Not included in the assessment was 40% evidence with unclear proportion of indirectness (country, specialized medical settings).

aa. Confidence interval encompasses little to no difference. The optimal information size was not quite met (386 events) but we did not think the concerns were serious and large sample size (>2000). We did not downrate this domain.

ab. Review authors broadly included studies of adult smokers but a mix of trial populations in this analysis: 42% motivated to quit (less than half are co-morbid populations: COPD, mental health, CVD, substance use), 21% were not reported, 8% unrestricted (general/mixed) populations, and the remainder were small frequencies of other populations (co-morbid, reduction in use, quitters).

ac. Co-interventions (both groups): Behavioural: most. Inclusive of counselling, (telephone) support, self-help, advice; 4% with specialized behavioural counselling. 25% NR.

ad. Variation in point estimates across evidence base, but confidence intervals overlap. Outlier estimates contributed little weight in the meta-analysis. I2=0%, p=0.98. We do not downrate this domain.

ae. 5.5% indirectness across the evidence (specialized medical settings). We do not downrate. Not included in the assessment was 51% evidence with unclear proportion of indirectness (country, specialized medical settings).

af. Confidence interval encompasses little to no difference. The optimal information size is not met (66 events), but large sample size (>2000). We did not downrate this domain.

ag. Review authors broadly included studies of adult smokers but a mix of trial populations in this analysis: 50% motivated to quit (half are co-morbid populations: COPD, mental health, CVD, substance use), 42% unrestricted (general/mixed) populations, and the remainder were small frequencies of other populations (co-morbid, reduction in use, quitters).

ah. Some variation in point estimates across evidence base, but confidence intervals overlap. Outlier estimates contributed little weight in the meta-analysis. I2=0%, p=0.90. We do not downrate this domain.

ai. 22% indirectness across the evidence (specialized medical settings). We downrate by -0.5. Not included in the assessment was 48% evidence with unclear proportion of indirectness (country, specialized medical settings).

aj. Confidence interval encompasses one range of effect (little to no difference to small but important harm). The optimal information size is met (465 events) and large sample size (>2000). We did not downrate this domain.

ak. Review authors broadly included studies of adult smokers but a mix of trial populations in this analysis: 50% motivated to quit (more than half are co-morbid populations: COPD, mental health, CVD, substance use), 38% unrestricted (general/mixed) populations, and the remainder were small frequencies of other populations (co-morbid, reduction in use, quitters).

al. Co-interventions (both groups): Behavioural: most. Inclusive of counselling, (telephone) support, self-help, advice; none with specialized behavioural counselling.

am. Some variation in point estimates across evidence base, but confidence intervals overlap. Outlier estimates contributed little weight in the meta-analysis. I2=0%, p=0.80. We do not downrate this domain.

an. 21% indirectness across the evidence (specialized medical settings). We downrate by -0.5. Not included in the assessment was 53% evidence with unclear proportion of indirectness (country, specialized medical settings).

ao. Confidence interval encompasses one range of effect (little to no difference to small but important harm). The optimal information size was not quite met (388 events), but large sample size (>2000). We did not downrate this domain.

ap. Review authors broadly included studies of adult smokers but a mix of trial populations in this analysis: 52% motivated to quit (more than half are co-morbid populations: COPD, mental health, CVD, substance use), 39% unrestricted (general/mixed) populations, and the remainder were small frequencies of other populations (mental health, quitters).

aq. Variation in point estimates across evidence base, but confidence intervals overlap. Outlier estimates contributed little weight in the meta-analysis. I2=0%, p=0.70. We do not downrate this domain.

ar. 8.1% indirectness across the evidence (specialized medical settings, inpatient). We do not downrate. Not included in the assessment was 14% evidence with unclear proportion of indirectness (country, specialized medical settings).

as. Confidence interval encompasses little to no difference. The optimal information size is not met (84 events), but large sample size (>2000). We did not downrate this domain

at. Review authors broadly included studies of adult smokers but a mix of trial populations in this analysis: 48% motivated to quit (half are co-morbid populations: COPD, CVD, mental health), 33% unrestricted (general/mixed) populations, and the remainder were small frequencies of other populations (co-morbid, quitters).

au. 67% of evidence at unclear risk of bias, mainly due to issues of selection bias and blinding. We downrate by -1.0.

av. Variation in point estimates across evidence base, but confidence intervals overlap. Outlier estimates contributed little weight in the meta-analysis. I2=0%, p=0.94. We do not downrate this domain.

aw. 43% indirectness across the evidence (specialized medical settings, inpatient, quitters). We downrate by -1.0. Not included in the assessment was 24% evidence with unclear proportion of indirectness (country, specialized medical settings).

ax. Confidence interval encompasses one range of effect (little to no difference to small but important harm). The optimal information size is not met (92 events), but large sample size (>2000). We did not downrate this domain.

ay. Downrated due to imprecision as confidence intervals do not rule out an increase in risk.

az. Half of studies 1 mg twice daily for 40 wk.

aaa. Review authors broadly included studies of adult smokers. 75% unrestricted (general/mixed) populations, and one study in people with mental health diagnoses.

aab. Co-interventions (both groups): All behavioural, inclusive of counselling and self-help; 25% specialized behavioural counselling.

aac. Given information, variation exists in the data and lends to some inconsistency. We downrate by -0.5.

aad. No issues of indirectness identified.

aae. Unable to assess confidence intervals. Unable to assess optimal information size and sample size not reported. We cannot rate this domain.

aaf. Difficult to assess due to nature of reported data. Comprehensive literature search. We do not downrate this domain.

# Farley 2012 {1469}

## Appendix K Table 18. Bupropion versus Placebo: Weight gain in smokers motivated to quit at baseline and abstinent at follow-up

| Bupropion (300 mg/day) compared to placebo in smokers motivated/wishing to quit at baseline and abstinent at follow-up  **Bibliography:** Farley 2012; Date of last search: September 2011 | | | | | | | | | | | |
| --- | --- | --- | --- | --- | --- | --- | --- | --- | --- | --- | --- |
| **Certainty assessment** | | | | | | | **Summary of findings** | | | | |
| **№ of participants (studies) Follow-up** | **Risk of bias** | **Inconsistency** | **Indirectness** | **Imprecision** | **Publication bias** | **Overall certainty of evidence** | **Study event rates (%)** | | **Relative effect (95% CI)** | **Anticipated absolute effects** | |
|  |  |  |  |  |  |  | **With placebo** | **With Bupropion (300 mg/day)** |  | **Risk with placebo** | **Risk difference with Bupropion (300 mg/day)** |
| **Weight gain in abstinent smokers (follow up: End of treatment)** ^a,b,c^  Mean (SD) weight change (kg) from baseline assessed in abstainers (continuous/sustained 28.5%, prolonged 42.9%, point prevalence 28.5% studies)  Smoking abstinence biochemically validated in all trials | | | | | | | | | | | |
| 869 (7 RCTs) | serious ^d^ | not serious ^e^ | not serious ^f^ | not serious ^g^ | none ^h^ | ⨁⨁⨁◯ MODERATE | 264 | 605 | - | The mean weight gain in abstinent smokers ranged from 2.32-4.0 kg | MD 1.12 kg lower (1.47 lower to 0.77 lower) |
| **Weight gain in abstinent smokers (follow up: 6 months)** ^i,j,k^  Mean (SD) weight change (kg) from baseline assessed in abstainers (continuous/sustained 75%, prolonged 25% studies)  Smoking abstinence biochemically validated in all trials | | | | | | | | | | | |
| 218 (4 RCTs) | serious ^l^ | not serious ^m^ | not serious ^n^ | serious ^o^ | none ^p^ | ⨁⨁◯◯  LOW | 63 | 155 | - | The mean weight gain in abstinent smokers ranged from 1.69-5.5 kg | MD 0.87 kg lower (2.21 lower to 0.47 higher) |
| **Weight gain in abstinent smokers (follow up: 12 months)** ^q,r,s^  Mean (SD) weight change (kg) from baseline assessed in abstainers (continuous/sustained 50%, prolonged 25%, point prevalence 25% studies)  Smoking abstinence biochemically validated in all trials | | | | | | | | | | | |
| 252 (4 RCTs) | serious ^t^ | not serious ^u^ | not serious ^v^ | serious ^w^ | none ^p^ | ⨁⨁◯◯  LOW | 82 | 170 | - | The mean weight gain in abstinent smokers ranged from 2.94-6.9 kg | MD 0.38 kg lower (2 lower to 1.24 higher) |

**CBT:** Cognitive behavioural therapy**; CI:** Confidence interval; **MD:** Mean difference

#### Explanations

a. Review authors state that all trials included in the review examined smokers motivated to quit. However, motivation to quit was not explicitly reported in review evidence tables for any of the trials included in this analysis. Five trials recruited general smokers, one trial hospital inpatients with cardiovascular disease, and one trial healthcare professionals.

b. Bupropion: 300 mg/day for 7 to 12 weeks. Behavioural co-intervention provided in all trials. In two 3-arm trials, participants assigned to the bupropion arm also received placebo to control for the effect of the other tested intervention. 5.1% of participants received a specialized behavioural counselling co-intervention (i.e., cognitive behavioural therapy).

c. Placebo: Review authors indicate the comparator is placebo; however, in one trial, control is advice with follow-up rather than placebo. Behavioural co-intervention provided in all trials. In two 3-arm trials, participants assigned to placebo bupropion also received additional placebo (placebo gum in one trial and placebo varenicline in another). 9.5% of participants received a specialized behavioural counselling co-intervention (i.e., cognitive behavioural therapy).

d. 74% of the evidence is at unclear risk of bias for two domains among selection, attrition, and abstinence definition bias; we downrate this domain by -1.0. Evidence at high risk of bias (7%) is not substantive enough for further downrating.

e. Little variation in point estimates and confidence intervals overlap (I2=0%, p=0.78). We do not downrate this domain.

f. 3% of evidence is indirect (i.e., inpatient setting). Due to reporting, directness could not be ascertained for one study contributing 59% of weight to the meta-analysis (recruited healthcare professionals but unclear if conducted in workplace setting and unclear countries of conduct). We do not downrate this domain.

g. Confidence interval encompasses little to no difference. The sample size is adequate (>400 participants for continuous outcome). We did not downrate this domain.

h. Although search not completely comprehensive and largest trial favours bupropion, there is not enough evidence that publication bias has been detected. We do not downrate this domain.

i. Bupropion: 300 mg/day for 7 weeks. Behavioural co-intervention provided in all trials. 3.9% of participants received a specialized behavioural counselling co-intervention (i.e., cognitive behavioural therapy).

j. Placebo: Review authors indicate the comparator is placebo; however, in one trial, control is advice with follow-up rather than placebo. Behavioural co-intervention provided in all trials. 20.6% of participants received a specialized behavioural counselling co-intervention (i.e., cognitive behavioural therapy).

k. Review authors state that all trials included in the review examined smokers motivated to quit. However, motivation to quit was not explicitly reported in review evidence tables for any of the trials included in this analysis. Two trials recruited general smokers, one trial inpatients, and one trial healthcare professionals.

l. 99% of the evidence is at unclear risk of bias for two or more domains, namely, selection, performance/detection, and attrition bias. There is no evidence at high risk of bias. We downrate this domain by -1.0.

m. Little variation in point estimates and confidence intervals overlap (I2=0%, p=0.92). We do not downrate this domain.

n. <1% of the evidence is indirect (inpatient setting). One study was conducted in a ‘high’ HDI country but contributes only 28% of weight to the meta-analysis. Due to reporting, directness could not be ascertained for one study contributing 61% of weight to the meta-analysis (recruited healthcare professionals but unclear if conducted in workplace setting and unclear countries of conduct). We do not downrate this domain.

o. Confidence interval encompasses little to no difference. The sample size is inadequate (total of 218 participants). We downrate this domain by -1.0.

p. Although search not completely comprehensive, all studies report negative findings. We do not downrate this domain.

q. Bupropion: 300 mg/day for 7 to 12 weeks. Behavioural co-intervention provided in all trials. 21.8% of participants received specialized behavioral counselling interventions (CBT with or without relapse prevention counselling).

r. Placebo: Behavioural co-intervention provided in all trials. 46.3% of participants received specialized behavioral counselling interventions (CBT with or without relapse prevention counselling).

s. Review authors state that all trials included in the review examined smokers motivated to quit. However, motivation to quit was not explicitly reported in review evidence tables for any of the trials included in this analysis. Two trials recruited general smokers, one trial hospital patients with cardiovascular disease, and one trial healthcare professionals.

t. 66% of the evidence is at unclear risk of bias for two domains, namely, selection and attrition bias; we downrate this domain by -1.0. Evidence at high risk of bias (13.2%) is not substantive enough for further downrating.

u. Little variation in point estimates and confidence intervals overlap. I2=0%, p=0.98. We do not downrate this domain.

v. 13% of the evidence is indirect (i.e., inpatient setting). Due to reporting, the proportion of directness could not be ascertained for one study contributing 57% of weight to the meta-analysis (recruited healthcare professionals but unclear if conducted in workplace setting and unclear countries of conduct). We do not downrate this domain.

w. Confidence interval encompasses little to no difference. The sample size is inadequate (total of 252 participants). We downrate this domain by -1.0.

| Summary of findings: | | | | | | |
| --- | --- | --- | --- | --- | --- | --- |
| **Bupropion (300 mg/day) compared to placebo in smokers motivated/wishing to quit at baseline and abstinent at follow-up** | | | | | | |
| **Patient or population**: smokers motivated/wishing to quit at baseline and abstinent at follow-up  **Setting**: No restriction  **Intervention**: Bupropion (300 mg/day)  **Comparison**: placebo | | | | | | |
| Outcomes | **Anticipated absolute effects^*^** (95% CI) | | Relative effect (95% CI) | № of participants  (studies) | Certainty of the evidence (GRADE) | Comments |
|  | **Risk with placebo** | **Risk with Bupropion (300 mg/day)** |  |  |  |  |
| Weight gain in abstinent smokers ^a,b,c^  Mean (SD) weight change (kg) from baseline assessed in abstainers (continuous/sustained 28.5%, prolonged 42.9%, point prevalence 28.5% studies)  Smoking abstinence biochemically validated in all trials  Follow up: End of treatment | The mean weight gain in abstinent smokers ranged from 2.32-4.0 kg | MD 1.12 kg lower (1.47 lower to 0.77 lower) | - | 869 (7 RCTs) | ⨁⨁⨁◯ MODERATE ^d,e,f,g,h^ | Fixed effects analysis.  AMSTAR-2: Critically low.  Date of last search: September 2011.  Not GRADEd by review authors. |
| Weight gain in abstinent smokers ^i,j,k^  Mean (SD) weight change (kg) from baseline assessed in abstainers (continuous/sustained 75%, prolonged 25% studies)  Smoking abstinence biochemically validated in all trials.  Follow up: 6 months | The mean weight gain in abstinent smokers ranged from 1.69-5.5 kg | MD 0.87 kg lower (2.21 lower to 0.47 higher) | - | 218 (4 RCTs) | ⨁⨁◯◯  LOW ^l,m,n,o,p^ | Fixed effects analysis.  AMSTAR-2: Critically low.  Date of last search: September 2011.  Not GRADEd by review authors. |
| Weight gain in abstinent smokers ^q,r,s^  Mean (SD) weight change (kg) from baseline assessed in abstainers (continuous/sustained 50%, prolonged 25%, point prevalence 25% studies)  Smoking abstinence biochemically validated in all trials.  Follow up: 12 months | The mean weight gain in abstinent smokers ranged from 2.94-6.9 kg | MD 0.38 kg lower (2 lower to 1.24 higher) | - | 252 (4 RCTs) | ⨁⨁◯◯  LOW ^p,t,u,v,w^ | Fixed effects analysis.  AMSTAR-2: Critically low.  Date of last search: September 2011.  Not GRADEd by review authors. |
| ***The risk in the intervention group** (and its 95% confidence interval) is based on the assumed risk in the comparison group and the **relative effect** of the intervention (and its 95% CI).   **CBT:** Cognitive behavioural therapy**; CI:** Confidence interval; **MD:** Mean difference | | | | | | |
| **GRADE Working Group grades of evidence** **High certainty:** We are very confident that the true effect lies close to that of the estimate of the effect **Moderate certainty:** We are moderately confident in the effect estimate: The true effect is likely to be close to the estimate of the effect, but there is a possibility that it is substantially different **Low certainty:** Our confidence in the effect estimate is limited: The true effect may be substantially different from the estimate of the effect **Very low certainty:** We have very little confidence in the effect estimate: The true effect is likely to be substantially different from the estimate of effect | | | | | | |

#### Explanations

a. Review authors state that all trials included in the review examined smokers motivated to quit. However, motivation to quit was not explicitly reported in review evidence tables for any of the trials included in this analysis. Five trials recruited general smokers, one trial hospital inpatients with cardiovascular disease, and one trial healthcare professionals.

b. Bupropion: 300 mg/day for 7 to 12 weeks. Behavioural co-intervention provided in all trials. In two 3-arm trials, participants assigned to the bupropion arm also received placebo to control for the effect of the other tested intervention. 5.1% of participants received a specialized behavioural counselling co-intervention (i.e., cognitive behavioural therapy).

c. Placebo: Review authors indicate the comparator is placebo; however, in one trial, control is advice with follow-up rather than placebo. Behavioural co-intervention provided in all trials. In two 3-arm trials, participants assigned to placebo bupropion also received additional placebo (placebo gum in one trial and placebo varenicline in another). 9.5% of participants received a specialized behavioural counselling co-intervention (i.e., cognitive behavioural therapy).

d. 74% of the evidence is at unclear risk of bias for two domains among selection, attrition, and abstinence definition bias; we downrate this domain by -1.0. Evidence at high risk of bias (7%) is not substantive enough for further downrating.

e. Little variation in point estimates and confidence intervals overlap (I2=0%, p=0.78). We do not downrate this domain.

f. 3% of evidence is indirect (i.e., inpatient setting). Due to reporting, directness could not be ascertained for one study contributing 59% of weight to the meta-analysis (recruited healthcare professionals but unclear if conducted in workplace setting and unclear countries of conduct). We do not downrate this domain.

g. Confidence interval encompasses little to no difference. The sample size is adequate (>400 participants for continuous outcome). We did not downrate this domain.

h. Although search not completely comprehensive and largest trial favours bupropion, there is not enough evidence that publication bias has been detected. We do not downrate this domain.

i. Bupropion: 300 mg/day for 7 weeks. Behavioural co-intervention provided in all trials. 3.9% of participants received a specialized behavioural counselling co-intervention (i.e., cognitive behavioural therapy).

j. Placebo: Review authors indicate the comparator is placebo; however, in one trial, control is advice with follow-up rather than placebo. Behavioural co-intervention provided in all trials. 20.6% of participants received a specialized behavioural counselling co-intervention (i.e., cognitive behavioural therapy).

k. Review authors state that all trials included in the review examined smokers motivated to quit. However, motivation to quit was not explicitly reported in review evidence tables for any of the trials included in this analysis. Two trials recruited general smokers, one trial inpatients, and one trial healthcare professionals.

l. 99% of the evidence is at unclear risk of bias for two or more domains, namely, selection, performance/detection, and attrition bias. There is no evidence at high risk of bias. We downrate this domain by -1.0.

m. Little variation in point estimates and confidence intervals overlap (I2=0%, p=0.92). We do not downrate this domain.

n. <1% of the evidence is indirect (inpatient setting). One study was conducted in a ‘high’ HDI country but contributes only 28% of weight to the meta-analysis. Due to reporting, directness could not be ascertained for one study contributing 61% of weight to the meta-analysis (recruited healthcare professionals but unclear if conducted in workplace setting and unclear countries of conduct). We do not downrate this domain.

o. Confidence interval encompasses little to no difference. The sample size is inadequate (total of 218 participants). We downrate this domain by -1.0.

p. Although search not completely comprehensive, all studies report negative findings. We do not downrate this domain.

q. Bupropion: 300 mg/day for 7 to 12 weeks. Behavioural co-intervention provided in all trials. 21.8% of participants received specialized behavioral counselling interventions (CBT with or without relapse prevention counselling).

r. Placebo: Behavioural co-intervention provided in all trials. 46.3% of participants received specialized behavioral counselling interventions (CBT with or without relapse prevention counselling).

s. Review authors state that all trials included in the review examined smokers motivated to quit. However, motivation to quit was not explicitly reported in review evidence tables for any of the trials included in this analysis. Two trials recruited general smokers, one trial hospital patients with cardiovascular disease, and one trial healthcare professionals.

t. 66% of the evidence is at unclear risk of bias for two domains, namely, selection and attrition bias; we downrate this domain by -1.0. Evidence at high risk of bias (13.2%) is not substantive enough for further downrating.

u. Little variation in point estimates and confidence intervals overlap. I2=0%, p=0.98. We do not downrate this domain.

v. 13% of the evidence is indirect (i.e., inpatient setting). Due to reporting, the proportion of directness could not be ascertained for one study contributing 57% of weight to the meta-analysis (recruited healthcare professionals but unclear if conducted in workplace setting and unclear countries of conduct). We do not downrate this domain.

w. Confidence interval encompasses little to no difference. The sample size is inadequate (total of 252 participants). We downrate this domain by -1.0.

## Appendix K Table 19. NRT versus Placebo: Weight gain in smokers motivated to quit at baseline and abstinent at follow-up

| Any type of NRT compared to placebo in smokers motivated/wishing to quit at baseline and abstinent at follow-up  **Bibliography:** Farley 2012; Date of last search: September 2011 | | | | | | | | | | | |
| --- | --- | --- | --- | --- | --- | --- | --- | --- | --- | --- | --- |
| **Certainty assessment** | | | | | | | **Summary of findings** | | | | |
| **№ of participants (studies) Follow-up** | **Risk of bias** | **Inconsistency** | **Indirectness** | **Imprecision** | **Publication bias** | **Overall certainty of evidence** | **Study event rates (%)** | | **Relative effect (95% CI)** | **Anticipated absolute effects** | |
|  |  |  |  |  |  |  | **With placebo** | **With Any type of NRT** |  | **Risk with placebo** | **Risk difference with Any type of NRT** |
| **Weight gain in abstinent smokers (follow up: End of treatment)** ^a,b,c^  Mean (SD) weight change (kg) assessed in abstainers (continuous/sustained 26%, prolonged 52.6%, point prevalence 15.8%, NR 5.3% studies)  Smoking abstinence biochemically validated in at least 94.7% of trials (NR for one trial) | | | | | | | | | | | |
| 2600 (19 RCTs) | serious ^d^ | very serious ^e^ | not serious ^f^ | not serious ^g^ | none ^h^ | ⨁◯◯◯  VERY LOW | 686 | 1914 | - | The mean weight gain in abstinent smokers was 1.1-5.6 kg | MD 0.69 kg lower (0.88 lower to 0.51 lower) |
| **Weight gain in abstinent smokers (follow up: 6 months)** ^i,j,k^  Mean (SD) weight change (kg) assessed in abstainers (continuous/sustained 33.3%, prolonged 66.7% studies)  Smoking abstinence biochemically validated in all trials | | | | | | | | | | | |
| 771 (9 RCTs) | serious ^l^ | not serious ^m^ | not serious ^n^ | not serious ^o^ | none ^p^ | ⨁⨁⨁◯  MODERATE | 275 | 496 | - | The mean weight gain in abstinent smokers was 2.58-5.8 kg | MD 0.37 kg lower (0.88 lower to 0.14 higher) |
| **Weight gain in abstinent smokers (follow up: 12 months)** ^q,r,s^  Mean (SD) weight change (kg) assessed in abstainers (continuous/sustained 26.7%, prolonged 73.3% studies)  Smoking abstinence biochemically validated in all trials | | | | | | | | | | | |
| 1334 (15 RCTs) | serious ^t^ | not serious ^u^ | not serious ^v^ | not serious ^w^ | none ^h^ | ⨁⨁⨁◯  MODERATE | 364 | 970 | - | The mean weight gain in abstinent smokers ranged from 3.0-8.3 kg | MD 0.42 kg lower (0.92 lower to 0.08 higher) |

**CI:** Confidence interval; **NR**: Not reported; **MD:** Mean difference

#### Explanations

a. Review authors state that all trials included in the review examined smokers motivated to quit. However, motivation to quit was not explicitly reported in review evidence tables for any of the trials included in this analysis. Fourteen trials recruited general smokers, two trials females only, one trial smokers who had relapsed in an earlier phase of the study, one trial low dependence smokers, and one trial high dependence smokers.

b. NRT: Gum (n=4), patch (n=10), inhaler (n=2), sublingual tablet (n=2), intranasal spray (n=1). Patch: Variation in dose across trials but most studies provided participants with either lower (e.g., 14 or 15 mg) and/or higher dose (e.g., 21/22 or 25 mg) based on dependence or preference. Gum: Dosing varied across trials including 2 mg ad libitum (1 trial), 10 to 12 pieces daily (1 trial), 2 mg followed by randomization to 7, 15, or 30 pieces daily (1 trial), and 2 mg 9-15 pieces or 4 mg 9-15 pieces (1 trial). Treatment duration for gum was from 8 weeks to 1 year (median = 12 weeks). Inhaler: Up to 6-month use of 2-10/day in one trial and minimum 4/day in the other trial. Sublingual tablet: Up to 24 weeks of 2 mg in one trial and 4 mg in the second trial. Intranasal spray: 0.5mg/dose for up to 1 year. Behavioural co-intervention provided in most trials. One trial examining NRT intranasal spray also provided all participants with NRT patch. 1.3% of participants received specialized behavioural counselling (CBT).

c. Placebo: Comparator is reported as ‘placebo’ by review authors; however, the control condition is group therapy or described as 'no gum' in one trial each. Behavioural co-intervention provided in most trials. One trial examining NRT intranasal spray provided both arms with NRT patch. 3.2% of participants received specialized behavioural counselling (CBT).

d. About 80% of evidence at unclear risk for selection and attrition biases. Minority of other bias issues. We downrate this domain by -1.0. Evidence at high risk of bias (5%) is not substantive enough for further downrating.

e. Statistical heterogeneity attributed to one trial reporting 4.3 kg difference between groups (I2=82%, p=<0.00001). Otherwise, risk estimates mostly consistent and confidence intervals overlapping. We downrate this domain by -2.0.

f. 3% of evidence is indirect (university setting). Due to reporting, directness could not be ascertained for one study contributing 20.5% of weight to the meta-analysis. It was conducted in 17 unspecified European countries. We do not downrate this domain.

g. Confidence interval encompasses little to no difference. The optimal information size is met (total of 2600 participants). We do not downrate this domain.

h. Although search not completely comprehensive, a mix of results observed. We do not downrate this domain.

i. NRT: Gum (n=2), patch (n=4), inhaler (n=1), sublingual tablets (n=2). Behavioural co-intervention provided in most trials. One trial examining NRT patch also provided all participants with NRT inhaler in addition to behavioural support. One trial examining NRT patch also provided all participants with NRT gum.

j. Placebo: Comparator is reported as ‘placebo’ by review authors; however, control condition is group therapy in one trial. Behavioural co-intervention provided in most trials. One trial examining NRT patch provided all participants (including controls) with NRT inhaler in addition to behavioural support. One trial examining NRT patch provided all participants (including controls) with NRT gum.

k. Review authors state that all trials included in the review examined smokers motivated to quit. However, motivation to quit was not explicitly reported in review evidence tables for any of the trials included in this analysis. Six trials recruited general smokers, one trial females only, one trial high dependency smokers, and one trial low dependency smokers.

l. 81% of the evidence is at unclear risk of bias for mainly selection and attrition bias. There is no evidence at high risk of bias. We downrate this domain by -1.0.

m. Little variation in point estimates and confidence intervals overlap. I2=0%, p=0.81. We do not downrate this domain.

n. No indirectness.

o. Confidence interval encompasses little to no difference. The optimal information size is met (total of 771 participants). We do not downrate this domain.

p. Although search not completely comprehensive, all studies report negative findings. We do not downrate this domain.

q. NRT: Gum (n=1), patch (n=6), intranasal spray (n=3), inhaler (n=2), sublingual tablet (n=3). Behavioural co-intervention provided in most trials. One trial examining NRT patch also provided all participants with NRT inhaler in addition to behavioural support. Two trials examining either NRT intranasal spray or NRT patch also provided all participants with a second type of NRT (i.e., NRT patch or NRT gum, respectively).

r. Placebo: Comparator is reported as 'placebo' by review authors; however, in one trial, control condition is group therapy. Behavioural co-intervention provided in most trials. One trial examining NRT patch provided all participants (including controls) with NRT inhaler in addition to behavioural support. Two trials examining either NRT intranasal spray or NRT patch provided all participants (including controls) with NRT patch or NRT gum, respectively.

s. Review authors state that all trials included in the review examined smokers motivated to quit. However, motivation to quit was not explicitly reported in review evidence tables for any of the trials included in this analysis. Twelve trials recruited general smokers, one trial females only, one trial high dependency smokers, and one trial low dependency smokers.

t. 55% of the evidence is at unclear risk of bias mainly for selection and attrition bias. There is no evidence at high risk of bias. We downrate this domain by -1.0.

u. Little variation in point estimates and confidence intervals overlap. I2=0%, p=0.85. We do not downrate this domain.

v. Due to reporting, directness could not be ascertained for one study contributing 27% of weight to the meta-analysis (conducted in 17 unspecified European countries). No indirectness for remaining trials. We do not downrate this domain.

w. Confidence interval encompasses little to no difference. The optimal information size is met (total of 1334 participants). We do not downrate this domain.

| Summary of findings: | | | | | | |
| --- | --- | --- | --- | --- | --- | --- |
| **Any type of NRT compared to placebo in smokers motivated/wishing to quit at baseline and abstinent at follow-up** | | | | | | |
| **Patient or population**: Smokers motivated/wishing to quit at baseline and abstinent at follow-up  **Setting**: No restriction  **Intervention**: Any type of NRT  **Comparison**: Placebo | | | | | | |
| Outcomes | **Anticipated absolute effects^*^** (95% CI) | | Relative effect (95% CI) | № of participants  (studies) | Certainty of the evidence (GRADE) | Comments |
|  | **Risk with placebo** | **Risk with Any type of NRT** |  |  |  |  |
| Weight gain in abstinent smokers ^a,b,c^  Mean (SD) weight change (kg) assessed in abstainers (continuous/sustained 26%, prolonged 52.6%, point prevalence 15.8%, NR 5.3% studies)  Smoking abstinence biochemically validated in at least 94.7% of trials (NR for one trial)  Follow up: End of treatment | The mean weight gain in abstinent smokers was 1.1-5.6 kg | MD 0.69 kg lower (0.88 lower to 0.51 lower) | - | 2600 (19 RCTs) | ⨁◯◯◯  VERY LOW ^d,e,f,g,h^ | Statistical heterogeneity attributed to one trial reporting 4.3 kg difference between groups. Estimate decreased when trial excluded from analysis (MD: -0.46 kg, 95% CI: -0.66 to -0.27; I2=0%).  Fixed effects analysis.  AMSTAR-2: Critically low.  Date of last search: September 2011.  Not GRADEd by review authors. |
| Weight gain in abstinent smokers ^i,j,k^  Mean (SD) weight change (kg) assessed in abstainers (continuous/sustained 33.3%, prolonged 66.7% studies)  Smoking abstinence biochemically validated in all trials  Follow up: 6 months | The mean weight gain in abstinent smokers was 2.58-5.8 kg | MD 0.37 kg lower (0.88 lower to 0.14 higher) | - | 771 (9 RCTs) | ⨁⨁⨁◯  MODERATE ^l,m,n,o,p^ | Fixed effects analysis.  AMSTAR-2: Critically low.  Date of last search: September 2011.  Not GRADEd by review authors. |
| Weight gain in abstinent smokers ^q,r,s^  Mean (SD) weight change (kg) assessed in abstainers (continuous/sustained 26.7%, prolonged 73.3% studies)  Smoking abstinence biochemically validated in all trials  Follow up: 12 months | The mean weight gain in abstinent smokers ranged from 3.0-8.3 kg | MD 0.42 kg lower (0.92 lower to 0.08 higher) | - | 1334 (15 RCTs) | ⨁⨁⨁◯  MODERATE ^h,t,u,v,w^ | Fixed effects analysis.  AMSTAR-2: Critically low.  Date of last search: September 2011.  Not GRADEd by review authors. |
| ***The risk in the intervention group** (and its 95% confidence interval) is based on the assumed risk in the comparison group and the **relative effect** of the intervention (and its 95% CI).   **CBT:** Cognitive behavioural therapy**; CI:** Confidence interval; **NR:** Not reported; **NRT:** Nicotine replacement therapy; **MD:** Mean difference | | | | | | |
| **GRADE Working Group grades of evidence** **High certainty:** We are very confident that the true effect lies close to that of the estimate of the effect **Moderate certainty:** We are moderately confident in the effect estimate: The true effect is likely to be close to the estimate of the effect, but there is a possibility that it is substantially different **Low certainty:** Our confidence in the effect estimate is limited: The true effect may be substantially different from the estimate of the effect **Very low certainty:** We have very little confidence in the effect estimate: The true effect is likely to be substantially different from the estimate of effect | | | | | | |

#### Explanations

a. Review authors state that all trials included in the review examined smokers motivated to quit. However, motivation to quit was not explicitly reported in review evidence tables for any of the trials included in this analysis. Fourteen trials recruited general smokers, two trials females only, one trial smokers who had relapsed in an earlier phase of the study, one trial low dependence smokers, and one trial high dependence smokers.

b. NRT: Gum (n=4), patch (n=10), inhaler (n=2), sublingual tablet (n=2), intranasal spray (n=1). Patch: Variation in dose across trials but most studies provided participants with either lower (e.g., 14 or 15 mg) and/or higher dose (e.g., 21/22 or 25 mg) based on dependence or preference. Gum: Dosing varied across trials including 2 mg ad libitum (1 trial), 10 to 12 pieces daily (1 trial), 2 mg followed by randomization to 7, 15, or 30 pieces daily (1 trial), and 2 mg 9-15 pieces or 4 mg 9-15 pieces (1 trial). Treatment duration was from 8 weeks to 1 year (median = 12 weeks). Inhaler: Up to 6-month use of 2-10/day in one trial and minimum 4/day in the other trial. Sublingual tablet: Up to 24 weeks of 2 mg in one trial and 4 mg in the second trial. Intranasal spray: 0.5mg/dose for up to 1 year. Behavioural co-intervention provided in most trials. One trial examining NRT intranasal spray also provided all participants with NRT patch. 1.3% of participants received specialized behavioural counselling (CBT).

c. Placebo: Comparator is reported as ‘placebo’ by review authors; however, the control condition is group therapy or described as 'no gum' in one trial each. Behavioural co-intervention provided in most trials. One trial examining NRT intranasal spray provided both arms with NRT patch. 3.2% of participants received specialized behavioural counselling (CBT).

d. About 80% of evidence at unclear risk for selection and attrition biases. Minority of other bias issues. We downrate this domain by -1.0. Evidence at high risk of bias (5%) is not substantive enough for further downrating.

e. Statistical heterogeneity attributed to one trial reporting 4.3 kg difference between groups (I2=82%, p=<0.00001). Otherwise, risk estimates mostly consistent and confidence intervals overlapping. We downrate this domain by -2.0.

f. 3% of evidence is indirect (university setting). Due to reporting, directness could not be ascertained for one study contributing 20.5% of weight to the meta-analysis. It was conducted in 17 unspecified European countries. We do not downrate this domain.

g. Confidence interval encompasses little to no difference. The optimal information size is met (total of 2600 participants). We do not downrate this domain.

h. Although search not completely comprehensive, a mix of results observed. We do not downrate this domain.

i. NRT: Gum (n=2), patch (n=4), inhaler (n=1), sublingual tablets (n=2). Behavioural co-intervention provided in most trials. One trial examining NRT patch also provided all participants with NRT inhaler in addition to behavioural support. One trial examining NRT patch also provided all participants with NRT gum.

j. Placebo: Comparator is reported as ‘placebo’ by review authors; however, control condition is group therapy in one trial. Behavioural co-intervention provided in most trials. One trial examining NRT patch provided all participants (including controls) with NRT inhaler in addition to behavioural support. One trial examining NRT patch provided all participants (including controls) with NRT gum.

k. Review authors state that all trials included in the review examined smokers motivated to quit. However, motivation to quit was not explicitly reported in review evidence tables for any of the trials included in this analysis. Six trials recruited general smokers, one trial females only, one trial high dependency smokers, and one trial low dependency smokers.

l. 81% of the evidence is at unclear risk of bias for mainly selection and attrition bias. There is no evidence at high risk of bias. We downrate this domain by -1.0.

m. Little variation in point estimates and confidence intervals overlap. I2=0%, p=0.81. We do not downrate this domain.

n. No indirectness.

o. Confidence interval encompasses little to no difference. The optimal information size is met (total of 771 participants). We do not downrate this domain.

p. Although search not completely comprehensive, all studies report negative findings. We do not downrate this domain.

q. NRT: Gum (n=1), patch (n=6), intranasal spray (n=3), inhaler (n=2), sublingual tablet (n=3). Behavioural co-intervention provided in most trials. One trial examining NRT patch also provided all participants with NRT inhaler in addition to behavioural support. Two trials examining either NRT intranasal spray or NRT patch also provided all participants with a second type of NRT (i.e., NRT patch or NRT gum, respectively).

r. Placebo: Comparator is reported as 'placebo' by review authors; however, in one trial, control condition is group therapy. Behavioural co-intervention provided in most trials. One trial examining NRT patch provided all participants (including controls) with NRT inhaler in addition to behavioural support. Two trials examining either NRT intranasal spray or NRT patch provided all participants (including controls) with NRT patch or NRT gum, respectively.

s. Review authors state that all trials included in the review examined smokers motivated to quit. However, motivation to quit was not explicitly reported in review evidence tables for any of the trials included in this analysis. Twelve trials recruited general smokers, one trial females only, one trial high dependency smokers, and one trial low dependency smokers.

t. 55% of the evidence is at unclear risk of bias mainly for selection and attrition bias. There is no evidence at high risk of bias. We downrate this domain by -1.0.

u. Little variation in point estimates and confidence intervals overlap. I2=0%, p=0.85. We do not downrate this domain.

v. Due to reporting, directness could not be ascertained for one study contributing 27% of weight to the meta-analysis (conducted in 17 unspecified European countries). No indirectness for remaining trials. We do not downrate this domain.

w. Confidence interval encompasses little to no difference. The optimal information size is met (total of 1334 participants). We do not downrate this domain.

## Appendix K Table 20. Varenicline (2mg/day) versus Placebo: Weight gain in smokers motivated to quit at baseline and abstinent at follow-up

| Varenicline (2 mg/day) compared to placebo in smokers motivated/wishing to quit at baseline and abstinent at follow-up  **Bibliography:** Farley 2012; Date of last search: September 2011. | | | | | | | | | | | |
| --- | --- | --- | --- | --- | --- | --- | --- | --- | --- | --- | --- |
| **Certainty assessment** | | | | | | | **Summary of findings** | | | | |
| **№ of participants (studies) Follow-up** | **Risk of bias** | **Inconsistency** | **Indirectness** | **Imprecision** | **Publication bias** | **Overall certainty of evidence** | **Study event rates (%)** | | **Relative effect (95% CI)** | **Anticipated absolute effects** | |
|  |  |  |  |  |  |  | **With placebo** | **With Varenicline (2 mg/day)** |  | **Risk with placebo** | **Risk difference with Varenicline (2 mg/day)** |
| **Weight gain in abstinent smokers (follow up: End of treatment)** ^a,b,c^  Mean (SD) weight change (kg) assessed in abstainers (continuous/sustained 63.6%, prolonged 36.4% studies)  Smoking abstinence biochemically validated in all trials | | | | | | | | | | | |
| 2008 (11 RCTs) | serious ^d^ | not serious ^e^ | not serious ^f^ | not serious ^g^ | none ^h^ | ⨁⨁⨁◯  MODERATE | 666 | 1342 | - | The mean weight gain in abstinent smokers ranged from 1.38-3.80 kg | MD 0.41 kg lower (0.63 lower to 0.19 lower) |
| **Weight gain in abstinent smokers (follow up: 6 months)** ^i,j^  Mean (SD) weight change (kg) assessed in abstainers (continuous/sustained 100% studies)  Smoking abstinence biochemically validated in the only trial included in this analysis | | | | | | | | | | | |
| 105 (1 RCT) | serious ^k^ | not serious ^l^ | not serious ^m^ | serious ^n^ | none ^o^ | ⨁⨁◯◯  LOW | 42 | 63 | - | The mean weight gain in abstinent smokers was 1.66 kg | MD 0.41 kg higher (0.79 lower to 1.61 higher) |
| **Weight gain in abstinent smokers (follow up: 12 months)** ^p,q^  Mean (SD) weight change (kg) assessed in abstainers (continuous/sustained 100% studies)  Smoking abstinence biochemically validated in all trials | | | | | | | | | | | |
| 151 (2 RCTs) | not serious ^r^ | not serious ^s^ | not serious ^t^ | serious ^u^ | none ^v^ | ⨁⨁⨁◯  MODERATE | 40 | 111 | - | The mean weight gain in abstinent smokers ranged from 3.9-5.2 kg | MD 1.11 kg higher (0.75 lower to 2.98 higher) |

**CI:** Confidence interval; **MD:** Mean difference

#### Explanations

a. Review authors state that all trials included in the review examined smokers motivated to quit. However, motivation to quit was explicitly reported in review evidence tables for only 27% of the trials included in this analysis. According to the information reported in the review evidence tables, seven trials recruited general smokers, one trial general smokers motivated to quit, one trial smokers with stable CVD who were motivated to quit, one trial smokers with COPD who were motivated to quit, and one trial successful quitters following open-label treatment with varenicline.

b. Varenicline: Treatment duration was 12 weeks in all but one trial (6 weeks). Behavioural co-intervention provided in most trials. In one 3-arm trial, participants assigned to the varenicline arm also received placebo to control for the effect of the other tested intervention.

c. Placebo: Behavioural co-intervention provided in most trials. In one 3-arm trial, participants assigned to placebo varenicline also received additional placebo (i.e., placebo bupropion) along with a behavioural support co-intervention.

d. 90% of the evidence is at unclear risk of bias for one domain, namely, attrition bias which was not evaluated by review authors. There is no evidence at high risk of bias and evidence at unclear risk for two domains (10%) is not substantive enough to be considered for downrating. We downrate this domain by -0.5 but also reflect the partial downrating (-0.5) from the inconsistency domain here.

e. Some variation in point estimates but confidence intervals mostly overlap (I2=42%, p=0.07). We downrate this domain by -0.5 but reflect this in the risk of bias domain.

f. Due to reporting, the proportion of indirect evidence could not be determined for two trials contributing a combined weight of 13% to the meta-analysis. One was conducted in 15 unspecified countries in Europe, Asia and the Americas. The second trial recruited from both high and very high ranking studies; however, proportion of participants recruited from each NR. No indirectness for remaining trials. We do not downrate this domain.

g. Confidence interval encompasses little to no difference. The optimal information size is met (>2000 participants). We do not downrate this domain.

h. Although search not completely comprehensive, a mix of results observed across studies. We do not downrate this domain.

i. Review authors state that all trials included in the review examined smokers motivated to quit. However, motivation to quit was not explicitly reported in the review evidence table for the only trial included in this analysis. According to the review evidence table, the trial recruited general smokers.

j. Treatment duration was 12 weeks. Behavioural co-intervention provided to both varenicline and placebo participants.

k. The only trial included in this analysis is at unclear risk of selection and attrition bias. We downrate this domain by -1.0.

l. Not applicable - one trial.

m. The only trial in this analysis recruited from both high and very high ranking studies; however, proportion of participants recruited from each NR. We do not downrate this domain.

n. Confidence interval encompasses little to no difference. The optimal information size is not met (<400 participants). We downrate this domain by -1.0.

o. Search not completely comprehensive but the only study included in this analysis reports negative findings. We do not downrate this domain.

p. Both trials in the analysis recruited smokers motivated to quit. One of the trials recruited participants with COPD.

q. Treatment duration was 12 weeks in both studies. Behavioural co-intervention provided to both arms in all trials.

r. 77% of the evidence at unclear risk of bias for one domain (i.e., attrition bias); we downrate this domain by -0.5. Evidence at unclear risk of bias for two domains (23%) is not substantive enough for further downrating.

s. Little variation in point estimates and confidence intervals overlap (I2=0%, p=0.72). We do not downrate this domain.

t. Due to reporting, directness could not be ascertained for one study contributing 76.8% of weight to the meta-analysis. It was conducted in 15 unspecified countries in Europe, Asia and the Americas. Remaining trial is not indirect. We do not downrate this domain.

u. Confidence interval encompasses little to no difference. The optimal information size is not met (<400 participants). We downrate this domain by -1.0. v. Although search is not completely comprehensive, both trials report negative findings. We do not downrate this domain.

| Summary of findings: | | | | | | |
| --- | --- | --- | --- | --- | --- | --- |
| **Varenicline (2 mg/day) compared to placebo in smokers motivated/wishing to quit at baseline and abstinent at follow-up** | | | | | | |
| **Patient or population**: Smokers motivated/wishing to quit at baseline and abstinent at follow-up  **Setting**: No restriction  **Intervention**: Varenicline (2 mg/day)  **Comparison**: Placebo | | | | | | |
| Outcomes | **Anticipated absolute effects^*^** (95% CI) | | Relative effect (95% CI) | № of participants  (studies) | Certainty of the evidence (GRADE) | Comments |
|  | **Risk with placebo** | **Risk with Varenicline (2 mg/day)** |  |  |  |  |
| Weight gain in abstinent smokers ^a,b,c^  Mean (SD) weight change (kg) assessed in abstainers (continuous/sustained 63.6%, prolonged 36.4% studies)  Smoking abstinence biochemically validated in all trials  Follow up: End of treatment | The mean weight gain in abstinent smokers ranged from 1.38-3.80 kg | MD 0.41 kg lower (0.63 lower to 0.19 lower) | - | 2008 (11 RCTs) | ⨁⨁⨁◯  MODERATE ^d,e,f,g,h^ | Fixed effects analysis.  AMSTAR-2: Critically low.  Date of last search: September 2011.  Not GRADEd by review authors. |
| Weight gain in abstinent smokers ^,I,j^  Mean (SD) weight change (kg) assessed in abstainers (continuous/sustained 100% studies)  Smoking abstinence biochemically validated in the only trial included in this analysis  Follow up: 6 months | The mean weight gain in abstinent smokers was 1.66 kg | MD 0.41 kg higher (0.79 lower to 1.61 higher) | - | 105 (1 RCT) | ⨁⨁◯◯  LOW ^k,l,m,n,o^ | AMSTAR-2: Critically low.  Date of last search: September 2011.  Not GRADEd by review authors. |
| Weight gain in abstinent smokers ^p,q^  Mean (SD) weight change (kg) assessed in abstainers (continuous/sustained 100% studies)  Smoking abstinence biochemically validated in all trials.  Follow up: 12 months | The mean weight gain in abstinent smokers ranged from 3.9-5.2 kg | MD 1.11 kg higher (0.75 lower to 2.98 higher) | - | 151 (2 RCTs) | ⨁⨁⨁◯  MODERATE ^r,s,t,u,v^ | Fixed effects analysis.  AMSTAR-2: Critically low.  Date of last search: September 2011.  Not GRADEd by review authors. |
| ***The risk in the intervention group** (and its 95% confidence interval) is based on the assumed risk in the comparison group and the **relative effect** of the intervention (and its 95% CI).   **CI:** Confidence interval; **MD:** Mean difference | | | | | | |
| **GRADE Working Group grades of evidence** **High certainty:** We are very confident that the true effect lies close to that of the estimate of the effect **Moderate certainty:** We are moderately confident in the effect estimate: The true effect is likely to be close to the estimate of the effect, but there is a possibility that it is substantially different **Low certainty:** Our confidence in the effect estimate is limited: The true effect may be substantially different from the estimate of the effect **Very low certainty:** We have very little confidence in the effect estimate: The true effect is likely to be substantially different from the estimate of effect | | | | | | |

#### Explanations

a. Review authors state that all trials included in the review examined smokers motivated to quit. However, motivation to quit was explicitly reported in review evidence tables for only 27% of the trials included in this analysis. According to the information reported in the review evidence tables, seven trials recruited general smokers, one trial general smokers motivated to quit, one trial smokers with stable CVD who were motivated to quit, one trial smokers with COPD who were motivated to quit, and one trial successful quitters following open-label treatment with varenicline.

b. Varenicline: Treatment duration was 12 weeks in all but one trial (6 weeks). Behavioural co-intervention provided in most trials. In one 3-arm trial, participants assigned to the varenicline arm also received placebo to control for the effect of the other tested intervention.

c. Placebo: Behavioural co-intervention provided in most trials. In one 3-arm trial, participants assigned to placebo varenicline also received additional placebo (i.e., placebo bupropion) along with a behavioural support co-intervention.

d. 90% of the evidence is at unclear risk of bias for one domain, namely, attrition bias which was not evaluated by review authors. There is no evidence at high risk of bias and evidence at unclear risk for two domains (10%) is not substantive enough to be considered for downrating. We downrate this domain by -0.5 but also reflect the partial downrating (-0.5) from the inconsistency domain here.

e. Some variation in point estimates but confidence intervals mostly overlap (I2=42%, p=0.07). We downrate this domain by -0.5 but reflect this in the risk of bias domain.

f. Due to reporting, the proportion of indirect evidence could not be determined for two trials contributing a combined weight of 13% to the meta-analysis. One was conducted in 15 unspecified countries in Europe, Asia and the Americas. The second trial recruited from both high and very high ranking studies; however, proportion of participants recruited from each NR. No indirectness for remaining trials. We do not downrate this domain.

g. Confidence interval encompasses little to no difference. The optimal information size is met (>2000 participants). We do not downrate this domain.

h. Although search not completely comprehensive, a mix of results observed across studies. We do not downrate this domain.

i. Review authors state that all trials included in the review examined smokers motivated to quit. However, motivation to quit was not explicitly reported in the review evidence table for the only trial included in this analysis. According to the review evidence table, the trial recruited general smokers.

j. Treatment duration was 12 weeks. Behavioural co-intervention provided to both varenicline and placebo participants.

k. The only trial included in this analysis is at unclear risk of selection and attrition bias. We downrate this domain by -1.0.

l. Not applicable - one trial.

m. The only trial in this analysis recruited from both high and very high ranking studies; however, proportion of participants recruited from each NR. We do not downrate this domain.

n. Confidence interval encompasses little to no difference. The optimal information size is not met (<400 participants). We downrate this domain by -1.0.

o. Search not completely comprehensive but the only study included in this analysis reports negative findings. We do not downrate this domain.

p. Both trials in the analysis recruited smokers motivated to quit. One of the trials recruited participants with COPD.

q. Treatment duration was 12 weeks in both studies. Behavioural co-intervention provided to both arms in all trials.

r. 77% of the evidence at unclear risk of bias for one domain (i.e., attrition bias); we downrate this domain by -0.5. Evidence at unclear risk of bias for two domains (23%) is not substantive enough for further downrating.

s. Little variation in point estimates and confidence intervals overlap (I2=0%, p=0.72). We do not downrate this domain.

t. Due to reporting, directness could not be ascertained for one study contributing 76.8% of weight to the meta-analysis. It was conducted in 15 unspecified countries in Europe, Asia and the Americas. Remaining trial is not indirect. We do not downrate this domain.

u. Confidence interval encompasses little to no difference. The optimal information size is not met (<400 participants). We downrate this domain by -1.0.

v. Although search is not completely comprehensive, both trials report negative findings. We do not downrate this domain.

## Appendix K Table 21. Varenicline (1 mg/day) versus Placebo: Weight gain in smokers motivated to quit at baseline and abstinent at follow-up

| Varenicline (1 mg/day) compared to placebo in smokers motivated/wishing to quit at baseline and abstinent at follow-up  **Bibliography:** Farley 2012; Date of last search: September 2011 | | | | | | | | | | | |
| --- | --- | --- | --- | --- | --- | --- | --- | --- | --- | --- | --- |
| **Certainty assessment** | | | | | | | **Summary of findings** | | | | |
| **№ of participants (studies) Follow-up** | **Risk of bias** | **Inconsistency** | **Indirectness** | **Imprecision** | **Publication bias** | **Overall certainty of evidence** | **Study event rates (%)** | | **Relative effect (95% CI)** | **Anticipated absolute effects** | |
|  |  |  |  |  |  |  | **With placebo** | **With Varenicline (1 mg/day)** |  | **Risk with placebo** | **Risk difference with Varenicline (1 mg/day)** |
| **Weight gain in abstinent smokers (follow up: End of treatment)** ^a,b^  Mean (SD) weight change (kg) assessed in abstainers (continuous/sustained 66.7%, prolonged 33.3% studies)  Smoking abstinence biochemically validated in all trials | | | | | | | | | | | |
| 254 (3 RCTs) | not serious ^c^ | serious ^d^ | not serious ^e^ | serious ^f^ | none ^g^ | ⨁⨁◯◯  LOW | 75 | 179 | - | The mean weight gain in abstinent smokers ranged from 1.48-4.0 kg | MD 0.12 kg lower (0.68 lower to 0.43 higher) |

**CI:** Confidence interval; **MD:** Mean difference

#### Explanations

a. Treatment duration was 12 weeks in two trials and 6 weeks in one trial. Behavioural co-intervention provided to both arms in all trials

b. Review authors state that all trials included in the review examined smokers motivated to quit. However, motivation to quit was not explicitly reported in review evidence tables for any of the trials included in this analysis. According to the review evidence tables, all trials recruited general smokers.

c. Evidence is at unclear risk of bias for one domain, namely, attrition bias which was not assessed by review authors. There is no evidence at high risk of bias and 15% of evidence at additional risk for selection bias is not substantive enough to consider for downrating. We downrate this domain by -0.5.

d. Variation in point estimates and limited overlap in confidence intervals (I2=60%, p=0.08). We downrate this domain by -1.0.

e. No indirectness.

f. Confidence interval encompasses little to no difference. The optimal information size is not met (<2000 participants). We downrate this domain by -1.0.

g. Although search is not completely comprehensive, a mix of results observed across studies. We do not downrate this domain.

| Summary of findings: | | | | | | |
| --- | --- | --- | --- | --- | --- | --- |
| **Varenicline (1 mg/day) compared to placebo in smokers motivated/wishing to quit at baseline and abstinent at follow-up** | | | | | | |
| **Patient or population**: Smokers motivated/wishing to quit at baseline and abstinent at follow-up  **Setting**: No restriction  **Intervention**: Varenicline (1 mg/day)  **Comparison**: Placebo | | | | | | |
| Outcomes | **Anticipated absolute effects^*^** (95% CI) | | Relative effect (95% CI) | № of participants  (studies) | Certainty of the evidence (GRADE) | Comments |
|  | **Risk with placebo** | **Risk with Varenicline (1 mg/day)** |  |  |  |  |
| Weight gain in abstinent smokers ^a,b^  Mean (SD) weight change (kg) assessed in abstainers (continuous/sustained 66.7%, prolonged 33.3% studies)  Smoking abstinence biochemically validated in all trials  Follow up: End of treatment | The mean weight gain in abstinent smokers ranged from 1.48-4.0 kg | MD 0.12 kg lower (0.68 lower to 0.43 higher) | - | 254 (3 RCTs) | ⨁⨁◯◯  LOW ^c,d,e,f,g^ | Fixed effects analysis.  AMSTAR-2: Critically low.  Date of last search: September 2011.  Not GRADEd by review authors. |
| ***The risk in the intervention group** (and its 95% confidence interval) is based on the assumed risk in the comparison group and the **relative effect** of the intervention (and its 95% CI).   **CI:** Confidence interval; **MD:** Mean difference | | | | | | |
| **GRADE Working Group grades of evidence** **High certainty:** We are very confident that the true effect lies close to that of the estimate of the effect **Moderate certainty:** We are moderately confident in the effect estimate: The true effect is likely to be close to the estimate of the effect, but there is a possibility that it is substantially different **Low certainty:** Our confidence in the effect estimate is limited: The true effect may be substantially different from the estimate of the effect **Very low certainty:** We have very little confidence in the effect estimate: The true effect is likely to be substantially different from the estimate of effect | | | | | | |

#### Explanations

a. Treatment duration was 12 weeks in two trials and 6 weeks in one trial. Behavioural co-intervention provided to both arms in all trials

b. Review authors state that all trials included in the review examined smokers motivated to quit. However, motivation to quit was not explicitly reported in review evidence tables for any of the trials included in this analysis. According to the review evidence tables, all trials recruited general smokers.

c. Evidence is at unclear risk of bias for one domain, namely, attrition bias which was not assessed by review authors. There is no evidence at high risk of bias and 15% of evidence at additional risk for selection bias is not substantive enough to consider for downrating. We downrate this domain by -0.5.

d. Variation in point estimates and limited overlap in confidence intervals (I2=60%, p=0.08). We downrate this domain by -1.0.

e. No indirectness.

f. Confidence interval encompasses little to no difference. The optimal information size is not met (<2000 participants). We downrate this domain by -1.0.

g. Although search is not completely comprehensive, a mix of results observed across studies. We do not downrate this domain.

# Hartmann-Boyce 2018 {332}

## Appendix K Table 22. NRT patch vs Placebo: Smoking cessation in relapsed smokers who are motivated to quit

| NRT patch compared to placebo patch in relapsed smokers who are motivated to quit  **Bibliography:** Hartmann-Boyce 2018; Date of last search July 2017 | | | | | | | | | | | |
| --- | --- | --- | --- | --- | --- | --- | --- | --- | --- | --- | --- |
| **Certainty assessment** | | | | | | | **Summary of findings** | | | | |
| **№ of participants (studies) Follow-up** | **Risk of bias** | **Inconsistency** | **Indirectness** | **Imprecision** | **Publication bias** | **Overall certainty of evidence** | **Study event rates (%)** | | **Relative effect (95% CI)** | **Anticipated absolute effects** | |
|  |  |  |  |  |  |  | **With Placebo patch** | **With Nicotine patch** |  | **Risk with Placebo patch** | **Risk difference with Nicotine patch** |
| **Abstinence/cessation (follow up: 6 months)** ^a,b,c^  Outcome measurement: continuous/sustained abstinence 100% studies  Biochemical validation: 100% studies | | | | | | | | | | | |
| 629 (1 RCT) | not serious ^d^ | not serious ^e^ | not serious ^f^ | very serious ^g^ | none ^h^ | ⨁◯◯◯  VERY LOW | NR | NR | RR 1.25 (0.34 to 4.60) | NA | **NA** |

**CI:** Confidence interval; **NA**: Not available; **NR**: Not reported; **NRT:** Nicotine replacement therapy; **RR:** Risk ratio

#### Explanations

a. Participants had relapsed after transdermal patch and behavioural counselling in earlier phase of trial but motivated to make a second attempt.

b. Co-intervention (both groups): Minimal additional behavioural support as per review authors.

c. Nicotine patch decreasing dose (21 mg/24 hr to 7mg/24 hr) over 12 weeks.

d. We downrated by -0.5 because of unclear risk of bias for selection bias.

e. One study only. We do not downrate.

f. An evaluation of the study-level information provided in the review signalled no issues of indirectness to the question of interest. We do not downrate this domain.

g. Confidence interval encompasses both harm (moderate) and benefit (large). Unable to assess event rates, but small sample size. We downrate this domain by -2.0.

h. Although one study for this analysis, authors conduct a publication bias assessment of the main analysis (not included in this overview) and did not signal concern despite asymmetry observed. We do not downrate this domain.

| Summary of findings: | | | | | | |
| --- | --- | --- | --- | --- | --- | --- |
| **NRT patch compared to placebo patch in relapsed smokers who are motivated to quit** | | | | | | |
| **Patient or population**: Relapsed smokers who are motivated to quit  **Setting**: No restriction  **Intervention**: NRT patch  **Comparison**: Placebo patch | | | | | | |
| Outcomes | **Anticipated absolute effects^*^** (95% CI) | | Relative effect (95% CI) | № of participants  (studies) | Certainty of the evidence (GRADE) | Comments |
|  | **Risk with Placebo patch** | **Risk with Nicotine patch** |  |  |  |  |
| Abstinence/cessation ^a,b,c^  Outcome measurement: continuous/sustained abstinence 100% studies  Biochemical validation: 100% studies  Follow up: 6 months | NA | **NA** | **RR 1.25** (0.34 to 4.60) | 629  (1 RCT) | ⨁◯◯◯  VERY LOW ^d,e,f,g,h^ | Authors state a significant increase in 28-day point prevalence abstinence (RR 2.49, 95% CI 1.11 to 5.57). Quit rates low in either analysis.  Total sample size 629 patients (not reported by group).  Date of last search: July 2017; authors assess the evidence base for NRT as a whole as stable and do not intend to update the review in the future.  AMSTAR-2: Critically low.  Authors do not GRADE this analysis. |
| ***The risk in the intervention group** (and its 95% confidence interval) is based on the assumed risk in the comparison group and the **relative effect** of the intervention (and its 95% CI).   **CI:** Confidence interval; **NA**: Not available; **RR:** Risk ratio | | | | | | |
| **GRADE Working Group grades of evidence** **High certainty:** We are very confident that the true effect lies close to that of the estimate of the effect **Moderate certainty:** We are moderately confident in the effect estimate: The true effect is likely to be close to the estimate of the effect, but there is a possibility that it is substantially different **Low certainty:** Our confidence in the effect estimate is limited: The true effect may be substantially different from the estimate of the effect **Very low certainty:** We have very little confidence in the effect estimate: The true effect is likely to be substantially different from the estimate of effect | | | | | | |

**Explanations**

a. Participants had relapsed after transdermal patch and behavioural counselling in earlier phase of trial but motivated to make a second attempt.

b. Co-intervention (both groups): Minimal additional behavioural support as per review authors.

c. Nicotine patch decreasing dose (21 mg/24 hr to 7mg/24 hr) over 12 weeks.

d. We downrated by -0.5 because of unclear risk of bias for selection bias.

e. One study only. We do not downrate.

f. An evaluation of the study-level information provided in the review signalled no issues of indirectness to the question of interest. We do not downrate this domain.

g. Confidence interval encompasses both harm (moderate) and benefit (large). Unable to assess event rates, but small sample size. We downrate this domain by -2.0.

h. Although one study for this analysis, authors conduct a publication bias assessment of the main analysis (not included in this overview) and did not signal concern despite asymmetry observed. We do not downrate this domain.

## Appendix K Table 23. NRT vs Placebo: Adverse events in smokers motivated/wishing to quit

| NRT compared to placebo in smokers motivated/wishing to quit  **Bibliography:** Hartmann-Boyce 2018; Date of last search July 2017 | | | | | | | | | | | |
| --- | --- | --- | --- | --- | --- | --- | --- | --- | --- | --- | --- |
| **Certainty assessment** | | | | | | | **Summary of findings** | | | | |
| **№ of participants (studies) Follow-up** | **Risk of bias** | **Inconsistency** | **Indirectness** | **Imprecision** | **Publication bias** | **Overall certainty of evidence** | **Study event rates (%)** | | **Relative effect (95% CI)** | **Anticipated absolute effects** | |
|  |  |  |  |  |  |  | **With Placebo** | **With Nicotine replacement therapy** |  | **Risk with Placebo** | **Risk difference with Nicotine replacement therapy** |
| **Adverse events (follow up: Not reported)** ^a,b,c,d^  Outcome measurement: Not reported | | | | | | | | | | | |
| NR (6 RCTs) | not serious to very serious ^e^ | not serious to very serious ^f^ | not serious to very serious ^g^ | not serious to very serious ^h^ | none ^i^ | unable to assess | For nicotine gum, most common adverse events are hiccoughs, gastrointestinal disturbances, jaw pain, and orodental problems. With nicotine patch, typically mild skin sensitivity and local skin irritation in up to 54% of patch users. Throat irritation, coughing, and oral burning are common with nicotine inhalator. With nasal spray, irritation and runny nose. For oral spray, hiccoughs and throat irritation. Symptoms associated with nicotine sublingual tablets include hiccoughs, burning and smarting sensation in the mouth, sore throat, coughing, dry lips, and mouth ulcers. Reactions to NRT are usually not severe enough to prompt discontinuation of treatment. Trials could not be pooled due to heterogeneity with respect to the nature, timing and duration of symptoms. | | | | |
| **Adverse events: Palpitations/chest pains (follow up: Range of follow-up times)** ^a,j,k,l^  Outcome measurement: Unclear/NR | | | | | | | | | | | |
| 11074 (15 RCTs) | serious ^m^ | not serious ^n^ | not serious ^o^ | not serious ^p^ | none ^q^ | ⨁⨁⨁◯  MODERATE | 62/4401 (1.4%) | 165/6673 (2.5%) | **OR 1.88** (1.37 to 2.57)  **RR 1.86**  (1.36 to 2.51) | 14 per 1,000 | **12 more per 1,000** (from 5 more to 21 more) |
| **Attrition (follow up: Not reported)** ^r,s,t^  Outcome measurement: Not reported/unclear | | | | | | | | | | | |
| NR (RCTs, NR) | not serious to very serious ^u^ | not serious ^v^ | not serious to very serious ^w^ | not serious to very serious ^x^ | none ^y^ | unable to assess | Authors state that attrition rates in NRT groups were generally similar to or lower than in control groups among included studies. | | | | |

**CI:** Confidence interval; **NR**: Not reported; **NRT**: Nicotine replacement therapy; **OR:** Odds ratio; **RR:** Risk ratio

#### Explanations

a. Included and excluded studies in this analysis.

b. Authors state that evidence relates to a motivated to quit population. Study characteristics not reported for 67% of studies in this analysis.

c. Co-interventions (both groups): 67% studies without information reported. Remaining studies with high level of behavioural support.

d. Formulation and dose not reported for majority of studies.

e. Majority of studies in this analysis were not evaluated for the risk of bias. We cannot rate this domain.

f. Difficult to assess this domain as NRT formulations were considered together and report on various adverse events. We cannot rate this domain.

g. Information not reported for majority of studies in this analysis. We cannot rate this domain.

h. Unclear sample size and unable to assess optimal information size and confidence intervals. We cannot rate this domain.

i. Authors did not signal concern despite funnel plot asymmetry in the main analysis. We do not downrate this domain.

j. Authors state evidence relates to a motivated to quit population. Explicitly declared in 47% of studies, while another 40% were excluded studies without study characteristics reported.

k. Co-interventions (both groups): High-intensity behavioural support provided in 33% studies, low-intensity (minimal/none) provided in 27%, and not reported in 40% of studies.

l. Varied NRT formulations and doses among nine studies; remaining studies NR.

m. 68% of evidence at unclear risk of bias for multiple domains (mainly selection bias plus blinding with or without attrition bias) or unknown because no bias assessments were undertaken by review authors. We downrate by -1.0.

n. Some variation in estimates but confidence intervals are overlapping. I2=10%, p=0.35. We do not downrate this domain.

o. 40% of studies without available information to assess. Of remaining studies, no issues of indirectness signalled. We do not downrate this domain.

p. Confidence interval encompasses one range of effect (little to no difference to small but important harm). The optimal information size not met (total of 227 events), but adequate sample size. We do not downrate this domain.

q. Mix of results among studies and authors did not signal concern despite funnel plot asymmetry in the main analysis. We do not downrate this domain.

r. Authors state evidence relates to a motivated to quit population. Majority of studies in analysis are not formally included in the review and study characteristics not reported.

s. Studies for this analysis not specified. Based on studies in the review, we presume a mix of high intensity and low intensity behavioural support.

t. Formulations and doses not reported.

u. Studies for this analysis not specified. We cannot rate this domain.

v. Because authors report little difference between groups across studies, we do not downrate this domain.

w. Studies for this analysis not specified. We cannot rate this domain.

x. Unclear sample size and number of studies and unable to assess optimal information size and confidence intervals. We cannot rate this domain.

y. Given findings for this outcome and that authors did not signal concern despite funnel plot asymmetry in the main analysis. We do not downrate this domain.

| Summary of findings: | | | | | | |
| --- | --- | --- | --- | --- | --- | --- |
| **NRT compared to placebo in smokers motivated/wishing to quit** | | | | | | |
| **Patient or population**: Smokers motivated/wishing to quit  **Setting**: No restriction  **Intervention**: NRT  **Comparison**: Placebo | | | | | | |
| Outcomes | **Anticipated absolute effects^*^** (95% CI) | | Relative effect (95% CI) | № of participants  (studies) | Certainty of the evidence (GRADE) | Comments |
|  | **Risk with Placebo** | **Risk with Nicotine replacement therapy** |  |  |  |  |
| Adverse events ^a,b,c,d^  Outcome measurement: Not reported  Follow up: Not reported | For nicotine gum, most common adverse events are hiccoughs, gastrointestinal disturbances, jaw pain, and orodental problems. With nicotine patch, typically mild skin sensitivity and local skin irritation in up to 54% of patch users. Throat irritation, coughing, and oral burning are common with nicotine inhalator. With nasal spray, irritation and runny nose. For oral spray, hiccoughs and throat irritation. Symptoms associated with nicotine sublingual tablets include hiccoughs, burning and smarting sensation in the mouth, sore throat, coughing, dry lips, and mouth ulcers. Reactions to NRT are usually not severe enough to prompt discontinuation of treatment. Trials could not be pooled due to heterogeneity with respect to the nature, timing and duration of symptoms. | | NR | NR  (6 RCTs) | unable to assess ^e,f,g,h,i^ | Date of last search: July 2017; authors assess the evidence base for NRT as a whole as stable and do not intend to update the review in the future.  AMSTAR-2: Critically low.  Authors do not GRADE this analysis. |
| Adverse events: Palpitations/chest pains ^a,j,k,l^  Outcome measurement: Unclear/NR  Follow up: Range of follow-up times | 14 per 1,000 | **26 per 1,000** (19 to 35) | **OR 1.88** (1.37 to 2.57)  **RR 1.86** (1.36 to 2.51) | 11074 (15 RCTs) | ⨁⨁⨁◯  MODERATE ^m,n,o,p,q^ | Authors remark that events were extremely rare. OR data convert to RR 1.86 (1.36 to 2.51).  Fixed effects meta-analysis. OR converts to RR 1.86 (95% CI 1.36 to 2.51).  Date of last search: July 2017; authors assess the evidence base for NRT as a whole as stable and do not intend to update the review in the future.  AMSTAR-2: Critically low.  Authors do not GRADE this analysis. |
| Attrition ^r,s,t^  Outcome measurement: Not reported/unclear  Follow up: Not reported | Authors state that attrition rates in NRT groups were generally similar to or lower than in control groups among included studies. | | NR | NR  (RCTs, NR) | unable to assess ^u,v,w,x,y^ | Date of last search: July 2017; authors assess the evidence base for NRT as a whole as stable and do not intend to update the review in the future.  AMSTAR-2: Critically low.  Authors do not GRADE this analysis. |
| ***The risk in the intervention group** (and its 95% confidence interval) is based on the assumed risk in the comparison group and the **relative effect** of the intervention (and its 95% CI).   **CI:** Confidence interval; **NR**: Not reported; **NRT**: Nicotine replacement therapy; **OR:** Odds ratio; **RR:** Risk ratio | | | | | | |
| **GRADE Working Group grades of evidence** **High certainty:** We are very confident that the true effect lies close to that of the estimate of the effect **Moderate certainty:** We are moderately confident in the effect estimate: The true effect is likely to be close to the estimate of the effect, but there is a possibility that it is substantially different **Low certainty:** Our confidence in the effect estimate is limited: The true effect may be substantially different from the estimate of the effect **Very low certainty:** We have very little confidence in the effect estimate: The true effect is likely to be substantially different from the estimate of effect | | | | | | |

**Explanations**

a. Included and excluded studies in this analysis.

b. Authors state that evidence relates to a motivated to quit population. Study characteristics not reported for 67% of studies in this analysis.

c. Co-interventions (both groups): 67% studies without information reported. Remaining studies with high level of behavioural support.

d. Formulation and dose not reported for majority of studies.

e. Majority of studies in this analysis were not evaluated for the risk of bias. We cannot rate this domain.

f. Difficult to assess this domain as NRT formulations were considered together and report on various adverse events. We cannot rate this domain.

g. Information not reported for majority of studies in this analysis. We cannot rate this domain.

h. Unclear sample size and unable to assess optimal information size and confidence intervals. We cannot rate this domain.

i. Authors did not signal concern despite funnel plot asymmetry in the main analysis. We do not downrate this domain.

j. Authors state evidence relates to a motivated to quit population. Explicitly declared in 47% of studies, while another 40% were excluded studies without study characteristics reported.

k. Co-interventions (both groups): High-intensity behavioural support provided in 33% studies, low-intensity (minimal/none) provided in 27%, and not reported in 40% of studies.

l. Varied NRT formulations and doses among nine studies; remaining studies NR.

m. 68% of evidence at unclear risk of bias for multiple domains (mainly selection bias plus blinding with or without attrition bias) or unknown because no bias assessments were undertaken by review authors. We downrate by -1.0.

n. Some variation in estimates but confidence intervals are overlapping. I2=10%, p=0.35. We do not downrate this domain.

o. 40% of studies without available information to assess. Of remaining studies, no issues of indirectness signalled. We do not downrate this domain.

p. Confidence interval encompasses one range of effect (little to no difference to small but important harm). The optimal information size not met (total of 227 events), but adequate sample size. We do not downrate this domain.

q. Mix of results among studies and authors did not signal concern despite funnel plot asymmetry in the main analysis. We do not downrate this domain.

r. Authors state evidence relates to a motivated to quit population. Majority of studies in analysis are not formally included in the review and study characteristics not reported.

s. Studies for this analysis not specified. Based on studies in the review, we presume a mix of high intensity and low intensity behavioural support.

t. Formulations and doses not reported.

u. Studies for this analysis not specified. We estimate risk of bias to range from -0.5 to -1.0.

v. Because authors report little difference between groups across studies, we do not downrate this domain.

w. Studies for this analysis not specified. We cannot rate this domain.

x. Unclear sample size and number of studies and unable to assess optimal information size and confidence intervals. We cannot rate this domain.

y. Given findings for this outcome and that authors did not signal concern despite funnel plot asymmetry in the main analysis. We do not downrate this domain.

# Hollands 2019 {3841}

## Appendix K Table 24. Interventions to increase adherence to medications for tobacco dependence versus Usual or standard care: Smoking cessation, adverse events, and change in emotional state (anxiety) in smokers motivated/wishing to quit or reduce smoking

| Interventions to increase adherence to medications for tobacco dependence compared to usual or standard care in smokers motivated/wishing to quit or reduce smoking  **Bibliography:** Hollands 2019; Date of last search: September 2018 | | | | | | | | | | | |
| --- | --- | --- | --- | --- | --- | --- | --- | --- | --- | --- | --- |
| **Certainty assessment** | | | | | | | **Summary of findings** | | | | |
| **№ of participants (studies) Follow-up** | **Risk of bias** | **Inconsistency** | **Indirectness** | **Imprecision** | **Publication bias** | **Overall certainty of evidence** | **Study event rates (%)** | | **Relative effect (95% CI)** | **Anticipated absolute effects** | |
|  |  |  |  |  |  |  | **With usual or standard care** | **With Interventions to increase adherence to medications for tobacco dependence** |  | **Risk with usual or standard care** | **Risk difference with Interventions to increase adherence to medications for tobacco dependence** |
| **Abstinence/Cessation (follow up: 6 months)** ^a,b,c^  Outcome measurement: point prevalence 75%, prolonged abstinence 25% studies  Biochemical validation: 75% studies | | | | | | | | | | | |
| 3593 (5 RCTs) | very serious ^d^ | serious ^e^ | not serious ^f^ | not serious ^g^ | none ^h^ | ⨁◯◯◯  VERY LOW | 361/1777 (20.3%) | 412/1816 (22.7%) | RR 1.16 (0.96 to 1.40) | 203 per 1,000 | **33 more per 1,000** (from 8 fewer to 81 more) |
| **Adverse events (follow up: Unclear/NR)** ^i,j,k,l^ | | | | | | | | | | | |
| Unclear/NR (4 RCTs) ^l,m^ | serious ^n^ | not serious ^o^ | not serious ^f^ | serious to very serious ^p^ | none ^q^ | unable to assess | Two studies reported no serious adverse events during the study period; one study reported no treatment related adverse events. The fourth study reported no difference in adverse events between groups. | | | | |
| **Change in emotional state - anxiety (follow up: 1-week and 6 months)** ^r,s,t^  Outcome measurement: STAI-6 | | | | | | | | | | | |
| Unclear/NR (1 RCT) ^u^ | not serious ^v^ | not serious ^w^ | not serious ^f^ | not serious to very serious ^x^ | none ^y^ | unable to assess | The study reports no difference between groups regarding levels of anxiety at both timepoints. | | | | |

**CI:** Confidence interval; **RR:** Risk ratio; **STAI:** Short-form Spielberger State-Trait Anxiety Inventory

#### Explanations

a. One trial recruited only males with erectile dysfunction. Remaining trials in this analysis recruited general smokers. Participants in all trials were motivated to quit or reduce smoking and agreed to receive medication and behavioural support.

b. Interventions: Interventions were aimed to increase adherence to tobacco cessation medications. As compared to the control condition, in 3 of 4 trials, the intervention included an additional component focusing on medication adherence with additional contact time. In the remaining trial, the nature of the intervention differed across arms but contact time was similar (i.e., tailoring NRT dosage based on genotype rather than phenotype). The intervention involved specialized behavioural counselling in 3 of 4 studies (i.e., counselling based on motivational interviewing techniques and 4R approach, counselling based on withdrawal-oriented therapy); this was received by 67.5% of participants in this analysis. The intervention was delivered by phone in one study and in-person in three studies. Trained counsellors delivered the intervention in three studies while nurses delivered the intervention in one study. All participants were receiving NRT.

c. Control: Eligible control conditions were usual or standard care which could consist of minimal support or varying degrees of behavioural support. All trials provided some behavioural support to control participants which ranged up to seven weekly sessions. In one trial, the behavioural support involved specialized behavioural counselling (i.e., based on withdrawal-oriented therapy) which was received by 21% of control participants in this analysis. All participants were receiving NRT.

d. 90% of evidence with substantive bias issues; 19% at unclear risk of bias for two domains (selection, performance/detection, and baseline imbalance) and 71% at high risk for one domain (biochemical validation) and unclear risk for two domains (selection and performance/detection). We downrate this domain by -2.0.

e. Variation in point estimates and confidence interval of one trial has little overlap with others (I2=72%, p=0.01). We downrate this domain by -1.5.

f. No indirectness.

g. Confidence interval encompasses two ranges of effect (little to no difference to moderate benefit). The optimal information size is met and there is an adequate sample size (>2000 participants). We downrate this domain by -0.5.

h. Although search not comprehensive, mix of results across trials. We do not downrate this domain.

i. All trials recruited general smokers who were motivated to quit or reduce smoking and agreed to receive medication and behavioural support.

j. Intervention: Interventions were aimed to increase adherence to tobacco cessation medications. In one trial, the intervention included an additional component focusing on medication adherence and additional contact time as compared to the control condition. In two trials, the nature of the intervention differed across arms but contact time was similar; one trial tailored NRT dosage based on genotype (versus phenotype in controls) and the second provided a personalized feedback component on medication use and adherence. The intervention involved specialized behavioural counselling in one study (i.e., counselling based on withdrawal-oriented therapy). The intervention was delivered by phone in one study and in-person in two studies. Trained counsellors delivered the intervention in two studies while nurses delivered the intervention in one study. All participants were receiving NRT.

k. Control: Eligible control conditions were usual or standard care which could consist of minimal support or varying degrees of behavioural support. All trials provided some behavioural support to control participants which ranged up to seven weekly sessions. In one trial, the behavioural support involved specialized behavioural counselling (i.e., based on withdrawal-oriented therapy). All participants were receiving NRT.

l. Timing of adverse event ascertainment not reported. Two trials followed participants for 6 months and one for up to 7 weeks for smoking cessation outcome.

m. Number of participants included in this analysis is not reported. A total of 1683 participants included across all three trials (Intervention = 849; Control: 834).

n. Two trials with substantive bias issues; one trial at unclear risk for two domains (selection and performance/detection bias) and one trial at high risk for one domain (attrition) and unclear risk for three domains (selection, performance/detection, selective reporting bias). We downrate this domain by -1.5.

o. Differences in how AEs are reported across trials (e.g., SAEs only versus all AEs) precludes adequate comparison of consistency. Overall, all studies report no events or no difference between groups. We do not downrate this domain.

p. Unable to assess confidence intervals. Unclear sample size analyzed but approximately 1683 total participants across all 3 trials and the optimal information size cannot be met. We cannot rate this domain.

q. Although search not comprehensive, negative studies included. We do not downrate this domain.

r. Trial recruited general smokers who were motivated to quit or reduce smoking and agreed to receive medication and behavioural support.

s. Intervention: Intervention was aimed at increasing adherence to tobacco cessation medication. In the only trial included in this analysis, the nature of the intervention differed across arms but contact time was similar; the intervention arm received tailored NRT dosage and rationale based on genotype. The intervention was delivered in-person by nurses and involved specialized behavioural counselling (i.e., counselling based on withdrawal-oriented therapy). All participants were receiving NRT.

t. Control: NRT dosage and rationale based on phenotype. The behavioural support involved specialized behavioural counselling (i.e., based on withdrawal-oriented therapy). All participants were receiving NRT.

u. Number of participants included in this analysis is not reported. The trial includes a total of 633 participants (Intervention: 315, Control: 318).

v. Trial at unclear risk of performance/detection bias. We downrate this domain by -0.5.

w. Not applicable - single study.

x. Unable to assess confidence intervals. Unclear sample size analyzed but approximately 633 total participants. The optimal information size may be able to be met, but not enough information to confirm. We cannot rate this domain.

y. Although search not comprehensive, trial reports negative findings. We do not downrate this domain.

| Summary of findings: | | | | | | |
| --- | --- | --- | --- | --- | --- | --- |
| **Interventions to increase adherence to medications for tobacco dependence compared to usual or standard care in smokers motivated/wishing to quit or reduce smoking** | | | | | | |
| **Patient or population**: Smokers motivated/wishing to quit or reduce smoking  **Setting**: No restriction  **Intervention**: Interventions to increase adherence to medications for tobacco dependence  **Comparison**: Usual or standard care | | | | | | |
| Outcomes | **Anticipated absolute effects^*^** (95% CI) | | Relative effect (95% CI) | № of participants  (studies) | Certainty of the evidence (GRADE) | Comments |
|  | **Risk with usual or standard care** | **Risk with Interventions to increase adherence to medications for tobacco dependence** |  |  |  |  |
| Abstinence/Cessation ^a,b,c^  Outcome measurement: point prevalence 75%, prolonged abstinence 25% studies.  Biochemical validation: 75% studies  Follow up: 6 months | 203 per 1,000 | **236 per 1,000** (195 to 284) | RR 1.16 ( 0.96 to 1.40) | 3593 (5 RCTs) | ⨁◯◯◯  VERY LOW ^d,e,f,g,h^ | Heterogeneity attributed to one trial; sensitivity analysis excluding this study yields I2 of 0% and effect estimate of greater magnitude (RR 1.63, 95%CI: 1.24, 2.14, n=2062). Using random effects model, magnitude of effect estimates increases but confidence interval is null inclusive (RR 1.36, 95%CI: 0.96, 1.94, I2=72%).  Fixed effects analysis.  Date of last search: September 2018.  AMSTAR-2: Low.  Review authors rated certainty as low. ^i^ |
| Adverse events ^j,k,l,m^  Follow up: Unclear/NR | One study each reported no serious adverse events, or no treatment related adverse events. The third study reported no difference in adverse events between groups. | |  | ( 4 RCTs) ^n^ | unable to assess ^f,o,p,q,r^ | Date of last search: September 2018.  AMSTAR-2: Low.  Not GRADEd by review authors. |
| Change in emotional state - anxiety ^s,t,u^  Outcome measurement: STAI-6  Follow up: 1-week and 6 months | The study reports no difference between groups regarding levels of anxiety at both timepoints. | |  | (1 RCT) ^v^ | unable to assess ^f,w,x,y,z^ | Date of last search: September 2018.  AMSTAR-2: Low.  Not GRADEd by review authors. |
| ***The risk in the intervention group** (and its 95% confidence interval) is based on the assumed risk in the comparison group and the **relative effect** of the intervention (and its 95% CI).   **CI:** Confidence interval; **RR:** Risk ratio | | | | | | |
| **GRADE Working Group grades of evidence** **High certainty:** We are very confident that the true effect lies close to that of the estimate of the effect **Moderate certainty:** We are moderately confident in the effect estimate: The true effect is likely to be close to the estimate of the effect, but there is a possibility that it is substantially different **Low certainty:** Our confidence in the effect estimate is limited: The true effect may be substantially different from the estimate of the effect **Very low certainty:** We have very little confidence in the effect estimate: The true effect is likely to be substantially different from the estimate of effect | | | | | | |

#### Explanations

a. One trial recruited only males with erectile dysfunction. Remaining trials in this analysis recruited general smokers. Participants in all trials were motivated to quit or reduce smoking and agreed to receive medication and behavioural support.

b. Interventions: Interventions were aimed to increase adherence to tobacco cessation medications. As compared to the control condition, in 3 of 4 trials, the intervention included an additional component focusing on medication adherence with additional contact time. In the remaining trial, the nature of the intervention differed across arms but contact time was similar (i.e., tailoring NRT dosage based on genotype rather than phenotype). The intervention involved specialized behavioural counselling in 3 of 4 studies (i.e., counselling based on motivational interviewing techniques and 4R approach, counselling based on withdrawal-oriented therapy); this was received by 67.5% of participants in this analysis. The intervention was delivered by phone in one study and in-person in three studies. Trained counsellors delivered the intervention in three studies while nurses delivered the intervention in one study. All participants were receiving NRT.

c. Control: Eligible control conditions were usual or standard care which could consist of minimal support or varying degrees of behavioural support. All trials provided some behavioural support to control participants which ranged up to seven weekly sessions. In one trial, the behavioural support involved specialized behavioural counselling (i.e., based on withdrawal-oriented therapy) which was received by 21% of control participants in this analysis. All participants were receiving NRT.

d. 90% of evidence with substantive bias issues; 19% at unclear risk of bias for two domains (selection, performance/detection, and baseline imbalance) and 71% at high risk for one domain (biochemical validation) and unclear risk for two domains (selection and performance/detection). We downrate this domain by -2.0.

e. Variation in point estimates and confidence interval of one trial has little overlap with others (I2=72%, p=0.01). We downrate this domain by -1.5.

f. No indirectness.

g. Confidence interval encompasses two ranges of effect (little to no difference to moderate benefit). The optimal information size is met and there is an adequate sample size (>2000 participants). We downrate this domain by -0.5.

h. Although search not comprehensive, mix of results across trials. We do not downrate this domain.

i. Review authors rate certainty of evidence as low (downrating for risk of bias and inconsistency).

j. All trials recruited general smokers who were motivated to quit or reduce smoking and agreed to receive medication and behavioural support.

k. Intervention: Interventions were aimed to increase adherence to tobacco cessation medications. In one trial, the intervention included an additional component focusing on medication adherence and additional contact time as compared to the control condition. In two trials, the nature of the intervention differed across arms but contact time was similar; one trial tailored NRT dosage based on genotype (versus phenotype in controls) and the second provided a personalized feedback component on medication use and adherence. The intervention involved specialized behavioural counselling in one study (i.e., counselling based on withdrawal-oriented therapy). The intervention was delivered by phone in one study and in-person in two studies. Trained counsellors delivered the intervention in two studies while nurses delivered the intervention in one study. All participants were receiving NRT.

l. Control: Eligible control conditions were usual or standard care which could consist of minimal support or varying degrees of behavioural support. All trials provided some behavioural support to control participants which ranged up to seven weekly sessions. In one trial, the behavioural support involved specialized behavioural counselling (i.e., based on withdrawal-oriented therapy). All participants were receiving NRT.

m. Timing of adverse event ascertainment not reported. Two trials followed participants for 6 months and one for up to 7 weeks for smoking cessation outcome.

n. Number of participants included in this analysis is not reported. A total of 1683 participants included across all three trials (Intervention = 849; Control: 834).

o. Two trials with substantive bias issues; one trial at unclear risk for two domains (selection and performance/detection bias) and one trial at high risk for one domain (attrition) and unclear risk for three domains (selection, performance/detection, selective reporting bias). We downrate this domain by -1.5.

p. Differences in how AEs are reported across trials (e.g., SAEs only versus all AEs) precludes adequate comparison of consistency. Overall, all studies report no events or no difference between groups. We do not downrate this domain.

q. Unable to assess confidence intervals. Unclear sample size analyzed but approximately 1683 total participants across all 3 trials and the optimal information size cannot be met. We cannot rate this domain.

r. Although search not comprehensive, negative studies included. We do not downrate this domain.

s. Trial recruited general smokers who were motivated to quit or reduce smoking and agreed to receive medication and behavioural support.

t. Intervention: Intervention was aimed at increasing adherence to tobacco cessation medication. In the only trial included in this analysis, the nature of the intervention differed across arms but contact time was similar; the intervention arm received tailored NRT dosage and rationale based on genotype. The intervention was delivered in-person by nurses and involved specialized behavioural counselling (i.e., counselling based on withdrawal-oriented therapy). All participants were receiving NRT.

u. Control: NRT dosage and rationale based on phenotype. The behavioural support involved specialized behavioural counselling (i.e., based on withdrawal-oriented therapy). All participants were receiving NRT.

v. Number of participants included in this analysis is not reported. The trial includes a total of 633 participants (Intervention: 315, Control: 318).

w. Trial at unclear risk of performance/detection bias. We downrate this domain by -0.5.

x. Not applicable - single study.

y. Unable to assess confidence intervals. Unclear sample size analyzed but approximately 633 total participants. The optimal information size may be able to be met, but not enough information to confirm. We cannot rate this domain.

z. Although search not comprehensive, trial reports negative findings. We do not downrate this domain.

# Howes 2020 {96}

## Appendix K Table 25. Bupropion versus Placebo: Smoking cessation and reduction in smokers not motivated/wishing to quit

| Bupropion compared to placebo in smokers not motivated/wishing to quit  **Bibliography:** Howes 2020; Date of last search: May 2019 | | | | | | | | | | | |
| --- | --- | --- | --- | --- | --- | --- | --- | --- | --- | --- | --- |
| **Certainty assessment** | | | | | | | **Summary of findings** | | | | |
| **№ of participants (studies) Follow-up** | **Risk of bias** | **Inconsistency** | **Indirectness** | **Imprecision** | **Publication bias** | **Overall certainty of evidence** | **Study event rates (%)** | | **Relative effect (95% CI)** | **Anticipated absolute effects** | |
|  |  |  |  |  |  |  | **With placebo** | **With Bupropion** |  | **Risk with placebo** | **Risk difference with Bupropion** |
| **Abstinence/Cessation (follow up: 6 months)** ^a,b,c^  Outcome measurement: unclear/NR  Biochemical validation: 100% studies | | | | | | | | | | | |
| 594 (1 RCT) | very serious ^d^ | not serious ^e^ | not serious ^f^ | very serious ^g^ | none ^h^ | ⨁◯◯◯  VERY LOW | 16/299 (5.4%) | 20/295 (6.8%) | RR 1.27 (0.67 to 2.40) | 54 per 1,000 | **14 more per 1,000** (from 18 fewer to 75 more) |
| **Reduction in cigarettes per day (follow up: 12 months)** ^a,b,c,i^  Outcome measurement: unclear/NR  Biochemical validation: unclear/NR | | | | | | | | | | | |
| 594 (1 RCT) | very serious ^d^ | not serious ^e^ | not serious ^f^ | not serious to very serious ^j^ | none ^h^ | unable to assess | No significant difference between groups. | | | | |
| **Reduction in cotinine >50% from baseline (follow up: 12 months)** ^a,b,c^  Outcome measurement: unclear/NR | | | | | | | | | | | |
| 327 (1 RCT) | very serious ^d^ | not serious ^e^ | not serious ^f^ | very serious ^k^ | none ^h^ | ⨁◯◯◯  VERY LOW | 8/174 (4.6%) | 3/153 (2.0%) | RR 0.43 (0.12 to 1.58) | 46 per 1,000 | **26 fewer per 1,000** (from 40 fewer to 27 more) |

**CI:** Confidence interval; **CPD:** Cigarettes per day; **NR:** Not reported; **RR:** Risk ratio

#### Explanations

a. Trial recruited smokers interested in reducing but not quitting. Those who became willing to quit entered the cessation phase of the trial which included weekly counselling for 7 weeks and then 19 weeks of follow-up.

b. Bupropion 300 mg/day for 26 weeks

c. Behavioural co-intervention provided to both trial arms.

d. Trial at high risk for attrition bias and unclear risk for both selection and performance/detection bias. Unclear whether reduction in cigarettes was biochemically validated. We downrate this domain by -2.0.

e. Not applicable - single study.

f. No indirectness.

g. Confidence interval encompasses both harm (small but important) and benefit (moderate). The optimal information size not met (total of 36 events) and inadequate sample size (<2000 participants). We downrate this domain by -2.0

h. Search not completely comprehensive but study reports negative findings. We do not downrate this domain.

i. Urine cotinine levels were measured in the trial and reported as a separate outcome. However, it is unclear whether reduced levels of cotinine at follow-up was used to validate self-reported reduction in CPD.

j. Unable to assess confidence intervals. Unable to assess optimal information size but a total of 594 participants. We cannot rate this domain.

k. Confidence interval encompasses both harm (small but important) and benefit (small but important). The optimal information size not met (total of 11 events) and inadequate sample size (<2000 participants). We downrate this domain by -2.0.

| Summary of findings: | | | | | | |
| --- | --- | --- | --- | --- | --- | --- |
| **Bupropion compared to placebo in smokers not motivated/wishing to quit** | | | | | | |
| **Patient or population**: Smokers not motivated/wishing to quit  **Setting**: No restriction  **Intervention**: Bupropion  **Comparison**: Placebo | | | | | | |
| Outcomes | **Anticipated absolute effects^*^** (95% CI) | | Relative effect (95% CI) | № of participants  (studies) | Certainty of the evidence (GRADE) | Comments |
|  | **Risk with placebo** | **Risk with Bupropion** |  |  |  |  |
| Abstinence/Cessation ^a,b,c^  Outcome measurement: unclear/NR.  Biochemical validation: 100% studies  Follow up: 6 months | 54 per 1,000 | **68 per 1,000** (36 to 128) | RR 1.27 (0.67 to 2.40) | 594 (1 RCT) | ⨁◯◯◯  VERY LOW ^d,e,f,g,h^ | Data also available in Lindson-Hawley 2016. Note that rating of attrition bias by review authors differs across reviews resulting in different ROB domain ratings.  AMSTAR-2 rating: Critically low.  Date of last search: May 2019.  Not GRADEd by review authors. |
| Reduction in cigarettes per day ^a,b,c,i^,  Outcome measurement: unclear/NR.  Biochemical validation: unclear/NR  Follow up: 12 months | No significant difference between groups. | |  | 594 (1 RCT) | ⨁◯◯◯  VERY LOW ^d,e,f,g,j^ | AMSTAR-2 rating: Critically low.  Date of last search: May 2019.  Not GRADEd by review authors. |
| Reduction in cotinine >50% from baseline ^a,b,c^  Outcome measurement: unclear/NR  Follow up: 12 months | 46 per 1,000 | **20 per 1,000** (6 to 73) | RR 0.43 (0.12 to 1.58) | 327 (1 RCT) | ⨁◯◯◯  VERY LOW ^d,e,f,g,k^ | Data also available in Lindson-Hawley 2016. Note that rating of attrition bias by review authors differs across reviews resulting in different ROB domain ratings.  AMSTAR-2 rating: Critically low.  Date of last search: May 2019.  Not GRADEd by review authors. |
| ***The risk in the intervention group** (and its 95% confidence interval) is based on the assumed risk in the comparison group and the **relative effect** of the intervention (and its 95% CI).   **CI:** Confidence interval; **CPD:** Cigarettes per day; **NR:** Not reported; **RR:** Risk ratio | | | | | | |
| **GRADE Working Group grades of evidence** **High certainty:** We are very confident that the true effect lies close to that of the estimate of the effect **Moderate certainty:** We are moderately confident in the effect estimate: The true effect is likely to be close to the estimate of the effect, but there is a possibility that it is substantially different **Low certainty:** Our confidence in the effect estimate is limited: The true effect may be substantially different from the estimate of the effect **Very low certainty:** We have very little confidence in the effect estimate: The true effect is likely to be substantially different from the estimate of effect | | | | | | |

#### Explanations

a. Trial recruited smokers interested in reducing but not quitting. Those who became willing to quit entered the cessation phase of the trial which included weekly counselling for 7 weeks and then 19 weeks of follow-up.

b. Bupropion 300 mg/day for 26 weeks

c. Behavioural co-intervention provided to both trial arms.

d. Trial at high risk for attrition bias and unclear risk for both selection and performance/detection bias. Unclear whether reduction in cigarettes was biochemically validated. We downrate this domain by -2.0.

e. Not applicable - single study.

f. No indirectness.

g. Confidence interval encompasses both harm (small but important) and benefit (moderate). The optimal information size not met (total of 36 events) and inadequate sample size (<2000 participants). We downrate this domain by -2.0

h. Search not completely comprehensive but study reports negative findings. We do not downrate this domain.

i. Urine cotinine levels were measured in the trial and reported as a separate outcome. However, it is unclear whether reduced levels of cotinine at follow-up was used to validate self-reported reduction in CPD.

j. Unable to assess confidence intervals. Unable to assess optimal information size but a total of 594 participants. We cannot rate this domain.

k. Confidence interval encompasses both harm (small but important) and benefit (small but important). The optimal information size not met (total of 11 events) and inadequate sample size (<2000 participants). We downrate this domain by -2.0.

## Appendix K Table 26. Bupropion versus Placebo: Change in emotional state (depressive symptoms) in general/mixed population of smokers

| Bupropion compared to placebo in general/mixed population of smokers  **Bibliography:** Howes 2020; Date of last search: May 2019 | | | | | | | | | | | |
| --- | --- | --- | --- | --- | --- | --- | --- | --- | --- | --- | --- |
| **Certainty assessment** | | | | | | | **Summary of findings** | | | | |
| **№ of participants (studies) Follow-up** | **Risk of bias** | **Inconsistency** | **Indirectness** | **Imprecision** | **Publication bias** | **Overall certainty of evidence** | **Study event rates (%)** | | **Relative effect (95% CI)** | **Anticipated absolute effects** | |
|  |  |  |  |  |  |  | **With placebo** | **With Bupropion** |  | **Risk with placebo** | **Risk difference with Bupropion** |
| **Change in emotional state (depressive symptoms) (follow up: specific timepoints NR)** ^a,b,c^  Outcome measurement: unclear/NR | | | | | | | | | | | |
| Unclear/NR (1 RCT) ^d^ | not serious ^e^ | not serious ^f^ | not serious ^g^ | serious to very serious ^h^ | none ^i^ | unable to assess | During treatment, most participants in both arms experienced reduction in depressive symptoms and this was sustained at follow-up. A between-group difference was observed for highly dependent smokers with greater reduction in the bupropion arm. The reduction was not sustained at follow-up. | | | | |

**CI:** Confidence interval; **NR:** Not reported

#### Explanations

a. Trial recruited general smokers and excluded those with psychiatric disorders.

b. Trial examined bupropion at 300 mg/day for 10 weeks.

c. Both arms received behavioural co-intervention.

d. Number of participants included in this analysis NR. Trial included 555 smokers total.

e. The trial is at unclear risk of performance/detection bias. We downrate this domain by -0.5.

f. Not applicable - single study.

g. No indirectness.

h. Unable to assess confidence intervals. Unable to assess optimal information size and sample size analyzed unclear but total of 555 total participants included. We cannot rate this domain.

i. Search strategy not completely comprehensive and study reports some positive findings, but lack of negative trials may be reflective of an under-researched area as opposed to suppression of findings. Given not enough information exists to strongly suspect publication bias, we do not downrate this domain.

| Summary of findings: | | | | | | |
| --- | --- | --- | --- | --- | --- | --- |
| **Bupropion compared to placebo in general/mixed population of smokers** | | | | | | |
| **Patient or population**: General/mixed population of smokers  **Setting**: No restriction  **Intervention**: Bupropion  **Comparison**: Placebo | | | | | | |
| Outcomes | **Anticipated absolute effects^*^** (95% CI) | | Relative effect (95% CI) | № of participants  (studies) | Certainty of the evidence (GRADE) | Comments |
|  | **Risk with placebo** | **Risk with Bupropion** |  |  |  |  |
| Change in emotional state (depressive symptoms) ^a,b,c^  Outcome measurement: unclear/NR  Follow up: specific timepoints NR | During treatment, most participants in both arms experienced reduction in depressive symptoms and this was sustained at follow-up. A between group difference was observed for highly dependent smokers with greater reduction in the bupropion arm. The reduction was not sustained at follow-up. | |  | Unclear/NR  (1 RCT) ^d^ | unable to assess ^e,f,g,h,i^ | AMSTAR-2 rating: Critically low.  Date of last search: May 2019.  Not GRADEd by review authors. |
| ***The risk in the intervention group** (and its 95% confidence interval) is based on the assumed risk in the comparison group and the **relative effect** of the intervention (and its 95% CI).   **CI:** Confidence interval; **NR:** Not reported | | | | | | |
| **GRADE Working Group grades of evidence** **High certainty:** We are very confident that the true effect lies close to that of the estimate of the effect **Moderate certainty:** We are moderately confident in the effect estimate: The true effect is likely to be close to the estimate of the effect, but there is a possibility that it is substantially different **Low certainty:** Our confidence in the effect estimate is limited: The true effect may be substantially different from the estimate of the effect **Very low certainty:** We have very little confidence in the effect estimate: The true effect is likely to be substantially different from the estimate of effect | | | | | | |

#### Explanations

a. Trial recruited general smokers and excluded those with psychiatric disorders.

b. Trial examined bupropion at 300 mg/day for 10 weeks.

c. Both arms received behavioural co-intervention.

d. Number of participants included in this analysis NR. Trial included 555 smokers total.

e. The trial is at unclear risk of performance/detection bias. We downrate this domain by -0.5.

f. Not applicable - single study.

g. No indirectness.

h. Unable to assess confidence intervals. Unable to assess optimal information size and sample size analyzed unclear but total of 555 total participants included. We cannot rate this domain.

i. Search strategy not completely comprehensive and study reports some positive findings, but lack of negative trials may be reflective of an under-researched area as opposed to suppression of findings. Given not enough information exists to strongly suspect publication bias, we do not downrate this domain.

## Appendix K Table 27. St John’s wort versus Placebo: Smoking cessation in smokers motivated/wishing to quit

| St John's wort compared to placebo in smokers motivated/wishing to quit  **Bibliography:** Howes 2020; Date of last search: May 2019 | | | | | | | | | | | |
| --- | --- | --- | --- | --- | --- | --- | --- | --- | --- | --- | --- |
| **Certainty assessment** | | | | | | | **Summary of findings** | | | | |
| **№ of participants (studies) Follow-up** | **Risk of bias** | **Inconsistency** | **Indirectness** | **Imprecision** | **Publication bias** | **Overall certainty of evidence** | **Study event rates (%)** | | **Relative effect (95% CI)** | **Anticipated absolute effects** | |
|  |  |  |  |  |  |  | **With placebo** | **With St John's wort** |  | **Risk with placebo** | **Risk difference with St John's wort** |
| **Abstinence/Cessation (follow up: 6 months)** ^a,b,c^  Outcome measurement: prolonged abstinence 100% studies  Biochemical validation: 100% studies | | | | | | | | | | | |
| 261 (2 RCTs) | not serious ^d^ | not serious ^e^ | not serious ^f^ | very serious ^g^ | none ^h^ | ⨁⨁◯◯  LOW | 6/111 (5.4%) | 6/150 (4.0%) | RR 0.81 (0.26 to 2.53) | 54 per 1,000 | **10 fewer per 1,000** (from 40 fewer to 83 more) |

**CI:** Confidence interval; **RR:** Risk ratio

#### Explanations

a. Both trials recruited general adult smokers motivated to quit.

b. One trial examined 900 mg/day of St John's wort for 14 weeks and the other examined 900 mg/day and 1800 mg/day for 12 weeks (arms collapsed in analysis).

c. Behavioural co-intervention provided to all arms in both trials.

d. Issues of bias are not substantive enough to warrant downrating.

e. Effect estimates vary but confidence intervals overlap (I2=29%, p=0.23). We do not downrate this domain.

f. No indirectness.

g. Confidence interval encompasses harm (moderate) to benefit (moderate). The optimal information size not met (total of 12 events) and inadequate sample size (<2000 participants). We downrate this domain by -2.0.

h. Search not completely comprehensive but trials report negative findings. We do not downrate this domain.

| Summary of findings: | | | | | | |
| --- | --- | --- | --- | --- | --- | --- |
| **St John's wort compared to placebo in smokers motivated/wishing to quit** | | | | | | |
| **Patient or population**: Smokers motivated/wishing to quit  **Setting**: No restriction  **Intervention**: St John's wort  **Comparison**: Placebo | | | | | | |
| Outcomes | **Anticipated absolute effects^*^** (95% CI) | | Relative effect (95% CI) | № of participants  (studies) | Certainty of the evidence (GRADE) | Comments |
|  | **Risk with placebo** | **Risk with St John's wort** |  |  |  |  |
| Abstinence/Cessation ^a,b,c^  Outcome measurement: prolonged abstinence 100% studies.  Biochemical validation: 100% studies  Follow up: 6 months | 54 per 1,000 | **44 per 1,000** (14 to 137) | RR 0.81 (0.26 to 2.53) | 261 (2 RCTs) | ⨁⨁◯◯  LOW ^d,e,f,g,h^ | Fixed effects.  AMSTAR-2: Critically low.  Date of last search: May 2019.  Not GRADEd by review authors. |
| ***The risk in the intervention group** (and its 95% confidence interval) is based on the assumed risk in the comparison group and the **relative effect** of the intervention (and its 95% CI).   **CI:** Confidence interval; **RR:** Risk ratio | | | | | | |
| **GRADE Working Group grades of evidence** **High certainty:** We are very confident that the true effect lies close to that of the estimate of the effect **Moderate certainty:** We are moderately confident in the effect estimate: The true effect is likely to be close to the estimate of the effect, but there is a possibility that it is substantially different **Low certainty:** Our confidence in the effect estimate is limited: The true effect may be substantially different from the estimate of the effect **Very low certainty:** We have very little confidence in the effect estimate: The true effect is likely to be substantially different from the estimate of effect | | | | | | |

#### Explanations

a. Both trials recruited general adult smokers motivated to quit.

b. One trial examined 900 mg/day of St John's wort for 14 weeks and the other examined 900 mg/day and 1800 mg/day for 12 weeks (arms collapsed in analysis).

c. Behavioural co-intervention provided to all arms in both trials.

d. Issues of bias are not substantive enough to warrant downrating. e. Effect estimates vary but confidence intervals overlap (I2=29%, p=0.23). We do not downrate this domain.

e. Effect estimates vary but confidence intervals overlap (I2=29%, p=0.23). We do not downrate this domain.

f. No indirectness.

g. Confidence interval encompasses harm (moderate) to benefit (moderate). The optimal information size not met (total of 12 events) and inadequate sample size (<2000 participants). We downrate this domain by -2.0.

h. Search not completely comprehensive but trials report negative findings. We do not downrate this domain.

## Appendix K Table 28. S-Adenosyl-L-Methionine (SAMe) versus Placebo: Smoking cessation in smokers motivated/wishing to quit

| S-Adenosyl-L-Methionine (SAMe) compared to placebo in smokers motivated/wishing to quit  **Bibliography:** Howes 2020; Date of last search: May 2019 | | | | | | | | | | | |
| --- | --- | --- | --- | --- | --- | --- | --- | --- | --- | --- | --- |
| **Certainty assessment** | | | | | | | **Summary of findings** | | | | |
| **№ of participants (studies) Follow-up** | **Risk of bias** | **Inconsistency** | **Indirectness** | **Imprecision** | **Publication bias** | **Overall certainty of evidence** | **Study event rates (%)** | | **Relative effect (95% CI)** | **Anticipated absolute effects** | |
|  |  |  |  |  |  |  | **With placebo** | **With S-Adenosyl-L-Methionine (SAMe)** |  | **Risk with placebo** | **Risk difference with S-Adenosyl-L-Methionine (SAMe)** |
| **Abstinence/Cessation (follow up: 6 months)** ^a,b,c^  Outcome measurement: point prevalence 100% studies  Biochemical validation: 100% studies | | | | | | | | | | | |
| 120 (1 RCT) | serious ^d^ | not serious ^e^ | not serious ^f^ | very serious ^g^ | none ^h^ | ⨁◯◯◯  VERY LOW | 5/40 (12.5%) | 7/80 (8.8%) | RR 0.70 (0.24 to 2.07) | 125 per 1,000 | **38 fewer per 1,000** (from 95 fewer to 134 more) |

**CI:** Confidence interval; **RR:** Risk ratio

#### Explanations

a. Trial recruited general adult smokers motivated to quit.

b. Trial examined both 800 mg/day and 1600 mg/day SAMe for 8 weeks (arms collapsed in analysis).

c. Behavioural co-intervention provided to all trial arms.

d. Trial at unclear risk for selection and performance/detection bias. We downrate this domain by -1.0.

e. Not applicable - single study.

f. No indirectness.

g. Confidence interval encompasses both harm (large) and benefit (large). The optimal information size not met (total of 12 events) and inadequate sample size (<2000 participants). We downrate this domain by -2.0.

h. Search not completely comprehensive but trial reports negative findings. We do not downrate this domain.

| Summary of findings: | | | | | | |
| --- | --- | --- | --- | --- | --- | --- |
| **S-Adenosyl-L-Methionine (SAMe) compared to placebo in smokers motivated/wishing to quit** | | | | | | |
| **Patient or population**: Smokers motivated/wishing to quit  **Setting**: No restriction  **Intervention**: S-Adenosyl-L-Methionine (SAMe)  **Comparison**: Placebo | | | | | | |
| Outcomes | **Anticipated absolute effects^*^** (95% CI) | | Relative effect (95% CI) | № of participants  (studies) | Certainty of the evidence (GRADE) | Comments |
|  | **Risk with placebo** | **Risk with S-Adenosyl-L-Methionine (SAMe)** |  |  |  |  |
| Abstinence/Cessation ^a,b,c^  Outcome measurement: point prevalence 100% studies.  Biochemical validation: 100% studies  Follow up: 6 months | 125 per 1,000 | **88 per 1,000** (30 to 259) | RR 0.70 (0.24 to 2.07) | 120 (1 RCT) | ⨁◯◯◯  VERY LOW ^d,e,f,g,h^ | AMSTAR-2 rating: Critically low.  Date of last search: May 2019.  Not GRADEd by review authors. |
| ***The risk in the intervention group** (and its 95% confidence interval) is based on the assumed risk in the comparison group and the **relative effect** of the intervention (and its 95% CI).   **CI:** Confidence interval; **RR:** Risk ratio | | | | | | |
| **GRADE Working Group grades of evidence** **High certainty:** We are very confident that the true effect lies close to that of the estimate of the effect **Moderate certainty:** We are moderately confident in the effect estimate: The true effect is likely to be close to the estimate of the effect, but there is a possibility that it is substantially different **Low certainty:** Our confidence in the effect estimate is limited: The true effect may be substantially different from the estimate of the effect **Very low certainty:** We have very little confidence in the effect estimate: The true effect is likely to be substantially different from the estimate of effect | | | | | | |

#### Explanations

a. Trial recruited general adult smokers motivated to quit.

b. Trial examined both 800 mg/day and 1600 mg/day SAMe for 8 weeks (arms collapsed in analysis).

c. Behavioural co-intervention provided to all trial arms.

d. Trial at unclear risk for selection and performance/detection bias. We downrate this domain by -1.0.

e. Not applicable - single study.

f. No indirectness.

g. Confidence interval encompasses both harm (large) and benefit (large). The optimal information size not met (total of 12 events) and inadequate sample size (<2000 participants). We downrate this domain by -2.0.

h. Search not completely comprehensive but trial reports negative findings. We do not downrate this domain.

# Lancaster 2017 {539}

## Appendix K Table 29. Individual counselling versus Minimal contact control: Smoking cessation in general/mixed population of smokers

| Individual counselling (no systematic pharmacotherapy) compared to minimal contact control (no systematic pharmacotherapy) in general/mixed population of smokers  **Bibliography:** Lancaster 2017; Date of last search: May 2016 | | | | | | | | | | | |
| --- | --- | --- | --- | --- | --- | --- | --- | --- | --- | --- | --- |
| **Certainty assessment** | | | | | | | **Summary of findings** | | | | |
| **№ of participants (studies) Follow-up** | **Risk of bias** | **Inconsistency** | **Indirectness** | **Imprecision** | **Publication bias** | **Overall certainty of evidence** | **Study event rates (%)** | | **Relative effect (95% CI)** | **Anticipated absolute effects** | |
|  |  |  |  |  |  |  | **With minimal contact control (no systematic pharmacotherapy)** | **With Individual counselling (no systematic pharmacotherapy)** |  | **Risk with minimal contact control (no systematic pharmacotherapy)** | **Risk difference with Individual counselling (no systematic pharmacotherapy)** |
| **Abstinence/cessation (follow up: 6+ months)** ^a,b,c^  Outcome measurement: point prevalence 41%, continuous/sustained abstinence 26%, NR 33% studies  Biochemical validation: 63% studies | | | | | | | | | | | |
| 11100 (27 RCTs) | not serious ^d^ | serious ^e^ | very serious ^f^ | not serious ^g^ | none ^h^ | ⨁◯◯◯  VERY LOW | 392/5581 (7.0%) | 604/5519 (10.9%) | RR 1.57 (1.40 to 1.77) | 70 per 1,000 | **40 more per 1,000** (from 28 more to 54 more) |

**CI:** Confidence interval; **NR:** Not reported; **RR:** Risk ratio

#### Explanations

a. Trials recruited general smokers (n=7), inpatients awaiting surgery or with various co-morbidities (n=9), smokers with co-morbidities (e.g., diabetes, hypertension/hypercholestrolaemia, cardiovascular disease) (n=6), smokers with substance use disorder (n=1), male veteran smokers (n=1), female smokers (n=1), postpartum smokers (n=1) and Aboriginal and Torres Strait Islander smokers (n=1).

b. Intervention: Individual counseling defined as at least one session of 10 or more minutes duration. The review restricted inclusion to counselling delivered by a smoking cessation specialist outside of routine clinical care. Additional behavioural and/or ‘other’ (e.g., computer-guided nicotine fading with contingent contract, cigarette substitute) co-interventions provided in majority of studies. Although this analysis excludes trials in which pharmacotherapy was offered to participants in all trial arms, it appears a few trials did offer NRT or a prescription for NRT to those in the intervention arm. Approximately 37.5% of intervention participants received specialized behavioural counselling (e.g., cognitive behavioural therapy, motivational interviewing, stage-based counselling, 5As).

c. Control: Minimal contact control was usual care or brief advice (up to 15 min) with/without self-help materials. Some trials provided additional behavioural (e.g., advice/counselling on pharmacotherapy) or ‘other’ (e.g., monetary rewards for cessation) co-interventions. NRT was made available to control participants in three trials. No control participants received specialized behavioural counselling.

d. Performance bias not assessed by review authors; they don't consider trials to be at high risk for this domain. Therefore, we consider trials to be at low risk for performance bias. 46% of the evidence at unclear risk of bias for one domain (mostly selection bias); we downrate this domain by -0.5. Evidence at greater risk of bias is not substantive enough for further downrating.

e. Point estimates vary and not all confidence intervals overlap (I2=50%, p=0.002). We downrate this domain by -1.0.

f. 88.2% of evidence is indirect, mainly due to setting (e.g., inpatient, specialized medical); we downrate this domain by -2.0. Due to reporting, extent of indirectness could not be determined for one trial that recruited from both community and workplace settings.

g. Confidence interval encompasses small but important benefit. The optimal information size is met (total of 996 events) and adequate sample size. We do not downrate this domain.

h. Although search not completely comprehensive, a mix of results observed. We do not downrate this domain.

| Summary of findings: | | | | | | |
| --- | --- | --- | --- | --- | --- | --- |
| **Individual counselling (no systematic pharmacotherapy) compared to minimal contact control (no systematic pharmacotherapy) in general/mixed population of smokers** | | | | | | |
| **Patient or population**: General/mixed population of smokers  **Setting**: No setting restriction. Review authors restrict inclusion to counselling delivered by a smoking cessation specialist outside of routine clinical care.  **Intervention**: Individual counselling (no systematic pharmacotherapy)  **Comparison**: Minimal contact control (no systematic pharmacotherapy) | | | | | | |
| Outcomes | **Anticipated absolute effects^*^** (95% CI) | | Relative effect (95% CI) | № of participants  (studies) | Certainty of the evidence (GRADE) | Comments |
|  | **Risk with minimal contact control (no systematic pharmacotherapy)** | **Risk with Individual counselling (no systematic pharmacotherapy)** |  |  |  |  |
| Abstinence/cessation ^a,b,c^  Outcome measurement: point prevalence 41%, continuous/sustained abstinence 26%, NR 33% studies  Biochemical validation: 63% studies  Follow up: 6+ months | 70 per 1,000 | **110 per 1,000** (98 to 124) | RR 1.57 (1.40 to 1.77) | 11100 (27 RCTs) | ⨁⨁◯◯  LOW ^d,e,f,g,h^ | Authors performed as a subgroup analysis; may be plausible but some doubt remains.  Fixed effects model.  AMSTAR-2: Critically low.  Date of last search: May 2016.  Review authors rated high certainty. |
| ***The risk in the intervention group** (and its 95% confidence interval) is based on the assumed risk in the comparison group and the **relative effect** of the intervention (and its 95% CI).   **CI:** Confidence interval; **NR:** Not reported; **RR:** Risk ratio | | | | | | |
| **GRADE Working Group grades of evidence** **High certainty:** We are very confident that the true effect lies close to that of the estimate of the effect **Moderate certainty:** We are moderately confident in the effect estimate: The true effect is likely to be close to the estimate of the effect, but there is a possibility that it is substantially different **Low certainty:** Our confidence in the effect estimate is limited: The true effect may be substantially different from the estimate of the effect **Very low certainty:** We have very little confidence in the effect estimate: The true effect is likely to be substantially different from the estimate of effect | | | | | | |

#### Explanations

a. Trials recruited general smokers (n=7), inpatients awaiting surgery or with various co-morbidities (n=9), smokers with co-morbidities (e.g., diabetes, hypertension/hypercholestrolaemia, cardiovascular disease) (n=6), smokers with substance use disorder (n=1), male veteran smokers (n=1), female smokers (n=1), postpartum smokers (n=1) and Aboriginal and Torres Strait Islander smokers (n=1).

b. Intervention: Individual counseling defined as at least one session of 10 or more minutes duration. The review restricted inclusion to counselling delivered by a smoking cessation specialist outside of routine clinical care. Additional behavioural and/or ‘other’ (e.g., computer-guided nicotine fading with contingent contract, cigarette substitute) co-interventions provided in majority of studies. Although this analysis excludes trials in which pharmacotherapy was offered to participants in all trial arms, it appears a few trials did offer NRT or a prescription for NRT to those in the intervention arm. Approximately 37.5% of intervention participants received specialized behavioural counselling (e.g., cognitive behavioural therapy, motivational interviewing, stage-based counselling, 5As).

c. Control: Minimal contact control was usual care or brief advice (up to 15 min) with/without self-help materials. Some trials provided additional behavioural (e.g., advice/counselling on pharmacotherapy) or ‘other’ (e.g., monetary rewards for cessation) co-interventions. NRT was made available to control participants in three trials. No control participants received specialized behavioural counselling.

d. Performance bias not assessed by review authors; they don't consider trials to be at high risk for this domain. Therefore, we consider trials to be at low risk for performance bias. 46% of the evidence at unclear risk of bias for one domain (mostly selection bias); we downrate this domain by -0.5. Evidence at greater risk of bias is not substantive enough for further downrating.

e. Point estimates vary and not all confidence intervals overlap (I2=50%, p=0.002). We downrate this domain by -1.0.

f. 88.2% of evidence is indirect, mainly due to setting (e.g., inpatient, specialized medical); we downrate this domain by -2.0. Due to reporting, extent of indirectness could not be determined for one trial that recruited from both community and workplace settings.

g. Confidence interval encompasses small but important benefit. The optimal information size is met (total of 996 events) and adequate sample size. We do not downrate this domain.

h. Although search not completely comprehensive, a mix of results observed. We do not downrate this domain.

# Lindson-Hawley 2016 {671}

## Appendix K Table 30. NRT versus Placebo: Smoking cessation and reduction in smokers not motivated/wishing to quit

| NRT compared to placebo in smokers not motivated/wishing to quit  **Bibliography:** Lindson-Hawley 2016; Date of last se arch: October 2015 | | | | | | | | | | | |
| --- | --- | --- | --- | --- | --- | --- | --- | --- | --- | --- | --- |
| **Certainty assessment** | | | | | | | **Summary of findings** | | | | |
| **№ of participants (studies) Follow-up** | **Risk of bias** | **Inconsistency** | **Indirectness** | **Imprecision** | **Publication bias** | **Overall certainty of evidence** | **Study event rates (%)** | | **Relative effect (95% CI)** | **Anticipated absolute effects** | |
|  |  |  |  |  |  |  | **With placebo** | **With NRT** |  | **Risk with placebo** | **Risk difference with NRT** |
| **Abstinence/cessation (follow up: range 12 months to 24 months)** ^a,b,c,d^  Outcome measurement: point prevalence 62.5%, continuous/sustained abstinence 25.0%, unclear/NR 12.5% studies  Biochemical validation: 87.5% | | | | | | | | | | | |
| 3081 (8 RCTs) | serious ^e^ | not serious ^f^ | not serious ^g^ | not serious ^h^ | none ^i^ | ⨁⨁⨁◯  MODERATE | 75/1488 (5.0%) | 159/1593 (10.0%) | RR 1.87 (1.43 to 2.44) | 50 per 1,000 | **44 more per 1,000** (from 22 more to 73 more) |
| **Reduction in cigarettes/day of >50% of baseline or cessation (follow up: 12+ months)** ^a,b,c,j^  Outcome measurement: continuous/sustained reduction 82.5%, unclear/NR 12.5% studies  Biochemical validation: 62.5% studies ^k^ | | | | | | | | | | | |
| 3081 (8 RCTs) | serious ^l^ | not serious ^m^ | not serious ^n^ | not serious ^o^ | none ^i^ | ⨁⨁⨁◯  MODERATE | 120/1488 (8.1%) | 250/1593 (15.7%) | RR 1.75 (1.44 to 2.13) | 81 per 1,000 | **60 more per 1,000** (from 35 more to 91 more) |

**CI:** Confidence interval; **NR:** Not reported; **NRT:** Nicotine replacement therapy; **RR:** Risk ratio

#### Explanations

a. Review eligibility criterion is people who smoke tobacco but have no immediate intention to quit all tobacco use. Four trials recruited participants not motivated/wishing to quit, three trials those not motivated/wishing to quit with at least one failed quit attempt, and one trial those with at least one failed quit attempt where motivation to quit was not required for enrollment.

b. NRT: Intervention used to assist smoking reduction. Type of NRT: Inhaler (n=2), gum (n=4), choice of NRT type (n=2). Inhaler: Participants in one trial used 6 to 12 cartridges daily for 4 months and were instructed to decrease thereafter (usage allowed up to 18 months). The second study provided 10 mg ad lib with recommendations to use 6 to 12 cartridges daily for up to 12 months (cessation encouraged after 6 months). NRT gum: Two trials provided either 2mg or 4mg gum according to dependence for 4 or 12 months, one trial provided only 4mg gum (6 to 24 pieces/day) for up to 12 months, and remaining trial provided gum for up to 9 months (dose not specified). Choice of NRT: One trial offered choice of 4mg gum (up to 24 pieces per day) or 10mg inhaler (6 to 12 cartridges) for up to 6 months with an additional 3 months of tapering. The second trial provided choice of patch, 4mg gum, inhaler or combination for 6 months. Behavioural co-intervention provided in most trials.

c. Placebo: Behavioural co-intervention provided in most trials.

d. For smoking cessation, authors preferred point prevalence over sustained/continuous abstinence as participants were not expected to quit at start of intervention.

e. 46% of the evidence is at unclear risk of bias for two or more domains, namely, selection, performance/detection, and attrition bias; therefore, we downrate this domain by -1.0. Evidence at high risk of bias (32%) not substantive enough for further downrating.

f. Some variation in point estimates but confidence intervals mostly overlap (I2=30%, p=0.19). We do not downrate this domain.

g. 22% of the evidence is indirect due to setting (i.e., medical specialist). We downrate this domain by -0.5.

h. Confidence interval encompasses one range of effect (small but important benefit to moderate benefit). The optimal information size not met (total of 234 events), but adequate sample size (>2000 participants). We did not downrate this domain.

i. Although search not completely comprehensive, a mix of results observed across trials. We do not downrate this domain.

j. For smoking reduction, review authors preferred sustained/continuous rates over point prevalence.

k. Biochemical validation of self-reported CPD reduction is unclear in two trials. In one trial, the outcome was validated at short-term follow-up but unclear at 24 months. In the other trial, while CO levels were measured as an outcome of the study, it is unclear whether reduced levels of CO at follow-up was used to validate reduction in CPD.

l. 44% of the evidence is at unclear risk of bias for two or more domains, namely, selection bias plus performance/detection bias, attrition bias, or biochemical validation. 45% of the evidence is at high risk of bias as biochemical validation of self-reported CPD was not carried out. We downrate this domain by -1.0.

m. Risk estimates vary but confidence intervals mostly overlap (I2=45%, p=0.08). We downrate this domain by -0.5.

n. 5% of the evidence is indirect due to setting (i.e., medical specialist). We do not downrate this domain.

o. Confidence interval encompasses small but important benefit. The optimal information size not met (total of 234 events), but adequate sample size (>2000 participants). We did not downrate this domain.

| Summary of findings: | | | | | | |
| --- | --- | --- | --- | --- | --- | --- |
| **NRT compared to placebo in smokers not motivated/wishing to quit** | | | | | | |
| **Patient or population**: Smokers not motivated/wishing to quit  **Setting**: No restriction  **Intervention**: NRT  **Comparison**: Placebo | | | | | | |
| Outcomes | **Anticipated absolute effects^*^** (95% CI) | | Relative effect (95% CI) | № of participants  (studies) | Certainty of the evidence (GRADE) | Comments |
|  | **Risk with placebo** | **Risk with NRT** |  |  |  |  |
| Abstinence/cessation ^a,b,c,d^  Outcome measurement: point prevalence 62.5%, continuous/sustained abstinence 25.0%, unclear/NR 12.5% studies  Biochemical validation: 87.5%  Follow up: range 12 months to 24 months | 50 per 1,000 | **94 per 1,000** (72 to 123) | RR 1.87 (1.43 to 2.44) | 3081 (8 RCTs) | ⨁⨁⨁◯  MODERATE ^e,f,g,h,i^ | Fixed effects analysis.  Date of last search: October 2015.  AMSTAR-2: Critically low.  Review authors rated certainty as low. ^j^ |
| Reduction in cigarettes/day of >50% of baseline or cessation ^a,b,c,k^  Outcome measurement: continuous/sustained reduction 82.5%, unclear/NR 12.5% studies  Biochemical validation: 62.5% studies ^l^  Follow up: 12+ months | 81 per 1,000 | **141 per 1,000** (116 to 172) | RR 1.75 (1.44 to 2.13) | 3081 (8 RCTs) | ⨁⨁⨁◯  MODERATE ^i,m,n,o,p^ | Heterogeneity reduced when two trials contributing the most weight were removed from the analysis (RR 2.77, 95% CI 1.88 to 4.08, I² = 9%; n=2233). Of the trials removed, one included participants who were more motivated to quit than reduce and the other trial did not biochemically validate reduction.  Fixed effects analysis.  Date of last search: October 2015.  AMSTAR-2: Critically low.  Not GRADEd by review authors. |
| ***The risk in the intervention group** (and its 95% confidence interval) is based on the assumed risk in the comparison group and the **relative effect** of the intervention (and its 95% CI).   **CI:** Confidence interval; **CO:** Carbon monoxide; **CPD:** Cigarettes per day; **NR:** Not reported; **NRT:** Nicotine replacement therapy; **RR:** Risk ratio | | | | | | |
| **GRADE Working Group grades of evidence** **High certainty:** We are very confident that the true effect lies close to that of the estimate of the effect **Moderate certainty:** We are moderately confident in the effect estimate: The true effect is likely to be close to the estimate of the effect, but there is a possibility that it is substantially different **Low certainty:** Our confidence in the effect estimate is limited: The true effect may be substantially different from the estimate of the effect **Very low certainty:** We have very little confidence in the effect estimate: The true effect is likely to be substantially different from the estimate of effect | | | | | | |

#### Explanations

a. Review eligibility criterion is people who smoke tobacco but have no immediate intention to quit all tobacco use. Four trials recruited participants not motivated/wishing to quit, three trials those not motivated/wishing to quit with at least one failed quit attempt, and one trial those with at least one failed quit attempt where motivation to quit was not required for enrollment.

b. NRT: Intervention used to assist smoking reduction. Type of NRT: Inhaler (n=2), gum (n=4), choice of NRT type (n=2). Inhaler: Participants in one trial used 6 to 12 cartridges daily for 4 months and were instructed to decrease thereafter (usage allowed up to 18 months). The second study provided 10 mg ad lib with recommendations to use 6 to 12 cartridges daily for up to 12 months (cessation encouraged after 6 months). NRT gum: Two trials provided either 2mg or 4mg gum according to dependence for 4 or 12 months, one trial provided only 4mg gum (6 to 24 pieces/day) for up to 12 months, and remaining trial provided gum for up to 9 months (dose not specified). Choice of NRT: One trial offered choice of 4mg gum (up to 24 pieces per day) or 10mg inhaler (6 to 12 cartridges) for up to 6 months with an additional 3 months of tapering. The second trial provided choice of patch, 4mg gum, inhaler or combination for 6 months. Behavioural co-intervention provided in most trials.

c. Placebo: Behavioural co-intervention provided in most trials.

d. For smoking cessation, authors preferred point prevalence over sustained/continuous abstinence as participants were not expected to quit at start of intervention.

e. 46% of the evidence is at unclear risk of bias for two or more domains, namely, selection, performance/detection, and attrition bias; we downrate this domain by -1.0. Evidence at high risk of bias (32%) not substantive enough for further downrating.

f. Some variation in point estimates but confidence intervals mostly overlap (I2=30%, p=0.19). We do not downrate this domain.

g. 22% of the evidence is indirect due to setting (i.e., medical specialist). We downrate this domain by -0.5.

h. Confidence interval encompasses one range of effect (small but important benefit to moderate benefit). The optimal information size not met (total of 234 events), but adequate sample size (>2000 participants). We did not downrate this domain.

i. Although search not completely comprehensive, a mix of results observed across trials. We do not downrate this domain.

j. Review authors rate certainty of evidence as low (double downrated imprecision domain due to small number of events and wide confidence intervals).

k. For smoking reduction, review authors preferred sustained/continuous rates over point prevalence.

l. Biochemical validation of self-reported CPD reduction is unclear in two trials. In one trial, the outcome was validated at short-term follow-up but unclear at 24 months. In the other trial, while CO levels were measured as an outcome of the study, it is unclear whether reduced levels of CO at follow-up was used to validate reduction in CPD.

m. 44% of the evidence is at unclear risk of bias for two or more domains, namely, selection bias plus performance/detection bias, attrition bias, or biochemical validation. 45% of the evidence is at high risk of bias as biochemical validation of self-reported CPD was not carried out. We downrate this domain by -1.0.

n. Risk estimates vary but confidence intervals mostly overlap (I2=45%, p=0.08). We downrate this domain by -0.5.

o. 5% of the evidence is indirect due to setting (i.e., medical specialist). We do not downrate this domain.

p. Confidence interval encompasses small but important benefit. The optimal information size not met (total of 234 events), but adequate sample size (>2000 participants). We did not downrate this domain.

## Appendix K Table 31. Bupropion versus Placebo: Smoking cessation, reduction, and serious adverse events in smokers not motivated/wishing to quit

| Bupropion compared to placebo in smokers not motivated/wishing to quit  **Bibliography:** Lindson-Hawley 2016; Date of last search: October 2015 | | | | | | | | | | | |
| --- | --- | --- | --- | --- | --- | --- | --- | --- | --- | --- | --- |
| **Certainty assessment** | | | | | | | **Summary of findings** | | | | |
| **№ of participants (studies) Follow-up** | **Risk of bias** | **Inconsistency** | **Indirectness** | **Imprecision** | **Publication bias** | **Overall certainty of evidence** | **Study event rates (%)** | | **Relative effect (95% CI)** | **Anticipated absolute effects** | |
|  |  |  |  |  |  |  | **With placebo** | **With Bupropion** |  | **Risk with placebo** | **Risk difference with Bupropion** |
| **Abstinence/Cessation (follow up: 6 months)** ^a,b,c,d^  Outcome measurement: unclear/NR  Biochemical validation: 100% studies | | | | | | | | | | | |
| 594 (1 RCT) | serious ^e^ | not serious ^f^ | not serious ^g^ | very serious ^h^ | none ^i^ | ⨁◯◯◯  VERY LOW | 16/299 (5.4%) | 20/295 (6.8%) | RR 1.27 (0.67 to 2.40) | 54 per 1,000 | **14 more per 1,000** (from 18 fewer to 75 more) |
| **Reduction in cigarettes/day of >50% of baseline or cessation (follow up: 12 months)** ^a,b,c,j^  Outcome measurement: unclear/NR  Biochemical validation: unclear/NR ^k^ | | | | | | | | | | | |
| 594 (1 RCT) | serious ^l^ | not serious ^f^ | not serious ^g^ | very serious ^m^ | none ^i^ | ⨁◯◯◯  VERY LOW | 28/299 (9.4%) | 28/295 (9.5%) | RR 1.01 (0.62 to 1.67) | 94 per 1,000 | **1 more per 1,000** (from 36 fewer to 63 more) |
| **Reduction in cotinine >50% (follow up: 12 months)** ^a,b,c,j^  Outcome measurement: unclear/NR | | | | | | | | | | | |
| 327 ^n^ (1 RCT) | serious ^e^ | not serious ^f^ | not serious ^g^ | very serious ^o^ | none ^i^ | ⨁◯◯◯  VERY LOW | 8/174 (4.6%) ^n^ | 3/153 (2.0%) ^n^ | RR 0.43 (0.12 to 1.58) | 46 per 1,000 ^n^ | **26 fewer per 1,000** (from 40 fewer to 27 more) |
| **Reduction in cotinine (follow up: 12 months)** ^a,b,c^  Mean reduction in cotinine from baseline | | | | | | | | | | | |
| Unclear/NR ^p^ (1 RCT) | serious ^e^ | not serious ^f^ | not serious ^g^ | serious to very serious ^q^ | none ^i^ | unable to assess | No significant difference between groups in mean urinary cotinine from baseline at 12-month follow-up (mean decrease: bupropion 82 ng/mL vs control 28 ng/mL, p=0.25). | | | | |
| **Serious adverse events (follow up: Unclear/NR)** ^a,b,c^ | | | | | | | | | | | |
| 594 (1 RCT) | serious ^e^ | not serious ^f^ | not serious ^g^ | very serious ^r^ | none ^i^ | ⨁◯◯◯  VERY LOW | 3/299 (1.0%) | 8/295 (2.7%) | RR 2.70 (0.72 to 10.09) | 10 per 1,000 | **17 more per 1,000** (from 3 fewer to 91 more) |

**CI:** Confidence interval; **CPD:** Cigarettes per day; **NR:** Not reported; **NRT:** Nicotine replacement therapy; **RR:** Risk ratio

#### Explanations

a. Review eligibility criterion is people who smoke tobacco but have no immediate intention to quit all tobacco use. The only trial in this analysis recruited smokers interested in reducing smoking and with at least two failed quit attempts one of which with NRT. Those who became willing to quit entered the cessation phase of the trial which included weekly counselling for 7 weeks and then 19 weeks of follow-up.

b. Bupropion: Intervention used to assist smoking reduction. 300 mg/day for 26 weeks. Behavioural co-intervention provided.

c. Placebo: Behavioural co-intervention provided.

d. For smoking cessation, authors preferred point prevalence over sustained/continuous abstinence as participants were not expected to quit at start of intervention. However, it is unclear what measure of cessation was reported in the only trial included in this analysis.

e. The only trial included in this analysis is at unclear risk for selection bias and performance/detection bias. We downrate this domain by -1.0.

f. Not applicable (single trial).

g. No indirectness.

h. Confidence interval encompasses both harm (small but important) and benefit (moderate). The optimal information size not met (total of 36 events) and inadequate sample size (<2000 participants). We downrate this domain by -2.0.

i. Although search not completely comprehensive, the only trial in this analysis reports negative findings. We do not downrate this domain.

j. For smoking reduction outcomes, review authors preferred sustained/continuous rates over point prevalence. However, unclear which was reported for this particular analysis.

k. Urine cotinine levels were measured in the trial and reported as a separate outcome. However, is unclear whether reduced levels of cotinine at follow-up was used to validate self-reported reduction in CPD.

l. The only trial included in this analysis is at unclear risk for selection bias and performance/detection bias. It is also unclear whether this outcome was biochemically validated. We downrate this domain by -1.0.

m. Confidence interval encompasses both harm (small but important) and benefit (small but important). The optimal information size not met (total of 56 events) and inadequate sample size (<2000 participants). We downrate this domain by -2.0.

n. This analysis excludes those who became willing to quit and entered the cessation phase of the trial.

o. Confidence interval encompasses both harm (small but important) and benefit (small but important). The optimal information size not met (total of 11 events) and inadequate sample size (<2000 participants). We downrate this domain by -2.0.

p. Number of participants included in this analysis is unclear. In particular, unclear whether those who became willing to quit were included in this analysis.

q. Unable to assess confidence intervals. Unable to assess optimal information size and sample size analyzed not reported. We cannot rate this domain.

r. Confidence interval encompasses three ranges of effect (little to no difference to large harm). The optimal information size not met (total of 11 events) and inadequate sample size (<2000 participants). We downrate this domain by -2.0.

| Summary of findings: | | | | | | |
| --- | --- | --- | --- | --- | --- | --- |
| **Bupropion compared to placebo in smokers not motivated/wishing to quit** | | | | | | |
| **Patient or population**: Smokers not motivated/wishing to quit  **Setting**: No restriction  **Intervention**: Bupropion  **Comparison**: Placebo | | | | | | |
| Outcomes | **Anticipated absolute effects^*^** (95% CI) | | Relative effect (95% CI) | № of participants  (studies) | Certainty of the evidence (GRADE) | Comments |
|  | **Risk with placebo** | **Risk with Bupropion** |  |  |  |  |
| Abstinence/Cessation ^a,b,c,d^  Outcome measurement: unclear/NR  Biochemical validation: 100% studies  Follow up: 6 months | 54 per 1,000 | **68 per 1,000** (36 to 128) | RR 1.27 (0.67 to 2.40) | 594 (1 RCT) | ⨁◯◯◯  VERY LOW ^e,f,g,h,i^ | Data also available in Hughes 2014. Note that rating of attrition bias by review authors differs across reviews resulting in different ROB domain ratings.  Date of last search: October 2015.  AMSTAR-2: Critically low.  Review authors rated certainty as low. ^j^ |
| Reduction in cigarettes/day of >50% of baseline or cessation ^a,b,c,k^  Outcome measurement: unclear/NR  Biochemical validation: unclear/NR ^l^  Follow up: 12 months | 94 per 1,000 | **95 per 1,000** (58 to 156) | RR 1.01 (0.62 to 1.67) | 594 (1 RCT) | ⨁◯◯◯  VERY LOW ^f,g,i,m,n^ | Date of last search: October 2015.  AMSTAR-2: Critically low.  Not GRADEd by review authors. |
| Reduction in cotinine >50% ^a,b,c,k^  Outcome measurement: unclear/NR  Follow up: 12 months | 46 per 1,000 ^o^ | **20 per 1,000** (6 to 73) ^o^ | RR 0.43 (0.12 to 1.58) | 327 ^o^ (1 RCT) | ⨁◯◯◯  VERY LOW ^e,f,g,i,p^ | Data also available in Hughes 2014. Note that rating of attrition bias by review authors differs across reviews resulting in different ROB domain ratings.  Date of last search: October 2015.  AMSTAR-2: Critically low.  Not GRADEd by review authors. |
| Reduction in cotinine ^a,b,c^  Mean reduction in cotinine from baseline  Follow up: 12 months | No significant difference between groups in mean urinary cotinine from baseline at 12-month follow-up (mean decrease: bupropion 82 ng/ML vs control 28 ng/mL, p=0.25). | |  | Unclear/NR ^q^  (1 RCT) | unable to assess ^e,f,g,i,r^ | Date of last search: October 2015.  AMSTAR-2: Critically low.  Not GRADEd by review authors. |
| Serious adverse events ^a,b,c^  Follow up: Unclear/NR | 10 per 1,000 | **27 per 1,000** (7 to 101) | RR 2.70 (0.72 to 10.09) | 594 (1 RCT) | ⨁◯◯◯  VERY LOW ^e,f,g,i,s^ | One of the serious adverse events potentially attributed to bupropion intervention.  Date of last search: October 2015.  AMSTAR-2: Critically low.  Not GRADEd by review authors. |
| ***The risk in the intervention group** (and its 95% confidence interval) is based on the assumed risk in the comparison group and the **relative effect** of the intervention (and its 95% CI).   **CI:** Confidence interval; **CPD:** Cigarettes per day; **NR:** Not reported; **NRT:** Nicotine replacement therapy; **RR:** Risk ratio | | | | | | |
| **GRADE Working Group grades of evidence** **High certainty:** We are very confident that the true effect lies close to that of the estimate of the effect **Moderate certainty:** We are moderately confident in the effect estimate: The true effect is likely to be close to the estimate of the effect, but there is a possibility that it is substantially different **Low certainty:** Our confidence in the effect estimate is limited: The true effect may be substantially different from the estimate of the effect **Very low certainty:** We have very little confidence in the effect estimate: The true effect is likely to be substantially different from the estimate of effect | | | | | | |

#### Explanations

a. Review eligibility criterion is people who smoke tobacco but have no immediate intention to quit all tobacco use. The only trial in this analysis recruited smokers interested in reducing smoking and with at least two failed quit attempts one of which with NRT. Those who became willing to quit entered the cessation phase of the trial which included weekly counselling for 7 weeks and then 19 weeks of follow-up.

b. Bupropion: Intervention used to assist smoking reduction. 300 mg/day for 26 weeks. Behavioural co-intervention provided.

c. Placebo: Behavioural co-intervention provided.

d. For smoking cessation, authors preferred point prevalence over sustained/continuous abstinence as participants were not expected to quit at start of intervention. However, it is unclear what measure of cessation was reported in the only trial included in this analysis.

e. The only trial included in this analysis is at unclear risk for selection bias and performance/detection bias. We downrate this domain by -1.0.

f. Not applicable (single trial).

g. No indirectness.

h. Confidence interval encompasses both harm (small but important) and benefit (moderate). The optimal information size not met (total of 36 events) and inadequate sample size (<2000 participants). We downrate this domain by -2.0.

i. Although search not completely comprehensive, the only trial in this analysis reports negative findings. We do not downrate this domain.

j. Review authors rate certainty of evidence as low (double downrated imprecision domain due to small number of events and small number of studies).

k. For smoking reduction outcomes, review authors preferred sustained/continuous rates over point prevalence. However, unclear which was reported for this particular analysis.

l. Urine cotinine levels were measured in the trial and reported as a separate outcome. However, is unclear whether reduced levels of cotinine at follow-up was used to validate self-reported reduction in CPD.

m. The only trial included in this analysis is at unclear risk for selection bias and performance/detection bias. It is also unclear whether this outcome was biochemically validated. We downrate this domain by -1.0.

n. Confidence interval encompasses both harm (small but important) and benefit (small but important). The optimal information size not met (total of 56 events) and inadequate sample size (<2000 participants). We downrate this domain by -2.0.

o. This analysis excludes those who became willing to quit and entered the cessation phase of the trial.

p. Confidence interval encompasses both harm (small but important) and benefit (small but important). The optimal information size not met (total of 11 events) and inadequate sample size (<2000 participants). We downrate this domain by -2.0.

q. Number of participants included in this analysis is unclear. In particular, unclear whether those who became willing to quit were included in this analysis.

r. Unable to assess confidence intervals. Unable to assess optimal information size and sample size analyzed not reported. We cannot rate this domain.

s. Confidence interval encompasses three ranges of effect (little to no difference to large harm). The optimal information size not met (total of 11 events) and inadequate sample size (<2000 participants). We downrate this domain by -2.0.

## Appendix K Table 32. Varenicline versus Placebo: Smoking cessation and adverse events in smokers not motivated/wishing to quit

| Varenicline compared to placebo in smokers not motivated/wishing to quit  **Bibliography:** Lindson-Hawley 2016; Date of last search: October 2015 | | | | | | | | | | | |
| --- | --- | --- | --- | --- | --- | --- | --- | --- | --- | --- | --- |
| **Certainty assessment** | | | | | | | **Summary of findings** | | | | |
| **№ of participants (studies) Follow-up** | **Risk of bias** | **Inconsistency** | **Indirectness** | **Imprecision** | **Publication bias** | **Overall certainty of evidence** | **Study event rates (%)** | | **Relative effect (95% CI)** | **Anticipated absolute effects** | |
|  |  |  |  |  |  |  | **With placebo** | **With Varenicline** |  | **Risk with placebo** | **Risk difference with Varenicline** |
| **Abstinence/cessation (follow up: 6 months)** ^a,b,c,d^  Outcome measurement: point prevalence 100% studies  Biochemical validation: 100% studies | | | | | | | | | | | |
| 218 (1 RCT) | serious ^e^ | not serious ^f^ | not serious ^g^ | very serious ^h^ | none ^i^ | ⨁◯◯◯  VERY LOW | 8/111 (7.2%) | 15/107 (14.0%) | RR 1.95 (0.86 to 4.40) | 72 per 1,000 | **68 more per 1,000** (from 10 fewer to 245 more) |
| **Adverse events (stopping medication due to adverse event) (follow up: Unclear/NR)** ^a,b,c^ | | | | | | | | | | | |
| Unclear/NR ^j^ (1 RCT) | serious ^e^ | not serious ^f^ | not serious ^g^ | not serious to very serious ^k^ | none ^i^ | unable to assess | No significant difference between groups (varenicline 12%, placebo 10%). | | | | |

**CI:** Confidence interval; **NR:** Not reported; **RR:** Risk ratio

#### Explanations

a. Review eligibility criterion is people who smoke tobacco but have no immediate intention to quit all tobacco use. The only trial in this analysis recruited general smokers who wished to eventually quit but had no immediate intention to do so.

b. Varenicline: Objective of trial was to evaluate effect of varenicline on inducing quit attempts in smokers not planning to quit. 2 mg/day for 2 to 8 weeks. Behavioural co-intervention provided.

c. Placebo: Behavioural co-intervention provided.

d. For smoking cessation, authors preferred point prevalence over sustained/continuous abstinence as participants were not expected to quit at start of intervention.

e. The only trial included in this analysis is at unclear risk for selection and attrition bias. We downrate this domain by -1.0.

f. Not applicable (single study).

g. No indirectness.

h. Confidence interval encompasses both harm (moderate) and benefit (large). The optimal information size not met (total of 23 events) and inadequate sample size (<2000 participants). We downrate this domain by -2.0.

i. Although search not completely comprehensive, the only trial in this analysis reports negative findings. We do not downrate this domain.

j. Number of participants included in this analysis unclear.

k. Unable to assess confidence intervals. Unable to assess optimal information size and unclear sample size analyzed. We cannot rate this domain.

| Summary of findings: | | | | | | |
| --- | --- | --- | --- | --- | --- | --- |
| **Varenicline compared to placebo in smokers not motivated/wishing to quit** | | | | | | |
| **Patient or population**: Smokers not motivated/wishing to quit  **Setting**: No restriction  **Intervention**: Varenicline  **Comparison**: Placebo | | | | | | |
| Outcomes | **Anticipated absolute effects^*^** (95% CI) | | Relative effect (95% CI) | № of participants  (studies) | Certainty of the evidence (GRADE) | Comments |
|  | **Risk with placebo** | **Risk with Varenicline** |  |  |  |  |
| Abstinence/cessation ^a,b,c,d^  Outcome measurement: point prevalence 100% studies;  Biochemical validation: 100% studies  Follow up: 6 months | 72 per 1,000 | **141 per 1,000** (62 to 317) | RR 1.95 (0.86 to 4.40) | 218 (1 RCT) | ⨁◯◯◯  VERY LOW ^e,f,g,h,i^ | Date of last search: October 2015.  AMSTAR-2: Critically low.  Review authors rated certainty as low. ^j^ |
| Adverse events (stopping medication due to adverse event) ^a,b,c^  Follow up: Unclear/NR | No significant difference between groups (varenicline 12%, placebo 10%). | |  | Unclear/NR ^k^  (1 RCT) | unable to assess ^e,f,g,i,l^ | Date of last search: October 2015.  AMSTAR-2: Critically low.  Not GRADEd by review authors. |
| ***The risk in the intervention group** (and its 95% confidence interval) is based on the assumed risk in the comparison group and the **relative effect** of the intervention (and its 95% CI).   **CI:** Confidence interval; **NR:** Not reported; **RR:** Risk ratio | | | | | | |
| **GRADE Working Group grades of evidence** **High certainty:** We are very confident that the true effect lies close to that of the estimate of the effect **Moderate certainty:** We are moderately confident in the effect estimate: The true effect is likely to be close to the estimate of the effect, but there is a possibility that it is substantially different **Low certainty:** Our confidence in the effect estimate is limited: The true effect may be substantially different from the estimate of the effect **Very low certainty:** We have very little confidence in the effect estimate: The true effect is likely to be substantially different from the estimate of effect | | | | | | |

#### Explanations

a. Review eligibility criterion is people who smoke tobacco but have no immediate intention to quit all tobacco use. The only trial in this analysis recruited general smokers who wished to eventually quit but had no immediate intention to do so.

b. Varenicline: Objective of trial was to evaluate effect of varenicline on inducing quit attempts in smokers not planning to quit. 2 mg/day for 2 to 8 weeks. Behavioural co-intervention provided.

c. Placebo: Behavioural co-intervention provided.

d. For smoking cessation, authors preferred point prevalence over sustained/continuous abstinence as participants were not expected to quit at start of intervention.

e. The only trial included in this analysis is at unclear risk for selection and attrition bias. We downrate this domain by -1.0.

f. Not applicable (single study).

g. No indirectness.

h. Confidence interval encompasses both harm (moderate) and benefit (large). The optimal information size not met (total of 23 events) and inadequate sample size (<2000 participants). We downrate this domain by -2.0.

i. Although search not completely comprehensive, the only trial in this analysis reports negative findings. We do not downrate this domain.

j. Review authors rate certainty of evidence as low (double downrated imprecision domain due to small number of events and small number of studies).

k. Number of participants included in this analysis unclear.

l. Unable to assess confidence intervals. Unable to assess optimal information size and unclear sample size analyzed. We cannot rate this domain.

## Appendix K Table 33. Telephone counselling plus self-help materials versus Usual care: Smoking cessation and reduction in smokers not motivated/wishing to quit

| Telephone-based individual counselling plus self-help materials compared to usual care in smokers not motivated/wishing to quit  **Bibliography:** Lindson-Hawley 2016; Date of last search: October 2015 | | | | | | | | | | | |
| --- | --- | --- | --- | --- | --- | --- | --- | --- | --- | --- | --- |
| **Certainty assessment** | | | | | | | **Summary of findings** | | | | |
| **№ of participants (studies) Follow-up** | **Risk of bias** | **Inconsistency** | **Indirectness** | **Imprecision** | **Publication bias** | **Overall certainty of evidence** | **Study event rates (%)** | | **Relative effect (95% CI)** | **Anticipated absolute effects** | |
|  |  |  |  |  |  |  | **With usual care** | **With Telephone-based individual counselling plus self-help materials** |  | **Risk with usual care** | **Risk difference with Telephone-based individual counselling plus self-help materials** |
| **Abstinence/cessation (follow up: 12 months)** ^a,b,c,d^  Outcome measurement: point prevalence 100% studies  Biochemical validation: 100% studies | | | | | | | | | | | |
| 320 (1 RCT) | not serious ^e^ | not serious ^f^ | not serious ^g^ | very serious ^h^ | none ^i^ | ⨁⨁◯◯  LOW | 7/156 (4.5%) | 11/164 (6.7%) | RR 1.49 (0.59 to 3.76) | 45 per 1,000 | **22 more per 1,000** (from 18 fewer to 124 more) |
| **Reduction in cigarettes/day of >50% of baseline or cessation (follow up: 12 months)** ^a,b,c,j^  Outcome measurement: unclear/NR  Biochemical validation: unclear/NR ^k^ | | | | | | | | | | | |
| 320 (1 RCT) | serious ^l^ | not serious ^f^ | not serious ^g^ | very serious ^m^ | none ^i^ | ⨁◯◯◯  VERY LOW | 29/156 (18.6%) | 41/164 (25.0%) | RR 1.34 (0.88 to 2.05) | 186 per 1,000 | **63 more per 1,000** (from 22 fewer to 195 more) |
| **Reduction in CO >50% (follow up: 12 months)** ^a,b,c,i^  Outcome measurement: unclear/NR | | | | | | | | | | | |
| 320 (1 RCT) | not serious ^e^ | not serious ^f^ | not serious ^g^ | very serious ^n^ | none ^i^ | ⨁⨁◯◯  LOW | 22/156 (14.1%) | 23/164 (14.0%) | RR 0.99 (0.58 to 1.71) | 141 per 1,000 | **1 fewer per 1,000** (from 59 fewer to 100 more) |
| **Reduction in number of cigarettes/day from baseline (follow up: 12 months)** ^a,b,c^  Mean reduction in number of cigarettes/day from baseline  Biochemical validation: unclear/NR ^j^ | | | | | | | | | | | |
| 320 (1 RCT) | serious ^l^ | not serious ^f^ | not serious ^g^ | serious to very serious ^o^ | none ^i^ | unable to assess | Number of cigarettes/day decreased from baseline in both groups (mean change from baseline (SD); Intervention: 21.2 (9.4), Usual care: 20.1 (8.9)). No difference between groups at 12 month follow-up (mean (SD): Intervention: 15.8 (10. 3), Usual care: 15.3 (9.2). | | | | |
| **Reduction in CO from baseline (follow up: 12 months)** ^a,b,c^  Mean reduction in CO from baseline | | | | | | | | | | | |
| 320 (1 RCT) | not serious ^e^ | not serious ^f^ | not serious ^g^ | serious to very serious ^p^ | none ^i^ | unable to assess | CO levels decreased from baseline in both groups (baseline mean (SD) - Intervention 29.8 (13.9), Usual care 29.8 (14.5); 12 months - Intervention: 24.9 (14.0). Usual care: 24.3 (13.8)). No significant between-group difference in the change from baseline. | | | | |

**CI:** Confidence interval; **CO:** Carbon monoxide; **CPD:** Cigarettes per day; **NR:** Not reported; **RR:** Risk ratio

#### Explanations

a. Review eligibility criterion is people who smoke tobacco but have no immediate intention to quit all tobacco use. The only trial in this analysis recruited smokers not interested in quitting who were scheduled to have outpatient surgery or a diagnostic procedure.

b. Intervention: Participants instructed to reduce smoking by 50% or more; cessation encouraged thereafter. Self-help materials were individually tailored newsletters and a targeted newsletter. No co-interventions provided.

c. Control: Usual care consisted of usual care plus generic health mailings. No co-interventions provided.

d. For smoking cessation, authors preferred point prevalence over sustained/continuous abstinence as participants were not expected to quit at start of intervention.

e. The only trial included in this analysis is at unclear risk of selection bias. We downrate this domain by -0.5.

f. Not applicable (single study).

g. No indirectness.

h. Confidence interval encompasses both harm (small but important) and benefit (large). The optimal information size not met (total of 18 events), inadequate sample size (<2000 participants). We downrate this domain by -2.0.

i. Although search not completely comprehensive, the only trial in this analysis reports negative findings. We do not downrate this domain.

j. For smoking reduction outcomes, review authors preferred sustained/continuous rates over point prevalence. However, unclear which was reported for this particular analysis.

k. CO levels were measured in the trial and reported as a separate outcome. However, it is unclear whether reduced levels of CO at follow-up was used to validate self-reported reduction in CPD.

l. The only trial included in this analysis is at unclear risk of selection bias. It is also unclear whether this outcome was biochemically validated. We downrate this domain by -1.0.

m. Confidence interval encompasses both harm (small but important) and benefit (moderate). The optimal information size not met (total of 70 events), inadequate sample size (<2000 participants). We downrate this domain by -2.0

n. Confidence interval encompasses both harm (moderate) and benefit (small but important). The optimal information size not met (total of 45 events), inadequate sample size (<2000 participants). We downrate this domain by -2.0

o. Unable to assess confidence intervals. 320 participants cannot meet the optimal information. We cannot rate this domain.

p. Unable to assess confidence intervals. 320 participants cannot meet the optimal information. We cannot rate this domain.

| Summary of findings: | | | | | | |
| --- | --- | --- | --- | --- | --- | --- |
| **Telephone-based individual counselling plus self-help materials compared to usual care in smokers not motivated/wishing to quit** | | | | | | |
| **Patient or population**: Smokers not motivated/wishing to quit  **Setting**: No restriction  **Intervention**: Telephone-based individual counselling plus self-help materials  **Comparison**: Usual care | | | | | | |
| Outcomes | **Anticipated absolute effects^*^** (95% CI) | | Relative effect (95% CI) | № of participants  (studies) | Certainty of the evidence (GRADE) | Comments |
|  | **Risk with usual care** | **Risk with Telephone-based individual counselling plus self-help materials** |  |  |  |  |
| Abstinence/cessation ^a,b,c,d^  Outcome measurement: point prevalence 100% studies  Biochemical validation: 100% studies  Follow up: 12 months | 45 per 1,000 | **67 per 1,000** (26 to 169) | RR 1.49 (0.59 to 3.76) | 320 (1 RCT) | ⨁⨁◯◯  LOW ^e,f,g,h,i^ | Date of last search: October 2015.  AMSTAR-2: Critically low.  Review authors rated certainty as very low. ^j^ |
| Reduction in cigarettes/day of >50% of baseline or cessation ^a,b,c,k^  Outcome measurement: unclear/NR;  Biochemical validation: unclear/NR ^l^  Follow up: 12 months | 186 per 1,000 | **249 per 1,000** (164 to 381) | RR 1.34 (0.88 to 2.05) | 320 (1 RCT) | ⨁◯◯◯  VERY LOW ^f,g,i,m,n^ | Date of last search: October 2015.  AMSTAR-2: Critically low.  Not GRADEd by review authors. |
| Reduction in CO >50% ^a,b,c,k^  Outcome measurement: unclear/NR  Follow up: 12 months | 141 per 1,000 | **140 per 1,000** (82 to 241) | RR 0.99 (0.58 to 1.71) | 320 (1 RCT) | ⨁⨁◯◯  LOW ^e,f,g,i,o^ | Date of last search: October 2015.  AMSTAR-2: Critically low.  Not GRADEd by review authors. |
| Reduction in number of cigarettes/day from baseline ^a,b,c^  Mean reduction in number of cigarettes/day from baseline  Biochemical validation: unclear/NR ^l^  Follow up: 12 months | Number of cigarettes/day decreased from baseline in both groups (mean change from baseline (SD); Intervention: 21.2 (9.4), Usual care: 20.1 (8.9)). No difference between groups at 12 month follow-up (mean (SD): Intervention: 15.8 (10. 3), Usual care: 15.3 (9.2). | |  | 320 (1 RCT) | unable to assess ^f,g,h,m,p^ | Date of last search: October 2015.  AMSTAR-2: Critically low.  Not GRADEd by review authors. |
| Reduction in CO from baseline ^a,b,c^  Mean reduction in CO from baseline  Follow up: 12 months | CO levels decreased from baseline in both groups (baseline mean (SD) - Intervention 29.8 (13.9), Usual care 29.8 (14.5); 12 months - Intervention: 24.9 (14.0). Usual care: 24.3 (13.8)). No significant between-group difference in the change from baseline. | |  | 320 (1 RCT) | unable to assess ^e,f,g,i,q^ | Date of last search: October 2015.  AMSTAR-2: Critically low.  Not GRADEd by review authors. |
| ***The risk in the intervention group** (and its 95% confidence interval) is based on the assumed risk in the comparison group and the **relative effect** of the intervention (and its 95% CI).   **CI:** Confidence interval; **CO:** Carbon monoxide; **CPD:** Cigarettes per day; **NR:** Not reported; **RR:** Risk ratio | | | | | | |
| **GRADE Working Group grades of evidence** **High certainty:** We are very confident that the true effect lies close to that of the estimate of the effect **Moderate certainty:** We are moderately confident in the effect estimate: The true effect is likely to be close to the estimate of the effect, but there is a possibility that it is substantially different **Low certainty:** Our confidence in the effect estimate is limited: The true effect may be substantially different from the estimate of the effect **Very low certainty:** We have very little confidence in the effect estimate: The true effect is likely to be substantially different from the estimate of effect | | | | | | |

#### Explanations

a. Review eligibility criterion is people who smoke tobacco but have no immediate intention to quit all tobacco use. The only trial in this analysis recruited smokers not interested in quitting who were scheduled to have outpatient surgery or a diagnostic procedure.

b. Intervention: Participants instructed to reduce smoking by 50% or more; cessation encouraged thereafter. Self-help materials were individually tailored newsletters and a targeted newsletter. No co-interventions provided.

c. Control: Usual care consisted of usual care plus generic health mailings. No co-interventions provided.

d. For smoking cessation, authors preferred point prevalence over sustained/continuous abstinence as participants were not expected to quit at start of intervention.

e. The only trial included in this analysis is at unclear risk of selection bias. We downrate this domain by -0.5.

f. Not applicable (single study).

g. No indirectness.

h. Confidence interval encompasses both harm (small but important) and benefit (large). The optimal information size not met (total of 18 events), inadequate sample size (<2000 participants). We downrate this domain by -2.0.

i. Although search not completely comprehensive, the only trial in this analysis reports negative findings. We do not downrate this domain.

j. Review authors rated the certainty of evidence as very low double downrating for imprecision (small number of events, small number of studies) and downrating one level for indirectness (participants scheduled for outpatient surgery therefore results may not be generalizable to general population).

k. For smoking reduction outcomes, review authors preferred sustained/continuous rates over point prevalence. However, unclear which was reported for this particular analysis.

l. CO levels were measured in the trial and reported as a separate outcome. However, it is unclear whether reduced levels of CO at follow-up was used to validate self-reported reduction in CPD.

m. The only trial included in this analysis is at unclear risk of selection bias. It is also unclear whether this outcome was biochemically validated. We downrate this domain by -1.0.

n. Confidence interval encompasses both harm (small but important) and benefit (moderate). The optimal information size not met (total of 70 events), inadequate sample size (<2000 participants). We downrate this domain by -2.0

o. Confidence interval encompasses both harm (moderate) and benefit (small but important). The optimal information size not met (total of 45 events), inadequate sample size (<2000 participants). We downrate this domain by -2.0

p. Unable to assess confidence intervals. 320 participants cannot meet the optimal information. We cannot rate this domain.

q. Unable to assess confidence intervals. 320 participants cannot meet the optimal information. We cannot rate this domain.

## Appendix K Table 34. Behavioural support (advice) plus NRT plus phone calls versus No Intervention: Smoking cessation and reduction in smokers not motivated/wishing to quit

| Behavioural support to reduce smoking (advice) plus NRT (offered if quit date set) plus phone calls compared to no intervention in smokers not motivated/wishing to quit  **Bibliography:** Lindson-Hawley 2016; Date of last search: October 2015 | | | | | | | | | | | |
| --- | --- | --- | --- | --- | --- | --- | --- | --- | --- | --- | --- |
| **Certainty assessment** | | | | | | | **Summary of findings** | | | | |
| **№ of participants (studies) Follow-up** | **Risk of bias** | **Inconsistency** | **Indirectness** | **Imprecision** | **Publication bias** | **Overall certainty of evidence** | **Study event rates (%)** | | **Relative effect (95% CI)** | **Anticipated absolute effects** | |
|  |  |  |  |  |  |  | **With no intervention** | **With Behavioural support to reduce smoking (advice) plus NRT (offered if quit date set) plus phone calls** |  | **Risk with no intervention** | **Risk difference with Behavioural support to reduce smoking (advice) plus NRT (offered if quit date set) plus phone calls** |
| **Abstinence/cessation (follow up: 6 months)** ^a,b,c,d^  Outcome measurement: point prevalence 100% studies  Biochemical validation: 0% studies | | | | | | | | | | | |
| Unclear/NR ^e^ (1 RCT) | very serious ^f^ | not serious ^g^ | not serious ^h^ | serious to very serious ^i^ | none ^j^ | ⨁◯◯◯  VERY LOW | Quit rate significantly higher in the intervention group compared to those receiving no intervention. | | | | |
| **Reduction in number cigarettes/day (follow up: 6 months)** ^a,b,c,k^  Outcome measurement: unclear/NR  Biochemical validation: 0% studies | | | | | | | | | | | |
| Unclear/NR ^l^ (1 RCT) | very serious ^f^ | not serious ^g^ | not serious ^h^ | serious to very serious ^i^ | none ^j^ | ⨁◯◯◯  VERY LOW | Reduction rate significantly higher in the intervention group compared to those receiving no intervention. | | | | |

**CI:** Confidence interval; **NR:** Not reported; **NRT:** Nicotine replacement therapy

#### Explanations

a. Review eligibility criterion is people who smoke tobacco but have no immediate intention to quit all tobacco use. The only trial in this analysis recruited general smokers not interested in cessation.

b. Intervention: Initial advice intervention aimed at encouraging reduction. Participants also advised to quit; those who agreed (i.e., set quit date) received cessation intervention. Participants offered choice of NRT gum or patch (dosage and duration not specified). No co-interventions provided.

c. Control: Assessment calls only. No co-interventions provided.

d. For smoking cessation, authors preferred point prevalence over sustained/continuous abstinence as participants were not expected to quit at start of intervention.

e. A total of 616 participants in this three-arm study; 419 total participants in the two arms compared in this analysis. However, unclear whether all 419 participants were analyzed.

f. The only trial included in this analysis is at unclear risk for selection and attrition bias. The trial is also at high risk for performance/detection bias as blinding was not possible due to the nature of the intervention and smoking outcomes were not biochemically validated. We downrate this domain by -2.0.

g. Not applicable (single study).

h. No indirectness.

i. Unable to assess confidence intervals. Unclear sample size analyzed but at most 419 total participants and the optimal information size cannot be met. We cannot rate this domain, but suggesting a range for rating based on available information to assist with overall certainty of evidence.

j. Search not completely comprehensive and only trial in this analysis reports positive findings, but lack of negative trials may be reflective of an under-researched area as opposed to suppression of findings. Given not enough information exists to strongly suspect publication bias, we do not downrate this domain.

k. For smoking reduction outcomes, review authors preferred sustained/continuous rates over point prevalence. However, unclear which was reported for this particular analysis.

l. Number of participants included in this analysis is unclear. Review authors indicate that those who successfully quit were excluded.

| Summary of findings: | | | | | | |
| --- | --- | --- | --- | --- | --- | --- |
| **Behavioural support to reduce smoking (advice) plus NRT (offered if quit date set) plus phone calls compared to no intervention in smokers not motivated/wishing to quit** | | | | | | |
| **Patient or population**: Smokers not motivated/wishing to quit  **Setting**: No restriction  **Intervention**: Behavioural support to reduce smoking (advice) plus NRT (offered if quit date set) plus phone calls  **Comparison**: No intervention | | | | | | |
| Outcomes | **Anticipated absolute effects^*^** (95% CI) | | Relative effect (95% CI) | № of participants  (studies) | Certainty of the evidence (GRADE) | Comments |
|  | **Risk with no intervention** | **Risk with Behavioural support to reduce smoking (advice) plus NRT (offered if quit date set) plus phone calls** |  |  |  |  |
| Abstinence/cessation ^a,b,c,d^  Outcome measurement: point prevalence 100% studies  Biochemical validation: 0% studies  Follow up: 6 months | Quit rate significantly higher in the intervention group compared to those receiving no intervention. | |  | Unclear/NR ^e^  (1 RCT) | ⨁◯◯◯  VERY LOW^f,g,h,I,j^ | Date of last search: October 2015.  AMSTAR-2: Critically low.  Not GRADEd by review authors. |
| Reduction in number cigarettes/day ^a,b,c,k^  Outcome measurement: unclear/NR  Biochemical validation: 0% studies  Follow up: 6 months | Reduction rate significantly higher in the intervention group compared to those receiving no intervention. | |  | Unclear/NR ^l^  (1 RCT) | ⨁◯◯◯  VERY LOW ^f,g,h,I,j^ | Date of last search: October 2015.  AMSTAR-2: Critically low.  Not GRADEd by review authors. |
| ***The risk in the intervention group** (and its 95% confidence interval) is based on the assumed risk in the comparison group and the **relative effect** of the intervention (and its 95% CI).   **CI:** Confidence interval; **NR:** Not reported; **NRT:** Nicotine replacement therapy | | | | | | |
| **GRADE Working Group grades of evidence** **High certainty:** We are very confident that the true effect lies close to that of the estimate of the effect **Moderate certainty:** We are moderately confident in the effect estimate: The true effect is likely to be close to the estimate of the effect, but there is a possibility that it is substantially different **Low certainty:** Our confidence in the effect estimate is limited: The true effect may be substantially different from the estimate of the effect **Very low certainty:** We have very little confidence in the effect estimate: The true effect is likely to be substantially different from the estimate of effect | | | | | | |

#### Explanations

a. Review eligibility criterion is people who smoke tobacco but have no immediate intention to quit all tobacco use. The only trial in this analysis recruited general smokers not interested in cessation.

b. Intervention: Initial advice intervention aimed at encouraging reduction. Participants also advised to quit; those who agreed (i.e., set quit date) received cessation intervention. Participants offered choice of NRT gum or patch (dosage and duration not specified). No co-interventions provided.

c. Control: Assessment calls only. No co-interventions provided.

d. For smoking cessation, authors preferred point prevalence over sustained/continuous abstinence as participants were not expected to quit at start of intervention.

e. A total of 616 participants in this three-arm study; 419 total participants in the two arms compared in this analysis. However, unclear whether all 419 participants were analyzed.

f. The only trial included in this analysis is at unclear risk for selection and attrition bias. The trial is also at high risk for performance/detection bias as blinding was not possible due to the nature of the intervention and smoking outcomes were not biochemically validated. We downrate this domain by -2.0.

g. Not applicable (single study).

h. No indirectness.

i. Unable to assess confidence intervals. Unclear sample size analyzed but at least 419 total participants and the optimal information size cannot be met. We cannot rate this domain.j. Search not completely comprehensive and only trial in this analysis reports positive findings, but lack of negative trials may be reflective of an under-researched area as opposed to suppression of findings. Given not enough information exists to strongly suspect publication bias, we do not downrate this domain.

k. For smoking reduction outcomes, review authors preferred sustained/continuous rates over point prevalence. However, unclear which was reported for this particular analysis.

l. Number of participants included in this analysis is unclear. Review authors indicate that those who successfully quit were excluded.

## Appendix K Table 35. E-cigarette versus Placebo: Smoking cessation, reduction, adverse events, and weight gain in smokers not motivated/wishing to quit

| Electronic cigarettes compared to placebo electronic cigarettes in smokers not motivated/wishing to quit  **Bibliography:** Lindson-Hawley 2016; Date of last search: October 2015 | | | | | | | | | | | |
| --- | --- | --- | --- | --- | --- | --- | --- | --- | --- | --- | --- |
| **Certainty assessment** | | | | | | | **Summary of findings** | | | | |
| **№ of participants (studies) Follow-up** | **Risk of bias** | **Inconsistency** | **Indirectness** | **Imprecision** | **Publication bias** | **Overall certainty of evidence** | **Study event rates (%)** | | **Relative effect (95% CI)** | **Anticipated absolute effects** | |
|  |  |  |  |  |  |  | **With placebo electronic cigarettes** | **With Electronic cigarettes** |  | **Risk with placebo electronic cigarettes** | **Risk difference with Electronic cigarettes** |
| **Abstinence/cessation (follow up: 12 months)** ^a,b,c,d^  Outcome measurement: defined as 'not even a puff' since previous visit  Biochemical validation: 100% studies | | | | | | | | | | | |
| 300 (1 RCT) | not serious ^e^ | not serious ^f^ | not serious ^g^ | very serious ^h^ | none ^i^ | ⨁⨁◯◯  LOW | 4/100 (4.0%) | 22/200 (11.0%) | RR 2.75 (0.97 to 7.76) | 40 per 1,000 | **70 more per 1,000** (from 1 fewer to 270 more) |
| **Reduction in cigarettes/day of >50% of baseline or cessation (follow up: 12 months)** ^a,b,c,j^  Outcome measurement: unclear/NR  Biochemical validation: unclear/NR ^k^ | | | | | | | | | | | |
| 300 (1 RCT) | serious ^l^ | not serious ^f^ | not serious ^g^ | very serious ^m^ | none ^i^ | ⨁◯◯◯  VERY LOW | 16/100 (16.0%) | 41/200 (20.5%) | RR 1.28 (0.76 to 2.17) | 160 per 1,000 | **45 more per 1,000** (from 38 fewer to 187 more) |
| **Reduction in number cigarettes/day (follow up: 12 months)** ^a,b,c^  Outcome measurement: unclear/NR  Biochemical validation: unclear/NR ^k^ | | | | | | | | | | | |
| 300 (1 RCT) | serious ^l^ | not serious ^f^ | not serious ^g^ | serious to very serious ^n^ | none ^i^ | unable to assess | No statistically significant difference in number of cigarettes/day between groups. Median CPD = 12 - 14 in all groups. | | | | |
| **Reduction in CO (follow up: 12 months)** ^a,b,c^  Outcome measurement: unclear/NR | | | | | | | | | | | |
| 300 (1 RCT) | not serious ^e^ | not serious ^f^ | not serious ^g^ | serious to very serious ^n^ | none ^i^ | unable to assess | No significant difference in CO between groups. Median CO = 15-17 ppm in all groups. | | | | |
| **Adverse events (follow up: Baseline, 3 months, 12 months)** ^a,b,c^ | | | | | | | | | | | |
| Unclear/NR ^o^ (1 RCT) | not serious ^e^ | not serious ^f^ | not serious ^g^ | not serious to very serious ^p^ | none ^i^ | unable to assess | Similar frequency of AEs across groups at all timepoints (baseline, week 12, week 52). Reduction in all symptoms from baseline to 12-month follow-up across groups (P < 0.001). Rates of shortness of breath were reduced from 20% to 4% from baseline to 2 weeks. | | | | |
| **Serious adverse events (follow up: 12 months)** ^a,b,c^ | | | | | | | | | | | |
| Unclear/NR ^o^ (1 RCT) | not serious ^e^ | not serious ^f^ | not serious ^g^ | not serious to very serious ^p^ | none ^i^ | unable to assess | No serious adverse events occurred. | | | | |
| **Weight gain (follow up: Unclear/NR)** ^a,b,c^ | | | | | | | | | | | |
| Unclear/NR ^o^ (1 RCT) | not serious ^e^ | not serious ^f^ | not serious ^g^ | not serious to very serious ^p^ | none ^i^ | unable to assess | No significant difference in weight change within or between groups. Uncertain but unlikely that the outcome is post-cessation weight gain (i.e., does not appear to be assessed in abstinent smokers only). | | | | |

**CI:** Confidence interval; **CO:** Carbon monoxide; **CPD:** Cigarettes per day; **NR:** Not reported; **RR:** Risk ratio

#### Explanations

a. Review eligibility criterion is people who smoke tobacco but have no immediate intention to quit all tobacco use. The only trial in this analysis recruited general smokers not interested in cessation.

b. E-cigarette: Intervention used to assist smoking reduction. All data is from a 3-arm trial in which two arms received e-cigarettes containing nicotine (either 7.2 mg or 5.4 mg supplied for 12 weeks); active arms combined in analyses. No co-interventions provided.

c. Placebo: Received e-cigarettes without nicotine. No co-interventions provided.

d. For smoking cessation, authors preferred point prevalence over sustained/continuous abstinence as participants were not expected to quit at start of intervention.

e. The only trial included in this analysis is at unclear risk for performance/detection bias due to inadequate reporting. We downrate this domain by -0.5.

f. Not applicable (single study).

g. No indirectness.

h. Confidence interval encompasses three ranges of effect (little to no difference to large benefit). The optimal information size not met (total of 26 events) and inadequate sample size (<2000 participants). We downrate this domain by -2.0.

i. Although search not completely comprehensive, only trial in this analysis reports negative findings. We do not downrate this domain.

j. For smoking reduction outcomes, review authors preferred sustained/continuous rates over point prevalence. However, unclear which was reported for this particular analysis.

k. CO levels were measured in the trial and reported as a separate outcome. It is unclear whether reduced levels of CO at follow-up was used to validate self-reported reduction in CPD.

l. The only trial included in this analysis is at unclear risk for performance/detection bias due to inadequate reporting. It is also unclear whether this outcome was biochemically validated. We downrate this domain by -1.0.

m. Confidence interval encompasses harm (small but important) to benefit (moderate). The optimal information size not met (total of 57 events) and inadequate sample size (<2000 participants). We downrate this domain by -2.0.

n. Unable to assess confidence intervals, but 300 total participants and the optimal information size cannot be met. We cannot rate this domain. o. Number of participants included in this analysis unclear/NR.

p. Sample size analyzed not reported and unable to assess optimal information size and confidence intervals. We cannot rate this domain.

| Summary of findings: | | | | | | |
| --- | --- | --- | --- | --- | --- | --- |
| **Electronic cigarettes compared to placebo electronic cigarettes in smokers not motivated/wishing to quit** | | | | | | |
| **Patient or population**: Smokers not motivated/wishing to quit  **Setting**: No restriction  **Intervention**: Electronic cigarettes  **Comparison**: Placebo electronic cigarettes | | | | | | |
| Outcomes | **Anticipated absolute effects^*^** (95% CI) | | Relative effect (95% CI) | № of participants  (studies) | Certainty of the evidence (GRADE) | Comments |
|  | **Risk with placebo electronic cigarettes** | **Risk with Electronic cigarettes** |  |  |  |  |
| Abstinence/cessation ^a,b,c,d^  Outcome measurement: defined as 'not even a puff' since previous visit  Biochemical validation: 100% studies  Follow up: 12 months | 40 per 1,000 | **110 per 1,000** (39 to 310) | RR 2.75 (0.97 to 7.76) | 300 (1 RCT) | ⨁⨁◯◯  LOW ^e,f,g,h,i^ | Date of last search: October 2015.  AMSTAR-2: Critically low.  Review authors rated certainty as low. ^j^ |
| Reduction in cigarettes/day of >50% of baseline or cessation ^a,b,c,k^  Outcome measurement: unclear/NR;  Biochemical validation: unclear/NR ^l^  follow up: 12 months | 160 per 1,000 | **205 per 1,000** (122 to 347) | RR 1.28 (0.76 to 2.17) | 300 (1 RCT) | ⨁◯◯◯  VERY LOW ^f,g,i,m,n^ | Date of last search: October 2015.  AMSTAR-2: Critically low.  Not GRADEd by review authors. |
| Reduction in number cigarettes/day ^a,b,c^  Outcome measurement: unclear/NR;  Biochemical validation: unclear/NR ^k^  Follow up: 12 months | No statistically significant difference in number of cigarettes/day between groups. Median CPD = 12 - 14 in all groups. | |  | 300 (1 RCT) | unable to assess ^f,g,i,m,o^ | Date of last search: October 2015.  AMSTAR-2: Critically low.  Not GRADEd by review authors. |
| Reduction in CO ^a,b,c^  Outcome measurement: unclear/NR  Follow up: 12 months | No significant difference in CO between groups. Median CO = 15-17 ppm in all groups. | |  | 300 (1 RCT) | unable to assess ^e,f,g,i,o^ | Date of last search: October 2015.  AMSTAR-2: Critically low.  Not GRADEd by review authors. |
| Adverse events ^a,b,c^  Follow up: Baseline, 3 months, 12 months | Similar frequency of AEs across groups at all timepoints (baseline, week 12, week 52). Reduction in all symptoms from baseline to 12-month follow-up across groups (P < 0.001). Rates of shortness of breath were reduced from 20% to 4% from baseline to 2 weeks. | |  | Unclear/NR ^p^  (1 RCT) | unable to assess ^e,f,g,i,q^ | Date of last search: October 2015.  AMSTAR-2: Critically low.  Not GRADEd by review authors. |
| Serious adverse events ^a,b,c^  Follow up: 12 months | No serious adverse events occurred. | |  | Unclear/NR ^p^  (1 RCT) | unable to assess ^e,f,g,i,q^ | Date of last search: October 2015.  AMSTAR-2: Critically low.  Not GRADEd by review authors. |
| Weight gain ^a,b,c^  Follow up: Unclear/NR | No significant difference in weight change within or between groups. Uncertain but unlikely that the outcome is post-cessation weight gain (i.e., does not appear to be assessed in abstinent smokers only). | |  | Unclear/NR ^p^  (1 RCT) | unable to assess ^e,f,g,i,q^ | Date of last search: October 2015.  AMSTAR-2: Critically low.  Not GRADEd by review authors. |
| ***The risk in the intervention group** (and its 95% confidence interval) is based on the assumed risk in the comparison group and the **relative effect** of the intervention (and its 95% CI).   **CI:** Confidence interval; **CO:** Carbon monoxide; **CPD:** Cigarettes per day; **NR:** Not reported; **RR:** Risk ratio | | | | | | |
| **GRADE Working Group grades of evidence** **High certainty:** We are very confident that the true effect lies close to that of the estimate of the effect **Moderate certainty:** We are moderately confident in the effect estimate: The true effect is likely to be close to the estimate of the effect, but there is a possibility that it is substantially different **Low certainty:** Our confidence in the effect estimate is limited: The true effect may be substantially different from the estimate of the effect **Very low certainty:** We have very little confidence in the effect estimate: The true effect is likely to be substantially different from the estimate of effect | | | | | | |

#### Explanations

a. Review eligibility criterion is people who smoke tobacco but have no immediate intention to quit all tobacco use. The only trial in this analysis recruited general smokers not interested in cessation.

b. E-cigarette: Intervention used to assist smoking reduction. All data is from a 3-arm trial in which two arms received e-cigarettes containing nicotine (either 7.2 mg or 5.4 mg supplied for 12 weeks); active arms combined in analyses. No co-interventions provided.

c. Placebo: Received e-cigarettes without nicotine. No co-interventions provided.

d. For smoking cessation, authors preferred point prevalence over sustained/continuous abstinence as participants were not expected to quit at start of intervention.

e. The only trial included in this analysis is at unclear risk for performance/detection bias due to inadequate reporting. We downrate this domain by -0.5.

f. Not applicable (single study).

g. No indirectness.

h. Confidence interval encompasses three ranges of effect (little to no difference to large benefit). The optimal information size not met (total of 26 events) and inadequate sample size (<2000 participants). We downrate this domain by -2.0.

i. Although search not completely comprehensive, only trial in this analysis reports negative findings. We do not downrate this domain.

j. Review authors rate certainty of evidence as low (double downrated imprecision domain due to small number of events and small number of studies).

k. For smoking reduction outcomes, review authors preferred sustained/continuous rates over point prevalence. However, unclear which was reported for this particular analysis.

l. CO levels were measured in the trial and reported as a separate outcome. It is unclear whether reduced levels of CO at follow-up was used to validate self-reported reduction in CPD.

m. The only trial included in this analysis is at unclear risk for performance/detection bias due to inadequate reporting. It is also unclear whether this outcome was biochemically validated. We downrate this domain by -1.0.

n. Confidence interval encompasses harm (small but important) to benefit (moderate). The optimal information size not met (total of 57 events) and inadequate sample size (<2000 participants). We downrate this domain by -2.0.

o. Unable to assess confidence intervals, but 300 total participants and the optimal information size cannot be met. We cannot rate this domain.

p. Number of participants included in this analysis unclear/NR.

q. Sample size analyzed not reported and unable to assess optimal information size and confidence intervals. We cannot rate this domain.

# Livingstone-Banks 2019 {1077}

## Appendix K Table 36. Non-tailored print-based self-help materials (no face-to-face contact) versus No materials/no intervention: Smoking cessation in general/mixed population of smokers

| Non-tailored print-based self-help materials (no face-to-face contact) compared to no materials/no intervention in general/mixed population of smokers  **Bibliography:** Livingstone-Banks 2019; Date of last search: March 2018 | | | | | | | | | | | |
| --- | --- | --- | --- | --- | --- | --- | --- | --- | --- | --- | --- |
| **Certainty assessment** | | | | | | | **Summary of findings** | | | | |
| **№ of participants (studies) Follow-up** | **Risk of bias** | **Inconsistency** | **Indirectness** | **Imprecision** | **Publication bias** | **Overall certainty of evidence** | **Study event rates (%)** | | **Relative effect (95% CI)** | **Anticipated absolute effects** | |
|  |  |  |  |  |  |  | **With no materials/no intervention** | **With Non-tailored print-based self-help materials (no face-to-face contact)** |  | **Risk with no materials/no intervention** | **Risk difference with Non-tailored print-based self-help materials (no face-to-face contact)** |
| **Abstinence/Cessation (follow up: 6+ months)** ^a,b,c^  Outcome measurement: point prevalence 27%, continuous/sustained 27%, unclear/NR 45% studies  Biochemical validation: 46% studies | | | | | | | | | | | |
| 13241 (11 RCTs) | not serious ^d^ | not serious ^e^ | not serious ^f^ | not serious ^g^ | none ^h^ | ⨁⨁⨁⨁  HIGH | 331/6518 (5.1%) | 416/6723 (6.2%) | RR 1.19 (1.04 to 1.37) | 51 per 1,000 | **10 more per 1,000** (from 2 more to 19 more) |

**CI:** Confidence interval; **NR:** Not reported; **RR:** Risk ratio

#### Explanations

a. As reported by review authors, participants in trials were not selected for interest in quitting smoking. Trials recruited general smokers (n=6), smokers motivated to quit (n=1), smokers not motivated to quit (n=1), females only (n=1), and males only (n=2).

b. Intervention: All trials in this analysis sent non-tailored materials to participants without personal contact. Additional behavioural co-intervention provided in one trial.

c. Control: Various control conditions across trials including no intervention, usual care, wait-list, letter apologizing for shortage of kits. No co-interventions provided to control arms across trials.

d. Unclear risk for selection bias for 66% of evidence. In addition, unclear or high risk of bias issues among performance/detection, attrition, or biochemical validation for 42% of evidence. We downrate this domain by -0.75.

e. Some variation in effect estimates but confidence intervals largely overlap (I2=0%, p=0.64). We do not downrate this domain.

f. Due to reporting, extent of indirectness could not be determined for three studies contributing a combined weight of 59%; the studies included smokers and recent quitters without reporting the proportion of each. Participants in one of these trials were also likely exposed to a community Quit and Win contest. No indirectness in remaining studies. We do not downrate this domain.

g. Confidence interval encompasses one range of effect (little to no difference to small but important benefit). The optimal information size is met (total of 747 events) and adequate sample size. We do not downrate this domain.

h. Search strategy is not completely comprehensive; however, most trials report negative findings and review authors state that no evidence of asymmetry for all analyses assessed (i.e., those with at least 10 studies). We do not downrate this domain.

| Summary of findings: | | | | | | |
| --- | --- | --- | --- | --- | --- | --- |
| **Non-tailored print-based self-help materials (no face-to-face contact) compared to no materials/no intervention in general/mixed population of smokers** | | | | | | |
| **Patient or population**: General/mixed population of smokers  **Setting**: No restriction  **Intervention**: Non-tailored print-based self-help materials (no face-to-face contact)  **Comparison**: No materials/no intervention | | | | | | |
| Outcomes | **Anticipated absolute effects^*^** (95% CI) | | Relative effect (95% CI) | № of participants  (studies) | Certainty of the evidence (GRADE) | Comments |
|  | **Risk with no materials/no intervention** | **Risk with Non-tailored print-based self-help materials (no face-to-face contact)** |  |  |  |  |
| Abstinence/Cessation ^a,b,c^  Outcome measurement: point prevalence 27%, continuous/sustained 27%, unclear/NR 45% studies  Biochemical validation: 46% studies  Follow up: 6+ months | 51 per 1,000 | **60 per 1,000** (52 to 70) | RR 1.19 (1.04 to 1.37) | 13241 (11 RCTs) | ⨁⨁⨁⨁  HIGH ^d,e,f,g,h^ | Three trials excluded from this analysis: two trials recruited treatment seeking smokers and observed high quit rates; they were excluded from the meta-analysis due to heterogeneity. The two studies were pooled in a separate analysis by review authors and are covered under a separate GRADE assessment. One trial provided insufficient data but suggested no difference between groups.  Authors performed this analysis as a subgroup analysis; may be plausible.  AMSTAR-2: Moderate.  Date of last search: March 2018.  Review authors rate as moderate certainty. ^i^ |
| ***The risk in the intervention group** (and its 95% confidence interval) is based on the assumed risk in the comparison group and the **relative effect** of the intervention (and its 95% CI).   **CI:** Confidence interval; **NR:** Not reported; **RR:** Risk ratio | | | | | | |
| **GRADE Working Group grades of evidence** **High certainty:** We are very confident that the true effect lies close to that of the estimate of the effect **Moderate certainty:** We are moderately confident in the effect estimate: The true effect is likely to be close to the estimate of the effect, but there is a possibility that it is substantially different **Low certainty:** Our confidence in the effect estimate is limited: The true effect may be substantially different from the estimate of the effect **Very low certainty:** We have very little confidence in the effect estimate: The true effect is likely to be substantially different from the estimate of effect | | | | | | |

#### Explanations

a. As reported by review authors, participants in trials were not selected for interest in quitting smoking. Trials recruited general smokers (n=6), smokers motivated to quit (n=1), smokers not motivated to quit (n=1), females only (n=1), and males only (n=2).

b. Intervention: All trials in this analysis sent non-tailored materials to participants without personal contact. Additional behavioural co-intervention provided in one trial.

c. Control: Various control conditions across trials including no intervention, usual care, wait-list, letter apologizing for shortage of kits. No co-interventions provided to control arms across trials.

d. Unclear selection bias for 66% of evidence. In addition, unclear or high risk of bias issues among performance/detection, attrition, or biochemical validation for 42% of evidence. We downrate this domain by -0.75.

e. Some variation in effect estimates but confidence intervals largely overlap (I2=0%, p=0.64). We do not downrate this domain.

f. Due to reporting, extent of indirectness could not be determined for three studies contributing a combined weight of 59%; the studies included smokers and recent quitters without reporting the proportion of each. Participants in one of these trials were also likely exposed to community Quit and Win contest. No indirectness in remaining studies. We do not downrate this domain.

g. Confidence interval encompasses one range of effect (little to no difference to small but important benefit). The optimal information size is met (total of 747 events) and adequate sample size. We do not downrate this domain.

h. Search strategy is not completely comprehensive; however, most trials report negative findings and review authors state that no evidence of asymmetry for all analyses assessed (i.e., those with at least 10 studies). We do not downrate this domain.

i. Review authors rate as moderate certainty (downrated one level for indirectness due to concerns about applicability of findings to low- and middle-income countries).

## Appendix K Table 37. Non-tailored print-based self-help materials (no face-to-face contact) versus No materials/no intervention: Smoking cessation in smokers motivated/wishing to quit

| Non-tailored print-based self-help materials (no face-to-face contact) compared to no materials/no intervention in smokers motivated to quit/wishing to quit  **Bibliography:** Livingstone-Banks 2019; Date of last search: March 2018 | | | | | | | | | | | |
| --- | --- | --- | --- | --- | --- | --- | --- | --- | --- | --- | --- |
| **Certainty assessment** | | | | | | | **Summary of findings** | | | | |
| **№ of participants (studies) Follow-up** | **Risk of bias** | **Inconsistency** | **Indirectness** | **Imprecision** | **Publication bias** | **Overall certainty of evidence** | **Study event rates (%)** | | **Relative effect (95% CI)** | **Anticipated absolute effects** | |
|  |  |  |  |  |  |  | **With no materials/no intervention** | **With Non-tailored print-based self-help materials (no face-to-face contact)** |  | **Risk with no materials/no intervention** | **Risk difference with Non-tailored print-based self-help materials (no face-to-face contact)** |
| **Abstinence/Cessation (follow up: 6 months)** ^a,b,c^  Outcome measurement: point prevalence 50%, continuous/sustained abstinence 50% studies  Biochemical validation: 50% studies | | | | | | | | | | | |
| 924 (2 RCTs) | very serious ^d^ | not serious ^e^ | not serious ^f^ | serious ^g^ | none ^h^ | ⨁◯◯◯  VERY LOW | 6/342 (1.8%) | 135/582 (23.2%) | RR 10.91 (5.03 to 23.66) | 18 per 1,000 | **174 more per 1,000** (from 71 more to 398 more) |

**CI:** Confidence interval; **RR:** Risk ratio

#### Explanations

a. Both studies enrolled treatment-seeking smokers.

b. Intervention: Both trials in this analysis sent non-tailored materials to participants without personal contact. No co-interventions provided.

c. Control: Wait list control in both trials. No co-interventions provided to control arm.

d. 100% of evidence with substantive bias issues: 8% at high risk for performance/detection bias and unclear risk for selection bias, 92% at high risk for two domains (performance/detection, biochemical validation) and unclear risk for remaining domains. We downrate this domain by -2.0.

e. Point estimates vary but confidence intervals overlap (I2=0%; p=0.42). We do not downrate this domain.

f. No indirectness.

g. Confidence interval encompasses large benefit. The optimal information size not met (total of 141 events) and inadequate sample size (<2000 participants). We downrate this domain by -1.0.

h. Search strategy not completely comprehensive and studies both report positive findings, but lack of negative trials may be reflective of an under-researched area as opposed to suppression of findings. Given not enough information exists to strongly suspect publication bias, we do not downrate this domain.

| Summary of findings: | | | | | | |
| --- | --- | --- | --- | --- | --- | --- |
| **Non-tailored print-based self-help materials (no face-to-face contact) compared to no materials/no intervention in smokers motivated to quit/wishing to quit** | | | | | | |
| **Patient or population**: Smokers motivated to quit/wishing to quit  **Setting**: No restriction  **Intervention**: Non-tailored print-based self-help materials (no face-to-face contact)  **Comparison**: No materials/no intervention | | | | | | |
| Outcomes | **Anticipated absolute effects^*^** (95% CI) | | Relative effect (95% CI) | № of participants  (studies) | Certainty of the evidence (GRADE) | Comments |
|  | **Risk with no materials/no intervention** | **Risk with Non-tailored print-based self-help materials (no face-to-face contact)** |  |  |  |  |
| Abstinence/Cessation ^a,b,c^  Outcome measurement: point prevalence 50%, continuous/sustained abstinence 50% studies  Biochemical validation: 50% studies  Follow up: 6 months | 18 per 1,000 | **191 per 1,000** (88 to 415) | RR 10.91 (5.03 to 23.66) | 924 (2 RCTs) | ⨁◯◯◯  VERY LOW ^d,e,f,g,h^ | AMSTAR-2: Moderate.  Date of last search: March 2018.  Not GRADEd by review authors. |
| ***The risk in the intervention group** (and its 95% confidence interval) is based on the assumed risk in the comparison group and the **relative effect** of the intervention (and its 95% CI).   **CI:** Confidence interval; **RR:** Risk ratio | | | | | | |
| **GRADE Working Group grades of evidence** **High certainty:** We are very confident that the true effect lies close to that of the estimate of the effect **Moderate certainty:** We are moderately confident in the effect estimate: The true effect is likely to be close to the estimate of the effect, but there is a possibility that it is substantially different **Low certainty:** Our confidence in the effect estimate is limited: The true effect may be substantially different from the estimate of the effect **Very low certainty:** We have very little confidence in the effect estimate: The true effect is likely to be substantially different from the estimate of effect | | | | | | |

#### Explanations

a. Both studies enrolled treatment-seeking smokers.

b. Intervention: Both trials in this analysis sent non-tailored materials to participants without personal contact. No co-interventions provided.

c. Control: Wait list control in both trials. No co-interventions provided to control arm.

d. 100% of evidence with substantive bias issues: 8% at high risk for performance/detection bias and unclear risk for selection bias, 92% at high risk for two domains (performance/detection, biochemical validation) and unclear risk for remaining domains. We downrate this domain by -2.0.

e. Point estimates vary but confidence intervals overlap (I2= 0%; p=0.42). We do not downrate this domain.

f. No indirectness.

g. Confidence interval encompasses large benefit. The optimal information size not met (total of 141 events) and inadequate sample size (<2000 participants). We downrate this domain by -1.0.

h. Search strategy not completely comprehensive and studies both report positive findings, but lack of negative trials may be reflective of an under-researched area as opposed to suppression of findings. Given not enough information exists to strongly suspect publication bias, we do not downrate this domain.

## Appendix K Table 38. Non-tailored print-based self-help materials (no face-to-face contact) versus Brief leaflet: Smoking cessation in general/mixed population of smokers

| Non-tailored print-based self-help materials (no face-to-face contact) compared to brief leaflet in general/mixed population of smokers  **Bibliography:** Livingstone-Banks 2019; Date of last search: March 2018 | | | | | | | | | | | |
| --- | --- | --- | --- | --- | --- | --- | --- | --- | --- | --- | --- |
| **Certainty assessment** | | | | | | | **Summary of findings** | | | | |
| **№ of participants (studies) Follow-up** | **Risk of bias** | **Inconsistency** | **Indirectness** | **Imprecision** | **Publication bias** | **Overall certainty of evidence** | **Study event rates (%)** | | **Relative effect (95% CI)** | **Anticipated absolute effects** | |
|  |  |  |  |  |  |  | **With brief leaflet** | **With Non-tailored print-based self-help materials (no face-to-face contact)** |  | **Risk with brief leaflet** | **Risk difference with Non-tailored print-based self-help materials (no face-to-face contact)** |
| **Abstinence/Cessation (follow up: 6+ months)** ^a,b,c^  Outcome measurement: continuous/sustained abstinence 50%, unclear/NR 50% studies  Biochemical validation: 17% studies | | | | | | | | | | | |
| 7023 (6 RCTs) ^d^ | serious ^e^ | not serious ^f^ | not serious ^g^ | not serious ^h^ | none ^i^ | ⨁⨁⨁◯  MODERATE | 208/2676 (7.8%) | 279/4347 (6.4%) | RR 0.87 (0.71 to 1.07) | 78 per 1,000 | **10 fewer per 1,000** (from 23 fewer to 5 more) |

**CI:** Confidence interval; **NR:** Not reported; **RR:** Risk ratio

#### Explanations

a. All trials recruited general smokers, except for one, where only a subgroup of participants were smokers.

b. Intervention: All trials in this analysis sent non-tailored materials to participants without personal contact. No co-interventions provided to intervention arms across trials.

c. Control: Brief leaflet considered to be a minimal print-based self-help intervention by authors. Behavioural co-intervention provided in all studies.

d. One study was a cluster RCT.

e. 100% of evidence with bias issues: 74% at high risk of bias for at least one domain (biochemical validation with or without selection bias) and with most also at unclear risk for selection bias with or without issues with attrition. Remaining evidence (26%) at unclear risk of selection bias only. We downrate this domain by -1.5.

f. Some variation in point estimates but confidence intervals largely overlap (I2= 21%; p=0.27). We do not downrate this domain.

g. No indirectness.

h. Confidence interval encompasses one range of effect (small but important harm to little to no difference). The optimal information size is met (total of 487 events) and adequate sample size. We do not downrate this domain.

i. Search was not completely comprehensive, but most trials report negative findings. We do not downrate this domain.

| Summary of findings: | | | | | | |
| --- | --- | --- | --- | --- | --- | --- |
| **Non-tailored print-based self-help materials (no face-to-face contact) compared to brief leaflet in general/mixed population of smokers** | | | | | | |
| **Patient or population**: General/mixed population of smokers  **Setting**: No restriction  **Intervention**: Non-tailored print-based self-help materials (no face-to-face contact)  **Comparison**: Brief leaflet | | | | | | |
| Outcomes | **Anticipated absolute effects^*^** (95% CI) | | Relative effect (95% CI) | № of participants  (studies) | Certainty of the evidence (GRADE) | Comments |
|  | **Risk with brief leaflet** | **Risk with Non-tailored print-based self-help materials (no face-to-face contact)** |  |  |  |  |
| Abstinence/Cessation ^a,b,c^  Outcome measurement: continuous/sustained abstinence 50%, unclear/NR 50% studies  Biochemical validation: 17% studies  Follow up: 6+ months | 78 per 1,000 | **68 per 1,000** (55 to 83) | RR 0.87 (0.71 to 1.07) | 7023 (6 RCTs) ^d^ | ⨁⨁⨁◯  MODERATE ^e,f,g,h,i^ | Authors performed this analysis as a subgroup analysis; may be plausible.  AMSTAR-2: Moderate.  Date of last search: March 2018.  Not GRADEd by review authors. |
| ***The risk in the intervention group** (and its 95% confidence interval) is based on the assumed risk in the comparison group and the **relative effect** of the intervention (and its 95% CI).   **CI:** Confidence interval; **NR:** Not reported; **RR:** Risk ratio | | | | | | |
| **GRADE Working Group grades of evidence** **High certainty:** We are very confident that the true effect lies close to that of the estimate of the effect **Moderate certainty:** We are moderately confident in the effect estimate: The true effect is likely to be close to the estimate of the effect, but there is a possibility that it is substantially different **Low certainty:** Our confidence in the effect estimate is limited: The true effect may be substantially different from the estimate of the effect **Very low certainty:** We have very little confidence in the effect estimate: The true effect is likely to be substantially different from the estimate of effect | | | | | | |

#### Explanations

a. All trials recruited general smokers, except for one, where only a subgroup of participants were smokers.

b. Intervention: All trials in this analysis sent non-tailored materials to participants without personal contact. No co-interventions provided to intervention arms across trials.

c. Control: Brief leaflet considered to be a minimal print-based self-help intervention by authors. Behavioural co-intervention provided in all studies.

d. One study was a cluster RCT.

e. 100% of evidence with bias issues: 74% at high risk of bias for at least one domain (biochemical validation with or without selection bias) and with most also at unclear risk for selection bias with or without issues with attrition. Remaining evidence (26%) at unclear risk of selection bias only. We downrate this domain by -1.5.

f. Point estimates similar and confidence intervals overlap except one outlier study contributing very little to the total (I2= 21%; p=0.27). We do not downrate this domain.

g. No indirectness.

h. Confidence interval encompasses one range of effect (small but important harm to little to no difference). The optimal information size is met (total of 487 events) and adequate sample size. We do not downrate this domain.

i. Search was not completely comprehensive, but most trials report negative findings. We do not downrate this domain.

## Appendix K Table 39. Non-tailored print-based self-help materials (with face-to-face contact) versus No intervention or leaflet only: Smoking cessation in general/mixed population of smokers

| Non-tailored print-based self-help materials given (with face-to-face contact) compared to no intervention or leaflet only in general/mixed population of smokers  **Bibliography:** Livingstone-Banks 2019; Date of last search: March 2018 | | | | | | | | | | | |
| --- | --- | --- | --- | --- | --- | --- | --- | --- | --- | --- | --- |
| **Certainty assessment** | | | | | | | **Summary of findings** | | | | |
| **№ of participants (studies) Follow-up** | **Risk of bias** | **Inconsistency** | **Indirectness** | **Imprecision** | **Publication bias** | **Overall certainty of evidence** | **Study event rates (%)** | | **Relative effect (95% CI)** | **Anticipated absolute effects** | |
|  |  |  |  |  |  |  | **With no intervention or leaflet only** | **With Non-tailored print-based self-help materials given (with face-to-face contact)** |  | **Risk with no intervention or leaflet only** | **Risk difference with Non-tailored print-based self-help materials given (with face-to-face contact)** |
| **Abstinence/Cessation (follow up: 6+ months)** ^a,b,c^  Outcome measurement: point prevalence: 50%, continuous/sustained abstinence 25%, unclear/NR 25% studies  Biochemical validation: 25% studies | | | | | | | | | | | |
| 2822 (4 RCTs) ^d^ | very serious ^e^ | not serious ^f^ | not serious ^g^ | not serious ^h^ | none ^i^ | ⨁⨁◯◯ LOW | 67/1426 (4.7%) | 107/1396 (7.7%) | RR 1.39 (1.03 to 1.88) | 47 per 1,000 | **18 more per 1,000** (from 1 more to 41 more) |

**CI:** Confidence interval; **NR:** Not reported; **RR:** Risk ratio

#### Explanations

a. All trials recruited general smokers.

b. Intervention: Investigators gave participants the materials in-person but did not provide advice to stop smoking. Most studies provided additional behavioural co-intervention.

c. Control: No intervention provided to control participants in three trials. One trial provided materials that were not specific to smoking plus a video focused on cholesterol education.

d. One trial was quasi-randomized and another was a cluster RCT.

e. 100% of the evidence at high risk for at least one domain, namely, selection bias, performance/detection bias, or biochemical validation; of this, a subset of evidence (69%) is at high risk for two or more domains. We downrate this domain by -2.0.

f. Point estimates similar and confidence intervals overlap except one outlier study contributing very little to the total (I2= 21%; p=0.27). We do not downrate this domain.

g. 24% of the evidence is indirect (medical specialist setting). We downrate this domain by -0.5.

h. Confidence interval encompasses two ranges of effect (little to no difference to moderate benefit). The optimal information size not met (total of 174 events), but sample size is adequate (>2000 participants). We downrate this domain by -0.5.

i. Search was not completely comprehensive, but all trials report negative findings. We do not downrate this domain.

| Summary of findings: | | | | | | |
| --- | --- | --- | --- | --- | --- | --- |
| **Non-tailored print-based self-help materials given (with face-to-face contact) compared to no intervention or leaflet only in general/mixed population of smokers** | | | | | | |
| **Patient or population**: general/mixed population of smokers  **Setting**: No restriction  **Intervention**: Non-tailored print-based self-help materials given (with face-to-face contact)  **Comparison**: No intervention or leaflet only | | | | | | |
| Outcomes | **Anticipated absolute effects^*^** (95% CI) | | Relative effect (95% CI) | № of participants  (studies) | Certainty of the evidence (GRADE) | Comments |
|  | **Risk with no intervention or leaflet only** | **Risk with Non-tailored print-based self-help materials given (with face-to-face contact)** |  |  |  |  |
| Abstinence/Cessation ^a,b,c^  Outcome measurement: point prevalence: 50%, continuous/sustained abstinence 25%, unclear/NR 25% studies  Biochemical validation: 25% studies  Follow up: 6+ months | 47 per 1,000 | **65 per 1,000** (48 to 88) | RR 1.39 (1.03 to 1.88) | 2822 (4 RCTs) ^d^ | ⨁⨁◯◯ LOW ^e,f,g,h,i^ | AMSTAR-2: Moderate.  Date of last search: March 2018.  Not GRADEd by review authors. |
| ***The risk in the intervention group** (and its 95% confidence interval) is based on the assumed risk in the comparison group and the **relative effect** of the intervention (and its 95% CI).   **CI:** Confidence interval; **RR:** Risk ratio | | | | | | |
| **GRADE Working Group grades of evidence** **High certainty:** We are very confident that the true effect lies close to that of the estimate of the effect **Moderate certainty:** We are moderately confident in the effect estimate: The true effect is likely to be close to the estimate of the effect, but there is a possibility that it is substantially different **Low certainty:** Our confidence in the effect estimate is limited: The true effect may be substantially different from the estimate of the effect **Very low certainty:** We have very little confidence in the effect estimate: The true effect is likely to be substantially different from the estimate of effect | | | | | | |

#### Explanations

a. All trials recruited general smokers.

b. Intervention: Investigators gave participants the materials in-person but did not provide advice to stop smoking. Most studies provided additional behavioural co-intervention.

c. Control: No intervention provided to control participants in three trials. One trial provided materials that were not specific to smoking plus a video focused on cholesterol education.

d. One trial was quasi-randomized and another was a cluster RCT.

e. 100% of the evidence at high risk for at least one domain, namely, selection bias, performance/detection bias, or biochemical validation; of this, a subset of evidence (69%) is at high risk for two or more domains. We downrate this domain by -2.0.

f. Some variation in point estimates but confidence intervals largely overlap (I2= 0%; p=0.73). We do not downrate this domain.

g. 24% of the evidence is indirect (medical specialist setting). We downrate this domain by -0.5.

h. Confidence interval encompasses two ranges of effect (little to no difference to moderate benefit). The optimal information size not met (total of 174 events), but sample size is adequate (>2000 participants). We downrate this domain by -0.5.

i. Search was not completely comprehensive, but all trials report negative findings. We do not downrate this domain.

## Appendix K Table 40. Individually tailored print-based self-help materials (no face-to-face) versus No materials/no intervention: Smoking cessation in general/mixed population of smokers

| Individually tailored print-based self-help materials (no face-to-face contact) compared to no materials/no intervention in general/mixed population of smokers  **Bibliography:** Livingstone-Banks 2019; Date of last search: March 2018 | | | | | | | | | | | |
| --- | --- | --- | --- | --- | --- | --- | --- | --- | --- | --- | --- |
| **Certainty assessment** | | | | | | | **Summary of findings** | | | | |
| **№ of participants (studies) Follow-up** | **Risk of bias** | **Inconsistency** | **Indirectness** | **Imprecision** | **Publication bias** | **Overall certainty of evidence** | **Study event rates (%)** | | **Relative effect (95% CI)** | **Anticipated absolute effects** | |
|  |  |  |  |  |  |  | **With no materials/no intervention** | **With Individually tailored print-based self-help materials (no face-to-face contact)** |  | **Risk with no materials/no intervention** | **Risk difference with Individually tailored print-based self-help materials (no face-to-face contact)** |
| **Abstinence/Cessation (follow up: 6+ months)** ^a,b,c^  Outcome measurement: point prevalence 10%, continuous/sustained abstinence 80%, unclear/NR 10% studies  Biochemical validation: 0% studies | | | | | | | | | | | |
| 14359 (10 RCTs) ^d^ | very serious ^e^ | not serious ^f^ | not serious ^g^ | not serious ^h^ | none ^i^ | ⨁⨁◯◯  LOW | 455/7573 (6.0%) | 501/6786 (7.4%) | RR 1.34 (1.19 to 1.51) | 60 per 1,000 | **20 more per 1,000** (from 11 more to 31 more) |

**CI:** Confidence interval; **NR:** Not reported; **RR:** Risk ratio

#### Explanations

a. As reported by review authors, participants in trials were not selected for interest in quitting smoking. Majority of trials recruited general smokers (n=8) with remainder recruiting smokers motivated to quit (n=1) or smokers not motivated to quit (n=1).

b. Intervention: Materials were tailored to the individual’s characteristics; several trials used computerized expert systems with tailoring according to baseline data. In all trials, materials were sent to participants without personal contact. No co-interventions provided.

c. Control: Across trials, control conditions included assessment only, thank you letters only, and no intervention or information. No co-interventions provided to control participants.

d. One trial was quasi-randomized.

e. 100% of evidence with substantial bias issues, namely pertaining to selection, performance/detection, and biochemical validation: 44% of evidence at high risk for two domains and at unclear risk for at least one domain, 53% at high risk for one domain and unclear risk for at least two domains, and 3% at high risk for one domain but at low risk for all others. Biochemical validation was high risk for all studies. We downrate this domain by -2.0.

f. Little variation in risk estimates and confidence intervals largely overlap (I2=0%, p=0.93). We do not downrate this domain.

g. Due to reporting, extent of indirectness could not be determined for one trial contributing 23% of weight to the analysis; the study recruited smokers >15 years of age but did not report the proportion of adolescents included. No indirectness in remaining studies. We do not downrate this domain.

h. Confidence interval encompasses small but important benefit. The optimal information size is met (total of 956 events) and adequate sample size. We do not downrate this domain.

i. Search strategy is not completely comprehensive; however, most trials report negative findings and review authors state that no evidence of asymmetry for all analyses assessed (i.e., those with at least 10 studies). We do not downrate this domain.

| Summary of findings: | | | | | | |
| --- | --- | --- | --- | --- | --- | --- |
| **Individually tailored print-based self-help materials (no face-to-face contact) compared to no materials/no intervention in general/mixed population of smokers** | | | | | | |
| **Patient or population**: General/mixed population of smokers  **Setting**: No restriction  **Intervention**: Individually tailored print-based self-help materials (no face-to-face contact)  **Comparison**: No materials/no intervention | | | | | | |
| Outcomes | **Anticipated absolute effects^*^** (95% CI) | | Relative effect (95% CI) | № of participants  (studies) | Certainty of the evidence (GRADE) | Comments |
|  | **Risk with no materials/no intervention** | **Risk with Individually tailored print-based self-help materials (no face-to-face contact)** |  |  |  |  |
| Abstinence/Cessation ^a,b,c^  Outcome measurement: point prevalence 10%, continuous/sustained abstinence 80%, unclear/NR 10% studies  Biochemical validation: 0% studies  Follow up: 6+ months | 60 per 1,000 | **81 per 1,000** (71 to 91) | RR 1.34 (1.19 to 1.51) | 14359 (10 RCTs) ^d^ | ⨁⨁◯◯  LOW ^e,f,g,h,i^ | Authors performed this analysis as a subgroup analysis; may be plausible but unlikely.  AMSTAR-2: Moderate.  Date of last search: March 2018.  Review authors rate as moderate certainty. ^j^ |
| ***The risk in the intervention group** (and its 95% confidence interval) is based on the assumed risk in the comparison group and the **relative effect** of the intervention (and its 95% CI).   **CI:** Confidence interval; **NR:** Not reported; **RR:** Risk ratio | | | | | | |
| **GRADE Working Group grades of evidence** **High certainty:** We are very confident that the true effect lies close to that of the estimate of the effect **Moderate certainty:** We are moderately confident in the effect estimate: The true effect is likely to be close to the estimate of the effect, but there is a possibility that it is substantially different **Low certainty:** Our confidence in the effect estimate is limited: The true effect may be substantially different from the estimate of the effect **Very low certainty:** We have very little confidence in the effect estimate: The true effect is likely to be substantially different from the estimate of effect | | | | | | |

#### Explanations

a. As reported by review authors, participants in trials were not selected for interest in quitting smoking. Majority of trials recruited general smokers (n=8) with remainder recruiting smokers motivated to quit (n=1) or smokers not motivated to quit (n=1).

b. Intervention: Materials were tailored to the individual’s characteristics; several trials used computerized expert systems with tailoring according to baseline data. In all trials, materials were sent to participants without personal contact. No co-interventions provided.

c. Control: Across trials, control conditions included assessment only, thank you letters only, and no intervention or information. No co-interventions provided to control participants.

d. One trial was quasi-randomized.

e. 100% of evidence with substantial bias issues, namely pertaining to selection, performance/detection, and biochemical validation: 44% of evidence at high risk for two domains and at unclear risk for at least one domain, 53% at high risk for one domain and unclear risk for at least two domains, and 3% at high risk for one domain but at low risk for all others. Biochemical validation was high risk for all studies. We downrate this domain by -2.0.

f. Little variation in risk estimates and confidence intervals largely overlap (I2=0%, p=0.93). We do not downrate this domain.

g. Due to reporting, extent of indirectness could not be determined for one trial contributing 23% of weight to the analysis; the study recruited smokers >15 years of age but did not report the proportion of adolescents included. No indirectness in remaining studies. We do not downrate this domain.

h. Confidence interval encompasses small but important benefit. The optimal information size is met (total of 956 events) and adequate sample size. We do not downrate this domain.

i. Search strategy is not completely comprehensive; however, most trials report negative findings and review authors state that no evidence of asymmetry for all analyses assessed (i.e., those with at least 10 studies). We do not downrate this domain.

j. Review authors rate as moderate certainty (downrated one level for risk of bias as all but one study at high or unclear risk of bias).

# Matkin 2019 {1228}

## Appendix K Table 41. Hotline and self-help materials versus Minimal intervention in general/mixed population of smokers

| Hotline and self-help materials compared to minimal intervention in general/mixed population of smokers  **Bibliography:** Matkin 2019; Date of last search: May 2018 | | | | | | | | | | | |
| --- | --- | --- | --- | --- | --- | --- | --- | --- | --- | --- | --- |
| **Certainty assessment** | | | | | | | **Summary of findings** | | | | |
| **№ of participants (studies) Follow-up** | **Risk of bias** | **Inconsistency** | **Indirectness** | **Imprecision** | **Publication bias** | **Overall certainty of evidence** | **Study event rates (%)** | | **Relative effect (95% CI)** | **Anticipated absolute effects** | |
|  |  |  |  |  |  |  | **With Minimal intervention** | **With Hotline and self-help materials** |  | **Risk with Minimal intervention** | **Risk difference with Hotline and self-help materials** |
| Abstinence/Cessation (follow up: 12-18 months) ^a,b,c^  Outcome assessment: continuous/sustained abstinence 100% studies  Biochemical validation: 50% studies | | | | | | | | | | | |
| 3327 (2 RCTs) | serious ^d^ | not serious ^e^ | not serious ^f^ | not serious ^g^ | none ^h^ | ⨁⨁◯◯  LOW | 54/1597 (3.4%) | 93/1730 (5.4%) | **RR 1.62** (1.16 to 2.25) | 34 per 1,000 | **21 more per 1,000** (from 5 more to 42 more) |

**CI:** Confidence interval; **RR:** Risk ratio

#### Explanations

a. One study included smokers motivated to quit/wishing to quit, and the other was a general population (i.e., not defined).

b. Hotline and self-help materials: One study was a combination intervention: promoted 24-hour hotline, daytime access to counsellors, and provision of American Lung Association self-help (ALA S-H) manuals. The second study comprised Quitline proactive contact, quit kits (national Quitline printed resources), individual face-to-face counselling with practice nurse, and three proactive telephone calls from an experienced counsellor. Pharmacotherapy (NRT patch) co-intervention offered in one of the two trials.

c. Minimal intervention: Review authors report the control as 'minimal intervention'. This consists of self-help manual in one trial (ALA S-H manual). In the second trial, primary care providers’ delivered their usual care, such as advice, referral to Quitline, or both. Pharmacotherapy (NRT patch) co-intervention in one of the two trials).

d. Evidence at high risk for performance bias due to nature of intervention and unclear risk for selection bias. 38% of evidence also at high risk for self-reported smoking. We downrate by -1.5.

e. Little variation in point estimates, and the confidence intervals overlap (I2=0%; p=0.73). We do not downrate this domain.

f. No indirectness. We do not downrate this domain.

g. Confidence interval encompasses two ranges of effect (little to no difference to moderate benefit). The optimal information size not met (total of 147 events), but adequate sample size. We downrate this domain by -0.5.

h. Search was not comprehensive but mixed results among studies. We do not downrate this domain.

| Summary of findings: | | | | | | |
| --- | --- | --- | --- | --- | --- | --- |
| **Hotline and self-help materials compared to minimal intervention in general/mixed population of smokers** | | | | | | |
| **Patient or population**: General/mixed population of smokers  **Setting**: No restriction  **Intervention**: Hotline and self-help materials  **Comparison**: Minimal intervention | | | | | | |
| Outcomes | **Anticipated absolute effects^*^** (95% CI) | | Relative effect (95% CI) | № of participants  (studies) | Certainty of the evidence (GRADE) | Comments |
|  | **Risk with Minimal intervention** | **Risk with Hotline and self-help materials** |  |  |  |  |
| Abstinence/Cessation ^a,b,c^  Outcome assessment: continuous/sustained abstinence 100% studies  Biochemical validation: 50% studies  Follow up: 12-18 months | 34 per 1,000 | **55 per 1,000** (39 to 76) | **RR 1.62** (1.16 to 2.25) | 3327 (2 RCTs) | ⨁⨁◯◯  LOW ^d,e,f,g,h^ | Date of last search: May 2018  AMSTAR-2: Low  Not GRADEd by review authors |
| ***The risk in the intervention group** (and its 95% confidence interval) is based on the assumed risk in the comparison group and the **relative effect** of the intervention (and its 95% CI).   **CI:** Confidence interval; **RR:** Risk ratio | | | | | | |
| **GRADE Working Group grades of evidence** **High certainty:** We are very confident that the true effect lies close to that of the estimate of the effect **Moderate certainty:** We are moderately confident in the effect estimate: The true effect is likely to be close to the estimate of the effect, but there is a possibility that it is substantially different **Low certainty:** Our confidence in the effect estimate is limited: The true effect may be substantially different from the estimate of the effect **Very low certainty:** We have very little confidence in the effect estimate: The true effect is likely to be substantially different from the estimate of effect | | | | | | |

#### Explanations

a. One study included smokers motivated to quit/wishing to quit, and the other was a general population (i.e., not defined)

b. Hotline and self-help materials: One study was a combination intervention: promoted 24-hour hotline, daytime access to counsellors, and provision of American Lung Association self-help (ALA S-H) manuals. The second study comprised Quitline proactive contact, quit kits (national Quitline printed resources), individual face-to-face counselling with practice nurse, and three proactive telephone calls from an experienced counsellor. Pharmacotherapy (NRT patch) co-intervention offered in one of the two trials.

c. Minimal intervention: Review authors report the control as 'minimal intervention'. This consists of self-help manual in one trial (ALA S-H manual). In the second trial, primary care providers’ delivered their usual care, such as advice, referral to Quitline, or both. Pharmacotherapy (NRT patch) co-intervention in one of the two trials.

d. Evidence at high risk for performance bias due to nature of intervention and unclear risk for selection bias. 38% of evidence also at high risk for self-reported smoking. We downrate by -1.5.

e. Little variation in point estimates, and the confidence intervals overlap (I2=0%; p=0.73). We do not downrate this domain.

f. No indirectness. We do not downrate this domain.

g. Confidence interval encompasses two ranges of effect (little to no difference to moderate benefit). The optimal information size not met (total of 147 events), but adequate sample size. We downrate this domain by -0.5.

h. Search was not comprehensive but mixed results among studies. We do not downrate this domain.

## Appendix K Table 42. Intensive telephone counselling versus Minimal telephone counselling in general/mixed population of smokers

| Intensive telephone counselling compared to minimal telephone counselling in general/mixed population of smokers  **Bibliography:** Matkin 2019; Date of last search: May 2018 | | | | | | | | | | | |
| --- | --- | --- | --- | --- | --- | --- | --- | --- | --- | --- | --- |
| **Certainty assessment** | | | | | | | **Summary of findings** | | | | |
| **№ of participants (studies) Follow-up** | **Risk of bias** | **Inconsistency** | **Indirectness** | **Imprecision** | **Publication bias** | **Overall certainty of evidence** | **Study event rates (%)** | | **Relative effect (95% CI)** | **Anticipated absolute effects** | |
|  |  |  |  |  |  |  | **With Minimal intervention** | **With Intensive telephone counselling** |  | **Risk with Minimal intervention** | **Risk difference with Intensive telephone counselling** |
| Abstinence/cessation (follow up: 6+ months) ^a,b,c^  Outcome assessment: point prevalence 67%, continuous/sustained abstinence 33% studies  Biochemical validation: 33% studies | | | | | | | | | | | |
| 2602 (3 RCTs) | very serious ^d^ | not serious ^e^ | not serious ^f^ | not serious ^g^ | none ^h^ | ⨁◯◯◯  VERY LOW | 299/1260 (23.7%) | 402/1342 (30.0%) | **RR 1.27** (1.12 to 1.44) | 237 per 1,000 | **64 more per 1,000** (from 28 more to 104 more) |

**CI:** Confidence interval; **RR:** Risk ratio

#### Explanations

a. Mix of trials in this analysis: largest trials in an undefined (general) population and remaining studies in smokers motivated to quit.

b. Intensive telephone counselling: moderate intensity of three to five calls across studies. Behavioural co-intervention provided in one trial; a second trial provided behavioural and pharmacotherapy to participants.

c. Minimal intervention: Minimal telephone counselling/low-intensity (single call) telephone counselling. Behavioural co-intervention provided in one trial; in a second trial, behavioural and pharmacotherapy co-intervention provided to participants.

d. Evidence at risk for performance bias due to the nature of the interventions. In addition, the majority of the evidence (66%) at risk for detection bias. We downrate by -2.0.

e. Little variation in point estimates, and the confidence intervals overlap (I2= 0%; p=0.80). We do not downrate this domain.

f. 33.4% of the evidence is indirect due to setting (inpatient). We downrate this domain by -0.5.

g. Confidence interval encompasses two ranges of effect (small but important benefit to large benefit). The optimal information size is met (total of 701 events) and adequate sample size. We downrate this domain by -0.5.

h. Search was not comprehensive but a mix of results among trials. We do not downrate this domain.

| Summary of findings: | | | | | | |
| --- | --- | --- | --- | --- | --- | --- |
| **Intensive telephone counselling compared to minimal telephone counselling in general/mixed population of smokers** | | | | | | |
| **Patient or population**: General/mixed population of smokers  **Setting**: No restriction  **Intervention**: Intensive telephone counselling  **Comparison**: Minimal telephone counselling | | | | | | |
| Outcomes | **Anticipated absolute effects^*^** (95% CI) | | Relative effect (95% CI) | № of participants  (studies) | Certainty of the evidence (GRADE) | Comments |
|  | **Risk with Minimal intervention** | **Risk with Intensive telephone counselling** |  |  |  |  |
| Abstinence/cessation ^a,b,c^  Outcome assessment: point prevalence 67%, continuous/sustained abstinence 33% studies  Biochemical validation: 33%, studies  Follow up: 6+ months | 237 per 1,000 | **301 per 1,000** (266 to 342) | **RR 1.27** (1.12 to 1.44) | 2602 (3 RCTs) | ⨁◯◯◯  VERY LOW ^d,e,f,g,h^ | Date of last search: May 2018  AMSTAR-2: Low  Not GRADEd by review authors |
| ***The risk in the intervention group** (and its 95% confidence interval) is based on the assumed risk in the comparison group and the **relative effect** of the intervention (and its 95% CI).   **CI:** Confidence interval; **RR:** Risk ratio | | | | | | |
| **GRADE Working Group grades of evidence** **High certainty:** We are very confident that the true effect lies close to that of the estimate of the effect **Moderate certainty:** We are moderately confident in the effect estimate: The true effect is likely to be close to the estimate of the effect, but there is a possibility that it is substantially different **Low certainty:** Our confidence in the effect estimate is limited: The true effect may be substantially different from the estimate of the effect **Very low certainty:** We have very little confidence in the effect estimate: The true effect is likely to be substantially different from the estimate of effect | | | | | | |

#### Explanations

a. Mix of trials in this analysis: largest trials in an undefined (general) population and remaining studies in smokers motivated to quit.

b. Intensive telephone counselling: moderate intensity of three to five calls across studies. Behavioural co-intervention provided in one trial; a second trial provided behavioural and pharmacotherapy to participants.

c. Minimal intervention: Minimal telephone counselling/low-intensity (single call) telephone counselling. Behavioural co-intervention provided in one trial; in a second trial, behavioural and pharmacotherapy co-intervention provided to participants.

d. Evidence at risk for performance bias due to the nature of the interventions. In addition, the majority of the evidence (66%) at risk for detection bias. We downrate by -2.0.

e. Little variation in point estimates, and the confidence intervals overlap (I2= 0%; p=0.80). We do not downrate this domain.

f. 33.4% of the evidence is indirect due to setting (inpatient). We downrate this domain by -0.5.

g. Confidence interval encompasses two ranges of effect (small but important benefit to large benefit). The optimal information size is met (total of 701 events) and adequate sample size. We downrate this domain by -0.5.

h. Search was not comprehensive but a mix of results among trials. We do not downrate this domain.

## Appendix K Table 43. Brief motivational telephone counselling versus Usual care telephone call in general/mixed population of smokers

| Brief motivational telephone counselling compared to usual care telephone call in general/mixed population of smokers  **Bibliography:** Matkin 2019; Date of last search: May 2018 | | | | | | | | | | | |
| --- | --- | --- | --- | --- | --- | --- | --- | --- | --- | --- | --- |
| **Certainty assessment** | | | | | | | **Summary of findings** | | | | |
| **№ of participants (studies) Follow-up** | **Risk of bias** | **Inconsistency** | **Indirectness** | **Imprecision** | **Publication bias** | **Overall certainty of evidence** | **Study event rates (%)** | | **Relative effect (95% CI)** | **Anticipated absolute effects** | |
|  |  |  |  |  |  |  | **With Usual care telephone call** | **With Brief motivational telephone counselling** |  | **Risk with Usual care telephone call** | **Risk difference with Brief motivational telephone counselling** |
| Abstinence/Cessation (follow up: 12 months) ^a,b^  Outcome assessment: point prevalence 100% studies  Biochemical validation: 0% studies | | | | | | | | | | | |
| 374 (1 RCT) | very serious ^c^ | not serious ^d^ | not serious ^e^ | very serious ^f^ | none ^g^ | ⨁◯◯◯  VERY LOW | 7/189 (3.7%) | 18/185 (9.7%) | **RR 2.63** (1.12 to 6.14) | 37 per 1,000 | **60 more per 1,000** (from 4 more to 190 more) |

**CI:** Confidence interval; **RR:** Risk ratio

#### Explanations

a. Brief motivational telephone counselling: Three 15-minute calls (baseline and weeks 2 and 4). No co-interventions provided.

b. Usual care telephone call: One five-minute call. No co-intervention provided.

c. High risk of performance and detection bias. Unclear risk for allocation concealment. We downrate by -2.0.

d. One study included in analysis. We do not downrate this domain.

e. No indirectness. We do not downrate this domain.

f. Confidence interval encompasses three ranges of effect (little to no difference to large benefit). The optimal information size not met (total of 25 events) and inadequate sample size (<2000 participants). We downrate this domain by -2.0.

g. Search was not comprehensive, and one study found. Lack of additional studies may be due to reasons other than publication bias. We do not downrate this domain

| Summary of findings: | | | | | | |
| --- | --- | --- | --- | --- | --- | --- |
| **Brief motivational telephone counselling compared to usual care telephone call in general/mixed population of smokers** | | | | | | |
| **Patient or population**: General/mixed population of smokers  **Setting**: No restriction  **Intervention**: Brief motivational telephone counselling  **Comparison**: Usual care | | | | | | |
| Outcomes | **Anticipated absolute effects^*^** (95% CI) | | Relative effect (95% CI) | № of participants  (studies) | Certainty of the evidence (GRADE) | Comments |
|  | **Risk with Usual care telephone call** | **Risk with Brief motivational telephone counselling** |  |  |  |  |
| Abstinence/Cessation ^a,b^  Outcome assessment: point prevalence 100% studies  Biochemical validation: 0% studies  Follow up: 12 months | 37 per 1,000 | **97 per 1,000** (41 to 227) | **RR 2.63** (1.12 to 6.14) | 374 (1 RCT) | ⨁◯◯◯  VERY LOW ^c,d,e,f,g^ | Date of last search: May 2018  AMSTAR-2: Low  Not GRADEd by review authors |
| ***The risk in the intervention group** (and its 95% confidence interval) is based on the assumed risk in the comparison group and the **relative effect** of the intervention (and its 95% CI).   **CI:** Confidence interval; **RR:** Risk ratio | | | | | | |
| **GRADE Working Group grades of evidence** **High certainty:** We are very confident that the true effect lies close to that of the estimate of the effect **Moderate certainty:** We are moderately confident in the effect estimate: The true effect is likely to be close to the estimate of the effect, but there is a possibility that it is substantially different **Low certainty:** Our confidence in the effect estimate is limited: The true effect may be substantially different from the estimate of the effect **Very low certainty:** We have very little confidence in the effect estimate: The true effect is likely to be substantially different from the estimate of effect | | | | | | |

#### Explanations

a. Brief motivational telephone counselling: Three 15-minute calls (baseline and weeks 2 and 4). No co-interventions provided.

b. Usual care telephone call: One five-minute call. No co-interventions provided.

c. High risk of performance and detection bias. Unclear risk for allocation concealment. We downrate by -2.0.

d. One study included in analysis. We do not downrate this domain.

e. No indirectness. We do not downrate this domain.

f. Confidence interval encompasses three ranges of effect (little to no difference to large benefit). The optimal information size not met (total of 25 events) and inadequate sample size (<2000 participants). We downrate this domain by -2.0.

g. Search was not comprehensive, and one study found. Lack of additional studies may be due to reasons other than publication bias. We do not downrate this domain.

## Appendix K Table 44. Telephone counselling for smoking reduction versus Usual care telephone call in general/mixed population of smokers

| Telephone counselling for smoking reduction compared to usual care telephone call in the general/mixed population  **Bibliography:** Matkin 2019; Date of last search: May 2018 | | | | | | | | | | | |
| --- | --- | --- | --- | --- | --- | --- | --- | --- | --- | --- | --- |
| **Certainty assessment** | | | | | | | **Summary of findings** | | | | |
| **№ of participants (studies) Follow-up** | **Risk of bias** | **Inconsistency** | **Indirectness** | **Imprecision** | **Publication bias** | **Overall certainty of evidence** | **Study event rates (%)** | | **Relative effect (95% CI)** | **Anticipated absolute effects** | |
|  |  |  |  |  |  |  | **With Usual care telephone call** | **With Telephone counselling for smoking reduction** |  | **Risk with Usual care telephone call** | **Risk difference with Telephone counselling for smoking reduction** |
| Abstinence/cessation (follow up: 12 months) ^a,b^  Outcome assessment: point prevalence 100% studies  Biochemical validation: 0% studies | | | | | | | | | | | |
| 375 (1 RCT) | very serious ^c^ | not serious ^d^ | not serious ^e^ | very serious ^f^ | none ^g^ | ⨁◯◯◯  VERY LOW | 7/189 (3.7%) | 16/186 (8.6%) | **RR 2.32** (0.98 to 5.52) | 37 per 1,000 | **49 more per 1,000** (from 1 fewer to 167 more) |

**CI:** Confidence interval; **RR:** Risk ratio

#### Explanations

a. Telephone counselling for smoking reduction: Three 15-minute calls (baseline and weeks 2 and 4. No co-interventions provided.

b. Usual care telephone call: One five-minute call. No co-interventions provided.

c. High risk of performance and detection bias. Unclear risk for allocation concealment. We downrate by -2.0.

d. One study included in analysis. We do not downrate this domain.

e. No indirectness. We do not downrate this domain.

f. Confidence interval encompasses three ranges of effect (little to no difference to large benefit). The optimal information size not met (total of 23 events) and inadequate sample size (<2000 participants). We downrate this domain by -2.0.

g. Search was not comprehensive, and one study found. Lack of additional studies may be due to reasons other than publication bias. We do not downrate this domain.

| Summary of findings: | | | | | | |
| --- | --- | --- | --- | --- | --- | --- |
| **Telephone counselling for smoking reduction compared to usual care telephone call in general/mixed population of smokers** | | | | | | |
| **Patient or population**: General/mixed population of smokers  **Setting**: No restriction  **Intervention**: Telephone counselling for smoking reduction  **Comparison**: Usual care telephone call | | | | | | |
| Outcomes | **Anticipated absolute effects^*^** (95% CI) | | Relative effect (95% CI) | № of participants  (studies) | Certainty of the evidence (GRADE) | Comments |
|  | **Risk with Usual care telephone call** | **Risk with Telephone counselling for smoking reduction** |  |  |  |  |
| Abstinence/cessation ^a,b^  Outcome assessment: point prevalence 100% studies  Biochemical validation: 0% studies  Follow up: 12 months | 37 per 1,000 | **86 per 1,000** (36 to 204) | **RR 2.32** (0.98 to 5.52) | 375 (1 RCT) | ⨁◯◯◯  VERY LOW ^c,d,e,f,g^ | Date of last search: May 2018  AMSTAR-2: Low  Not GRADEd by review authors |
| ***The risk in the intervention group** (and its 95% confidence interval) is based on the assumed risk in the comparison group and the **relative effect** of the intervention (and its 95% CI).   **CI:** Confidence interval; **RR:** Risk ratio | | | | | | |
| **GRADE Working Group grades of evidence** **High certainty:** We are very confident that the true effect lies close to that of the estimate of the effect **Moderate certainty:** We are moderately confident in the effect estimate: The true effect is likely to be close to the estimate of the effect, but there is a possibility that it is substantially different **Low certainty:** Our confidence in the effect estimate is limited: The true effect may be substantially different from the estimate of the effect **Very low certainty:** We have very little confidence in the effect estimate: The true effect is likely to be substantially different from the estimate of effect | | | | | | |

#### Explanations

a. Telephone counselling for smoking reduction: Three 15-minute calls (baseline and weeks 2 and 4). No co-interventions provided.

b. Usual care telephone call: One five-minute call. No co-interventions provided.

c. High risk of performance and detection bias. Unclear risk for allocation concealment. We downrate by -2.0.

d. One study included in analysis. We do not downrate this domain.

e. No indirectness. We do not downrate this domain.

f. Confidence interval encompasses three ranges of effect (little to no difference to large benefit). The optimal information size not met (total of 23 events) and inadequate sample size (<2000 participants). We downrate this domain by -2.0.

g. Search was not comprehensive, and one study found. Lack of additional studies may be due to reasons other than publication bias. We do not downrate this domain.

# Posadzki 2016 {659}

## Appendix K Table 45. Interactive voice response (IVR) systems versus No intervention: Smoking cessation in general/mixed population of smokers

| Interactive voice response (IVR) systems compared to no intervention in general/mixed population of smokers  **Bibliography:** Posadzki 2016; Date of last search: May/June 2015 | | | | | | | | | | | |
| --- | --- | --- | --- | --- | --- | --- | --- | --- | --- | --- | --- |
| **Certainty assessment** | | | | | | | **Summary of findings** | | | | |
| **№ of participants (studies) Follow-up** | **Risk of bias** | **Inconsistency** | **Indirectness** | **Imprecision** | **Publication bias** | **Overall certainty of evidence** | **Study event rates (%)** | | **Relative effect (95% CI)** | **Anticipated absolute effects** | |
|  |  |  |  |  |  |  | **With no intervention** | **With Interactive voice response (IVR) systems** |  | **Risk with no intervention** | **Risk difference with Interactive voice response (IVR) systems** |
| **Abstinence/Cessation (follow up: 24 months)** ^a,b,c^  Outcome measurement: unclear/NR 100% studies  Biochemical validation: 100% studies | | | | | | | | | | | |
| Unclear/NR (1 RCT) | serious ^d^ | not serious ^e^ | not serious ^f^ | not serious to very serious ^g^ | none ^h^ | unable to assess | Little to no difference between groups at 24-month follow-up (21.7% of intervention group versus 42.9% of control group; P = 0.13). | | | | |

**ATCS:** Automated telephone communication system; **CI:** Confidence interval; **IVR:** Interactive voice response; **NR:** Not reported

#### Explanations

a. The only trial in this analysis recruited general smokers.

b. Intervention: IVR is a type of automated telephone communication systems (ATCS). Participants received IVR calls on their quit date, on days 3, 8, and 11, and then every two weeks between weeks 13 and 52. Calls were 3 to 5 minutes long. Prior to randomization, participants had received varenicline for 12 weeks (0.5 mg on days 1-3, 1 mg on days 4-7, and 2 mg until end of week 12) and IVR.

c. Control: In the first phase of the study (i.e., prior to randomization), participants received varenicline for 12 weeks (0.5 mg on days 1-3, 1 mg on days 4-7, and 2 mg until end of week 12) and IVR. After randomization, participants in control group no longer received IVR.

d. The only trial in this analysis is at unclear risk for selection, attrition, selective reporting, performance and detection bias. We downrate this domain by -1.0.

e. Not applicable - single study.

f. No indirectness.

g. Unable to assess confidence intervals. Unable to assess optimal information size and unclear sample size analyzed. We cannot rate this domain.

h. Search was comprehensive and the only trial in this analysis reports negative findings. We do not downrate this domain.

| Summary of findings: | | | | | | |
| --- | --- | --- | --- | --- | --- | --- |
| **Interactive voice response (IVR) systems compared to no intervention in general/mixed population of smokers** | | | | | | |
| **Patient or population**: General/mixed population of smokers  **Setting**: No restriction  **Intervention**: Interactive voice response (IVR) systems  **Comparison**: No intervention | | | | | | |
| Outcomes | **Anticipated absolute effects^*^** (95% CI) | | Relative effect (95% CI) | № of participants  (studies) | Certainty of the evidence (GRADE) | Comments |
|  | **Risk with no intervention** | **Risk with Interactive voice response (IVR) systems** |  |  |  |  |
| Abstinence/Cessation ^a,b,c^  Outcome measurement: unclear/NR 100% studies  Biochemical validation: 100% studies  Follow up: 24 months | Little to no difference between groups at 24-month follow-up (21.7% of intervention group versus 42.9% of control group; P = 0.13). | |  | Unclear/NR  (1 RCT) | unable to assess ^d,e,f,g,h^ | AMSTAR-2: Critically low.  Date of last search: May/June 2015.  Review authors rated as low certainty. ^i^ |
| ***The risk in the intervention group** (and its 95% confidence interval) is based on the assumed risk in the comparison group and the **relative effect** of the intervention (and its 95% CI).   **ATCS:** Automated telephone communication system; **CI:** Confidence interval; **IVR:** Interactive voice response; **NR:** Not reported | | | | | | |
| **GRADE Working Group grades of evidence** **High certainty:** We are very confident that the true effect lies close to that of the estimate of the effect **Moderate certainty:** We are moderately confident in the effect estimate: The true effect is likely to be close to the estimate of the effect, but there is a possibility that it is substantially different **Low certainty:** Our confidence in the effect estimate is limited: The true effect may be substantially different from the estimate of the effect **Very low certainty:** We have very little confidence in the effect estimate: The true effect is likely to be substantially different from the estimate of effect | | | | | | |

#### Explanations

a. The only trial in this analysis recruited general smokers.

b. Intervention: IVR is a type of automated telephone communication systems (ATCS). Participants received IVR calls on their quit date, on days 3, 8, and 11, and then every two weeks between weeks 13 and 52. Calls were 3 to 5 minutes long. Prior to randomization, participants had received varenicline for 12 weeks (0.5 mg on days 1-3, 1 mg on days 4-7, and 2 mg until end of week 12) and IVR.

c. Control: In the first phase of the study (i.e., prior to randomization), participants received varenicline for 12 weeks (0.5 mg on days 1-3, 1 mg on days 4-7, and 2 mg until end of week 12) and IVR. After randomization, participants in control group no longer received IVR.

d. The only trial in this analysis is at unclear risk for selection, attrition, selective reporting, performance and detection bias. We downrate this domain by -1.0.

e. Not applicable - single study.

f. No indirectness.

g. Unable to assess confidence intervals. Unable to assess optimal information size and unclear sample size analyzed. We cannot rate this domain.

h. Search was comprehensive and the only trial in this analysis reports negative findings. We do not downrate this domain.

i. Review authors rated as low certainty; justification not reported.

# Stead 2013 {1998}

## Appendix K Table 46. Physician advice (minimal or intensive interventions) versus No advice (or usual care): Smoking cessation in general/mixed population of smokers

| Physician advice (minimal or intensive interventions) compared to no advice (or usual care) in general/mixed population of smokers  **Bibliography:** Stead 2013; Date of last search: January & February 2013 | | | | | | | | | | | |
| --- | --- | --- | --- | --- | --- | --- | --- | --- | --- | --- | --- |
| **Certainty assessment** | | | | | | | **Summary of findings** | | | | |
| **№ of participants (studies) Follow-up** | **Risk of bias** | **Inconsistency** | **Indirectness** | **Imprecision** | **Publication bias** | **Overall certainty of evidence** | **Study event rates (%)** | | **Relative effect (95% CI)** | **Anticipated absolute effects** | |
|  |  |  |  |  |  |  | **With no advice (or usual care)** | **With Physician advice (minimal or intensive interventions)** |  | **Risk with no advice (or usual care)** | **Risk difference with Physician advice (minimal or intensive interventions)** |
| **Abstinence/cessation (follow up: 6+ months)** ^a,b,c^  Outcome assessment: point prevalence 46%, continuous/sustained abstinence 42%, unclear/NR 12% studies  Biochemical validation 15% studies | | | | | | | | | | | |
| 22239 (26 RCTs) ^d^ | very serious ^e^ | not serious ^f^ | not serious ^g^ | not serious ^h^ | none ^i^ | ⨁⨁◯◯  LOW | 462/9656 (4.8%) | 1008/12583 (8.0%) | **RR 1.76** (1.58 to 1.96) | 48 per 1,000 | **36 more per 1,000** (from 28 more to 46 more) |

**COPD:** Chronic obstructive pulmonary disease; **CI:** Confidence interval; **NR:** Not reported; **RR:** Risk ratio

#### Explanations

a. Studies recruited general smokers (n=20), females only (n=2), smokers with COPD (n=1), and smokers >60 or >65 years (n=2).

b. Intervention: Physician advice involved a 'stop smoking' message delivered verbally. Interventions were defined as minimal if delivered during a single session of <20-minute duration plus up to one follow-up visit with or without a leaflet. Intensive interventions involved a longer initial consultation, use of additional materials other than a leaflet, or more than one follow-up visit. Pharmacotherapy co-intervention provided in some of the studies.

c. Control: Denoted as 'no advice (usual care)' by review authors. According to evidence tables, various control conditions across trials including smoking related written materials, 'control' (not further specified), 'no advice' (not further specified), self-monitoring of smoking, usual care, no intervention, and questionnaires. Additional behavioural and/or pharmacotherapy co-interventions provided to control participants in some studies.

d. Seven studies were cluster RCTs.

e. Review authors did not assess performance/detection bias so we consider this domain unclear for all trials. 100% of evidence with substantive bias issues mostly with selection bias and biochemical validation: 91% at high risk for two domains or at high risk for one domain and unclear risk for two or more domains (including attrition); 9% at high and unclear risk for one domain each or at unclear risk for two or more domains. We downrate this domain by -2.0.

f. Variation in point estimates, but confidence intervals mostly overlap (I2= 40%; p=0.02). We downrate this domain by -0.5.

g. 13% of the evidence is indirect (i.e., specialized medical setting, incentive provided for quitting). Due to reporting, extent of indirectness could not be ascertained for one study contributing 1% of weight to the meta-analysis; the study recruited participants >15 years but proportion of those less than 18 years of age is NR. We do not downrate this domain.

h. Confidence interval encompasses moderate benefit. The optimal information size is met (total of 1470 events) and adequate sample size. We do not downrate this domain.

i. Search was not comprehensive but mix of results across trials. We do not downrate this domain.

| Summary of findings: | | | | | | |
| --- | --- | --- | --- | --- | --- | --- |
| **Physician advice (minimal or intensive interventions) compared to no advice (or usual care) in general/mixed population of smokers** | | | | | | |
| **Patient or population**: General/mixed population of smokers  **Setting**: No restriction  **Intervention**: Physician advice (minimal or intensive interventions)  **Comparison**: No advice (or usual care) | | | | | | |
| Outcomes | **Anticipated absolute effects^*^** (95% CI) | | Relative effect (95% CI) | № of participants  (studies) | Certainty of the evidence (GRADE) | Comments |
|  | **Risk with no advice (or usual care)** | **Risk with Physician advice (minimal or intensive interventions)** |  |  |  |  |
| Abstinence/cessation ^a,b,c^  Outcome assessment: point prevalence 46%, continuous/sustained abstinence 42%, unclear/NR 12% studies  Biochemical validation 15% studies  Follow up: 6+ months | 48 per 1,000 | **84 per 1,000** (76 to 94) | **RR 1.76** (1.58 to 1.96) | 22239 (26 RCTs) ^d^ | ⨁⨁◯◯  LOW ^e,f,g,h,i^ | No difference between minimal and intensive advice according to subgroup analysis (Test for subgroup differences: Chi2 = 1.03, df = 1 (P = 0.31), I2 =3%).  Fixed effects analysis  Date of last search: January/February 2013.  AMSTAR-2: Critically low  Not GRADEd by review authors |
| ***The risk in the intervention group** (and its 95% confidence interval) is based on the assumed risk in the comparison group and the **relative effect** of the intervention (and its 95% CI).   **COPD:** Chronic obstructive pulmonary disease; **CI:** Confidence interval; **NR:** Not reported; **RR:** Risk ratio | | | | | | |
| **GRADE Working Group grades of evidence** **High certainty:** We are very confident that the true effect lies close to that of the estimate of the effect **Moderate certainty:** We are moderately confident in the effect estimate: The true effect is likely to be close to the estimate of the effect, but there is a possibility that it is substantially different **Low certainty:** Our confidence in the effect estimate is limited: The true effect may be substantially different from the estimate of the effect **Very low certainty:** We have very little confidence in the effect estimate: The true effect is likely to be substantially different from the estimate of effect | | | | | | |

#### Explanations

a. Studies recruited general smokers (n=20), females only (n=2), smokers with COPD (n=1), and smokers >60 or >65 years (n=2).

b. Intervention: Physician advice involved a 'stop smoking' message delivered verbally. Interventions were defined as minimal if delivered during a single session of <20-minute duration plus up to one follow-up visit with or without a leaflet. Intensive intervention involved a longer initial consultation, use of additional materials other than a leaflet, or more than one follow-up visit. Pharmacotherapy co-intervention provided in some of the studies.

c. Control: Denoted as 'no advice (usual care)' by review authors. According to evidence tables, various control conditions across trials including smoking related written materials, 'control' (not further specified), 'no advice' (not further specified), self-monitoring of smoking, usual care, no intervention, and questionnaires. Additional behavioural and/or pharmacotherapy co-interventions provided to control participants in some studies.

d. Seven studies were cluster RCTs.

e. Review authors did not assess performance/detection bias so we consider this domain unclear for all trials. 100% of evidence with substantive bias issues mostly with selection bias and biochemical validation: 91% at high risk for two domains or at high risk for one domain and unclear risk for two or more domains (including attrition); 9% at high and unclear risk for one domain each or at unclear risk for two or more domains. We downrate this domain by -2.0.

f. Variation in point estimates, but confidence intervals mostly overlap (I2= 40%; p=0.02). We downrate this domain by -0.5.

g. 13% of the evidence is indirect (i.e., specialized medical setting, incentive provided for quitting). Due to reporting, extent of indirectness could not be ascertained for one study contributing 1% of weight to the meta-analysis; the study recruited participants >15 years but proportion of those less than 18 years of age is NR. We do not downrate this domain.

h. Confidence interval encompasses moderate benefit. The optimal information size is met (total of 1470 events) and adequate sample size. We do not downrate this domain.

i. Search was not comprehensive but mix of results across trials. We do not downrate this domain.

## Appendix K Table 47. Physician advice with follow-up versus Minimal intervention/advice with single visit: Smoking cessation in general/mixed population of smokers

| Physician advice with follow-up compared to minimal intervention/advice with single visit in general/mixed population of smokers  **Bibliography:** Stead 2013; Date of last search: January & February 2013 | | | | | | | | | | | |
| --- | --- | --- | --- | --- | --- | --- | --- | --- | --- | --- | --- |
| **Certainty assessment** | | | | | | | **Summary of findings** | | | | |
| **№ of participants (studies) Follow-up** | **Risk of bias** | **Inconsistency** | **Indirectness** | **Imprecision** | **Publication bias** | **Overall certainty of evidence** | **Study event rates (%)** | | **Relative effect (95% CI)** | **Anticipated absolute effects** | |
|  |  |  |  |  |  |  | **With Minimal intervention/advice with single visit** | **With Physician advice with follow-up** |  | **Risk with Minimal intervention/advice with single visit** | **Risk difference with Physician advice with follow-up** |
| **Abstinence/cessation (follow up: 6+ months)** ^a,b,c^  Outcome assessment: point prevalence 40%, continuous/sustained abstinence 60% studies  Biochemical validation 60% studies | | | | | | | | | | | |
| 1254 (5 RCTs) | very serious ^d^ | not serious ^e^ | not serious ^f^ | serious ^g^ | none ^h^ | ⨁◯◯◯  VERY LOW | 47/521 (9.0%) | 84/733 (11.5%) | **RR 1.52** (1.08 to 2.14) | 90 per 1,000 | **47 more per 1,000** (from 7 more to 103 more) |

**CI:** Confidence interval; **RR:** Risk ratio

#### Explanations

a. Four studies recruited general smokers and one recruited general smokers motivated to quit.

b. Intervention: Unclear how many follow-up visits are provided to the intervention arm. Pharmacotherapy co-intervention provided in one study.

c. Control: Pharmacotherapy co-intervention provided in one study.

d. Review authors did not assess performance/detection bias so we consider this domain unclear for all trials. 90% of evidence with substantive bias issues: 29% at unclear risk for two domains (selection and performance/detection bias) and 61% at high risk for one domain (selection bias or biochemical validation) and at unclear risk for two or more domains. We downrate this domain by -2.0.

e. Little variation in point estimates, and the confidence intervals overlap (I2= 0%; p=0.79). We do not downrate this domain.

f. No indirectness. We do not downrate this domain.

g. Confidence interval encompasses one range of effect (small but important benefit to moderate benefit). The optimal information size not met (total of 131 events) and inadequate sample size (<2000 participants). We downrate this domain by -1.0.

h. Search was not comprehensive but trials report negative findings. We do not downrate this domain.

| Summary of findings: | | | | | | |
| --- | --- | --- | --- | --- | --- | --- |
| **Physician advice with follow-up compared to minimal intervention/advice with single visit in general/mixed population of smokers** | | | | | | |
| **Patient or population**: General/mixed population of smokers  **Setting**: No restriction  **Intervention**: Physician advice with follow-up  **Comparison**: Minimal intervention/advice with single visit | | | | | | |
| Outcomes | **Anticipated absolute effects^*^** (95% CI) | | Relative effect (95% CI) | № of participants  (studies) | Certainty of the evidence (GRADE) | Comments |
|  | **Risk with Minimal intervention/advice with single visit** | **Risk with Physician advice with follow-up** |  |  |  |  |
| Abstinence/cessation ^a,b,c^  Outcome assessment: point prevalence 40%, continuous/sustained abstinence 60% studies  Biochemical validation 60% studies  Follow up: 6+ months | 90 per 1,000 | **137 per 1,000** (97 to 193) | **RR 1.52** (1.08 to 2.14) | 1254 (5 RCTs) | ⨁◯◯◯  VERY LOW ^d,e,f,g,h^ | Fixed effects analysis  Date of last search: January/February 2013  AMSTAR-2: Critically low  Not GRADEd by review authors |
| ***The risk in the intervention group** (and its 95% confidence interval) is based on the assumed risk in the comparison group and the **relative effect** of the intervention (and its 95% CI).   **CI:** Confidence interval; **RR:** Risk ratio | | | | | | |
| **GRADE Working Group grades of evidence** **High certainty:** We are very confident that the true effect lies close to that of the estimate of the effect **Moderate certainty:** We are moderately confident in the effect estimate: The true effect is likely to be close to the estimate of the effect, but there is a possibility that it is substantially different **Low certainty:** Our confidence in the effect estimate is limited: The true effect may be substantially different from the estimate of the effect **Very low certainty:** We have very little confidence in the effect estimate: The true effect is likely to be substantially different from the estimate of effect | | | | | | |

#### Explanations

a. Four studies recruited general smokers and one recruited general smokers motivated to quit.

b. Intervention: Unclear how many follow-up visits are provided to the intervention arm. Pharmacotherapy co-intervention provided in one study.

c. Control: Pharmacotherapy co-intervention provided in one study.

d. Review authors did not assess performance/detection bias so we consider this domain unclear for all trials. 90% of evidence with substantive bias issues: 29% at unclear risk for two domains (selection and performance/detection bias) and 61% at high risk for one domain (selection bias or biochemical validation) and at unclear risk for two or more domains. We downrate this domain by -2.0.

e. Little variation in point estimates, and the confidence intervals overlap (I2= 0%; p=0.79). We do not downrate this domain.

f. No indirectness. We do not downrate this domain.

g. Confidence interval encompasses one range of effect (small but important benefit to moderate benefit). The optimal information size not met (total of 131 events) and inadequate sample size (<2000 participants). We downrate this domain by -1.0.

h. Search was not comprehensive but trials report negative findings. We do not downrate this domain.

## Appendix K Table 48. Intensive advice versus Minimal advice: Smoking cessation in general/mixed population of smokers

| Intensive advice compared to minimal advice in general/mixed population of smokers  **Bibliography:** Stead 2013; Date of last search: January & February 2013 | | | | | | | | | | | |
| --- | --- | --- | --- | --- | --- | --- | --- | --- | --- | --- | --- |
| **Certainty assessment** | | | | | | | **Summary of findings** | | | | |
| **№ of participants (studies) Follow-up** | **Risk of bias** | **Inconsistency** | **Indirectness** | **Imprecision** | **Publication bias** | **Overall certainty of evidence** | **Study event rates (%)** | | **Relative effect (95% CI)** | **Anticipated absolute effects** | |
|  |  |  |  |  |  |  | **With Minimal advice** | **With Intensive advice** |  | **Risk with Minimal advice** | **Risk difference with Intensive advice** |
| **Abstinence/cessation (follow up 6+ months)** ^a,b,c^  Outcome assessment: point prevalence 47%, continuous/sustained abstinence 53% studies  Biochemical validation 60% studies | | | | | | | | | | | |
| 9775 (15 RCTs) ^d^ | very serious ^e^ | not serious ^f^ | serious ^g^ | not serious ^h^ | none ^i^ | ⨁◯◯◯  VERY LOW | 305/4036 (7.6%) | 620/5739 (10.8%) | **RR 1.37** (1.20 to 1.56) | 76 per 1,000 | **28 more per 1,000** (from 15 more to 42 more) |

**CI:** Confidence interval; **CVD:** Cardiovascular disease; **RR:** Risk ratio

#### Explanations

a. Studies recruited unselected/general smokers (n=10) or high-risk populations (e.g., smoking related diseases, diabetes, asbestos-exposed, CVD) (n=5).

b. Intervention: Intensive interventions involved a longer initial consultation, use of additional materials other than a leaflet, or more than one follow-up visit. Pharmacotherapy co-intervention provided in one study.

c. Control: Interventions were defined as minimal if delivered during a single session of <20-minute duration plus up to one follow-up visit with or without a leaflet. Pharmacotherapy co-intervention provided in one study.

d. One study was a cluster RCT.

e. Review authors did not assess performance/detection bias so we consider this domain unclear for all trials. 98% of evidence with substantive bias issues: 40% at unclear risk for two or more domains (selection, attrition, performance/detection), 58% at high risk for two domains (selection bias, biochemical validation) or at high risk for one of the aforementioned domains and unclear risk for two or more domains. We downrate this domain by -2.0.

f. Variation in point estimates, but confidence intervals mostly overlap (I2=32%; p=0.12). We downrate by -0.5.

g. 52% of the evidence is indirect (workplace, schools, medical specialist settings). We downrate this domain by -1.0.

h. Confidence interval encompasses small but important benefit. The optimal information size is met (total of 925 events) and adequate sample size. We do not downrate this domain.

i. Search was not comprehensive but mix of results across trials. We do not downrate this domain.

| Summary of findings: | | | | | | |
| --- | --- | --- | --- | --- | --- | --- |
| **Intensive advice compared to Minimal advice in general/mixed population of smokers** | | | | | | |
| **Patient or population**: General/mixed population of smokers  **Setting**: No restriction  **Intervention**: Intensive advice  **Comparison**: Minimal advice | | | | | | |
| Outcomes | **Anticipated absolute effects^*^** (95% CI) | | Relative effect (95% CI) | № of participants  (studies) | Certainty of the evidence (GRADE) | Comments |
|  | **Risk with Minimal advice** | **Risk with Intensive advice** |  |  |  |  |
| Abstinence/cessation ^a,b,c^  Outcome assessment: point prevalence 47%, continuous/sustained abstinence 53% studies  Biochemical validation 60% studies  Follow up 6+ months | 76 per 1,000 | **104 per 1,000** (91 to 118) | **RR 1.37** (1.20 to 1.56) | 9775 (15 RCTs) ^d^ | ⨁◯◯◯  VERY LOW ^e,f,g,h,i^ | Fixed effects analysis  Date of last search: January/February 2013  AMSTAR-2: Critically low  Not GRADEd by review authors |
| ***The risk in the intervention group** (and its 95% confidence interval) is based on the assumed risk in the comparison group and the **relative effect** of the intervention (and its 95% CI).   **CI:** Confidence interval; **CVD:** Cardiovascular disease; **RR:** Risk ratio | | | | | | |
| **GRADE Working Group grades of evidence** **High certainty:** We are very confident that the true effect lies close to that of the estimate of the effect **Moderate certainty:** We are moderately confident in the effect estimate: The true effect is likely to be close to the estimate of the effect, but there is a possibility that it is substantially different **Low certainty:** Our confidence in the effect estimate is limited: The true effect may be substantially different from the estimate of the effect **Very low certainty:** We have very little confidence in the effect estimate: The true effect is likely to be substantially different from the estimate of effect | | | | | | |

#### Explanations

a. Studies recruited unselected/general smokers (n=10) or high-risk populations (e.g., smoking related diseases, diabetes, asbestos-exposed, CVD) (n=5).

b. Intervention: Intensive interventions involved a longer initial consultation, use of additional materials other than a leaflet, or more than one follow-up visit. Pharmacotherapy co-intervention provided in one study.

c. Control: Interventions were defined as minimal if delivered during a single session of <20-minute duration plus up to one follow-up visit with or without a leaflet. Pharmacotherapy co-intervention provided in one study.

d. One study was a cluster RCT.

e. Review authors did not assess performance/detection bias so we consider this domain unclear for all trials. 98% of evidence with substantive bias issues: 40% at unclear risk for two or more domains (selection, attrition, performance/detection), 58% at high risk for two domains (selection bias, biochemical validation) or at high risk for one of the aforementioned domains and unclear risk for two or more domains. We downrate this domain by -2.0.

f. Variation in point estimates, but confidence intervals mostly overlap (I2= 32%; p=0.12). We downrate by -0.5.

g. 52% of the evidence is indirect (workplace, schools, medical specialist settings). We downrate this domain by -1.0.

h. Confidence interval encompasses small but important benefit. The optimal information size is met (total of 925 events) and adequate sample size. We do not downrate this domain.

i. Search was not comprehensive but mix of results across trials. We do not downrate this domain.

# Stead 2016 {1356}

## Appendix K Table 49. Combined pharmacotherapy and behavioural interventions versus Usual care or minimal intervention: Smoking cessation in the general/mixed population

| Combined pharmacotherapy and behavioural interventions compared to usual care or minimal intervention in general/mixed population of smokers  **Bibliography:** Stead, 2016; Date of last search: July 2015 | | | | | | | | | | | |
| --- | --- | --- | --- | --- | --- | --- | --- | --- | --- | --- | --- |
| **Certainty assessment** | | | | | | | **Summary of findings** | | | | |
| **№ of participants (studies) Follow-up** | **Risk of bias** | **Inconsistency** | **Indirectness** | **Imprecision** | **Publication bias** | **Overall certainty of evidence** | **Study event rates (%)** | | **Relative effect (95% CI)** | **Anticipated absolute effects** | |
|  |  |  |  |  |  |  | **With Usual care or minimal intervention** | **With Combined pharmaco-therapy and behavioural interventions** |  | **Risk with Usual care or minimal intervention** | **Risk difference with Combined pharmaco-therapy and behavioural interventions** |
| **Abstinence/cessation (follow up: 6+ months)** ^a,b,c^  Outcome measurement: point prevalence 60%, continuous/sustained abstinence 28%, prolonged abstinence 8%, unclear/NR 4% studies  Biochemical validation: 65% studies | | | | | | | | | | | |
| 19488 (52 RCTs) | very serious ^d^ | not serious ^e^ | not serious ^f^ | not serious ^g^ | none ^h^ | ⨁⨁◯◯  LOW | 808/9418 (8.6%) | 1529/10070 (15.2%) | **RR 1.83** (1.68 to 1.98) | 86 per 1,000 | **71 more per 1,000** (from 58 more to 84 more) |

**CI:** Confidence interval; **COPD:** Chronic obstructive pulmonary disease; **CVD:** Cardiovascular disease; **HIV:** Human immunodeficiency virus; **NR:** Not reported; **RR:** Risk ratio

#### Explanations

a. Trials took place in various settings consisting of a mix of populations: 38% of studies in smokers motivated to quit (inclusive of co-morbidities such as mental health, inpatients, COPD, substance use, and CVD), 17% in unselected (general) smokers, small frequencies of co-morbidities (mental health, diabetes, CVD, cancer, HIV, substance use, and surgical or hospital patients), women-only studies, and one study in smokers of low socioeconomic status.

b. The typical intervention involved multiple contacts with a specialist cessation counsellor, with most using pharmacotherapy and receiving multiple contacts, but with variation across studies. Additional behavioural with or without pharmacological co-intervention provided in a few trials.

c. Usual care or minimal intervention was typically brief advice and self-help materials. Additional behavioural and/or pharmacological co-intervention provided in some trials. Unclear whether co-intervention provided in some trials.

d. 84% of evidence with substantial issues of bias: high risk for lack of biochemical validation, unclear risk for performance bias, and unclear risks for randomization sequence and/or concealment; 46% of the evidence at very serious risk of bias. We downrate by -1.5 for risk of bias but also account for issues of indirectness in the rating of this domain.

e. Point estimates are mostly consistent, and outlier estimates contribute little weight in the meta-analysis. Confidence intervals overlap somewhat. I2=36%, and the low p=0.006 value could be reflective of an overpowered analysis. We downrate this domain by -0.5.

f. 29.9% of the evidence, is indirect due to setting (inpatient, medical specialist, workplace) or irrelevant intervention (nortriptyline). We downrate this domain by -0.5 but reflect this in the risk of bias domain.

g. Confidence interval encompasses moderate benefit. The optimal information size is met and there is a large sample size (>2000 participants). We did not downrate this domain.

h. Search not completely comprehensive, and a mix of findings across studies. According to the review authors, the funnel plot was asymmetric (with smaller studies showing larger effects); however once removing these studies, it did not change the pooled estimate. We do not downrate this domain.

| Summary of findings: | | | | | | |
| --- | --- | --- | --- | --- | --- | --- |
| **Combined pharmacotherapy and behavioural interventions compared to usual care or minimal intervention in general/mixed population of smokers** | | | | | | |
| **Patient or population**: General/mixed population of smokers  **Setting**: No restriction  **Intervention**: Combined pharmacotherapy and behavioural interventions  **Comparison**: Usual care or minimal intervention | | | | | | |
| Outcomes | **Anticipated absolute effects^*^** (95% CI) | | Relative effect (95% CI) | № of participants  (studies) | Certainty of the evidence (GRADE) | Comments |
|  | **Risk with Usual care or minimal intervention** | **Risk with Combined pharmacotherapy and behavioural interventions** |  |  |  |  |
| Abstinence/cessation ^a,b,c^  Outcome measurement: point prevalence 60%, continuous/sustained abstinence 28%, prolonged abstinence 8%, unclear/NR 4% studies  Biochemical validation: 65% studies  Follow up: 6+ months | 86 per 1,000 | **157 per 1,000** (144 to 170) | **RR 1.83** (1.68 to 1.98) | 19488 (52 RCTs) | ⨁⨁◯◯  LOW ^d,e,f,g,h^ | Fixed effects analysis.  Date of last search: July 2015.  AMSTAR 2: Low  Authors rate as high certainty. |
| ***The risk in the intervention group** (and its 95% confidence interval) is based on the assumed risk in the comparison group and the **relative effect** of the intervention (and its 95% CI).   **CI:** Confidence interval; **COPD:** Chronic obstructive pulmonary disease; **CVD:** Cardiovascular disease; **HIV:** Human immunodeficiency virus; **NR:** Not reported; **RR:** Risk ratio | | | | | | |
| **GRADE Working Group grades of evidence** **High certainty:** We are very confident that the true effect lies close to that of the estimate of the effect **Moderate certainty:** We are moderately confident in the effect estimate: The true effect is likely to be close to the estimate of the effect, but there is a possibility that it is substantially different **Low certainty:** Our confidence in the effect estimate is limited: The true effect may be substantially different from the estimate of the effect **Very low certainty:** We have very little confidence in the effect estimate: The true effect is likely to be substantially different from the estimate of effect | | | | | | |

#### Explanations

a. Trials took place in various settings consisting of a mix of populations: 38% of studies in smokers motivated to quit (inclusive of co-morbidities such as mental health, inpatients, COPD, substance use, and CVD), 17% in unselected (general) smokers, small frequencies of co-morbidities (mental health, diabetes, CVD, cancer, HIV, substance use, and surgical or hospital patients), women-only studies, and one study in smokers of low socioeconomic status.

b. The typical intervention involved multiple contacts with a specialist cessation counsellor, with most using pharmacotherapy and receiving multiple contacts, but with variation across studies. Additional behavioural with or without pharmacological co-intervention provided in a few trials.

c. Usual care or minimal intervention was typically brief advice and self-help materials. Additional behavioural and/or pharmacological co-intervention provided in some trials. Unclear whether co-intervention provided in some trials.

d. 84% of evidence with substantial issues of bias: high risk for lack of biochemical validation, unclear risk for performance bias, and unclear risks for randomization sequence and/or concealment; 46% of the evidence at very serious risk of bias. We downrate by -1.5 for risk of bias but also account for issues of indirectness in the rating of this domain.

e. Point estimates are mostly consistent, and outlier estimates contribute little weight in the meta-analysis. Confidence intervals overlap somewhat. I2=36%, and the low p=0.006 value could be reflective of an overpowered analysis. We downrate this domain by -0.5.

f. 29.9% of the evidence, is indirect due to setting (inpatient, medical specialist, workplace) or irrelevant intervention (nortriptyline). We downrate this domain by -0.5 but reflect this in the risk of bias domain.

g. Confidence interval encompasses moderate benefit. The optimal information size is met and there is a large sample size (>2000 participants). We did not downrate this domain.

h. Search not completely comprehensive, and a mix of findings across studies. According to the review authors, the funnel plot was asymmetric (with smaller studies showing larger effects); however once removing these studies, it did not change the pooled estimate. We do not downrate this domain.

## Appendix K Table 50. Combined pharmacotherapy and behavioural interventions versus Usual care or no intervention: Smoking cessation in general/mixed population of smokers

| Combined pharmacotherapy and behavioural interventions compared to usual care or no intervention in general/mixed population of smokers  **Bibliography:** Stead, 2016; Date of last search: July 2015 | | | | | | | | | | | |
| --- | --- | --- | --- | --- | --- | --- | --- | --- | --- | --- | --- |
| **Certainty assessment** | | | | | | | **Summary of findings** | | | | |
| **№ of participants (studies) Follow-up** | **Risk of bias** | **Inconsistency** | **Indirectness** | **Imprecision** | **Publication bias** | **Overall certainty of evidence** | **Study event rates (%)** | | **Relative effect (95% CI)** | **Anticipated absolute effects** | |
|  |  |  |  |  |  |  | **With Usual care or no intervention** | **With Combined pharmaco-therapy and behavioural interventions** |  | **Risk with Usual care or no intervention** | **Risk difference with Combined pharmaco-therapy and behavioural interventions** |
| **Abstinence/Cessation (follow up: 12 months)** ^a,b,c^  Outcome measurement: point prevalence 100% studies  Biochemical validation: 100% studies | | | | | | | | | | | |
| 5887 (1 RCT) | not serious ^d^ | not serious ^e^ | serious ^f^ | not serious ^g^ | none ^h^ | ⨁⨁⨁◯  MODERATE | 177/1964 (9.0%) | 1373/3923 (35.0%) | **RR 3.88** (3.35 to 4.50) | 90 per 1,000 | **260 more per 1,000** (from 212 more to 315 more) |

**CI:** Confidence interval; **COPD:** Chronic obstructive pulmonary disease; **RR:** Risk ratio

#### Explanations

a. Healthy smokers with mild airway obstruction were recruited. Providers: specialist counsellors.

b. Combined pharmacotherapy and behavioural interventions: Consisted of 12 group sessions over 10 weeks, with advice from physician on risk for COPD, 2 mg of nicotine gum for 6 months. Intervention participants were also randomized to bronchodilator or placebo. No co-interventions.

c. Usual care or no intervention: No co-interventions provided.

d. Unclear risk for performance bias. We downrate by -0.5.

e. One study included in analysis. We do not downrate this domain

f. We reflect authors’ indication that the intervention tested in this study may not be applicable to a real-world treatment setting.

g. Confidence interval encompasses large benefit. The optimal information size is met and there is a large sample size (>2000 participants). We did not downrate this domain.

h. Search was not comprehensive, but no publication bias concerns overall with the evidence based for combined behavioural and pharmacotherapy (refer to related analysis). We do not downrate this domain.

| Summary of findings: | | | | | | |
| --- | --- | --- | --- | --- | --- | --- |
| **Combined pharmacotherapy and behavioural interventions compared to usual care or no intervention in general/mixed population of smokers** | | | | | | |
| **Patient or population**: General/mixed population of smokers  **Setting**: Not restricted  **Intervention**: Combined pharmacotherapy and behavioural interventions  **Comparison**: Usual care or no intervention | | | | | | |
| Outcomes | **Anticipated absolute effects^*^** (95% CI) | | Relative effect (95% CI) | № of participants  (studies) | Certainty of the evidence (GRADE) | Comments |
|  | **Risk with Usual care or no intervention** | **Risk with Combined pharmacotherapy and behavioural interventions** |  |  |  |  |
| Abstinence/cessation ^a,b,c^  Outcome measurement: point prevalence 100% studies  Biochemical validation: 100% studies  Follow up: 12 months | 90 per 1,000 | **350 per 1,000** (302 to 406) | **RR 3.88** (3.35 to 4.50) | 5887 (1 RCT) | ⨁⨁⨁◯  MODERATE ^d,e,f,g,h^ | Date of last search: July 2015.  AMSTAR 2: Low  Authors rate as moderate certainty. ^i^ |
| ***The risk in the intervention group** (and its 95% confidence interval) is based on the assumed risk in the comparison group and the **relative effect** of the intervention (and its 95% CI).   **CI:** Confidence interval; **COPD:** Chronic obstructive pulmonary disease; **RR:** Risk ratio | | | | | | |
| **GRADE Working Group grades of evidence** **High certainty:** We are very confident that the true effect lies close to that of the estimate of the effect **Moderate certainty:** We are moderately confident in the effect estimate: The true effect is likely to be close to the estimate of the effect, but there is a possibility that it is substantially different **Low certainty:** Our confidence in the effect estimate is limited: The true effect may be substantially different from the estimate of the effect **Very low certainty:** We have very little confidence in the effect estimate: The true effect is likely to be substantially different from the estimate of effect | | | | | | |

#### Explanations

a. Healthy smokers with mild airway obstruction were recruited. Providers: specialist counsellors.

b. Combined pharmacotherapy and behavioural interventions: Consisted of 12 group sessions over 10 weeks, with advice from physician on risk for COPD, 2 mg of nicotine gum for 6 months. Intervention participants were also randomized to bronchodilator or placebo. No co-interventions.

c. Usual care or no intervention: No co-interventions provided.

d. Unclear risk for performance bias. We downrate by -0.5.

e. One study included in analysis. We do not downrate this domain

f. We reflect authors’ indication that the intervention tested in this study may not be applicable to a real-world treatment setting.

g. Confidence interval encompasses large benefit. The optimal information size is met and there is a large sample size (>2000 participants). We did not downrate this domain.

h. Search was not comprehensive, but no publication bias concerns overall with the evidence based for combined behavioural and pharmacotherapy (refer to related analysis). We do not downrate this domain.

i. Authors downrate due to indirectness as this study had evaluated an intensive intervention that may not be generalizable to a real-world treatment programme.

# Stead 2017 {538}

## Appendix K Table 51. Group therapy vs No intervention: Smoking cessation in general/mixed population of smokers

| Group behaviour therapy compared to no intervention in general/mixed population of smokers  **Bibliography:** Stead 2017; Date of last search May 2016 | | | | | | | | | | | |
| --- | --- | --- | --- | --- | --- | --- | --- | --- | --- | --- | --- |
| **Certainty assessment** | | | | | | | **Summary of findings** | | | | |
| **№ of participants (studies) Follow-up** | **Risk of bias** | **Inconsistency** | **Indirectness** | **Imprecision** | **Publication bias** | **Overall certainty of evidence** | **Study event rates (%)** | | **Relative effect (95% CI)** | **Anticipated absolute effects** | |
|  |  |  |  |  |  |  | **With no intervention** | **With Group behaviour therapy** |  | **Risk with no intervention** | **Risk difference with Group behaviour therapy** |
| **Abstinence/Cessation (follow up: 6+ months)** ^a,b,c,d^  Outcome measurement: Unclear/NR 67%, point prevalence 22%, continuous/sustained abstinence 11% studies  Biochemical validation: 11% | | | | | | | | | | | |
| 1098 (9 RCTs) | very serious ^e^ | serious ^f^ | not serious ^g^ | serious ^h^ | none ^i^ | ⨁◯◯◯  VERY LOW | 32/475 (6.7%) | 119/623 (19.1%) | RR 2.60 (1.80 to 3.76) | 67 per 1,000 | **108 more per 1,000** (from 54 more to 186 more) |

**CI:** Confidence interval; **NR:** Not reported; **RR:** Risk ratio

#### Explanations

a. Majority (78%) of studies in smokers not otherwise specified and deemed general/mixed. Two remaining studies in substance use population and mental illness, respectively.

b. As per eligibility, group therapy was delivered over at least two sessions. No intervention or minimal contact control.

c. Co-interventions (Intervention arm): Pharmacotherapy and other co-interventions provided in some studies. Other co-interventions provided in some studies. Unclear whether co-interventions provided in most studies. 4.7% received specialized behavioural counselling.

d. Co-interventions (Control arm): Pharmacotherapy and other co-interventions provided in some studies. Behavioural and other co-interventions provided in some studies. Unclear whether co-interventions provided in most studies.

e. Majority of evidence with substantial bias issues: 95% high risk for detection bias, performance bias not assessed but nature of intervention lends to high risk, and studies at unclear-to-high risk for selection bias. We downrate by -2.0.

f. Variation in point estimates and confidence intervals do not all overlap. I2=55%, p=0.02. We downrate by -1.0.

g. An evaluation of the study-level information provided in the review signalled no issues of indirectness to the question of interest. We do not downrate this domain.

h. Confidence interval encompasses one range of effect (moderate benefit to large benefit). The optimal information is not met (total of 151 events) and inadequate sample size (<2000 participants). We downrate this domain by -1.0.

i. Although authors state that they cannot exclude the possibility of publication bias and acknowledge the possibility of other published or unpublished studies, there is a mix of results among studies and has not been detected. We do not downrate this domain.

| Summary of findings: | | | | | | |
| --- | --- | --- | --- | --- | --- | --- |
| **Group behaviour therapy compared to no intervention in general/mixed population of smokers** | | | | | | |
| **Patient or population**: general/mixed population of smokers  **Setting**: Not restricted  **Intervention**: Group behaviour therapy  **Comparison**: no intervention | | | | | | |
| Outcomes | **Anticipated absolute effects^*^** (95% CI) | | Relative effect (95% CI) | № of participants  (studies) | Certainty of the evidence (GRADE) | Comments |
|  | **Risk with no intervention** | **Risk with Group behaviour therapy** |  |  |  |  |
| Abstinence/Cessation ^a,b,c,d^  Outcome measurement: Unclear/NR 67%, point prevalence 22%, continuous/sustained abstinence 11% studies  Biochemical validation: 11%  Follow up: 6+ months | 67 per 1,000 | **175 per 1,000** (121 to 253) | RR 2.60 (1.80 to 3.76) | 1098 (9 RCTs) | ⨁◯◯◯  VERY LOW ^e,f,g,h,i^ | Fixed effects meta-analysis.  Date of last search: May 2016.  AMSTAR-2: Critically low.  Authors rate as low certainty. ^j^ |
| ***The risk in the intervention group** (and its 95% confidence interval) is based on the assumed risk in the comparison group and the **relative effect** of the intervention (and its 95% CI).   **CI:** Confidence interval; **NR:** Not reported; **RR:** Risk ratio | | | | | | |
| **GRADE Working Group grades of evidence** **High certainty:** We are very confident that the true effect lies close to that of the estimate of the effect **Moderate certainty:** We are moderately confident in the effect estimate: The true effect is likely to be close to the estimate of the effect, but there is a possibility that it is substantially different **Low certainty:** Our confidence in the effect estimate is limited: The true effect may be substantially different from the estimate of the effect **Very low certainty:** We have very little confidence in the effect estimate: The true effect is likely to be substantially different from the estimate of effect | | | | | | |

#### Explanations

a. Majority (78%) of studies in smokers not otherwise specified and deemed general/mixed. Two remaining studies in substance use population and mental illness, respectively.

b. As per eligibility, group therapy was delivered over at least two sessions. No intervention or minimal contact control.

c. Co-interventions (Intervention arm): Pharmacotherapy and other co-interventions provided in some studies. Other co-interventions provided in some studies. Unclear whether co-interventions provided in most studies. 4.7% received specialized behavioural counselling.

d. Co-interventions (Control arm): Pharmacotherapy and other co-interventions provided in some studies. Behavioural and other co-interventions provided in some studies. Unclear whether co-interventions provided in most studies.

e. Majority of evidence with substantial bias issues: 95% high risk for detection bias, performance bias not assessed but nature of intervention lends to high risk, and studies at unclear-to-high risk for selection bias. We downrate by -2.0.

f. Variation in point estimates and confidence intervals do not all overlap. I2=55%, p=0.02. We downrate by -1.0.

g. An evaluation of the study-level information provided in the review signalled no issues of indirectness to the question of interest. We do not downrate this domain.

h. Confidence interval encompasses one range of effect (moderate benefit to large benefit). The optimal information is not met (total of 151 events) and inadequate sample size (<2000 participants). We downrate this domain by -1.0.

i. Although authors state that they cannot exclude the possibility of publication bias and acknowledge the possibility of other published or unpublished studies, there is a mix of results among studies and has not been detected. We do not downrate this domain.

j. Review authors downgraded twice. Most studies at unclear risk of bias for allocation and concealment. Lack of biochemical validation in most studies.

# Taylor 2017 {411}

## Appendix K Table 52. Interactive and tailored internet interventions versus Non-active controls: Smoking cessation in general/mixed population of smokers

| Interactive and tailored internet interventions compared to non-active controls in general/mixed population of smokers  **Bibliography:** Taylor 2017; Date of last search: August 2016 | | | | | | | | | | | |
| --- | --- | --- | --- | --- | --- | --- | --- | --- | --- | --- | --- |
| **Certainty assessment** | | | | | | | **Summary of findings** | | | | |
| **№ of participants (studies) Follow-up** | **Risk of bias** | **Inconsistency** | **Indirectness** | **Imprecision** | **Publication bias** | **Overall certainty of evidence** | **Study event rates (%)** | | **Relative effect (95% CI)** | **Anticipated absolute effects** | |
|  |  |  |  |  |  |  | **With non-active controls** | **With Interactive and tailored internet interventions** |  | **Risk with non-active controls** | **Risk difference with Interactive and tailored internet interventions** |
| **Abstinence/Cessation (follow up: 6-12 months)** ^a,b,c,d^  Outcome measurement: point prevalence 37.5%, continuous/sustained abstinence 50%, prolonged abstinence 12.5% studies  Biochemical validation: 25% studies | | | | | | | | | | | |
| 6786 (8 RCTs) ^e^ | very serious ^f^ | serious ^g^ | serious ^h^ | not serious ^i^ | none ^j^ | ⨁◯◯◯  VERY LOW | 356/2766 (12.9%) | 516/4020 (12.8%) | RR 1.15 (1.01 to 1.30) | 129 per 1,000 | **19 more per 1,000** (from 1 more to 39 more) |

**CI:** Confidence interval; **COPD:** Chronic obstructive pulmonary disease; **RR:** Risk ratio

#### Explanations

a. Two studies each recruited inpatients, general smokers, and general smokers motivated to quit. The remaining two studies recruited childhood cancer survivors and male smokers with COPD.

b. Interactive interventions 'involved a two-way flow of information between the internet and the participant'. Tailored refers to 'programmes that were adapted to a participant's characteristics'.

c. Intervention arm: One study provided NRT as an adjunct intervention. Another study offered free nicotine patches and bupropion to all participants and their partners who wanted to quit. No co-interventions provided in remaining studies.

d. Control arm: Across studies, 'non-active controls' consisted of printed self-help guides or usual care. One study offered free nicotine patches and bupropion to all participants, including controls, and their partners who wanted to quit. No co-interventions provided to control arm in remaining studies.

e. One study described as 'quasi-randomized' by review authors.

f. 97% of evidence is at high risk for at least one domain (selection, attrition, biochemical validation). Performance/detection bias was not assessed by review authors. Due to the nature of the intervention and control, blinding may not have been achievable. Given the uncertainty, a range of risk may be representative. We downrate this domain by -1.5 to -2.0 but also account for additional issues of indirectness in this domain.

g. Some variation in effect estimates and confidence intervals of some studies do not overlap with others (I2=58%, p=0.02). We downrate this domain by -1.0.

h. 68% of the evidence is indirect (inpatient or medical specialist setting, country of conduct); we downrate this domain by -1.5. Due to reporting, extent of indirectness could not be determined for one study which recruited smokers and recent quitters without specifying the proportion of each.

i. Confidence interval encompasses one range of effect (little to no difference to small but important benefit). The optimal information size is met (total of 872 events) and adequate sample size. We do not downrate this domain.

j. Search strategy is comprehensive and mix of findings across studies. We do not downrate this domain.

| Summary of findings: | | | | | | |
| --- | --- | --- | --- | --- | --- | --- |
| **Interactive and tailored internet interventions compared to non-active controls in general/mixed population of smokers** | | | | | | |
| **Patient or population**: General/mixed population of smokers  **Setting**: No restriction  **Intervention**: Interactive and tailored internet interventions  **Comparison**: Non-active controls | | | | | | |
| Outcomes | **Anticipated absolute effects^*^** (95% CI) | | Relative effect (95% CI) | № of participants  (studies) | Certainty of the evidence (GRADE) | Comments |
|  | **Risk with non-active controls** | **Risk with Interactive and tailored internet interventions** |  |  |  |  |
| Abstinence/Cessation ^a,b,c,d^  Outcome measurement: point prevalence 37.5%, continuous/sustained abstinence 50%, prolonged abstinence 12.5% studies.  Biochemical validation: 25% studies  Follow up: 6-12 months | 129 per 1,000 | **148 per 1,000** (130 to 167) | RR 1.15 (1.01 to 1.30) | 6786 (8 RCTs) ^e^ | ⨁◯◯◯  VERY LOW ^f,g,h,i,j^ | Fixed effects analysis.  AMSTAR-2: Critically low.  Date of last search: August 2016.  Review authors rated certainty as low. ^k^ |
| ***The risk in the intervention group** (and its 95% confidence interval) is based on the assumed risk in the comparison group and the **relative effect** of the intervention (and its 95% CI).   **CI:** Confidence interval; **COPD:** Chronic obstructive pulmonary disease; **RR:** Risk ratio | | | | | | |
| **GRADE Working Group grades of evidence** **High certainty:** We are very confident that the true effect lies close to that of the estimate of the effect **Moderate certainty:** We are moderately confident in the effect estimate: The true effect is likely to be close to the estimate of the effect, but there is a possibility that it is substantially different **Low certainty:** Our confidence in the effect estimate is limited: The true effect may be substantially different from the estimate of the effect **Very low certainty:** We have very little confidence in the effect estimate: The true effect is likely to be substantially different from the estimate of effect | | | | | | |

#### Explanations

a. Two studies each recruited inpatients, general smokers, and general smokers motivated to quit. The remaining two studies recruited childhood cancer survivors and male smokers with COPD.

b. Interactive interventions 'involved a two-way flow of information between the internet and the participant'. Tailored refers to 'programmes that were adapted to a participant's characteristics'.

c. Intervention arm: One study provided NRT as an adjunct intervention. Another study offered free nicotine patches and bupropion to all participants and their partners who wanted to quit. No co-interventions provided in remaining studies.

d. Control arm: Across studies, 'non-active controls' consisted of printed self-help guides or usual care. One study offered free nicotine patches and bupropion to all participants, including controls, and their partners who wanted to quit. No co-interventions provided to control arm in remaining studies.

e. One study described as 'quasi-randomized' by review authors.

f. 97% of evidence is at high risk for at least one domain (selection, attrition, biochemical validation). Performance/detection bias was not assessed by review authors. Due to the nature of the intervention and control, blinding may not have been achievable. Given the uncertainty, a range of risk may be representative. We downrate this domain by -1.5 to -2.0 but also account for additional issues of indirectness in this domain.

g. Some variation in effect estimates and confidence intervals of some studies do not overlap with others (I2=58%, p=0.02). We downrate this domain by -1.0.

h. 68% of the evidence is indirect (inpatient or medical specialist setting, country of conduct); we downrate this domain by -1.5. Due to reporting, extent of indirectness could not be determined for one study which recruited smokers and recent quitters without specifying the proportion of each.

i. Confidence interval encompasses one range of effect (little to no difference to small but important benefit). The optimal information size is met (total of 872 events) and adequate sample size. We do not downrate this domain.

j. Search strategy is comprehensive and mix of findings across studies. We do not downrate this domain.

k. Review authors rated certainty as low (downrated one level each for risk of bias and inconsistency).

## Appendix K Table 53. Internet plus behavioural support versus Non-internet-based non-active controls: Smoking cessation in general/mixed population of smokers

| Internet plus behavioural support compared to non-internet-based non-active controls in general/mixed population of smokers  **Bibliography:** Taylor 2017; Date of last search: August 2016 | | | | | | | | | | | |
| --- | --- | --- | --- | --- | --- | --- | --- | --- | --- | --- | --- |
| **Certainty assessment** | | | | | | | **Summary of findings** | | | | |
| **№ of participants (studies) Follow-up** | **Risk of bias** | **Inconsistency** | **Indirectness** | **Imprecision** | **Publication bias** | **Overall certainty of evidence** | **Study event rates (%)** | | **Relative effect (95% CI)** | **Anticipated absolute effects** | |
|  |  |  |  |  |  |  | **With non-internet-based non-active controls** | **With Internet plus behavioural support** |  | **Risk with non-internet-based non-active controls** | **Risk difference with Internet plus behavioural support** |
| **Abstinence/Cessation (follow up: 6-12 months)** ^a,b,c^  Outcome measurement: point prevalence 20%, continuous/sustained abstinence 20%, prolonged abstinence 60% studies  Biochemical validation: 40% studies | | | | | | | | | | | |
| 2334 (5 RCTs) | serious to very serious ^d^ | serious ^e^ | not serious ^f^ | not serious ^g^ | none ^h^ | ⨁◯◯◯  VERY LOW | 75/966 (7.8%) | 164/1368 (12.0%) | RR 1.69 (1.30 to 2.18) | 78 per 1,000 | **54 more per 1,000** (from 23 more to 92 more) |

**CI:** Confidence interval; **RR:** Risk ratio

#### Explanations

a. All studies recruited general smokers. In one trial, participants were motivated to quit.

b. Intervention arm: Internet intervention plus behavioural support provided by nurses, peer coaches, or tobacco treatment specialists. In two trials, all participants were using or offered pharmacotherapy.

c. Control arm: Across studies, ‘non-active control’ consisted of usual care, printed self-help guides, and standard smoking cessation advice. In two trials, all participants including controls were using or offered pharmacotherapy.

d. 84% of the evidence is at high risk for one domain (selection bias or biochemical validation). Performance/detection bias was not assessed by review authors; due to the nature of the intervention and control, blinding may not have been achievable. Given the uncertainty, a range of risk may be representative. We downrate this domain by -1.5 to -2.0.

e. Effect estimates vary and confidence intervals of some studies do not overlap with others (I2=60%, p=0.04). We downrate this domain by -1.0.

f. Due to reporting, extent of indirectness could not be determined for one study which recruited smokers and recent quitters without specifying the proportion of each. No sources of indirectness in remaining studies. We do not downrate this domain.

g. Confidence interval encompasses two ranges of effect (small but important benefit to large benefit). The optimal information size not met (total of 239 events), but adequate sample size. We downrate this domain by -0.5.

h. Search strategy is comprehensive and mix of findings across studies. We do not downrate this domain.

| Summary of findings: | | | | | | |
| --- | --- | --- | --- | --- | --- | --- |
| **Internet plus behavioural support compared to non-internet-based non-active controls in general/mixed population of smokers** | | | | | | |
| **Patient or population**: General/mixed population of smokers  **Setting**: No restriction  **Intervention**: Internet plus behavioural support  **Comparison**: Non-internet based non-active controls | | | | | | |
| Outcomes | **Anticipated absolute effects^*^** (95% CI) | | Relative effect (95% CI) | № of participants  (studies) | Certainty of the evidence (GRADE) | Comments |
|  | **Risk with non-internet-based non-active controls** | **Risk with Internet plus behavioural support** |  |  |  |  |
| Abstinence/Cessation ^a,b,c^  Outcome measurement: point prevalence 20%, continuous/sustained abstinence 20%, prolonged abstinence 60%.  Biochemical validation: 40%  Follow up: 6-12 months | 78 per 1,000 | **131 per 1,000** (101 to 169) | RR 1.69 (1.30 to 2.18) | 2334 (5 RCTs) | unable to assess ^d,e,f,g,h^ | Fixed effects analysis.  AMSTAR-2: Critically low.  Date of last search: August 2016.  Review authors rated certainty as moderate. ^i^ |
| ***The risk in the intervention group** (and its 95% confidence interval) is based on the assumed risk in the comparison group and the **relative effect** of the intervention (and its 95% CI).   **CI:** Confidence interval; **RR:** Risk ratio | | | | | | |
| **GRADE Working Group grades of evidence** **High certainty:** We are very confident that the true effect lies close to that of the estimate of the effect **Moderate certainty:** We are moderately confident in the effect estimate: The true effect is likely to be close to the estimate of the effect, but there is a possibility that it is substantially different **Low certainty:** Our confidence in the effect estimate is limited: The true effect may be substantially different from the estimate of the effect **Very low certainty:** We have very little confidence in the effect estimate: The true effect is likely to be substantially different from the estimate of effect | | | | | | |

#### Explanations

a. All studies recruited general smokers. In one trial, participants were motivated to quit.

b. Intervention arm: Internet intervention plus behavioural support provided by nurses, peer coaches, or tobacco treatment specialists. In two trials, all participants were using or offered pharmacotherapy.

c. Control arm: Across studies, ‘non-active control’ consisted of usual care, printed self-help guides, and standard smoking cessation advice. In two trials, all participants including controls were using or offered pharmacotherapy.

d. 84% of the evidence is at high risk for one domain (selection bias or biochemical validation). Performance/detection bias was not assessed by review authors; due to the nature of the intervention and control, blinding may not have been achievable. Given the uncertainty, a range of risk may be representative. We downrate this domain by -1.5 to -2.0.

e. Effect estimates vary and confidence intervals of some studies do not overlap with others (I2=60%, p=0.04). We downrate this domain by -1.0.

f. Due to reporting, extent of indirectness could not be determined for one study which recruited smokers and recent quitters without specifying the proportion of each. No sources of indirectness in remaining studies. We do not downrate this domain.

g. Confidence interval encompasses two ranges of effect (small but important benefit to large benefit). The optimal information size not met (total of 239 events), but adequate sample size. We downrate this domain by -0.5.

h. Search strategy is comprehensive and mix of findings across studies. We do not downrate this domain.

i. Review authors rate certainty as moderate (downrated one level for inconsistency).

# Tsoi 2013 {1698}

## Appendix K Table 54. Bupropion vs Placebo: Smoking cessation, reduction, change in mental state, and adverse events in smokers with schizophrenia or schizoaffective disorder

| Bupropion compared to placebo in smokers with schizophrenia or schizoaffective disorder  **Bibliography:** Tsoi 2013; Date of last search: October/November 2012 | | | | | | | | | | | |
| --- | --- | --- | --- | --- | --- | --- | --- | --- | --- | --- | --- |
| **Certainty assessment** | | | | | | | **Summary of findings** | | | | |
| **№ of participants (studies) Follow-up** | **Risk of bias** | **Inconsistency** | **Indirectness** | **Imprecision** | **Publication bias** | **Overall certainty of evidence** | **Study event rates (%)** | | **Relative effect (95% CI)** | **Anticipated absolute effects** | |
|  |  |  |  |  |  |  | **With placebo** | **With Bupropion** |  | **Risk with placebo** | **Risk difference with Bupropion** |
| **Abstinence/cessation (follow up: 6 months)** ^a,b,c^  Trials had a primary aim of smoking cessation  Outcome measurement: point prevalence 40%, continuous/sustained abstinence 60% of studies  Biochemical validation: 100% studies | | | | | | | | | | | |
| 214 (5 RCTs) | serious ^d^ | not serious ^e^ | not serious ^f^ | very serious ^g^ | none ^h^ | ⨁◯◯◯  VERY LOW | 4/108 (3.7%) | 14/106 (13.2%) | RR 2.78 (1.02 to 7.58) | 37 per 1,000 | **66 more per 1,000** (from 1 more to 244 more) |
| **Reduction in number of cigarettes per day from baseline (follow up: 6 months)** ^a,i^  Trials had a primary aim of smoking cessation  Outcome measurement: unclear/NR  Biochemical validation: unclear/NR ^j^ | | | | | | | | | | | |
| 104 (2 RCTs) ^k^ | serious ^l^ | not serious ^m^ | not serious ^f^ | serious ^n^ | none ^h^ | ⨁⨁◯◯  LOW | 54 | 50 | - | The mean change in number of cigarettes per day from baseline ranged from **-2.9 to -6.0** | MD **0.4 higher** (5.72 lower to 6.53 higher) |
| **Expired CO level (follow up: 6 months)** ^a,i,o^  Trials had a primary aim of smoking cessation  Outcome measurement: unclear/NR | | | | | | | | | | | |
| 123 (3 RCTs) ^k^ | serious ^p^ | very serious ^q^ | not serious ^f^ | serious ^r^ | none ^s^ | ⨁◯◯◯  VERY LOW | 63 | 60 | - | The mean expired CO level ranged from **20-27** ppm | MD **5.55 ppm lower** (17.89 lower to 6.78 higher) |
| **Change in mental state: Positive symptoms (e.g., hallucinations, delusions) (follow up: End of treatment)** ^a,t,u^  Trials had a primary aim of smoking cessation  Outcome measurement: Effects on mental state measured by PANSS and BDI in one trial and by PANSS, SANS, HAM-D and HAM-A in the other. | | | | | | | | | | | |
| 85 (2 RCTs) | serious ^v^ | not serious ^w^ | not serious ^f^ | serious ^x^ | none ^h^ | ⨁⨁◯◯  LOW | 44 | 41 | - | - | SMD **0.24 SD lower** (0.66 lower to 0.19 higher) |
| **Change in mental state: Negative symptoms (e.g. anhedonia, avolition) (follow up: End of treatment)** ^a,u,y^  Trials had a primary aim of smoking cessation  Outcome measurement: Effects on mental state measured using PANSS, SANS, HAM-D, and HAM-A or STAI in two trials. The third trial used PANSS and BDI. | | | | | | | | | | | |
| 136 (3 RCTs) | serious ^z^ | not serious ^aa^ | not serious ^f^ | very serious ^ab^ | none ^h^ | ⨁◯◯◯  VERY LOW | 70 | 66 | - | - | SMD **0.12 SD lower** (0.46 lower to 0.22 higher) |
| **Change in mental state: Depressive symptoms (follow up: End of treatment)** ^a,u,y^  Trials had a primary aim of smoking cessation  Outcome measurement: Effects on mental state measured using PANSS, SANS, HAM-D, and HAM-A or STAI in two trials. The third trial used PANSS and BDI. | | | | | | | | | | | |
| 136 (3 RCTs) | serious ^z^ | not serious ^ac^ | not serious ^f^ | serious ^ad^ | none ^h^ | ⨁⨁◯◯  LOW | 70 | 66 | - | - | SMD **0.16 SD lower** (0.5 lower to 0.18 higher) |
| **Adverse events (follow up: Unclear/NR)** ^ae,af,ag^  Trials had a primary aim of smoking cessation | | | | | | | | | | | |
| Unclear/NR (7 RCTs) ^ag,ah^ | serious ^ai^ | not serious ^aj^ | not serious ^ak^ | serious to very serious ^al^ | none ^s^ | unable to assess | Review authors report various adverse events collectively.  In one study, a participant who took bupropion had a seizure; authors reported that this was likely unrelated to bupropion treatment. No seizures reported in remaining trials.  One study reported that one participant receiving bupropion (3%) and two receiving placebo (7%) experienced a psychotic breakdown; authors did not consider this related to study treatment. Another study reported recurrence of psychotic symptoms in two participants but results were not reported by study arm. One study reported no serious adverse events.  One trial reported higher rates of dry mouth in the bupropion arm compared to placebo and another study reported that significantly more bupropion participants (also receiving NRT) experienced poor concentration, jitteriness, light-headedness, muscle stiffness and frequent nocturnal awakening. A third study reported that, compared to placebo, the bupropion arm had higher rates of insomnia, dry mouth and sweatiness. A fourth study reported an allergic reaction in one participant receiving bupropion. A fifth study reported no significant differences in major adverse events measured by the Side Effect Checklist (e.g., restlessness, insomnia, dry mouth, sedation); however, five participants on bupropion withdrew from the trial due to side effects, including rash (n=1), restlessness and increased anxiety (n=2), worsening of psychosis (n=1), and the aforementioned seizure. The sixth study reported two participant withdrawals in the bupropion arm (also receiving NRT) due to insomnia and dizziness. | | | | |
| **Change in mental state: Positive symptoms (e.g. hallucinations, delusions), negative symptoms (e.g. anhedonia, avolition), psychiatric symptoms (follow up: End of treatment)** ^am,an^  Trials had a primary aim of smoking reduction  Outcome measurement: Effects on mental state measured by PANSS and BPRS in one trial; by BPRS, PANSS, SAPS SANS, HAM-D, and BDI in one trial; and by PANSS in the remaining trial. | | | | | | | | | | | |
| Unclear/NR (3 RCTs) ^ao,ap^ | very serious ^aq^ | not serious ^ar^ | not serious ^as^ | serious to very serious ^at^ | none ^h^ | ⨁◯◯◯  VERY LOW | One study reported no worsening of positive and negative symptoms in the bupropion arm. A second study reported no significant difference between groups regarding change in positive and negative symptoms. The third study reported no increase in psychiatric symptoms in the bupropion arm. | | | | |
| **Adverse events (follow up: Unclear/NR)** ^am,an,au^  Trials had a primary aim of smoking reduction | | | | | | | | | | | |
| Unclear/NR (3 RCTs) ^au,av^ | serious ^aw^ | not serious ^ax^ | serious ^ay^ | serious to very serious ^az^ | none ^h^ | ⨁◯◯◯  VERY LOW | Two trials reported no adverse events related to bupropion. One trial reported no significant difference between groups in adverse events and no seizures or suicidal behaviour in the bupropion arm. | | | | |

**BDI:** Beck Depression Inventory; **BPRS:** Brief Psychiatric Rating Scale; **CBT:** Cognitive behavioural therapy; **CI:** Confidence interval; **CO:** Carbon monoxide; **CPD:** Cigarettes per day; **HAM-D:** Hamilton Depression Rating Scale; **HAM-A:** Hamilton Anxiety Rating Scale; **HDI:** Human Development Index; **MD:** Mean difference; **NR:** Not reported; **NRT:** Nicotine replacement therapy; **RR:** Risk ratio; **PANSS:** Positive And Negative Syndrome Scale; **SANS:** Scale for the Assessment of Negative Symptoms; **SAPS:** Scale for the Assessment of Positive Symptoms; **SMD:** Standardised mean difference; **STAI:** State and Trait Anxiety Inventory

#### Explanations

a. Participants in all trials were interested in quitting smoking.

b. Bupropion: Most trials examined 300 mg/day for about 10 or 12 weeks; one trial provided 150 mg/day for 12 weeks. Behavioural co-intervention provided in all trials. In two trials, participants also received NRT patch with or without NRT gum as a co-intervention. 72% of participants in this analysis received specialized behavioural counselling (CBT or relapse prevention).

c. Placebo: Behavioural co-intervention provided in all trials. In two trials, participants also received NRT patch with or without NRT gum as a co-intervention. 73% of participants received specialized behavioural counselling (CBT or relapse prevention).

d. 58% of evidence with substantive bias issues: 47% at unclear risk of bias for two or more domains (i.e., selection, performance/detection, or attrition bias) and 11% at high risk for one domain (i.e., attrition). We downrate this domain by -1.0.

e. Some variation in point estimates but confidence intervals overlap (I2=0%, p=0.90). We do not downrate this domain.

f. No indirectness.

g. Confidence interval encompasses three ranges of effect (little to no difference to large benefit). The optimal information size not met (total of 18 events) and inadequate sample size (<2000 participants). We downrate this domain by -2.0.

h. Search strategy not completely comprehensive but all trials report negative findings. We do not downrate this domain.

i. Bupropion and Placebo: Both trials included in the reduction in CPD analysis provided 300 mg/day for about 12 weeks. For expired CO, two trials provided 300 mg/day and one provided 150 mg/day for about 12 weeks. Behavioural co-intervention provided to both arms in all trials. In one of the trials, participants in both arms also received NRT patch and NRT gum as a co-intervention. All participants in this analysis received specialized behavioural counselling (CBT).

j. Reduction in terms of expired CO is also reported by both trials; however, unclear whether reduced levels of CO at follow-up was used to validate reduction in self-reported CPD.

k. Review authors indicate that abstainers as well as continued smokers are likely included in smoking reduction outcome measures.

l. 100% of the evidence with substantive bias issues: 92% of evidence at unclear risk for both performance/detection and attrition bias and all of the evidence at unclear risk of bias attributed to lack of biochemical validation. We downrate this domain by -1.0.

m. Point estimates vary but confidence intervals overlap (I2=0%, p=0.51). We do not downrate this domain.

n. Confidence interval encompasses little to no difference. The optimal information size not met and inadequate sample size (<400 participants for a continuous outcome). We downrate this domain by -1.0.

o. Review authors combine change-from-baseline measurements and final measurements if mean difference was used. Two trials in this analysis used final measurements and one trial used change-from-baseline.

p. 63% of evidence with substantive bias issues: 35% at unclear risk for performance/detection and attrition bias and 28% at high risk for attrition bias. We downrate this domain by -1.0.

q. Point estimates vary and CI from one trial does not overlap with the CIs from the others (I2=83%, p=0.003). We downrate this domain by -2.0.

r. Confidence interval encompasses one range of effect (little to no difference to small benefit). The optimal information size not met and inadequate sample size (<400 participants for a continuous outcome). We downrate this domain by -1.0.

s. Although search strategy not completely comprehensive, mix of results across trials. We do not downrate this domain.

t. Bupropion and Placebo: Both trials provided 300 mg/day of bupropion for about 10 or 12 weeks. Behavioural co-intervention provided to both arms in both trials. All participants in this analysis received specialized behavioural counselling (CBT or relapse prevention).

u. Final measurement data reported in studies used by review authors to estimate change in symptoms.

v. 100% of the evidence is at unclear risk of bias for two or more domains, namely, selection, performance/detection, and attrition bias. We downrate this domain by -1.0.

w. Little variation in point estimates and confidence intervals overlap (I2=0%, p=0.51). We do not downrate this domain.

x. Confidence interval encompasses two ranges of effect (little to no difference to moderate benefit). The optimal information size not met and inadequate sample size (<400 participants for a continuous outcome). We downrate this domain by -1.5.

y. Bupropion and Placebo: All trials examined 300 mg/day of bupropion for about 10 or 12 weeks. Behavioural co-intervention provided to both arms in all trials. In one trial, participants in both arms also received NRT patch and NRT gum as a co-intervention. All participants in this analysis received specialized behavioural counselling (CBT or relapse prevention).

z. 62% of evidence at unclear risk of bias for two or more domains, namely, selection, performance/detection, and attrition bias. We downrate this domain by -1.0.

aa. Little variation in point estimates and confidence intervals overlap (I2=0%, p=0.88). We do not downrate this domain.

ab. Confidence interval encompasses both harm (small but important) and benefit (moderate). The optimal information size not met and inadequate sample size (<400 participants for a continuous outcome). We downrate this domain by -2.0.

ac. Little variation in point estimates and confidence intervals overlap (I2=0%, p=0.80). We do not downrate this domain.

ad. Confidence interval encompasses two ranges of effect (little to no difference to moderate benefit). The optimal information size not met and inadequate sample size (<400 participants for a continuous outcome). We downrate this domain by -1.5.

ae. Participants in six trials were interested in quitting. Remaining trial recruited participants interested in reducing smoking consumption.

af. Bupropion and Placebo: Most trials provided 300 mg/day for about 4, 10, or 12 weeks; one trial provided 150 mg/day for 12 weeks. Behavioural co-intervention provided to all participants in all but one trial. In two trials, participants in both arms also received NRT patch with or without NRT gum. In four of the trials, the behavioural co-intervention was specialized behavioural counselling (i.e., CBT or relapse prevention).

ag. Timing of adverse event ascertainment unclear/NR. Five trials followed participants for at least 6 months for smoking outcomes while two trials did not follow participants beyond end of treatment.

ah. Number of participants included in this trial not explicitly reported by review authors. Approximately 340 total participants across trials (170 per arm).

ai. Six trials with serious limitations; four trials at unclear risk for two or three domains among selection, performance/detection and attrition, one trial at high risk for attrition bias, and one trial with very serious limitations (high risk for selection bias and unclear for performance/detection and attrition). We downrate this domain by -1.0.

aj. Effect estimates not reported in the review; comparison of direction and magnitude of results not possible. Narrative data suggestive of some inconsistency across trials for some adverse events, but may not be appreciable. We downrate this domain by -0.5.

ak. One trial was conducted in a high HDI country and recruited participants from a psychiatric inpatient unit. The trial included a total of 80 participants but unclear if all were included in this analysis. No indirectness in remaining studies. We do not downrate this domain.

al. Unable to assess confidence intervals. Unclear sample size analyzed but approximately 340 total participants and the optimal information size cannot be met. We cannot rate this domain.

am. Participants in two trials were interested in quitting or reducing smoking. Participants’ interest in quitting/reducing not reported in remaining trial.

an. Bupropion and Placebo: Studies provided 300 mg/day of bupropion for about 22 days to 14 weeks, across trials; dose and duration of intervention for one study unclear. One trial provided both arms with a specialized behavioural counselling co-intervention (CBT). In a second study, both arms received non-contingent reinforcement. No co-interventions provided in remaining trial.

ao. Number of participants included in this analysis is not clearly reported in the review. Two of the three trials included a combined 71 participants. The remaining study was a four-arm trial which included a total of 57 participants; the number of participants from the two arms included in this analysis is unclear.

ap. One study is a cross-over trial.

aq. Two trials at high risk for one domain (i.e., selection or selective reporting bias) and at unclear risk for two or more other domains. Remaining trial at unclear risk for three domains (i.e., selection, performance/detection, and attrition bias). We downrate this domain by -2.0.

ar. Effect estimates not reported in the review; comparison of direction and magnitude of results not possible. Overall, results consistently show no difference between groups or no worsening in symptoms. We do not downrate this domain.

as. One four-arm trial provided non-contingent reinforcement as a co-intervention. Number of participants from this trial that contribute to this analysis is unclear. No indirectness in remaining studies. We downrate this domain by -0.5.

at. Unable to assess confidence intervals. Unclear sample size analyzed but 2/3 trials included a combined 71 participants and the remaining study included a total of 57 participants. The optimal information size cannot be met. We cannot rate this domain.

au. Timepoint of follow-up for this outcome not clearly reported but likely occurred at end of treatment. Treatment duration ranged from 22 days to 14 weeks across trials.

av. Number of participants included in this analysis is not clearly reported in the review. Two of the three trials included a combined 93 participants. The remaining study was a four-arm trial which included a total of 57 participants; the number of participants from the two arms included in this analysis is unclear.

aw. Two trials at unclear risk for three domains (i.e., selection, performance/detection, and attrition bias). Remaining trial at high risk for selection bias and unclear risk for performance/detection and attrition bias. We downrate this domain by -1.0.

ax. Overall, results show no events or no difference between groups. We do not downrate this domain.

ay. One four-arm trial provided non-contingent reinforcement as co-intervention. A second trial was conducted in a country with a high HDI rating and recruited from a psychiatric in-patient unit. Number of participants from each of these trials contributing to the analysis is unclear. No indirectness in remaining study. We downrate this domain by -1.5.

az. Unable to assess confidence intervals. Unclear sample size analyzed but approximately 2/3 trials included a combined 93 participants and the remaining study included a total of 57 participants. The optimal information size cannot be met. We cannot rate this domain.

| Summary of findings: | | | | | | |
| --- | --- | --- | --- | --- | --- | --- |
| **Bupropion compared to placebo in smokers with schizophrenia or schizoaffective disorder** | | | | | | |
| **Patient or population**: Smokers with schizophrenia or schizoaffective disorder  **Setting**: No restriction  **Intervention**: Bupropion  **Comparison**: Placebo | | | | | | |
| Outcomes | **Anticipated absolute effects^*^** (95% CI) | | Relative effect (95% CI) | № of participants  (studies) | Certainty of the evidence (GRADE) | Comments |
|  | **Risk with placebo** | **Risk with Bupropion** |  |  |  |  |
| Abstinence/cessation ^a,b,c^  Trials had a primary aim of smoking cessation.  Outcome measurement: point prevalence 40%, continuous/sustained abstinence 60% of studies  Biochemical validation: 100% studies  Follow up: 6 months | 37 per 1,000 | **103 per 1,000** (38 to 281) | RR 2.78 (1.02 to 7.58) | 214 (5 RCTs) | ⨁◯◯◯  VERY LOW ^d,e,f,g,h^ | NNT: 15 (4 to 1350).  One trial reported that 3 additional participants were abstinent at 2-year follow-up; of the 4 total participants that quit, 3 received bupropion during the trial or during the follow-up period. One other study reported that of the 5 bupropion participants that were abstinent at 6 months, 2 relapsed at 12 month follow-up. Including this 12-month follow-up data would reduce the effect estimate and the CI would cross the null.  Date of last search: October/November 2012.  AMSTAR-2: Moderate.  Not GRADEd by review authors. |
| Reduction in number of cigarettes per day from baseline ^a,i^  Trials had a primary aim of smoking cessation.  Outcome measurement: unclear/NR  Biochemical validation: unclear/NR ^j^  Follow up: 6 months | The mean reduction in number of cigarettes per day from baseline ranged from **-2.9 to -6.0** | MD **0.4 higher** (5.72 lower to 6.53 higher) | - | 104 (2 RCTs) ^k^ | ⨁⨁◯◯  LOW ^f,h,l,m,n^ | Date of last search: October/November 2012.  AMSTAR-2: Moderate.  Not GRADEd by review authors. |
| Expired CO level ^a,i,o^  Trials had a primary aim of smoking cessation.  Outcome measurement: unclear/NR  Follow up: 6 months | The mean expired CO level ranged from **20-27** ppm | MD **5.55 ppm lower** (17.89 lower to 6.78 higher) | - | 123 (3 RCTs) ^k^ | ⨁◯◯◯  VERY LOW ^f,p,q,r,s^ | Mean expired CO level at 6-month follow up (n=3) is 18.8 ppm and 22.7 ppm in bupropion and placebo groups, respectively. Review authors report that heterogeneity largely attributed to one trial reporting higher average CO levels in bupropion arm (Mean (SD): 26 ppm (16)) compared to placebo (20 ppm (12)).  Date of last search: October/November 2012.  AMSTAR-2: Moderate.  Not GRADEd by review authors. |
| Change in mental state: Positive symptoms (e.g., hallucinations, delusions) ^a,t,u^  Trials had a primary aim of smoking cessation.  Outcome measurement: Effects on mental state measured by PANSS and BDI in one trial and by PANSS, SANS, HAM-D and HAM-A in the other.  Follow up: End of treatment | - | SMD **0.24 SD lower** (0.66 lower to 0.19 higher) | - | 85 (2 RCTs) | ⨁⨁◯◯  LOW ^f,h,v,w,x^ | Three additional trials provide corroborating evidence of no difference between groups in symptoms but data incompletely reported. One trial not included in the meta-analysis reported greater stability of psychotic symptoms in the bupropion arm during quit attempt. Three additional studies reported no effect of smoking abstinence on positive symptoms.  Date of last search: October/November 2012.  AMSTAR-2: Moderate.  Not GRADEd by review authors. |
| Change in mental state: Negative symptoms (e.g. anhedonia, avolition) ^a,u,y^  Trials had a primary aim of smoking cessation.  Outcome measurement: Effects on mental state measured using PANSS, SANS, HAM-D, and HAM-A or STAI in two trials. The third trial used PANSS and BDI.  Follow up: End of treatment | - | SMD **0.12 SD lower** (0.46 lower to 0.22 higher) | - | 136 (3 RCTs) | ⨁◯◯◯  VERY LOW ^f,h,z,aa,ab^ | Three additional trials provide corroborating evidence of no difference between groups in symptoms but data incompletely reported. One trial not included in the meta-analysis reported improvement in negative symptoms in the bupropion arm during quit attempt. Three additional studies reported no effect of smoking abstinence on negative symptoms.  Date of last search: October/November 2012.  AMSTAR-2: Moderate.  Not GRADEd by review authors. |
| Change in mental state: Depressive symptoms ^a,u,y^  Trials had a primary aim of smoking cessation.  Outcome measurement: Effects on mental state measured using PANSS, SANS, HAM-D, and HAM-A or STAI in two trials. The third trial used PANSS and BDI.  Follow up: End of treatment | - | SMD **0.16 SD lower** (0.5 lower to 0.18 higher) | - | 136 (3 RCTs) | ⨁⨁◯◯  LOW ^f,h,z,ac,ad^ | Three additional trials provide corroborating evidence of no difference between groups in symptoms but data incompletely reported. One trial not included in the meta-analysis reported greater stability of depressive symptoms in the bupropion arm during quit attempt. Three additional studies reported no effect of smoking abstinence on depressive symptoms.  Date of last search: October/November 2012.  AMSTAR-2: Moderate.  Not GRADEd by review authors. |
| Adverse events ^ae,af,ag^  Trials had a primary aim of smoking cessation.  Follow up: Unclear/NR | Review authors report various adverse events collectively.  In one study, a participant who took bupropion had a seizure; authors reported that this was likely unrelated to bupropion treatment. No seizures reported in remaining trials.  One study reported that one participant receiving bupropion (3%) and two receiving placebo (7%) experienced a psychotic breakdown; authors did not consider this related to study treatment. Another study reported recurrence of psychotic symptoms in two participants but results were not reported by study arm. One study reported no serious adverse events.  One trial reported higher rates of dry mouth in the bupropion arm compared to placebo and another study reported that significantly more bupropion participants (also receiving NRT) experienced poor concentration, jitteriness, light-headedness, muscle stiffness and frequent nocturnal awakening. A third study reported that, compared to placebo, the bupropion arm had higher rates of insomnia, dry mouth and sweatiness. A fourth study reported an allergic reaction in one participant receiving bupropion. A fifth study reported no significant differences in major adverse events measured by the Side Effect Checklist (e.g., restlessness, insomnia, dry mouth, sedation); however, five participants on bupropion withdrew from the trial due to side effects, including rash (n=1), restlessness and increased anxiety (n=2), worsening of psychosis (n=1), and the aforementioned seizure. The sixth study reported two participant withdrawals in the bupropion arm (also receiving NRT) due to insomnia and dizziness. | |  | Unclear/NR  (7 RCTs) ^ag,ah^ | unable to assess ^s,ai,aj,ak,al^ | Date of last search: October/November 2012.  AMSTAR-2: Moderate.  Not GRADEd by review authors. |
| Change in mental state: Positive symptoms (e.g. hallucinations, delusions), negative symptoms (e.g. anhedonia, avolition), psychiatric symptoms ^am,an^  Trials had a primary aim of smoking reduction.  Outcome measurement: Effects on mental state measured by PANSS and BPRS in one trial; by BPRS, PANSS, SAPS SANS, HAM-D, and BDI in one trial; and by PANSS in the remaining trial.  Follow up: End of treatment | One study reported no worsening of positive and negative symptoms in the bupropion arm. A second study reported no significant difference between groups regarding change in positive and negative symptoms. The third study reported no increase in psychiatric symptoms in the bupropion arm. | |  | Unclear/NR  (3 RCTs) ^ao,ap^ | ⨁◯◯◯  VERY LOW ^h,aq,ar,as,at^ | Date of last search: October/November 2012.  AMSTAR-2: Moderate.  Not GRADEd by review authors. |
| Adverse events ^am,an,au^  Trials had a primary aim of smoking reduction.  Follow up: Unclear/NR | Two trials reported no adverse events related to bupropion. One trial reported no significant difference between groups in adverse events and no seizures or suicidal behaviour in the bupropion arm. | |  | Unclear/NR  (3 RCTs) ^au,av^ | ⨁◯◯◯  VERY LOW ^h,aw,ax,ay,az^ | Date of last search: October/November 2012.  AMSTAR-2: Moderate.  Not GRADEd by review authors. |
| ***The risk in the intervention group** (and its 95% confidence interval) is based on the assumed risk in the comparison group and the **relative effect** of the intervention (and its 95% CI).   **BDI:** Beck Depression Inventory; **BPRS:** Brief Psychiatric Rating Scale; **CBT:** Cognitive behavioural therapy; **CI:** Confidence interval; **CO:** Carbon monoxide; **CPD:** Cigarettes per day; **HAM-D:** Hamilton Depression Rating Scale; **HAM-A:** Hamilton Anxiety Rating Scale; **HDI:** Human Development Index; **MD:** Mean difference; **NNT:** Number needed to treat; **NR:** Not reported; **NRT:** Nicotine replacement therapy; **RR:** Risk ratio; **PANSS:** Positive And Negative Syndrome Scale; **SANS:** Scale for the Assessment of Negative Symptoms; **SAPS:** Scale for the Assessment of Positive Symptoms; **SMD:** Standardised mean difference; **STAI:** State and Trait Anxiety Inventory | | | | | | |
| **GRADE Working Group grades of evidence** **High certainty:** We are very confident that the true effect lies close to that of the estimate of the effect **Moderate certainty:** We are moderately confident in the effect estimate: The true effect is likely to be close to the estimate of the effect, but there is a possibility that it is substantially different **Low certainty:** Our confidence in the effect estimate is limited: The true effect may be substantially different from the estimate of the effect **Very low certainty:** We have very little confidence in the effect estimate: The true effect is likely to be substantially different from the estimate of effect | | | | | | |

#### Explanations

a. Participants in all trials were interested in quitting smoking.

b. Bupropion: Most trials examined 300 mg/day for about 10 or 12 weeks; one trial provided 150 mg/day for 12 weeks. Behavioural co-intervention provided in all trials. In two trials, participants also received NRT patch with or without NRT gum as a co-intervention. 72% of participants in this analysis received specialized behavioural counselling (CBT or relapse prevention).

c. Placebo: Behavioural co-intervention provided in all trials. In two trials, participants also received NRT patch with or without NRT gum as a co-intervention. 73% of participants received specialized behavioural counselling (CBT or relapse prevention).

d. 58% of evidence with substantive bias issues: 47% at unclear risk of bias for two or more domains (i.e., selection, performance/detection, or attrition bias) and 11% at high risk for one domain (i.e., attrition). We downrate this domain by -1.0.

e. Some variation in point estimates but confidence intervals overlap (I2=0%, p=0.90). We do not downrate this domain.

f. No indirectness.

g. Confidence interval encompasses three ranges of effect (little to no difference to large benefit). The optimal information size not met (total of 18 events) and inadequate sample size (<2000 participants). We downrate this domain by -2.0.

h. Search strategy not completely comprehensive but all trials report negative findings. We do not downrate this domain.

i. Bupropion and Placebo: Both trials included in the reduction in CPD analysis provided 300 mg/day for about 12 weeks. For expired CO, two trials provided 300 mg/day and one provided 150 mg/day for about 12 weeks. Behavioural co-intervention provided to both arms in all trials. In one of the trials, participants in both arms also received NRT patch and NRT gum as a co-intervention. All participants in this analysis received specialized behavioural counselling (CBT).

j. Reduction in terms of expired CO is also reported by both trials; however, unclear whether reduced levels of CO at follow-up was used to validate reduction in self-reported CPD.

k. Review authors indicate that abstainers as well as continued smokers are likely included in smoking reduction outcome measures.

l. 100% of the evidence with substantive bias issues: 92% of evidence at unclear risk for both performance/detection and attrition bias and all of the evidence at unclear risk of bias attributed to lack of biochemical validation. We downrate this domain by -1.0.

m. Point estimates vary but confidence intervals overlap (I2=0%, p=0.51). We do not downrate this domain.

n. Confidence interval encompasses little to no difference. The optimal information size not met and inadequate sample size (<400 participants for a continuous outcome). We downrate this domain by -1.0.

o. Review authors combine change-from-baseline measurements and final measurements if mean difference was used. Two trials in this analysis used final measurements and one trial used change-from-baseline.

p. 63% of evidence with substantive bias issues: 35% at unclear risk for performance/detection and attrition bias and 28% at high risk for attrition bias. We downrate this domain by -1.0.

q. Point estimates vary and CI from one trial does not overlap with the CIs from the others (I2=83%, p=0.003). We downrate this domain by -2.0.

r. Confidence interval encompasses one range of effect (little to no effect to small benefit). The optimal information size not met and inadequate sample size (<400 participants for a continuous outcome). We downrate this domain by -1.0.

s. Although search strategy not completely comprehensive, mix of results across trials. We do not downrate this domain.

t. Bupropion and Placebo: Both trials provided 300 mg/day of bupropion for about 10 or 12 weeks. Behavioural co-intervention provided to both arms in both trials. All participants in this analysis received specialized behavioural counselling (CBT or relapse prevention).

u. Final measurement data reported in studies used by review authors to estimate change in symptoms.

v. 100% of the evidence is at unclear risk of bias for two or more domains, namely, selection, performance/detection, and attrition bias. We downrate this domain by -1.0.

w. Little variation in point estimates and confidence intervals overlap (I2=0%, p=0.51). We do not downrate this domain.

x. Confidence interval encompasses two ranges of effect (little to no difference to moderate benefit). The optimal information size not met and inadequate sample size (<400 participants for a continuous outcome). We downrate this domain by -1.5.

y. Bupropion and Placebo: All trials examined 300 mg/day of bupropion for about 10 or 12 weeks. Behavioural co-intervention provided to both arms in all trials. In one trial, participants in both arms also received NRT patch and NRT gum as a co-intervention. All participants in this analysis received specialized behavioural counselling (CBT or relapse prevention).

z. 62% of evidence at unclear risk of bias for two or more domains, namely, selection, performance/detection, and attrition bias. We downrate this domain by -1.0.

aa. Little variation in point estimates and confidence intervals overlap (I2=0%, p=0.88). We do not downrate this domain.

ab. Confidence interval encompasses both harm (small but important) and benefit (moderate). The optimal information size not met and inadequate sample size (<400 participants for a continuous outcome). We downrate this domain by -2.0.

ac. Little variation in point estimates and confidence intervals overlap (I2=0%, p=0.80). We do not downrate this domain.

ad. Confidence interval encompasses two ranges of effect (little to no difference to moderate benefit). The optimal information size not met and inadequate sample size (<400 participants for a continuous outcome). We downrate this domain by -1.5.

ae. Participants in six trials were interested in quitting. Remaining trial recruited participants interested in reducing smoking consumption.

af. Bupropion and Placebo: Most trials provided 300 mg/day for about 4, 10, or 12 weeks; one trial provided 150 mg/day for 12 weeks. Behavioural co-intervention provided to all participants in all but one trial. In two trials, participants in both arms also received NRT patch with or without NRT gum. In four of the trials, the behavioural co-intervention was specialized behavioural counselling (i.e., CBT or relapse prevention).

ag. Timing of adverse event ascertainment unclear/NR. Five trials followed participants for at least 6 months for smoking outcomes while two trials did not follow participants beyond end of treatment.

ah. Number of participants included in this trial not explicitly reported by review authors. Approximately 340 total participants across trials (170 per arm).

ai. Six trials with serious limitations; four trials at unclear risk for two or three domains among selection, performance/detection and attrition, one trial at high risk for attrition bias, and one trial with very serious limitations (high risk for selection bias and unclear for performance/detection and attrition). We downrate this domain by -1.0.

aj. Effect estimates not reported in the review; comparison of direction and magnitude of results not possible. Narrative data suggestive of some inconsistency across trials for some adverse events, but may not be appreciable. We downrate this domain by -0.5.

ak. One trial was conducted in a high HDI country and recruited participants from a psychiatric inpatient unit. The trial included a total of 80 participants but unclear if all were included in this analysis. No indirectness in remaining studies. We do not downrate this domain.

al. Unable to assess confidence intervals. Unclear sample size analyzed but approximately 340 total participants and the optimal information size cannot be met. We cannot rate this domain.

am. Participants in two trials were interested in quitting or reducing smoking. Participants’ interest in quitting/reducing not reported in remaining trial.

an. Bupropion and Placebo: Studies provided 300 mg/day of bupropion for about 22 days to 14 weeks, across trials; dose and duration of intervention for one study unclear. One trial provided both arms with a specialized behavioural counselling co-intervention (CBT). In a second study, both arms received non-contingent reinforcement. No co-interventions provided in remaining trial.

ao. Number of participants included in this analysis is not clearly reported in the review. Two of the three trials included a combined 71 participants. The remaining study was a four-arm trial which included a total of 57 participants; the number of participants from the two arms included in this analysis is unclear.

ap. One study is a cross-over trial.

aq. Two trials at high risk for one domain (i.e., selection or selective reporting bias) and at unclear risk for two or more other domains. Remaining trial at unclear risk for three domains (i.e., selection, performance/detection, and attrition bias). We downrate this domain by -2.0.

ar. Effect estimates not reported in the review; comparison of direction and magnitude of results not possible. Overall, results consistently show no difference between groups or no worsening in symptoms. We do not downrate this domain.

as. One four-arm trial provided non-contingent reinforcement as a co-intervention. Number of participants from this trial that contribute to this analysis is unclear. No indirectness in remaining studies. We downrate this domain by -0.5.

at. Unable to assess confidence intervals. Unclear sample size analyzed but 2/3 trials included a combined 71 participants and the remaining study included a total of 57 participants. The optimal information size cannot be met. We cannot rate this domain.

au. Timepoint of follow-up for this outcome not clearly reported but likely occurred at end of treatment. Treatment duration ranged from 22 days to 14 weeks across trials.

av. Number of participants included in this analysis is not clearly reported in the review. Two of the three trials included a combined 93 participants. The remaining study was a four-arm trial which included a total of 57 participants; the number of participants from the two arms included in this analysis is unclear.

aw. Two trials at unclear risk for three domains (i.e., selection, performance/detection, and attrition bias). Remaining trial at high risk for selection bias and unclear risk for performance/detection and attrition bias. We downrate this domain by -1.0.

ax. Overall, results show no events or no difference between groups. We do not downrate this domain.

ay. One four-arm trial provided non-contingent reinforcement as co-intervention. A second trial was conducted in a country with a high HDI rating and recruited from a psychiatric in-patient unit. Number of participants from each of these trials contributing to the analysis is unclear. No indirectness in remaining study. We downrate this domain by -1.5.

az. Unable to assess confidence intervals. Unclear sample size analyzed but approximately 2/3 trials included a combined 93 participants and the remaining study included a total of 57 participants. The optimal information size cannot be met. We cannot rate this domain.

## Appendix K Table 55. Varenicline vs Placebo: Smoking cessation, reduction, change in mental state, and adverse events in smokers with schizophrenia or schizoaffective disorder

| Varenicline compared to placebo in smokers with schizophrenia or schizoaffective disorder  **Bibliography:** Tsoi 2013; Date of last search: October/November 2012 | | | | | | | | | | | |
| --- | --- | --- | --- | --- | --- | --- | --- | --- | --- | --- | --- |
| **Certainty assessment** | | | | | | | **Summary of findings** | | | | |
| **№ of participants (studies) Follow-up** | **Risk of bias** | **Inconsistency** | **Indirectness** | **Imprecision** | **Publication bias** | **Overall certainty of evidence** | **Study event rates (%)** | | **Relative effect (95% CI)** | **Anticipated absolute effects** | |
|  |  |  |  |  |  |  | **With placebo** | **With Varenicline** |  | **Risk with placebo** | **Risk difference with Varenicline** |
| **Abstinence/Cessation (follow up: 6 months)** ^a,b^  Trial had a primary aim of smoking cessation  Outcome measurement: point prevalence abstinence 100% of studies  Biochemical validation: 100% studies | | | | | | | | | | | |
| 128 (1 RCT) | serious ^c^ | not serious ^d^ | not serious ^e^ | very serious ^f^ | none ^g^ | ⨁◯◯◯  VERY LOW | 1/43 (2.3%) | 10/85 (11.8%) | RR 5.06 (0.67 to 38.24) | 23 per 1,000 | **94 more per 1,000** (from 8 fewer to 866 more) |
| **Reduction in number of cigarettes/day from baseline (follow up: 6 months)** ^a,b^  Trial had a primary aim of smoking cessation  Outcome measurement: unclear/NR  Biochemical validation: unclear/NR | | | | | | | | | | | |
| Unclear/NR (1 RCT) ^h^ | serious ^i^ | not serious ^d^ | not serious ^e^ | serious to very serious ^j^ | none ^g^ | unable to assess | Among continued smokers, there was no statistically significant difference between groups in reduction of cigarettes per day from baseline. | | | | |
| **Change in mental state: Positive (e.g. hallucinations, delusions), negative (e.g. anhedonia, avolition), and depressive symptoms (follow up: End of treatment)** ^k,l^  Trials had a primary aim of smoking cessation  Outcome measurement: Effects on mental state measured using PANSS, SAS, Columbia Suicide Severity Rating Scale (C-SSRS) and Clinical Global Impression (CGI) in one trial. The other trial used BPRS and CDSS. | | | | | | | | | | | |
| Unclear/NR (2 RCTs) ^m^ | serious ^n^ | not serious ^o^ | not serious ^e^ | serious to very serious ^p^ | none ^g^ | unable to assess | Both trials reported on positive symptoms and one trial each on negative and depressive symptoms. There was no significant difference between groups for all symptoms. | | | | |
| **Adverse events (follow up: Unclear/NR)** ^k,l^  Trials had a primary aim of smoking cessation | | | | | | | | | | | |
| Unclear/NR (2 RCTs) ^m^ | serious ^n^ | not serious ^q^ | not serious ^e^ | serious to very serious ^p^ | none ^r^ | unable to assess | One trial reported no suicidal ideation. This study reported exacerbation of side effects in the varenicline arm, namely, constipation, insomnia, and nausea.  In the second trial, two participants with a history of suicide attempts assigned to varenicline were hospitalized; one of the participants overdosed and had a seizure resulting in hospitalization. In total, the trial reported 13 serious adverse events occurring in 9 participants assigned to varenicline and 1 participant assigned to placebo (2 varenicline participants experienced 3 serous adverse events related to treatment). One death from accidental drowning occurred in the varenicline arm during the long-term follow-up period (off-treatment); event not related to treatment according to authors. No treatment-related adverse events occurred in placebo arm.  This second trial also reported no difference between groups regarding other adverse events including neuropsychiatric SAEs and study discontinuation. The most common adverse events occurring in the varenicline arm were nausea (23.8%), headache (10.7%), and vomiting (10.7%). | | | | |
| **Adverse events (follow up: Unclear/NR)** ^s,t,u^  Trials examined varenicline for purposes other than smoking cessation, reduction, or relapse  The primary aim of the trials was to examine the effect on cognitive function (n=2) or alcohol dependence (n=1). | | | | | | | | | | | |
| Unclear/NR (3 RCTs) ^u,v^ | serious ^w^ | serious ^x^ | not serious ^y^ | serious to very serious ^z^ | none ^r^ | ⨁◯◯◯  VERY LOW | Data from two trials is inclusive of both smokers and non-smokers; remaining trial recruited smokers with alcohol dependence and had a small sample due to recruitment issues (n=10).  In one trial recruiting participants with both smoking and alcohol dependence, one participant assigned to varenicline withdrew from the trial due to passive suicidal ideation (7 days after starting varenicline), vomiting and irritability. Remaining two trials reported that among smokers and non-smokers, no participants had suicidal ideation and no increase in suicidal ideation occurred in those assigned varenicline, respectively.  One trial reported a trend toward reduced psychosis in the varenicline arm compared to placebo; both smokers and non-smokers were included in this analysis but study authors report no difference in treatment effect related to smoking status. In the second trial of smokers and non-smokers, two participants in each study arm withdrew due to exacerbation of psychotic symptoms.  One trial reported that among smokers and non-smokers, there was no difference between varenicline and placebo groups regarding common side effects of varenicline. However, the two other trials reported higher rates of common side effects in the varenicline arm (e.g., nausea, headache, vomiting, abdominal pain). In one of these studies, one patient withdrew due to nausea and vomiting.  Given what was reported, the one trial in smokers with alcohol dependence raised concerns regarding the safety and tolerability of varenicline in schizophrenic patients. | | | | |
| **Change in mental state: Positive (e.g. hallucinations, delusions), negative (e.g. anhedonia, avolition), depressive symptoms, and general symptoms of schizophrenia (follow up: Unclear/NR)** ^s,t,u^  Trials examined varenicline for purposes other than smoking cessation, reduction, or relapse  The primary aim of the trials was to examine the effect on cognitive function (n=2) or alcohol dependence (n=1). Outcome measurement: One trial measured effects on mental state using BPRS, SANS, HAM-D, and CGI. A second trial used PANSS, SANS, HAM-D, and CGI. Remaining trial used PANSS only. | | | | | | | | | | | |
| Unclear/NR (3 RCTs) ^u,v^ | serious ^w^ | not serious ^aa^ | not serious ^ab^ | serious to very serious ^ac^ | none ^g^ | unable to assess | Data from one trial in this analysis is inclusive of both smokers and non-smokers; remaining trials either only recruited smokers or reported data on these outcomes for smokers only. One study had a small sample due to recruitment issues (n=10).  Of the two trials reporting on change in positive symptoms, one reported no significant changes in symptoms among smokers and the other no significant difference between groups (inclusive of smokers and non-smokers).  For negative symptoms, one trial reported no significant change in symptoms among smokers and two trials reported no significant difference between groups (smokers and non-smokers included in the analysis for one of these two studies).  One trial reported no significant depressive symptoms among smokers and non-smokers during the study while another reported no significant difference between smokers in each group.  One trial also reported no significant change in general symptoms of schizophrenia among smokers. | | | | |

**AEs:** Adverse events; **BPRS:** Brief Psychiatric Rating Scale; **CDSS:** Calgary Depression Rating Scale; **CGI:** Clinical Global Impression; **CI:** Confidence interval; **CPD:** Cigarettes per day; **C-SSRS:** Columbia Suicide Severity Rating Scale; **HAM-D:** Hamilton Depression Rating Scale; **NR:** Not reported; **PANSS:** Positive And Negative Syndrome Scale; **RR:** Risk ratio; **SAEs:** Serious adverse events; **SANS:** Scale for the Assessment of Negative Symptoms

#### Explanations

a. All participants were interested in quitting.

b. Varenicline and Placebo: Trial examined 2 mg/day varenicline for about 12 weeks. Both arms received behavioural co-intervention.

c. The only trial included in this analysis is at unclear risk of selection, attrition, and performance/detection bias. We downrate this domain by -1.0.

d. Not applicable (single study).

e. No indirectness.

f. Confidence interval encompasses both harm (small but important) and benefit (large benefit). The optimal information size not met (total of 11 events), inadequate sample size (<2000 participants). We downrate this domain by -2.0.

g. Although search strategy is not completely comprehensive, trials reports negative findings. We do not downrate this domain.

h. Number of participants included in this analysis not explicitly reported by review authors. A total of 128 participants (varenicline = 85, placebo = 43) included in the trial. Analysis excludes abstainers.

i. The only trial included in this analysis is at unclear risk of selection, attrition, and performance/detection bias. Reduction in terms of expired CO was measured in the trial, however, it is unclear whether reduced levels of CO at follow-up was used to validate reduction in self-reported CPD. We downrate this domain by -1.0.

j. Unable to assess confidence intervals. Unable to assess optimal information size and sample size analyzed not reported but total of 128 total participants included. We cannot rate this domain.

k. All participants in one trial were interested in quitting. Participants’ interest in quitting not reported in one of the studies.

l. Varenicline and Placebo: Both trials examined 2 mg/day of varenicline for about 12 weeks. Behavioural co-intervention provided to both arms in both trials. In one of the trials, participants received counselling based on the American Lung Association Freedom from Smoking Program.

m. In total, there were 137 participants across both studies (varenicline = 89, placebo = 48). However, the number of participants included in this analysis unclear/NR.

n. Both trials included in this analysis are at unclear risk for three or more domains (i.e., selection, performance/detection, attrition, or selective reporting bias). We downrate this domain by -1.0.

o. Effect estimates not reported in the review; comparison of direction and magnitude of results not possible. Overall, results consistently show no difference between groups for all symptoms. We do not downrate this domain.

p. Unable to assess confidence intervals. Unable to assess optimal information size and sample size analyzed unclear but total of 137 total participants included. We cannot rate this domain.

q. There may be some variability in adverse events between groups but types and frequencies of adverse events are sparse. We downrate this domain by -0.5.

r. Search strategy not completely comprehensive but trials with negative findings included. We do not downrate this domain.

s. Participants in one trial were interested in reducing or quitting smoking. In remaining trials, smokers were not interested in quitting or interest in quitting was uncertain.

t. Varenicline and Placebo: Two trials examined 2 mg/day of varenicline for about 8 weeks; remaining trial examined 1 mg/day for the same duration. One small trial (n=10) provided participants of both arms with incentive and specialized behavioural counselling co-intervention (i.e., motivational interviewing for quitting or reducing smoking and drinking). Remaining trials provided no co-interventions.

u. Timepoint of follow-up for this outcome not reported in the review. Treatment duration in all trials is 8 weeks with one trial following participants to at least 10 weeks.

v. Number of participants included in this analysis is not clearly reported in the review. Overall, a total of 199 participants included across the three trials and of these, 113 participants were smokers (number of participants per arm NR). One trial was terminated due to low enrolment (n=10) and drop-outs/losses (n=6).

w. All trials at unclear risk of selection, performance/detection, and attrition bias. One of the trials is also at high risk of selective reporting bias. We downrate this domain by -1.0.

x. Effect estimates not reported in the review; comparison of direction and magnitude of results not possible. Narrative data suggestive of inconsistency across trials for some adverse events. We downrate this domain by -0.5 but also reflect the partial downrating (-0.5) from the indirectness domain here.

y. One trial provided incentive as co-intervention; we downrate this domain by -0.5 but reflect this in the inconsistency domain. Remaining two trials include both smokers and non-smokers; 45% of all participants across trials were non-smokers, however, unclear how many participants were included in this analysis and the proportion of which were non-smokers. No further downrating.

z. Unable to assess confidence intervals. Unable to assess optimal information size and sample size analyzed unclear but total of 199 total participants included. We cannot rate this domain, but suggesting a range for rating based on available information to assist with overall certainty of evidence.

aa. Effect estimates not reported in the review; comparison of direction and magnitude of results not possible. Trials consistently report no change in symptoms or no difference between groups. We do not downrate this domain.

ab. One trial provided incentive as co-intervention; we downrate this domain by -0.5. Outcome data for one trial is inclusive of smokers and non-smokers; however, it is unclear how many participants were included in this analysis and the proportion of which were non-smokers. No further downrating.

ac. Unable to assess confidence intervals. Unable to assess optimal information size and sample size analyzed unclear but total of 199 total participants included. We cannot rate this domain.

| Summary of findings: | | | | | | |
| --- | --- | --- | --- | --- | --- | --- |
| **Varenicline compared to placebo in smokers with schizophrenia or schizoaffective disorder** | | | | | | |
| **Patient or population**: Smokers with schizophrenia or schizoaffective disorder  **Setting**: No restriction  **Intervention**: Varenicline  **Comparison**: Placebo | | | | | | |
| Outcomes | **Anticipated absolute effects^*^** (95% CI) | | Relative effect (95% CI) | № of participants  (studies) | Certainty of the evidence (GRADE) | Comments |
|  | **Risk with placebo** | **Risk with Varenicline** |  |  |  |  |
| Abstinence/Cessation ^a,b^  Trial had a primary aim of smoking cessation  Outcome measurement: Point prevalence abstinence 100% of studies  Biochemical validation: 100% studies  Follow up: 6 months | 23 per 1,000 | **118 per 1,000** (16 to 889) | RR 5.06 (0.67 to 38.24) | 128 (1 RCT) | ⨁◯◯◯  VERY LOW ^c,d,e,f,g^ | Date of last search: October/November 2012.  AMSTAR-2: Moderate.  Not GRADEd by review authors. |
| Reduction in number of cigarettes/day from baseline ^a,b^  Trial had a primary aim of smoking cessation  Outcome measurement: Unclear/NR  Biochemical validation: Unclear/NR  Follow up: 6 months | Among continued smokers, there was no statistically significant difference between groups in reduction of cigarettes per day from baseline. | |  | Unclear/NR  (1 RCT) ^h^ | unable to assess ^d,e,g,i,j^ | Date of last search: October/November 2012.  AMSTAR-2: Moderate.  Not GRADEd by review authors.. |
| Change in mental state: Positive (e.g. hallucinations, delusions), negative (e.g. anhedonia, avolition), and depressive symptoms ^k,l^  Trials had a primary aim of smoking cessation  Outcome measurement: Effects on mental state measured using PANSS, SAS, Columbia Suicide Severity Rating Scale (C-SSRS) and Clinical Global Impression (CGI) in one trial. The other trial used BPRS and CDSS.  Follow up: End of treatment | Both trials reported on positive symptoms and one trial each on negative and depressive symptoms. There was no significant difference between groups for all symptoms. | |  | Unclear/NR  (2 RCTs) ^m^ | unable to assess ^e,g,n,o,p^ | Date of last search: October/November 2012.  AMSTAR-2: Moderate.  Not GRADEd by review authors. |
| Adverse events ^k,l^  Trials had a primary aim of smoking cessation.  Follow up: Unclear/NR | One trial reported no suicidal ideation. This study reported exacerbation of side effects in the varenicline arm, namely, constipation, insomnia, and nausea.  In the second trial, two participants with a history of suicide attempts assigned to varenicline were hospitalized; one of the participants overdosed and had a seizure resulting in hospitalization. In total, the trial reported 13 serious adverse events occurring in 9 participants assigned to varenicline and 1 participant assigned to placebo (2 varenicline participants experienced 3 serous adverse events related to treatment). One death from accidental drowning occurred in the varenicline arm during the long-term follow-up period (off-treatment); event not related to treatment according to authors. No treatment-related adverse events occurred in placebo arm.  This second trial also reported no difference between groups regarding other adverse events including neuropsychiatric SAEs and study discontinuation. The most common adverse events occurring in the varenicline arm were nausea (23.8%), headache (10.7%), and vomiting (10.7%). | |  | Unclear/NR  (2 RCTs) ^m^ | unable to assess ^e,n,p,q,r^ | Date of last search: October/November 2012.  AMSTAR-2: Moderate.  Not GRADEd by review authors. |
| Adverse events ^s,t,u^  Trials examined varenicline for purposes other than smoking cessation, reduction, or relapse  The primary aim of the trials was to examine the effect on cognitive function (n=2) or alcohol dependence (n=1).  Follow up: Unclear/NR | Data from two trials is inclusive of both smokers and non-smokers; remaining trial recruited smokers with alcohol dependence and had a small sample due to recruitment issues (n=10).  In one trial recruiting participants with both smoking and alcohol dependence, one participant assigned to varenicline withdrew from the trial due to passive suicidal ideation (7 days after starting varenicline), vomiting and irritability. Remaining two trials reported that among smokers and non-smokers, no participants had suicidal ideation and no increase in suicidal ideation occurred in those assigned varenicline, respectively.  One trial reported a trend toward reduced psychosis in the varenicline arm compared to placebo; both smokers and non-smokers were included in this analysis but study authors report no difference in treatment effect related to smoking status. In the second trial of smokers and non-smokers, two participants in each study arm withdrew due to exacerbation of psychotic symptoms.  One trial reported that among smokers and non-smokers, there was no difference between varenicline and placebo groups regarding common side effects of varenicline. However, the two other trials reported higher rates of common side effects in the varenicline arm (e.g., nausea, headache, vomiting, abdominal pain). In one of these studies, one patient withdrew due to nausea and vomiting.  Given what was reported, the one trial in smokers with alcohol dependence raised concerns regarding the safety and tolerability of varenicline in schizophrenic patients. | |  | Unclear/NR  (3 RCTs) ^u,v^ | ⨁◯◯◯  VERY LOW ^r,w,x,y,z^ | Review authors note that across all varenicline trials in the review, 2 of 144 participants on varenicline experienced suicidal ideation or behaviour. This includes one participant who overdosed in a smoking cessation trial (see above GRADE assessment) as well as one participant with passive suicidal ideation in a trial examining varenicline for purposes other than smoking cessation (as reported in this GRADE assessment).  Date of last search: October/November 2012.  AMSTAR-2: Moderate. Not GRADEd |
| Change in mental state: Positive (e.g. hallucinations, delusions), negative (e.g. anhedonia, avolition), depressive symptoms, and general symptoms of schizophrenia ^s,t,u^  Trials examined varenicline for purposes other than smoking cessation, reduction, or relapse. The primary aim of the trials was to examine the effect on cognitive function (n=2) or alcohol dependence (n=1).  Outcome measurement: One trial measured effects on mental state using BPRS, SANS, HAM-D, and CGI. A second trial used PANSS, SANS, HAM-D, and CGI. Remaining trial used PANSS only.  Follow up: Unclear/NR | Data from one trial in this analysis is inclusive of both smokers and non-smokers; remaining trials either only recruited smokers or reported data on these outcomes for smokers only. One study had a small sample due to recruitment issues (n=10).  Of the two trials reporting on change in positive symptoms, one reported no significant changes in symptoms among smokers and the other no significant difference between groups (inclusive of smokers and non-smokers).  For negative symptoms, one trial reported no significant change in symptoms among smokers and two trials reported no significant difference between groups (smokers and non-smokers included in the analysis for one of these two studies).  One trial reported no significant depressive symptoms among smokers and non-smokers during the study while another reported no significant difference between smokers in each group.  One trial also reported no significant change in general symptoms of schizophrenia among smokers. | |  | Unclear/NR  (3 RCTs) ^u,v^ | unable to assess ^g,w,aa,ab,ac^ | Date of last search: October/November 2012.  AMSTAR-2: Moderate.  Not GRADEd by review authors. |
| ***The risk in the intervention group** (and its 95% confidence interval) is based on the assumed risk in the comparison group and the **relative effect** of the intervention (and its 95% CI).   **AEs:** Adverse events; **BPRS:** Brief Psychiatric Rating Scale; **CDSS:** Calgary Depression Rating Scale; **CGI:** Clinical Global Impression; **CI:** Confidence interval; **CPD:** Cigarettes per day; **C-SSRS:** Columbia Suicide Severity Rating Scale; **HAM-D:** Hamilton Depression Rating Scale; **NR:** Not reported; **PANSS:** Positive And Negative Syndrome Scale; **RR:** Risk ratio;  **SAEs:** Serious adverse events; **SANS:** Scale for the Assessment of Negative Symptoms | | | | | | |
| **GRADE Working Group grades of evidence** **High certainty:** We are very confident that the true effect lies close to that of the estimate of the effect **Moderate certainty:** We are moderately confident in the effect estimate: The true effect is likely to be close to the estimate of the effect, but there is a possibility that it is substantially different **Low certainty:** Our confidence in the effect estimate is limited: The true effect may be substantially different from the estimate of the effect **Very low certainty:** We have very little confidence in the effect estimate: The true effect is likely to be substantially different from the estimate of effect | | | | | | |

#### Explanations

a. All participants were interested in quitting.

b. Varenicline and Placebo: Trial examined 2 mg/day varenicline for about 12 weeks. Both arms received behavioural co-intervention.

c. The only trial included in this analysis is at unclear risk of selection, attrition, and performance/detection bias. We downrate this domain by -1.0.

d. Not applicable (single study).

e. No indirectness.

f. Confidence interval encompasses both harm (small but important) and benefit (large benefit). The optimal information size not met (total of 11 events), inadequate sample size (<2000 participants). We downrate this domain by -2.0.

g. Although search strategy is not completely comprehensive, trials reports negative findings. We do not downrate this domain.

h. Number of participants included in this analysis not explicitly reported by review authors. A total of 128 participants (varenicline = 85, placebo = 43) included in the trial. Analysis excludes abstainers.

i. The only trial included in this analysis is at unclear risk of selection, attrition, and performance/detection bias. Reduction in terms of expired CO was measured in the trial, however, it is unclear whether reduced levels of CO at follow-up was used to validate reduction in self-reported CPD. We downrate this domain by -1.0.

j. Unable to assess confidence intervals. Unable to assess optimal information size and sample size analyzed not reported but total of 128 total participants included. We cannot rate this domain.

k. All participants in one trial were interested in quitting. Participants’ interest in quitting not reported in one of the studies.

l. Varenicline and Placebo: Both trials examined 2 mg/day of varenicline for about 12 weeks. Behavioural co-intervention provided to both arms in both trials. In one of the trials, participants received counselling based on the American Lung Association Freedom from Smoking Program.

m. In total, there were 137 participants across both studies (varenicline = 89, placebo = 48). However, the number of participants included in this analysis unclear/NR.

n. Both trials included in this analysis are at unclear risk for three or more domains (i.e., selection, performance/detection, attrition, or selective reporting bias). We downrate this domain by -1.0.

o. Effect estimates not reported in the review; comparison of direction and magnitude of results not possible. Overall, results consistently show no difference between groups for all symptoms. We do not downrate this domain.

p. Unable to assess confidence intervals. Unable to assess optimal information size and sample size analyzed unclear but total of 137 total participants included. We cannot rate this domain.

q. There may be some variability in adverse events between groups but types and frequencies of adverse events are sparse. We downrate this domain by -0.5.

r. Search strategy not completely comprehensive but trials with negative findings included. We do not downrate this domain.

s. Participants in one trial were interested in reducing or quitting smoking. In remaining trials, smokers were not interested in quitting or interest in quitting was uncertain.

t. Varenicline and Placebo: Two trials examined 2 mg/day of varenicline for about 8 weeks; remaining trial examined 1 mg/day for the same duration. One small trial (n=10) provided participants of both arms with incentive and specialized behavioural counselling co-intervention (i.e., motivational interviewing for quitting or reducing smoking and drinking). Remaining trials provided no co-interventions.

u. Timepoint of follow-up for this outcome not reported in the review. Treatment duration in all trials is 8 weeks with one trial following participants to at least 10 weeks.

v. Number of participants included in this analysis is not clearly reported in the review. Overall, a total of 199 participants included across the three trials and of these, 113 participants were smokers (number of participants per arm NR). One trial was terminated due to low enrolment (n=10) and drop-outs/losses (n=6).

w. All trials at unclear risk of selection, performance/detection, and attrition bias. One of the trials is also at high risk of selective reporting bias. We downrate this domain by -1.0.

x. Effect estimates not reported in the review; comparison of direction and magnitude of results not possible. Narrative data suggestive of inconsistency across trials for some adverse events. We downrate this domain by -0.5 but also reflect the partial downrating (-0.5) from the indirectness domain here.

y. One trial provided incentive as co-intervention; we downrate this domain by -0.5 but reflect this in the inconsistency domain. Remaining two trials include both smokers and non-smokers; 45% of all participants across trials were non-smokers, however, unclear how many participants were included in this analysis and the proportion of which were non-smokers. No further downrating.

z. Unable to assess confidence intervals. Unable to assess optimal information size and sample size analyzed unclear but total of 199 total participants included. We cannot rate this domain.

aa. Effect estimates not reported in the review; comparison of direction and magnitude of results not possible. Trials consistently report no change in symptoms or no difference between groups. We do not downrate this domain.

ab. One trial provided incentive as co-intervention; we downrate this domain by -0.5. Outcome data for one trial is inclusive of smokers and non-smokers; however, it is unclear how many participants were included in this analysis and the proportion of which were non-smokers. No further downrating.

ac. Unable to assess confidence intervals. Unable to assess optimal information size and sample size analyzed unclear but total of 199 total participants included. We cannot rate this domain.

## Appendix K Table 56. NRT patch vs Placebo patch: Change in mental state and adverse events in smokers with schizophrenia or schizoaffective disorder

| NRT patch compared to placebo patch in smokers with schizophrenia or schizoaffective disorder  **Bibliography:** Tsoi 2013; Date of last search: October/November 2012 | | | | | | | | | | | |
| --- | --- | --- | --- | --- | --- | --- | --- | --- | --- | --- | --- |
| **Certainty assessment** | | | | | | | **Summary of findings** | | | | |
| **№ of participants (studies) Follow-up** | **Risk of bias** | **Inconsistency** | **Indirectness** | **Imprecision** | **Publication bias** | **Overall certainty of evidence** | **Study event rates (%)** | | **Relative effect (95% CI)** | **Anticipated absolute effects** | |
|  |  |  |  |  |  |  | **With placebo patch** | **With NRT patch** |  | **Risk with placebo patch** | **Risk difference with NRT patch** |
| **Change in mental state (follow up: Unclear/NR)** ^a,b,c^  Trials had a primary aim of smoking reduction  Outcome measurement: One trial measured effects on mental state using BPRS, SANS, HAM-D. Review authors indicate effects on mental state were not measured in the second trial; this outcome appears to have been ascertained via observation. | | | | | | | | | | | |
| Unclear/NR (2 RCTs) ^c,d,e^ | serious ^f^ | not serious ^g^ | not serious ^h^ | serious to very serious ^i^ | none ^j^ | unable to assess | One trial reported no difference in psychiatric symptoms between NRT and placebo phases. In the second trial, no participant experienced a change in subjective experience or mental status. | | | | |
| **Adverse events (follow up: Unclear/NR)** ^b,k,l^  Trial had a primary aim of smoking reduction | | | | | | | | | | | |
| Unclear/NR (1 RCT) ^l,m^ | very serious ^n^ | not serious ^o^ | not serious ^p^ | serious to very serious ^q^ | none ^r^ | ⨁◯◯◯  VERY LOW | One cross-over trial reported that 60% of participants experienced an increase in abnormal involuntary movement when using NRT patch. The increase was reported as statistically significant when participants were smoking and using NRT patch. | | | | |

**BPRS:** Brief Psychiatric Rating Scale; **CI:** Confidence interval; **HAM-D:** Hamilton Depression Rating Scale; **NR:** Not reported; **NRT:** Nicotine replacement therapy; **SANS:** Scale for the Assessment of Negative Symptoms

#### Explanations

a. Participants in both trials were not interested in quitting.
b. No co-interventions provided to either arm.
c. Timing of outcome assessment unclear/NR. Treatment duration was 7 hours in one trial (8mg) and 32 hours (22 mg/day) in the second.
d. Number of participants in this analysis not explicitly reported by authors. A total of 20 participants with schizophrenia and schizoaffective disorder included across trials. Number of participants per study arm not reported.
e. Both studies were cross-over trials.
f. One trial at unclear risk for three domains (i.e., selection, performance/detection, and attrition bias). The second study at high risk for selective reporting bias and at unclear risk for performance/detection and attrition. We downrate this domain by -1.5.
g. Effect estimates not reported. Overall, results show no changes or no difference between phases. We do not downrate this domain.
h. One trial recruited both inpatients and outpatients but proportion from each setting not reported. No indirectness in remaining study. We do not downrate this domain.
i. Unable to assess confidence intervals. Unclear sample size analyzed but a total of 20 participants and the optimal information size cannot be met. We cannot rate this domain.
j. Search strategy not completely comprehensive but trials report negative findings. We do not downrate this domain.
k. Participants were not interested in quitting.
l. Timing of outcome assessment is unclear/NR. Treatment duration was 32 hours (22 mg/day).
m. Number of participants in this analysis not explicitly reported by authors. A total of 10 participants were included in the trial. Number of participants per study arm not reported.
n. The only trial in this analysis is at unclear risk for performance/detection bias and attrition bias. The trial is also at high risk for selective reporting bias. We downrate this domain by -2.0.
o. Not applicable (single study).
p. No indirectness.
q. Unable to assess confidence intervals. Unclear sample size analyzed but a total of 10 participants and the optimal information size cannot be met. We cannot rate this domain.
r. Search strategy not completely comprehensive and trial reports positive findings. Given the outcome is adverse events, publication bias not strongly suspected.

| Summary of findings: | | | | | | |
| --- | --- | --- | --- | --- | --- | --- |
| **NRT patch compared to placebo patch in smokers with schizophrenia or schizoaffective disorder** | | | | | | |
| **Patient or population**: Smokers with schizophrenia or schizoaffective disorder  **Setting**: No restriction  **Intervention**: NRT patch  **Comparison**: Placebo patch | | | | | | |
| Outcomes | **Anticipated absolute effects^*^** (95% CI) | | Relative effect (95% CI) | № of participants  (studies) | Certainty of the evidence (GRADE) | Comments |
|  | **Risk with placebo patch** | **Risk with NRT patch** |  |  |  |  |
| Change in mental state ^a,b,c^  Trials had a primary aim of smoking reduction. Outcome measurement: One trial measured effects on mental state using BPRS, SANS, HAM-D.  Review authors indicate effects on mental state were not measured in the second trial; this outcome appears to have been ascertained via observation.  Follow up: Unclear/NR | One trial reported no difference in psychiatric symptoms between NRT and placebo phases. In the second trial, no participant experienced a change in subjective experience or mental status. | |  | Unclear/NR  (2 RCTs) ^d,e^ | unable to assess ^f,g,h,i,j^ | Date of last search: October/November 2012.  AMSTAR-2: Moderate.  Not GRADEd by review authors. |
| Adverse events ^b,k,l^  Trial had a primary aim of smoking reduction.  Follow up: Unclear/NR | One cross-over trial reported that 60% of participants experienced an increase in abnormal involuntary movement when using NRT patch. The increase was reported as statistically significant when participants were smoking and using NRT patch. | |  | Unclear/NR  (1 RCT) ^m^ | ⨁◯◯◯  VERY LOW ^n,o,p,q,r^ | Date of last search: October/November 2012.  AMSTAR-2: Moderate.  Not GRADEd by review authors. |
| ***The risk in the intervention group** (and its 95% confidence interval) is based on the assumed risk in the comparison group and the **relative effect** of the intervention (and its 95% CI).   **BPRS:** Brief Psychiatric Rating Scale; **CI:** Confidence interval; **HAM-D:** Hamilton Depression Rating Scale; **NR:** Not reported; **NRT:** Nicotine replacement therapy; **SANS:** Scale for the Assessment of Negative Symptoms | | | | | | |
| **GRADE Working Group grades of evidence** **High certainty:** We are very confident that the true effect lies close to that of the estimate of the effect **Moderate certainty:** We are moderately confident in the effect estimate: The true effect is likely to be close to the estimate of the effect, but there is a possibility that it is substantially different **Low certainty:** Our confidence in the effect estimate is limited: The true effect may be substantially different from the estimate of the effect **Very low certainty:** We have very little confidence in the effect estimate: The true effect is likely to be substantially different from the estimate of effect | | | | | | |

#### Explanations

a. Participants in both trials were not interested in quitting.
b. No co-interventions provided to either arm.
c. Timing of outcome assessment unclear/NR. Treatment duration was 7 hours in one trial (8mg) and 32 hours (22 mg/day) in the second.
d. Number of participants in this analysis not explicitly reported by authors. A total of 20 participants with schizophrenia and schizoaffective disorder included across trials. Number of participants per study arm not reported.
e. Both studies were cross-over trials.
f. One trial at unclear risk for three domains (i.e., selection, performance/detection, and attrition bias). The second study at high risk for selective reporting bias and at unclear risk for performance/detection and attrition. We downrate this domain by -1.5.
g. Effect estimates not reported. Overall, results show no changes or no difference between phases. We do not downrate this domain.
h. One trial recruited both inpatients and outpatients but proportion from each setting not reported. No indirectness in remaining study. We do not downrate this domain.
i. Unable to assess confidence intervals. Unclear sample size analyzed but a total of 20 participants and the optimal information size cannot be met. We cannot rate this domain.
j. Search strategy not completely comprehensive but trials report negative findings. We do not downrate this domain.
k. Participants were not interested in quitting.
l. Timing of outcome assessment is unclear/NR. Treatment duration was 32 hours (22 mg/day).
m. Number of participants in this analysis not explicitly reported by authors. A total of 10 participants were included in the trial. Number of participants per study arm not reported.
n. The only trial in this analysis is at unclear risk for performance/detection bias and attrition bias. The trial is also at high risk for selective reporting bias. We downrate this domain by -2.0.
o. Not applicable (single study).
p. No indirectness.
q. Unable to assess confidence intervals. Unclear sample size analyzed but a total of 10 participants and the optimal information size cannot be met. We cannot rate this domain.
r. Search strategy not completely comprehensive and trial reports positive findings. Given the outcome is adverse events, publication bias not strongly suspected.

## Appendix K Table 57. Individual smoking cessation intervention (cognitive behavioural therapy and motivational interviewing) plus NRT patch versus Routine care: Smoking cessation and reduction in smokers with schizophrenia or schizoaffective disorder

| Individual smoking cessation intervention based on cognitive behavioural therapy and motivational interviewing plus NRT patch compared to routine care in smokers with schizophrenia or schizoaffective disorder  **Bibliography:** Tsoi 2013; Date of last search: October/November 2012 | | | | | | | | | | | |
| --- | --- | --- | --- | --- | --- | --- | --- | --- | --- | --- | --- |
| **Certainty assessment** | | | | | | | **Summary of findings** | | | | |
| **№ of participants (studies) Follow-up** | **Risk of bias** | **Inconsistency** | **Indirectness** | **Imprecision** | **Publication bias** | **Overall certainty of evidence** | **Study event rates (%)** | | **Relative effect (95% CI)** | **Anticipated absolute effects** | |
|  |  |  |  |  |  |  | **With routine care** | **With Individual smoking cessation intervention based on cognitive behavioural therapy and motivational interviewing plus NRT patch** |  | **Risk with routine care** | **Risk difference with Individual smoking cessation intervention based on cognitive behavioural therapy and motivational interviewing plus NRT patch** |
| **Abstinence/Cessation (follow up: 6 months, 12 months, 4 years)** ^a,b,c^  Trial had a primary aim of smoking cessation  Outcome measurement: point prevalence and continuous/sustained abstinence  Biochemical validation: 100% studies | | | | | | | | | | | |
| Unclear/NR (1 RCT) ^d^ | serious ^e^ | not serious ^f^ | not serious ^g^ | serious to very serious ^h^ | none ^i^ | unable to assess | No statistically significant difference between groups in point prevalence or continuous abstinence rates at all follow-up time points. Threshold for statistical significance was p<0.01. | | | | |
| **Reduction in cigarettes/day of >50% of baseline (follow up: 6 months, 12 months, 4 years)** ^a,b,c^  Trial had a primary aim of smoking cessation  Outcome measurement: unclear/NR  Biochemical validation: 0% studies | | | | | | | | | | | |
| Unclear/NR (1 RCT) ^d^ | serious ^j^ | not serious ^f^ | not serious ^g^ | serious to very serious ^h^ | none ^i^ | unable to assess | No statistically significant difference between groups in smoking reduction at all follow-up time points. | | | | |

**CI:** Confidence interval; **CPD:** Cigarettes per day; **NR:** Not reported; **NRT:** Nicotine replacement therapy

#### Explanations

a. Trial recruited participants with psychotic disorders of mixed diagnoses however, subgroup data reported for those with schizophrenia and schizoaffective disorder. All participants were interested in quitting.
b. Intervention: Involved eight hours of individual contact for eight weeks. NRT patch provided for about 10 weeks (21 mg, 14 mg, 7 mg titrated down). Booklets on smoking cessation provided to participants as co-intervention.
c. Control: No extra contact time. Participants received booklets on smoking cessation as co-intervention.
d. Total of 169 participants included in the analysis but number per arm not reported.
e. The only trial in this analysis is at high risk of performance bias as blinding of participants was not possible due to the nature of the intervention. Abstinence was biochemically validated, however, participants that self-reported as abstinent were classified as such even if their expired CO level was greater than 10 ppm. The trial is also at unclear risk of selection bias. We downrate this domain by -1.5.
f. Not applicable (single study).
g. No indirectness.
h. Unable to assess confidence intervals. Unclear sample size analyzed but approximately 169 total participants and the optimal information size cannot be met. We cannot rate this domain.
i. Search strategy not completely comprehensive but trial reports negative findings. We do not downrate this domain.
j. The only trial in this analysis is at high risk of performance bias as blinding of participants was not possible due to the nature of the intervention. It is unclear whether self-reported CPD was biochemically validated. The trial is also at unclear risk of selection bias. We downrate this domain by -1.5.

| Summary of findings: | | | | | | |
| --- | --- | --- | --- | --- | --- | --- |
| **Individual smoking cessation intervention based on cognitive behavioural therapy and motivational interviewing plus NRT patch compared to routine care in smokers with schizophrenia or schizoaffective disorder** | | | | | | |
| **Patient or population**: Smokers with schizophrenia or schizoaffective disorder  **Setting**: No restriction  **Intervention**: Individual smoking cessation intervention based on cognitive behavioural therapy and motivational interviewing plus NRT patch  **Comparison**: Routine care | | | | | | |
| Outcomes | **Anticipated absolute effects^*^** (95% CI) | | Relative effect (95% CI) | № of participants  (studies) | Certainty of the evidence (GRADE) | Comments |
|  | **Risk with routine care** | **Risk with Individual smoking cessation intervention based on cognitive behavioural therapy and motivational interviewing plus NRT patch** |  |  |  |  |
| Abstinence/Cessation ^a,b,c^  Trial had a primary aim of smoking cessation.  Outcome measurement: point prevalence and continuous/sustained abstinence  Biochemical validation: 100% studies  Follow up: 6 months, 12 months, 4 years | No statistically significant difference between groups in point prevalence or continuous abstinence rates at all follow-up time points. Threshold for statistical significance was p<0.01. | |  | Unclear/NR  (1 RCT) ^d^ | unable to assess ^e,f,g,h,i^ | Date of last search: October/November 2012.  AMSTAR-2: Moderate.  Not GRADEd by review authors. |
| Reduction in cigarettes/day of >50% of baseline ^a,b,c^  Trial had a primary aim of smoking cessation.  Outcome measurement: unclear/NR  Biochemical validation: 0% studies  Follow up: 6 months, 12 months, 4 years | No statistically significant difference between groups in smoking reduction at all follow-up time points. | |  | Unclear/NR  (1 RCT) ^d^ | unable to assess ^f,g,h,i,j^ | Date of last search: October/November 2012.  AMSTAR-2: Moderate.  Not GRADEd by review authors. |
| ***The risk in the intervention group** (and its 95% confidence interval) is based on the assumed risk in the comparison group and the **relative effect** of the intervention (and its 95% CI).   **CI:** Confidence interval; **CPD:** Cigarettes per day; **NR:** Not reported; **NRT:** Nicotine replacement therapy | | | | | | |
| **GRADE Working Group grades of evidence** **High certainty:** We are very confident that the true effect lies close to that of the estimate of the effect **Moderate certainty:** We are moderately confident in the effect estimate: The true effect is likely to be close to the estimate of the effect, but there is a possibility that it is substantially different **Low certainty:** Our confidence in the effect estimate is limited: The true effect may be substantially different from the estimate of the effect **Very low certainty:** We have very little confidence in the effect estimate: The true effect is likely to be substantially different from the estimate of effect | | | | | | |

#### Explanations

a. Trial recruited participants with psychotic disorders of mixed diagnoses however, subgroup data reported for those with schizophrenia and schizoaffective disorder. All participants were interested in quitting.
b. Intervention: Involved eight hours of individual contact for eight weeks. NRT patch provided for about 10 weeks (21 mg, 14 mg, 7 mg titrated down). Booklets on smoking cessation provided to participants as co-intervention.
c. Control: No extra contact time. Participants received booklets on smoking cessation as co-intervention.
d. Total of 169 participants included in the analysis but number per arm not reported.
e. The only trial in this analysis is at high risk of performance bias as blinding of participants was not possible due to the nature of the intervention. Abstinence was biochemically validated, however, participants that self-reported as abstinent were classified as such even if their expired CO level was greater than 10 ppm. The trial is also at unclear risk of selection bias. We downrate this domain by -1.5.
f. Not applicable (single study).
g. No indirectness.
h. Unable to assess confidence intervals. Unclear sample size analyzed but approximately 169 total participants and the optimal information size cannot be met. We cannot rate this domain.
i. Search strategy not completely comprehensive but trial reports negative findings. We do not downrate this domain.
j. The only trial in this analysis is at high risk of performance bias as blinding of participants was not possible due to the nature of the intervention. It is unclear whether self-reported CPD was biochemically validated. The trial is also at unclear risk of selection bias. We downrate this domain by -1.5.

# Van der Meer 2013 {1223}

## Appendix K Table 58. Bupropion versus Placebo: Smoking cessation in smokers with current depression

| Bupropion compared to placebo in smokers with current depression  **Bibliography:** van der Meer 2013; Date of last search: April 2013 | | | | | | | | | | | |
| --- | --- | --- | --- | --- | --- | --- | --- | --- | --- | --- | --- |
| **Certainty assessment** | | | | | | | **Summary of findings** | | | | |
| **№ of participants (studies) Follow-up** | **Risk of bias** | **Inconsistency** | **Indirectness** | **Imprecision** | **Publication bias** | **Overall certainty of evidence** | **Study event rates (%)** | | **Relative effect (95% CI)** | **Anticipated absolute effects** | |
|  |  |  |  |  |  |  | **With placebo** | **With Bupropion** |  | **Risk with placebo** | **Risk difference with Bupropion** |
| **Abstinence/Cessation (follow up: 6-12 months)** ^a,b,c^  Outcome measurement: point prevalence 20%, continuous/sustained abstinence 60%, prolonged 20% studies  Biochemical validation: 100% studies | | | | | | | | | | | |
| 410 (5 RCTs) | serious ^d^ | not serious ^e^ | not serious ^f^ | very serious ^g^ | none ^h^ | ⨁◯◯◯  VERY LOW | 23/206 (11.2%) | 31/204 (15.2%) | RR 1.37 (0.83 to 2.27) | 112 per 1,000 | **41 more per 1,000** (from 19 fewer to 142 more) |

**CI:** Confidence interval; **RR:** Risk ratio

#### Explanations

a. Studies examined general smokers (n=1), African-American smokers (n=1), female smokers motivated to quit (n=1), hospitalized smokers with acute cardiovascular disease (n=1) and smokers with cancer (n=1). All studies had subgroups of participants with mild depressive symptoms; subgroups were prestated in two studies and post hoc in three studies.
b. Bupropion: Across trials, 300mg/day for 7 weeks to 6 months.
c. Bupropion and Placebo: All studies provided a behavioural co-intervention to both arms; one study also provided NRT patch to both arms. Across studies, at least 48% of participants in each arm received specialized behavioural counselling (CBT or relapse prevention counselling).
d. 50% of the evidence is at unclear risk of bias for at least one domain or at high and unclear risk for one domain each (primarily selection and attrition bias). In all but one trial, it was clear that participants and staff were blind to medication condition but not for the psychosocial components; for the purpose of this comparison, such studies are considered to be at low risk of performance/detection bias. We downrate this domain by -0.5 but also reflect the partial downrating (-0.5) in the indirectness domain here.
e. Effect estimates vary but confidence intervals largely overlap (I2=29%, p=0.23). We do not downrate this domain.
f. 24% of the evidence is indirect (inpatient setting); we downrate this domain by -0.5 but reflect this in the risk of bias domain. One other trial was conducted in a hospital setting but unclear with participants were in- or out-patients.
g. Confidence interval encompasses both harm (small but important) and benefit (large). The optimal information size not met (total of 54 events) and inadequate sample size (<2000 participants). We downrate this domain by -2.0.
h. Although search is not completely comprehensive, all studies report negative findings. We do not downrate this domain.

| Summary of findings: | | | | | | |
| --- | --- | --- | --- | --- | --- | --- |
| **Bupropion compared to placebo in smokers with current depression** | | | | | | |
| **Patient or population**: Smokers with current depression  **Setting**: No restriction  **Intervention**: Bupropion  **Comparison**: Placebo | | | | | | |
| Outcomes | **Anticipated absolute effects^*^** (95% CI) | | Relative effect (95% CI) | № of participants  (studies) | Certainty of the evidence (GRADE) | Comments |
|  | **Risk with placebo** | **Risk with Bupropion** |  |  |  |  |
| Abstinence/Cessation ^a,b,c^  Outcome measurement: point prevalence 20%, continuous/sustained abstinence 60%, prolonged 20% studies  Biochemical validation: 100% studies  Follow up: 6-12 months | 112 per 1,000 | **153 per 1,000** (93 to 253) | RR 1.37 (0.83 to 2.27) | 410 (5 RCTs) | ⨁◯◯◯  VERY LOW ^d,e,f,g,h^ | Data for patients with current depression derived from subgroup analyses in all studies; subgroups were post hoc in three of the five studies.  Fixed effects analysis.  AMSTAR-2: Critically low.  Date of last search: April 2013.  Review authors rated certainty as low. ^i^ |
| ***The risk in the intervention group** (and its 95% confidence interval) is based on the assumed risk in the comparison group and the **relative effect** of the intervention (and its 95% CI).   **CI:** Confidence interval; **RR:** Risk ratio | | | | | | |
| **GRADE Working Group grades of evidence** **High certainty:** We are very confident that the true effect lies close to that of the estimate of the effect **Moderate certainty:** We are moderately confident in the effect estimate: The true effect is likely to be close to the estimate of the effect, but there is a possibility that it is substantially different **Low certainty:** Our confidence in the effect estimate is limited: The true effect may be substantially different from the estimate of the effect **Very low certainty:** We have very little confidence in the effect estimate: The true effect is likely to be substantially different from the estimate of effect | | | | | | |

#### Explanations

a. Studies examined general smokers (n=1), African-American smokers (n=1), female smokers motivated to quit (n=1), hospitalized smokers with acute cardiovascular disease (n=1) and smokers with cancer (n=1). All studies had subgroups of participants with mild depressive symptoms; subgroups were prestated in two studies and post hoc in three studies.
b. Bupropion: Across trials, 300mg/day for 7 weeks to 6 months.
c. Bupropion and Placebo: All studies provided a behavioural co-intervention to both arms; one study also provided NRT patch to both arms. Across studies, at least 48% of participants in each arm received specialized behavioural counselling (CBT or relapse prevention counselling).
d. 50% of the evidence is at unclear risk of bias for at least one domain or at high and unclear risk for one domain each (primarily selection and attrition bias). In all but one trial, it was clear that participants and staff were blind to medication condition but not for the psychosocial components; for the purpose of this comparison, such studies are considered to be at low risk of performance/detection bias. We downrate this domain by -0.5 but also reflect the partial downrating (-0.5) in the indirectness domain here.
e. Effect estimates vary but confidence intervals largely overlap (I2=29%, p=0.23). We do not downrate this domain.
f. 24% of the evidence is indirect (inpatient setting); we downrate this domain by -0.5 but reflect this in the risk of bias domain. One other trial was conducted in a hospital setting but unclear with participants were in- or out-patients.
g. Confidence interval encompasses both harm (small but important) and benefit (large). The optimal information size not met (total of 54 events) and inadequate sample size (<2000 participants). We downrate this domain by -2.0.
h. Although search is not completely comprehensive, all studies report negative findings. We do not downrate this domain.
i. Review authors rated the certainty as low; downrated for risk of bias (all studies with some unclear risk) and imprecision (small number of events and wide CIs),

## Appendix K Table 59. Bupropion versus Placebo: Smoking cessation in smokers with past depression

| Bupropion compared to placebo in smokers with past depression  **Bibliography:** van der Meer 2013; Date of last search: April 2013 | | | | | | | | | | | |
| --- | --- | --- | --- | --- | --- | --- | --- | --- | --- | --- | --- |
| **Certainty assessment** | | | | | | | **Summary of findings** | | | | |
| **№ of participants (studies) Follow-up** | **Risk of bias** | **Inconsistency** | **Indirectness** | **Imprecision** | **Publication bias** | **Overall certainty of evidence** | **Study event rates (%)** | | **Relative effect (95% CI)** | **Anticipated absolute effects** | |
|  |  |  |  |  |  |  | **With placebo** | **With Bupropion** |  | **Risk with placebo** | **Risk difference with Bupropion** |
| **Abstinence/Cessation (follow up: 6-12 months)** ^a,b,c^  Outcome measurement: point prevalence 75%, continuous/sustained abstinence 25% studies  Biochemical validation: 100% studies | | | | | | | | | | | |
| 404 (4 RCTs) | serious ^d^ | not serious ^e^ | not serious ^f^ | serious ^g^ | none ^h^ | ⨁⨁◯◯  LOW | 24/195 (12.3%) | 56/209 (26.8%) | RR 2.04 (1.31 to 3.18) | 123 per 1,000 | **128 more per 1,000** (from 38 more to 268 more) |

**CI:** Confidence interval; **RR:** Risk ratio

#### Explanations

a. All studies recruit general smokers and report post hoc subgroup data for participants with past depression.
b. Bupropion: Across trials, 300 mg/day for 7 to 12 weeks. All studies provide a behavioural co-intervention to participants receiving bupropion; one four arm trial also provided either NRT or placebo patch (study reports two comparisons entered separately in meta-analysis: (1) bupropion plus nicotine patch versus placebo tablet plus nicotine patch, (2) bupropion plus placebo patch versus placebo tablet plus placebo patch). 22% of participants received specialized behavioural counselling (CBT).
c. Placebo: All studies provide a behavioural co-intervention to participants in this arm; one four arm trial also provided either NRT or placebo patch. 32% of participants received specialized behavioural counselling (CBT).
d. 90% of the evidence is at unclear risk of bias for at least one domain (selection, performance/detection, and attrition bias). A small proportion of evidence is at high risk of bias (10%). In two trials, it was clear that participants and staff were blind to medication condition but not for the psychosocial components; for the purpose of this comparison, such studies are considered to be at low risk of performance/detection bias. We downrate this domain by -0.5 but also reflect the partial downrating (-0.5) from the inconsistency domain here.
e. Effect estimates vary but confidence intervals mostly overlap (I2=44%, p=0.13). We downrate this domain by -0.5 but reflect this in the risk of bias domain.
f. Due to reporting, directness could not be ascertained for two studies. One study was in a hospital setting but unclear whether participants were in- or out- patients. The other recruited participants from a mix of settings; proportion of participants from university setting NR. We do not downrate this domain.
g. Confidence interval encompasses one range of effect (moderate benefit to large benefit). The optimal information size not met (total of 80 events) and inadequate sample size (<2000 participants). We downrate this domain by -1.0.
h. Although search is not completely comprehensive, mix of findings reported across studies. We do not downrate this domain.

| Summary of findings: | | | | | | |
| --- | --- | --- | --- | --- | --- | --- |
| **Bupropion compared to placebo in smokers with past depression** | | | | | | |
| **Patient or population**: Smokers with past depression  **Setting**: No restriction  **Intervention**: Bupropion  **Comparison**: Placebo | | | | | | |
| Outcomes | **Anticipated absolute effects^*^** (95% CI) | | Relative effect (95% CI) | № of participants  (studies) | Certainty of the evidence (GRADE) | Comments |
|  | **Risk with placebo** | **Risk with Bupropion** |  |  |  |  |
| Abstinence/Cessation ^a,b,c^  Outcome measurement: point prevalence 75%, continuous/sustained abstinence 25% studies  Biochemical validation: 100% studies  Follow up: 6-12 months | 123 per 1,000 | **251 per 1,000** (161 to 391) | RR 2.04 (1.31 to 3.18) | 404 (4 RCTs) | ⨁⨁◯◯  LOW ^d,e,f,g,h^ | Review authors caution that data for patients with past depression derived from post hoc subgroup analyses in all studies.  Fixed effects analysis.  AMSTAR-2: Critically low.  Date of last search: April 2013.  Review authors rated the certainty as low. ^i^ |
| ***The risk in the intervention group** (and its 95% confidence interval) is based on the assumed risk in the comparison group and the **relative effect** of the intervention (and its 95% CI).   **CI:** Confidence interval; **RR:** Risk ratio | | | | | | |
| **GRADE Working Group grades of evidence** **High certainty:** We are very confident that the true effect lies close to that of the estimate of the effect **Moderate certainty:** We are moderately confident in the effect estimate: The true effect is likely to be close to the estimate of the effect, but there is a possibility that it is substantially different **Low certainty:** Our confidence in the effect estimate is limited: The true effect may be substantially different from the estimate of the effect **Very low certainty:** We have very little confidence in the effect estimate: The true effect is likely to be substantially different from the estimate of effect | | | | | | |

#### Explanations

a. All studies recruit general smokers and report post hoc subgroup data for participants with past depression.
b. Bupropion: Across trials, 300 mg/day for 7 to 12 weeks. All studies provide a behavioural co-intervention to participants receiving bupropion; one four arm trial also provided either NRT or placebo patch (study reports two comparisons entered separately in meta-analysis: (1) bupropion plus nicotine patch versus placebo tablet plus nicotine patch, (2) bupropion plus placebo patch versus placebo tablet plus placebo patch). 22% of participants received specialized behavioural counselling (CBT).
c. Placebo: All studies provide a behavioural co-intervention to participants in this arm; one four arm trial also provided either NRT or placebo patch. 32% of participants received specialized behavioural counselling (CBT).
d. 90% of the evidence is at unclear risk of bias for at least one domain (selection, performance/detection, and attrition bias). A small proportion of evidence is at high risk of bias (10%). In two trials, it was clear that participants and staff were blind to medication condition but not for the psychosocial components; for the purpose of this comparison, such studies are considered to be at low risk of performance/detection bias. We downrate this domain by -0.5 but also reflect the partial downrating (-0.5) from the inconsistency domain here.
e. Effect estimates vary but confidence intervals mostly overlap (I2=44%, p=0.13). We downrate this domain by -0.5 but reflect this in the risk of bias domain.
f. Due to reporting, directness could not be ascertained for two studies. One study was in a hospital setting but unclear whether participants were in- or out- patients. The other recruited participants from a mix of settings; proportion of participants from university setting NR. We do not downrate this domain.
g. Confidence interval encompasses one range of effect (moderate benefit to large benefit). The optimal information size not met (total of 80 events) and inadequate sample size (<2000 participants). We downrate this domain by -1.0.
h. Although search is not completely comprehensive, mix of findings reported across studies. We do not downrate this domain.
i. Review authors rated the certainty as low; downrated for risk of bias (all studies with some unclear or high risk) and imprecision (small number of events).

## Appendix K Table 60. NRT gum versus Placebo: Smoking cessation in smokers with current depression

| NRT gum compared to placebo in smokers with current depression  **Bibliography:** van der Meer 2013; Date of last search: April 2013 | | | | | | | | | | | |
| --- | --- | --- | --- | --- | --- | --- | --- | --- | --- | --- | --- |
| **Certainty assessment** | | | | | | | **Summary of findings** | | | | |
| **№ of participants (studies) Follow-up** | **Risk of bias** | **Inconsistency** | **Indirectness** | **Imprecision** | **Publication bias** | **Overall certainty of evidence** | **Study event rates (%)** | | **Relative effect (95% CI)** | **Anticipated absolute effects** | |
|  |  |  |  |  |  |  | **With placebo** | **With NRT gum** |  | **Risk with placebo** | **Risk difference with NRT gum** |
| **Abstinence/Cessation (follow up: 12 months)** ^a,b,c^  Outcome measurement: continuous/sustained abstinence 100% studies  Biochemical validation: 100% studies | | | | | | | | | | | |
| 196 (1 RCT) | serious ^d^ | not serious ^e^ | not serious ^f^ | very serious ^g^ | none ^h^ | ⨁◯◯◯  VERY LOW | 4/70 (5.7%) | 19/126 (15.1%) | RR 2.64 (0.93 to 7.45) | 57 per 1,000 | **94 more per 1,000** (from 4 fewer to 369 more) |

**CI:** Confidence interval; **NRT:** Nicotine replacement therapy; **RR:** Risk ratio

#### Explanations

a. The only trial in this analysis recruited general smokers but reports post hoc subgroup data for participants with current depression.
b. NRT gum: 2 or 4 mg with recommendation of 9-15 pieces per day for 2 months followed by weaning.
c. Behavioural co-intervention provided to both study arms.
d. The only study in this analysis is at unclear risk of bias for three domains (selection, performance/detection, and attrition bias). We downrate this domain by -1.0.
e. Not applicable - single study.
f. No indirectness.
g. Confidence interval encompasses three ranges of effect (little to no difference to large benefit). The optimal information size not met (total of 23 events) and inadequate sample size (<2000 participants). We downrate this domain by -2.0.
h. Search is not completely comprehensive but trial reports negative findings. We do not downrate this domain.

| Summary of findings: | | | | | | |
| --- | --- | --- | --- | --- | --- | --- |
| **NRT gum compared to placebo in smokers with current depression** | | | | | | |
| **Patient or population**: Smokers with current depression  **Setting**: No restriction  **Intervention**: NRT gum  **Comparison**: Placebo | | | | | | |
| Outcomes | **Anticipated absolute effects^*^** (95% CI) | | Relative effect (95% CI) | № of participants  (studies) | Certainty of the evidence (GRADE) | Comments |
|  | **Risk with placebo** | **Risk with NRT gum** |  |  |  |  |
| Abstinence/Cessation ^a,b,c^  Outcome measurement: continuous/sustained abstinence 100% studies  Biochemical validation: 100% studies  Follow up: 12 months | 57 per 1,000 | **151 per 1,000** (53 to 426) | RR 2.64 (0.93 to 7.45) | 196 (1 RCT) | ⨁◯◯◯  VERY LOW ^d,e,f,g,h^ | Data for participants with current depression derived from post hoc subgroup analysis,  AMSTAR-2: Critically low.  Date of last search: April 2013.  Not GRADEd by review authors. |
| ***The risk in the intervention group** (and its 95% confidence interval) is based on the assumed risk in the comparison group and the **relative effect** of the intervention (and its 95% CI).   **CI:** Confidence interval; **NRT:** Nicotine replacement therapy; **RR:** Risk ratio | | | | | | |
| **GRADE Working Group grades of evidence** **High certainty:** We are very confident that the true effect lies close to that of the estimate of the effect **Moderate certainty:** We are moderately confident in the effect estimate: The true effect is likely to be close to the estimate of the effect, but there is a possibility that it is substantially different **Low certainty:** Our confidence in the effect estimate is limited: The true effect may be substantially different from the estimate of the effect **Very low certainty:** We have very little confidence in the effect estimate: The true effect is likely to be substantially different from the estimate of effect | | | | | | |

#### Explanations

a. The only trial in this analysis recruited general smokers but reports post hoc subgroup data for participants with current depression.
b. NRT gum: 2 or 4 mg with recommendation of 9-15 pieces per day for 2 months followed by weaning.
c. Behavioural co-intervention provided to both study arms.
d. The only study in this analysis is at unclear risk of bias for three domains (selection, performance/detection, and attrition bias). We downrate this domain by -1.0.
e. Not applicable - single study.
f. No indirectness.
g. Confidence interval encompasses three ranges of effect (little to no difference to large benefit). The optimal information size not met (total of 23 events) and inadequate sample size (<2000 participants). We downrate this domain by -2.0.
h. Search is not completely comprehensive but trial reports negative findings. We do not downrate this domain.

## Appendix K Table 61. NRT versus Placebo: Smoking cessation in smokers with past depression

| NRT compared to placebo in smokers with past depression  **Bibliography:** van der Meer 2013; Date of last search: April 2013 | | | | | | | | | | | |
| --- | --- | --- | --- | --- | --- | --- | --- | --- | --- | --- | --- |
| **Certainty assessment** | | | | | | | **Summary of findings** | | | | |
| **№ of participants (studies) Follow-up** | **Risk of bias** | **Inconsistency** | **Indirectness** | **Imprecision** | **Publication bias** | **Overall certainty of evidence** | **Study event rates (%)** | | **Relative effect (95% CI)** | **Anticipated absolute effects** | |
|  |  |  |  |  |  |  | **With placebo** | **With NRT** |  | **Risk with placebo** | **Risk difference with NRT** |
| **Abstinence/Cessation (follow up: 6+ months)** ^a,b,c^  Outcome measurement: point prevalence 67%, continuous/sustained abstinence 33% studies  Biochemical validation: 100% studies | | | | | | | | | | | |
| 432 (3 RCTs) | not serious ^d^ | not serious ^e^ | not serious ^f^ | very serious ^g^ | none ^h^ | ⨁⨁◯◯  LOW | 49/196 (25.0%) | 65/236 (27.5%) | RR 1.17 (0.85 to 1.60) | 250 per 1,000 | **42 more per 1,000** (from 38 fewer to 150 more) |

**CI:** Confidence interval; **NRT:** Nicotine replacement therapy; **RR:** Risk ratio

#### Explanations

a. All studies recruit general smokers and report subgroup data for participants with past depression; subgroup analysis was pre-stated in one study and post hoc in two.
b. One study examined NRT gum and another NRT patch. The third study examined NRT patch, lozenge, and patch plus lozenge (arms entered separately in meta-analysis). NRT gum: 2 mg for 8 weeks and tapering to week 11; NRT patch (both trials): 21, 14, and 7 mg titrated down during 8 weeks after quit date; NRT lozenge: 2 or 4 mg according to dependence for 12 weeks.
c. NRT and Placebo: All studies provided a behavioural co-intervention to both arms; one four arm trial also provided bupropion or placebo tablets (study reports two comparisons entered separately in meta-analysis: (1) bupropion plus nicotine patch versus bupropion plus placebo patch, (2) placebo tablet plus nicotine patch versus placebo tablet plus placebo patch). In one study, one of the behavioural co-interventions provided was mood management; proportion of participants receiving this co-intervention is unclear.
d. 90% of the evidence is at unclear risk of bias for one domain (performance/detection or attrition bias). In one trial, it was clear that participants and staff were blind to medication condition but not for the psychosocial components; for the purpose of this comparison, this study is considered to be at low risk of performance/detection bias. Remaining evidence (10%) is at unclear risk for three domains (selection, performance/detection, and attrition bias). We downrate this domain by -0.5.
e. Little variation in effect estimates and confidence intervals largely overlap (I2=0%, p=0.95). We do not downrate this domain.
f. 10% of the evidence is indirect (university setting). We do not downrate this domain.
g. Confidence interval encompasses both harm (moderate) and benefit (large). The optimal information size not met (total of 114 events) and inadequate sample size (<2000 participants). We downrate this domain by -2.0.
h. Search is not completely comprehensive, but all studies report negative findings. We do not downrate this domain.

| Summary of findings: | | | | | | |
| --- | --- | --- | --- | --- | --- | --- |
| **NRT compared to placebo in smokers with past depression** | | | | | | |
| **Patient or population**: Smokers with past depression  **Setting**: No restriction  **Intervention**: NRT  **Comparison**: Placebo | | | | | | |
| Outcomes | **Anticipated absolute effects^*^** (95% CI) | | Relative effect (95% CI) | № of participants  (studies) | Certainty of the evidence (GRADE) | Comments |
|  | **Risk with placebo** | **Risk with NRT** |  |  |  |  |
| Abstinence/Cessation ^a,b,c^  Outcome measurement: point prevalence 67%, continuous/sustained abstinence 33% studies  Biochemical validation: 100% studies  Follow up: 6+ months | 250 per 1,000 | **293 per 1,000** (213 to 400) | RR 1.17 (0.85 to 1.60) | 432 (3 RCTs) | ⨁⨁◯◯  LOW ^d,e,f,g,h^ | Data for patients with past depression derived from subgroup analyses in all studies; subgroups were post hoc in two of the three studies.  Fixed effects analysis.  AMSTAR-2: Critically low.  Date of last search: April 2013.  Not GRADEd by review authors. |
| ***The risk in the intervention group** (and its 95% confidence interval) is based on the assumed risk in the comparison group and the **relative effect** of the intervention (and its 95% CI).   **CI:** Confidence interval; **NRT:** Nicotine replacement therapy; **RR:** Risk ratio | | | | | | |
| **GRADE Working Group grades of evidence** **High certainty:** We are very confident that the true effect lies close to that of the estimate of the effect **Moderate certainty:** We are moderately confident in the effect estimate: The true effect is likely to be close to the estimate of the effect, but there is a possibility that it is substantially different **Low certainty:** Our confidence in the effect estimate is limited: The true effect may be substantially different from the estimate of the effect **Very low certainty:** We have very little confidence in the effect estimate: The true effect is likely to be substantially different from the estimate of effect | | | | | | |

#### Explanations

a. All studies recruit general smokers and report subgroup data for participants with past depression; subgroup analysis was prestated in one study and post hoc in two.
b. One study examined NRT gum and another NRT patch. The third study examined NRT patch, lozenge and patch plus lozenge (arms entered separately in meta-analysis). NRT gum: 2 mg for 8 weeks and tapering to week 11; NRT patch (both trials): 21, 14, and 7 mg titrated down during 8 weeks after quit date; NRT lozenge: 2 or 4 mg according to dependence for 12 weeks.
c. NRT and Placebo: All studies provided a behavioural co-intervention to both arms; one four arm trial also provided bupropion or placebo tablets (study reports two comparisons entered separately in meta-analysis: (1) bupropion plus nicotine patch versus bupropion plus placebo patch, (2) placebo tablet plus nicotine patch versus placebo tablet plus placebo patch). In one study, one of the behavioural co-interventions provided was mood management; proportion of participants receiving this co-intervention is unclear.
d. 90% of the evidence is at unclear risk of bias for one domain (performance/detection or attrition bias). In one trial, it was clear that participants and staff were blind to medication condition but not for the psychosocial components; for the purpose of this comparison, this study is considered to be at low risk of performance/detection bias. Remaining evidence (10%) is at unclear risk for three domains (selection, performance/detection, and attrition bias). We downrate this domain by -0.5.
e. Little variation in effect estimates and confidence intervals largely overlap (I2=0%, p=0.95). We do not downrate this domain.
f. 10% of the evidence is indirect (university setting). We do not downrate this domain.
g. Confidence interval encompasses both harm (moderate) and benefit (large). The optimal information size not met (total of 114 events) and inadequate sample size (<2000 participants). We downrate this domain by -2.0.
h. Search is not completely comprehensive but all studies report negative findings. We do not downrate this domain.

## Appendix K Table 62. Standard treatment plus extended NRT and extended CBT versus Standard treatment: Smoking cessation in smokers with past depression

| Standard treatment plus extended NRT and extended CBT compared to standard treatment in smokers with past depression  **Bibliography:** van der Meer 2013; Date of last search: April 2013 | | | | | | | | | | | |
| --- | --- | --- | --- | --- | --- | --- | --- | --- | --- | --- | --- |
| **Certainty assessment** | | | | | | | **Summary of findings** | | | | |
| **№ of participants (studies) Follow-up** | **Risk of bias** | **Inconsistency** | **Indirectness** | **Imprecision** | **Publication bias** | **Overall certainty of evidence** | **Study event rates (%)** | | **Relative effect (95% CI)** | **Anticipated absolute effects** | |
|  |  |  |  |  |  |  | **With standard treatment** | **With Standard treatment plus extended NRT and extended CBT** |  | **Risk with standard treatment** | **Risk difference with Standard treatment plus extended NRT and extended CBT** |
| **Abstinence/Cessation (follow up: Unclear/NR)** ^a,b,c,d^  Outcome measurement: point prevalence 100% studies  Biochemical validation: 100% studies | | | | | | | | | | | |
| 57 (1 RCT) ^d^ | serious ^e^ | not serious ^f^ | not serious ^g^ | very serious ^h^ | none ^i^ | ⨁◯◯◯  VERY LOW | 8/29 (27.6%) | 15/28 (53.6%) | RR 1.94 (0.98 to 3.85) | 276 per 1,000 | **259 more per 1,000** (from 6 fewer to 786 more) |

**CI:** Confidence interval; **NR:** Not reported; **NRT:** Nicotine replacement therapy; **RR:** Risk ratio

#### Explanations

a. The only trial in this analysis recruited general smokers but reports subgroup data for participants with past depression.
b. Standard treatment consisted of “12 weeks of sustained release bupropion (150 mg/d first; from week 2 300 mg/d) and 10 weeks of 2 mg and 4 mg nicotine gum, and received 5 group counselling sessions (week 1, 3 (2 sessions), 5 and 8) from a counsellor and a self-help manual. No further treatment after week 12.” No other co-interventions provided.
c. In addition to standard treatment, the intervention arm received NRT until week 52 and 11 individual CBT sessions from week 10 to 52. No other co-interventions provided.
d. Study had multiple follow-up timepoints; unclear which timepoint this data pertains to. Longest timepoint of follow-up was 24 months.
e. The only study in this analysis is at high risk of bias for performance/detection bias (participants not blind and unclear for study staff). We downrate this domain by -1.0.
f. Not applicable - single study.
g. No indirectness.
h. Confidence interval encompasses three ranges of effect (little to no difference to large benefit). The optimal information size is not met (total of 23 events) and inadequate sample size (<2000 participants). We downrate this domain by -2.0.
i. Search is not completely comprehensive, but study reports negative findings. We do not downrate this domain.

| Summary of findings: | | | | | | |
| --- | --- | --- | --- | --- | --- | --- |
| **Standard treatment plus extended NRT and extended CBT compared to standard treatment in smokers with past depression** | | | | | | |
| **Patient or population**: Smokers with past depression  **Setting**: No restriction  **Intervention**: Standard treatment plus extended NRT and extended CBT  **Comparison**: Standard treatment | | | | | | |
| Outcomes | **Anticipated absolute effects^*^** (95% CI) | | Relative effect (95% CI) | № of participants  (studies) | Certainty of the evidence (GRADE) | Comments |
|  | **Risk with standard treatment** | **Risk with Standard treatment plus extended NRT and extended CBT** |  |  |  |  |
| Abstinence/Cessation ^a,b,c^  Outcome measurement: point prevalence 100% studies  Biochemical validation: 100% studies  Follow up: Unclear/NR ^d^ | 276 per 1,000 | **535 per 1,000** (270 to 1,000) | RR 1.94 (0.98 to 3.85) | 57 (1 RCT) | ⨁◯◯◯  VERY LOW ^e,f,g,h,i^ | Data for participants with current depression derived from prestated subgroup analysis.  AMSTAR-2: Critically low.  Date of last search: April 2013.  Not GRADEd by review authors. |
| ***The risk in the intervention group** (and its 95% confidence interval) is based on the assumed risk in the comparison group and the **relative effect** of the intervention (and its 95% CI).   **CI:** Confidence interval; **NR:** Not reported; **NRT:** Nicotine replacement therapy; **RR:** Risk ratio | | | | | | |
| **GRADE Working Group grades of evidence** **High certainty:** We are very confident that the true effect lies close to that of the estimate of the effect **Moderate certainty:** We are moderately confident in the effect estimate: The true effect is likely to be close to the estimate of the effect, but there is a possibility that it is substantially different **Low certainty:** Our confidence in the effect estimate is limited: The true effect may be substantially different from the estimate of the effect **Very low certainty:** We have very little confidence in the effect estimate: The true effect is likely to be substantially different from the estimate of effect | | | | | | |

#### Explanations

a. The only trial in this analysis recruited general smokers but reports subgroup data for participants with past depression.
b. Standard treatment consisted of “12 weeks of sustained release bupropion (150 mg/d first; from week 2 300 mg/d) and 10 weeks of 2 mg and 4 mg nicotine gum, and received 5 group counselling sessions (week 1, 3 (2 sessions), 5 and 8) from a counsellor and a self-help manual. No further treatment after week 12.” No other co-interventions provided.
c. In addition to standard treatment, the intervention arm received NRT until week 52 and 11 individual CBT sessions from week 10 to 52. No other co-interventions provided.
d. Study had multiple follow-up timepoints; unclear which timepoint this data pertains to. Longest timepoint of follow-up was 24 months.
e. The only study in this analysis is at high risk of bias for performance/detection bias (participants not blind and unclear for study staff). We downrate this domain by -1.0.
f. Not applicable - single study.
g. No indirectness.
h. Confidence interval encompasses three ranges of effect (little to no difference to large benefit). The optimal information size is not met (total of 23 events) and inadequate sample size (<2000 participants). We downrate this domain by -2.0.
i. Search is not completely comprehensive but study reports negative findings. We do not downrate this domain.

# Vodoplivec-Jamsek 2012 {1343}

## Appendix K Table 63. Mobile phone short message service vs Control: Smoking cessation in smokers motivated/wishing to quit

| Mobile phone short message service compared to control in smokers motivated/wishing to quit  **Bibliography:** Vodoplivec-Jamsek 2012; Date of last search: June 2009 | | | | | | | | | | | |
| --- | --- | --- | --- | --- | --- | --- | --- | --- | --- | --- | --- |
| **Certainty assessment** | | | | | | | **Summary of findings** | | | | |
| **№ of participants (studies) Follow-up** | **Risk of bias** | **Inconsistency** | **Indirectness** | **Imprecision** | **Publication bias** | **Overall certainty of evidence** | **Study event rates (%)** | | **Relative effect (95% CI)** | **Anticipated absolute effects** | |
|  |  |  |  |  |  |  | **With Control** | **With Mobile phone SMS** |  | **Risk with Control** | **Risk difference with Mobile phone SMS** |
| **Abstinence/cessation (follow up: 6 months)** ^a,b,c,d^  Outcome measurement: point prevalence 100% studies  Biochemical validation: 0% studies | | | | | | | | | | | |
| 1705 (1 RCT) | serious ^e^ | not serious ^f^ | not serious ^g^ | very serious ^h^ | none ^i^ | ⨁◯◯◯  VERY LOW | 202/853 (23.7%) | 216/852 (25.4%) | RR 1.07 (0.91 to 1.26) | 237 per 1,000 | **17 more per 1,000** (from 21 fewer to 62 more) |
| **Abstinence/cessation (follow up: 6 months)** ^a,b,c,d,j^  Outcome measurement: point prevalence 100% studies  Biochemical validation: 0% studies | | | | | | | | | | | |
| 1705 (1 RCT) | serious ^e^ | not serious ^f^ | not serious ^g^ | serious ^k^ | none ^i^ | ⨁⨁◯◯  LOW | 233/853 (27.3%) | 298/852 (35.0%) | RR 1.28 (1.11 to 1.48) | 273 per 1,000 | **76 more per 1,000** (from 30 more to 131 more) |
| **Abstinence/cessation (follow up: 6 months)** ^a,b,c,d,l^  Outcome measurement: continuous/sustained abstinence: 100% studies  Biochemical validation: 0% studies | | | | | | | | | | | |
| 1705 (1 RCT) | serious ^e^ | not serious ^f^ | not serious ^g^ | serious ^m^ | none ^i^ | ⨁⨁◯◯  LOW | 39/853 (4.6%) | 64/852 (7.5%) | RR 1.64 (1.12 to 2.42) | 46 per 1,000 | **29 more per 1,000** (from 5 more to 65 more) |
| **Abstinence/cessation (follow up: 6 months)** ^a,b,c,d,n^  Outcome measurement: continuous, complete abstinence 100% studies  Biochemical validation: 0% studies | | | | | | | | | | | |
| 1705 (1 RCT) | serious ^e^ | not serious ^f^ | not serious ^g^ | serious ^o^ | none ^i^ | ⨁⨁◯◯  LOW | 26/853 (3.0%) | 39/852 (4.6%) | RR 1.50 (0.92 to 2.44) | 30 per 1,000 | **15 more per 1,000** (from 2 fewer to 44 more) |
| **Adverse events: Car crash (follow up: 6 months)** ^a,b,c,d^  Outcome measurement: Not reported | | | | | | | | | | | |
| 1705 (1 RCT) | not serious ^p^ | not serious ^f^ | not serious ^g^ | very serious ^q^ | none ^i^ | ⨁⨁◯◯  LOW | 43/853 (5.0%) | 38/852 (4.5%) | RR 0.88 (0.58 to 1.35) | 50 per 1,000 | **6 fewer per 1,000** (from 21 fewer to 18 more) |
| **Adverse events: Pain in thumb/finger joints (follow up: 6 months)** ^a,b,c,d^  Outcome measurement: Not reported | | | | | | | | | | | |
| 1705 (1 RCT) | not serious ^p^ | not serious ^f^ | not serious ^g^ | very serious ^r^ | none ^i^ | ⨁⨁◯◯  LOW | 48/853 (5.6%) | 52/852 (6.1%) | RR 1.08 (0.74 to 1.59) | 56 per 1,000 | **5 more per 1,000** (from 15 fewer to 33 more) |

**CI:** Confidence interval; **RR:** Risk ratio; **SMS**: short message service

#### Explanations

a. Current daily smokers from community in New Zealand. Owners of mobile phone.
b. Text messaging intervention: Sent regular, personalized text messages with smoking cessation advice, support, and distraction, which decreased in frequency over time. Messages sent between healthcare provider or buddy (lay health worker or peer supported) and participant.
c. Control: Sham text messaging. One text message biweekly thanking participants for involvement and reminder of free month of text messaging if they complete follow-up.
d. Co-interventions (both groups): unclear/not reported.
e. High risk for self-report of cessation measure and unclear risk for selective reporting. We downrate by -1.5.
f. One study only. We do not downrate.
g. An evaluation of the study-level information provided in the review signalled no issues of indirectness to the question of interest. We do not downrate this domain.
h. Confidence interval encompasses both harm (small but important) and benefit (moderate). The optimal information size is met (total of 418 events), but inadequate sample size (<2000 participants). We downrate this domain by -2.0.
i. Evidence base too small to formally evaluate. Comprehensive literature search. Unlikely that studies are missing.
j. Point prevalence using last outcome carried forward.
k. Confidence interval encompasses two ranges of effect (small but important benefit to large benefit). The optimal information size is met (total of 531 events), but inadequate sample size (<2000 participants). We downrate this domain by -1.5.
l. Allowing three or fewer lapses of two or fewer cigarettes per lapse.
m. Confidence interval encompasses two ranges of effect (little to no difference to moderate benefit). The optimal information size not met (total of 103 events), inadequate sample size (<2000 participants). We downrate this domain by -1.5.
n. No lapses allowed.
o. Confidence interval encompasses two ranges of effect (little to no difference to moderate benefit). The optimal information size not met (total of 65 events), inadequate sample size (<2000 participants). We downrate this domain by -1.5.
p. Unclear risk of bias for selective reporting. We downrate by -0.5.
q. Confidence interval encompasses both benefit (small but important) and harm (small but important). The optimal information size not met (total of 100 events), inadequate sample size (<2000 participants). We downrate this domain by -2.0.

r. Confidence interval encompasses both benefit (small but important) and harm (small but important). The optimal information size not met (total of 81 events), inadequate sample size (<2000 participants). We downrate this domain by -2.0.

| Summary of findings: | | | | | | |
| --- | --- | --- | --- | --- | --- | --- |
| **Mobile phone short message service compared to control in smokers motivated/wishing to quit** | | | | | | |
| **Patient or population**: Smokers motivated/wishing to quit  **Setting**: Not restricted  **Intervention**: Mobile phone short message service  **Comparison**: Control | | | | | | |
| Outcomes | **Anticipated absolute effects^*^** (95% CI) | | Relative effect (95% CI) | № of participants  (studies) | Certainty of the evidence (GRADE) | Comments |
|  | **Risk with Control** | **Risk with Mobile phone SMS** |  |  |  |  |
| Abstinence/cessation ^a,b,c,d^  Outcome measurement: point prevalence 100% studies  Biochemical validation: 0% studies  Follow up: 6 months | 237 per 1,000 | **253 per 1,000** (215 to 298) | RR 1.07 (0.91 to 1.26) | 1705 (1 RCT) | ⨁◯◯◯  VERY LOW ^e,f,g,h,i^ | Authors state no difference between groups.  Date of last search: June 2009.  AMSTAR-2: Low.  Authors do not GRADE this analysis. |
| Abstinence/cessation ^a,b,c,d,j^  Outcome measurement: point prevalence 100% studies  Biochemical validation: 0% studies  Follow up: 6 months | 273 per 1,000 | **350 per 1,000** (303 to 404) | RR 1.28 (1.11 to 1.48) | 1705 (1 RCT) | ⨁⨁◯◯  LOW ^e,f,g,i,k^ | Authors state that a difference between groups observed when using last outcome carried forward.  Date of last search: June 2009.  AMSTAR-2: Low.  Authors rate as high certainty. |
| Abstinence/cessation ^a,b,c,d,l^  Outcome measurement: continuous/sustained abstinence 100% studies  Biochemical validation: 0% studies  Follow up: 6 months | 46 per 1,000 | **75 per 1,000** (51 to 111) | RR 1.64 (1.12 to 2.42) | 1705 (1 RCT) | ⨁⨁◯◯  LOW ^e,f,g,i,m^ | Authors state that cessation higher with text messaging when allowing lapses during continuous abstinence.  Date of last search: June 2009.  AMSTAR-2: Low.  Authors rate as high certainty. |
| Abstinence/cessation ^a,b,c,d,n^  Outcome measurement: continuous/sustained, complete abstinence 100% studies  Biochemical validation: 0% studies  Follow up: 6 months | 30 per 1,000 | **46 per 1,000** (28 to 74) | RR 1.50 (0.92 to 2.44) | 1705 (1 RCT) | ⨁⨁◯◯  LOW ^e,f,g,i,o^ | Authors state no difference between groups when continuous cessation required no lapses.  Date of last search: June 2009.  AMSTAR-2: Low.  Authors rate as high certainty. |
| Adverse events: Car crash ^a,b,c,d^  Outcome measurement: Not reported  Follow up: 6 months | 50 per 1,000 | **44 per 1,000** (29 to 68) | RR 0.88 (0.58 to 1.35) | 1705 (1 RCT) | ⨁⨁◯◯  LOW ^f,g,i,p,q^ | Authors state no difference between groups in rate of car crashes; 5 crashes occurred while texting or thereafter whereas 3 others occurred while smoking.  Date of last search: June 2009.  AMSTAR-2: Low.  Authors rate as moderate certainty. ^r^ |
| Adverse events: Pain in thumb/finger joints ^a,b,c,d^  Outcome measurement: Not reported  Follow up: 6 months | 56 per 1,000 | **61 per 1,000** (42 to 89) | RR 1.08 (0.74 to 1.59) | 1705 (1 RCT) | ⨁⨁◯◯  LOW ^f,g,i,p,s^ | Authors state no difference between groups for occurrence of pain in thumb or finger joints during texting.  Date of last search: June 2009.  AMSTAR-2: Low.  Authors rate as moderate certainty. ^r^ |
| ***The risk in the intervention group** (and its 95% confidence interval) is based on the assumed risk in the comparison group and the **relative effect** of the intervention (and its 95% CI).   **CI:** Confidence interval; **RR:** Risk ratio; **SMS**: Short message service | | | | | | |
| **GRADE Working Group grades of evidence** **High certainty:** We are very confident that the true effect lies close to that of the estimate of the effect **Moderate certainty:** We are moderately confident in the effect estimate: The true effect is likely to be close to the estimate of the effect, but there is a possibility that it is substantially different **Low certainty:** Our confidence in the effect estimate is limited: The true effect may be substantially different from the estimate of the effect **Very low certainty:** We have very little confidence in the effect estimate: The true effect is likely to be substantially different from the estimate of effect | | | | | | |

#### Explanations

a. Current daily smokers from community in New Zealand. Owners of mobile phone.
b. Text messaging intervention: Sent regular, personalized text messages with smoking cessation advice, support, and distraction, which decreased in frequency over time. Messages sent between healthcare provider or buddy (lay health worker or peer supported) and participant.
c. Control: Sham text messaging. One text message biweekly thanking participants for involvement and reminder of free month of text messaging if they complete follow-up.
d. Co-interventions (both groups): unclear/not reported.
e. High risk for self-report of cessation measure and unclear risk for selective reporting. We downrate by -1.5.
f. One study only. We do not downrate.
g. An evaluation of the study-level information provided in the review signalled no issues of indirectness to the question of interest. We do not downrate this domain.
h. Confidence interval encompasses both harm (small but important) and benefit (moderate). The optimal information size is met (total of 418 events), but inadequate sample size (<2000 participants). We downrate this domain by -2.0.
i. Evidence base too small to formally evaluate. Comprehensive literature search. Unlikely that studies are missing.
j. Point prevalence using last outcome carried forward.
k. Confidence interval encompasses two ranges of effect (small but important benefit to large benefit). The optimal information size is met (total of 531 events), but inadequate sample size (<2000 participants). We downrate this domain by -1.5.
l. Allowing three or fewer lapses of two or fewer cigarettes per lapse.
m. Confidence interval encompasses two ranges of effect (little to no difference to moderate benefit). The optimal information size not met (total of 103 events), inadequate sample size (<2000 participants). We downrate this domain by -1.5.
n. No lapses allowed.
o. Confidence interval encompasses two ranges of effect (little to no difference to moderate benefit). The optimal information size not met (total of 65 events), inadequate sample size (<2000 participants). We downrate this domain by -1.5.
p. Unclear risk of bias for selective reporting. We downrate by -0.5.
q. Confidence interval encompasses both benefit (small but important) and harm (small but important). The optimal information size not met (total of 100 events), inadequate sample size (<2000 participants). We downrate this domain by -2.0.

r. Authors downrate for very low numbers of adverse events reported in both groups.

s. Confidence interval encompasses both benefit (small but important) and harm (small but important). The optimal information size not met (total of 81 events), inadequate sample size (<2000 participants). We downrate this domain by -2.0.

# White 2014 {1618}

## Appendix K Table 64. Acupuncture versus Sham: Smoking cessation in smokers motivated/wishing to quit

| Acupuncture compared to sham acupuncture in smokers motivated/wishing to quit  **Bibliography:** White 2014; Date of last search: September/October 2016 | | | | | | | | | | | |
| --- | --- | --- | --- | --- | --- | --- | --- | --- | --- | --- | --- |
| **Certainty assessment** | | | | | | | **Summary of findings** | | | | |
| **№ of participants (studies) Follow-up** | **Risk of bias** | **Inconsistency** | **Indirectness** | **Imprecision** | **Publication bias** | **Overall certainty of evidence** | **Study event rates (%)** | | **Relative effect (95% CI)** | **Anticipated absolute effects** | |
|  |  |  |  |  |  |  | **With sham acupuncture** | **With Acupuncture** |  | **Risk with sham acupuncture** | **Risk difference with Acupuncture** |
| **Abstinence/cessation (follow up: 6-12 months)** ^a,b,c^  Outcome measurement: point prevalence 19%, continuous/sustained abstinence 36%, unclear/NR 46% studies  Biochemical validation 27% | | | | | | | | | | | |
| 1892 (11 RCTs) | serious ^d^ | not serious ^e^ | not serious ^f^ | serious ^g^ | none ^h^ | ⨁⨁◯◯  LOW | 97/895 (10.8%) | 122/997 (12.2%) | RR 1.10 (0.86 to 1.40) | 108 per 1,000 | **11 more per 1,000** (from 15 fewer to 43 more) |

**CI:** Confidence interval; **NR:** Not reported; **RR:** Risk ratio

#### Explanations

a. Population eligibility criterion of review is smokers wishing to quit. However, motivation or interest in quitting is not explicitly stated in review evidence tables for any of the trials included in this analysis. All studies recruited general smokers.
b. Acupuncture: All trials selected acupuncture points (i.e., anatomic sites) for smoking cessation. Two studies used facial acupuncture, five used auricular acupuncture alone with/without continuous stimulation (i.e., needle or pressure device), and four used combined body and auricular acupuncture with/without continuous stimulation (i.e., indwelling needle or seed). Behavioural co-intervention provided in some trials, pharmacotherapy in one study, and ‘other’ (i.e., placebo nicotine gum) in one study.
c. Sham: Two trials, representing 4% of the evidence, used potentially active acupuncture points for the sham arm. Behavioural co-intervention provided to control arm in some trials, pharmacotherapy in one study, and ‘other’ (i.e., placebo nicotine gum) in one study.
d. 95% of evidence with substantive bias issues; 86% at high risk of bias for one domain (biochemical validation) and unclear risk for two domains (selection, attrition bias), 10% of evidence at high risk of bias for one domain +/- unclear risk in one domain. Also, there is little to no difference, as it was felt the key risk of bias elements (e.g., biochemical validation) would likely exaggerate the effect We downrate this domain by -1.0.
e. Point estimates vary but confidence intervals largely overlap (I2=23%, p=0.23). We do not downrate this domain.
f. 0.5% of evidence is indirect (i.e., workplace setting). We do not downrate in this domain.
g. The point estimate and lower CI has little to no difference which suggest fewer concerns regarding imprecision. We downrate this domain by -1.0.
h. Although search not completely comprehensive, all studies report negative findings. Authors detected funnel plot asymmetry for evidence with short follow-up (<6 months); no assessment for long-term follow-up. We do not downrate this domain.

| Summary of findings: | | | | | | |
| --- | --- | --- | --- | --- | --- | --- |
| **Acupuncture compared to sham acupuncture in smokers motivated/wishing to quit** | | | | | | |
| **Patient or population**: Smokers motivated/wishing to quit  **Setting**: No restriction  **Intervention**: Acupuncture  **Comparison**: Sham acupuncture | | | | | | |
| Outcomes | **Anticipated absolute effects^*^** (95% CI) | | Relative effect (95% CI) | № of participants  (studies) | Certainty of the evidence (GRADE) | Comments |
|  | **Risk with sham acupuncture** | **Risk with Acupuncture** |  |  |  |  |
| Abstinence/cessation ^a,b,c^  Outcome measurement: point prevalence 19%, continuous/sustained abstinence 36%, unclear/NR 46% studies  Biochemical validation 27%  Follow up: 6-12 months | 108 per 1,000 | **119 per 1,000** (93 to 152) | RR 1.10 (0.86 to 1.40) | 1892 (11 RCTs) | ⨁◯◯◯  VERY LOW ^d,e,f,g,h^ | Sensitivity analysis excluding studies which used potentially active acupuncture points for the sham arm yielded similar results.  Fixed effects analysis.  Date of last search: September/October 2016.  AMSTAR-2: Low.  Review authors rated certainty as moderate. ^i^ |
| ***The risk in the intervention group** (and its 95% confidence interval) is based on the assumed risk in the comparison group and the **relative effect** of the intervention (and its 95% CI).   **CI:** Confidence interval; **NR:** Not reported; **RR:** Risk ratio | | | | | | |
| **GRADE Working Group grades of evidence** **High certainty:** We are very confident that the true effect lies close to that of the estimate of the effect **Moderate certainty:** We are moderately confident in the effect estimate: The true effect is likely to be close to the estimate of the effect, but there is a possibility that it is substantially different **Low certainty:** Our confidence in the effect estimate is limited: The true effect may be substantially different from the estimate of the effect **Very low certainty:** We have very little confidence in the effect estimate: The true effect is likely to be substantially different from the estimate of effect | | | | | | |

#### Explanations

a. Population eligibility criterion of review is smokers wishing to quit. However, motivation or interest in quitting is not explicitly stated in review evidence tables for any of the trials included in this analysis. All studies recruited general smokers.
b. Acupuncture: All trials selected acupuncture points (i.e., anatomic sites) for smoking cessation. Two studies used facial acupuncture, five used auricular acupuncture alone with/without continuous stimulation (i.e., needle or pressure device), and four used combined body and auricular acupuncture with/without continuous stimulation (i.e., indwelling needle or seed). Behavioural co-intervention provided in some trials, pharmacotherapy in one study, and ‘other’ (i.e., placebo nicotine gum) in one study.
c. Sham: Two trials, representing 4% of the evidence, used potentially active acupuncture points for the sham arm. Behavioural co-intervention provided to control arm in some trials, pharmacotherapy in one study, and ‘other’ (i.e., placebo nicotine gum) in one study.
d. 95% of evidence with substantive bias issues; 86% at high risk of bias for one domain (biochemical validation) and unclear risk for two domains (selection, attrition bias), 10% of evidence at high risk of bias for one domain +/- unclear risk in one domain. We downrate this domain by -2.0.
e. Point estimates vary but confidence intervals largely overlap (I2=23%, p=0.23). We do not downrate this domain.
f. 0.5% of evidence is indirect (i.e., workplace setting). We do not downrate in this domain.
g. Confidence interval encompasses both harm (important) and benefit (moderate). The optimal information size not met (total of 219 events) and inadequate sample size (<2000 participants). We downrate this domain by -2.0.
h. Although search not completely comprehensive, all studies report negative findings. Authors detected funnel plot asymmetry for evidence with short follow-up (<6 months); no assessment for long-term follow-up. We do not downrate this domain.
i. Review authors rate certainty of evidence as moderate; they downrate for publication bias according to funnel plot asymmetry at short-term follow-up (<6 months). They do not downrate for risk of bias as results are null-inclusive.

## Appendix K Table 65. Acupuncture versus Waiting list/no intervention: Smoking cessation in smokers motivated/wishing to quit

| Acupuncture compared to waiting list/no intervention in smokers motivated/wishing to quit  **Bibliography:** White 2014; Date of last search: September/October 2016 | | | | | | | | | | | |
| --- | --- | --- | --- | --- | --- | --- | --- | --- | --- | --- | --- |
| **Certainty assessment** | | | | | | | **Summary of findings** | | | | |
| **№ of participants (studies) Follow-up** | **Risk of bias** | **Inconsistency** | **Indirectness** | **Imprecision** | **Publication bias** | **Overall certainty of evidence** | **Study event rates (%)** | | **Relative effect (95% CI)** | **Anticipated absolute effects** | |
|  |  |  |  |  |  |  | **With waiting list/no intervention** | **With Acupuncture** |  | **Risk with waiting list/no intervention** | **Risk difference with Acupuncture** |
| **Abstinence/Cessation (follow up: 6-12 months)** ^a,b^  Outcome measurement: continuous/sustained abstinence 33%, unclear/NR 67% studies  Biochemical validation 0% | | | | | | | | | | | |
| 393 (3 RCTs) | very serious ^c^ | serious ^d^ | not serious ^e^ | very serious ^f^ | none ^g^ | ⨁◯◯◯  VERY LOW | 15/196 (7.7%) | 27/197 (13.7%) | RR 1.79 (0.98 to 3.28) | 77 per 1,000 | **60 more per 1,000** (from 2 fewer to 174 more) |

**CI:** Confidence interval; **NR:** Not reported; **RR:** Risk ratio

#### Explanations

a. Population eligibility criterion was smokers wishing to quit. However, motivation or interest in quitting was specified in review evidence tables for only one of the trials included in this analysis. Remaining studies recruited general smokers with no indication of motivation/interest to quit.
b. All trials selected acupuncture points (i.e., anatomic sites) for smoking cessation. One study used facial acupuncture and two used auricular acupuncture alone with or without continuous stimulation (i.e., needle or pressure device). One study provided a behavioural co-intervention to both study arms. No co-interventions in remaining studies.
c. 100% of evidence with substantive bias issues; 67% at high risk for one or more domains (performance/detection, biochemical validation) and unclear risk for two domains (selection, attrition bias), 33% of evidence at high risk for one domain and unclear risk for another. We downrate this domain by -2.0.
d. Effect estimates vary and the confidence intervals overlap somewhat (I2=57%, p=0.10). We downrate this domain by -1.0.
e. No indirectness.
f. Confidence intervals encompass three effect ranges (little to no difference as well as large benefit). The optimal information size not met (total of 42 events) and inadequate sample size (<2000 participants). We downrate this domain by -2.0.
g. Search strategy is not completely comprehensive, but all studies report negative findings. We do not downrate this domain.

| Summary of findings: | | | | | | |
| --- | --- | --- | --- | --- | --- | --- |
| **Acupuncture compared to waiting list/no intervention in smokers motivated/wishing to quit** | | | | | | |
| **Patient or population**: Smokers motivated/wishing to quit  **Setting**: No restriction  **Intervention**: Acupuncture  **Comparison**: Waiting list/no intervention | | | | | | |
| Outcomes | **Anticipated absolute effects^*^** (95% CI) | | Relative effect (95% CI) | № of participants  (studies) | Certainty of the evidence (GRADE) | Comments |
|  | **Risk with waiting list/no intervention** | **Risk with Acupuncture** |  |  |  |  |
| Abstinence/Cessation ^a,b^  Outcome measurement: continuous/sustained abstinence 33%, unclear/NR 67% studies  Biochemical validation 0%  Follow up: 6-12 months | 77 per 1,000 | **137 per 1,000** (75 to 251) | RR 1.79 (0.98 to 3.28) | 393 (3 RCTs) | ⨁◯◯◯  VERY LOW ^c,d,e,f,g^ | Fixed effects.  AMSTAR-2: Low.  Date of last search: September/October 2016.  Not GRADEd by review authors. |
| ***The risk in the intervention group** (and its 95% confidence interval) is based on the assumed risk in the comparison group and the **relative effect** of the intervention (and its 95% CI).   **CI:** Confidence interval; **NR:** Not reported; **RR:** Risk ratio | | | | | | |
| **GRADE Working Group grades of evidence** **High certainty:** We are very confident that the true effect lies close to that of the estimate of the effect **Moderate certainty:** We are moderately confident in the effect estimate: The true effect is likely to be close to the estimate of the effect, but there is a possibility that it is substantially different **Low certainty:** Our confidence in the effect estimate is limited: The true effect may be substantially different from the estimate of the effect **Very low certainty:** We have very little confidence in the effect estimate: The true effect is likely to be substantially different from the estimate of effect | | | | | | |

#### Explanations

a. Population eligibility criterion was smokers wishing to quit. However, motivation or interest in quitting was specified in review evidence tables for only one of the trials included in this analysis. Remaining studies recruited general smokers with no indication of motivation/interest to quit.
b. All trials selected acupuncture points (i.e., anatomic sites) for smoking cessation. One study used facial acupuncture and two used auricular acupuncture alone with or without continuous stimulation (i.e., needle or pressure device). One study provided a behavioural co-intervention to both study arms. No co-interventions in remaining studies.
c. 100% of evidence with substantive bias issues; 67% at high risk for one or more domains (performance/detection, biochemical validation) and unclear risk for two domains (selection, attrition bias), 33% of evidence at high risk for one domain and unclear risk for another. We downrate this domain by -2.0.
d. Effect estimates vary and the confidence intervals overlap somewhat (I2=57%, p=0.10). We downrate this domain by -1.0.
e. No indirectness.
f. Confidence intervals encompass three effect ranges (little to no difference as well as large benefit). The optimal information size not met (total of 42 events) and inadequate sample size (<2000 participants). We downrate this domain by -2.0.
g. Search strategy is not completely comprehensive, but all studies report negative findings. We do not downrate this domain.

## Appendix K Table 66. Continuous auricular stimulation versus Sham: Smoking cessation in smokers motivated/wishing to quit

| Continuous auricular stimulation compared to sham stimulation in smokers motivated/wishing to quit  **Bibliography:** White 2014; Date of last search: September/October 2016 | | | | | | | | | | | |
| --- | --- | --- | --- | --- | --- | --- | --- | --- | --- | --- | --- |
| **Certainty assessment** | | | | | | | **Summary of findings** | | | | |
| **№ of participants (studies) Follow-up** | **Risk of bias** | **Inconsistency** | **Indirectness** | **Imprecision** | **Publication bias** | **Overall certainty of evidence** | **Study event rates (%)** | | **Relative effect (95% CI)** | **Anticipated absolute effects** | |
|  |  |  |  |  |  |  | **With sham stimulation** | **With Continuous auricular stimulation** |  | **Risk with sham stimulation** | **Risk difference with Continuous auricular stimulation** |
| **Abstinence/Cessation (follow up: 6-12 months)** ^a,b,c^  Outcome measurement: point prevalence 17%, continuous/sustained abstinence 33%, unclear/NR 50% studies  Biochemical validation 50% | | | | | | | | | | | |
| 570 (6 RCTs) | very serious ^d^ | not serious ^e^ | not serious ^f^ | very serious ^g^ | none ^h^ | ⨁◯◯◯  VERY LOW | 16/284 (5.6%) | 29/286 (10.1%) | RR 1.47 (0.79 to 2.74) | 56 per 1,000 | **26 more per 1,000** (from 12 fewer to 98 more) |

**CI:** Confidence interval; **NR:** Not reported; **RR:** Risk ratio

#### Explanations

a. Population eligibility criterion was smokers wishing to quit. However, motivation or interest in quitting was not specified in review evidence tables for any of the trials included in this analysis. All studies recruited general smokers.
b. Intervention: Four studies used indwelling needles and remainder used continuous acupressure. Two studies provided a behavioural co-intervention.
c. Sham: One trial, representing 5% of the evidence, used potentially active acupuncture points for the sham arm. Two studies provided a behavioural co-intervention.
d. 64% of evidence at high risk for biochemical validation with a subset (37%) also at unclear risk for selection and attrition bias. We downrate this domain by -1.5 but also reflect the partial downrating for publication bias here.
e. Point estimates vary but confidence intervals mostly overlap (I2=22%, p=0.27). We do not downrate this domain.
f. 5% of evidence is indirect (i.e., workplace setting). We do not downrate this domain.
g. Confidence interval encompasses both harm (small but important) and benefit (large). The optimal information size not met (total of 45 events) and inadequate sample size (<2000 participants). We downrate this domain by -2.0.
h. Search not completely comprehensive but all studies report negative findings. However, authors detected funnel plot asymmetry for long-term follow-up despite variation of effects across studies. We downrate this domain by -0.5 but reflect this in the risk of bias domain.

| Summary of findings: | | | | | | |
| --- | --- | --- | --- | --- | --- | --- |
| **Continuous auricular stimulation compared to sham stimulation in smokers motivated/wishing to quit** | | | | | | |
| **Patient or population**: Smokers motivated/wishing to quit  **Setting**: No restriction  **Intervention**: Continuous auricular stimulation  **Comparison**: Sham stimulation | | | | | | |
| Outcomes | **Anticipated absolute effects^*^** (95% CI) | | Relative effect (95% CI) | № of participants  (studies) | Certainty of the evidence (GRADE) | Comments |
|  | **Risk with sham stimulation** | **Risk with Continuous auricular stimulation** |  |  |  |  |
| Abstinence/Cessation ^a,b,c^  Outcome measurement: point prevalence 17%, continuous/sustained abstinence 33%, unclear/NR 50% studies.  Biochemical validation 50%  Follow up: 6-12 months | 56 per 1,000 | **83 per 1,000** (45 to 154) | RR 1.47 (0.79 to 2.74) | 570 (6 RCTs) | ⨁◯◯◯  VERY LOW ^d,e,f,g,h^ | Fixed effects.  AMSTAR-2: Low.  Date of last search: September/October 2016.  Not GRADEd by review authors. |
| ***The risk in the intervention group** (and its 95% confidence interval) is based on the assumed risk in the comparison group and the **relative effect** of the intervention (and its 95% CI).   **CI:** Confidence interval; **NR:** Not reported; **RR:** Risk ratio | | | | | | |
| **GRADE Working Group grades of evidence** **High certainty:** We are very confident that the true effect lies close to that of the estimate of the effect **Moderate certainty:** We are moderately confident in the effect estimate: The true effect is likely to be close to the estimate of the effect, but there is a possibility that it is substantially different **Low certainty:** Our confidence in the effect estimate is limited: The true effect may be substantially different from the estimate of the effect **Very low certainty:** We have very little confidence in the effect estimate: The true effect is likely to be substantially different from the estimate of effect | | | | | | |

#### Explanations

a. Population eligibility criterion was smokers wishing to quit. However, motivation or interest in quitting was not specified in review evidence tables for any of the trials included in this analysis. All studies recruited general smokers.
b. Intervention: Four studies used indwelling needles and remainder used continuous acupressure. Two studies provided a behavioural co-intervention.
c. Sham: One trial, representing 5% of the evidence, used potentially active acupuncture points for the sham arm. Two studies provided a behavioural co-intervention.
d. 64% of evidence at high risk for biochemical validation with a subset (37%) also at unclear risk for selection and attrition bias. We downrate this domain by -1.5 but also reflect the partial downrating for publication bias here.
e. Point estimates vary but confidence intervals mostly overlap (I2=22%, p=0.27). We do not downrate this domain.
f. 5% of evidence is indirect (i.e., workplace setting). We do not downrate this domain.
g. Confidence interval encompasses both harm (small but important) and benefit (large). The optimal information size not met (total of 45 events) and inadequate sample size (<2000 participants). We downrate this domain by -2.0.
h. Search not completely comprehensive but all studies report negative findings. However, authors detected funnel plot asymmetry for long-term follow-up despite variation of effects across studies. We downrate this domain by -0.5 but reflect this in the risk of bias domain.

## Appendix K Table 67. Laser therapy versus Sham: Smoking cessation in smokers motivated/wishing to quit

| Laser therapy compared to sham laser in smokers motivated/wishing to quit  **Bibliography:** White 2014; Date of last search: September/October 2016 | | | | | | | | | | | |
| --- | --- | --- | --- | --- | --- | --- | --- | --- | --- | --- | --- |
| **Certainty assessment** | | | | | | | **Summary of findings** | | | | |
| **№ of participants (studies) Follow-up** | **Risk of bias** | **Inconsistency** | **Indirectness** | **Imprecision** | **Publication bias** | **Overall certainty of evidence** | **Study event rates (%)** | | **Relative effect (95% CI)** | **Anticipated absolute effects** | |
|  |  |  |  |  |  |  | **With sham laser** | **With Laser therapy** |  | **Risk with sham laser** | **Risk difference with Laser therapy** |
| **Abstinence/Cessation (follow up: 6-12 months)** ^a,b,c^  Outcome measurement: unclear/NR 100% studies  Biochemical validation 0% | | | | | | | | | | | |
| 613 (2 RCTs) | serious ^d^ | very serious ^e^ | not serious ^f^ | very serious ^g^ | none ^h^ | ⨁◯◯◯  VERY LOW | Studies were heterogenous and could not be quantitatively synthesized (I2=97%). Review authors report that heterogeneity could be due to populations recruited and dose of laser administered.  Results from one study were null-inclusive (RR 1.03, 95% CI: 0.55, 1.94; laser vs sham 10.3% vs 10%; RD 3 more per 1,000, 95% CI 45 fewer to 94 more). The other study reported results favouring the intervention (RR 14.18, 95% CI: 5.92, 33.94; laser vs sham 55.4% vs 3.9%, RD 515 more per 1,000, 95% CI 192 to 1,000 more). | | | | |

**CI:** Confidence interval

#### Explanations

a. Population eligibility criterion was smokers wishing to quit. However, motivation or interest in quitting was specified in review evidence tables for only one of the trials included in this analysis. One study recruited participants from a socioeconomically deprived area and the other recruited general smokers motivated to quit.
b. Laser therapy: Only one study reported the dose of laser used which was 50mW for 14 minutes. One study provided a behavioural co-intervention.
c. Sham laser: One study provided a behavioural co-intervention.
d. Both studies with substantive bias issues; one study at unclear risk for three domains (selection, attrition, biochemical validation) and the other study at high risk for those three domains. We downrate by -1.5.
e. Effect estimates vary, and confidence intervals do not overlap (I2=97%). We downrate by -2.0.
f. No indirectness.
g. Confidence intervals encompass small to moderate harm in one study and large benefit in the other study. We downrate this domain by -2.0.
h. Search strategy is not completely comprehensive but mix of results across studies. We do not downrate this domain.

| Summary of findings: | | | | | | |
| --- | --- | --- | --- | --- | --- | --- |
| **Laser therapy compared to sham laser in smokers motivated/wishing to quit** | | | | | | |
| **Patient or population**: Smokers motivated/wishing to quit  **Setting**: No restriction  **Intervention**: Laser therapy  **Comparison**: Sham laser | | | | | | |
| Outcomes | **Anticipated absolute effects^*^** (95% CI) | | Relative effect (95% CI) | № of participants  (studies) | Certainty of the evidence (GRADE) | Comments |
|  | **Risk with sham laser** | **Risk with Laser therapy** |  |  |  |  |
| Abstinence/Cessation ^a,b,c^  Outcome measurement: unclear/NR 100% studies  Biochemical validation 0%  Follow up: 6-12 months | Studies were heterogenous and could not be quantitatively synthesized (I2=97%). Review authors report that heterogeneity could be due to populations recruited and dose of laser administered. Results from one study were null-inclusive (RR 1.03, 95% CI: 0.55, 1.94). The other study reported results favouring the intervention (RR 14.18, 95% CI: 5.92, 33.94). | |  | 613 (2 RCTs) | ⨁◯◯◯  VERY LOW ^d,e,f,g,h^ | AMSTAR-2: Low.  Date of last search: September/October 2016.  Not GRADEd by review authors. |
| ***The risk in the intervention group** (and its 95% confidence interval) is based on the assumed risk in the comparison group and the **relative effect** of the intervention (and its 95% CI).   **CI:** Confidence interval; **NR:** Not reported | | | | | | |
| **GRADE Working Group grades of evidence** **High certainty:** We are very confident that the true effect lies close to that of the estimate of the effect **Moderate certainty:** We are moderately confident in the effect estimate: The true effect is likely to be close to the estimate of the effect, but there is a possibility that it is substantially different **Low certainty:** Our confidence in the effect estimate is limited: The true effect may be substantially different from the estimate of the effect **Very low certainty:** We have very little confidence in the effect estimate: The true effect is likely to be substantially different from the estimate of effect | | | | | | |

#### Explanations

a. Population eligibility criterion was smokers wishing to quit. However, motivation or interest in quitting was specified in review evidence tables for only one of the trials included in this analysis. One study recruited participants from a socioeconomically deprived area and the other recruited general smokers motivated to quit.
b. Laser therapy: Only one study reported the dose of laser used which was 50mW for 14 minutes. One study provided a behavioural co-intervention. study
c. Sham laser: One study provided a behavioural co-intervention.
d. Both studies with substantive bias issues; one study at unclear risk for three domains (selection, attrition, biochemical validation) and the other study at high risk for those three domains. We downrate by -1.5.
e. Effect estimates vary, and confidence intervals do not overlap (I2=97%). We downrate by -2.0.
f. No indirectness.
g. Confidence intervals encompass small to moderate harm in one study and large benefit in the other study. We downrate this domain by -2.0.
h. Search strategy is not completely comprehensive but mix of results across studies. We do not downrate this domain.

## Appendix K Table 68. Electrostimulation versus Sham: Smoking cessation in smokers motivated/wishing to quit

| Electrostimulation compared to sham electrostimulation in smokers motivated/wishing to quit  **Bibliography:** White 2014; Date of last search: September/October 2016 | | | | | | | | | | | |
| --- | --- | --- | --- | --- | --- | --- | --- | --- | --- | --- | --- |
| **Certainty assessment** | | | | | | | **Summary of findings** | | | | |
| **№ of participants (studies) Follow-up** | **Risk of bias** | **Inconsistency** | **Indirectness** | **Imprecision** | **Publication bias** | **Overall certainty of evidence** | **Study event rates (%)** | | **Relative effect (95% CI)** | **Anticipated absolute effects** | |
|  |  |  |  |  |  |  | **With sham electrostimulation** | **With Electrostimulation** |  | **Risk with sham electrostimulation** | **Risk difference with Electrostimulation** |
| **Abstinence/Cessation (follow up: 6-12 months)** ^a,b,c^  Outcome measurement: unclear/NR 100% studies  Biochemical validation 50% | | | | | | | | | | | |
| 405 (2 RCTs) | very serious ^d^ | not serious ^e^ | not serious ^f^ | very serious ^g^ | none ^h^ | ⨁◯◯◯  VERY LOW | 38/146 (26.0%) | 55/259 (21.2%) | RR 0.87 (0.61 to 1.23) | 260 per 1,000 | **34 fewer per 1,000** (from 102 fewer to 60 more) |

**CI:** Confidence interval; **NR:** Not reported; **RR:** Risk ratio

#### Explanations

a. Population eligibility criterion was smokers wishing to quit. However, motivation or interest in quitting was not clearly specified in review evidence tables for either study. Both appear to recruit general smokers.
b. Intervention: Electrostimulation administered through surface electrodes over the mastoid bone in one study and to the ear in the other. No co-interventions provided in both studies.
c. Control: No co-interventions provided to control arms in either study.
d. 79% of the evidence at high risk of bias for attrition and at unclear risk for all other domains (selection, performance/detection, and biochemical validation). Remaining evidence at unclear risk for two domains (selection and attrition bias). We downrate this domain by -2.0.
e. Effect estimates vary but some overlap in confidence intervals (I2=46%, p=0.17). We downrate this domain by -0.5.
f. No indirectness.
g. Confidence interval encompasses both harm (large) and benefit (moderate). The optimal information size not met (total of 93 events) and inadequate sample size (<2000 participants). We downrate this domain by -2.0.
h. Search not completely comprehensive but both studies report negative findings. We do not downrate this domain.

| Summary of findings: | | | | | | |
| --- | --- | --- | --- | --- | --- | --- |
| **Electrostimulation compared to sham electrostimulation in smokers motivated/wishing to quit** | | | | | | |
| **Patient or population**: Smokers motivated/wishing to quit  **Setting**: No restriction  **Intervention**: Electrostimulation  **Comparison**: Sham electrostimulation | | | | | | |
| Outcomes | **Anticipated absolute effects^*^** (95% CI) | | Relative effect (95% CI) | № of participants  (studies) | Certainty of the evidence (GRADE) | Comments |
|  | **Risk with sham electrostimulation** | **Risk with Electrostimulation** |  |  |  |  |
| Abstinence/Cessation ^a,b,c^  Outcome measurement: unclear/NR 100% studies  Biochemical validation: 50%  Follow up: 6-12 months | 260 per 1,000 | **226 per 1,000** (159 to 320) | RR 0.87 (0.61 to 1.23) | 405 (2 RCTs) | ⨁◯◯◯  VERY LOW ^d,e,f,g,h^ | Fixed effects.  AMSTAR-2: Low.  Date of last search: September/October 2016.  Not GRADEd by review authors. |
| ***The risk in the intervention group** (and its 95% confidence interval) is based on the assumed risk in the comparison group and the **relative effect** of the intervention (and its 95% CI).   **CI:** Confidence interval; **NR:** Not reported; **RR:** Risk ratio | | | | | | |
| **GRADE Working Group grades of evidence** **High certainty:** We are very confident that the true effect lies close to that of the estimate of the effect **Moderate certainty:** We are moderately confident in the effect estimate: The true effect is likely to be close to the estimate of the effect, but there is a possibility that it is substantially different **Low certainty:** Our confidence in the effect estimate is limited: The true effect may be substantially different from the estimate of the effect **Very low certainty:** We have very little confidence in the effect estimate: The true effect is likely to be substantially different from the estimate of effect | | | | | | |

#### Explanations

a. Population eligibility criterion was smokers wishing to quit. However, motivation or interest in quitting was not clearly specified in review evidence tables for either study. Both appear to recruit general smokers.
b. Intervention: Electrostimulation administered through surface electrodes over the mastoid bone in one study and to the ear in the other. No co-interventions provided in both studies.
c. Control: No co-interventions provided to control arms in either study.
d. 79% of the evidence at high risk of bias for attrition and at unclear risk for all other domains (selection, performance/detection, and biochemical validation). Remaining evidence at unclear risk for two domains (selection and attrition bias). We downrate this domain by -2.0.
e. Effect estimates vary but some overlap in confidence intervals (I2=46%, p=0.17). We downrate this domain by -0.5.
f. No indirectness.
g. Confidence interval encompasses both harm (large) and benefit (moderate). The optimal information size not met (total of 93 events) and inadequate sample size (<2000 participants). We downrate this domain by -2.0.
h. Search not completely comprehensive but both studies report negative findings. We do not downrate this domain.

# Whittaker 2019 {1803}

## Appendix K Table 69. Mobile phone-based interventions versus Usual care: Smoking cessation in smokers motivated/wishing to quit

| Mobile phone-based interventions compared to usual care in smokers motivated/wishing to quit  **Bibliography:** Whittaker 2019; Date of last search: October 2018 | | | | | | | | | | | |
| --- | --- | --- | --- | --- | --- | --- | --- | --- | --- | --- | --- |
| **Certainty assessment** | | | | | | | **Summary of findings** | | | | |
| **№ of participants (studies) Follow-up** | **Risk of bias** | **Inconsistency** | **Indirectness** | **Imprecision** | **Publication bias** | **Overall certainty of evidence** | **Study event rates (%)** | | **Relative effect (95% CI)** | **Anticipated absolute effects** | |
|  |  |  |  |  |  |  | **With usual care** | **With Mobile phone-based interventions** |  | **Risk with usual care** | **Risk difference with Mobile phone-based interventions** |
| **Abstinence/Cessation (follow up: 6+ months)** ^a,b,c^  Outcome measurement: point prevalence 58%, continuous/sustained abstinence 41% studies  Biochemical validation 50% studies | | | | | | | | | | | |
| 11885 (12 RCTs) ^d^ | serious ^e^ | serious ^f^ | not serious ^g^ | not serious ^h^ | none ^i^ | ⨁⨁◯◯  LOW | 320/5760 (5.6%) | 567/6125 (9.3%) | RR 1.67 (1.46 to 1.90) | 56 per 1,000 | **37 more per 1,000** (from 26 more to 50 more) |

**CI:** Confidence interval; **RR:** Risk ratio

#### Explanations

a. 75% of trials examined general smokers who were motivated/interested in quitting, 17% recruited HIV-positive smokers interested in quitting, and 8% recruited general smokers where motivation/interest in quitting was not specified.
b. Intervention: Nearly all studies had text messaging (SMS) as the main component of the intervention; however, one trial differed in that participants received mobile phone-based counselling (CBT and motivational). In-person visits or assessments were provided in addition to SMS in five studies. Five studies provided additional behavioural co-interventions (e.g., booklets) and of these, three studies also offered or made pharmacotherapy available to participants as part of usual care. 4% of intervention participants in this analysis received specialized behavioural counselling (CBT).
c. Usual care: Comparator is described as ‘usual care’ by review authors in the discussion section of the report. Various control conditions across trials including no intervention, text messages, written/internet untailored materials, untailored messages, and standard cessation advice and treatment. One trial provided a behavioral co-intervention.
d. One study was a cluster randomized trial.
e. 100% of evidence at high risk of bias for at least one domain, mainly performance bias but also attrition and biochemical validation; a subset of this (35%) is at high risk for two or more domains. We downrate this domain by -1.0.
f. Confidence intervals mostly overlap but point estimates vary and I2= 59%, p=0.005. As reported by authors, sensitivity analyses did not account for the heterogeneity. We downrate this domain by -1.0.
g. 11% of evidence is indirect (medical specialist setting, schools). Due to reporting, extent of indirectness could not be determined for five trials which recruited participants aged ≥16 years and/or the mean age of participants was 18 years; these studies did not report the proportion of adolescents included. We do not downrate this domain.
h. Confidence interval encompasses one range of effect (small but important benefit to moderate benefit). The optimal information size is met (total of 887 events) and adequate sample size. We do not downrate this domain.
i. i. Search was comprehensive, and trials reported a mix of positive and negative findings. We do not downrate this domain.

| Summary of findings: | | | | | | |
| --- | --- | --- | --- | --- | --- | --- |
| **Mobile phone-based interventions compared to usual care in smokers motivated/wishing to quit** | | | | | | |
| **Patient or population**: Smokers motivated/wishing to quit  **Setting**: No restriction  **Intervention**: Mobile phone-based interventions  **Comparison**: Usual care | | | | | | |
| Outcomes | **Anticipated absolute effects^*^** (95% CI) | | Relative effect (95% CI) | № of participants  (studies) | Certainty of the evidence (GRADE) | Comments |
|  | **Risk with usual care** | **Risk with Mobile phone-based interventions** |  |  |  |  |
| Abstinence/Cessation ^a,b,c^  Outcome measurement: point prevalence 58%, continuous/sustained abstinence 41% studies  Biochemical validation 50% studies  Follow up: 6+ months | 56 per 1,000 | **93 per 1,000** (81 to 106) | RR 1.67 (1.46 to 1.90) | 11885 (12 RCTs) ^d^ | ⨁⨁◯◯  LOW ^e,f,g,h,i^ | Fixed effects analysis.  Post-hoc random effects analysis: RR 1.42 (95% CI: 1.11, 1.83).  Sensitivity analysis removing trials with more active control conditions (i.e., standard cessation advice and treatment) did not change results appreciably (RR 1.66, 95%CI: 1.45, 1.91; I2=66%; 10 studies, 11,176 participants).  AMSTAR-2: Critically low.  Date of last search: October 2018.  Review authors rated certainty as moderate. ^j^ |
| ***The risk in the intervention group** (and its 95% confidence interval) is based on the assumed risk in the comparison group and the **relative effect** of the intervention (and its 95% CI).   **CI:** Confidence interval; **RR:** Risk ratio | | | | | | |
| **GRADE Working Group grades of evidence** **High certainty:** We are very confident that the true effect lies close to that of the estimate of the effect **Moderate certainty:** We are moderately confident in the effect estimate: The true effect is likely to be close to the estimate of the effect, but there is a possibility that it is substantially different **Low certainty:** Our confidence in the effect estimate is limited: The true effect may be substantially different from the estimate of the effect **Very low certainty:** We have very little confidence in the effect estimate: The true effect is likely to be substantially different from the estimate of effect | | | | | | |

#### Explanations

a. 75% of trials examined general smokers who were motivated/interested in quitting, 17% recruited HIV-positive smokers interested in quitting, and 8% recruited general smokers where motivation/interest in quitting was not specified.
b. Intervention: Nearly all studies had text messaging (SMS) as the main component of the intervention; however, one trial differed in that participants received mobile phone-based counselling (CBT and motivational). In-person visits or assessments were provided in addition to SMS in five studies. Five studies provided additional behavioural co-interventions (e.g., booklets) and of these, three studies also offered or made pharmacotherapy available to participants as part of usual care. 4% of intervention participants in this analysis received specialized behavioural counselling (CBT).
c. Usual care: Comparator is described as ‘usual care’ by review authors in the discussion section of the report. Various control conditions across trials including no intervention, text messages, written/internet untailored materials, untailored messages, and standard cessation advice and treatment. One trial provided a behavioral co-intervention.
d. One study was a cluster randomized trial.
e. 100% of evidence at high risk of bias for at least one domain, mainly performance bias but also attrition and biochemical validation; a subset of this (35%) is at high risk for two or more domains. We downrate this domain by -1.0.
f. Confidence intervals mostly overlap but point estimates vary and I2= 59%, p=0.005. As reported by authors, sensitivity analyses did not account for the heterogeneity. We downrate this domain by -1.0.
g. 11% of evidence is indirect (medical specialist setting, schools). Due to reporting, extent of indirectness could not be determined for five trials which recruited participants aged ≥16 years and/or the mean age of participants was 18 years; these studies did not report the proportion of adolescents included. We do not downrate this domain.
h. Confidence interval encompasses one range of effect (small but important benefit to moderate benefit). The optimal information size is met (total of 887 events) and adequate sample size. We do not downrate this domain.
i. Search was comprehensive, and trials reported a mix of positive and negative findings. We do not downrate this domain.
j. j. Review authors rate certainty of evidence as moderate (downrated inconsistency domain due to heterogeneity; sensitivity analyses did not account for majority of heterogeneity).
