# Supplementary material for: Effectiveness of smoking cessation interventions among adults: an overview of systematic reviews
Source: Syst Rev. 2024 Jul 12;13:179. doi: 10.1186/s13643-024-02570-9 (PMC11242003; doi:10.1186/s13643-024-02570-9)
Supplement: Supplementary file 13 — Additional file 13. Subgroup data for included analyses. [file 13643_2024_2570_MOESM13_ESM.docx]

**Additional file 13. Sub-group data for included analyses**

Table of Contents

[Farley 2012 {1469} 2](#_Toc34436150)

[Lindson-Hawley 2016 {671} 5](#_Toc34436151)

[Tsoi 2013 {1698} 6](#_Toc34436152)

[Van der Meer 2013 {1223} 7](#_Toc34436153)

[Livingstone-Banks 2019 {1077} 8](#_Toc34436154)

[Stead 2013 {1998} 9](#_Toc34436155)

[Whittaker 2016 {1803} 11](#_Toc34436156)

[White 2014 {1618} 12](#_Toc34436157)

[Stead 2016 {1356} 13](#_Toc34436158)

#

# Farley 2012 {1469}

| Review | Subgroup analysis | Subgroup | Outcome. | Outcome definition | Timepoint of follow-up | Number of studies in subgroup | Comparison | Intervention arm - number of events | Intervention arm - total sample size | Comparator arm - number of events | Comparator arm - total sample size | Type of model | Pooled risk estimate | Heterogeneity statistic: Chi-square value; p-value | Heterogeneity statistic: I-square value (%) | Subgroup GRADEd by review authors |
| --- | --- | --- | --- | --- | --- | --- | --- | --- | --- | --- | --- | --- | --- | --- | --- | --- |
| Farley 2012 {1469} | By NRT type (end of treatment f/u) | NRT Gum | Weight gain | Mean (SD) change in body weight (kg) from baseline to follow up in abstainers only | Other (e.g., '9 months'; 'At end of pregnancy', 'At longest postpartum follow-up',etc.) | 4 | Any type of NRT vs Placebo | N/A - continuous outcome | 254 | N/A - continuous outcome | 91 | Fixed effects | Mean Difference: -0.58 (95% CI: -1.02, -0.13) | 0.57; p=0.90 | 0% | No |
| Farley 2012 {1469} | By NRT type (end of treatment f/u) | NRT Patch | Weight gain | Mean (SD) change in body weight (kg) from baseline to follow up in abstainers only | Other (e.g., '9 months'; 'At end of pregnancy', 'At longest postpartum follow-up',etc.) | 10 | Any type of NRT vs Placebo | N/A - continuous outcome | 1244 | N/A - continuous outcome | 375 | Fixed effects | Mean Difference: -0.82 (95% CI: -1.06, -0.58) | 92.55; p<0.00001 | 90% | No |
| Farley 2012 {1469} | By NRT type (end of treatment f/u) | NRT Inhaler | Weight gain | Mean (SD) change in body weight (kg) from baseline to follow up in abstainers only | Other (e.g., '9 months'; 'At end of pregnancy', 'At longest postpartum follow-up',etc.) | 2 | Any type of NRT vs Placebo | N/A - continuous outcome | 71 | N/A - continuous outcome | 40 | Fixed effects | Mean Difference: -0.37 (95% CI: -1.19, 0.45) | 0.13; p=0.72 | 0% | No |
| Farley 2012 {1469} | By NRT type (end of treatment f/u) | NRT Sub-lingual tablet | Weight gain | Mean (SD) change in body weight (kg) from baseline to follow up in abstainers only | Other (e.g., '9 months'; 'At end of pregnancy', 'At longest postpartum follow-up',etc.) | 2 | Any type of NRT vs Placebo | N/A - continuous outcome | 316 | N/A - continuous outcome | 162 | Fixed effects | Mean Difference: -0.48 (95% CI: -0.99, 0.03) | 1.43; p=0.23 | 30% | No |
| Farley 2012 {1469} | By NRT type (end of treatment f/u) | NRT Intranasal spray | Weight gain | Mean (SD) change in body weight (kg) from baseline to follow up in abstainers only | Other (e.g., '9 months'; 'At end of pregnancy', 'At longest postpartum follow-up',etc.) | 1 | Any type of NRT vs Placebo | N/A - continuous outcome | 29 | N/A - continuous outcome | 18 | Fixed effects | Mean Difference: 0.90 (95% CI: -1.54, 3.34) | N/A - single study included in subgroup | N/A - single study included in subgroup | No |
| Farley 2012 {1469} | By NRT type (6 month f/u) | NRT Patch | Weight gain | Mean (SD) change in body weight (kg) from baseline to follow up in abstainers only | 6 months (26 weeks) | 4 | Any type of NRT vs Placebo | N/A - continuous outcome | 174 | N/A - continuous outcome | 108 | Fixed effects | Mean Difference: -0.31 (95% CI: -1.09, 0.47) | 3.23; p=0.36 | 7% | No |
| Farley 2012 {1469} | By NRT type (6 month f/u) | NRT Inhaler | Weight gain | Mean (SD) change in body weight (kg) from baseline to follow up in abstainers only | 6 months (26 weeks) | 1 | Any type of NRT vs Placebo | N/A - continuous outcome | 35 | N/A - continuous outcome | 22 | Fixed effects | Mean Difference: -0.60 (95% CI: -1.98, 0.78) | N/A - single study included in subgroup | N/A - single study included in subgroup | No |
| Farley 2012 {1469} | By NRT type (6 month f/u) | NRT Sub-lingual tablet | Weight gain | Mean (SD) change in body weight (kg) from baseline to follow up in abstainers only | 6 months (26 weeks) | 2 | Any type of NRT vs Placebo | N/A - continuous outcome | 217 | N/A - continuous outcome | 112 | Fixed effects | Mean Difference: -0.19 (95% CI: -1.09, 0.72) | 0.06; p=0.80 | 0% | No |
| Farley 2012 {1469} | By NRT type (6 month f/u) | NRT Gum | Weight gain | Mean (SD) change in body weight (kg) from baseline to follow up in abstainers only | 6 months (26 weeks) | 2 | Any type of NRT vs Placebo | N/A - continuous outcome | 70 | N/A - continuous outcome | 33 | Fixed effects | Mean Difference: -0.83 (95% CI: -2.35, 0.69) | 0.51; p=0.48 | 0% | No |
| Farley 2012 {1469} | By NRT type (12 month f/u) | NRT Gum | Weight gain | Mean (SD) change in body weight (kg) from baseline to follow up in abstainers only | 12 months (52 weeks) | 1 | Any type of NRT vs Placebo | N/A - continuous outcome | 34 | N/A - continuous outcome | 15 | Fixed effects | Mean Difference: -0.07 (95% CI: -3.07, 2.93) | N/A - single study in subgroup | N/A - single study in subgroup | No |
| Farley 2012 {1469} | By NRT type (12 month f/u) | NRT Patch | Weight gain | Mean (SD) change in body weight (kg) from baseline to follow up in abstainers only | 12 months (52 weeks) | 6 | Any type of NRT vs Placebo | N/A - continuous outcome | 607 | N/A - continuous outcome | 163 | Fixed effects | Mean Difference: -0.23 (95% CI: -0.90, 0.45) | 1.00; p=0.96 | 0% | No |
| Farley 2012 {1469} | By NRT type (12 month f/u) | NRT Inhaler | Weight gain | Mean (SD) change in body weight (kg) from baseline to follow up in abstainers only | 12 months (52 weeks) | 2 | Any type of NRT vs Placebo | N/A - continuous outcome | 59 | N/A - continuous outcome | 31 | Fixed effects | Mean Difference: -1.03 (95% CI: -2.23, 0.17) | 0.06; p=0.80 | 0% | No |
| Farley 2012 {1469} | By NRT type (12 month f/u) | NRT Sub-lingual tablet | Weight gain | Mean (SD) change in body weight (kg) from baseline to follow up in abstainers only | 12 months (52 weeks) | 3 | Any type of NRT vs Placebo | N/A - continuous outcome | 194 | N/A - continuous outcome | 109 | Fixed effects | Mean Difference: 0.27 (95% CI: -0.99, 1.54) | 1.21; p=0.55 | 0% | No |
| Farley 2012 {1469} | By NRT type (12 month f/u) | NRT Intranasal spray | Weight gain | Mean (SD) change in body weight (kg) from baseline to follow up in abstainers only | 12 months (52 weeks) | 3 | Any type of NRT vs Placebo | N/A - continuous outcome | 76 | N/A - continuous outcome | 46 | Fixed effects | Mean Difference: -1.55 (95% CI: -3.09, 0.00) | 1.87; p=0.39 | 0% | No |

# Lindson-Hawley 2016 {671}

| Review | Subgroup analysis | Subgroup | Outcome. | Timepoint of follow-up | Comparison | Number of studies in subgroup | Intervention arm - number of events | Intervention arm - total sample size | Comparator arm - number of events | Comparator arm - total sample size | Type of model | Pooled risk estimate | Heterogeneity statistic: Chi-square value; p-value | Heterogeneity statistic: I-square value (%) | Subgroup GRADEd by review authors |
| --- | --- | --- | --- | --- | --- | --- | --- | --- | --- | --- | --- | --- | --- | --- | --- |
| Lindson-Hawley 2016 {671} | By type of NRT | Nicotine gum | Tobacco smoking reduction | 12+ months (52+ weeks) | Nicotine gum versus placebo | 4 | 58 | 704 | 23 | 700 | Fixed effects | Risk ratio: 2.50 (95% CI: 1.57, 4.00) | 4.53; P = 0.21 | 34% | No |
| Lindson-Hawley 2016 {671} | By type of NRT | Nicotine inhaler | Tobacco smoking reduction | 12+ months (52+ weeks) | Nicotine inhaler versus placebo | 2 | 34 | 415 | 10 | 414 | Fixed effects | Risk ratio: 3.39 (95% CI: 1.70, 6.77) | 0.05; P = 0.82 | 0.0% | No |
| Lindson-Hawley 2016 {671} | By type of NRT | Choice of NRT | Tobacco smoking reduction | 12+ months (52+ weeks) | Choice of NRT type versus placebo | 2 | 158 | 474 | 87 | 374 | Fixed effects | Risk ratio: 1.40 (95% CI: 1.11, 1.75) | 0.06; P = 0.80 | 0.0% | No |
| Lindson-Hawley 2016 {671} | By type of NRT | Choice of NRT | Tobacco smoking abstinence/cessation | 12+ months (52+ weeks) | Choice of NRT type versus placebo | 2 | 70 | 474 | 34 | 374 | Fixed effects | Risk ratio: 1.56 (95% CI: 1.04, 2.33) | 1.62; P = 0.20 | 38% | No |
| Lindson-Hawley 2016 {671} | By type of NRT | Nicotine gum | Tobacco smoking abstinence/cessation | 12+ months (52+ weeks) | Nicotine gum versus placebo | 4 | 51 | 704 | 21 | 700 | Fixed effects | Risk ratio: 2.38 (95% CI: 1.46, 3.89) | 1.58; P = 0.66 | 0% | No |
| Lindson-Hawley 2016 {671} | By type of NRT | Nicotine inhaler | Tobacco smoking abstinence/cessation | 12+ months (52+ weeks) | Nicotine inhaler versus placebo | 2 | 38 | 415 | 20 | 414 | Fixed effects | Risk ratio: 1.90 (95% CI: 1.13, 3.20) | 5.01; P = 0.03 | 80% | No |

# Tsoi 2013 {1698}

| Review | Subgroup analysis | Subgroup | Outcome | Timepoint of follow-up. | Comparison | Number of studies in subgroup | Intervention arm - number of events | Intervention arm - total sample size | Comparator arm - number of events | Comparator arm - total sample size | Type of model | Pooled risk estimate | Heterogeneity statistic: Chi-square value; p-value | Heterogeneity statistic: I-square value (%) | Subgroup GRADEd by review authors |
| --- | --- | --- | --- | --- | --- | --- | --- | --- | --- | --- | --- | --- | --- | --- | --- |
| Tsoi 2013 {1698} | By NRT co-intervention in bupropion trials | No NRT co-intervention | Tobacco smoking abstinence/cessation | 6 months (26 weeks) | Bupropion vs Placebo | 3 | 5 | 51 | 2 | 53 | Random effects | Risk ratio: 2.19 (95% CI: 0.50, 9.63) | 0.34; p=0.85 | 0% | No |
| Tsoi 2013 {1698} | By NRT co-intervention in bupropion trials | NRT co-intervention | Tobacco smoking abstinence/cessation | 6 months (26 weeks) | Bupropion + NRT patch vs Placebo + NRT patch | 2 | 9 | 55 | 2 | 55 | Random effects | Risk ratio: 3.41 (95% CI: 0.87, 13.30) | 0.56; p=0.46 | 0% | No |

# Van der Meer 2013 {1223}

| Review | Subgroup analysis | Subgroup | Outcome. | Timepoint of follow-up | Comparison | Number of studies in subgroup | Intervention arm - number of events | Intervention arm - total sample size | Comparator arm - number of events | Comparator arm - total sample size | Type of model | Pooled risk estimate | Heterogeneity statistic: Chi-square value; p-value | Heterogeneity statistic: I-square value (%) | Subgroup GRADEd by review authors |
| --- | --- | --- | --- | --- | --- | --- | --- | --- | --- | --- | --- | --- | --- | --- | --- |
| van der Meer 2013 {1223} | By additional pharmacotherapy | Bupropion alone (no additional pharmacotherapy) | Tobacco smoking abstinence/cessation | 6-12 months | Bupropion vs Placebo (current depression) | 4 | 27 | 176 | 21 | 179 | Fixed effects | Risk ratio: 1.32 (95% CI: 0.78, 2.24) | 5.37; p=0.15 | 44% | No |
| van der Meer 2013 {1223} | By additional pharmacotherapy | Bupropion plus NRT | Tobacco smoking abstinence/cessation | 6 months | Bupropion plus NRT vs Placebo plus NRT (current depression) | 1 | 4 | 28 | 2 | 27 | Fixed effects | Risk ratio: 1.93 (95% CI: 0.38, 9.68) | N/A - single study | N/A - single study | No |
| van der Meer 2013 {1223} | By additional pharmacotherapy | Bupropion alone (no additional pharmacotherapy) | Tobacco smoking abstinence/cessation | 6-12 months | Bupropion vs Placebo (past depression) | 4 | 40 | 166 | 21 | 151 | Fixed effects | Risk ratio: 1.57 (95% CI: 0.96, 2.58) | 2.59; p=0.46 | 0% | No |
| van der Meer 2013 {1223} | By additional pharmacotherapy | Bupropion plus NRT | Tobacco smoking abstinence/cessation | 12 months | Bupropion plus NRT vs Placebo plus NRT (past depression) | 1 | 16 | 43 | 3 | 44 | Fixed effects | Risk ratio: 5.46 (95% CI: 1.71, 17.40) | N/A - single study | N/A - single study | No |

# Livingstone-Banks 2019 {1077}

| Review | Subgroup analysis | Subgroup | Outcome. | Timepoint of follow-up | Comparison | Number of studies in subgroup | Intervention arm - number of events | Intervention arm - total sample size | Comparator arm - number of events | Comparator arm - total sample size | Type of model | Pooled risk estimate | Heterogeneity statistic: Chi-square value; p-value | Heterogeneity statistic: I-square value (%) | Subgroup GRADEd by review authors |
| --- | --- | --- | --- | --- | --- | --- | --- | --- | --- | --- | --- | --- | --- | --- | --- |
| Livingstone-Banks 2019 {1077} | By control condition | Control group given no materials/no intervention | Tobacco smoking abstinence/cessation | 6+ months (26+ weeks) | Non-tailored print-based self-help materials given with face-to-face contact versus No materials or leaflet only | 3 | 34 | 746 | 27 | 922 | Random effects | Risk ratio: 1.35 (95% CI: 0.80, 2.26) | 1.28; P = 0.53 | 0% | No |
| Livingstone-Banks 2019 {1077} | By control condition | Control group given leaflet/pamphlet (received materials that were not specific to smoking plus a video focused on cholesterol education) | Tobacco smoking abstinence/cessation | 6 months (26 weeks) | Non-tailored print-based self-help materials given with face-to-face contact versus No materials or leaflet only | 1 | 73 | 650 | 40 | 504 | Random effects | Risk ratio: 1.42 (95% CI: 0.98, 2.04) | not applicable- single study | not applicable- single study | No |

# Stead 2013 {1998}

| Review | Subgroup analysis | Subgroup | Outcome | Timepoint of follow-up. | Comparison | Number of studies in subgroup | Intervention arm - number of events | Intervention arm - total sample size | Comparator arm - number of events | Comparator arm - total sample size | Type of model | Pooled risk estimate | Heterogeneity statistic: Chi-square value; p-value | Heterogeneity statistic: I-square value (%) | Subgroup GRADEd by review authors |
| --- | --- | --- | --- | --- | --- | --- | --- | --- | --- | --- | --- | --- | --- | --- | --- |
| Stead 2013 {1998} | By intensity of intervention (advice) | Intensive intervention | Tobacco smoking abstinence/cessation | 6+ months (26+ weeks) | Physician advice (intensive) versus No advice (or usual care) | 11 | 553 | 4670 | 246 | 3845 | Fixed effects | Risk ratio: 1.86 (95% CI 1.60, 2.15) | 20.15; P = 0.03 | 50% | No |
| Stead 2013 {1998} | By intensity of intervention (advice) | Minimal intervention | Tobacco smoking abstinence/cessation | 6+ months (26+ weeks) | Physician advice (minimal) versus No advice (or usual care) | 17 | 455 | 7913 | 216 | 5811 | Fixed effects | Risk ratio: 1.66 (95% CI 1.42, 1.94) | 23.33; P = 0.11 | 31% | No |
| Stead 2013 {1998} | By number of advice sessions | One visit | Tobacco smoking abstinence/cessation | 6+ months (26+ weeks) | Physician advice (one visit) versus No advice (or usual care) | 18 | 484 | 8199 | 284 | 6476 | Fixed effects | Risk ratio: 1.55 (95% CI: 1.35,1.79) | 25.96; p= 0.08 | 35% | No |
| Stead 2013 {1998} | By number of advice sessions | More than one visit | Tobacco smoking abstinence/cessation | 6+ months (26+ weeks) | Physician advice (more than one visit) versus No advice (or usual care) | 3 | 122 | 944 | 74 | 919 | Fixed effects | Risk ratio: 1.60 (95% CI: 1.21,2.11) | 5.78; p=0.06 | 65% | No |
| Stead 2013 {1998} | By number of advice sessions | Multiple visits | Tobacco smoking abstinence/cessation | 6+ months (26+ weeks) | Physician advice (multiple visits) versus No advice (or usual care) | 6 | 312 | 2453 | 132 | 2057 | Fixed effects | Risk ratio: 2.27 (95% CI: 1.87,2.75) | 6.80; p= 0.24 | 26% | No |
| Stead 2013 {1998} | By aids as adjuncts to advice | Aids used | Tobacco smoking abstinence/cessation | 6+ months (26+ weeks) | Physician advice with aids versus No advice (or usual care) | 10 | 357 | 3640 | 230 | 3650 | Fixed effects | Risk ratio: 1.71 (95% CI: 1.46,2.01) | 14.58; p=0.10 | 38% | No |
| Stead 2013 {1998} | By aids as adjuncts to advice | Aids not used | Tobacco smoking abstinence/cessation | 6+ months (26+ weeks) | Physician advice with no aids versus No advice (or usual care) | 17 | 570 | 8113 | 298 | 6405 | Fixed effects | Risk ratio: 1.78 (95% CI: 1.56,2.04) | 30.15; p=0.02 | 47% | No |
| Stead 2013 {1998} | By population | Unselected populations | Tobacco smoking abstinence/cessation | 6+ months (26+ weeks) | Intensive advice versus minimal advice | 10 | 405 | 3907 | 178 | 2095 | Fixed effects | Risk ratio: 1.20 (95% CI 1.02, 1.43) | 8.72; P = 0.46 | 0% | No |
| Stead 2013 {1998} | By population) | High risk populations | Tobacco smoking abstinence/cessation | 6+ months (26+ weeks) | Intensive advice versus minimal advice | 5 | 215 | 1832 | 127 | 1941 | Fixed effects | Risk ratio: 1.65 (95% CI 1.35, 2.03) | 5.05; P = 0.28 | 21% | No |

# Whittaker 2016 {1803}

(sensitivity analyses)

| Review | Sensitivity analysis | Outcome. | Timepoint of follow-up | Comparison | Number of studies | Intervention arm - number of events | Intervention arm - total sample size | Comparator arm - number of events | Comparator arm - total sample size | Type of model | Pooled risk estimate | Heterogeneity statistic: Chi-square value; p-value | Heterogeneity statistic: I-square value (%) | Subgroup GRADEd by review authors |
| --- | --- | --- | --- | --- | --- | --- | --- | --- | --- | --- | --- | --- | --- | --- |
| Whittaker 2019 {1803} | Biochemically verified 26-week abstinence | Tobacco smoking abstinence/cessation | 6 months (26 weeks) | Mobile phone-based interventions vs Usual care | 6 | 330 | 3707 | 177 | 3653 | Fixed effects | Risk ratio: 1.83 (95% CI: 1.54, 2.19) | 17.40; P = 0.004 | 71% | No |
| Whittaker 2019 {1803} | 26-week continuous abstinence | Tobacco smoking abstinence/cessation | 6 months (26 weeks) | Mobile phone-based interventions vs Usual care | 8 | 510 | 5527 | 276 | 5152 | Fixed effects | Risk ratio: 1.72 (95% CI: 1.50, 1.98) | 21.54; P = 0.003 | 68% | No |
| Whittaker 2019 {1803} | 26-week 7-day point prevalence | Tobacco smoking abstinence/cessation | 6 months (26 weeks) | Mobile phone-based interventions vs Usual care | 7 | 368 | 1948 | 307 | 1940 | Fixed effects | Risk ratio: 1.18 (95% CI: 1.03, 1.35) | 7.87; P = 0.25 | 24% | No |
| Whittaker 2019 {1803} | Text messaging-only interventions (excluding trials with in-person contact) | Tobacco smoking abstinence/cessation | 6 months (26 weeks) | Text messaging-only interventions vs Usual care | 7 | 468 | 5134 | 267 | 4753 | Fixed effects | Risk ratio: 1.69 (95% CI: 1.46, 1.95) | 22.80; P = 0.00087 | 74% | No |
| Whittaker 2019 {1803} | Text messaging plus face-to-face interventions (excluding trials without in-person contact) | Tobacco smoking abstinence/cessation | 6 months (26 weeks) | Text messaging plus face-to-face interventions vs Usual care | 5 | 89 | 988 | 59 | 1007 | Fixed effects | Risk ratio: 1.54 (95% CI: 1.12, 2.11) | 3.62; P = 0.46 | 0% | No |
| Whittaker 2019 {1803} | Minimal control conditions (excluding trials with standard cessation advice and treatment) | Tobacco smoking abstinence/cessation | 6 months (26 weeks) | Mobile phone-based interventions vs Usual care | 10 | 531 | 5772 | 299 | 5404 | Fixed effects | Risk ratio: 1.66 (95% CI: 1.45, 1.91) | NR | 66% | No |

# White 2014 {1618}

| Review | Subgroup analysis | Subgroup | Outcome. | Timepoint of follow-up | Comparison | Number of studies in subgroup | Intervention arm - number of events | Intervention arm - total sample size | Comparator arm - number of events | Comparator arm - total sample size | Type of model | Pooled risk estimate | Heterogeneity statistic: Chi-square value; p-value | Heterogeneity statistic: I-square value (%) | Subgroup GRADEd by review authors |
| --- | --- | --- | --- | --- | --- | --- | --- | --- | --- | --- | --- | --- | --- | --- | --- |
| White 2014 {1618}; | By type of continuous auricular stimulation | Continuous acupressure | Tobacco smoking abstinence/cessation | 6-12 months | Continuous auricular stimulation vs Sham stimulation | 2 | 10 | 66 | 0 | 58 | Fixed effects | Risk ratio: 9.45 (95% CI: 1.26-70.92) | 0.01; p=0.92 | 0% | No |
| White 2014 {1618}; | By type of continuous auricular stimulation | Indwelling needles | Tobacco smoking abstinence/cessation | 6-12 months | Continuous auricular stimulation vs. Sham stimulation | 4 | 19 | 220 | 16 | 226 | Fixed effects | Risk ratio: 1.20 (95% CI: 0.62-2.32) | 2.76; p=0.43 | 0% | No |

# Stead 2016 {1356}

| Review | Subgroup analysis | Subgroup | Outcome | Timepoint of follow-up. | Comparison | Number of studies in subgroup | Intervention arm - number of events | Intervention arm - total sample size | Comparator arm - number of events | Comparator arm - total sample size | Type of model | Pooled risk estimate | Heterogeneity statistic: Chi-square value; p-value | Heterogeneity statistic: I-square value (%) | Subgroup GRADEd by review authors |
| --- | --- | --- | --- | --- | --- | --- | --- | --- | --- | --- | --- | --- | --- | --- | --- |
| Stead 2016 {1356} | By setting | Lung Health Study (community) | Tobacco smoking abstinence/cessation | 6+ months (26+ weeks) | Combined pharmacotherapy and behavioural interventions for smoking cessation vs minimal intervention or usual care | 1 | 1373 | 3923 | 177 | 1964 | Fixed effects | Risk ratio: 3.88 (95% CI: 3.35, 4.50) - single study included in analysis (not a pooled risk estimate) | not applicable | not applicable | Yes |
| Stead 2016 {1356} | By setting | Recruited in health care setting | Tobacco smoking abstinence/cessation | 6+ months (26+ weeks) | Combined pharmacotherapy and behavioural interventions for smoking cessation vs minimal intervention or usual care | 43 | 1061 | 7180 | 513 | 6683 | Fixed effects | Risk ratio: 1.97 (95% CI: 1.79, 2.18) | 68.33; P = 0.01 | 39% | No |
| Stead 2016 {1356} | By setting | Recruited from community settings | Tobacco smoking abstinence/cessation | 6+ months (26+ weeks) | Combined pharmacotherapy and behavioural interventions for smoking cessation vs minimal intervention or usual care | 8 | 411 | 2389 | 283 | 2517 | Fixed effects | Risk ratio: 1.53 (95% CI: 1.33, 1.76) | 7.89; P = 0.34 | 11% | No |
| Stead 2016 {1356} | By number of sessions | Lung Health Study (over 8 sessions) | Tobacco smoking abstinence/cessation | 6+ months (26+ weeks) | Combined pharmacotherapy and behavioural interventions for smoking cessation vs minimal intervention or usual care | 1 | 1373 | 3923 | 177 | 1964 | Fixed effects | Risk ratio: 3.88 (95% CI: 3.35, 4.50) - single study included in analysis (not a pooled risk estimate | not applicable | not applicable | Yes |
| Stead 2016 {1356} | By number of sessions | 0 sessions | Tobacco smoking abstinence/cessation | 6+ months (26+ weeks) | Combined pharmacotherapy and behavioural interventions for smoking cessation vs minimal intervention or usual care | 1 | 42 | 500 | 42 | 523 | Fixed effects | Risk ratio: 1.05 (95% CI: 0.69, 1.58) - single study included in analysis (not a pooled risk estimate) | not applicable | not applicable | No |
| Stead 2016 {1356} | By number of sessions | 1-3 sessions | Tobacco smoking abstinence/cessation | 6+ months (26+ weeks) | Combined pharmacotherapy and behavioural interventions for smoking cessation vs minimal intervention or usual care | 10 | 303 | 2202 | 134 | 1830 | Fixed effects | Risk ratio: 1.94 (95% CI: 1.60, 2.36) | 5.53; P = 0.79 | 0% | No |
| Stead 2016 {1356} | By number of sessions | 4-8 sessions | Tobacco smoking abstinence/cessation | 6+ months (26+ weeks) | Combined pharmacotherapy and behavioural interventions for smoking cessation vs minimal intervention or usual care | 28 | 991 | 6174 | 547 | 5989 | Fixed effects | Risk ratio: 1.81 (95% CI: 1.64, 1.99) | 46.46; P = 0.01 | 42% | No |
| Stead 2016 {1356} | By number of sessions | Over 8 sessions | Tobacco smoking abstinence/cessation | 6+ months (26+ weeks) | Combined pharmacotherapy and behavioural interventions for smoking cessation vs minimal intervention or usual care | 13 | 193 | 1194 | 85 | 1076 | Fixed effects | Risk ratio: 2.10 (95% CI: 1.65, 2.68) | 20.75; P = 0.05 | 42% | No |
| Stead 2016 {1356} | By duration of contact | No personal contact scheduled | Tobacco smoking abstinence/cessation | 6+ months (26+ weeks) | Combined pharmacotherapy and behavioural interventions for smoking cessation vs minimal intervention or usual care | 1 | 42 | 500 | 42 | 523 | Fixed effects | Risk ratio: 1.05 (95% CI: 0.69, 1.58) - single study included in analysis (not a pooled risk estimate) | not applicable | not applicable | No |
| Stead 2016 {1356} | By duration of contact | Up to 30 minutes | Tobacco smoking abstinence/cessation | 6+ months (26+ weeks) | Combined pharmacotherapy and behavioural interventions for smoking cessation vs minimal intervention or usual care | 5 | 106 | 948 | 55 | 771 | Fixed effects | Risk ratio: 1.70 (95% CI: 1.24, 2.33) | 0.98; P = 0.91 | 0% | No |
| Stead 2016 {1356} | By duration of contact | 31-90 minutes | Tobacco smoking abstinence/cessation | 6+ months (26+ weeks) | Combined pharmacotherapy and behavioural interventions for smoking cessation vs minimal intervention or usual care | 17 | 696 | 4494 | 350 | 4224 | Fixed effects | Risk ratio: 1.96 (95% CI: 1.74, 2.21) | 35.90; P = 0.003 | 55% | No |
| Stead 2016 {1356} | By duration of contact | 91-300 minutes | Tobacco smoking abstinence/cessation | 6+ months (26+ weeks) | Combined pharmacotherapy and behavioural interventions for smoking cessation vs minimal intervention or usual care | 22 | 509 | 2923 | 268 | 2835 | Fixed effects | Risk ratio: 1.84 (95% CI: 1.60, 2.11) | 26.53; P = 0.19 | 21% | No |
| Stead 2016 {1356} | By duration of contact | Over 300 minutes | Tobacco smoking abstinence/cessation | 6+ months (26+ weeks) | Combined pharmacotherapy and behavioural interventions for smoking cessation vs minimal intervention or usual care | 7 | 176 | 1205 | 93 | 1065 | Fixed effects | Risk ratio: 1.70 (95% CI: 1.34, 2.16) | 9.93; P = 0.13 | 40% | No |
| Stead 2016 {1356} | By duration of contact | Lung Health Study (over 300 mins) | Tobacco smoking abstinence/cessation | 6+ months (26+ weeks) | Combined pharmacotherapy and behavioural interventions for smoking cessation vs minimal intervention or usual care | 1 | 1373 | 3923 | 177 | 1964 | Fixed effects | Risk ratio: 3.88 (95% CI: 3.35, 4.50) - single study included in analysis (not a pooled risk estimate | not applicable | not applicable | Yes |
| Stead 2016 {1356} | By motivation to quit | Not explicitly selected | Tobacco smoking abstinence/cessation | 6+ months (26+ weeks) | Combined pharmacotherapy and behavioural interventions for smoking cessation vs minimal intervention or usual care | 10 | 213 | 1159 | 74 | 1103 | Fixed effects | Risk ratio: 2.71 ((95% CI: 2.11, 3.49) | 23.18; P = 0.01 | 61% | No |
| Stead 2016 {1356} | By motivation to quit | Not selected | Tobacco smoking abstinence/cessation | 6+ months (26+ weeks) | Combined pharmacotherapy and behavioural interventions for smoking cessation vs minimal intervention or usual care | 20 | 672 | 5196 | 411 | 4942 | Fixed effects | Risk ratio: 1.60 (95% CI: 1.42, 1.80) | 18.90; P = 0.46 | 0% | No |
| Stead 2016 {1356} | By motivation to quit | Selected for motivation | Tobacco smoking abstinence/cessation | 6+ months (26+ weeks) | Combined pharmacotherapy and behavioural interventions for smoking cessation vs minimal intervention or usual care | 22 | 644 | 3715 | 323 | 3373 | Fixed effects | Risk ratio: 1.90 (95% CI: 1.68, 2.15) | 29.60; P = 0.10 | 29% | No |
| Stead 2016 {1356} | By motivation to quit | Lung Health Study (unselected) | Tobacco smoking abstinence/cessation | 6+ months (26+ weeks) | Combined pharmacotherapy and behavioural interventions for smoking cessation vs minimal intervention or usual care | 1 | 1373 | 3923 | 177 | 1964 | Fixed effects | Risk ratio: 3.88 (95% CI: 3.35, 4.50) - single study included in analysis (not a pooled risk estimate) | not applicable | not applicable | Yes |
