# Supplementary material for: Effectiveness of smoking cessation interventions among adults: an overview of systematic reviews
Source: Syst Rev. 2024 Jul 12;13:179. doi: 10.1186/s13643-024-02570-9 (PMC11242003; doi:10.1186/s13643-024-02570-9)
Supplement: Supplementary file 14 — Additional file 14. Tobacco effect judgements. [file 13643_2024_2570_MOESM14_ESM.docx]

# **Additional file 14.**

# Procedure

- Judgments on effect size are to be made using the **absolute difference** between intervention and control groups. GRADE discourages the use of relative effects to make these judgments, since the same relative effect could have drastically different absolute impacts depending on the baseline risk of an outcome.
- Judgments must be made for the point estimate, lower confidence interval (CI), and upper CI **separately**. The rater should judge the size of effect for each estimate, **as if that given integer represents the true effect.**
- This is done for each outcome for a given intervention/comparator and population.
- Contextualizing the judgment of effect size – Factors that may impact the judgment:
  - **Magnitude of health benefit from successful quitting.** To assess the benefit for the smoking cessation outcome, it is important to consider the downstream health benefits of successful quitting. Quitting at 30 years of age increases life expectancy by a decade while quitting at 40 and 50 years of age increases expectancy by 9 and 6 years, respectively (1). For every two individuals who quit smoking tobacco, one will avoid a tobacco-related death (2). Quitting before age 40 reduces the excess risk of premature disease and death by 90% (3). Ten years after quitting smoking, the risk of developing lung cancer is cut in half and the risk of cancers of the mouth, throat, esophagus, bladder, cervix, and pancreas are decreased (4,5). Risk for coronary heart disease decreases by half after 1 year and is at normal levels by 15 years (4). Risk of stroke is also greatly reduced after 2-4 years (4). Quitting smoking also leads to improvements in mental health, overall quality of life (4). People who quit smoking after having a heart attack reduce their risk of having a second heart attack by about half (6).

Therefore, the judgment of benefit for smoking cessation for a given intervention is in large part a product of the increased likelihood of quitting, and the large anticipated health benefit that would follow (e.g., a 10 in 1000 increased chance of quitting is a 10 in 1000 chance of obtaining the above health benefits and may therefore be considered an important benefit despite the small absolute increase in likelihood of quitting). The judgement may also be influenced by other risk factors. For example, there may be a differential benefit for those with mental health conditions (e.g., schizophrenia) given higher rates of smoking and smoking-related mortality, greater levels of nicotine dependence, and greater challenges with quitting in some of these populations (7).

- - **Harms related to smoking.** Related to the above, previous discussions with our experts have indicated that almost *any* increase in quit rate is important given how harmful it is to continue smoking. Men who smoke are 22 times more likely to get cancer, while women who smoke are at 12 times the risk (4). Tobacco smoking is a cause of cancers of the mouth and throat, lungs, liver, colon and rectum, stomach, bladder, blood, pancreas, kidneys, and cervix (8). Tobacco smoking is also a cause of metabolic disorders such as diabetes (8). The long list of conditions linked to smoking also includes blindness, cataracts, pneumonia, erectile dysfunction, reduced immune function, and overall diminished health (8). These harms are not captured in our review, but should be considered. This should also be considered in the case where an intervention may reduce the likelihood of quitting (i.e., effect is on the side of harms).
  - **Trade off of benefits versus harms/ adverse effects of the intervention.** For example, if two interventions both increase smoking quit rate by 5%, but one has more identified harms than the other, you may rate the effect sizes differently. Similarly, if two interventions have similar adverse events, but one has a great effect on benefits, you may judge the effect sizes for those adverse event outcomes differently.
  - **Importance and nature of the outcome** (see outcome rating below). For example, a 10% absolute increase in a very important outcome (e.g., death) may be considered large, whereas a 10% increase in a less important outcome (e.g., headache) may be considered trivial.
  - **Other elements of the intervention not captured under recorded harms/adverse events**. For example, the same absolute effect might be judged differently for two different interventions if one is clearly more invasive or resource intensive.

**References**

1. Jha P, Peto R. Global effects of smoking, of quitting, and of taxing tobacco. N Engl J Med. 2014 Jan 2; 370(1):60-8.
2. Lam TH. Absolute risk of tobacco deaths: one in two smokers will be killed by smoking: comment on "Smoking and all-cause mortality in older people". Arch Intern Med. 2012 Jun 11; 172(11):845-6.
3. Reid R, Pritchard G, Walker K, Aitken D, Mullen K, Pipe A. Managing smoking cessation. CMAJ. 2016 Dec;188(17-18):E484-E492.
4. Patnode CD, Henderson JT, Thompson JH, Senger CA, Fortmann SP, Whitlock EP. Behavioral Counseling and Pharmacotherapy Interventions for Tobacco Cessation in Adults, Including Pregnant Women: A Review of Reviews for the U.S. Preventive Services Task Force. 2015 Sep.
5. Word Health Organization. Tobacco Free Initiative (TFI): Fact sheet about health benefits of smoking cessation. 2018. Available at: <http://www.who.int/tobacco/quitting/benefits/en/>
6. Stead LF, Buitrago D, Preciado N, Sanchez G, Hartmann-Boyce J, Lancaster T. Physician advice for smoking cessation. Cochrane Database of Systematic Reviews 2013, Issue 5. Art. No.: CD000165.
7. Caponnetto, P., Polosa, R., Robson, D., & Bauld, L. (2020). Tobacco smoking, related harm and motivation to quit smoking in people with schizophrenia spectrum disorders. Health psychology research, 8(1), 9042.
8. U.S. Department of Health and Human Services. The Health Consequences of Smoking—50 Years of Progress: A Report of the Surgeon General. Atlanta, GA: U.S. Department of Health and Human Services, Centers for Disease Control and Prevention, National Center for Chronic Disease Prevention and Health Promotion, Office on Smoking and Health; 2014. Accessed at [www.surgeongeneral.gov/library/reports/50-years-of-progress/full-report.pdf](http://www.surgeongeneral.gov/library/reports/50-years-of-progress/full-report.pdf)

Table of Contents

[Procedure 1](#_Toc127954792)

[Outcome ratings – for reference 6](#_Toc127954793)

[Table template 6](#_Toc127954794)

[Behavioural interventions 7](#_Toc127954795)

[Physician advice 7](#_Toc127954796)

[1. Physician advice (minimal or intensive interventions)^a^ versus no advice (or usual care) in general/mixed population of smokers 7](#_Toc127954797)

[2. Physician advice with follow-up^a^ versus Minimal intervention/advice with single visit in general/mixed population of smokers 7](#_Toc127954798)

[Counselling 8](#_Toc127954799)

[3. Intensive advice^a^ versus Minimal advice in general/mixed population of smokers 8](#_Toc127954800)

[4. Stage-based individual counselling and/or advice in a general/mixed population of smokers 9](#_Toc127954801)

[5. Individual counselling^a^ versus minimal contact in general/mixed population 10](#_Toc127954802)

[6. Group therapy^a^ vs No intervention in general/mixed population of smokers 10](#_Toc127954803)

[Tailored and non-tailored print-based material 11](#_Toc127954804)

[7. Individually tailored print-based self-help materials (no face-to-face) versus No materials/no intervention in general/mixed population of smokers 11](#_Toc127954805)

[8. Non-tailored print-based self-help materials (no face-to-face contact) in general/mixed population of smokers 12](#_Toc127954806)

[9. Non-tailored print-based self-help materials^a^ (no face-to-face contact) Versus No materials/no intervention in smokers motivated/willing to quit 13](#_Toc127954807)

[10. Non-tailored print-based self-help materials^a^ (WITH face-to-face contact) versus No intervention or leaflet only in general/mixed population of smokers 13](#_Toc127954808)

[11. Stage-based expert systems or tailored self-help materials versus Assessment only in general/mixed population of smokers 14](#_Toc127954809)

[Computer and internet counselling 14](#_Toc127954810)

[12. Interactive internet/computer programs versus usual care or non-active control in general/mixed population of smokers 14](#_Toc127954811)

[Mobile phone, hotline, and telephone counselling 16](#_Toc127954812)

[13. Interactive voice response (IVR) systems^a^ versus No intervention: Smoking cessation in general/mixed population of smokers 16](#_Toc127954813)

[14. Mobile phone short message service (SMS)^a^ vs control in smokers motivated/wishing to quit 17](#_Toc127954814)

[15. Mobile phone based interventions^a^ versus usual care in smokers motivated/wishing to quit 18](#_Toc127954815)

[16. Hotline and self-help materials^a^ versus Minimal intervention in general/mixed population of smokers 19](#_Toc127954816)

[17. Telephone counselling^a^ versus Usual care telephone call in general/mixed population of smokers 19](#_Toc127954817)

[18. Intensive telephone counselling^a^ versus Minimal telephone counselling in general/mixed population of smokers 20](#_Toc127954818)

[19. Stage-based telephone counselling^a^ versus usual care in general/mixed population of smokers 20](#_Toc127954819)

[20. Telephone counselling^a^ plus self-help material versus usual care in smokers NOT willing/motivated to quit 21](#_Toc127954820)

[Pharmacological approaches 23](#_Toc127954821)

[NRT 23](#_Toc127954822)

[21. NRT versus placebo in smokers who are motivated/wishing to quit 23](#_Toc127954823)

[22. NRT with or without additional advice/phone calls in relapsed smokers who are NOT motivated/wishing to quit 26](#_Toc127954824)

[23. NRT versus placebo or usual care in smokers with current or past depression 27](#_Toc127954825)

[24. NRT patch versus placebo patch in smokers with schizophrenia or schizoaffective disorder 28](#_Toc127954826)

[Cytisine 29](#_Toc127954827)

[25. Cytisine versus placebo in a general/mixed population of smokers 29](#_Toc127954828)

[26. Cytisine versus placebo in smokers motivated/wishing to quit 29](#_Toc127954829)

[Varenicline 30](#_Toc127954830)

[27. Varenicline versus Placebo In general/mixed population of smokers 30](#_Toc127954831)

[28. Varenicline versus Placebo in smokers motivated to quit 34](#_Toc127954832)

[29. Varenicline^a^ versus Placebo in smokers NOT motivated/wishing to quit 35](#_Toc127954833)

[30. Varenicline^a^ versus placebo in smokers with depression and motivated/willing to quit 36](#_Toc127954834)

[31. Varenicline versus placebo in smokers with schizophrenia, schizoaffective, bipolar, or other psychiatric disorder 37](#_Toc127954835)

[Bupropion 40](#_Toc127954836)

[32. Bupropion versus Placebo in a mixed population of smokers 40](#_Toc127954837)

[33. Bupropion versus Placebo in smokers motivated/wishing to quit at baseline and abstinent at follow-up 40](#_Toc127954838)

[34. Bupropion^a^ versus Placebo in smokers who are NOT motivated/ wishing to quit 41](#_Toc127954839)

[35. Bupropion versus placebo in smokers with current or past depression 43](#_Toc127954840)

[36. Bupropion versus placebo in smokers with schizophrenia or schizoaffective disorder 44](#_Toc127954841)

[Mixed approaches 48](#_Toc127954842)

[37. Combined pharmacotherapy and behavioural interventions versus Usual care or no/minimal intervention in the general/mixed population 48](#_Toc127954843)

[38. Interventions to increase adherence to medications for tobacco dependence compared to usual or standard care in smokers motivated/wishing to quit or reduce smoking 49](#_Toc127954844)

[39. Standard treatment plus extended NRT and extended CBT^a^ versus Standard treatment: Smoking cessation in smokers with past depression 50](#_Toc127954845)

[40. Individual smoking cessation intervention (cognitive behavioural therapy and motivational interviewing) plus NRT patch^a^ versus Routine care smokers with schizophrenia or schizoaffective disorder 51](#_Toc127954846)

[Alternative therapies 52](#_Toc127954847)

[41. Hypnotherapy^a^ versus Placebo drug in smokers motivated/wishing to quit 52](#_Toc127954848)

[42. St John’s Wort^a^ versus Placebo drug in smokers motivated/wishing to quit 52](#_Toc127954849)

[43. S-Adenosyl-L-Methionine (SAMe)^a^ versus Placebo drug in smokers motivated/wishing to quit 52](#_Toc127954850)

[44. Acupuncture in smokers motivated/wishing to quit 53](#_Toc127954851)

[45. Continuous auricular stimulation^a^ versus Sham stimulation in smokers motivated/wishing to quit 54](#_Toc127954852)

[46. Laser therapy^a^ versus Sham laser in smokers motivated/wishing to quit 54](#_Toc127954853)

[47. Electrostimulation^a^ versus Sham in smokers motivated/wishing to quit 55](#_Toc127954854)

# Outcome ratings – for reference

| **Number** | **Outcome** | **Median patient rating** | **Mean WG rating** |
| --- | --- | --- | --- |
| 1 | Smoking cessation | 8/10 (critical) | 8.8/10 (critical) |
| 2 | Smoking reduction | 7/10 (critical) | 5.0/10 (important) |
| 3 | Quality of life | 8/10 (critical) | 5.5/10 (important) |
| 4 | Relapse (there was nothing coded as this) | 6/10 (important) | 7/10 (important) |
| 5 | Adverse events | 7/10 (important) | 6.5/10 (important) |
| 6 | Weight gain | 5/10 (important) | 6.0/10 (important) |
| 7 | Change in mental state | 8/10 (critical) | 5.5/10 (important) |
| 8 | Loss of social group/relationship (there was nothing coded as this) | 5/10 (important) | 3.5/10 (of limited importance) |

Table template **–** Highlighted sections demonstrate where judgments must be made.

| Intervention XX versus XX in XX population  Any additional details worth noting | | | | | | | | | | |
| --- | --- | --- | --- | --- | --- | --- | --- | --- | --- | --- |
| **Outcome(s)**  **(Follow up (fu): time)**  **# of participants (# studies)** | | **Median Patient rating** | **Mean WG rating** | **Relative effect (Risk Ratio (RR), 95%CI)** | **Baseline (control) risk** | **Absolute difference with intervention** | **Assessment of absolute difference** | | | |
|  |  |  |  |  |  |  | **Point estimate** | **Lower 95% CI** | **Upper 95%CI** | **Comments on judgment** |
| Outcome number | Outcome (fu:)  N (n RCTs) | X/10  (rating) | X.X/10  (rating) | RR X.XX (X.XX to X.XX) | 70 per 1000 | **XX more per 1000** (from XX more to XX more) | Choose an item. | Choose an item. | Choose an item. |  |

# Behavioural interventions

# Physician advice

| Physician advice (minimal or intensive interventions)^a^ versus no advice (or usual care) in general/mixed population of smokers | | | | | | | | | | |
| --- | --- | --- | --- | --- | --- | --- | --- | --- | --- | --- |
| **Outcome(s)**  **(Follow up (fu): time)**  **# of participants (# studies)** | | **Median Patient rating** | **Mean WG rating** | **Relative effect (95%CI)** | **Baseline (control) risk** | **Absolute difference with intervention** | **Assessment of absolute difference** | | | |
|  |  |  |  |  |  |  | **Point estimate** | **Lower 95% CI** | **Upper 95%CI** | **Comments on judgment** |
| 1 | Smoking cessation (fu: 6+ mo)  22239 (26 RCTs) | 8/10  (critical) | 8.8/10  (critical) | RR 1.76 (1.58 to 1.96) | 48 per 1000 | **36 more per 1,000** (from 28 more to 46 more) | Moderate benefit | Moderate benefit | Moderate benefit | Point estimate, lower CI and upper CI are moderate benefit considering the health benefits of quitting smoking, baseline risks and ease of the intervention. No evidence on harms available from systematic review, but would anticipate very minimal harms, if any, from providing advice to patients. |

^a^ **Intervention:** Physician advice involved a 'stop smoking' message delivered verbally. Interventions were defined as minimal if delivered during a single session of <20-minute duration plus up to one follow-up visit with or without a leaflet. Intensive interventions involved a longer initial consultation, use of additional materials other than a leaflet, or more than one follow-up visit. Pharmacotherapy co-intervention provided in some of the studies. **Control:** Denoted as 'no advice (usual care)' by review authors. According to evidence tables, various control conditions across trials including smoking related written materials, 'control' (not further specified), 'no advice' (not further specified), self-monitoring of smoking, usual care, no intervention, and questionnaires. Additional behavioural and/or pharmacotherapy co-interventions provided to control participants in some studies

| Physician advice with follow-up^a^ versus Minimal intervention/advice with single visit in general/mixed population of smokers | | | | | | | | | | |
| --- | --- | --- | --- | --- | --- | --- | --- | --- | --- | --- |
| **Outcome(s)**  **(Follow up (fu): time)**  **# of participants (# studies)** | | **Median Patient rating** | **Mean WG rating** | **Relative effect (95%CI)** | **Baseline (control) risk** | **Absolute difference with intervention** | **Assessment of absolute difference** | | | |
|  |  |  |  |  |  |  | **Point estimate** | **Lower 95% CI** | **Upper 95%CI** | **Comments on judgment** |
| 1 | Smoking cessation (fu: 6+ mo)  1254 (5 RCTs) | 8/10  (critical) | 8.8/10  (critical) | RR 1.52 (1.08 to 2.14) | 90 per 1,000 | **47 more per 1,000** (from 7 more to 103 more) | Small but important benefit | Small but important benefit | Moderate benefit | Lower CI and point estimate provide a small but important benefit given the benefits of quitting, baseline risk, but greater demands of intervention (i.e., follow-up, and opportunity costs for the physician). Upper CI could be considered moderate based on the same factors. No evidence on harms available from systematic review, but would anticipate very minimal harms, if any, from providing advice to patients. |

^a^**Intervention:** Unclear how many follow-up visits are provided to the intervention arm. Pharmacotherapy co-intervention provided in one study. **Control**: Pharmacotherapy co-intervention provided in one study.

# Counselling

| Intensive advice^a^ versus Minimal advice in general/mixed population of smokers | | | | | | | | | | |
| --- | --- | --- | --- | --- | --- | --- | --- | --- | --- | --- |
| **Outcome(s)**  **(Follow up (fu): time)**  **# of participants (# studies)** | | **Median Patient rating** | **Mean WG rating** | **Relative effect (95%CI)** | **Baseline (control) risk** | **Absolute difference with intervention** | **Assessment of absolute difference** | | | |
|  |  |  |  |  |  |  | **Point estimate** | **Lower 95% CI** | **Upper 95%CI** | **Comments on judgment** |
| 1 | Smoking cessation (fu: 6+ mo)  9775 (15 RCTs) | 8/10  (critical) | 8.8/10  (critical) | RR 1.37 (1.20 to 1.56) | 76 per 1,000 | **28 more per 1,000** (from 15 more to 42 more) | Small but important benefit | Small but important benefit | Small but important benefit | Point estimate and CI suggest small but important benefit given the baseline risk (upper CI is a roughly 30% increase), and greater demands of intervention (i.e., intensive advice versus minimal advice). No evidence on harms available from systematic review, but would anticipate very minimal harms, if any, from providing advice to patients. |

^a^ **Intervention:** Intensive interventions involved a longer initial consultation, use of additional materials other than a leaflet, or more than one follow-up visit. Pharmacotherapy co-intervention provided in one study. **Control:** Interventions were defined as minimal if delivered during a single session of <20-minute duration plus up to one follow-up visit with or without a leaflet. Pharmacotherapy co-intervention provided in one study.

| Stage-based individual counselling and/or advice in a general/mixed population of smokers Intervention usually included follow up calls and was delivered by a physician, healthcare worker, or trained counsellor. | | | | | | | | | | |
| --- | --- | --- | --- | --- | --- | --- | --- | --- | --- | --- |
| **Outcome(s)**  **(Follow up (fu): time)**  **# of participants (# studies)** | | **Median Patient rating** | **WG rating** | **Relative effect (95%CI)** | **Baseline (control) risk** | **Absolute difference with intervention** | **Assessment of absolute difference** | | | |
|  |  |  |  |  |  |  | **Point estimate** | **Lower 95% CI** | **Upper 95%CI** | **Comments on judgment** |
| 1 | *Compared to usual care^a^*  Smoking cessation  (fu: 6 mo)  3293  (7 RCTs) | 8/10  (critical) | 8.8/10  (critical) | RR 1.19 (0.99 to 1.42) | 110 per 1000 | **21 more per 1000** (from 1 fewer to 46 more) | Little to no difference | Little to no difference | Small but important benefit | Upper CI suggest small but important benefit considering the baseline risk, the demands of the intervention (most studies provided counseling tailored to state of change as well as with follow-up phone calls, stage-based intervention will require more training and time on the part of the provider). Lower CI suggests little to no difference (1 less person per 1000 quitting smoking versus usual care, which in some studies involved active intervention such as advice), as does point estimate due to rationale above. No evidence on harms available from systematic review, but would anticipate very minimal harms given the type of intervention. |
|  | *Compared to assessment only^b^*  Smoking cessation  (fu: 6+ mo)  3056 (3 RCTs) | 8/10  (critical) | 8.8/10  (critical) | RR 1.28 (0.95 to 1.73) | 43 per 1000 | **12 more per 1000** (from 2 fewer to 31 more) | Little to no difference | Little to no difference | Small but important benefit | Point estimate and lower CI considered little to no difference, and upper CI considered small benefit based on rationale provided above. |

^a^ **Intervention:** One trial provided both stage-based counselling and advice; other trials offered either counselling or advice tailored to stage of change. Intervention usually includes follow-up phone call(s) and typically delivered by a physician, healthcare worker, or trained counsellor. Behavioural co-intervention provided in half of the studies; of these, three studies also recommended pharmacotherapy (NRT). One study encouraged pharmacotherapy (NRT or bupropion) without a behavioural co-intervention and another provided a lung function test and CO test feedback with individualized newsletter of the data. 13% of intervention participants in this analysis received specialized behavioural counselling (motivational interviewing). **Control:** Usual care varies across trial. In some trials, active smoking cessation interventions provided as part of or in conjunction with usual care. For example, one study provided controls with general smoking cessation advice and all study participants were encouraged to use NRT or bupropion. In another study, all participants (including controls), completed group smoking cessation programme, received telephone calls from a counsellor, and possibly recommended pharmacotherapy. In a third study, usual care consisted of brief motivational interviewing and a self-help manual. 13% of controls in this analysis received specialized behavioural counselling (motivational interviewing).

^b^ **Intervention:** One trial provided both stage-based counselling and advice; other trials offered either counselling or advice tailored to stage of change. Intervention usually includes follow-up phone call(s) and typically delivered by a physician, healthcare worker, or trained counsellor. Behavioural co-intervention provided in half of the studies; of these, three studies also recommended pharmacotherapy (NRT). One study encouraged pharmacotherapy (NRT or bupropion) without a behavioural co-intervention and another provided a lung function test and CO test feedback with individualized newsletter of the data. 13% of intervention participants in this analysis received specialized behavioural counselling (motivational interviewing). **Control:** Usual care varies across trial. In some trials, active smoking cessation interventions provided as part of or in conjunction with usual care. For example, one study provided controls with general smoking cessation advice and all study participants were encouraged to use NRT or bupropion. In another study, all participants (including controls) completed group smoking cessation programme, received telephone calls from a counsellor, and possibly recommended pharmacotherapy. In a third study, usual care consisted of brief motivational interviewing and a self-help manual. 13% of controls in this analysis received specialized behavioural counselling (motivational interviewing).

| Individual counselling^a^ versus minimal contact in general/mixed population | | | | | | | | | | |
| --- | --- | --- | --- | --- | --- | --- | --- | --- | --- | --- |
| **Outcome(s)**  **(Follow up (fu): time)**  **# of participants (# studies)** | | **Median Patient rating** | **Mean WG rating** | **Relative effect (95%CI)** | **Baseline (control) risk** | **Absolute difference with intervention** | **Assessment of absolute difference** | | | |
|  |  |  |  |  |  |  | **Point estimate** | **Lower 95% CI** | **Upper 95%CI** | **Comments on judgment** |
| 1 | Smoking cessation (fu: 6+ mo)  11100 (27 RCTs) | 8/10  (critical) | 8.8/10  (critical) | RR 1.57 (1.40 to 1.77) | 70 per 1000 | **40 more per 1000** (from 28 more to 54 more) | Small but important benefit | Small but important benefit | Small but important benefit | Point estimate, lower CI and upper CI provide a small but important benefit considering the benefits of quitting, baseline risk, and greater demands of intervention (i.e., individual counselling is relatively resource intensive). No evidence on harms available from systematic review, but would anticipate very minimal harms from providing counselling. |

^a^ **Intervention:** Individual counseling defined as at least one session of 10 or more minutes duration. The review restricted inclusion to counselling delivered by a smoking cessation specialist outside of routine clinical care. Additional behavioural and/or ‘other’ (e.g., computer-guided nicotine fading with contingent contract, cigarette substitute) co-interventions provided in majority of studies. Although this analysis excludes trials in which pharmacotherapy was offered to participants in all trial arms, it appears a few trials did offer NRT or a prescription for NRT to those in the intervention arm. Approximately 37.5% of intervention participants received specialized behavioural counselling (e.g., cognitive behavioural therapy, motivational interviewing, stage-based counselling, 5As). **Control:** Minimal contact control was usual care or brief advice (up to 15 min) with/without self-help materials. Some trials provided additional behavioural (e.g., advice/counselling on pharmacotherapy) or ‘other’ (e.g., monetary rewards for cessation) co-interventions. NRT was made available to control participants in three trials. No control participants received specialized behavioural counselling.

| Group therapy^a^ vs No intervention in general/mixed population of smokers Pharmacotherapy and other behavioural co-interventions provided in some studies, but no further details available | | | | | | | | | | |
| --- | --- | --- | --- | --- | --- | --- | --- | --- | --- | --- |
| **Outcome(s)**  **(Follow up (fu): time)**  **# of participants (# studies)** | | **Median Patient rating** | **Mean WG rating** | **Relative effect (95%CI)** | **Baseline (control) risk** | **Absolute difference with intervention** | **Assessment of absolute difference** | | | |
|  |  |  |  |  |  |  | **Point estimate** | **Lower 95% CI** | **Upper 95%CI** | **Comments on judgment** |
| 1 | Smoking cessation (fu: 6+ mo)  1098 (9 RCTs) | 8/10  (critical) | 8.8/10  (critical) | RR 2.60 (1.80 to 3.76) | 67 per 1000 | **108 more per 1000** (from 54 more to 186 more) | Moderate benefit | Moderate benefit | Large benefit | Lower CI and point estimate considered moderate benefit given the baseline risks and greater level of required involvement from patients and providers which may exceed capacity for many practices. Upper CI provides a large benefit given the relatively large increased likelihood of quitting. No evidence on harms available from systematic review, but would anticipate very minimal harms from providing group therapy. |

^a^ **Intervention:** As per review eligibility, group therapy was delivered over at least two sessions. **Control:** No intervention or minimal contact control.

# Tailored and non-tailored print-based material

| Individually tailored print-based self-help materials (no face-to-face)^a^ versus No materials/no intervention in general/mixed population of smokers | | | | | | | | | | |
| --- | --- | --- | --- | --- | --- | --- | --- | --- | --- | --- |
| **Outcome(s)**  **(Follow up (fu): time)**  **# of participants (# studies)** | | **Median Patient rating** | **WG rating** | **Relative effect (95%CI)** | **Baseline (control) risk** | **Absolute difference with intervention** | **Assessment of absolute difference** | | | |
|  |  |  |  |  |  |  | **Point estimate** | **Lower 95% CI** | **Upper 95%CI** | **Comments on judgment** |
| 1 | Smoking cessation (fu: 6+mo)  14359 (10 RCTs) | 8/10  (critical) | 8.8/10  (critical) | RR 1.34 (1.19 to 1.51) | 60 per 1,000 | **20 more per 1,000** (from 11 more to 31 more) | Small but important benefit | Small but important benefit | Small but important benefit | Point estimate, lower CI and upper CI suggest small but important benefit considering the benefits of quitting, baseline risk, and likely lack of harms. No evidence on harms available from systematic review, but would anticipate very minimal harms, if any, from providing tailored self-help materials. |

^a^ **Intervention:** Materials were tailored to the individual’s characteristics; several trials used computerized expert systems with tailoring according to baseline data. In all trials, materials were sent to participants without personal contact. No co-interventions provided. Control: Across trials, control conditions included assessment only, thank you letters only, and no intervention or information. No co-interventions provided to control participants.

| Non-tailored print-based self-help materials (no face-to-face contact) in general/mixed population of smokers | | | | | | | | | | |
| --- | --- | --- | --- | --- | --- | --- | --- | --- | --- | --- |
| **Outcome(s)**  **(Follow up (fu): time)**  **# of participants (# studies)** | | **Median Patient rating** | **WG rating** | **Relative effect (95%CI)** | **Baseline (control) risk** | **Absolute difference with intervention** | **Assessment of absolute difference** | | | |
|  |  |  |  |  |  |  | **Point estimate** | **Lower 95% CI** | **Upper 95%CI** | **Comments on judgment** |
| 1 | *Versus No materials/no intervention^a^*  Smoking cessation  (fu: 6+ mo)  13241 (11 RCTs) | 8/10  (critical) | 8.8/10  (critical) | RR 1.19 (1.03 to 1.37) | 51 per 1,000 | **10 more per 1,000** (from 2 more to 19 more) | Small but important benefit | Little to no difference | Small but important benefit | Point estimate and upper CI suggest small but important benefit given the benefits of quitting, baseline risk, and considering the relative ease of intervention. No evidence on harms available from systematic review, but would anticipate very minimal harms, if any, from providing general self-help material with no face-to-face contact. |
|  | *Versus brief leaflet^b^*  Smoking cessation  (fu: 6+ mo)  7023 (6 RCTs) | 8/10  (critical) | 8.8/10  (critical) | RR 0.87 (0.71 to 1.07) | 78 per 1,000 | **10 fewer per 1,000** (from 23 fewer to 5 more) | Small but important harm | Small but important harm | Little to no difference | Given the baseline risk, and harms of smoking, point estimate and lower CI are small but important harm because the intervention leads to fewer people quitting. Upper CI considered little to no difference considering the baseline risk. No evidence on harms available from systematic review, but would anticipate very minimal harms, if any, from providing general self-help material with no face-to-face contact. |

^a^ **Intervention:** All trials in this analysis sent non-tailored materials to participants without personal contact. Additional behavioural co-intervention provided in one trial. **Control:** Various control conditions across trials including no intervention, usual care, wait-list, letter apologizing for shortage of kits. No co-interventions provided to control arms across trials.

^b^ **Intervention:** All trials in this analysis sent non-tailored materials to participants without personal contact. No co-interventions provided to intervention arms across trials. **Control:** Brief leaflet considered to be a minimal print-based self-help intervention by authors. Behavioural co-intervention provided in all studies.

| Non-tailored print-based self-help materials^a^ (no face-to-face contact) Versus No materials/no intervention in smokers motivated/willing to quit | | | | | | | | | | |
| --- | --- | --- | --- | --- | --- | --- | --- | --- | --- | --- |
| **Outcome(s)**  **(Follow up (fu): time)**  **# of participants (# studies)** | | **Median Patient rating** | **WG rating** | **Relative effect (95%CI)** | **Baseline (control) risk** | **Absolute difference with intervention** | **Assessment of absolute difference** | | | |
|  |  |  |  |  |  |  | **Point estimate** | **Lower 95% CI** | **Upper 95%CI** | **Comments on judgment** |
| 1 | Smoking cessation  (fu: 6 mo)  924 (2 RCTs) | 8/10  (critical) | 8.8/10  (critical) | RR 10.91 (5.03 to 23.66) | 18 per 1,000 | **174 more per 1,000** (from 71 more to 398 more) | Large benefit | Large benefit | Large benefit | Increase in quit rate is large relative given the benefits of quitting and baseline risk (absolute increase over baseline of 17%). Point estimate, lower CI and upper CI all suggest large benefit considering the baseline risks and ease of the intervention. No evidence on harms available from systematic review, but would anticipate very minimal harms, if any, from providing general self-help material without face-to-face contact. |

^a^ **Intervention:** Both trials in this analysis sent non-tailored materials to participants without personal contact. No co-interventions provided. **Control:** Wait list control in both trials. No co-interventions provided to control arm.

| Non-tailored print-based self-help materials^a^ (WITH face-to-face contact) versus No intervention or leaflet only in general/mixed population of smokers | | | | | | | | | | |
| --- | --- | --- | --- | --- | --- | --- | --- | --- | --- | --- |
| **Outcome(s)**  **(Follow up (fu): time)**  **# of participants (# studies)** | | **Median Patient rating** | **WG rating** | **Relative effect (95%CI)** | **Baseline (control) risk** | **Absolute difference with intervention** | **Assessment of absolute difference** | | | |
|  |  |  |  |  |  |  | **Point estimate** | **Lower 95% CI** | **Upper 95%CI** | **Comments on judgment** |
| 1 | Smoking cessation (fu: 6+mo)  2822 (4 RCTs) | 8/10  (critical) | 8.8/10  (critical) | RR 1.39 (1.03 to 1.88) | 47 per 1,000 | **18 more per 1,000** (from 1 more to 41 more) | Small but important benefit | Little to no difference | Moderate benefit | Point estimate is considered a small but important benefit, and upper CI a moderate benefit, given the baseline risk, ease of intervention (face-to-face contact was simply to hand out materials), benefits of quitting. Lower CI suggests little to no difference. No evidence on harms available from systematic review, but would anticipate very minimal harms, if any, from providing general self-help material in person. |

^a^ **Intervention:** Investigators gave participants the materials in-person but did not provide advice to stop smoking. Most studies provided additional behavioural co-intervention. **Control:** No intervention provided to control participants in three trials. One trial provided materials that were not specific to smoking plus a video focused on cholesterol education.

| Stage-based expert systems or tailored self-help materials versus Assessment only in general/mixed population of smokers | | | | | | | | | | |
| --- | --- | --- | --- | --- | --- | --- | --- | --- | --- | --- |
| **Outcome(s)**  **(Follow up (fu): time)**  **# of participants (# studies)** | | **Median Patient rating** | **WG rating** | **Relative effect (95%CI)** | **Baseline (control) risk** | **Absolute difference with intervention** | **Assessment of absolute difference** | | | |
|  |  |  |  |  |  |  | **Point estimate** | **Lower 95% CI** | **Upper 95%CI** | **Comments on judgment** |
| 1 | Smoking cessation (fu: 6+mo)^a^  13597  (10 RCTs) | 8/10  (critical) | 8.8/10  (critical) | RR 1.35 (1.19 to 1.52) | 64 per 1000 | **22 more per 1000**  (from 12 to 33 more) | Small but important benefit | Small but important benefit | Small but important benefit | Point estimate, and CI represent a small but important benefit given the benefits of quitting, baseline risk, and greater demands of intervention (i.e., individualized reports and follow-up is relatively resource intensive compared to, for example, basic advice or counseling). No evidence on harms available from systematic review, but would anticipate very minimal harms, if any, from providing tailored self-help material. |
|  | Smoking cessation (fu: 14 mo)^b^  Unclear  (1 RCT) | 8/10  (critical) | 8.8/10  (critical) | Sig diff between groups with more quitters in the intervention arm (OR 3.74 , 95%CI: NR) | | | Large benefit | Unable to assess | Unable to assess | Insufficient data from review to determine imprecision. Data suggests a potentially large benefit (almost 4 times more likely to quit), but is difficult to interpret due to lack of baseline risk. No evidence on harms available from systematic review, but would anticipate very minimal harms, if any, from intervention. |

^a^ **Intervention:** The intervention involved personalised reports or letters matched to stage of change. Letters/reports often produced electronically according to questionnaires or interviews. The intervention may include self-help materials (tailored or standard) and/or follow-up phone calls. Behavioural co-intervention provided in one trial. In another trial, 25% of the intervention arm used NRT by 2-year follow-up. 4% of participants in the intervention arm received specialized behavioural counselling (i.e., motivational interviewing). **Control:** Review authors consider 'assessment only' as a no intervention control. Across trials, control conditions included 'assessment only', no intervention, a letter confirming no self-help information will be sent, and non-smoking related healthcare intervention including 3-5 minute dietary advice. In one trial, control participants did not receive smoking cessation advice but 21% had used NRT by 2-year follow-up. No control participants in this analysis received specialized behavioural counselling.

^b^ **Intervention:** Computer-generated tailored letter addressing (a) outcomes of smoking and quitting and (b) self-efficacy, active skills to quit, boosting confidence, coping skills. No co-interventions provided. **Control:** Participants received letter confirming no self-help information would be sent. No co-intervention provided.

# Computer and internet counselling

| Interactive internet/computer programs versus usual care or non-active control in general/mixed population of smokers | | | | | | | | | | |
| --- | --- | --- | --- | --- | --- | --- | --- | --- | --- | --- |
| **Outcome(s)**  **(Follow up (fu): time)**  **# of participants (# studies)** | | **Median Patient rating** | **WG rating** | **Relative effect (95%CI)** | **Baseline (control) risk** | **Absolute difference with intervention** | **Assessment of absolute difference** | | | |
|  |  |  |  |  |  |  | **Point estimate** | **Lower 95% CI** | **Upper 95%CI** | **Comments on judgment** |
| 1 | *Stage-based interactive computer programmes vs usual care^a^*  Smoking cessation  (fu: 12+ mo)  1702  (2 RCTs) | 8/10  (critical) | 8.8/10  (critical) | RR 1.14 (0.81 to 1.59) | 70 per 1000 | **10 more per 1000** (from 13 fewer to 41 more) | Small but important benefit | Small but important harm | Small but important benefit | Point estimate and upper CI suggest small but important benefit given benefits of quitting, baseline risk, but higher demand of intervention (it included supervision and feedback). Lower CI suggests small harm based on the same factors. No evidence on harms available from systematic review, but would anticipate very minimal harms, if any, from providing interactive care via computer programs. |
|  | *Interactive and tailored internet vs non-active controls (printed self-help guides or usual care)^b^*  Smoking cessation (fu: 6-12 mo)  6786 (8 RCTs) | 8/10  (critical) | 8.8/10  (critical) | RR 1.15 (1.01 to 1.30) | 129 per 1,000 | **19 more per 1,000** (from 1 more to 39 more) | Small but important benefit | Little to no difference | Small but important benefit | Point estimate and upper CI suggest small but important benefit given the benefits of quitting, baseline risk, and demands of intervention (internet programs tailored to personal characteristics that provide two-way flow of information). Lower CI suggests little to no difference given the above. No evidence on harms available from systematic review, but would anticipate very minimal harms, if any, from providing interactive programs via the internet. |
|  | *Internet plus behavioural support vs non-internet based, non-active controls (usual care, printed self-help guides, standard smoking cessation advice)^c^*  Smoking cessation (fu: 6-12 mo)  2334 (5 RCTs) | 8/10  (critical) | 8.8/10  (critical) | RR 1.69 (1.30 to 2.18) | 78 per 1,000 | **54 more per 1,000** (from 23 more to 92 more) | Moderate benefit | Small but important benefit | Large benefit | Point estimate suggests moderate benefit and upper CI suggests large benefit given the benefits of quitting, baseline risk, and resources involved in intervention (i.e. follow-up counseling from nurses, coaches along with internet intervention). Lower CI suggests small but important benefit. No evidence on harms available from systematic review, but would anticipate very minimal harms, if any, from providing an internet program with additional support. |

^a^ **Intervention** typically delivered within fixed time frame and sessions may include supervision with oral and written feedback provided. No co-interventions provided in either trial. **Comparator** described as usual care by review authors. In one study, usual care group received advice plus self-help materials. In the second school-based study, usual care was 'standard English curriculum on smoking i.e. smoking quizzes and advice on persuading people to quit'. No co-interventions provided in either study.

^b^ **Intervention:** One study provided NRT as an adjunct intervention. Another study offered free nicotine patches and bupropion to all participants and their partners who wanted to quit. No co-interventions provided in remaining studies. **Control:** Across studies, 'non-active controls' consisted of printed self-help guides or usual care. One study offered free nicotine patches and bupropion to all participants, including controls, and their partners who wanted to quit. No co-interventions provided to control arm in remaining studies.

^c^ **Intervention**: Internet intervention plus behavioural support provided by nurses, peer coaches, or tobacco treatment specialists. In two trials, all participants were using or offered pharmacotherapy. **Control**: Across studies, ‘non-active control’ consisted of usual care, printed self-help guides, and standard smoking cessation advice. In two trials, all participants including controls were using or offered pharmacotherapy.

# Mobile phone, hotline, and telephone counselling

| Interactive voice response (IVR) systems^a^ versus No intervention: Smoking cessation in general/mixed population of smokers | | | | | | | | | | |
| --- | --- | --- | --- | --- | --- | --- | --- | --- | --- | --- |
| **Outcome(s)**  **(Follow up (fu): time)**  **# of participants (# studies)** | | **Median Patient rating** | **WG rating** | **Relative effect (95%CI)** | **Baseline (control) risk** | **Absolute difference with intervention** | **Assessment of absolute difference** | | | |
|  |  |  |  |  |  |  | **Point estimate** | **Lower 95% CI** | **Upper 95%CI** | **Comments on judgment** |
| 1 | Smoking cessation (fu: 24 mo)  NR (1 RCT) | 8/10  (critical) | 8.8/10  (critical) | Little to no difference between groups at 24-month follow-up (21.7% of intervention group versus 42.9% of control group; P = 0.13). | | | Large harm | Unable to asses | Unable to asses | Insufficient data from review to determine imprecision. Review authors characterized as little to no difference but point estimate suggests large harm (much lower percentage of quitters in intervention compared to control). Lack of statistical significance in results suggest imprecision. |

^a^ **Intervention:** IVR is a type of automated telephone communication systems (ATCS). Participants received IVR calls on their quit date, on days 3, 8, and 11, and then every two weeks between weeks 13 and 52. Calls were 3 to 5 minutes long. Prior to randomization, participants had received varenicline for 12 weeks (0.5 mg on days 1-3, 1 mg on days 4-7, and 2 mg until end of week 12) and IVR. **Control:** In the first phase of the study (i.e., prior to randomization), participants received varenicline for 12 weeks (0.5 mg on days 1-3, 1 mg on days 4-7, and 2 mg until end of week 12) and IVR. After randomization, participants in control group no longer received IVR.

| Mobile phone short message service (SMS)^a^ vs control in smokers motivated/wishing to quit | | | | | | | | | | |
| --- | --- | --- | --- | --- | --- | --- | --- | --- | --- | --- |
| **Outcome(s)**  **(Follow up (fu): time)**  **# of participants (# studies)** | | **Median Patient rating** | **WG rating** | **Relative effect (95%CI)** | **Baseline (control) risk** | **Absolute difference with intervention** | **Assessment of absolute difference** | | | |
|  |  |  |  |  |  |  | **Point estimate** | **Lower 95% CI** | **Upper 95%CI** | **Comments on judgment** |
| 1 | Smoking cessation (fu: 6 mo) – *Point prevalence with* *missing data considered as still smoking*  1705 (1 RCT) | 8/10  (critical) | 8.8/10  (critical) | RR 1.07 (0.91 to 1.26) | 237 per 1,000 | **17 more per 1,000** (from 21 fewer to 62 more) | Small but important benefit | Small but important harm | Moderate benefit | Point estimate and upper CI small but important benefit considering benefits of quitting, little to no potential harms identified below, baseline risk, ease of intervention (text messages only). Lower CI suggests small but important harm given fewer individuals will quit smoking. |
|  | Smoking cessation (fu: 6 mo) - *Point prevalence using last measurement carried forward where data was missing at 6 months*  1705 (1 RCT) | 8/10  (critical) | 8.8/10  (critical) | RR 1.28 (1.11 to 1.48) | 273 per 1,000 | **76 more**  **per 1,000** (from 30 more to 131 more) | Large benefit | Small but important benefit | Large benefit | Point estimate and upper CI suggest large benefit considering benefits of quitting, little to no risk of potential harms identified below, baseline risk, ease of intervention (text messages only). Lower CI suggests small but important benefit based on same rationale. |
|  | Smoking cessation (fu: 6 mo) - *Allowing three or fewer lapses of two or fewer cigarettes per lapse.*  1705 (1 RCT) | 8/10  (critical) | 8.8/10  (critical) | RR 1.64 (1.12 to 2.42) | 46 per 1,000 | **29 more per 1,000** (from 5 more to 65 more) | Small but important benefit | Little to no difference | Moderate benefit | Point estimate considered small but important benefit and upper CI moderate benefit considering benefits of quitting, little to no risk of potential harms identified below, baseline risk, ease of intervention (text messages only). Lower CI suggests a small but important benefit based on same rationale. |
|  | Smoking cessation (fu: 6 mo) – *Complete continuous abstinence (no lapses allowed)*  1705 (1 RCT) | 8/10  (critical) | 8.8/10  (critical) | RR 1.50 (0.92 to 2.44) | 30 per 1,000 | **15 more per 1,000** (from 2 fewer to 44 more) | Small but important benefit | Little to no difference | Moderate benefit | Point estimate is judged as a small benefit, and upper CI as moderate benefit, considering benefits of quitting, little to no risk of potential harms identified below, baseline risk, ease of intervention (text messages only). Lower CI suggests little to no difference based on same rationale. |
| 5 | Adverse events (car crash)  (fu: 6 mo)  1705 (1 RCT) | 7/10 (important) | 6.5/10 (important) | RR 0.88 (0.58 to 1.35) | 50 per 1,000 | **6 fewer per 1,000** (from 21 fewer to 18 more) | Little to no difference | Small but important benefit | Small but important harm | Considering potential severity of the outcome, potential offset of smoking cessation benefits, and baseline risk, upper CI suggests small but important harm. Lower CI suggests small but important benefit, and point estimate suggest little to no difference considering the same factors. |
|  | Adverse events (pain in thumb/finger joints)  (fu: 6 mo)  1705 (1 RCT) | 7/10 (important) | 6.5/10 (important) | RR 1.08 (0.74 to 1.59) | 56 per 1,000 | **5 more per 1,000** (from 15 fewer to 33 more) | Little to no difference | Small but important benefit | Small but important harm | Considering relatively low severity of the outcome, offset of smoking cessation benefits, and baseline risk, point estimate suggests little to no difference. Lower CI considered small benefit due to added benefit of smoking cessation. Upper CI considered small harm due to low severity of the outcome (which could lead to discontinuation), but potential smoking cessation benefits. |

^a^ **Text messaging intervention**: Sent regular, personalized text messages with smoking cessation advice, support, and distraction, which decreased in frequency over time. Messages sent between healthcare provider or buddy (lay health worker or peer supported) and participant. **Control:** Sham text messaging. One text message biweekly thanking participants for involvement and reminder of free month of text messaging if they complete follow-up.

| Mobile phone based interventions^a^ versus usual care in smokers motivated/wishing to quit | | | | | | | | | | |
| --- | --- | --- | --- | --- | --- | --- | --- | --- | --- | --- |
| **Outcome(s)**  **(Follow up (fu): time)**  **# of participants (# studies)** | | **Median Patient rating** | **WG rating** | **Relative effect (95%CI)** | **Baseline (control) risk** | **Absolute difference with intervention** | **Assessment of absolute difference** | | | |
|  |  |  |  |  |  |  | **Point estimate** | **Lower 95% CI** | **Upper 95%CI** | **Comments on judgment** |
| 1 | Smoking cessation (fu: 6+ mo)  11885 (12 RCTs) | 8/10  (critical) | 8.8/10  (critical) | RR 1.67 (1.46 to 1.90) | 56 per 1,000 | **37 more per 1,000** (from 26 more to 50 more) | Moderate benefit | Small but important benefit | Moderate benefit | Considering benefits of quitting, indirect evidence of lack of potential harms identified in studies of SMS-only interventions, baseline risk, point estimate and upper CI suggest moderate benefit. Lower CI considered a small but important benefit based on the same rationale. |

^a^ **Intervention:** Nearly all studies had text messaging (SMS) as the main component of the intervention; however, one trial differed in that participants received mobile phone-based counselling (CBT and motivational). In-person visits or assessments were provided in addition to SMS in five studies. Five studies provided additional behavioural co-interventions (e.g., booklets) and of these, three studies also offered or made pharmacotherapy available to participants as part of usual care. 4% of intervention participants in this analysis received specialized behavioural counselling (CBT). **Usual care:** Comparator is described as ‘usual care’ by review authors in the discussion section of the report. Various control conditions across trials including no intervention, text messages, written/internet untailored materials, untailored messages, and standard cessation advice and treatment. One trial provided a behavioral co-intervention.

| Hotline and self-help materials^a^ versus Minimal intervention in general/mixed population of smokers | | | | | | | | | | |
| --- | --- | --- | --- | --- | --- | --- | --- | --- | --- | --- |
| **Outcome(s)**  **(Follow up (fu): time)**  **# of participants (# studies)** | | **Median Patient rating** | **WG rating** | **Relative effect (95%CI)** | **Baseline (control) risk** | **Absolute difference with intervention** | **Assessment of absolute difference** | | | |
|  |  |  |  |  |  |  | **Point estimate** | **Lower 95% CI** | **Upper 95%CI** | **Comments on judgment** |
| 1 | Smoking cessation (fu: 12-18 mo)  3327 (2 RCTs) | 8/10  (critical) | 8.8/10  (critical) | RR 1.62 (1.16 to 2.25) | 34 per 1,000 | **21 more per 1,000** (from 5 more to 42 more) | Small but important benefit | Little to no difference | Moderate benefit | Point estimate considered small benefit and upper CI considered moderate benefit considering benefits of quitting, baseline risk, and given relative ease of intervention (although no information given on how often hotline was used) and lack of harms from the intervention. Lower CI suggests little to no difference. No evidence on harms available from systematic review, but would anticipate very minimal harms, if any, from providing access to a hotline. |

^a^ **Hotline and self-help materials**: One study was a combination intervention: promoted 24-hour hotline, daytime access to counsellors, and provision of American Lung Association self-help (ALA S-H) manuals. The second study comprised Quitline proactive contact, quit kits (national Quitline printed resources), individual face-to-face counselling with practice nurse, and three proactive telephone calls from an experienced counsellor. Pharmacotherapy (NRT patch) co-intervention offered in one of the two trials. **Minimal intervention:** Review authors report the control as 'minimal intervention'. This consists of self-help manual in one trial (ALA S-H manual). In the second trial, primary care providers’ delivered their usual care, such as advice, referral to Quitline, or both. Pharmacotherapy (NRT patch) co-intervention in one of the two trials).

| Telephone counselling^a^ versus Usual care telephone call in general/mixed population of smokers | | | | | | | | | | |
| --- | --- | --- | --- | --- | --- | --- | --- | --- | --- | --- |
| **Outcome(s)**  **(Follow up (fu): time)**  **# of participants (# studies)** | | **Median Patient rating** | **WG rating** | **Relative effect (95%CI)** | **Baseline (control) risk** | **Absolute difference with intervention** | **Assessment of absolute difference** | | | |
|  |  |  |  |  |  |  | **Point estimate** | **Lower 95% CI** | **Upper 95%CI** | **Comments on judgment** |
| 1 | Smoking cessation (fu: 12 mo) – *counselling for smoking reduction*  375 (1 RCT) | 8/10  (critical) | 8.8/10  (critical) | RR 2.32 (0.98 to 5.52) | 37 per 1,000 | **49 more per 1,000** (from 1 fewer to 167 more) | Moderate benefit | Little to no difference | Large benefit | Point estimate considered moderate benefit and upper CI considered large benefit considering benefits of quitting, baseline risk, and relative ease of intervention (i.e., 45 minutes of total counselling time). Lower CI is considered little to no difference. No evidence on harms available from systematic review, but would anticipate very minimal harms, if any, from providing telephone counselling. |
|  | Smoking cessation (fu: 12 mo) - *brief motivational telephone counselling*  374 (1 RCT) | 8/10  (critical) | 8.8/10  (critical) | RR 2.63 (1.12 to 6.14) | 37 per 1,000 | **60 more per 1,000** (from 4 more to 190 more) | Moderate benefit | Little to no difference | Large benefit | Point estimate considered moderate benefit given benefits of quitting, baseline risk, and ease of intervention (i.e., 45 minutes of total counselling time). Upper CI is considered large (19% absolute increase in likelihood of quitting over baseline). Lower CI suggests little to no difference. No evidence on harms available from systematic review, but would anticipate very minimal harms, if any, from providing telephone counselling. |

^a^ **Telephone counselling**: Three 15-minute calls (baseline and weeks 2 and 4). No co-interventions provided. **Usual care telephone call**: One five-minute call. No co-intervention provided.

| Intensive telephone counselling^a^ versus Minimal telephone counselling in general/mixed population of smokers | | | | | | | | | | |
| --- | --- | --- | --- | --- | --- | --- | --- | --- | --- | --- |
| **Outcome(s)**  **(Follow up (fu): time)**  **# of participants (# studies)** | | **Median Patient rating** | **WG rating** | **Relative effect (95%CI)** | **Baseline (control) risk** | **Absolute difference with intervention** | **Assessment of absolute difference** | | | |
|  |  |  |  |  |  |  | **Point estimate** | **Lower 95% CI** | **Upper 95%CI** | **Comments on judgment** |
| 1 | Smoking cessation (fu: 6+ mo)  2602 (3 RCTs) | 8/10  (critical) | 8.8/10  (critical) | RR 1.27 (1.12 to 1.44) | 237 per 1,000 | **64 more per 1,000** (from 28 more to 104 more) | Moderate benefit | Small but important benefit | Large benefit | Considering the benefits of quitting, baseline risk and higher demands of intervention, point estimate considered a moderate benefit and lower CI considered small but important benefit. Upper CI considered a large benefit based on the same rationale. No evidence on harms available from systematic review, but would anticipate very minimal harms, if any, from providing telephone counselling, especially since the control group also received some counselling. |

^a^ **Intensive telephone counselling**: moderate intensity of three to five calls across studies. Behavioural co-intervention provided in one trial; a second trial provided behavioural and pharmacotherapy to participants. **Minimal intervention**: Minimal telephone counselling/low-intensity (single call) telephone counselling. Behavioural co-intervention provided in one trial; in a second trial, behavioural and pharmacotherapy co-intervention provided to participants.

| Stage-based telephone counselling^a^ versus usual care in general/mixed population of smokers | | | | | | | | | | |
| --- | --- | --- | --- | --- | --- | --- | --- | --- | --- | --- |
| **Outcome(s)**  **(Follow up (fu): time)**  **# of participants (# studies)** | | **Median Patient rating** | **WG rating** | **Relative effect (95%CI)** | **Baseline (control) risk** | **Absolute difference with intervention** | **Assessment of absolute difference** | | | |
|  |  |  |  |  |  |  | **Point estimate** | **Lower 95% CI** | **Upper 95%CI** | **Comments on judgment** |
| 1 | Smoking cessation  (fu: 12 mo)  318  (1 RCT) | 8/10  (critical) | 8.8/10  (critical) | RR 1.27 (0.56 to 2.89) | 60 per 1000 | **16 more per 1000** (from 27 fewer to 114 more) | Little to no difference | Moderate harm | Moderate benefit | Given the demands of the intervention point estimate considered little to no difference. Lower CI caused fewer people to quit and is considered moderate harm given the associated harms of continuing to smoke. Upper CI considered a moderate benefit. No evidence on harms available from systematic review, but would anticipate minimal harms, other than the potential to not treat, from providing stage-based telephone counselling. |

^a^ **Intervention:** Assessments and counselling delivered over the phone with advice tailored to stage of change. Counselling was based on specialized approaches (5As, motivational interviewing, 5Rs). Those attempting to quit received quit pack and encouraged to use NRT. Those not ready to quit received motivational intervention to advance stage of change. **Control:** Participants received usual care including free quit kits. No co-interventions provided.

| Telephone counselling^a^ plus self-help material versus usual care in smokers NOT willing/motivated to quit | | | | | | | | | | |
| --- | --- | --- | --- | --- | --- | --- | --- | --- | --- | --- |
| **Outcome(s)**  **(Follow up (fu): time)**  **# of participants (# studies)** | | **Median Patient rating** | **WG rating** | **Relative effect (95%CI)** | **Baseline (control) risk** | **Absolute difference with intervention** | **Assessment of absolute difference** | | | |
|  |  |  |  |  |  |  | **Point estimate** | **Lower 95% CI** | **Upper 95%CI** | **Comments on judgment** |
| 1 | Smoking cessation  (fu: 12 mo)  320 (1 RCT) | 8/10  (critical) | 8.8/10  (critical) | RR 1.49 (0.59 to 3.76) | 45 per 1,000 | **22 more per 1,000** (from 18 fewer  to124 more) | Small but important benefit | Small but important harm | Large benefit | Point estimate suggests small benefit given benefits of quitting, potentially challenging population (i.e., not willing/motivated to quit), and given baseline risk. Upper CI suggest large benefit for same reasons. Lower CI suggests small harm given fewer patients quit smoking, and given harms of continuing to smoke. No evidence on other harms available from systematic review, but would anticipate very minimal harms, if any, from providing telephone counselling and self-help material. |
| 2 | Smoking reduction (>50% of baseline or cessation  (fu: 12 mo)  320 (1 RCT) | 7/10 (critical) | 5/10 (important) | RR 1.34 (0.88 to 2.05) | 186 per 1,000 | **63 more per 1,000** (from 22 fewer  to 195 more) | Small but important benefit | Small but important harm | Moderate benefit | Point estimate suggests small benefit given that those reducing by 50% are still continuing to smoke (still significantly increased morbidity and mortality, unless this leads to quitting, which could potentially follow), that the data here likely includes those who quit completely (i.e., number of reducers is likely smaller than indicated), and considering more challenging population (i.e., not willing/motivated to quit) and the relative ease of the intervention. Upper CI suggest a moderate benefit based on the same rationale. Lower CI suggests small harm (2% less individuals reducing their smoking) given harms of continuing to smoke at greater levels. No evidence on harms available from systematic review, but would anticipate very minimal harms, if any, from providing telephone counselling and self-help materials. |
|  | Smoking reduction (CO by >50%)  (fu: 12 mo)  320 (1 RCT) | 7/10 (critical) | 5/10 (important) | RR 0.99 (0.58 to 1.71) | 141 per 1,000 | **1 fewer per 1,000** (from 59 fewer to 100 more) | Little to no difference | Moderate harm | Small but important benefit | Point estimate suggests little to no difference (1 less person per 1000 reducing their CO by 50%). Lower CI is a moderate harm (6% fewer patients reducing their CO by 50%) due to harms of continuing to smoke, and upper CI is small benefit. Judgements were based on baseline risk, population (i.e., not willing/motivated to quit), and outcome (reduction in smoking, which still causes significantly increased morbidity and mortality, as opposed to quitting) and that these results likely also include quitters. No evidence on harms available from systematic review, but would anticipate very minimal harms, if any, from providing telephone counselling and self-help materials. |
|  | Smoking reduction (# of cigarettes per day from baseline)  (fu: 12 mo)  320 (1 RCT) | 7/10 (critical) | 5/10 (important) | Number of cigarettes/day decreased from baseline in both groups (mean change from baseline (SD); Intervention: 21.2 (9.4), Usual care: 20.1 (8.9)). No difference between groups at 12-month follow-up (mean (SD): Intervention: 15.8 (10. 3), Usual care: 15.3 (9.2). | | | Little to no difference | Unable to asses | Unable to asses | Insufficient data from review to determine imprecision. Data suggests little to no difference between groups. |
|  | Reduction in CO from baseline (fu: 12 months) | 7/10 (critical) | 5/10 (important) | CO levels decreased from baseline in both groups (baseline mean (SD) - Intervention 29.8 (13.9), Usual care 29.8 (14.5); 12 months - Intervention: 24.9 (14.0). Usual care: 24.3 (13.8)). No significant between-group difference in the change from baseline. | | | Little to no difference | Unable to asses | Unable to asses | Insufficient data from review to determine imprecision. Data suggests little to no difference between groups. |

^a^ **Intervention:** Participants instructed to reduce smoking by 50% or more; cessation encouraged thereafter. Self-help materials were individually tailored newsletters and a targeted newsletter. No co-interventions provided. **Control:** Usual care consisted of usual care plus generic health mailings. No co-interventions provided.

# Pharmacological approaches

# NRT

| NRT versus placebo in smokers who are motivated/wishing to quit | | | | | | | | | | |
| --- | --- | --- | --- | --- | --- | --- | --- | --- | --- | --- |
| **Outcome(s)**  **(Follow up (fu): time)**  **# of participants (# studies)** | | **Median Patient rating** | **WG rating** | **Relative effect (95%CI)** | **Baseline (control) risk** | **Absolute difference with intervention** | **Assessment of absolute difference** | | | |
|  |  |  |  |  |  |  | **Point estimate** | **Lower 95% CI** | **Upper 95%CI** | **Comments on judgment** |
| 1 | *In relapsed smokers motivated to quit*  *NRT patch versus placebo patch^a^*  Smoking cessation  (fu: 6 mo)  629 (1 RCT) | 8/10  (critical) | 8.8/10  (critical) | RR 1.25 (0.34 to 4.60) | Not available | Not available | Small but important benefit | Moderate harm | Large benefit | Baseline risk and absolute increase in quit rate were not reported. Relative effect suggests potential small benefit with potential range from moderate harm to large benefit due to health benefits of quitting smoking, but difficult to interpret without baseline risk. Information on harms suggests that harms identified are minimal. |
| 5.1 | *Various forms of NRT^b^*  Adverse events  (fu: not reported)  NR (6 RCTs) | 6.5/10 (important) | 7/10 (important) | For nicotine gum, most common adverse events are hiccoughs, gastrointestinal disturbances, jaw pain, and orodental problems. With nicotine patch, typically mild skin sensitivity and local skin irritation in up to 54% of patch users. Throat irritation, coughing, and oral burning are common with nicotine inhalator. With nasal spray, irritation and runny nose. For oral spray, hiccoughs and throat irritation. Symptoms associated with nicotine sublingual tablets include hiccoughs, burning and smarting sensation in the mouth, sore throat, coughing, dry lips, and mouth ulcers. Reactions to NRT are usually not severe enough to prompt discontinuation of treatment. Trials could not be pooled due to heterogeneity with respect to the nature, timing and duration of symptoms. | | | Little to no difference | Unable to assess | Unable to assess | Increase in adverse advents identified considered trivial (little to no difference) when taking into account relative severity (not severe enough to prompt discontinuation) and potential benefits of quitting smoking (indirect evidence from relapsed smokers, and from those not motivated to quit). However, difficult to interpret due to lack of baseline risk to compare for absolute differences. Insufficient data to determine imprecision. |
| 5.2 | *Various forms of NRT^c^*  Adverse events (palpitations/chest pains)  (fu: range of times)  11074 (15 RCTs) | 7/10 (important) | 6.5/10 (important) | **OR 1.88** (1.37 to 2.57)  **RR 1.86**  (1.36 to 2.51) | 14 per 1,000 | **12 more per 1,000** (from 5 more to 21 more) | Small but important harm | Little to no difference | Small but important harm | When taking into account potential benefits for smoking cessation (indirect evidence from relapsed smokers, and from those not motivated to quit), and nature of adverse event (may take the patient to ER or require significant medical work up), increase in palpitations/chest pains is considered a small but important harm for point estimate and upper CI. Lower CI is considered little to no difference. |
| 5.3 | *Forms of NRT not reported*  Adverse events (attrition)  (fu: not reported) | 7/10 (important) | 6.5/10 (important) | Authors state that attrition rates in NRT groups were generally similar to or lower than in control groups among included studies. | | | Little to no difference | Unable to asses | Unable to asses | Insufficient data from review to determine imprecision. Data suggests little to no difference between groups but is difficult to interpret. |
| 6 | Weight gain  (fu: end of treatment)^d^  2600 (19 RCTs) | 5/10 (important) | 6.0/10 (important) | - | The mean weight gain in abstinent smokers was 1.1-5.6 kg | **MD 0.69 kg lower** (0.88 lower to 0.51 lower) | Little to no difference | Little to no difference | Little to no difference | Abstinent smokers gained up to 5.6 kg, and this may be reduced by up to ~0.9 kg with NRT. Point estimate, lower CI, and upper CI are considered trivial (little to no difference) weight change and could be due to daily fluctuations. |
|  | Weight gain  (fu: 6 mo)^e^  771 (9 RCTs) | 5/10 (important) | 6.0/10 (important) | - | The mean weight gain in abstinent smokers was 2.58-5.8 kg | **MD 0.37 kg lower** (0.88 lower to 0.14 higher) | Little to no difference | Little to no difference | Little to no difference | Abstinent smokers gained up to 5.8 kg, and this may be reduced by up to ~0.9 kg or increased by up to ~0.1kg with NRT. Point estimate, lower CI, and upper CI are considered trivial (little to no difference) weight change and could be due to daily fluctuations. |
|  | Weight gain  (fu: 12 mo)^f^  1334 (15 RCTs) | 5/10 (important) | 6.0/10 (important) | - | The mean weight gain in abstinent smokers ranged from 3.0-8.3 kg | **MD 0.42 kg lower** (0.92 lower to 0.08 higher) | Little to no difference | Little to no difference | Little to no difference | Abstinent smokers gained up to 8.3 kg, and this may be reduced by up to ~0.9 kg or increased by up to ~0.1 kg with NRT. Point estimate, lower CI, and upper CI are considered trivial (little to no difference) weight change and could be due to daily fluctuations. |

^a^ **Intervention:** nicotine patch decreasing dose (21 mg/24 hr to 7mg/24 hr) over 12 weeks. **Control:** placebo patch. **Co-intervention (both groups)**: Minimal additional behavioural support as per review authors.

^b^ **Intervention:** Formulation and dose not reported for majority of studies. **Control:** Placebo. **Co-interventions (both groups):** 67% studies without information reported. Remaining studies with high level of behavioural support.

^c.^ **Intervention:** Varied NRT formulations and doses among nine studies; remaining studies NR. **Control:** Placebo. **Co-interventions (both groups):** High-intensity behavioural support provided in 33% studies, low-intensity (minimal/none) provided in 27%, and not reported in 40% of studies.

^d^ **NRT:** Gum (n=4), patch (n=10), inhaler (n=2), sublingual tablet (n=2), intranasal spray (n=1). Patch: Variation in dose across trials but most studies provided participants with either lower (e.g., 14 or 15 mg) and/or higher dose (e.g., 21/22 or 25 mg) based on dependence or preference. Gum: Dosing varied across trials including 2 mg ad libitum (1 trial), 10 to 12 pieces daily (1 trial), 2 mg followed by randomization to 7, 15, or 30 pieces daily (1 trial), and 2 mg 9-15 pieces or 4 mg 9-15 pieces (1 trial). Treatment duration for gum was from 8 weeks to 1 year (median = 12 weeks). Inhaler: Up to 6-month use of 2-10/day in one trial and minimum 4/day in the other trial. Sublingual tablet: Up to 24 weeks of 2 mg in one trial and 4 mg in the second trial. Intranasal spray: 0.5mg/dose for up to 1 year. Behavioural co-intervention provided in most trials. One trial examining NRT intranasal spray also provided all participants with NRT patch. 1.3% of participants received specialized behavioural counselling (CBT). **Placebo:** Comparator is reported as ‘placebo’ by review authors; however, the control condition is group therapy or described as 'no gum' in one trial each. Behavioural co-intervention provided in most trials. One trial examining NRT intranasal spray provided both arms with NRT patch. 3.2% of participants received specialized behavioural counselling (CBT).

^e^ **NRT:** Gum (n=2), patch (n=4), inhaler (n=1), sublingual tablets (n=2). Behavioural co-intervention provided in most trials. One trial examining NRT patch also provided all participants with NRT inhaler in addition to behavioural support. One trial examining NRT patch also provided all participants with NRT gum. **Placebo:** Comparator is reported as ‘placebo’ by review authors; however, control condition is group therapy in one trial. Behavioural co-intervention provided in most trials. One trial examining NRT patch provided all participants (including controls) with NRT inhaler in addition to behavioural support. One trial examining NRT patch provided all participants (including controls) with NRT gum.

^f^ **NRT**: Gum (n=1), patch (n=6), intranasal spray (n=3), inhaler (n=2), sublingual tablet (n=3). Behavioural co-intervention provided in most trials. One trial examining NRT patch also provided all participants with NRT inhaler in addition to behavioural support. Two trials examining either NRT intranasal spray or NRT patch also provided all participants with a second type of NRT (i.e., NRT patch or NRT gum, respectively). **Placebo:** Comparator is reported as 'placebo' by review authors; however, in one trial, control condition is group therapy. Behavioural co-intervention provided in most trials. One trial examining NRT patch provided all participants (including controls) with NRT inhaler in addition to behavioural support. Two trials examining either NRT intranasal spray or NRT patch provided all participants (including controls) with NRT patch or NRT gum, respectively.

| NRT with or without additional advice/phone calls in relapsed smokers who are NOT motivated/wishing to quit | | | | | | | | | | | | |
| --- | --- | --- | --- | --- | --- | --- | --- | --- | --- | --- | --- | --- |
| **Outcome(s)**  **(Follow up (fu): time)**  **# of participants (# studies)** | | **Median Patient rating** | **WG rating** | **Relative effect (95%CI)** | **Baseline (control) risk** | | **Absolute difference with intervention** | | **Assessment of absolute difference** | | | |
|  |  |  |  |  |  |  |  |  | **Point estimate** | **Lower 95% CI** | **Upper 95%CI** | **Comments on judgment** |
| 1 | *NRT plus advice/phone calls^a^*  Gum or patch  Smoking cessation  (fu: 6 mo)  NR (1 RCT) | 8/10  (critical) | 8.8/10  (critical) | Quit rate significantly higher in the intervention group compared to those receiving no intervention. | | | | | Small but important benefit | Unable to assess | Unable to assess | Insufficient data from review to determine imprecision. Results suggests small but important benefit due to health benefits of quitting, but are difficult to interpret. No harms identified in this review. In other populations, data on harms was difficult to interpret but not judged as sufficient to reduce benefit to trivial due to harms associated with continuing to smoke. |
|  | *NRT ^b^*  Various forms  Smoking cessation  (fu: 12-24 mo)  3081 (8 RCTs) | 8/10  (critical) | 8.8/10  (critical) | RR 1.87 (1.43 to 2.44) | | 50 per 1000 | | **44 more per 1000** (from 22 more to 73 more) | Moderate benefit | Small but important benefit | Moderate benefit | Increase in quit rate (point estimate and upper CI) is judged as moderate benefit when considering benefits of quitting, baseline risk, potentially more challenging population (not motivated to quit). Lower CI is judged as a small benefit based on the same rationale. No harms identified in this review. In other populations, data on harms was difficult to interpret but harms appear trivial to small and therefore not judged as sufficient to reduce to small but important benefit due to harms associated with continuing to smoke. |
| 2 | *NRT plus advice/phone calls^a^*  Gum or patch  Smoking reduction (# of cigarettes per day)  (fu: 6 mo)  NR (1 RCT) | 7/10 (critical) | 5/10 (important) | Reduction rate significantly higher in the intervention group compared to those receiving no intervention. | | | | | Small but important benefit | Unable to assess | Unable to assess | Insufficient data from review to determine imprecision. Results suggests small but important benefit , but is difficult to interpret (reduction in smoking, which [still causes significantly increased morbidity and mortality](https://www.ncbi.nlm.nih.gov/pmc/articles/PMC2865193/), as opposed to quitting). No harms identified in this review. In other populations, data on harms was difficult to interpret but not judged as sufficient to reduce benefit to trivial. |
|  | *NRT^b^*  Various forms  Smoking reduction (>50% of baseline or cessation)  (fu: 12+ mo)  3081 (8 RCTs) | 7/10  (critical) | 5.0/10 (important) | RR 1.75 (1.44 to 2.13) | | 81 per 1000 | | **60 more per 1000** (from 35 more to 91 more) | Small but important benefit | Small but important benefit | Small but important benefit | Point estimate and CI suggests small benefit given that those reducing by 50% are still continuing to smoke (reduction in smoking [still causes significantly increased morbidity and mortality](https://www.ncbi.nlm.nih.gov/pmc/articles/PMC2865193/), as opposed to quitting), that the data here likely includes those who quit completely (i.e., number of reducers is likely smaller than indicated), and indirect evidence of trivial to small harms seen in other populations. |

^a^ **Intervention:** Initial advice intervention aimed at encouraging reduction. Participants also advised to quit; those who agreed (i.e., set quit date) received cessation intervention. Participants offered choice of NRT gum or patch (dosage and duration not specified). No co-interventions provided. **Control:** Assessment calls only. No co-interventions provided.

^b^ **NRT:** Intervention used to assist smoking reduction. Type of NRT: Inhaler (n=2), gum (n=4), choice of NRT type (n=2). Inhaler: Participants in one trial used 6 to 12 cartridges daily for 4 months and were instructed to decrease thereafter (usage allowed up to 18 months). The second study provided 10 mg ad lib with recommendations to use 6 to 12 cartridges daily for up to 12 months (cessation encouraged after 6 months). NRT gum: Two trials provided either 2mg or 4mg gum according to dependence for 4 or 12 months, one trial provided only 4mg gum (6 to 24 pieces/day) for up to 12 months, and remaining trial provided gum for up to 9 months (dose not specified). Choice of NRT: One trial offered choice of 4mg gum (up to 24 pieces per day) or 10mg inhaler (6 to 12 cartridges) for up to 6 months with an additional 3 months of tapering. The second trial provided choice of patch, 4mg gum, inhaler or combination for 6 months. Behavioural co-intervention provided in most trials. **Placebo:** Behavioural co-intervention provided in most trials.

| NRT versus placebo or usual care in smokers with current or past depression | | | | | | | | | | |
| --- | --- | --- | --- | --- | --- | --- | --- | --- | --- | --- |
| **Outcome(s)** | | **Median Patient rating** | **WG rating** | **Relative effect (95%CI)** | **Baseline (control) risk** | **Absolute difference with intervention** | **Assessment of absolute difference** | | | |
|  |  |  |  |  |  |  | **Point estimate** | **Lower 95% CI** | **Upper 95%CI** | **Comments on judgment** |
| 1 | *NRT gum in those with current depression^a^*  Smoking cessation (fu: 12 mo)  196 (1 RCT) | 8/10 (critical) | 8.8/10 (critical) | RR 2.64 (0.93 to 7.45) | 57 per 1,000 | **94 more per 1,000** (from 4 fewer to 369 more) | Large benefit | Little to no difference | Large benefit | Point estimate and upper CI represent large benefits when considering the health benefits of quitting baseline risk, challenging population, and intervention harms that appear trivial to small. Lower CI suggests little to no difference (4 fewer quitters per 1000). No harms identified in this review. In other populations, data on harms was difficult to interpret but not judged as sufficient to reduce to moderate benefit due to harms associated with continuing to smoke. |
|  | *NRT gum in those with past depression^b^*  Smoking cessation (fu: 6+ mo)  432 (3 RCT) | 8/10 (critical) | 8.8/10 (critical) | RR 1.17 (0.85 to 1.60) | 250 per 1,000 | **42 more per 1,000** (from 38 fewer  to 150 more) | Moderate benefit | Moderate harm | Large benefit | Point estimate is judged as moderate benefit when considering the health benefits of quitting, challenging population, and few identified harms. Lower CI suggests moderate harm due to harms of continuing to smoke (fewer patients quitting). Upper CI suggests large benefit. No harms identified in this review. In other populations, data on harms was difficult to interpret but not judged as sufficient to reduce point estimate to small but important benefit due to harms associated with continuing to smoke. |

^a^ **NRT gum:** 2 or 4 mg with recommendation of 9-15 pieces per day for 2 months followed by weaning. **Control:** Placebo. **Behavioural co-intervention** provided to both study arms.

^b^ **NRT:** One study examined NRT gum and another NRT patch. The third study examined NRT patch, lozenge and patch plus lozenge (arms entered separately in meta-analysis). NRT gum: 2 mg for 8 weeks and tapering to week 11; NRT patch (both trials): 21, 14, and 7 mg titrated down during 8 weeks after quit date; NRT lozenge: 2 or 4 mg according to dependence for 12 weeks. **Control:** Placebo. **NRT and Placebo**: All studies provided a behavioural co-intervention to both arms; one four arm trial also provided bupropion or placebo tablets (study reports two comparisons entered separately in meta-analysis: (1) bupropion plus nicotine patch versus bupropion plus placebo patch, (2) placebo tablet plus nicotine patch versus placebo tablet plus placebo patch). In one study, one of the behavioural co-interventions provided was mood management; proportion of participants receiving this co-intervention is unclear.

| NRT patch versus placebo patch in smokers with schizophrenia or schizoaffective disorder | | | | | | | | | | |
| --- | --- | --- | --- | --- | --- | --- | --- | --- | --- | --- |
| **Outcome(s)** | | **Median Patient rating** | **WG rating** | **Relative effect (95%CI)** | **Baseline (control) risk** | **Absolute difference with intervention** | **Assessment of absolute difference** | | | |
|  |  |  |  |  |  |  | **Point estimate** | **Lower 95% CI** | **Upper 95%CI** | **Comments on judgment** |
| 5 | *NRT patch*  Adverse events^a^ (fu: NR)  NR (1 RCT) | 7/10 (important) | 6.5/10 (important) | One cross-over trial reported that 60% of participants experienced an increase in abnormal involuntary movement when using NRT patch. The increase was reported as statistically significant when participants were smoking and using NRT patch. | | | Small but important harm | Unable to assess | Unable to assess | Insufficient data from review to determine imprecision. Data from one cross-over trial suggests an increase in abnormal movement (twitching) but considered small but important harm due to relatively mild nature of adverse event and offset of potential benefits of quitting smoking (indirect evidence from other populations). |
| 7 | *NRT patch*  Change in mental state^b^  (fu: NR)  NR (2 RCT) | 8/10 (critical) | 5.5 /10 (important) | One trial reported no difference in psychiatric symptoms between NRT and placebo phases. In the second trial, no participant experienced a change in subjective experience or mental status. | | | Little to no difference | Unable to assess | Unable to assess | Insufficient data from review to determine imprecision. Data from one trial suggests little to no difference between groups but is difficult to interpret; a second trial reported no changes in mental status in any participant. |

^a^ Timing of outcome assessment is unclear/not reported. Treatment duration was 32 hours (22 mg/day). **Control:** placebo. No co-interventions provided to either arm.

^b^ Timing of outcome assessment unclear/NR. Treatment duration was 7 hours in one trial (8mg) and 32 hours (22 mg/day) in the second. **Control:** placebo. No co-interventions provided to either arm. **Outcome measurement:** One trial measured effects on mental state using the Brief Psychiatric Rating Scale (BPRS), the Hamilton Depression Rating Scale (HAM-D), and the Scale for the Assessment of Negative Symptoms (SANS). Review authors indicate effects on mental state were not measured in the second trial; this outcome appears to have been ascertained via observation.

# Cytisine

| Cytisine versus placebo in a general/mixed population of smokers | | | | | | | | | | |
| --- | --- | --- | --- | --- | --- | --- | --- | --- | --- | --- |
| **Outcome(s)**  **(Follow up (fu): time)**  **# of participants (# studies)** | | **Median Patient rating** | **WG rating** | **Relative effect (95%CI)** | **Baseline (control) risk** | **Absolute difference with intervention** | **Assessment of absolute difference** | | | |
|  |  |  |  |  |  |  | **Point estimate** | **Lower 95% CI** | **Upper 95%CI** | **Comments on judgment** |
| 1 | Smoking cessation^a^  (fu: 2 yrs)  1214 (1 RCT) | 8/10  (critical) | 8.8/10  (critical) | RR 1.61 (1.24 to 2.08) | 130 per 1000 | **79 more per 1000**  (from 31 more to 141 more) | Moderate benefit | Small but important benefit | Large benefit | Increase in quit rate moderate when considering health benefits of quitting, baseline risk, and lack of harms. Lower CI suggests small but important benefit and upper CI suggests large benefit based on same factors. Data on adverse events suggests trivial (little to no difference) harms. |
| 5 | Adverse events^b^  (fu: NR)  NR (3 RCTs) | 7/10 (important) | 6.5/10  (important) | Adverse events largely similar between groups. Similar rates of mild adverse events (nausea, restlessness, insomnia, irritability) in abstinent smokers at 4 weeks in one study between groups (23.4% vs 20%); longer term information not reported. A total of 10 events (e.g., dyspepsia, nausea, and headache) from four people in each group from a second study. The third study reported higher rates of gastrointestinal disorders with Cytisine (13.8% vs 8.1%, p=0.02). | | | Little to no difference | Unable to asses | Unable to asses | Insufficient data from review to determine imprecision. Data from included studies suggest little to no difference between groups but is difficult to interpret. May be some increase in relatively mild harms such as GI disorders which could be important to patients, but still considered trivial given potential benefit on smoking cessation. |

^a^ **Intervention:** 1.5 mg tablets (variable per day doses) for 25-day period with behavioural support kept to a minimum. **Control:** Placebo. Study-level description did not report co-interventions.

^b^ **Intervention:** 1.5 mg tables (varied per-day doses across studies) for 20- or 25-day period, with or without minimal behavioural support. **Control:** Placebo. **Co-interventions (both groups)**: Behavioural provided in most studies (total n=3 trials), inclusive of counselling and support.

| Cytisine versus placebo in smokers motivated/wishing to quit | | | | | | | | | | |
| --- | --- | --- | --- | --- | --- | --- | --- | --- | --- | --- |
| **Outcome(s)**  **(Follow up (fu): time)**  **# of participants (# studies)** | | **Median Patient rating** | **WG rating** | **Relative effect (95%CI)** | **Baseline (control) risk** | **Absolute difference with intervention** | **Assessment of absolute difference** | | | |
|  |  |  |  |  |  |  | **Point estimate** | **Lower 95% CI** | **Upper 95%CI** | **Comments on judgment** |
| 1 | Smoking cessation  (fu: 6+ mo)  937 (2 RCTs) | 8/10  (critical) | 8.8/10  (critical) | RR 3.98 (2.01 to 7.87) | 21 per 1000 | **64 more per 1000**  (from 22 more to 147 more) | Moderate benefit | Small but important benefit | Large benefit | Increase in quit rate moderate when considering health benefits of quitting, and baseline risk. Lower CI suggests small but important benefit and upper CI suggests large benefit based on same rationale. No information on harms in this review. In a mixed population of smokers, data on adverse events suggests trivial (little to no difference) harms. |

^a^ **Intervention:** 1.5 mg tablets (variable per day doses) for 25-day period with behavioural support kept to a minimum. **Control:** Placebo. **Co-interventions (both groups):** Authors state that behavioural support kept to a minimum, but counselling/support provided in both studies.

Varenicline

| Varenicline versus Placebo In general/mixed population of smokers | | | | | | | | | | |
| --- | --- | --- | --- | --- | --- | --- | --- | --- | --- | --- |
| **Outcome(s)**  **(Follow up (fu): time)**  **# of participants (# studies)** | | **Median Patient rating** | **WG rating** | **Relative effect (95%CI)** | **Baseline (control) risk** | **Absolute difference with intervention** | **Assessment of absolute difference** | | | |
|  |  |  |  |  |  |  | **Point estimate** | **Lower 95% CI is** | **Upper 95%CI** | **Comments on judgment** |
| 1 | Smoking cessation (6 mo f/u)^a^  1mg 2x per day  12304 (25 RCTs) | 8/10  (critical) | 8.8/10  (critical) | RR 2.25 (2.08 to 2.44) | 124 per 1000 | **156 more per 1000** (from 134 more to 179 more) | Large benefit | Large benefit | Large benefit | Increase in quit rate (point estimate and CI) is very large relative to baseline (12.4% quit rate in the control group and 28.5% quit rate in the intervention group) and considering the health benefits of quitting. Some harms are identified below but not judged as sufficient to reduce to moderate benefit due to harms associated with continuing to smoke. |
|  | Smoking cessation (6+ mo f/u)^a^  1mg 2x per day  12625 (27 RCTs) | 8/10  (critical) | 8.8/10  (critical) | RR 2.24 (2.06 to 2.43) | 111 per 1000 | **138 more per 1000** (from 118 more to 159 more) | Large benefit | Large benefit | Large benefit | Increase in quit rate (point estimate and CI) is very large relative to baseline (11.1% quit rate in the control group and 24.9% quit rate in the intervention group) and considering health benefits of quitting. Some harms are identified below but not judged as sufficient to reduce to moderate benefit due to harms associated with continuing to smoke. |
|  | Smoking cessation (Long term varenicline use (6-12 mo)^b^  (fu: 6-12 mo)  1mg 2x per day  2170 (4 studies) | 8/10  (critical) | 8.8/10  (critical) | RR 3.64 (2.81 to 4.72) | 67 per 1000 | **177 more per 1000**  (from 121 more to 249 more) | Large benefit | Large benefit | Large benefit | Increase in quit rate (point estimate and CI) is very large relative to baseline (6.7% quit rate in the control group and 24.4% quit rate in the intervention group) and considering health benefits of quitting. Some harms are identified below but not judged as sufficient to reduce to moderate benefit due to harms associated with continuing to smoke. Intervention is more involved for patients in this case due to long-term use of varenicline, but have not judged this to be sufficient to change judgements on effect size. |
|  | Smoking cessation (low dose varenicline)^c^  (fu: 12 mo)  mean: 1.35 mg/d  1266 (4 RCTs) | 8/10  (critical) | 8.8/10  (critical) | RR 2.08 (1.56 to 2.78) | 102 per 1000 | **111 more per 1000** (from 57 more to 182 more) | Large benefit | Moderate benefit | Large benefit | Increase in quit rate (point estimate and upper CI) is very large relative to baseline (10.2% quit rate in the control group and 21.3% quit rate in the intervention group) and considering health benefit of quitting. Lower CI suggest a moderate benefit considering the same factors. Some harms are identified below but not judged as sufficient to reduce to moderate benefit due to harms associated with continuing to smoke. Lower dose may [attenuate some harms such as nausea](https://www.pfizer.ca/sites/default/files/201902/Champix_PM_221214_22Jan2019_EN.pdf). |
|  | Smoking cessation (variable dosing: option to reduce dosing if side effects occurred)^d^  (fu: 6-12 mo)  1789 (6 RCTs) | 8/10  (critical) | 8.8/10  (critical) | RR 2.29 (1.81 to 2.89) | 97 per 1000 | **125 more per 1000** (from 78 more to 183 more) | Large benefit | Moderate benefit | Large benefit | Increase in quit rate is very large relative to baseline (9.7% quit rate in the control group and 22.2% quit rate in the intervention group) and considering health benefits of quitting. Lower CI suggest a moderate benefit considering baseline risk. Some harms are identified below but not judged as sufficient to reduce to moderate benefit due to harms associated with continuing to smoke. |
| 5. Adverse events | | | | | | | | | | |
| 5.1 | Nausea  (fu: range of times)^e^  1mg 2x per day  14963 (32 RCTs) | 7/10 (important) | 6.5/10 (important) | RR 3.27 (3.00 to 3.55) | 85 per 1000 | **192 more per 1000** (from 169 more to 216 more) | Small but important harm | Small but important harm | Small but important harm | Nausea increased to important extent (almost 30% of individuals getting nausea with intervention) which could have impact on adherence or discontinuation, but considered small due to relatively mild nature of adverse event, which may be mitigated by taking on a full stomach with water [or reducing dosage](https://www.pfizer.ca/sites/default/files/201902/Champix_PM_221214_22Jan2019_EN.pdf), and offset of large benefits from quitting smoking. |
| 5.2 | Insomnia  (fu: range of times) ^e^  1mg 2x per day  14447 (29 RCTs) | 7/10 (important) | 6.5/10 (important) | RR 1.49 (1.35 to 1.65) | 83 per 1000 | **41 more per 1000** (from 29 more to 54 more) | Small but important harm | Small but important harm | Small but important harm | Increase in insomnia considered small but important when taking into account nature of the adverse event, baseline risk, and offset of large benefits from quitting smoking. |
| 5.3 | Abnormal dreams  (fu: range of times) ^e^  1mg 2x per day  13682 (26 RCTs) | 7/10 (important) | 6.5/10 (important) | RR 2.12 (1.88 to 2.38) | 57 per 1000 | **64 more per 1000** (from 50 more to 79 more) | Small but important harm | Small but important harm | Small but important harm | Increase in abnormal dreams outcome considered small when taking into account nature of the adverse event, baseline risk and potential benefits of quitting smoking. Harm may also be mitigated by [altering timing of dosage in the evening](https://www.uptodate.com/contents/pharmacotherapy-for-smoking-cessation-in-adults#H3923734249). |
| 5.4 | Headache  (fu: range of times) ^e^  1mg 2x per day  13835 (25 studies) | 7/10 (important) | 6.5/10 (important) | RR 1.17 (1.07 to 1.29) | 102 per 1000 | **17 more per**  **1000** (from 7 more to 30 more) | Little to no difference | Little to no difference | Little to no difference | When taking into account potential benefits, nature of adverse effect and baseline risk (10% in control group, increased 2-3% with intervention), increase in headaches considered trivial (little to no difference), particularly due to the potential benefits of quitting smoking. |
| 5.5 | Serious adverse events (1 or more)  (fu: range of times) ^e^  1mg 2x per day  15370 (29 RCTs) | 7/10 (important) | 6.5/10 (important) | RR 1.25 (1.04 to 1.49) | 27 per 1000 | **7 more per 1000** (from 1 more to 13 more) | Small but important harm | Little to no difference | Small but important harm | Considering severity of the outcome, potential offset of smoking cessation benefits, and baseline risk, point estimate and upper CI suggest small but important harm. Lower CI suggests little no difference. |
|  | Serious adverse events (1 or more during or immediately after treatment) ^e^  (fu: range of times)  1mg 2x per day  15000 (26 RCTs) | 7/10 (important) | 6.5/10 (important) | RR 1.25 (1.02 to 1.52) | 23 per 1000 | **6 more per 1000** (from 0 fewer to 12 more) | Small but important harm | Little to no difference | Small but important harm | Considering severity of the outcome, potential offset of smoking cessation benefits, and baseline risk, point estimate and upper CI suggest small but important harm. Lower CI suggests little no difference. |
|  | Serious adverse events (Cardiac, including death) ^e^  (fu: range of times)  1mg 2x per day  8587 (21 RCTs) | 7/10 (important) | 6.5/10 (important) | RR 1.36 (0.91 to 2.04) | 9 per 1000 | **3 more per 1000** (from 1 fewer to 9 more) | Little to no difference | Little to no difference | Small but important harm | Considering severity of the outcome, potential offset of smoking cessation benefits, and baseline risk, upper CI suggest small but important harm. Point estimate and lower CI suggests little no difference. |
| 5.6 | Discontinuation  (fu: range of times)^f^  1mg 2x per day  NR (4RCTs) | 7/10 (important) | 6.5 (important) | Across three studies, treatment discontinuation ranged from 9.5% to 28% with varenicline and from 8% to 10% in the placebo group.  In the fourth study, where study discontinuation was assessed during the 12-week varenicline open label phase, 32% exited the study because of discontinuation, non-adherence to protocol, and relapse. | | | Small but important harm  to  Moderate harm | Unable to assess | Unable to assess | Small differences in discontinuation could be important if it means individual returns to smoking, given the associated harm. No 95% CI available, but ranges reported in studies include small to moderate harms (e.g., if ~30% end up discontinuing) based on this rationale. |
| 7 | Change in mental state (Neuropsychiatric events: depression, suicidal ideation, not causing death) ^e^  (fu: range of times)  8955 (23 RCTs) | 8/10 (critical) | 5.5/10 (important) | RR 0.82 (0.57 to 1.19) | 11 per 1000 | **2 fewer per 1000** (from 5 fewer to 2 more) | Little to no difference | Little to no difference | Little to no difference | Considering severity of the outcome, and baseline risk, point estimate and CIs are considered little to no difference due to the offset of smoking cessation benefits. |
|  | Depression  (fu: range of times) ^e^  16189 (36 studies) | 7/10 (important) | 6.5/10 (important) | RR 0.94 (0.77 to 1.14) | 24 per 1000 | **1 fewer per 1000** (from 6 fewer to 3 more) | Little to no difference | Little to no difference | Little to no difference | Considering severity of the outcome, and baseline risk, point estimate and CIs are considered little to no difference due to the offset of smoking cessation benefits. |
|  | Suicidal ideation  (fu: range of times) ^e^ | 7/10 (important) | 6.5/10 (important) | RR 0.68 (0.43 to 1.07) | 7 per 1000 | **2 fewer per 1000** (from 4 fewer to 0 fewer) | Little to no difference | Little to no difference | Little to no difference | Considering severity of the outcome, and baseline risk, point estimate and CIs are considered little to no difference due to the offset of smoking cessation benefits. |

^a^ **Intervention:** Varenicline 1 mg twice daily for 12 weeks except two studies (8 week). **Control:** Placebo. **Co-interventions (both groups):** Behavioural provided in most studies, inclusive of counselling, (telephone) support, self-help, advice. For the longer analysis of 6+ months follow-up, 2.7% of participants across studies allocated to varenicline and 3.4% in the placebo group received specialized counselling (CBT, relapse prevention, MI). For the analysis at 6 months, 1.3% and 1.8% in the varenicline and placebo groups, respectively, received specialized counselling (MI). For both analyses, few studies were unclear/not reported for co-interventions.

^b^ **Intervention:** Extended varenicline treatment at 1 mg twice daily for 6-12 months. **Control:** Placebo. **Co-interventions (both groups):** Behavioural provided in most studies, inclusive of counselling, self-help, advice, and support. One study unclear/not reported.

^c^ **Intervention:** half dosage (1mg/d) in three studies; one at participants’ discretion (0.5 to 2.0 mg/d) but mean modal varenicline dose 1.35 mg/d and placebo 1.63 mg/d. **Control:** Placebo. **Co-interventions (both groups):** Behavioural for all studies, inclusive of counselling and self-help.

^d^ **Intervention:** Option to reduce varenicline dosage to moderate side effects, at discretion. **Control:** Placebo. **Co-interventions (both groups):** Behavioural for all studies, inclusive of counselling, (telephone) support, and self-help. 9.8% participants in the varenicline group and 11.7% in the placebo group received specialized counselling (MI).

^e^ **Intervention:** Most studies 1 mg twice daily for 12 weeks. **Control:** Placebo. **Co-interventions (both groups):** Behavioural co-interventions common.

^f^ **Intervention:** Half of studies 1 mg twice daily for 40 wk. **Control:** Placebo. **Co-interventions (both groups):** All behavioural, inclusive of counselling and self-help; 25% specialized behavioural counselling.

| Varenicline versus Placebo in smokers motivated to quit | | | | | | | | | | |
| --- | --- | --- | --- | --- | --- | --- | --- | --- | --- | --- |
| **Outcome(s)**  **(Follow up (fu): time)**  **# of participants (# studies)** | | **Median Patient rating** | **Mean WG rating** | **Relative effect (95%CI)** | **Baseline (control) risk** | **Absolute difference with intervention** | **Assessment of absolute difference** | | | |
|  |  |  |  |  |  |  | **Point estimate** | **Lower 95% CI** | **Upper 95%CI** | **Comments on judgment** |
| 1 | *In smokers reducing to quit^a^*  Smoking cessation (fu: 12 mo)  1510 (1 RCTs) | 8/10  (critical) | 8.8/10  (critical) | RR 3.99 (2.93 to 5.44) | 60 per 1000 | **179 more per 1000** (from 116 more to 266 more) | Large benefit | Large benefit | Large benefit | Increase in quit rate (point estimate and CI) is very large relative to baseline (6.0% quit rate in the control group and 23.9% quit rate in the intervention group) and considering the health benefits of quitting. Some harms are identified below and elsewhere but not judged as sufficient to reduce to moderate benefit due to harms associated with continuing to smoke/benefits of quitting. |
|  | *In smokers who failed to quit previously but motivated/wishing to try again^b^*  Smoking cessation (fu: 12 mo)  494 (1 RCT) | 8/10  (critical) | 8.8/10  (critical) | RR 6.15 (2.98 to 12.70) | 33 per 1000 | **168 more per 1000** (from 65 more to 382 more) | Large benefit | Moderate benefit | Large benefit | Increase in quit rate (point estimate and upper CI) is very large relative to baseline (3.3% quit rate in the control group and 20.1% quit rate in the intervention group) and considering the health benefits of quitting. Lower CI is moderate benefit considering baseline risk and potential harms. Some harms are identified below and elsewhere but not judged as sufficient to reduce point estimate/upper CI to moderate benefit due to harms associated with continuing to smoke/benefits of quitting. |
| 6 | Weight gain  (fu: end of treatment)^c^  2mg/day  2008 (11 RCTs) | 6.1/10 (important) | 5/10 (important) | - | The mean weight gain in abstinent smokers ranged from 1.38-3.80 kg | MD 0.41 kg lower (0.63 lower to 0.19 lower) | Little to no difference | Little to no difference | Little to no difference | Abstinent smokers gained up to 3.8kg, and this may be reduced by ~0.5 kg with varenicline. Point estimate, lower CI, or upper CI are considered trivial (little to no difference) weight change and may be due to daily fluctuations |
|  | Weight gain  (fu: 12 mo)  2mg/day  151 (2 RCT) | 6.1/10 (important) | 5/10 (important) | - | The mean weight gain in abstinent smokers ranged from 3.9-5.2 kg | MD 1.11 kg  higher (0.75 lower to 2.98 kg higher) | Little to no difference | Little to no difference | Little to no difference | Abstinent smokers gained up to 5.2 kg, and this may be increased by ~1.1 kg with varenicline. Point estimate and CI are considered trivial (little to no difference) weight change and may be due to daily fluctuations. Upper CI could be important difference in weight gain (3kg of additional weight gain compared with placebo), considering patient importance of outcome, however, we judge this as trivial (little to no difference) due to the potential benefits on smoking cessation and harms of continuing to smoke. |
|  | Weight gain  (fu: 6 mo)^d^  2mg/day  105 (1 RCT) | 6.1/10 (important) | 5/10 (important) | - | The mean weight gain in abstinent smokers was 1.66 kg | MD 0.41 kg higher (0.79 lower to  1.61 kg higher) | Little to no difference | Little to no difference | Little to no difference | Abstinent smokers gained up to 1.66 kg, and this may be reduced by ~0.4 kg with varenicline. Point estimate or lower CI are considered trivial (little to no difference) weight change and may be due to daily fluctuations. Upper CI is could be an important difference in weight gain (1.6kg of additional weight gain compared with placebo) considering patient importance of outcome however, we judge this as trivial (little to no difference) due to the potential benefits on smoking cessation and harms of continuing to smoke. |
|  | Weight gain  (fu: end of treatment)^d^  1 mg/day  254 (3 RCTs) | 6.1/10 (important) | 5/10 (important) | - | The mean weight gain in abstinent smokers ranged from 1.48-4.0 kg | MD 0.12 kg lower (0.68 lower to 0.43 higher) | Little to no difference | Little to no difference | Little to no difference | Abstinent smokers gained up to 4.0 kg, and this may be reduced by ~0.1 kg with varenicline. Point estimate, lower CI, or upper CI are considered trivial (little to no difference) weight change and could be due to daily fluctuations. |

^a^ **Varenicline:** Treatment course 6 months. Participant not willing to quit abruptly but were interested in quitting in the following three months. **Control:** placebo. **Co-interventions (both groups):** Behavioural (counselling and self-help).

^b^ **Varenicline:** 1 mg twice daily for 12 weeks. **Control:** placebo. **Co-interventions (both groups):** Behavioural (counselling).

^c^ **Varenicline:** Treatment duration was 12 weeks in all but one trial (6 weeks). Behavioural co-intervention provided in most trials. In one 3-arm trial, participants assigned to the varenicline arm also received placebo to control for the effect of the other tested intervention. **Placebo:** Behavioural co-intervention provided in most trials. In one 3-arm trial, participants assigned to placebo varenicline also received additional placebo (i.e., placebo bupropion) along with a behavioural support co-intervention.

^d^ **Varenicline:** Treatment duration was 12 weeks. **Control:** placebo. **Behavioural co-intervention** provided to both varenicline and placebo participants in all trials.

| Varenicline^a^ versus Placebo in smokers NOT motivated/wishing to quit | | | | | | | | | | |
| --- | --- | --- | --- | --- | --- | --- | --- | --- | --- | --- |
| **Outcome(s)**  **(Follow up (fu): time)**  **# of participants (# RCTs)** | | **Median Patient rating** | **Mean WG rating** | **Relative effect (95%CI)** | **Baseline (control) risk** | **Absolute difference with intervention** | **Assessment of absolute difference** | | | |
|  |  |  |  |  |  |  | **Point estimate** | **Lower 95% CI** | **Upper 95%CI** | **Comments on judgment** |
| 1 | Smoking cessation (fu: 6 mo)  218 (1 RCTs) | 8/10  (critical) | 8.8/10  (critical) | RR 1.95 (0.86 to 4.40) | 72 per 1000 | **68 more per 1000** (from 20 fewer to 245 more) | Moderate benefit | Moderate harm | Large benefit | Point estimate suggests moderate increase in quit rate, given baseline risk and considering benefits of quitting (7.2% quit rate in the control group and 14.0% quit rate in the intervention group). Very few harms identified to offset this effect, although harms identified for general population of smokers are likely relevant here as well, but not judged as sufficient to change the judgment for the point estimate. Lower CI is a moderate harm, considering the negative impacts of continuing to smoke and potential harms of intervention. Upper CI would be considered a large benefit, given the baseline risk and health benefits of quitting, even after considering potential harms. |
| 5 | Stopping medication  (fu: NR)  NR (1 RCT) | 7/10 (important) | 6.5/10 (important) | No significant difference between groups (vareniciline 12%, placebo 10%) | | | Little to no difference | Unable to assess | Unable to assess | Insufficient data from review to determine imprecision. Data suggests little to no difference between groups but is difficult to interpret. |

^a^ **Varenicline:** Objective of trial was to evaluate effect of varenicline on inducing quit attempts in smokers not planning to quit. 2 mg/day for 2 to 8 weeks. **Control:** Placebo. Behavioural co-intervention provided to both groups.

| Varenicline^a^ versus placebo in smokers with depression and motivated/willing to quit | | | | | | | | | | |
| --- | --- | --- | --- | --- | --- | --- | --- | --- | --- | --- |
| **Outcome(s)**  **(Follow up (fu): time)**  **# of participants (# studies)** | | **Median Patient rating** | **WG rating** | **Relative effect (95%CI)** | **Baseline (control) risk** | **Absolute difference with intervention** | **Assessment of absolute difference** | | | |
|  |  |  |  |  |  |  | **Point estimate** | **Lower 95% CI** | **Upper 95%CI** | **Comments on judgment** |
| 1 | Smoking cessation  (fu: 12 mo)  1mg 2x per day  523 (1 RCT) | 8/10  (critical) | 8.8/10  (critical) | RR 1.97 (1.28 to 3.01) | 104 per 1000 | **101 more per 1000** (from 29 more to 209 more) | Large benefit | Small but important benefit | Large benefit | Increase in quit rate is large given baseline (10.4% quit rate in the control group and 20.5% quit rate in the intervention group) and health benefits of quitting. While no harms were identified in this population, some were harms identified in the general population likely apply here as well (e.g., nausea, headaches). These are not judged as sufficient to reduce the point estimate or upper CI to moderate benefit due to harms associated with continuing to smoke. Lower CI suggest a small but important benefit given benefits of quitting and potential harms of the intervention. |

^a^ **Intervention:** 1 mg twice daily for 12 weeks. **Control:** placebo. **Co-interventions (both groups):** Behavioural (counselling, (telephone) support).

| Varenicline versus placebo in smokers with schizophrenia, schizoaffective, bipolar, or other psychiatric disorder | | | | | | | | | | |
| --- | --- | --- | --- | --- | --- | --- | --- | --- | --- | --- |
| **Outcome(s)**  **(Follow up (fu): time)**  **# of participants (# studies)** | | **Median Patient rating** | **WG rating** | **Relative effect (95%CI)** | **Baseline (control) risk** | **Absolute difference with intervention** | **Assessment of absolute difference** | | | |
|  |  |  |  |  |  |  | **Point estimate** | **Lower 95% CI** | **Upper 95%CI** | **Comments on judgment** |
| 1 | Smoking cessation^a^  (fu: 6 mo)  1mg 2x per day  2332 (4 RCTs) | 8/10  (critical) | 8.8/10  (critical) | RR 2.28 (1.82 to 2.87) | 81 per 1000 | **104 more per 1000** (from 67 more to 152 more) | Large benefit | Moderate benefit | Large benefit | Increase in quit rate is large considering the baseline (8.1% quit rate in the control group and 18.5% quit rate in the intervention group) and considering health benefits of quitting. While few harms were identified in this population, there may be a small increase in adverse events, and some harms identified for general population likely apply here as well (e.g., nausea, headaches). These are not judged as sufficient to reduce the point estimate or upper CI to moderate benefit due to harms associated with continuing to smoke. Lower CI however suggest a moderate benefit given health benefits of quitting and potential harms of treatment. |
|  | Smoking cessation  (fu: 6 mo)^b^  128 (1 RCT) | 8/10  (critical) | 8.8/10  (critical) | RR 5.06 (0.67 to 38.24) | 23 per 1,000 | **94 more per 1,000** (from 8 fewer to 866 more) | Large benefit | Small but important harm | Large benefit | Increase in quit rate was large considering the baseline and considering the health benefits of quitting (2.3% quit rate in the control group and 11.7% quit rate in the intervention group). While few harms were identified in this population, there may be a small increase in adverse events, and some harms identified for general population likely apply here as well (e.g., nausea, headaches). These are not judged as sufficient to reduce the point estimate or upper CI to moderate benefit due to harms associated with continuing to smoke. Lower CI suggest a small but important harm given harms of continuing to smoke. |
| 2 | Smoking reduction (# cigarettes per day from baseline) (fu: 6 mo)^b^  NR (1 RCT) | 7/10  (critical) | 5.0/10  (important) | Among continued smokers, there was no statistically significant difference between groups in reduction of cigarettes per day from baseline. | | | Little to no difference | Unable to assess | Unable to assess | Insufficient data from review to determine imprecision. Data suggests little to no difference between groups but is difficult to interpret. |
| 5 | Adverse events  (fu: NR)^c^  NR (2 RCTs)  *All trials were aimed at smoking cessation* | 7/10 (important) | 7/10 (important) | One trial reported no suicidal ideation. This study reported exacerbation of side effects in the varenicline arm, namely, constipation, insomnia, and nausea.  In the second trial, two participants with a history of suicide attempts assigned to varenicline were hospitalized; one of the participants overdosed and had a seizure resulting in hospitalization. In total, the trial reported 13 serious adverse events occurring in 9 participants assigned to varenicline and 1 participant assigned to placebo (2 varenicline participants experienced 3 serous adverse events related to treatment). One death from accidental drowning occurred in the varenicline arm during the long-term follow-up period (off-treatment); event not related to treatment according to authors. No treatment-related adverse events occurred in placebo arm.  This second trial also reported no difference between groups regarding other adverse events including neuropsychiatric SAEs and study discontinuation. The most common adverse events occurring in the varenicline arm were nausea (23.8%), headache (10.7%), and vomiting (10.7%). | | | Small but important harm | Unable to assess | Unable to assess | Insufficient data from review to determine imprecision. Data suggests potential small but important difference between groups for serious adverse events, but is difficult to interpret. |
|  | Adverse events  (fu: NR)^d^  NR (3 RCTs)  *Data from two trials is inclusive of both smokers and non-smokers; remaining trial recruited smokers with alcohol dependence and had a small sample due to recruitment issues. All trials in this analysis were not aimed at smoking cessation* | 7/10 (important) | 7/10 (important) | In one trial recruiting participants with both smoking and alcohol dependence, one participant assigned to varenicline withdrew from the trial due to passive suicidal ideation (7 days after starting varenicline), vomiting and irritability. Remaining two trials reported that among smokers and non-smokers, no participants had suicidal ideation and no increase in suicidal ideation occurred in those assigned varenicline, respectively.  One trial reported a trend toward reduced psychosis in the varenicline arm compared to placebo; both smokers and non-smokers were included in this analysis but study authors report no difference in treatment effect related to smoking status. In the second trial of smokers and non-smokers, two participants in each study arm withdrew due to exacerbation of psychotic symptoms.  One trial reported that among smokers and non-smokers, there was no difference between varenicline and placebo groups regarding common side effects of varenicline. However, the two other trials reported higher rates of common side effects in the varenicline arm (e.g., nausea, headache, vomiting, abdominal pain). In one of these studies, one patient withdrew due to nausea and vomiting.  Given what was reported, the one trial in smokers with alcohol dependence raised concerns regarding the safety and tolerability of varenicline in schizophrenic patients. | | | Little to no difference | Unable to assess | Unable to assess | Insufficient data from review to determine imprecision. Data suggests little to no difference between groups but is difficult to interpret.  Data from one trial suggest potential important increase in common side effects such as nausea, similar to what was seen in studies in general population. |
| 7 | Change in mental state (positive, negative, and/or depression)^b,e^  (fu: end of treatment)  NR (2 RCTs)  *All trials were aimed at smoking cessation* | 8/10 (critical) | 5.5 (important) | Both trials reported on positive symptoms and one trial each on negative and depressive symptoms. There was no significant difference between groups for all symptoms. | | | Little to no difference | Unable to assess | Unable to assess | Insufficient data from review to determine imprecision. Data suggests little to no difference between groups but is difficult to interpret. |
|  | Change in mental state (positive, negative, depression, and/or general symptoms of schizophrenia)^f^  (fu: end of treatment)  NR (3 RCTs)  *All trials in this analysis were not aimed at smoking cessation* | 8/10 (critical) | 5.5 (important) | Data from one trial in this analysis is inclusive of both smokers and non-smokers; remaining trials either only recruited smokers or reported data on these outcomes for smokers only. One study had a small sample due to recruitment issues (n=10).  Of the two trials reporting on change in positive symptoms, one reported no significant changes in symptoms among smokers and the other no significant difference between groups (inclusive of smokers and non-smokers).  For negative symptoms, one trial reported no significant change in symptoms among smokers and two trials reported no significant difference between groups (smokers and non-smokers included in the analysis for one of these two studies).  One trial reported no significant depressive symptoms among smokers and non-smokers during the study while another reported no significant difference between smokers in each group.  One trial also reported no significant change in general symptoms of schizophrenia among smokers. | | | Little to no difference | Unable to assess | Unable to assess | Insufficient data from review to determine imprecision. Data suggests little to no difference between groups but is difficult to interpret. |

^a^ **Intervention:** 1 mg twice daily for 12 weeks except one study (5% evidence) with open label phase varenicline (12 wks) and varenicline vs placebo (40 wks) provided following abstinence. **Control:** Placebo. **Co-interventions (both groups):** Behavioural for all studies, inclusive of counselling, telephone support, and specialized behavioural therapy (CBT and relapse prevention, 3.4% participants in varenicline and 4.1% in placebo groups).

^b^ **Intervention:** 2 mg per day for about 12 weeks. **Control:** Placebo. **Co-interventions (both groups):** Both arms received behavioural co-intervention

^c^ **Intervention:** Both trials examined 2 mg/day of varenicline for about 12 weeks. **Control:** Placebo. **Behavioural co-intervention:** provided to both arms in both trials. In one of the trials, participants received counselling based on the American Lung Association Freedom from Smoking Program.

^d^ **Intervention:** Two trials examined 2 mg/day of varenicline for about 8 weeks; remaining trial examined 1 mg/day for the same duration. **Control:** Placebo. **Behavioural co-intervention:** One small trial (n=10) provided participants of both arms with incentive and specialized behavioural counselling co-intervention (i.e., motivational interviewing for quitting or reducing smoking and drinking). Remaining trials provided no co-interventions.

^e^ **Outcome measurement:** Effects on mental state measured using the Positive And Negative Syndrome Scale (PANSS), Scale for the Assessment of Negative Symptoms (SANS), Columbia Suicide Severity Rating Scale (C-SSRS), and Clinical Global Impression (CGI) in one trial. The other trial used the Brief Psychiatric Rating Scale (BPRS) and Calgary Depression Rating Scale (CDSS).

^f^ **Outcome measurement:** One trial measured effects on mental state using the Brief Psychiatric Rating Scale (BPRS), Scale for the Assessment of Negative Symptoms (SANS), Hamilton Depression Rating Scale (HAM-D), and Clinical Global Impression (CGI). A second trial used the Positive And Negative Syndrome Scale (PANSS), Scale for the Assessment of Negative Symptoms (SANS), Hamilton Depression Rating Scale (HAM-D), and Clinical Global Impression (CGI). Remaining trial used PANSS only.

# Bupropion

| Bupropion versus Placebo in a mixed population of smokers | | | | | | | | | | |
| --- | --- | --- | --- | --- | --- | --- | --- | --- | --- | --- |
| **Outcome(s)**  **(Follow up (fu): time)**  **# of participants (# studies)** | | **Median Patient rating** | **WG rating** | **Relative effect (95%CI)** | **Baseline (control) risk** | **Absolute difference with intervention** | **Assessment of absolute difference** | | | |
|  |  |  |  |  |  |  | **Point estimate** | **Lower 95% CI is** | **Upper 95%CI** | **Comments on judgment** |
| 7 | Change in mental state (Depression) (fu: no reported)  NR (1RCT) | 8/10 (critical) | 5.5 (important) | During treatment, most participants in both arms experienced reduction in depressive symptoms and this was sustained at follow-up. A between-group difference was observed for highly dependent smokers with greater reduction in the bupropion arm. The reduction was not sustained at follow-up. | | | Little to no difference | Unable to assess | Unable to assess | Insufficient data from review to determine imprecision. Data suggests little to no difference between groups at follow-up but is difficult to interpret. |

^a^ **Intervention:** Trial examined bupropion at 300 mg/day for 10 weeks. **Control:** Placebo. Both arms received behavioural co-intervention. **Outcome measurement** method not reported.

| Bupropion versus Placebo in smokers motivated/wishing to quit at baseline and abstinent at follow-up Review authors state that all trials included in the review examined smokers motivated to quit, however this was not explicitly stated in any of the trials included here | | | | | | | | | | |
| --- | --- | --- | --- | --- | --- | --- | --- | --- | --- | --- |
| **Outcome(s)**  **(Follow up (fu): time)**  **# of participants (# studies)** | | **Median Patient rating** | **WG rating** | **Relative effect (95%CI)** | **Baseline (control) risk** | **Absolute difference with intervention** | **Assessment of absolute difference** | | | |
|  |  |  |  |  |  |  | **Point estimate** | **Lower 95% CI is** | **Upper 95%CI** | **Comments on judgment** |
| 6 | Weight gain  (fu: end of treatment)^a^  869 (7 RCTs) | 6.1/10 (important) | 5/10 (important) | - | The mean weight gain in abstinent smokers ranged from 2.32-4.0 kg | MD 1.12 kg lower (1.47 lower to 0.77 lower) | Little to no difference | Little to no difference | Little to no difference | Abstinent smokers gained up to 4.0 kg, and this may be reduced by ~1.1 kg with bupropion. Point estimate, or CI are not considered important or clinically significant weight change and could partially be due to daily fluctuations. |
|  | Weight gain  (fu: 6 mo)^c^  218 (4 RCTs) | 6.1/10 (important) | 5/10 (important) | - | The mean weight gain in abstinent smokers ranged from 1.69-5.5 kg | MD 0.87 kg lower (2.21 lower to 0.47 higher) | Little to no difference | Little to no difference | Little to no difference | Abstinent smokers gained up to 5.5 kg, and this may be reduced by ~1 kg with bupropion. Point estimate, and CI are not considered important or clinically significant weight change and could partially be due to daily fluctuations. |
|  | Weight gain  (fu: 12 mo)^b^  252 (4 RCTs) | 6.1/10 (important) | 5/10 (important) | - | The mean weight gain in abstinent smokers ranged from 2.94-6.9 kg | MD 0.38 kg lower (2.00 lower to 1.24 higher) | Little to no difference | Little to no difference | Little to no difference | Abstinent smokers gained up to 6.9 kg, and this may be reduced by ~0.4 kg with bupropion. Point estimate and CI are not considered important or clinically significant weight gain and could be partially due to daily fluctuations. |

^a^ **Bupropion:** 300 mg/day for 7 to 12 weeks. Behavioural co-intervention provided in all trials. In two 3-arm trials, participants assigned to the bupropion arm also received placebo to control for the effect of the other tested intervention. 5.1% of participants received a specialized behavioural counselling co-intervention (i.e., cognitive behavioural therapy). **Placebo:** Review authors indicate the comparator is placebo; however, in one trial, control is advice with follow-up rather than placebo. Behavioural co-intervention provided in all trials. In two 3-arm trials, participants assigned to placebo bupropion also received additional placebo (placebo gum in one trial and placebo varenicline in another). 9.5% of participants received a specialized behavioural counselling co-intervention (i.e., cognitive behavioural therapy).

^b^ **Bupropion:** 300 mg/day for 7 weeks. Behavioural co-intervention provided in all trials. 3.9% of participants received a specialized behavioural counselling co-intervention (i.e., cognitive behavioural therapy). **Placebo:** Review authors indicate the comparator is placebo; however, in one trial, control is advice with follow-up rather than placebo. Behavioural co-intervention provided in all trials. 20.6% of participants received a specialized behavioural counselling co-intervention (i.e., cognitive behavioural therapy).

^c^ **Bupropion:** 300 mg/day for 7 to 12 weeks. Behavioural co-intervention provided in all trials. 21.8% of participants received specialized behavioral counselling interventions (CBT with or without relapse prevention counselling). **Placebo:** Behavioural co-intervention provided in all trials. 46.3% of participants received specialized behavioral counselling interventions (CBT with or without relapse prevention counselling).

| Bupropion^a^ versus Placebo in smokers who are NOT motivated/ wishing to quit | | | | | | | | | | |
| --- | --- | --- | --- | --- | --- | --- | --- | --- | --- | --- |
| **Outcome(s)**  **(Follow up (fu): time)**  **# of participants (# studies)** | | **Median Patient rating** | **WG rating** | **Relative effect (95%CI)** | **Baseline (control) risk** | **Absolute difference with intervention** | **Assessment of absolute difference** | | | |
|  |  |  |  |  |  |  | **Point estimate** | **Lower 95% CI is** | **Upper 95%CI** | **Comments on judgment** |
| 1 | Smoking cessation (fu: 6 mo)  594 (1 RCT) | 8/10  (critical) | 8.8/10  (critical) | RR 1.27 (0.67 to 2.40) | 54 per 1000 | **14 more per 1,000** (from 18 fewer to 75 more) | Small but important benefit | Small but important harm | Moderate benefit | Considering benefits of quitting smoking, potential small to large increase in adverse events, and baseline risk, point estimate is small benefit given the baseline risk, however CI includes small harm and moderate benefit. |
| 2 | Smoking reduction (>50% of baseline or cessation)  (fu: 12 mo)  594 (1 RCT) | 7/10  (critical) | 5.0/10  (important) | RR 1.01 (0.62 to 1.67) | 94 per 1000 | **1 more per 1000** (from 36 fewer to 63 more) | Little to no difference | Small but important harm | Small but important benefit | Considering potential small to large increase in adverse events, point estimate (1 more individual per 1000 reducing smoking by 50%) is judged to be little to no difference. CI includes small harm (fewer patients quitting) and small benefit, considering the baseline risk, and taking into account harms associated with smoking/benefits of quitting. |
|  | Smoking reduction (in cotinine by >50% of baseline)  (fu: 12 mo)  327 (1 RCT) | 7/10  (critical) | 5.0/10  (important) | RR 0.43 (0.12 to 1.58) | 46 per 1000 | **26 fewer per 1000** (from 40 fewer to 27 more) | Small but important harm | Small but important harm | Small but important benefit | Point estimate and lower CI suggest small but important harm with fewer patients reducing their smoking than in the control group. Upper CI could be a small benefit considering the control group risk of reducing smoking, and given that light smoking [still causes significantly increased morbidity and mortality](https://www.ncbi.nlm.nih.gov/pmc/articles/PMC2865193/), as opposed to quitting. |
|  | Smoking reduction (cotinine) (fu: 12 mo)  327 (1 RCT) | 7/10  (critical) | 5.0/10  (important) | No significant difference between groups in mean urinary cotinine from baseline at 12-month follow-up (mean decrease: bupropion 82 ng/mL vs control 28 ng/mL, p=0.25). | | | Little to no difference | Unable to asses | Unable to asses | Insufficient data from review to determine imprecision. Data suggests little to no difference between groups at follow-up but is difficult to interpret. |
|  | Smoking reduction (# cigarettes per day  (fu: 12 mo)  594 (1 RCT) | 7/10  (critical) | 5.0/10  (important) | No significant difference between groups | | | Little to no difference | Unable to asses | Unable to asses | Insufficient data from review to determine imprecision. Data suggests little to no difference between groups at follow-up but is difficult to interpret. |
| 5 | Serious adverse events  594 (1 RCT) | 7/10 (important) | 7/10 (important) | RR 2.70 (0.72 to 10.09) | 10 per 1,000 | **17 more per 1,000** (from 3 fewer to 91 more) | Small but important harm | Little to no difference | Large harm | When taking into account potential benefits from cessation, potential severity of adverse effect, and baseline risk, point estimate considered a small increase (harm), but CI ranges from little to no difference to large harm (particularly relative to the baseline risk). The study authors also note that only one serious event was thought to be related to bupropion treatment. |

^a^ **Intervention:** Bupropion 300 mg/day for 26 weeks. **Control:** Placebo. Behavioural co-intervention provided to both trial arms.

| Bupropion versus placebo in smokers with current or past depression | | | | | | | | | | |
| --- | --- | --- | --- | --- | --- | --- | --- | --- | --- | --- |
| **Outcome(s)**  **(Follow up (fu): time)**  **# of participants (# studies)** | | **Median Patient rating** | **WG rating** | **Relative effect (95%CI)** | **Baseline (control) risk** | **Absolute difference with intervention** | **Assessment of absolute difference** | | | |
|  |  |  |  |  |  |  | **Point estimate** | **Lower 95% CI** | **Upper 95%CI** | **Comments on judgment** |
| 1 | *In those with current depression^a^*  Smoking cessation (fu: 6-12 mo)  410 (5 RCTs) | 8/10  (critical) | 8.8/10  (critical) | RR 1.37 (0.83 to 2.27) | 112 per 1,000 | **41 more per 1,000** (from 19 fewer to 142 more) | Moderate benefit | Small but important harm | Large benefit | Increase in quit rate considered moderate benefit when taking into account benefits of quitting, baseline risk, potential harms of intervention, and more challenging population (11.2% quit rate in the control group and 15.2% quit rate in the intervention group). While no harms were identified in this population, there is indirect evidence of potential small to large increase in adverse events from smokers who are not motivated to quit. Judgement for upper CI is a large benefit despite potential harms due to health benefits of quitting. Lower CI suggests a small harm due to the harms associated with more people continuing to smoke. |
|  | *In those with past depression^b^*  Smoking cessation (fu: 6-12 mo)  404 (4 RCTs) | 8/10  (critical) | 8.8/10  (critical) | RR 2.04 (1.31 to 3.18) | 123 per 1,000 | **128 more per 1,000** (from 38 more to 268 more) | Large benefit | Moderate benefit | Large benefit | Increase in quit rate judged as large given the baseline risk (12.3% quit rate in the control group and 26.8% quit rate in the intervention group) and considering the benefits of quitting and more challenging population. Judgement for point estimate and upper CI remained a large benefit after considering potential harms, using evidence of potential small to large increase in adverse events from smokers who are not motivated to quit. Lower CI suggests a moderate benefit, taking into account those harms, as well as the more challenging population. |

^a^ **Bupropion:** Across trials, 300mg/day for 7 weeks to 6 months. **Control:** Placebo. **Co-interventions (both arms):** All studies provided a behavioural co-intervention to both arms; one study also provided NRT patch to both arms. Across studies, at least 48% of participants in each arm received specialized behavioural counselling (CBT or relapse prevention counselling).

^b^ **Bupropion:** Across trials, 300 mg/day for 7 to 12 weeks. All studies provide a behavioural co-intervention to participants receiving bupropion; one four arm trial also provided either NRT or placebo patch (study reports two comparisons entered separately in meta-analysis: (1) bupropion plus nicotine patch versus placebo tablet plus nicotine patch, (2) bupropion plus placebo patch versus placebo tablet plus placebo patch). 22% of participants received specialized behavioural counselling (CBT). **Placebo:** All studies provide a behavioural co-intervention to participants in this arm; one four arm trial also provided either NRT or placebo patch. 32% of participants received specialized behavioural counselling (CBT).

| Bupropion versus placebo in smokers with schizophrenia or schizoaffective disorder | | | | | | | | | | |
| --- | --- | --- | --- | --- | --- | --- | --- | --- | --- | --- |
| **Outcome(s)**  **(Follow up (fu): time)**  **# of participants (# studies)** | | **Median Patient rating** | **WG rating** | **Relative effect (95%CI)** | **Baseline (control) risk** | **Absolute difference with intervention** | **Assessment of absolute difference** | | | |
|  |  |  |  |  |  |  | **Point estimate** | **Lower 95% CI** | **Upper 95%CI** | **Comments on judgment** |
| 1 | Smoking cessation^a^  (fu: 6 mo)  214 (5RCTs) | 8/10  (critical) | 8.8/10  (critical) | RR 2.78 (1.02 to 7.58) | 37 per 1,000 | **66 more per 1,000** (from 1 more to 244 more) | Moderate benefit | Little to no difference | Large benefit | Increase in quit rate judges as moderate given the benefits of quitting, and baseline risk (3.7% quit rate in the control group and 13.2% in the intervention group), particular challenges of the population, and potential harms of intervention. Judgement for upper CI is a large benefit based on the same factors. Lower CI suggests little to no difference. Adverse events data for this population suggests little to no difference. However, there is indirect evidence from smokers without schizophrenia and not motivated to quit, of a small to large increase in serious adverse events. |
| 2 | Smoking reduction (expired CO level)^b^  (fu: 6 mo)  123 (3 RCTs) | 8/10  (critical) | 8.8/10  (critical) | - | The mean expired CO level ranged from **20-27** ppm | **MD** **5.55 ppm lower** (17.89 lower to 6.78 higher) | Little to no difference | Small but important benefit | Little to no difference | Considering adverse events, point estimate and upper CI are judged as little to no difference. While the difference could correspond to a significant reduction in cigarettes per day, see:  <https://ajph.aphapublications.org/doi/pdf/10.2105/AJPH.67.6.545>  [little impact on health would be anticipated unless this reduction ultimately leads to quitting](https://www.ncbi.nlm.nih.gov/pmc/articles/PMC2865193/).  Adverse events data for this population suggests little to no difference. However, there is indirect evidence from smokers without schizophrenia and not motivated to quit, of a small to large increase in adverse events. Judgement for lower CI remained a small benefit as it would almost guarantee that CO levels are reduced to those of a non-smoker (under 9ppm), regardless of baseline level. |
|  | Smoking reduction (# cigarettes per day from baseline)^b^  (fu: 6 mo)  104 (2 RCTs) | 7/10  (critical) | 5.0/10  (important) | - | The mean change in number of cigarettes per day from baseline ranged from **-2.9 to -6.0** | **MD** **0.4 higher** (5.72 lower to 6.53 higher) | Little to no difference | Little to no difference | Little to no difference | The mean difference does not suggest an important difference between groups (i.e., less than a half a cigarette per day more) and could be a result of daily fluctuations. The lower and upper CI also suggest little to no difference given the lack of health benefits from reducing smoking without quitting. While baseline number of cigarettes per day was not reported, the national average in Canada is ~13 cigarettes per day (<https://uwaterloo.ca/tobacco-use-canada/adult-tobacco-use/smoking-canada/cigarette-consumption>). Based on this, the lower and upper CI would each represent an almost 50% reduction. Potential harms did not change these judgements. |
| 5 | Adverse events^c^  (fu: NR)  *Smoking cessation trials*  NR (7 RCTs) | 7/10 (important) | 7/10 (important) | Review authors report various adverse events collectively.  In one study, a participant who took bupropion had a seizure; authors reported that this was likely unrelated to bupropion treatment. No seizures reported in remaining trials.  One study reported that one participant receiving bupropion (3%) and two receiving placebo (7%) experienced a psychotic breakdown; authors did not consider this related to study treatment. Another study reported recurrence of psychotic symptoms in two participants but results were not reported by study arm. One study reported no serious adverse events.  One trial reported higher rates of dry mouth in the bupropion arm compared to placebo and another study reported that significantly more bupropion participants (also receiving NRT) experienced poor concentration, jitteriness, light-headedness, muscle stiffness and frequent nocturnal awakening. A third study reported that, compared to placebo, the bupropion arm had higher rates of insomnia, dry mouth and sweatiness. A fourth study reported an allergic reaction in one participant receiving bupropion. A fifth study reported no significant differences in major adverse events measured by the Side Effect Checklist (e.g., restlessness, insomnia, dry mouth, sedation); however, five participants on bupropion withdrew from the trial due to side effects, including rash (n=1), restlessness and increased anxiety (n=2), worsening of psychosis (n=1), and the aforementioned seizure. The sixth study reported two participant withdrawals in the bupropion arm (also receiving NRT) due to insomnia and dizziness. | | | Little to no difference | Unable to asses | Unable to asses | Insufficient data from review to determine imprecision. Data suggests little to no difference between groups but is difficult to interpret. Increase in adverse events listed (e.g., dry mouth) considered small, but interpreted as little to no difference when taking into account potential benefits of quitting smoking. |
|  | Adverse events^d^  (fu: NR)  *Smoking reduction trials*  NR (3 RCTs) | 7/10 (important) | 7/10 (important) | Two trials reported no adverse events related to bupropion. One trial reported no significant difference between groups in adverse events and no seizures or suicidal behaviour in the bupropion arm. | | | Little to no difference | Unable to asses | Unable to asses | Insufficient data from review to determine imprecision. Data suggests little to no difference between groups. |
| 7 | Change in mental state (negative symptoms, e.g., anhedonia, avolition)^e^  (fu: end of treatment)  136 (3 RCTs) | 8/10 (critical) | 5.5 (important) | - | - | SMD **0.12 lower** (0.46 lower to 0.22 higher) | Little to no difference | Moderate benefit | Small but important harm | Measured using the Positive and Negative Syndrome Scale (PANSS: <https://en.wikipedia.org/wiki/Positive_and_Negative_Syndrome_Scale>) or the Scale for the Assessment of Negative Symptoms (https://www.ncbi.nlm.nih.gov/pmc/articles/PMC5805140/).  SMD of 0.2 is generally considered to be a small effect size, while 0.5 would be moderate : <https://www.ncbi.nlm.nih.gov/pmc/articles/PMC2730804/>  Judgments have been made accordingly. Upper CI is considered a small harm despite potential offset of smoking cessation improvement due to potential severity of outcome. |
|  | Change in mental state (positive symptoms, e.g., hallucinations, delusions)^f^  (fu: end of treatment)  *Smoking cessation trials*  85 (2 RCTs) | 8/10 (critical) | 5.5 (important) | - | - | SMD **0.24 lower** (0.66 lower to 0.19 higher | Small but important benefit | Moderate benefit | Little to no difference | Measured using the Positive and Negative Syndrome Scale (PANSS: <https://en.wikipedia.org/wiki/Positive_and_Negative_Syndrome_Scale>).  SMD of 0.2 is generally considered to be a small effect size, while 0.5 would be moderate : <https://www.ncbi.nlm.nih.gov/pmc/articles/PMC2730804/>  Judgments have been made accordingly. However given potential offset of smoking cessation improvement, have considered the upper CI to be little to no difference. |
|  | Change in mental state (positive symptoms, e.g., hallucinations, delusions)^g^  (fu: end of treatment)  *Smoking reduction trials*  Unclear/NR  (3 RCTs) | 8/10 (critical) | 5.5 (important) | One study reported no worsening of positive and negative symptoms in the bupropion arm. A second study reported no significant difference between groups regarding change in positive and negative symptoms. The third study reported no increase in psychiatric symptoms in the bupropion arm. | | | Little to no difference | Unable to determine | Unable to determine | Insufficient data from review to determine imprecision. Data suggests little to no difference between groups. |
|  | Change in mental state (depression)^e^  (fu: end of treatment)  136 (3 RCTs) | 8/10 (critical) | 5.5 (important) | - | - | SMD **0.16 lower** (0.5 lower to 0.18 higher) | Small but important benefit | Moderate benefit | Little to no difference | Measured using the Beck Depression Inventory (<https://www.apa.org/pi/about/publications/caregivers/practice-settings/assessment/tools/beck-depression>) or the Hamilton Depression Rating Scale (<https://en.wikipedia.org/wiki/Hamilton_Rating_Scale_for_Depression>)  SMD of 0.2 is generally considered to be a small effect size, while 0.5 would be moderate : <https://www.ncbi.nlm.nih.gov/pmc/articles/PMC2730804/>  Judgments have been made accordingly. However given potential offset of smoking cessation improvement, have considered the upper CI to be little to no difference. |

^a^ **Bupropion:** Most trials examined 300 mg/day for about 10 or 12 weeks; one trial provided 150 mg/day for 12 weeks. Control: Placebo. Behavioural co-intervention provided in all trials. In two trials, participants also received NRT patch with or without NRT gum as a co-intervention. 72% of participants in this analysis received specialized behavioural counselling (CBT or relapse prevention). **Placebo:** Behavioural co-intervention provided in all trials. In two trials, participants also received NRT patch with or without NRT gum as a co-intervention. 73% of participants received specialized behavioural counselling (CBT or relapse prevention).

^b^ **Bupropion and Placebo**: Both trials included in the reduction in CPD analysis provided 300 mg/day for about 12 weeks. For expired CO, two trials provided 300 mg/day and one provided 150 mg/day for about 12 weeks. Behavioural co-intervention provided to both arms in all trials. In one of the trials, participants in both arms also received NRT patch and NRT gum as a co-intervention. All participants in this analysis received specialized behavioural counselling (CBT).

^c^ **Bupropion and Placebo**: Most trials provided 300 mg/day for about 4, 10, or 12 weeks; one trial provided 150 mg/day for 12 weeks. Behavioural co-intervention provided to all participants in all but one trial. In two trials, participants in both arms also received NRT patch with or without NRT gum. In four of the trials, the behavioural co-intervention was specialized behavioural counselling (i.e., CBT or relapse prevention).

^d^ **Bupropion and Placebo**: Studies provided 300 mg/day of bupropion for about 22 days to 14 weeks, across trials; dose and duration of intervention for one study unclear. One trial provided both arms with a specialized behavioural counselling co-intervention (CBT). In a second study, both arms received non-contingent reinforcement. No co-interventions provided in remaining trial.

^e^ **Bupropion and Placebo**: All trials examined 300 mg/day of bupropion for about 10 or 12 weeks. Behavioural co-intervention provided to both arms in all trials. In one trial, participants in both arms also received NRT patch and NRT gum as a co-intervention. All participants in this analysis received specialized behavioural counselling (CBT or relapse prevention).

^f^ **Bupropion and Placebo**: Both trials provided 300 mg/day of bupropion for about 10 or 12 weeks. Behavioural co-intervention provided to both arms in both trials. All participants in this analysis received specialized behavioural counselling (CBT or relapse prevention).

^g^ **Bupropion and Placebo:** Studies provided 300 mg/day of bupropion for about 22 days to 14 weeks, across trials; dose and duration of intervention for one study unclear. One trial provided both arms with a specialized behavioural counselling co-intervention (CBT). In a second study, both arms received non-contingent reinforcement. No co-interventions provided in remaining trial.

# Mixed approaches

| Combined pharmacotherapy and behavioural interventions versus Usual care or no/minimal intervention in the general/mixed population | | | | | | | | | | |
| --- | --- | --- | --- | --- | --- | --- | --- | --- | --- | --- |
| **Outcome(s)**  **(Follow up (fu): time)**  **# of participants (# studies)** | | **Median Patient rating** | **WG rating** | **Relative effect (95%CI)** | **Baseline (control) risk** | **Absolute difference with intervention** | **Assessment of absolute difference** | | | |
|  |  |  |  |  |  |  | **Point estimate** | **Lower 95% CI** | **Upper 95%CI** | **Comments on judgment** |
| 1 | Smoking cessation (fu: 6+ mo)^a^  *versus Usual care or minimal intervention*  19488 (52 RCTs) | 8/10  (critical) | 8.8/10  (critical) | RR 1.83 (1.68 to 1.98) | 86 per 1,000 | **71 more per 1,000** (from 58 more to 84 more) | Moderate benefit | Moderate benefit | Moderate benefit | Point estimate and CI considered moderate benefits when considering baseline risks, few harms of intervention, health benefits of quitting, and greater involvement of intervention. No harms identified in this review. However, indirect evidence from other populations and interventions suggests some potential harms from pharmacotherapy, although not judged as sufficient to reduce to small benefit, due to harms associated with continuing to smoke. |
|  | Smoking cessation (fu: 12 mo)^b^  *versus Usual care or no intervention*  5887 (1 RCT) | 8/10  (critical) | 8.8/10  (critical) | RR 3.88 (3.35 to 4.50) | 90 per 1000 | **260 more per 1000** (from 212 more to 315 more) | Large benefit | Large benefit | Large benefit | Increase in quit rate (point estimate and CI) is large considering the baseline (9.0% in the control group and 35.0% in the intervention group) and the health benefits of quitting. No harms identified in this review. However, indirect evidence from other populations and interventions suggests some potential harms, but not judged as sufficient to reduce to moderate benefit, due to harms associated with continuing to smoke. |

^a^ **Intervention:** the typical intervention involved multiple contacts with a specialist cessation counsellor, with most using pharmacotherapy and receiving multiple contacts, but with variation across studies. Additional behavioural with or without pharmacological co-intervention provided in a few trials. **Control:** Usual care or minimal intervention was typically brief advice and self-help materials. Additional behavioural and/or pharmacological co-intervention provided in some trials. Unclear whether co-intervention provided in some trials.

^b^ **Intervention**: Consisted of 12 group sessions over 10 weeks, with advice from physician on risk for COPD, 2 mg of nicotine gum for 6 months. Intervention participants were also randomized to bronchodilator or placebo. No co-interventions. **Usual care or no intervention:** No co-interventions provided.

| Interventions to increase adherence to medications for tobacco dependence compared to usual or standard care in smokers motivated/wishing to quit or reduce smoking | | | | | | | | | | |
| --- | --- | --- | --- | --- | --- | --- | --- | --- | --- | --- |
| **Outcome(s)**  **(Follow up (fu): time)**  **# of participants (# studies)** | | **Median Patient rating** | **Mean WG rating** | **Relative effect (95%CI)** | **Baseline (control) risk** | **Absolute difference with intervention** | **Assessment of absolute difference** | | | |
|  |  |  |  |  |  |  | **Point estimate** | **Lower 95% CI** | **Upper 95%CI** | **Comments on judgment** |
| 1 | Smoking cessation (fu: 6 mo)^a^  3049 (4 RCTs) | 8/10 (critical) | 8.8/10 (critical) | RR 1.16 (1.01 to 1.34) | 171 per 1000 | **27 more per 1000** (from 2 more to 58 more) | Small but important benefit | Little to no difference | Moderate benefit | Increase in quit rate is small benefit when considering the health benefits of quitting, baseline risk, and given the higher demands of the intervention (17.1% in the control group and 19.9% in the intervention group). Evidence of harms was unclear but suggest little to no difference. Upper CI is considered a moderate benefit when taking into account demands of intervention, and health benefits of quitting. |
| 5 | Adverse events  (fu: unclear)^b^  NR (3 RCTs) | 7/10 (important) | 6.5/10 (important) | One study each reported no serious adverse events or no treatment related adverse events. The third study reported no difference in adverse events between groups. | | | Little to no difference | Unable to assess | Unable to assess | Insufficient data from review to determine imprecision. Data suggests little to no difference between groups but is difficult to interpret. |
| 7 | Change in mental state (anxiety)^c^  (fu: 1-week to 6 mo)  NR (1 RCT) | 8/10 (important) | 5.5/10 (important) | The study reports no difference between groups regarding levels of anxiety at both time points. | | | Little to no difference | Unable to assess | Unable to assess | Insufficient data from review to determine imprecision. Data suggests little to no difference between groups but is difficult to interpret. |

^a^ **Interventions:** Interventions were aimed to increase adherence to tobacco cessation medications. As compared to the control condition, in 3 of 4 trials, the intervention included an additional component focusing on medication adherence with additional contact time. In the remaining trial, the nature of the intervention differed across arms but contact time was similar (i.e., tailoring NRT dosage based on genotype rather than phenotype). The intervention involved specialized behavioural counselling in 3 of 4 studies (i.e., counselling based on motivational interviewing techniques and 4R approach, counselling based on withdrawal-oriented therapy); this was received by 67.5% of participants in this analysis. The intervention was delivered by phone in one study and in-person in three studies. Trained counsellors delivered the intervention in three studies while nurses delivered the intervention in one study. All participants were receiving NRT. **Control:** Eligible control conditions were usual or standard care which could consist of minimal support or varying degrees of behavioural support. All trials provided some behavioural support to control participants which ranged up to seven weekly sessions. In one trial, the behavioural support involved specialized behavioural counselling (i.e., based on withdrawal-oriented therapy) which was received by 21% of control participants in this analysis. All participants were receiving NRT.

^b^ **Intervention:** Interventions were aimed to increase adherence to tobacco cessation medications. In one trial, the intervention included an additional component focusing on medication adherence and additional contact time as compared to the control condition. In two trials, the nature of the intervention differed across arms but contact time was similar; one trial tailored NRT dosage based on genotype (versus phenotype in controls) and the second provided a personalized feedback component on medication use and adherence. The intervention involved specialized behavioural counselling in one study (i.e., counselling based on withdrawal-oriented therapy). The intervention was delivered by phone in one study and in-person in two studies. Trained counsellors delivered the intervention in two studies while nurses delivered the intervention in one study. All participants were receiving NRT. **Control:** Eligible control conditions were usual or standard care which could consist of minimal support or varying degrees of behavioural support. All trials provided some behavioural support to control participants which ranged up to seven weekly sessions. In one trial, the behavioural support involved specialized behavioural counselling (i.e., based on withdrawal-oriented therapy). All participants were receiving NRT.

^c^ **Intervention:** Intervention was aimed at increasing adherence to tobacco cessation medication. In the only trial included in this analysis, the nature of the intervention differed across arms but contact time was similar; the intervention arm received tailored NRT dosage and rationale based on genotype. The intervention was delivered in-person by nurses and involved specialized behavioural counselling (i.e., counselling based on withdrawal-oriented therapy). All participants were receiving NRT. **Control:** NRT dosage and rationale based on phenotype. The behavioural support involved specialized behavioural counselling (i.e., based on withdrawal-oriented therapy). All participants were receiving NRT.

| Standard treatment plus extended NRT and extended CBT^a^ versus Standard treatment: Smoking cessation in smokers with past depression | | | | | | | | | | |
| --- | --- | --- | --- | --- | --- | --- | --- | --- | --- | --- |
| **Outcome(s)**  **(Follow up (fu): time)**  **# of participants (# studies)** | | **Median Patient rating** | **WG rating** | **Relative effect (95%CI)** | **Baseline (control) risk** | **Absolute difference with intervention** | **Assessment of absolute difference** | | | |
|  |  |  |  |  |  |  | **Point estimate** | **Lower 95% CI** | **Upper 95%CI** | **Comments on judgment** |
| 1 | Smoking cessation (fu: NR)  57 (1 RCT) | 8/10  (critical) | 8.8/10  (critical) | RR 1.94 (0.98 to 3.85) | 276 per 1,000 | **259 more per 1,000** (from 6 fewer to 786 more) | Large benefit | Little to no difference | Large benefit | Increase in quit rate was very large even considering high baseline quit rate (27.6% quit rate in the control group and 53.6% quit rate in the intervention group), and considering health benefits of quitting. Judgement for point estimate and upper CI remained a large benefit after considering indirect evidence of potential harms from NRT in other populations. Lower CI suggests little to no difference given baseline risk. |

^a^ **Intervention and control**: Standard treatment consisted of “12 weeks of sustained release bupropion (150 mg/d first; from week 2 300 mg/d) and 10 weeks of 2 mg and 4 mg nicotine gum, and received 5 group counselling sessions (week 1, 3 (2 sessions), 5 and 8) from a counsellor and a self-help manual. No further treatment after week 12.” No other co-interventions provided. In addition to standard treatment, the intervention arm received NRT until week 52 and 11 individual CBT sessions from week 10 to 52. No other co-interventions provided.

| Individual smoking cessation intervention (cognitive behavioural therapy and motivational interviewing) plus NRT patch^a^ versus Routine care smokers with schizophrenia or schizoaffective disorder | | | | | | | | | | |
| --- | --- | --- | --- | --- | --- | --- | --- | --- | --- | --- |
| **Outcome(s)** | | **Median Patient rating** | **WG rating** | **Relative effect (95%CI)** | **Baseline (control) risk** | **Absolute difference with intervention** | **Assessment of absolute difference** | | | |
|  |  |  |  |  |  |  | **Point estimate** | **Lower 95% CI** | **Upper 95%CI** | **Comments on judgment** |
| 1 | *NRT patch*  Smoking cessation (fu: 6 mo, 12 mo, 4 yr)  NR (1 RCT) | 8/10 (critical) | 8.8/10 (critical) | No statistically significant difference between groups in point prevalence or continuous abstinence rates at all follow-up time points. Threshold for statistical significance was p<0.01. | | | Little to no difference | Unable to assess | Unable to assess | Insufficient data from review to determine imprecision. Data from review suggests little to no difference between groups but is difficult to interpret. |
| 2 | *NRT patch*  Smoking reduction (>50% of baseline)  (fu: 6 mo, 12 mo, 4 yr)  NR (1 RCT) | 7/10 (critical) | 5/10 (important) | No statistically significant difference between groups in smoking reduction at all follow-up time points. | | | Little to no difference | Unable to assess | Unable to assess | Insufficient data from review to determine imprecision. Data suggests little to no difference between groups but is difficult to interpret. |

^a^ **Intervention**: Involved eight hours of individual contact for eight weeks. NRT patch provided for about 10 weeks (21 mg, 14 mg, 7 mg titrated down). Booklets on smoking cessation provided to participants as co-intervention. **Control:** No extra contact time. Participants received booklets on smoking cessation as co-intervention.

# Alternative therapies

| Hypnotherapy^a^ versus Placebo drug in smokers motivated/wishing to quit | | | | | | | | | | |
| --- | --- | --- | --- | --- | --- | --- | --- | --- | --- | --- |
| **Outcome(s)**  **(Follow up (fu): time)**  **# of participants (# studies)** | | **Median Patient rating** | **Mean**  **WG rating** | **Relative effect (95%CI)** | **Baseline (control) risk** | **Absolute difference with intervention** | **Assessment of absolute difference** | | | |
|  |  |  |  |  |  |  | **Point estimate** | **Lower 95% CI** | **Upper 95%CI** | **Comments on judgment** |
| 1 | Smoking cessation  (fu: 12 mo)  114  (1 RCT) | 8/10  (critical) | 8.8/10  (critical) | RR 0.83 (0.27 to 2.58) | 105 per 1000 | **18 fewer per 1000**  (from 77 fewer to 166 more) | Small but important harm | Moderate harm | Large benefit | Point estimate is judged as a small harm given that fewer patients would quit smoking, and given the harms associated with continuing to smoke. Lower CI is judged as a moderate harm based on the same rationale. The upper CI is judged as a large benefit due to health benefits of quitting. No evidence identified on harms. |

^a^ **Hypnotherapy:** Number of sessions and duration not reported. No co-interventions provided. **Placebo drug:** No co-interventions provided.

| St John’s Wort^a^ versus Placebo drug in smokers motivated/wishing to quit | | | | | | | | | | |
| --- | --- | --- | --- | --- | --- | --- | --- | --- | --- | --- |
| **Outcome(s)**  **(Follow up (fu): time)**  **# of participants (# studies)** | | **Median Patient rating** | **Mean**  **WG rating** | **Relative effect (95%CI)** | **Baseline (control) risk** | **Absolute difference with intervention** | **Assessment of absolute difference** | | | |
|  |  |  |  |  |  |  | **Point estimate** | **Lower 95% CI** | **Upper 95%CI** | **Comments on judgment** |
| 1 | Smoking cessation  (fu: 6 mo)  261 (2 RCTs) | 8/10  (critical) | 8.8/10  (critical) | RR 0.81 (0.26 to 2.53) | 54 per 1000 | **10 fewer per 1000**  (from 40 fewer to 83 more) | Small but important harm | Moderate harm | Moderate benefit | Point estimate is judged as a small harm given that fewer patients would quit smoking, and given the harms associated with continuing to smoke. Lower CI is judged as a moderate harm based on the same rationale. The upper CI is judged as a moderate benefit given health benefits of quitting smoking. No evidence identified on harms was identified. |

^a^ **Intervention:** One trial examined 900 mg/day of St John's wort for 14 weeks and the other examined 900 mg/day and 1800 mg/day for 12 weeks (arms collapsed in analysis). **Control:** placebo. Behavioural co-intervention provided to all arms in both trials.

| S-Adenosyl-L-Methionine (SAMe)^a^ versus Placebo drug in smokers motivated/wishing to quit | | | | | | | | | | |
| --- | --- | --- | --- | --- | --- | --- | --- | --- | --- | --- |
| **Outcome(s)**  **(Follow up (fu): time)**  **# of participants (# studies)** | | **Median Patient rating** | **Mean**  **WG rating** | **Relative effect (95%CI)** | **Baseline (control) risk** | **Absolute difference with intervention** | **Assessment of absolute difference** | | | |
|  |  |  |  |  |  |  | **Point estimate** | **Lower 95% CI** | **Upper 95%CI** | **Comments on judgment** |
| 1 | Smoking cessation  (fu: 6 mo)  120 (1 RCT) | 8/10  (critical) | 8.8/10  (critical) | RR 0.70 (0.24 to 2.07) | 125 per 1,000 | **38 fewer per 1,000** (from 95 fewer to 134 more) | Moderate harm | Large harm | Large benefit | Point estimate is judged as a moderate harm given that fewer patients would quit smoking, and given the harms associated with continuing to smoke. Lower CI is judged as a large harm based on the same rationale. The upper CI is judged as a large benefit given the health benefits of quitting. No evidence identified on harms. |

^a^ **Intervention:** Trial examined both 800 mg/day and 1600 mg/day SAMe for 8 weeks (arms collapsed in analysis). **Control:** placebo. Behavioural co-intervention provided to all trial arms.

| Acupuncture in smokers motivated/wishing to quit | | | | | | | | | | |
| --- | --- | --- | --- | --- | --- | --- | --- | --- | --- | --- |
| **Outcome(s)**  **(Follow up (fu): time)**  **# of participants (# studies)** | | **Median Patient rating** | **Mean**  **WG rating** | **Relative effect (95%CI)** | **Baseline (control) risk** | **Absolute difference with intervention** | **Assessment of absolute difference** | | | |
|  |  |  |  |  |  |  | **Point estimate** | **Lower 95% CI** | **Upper 95%CI** | **Comments on judgment** |
| 1 | *Versus sham acupuncture*  Smoking cessation  (fu: 6-12 mo)^a^  1892 (11 RCTs) | 8/10  (critical) | 8.8/10  (critical) | RR 1.10 (0.86 to 1.40) | 108 per 1,000 | **11 more per 1,000** (from 15 fewer to 43 more) | Little to no difference | Little to no difference | Moderate benefit | Point estimate is judged to be little to no difference, and upper CI a moderate benefit, given size of absolute effects compared to the high baseline risk, lack of evidence of harms, and health benefits of quitting. Lower CI is considered a little to no difference given the harms of continuing to smoke. No evidence identified on harms of the intervention. |
|  | *Versus waiting list/no intervention*  Smoking cessation  (fu: 6-12 mo)^b^  393 (3 RCTs) | 8/10  (critical) | 8.8/10  (critical) | RR 1.79 (0.98 to 3.28) | 77 per 1,000 | **60 more per 1,000** (from 2 fewer to 174 more) | Moderate benefit | Little to no difference | Large benefit | Point estimate is judged to be a moderate benefit given the baseline risk, lack of evidence of harms, and health benefits of quitting. Upper CI is judged as a large benefit based on same rationale. Lower CI is considered little to no difference. |

^a^ **Acupuncture:** All trials selected acupuncture points (i.e., anatomic sites) for smoking cessation. Two studies used facial acupuncture, five used auricular acupuncture alone with/without continuous stimulation (i.e., needle or pressure device), and four used combined body and auricular acupuncture with/without continuous stimulation (i.e., indwelling needle or seed). Behavioural co-intervention provided in some trials, pharmacotherapy in one study, and ‘other’ (i.e., placebo nicotine gum) in one study. **Sham**: Two trials, representing 4% of the evidence, used potentially active acupuncture points for the sham arm. Behavioural co-intervention provided to control arm in some trials, pharmacotherapy in one study, and ‘other’ (i.e., placebo nicotine gum) in one study.

^b^ **Acupuncture:** All trials selected acupuncture points (i.e., anatomic sites) for smoking cessation. One study used facial acupuncture and two used auricular acupuncture alone with or without continuous stimulation (i.e., needle or pressure device). One study provided a behavioural co-intervention to both study arms. No co-interventions in remaining studies.

| Continuous auricular stimulation^a^ versus Sham stimulation in smokers motivated/wishing to quit | | | | | | | | | | |
| --- | --- | --- | --- | --- | --- | --- | --- | --- | --- | --- |
| **Outcome(s)**  **(Follow up (fu): time)**  **# of participants (# studies)** | | **Median Patient rating** | **Mean**  **WG rating** | **Relative effect (95%CI)** | **Baseline (control) risk** | **Absolute difference with intervention** | **Assessment of absolute difference** | | | |
|  |  |  |  |  |  |  | **Point estimate** | **Lower 95% CI** | **Upper 95%CI** | **Comments on judgment** |
| 1 | Smoking cessation  (fu: 6-12 mo)  570 (6 RCTs) | 8/10  (critical) | 8.8/10  (critical) | RR 1.47 (0.79 to 2.74) | 56 per 1,000 | **26 more per 1,000** (from 12 fewer to 98 more) | Small but important benefit | Small but important harm | Large benefit | Point estimate is judged to be a small but important benefit given the baseline risk, lack of evidence of harms, and health benefits of quitting. Upper CI is judged as a large benefit (almost 10% absolute increase in quit rate) based on same rationale. Lower CI is considered a small but important harm due to harms of continuing to smoke. No evidence identified on harms. |

^a^ **Intervention:** Four studies used indwelling needles and remainder used continuous acupressure. Two studies provided a behavioural co-intervention. **Sham:** One trial, representing 5% of the evidence, used potentially active acupuncture points for the sham arm. Two studies provided a behavioural co-intervention.

| Laser therapy^a^ versus Sham laser in smokers motivated/wishing to quit | | | | | | | | | | |
| --- | --- | --- | --- | --- | --- | --- | --- | --- | --- | --- |
| **Outcome(s)**  **(Follow up (fu): time)**  **# of participants (# studies)** | | **Median Patient rating** | **Mean**  **WG rating** | **Relative effect (95%CI)** | **Baseline (control) risk** | **Absolute difference with intervention** | **Assessment of absolute difference** | | | |
|  |  |  |  |  |  |  | **Point estimate** | **Lower 95% CI** | **Upper 95%CI** | **Comments on judgment** |
| 1 | Smoking cessation  (fu: 6-12 mo)  613 (2 RCTs) | 8/10  (critical) | 8.8/10  (critical) | Studies were heterogeneous and could not be quantitatively synthesized (I2=97%). Review authors report that heterogeneity could be due to populations recruited and dose of laser administered.  Results from one study were null-inclusive (RR 1.03, 95% CI: 0.55, 1.94; laser vs sham 10.3% vs 10%; RD 3 more per 1,000, 95% CI 45 fewer to 94 more). The other study reported results favouring the intervention (RR 14.18, 95% CI: 5.92, 33.94; laser vs sham 55.4% vs 3.9%, RD 515 more per 1,000, 95% CI 192 to 1,000 more). | | | Little to no difference | Moderate harm | Moderate benefit | Given the implausibility of the results of the second study (500+ additional quitters per 1000 participants), only the results from the first study are considered in the judgement of benefit. Point estimate is judged to be little to no difference. Lower CI is judged as a moderate harm (45 fewer quitters, due to harms of continuing to smoke) and upper CI is considered a moderate benefit (94 more quitters, due to health benefits of quitting). No evidence identified on harms. |

^a^ **Laser therapy:** Only one study reported the dose of laser used which was 50mW for 14 minutes. One study provided a behavioural co-intervention. **Sham laser:** One study provided a behavioural co-intervention.

| Electrostimulation^a^ versus Sham in smokers motivated/wishing to quit | | | | | | | | | | |
| --- | --- | --- | --- | --- | --- | --- | --- | --- | --- | --- |
| **Outcome(s)**  **(Follow up (fu): time)**  **# of participants (# studies)** | | **Median Patient rating** | **Mean**  **WG rating** | **Relative effect (95%CI)** | **Baseline (control) risk** | **Absolute difference with intervention** | **Assessment of absolute difference** | | | |
|  |  |  |  |  |  |  | **Point estimate** | **Lower 95% CI** | **Upper 95%CI** | **Comments on judgment** |
| 1 | Smoking cessation  (fu: 6-12 mo)  405 (2 RCTs) | 8/10  (critical) | 8.8/10  (critical) | RR 0.87 (0.61 to 1.23) | 260 per 1,000 | **34 fewer per 1,000** (from 102 fewer to 60 more) | Small but important harm | Large harm | Moderate benefit | Point estimate suggests a small harm (fewer patients quitting smoking) due to harms of continuing to smoke. Lower CI suggests a large harm for the same reason. Upper CI suggests a moderate benefit given baseline risk and health benefits of quitting. No evidence identified on harms. |

^a^ **Intervention:** Electrostimulation administered through surface electrodes over the mastoid bone in one study and to the ear in the other. No co-interventions provided in both studies. **Control:** No co-interventions provided to control arms in either study.
