# Supplementary material for: Effectiveness of smoking cessation interventions among adults: an overview of systematic reviews
Source: Syst Rev. 2024 Jul 12;13:179. doi: 10.1186/s13643-024-02570-9 (PMC11242003; doi:10.1186/s13643-024-02570-9)
Supplement: Supplementary file 15 — Additional file 15. Review characteristics. [file 13643_2024_2570_MOESM15_ESM.docx]

**Additional file 15.1: Review characteristics tables**

| **Author Year {refID};**  **Country;**  **Funding;**  **COI;** | **Date of last search;**  **Databases searched;**  **Total number of studies included in the review;**  **Total number of participants;**  **AMSTAR 2 overall rating** | **Populations for which data is reported** | **Comparisons of relevance to the overview;**  **Intervention delivery;**  **Setting;** | **Outcome(s)** | **General study characteristics** |
| --- | --- | --- | --- | --- | --- |
| **Barnes 2019 {3836};**  New Zealand;  University of Dundee, Department of Medicine; Department of Primary Health Care, Oxford University; Wellcome Trust; NHS Research and Development National Cancer Programme;  Review authors declare having no conflicts of interest | July 2018;  Cochrane Tobacco Addiction Group Specialized Register, MEDLINE, EMBASE, AMED, ISI Science Citation and Social Science Citation Indexes, Web of Science. The CISCOM database was no longer available for the 2010 update;  14;  Approximately 1,926;  AMSTAR 2: Critically low | Smokers motivated to quit/wishing to quit ^a^ | **(1) Hypnotherapy alone vs. placebo drug alone**  (2) Hypnotherapy vs. Brief attention/cessation advice  (3) Hypnotherapy vs. Psychological treatments (4) Hypnotherapy plus Other treatments ^b^ vs. Other treatments ^b^ (5) Hypnotherapy alone or plus other treatments vs. Control ^c^  NR;  No setting restriction | Tobacco smoking abstinence/cessation  Adverse events | Across trials mean age was between 30-40 years, participants smoked between 20 and 40 CPD, and there were more females than males.  All studies conducted in countries very high on HDI (i.e., USA, Canada, UK, Australia). |

Abbreviations: AMED: Allied and Alternative Medicine database; CISCOM: Centralised Information Service for Complementary Medicine; CPD: cigarettes per day; NR: not reported
**Bolded** comparisons are selected for GRADEing as they examine a relevant comparator (e.g., placebo, usual care) and population (e.g., general/mixed population of smokers)

^a^ The population eligibility criterion of the review is “people who wish to stop smoking”. However, interest or wish to quit is not explicitly reported in review evidence tables for all included trials.

^b^ “Other” treatments in both arms are identical and include self-help materials plus supportive calls, group counselling, or rapid smoking plus counselling.

^c^ Various control conditions across trials including but not limited to: no treatment waiting list, brief attention/cessation advice, psychological treatments (e.g., counselling), psychological treatment plus nicotine patch plus counselling

| **Author Year {refID};**  **Country;**  **Funding;**  **COI;** | **Date of last search;**  **Databases searched;**  **Total number of studies included in the review;**  **Total number of participants;**  **AMSTAR 2 overall rating** | **Populations for which data is reported** | **Comparisons of relevance to the overview;**  **Intervention delivery;**  **Setting;** | **Outcome(s)** | **General study characteristics** |
| --- | --- | --- | --- | --- | --- |
| **Cahill 2010 {1652};**  UK;  Department of Primary Health Care, Oxford University, UK;  Review authors declare having no conflicts of interest | August 2010;  Cochrane Tobacco Addiction Review Group Specialized Register (CENTRAL, MEDLINE, EMBASE, PsycINFO);  41;  >33,000;  AMSTAR 2: Critically low | General/Mixed population of smokers (i.e., not a specifically defined subpopulation)  Gender (males, females) | **(1) Stage-based expert systems or tailored self-help materials versus Assessment only ^a^**  **(2)** **Stage-based expert systems suitable for those in precontemplation or contemplation versus No intervention (3)** **Stage-based interactive computer programmes versus Usual care (4)** **Stage-based telephone counselling versus Usual care** **(5)** **Stage-based individual counselling and/or advice vs Usual care (6)** **Stage-based individual counselling or advice vs Assessment only ^b^** (7) Stage-based self-help materials versus Standard self-help materials  (8) Stage-based self-help materials versus Usual care ^c^  (9) Stage-based expert systems or tailored self-help materials versus Standard self-help materials  (10) Stage-based expert systems or tailored self-help materials versus Usual care ^d^  (11) Stage-based telephone counselling versus Standard self-help control  (12) Higher 'dose'/number of contacts of stage-based self-help intervention versus Lower dose stage-based self-help intervention (13) Stage-based individual counselling vs Standard self-help materials (14) Stage-based individual counselling vs Standard advice    NR;  No setting restriction | Tobacco smoking abstinence/cessation | Setting of studies varied and included both relevant and irrelevant settings. Eleven studies were population-based. Others were set in clinics or in outpatient departments (n=9), antenatal clinics (n=3), hospital wards (n=3), family practices (n=5). Six studies were in a school setting (secondary schools, colleges). Remainder were telephone quitline (n=2) or workplace (n=2) studies.  All studies were conducted in very high HDI countries (USA, UK, Australia, Netherlands, Germany, Belgium, Canada, Finland, Switzerland, Taiwan, Japan). |

Abbreviations: HDI: Human Development Index; NR: not reported
**Bolded** comparisons are selected for GRADEing as they examine a relevant comparator (e.g., placebo, usual care) and population (e.g., general/mixed population of smokers)

^a^ Review authors treat 'assessment only' as a no intervention control. Across trials, 'assessment only' is defined as: 'assessment only', no intervention, a letter confirming no self-help information will be sent, and non-smoking related healthcare intervention including 3-5 minute dietary advice.

^b^ Review authors treat 'assessment only' as a no intervention control. Across trials, 'assessment only' is defined as 'measurement only' in two trials and 'interventions for hypertension or hypercholesterolaemia' in one trial.

^c^ Males only

^b^ Females only

| **Author Year {refID};**  **Country;**  **Funding;**  **COI;** | **Date of last search;**  **Databases searched;**  **Total number of studies included in the review;**  **Total number of participants;**  **AMSTAR 2 overall rating** | **Populations for which data is reported** | **Comparisons of relevance to the overview;**  **Intervention delivery;**  **Setting;** | **Outcome(s)** | **General study characteristics** |
| --- | --- | --- | --- | --- | --- |
| **Cahill 2016 {1960};**  UK;  Department of Primary Care Health Sciences, University of Oxford, UK; National School for Health Research School for Primary Care Research, UK.; NHS Research and Development Fund, UK;  Review authors declare having conflicts of interest | May 2015;  Cochrane Tobacco Addiction Group specialized register inclusive of searches from MEDLINE, Embase, and PsycINFO;  44;  3,461;  AMSTAR 2: Low | General/Mixed population of smokers (i.e., not a specifically defined subpopulation)  Smokers motivated/wishing to quit  Smokers reducing to quit  Smoker with schizophrenia, bipolar, or other psychiatric disorder  Smokers with depression and motivated/wishing to quit  Smokers who previously failed to quit on varenicline but are motivated/wishing to try again  Cardiovascular disease  Chronic obstructive pulmonary disease  Asthma  Substance use disorder  HIV  Diabetes  Pregnancy  Head and neck cancer  Light smokers  Gender and ethnicity specific  Alcohol dependent | **(1) Varenicline vs Placebo (2) Cytisine vs Placebo** **(3) Non-standard dose varenicline versus Placebo**  **(4) Long-term varenicline use vs Placebo**  **(5) Low-dose varenicline vs. Placebo**  **(6) Varenicline varied dosing vs. Placebo**  **(7) Varenicline preloading vs. Placebo**†  (8) Cytisine vs NRT (9) Varenicline vs Bupropion (10) Standard dose varenicline versus Low dose (11) Varenicline vs NRT  (12) Combination varenicline treatment vs. single-therapy treatment†  (13) Varenicline tablets vs. other formulations†  NR;  No setting restriction | Tobacco smoking abstinence/cessation  Abnormal dreams  Adverse events  Cardiac SAEs, including deaths  Depression  Headache  Insomnia  Nausea  Neuropsychiatric SAEs (not deaths)  SAEs ^a^  Suicidal ideation  Harm reduction  Treatment discontinuation | **Cytisine trials:**  Trials were conducted in countries ranked very high and medium on HDI (Germany, Kyrgyzstan, and Poland.) Two studies were in smoking cessation clinics, and the third in a workplace setting. One comparative effectiveness non-inferiority trial studied a New Zealand national smoking quitline.  **Varenicline trials:**  Seventeen studies were set in countries ranked very high and high on the HDI. Ten studies were conducted in multiple countries. The trials were conducted in smoking cessation clinics, hospitals, universities and other research centres.  Participants in the majority of the trials were adult smokers, willing to make a quit attempt. Several trials recruited hospital inpatients (n=3) and disease-specific patient groups (CVD, n=1; acute coronary syndrome, n=1; COPD: n=1; asthma: n=1; substance use disorder: n=2; alcohol abuse: n=1; depression: n=1; bipolar/schizophrenia; schizoaffective disorder: n=3). One study enrolled two cohorts of adult smokers with and without histories of psychiatric disorders. Two trials targeted subgroups of smokers who were failing to respond to smoking cessation pharmacotherapies, either by increasing the dosage (n=1) or by switching to different medications n=1). Four studies focused on previous quitters. One was a reduction to quit trial. |

Abbreviations: HDI: Human Development Index; NR: not reported; NRT: nicotine replacement therapy; SAEs: serious adverse events
**Bolded** comparisons are selected for GRADEing as they examine a relevant comparator (e.g., placebo, usual care) and population (e.g., general/mixed population of smokers)

† No studies were found addressing these comparisons.

^a^ Varenicline trials only

| **Author Year {refID};**  **Country;**  **Funding;**  **COI;** | **Date of last search;**  **Databases searched;**  **Total number of studies included in the review;**  **Total number of participants;**  **AMSTAR 2 overall rating** | **Populations for which data is reported** | **Comparisons of relevance to the overview;**  **Intervention delivery;**  **Setting;** | **Outcome(s)** | **General study characteristics** |
| --- | --- | --- | --- | --- | --- |
| **Farley 2012 {1469};**  UK;  University of Birmingham, UK; The UK Centre for Tobacco Control Studies, UK; Queen Mary’s University of London, UK; National Institute of Health Research, UK;  Review authors declare having conflicts of interest | September 2011;  Searched for trials from existing Cochrane reviews ^a^ in Issue 9, 2011 of Cochrane Library; recency of individual review search dates ranging Oct 2007-2011. To identify recent trials published since these reviews, review authors also searched CENTRAL Issue 4, 2011;  54;  NR;  AMSTAR 2: Critically low | Smokers motivated/wishing to quit ^b^ at baseline and abstinence at follow-up | **(1) Bupropion vs. Placebo**  **(2) Any type of NRT vs. Placebo**  **(3) Varenicline (dose 1) vs. Placebo**  **(4) Varenicline (dose 2) vs. Placebo**  (5) Higher vs lower dose Bupropion (2 comparisons)  (6) Exercise plus Smoking cessation programme vs. Smoking cessation programme only  (7) Higher vs. lower dose NRT  (8) Higher vs. lower dose NRT patch  (9) Comparison of different NRT types (2 comparisons)  (10) Longer vs. Shorter course NRT patch  (11) Varenicline vs. Bupropion  (12) Varenicline vs. NRT patch  NR;  No setting restriction | Weight gain in abstinent smokers | Trials typically recruited community volunteers; others recruited from primary care and one from inpatient hospital setting.  Average of 20-30 CPD. All participants motivated to quit.  Baseline mean BMI of 24-25 or mean weight ≤ 85 kg across the 23 trials (43%) reporting this data. |

Abbreviations: BMI: body mass index; CPD: cigarettes per day; NR: not reported; NRT: nicotine replacement therapy
**Bolded** comparisons are selected for GRADEing as they examine a relevant comparator (e.g., placebo, usual care) and population (e.g., general/mixed population of smokers)

^a^ Antidepressants for smoking cessation (latest search, Jul 2009) Hughes 2007; Exercise interventions for smoking cessation (latest search, July 2008) Ussher 2008; Nicotine replacement therapy for smoking cessation (latest search, Oct 2007) Stead 2008; Cannabinoid type 1 receptor antagonists (rimonabant) for smoking cessation (latest search, Jan 2011) Cahill 2011a; and Nicotine receptor partial agonists for smoking cessation (latest search, Oct 2010) Cahill 2011b. Not all reviews listed here are of relevance.

^b^ Review authors state that all trials included in the review examined smokers motivated to quit. However, motivation to quit not explicitly reported in review evidence tables for most trials.

| **Author Year {refID};**  **Country;**  **Funding;**  **COI;** | **Date of last search;**  **Databases searched;**  **Total number of studies included in the review;**  **Total number of participants;**  **AMSTAR 2 overall rating** | **Populations for which data is reported** | **Comparisons of relevance to the overview;**  **Intervention delivery;**  **Setting;** | **Outcome(s)** | **General study characteristics** |
| --- | --- | --- | --- | --- | --- |
| **Hartmann-Boyce 2018 {332};**  UK;  Nuffield Department of Primary Care Health Sciences, University of Oxford; National Institute for Health Research School for Primary Care Research; National Institute for Health Research (NIHR);  Review authors declare having conflicts of interest | July 2017;  Cochrane Tobacco Addiction Group Specialized Register (CENTRAL, MEDLINE, Embase, PsycINFO). Previous review versions: Cancerlit, Health Planning and Administration, Social Scisearch, Smoking & Health, and Dissertation Abstracts;  136;  Over 64,000;  AMSTAR 2: Critically low | Smokers motivated/wishing to quit  Pregnant and postpartum women  Relapsed smokers motivated to quit | **(1)** **Nicotine patch vs. Placebo patch (2)** **NRT vs. Placebo**  (3) Any NRT vs. Placebo or other control group not receiving any type of NRT ^a^  NR;  No setting restriction | Tobacco smoking abstinence/cessation  Adverse events (all)  Adverse events (palpitations, tachycardia, chest pain)  Adverse events (attrition) | Most studies (n=134) in adults (average age 40-50), similar numbers of men and women. Two trials in adolescents.  People typically smoked at least 15 CPD at baseline. One trial, people smoked ≤ 10 CPD. Two trials people smoked ≥ 30 CPD.  Various countries of conduct, including those less than very high on HDI.  Studies randomized therapists rather than smokers as eligibility criterion; unclear whether it applies to all studies. |

Abbreviations: CPD: cigarettes per day; HDI: Human Development Index; NR: not reported; NRT: nicotine replacement therapy

**Bolded** comparisons are selected for GRADEing as they examine a relevant comparator (e.g., placebo, usual care) and population (e.g., general/mixed population of smokers)

^a^Comparator includes both active and inactive controls.

| **Author Year {refID};**  **Country;**  **Funding;**  **COI;** | **Date of last search;**  **Databases searched;**  **Total number of studies included in the review;**  **Total number of participants;**  **AMSTAR 2 overall rating** | **Populations for which data is reported** | **Comparisons of relevance to the overview;**  **Intervention delivery;**  **Setting;** | **Outcome(s)** | **General study characteristics** |
| --- | --- | --- | --- | --- | --- |
| **Hollands 2019 {3841};**  UK;  King’s College London (Database access); University of Cambridge (Computer use, database access)  No external sources of support.;  Review authors declare having conflicts of interest | September 2018;  Cochrane Tobacco Addiction Group Specialized Register, CENTRAL, MEDLINE, EMBASE, PsycINFO;  10;  3655;  AMSTAR 2: Low | Smokers motivated/wishing to quit or reduce smoking ^a^ | **(1) Interventions to increase adherence to medications for tobacco dependence vs Usual or standard care** ^b^  NR;  No setting restriction. Intervention delivered in-person (n=7), by phone (n=1) or by automated telephone call (n=1_. Intervention delivered by trained counsellors (n=7), nurses (n=2) or CBT therapists (n=1). | Tobacco smoking abstinence/cessation  Adverse events  Change in emotional state: anxiety | Participants were typically healthy smokers but one study included males with erectile dysfunction. Participants in all trials were motivated to quit or reduce smoking and agreed to receive medication and behavioural support.  Mean age ranged from 34.6 to 49 years. Two trials were all female and one trial recruited only males. In the other trials, % female ranged from 7.5 to 62.5.  Location of trials: USA (n=7), Hong Kong (n=2), UK (n=1). |

Abbreviations: CBT=Cognitive behavioural therapy; NR: not reported
**Bolded** comparisons are selected for GRADEing as they examine a relevant comparator (e.g., placebo, usual care) and population (e.g., general/mixed population of smokers)

^a^ Motivated to quit or reduce smoking and who had agreed to receive medication and behavioural support to assist them in doing so.

^b^ Eligible control conditions were usual or standard care which could consist of minimal support or varying degrees of behavioural support. The trials all provided some behavioural support to participants in the control arm.

| **Author Year {refID};**  **Country;**  **Funding;**  **COI;** | **Date of last search;**  **Databases searched;**  **Total number of studies included in the review;**  **Total number of participants;**  **AMSTAR 2 overall rating** | **Populations for which data is reported** | **Comparisons of relevance to the overview;**  **Intervention delivery;**  **Setting;** | **Outcome(s)** | **General study characteristics** |
| --- | --- | --- | --- | --- | --- |
| **Howes 2020 {96};**  USA;  Department of Primary Health Care, Oxford University, UK; National Institute for Health Research School for Primary Care Research, UK; National Institute on Drug Abuse (NIDA), USA; NHS Research and Development Programme, UK;  Review authors declare having conflicts of interest | May 2019;  Cochrane Tobacco Addiction Group’s Specialised Register (CENTRAL, MEDLINE, EMBASE, and PsycINFO);  115;  NR;  AMSTAR 2: Critically low | General/Mixed population of smokers (i.e., not a specifically defined subpopulation)  Smokers motivated/wishing to quit  Smokers not motivated/wishing to quit  Current or past depression  Pregnancy  Smokers who failed to quit using NRT  Age ^a^  Gender ^b^ | **(1) Bupropion vs Placebo (2) St John’s wort vs Placebo (3) SAMe vs Placebo**  (4) Bupropion vs Placebo or no pharmacotherapy control (5) Higher dose bupropion vs Lower dose bupropion  (6) Bupropion plus NRT vs. NRT alone (7) Bupropion vs NRT (8) Bupropion vs Varenicline  (9) Bupropion with/without NRT vs Placebo or NRT  (10) Bupropion with/without NRT (non-comparative or mostly non-comparative findings ^c^)  NR;  No setting restriction | Tobacco smoking abstinence/cessation  Tobacco smoking reduction: (i) reduction in CPD, (ii) reduction in continine >50% from baseline at 1 year  Change in emotional state: depressive symptoms  Adverse events: (i) major malformation, spontaneous abortion, infant exposure, (ii) overdose and death, (iii) psychiatric adverse events, (iv) seizures, (v) serious adverse events | Majority of studies excluded smokers with current depression but included those with a history of past depression. One study restricted inclusion to those with current depression.  **Bupropion studies:**  Majority of studies conducted in North America; others conducted in Europe, Brazil, Australia, Israel, New Zealand, Pakistan, Turkey, or recruited from multiple continents. Various population groups examined including COPD, schizophrenia, PTSD, cancer, suspected TB, alcoholism, cardiovascular disease, inpatients, adolescents, smokers awaiting surgery, healthcare workers, African-Americans, Maori, smokers who failed to quit on NRT or bupropion, and smokers wanting to reduce but not quit. Across trials, smokers with past depression was generally between 20 and 30% (range 6-44%). |

Abbreviations: COPD: chronic obstructive pulmonary disease; CPD: cigarettes per day; NR: not reported; NRT: nicotine replacement therapy; PTSD: post traumatic stress disorder; SAMe: S-Adenosyl-Methionine; TB: tuberculosis
**Bolded** comparisons are selected for GRADEing as they examine a relevant comparator (e.g., placebo, usual care) and population (e.g., general/mixed population of smokers)

^a^ Treatment effects between older and younger smokers

^b^ Treatment effects by gender

^c^ Comparative studies include a mix of active and inactive control conditions

| **Author Year {refID};**  **Country;**  **Funding;**  **COI;** | **Date of last search;**  **Databases searched;**  **Total number of studies included in the review;**  **Total number of participants;**  **AMSTAR 2 overall rating** | **Populations for which data is reported** | **Comparisons of relevance to the overview;**  **Intervention delivery;**  **Setting;** | **Outcome(s)** | **General study characteristics** |
| --- | --- | --- | --- | --- | --- |
| **Khanna, 2016 {310};**  UK;  Huddersfield University, UK; Queen Margaret University, UK;  Review authors declare having no conflicts of interest | April 2015;  Cochrane Schizophrenia Group Trials Register, BNI, CINHAL, EMBASE, MEDLINE, and PsycINFO;  0;  0;  AMSTAR 2: Moderate | Serious mental illness^a^ | Advice versus standard care†;  NR;  No setting restriction | N/A – no studies found | N/A – no studies found |

Abbreviations: BNI: British Nursing Index; NR: Not reported; N/A: Not applicable.

†No studies were found addressing these comparisons.

^a^ For example, schizophrenia, schizophrenia-like disorders, bipolar disorder, or serious affective disorders.

| **Author Year {refID};**  **Country;**  **Funding;**  **COI;** | **Date of last search;**  **Databases searched;**  **Total number of studies included in the review;**  **Total number of participants;**  **AMSTAR 2 overall rating** | **Populations for which data is reported** | **Comparisons of relevance to overview;**  **Intervention delivery;**  **Setting;** | **Outcome(s)** | **General study characteristics** |
| --- | --- | --- | --- | --- | --- |
| **Lancaster 2017 {539};**  UK;  Oxford University Department of Primary Health Care; National Institute for Health Research School for Primary Care Research; NHS Research and Development Programme, UK;  Review authors declare having no conflicts of interest | May 2016;  Cochrane Tobacco Addiction Group Specialized Register (CENTRAL, MEDLINE, EMBASE, PsycINFO);  49;  Around 19,000;  AMSTAR 2: Critically low | General/Mixed population of smokers (i.e., not a specifically defined subpopulation)  Trials recruiting pregnant women excluded. | **(1) Individual counselling vs Minimal contact control ^a^** (2) Individual counselling plus pharmacotherapy vs Minimal contact control plus pharmacotherapy (3) More intensive vs less intensive counselling (4) Comparison between counselling approaches;  Specialist counsellors (counselling not involved in routine clinical care);  No setting restriction | Tobacco smoking abstinence/cessation | 37% (n=18) relevant studies, inclusive of, primary care, dental, community, lung cancer screening, post partum, and periodic health exam, Planned Parenthood clinic.  4% (n=2) studies of mixed relevance: worksite and inpatients  51% (n=25) irrelevant, inclusive of medical/surgical hospital inpatients, substance use residential rehabilitation, and worksite.  Characteristics of remaining studies (n=4) NR in aggregate by authors.  Motivation to quit not part of eligibility, but authors state was sometimes difficult to assess.  Various countries of conduct, including those less than very high on HDI. |

Abbreviations: HDI: Human Development Index; NR: not reported

**Bolded** comparisons are selected for GRADEing as they examine a relevant comparator (e.g., placebo, usual care) and population (e.g., general/mixed population of smokers)

^a^ No systematic pharmacotherapy in either group

| **Author Year {refID};**  **Country;**  **Funding;**  **COI;** | **Date of last search;**  **Databases searched;**  **Total number of studies included in the review;**  **Total number of participants;**  **AMSTAR 2 overall rating** | **Populations for which data is reported** | **Comparisons of relevance to the overview;**  **Intervention delivery;**  **Setting;** | **Outcome(s)** | **General study characteristics** |
| --- | --- | --- | --- | --- | --- |
| **Lindson-Hawley 2016 {671};**  UK;  Nuffield Department of Primary Care Health Sciences, University of Oxford; Public Health, Epidemiology & Biostatistics, University of Birmingham; National Institute for Health Research;  Review authors declare having conflicts of interest | October 2015;  Cochrane Tobacco Addiction Group Trials Register (CENTRAL, MEDLINE, Embase, PsycINFO);  24;  NR;  AMSTAR 2: Critically low | Smokers not motivated to quit/not wishing to quit ^a^ | **(1) NRT ^b^ vs Placebo;** **(2)** **Bupropion ^b^ vs Placebo (3) Varenicline ^b^ vs Placebo (4) E-cigarettes ^b^ vs Placebo (non-nicotine) electronic cigarettes** **(5) Telephone-based individual counselling plus self-help materials ^b^ vs Usual care**  **(6) Behavioural support to reduce smoking (advice) plus NRT ^c^ plus phone calls vs No intervention** (7) Behavioural support to reduce smoking (advice) plus NRT ^c^ plus phone calls vs Motivational interviewing ^d^ plus NRT ^c^ plus self-help materials plus counsellor call (8) Behavioural support to reduce smoking by >50% plus NRT plus brief advice to quit plus self-help materials vs. Brief advice to quit at initial visit plus offer of NRT ^c^ (no further support)  (9) Computerized programme ^b^ vs self-help reduction guide  (10) NRT ^b^ vs Placebo/No NRT ^e^ (11) NRT alone or with counselling ^b^ vs Placebo, folic acid (placebo) or brief advice  (12) NRT plus counselling ^b^ vs Brief cessation advice  NR;  No setting restriction | Tobacco smoking abstinence/cessation  Tobacco smoking reduction  Adverse events  Serious adverse events  Stopping medication due to adverse events  Weight gain | Most studies recruited via community advertising.  One study recruited adolescents and another recruited hospitalized patients.  Most studies excluded people motivated to quit but criteria for ascertaining motivation varied. One study recruited those motivated to quit and in one study, motivation to quit was not required.  Generally, participants had no immediate intention to quit but motivation to do so in the long-term was often high.  Generally, participants smoked between 20 and 30 CPD. |

Abbreviations: CPD: cigarettes per day; HDI: Human Development Index; NR: not reported; NRT: nicotine replacement therapy
**Bolded** comparisons are selected for GRADEing as they examine a relevant comparator (e.g., placebo, usual care) and population (e.g., general/mixed population of smokers)

^a^ Review eligibility criterion is people who smoke tobacco but have no immediate intention to quit all tobacco use.

^b^ To assist smoking reduction

^c^ NRT offered if quit date set

^d^ Intended to increase interest in quitting

^e^ Comparator as reported by review authors. Comparator includes both active and inactive comparators.

| **Author Year {refID};**  **Country;**  **Funding;**  **COI;** | **Date of last search;**  **Databases searched;**  **Total number of studies included in the review;**  **Total number of participants;**  **AMSTAR 2 overall rating** | **Populations for which data is reported** | **Comparisons of relevance to the overview;**  **Intervention delivery;**  **Setting;** | **Outcome(s)** | **General study characteristics** |
| --- | --- | --- | --- | --- | --- |
| **Livingstone-Banks 2019 {1077};**  UK;  Nuffield Department of Primary Care Health Sciences, University of Oxford, UK.; National Institute for Health Research (NIHR) School for Primary Care Research, UK.; National Institute for Health Research (NIHR) Cochrane Programme Grant, UK.; National Institute for Health Research (NIHR) Community Healthcare Medtech and In Vitro Diagnostics Cooperative (MIC), UK; NIHR Biomedical Research Centre, Oxford, UK;  Review authors declare having no conflicts of interest | March 2018;  Cochrane Tobacco Addiction Review Group Specialised Register (CENTRAL, MEDLINE; Embase, and PsycINFO);  75;  NR;  AMSTAR 2: Moderate | General/Mixed population of smokers (i.e., not a specifically defined sub-population)  Smokers motivated/wishing to quit ^a^  Trials recruiting pregnant women excluded. | **(1) Non-tailored print-based self-help materials (no face-to-face contact) versus No materials/no intervention**  **(2) Non-tailored print-based self-help materials (no face-to-face contact) versus Brief leaflet ^b^**  **(3) Non-tailored print-based self-help materials (with face-to-face contact ^c^ ) versus No intervention or leaflet only ^b^**  **(4) Individually tailored print-based self-help materials (no face-to-face contact) vs No materials/no intervention**  (5) Individually tailored print-based self-help materials (no face-to-face contact) vs Brief advice  (6) Individually tailored materials as an adjunct to advice  (7) Self-help materials with nicotine patch or placebo patch plus additional video vs Self-help materials with nicotine patch or placebo patch without additional video (8) Manual targeted at a particular population vs Standard self-help manual (9) Self-help materials plus NRT vs NRT alone (10) Individually tailored self-help materials vs Standard or stage-matched materials (groups matched for number of contacts) (11) Tailored self-help materials (multiple mailings) vs Standard self-help or stage-matched materials (single mailing) (12) Individually tailored self-help vs No self-help materials ^d^  (13) Non-tailored self-help materials vs No self-help materials ^d^ (14) Non-tailored self-help materials plus advice vs Brief advice alone  (15) One type of self-help material versus Another type (16) Non-tailored self-help materials plus additional written materials vs Self-help materials/programme without additional written materials  NR;  No setting restriction | Tobacco smoking abstinence/cessation | Nearly all studies conducted in high-income countries.  Other relevant information not reported in aggregate by review authors (i.e. study characteristics reported by intervention category only). |

Abbreviations: NR: not reported; NRT: nicotine replacement therapy
**Bolded** comparisons are selected for GRADEing as they examine a relevant comparator (e.g., placebo, usual care) and population (e.g., general/mixed population of smokers)

^a^ Smokers seeking treatment

^b^ Leaflet considered a minimal print-based self-help intervention by review authors

^c^ Materials delivered in-person instead of by post

^d^ Comparator includes both active and inactive controls (e.g., no intervention, brief advice control)

| **Author Year {refID};**  **Country;**  **Funding;**  **COI;** | **Date of last search;**  **Databases searched;**  **Total number of studies included in the review;**  **Total number of participants;**  **AMSTAR 2 overall rating** | **Populations for which data is reported** | **Comparisons of relevance to the overview;**  **Intervention delivery;**  **Setting;** | **Outcome(s)** | **General study characteristics** |
| --- | --- | --- | --- | --- | --- |
| **Matkin 2019 {1228};**  UK;  Internal sources: National Institute for Health Research (NIHR) School for Primary Care Research, UK.; Department of Primary Care Health Sciences, University of Oxford, UK; NIHR Community Healthcare Medtech and In Vitro Diagnostics Cooperative (MIC), UK; Partial funding: NIHR Biomedical Research Centre, Oxford, UK. External sources: NHS Research & Development Programme, UK.;  Review authors declare having no conflicts of interest | May 2018;  Cochrane Tobacco Addiction Group Specialised Register (CENTRAL, MEDLINE, Embase, PsycINFO), WHO ICTRP, ClinicalTrials.gov;  104;  111,653;  AMSTAR 2: Low | General/Mixed population of smokers (i.e., not a specifically defined subpopulation)  Smokers motivated to quit/wishing to quit.  Ethnicity | **(1) Hotline and self-help materials vs Minimal intervention ^a^ (2) Intensive telephone counselling vs Minimal intervention (3) Brief motivational telephone counselling vs Usual care telephone call**  **(4) Telephone counselling for smoking reduction vs Usual care telephone call**  (5) Telephone counselling (proactive) vs Self-help materials or brief counselling (6) Telephone counselling (reactive) vs Self-help materials (7) Telephone counselling (tailored) vs Telephone counselling (standard) (8) Stage-based counselling vs General information/standard support ^b^ (9) Telephone counselling (Acceptance and Commitment Therapy) plus NRT vs Telephone counselling (Cognitive Behavioural Therapy) plus NRT (10) Telephone counselling (Motivational Interviewing) vs Telephone counselling (standard) (11) Telephone counselling (reactive or proactive) vs Provider counselling (quitline service) (12) Telephone counselling (proactive) vs Telephone counselling (reactive) (13) Telephone counselling (proactive) vs Self-help materials (14) Proactive counselling, not initiated by calls to quitlines vs Control ^c^ (15) Telephone counselling (smoking reduction) vs Telephone counselling (brief motivational counselling) (16) Telephone counseling (tailored) vs Referral to a state tobacco quitline (17) Brief quitline facilitation intervention vs Brief advice  NR;  No setting restriction | Tobacco smoking abstinence/cessation | Participants were mostly older adults with an average age in the 40s. Two studies in adolescents, one in young adults (18-24 years), and three recruited older people (over 50, 60, and 65 years, respectively). Four studies in pregnant women and five in women-only. Four studies in mainly men.  One study was culturally tailored for Chinese, Korean and Vietnamese smokers and one recruited Arabic smokers in Australia.  Most trials were conducted in North America.  The number, duration, and content of telephone calls varied across studies. |

Abbreviations: ICTRP: International Clinical Trials Registry Platform; NR: not reported; NRT: nicotine replacement therapy; WHO: World Health Organization
**Bolded** comparisons are selected for GRADEing as they examine a relevant comparator (e.g., placebo, usual care) and population (e.g., general/mixed population of smokers)

^a^ Review authors report the comparator as 'minimal intervention'. This consists of usual care in one trial and a self-help manual in the other trial.

^b^ Control condition is non-specific brief counselling. Callers to hotline received general information based on fact sheets, and sent S-H material.

^c^ Various control conditions across trials including minimal intervention without systematic pharmacotherapy (n=33), brief intervention/counselling without systematic pharmacotherapy (n=13), usual care or a behavioural intervention (e.g., self-help materials, access to a website, physician advice) with systematic offer or provision of pharmacotherapy (i.e., NRT, varenicline, 'any cessation medication') (n=18), and incentives (n=1).

| **Author Year {refID};**  **Country;**  **Funding;**  **COI;** | **Date of last search;**  **Databases searched;**  **Total number of studies included in the review;**  **Total number of participants;**  **AMSTAR 2 overall rating** | **Populations for which data is reported** | **Comparisons of relevance to the overview;**  **Intervention delivery;**  **Setting;** | **Outcome(s)** | **General study characteristics** |
| --- | --- | --- | --- | --- | --- |
| **Posadzki 2016 {659};**  Singapore;  NR;  Conflicts of interest not declared | May/June 2015;  CENTRAL, MEDLINE, Embase, PsycINFO, Web of Science, ASSIA ProQuest, CINAHL, GlobalHealth, WHOLIS, LILACS;  132 (10 studies on ATCS for managing tobacco dependence);  4,669,689;  AMSTAR 2: Critically low | General/Mixed population of smokers (i.e., not a specifically defined subpopulation) | **(1) ATCS, IVR systems vs. No intervention**  (2) ATCS (ACTS Plus, IVR systems) vs. No calls, Usual care, Inactive IVR system ^a^  NR;  No setting restriction | Tobacco smoking abstinence/cessation | Two studies (2%) were from low-income countries, one (1%) from a middle-income country, and the remaining 129 studies (97%) from high-income countries.  14 studies (11%) took place in the 1990s and 118 (89%) date from 2000 onwards.  In the subset of studies with an aim of managing tobacco dependence (n=10), five were conducted in the USA, three in Canada, one in Norway, and one in Taiwan. Mean age of participants was between 20 and 54 years across trials. In one trial, a substantial proportion of participants had a chronic condition and in a second trial, participants had acute coronary syndrome and received the intervention after being discharged from hospital. |

Abbreviations: ASSIA: Applied Social Sciences Index and Abstracts; ATCS: automated telephone communication systems; IVR: interactive voice response; LILACS: Latin America and Caribbean Health Sciences Literature; NR: not reported; WHOLIS: World Health Organization library database
**Bolded** comparisons are selected for GRADEing as they examine a relevant comparator (e.g., placebo, usual care) and population (e.g., general/mixed population of smokers)

^a^ Mix of active (self-help interventions such as stage-based manuals) and inactive (inactive IVR system, usual care, no intervention) control conditions.

| **Author Year {refID};**  **Country;**  **Funding;**  **COI;** | **Date of last search;**  **Databases searched;**  **Total number of studies included in the review;**  **Total number of participants;**  **AMSTAR 2 overall rating** | **Populations for which data is reported** | **Comparisons of relevance to the overview;**  **Intervention delivery;**  **Setting;** | **Outcome(s)** | **General study characteristics** |
| --- | --- | --- | --- | --- | --- |
| **Stead 2013 {1998};**  UK;  University of Oxford, Department of Primary Health Care, UK; National School for Health Research School for Primary Care Research, UK; NHS Research and Development Programme, UK;  Review authors declare having no conflicts of interest | January/February 2013  Cochrane Tobacco Addiction Group specialised register (MEDLINE, EMBASE and PsycINFO, CENTRAL),  Latin American databases through BVS (Virtual Library inHealth) which covered six databases (Lilacs, Biblioteca Cochrane, Wholis, Leyes, Scielo, Inbiomed);  42;  Over 31,000;  AMSTAR 2: Critically low | General/Mixed population of smokers  Trials recruiting pregnant women excluded. | **(1) Physician advice (minimal or intensive interventions) vs No advice (or usual care) ^a^ (2) Physician advice with follow-up vs Minimal intervention/advice with single visit**  **(3) Intensive advice versus Minimal advice**  (4) Motivational counselling versus Brief advice  (5) Physician advice (autonomy-supporting style) vs Physician advice (controlling style) (6) Brief advice vs Computer-generated tailored letters and selected self-help manuals  NR;  No setting restriction | Tobacco smoking abstinence/cessation | Most participants were from unselected populations; however, in some trials, participants had co-morbidities (e.g., diabetes, asbestos exposed, cardiovascular disease). |

Abbreviations: CVD: cardiovascular disease; Lilacs: Latin America and Caribbean Health Sciences Literature; NR: not reported; Scielo: Scientific Electronic Library Online; Wholis: World Health Organization library database
**Bolded** comparisons are selected for GRADEing as they examine a relevant comparator (e.g., placebo, usual care) and population (e.g., general/mixed population of smokers)

^a^ Comparator described as ‘no advice (or usual care)’ by review authors. Across trials, control conditions include smoking-related written materials, 'control' (not further specified), 'no advice' (not further specified), self-monitoring of smoking, usual care, no intervention, questionnaires.

| **Author Year {refID};**  **Country;**  **Funding;**  **COI;** | **Date of last search;**  **Databases searched;**  **Total number of studies included in the review;**  **Total number of participants;**  **AMSTAR 2 overall rating** | **Populations for which data is reported** | **Comparisons of relevance to the overview;**  **Intervention delivery;**  **Setting;** | **Outcome(s)** | **General study characteristics** |
| --- | --- | --- | --- | --- | --- |
| **Stead 2016 {1356};**  UK;  Nuffield Department of Primary Care Health Sciences, University of Oxford, UK.; NHS, National Institute for Health Research, UK; National School for Health Research, School for Primary Care Research, UK;  Review authors declare having no conflicts of interest | July 2015;  Cochrane Tobacco Addiction Specialised Register (CENTRAL, MEDLINE, EMBASE, and PsycINFO), MEDLINE;  53;  More than 25,000;  AMSTAR 2: Low | General/Mixed population of smokers (i.e., not a specifically defined subpopulation),  Trials recruiting pregnant women excluded. | **(1) Combined pharmacotherapy and behavioural interventions vs Usual care or minimal intervention  (2) Combined pharmacotherapy and behavioural interventions vs Usual care/no intervention**  Most counselling and support provided by specialist counsellors. Primary care professionals provided counselling/support in a small subset of studies.  No setting restriction | Tobacco smoking abstinence/cessation | Trials conducted in a wide range of settings and included patients with mental illness and smoking related diseases.  Females made up of 35 to 65% of the population. Two trials recruited women only, whereas one trial recruited men only.  A few trials had higher proportion of men (Veterans Administration medical system and a Spanish workplace trial).  Average age across trials typically low 40s to mid 50s. |

Abbreviations: NR: not reported
**Bolded** comparisons are selected for GRADEing as they examine a relevant comparator (e.g., placebo, usual care) and population (e.g., general/mixed population of smokers)

| **Author Year {refID};**  **Country;**  **Funding;**  **COI;** | **Date of last search;**  **Databases searched;**  **Total number of studies included in the review;**  **Total number of participants;**  **AMSTAR 2 overall rating** | **Populations for which data is reported** | **Comparisons of relevance to the overview;**  **Intervention delivery;**  **Setting;** | **Outcome(s)** | **General study characteristics** |
| --- | --- | --- | --- | --- | --- |
| **Stead 2017 {538};**  UK;  Internal sources: Department of Primary Health Care, Oxford University, UK; National Institute for Health Research (NIHR) School for Primary Care Research, UK.  External sources: NHS Research and Development National Cancer Programme, England, UK; Predoctoral Individual National Research Service Award (F31 HL129494), USA;  Review authors declare having no conflicts of interest | May 2016;  Cochrane Tobacco Addiction Group Specialized Register (CENTRAL, MEDLINE, Embase, PsycINFO);  66;  NR;  AMSTAR 2: Critically low | General/Mixed population of smokers (i.e., not a specifically defined sub-population)  Trials recruiting pregnant women excluded. | **(1) Group vs No intervention ^a^**  (2) Group programme vs Self-help (3) Group programme vs Brief support  (4) Group therapy plus pharmacotherapy vs Pharmacotherapy and brief support (5) Group programme vs Individual therapy (6) Mood management vs Standard programme ^b^ (7) Skills training with additional components vs Basic skills programme (8) Manipulating group dynamics vs Different approach to manipulating group dynamics (9) Three-way comparison: ALA Freedom from Smoking programme, ACS Freshstart programme, and laboratory-derived programme (10) Therapist-administered programme using one of three manuals vs Self-administered programme using same three manuals as intervention group ^c^ (11) Five-week, 10-session group counselling vs five-session group counselling (12) Manipulated pre-quit expectations (optimistic vs balanced)  NR;  No setting restriction | Tobacco smoking abstinence/cessation | A majority of studies recruited community volunteers. Three studies recruited from primary care settings, while others recruited patients with cardiovascular health problems, diabetes, schizophrenia, or those in outpatient/inpatient alcohol treatment programmes.  Four studies recruited employees at worksites. Two studies recruited only women, one study recruited only men, and two studies recruited only African-American smokers.  Generalizability was not specified. |

Abbreviations: ACS: American Cancer Society; ALA: American Lung Association; NR: not reported
**Bolded** comparisons are selected for GRADEing as they examine a relevant comparator (e.g., placebo, usual care) and population (e.g., general/mixed population of smokers)

^a^ No or minimal intervention.

^b^ Group counselling (different from intervention arm).

^c^ No comparison specified. Reflected as different programmes in the narrative description.

| **Author Year {refID};**  **Country;**  **Funding;**  **COI;** | **Date of last search;**  **Databases searched;**  **Total number of studies included in the review;**  **Total number of participants;**  **AMSTAR 2 overall rating** | **Populations for which data is reported** | **Comparisons of relevance to the overview;**  **Intervention delivery;**  **Setting;** | **Outcome(s)** | **General study characteristics** |
| --- | --- | --- | --- | --- | --- |
| **Taylor 2017 {411};**  Singapore;  Department of Primary Care and Social Medicine, Imperial College London; Ministry of Science, Education and Sport; Department of Primary Care Health Sciences, Oxford University (Editorial base for the Cochrane Tobacco Addiction Group); National Institute for Health Research School for Primary Care Research; Support for the Department of Primary Health Care, Oxford University; Lee Kong Chian School of Medicine, Nanyang Technological University, Singapore eLearning grant; NHS Connecting for Health Evaluation Programme (NHS CFHEP 001); NHS Research and Development Programme (Infrastructure funding for the Cochrane Tobacco Addiction Group);  Review authors declare having no conflicts of interest | August 2016;  Specialised register of the Cochrane Tobacco Addiction Group, (CENTRAL, MEDLINE, Embase, PsycINFO);  67;  > 110,000;  AMSTAR 2: Critically low | General/Mixed population of smokers (i.e., not a specifically defined subpopulation)  Smokers motivated to quit/wishing to quit  Ethnicity ^a^ | **(1)** **Internet (interactive and tailored) vs Non-active control ^b^** **(2) Internet plus behavioural support vs Non-Internet-based, non-active control ^c^**  (3) Internet (interactive, not tailored) vs Non-active control ^d^ (4) Internet vs Active control ^e^ (5) Internet plus behavioural support vs Non-Internet-based active control ^e^ (6) Tailored/interactive internet programmes vs Not tailored/interactive internet programmes (7) Tailored vs non-tailored Internet-based messages (8) Printed list of websites vs Self-help written materials (9) Multiple comparisons consisting of 16 variations of the ’Q2’ intervention ^f^ (10) Full website vs 'Lite website' (11) 'Action Planning' internet intervention vs 'Computer tailored' internet intervention (no action planning advice) (12) QuitCoach with Rapid Implementation vs QuitCoach without Rapid Implementation (13) Interactive and tailored internet intervention with motivational messages vs Interactive and tailored internet intervention without motivational messages (14) Interactive and tailored internet intervention with motivational messages and personal online support from trained tobacco treatment specialists and a link to an online support group vs Interactive and tailored internet intervention without motivational messages, without personal online support from trained tobacco treatment specialists, and without online support group (15) Internet-based interventions vs Control ^g^  (16) Other comparisons between internet interventions  (17) Increasing tailoring depth of internet programme ^h^  NR;  No setting restriction | Tobacco smoking abstinence/cessation  Adverse events | Most studies recruited adults, three studies recruited adolescents only and seven studies recruited young adults, university or college students.  Most studies recruited via web. Others recruited by advertising (e.g. billboards, television), from health care settings, or other settings (e.g., health surveys, educational institutions).  Some studies recruited subpopulations including adults who were childhood cancer survivors (n=1), Korean Americans only (n=2), military veterans and their families (n=2), participants with chronic physical conditions (n=4), pregnant smokers (n=1), and hospitalized patients (n=1).  Sample sizes ranged from less than 70 to nearly 12,000. There were more women than men and the mean age ranged from 16 to 63 years.  Participants offered financial compensation for completing assessments in 21 studies.  Almost all studies were conducted in countries that are very high on the HDI. |

Abbreviations: HDI: Human Development Index; NR: not reported
**Bolded** comparisons are selected for GRADEing as they examine a relevant comparator (e.g., placebo, usual care) and population (e.g., general/mixed population of smokers)

^a^ Korean-Americans
^b^ Mix of trials with usual care or printed self-help control conditions. Review authors consider these 'non-active' controls.
^c^ All trials examined a 'non-active' control condition, as defined by review authors.

^d^ The only trial included in this analysis provided control group with booklet with the same content as the internet intervention. Review authors consider this to be a non-active control. Trial participants were Korean-Americans.
^e^ ‘Active control', as defined by review authors, consist of interventions that were more intensive than usual care or self-help only.

^f^ No comparison specified. Narrative synthesis describes results generally across all experimental conditions.

^g^ Comparator includes both active and inactive control conditions (e.g., internet-based intervention, 'usual smoking cessation treatment', in-person counselling, usual care consisting of counselling and information to quit).

^h^ Trial compared multiple conditions in a fractional factorial design.

| **Author Year {refID};**  **Country;**  **Funding;**  **COI;** | **Date of last search;**  **Databases searched;**  **Total number of studies included in the review;**  **Total number of participants;**  **AMSTAR 2 overall rating** | **Populations for which data is reported** | **Comparisons of relevance to the overview;**  **Intervention delivery;**  **Setting;** | **Outcome(s)** | **General study characteristics** |
| --- | --- | --- | --- | --- | --- |
| **Tsoi 2013 {1698};**  UK;  Nottinghamshire Healthcare NHS Trust; Division of Psychiatry, University of Nottingham; Academic Clinical Psychiatry, University of Sheffield; School of Public Health, University of Sydney; NHS National Institute for Health Research;  Review authors declare having no conflicts of interest | October/November 2012;  Cochrane Tobacco Addiction Group Specialised Register, MEDLINE, MEDLINE In-Process & Other Non-Indexed Citations, EMBASE, PsycINFO, CINAHL Plus with Full Text, ISI Web of Science with Conference Proceedings, BIOSIS Previews;  34;  NR;  AMSTAR 2: Moderate | Schizophrenia or schizoaffective disorder | **(1) Bupropion (used primarily for cessation) vs Placebo**  **(2) Bupropion (used primarily for reduction) vs Placebo (3) Varenicline (used primarily for cessation) vs Placebo**  **(4) Varenicline (for other uses ^a^) vs Placebo (5) NRT (used primarily for cessation) vs Placebo patch** **(6) Individual smoking cessation intervention (based on CBT and MI) plus NRT vs Routine care**  (7) ALA programme in group setting vs specialised smoking cessation group therapy designed for schizophrenia (8) TANS vs MM (9) Group counselling vs Individual counselling†  (10) Telephone counselling vs Any control†  (11) Physician advice vs Any control†  (12) Self-help interventions vs Any control†  (13) Varenicline used primarily for smoking reduction vs Any control†  NR;  No setting restriction | Tobacco smoking abstinence/cessation  Tobacco smoking reduction: (i) Change in number of CPD from baseline, (ii) Expired CO levels  Changes in mental state: (i) positive symptoms ^b^; (ii) negative symptoms ^c^; (iii) depressive symptoms; (iv) psychiatric symptoms  Adverse events (e.g., all AEs, serious AEs, medication side effects) | Trials with primary aim of cessation, reduction, relapse prevention (n=26):   - Most studies recruited from the community. Other studies recruited from inpatient setting (n=4) and from both hospital and community (n=1). - One study recruited patients with both nicotine and alcohol dependence but many trials excluded participants with non-nicotine SUDs. - Trials recruiting smokers with various psychiatric diagnoses were included (n=3) if data was reported separately for those with schizophrenia or schizoaffective disorder. - All participants wanted to quit or reduce smoking in 16 trials. Thirteen studies had target quit dates. - Most studies conducted in USA. Remaining conducted in Australia, Canada, Iran, Israel, Taiwan, and China.   Trials without primary aim of cessation, reduction, relapse prevention (n=8):   - Primary aim of these studies varied but included examining varenicline for alcohol dependence or cognitive function. |

Abbreviations: ALA: American Lung Association; CBT: cognitive behavioural therapy; CPD: cigarettes per day; HDI: Human Development Index; MI: motivational interviewing; MM: medication management; NR: not reported; SUDs: substance use disorders; TANS: Treatment of Addiction to Nicotine in Schizophrenia; **Bolded** comparisons are selected for GRADEing as they examine a relevant comparator (e.g., placebo, usual care) and population (e.g., general/mixed population of smokers)

†No studies were found addressing these comparisons.

^a^ Used for reasons other than smoking cessation or reduction

^b^ Positive symptoms (e.g. hallucinations, delusions)

^c^ Negative symptoms (e.g. anhedonia, avolition)

| **Author Year {refID};**  **Country;**  **Funding;**  **COI;** | **Date of last search;**  **Databases searched;**  **Total number of studies included in the review;**  **Total number of participants;**  **AMSTAR 2 overall rating** | **Populations for which data is reported** | **Comparisons of relevance to the overview;**  **Intervention delivery;**  **Setting;** | **Outcome(s)** | **General study characteristics** |
| --- | --- | --- | --- | --- | --- |
| **van der Meer 2013 {1223};**  Netherlands;  STIVORO - for a smoke-free future; Vrije Universiteit Amsterdam.; Trimbos-Instituut, Netherlands Institute of Mental Health and Addiction;  Review authors declare having no conflicts of interest | April 2013;  Tobacco Addiction Group Specialised Register, CENTRAL, MEDLINE, EMBASE, PsycINFO;  49;  NR;  AMSTAR 2: Critically low | Current or past depression | **(1) Bupropion vs Placebo**  **(2) NRT vs Placebo**  **(3) Standard treatment ^a^ plus extended NRT plus extended CBT vs Standard treatment ^a^**  (4) Psychosocial mood management ^b^ vs Control ^c^ (5) Psychosocial interventions without specific components for depression vs ‘Standard smoking cessation counselling’  (6) Concurrent smoking cessation treatment (individual counselling plus NRT) vs Delayed smoking cessation ^d^ (7) Nurse managed lay led by protocol vs Personalised letter written by a physician (8) ‘Other pharmacotherapy’ (i.e., no antidepressants or NRT) ^e^ vs Placebo  NR;  No setting restriction | Tobacco smoking abstinence/cessation | Many studies included subgroups with current and past depression (prestated stratification or post hoc subgroups).  No relevant aggregate information provided by review authors (i.e. study characteristics reported by intervention and population categories). |

Abbreviations: NR: not reported; NRT: nicotine replacement therapy
**Bolded** comparisons are selected for GRADEing as they examine a relevant comparator (e.g., placebo, usual care) and population (e.g., general/mixed population of smokers)

^a^ Standard treatment: 12 wks of sustained release bupropion (150 mg/d first; from wk 2 300 mg/d) and 10 wks of 2 mg and 4 mg nicotine gum, and received 5 group counselling sessions (wk 1, 3 (2 sessions), 5 and 8) from a counsellor and a self-help manual. No further treatment after wk 12.

^b^ Psychosocial mood management component is cognitive behavioural therapy for depression in almost all trials. In one trial each, the component is cognitive behavioural analysis system of psychotherapy and hypnosis skills.

^c^ ‘Control’ as reported by review authors. Various control conditions across trials including group sessions of standard cognitive behavioural smoking cessation treatment and patient manual; standard cognitive-behavioral smoking cessation treatment;

standard behavioral counselling; time and contact health education control focused on health and wellness; telephone counselling; health education; etc.

^d^ Same as experimental arm but 6 months after study enrolment

^e^ Naltrexone (n=2); Bupropion plus NRT patch (n=1).

| **Author Year {refID};**  **Country;**  **Funding;**  **COI;** | **Date of last search;**  **Databases searched;**  **Total number of studies included in the review;**  **Total number of participants;**  **AMSTAR 2 overall rating** | **Populations for which data is reported** | **Comparisons of relevance to the overview;**  **Intervention delivery;**  **Setting;** | **Outcome(s)** | **General study characteristics** |
| --- | --- | --- | --- | --- | --- |
| **Vodopivec-Jamsek 2012 {1343};**  Slovenia;  eHealth Unit, Department of Primary Care and Social Medicine, Imperial College, UK.; Centre for Health Management, Tanaka Business School, Imperial College, UK.; Department of Family Medicine, Faculty of Medicine, University of Ljubljana, Slovenia.; Imperial College London, UK.; Ministry of Higher Education, Science and Technology, Slovenia;  NR | June 2009;  CENTRAL, MEDLINE, EMBASE, PsycINFO, CINAHL, LILACS, African Health Anthology;  4;  1,933;  AMSTAR 2: Low | Smokers motivated to quit/wishing to quit | **(1) Mobile phone SMS vs Placebo ^a^**  NR;  No setting restriction | Tobacco smoking abstinence/cessation  Adverse events;  (i) rates of car crash;  (ii) pain in thumb/finger joints from texting | The review had broadly included interventions addressing any type of preventive health care.  One of four included studies addressed smoking cessation. This study included current smokers over 16 years of age. Participants came from a range of income levels: 26% from low, 42% from middle and 31% from higher income level. The study was conducted in New Zealand and included 21% Maori and 79% Non-Maori participants.  There was no restriction  on the use of other smoking cessation strategies by study participants. |

Abbreviations: LILACS: Latin America and Caribbean Health Sciences Literature; NR: not reported; SMS: short message service
**Bolded** comparisons are selected for GRADEing as they examine a relevant comparator (e.g., placebo, usual care) and population (e.g., general/mixed population of smokers)
^a^ Sham SMS – One text message biweekly thanking participants for involvement, providing study centre contact details, reminder of free month of text messaging if they complete follow-up (whether they quit or not), and time until end of free text service.

| **Author Year {refID};**  **Country;**  **Funding;**  **COI;** | **Date of last search;**  **Databases searched;**  **Total number of studies included in the review;**  **Total number of participants;**  **AMSTAR 2 overall rating** | **Populations for which data is reported** | **Comparisons of relevance to the overview;**  **Intervention delivery;**  **Setting;** | **Outcome(s)** | **General study characteristics** |
| --- | --- | --- | --- | --- | --- |
| **White 2014 {1618};**  UK;  General Practice & Primary Care, Peninsula Medical School; NAFKAM National Research Centre for Complementary Medicine; NHS Research and Development National Cancer Programme; DH National Coordinating Centre for Research Capacity Development;  Review authors declare having conflicts of interest | September/October 2016;  Cochrane Tobacco Addiction Group Specialized Register (CENTRAL, MEDLINE, EMBASE, PsycINFO), AMED, CNKI, Wangfang Data, SinoMed^a^ and VIP;  38;  NR;  AMSTAR 2: Low | Smokers motivated to quit/wishing to quit^b^ | **(1) Acupuncture vs. Wait list/no intervention (2) Acupuncture vs. Sham acupuncture (3) Continuous auricular stimulation vs. Sham stimulation (4) Laser therapy vs. Sham laser (5) Electrostimulation vs. Sham electrostimulation; (6) Acupressure vs. Sham acupressure† (7) Laser therapy vs. wait-list/no intervention† (8) Electrostimulation vs. wait-list†**  (9) Acupuncture vs. NRT (10) Acupuncture vs. Counselling and psychological approaches (11) Acupuncture vs. Interventions of unknown effectiveness  NR;  No setting restriction | Tobacco smoking abstinence/cessation | This review was inclusive of Chinese-language literature. Authors otherwise do not summarize information on patient or report characteristics of included studies. |

Abbreviations: AMED: Allied and Complementary Medicine Database; CKNI: Chinese National Knowledge Infrastructure; NR: not reported; NRT: nicotine replacement therapy

**Bolded** comparisons are selected for GRADEing as they examine a relevant comparator (e.g., placebo, usual care) and population (e.g., general/mixed population of smokers)

†No studies were found addressing these comparisons.

^a^ Formerly Chinese Biomedical Database.

^b^ Population eligibility criterion of review is smokers wishing to quit. However, motivation/wish/interest to quit not explicitly reported in review evidence tables for all included trials.

| **Author Year {refID};**  **Country;**  **Funding;**  **COI;** | **Date of last search;**  **Databases searched;**  **Total number of studies included in the review;**  **Total number of participants;**  **AMSTAR 2 overall rating** | **Populations for which data is reported** | **Comparisons of relevance to the overview;**  **Intervention delivery;**  **Setting;** | **Outcome(s)** | **General study characteristics** |
| --- | --- | --- | --- | --- | --- |
| **Whittaker 2019 {1803};**  New Zealand;  National Institute for Health Innovation (Auckland Uniservices), New Zealand; Cancer Council Victoria, Australia;  Review authors declare having conflicts of interest | October 2018;  Specialised Register of the Cochrane Tobacco Addiction Review Group (CENTRAL, MEDLINE, EMBASE, and PsycINFO);  26;  33,849;  AMSTAR 2: Critically low | Smokers motivated/wishing to quit | **(1) Mobile phone-based interventions vs. Usual care ^a^**  NR;  No setting restriction | Tobacco smoking abstinence/cessation | Three studies used online recruitment methods, one recruited in vocational schools, one from primary care practices, and two from HIV clinics.  Three studies recruited younger participants (mean age 18.2-27 years); age of participants in remaining trials ranged from 30.7 to 44.8 years. Slightly more women in most trials. Occasional smokers were eligible for inclusion in only one trial; remaining trials only included daily smokers. Generally, level of nicotine dependence was similar across trials; however, participants in one trial appeared to have a higher level of dependence.  Most studies conducted in high-income countries. |

Abbreviations: HIV: human immunodeficiency virus; NR: not reported
**Bolded** comparisons are selected for GRADEing as they examine a relevant comparator (e.g., placebo, usual care) and population (e.g., general/mixed population of smokers)

^a^ Comparator is described as ‘usual care’ by review authors in the discussion section of the report. Various control conditions across trials including no intervention, text messages, written/internet untailored materials, untailored messages, and standard cessation advice and treatment.

## Additional file 15.2. Review eligibility criteria

| **Author Year {refID}** | **Eligibility criteria** |
| --- | --- |
| **Barnes, 2019 {3836}** | “Types of studies: Randomized controlled trials.  Types of participants: People who wish to stop smoking, irrespective of gender, number of years smoking, or level of nicotine dependence.  Types of interventions: We considered any trial of hypnotherapy for smoking cessation. We included studies comparing hypnotherapy with no treatment or with any other therapeutic interventions. We reported the type and duration of therapy.  Types of outcome measures: The primary outcome was abstinence (continuous, point prevalence or prolonged) from smoking, assessed at follow up at least six months from the start of treatment. Validated abstinence based on biochemical markers, and abstinence based on self-report by telephone and postal questionnaires were accepted. We also looked for any adverse events reported in the studies.” |
| **Cahill, 2010 {1652}** | “Types of studies: Randomized or quasi-randomized controlled trials.  Types of participants: Smokers, of any age, race or gender.  Types of interventions: Any intervention using a stage-based design to influence a change in smoking behaviour. We have attempted to distinguish where possible, between those studies which substantively modify the intervention to fit the estimated state of change, and those which simply assess participants’ stage of change or use the stage-based model to frame the approach without greatly affecting the content of the intervention. The latter group have not been treated as included studies. The intervention may be compared with a non-stage-based control (lower or equal intensity), or with a no-intervention control or usual care group.  Types of outcome measures: The primary outcome is smoking cessation at least six months after the start of the intervention, and longer wherever the data are reported. Many of the studies in this review are cessation induction studies, i.e. aiming to persuade mostly reluctant quitters to try and stop, and often supporting them as they make a quit attempt. Assessment of abstinence is therefore made after the period of cessation induction, where that distinction is reported. Secondary outcomes include any adverse effects of the interventions. We have not included movement through stages of change as an outcome of interest, since the primary concern of this review and of the included studies is achievement and maintenance of smoking cessation (Prochaska 2006). However, we consider movement through stages of change in the Discussion section, as a marker of the validity of the model for smoking cessation.” |
| **Cahill, 2016 {1960}** | “Types of studies: Randomized controlled trials.  Types of participants: Adult smokers. Trials which target users of smokeless tobacco are not included in this review, but are listed among the Excluded Studies. Interventions for smokeless tobacco use cessation are covered in a companion review (Ebbert 2011).  Types of interventions: Selective nicotine receptor partial agonists, including cytisine, dianicline and varenicline, or any other in this class of drug as they reach Phase 3 trial stage. The efficacy of lobeline is covered in an earlier Cochrane review (Stead 2003). For this update, and in anticipation of current ongoing trials reaching publication, we have extended the range of analyses to cover the following intervention types and subgroups:  I. Varenicline versus other pharmacotherapies:  1. Varenicline versus placebo  2. Varenicline versus bupropion  3. Varenicline versus NRT  4. Varenicline versus mecamylamine  5. Combination treatments (e.g. varenicline + NRT) versus single-therapy treatment, where the addition of varenicline is the intervention being tested  6. Varenicline tablets versus other formulations (e.g. patch, in  solution)  II. Variations in usage:  1. Flexible quit dates  2. Variable dosages  3. Preloading (before TQD)  4. Reducing to quit  5. Maintenance therapy (relapse prevention)  6. Harm reduction  III. Specific patient groups:  1. Cardiovascular disease (CVD)  2. Chronic obstructive pulmonary disease (COPD)  3. Asthma  4. Schizophrenia/bipolar/psychiatric disorder  5. Depression  6. Substance use disorder/methadone-maintained  7. Alcohol-dependent smokers  8. HIV  9. Diabetes  10. Head and neck cancer  11. Varenicline in pregnancy  12. Long-term use of NRT  IV. Settings/subgroups:  1. Hospital inpatients/perioperative patients  2. Smokers who have previously failed to quit on varenicline  or NRT or bupropion  3. Light or heavy smokers  4. Varenicline by gender  5. Varenicline in ethnic groups  We have not considered for inclusion any trials of varenicline used for conditions other than smoking cessation, such as alcoholism, cocaine dependence, Parkinson’s disease, spinocerebellar degeneration, etc.  Types of outcome measures: A minimum of six months abstinence is the primary outcome measure. We have used sustained cessation rates in preference to point prevalence, and we have preferred biochemically verified rates to rates based on self-report of quitting. In analysis, we treat  participants lost to follow-up as continuing smokers. We have recorded any adverse effects of treatment.” |
| **Farley 2012 {1469}** | “Types of studies: Randomized controlled trials  Type of participants: Adult smokers attempting to quit smoking.  Type of interventions: Smoking cessation interventions that are not designed primarily to limit post cessation weight gain but which might plausibly  influence it, i.e. antidepressants, exercise, nicotine replacement therapy (NRT), rimonabant and varenicline.  Type of outcome measures: There are two primary outcome measures: (i) Smoking status six months or more after quitting; (ii)Mean (SD) change in body weight (kg) from baseline to follow-up in abstainers only.*”  * Effects of these interventions on smoking are reported in the parent Cochrane reviews and therefore this review only reports on the effects of interventions on weight change. |
| **Hartmann-Boyce, 2018 {332}** | “Types of studies: Randomized controlled trials. We also include trials where allocation to treatment was by a quasi-randomized method, but use appropriate sensitivity analysis to determine whether their inclusion alters the results.  Types of participants: We include men or women who smoked and were motivated to quit, irrespective of the setting from which they were recruited or their initial level of nicotine dependence, or both. We included studies that randomized therapists, rather than smokers, to offer NRT or a control, provided that the specific aim of the study was to examine the effect of NRT on smoking cessation.  Types of interventions: Comparisons of NRT (including chewing gum, transdermal patches, nasal and oral spray, inhalators and tablets or lozenges) versus placebo or no NRT control. The terms ’inhaler’ and ’inhalator’ (an oral device which delivers nicotine to the buccal mucosa by sucking) are used interchangeably in the literature. We have used the term ’inhalator’ throughout the rest of this review. In some analyses we categorized the trials into groups depending on the level of additional support provided (low or high). The definition of the low-intensity category was intended to identify a level of support that could be offered as part of the provision of routine medical care. If the duration of time spent with the smoker (including assessment for the trial) exceeded 30 minutes at the initial consultation or the number of further assessment and reinforcement visits exceeded two, we categorized the level of additional support as high. The high-intensity category included trials where there were a large number of visits to the clinic or trial centre, but these were often brief, spread over an extended period during treatment and follow-up, and did not include a specific counselling component. To provide a more fine-grained analysis and to distinguish between high-intensity group-based support and other trials within the high-intensity category, we have therefore specified where the support included multi-session group-based counselling with frequent sessions around the quit date.  Types of outcome measures: The review evaluates the effects of NRT versus control on smoking cessation, rather than on withdrawal symptoms. We excluded trials that followed up participants for less than six months, except for trials amongst pregnant women, where the interval between enrolment and delivery may have been shorter (if less than six months, these were excluded from the main analysis). For each study we chose the strictest available criteria to define abstinence. For example, in studies where biochemical validation of cessation was available, we regard only those participants who met the criteria for biochemically-confirmed abstinence as being abstinent. Wherever possible we chose a measure of sustained cessation rather than point prevalence. We regard people who were lost to follow-up as being continuing smokers. For the 2012 update and for this current update we collected data on adverse events in both the included and excluded studies, where they were reported. We have not attempted to pool these findings, apart from one meta-analysis of reports of palpitations, tachycardia or chest pains.” |
| **Hollands, 2015 {916}** | “Types of studies: Randomised, cluster-randomised or quasi-randomised studies in which participants using active pharmacological treatment for smoking cessation were allocated to an intervention arm or a control arm. Pharmacological treatments comprised those that are prescribed to increase cessation rates (e.g. NRT, bupropion, nortriptyline, varenicline and combination regimens).  Types of participants: Adult individuals (18 years and over) defined as smokers at point of entry into the trial.  Types of interventions: Interventions to increase adherence may vary significantly in their nature, with a workable taxonomy provided in a previous Cochrane review (Haynes 2008). This taxonomy is provided in Appendix 1. The nature of the interventions considered in the current review was not specified beyond reference to exclusion criteria. Eligible interventions comprised any intervention that differed from standard care administered to smokers, and where the differing intervention content had a clear principal focus on increasing adherence to medications for tobacco dependence, reflected in described content and stated aims. We did not include interventions that systematically alter the active pharmacological characteristics of a given medication, such as dose strength, length of treatment or means of delivery. Interventions that include the use of financial incentives were not eligible. Acceptable comparison groups were those that provided standard or usual care. Depending on setting, this can comprise minimal support or varying degrees of behavioural support.  Types of outcome measures: To be considered for inclusion, studies must have used a measure of adherence behaviour allowing some assessment of the degree of adherence. This was defined as a continuous measure - such as the amount of medication consumed over a given treatment period - or as a dichotomous outcome, indicating whether the treatment is being used to a specified degree (e.g. adherence for x number of days, or x amount of medication consumed). This is in contrast to a single binary measure without nuance (i.e. any amount of medication at any time vs. non-use), which was not considered an appropriate measure. Adherence could be measured by means of a behavioural endpoint using an electronic measure, pill counts by a third party, or through a self-report or questionnaire measure (or combinations thereof  Primary outcomes Primary outcome:  • Adherence to medication for tobacco dependence Where treatment periods were assessed at multiple timepoints, the longest timepoint reported was used. Where multiple measures of adherence were reported, we have used the most stringent measure that is available.  Secondary outcomes:  • Abstinence from smoking measured near or at a time point relevant to the measure of adherence Where multiple measures of abstinence were reported, we used the most stringent. If there were data from multiple timepoints, we report data measured near or at a timepoint relevant to the measure of adherence. In addition, we also report abstinence at the longest available timepoint should that differ, in order to assess the long-term benefit of the intervention on cessation rates.  • Factors plausibly associated with increases in adherence such as, but not limited to: - intention or motivation to change health behaviour - attitudes towards treatment, or understanding of the treatment  • Adverse events Any adverse events or harms reported in included trials were noted, including clinical levels of depression or anxiety.” |
| **Hughes 2014 {1147}** | “Types of studies: For efficacy, we examined randomized trials comparing antidepressant with placebo or with an alternative therapeutic control,  or comparing different dosages of an antidepressant, that reported six-month or longer follow-ups. For safety, we examined data from randomized controlled trials comparing antidepressant with placebo or no pharmacotherapy controls, and also considered observational data. Studies were included irrespective of their publication status and language of publication.  Type of participants: Current cigarette smokers, or recent quitters (for trials of relapse prevention).  Type of interventions: Treatment with any medication with antidepressant properties to aid a smoking cessation attempt or to prevent relapse, or to reduce the number of cigarettes smoked and aid subsequent cessation. Trials in which all participants received the same pharmacotherapy regimen but different behavioural support were not included.  Type of outcome measures: Efficacy was measured via a) abstinence from smoking or b) incidence of reducing cigarette consumption to 50% or less of baseline, both assessed at follow-up at least six months from start of treatment. Safety was assessed by incidence of serious and other adverse events, and drop-outs due to adverse events.” |
| **Khanna, 2016 {310}** | “Types of studies: We considered all relevant randomised controlled trials (RCTs) and economic evaluations conducted alongside any included RCTs. We excluded quasi-randomised studies, such as studies that allocated participants by using alternate days of the week. If we had encountered trials that suggested or implied the trial was randomised and where the demographic details of each group’s participants were similar, we would have included them and conducted a sensitivity analysis to evaluate the effect of the presence or absence of these data. We have summarised the literature screening process in Figure 3.  Type of participants: A requirement was that a majority of participants were within the age range 18 to 65 years and suffering from serious mental illness, preferably as defined by National Institute of Mental Health (NIMH 1987), but in the absence of that, from diagnosed illness such as schizophrenia, schizophrenia-like disorders, bipolar disorder, or serious affective disorders. If the trials included participants with a range of serious mental illness we would have included them if the majority had schizophrenia, we would not have included trials that only randomised people with bipolar or serious affective disorders. We did not consider substance abuse to be serious mental illness in its own right, however those dealing with a dual diagnosis population i.e. those with serious mental illness plus substance abuse were eligible. We would not have included studies focusing on dementia, personality disorder and mental retardation, as these are not covered by our definition of serious mental illness. Despite the fact that personality disorder is now included in the NIMH 1987 definition we intended to exclude it from this review for the following reasons; the diagnosis  of personality disorder has low interrater reliability (Zimmerman 1994); the duration of treatment can be assessed much more precisely than duration of illness (Schinnar 1990); there is insufficient information given on how to operationalise the disability criterion in both the original NIMH 1987 definition and in the further work of Schinnar 1990.  Type of interventions:   1. **Smoking cessation advice:** We have found it difficult to find a useful definition of ’advice’. In the context of this review we define ’advice’ as preventative information (Greenlund 2002) or counsel (OED) that leaves the recipient to make the final decision. Advice may be directional but not paternalistic in its delivery. We do not consider that programmes of learning and educational or training groups fall into the definition of ’advice’. Advice should have at least a suggestion of: i. an educative component; ii. a preventative aim; and iii. an ethos of self-empowerment. We did not consider effects of training programmes as these are the focus of another Cochrane review (Tsoi 2013). 2. **Standard care:** Care in which smoking cessation advice is not specifically emphasised above and beyond the care that would be expected for people suffering from serious mental illness.   Type of outcome measures: For the purposes of this review we aimed to divide outcomes into four time periods: i. immediate (within one week); ii. short term (one week to six months); iii. medium term (six months to one year); and iv. long-term (over one year).  **Primary Outcomes:**   1. Smoking cessation awareness: 1.1 Raised awareness of common problems associated with smoking 2. Smoking behaviour: 2.1 Substantial reduction in smoking behaviour 3. Quality of life: 3.1 Healthy days   **Secondary Outcome:**  1. Adverse events:  1.1 Number of participants with at least one adverse effect  1.2 Clinically important specific adverse effects (withdrawal, irritability, weight gain, reduced appetite, insomnia, anxiety, craving, depression, decreased concentration)  1.3 Average endpoint in specific adverse effects  1.4 Average change in specific adverse effects  1.5 Death: natural or suicide  2. Service use:  2.1 Hospital admission  2.2 Emergency medical treatment  2.3 Use of emergency services  3. Financial dependency:  3.1 Claiming unemployment benefit  3.2 Claiming financial assistance because of a physical disability  4. Social:  4.1 Unemployment  4.2 Social isolation as a result of preventable incapacity  4.3 Increased burden to caregivers  5. Economic:  5.1 Increased costs of health care  5.2 Days off sick from work  5.3 Contribution to society  5.4 Family claiming carers’ allowance  6. Leaving the trial early:  6.1 Any reason  6.2 Adverse events  6.3 Inefficacy of treatment  7. Quality of life:  7.1 Loss of independence  7.2 Loss of skills in activities of daily living (ADL)  7.3 Loss of earnings  7.4 Loss of social status  8. Global state:  8.1 Clinically important change in global state (as defined by individual  trials)  8.2 Relapse (as defined by the individual trials)  9. Mental state:  9.1 Clinically important change in general mental state score  9.2 Average endpoint general mental state score  9.3 Average change in general mental state score  9.4 Clinically important change in specific symptoms (positive  symptoms of schizophrenia, negative symptoms of schizophrenia)  9.5 Average endpoint specific symptom score  9.6 Average change in specific symptom score.” |
| **Lancaster 2017 {539}** | “Types of studies: Randomized or quasi-randomized controlled trials (RCTs) with a minimum follow-up of six months, where at least one treatment arm consisted of an unconfounded intervention from a counsellor.  Types of participants: Any smokers, except pregnant women (smoking cessation interventions in pregnancy are addressed by a separate review, Chamberlain 2013). We also exclude trials recruiting only children and adolescents.  Types of interventions: We defined individual counselling as a face-to-face encounter between a smoker and a counsellor trained in assisting smoking cessation. We include studies that evaluate the effect of counselling as an addition to pharmacotherapy. We include studies comparing different counselling approaches if they are not covered by other Cochrane Reviews of specific interventions. Comparisons between individual counselling and behavioural therapy conducted in groups are covered in the Cochrane Review of group behavioural therapy (Stead 2017).  Types of outcome measures: The outcome was smoking cessation at the longest reported follow-up. We used sustained abstinence where available, or multiple point prevalence. We included studies using self-report with or without biochemically-validated cessation, and performed sensitivity analyses to determine whether the estimates differed significantly in studies without verification.” |
| **Lindson-Hawley, 2016 {671}** | “Types of studies: Randomized or quasi-randomized controlled trials.  Types of participants: People who smoke tobacco, but have no immediate intention to quit all tobacco use. We included trials which did not assess motivation if an aim was to reduce cigarette consumption, but not to quit entirely.  Types of interventions: Interventions to reduce the amount smoked, or to reduce harm from smoking by means other than cessation, including switching to a potential reduced-exposure product (PREP), or making other changes to cigarette characteristics. We excluded interventions where a reduction in the number of cigarettes smoked over a short period, or a change in type of cigarette smoked (e.g. nicotine fading), was intended as a precursor to quitting completely. We deemed studies eligible for inclusion if they compared these interventions to any ’standard control’, such as brief advice, no treatment or placebo, or compared one type of harm reduction intervention to another.  Types of outcome measures:  Primary outcomes  The preferred primary outcome was long-term change in health status, but we expected that this was unlikely to be assessed in randomized trials. The most appropriate proxy indicator to demonstrate a reduction in toxin intake from tobacco use, sufficient to lead to a clinically useful long-term health benefit is not known (Hatsukami 2005a). We have therefore considered and extracted any attempt made to measure a health marker. In the absence of better health indicators, we have also assessed both the change in smoking rate from baseline and smoking cessation as primary outcomes. For smoking reduction outcomes, we preferred prolonged or continuous rates to point prevalence rates. Where studies did not incorporate abstinent participants in their reduction rates we have done this for the purposes of our metaanalyses, as participants who have quit smoking have by definition reduced to zero. As there was no expectation that participants would quit at the start of the intervention, we have favoured measures of abstinence based on behaviour towards the end of the follow-up period (i.e. point prevalence rates) over continuous or sustained abstinence rates (Hughes 2003). We preferred biochemically-validated rates to self-reported rates for both reduction and cessation outcomes. To be eligible for inclusion, a study had to report at least one of these outcomes at least six months following baseline.  Secondary outcomes  We extracted information on any biochemical indicators of the amount of tobacco use, and on adverse events when the intervention being tested included the use of a pharmaceutical, nicotine or tobacco-based substitute for tobacco smoking.” |
| **Livingstone-Banks, 2019 {1077}** | “Types of studies: We sought randomised controlled trials with a minimum follow-up of six months, where at least one arm comprised a print-based  self-help intervention without repeated face-to-face therapist contact compared with another print-based self-help intervention or with a minimal control. We included studies that allocated participants to treatment via a quasi-randomised method, but, where appropriate, we used sensitivity analysis to determine whether inclusion of these studies altered the results.  Type of participants: We included any smokers except pregnant smokers and adolescent smokers. Separate Cochrane Reviews have evaluated interventions in pregnant smokers (Coleman 2015; Chamberlain 2017), and in adolescent smokers (Fanshawe 2017).  Type of interventions: We defined a ’self-help intervention’ as any manual or programme designed to be used by individuals to assist a quit attempt not aided by health professionals, counsellors, or group support. This review primarily covers written materials such as booklets and leaflets, but information could also have been provided via audio or video or a similar medium. Separate reviews cover interventions designed to be delivered via the internet, or via mobile phone (Taylor 2017; Whittaker 2016). Materials could be aimed at smokers in general; could target particular populations of smokers, for example, those of different ages or ethnic groups; or could be tailored to individual smoker characteristics. We did not include brief leaflets on the health effects of smoking - we considered them to be a control intervention if compared with a more substantial manual. We considered interventions with a single session of minimal face-to face contact for the purpose of supplying the self-help programme materials as self-help alone. Where a face-to-face meeting included discussion of programme content, we categorised this as brief advice in addition to self-help materials. We excluded interventions that provided repeated sessions of advice in addition to self-help materials. Separate Cochrane Reviews cover telephone counselling or hotlines as adjuncts to self-help materials (Stead 2013b), and interventions aimed at relapse prevention (Hajek 2013).  Type of outcome measures: We used sustained abstinence, or point prevalence, where available. We included studies that used self-report of cessation alone or biochemically validated cessation.” |
| **Matkin, 2019 {1228}** | “Types of studies: Randomised or quasi-randomised controlled trials (RCTs), with the unit of allocation being one of the following: the individual  smoker; counsellor; group; intervention site; or geographical area.  Types of participants: Individuals who were current smokers at the time of inclusion in the trial. We included trials with a mixture of current smokers and recent quitters if the recent quitters were only a small proportion of the entire study population. The definition of recent quitters was that used by the trial recruitment protocols, or by the participants themselves. We excluded trials that exclusively recruited quitters or were focused on telephone counselling as an intervention for relapse, as they fall within the scope of a separate Cochrane Review on preventing relapse (Livingstone-Banks 2019b). We included trials recruiting exclusively teenagers or pregnant women, but we considered them as a potential source of heterogeneity in meta-analyses. There are separate Cochrane Reviews for these population groups (Chamberlain 2017; Fanshawe 2017).  Types of interventions: Provision of proactive or reactive telephone counselling to assist smoking cessation, to any population. We excluded studies if the contribution of the telephone component could not be evaluated independently of another therapy. We included studies that compared a combination of telephone counselling and self-help materials versus no telephone counselling, as the effect of self-help materials alone is limited (Livingstone-Banks 2019a). We also included studies in which the effect of telephone counselling as an adjunct to another smoking cessation treatment was evaluated, e.g. print-based self-help, brief face-to-face intervention, pharmacotherapy, or incentives. We also included studies that compared different modalities or strategies of telephone counselling, and different theories of behavioural change.  Types of outcome measures: Long-term smoking cessation (i.e. at least six months after the start of intervention). We excluded trials with shorter follow-up. We used the strictest definition of smoking cessation available in a trial and biochemically-validated abstinence data whenever available.” |
| **Posadzaki 2016 {659}** | “Types of studies: We included RCTs, cluster RCTs, quasi-RCTs, interrupted time series (ITS) and controlled before-and-after (CBA) studies. We included CBA and ITS studies because they are often used to draw conclusions about ’promising interventions’ ready for trial when RCTs may be too expensive or simply impractical or where there are insufficient RCTs on a particular type of intervention (Centre for Reviews and Dissemination 2008; Higgins 2011; Jackson 2005). Interrupted time series designs can address cyclical trends (i.e. the outcome may be increasing or decreasing over time such as seasonal or other cyclical observations). To be considered for inclusion, these studies must have met the criteria specified by the Cochrane Effective Practice and Organisation of Care Review Group (EPOC) (Ryan 2009). For CBA designs, the timing of data collection for the control and intervention groups had to have been the same, there must have been at least two intervention sites and two control sites, and both groups would have been comparable on key characteristics related to demographics and intervention context. For ITS designs, the studies had to use a clearly defined point in time when the intervention occurred and at least three data points before and three after the intervention.  Type of participants:  • We included consumers, including carers, who received ATCS for prevention or management of long-term conditions, regardless of age, sex, education, marital status, employment status, or income.  • For management of long-term conditions, we included consumers who had one or more concurrent long-term conditions (i.e. multimorbidity).  • We included consumers in all settings.  Type of interventions: The ATCS interventions included in this review included the following.  • Unidirectional ATCS: non-interactive ATCS enabling one-way voice communication.  • Interactive ATCS: systems that enable two-way, real-time communication, such as interactive voice response systems or IVR.  • ATCS Plus: interactive ATCS systems including additional functions.  The review also included several multimodal/complex ATCS interventions, defined as any type of ATCS (unidirectional, IVR or ATCS Plus) delivered as part of a complex, multimodal package. We included studies that evaluated either unidirectional ATCS or interactive ATCS. We also included studies that compared ATCS interventions (e.g. unidirectional ATCS versus interactive ATCS and/or ATCS Plus) to compare the effects of different intervention designs on preventive healthcare or management of long-term conditions. Interactive ATCS had an automated function such as automated tailored feedback based on individual progress monitoring (e.g. comparison to norms or goals, reinforcing messages, coping messages, and automated follow-up messages). Although our protocol (Cash-Gibson 2012) indicated that we would include ATCS Plus interventions only if the study explicitly reported that the effects of the intervention could be attributed to the ATCS component, in the review we included all types of ATCS Plus interventions as, in a complex intervention such as this, it would be impossible to attribute the intervention effect to one of the intervention components. We also included studies that delivered any type of ATCS (unidirectional, IVR, or ATCS Plus) as part of a complex, multimodal (package) intervention. The interventions were delivered for one or more types of prevention or one or more types of management for long-term conditions, as illustrated in Figure 1 and Figure 5, respectively.  We excluded studies in which interventions:  • targeted health professionals or teachers for educational purposes;  • were exclusively for the purpose of electronic history-taking or data collection or risk assessment with no health promotion or interactive elements;  • involved only a non-ATCS component such as face-to-face communication or written communication;  • were web-based interventions that were accessed via a mobile phone.  Comparisons were made against various controls or standard or enhanced forms of usual care (i.e. no ATCS intervention). We also included comparisons of one type of ATCS against another, or the same type of ATCS that was delivered via different delivery modes (e.g. landline telephone versus mobile phone). As part of this review, we piloted and applied the intervention Complexity Assessment Tool for Systematic Reviews version 1 (iCAT˙SR) for assessing complex, multimodal interventions and reported results narratively/qualitatively (Lewin 2015).  Type of outcome measures:  Primary outcomes  Primary outcomes consisted of health behaviour and clinical outcomes (defined below). For each study, we included all relevant primary outcomes, as these are likely to be most meaningful to clinicians, consumers, the general public, administrators and policymakers (Chandler 2013). Given the wide spread of the included studies and the fact that this review represents the first attempt to systematically assess all relevant evidence on broadly defined ATCS interventions, we felt that it was important to capture and report as much relevant information on outcomes and effects of interventions as possible, in order to assist with comprehensively mapping where the evidence lies and how it has been assessed. In future updates to this review, we may consider modifying this approach to focus on a smaller number and range of outcomes if this is likely to improve the clarity and meaningfulness of the collected data. We reported the following outcomes in ’Summaries of findings’ tables.  1. Health behaviour outcomes (category)  • Changes in health-enhancing behaviour (e.g. physical activity, adherence to medications/uptake of recommended laboratory or other testing)  • Risk-taking behaviour (e.g. tobacco consumption). This outcome was either self-reported or collected using a validated questionnaire that was either self-administered or completed in an interview. In studies that measured the same outcome using both a self-reported measure and an objective measure, we used the objective measure. For example, if a study on physical activity measured Metabolic Equivalent of Task (MET) scores using a self reported, seven-day physical activity recall as well as a pedometer, we used the score obtained from the (objective) pedometer  2. Clinical outcomes (category)  • Physiological measures (e.g. blood pressure)  • Blood biochemistry (e.g. glucose levels)  Secondary outcomes  For each study, we selected all relevant secondary outcomes as these were also meaningful for the various stakeholders.  1. Process outcomes (category)  • Change in acceptability of service (e.g. consumer accessibility and usability of the interventions to apply information and support supplied through ATCS)  • Satisfaction (e.g. patient and carer satisfaction with the intervention)  • Cost-effectiveness  2. Cognitive outcomes (category)  • Changes in knowledge (i.e. accurate risk knowledge and perception)  • Attitude and intention to change  • Self-efficacy (i.e. a person’s belief in their capacity to carry out a specific action)  3. Patient-centred outcomes (category)  • Quality of life  4. Adverse outcomes  • Unintended adverse events attributable to the intervention” |
| **Stead, 2013 {1998}** | “Types of studies: Randomised controlled trials. Trials where allocation to treatment was by a quasi-randomised method were also included, but appropriate sensitivity analysis was used to determine whether their inclusion altered the results. Studies which used historical controls were excluded  Types of participants: Participants could be smokers of either gender recruited in any setting, the only exception being trials which only recruited pregnant women. These were excluded since they are reviewed elsewhere (Lumley 2009).  Types of interventions: We included trials if they compared physician advice to stop smoking versus no advice (or usual care), or compared differing levels of physician advice to stop smoking. We defined advice as verbal instructions from the physician with a ’stop smoking’ message irrespective  of whether or not information was provided about the harmful effects of smoking. Therapists were physicians, or physicians supported by another  healthcare worker. Trials which randomised therapists rather than smokers were included. We defined trials where advice was provided (with or without a leaflet) during a single consultation lasting less than 20 minutes plus up to one follow-up visit as minimal intervention. We defined  a trial as intensive when the intervention involved a greater time commitment at the initial consultation, the use of additional materials other than a leaflet, or more than one follow-up visit. We considered adjunctive aids to advice as additional strategies other than simple leaflets (e.g. demonstration of expired carbon monoxide or pulmonary function tests, self help manuals).  Types of outcome measures: The principal outcome used in the review was smoking cessation rather than reduction in withdrawal symptoms, or reduction in amount of cigarettes smoked. In each study we used the strictest available criteria to define abstinence. That is, we used rates of sustained cessation rather than point prevalence abstinence where possible. Where biochemical validation was used, we classified only those people meeting the biochemical criteria for cessation as abstainers; and where participants were lost to follow-up, they were regarded as continuing smokers. We required a minimum follow-up of at least six months for inclusion, and used the longest follow-up reported. A secondary outcome was the effect of smoking advice on subsequent mortality and morbidity.” |
| **Stead 2016 {1356}** | “Types of studies: Randomized or quasi-randomized controlled trials. We did not exclude studies on the basis of publication status or language of publication.  Types of participants: We included trials that recruited people who smoke in any setting, with the exception of trials which only recruit pregnant women or adolescents. These populations are considered in specific reviews. Trial participants did not need to be selected according to their interest in quitting or their suitability for pharmacotherapy.  Types of interventions: We included interventions for increasing smoking cessation that included behavioural support and the availability of pharmacotherapy, regardless of type of pharmacotherapy. We excluded trials where fewer than 20% of participants were eligible for or used pharmacotherapy. The provision of written information or brief instructions on correct use of the pharmacotherapy was not regarded as behavioural support. The control group should not have been systematically offered pharmacotherapy but we did not exclude studies where some control group participants obtained medication from other sources. Control group participants could be offered usual care, self-help materials or brief advice on quitting, but support had to have been of a lower intensity than that given to intervention participants.  Types of outcome measures: Following the standard methodology of the Cochrane Tobacco Addiction Group, the primary outcome is smoking cessation at the longest follow-up using the strictest definition of abstinence, that is, preferring sustained over point prevalence abstinence and using biochemically validated rates where available. We also noted any other abstinence outcomes reported and conducted sensitivity analyses to test if the choice of outcome affected the results of meta-analysis. We excluded trials reporting less than six months follow-up from the start of intervention.” |
| **Stead 2017 {538}** | “Types of studies: Trials were eligible for inclusion if participants were randomly allocated to treatment conditions. We included trials of worksite smoking cessation programmes which randomized worksites to different programmes. We also included studies that randomized therapists, rather than smokers, to offer group therapy or control, provided that the specific aim of the study was to examine the effect of group therapy on smoking cessation.  Type of participants: Adult smokers of either gender, irrespective of their initial level of nicotine dependency, recruited from any setting, with the exception of trials recruiting pregnant women in antenatal care settings, since interventions for pregnant women are reviewed separately (Chamberlain 2013). Interventions recruiting only adolescent smokers are also reviewed separately (Grimshaw 2013).  Type of interventions: We considered studies in which smokers met for scheduled meetings and received some form of behavioural intervention, such as information, advice and encouragement or cognitive behavioural therapy (CBT) delivered over at least two sessions. We excluded studies of interventions where participants met once for an orientation or information session. We excluded studies which covered group meetings but which were primarily investigating the efficacy of aversive smoking, acupuncture, hypnotherapy, exercise or partner support, unless there were other relevant arms. Trials investigating these specific components have been separately reviewed by Hajek 2001, White 2014, Barnes 2019, Ussher 2014 and Park 2012 respectively. We exclude trials of components to prevent relapse, as they are covered by a separate review (Hajek 2013). Trials in which smokers received group therapy in addition to active or placebo pharmacotherapy were excluded unless there were other relevant arms. The effect of nicotine replacement therapy (NRT) is evaluated in a separate review (Stead 2012), but we include studies which tested group therapy as an adjunct to nicotine replacement.  Type of outcome measures: The main outcome was abstinence from cigarettes at follow-up at least six months after the start of treatment. We excluded trials that reported only shorter follow-up or had no measurement of smoking cessation. In each study we used the strictest available criteria to define abstinence. For example, in studies where biochemical validation of cessation was available, we counted as abstinent only those participants who met the criteria for biochemically-confirmed abstinence. Wherever possible, we used a sustained cessation rate, rather than point prevalence. Where participants were lost to follow-up, we regarded them as being continuing smokers.” |
| **Taylor, 2017 {411}** | “Types of studies: Randomised or quasi-randomised controlled trials. Examples of quasi-random methods of assignment include alternation, date of birth, and medical record number. There were no restrictions by language.  Types of participants: Current smokers, with no exclusions by age, gender, ethnicity, language spoken or health status. We analyse studies in adolescents and young adults separately from the studies in adults, as both subgroups have particular needs which warrant separate investigation.  Types of interventions: We included studies evaluating Internet interventions in all settings and from all types of providers. There was no exclusion by intervention method or duration. We included trials where the Internet intervention was evaluated with an additional behavioural intervention/support component, or delivered alongside pharmacotherapy such as nicotine replacement therapy (NRT), bupropion or varenicline. The trials compared different types and combinations of intervention. The trials compared Internet-based programmes to no treatment or to other forms of treatment, such as self-help booklets. We included trials of interactive, tailored and non-interactive interventions that focused on standard approaches to information delivery. Interactive interventions were not necessarily personalised. We defined tailored interventions as programmes that were adapted to a participant’s characteristics, and interactive interventions as those which involved a two-way flow of information between the Internet and the participant. Personalised interventions can vary considerably, from minimal personalisation to those which have been developed based on theoretical models relevant to desired treatment outcomes, such as self efficacy. The interventions used in each study were fully described, illustrating the heterogeneity of the interventions (e.g. in relation to varying content, intensity, number of sessions, and duration of contact time). We excluded trials that used the Internet solely for recruitment and not for delivery of smoking cessation treatment. We also excluded trials where Internet-based programmes were used to remind participants of appointments for treatment that is not conducted online (e.g. face-to-face counselling, or pharmacotherapy). Text messaging, and smart-phone application interventions are covered in a Cochrane Review of mobile phone interventions (Whittaker 2016), and a review of video-based interventions is currently in progress with the Cochrane Tobacco Addiction Group (Tzelepis 2017). We therefore do not address these interventions in this review.  Types of outcome measures: The primary outcome is smoking cessation at least six months after the start of the intervention, and longer wherever the data were available. Where studies did not have follow-up of six months or longer, we report shorter-term outcomes narratively. We preferred sustained or prolonged cessation over point prevalence abstinence, but did not exclude studies which only reported the latter. We included studies that relied on self-reported cessation, as well as those that required biochemical validation of abstinence, but preferred biochemically validated rates where available. Where reported, we extracted data on user satisfaction rates, intervention costs, adverse outcomes, use of the Internet site or programme use, self-efficacy, use of NRT or other pharmacotherapies, reductions in the number of cigarettes or in smoking frequency, and the impact of Internet programme completion on smoking cessation.” |
| **Tsoi, 2013 {1698}** | “Types of studies: We included randomised controlled trials (RCTs) or quasi-randomised controlled trials.  Type of participants: We included adult smokers with a current diagnosis of schizophrenia according to the criteria of the International Classification of Diseases (ICD) (World Health Organization 2003) or Diagnostic and Statistical Manual of Mental Disorders (DSM) (American Psychiatric Association 1994). Smokers with a diagnosis of schizoaffective disorder were also included, because certain core symptoms are the same as in schizophrenia. We did not exclude patients with a diagnosis of schizophrenia or schizoaffective disorder who had other substance misuse disorder or additional psychiatric disorders, as individuals with schizophrenia have high prevalence of substance misuse disorders (Dixon 1999). If a study was conducted in a group of participants with mixed psychiatric diagnoses, we included that trial only when separate data for people with schizophrenia or schizoaffective disorder were available. We included people who may or may not have expressed an interest in stopping or reducing smoking. We reported whether or not participants in a study wanted to stop or reduce smoking.  Type of interventions: We included both pharmacological and non-pharmacological interventions (alone or in combination) specific to smoking cessation or reduction. We included interventions intended for another purpose (e.g. antipsychotics for treating schizophrenia) if smoking abstinence or reduction outcomes were reported. We reported the results of these trials separately and they did not contribute to any meta-analysis, since they were not designed to test the efficacy of the intervention for smoking cessation or reduction. The control condition could be another intervention (pharmacological or nonpharmacological), placebo, or usual care.  Type of outcome measures:  **Primary outcome:**  Smoking abstinence at longest follow-up: The primary outcome was abstinence from smoking assessed at least sixmonths fromthe start of the intervention, according to the ’Russell Standard’ (i.e. a common standard for outcome criteria in smoking cessation trials; West 2005). The United States Department of Health and Human Services (USDHHS) Tobacco Use and Dependence Guideline Panel also suggested a minimum of six months as an adequate period of abstinence to assess treatment differences in the longer term (Fiore 2008). Abstinence could be assessed by self report or with biochemical verification. For data synthesis, we chose the strictest definition of abstinence in each trial, preferring sustained abstinence over point prevalence if both were reported. In studies that used biochemical validation of abstinence, only people whose self reports could be validated were classified as abstinent.  Changes in mental state: Change in mental state was measured by change in positive symptoms (e.g. hallucinations, delusions), negative symptoms (e.g. anhedonia, avolition), and depressive symptoms.  **Secondary outcome:**  Smoking abstinence at the end of the intervention: This was measured as for the primary abstinence outcome.  Reduction of smoking behaviour or dependence: This was assessed at the end of the intervention and during the follow-up period after the end of the intervention, if data were available. Measures could include any of the following: percentage change in cigarettes per day (CPD) from baseline level; absolute number of cigarettes foregone; incidence of achieving at least a 50% reduction in CPD; reduction of expired carbon monoxide (CO) level; or reduction of scores on scale measures of nicotine dependence (e.g. Fagerström Test for Nicotine Dependence (FTND)).  Other adverse events: We recorded and assessed any other reported adverse events.” |
| **van der Meer 2013 {1223}** | “Types of studies: We examined randomised controlled trials (RCTs).  Type of participants: We included adult smokers with current or past depression. Current depression was defined as current major depression (according to the DSM-IV criteria) or depressive symptoms (use of multi item scales as measures of depression, for example Beck Depression Inventory or Center for Epidemiologic Studies Depression Scale, or use of a single item question as a measure of depression). Past depression was defined as past major depression (according to the DSM-IV criteria) or depressive symptoms (use of multi-item scales as measures of depression or use of a single item question as a measure of depression). Studies with subgroups (pre-stated stratification or post hoc) of participants with current or past depression were also included when separate data were available. By ’pre-stated’, we mean that researchers explicitly state, a priori, that they want to analyse the effectiveness of the smoking cessation intervention for the depression subgroup. By ’post hoc’, we mean that the researchers did not explicitly state this analysis a priori.  Type of interventions: RCTs were included when the effectiveness of any pharmacological or psychosocial intervention, or a combination of both types, was assessed as an aid to smoking cessation in smokers with current or past depression. Comparisons investigated (separately for smokers with past or current depression) the following.  *Interventions with specific mood management components for depression*  • Psychosocial mood management versus control  • Antidepressants for smoking cessation versus placebo  Psychosocial mood management was defined as: group or individual counselling, self help or exercise, or hypnosis intended to influence negative mood and improve depression symptoms above and beyond standard smoking cessation counselling.  *Interventions without specific mood management components for depression*  • Psychosocial interventions versus standard smoking cessation counselling  • Nicotine replacement therapy (NRT) versus placebo  • Other pharmacotherapy (no antidepressants or NRT) versus placebo  • Other interventions (smoking cessation interventions that did not fit within one of the other comparisons)  Type of outcome measures:  The primary outcome was smoking status at a minimum of six months from the quit day. We used sustained cessation rates in preference to point prevalence, where available. By sustained cessation we meant either continuous abstinence from quit date or prolonged abstinence (continuous abstinence following a short period of grace in which lapses are not regarded as treatment failure) (Hughes 2003). We used biochemically validated cessation in preference to self-reported quitting. The strictest definition of smoking cessation reported was used in the meta-analysis. We examined  types of abstinence (continuous, prolonged, point prevalence) and levels of verification (biochemical, self report) as potential modifiers of outcome. Participants lost to follow-up were assumed to be continuing smoking.” |
| **Vodopivec-Jamsek, 2012 {1343}** | “Types of studies: We included randomised controlled trials (RCTs), quasi-randomised controlled trials (QRCTs), controlled before and after studies (CBA) and interrupted time series (ITS) with at least three time points before and after the intervention. We define QRCT as a controlled trial in which the participant allocation is not truly random, such as allocation by date of birth or the order in which participants are included in the study. We  included QRCT, CBA and ITS designs because our initial literature searching suggested that only a small number of RCTs on mobile phone messaging interventions exist.  Types of participants: We included all study participants regardless of age, gender and ethnicity, as well as all types and stages of diseases. We included studies in all settings i.e. primary care settings (services of primary health care), outpatient settings (outpatient clinics), community settings (public health services, anywhere where a person can use a mobile phone) and hospital settings. We did not exclude studies according to the type of healthcare provider (e.g. nurse, doctor, allied staff).  Types of interventions: We included interventions using SMS or MMS as a mode of delivery for any type of preventive health care. The messaging needed to be between healthcare provider (either in person or automated) or a ’treatment buddy’ (i.e. lay health worker or peer supporter) and participant, regardless of who sent the first message. We excluded studies of mobile phone messaging to people other than those who were about to take part in preventive health care, or messaging between two healthcare providers. We also excluded studies in which mobile phone messaging was a part of a multifaceted intervention, as it would not be possible to separate the effects of messaging alone. We aimed to make comparisons between mobile phone messaging and no intervention, as well as other modes of communication such as face-to-face, postal letters, calls to land line or mobile, email or via electronic health records; and if applicable, automated  versus personal text messaging.  Types of outcome measures: A number of processes and outcomes may be affected by mobile phone messaging interventions that aim to facilitate communication between healthcare service users and healthcare providers.  **Primary outcomes**  As primary outcomes we included all outcomes related to health status or health behaviour, such as adoption of healthier lifestyles (e.g. smoking cessation, increased physical activity, weight control, nutrition and stress management), or improved quality of life.  **Secondary outcomes**  • User (patient, carer or healthcare provider) evaluation of the intervention, including satisfaction, readiness to use, timeliness, availability and/or convenience;  • Health service utilisation following the intervention;  • Costs (direct and indirect) of the intervention;  • User (patient, carer or healthcare provider) perceptions of  safety;  • Potential harms or adverse effects of the intervention, such as misreading or misinterpretation of data, transmission of inaccurate data, loss of verbal and non-verbal communication cues, issues of privacy and disclosure, or failure or delay in the message delivery.” |
| **White 2014 {1618}** | “Types of studies: All randomized controlled trials comparing acupuncture, acupressure, laser therapy or electrostimulation with either no intervention, or a sham form of the intervention, or another intervention for smoking cessation.  Type of participants: Tobacco smokers of any age who wished to stop smoking.  Type of interventions: Non-pharmacological stimulation interventions involving needle puncture, finger pressure or laser therapy in areas of the body described by the study’s author as acupuncture points, which includes points on the ear, face and body, or the related intervention of electrostimulation to the head region, through surface electrodes. Had we located any, studies using a Western acupuncture approach would have been considered separately from those using a traditional approach.  Type of outcome measures: Complete abstinence from smoking. The review has not been limited to studies where the outcome was confirmed biochemically.” |
| **Whittaker, 2016 {1803}** | “Types of studies: Randomised or quasi-randomised trials.  Types of participants: Any smokers who want to quit smoking.  Types of interventions: We included studies that examined any type of mobile phone based intervention for smoking cessation. This included any intervention aimed at mobile phone users, based around delivery via mobile phone, and using any functions or applications that could be used or sent via a mobile phone. We excluded trials where mobile phones were seen as an adjunct to face-to-face or Internet- based programmes, such as to remind participants of appointments or where the effects of the various components of a multifaceted programme could not be separated.  Types of outcome measures: The primary outcome was smoking abstinence at six months or longer from the start of the intervention. When available, we preferred sustained abstinence to point prevalence abstinence and biochemically validated results to self report.” |
