# Supplementary material for: Effectiveness of smoking cessation interventions among adults: an overview of systematic reviews
Source: Syst Rev. 2024 Jul 12;13:179. doi: 10.1186/s13643-024-02570-9 (PMC11242003; doi:10.1186/s13643-024-02570-9)
Supplement: Supplementary file 16 — Additional file 16. AMSTAR 2 rating of included reviews. [file 13643_2024_2570_MOESM16_ESM.docx]

## Additional file 16. AMSTAR 2 ratings for included systematic reviews

| **Author; Year**  **{RefID}** | **AMSTAR 2 Items** | | | | | | | | | | | | | | | | | | **Overall AMSTAR Rating** |
| --- | --- | --- | --- | --- | --- | --- | --- | --- | --- | --- | --- | --- | --- | --- | --- | --- | --- | --- | --- |
|  | **1** | **2*** | **3** | **4*** | **5** | **6** | **7*** | **8** | **9*** | | **10** | **11*** | | **12** | **13*** | **14** | **15*** | **16** |  |
|  |  |  |  |  |  |  |  |  | **a** | **b** |  | **a** | **b** |  |  |  |  |  |  |
| **Barnes, 2019 {3836}** | Yes | P^1^ | No | P^1^ | Yes | Yes | Yes | Yes | Yes | N/A^2^ | Yes | No | N/A^2^ | No | No | Yes | Yes | Yes | Critically low |
| **Cahill, 2010 {1652}** | Yes | P^1^ | No | P^1^ | No | Yes | Yes | Yes | P^1^ | N/A^2^ | Yes | No | N/A^2^ | Yes | No | Yes | Yes | Yes | Critically low |
| **Cahill, 2016 {1960}** | Yes | P^1^ | No | Yes | No | Yes | Yes | Yes | Yes | N/A^2^ | Yes | No | N/A^2^ | Yes | Yes | Yes | Yes | No | Low |
| **Farley 2012 {1469}** | No | P^1^ | No | No | Yes | Yes | Yes | Yes | P^1^ | N/A^2^ | No | No | N/A^2^ | No | No | Yes | No | No | Critically low |
| **Hartmann-Boyce, 2018 {332}** | Yes | Yes | No | P^1^ | No | Yes | Yes | Yes | P^1^ | No | Yes | No | No | Yes | Yes | Yes | Yes | No | Critically low |
| **Hollands, 2019 {3841}** | Yes | Yes | No | P^1^ | Yes | Yes | Yes | Yes | Yes | N/A^2^ | Yes | Yes | N/A^2^ | No | Yes | Yes | Yes | Yes | Moderate |
| **Howes 2020 {96}** | Yes | Yes | No | P^1^ | No | Yes | Yes | Yes | P^1^ | No | Yes | No | N/A^3^ | Yes | No | Yes | Yes | No | Critically low |
| **Khanna, 2016 {310}** | Yes | Yes | No | P^1^ | No | N/A^4^ | Yes | N/A^4^ | N/A^4^ | N/A^4^ | N/A^4^ | N/A^4^ | N/A^4^ | N/A^4^ | N/A^4^ | N/A^4^ | N/A^4^ | Yes | Moderate |
| **Lancaster 2017 {539}** | Yes | P^1^ | No | P^1^ | No | No | Yes | Yes | P^1^ | N/A^2^ | No | No | N/A^2^ | Yes | Yes | Yes | No | Yes | Critically low |
| **Lindson-Hawley, 2016 {671}** | Yes | Yes | No | P^1^ | Yes | Yes | Yes | Yes | P^1^ | NA | Yes | No | NA | No | Yes | Yes | No | No | Critically low |
| **Livingstone-Banks, 2019 {1077}** | Yes | Yes | No | P^1^ | No | No | Yes | Yes | P^1^ | N/A^2^ | Yes | Yes | N/A^2^ | Yes | Yes | Yes | Yes | Yes | Moderate |
| **Matkin, 2019 {1228}** | Yes | Yes | No | P^1^ | No | Yes | Yes | Yes | P^1^ | N/A^2^ | Yes | Yes | N/A^2^ | Yes | No | Yes | Yes | Yes | Low |
| **Posadzki 2016 {659}** | Yes | P^1^ | Yes | Yes | Yes | Yes | Yes | Yes | Yes | No | Yes | Yes | No | No | Yes | Yes | No | No | Critically low |
| **Stead, 2013 {1998}** | Yes | P^1^ | No | No | No | Yes | Yes | Yes | No | NA^2^ | No | No | NA^2^ | Yes | Yes | Yes | No | Yes | Critically low |
| **Stead 2016 {1356}** | Yes | Yes | No | P^1^ | No | Yes | Yes | Yes | No | N/A^2^ | No | Yes | N/A^2^ | Yes | Yes | Yes | Yes | Yes | Low |
| **Stead, 2017 {538}** | Yes | P^1^ | No | P^1^ | No | No | Yes | Yes | No | N/A^2^ | No | No | N/A^2^ | No | Yes | Yes | No | Yes | Critically low |
| **Taylor, 2017 {411}** | Yes | P^1^ | No | Yes | Yes | Yes | Yes | Yes | No | No | Yes | No | No | No | Yes | Yes | Yes | Yes | Critically low |
| **Tsoi, 2013 {1698}** | Yes | P^1^ | No | P^1^ | Yes | Yes | Yes | Yes | Yes | N/A^2^ | Yes | Yes | N/A^2^ | No | Yes | Yes | Yes | Yes | Moderate |
| **van der Meer 2013 {1223}** | Yes | P^1^ | No | P^1^ | Yes | Yes | Yes | Yes | P^1^ | N/A^2^ | No | No | N/A^2^ | No | No | Yes | No | Yes | Critically low |
| **Vodopivec-Jamsek, 2012 {1343}** | Yes | Yes | No | Yes | Yes | Yes | Yes | Yes | Yes | N/A^2^ | No | N/A^3^ | N/A^2^ | N/A^3^ | No | Yes | N/A^3^ | No | Low |
| **White 2014 {1618}** | Yes | P^1^ | No | P^1^ | No | No | Yes | Yes | P^1^ | N/A^2^ | No | Yes | N/A^2^ | No | Yes | Yes | Yes | No | Low |
| **Whittaker, 2019 {1803}** | Yes | No | No | Yes | Yes | Yes | Yes | Yes | Yes | NA | Yes | Yes | NA | Yes | Yes | Yes | Yes | Yes | Moderate |

*Critical domains

^1^ P = Partial Yes; ^2^ Not applicable – includes only RCTs; ^3^ Not applicable – no meta-analysis conducted; ^4^ Not applicable – no studies identified (empty review)
