# Supplementary material for: Effectiveness of smoking cessation interventions among adults: an overview of systematic reviews
Source: Syst Rev. 2024 Jul 12;13:179. doi: 10.1186/s13643-024-02570-9 (PMC11242003; doi:10.1186/s13643-024-02570-9)
Supplement: Supplementary file 17 — Additional file 17. Included analyses and results. [file 13643_2024_2570_MOESM17_ESM.docx]

**Additional file 17 – Included Analyses**

##### Figure 2a: Included analyses - pharmacotherapy interventions

|  |  |  |  |  | **GRADE rating** | | | | |  |
| --- | --- | --- | --- | --- | --- | --- | --- | --- | --- | --- |
| **Review, AMSTAR-2 Rating** | **Populations** | **Intervention** | **Comparator** | **Timepoint** | **Abstinence** | **Reduction** | **AE** | **Wt gain** | **∆ Emot State** | **Results; Number of studies; Number of participants** |
| **Cahill 2016** [60]**,** Low | MtQ | Cytisine | Placebo | 6+ months | MOD |  |  |  |  | RD 64 more per 1,000 (22 more to 147 more); n=2; N=937 |
|  | G/M | Cytisine | Placebo | 2 years | V. LOW |  |  |  |  | RD 79 more per 1,000 (31 more to 141 more); n=1; N=1214 |
|  | G/M | Cytisine | Placebo | NR |  |  | UTD |  |  | Adverse events similar between groups; n=3; N=NR |
|  | G/M | Varenicline | Placebo | 6+ months | MOD |  |  |  |  | RD 138 more per 1,000 (118 more to 159 more); n=27; N=12625 |
|  | G/M | Varenicline | Placebo | 6 months | MOD |  |  |  |  | RD 156 more per 1,000 (134 more to 179 more); n=25; N=12304 |
|  | G/M | Long-term Varenicline | Placebo | 6-12 months | V. LOW |  |  |  |  | RD 177 more per 1,000 (121 more to 249 more); n=4; N=2170 |
|  | G/M | Low-dose Varenicline | Placebo | 12 months | V. LOW |  |  |  |  | RD 111 more per 1,000 (57 more to 182 more); n=4; N=1266 |
|  | G/M | Varied dose Varenicline | Placebo | 12 months | LOW |  |  |  |  | RD 125 more per 1,000 (78 more to 183 more); n=6; N=1789 |
|  | G/M | Varenicline preloading | Placebo | NA |  |  |  |  |  | No studies found. |
|  | RtQ | Varenicline | Placebo | 12 months | MOD |  |  |  |  | RD 179 more per 1,000 (116 more to 266 more); n=1; N=1510 |
|  | Ml | Varenicline | Placebo | 6 months | HIGH |  |  |  |  | RD 104 more per 1,000 (67 more to 152 more); n=4; N=2332 |
|  | MI+MtQ | Varenicline | Placebo | 12 months | MOD |  |  |  |  | RD 101 more per 1,000 (29 more to 209 more); n=1; N=523 |
|  | FQA+MtQ | Varenicline | Placebo | 12 months | LOW |  |  |  |  | RD 168 more per 1,000 (65 more to 382 more); n=1; N=494 |
|  | G/M | Varenicline | Placebo | NA |  |  |  |  |  | No studies found^1^. |
|  | G/M | Varenicline | Placebo | Range |  |  | HIGH^2^ |  |  | RD 192 more per 1,000 (169 more to 216 more); n=32; N=14963 |
|  | G/M | Varenicline | Placebo | Range |  |  | HIGH^3^ |  |  | RD 41 more per 1,000 (29 more to 54 more); n=29; N=14447 |
|  | G/M | Varenicline | Placebo | Range |  |  | MOD^4^ |  |  | RD 64 more per 1,000 (50 more to 79 more); n=26; N=13682 |
|  | G/M | Varenicline | Placebo | Range |  |  | HIGH^5^ |  |  | RD 17 more per 1,000 (7 more to 30 more); n=25; N=13835 |
|  | G/M | Varenicline | Placebo | Range |  |  | HIGH^6^ |  |  | RD 1 fewer per 1,000 (6 fewer to 3 more); n=36; N=16189 |
|  | G/M | Varenicline | Placebo | Range |  |  | HIGH^7^ |  |  | RD 2 fewer per 1,000 (0 fewer to 4 fewer); n=24; N=11193 |
|  | G/M | Varenicline | Placebo | Range |  |  | HIGH^8^ |  |  | RD 7 more per 1,000 (1 more to 13 more); n=29; N=15370 |
|  | G/M | Varenicline | Placebo | Range |  |  | HIGH^9^ |  |  | RD 6 more per 1,000 (0 fewer to 12 more); n=26; N=15000 |
|  | G/M | Varenicline | Placebo | Range |  |  | HIGH^10^ |  |  | RD 2 fewer per 1,000 (5 fewer to 2 more); n=23; N=8955 |
|  | G/M | Varenicline | Placebo | Range |  |  | LOW^11^ |  |  | RD 3 more per 1,000 (1 fewer to 9 more); n=21; N=8587 |
|  | G/M | Varenicline | Placebo | Range |  |  | UTD^12^ |  |  | 3 of 4 studies 9.5-28% (Varen) vs 8-10% (Plac); n=4; N=NR |
| **Farley 2012** [58]**,** Critically low | MtQ | Bupropion | Placebo | End of treatment |  |  |  | MOD |  | MD 1.12 kg lower (1.47 lower to 0.77 lower); n=7; N=869 |
|  | MtQ | Bupropion | Placebo | 6 months |  |  |  | LOW |  | MD 0.87 kg lower (2.21 lower to 0.47 higher); n=4; N=218 |
|  | MtQ | Bupropion | Placebo | 12 months |  |  |  | LOW |  | MD 0.38 kg lower (2.00 lower to 1.24 higher); n=4; N=252 |
|  | MtQ | All types of NRT | Placebo | End of treatment |  |  |  | V. LOW |  | MD 0.69 kg lower (0.88 lower to 0.51 lower); n=19; N=2600 |
|  | MtQ | All types of NRT | Placebo | 6 months |  |  |  | MOD |  | MD 0.37 kg lower (0.88 lower to 0.14 higher); n=9; N=771 |
|  | MtQ | All types of NRT | Placebo | 12 months |  |  |  | MOD |  | MD 0.42 kg lower (0.92 lower to 0.08 higher); n=15; N=1334 |
|  | MtQ | Varenicline 2 mg/day | Placebo | End of treatment |  |  |  | MOD |  | MD 0.41 kg lower (0.63 lower to 0.19 lower); n=11; N=2008 |
|  | MtQ | Varenicline 2 mg/day | Placebo | 6 months |  |  |  | LOW |  | MD 0.41 kg higher (0.79 lower to 1.61 higher); n=1; N=105 |
|  | MtQ | Varenicline 2 mg/day | Placebo | 12 months |  |  |  | MOD |  | MD 1.11 kg higher (0.75 lower to 2.98 higher); n=2; N=151 |
|  | MtQ | Varenicline 1 mg/day | Placebo | End of treatment |  |  |  | LOW |  | MD 0.12 kg lower (0.68 lower to 0.43 higher); n=3; N=254 |
| **Hartmann-Boyce 2018** [52]**,** Critically low | RMtQ | NRT patch | Placebo patch | 6 months | V. LOW |  |  |  |  | RD data NA; RR 1.25 (0.34 to 4.60); n=1; N=629 |
|  | MtQ | NRT | Placebo | Unclear/NR |  |  | UTD |  |  | Various common events across formulations; n=6; N=NR |
|  | MtQ | NRT | Placebo | Unclear/NR |  |  | MOD^25^ |  |  | RD 12 more per 1,000 (5 to 21 more); n=15; N=11,074 |
|  | MtQ | NRT | Placebo | Unclear/NR |  |  | UTD^26^ |  |  | NRT generally similar to or lower than placebo; n=NR; N=NR |
| **Howes 2020** [65]**,** Critically low | NMtQ | Bupropion | Placebo | 6 months | V. LOW |  |  |  |  | RD 14 more per 1000 (18 fewer to 75 more); n=1; N=594 |
|  | NMtQ | Bupropion | Placebo | Unclear/NR |  | UTD^13^ |  |  |  | No difference; n=1; N=594 |
|  | NMtQ | Bupropion | Placebo | 12 months |  | V. LOW^14^ |  |  |  | RD 26 fewer per 1000 (40 fewer to 27 more); n=1; N=327 |
|  | G/M | Bupropion | Placebo | Unclear/NR |  |  |  |  | UTD^15^ | Reduction in both arms sustained at f/u. In HDS, greater reduction in bupropion arm but not sustained; n=1; N=NR |
| **Lindson-Hawley 2016** [54]**,** Critically low | NMtQ | Bupropion | Placebo | 6 months | V. LOW |  |  |  |  | RD 14 more per 1,000 (18 fewer to 75 more); n=1; N=594 |
|  | NMtQ | Bupropion | Placebo | 12 months |  | V. LOW^16^ |  |  |  | RD 1 more per 1,000 (36 fewer to 63 more); n=1; N=594 |
|  | NMtQ | Bupropion | Placebo | 12 months |  | V. LOW^14^ |  |  |  | RD 26 fewer per 1,000 (40 fewer to 27 more); n=1; N=327 |
|  | NMtQ | Bupropion | Placebo | 12 months |  | UTD^17^ |  |  |  | No difference; n=1; N=Unclear/NR |
|  | NMtQ | Bupropion | Placebo | Unclear/NR |  |  | V. LOW |  |  | RD 17 more per 1,000 (3 fewer to 91 more); n=1; N=594 |
|  | NMtQ | Varenicline | Placebo | 6 months | V. LOW |  |  |  |  | RD 68 more per 1,000 (10 fewer to 245 more); n=1; N=218 |
|  | NMtQ | Varenicline | Placebo | Unclear/NR |  |  | UTD |  |  | No difference; n=1; N=Unclear/NR |
|  | NMtQ | NRT | Placebo | 12-24 months | MOD |  |  |  |  | RD 44 more per 1,000 (22 more to 73 more); n=8; N=3081 |
|  | NMtQ | NRT | Placebo | 12+ months |  | MOD |  |  |  | RD 60 more per 1,000 (35 more to 91 more); n=8; N=3081 |
| **Tsoi 2013** [57]**,** Moderate | Ml | Bupropion | Placebo | 6 months | V. LOW |  |  |  |  | RD 66 more per 1,000 (1 more to 244 more); n=5; N=214; Cessation trials |
|  | Ml | Bupropion | Placebo | 6 months |  | LOW^13^ |  |  |  | MD 0.4 higher (5.72 lower to 6.53 higher); n=2; N=104; Cessation trials |
|  | Ml | Bupropion | Placebo | 6 months |  | V. LOW^18^ |  |  |  | MD 5.55 ppm lower (17.89 lower to 6.78 higher); n=3; N=123; Cessation trials |
|  | Ml | Bupropion | Placebo | End of treatment |  |  |  |  | LOW^19^ | SMD 0.24 SD lower (0.66 lower to 0.19 higher); n=2; N=85; Cessation trials |
|  | Ml | Bupropion | Placebo | End of treatment. |  |  |  |  | V. LOW^20^ | SMD 0.12 SD lower (0.46 lower to 0.22 higher); n=3; N=136; Cessation trials |
|  | Ml | Bupropion | Placebo | End of treatment |  |  |  |  | LOW^15^ | SMD 0.16 lower SD (0.50 lower to 0.18 higher); n=3; N=136; Cessation trials |
|  | Ml | Bupropion | Placebo | Unclear/NR |  |  | UTD |  |  | Mix of results (see GRADE); n=7; N=NR; Cessation trials |
|  | Ml | Bupropion | Placebo | End of treatment |  |  |  |  | V. LOW^21^ | No difference or no worsening; n=3; N=NR; Reduction trials |
|  | Ml | Bupropion | Placebo | Unclear/NR |  |  | V. LOW |  |  | No events or no difference; n=3; N=NR; Reduction trials |
|  | Ml | Varenicline | Placebo | 6 months | V. LOW |  |  |  |  | RD 94 more per 1,000 (8 fewer to 866 more); n=1; N=128; Cessation trials |
|  | Ml | Varenicline | Placebo | 6 months |  | UTD^13^ |  |  |  | No difference; n=1; N=NR; Cessation trials |
|  | Ml | Varenicline | Placebo | End of treatment |  |  |  |  | UTD^22^ | No difference; n=2; N=NR; Cessation trials |
|  | Ml | Varenicline | Placebo | Unclear/NR |  |  | UTD |  |  | See GRADE; n=2; N=NR; Cessation trials |
|  | Ml | Varenicline | Placebo | Unclear/NR |  |  | V. LOW |  |  | Mix of results (see GRADE); n=3; N=NR: Varenicline for other uses |
|  | Ml | Varenicline | Placebo | Unclear/NR |  |  |  |  | UTD^23^ | No change or no difference; n=3; N=NR; Varenicline for other uses |
|  | Ml | NRT | Placebo | Unclear/NR |  |  |  |  | UTD^24^ | No change or no difference; n=2; N=NR; Reduction trials |
|  | Ml | NRT | Placebo | Unclear/NR |  |  | V. LOW |  |  | See GRADE; n=1; N=NR; Reduction trials |
|  | Ml | NRT | Placebo | 6 months |  |  |  |  |  | No studies reporting on abstinence found. |
|  | Ml | NRT | Placebo | 6 months |  |  |  |  |  | No studies reporting on reduction (expired CO) found. |
| **van der Meer 2013** [56]**,** Critically low | Ml | Bupropion | Placebo | 6-12 months | V. LOW |  |  |  |  | Current depression: RD 41 more per 1000 (19 fewer to 142 more); n=5; N=410 |
|  | Ml | Bupropion | Placebo | 6-12 months | LOW |  |  |  |  | Past depression: RD 128 more per 1000 (38 more to 268 more); n=4; N=404 |
|  | Ml | NRT gum | Placebo | 12 months | V. LOW |  |  |  |  | Current depression: RD 94 more per 1000 (4 fewer to 369 more); n=1; N=196 |
|  | MI | NRT | Placebo | 6+ months | LOW |  |  |  |  | Past depression: RD 42 more per 1000 (38 fewer to 150 more); n=3; N=432 |

Abbreviations AE=Adverse events; Antidepress=Antidepressants; Cess=Cessation; CO=Carbon monoxide; Emot=Emotional; f/u=Follow-up; FQA=failed quit attempt; G/M=General/mixed population of smokers; HDS=Highly dependant smokers; MD=Mean difference; MI=Mental illness; MOD: Moderate MtQ=Motivated to quit; NMtQ=Not motivated to quit; NR=Not reported; NRT=Nicotine replacement therapy; Pharm=Pharmacotherapy; PQ=previous quitters; RMtQ: Relapsed and motivated to quit; Reducing to quit=RtQ; SMD: Standardized mean difference; UTD: Unable to determine Wt=Weight; V. LOW=Very low

1. Harm reduction
2. Nausea
3. Insomnia
4. Abnormal dreams
5. Headache
6. Depression
7. Suicidal ideation
8. At least one serious adverse event
9. At least one serious adverse event during or immediately after treatment
10. Neuropsychiatric events (not deaths)
11. Cardiac serious adverse events, including deaths
12. Treatment discontinuation
13. Reduction in number of cigarettes per day from baseline
14. Reduction in cotinine >50%
15. Change in emotional/mental state: Depressive symptoms
16. Reduction in cigarettes/day of >50% of baseline or cessation
17. Reduction in cotinine (mean reduction from baseline)
18. Reduction in expired CO
19. Change in mental state: Positive symptoms
20. Change in mental state: Negative symptoms
21. Change in mental state: Positive, negative, and psychiatric symptoms
22. Change in mental state: Positive, negative, and depressive symptoms
23. Change in mental state: Positive, negative, depressive, and general symptoms of schizophrenia
24. Change in mental state: Psychiatric symptoms, subjective experience, or mental status
25. Palpitations, chest pain
26. Attrition

##### Figure 2b: Included analyses - behavioural interventions

|  |  |  |  |  | **GRADE rating** | | | | |  |
| --- | --- | --- | --- | --- | --- | --- | --- | --- | --- | --- |
| **Review AMSTAR-2 Rating** | **Populations** | **Intervention** | **Comparator** | **Timepoint** | **Abstinence** | **Reduction** | **AE** | **Wt gain** | **∆ Emot State** | **Results; Number of studies; Number of participants** |
|  |  |  |  |  |  |  |  |  |  |  |
| **Cahill 2010** [66], Critically low | G/M | SB expert systems or tailored  S-H mat. | Assessment only | 6+ months | V. LOW |  |  |  |  | RD 22 more per 1,000 (12 more to 33 more); n=10; N=13597 |
|  | G/M | SB expert systems pCon or Con | No Intervention | 14 months | V. LOW |  |  |  |  | Favours intervention; n=1; N=NR |
|  | G/M | SB interactive computer programme | Usual care | 12+ months | V. LOW |  |  |  |  | RD 10 more per 1,000 (13 fewer to 41 more); n=2; N=1702 |
|  | G/M | SB telephone counselling | Usual care | 12 months | V. LOW |  |  |  |  | RD 16 more per 1,000 (27 fewer to 114 more); n=1; N=318 |
|  | G/M | SB individual counselling and/or advice | Usual care | 6+ months | MOD |  |  |  |  | RD 21 more per 1,000 (1 fewer to 46 more); n=7; N=3293 |
|  | G/M | SB individual counselling or advice | Assessment only | 6+ months | V. LOW |  |  |  |  | RD 12 more per 1,000 (2 fewer to 31 more); n=3; N=3056 |
| **Hollands 2019** [64]. Low | MtQ | Interv. to increase adher. to meds | Usual/Standard care | 6 months | V. LOW |  |  |  |  | RD 33 more per 1,000 (8 fewer to 81 more); n=5; N=3593 |
|  | MtQ | Interv. to increase adher. to meds | Usual/Standard care | Unclear/NR |  |  | UTD |  |  | No events or no difference between groups; n=3; N=NR |
|  | MtQ | Interv. to increase adher. to meds | Usual/Standard care | 1-week and 6 months |  |  |  |  | UTD | No difference between groups; n=1; N=NR |
| **Lancaster 2017** [67]**,** Critically low | G/M | Individual counselling | Minimal contact | 6+ months | V. LOW |  |  |  |  | RD 40 more per 1,000 (28 more to 54 more); n=27; N=11,100 |
| **Livingstone-Banks 2019** [68], Moderate | G/M | Non-tailored S-H  (no F2F contact) | No materials/No intervention | 6+ months | HIGH |  |  |  |  | RD 10 more per 1,000 (2 more to 19 more); n=11; N=13,241 |
|  | MtQ | Non-tailored S-H  (no F2F contact) | No materials/No intervention | 6 months | V. LOW |  |  |  |  | RD 174 more per 1,000 (71 more to 398 more); n=2; N=924 |
|  | G/M | Non-tailored S-H  (no F2F contact) | Brief leaflet | 6+ months | MOD |  |  |  |  | RD 10 fewer per 1,000 (23 fewer to 5 more); n=6; N=7023 |
|  | G/M | Non-tailored S-H  (with F2F contact) | No intervention or leaflet only | 6+ months | LOW |  |  |  |  | RD 18 more per 1,000 (1 more to 41 more); n=4; N=2822 |
|  | G/M | Individually tailored S-H  (no F2F contact) | No materials/No intervention | 6+ months | LOW |  |  |  |  | RD 20 more per 1,000 (11 more to 31 more); n=10; N=14,359 |
| **Matkin 2019** [69], Low | G/M | Intensive telephone counselling | Minimal telephone counselling | 6+ months | V. LOW |  |  |  |  | RD 64 more per 1,000 (28 more to 104 more); n=3; N=2,602 |
|  | G/M | Brief motivational telephone counselling | Usual care telephone call | 12 months | V. LOW |  |  |  |  | RD 60 more per 1,000 (4 more to 190 more); n=1; N=374 |
|  | G/M | Telephone counselling for reduction | Usual care telephone call | 12 months | V. LOW |  |  |  |  | RD 49 per 1,000 (1 fewer to 167 more); n=1; N=375 |
| **Posadzki 2016** [70], Critically low | G/M | ATCS, Interactive voice response system | No intervention | 24 months | UTD |  |  |  |  | Little to no difference between groups (Interv: 21.7%, Ctrl.: 42.9%; P = 0.13); n=1; N=NR |
| **Stead 2013** [71], Critically low | G/M | Physician advice (minimal or intensive) | No advice (or usual care) | 6+ months | LOW |  |  |  |  | RD 36 more per 1,000 (28 more to 46 more); n=26; N=22,239 |
|  | G/M | Physician advice + follow-up | Minimal advice with 1 visit | 6+ months | V. LOW |  |  |  |  | RD 47 more per 1,000 (7 more to 103 more); n=5; N=1254 |
|  | G/M | Intensive advice | Minimal advice | 6+ months | V. LOW |  |  |  |  | RD 28 more per 1,000 (15 more to 42 more); n=15; N=9775 |
| **Stead 2017** [73], Critically low | G/M | Group therapy | No intervention (or minimal contact) | 6+ months | V. LOW |  |  |  |  | RD 108 more per 1,000 (54 more to 186 more); n=9; N=1098 |
| **Taylor 2017** [74], Critically low | G/M | Internet (Interactive + Tailored) | Non-active Control | 6-12 months | V. LOW |  |  |  |  | RD 19 more per 1,000 (1 more to 39 more); n=8; N=6786 |
| **Vodopivec-Jamsek 2012** [63] Low | MtQ | Mobile phone SMS | Control | 6 months | V. LOW |  |  |  |  | RD 17 more per 1,000 (21 fewer to 62 more); n=1; N=1705^1^ |
|  | MtQ | Mobile phone SMS | Control | 6 months | LOW |  |  |  |  | RD 76 more per 1,000 (30 more to 131 more); n=1; N=1705^2^ |
|  | MtQ | Mobile phone SMS | Control | 6 months | LOW |  |  |  |  | RD 29 more per 1,000 (5 more to 65 more); n=1; N=1705^3^ |
|  | MtQ | Mobile phone SMS | Control | 6 months | LOW |  |  |  |  | RD 15 more per 1,000 (2 fewer to 44 more); n=1; N=1705^4^ |
|  | MtQ | Mobile phone SMS | Control | 6 months |  |  | LOW^5^ |  |  | RD 6 fewer per 1,000 (21 fewer to 18 more); n=1; N=1705 |
|  | MtQ | Mobile phone SMS | Control | 6 months |  |  | LOW^6^ |  |  | RD 5 more per 1,000 (15 fewer to 33 more); n=1; N=1705 |
| **Whittaker 2019** [75], Moderate | MtQ | Mobile phone-based intervention | Usual care | 6 months | LOW |  |  |  |  | RD 37 more per 1,000 (26 more to 50 more); n=12; N=11,885 |

Abbreviations: ACTS= automated telephone communication systems; Adher=Adherence; Adv=Advice; AE=Adverse events; Auto=Automated; Con=Contemplation; Cont=Contact; Emot=Emotional; F2F= Face to face; G/M=General/mixed population of smokers; Grp=Group; Interv=Intervention; IVR=Interactive voice response; Meds=Medications; MOD=Moderate; NMtQ=Not motivated to quit; NR=Not reported; PCon=Precontemplation; SB=Stage-based; S-H=Self-help; SMS=Short messaging service; UTD=Unable to determine; Wt=Weight; V. LOW=Very low

1. Point prevalence analysis undertaken
2. Point prevalence analysis with last outcome carried forward
3. Continuous analysis undertaken, allowing for defined lapses in smoking
4. Continuous analysis undertaken, requiring complete abstinence
5. Car crash
6. Pain in thumb/finger joints from texting

##### Figure 2c: Included analyses - other therapy interventions

|  |  |  |  |  | | **GRADE rating** | | | |  |
| --- | --- | --- | --- | --- | --- | --- | --- | --- | --- | --- |
| **Review, AMSTAR-2 Rating** | **Populations** | **Intervention** | **Comparator** | **Timepoint** | **Abstinence** | **Reduction** | **AE** | **Wt gain** | **∆ Emot State** | **Results; Number of studies; Number of participants** |
| **Barnes 2019** [51], Critically low | MtQ | Hypnotherapy | Placebo drug | 12 months | V. LOW |  |  |  |  | RD 18 fewer per 1,000 (77 fewer to 166 more); n=1; N=114 |
| **White 2014** [53], Low | MtQ | Acupuncture | Wait list/No intervention | 6-12 months | V. LOW |  |  |  |  | RD 60 more per 1,000 (2 fewer to 174 more); n=3; N=393 |
|  | MtQ | Acupuncture | Sham | 6-12 months | LOW |  |  |  |  | RD 11 more per 1,000 (15 fewer to 43 more); n=11; N=1892 |
|  | MtQ | CAS | Sham | 6-12 months | V. LOW |  |  |  |  | RD 26 more per 1,000 (12 fewer to 98 more); n=6; N=570 |
|  | MtQ | Laser therapy | Sham | 6-12 months | V. LOW |  |  |  |  | Null-inclusive findings in one study and results favouring intervention in second study; n=2; N=613 |
|  | MtQ | Electrostimulation | Sham | 6-12 months | V. LOW |  |  |  |  | RD 34 fewer per 1,000 (102 fewer to 60 more); n=2; N=405 |
|  | MtQ | Acupressure | Sham | 6-12 months |  | | | | | No studies found. |
|  | MtQ | Laser therapy | Wait list/No intervention | 6-12 months |  | | | | | No studies found. |
|  | MtQ | Electrostimulation | Wait list/No intervention | 6-12 months |  | | | | | No studies found. |
| **Howes 2020** [65]**,** Critically low | MtQ | SJW | Placebo | 6 months | LOW |  |  |  |  | RD 10 fewer per 1000 (40 fewer to 83 more); n=2; N=261 |
|  | MtQ | SAMe | Placebo | 6 months | V. LOW |  |  |  |  | RD 38 fewer per 1000 (95 fewer to 134 more); n=1; N=120 |

Abbreviations: AE=Adverse events; CAS=Continuous auricular stimulation; Emot=Emotional; MOD=Moderate; MtQ=Motivated to quit; NMtQ=Not motivated to quit; NR=Not reported; SJW=St. John’s Wort; SAMe=S-Adenosyl-Methionine; UTD= Unable to determine; Wt=Weight; V. LOW=Very low

##### Figure 2d: Included analyses - combination interventions

|  |  |  |  |  | **GRADE rating** | | | | |  |
| --- | --- | --- | --- | --- | --- | --- | --- | --- | --- | --- |
| **Review, AMSTAR-2 Rating** | **Populations** | **Intervention** | **Comparator** | **Timepoint** | **Abstinence** | **Reduction** | **AE** | **Wt gain** | **∆ Emot State** | **Results; Number of studies; Number of participants** |
| **Lindson-Hawley 2016** [54], Critically low | NMtQ | Behavioural support (advice) + NRT + phone calls | No intervention | 6 months | V. LOW |  |  |  |  | Favours interv.; n=1; N=NR |
|  | NMtQ | Behavioural support (advice) + NRT + phone calls | No intervention | 6 months |  | V. LOW |  |  |  | Favours interv.; n=1; N=NR |
|  | NMtQ | Individual telephone counselling plus S-H mat. | Usual care | 12 months | LOW |  |  |  |  | RD 22 more per 1,000 (18 fewer to 124 more); n=1; N=320 |
|  | NMtQ | Individual telephone counselling plus S-H mat. | Usual care | 12 months |  | V. LOW^1^ |  |  |  | RD 63 more per 1,000 (22 fewer to 195 more); n=1; N=320 |
|  | NMtQ | Individual telephone counselling plus S-H mat. | Usual care | 12 months |  | LOW^2^ |  |  |  | RD 1 fewer per 1,000 (59 fewer to 100 more); n=1; N=320 |
|  | NMtQ | Individual telephone counselling plus S-H mat. | Usual care | 12 months |  | UTD^3^ |  |  |  | No difference; n=1; N=320 |
|  | NMtQ | Individual telephone counselling plus S-H mat. | Usual care | 12 months |  | UTD^4^ |  |  |  | No difference; n=1; N=320 |
| **Matkin 2019** [69], Low | G/M | Telephone hotline & S-H | Minimal intervention | 12-18 months | LOW |  |  |  |  | RD 21 more per 1,000 (5 more to 42 more); n=2; N=3,327 |
| **Stead 2016** [72], Low | G/M | Combined behavioural + pharmacotherapy | Usual care or minimal intervention | 6+ months | LOW |  |  |  |  | RD 71 more per 1,000 (58 more to 84 more); n=52; N=19488 |
|  | G/M | Combined behavioural + pharmacotherapy | Usual care or no intervention | 12 months | MOD |  |  |  |  | RD 260 more per 1,000 (212 more to 315 more); n=1; N=5887 |
| **Taylor 2017** [74], Critically low | G/M | Internet + behavioural support | Non-internet, non-active control. | 6-12 months | V. LOW |  |  |  |  | RD 54 more per 1,000 (23 more to 92 more); n=5; N=2334 |
| **Tsoi 2013** [57]**,** Moderate | MI | Individual smoking cessation intervention  (CBT and MI + NRT | Routine Care | 6 months, 12 months & 4 years | UTD |  |  |  |  | No difference; n=1; N=NR; Cessation trials |
|  | MI | Individual smoking cessation intervention  (CBT and MInt) + NRT | Routine Care | 6 months, 12 months & 4 years |  | UTD |  |  |  | No difference; n=1; N=NR; Cessation trials |
| **van der Meer 2013** [56]**,** Critically low | MI | Standard treatment + extended NRT + extended CBT | Standard treatment | Unclear/NR | V. LOW |  |  |  |  | Past depression: RD 259 more per 1000 (6 fewer to 786 more) |

Abbreviations: Adv=Advice; AE=Adverse events; CBT=Cognitive behavioural therapy; Emot=Emotional; G/M=General/mixed population of smokers; MI=Mental illness; MInt=Motivational Interviewing; MOD=Moderate; MtQ=Motivated to quit; NMtQ=Not motivated to quit; NR=Not reported; NRT=Nicotine replacement therapy; UTD=Unable to determine; Wt=Weight; V. LOW=Very low

1. Reduction in cigarettes/day of >50% of baseline or cessation
2. Reduction in CO >50%
3. Reduction in number of cigarettes/day from baseline (mean reduction from baseline)

Reduction in CO from baseline (Mean reduction from baseline)

##### Figure 2e: Included analyses - electronic cigarettes

|  |  |  |  |  | **GRADE rating** | | | | | |  |
| --- | --- | --- | --- | --- | --- | --- | --- | --- | --- | --- | --- |
| **Review, AMSTAR-2 Rating** | **Populations** | **Intervention** | **Comparator** | **Timepoint** | **Abstinence** | **Reduction** | **QoL** | **AE** | **Wt gain** | **∆ Emot State** | **Results; Number of studies; Number of participants** |
| **Lindson-Hawley 2016** [54],  Critically low | NMtQ | E-cig. | Placebo e-cigs | 12 months | LOW |  |  |  |  |  | RD 70 more per 1,000 (1 fewer to 270 more); n=1; N=300 |
|  | NMtQ | E-cig. | Placebo e-cigs | 12 months |  | V. LOW^1^ |  |  |  |  | RD 45 more per 1,000 (38 fewer to 187 more); n=1; N=300 |
|  | NMtQ | E-cig. | Placebo e-cigs | 12 months |  | UTD^2^ |  |  |  |  | No difference; n=1; N=300 |
|  | NMtQ | E-cig. | Placebo e-cigs | 12 months |  | UTD^3^ |  |  |  |  | No difference; n=1; N=300 |
|  | NMtQ | E-cig. | Placebo e-cigs | Unclear/NR |  |  |  | UTD^4^ |  |  | No difference; n=1; N=NR |
|  | NMtQ | E-cig. | Placebo e-cigs | Unclear/NR |  |  |  | UTD^5^ |  |  | No events in either arm; n=1; N=NR |
|  | NMtQ | E-cig. | Placebo e-cigs | Unclear/NR |  |  |  |  | UTD |  | No difference; n=1; N=NR |
| **Hersi 2020** (unpublished),  N/A | G/M | E-cig with nicotine | No intervention | 12 to 16 weeks |  |  |  | V. LOW^4,5^ |  |  | See GRADE table; n=2; N=1808 |
|  | G/M | E-cig with nicotine | No intervention | 12 to 16 weeks |  |  |  |  | V. LOW |  | MD 0.2 kg higher (3.53 lower to 3.93 higher); n=1; N=408 |
|  | G/M | E-cig with nicotine | Waitlist | 1 to 8 weeks |  |  |  | V. LOW ^6^ |  |  | No difference; n=1; N=48 |
|  | G/M | E-cig with nicotine + usual care | Usual care | 6 months | V. LOW |  |  |  |  |  | RD 100 more per 1,000 (from 18 fewer to 649 more); n=1; N=80 |
|  | G/M | E-cig with nicotine + usual care | Usual care | 6 months |  | V. LOW ^3,7,8^ |  |  |  |  | CO: MD 6.2 ppm lower (12.21 lower to 0.19 lower); n=1; N=80  SC: MD 25.1 ng/mL lower (93.48 lower to 43.28 higher); n=1; N=80  SA: MD 0.6 ng/mL lower (1.54 lower to 0.34 higher); n=1; N=80 |
|  | G/M | E-cig with nicotine + usual care | Usual care | 6 months |  |  | V. LOW |  |  |  | MD 1.4 points higher (5.9 lower to 8.7 higher); n=1; N=80 |
|  | G/M | E-cig with nicotine + usual care | Usual care | 6 months |  |  |  | V. LOW |  |  | See GRADE table; n=1; N=80 |
|  | G/M | E-cig with nicotine + usual care | Usual care | 6 months |  |  |  |  |  | V. LOW | MD 0 points (4.4 lower to 4.4 higher); n=1; N=80 |
|  | MtQ | E-cig with nicotine + standard care | E-cigarette with no nicotine + standard care | 24 weeks | V. LOW |  |  |  |  |  | RD 100 more per 1,000 (from 59 fewer to 871 more); n=1; N=40 |
|  | MtQ | E-cig with nicotine + standard care | E-cigarette with no nicotine + standard care | 24 weeks |  | V. LOW ^2^ |  |  |  |  | MD 2.54 higher (4.62 lower to 9.7 higher); n=1; N=40 |
|  | MtQ | E-cig with nicotine + standard care | E-cigarette with no nicotine + standard care | 24 weeks |  |  |  | V. LOW |  |  | See GRADE tables; n=1; N=40 |
|  | NMtQ | E-cigarettes with nicotine | E-cigarettes with no nicotine | 24 weeks | V. LOW |  |  |  |  |  | 60 more per 1,000 (from 7 fewer to 232 more); n=1; N=300 |
|  | NMtQ | E-cigarettes with nicotine | E-cigarettes with no nicotine | 52 weeks | V. LOW |  |  |  |  |  | 70 more per 1,000 (from 1 fewer to 270 more); n=1; N=300 |
|  | NMtQ | E-cigarettes with nicotine | E-cigarettes with no nicotine | 24 weeks |  | V. LOW ^1^ |  |  |  |  | 30 more per 1,000 (from 47 fewer to 162 more); n=1; N=300 |
|  | NMtQ | E-cigarettes with nicotine | E-cigarettes with no nicotine | 52 weeks |  | V. LOW ^1^ |  |  |  |  | 25 fewer per 1,000 (from 72 fewer to 68 more); n=1; N=300 |
|  | NMtQ | E-cigarettes with nicotine | E-cigarettes with no nicotine | 52 weeks |  | UTD^3^ |  |  |  |  | See GRADE table; n=1; N=183 |
|  | NMtQ | E-cigarettes with nicotine | E-cigarettes with no nicotine | 52 weeks |  |  |  | UTD |  |  | See GRADE table; n=1; N=NR |
|  | NMtQ | E-cigarettes with nicotine | E-cigarettes with no nicotine | 52 weeks |  |  |  |  | V. LOW |  | See GRADE table; n=1; N=NR |

Abbreviations: AE=Adverse event; E-cig=Electronic cigarette; Emot=Emotional; G/M= General/mixed population of smokers; MOD=Moderate; MtQ=Motivated to quit; NMtQ=Not motivated to quit; NR=Not reported; UTD=Unable to determine;

Wt=Weight; V. LOW=Very low

1. Reduction in cigarettes/day of >50% of baseline or cessation
2. Reduction in number cigarettes/day
3. Reduction in CO
4. Adverse events (general)
5. Serious adverse events
6. Complaints
7. Salivary cotinine (SC)
8. Salivary anabasine (SA)
