# Supplementary material for: Effectiveness of smoking cessation interventions among adults: an overview of systematic reviews
Source: Syst Rev. 2024 Jul 12;13:179. doi: 10.1186/s13643-024-02570-9 (PMC11242003; doi:10.1186/s13643-024-02570-9)
Supplement: Supplementary file 18 — Additional file 18. Stakeholders’ Feedback. [file 13643_2024_2570_MOESM18_ESM.docx]

## **Additional file 18.** **Stakeholder feedback**

**Document sent to stakeholders**

Thank you again for reviewing the evidence review manuscript: ***Effectiveness of smoking cessation interventions among adults: an overview of systematic reviews***. This overview of reviews focusses on the benefits and harms of interventions required to promote cessation of tobacco smoking in adults aged 18 years and older. These results will then be used to help inform guidelines for the Canadian Task Force on Preventive Health Care’s (CTFPHC).

**INSTRUCTIONS:**

- Please use this form to provide feedback on the evidence review and return this form no later than **January 15, 2024,** to **taskforce.admin@CTFPHC.onmicrosoft.com**
- If you have any questions related to the review process, contact Greg Traversy at [**gregory.traversy@phac-aspc.gc.ca**](mailto:gregory.traversy@phac-aspc.gc.ca)
- Please check the appropriate box to answer the questions and elaborate in the space provided if necessary.

| **Question 1** | **Yes** | **No** |
| --- | --- | --- |
| Are the objectives and methods of this evidence review clear? | **​​☐​** | **​​☐​** |
|  | **Comments:** | |
| **Question 2** | **Yes** | **No** |
| Were the results clearly stated? | **​​☐​** | **​​☐​** |
|  | **Comments:** | |
| **Question 3** | **Yes** | **No** |
| Are the conclusions in the review supported by the data that were reviewed? | **​​☐​** | **​​☐​** |
|  | **Comments:** | |
| **Question 4** |  | |
| Do you have any additional comments? |  | |

| **Question** | **Comment** | **Response** | **Reviewer** |
| --- | --- | --- | --- |
| **Question 1**  Are the objectives and methods of these evidence reviews clear? | Strengths: use of PRIOR checklist & PROSPERO registration of protocol. Weakness: not assessing primary studies for risk of bias, overlap, accuracy, etc. (addressed in conclusion). | Thank you for your comment. | Reviewer#1 |
| **Question 2**  Were the results clearly stated? | Exceptionally detailed. A strength is the consistent order in which the results are presented for each intervention group. | Thank you for your comment. | Reviewer#1 |
| **Question 3**  Are the conclusions in the review supported by the data that were reviewed? | They clearly address the limitations of both the review and the quality of the data that exists. Hopefully the guidelines will explicitly state the weakness of the data underlying the recommendations. | Thank you for your comment. | Reviewer#1 |
| **Question 4**  Do you have any additional comments? | Why was CINAHL not included in the databases included? Happy to see the RNAO guidelines included but there is potentially nursing literature that was relevant and missed | Thank you for your comment. We looked at strategies produced by two other groups (USPSTF and Hartmann Boyce), and neither of these used CINAHL in their searches. | Reviewer#1 |
| **Question 1**  Are the objectives and methods of these evidence reviews clear? | **Comments:**  **Abstract/** The objective of the study is clear. However, I think it would be preferable to identity the first section of the abstract by the term “objective” or to provide information on why it is important to address and provide a Canadian guidance on smoking cessation in the “background” section.  In the “methods” section, the search strategy and quality assessment of the retrieved articles is well-described, but the general method employed is not mentioned (see comment below on the methods section of the manuscript).  **Introduction /**  Definition: I would have added a definition of what smoking is/what defines a smoker (i.e., nicotine dependance)  Argument: Even if we can assume/deduce that “There is a need to develop guidance to address smoking cessation strategies relevant to the Canadian context”, I think the explanation can be formulated more clearly: e.g., Canadian guidelines dating back to more than 5 to 10 years, new smoking cessation methods (approved or not), variation in the international recommendations …  Please see comments directly on the draft. I made some minor suggestions.  **Methods /** It would be relevant to standardize the use of the terms employed to describe the methods, in order to avoid confusion. For example, the title announces an overview of systematic reviews, but in the objective section of the abstract, the term “review” is used, in the objective section of the manuscript itself, it is “overview of reviews”, and in the methods section, it says “evidence review”. (See specific comments in the text).  It would be relevant to explain the following: why you restricted the search from 2008 up to 2020? And why you are not updating the results for 2020 up to now? When the results of this extended and rigorous work will be published, there will be a 4-year gap between the latest evidence and the recommendations made from the results of this work. I think this is important, especially for studies related to e-cigarette/vaping, to ensure up-to-date clinical practice guidelines.  Given that the methods section is 8 pages long (excluding the many appendices), it would be easier for the reader to have a schematic view of the research process in which the relevant appendix is identified for quick reference.  Please see specific comments on the eligibility criteria in appendix F. | Thank you for your comments. We have remained consistent with the abstract criteria mentioned in the Journal of Systematic reviews.  Thanks for your comments, we have considered your notes in the introduction and the methods section.  Please find the details on the search strategy in D. We have done a search update for the part -II on E-cigs but not for the overview of reviews since evidence on other interventions is not moving as fast.  We remained consistent with the methodology mentioned in part II, e-cigarettes.  We have made those changes as advised in Appendix F. | Reviewer#2 |
| **Question 2**  Were the results clearly stated? | I suggest presenting the results in a more synthetic manner in the text as this section is over than 50 pages long (versus a discussion of 2 pages). Would it be feasible to have a table summarizing benefits/harms for each type of intervention (pharmacotherapy, behavioral, other therapy, e-cigarette, combination intervention), along with the main conclusion/the certainty of evidence for each? More details can be/are already available in the appendix.  Appendix Q is very informative. | Thank you for your notes. We have aligned the results presented here with the results of the second part of the evidence review (i.e., e-cigarettes) for consistency.  We also understand that a summary table of interventions will be included in the guideline that this review is informing. | Reviewer#2 |
| **Question 3**  Are the conclusions in the review supported by the data that were reviewed? | **Comments:**  May wish to add: some elements of a discussion regarding results that consider recent (2021-2024) systematic reviews if an update is not planned or feasible.  Also, it could be relevant to add what practical implications can be derived from the results of this work. What is different or new in comparison with the latest Canadian guidelines? | Thank you for your comments on recent searches. We checked the Cochrane tobacco reviews, and three of the twenty-two included Cochrane reviews underwent a search update (i.e., Cahill 2016, Farley 2012 and Howes 2020); however, the main conclusion remained unchanged in two of the reviews. For Cahill 2016, conclusions were updated in terms of high evidence certainty surrounding varenicline and moderate evidence certainty surrounding cytisine helping more people quit smoking in comparison to placebo at the longest follow-up (6+ months). We addressed it as one of our study limitations in the discussion section. H.  Regarding differences with previous Canadian or other guidelines, this will be addressed in the Canadian Task Force on Preventive Health Care guideline informed by this review. | Reviewer#2 |
| **Question 4**  Do you have any additional comments? | No | NA | Reviewer#2 |
| **Question 1**  Are the objectives and methods of these evidence reviews clear? | The objectives of the systematic review were clear, outlining the questions that the paper aimed to address.  The paper has explained well for methods used. However, there was limited information on the randomization process of the included studies, including whether participants were aware of their treatment group and how this awareness might have influenced the study’s outcome. In addition, what had been done to control/mitigate potential confounders (for instance such as family support, physical exercise, educational level of participants etc.) in those studies. It was not very clear if included studies followed the rules/principles of RCT. | Thank you for your comments on the objectives of the paper. Since the paper is an overview of reviews, we have included Cochrane reviews as our primary studies. Therefore, the detailed RoB assessment of studies under each included systematic review can be accessed in Appendix J. | Reviewer#3 |
| **Question 2**  Were the results clearly stated? | Results of the systematic review were clearly stated and discussed. It would be helpful for readers if researchers could condense (summarize it a bit) the results. | Thank you for your notes. We have aligned with the results summarized in part II of the evidence review, i.e., E-Cigs systematic review. | Reviewer#3 |
| **Question 3**  Are the conclusions in the review supported by the data that were reviewed? | Conclusion was supported by the results. Researchers’ acknowledgement of the short duration of the studies was crucial, as it help readers/policy makers to take the result of this review with caution.  As quote from the paper: “It is also important to note that the evidence examined does not provide clarity regarding ideal intervention strategies, nor the long-term impact of these interventions for preventing smoking. We also caution readers to avoid indirect comparisons across the analyses reported within this document, whether across categories or within a category where differences in dose and duration of treatment may be reported.” | Thank you for this note. | Reviewer#3 |
| **Question 1**  Are the objectives and methods of these evidence reviews clear? | The methods used are consistent with current procedures employed in reviews and the objectives of both papers are clearly stated. However, the reader who is aware of other published work in this field (e.g. Hartmann-Boyce et al., 2022; Lindson et al., 2023) may wonder why the reviews refer to the 2016-2020 period, therefore excluding recent research. | Thank you for your notes. We have done a search update for E-cigs specifically in part II of the evidence review, which is a systematic review of e-cigs as an intervention for smoking cessation. We will be incorporating results from those new studies in part-II.  For other interventions mentioned in this overview of reviews, the evidence is not moving as fast. However, we checked the Cochrane tobacco reviews, and three of the twenty-two included Cochrane reviews underwent a search update (i.e., Cahill 2016, Farley 2012 and Howes 2020); however, the main conclusion remained unchanged in two of the reviews. For Cahill 2016, conclusions were updated in terms of high evidence certainty surrounding varenicline and moderate evidence certainty surrounding cytisine helping more people quit smoking in comparison to placebo at the longest follow-up (6+ months). We addressed it as one of our study limitations in the discussion section. H. | Reviewer#4 |
| **Question 2**  Were the results clearly stated? | There are some discrepancies in the way results are reported. It would be preferable to use the same exact manner for all sections of the reviews. | Thank you for your comment, we have reviewed the formatting. | Reviewer#4 |
| **Question 3**  Are the conclusions in the review supported by the data that were reviewed? | The discussion and conclusion sections could benefit from the addition of recommendations based on the findings of the reviews. What does it mean concretely from a clinical standpoint to have low to very low evidence certainty, and to what extent and purposes can these observations be applied to current smoking cessation practices? | Thank you for this note, we have clarified in the discussion section. | Reviewer#4 |
| **Question 4**  Do you have any additional comments? | In the Background section of both reviews, it would be preferable to refer to CCHS data instead of CTNS. It is widely acknowledged that CCHS provides more valid estimates of tobacco use prevalence in Canada. | Thank you for this note. Since the CTNS data is the recent one (i.e., 2022), we preferred using it. | Reviewer#4 |
| **Question 4**  Do you have any additional comments? | Smoking interventions in adults have been reviewed. How does this compare to other reviews of studies? Similar methodology? | Thank you, this is an important point to consider. We have added the comparative results from the USPSTF review in the discussion part. | Reviewer#5 |
| **Question 3**  Are the conclusions in the review supported by the data that were reviewed? | **The first sentence of the conclusion states “**This review provides a comprehensive synthesis of the current evidence on the effectiveness of smoking cessation interventions for preventing smoking in adults.” I would argue that due to the date of last search being 2020 and exclusion of more recent reviews, including non-Cochrane reviews, this sentence should be modified and tempered. In addition, the review does not summarize evidence for preventing smoking in adults. It summarizes evidence for assisting adults to quit smoking. | Thank you for your notes. The sentence has been reworded in the conclusion part. Also, for other interventions mentioned in this overview of reviews, We checked the Cochrane tobacco reviews, and three of the twenty-two included Cochrane reviews underwent a search update (i.e., Cahill 2016, Farley 2012 and Howes 2020); however, the main conclusion remained unchanged in two of the reviews. For Cahill 2016, conclusions were updated in terms of high evidence certainty surrounding varenicline and moderate evidence certainty surrounding cytisine helping more people quit smoking in comparison to placebo at the longest follow-up (6+ months). We addressed it as one of our study limitations in the discussion section. | Reviewer#6 |
| **Question 4**  Do you have any additional comments? | I think that the search date is quite outdated and should be updated. This review of evidence will be published four years after the latest studies were included and potentially misses any new evidence that may inform the question, thus reducing the value and effectiveness of this review.  Page 8 first paragraph: please review punctuation used in section describing sub-populations used in CAN-ADAPT recommendations. | Thank you for your notes, we have done a search update with the part-II (i.e., e-cigs) of this evidence review. | Reviewer#6 |
| **Question 2**  Were the results clearly stated? | This was an impressive review of a large amount of data and number of comparisons. The discussion section was very helpful in summarizing the review results at a higher level. | Thank you for your comments. | Reviewer#7 |
| **Question 4**  Do you have any additional comments? | One minor point. I made note different wording that was used on page 26, specifically “Varenicline *may result* in a large abstinence rate at 12-months follow-up in smokers who previously failed to quit on varenicline but were motivated to try again…” Other outcomes were not worded this way, which made me wonder if the authors were trying to imply or communicate something different about this analysis. | Thank you for your comment, we have reviewed the formatting for maintaining the consistency. | Reviewer#7 |
| **Question 1**  Are the objectives and methods of these evidence reviews clear? | The paper/methods should clarify that nicotine vapes are not actually ‘approved smoking cessation devices’ rather they are nicotine delivery systems – smoking cessation is a marketing claim, some may switch from smoking, but others the reverse. | Thank you for this note. We have given detailed information on e-cigarettes and acknowledge nicotine vapes as not approved smoking cessation intervention in Appendix A. | Reviewer#8 |
| **Question 4**  Do you have any additional comments? | This is a good paper**.** In general, the benefit/harm statements need to be bracketed to the narrow relationship to cessation claims – there is no real analysis of health benefit/harm, i.e., broader health endpoints, cancer risk, etc. | Thank you for this note. | Reviewer#8 |
| **Question 1**  Are the objectives and methods of these evidence reviews clear? | Introduction could be strengthened by stating if the scope is only Canada or international – also, age specification of adult. Flagged in Tobacco doc. | Thank you for this note. We have already acknowledged addressing the smoking cessation strategies relevant to the Canadian context under the section of current guideline recommendations in the background. We have specified the age of the included population group in the objective of the abstract and the eligibility criteria table in Appendix F. | Reviewer#9 |
| **Question 2**  Were the results clearly stated? | very | Thank you. | Reviewer#9 |
| **Question 3**  Are the conclusions in the review supported by the data that were reviewed? | This review provides a comprehensive synthesis of the current evidence on the effectiveness of smoking cessation interventions for preventing smoking in adults. | Thank you for this note. | Reviewer#9 |
| **Question 4**  Do you have any additional comments? | One sentence in particular was quite long – I feel the reader would benefit from the addition or repetition of some few key words, so you don’t have to go back to the start of sentence to remember the point. I flagged the example in Tobacco doc. Appendix J (file 10) – missing a key to define colour coding?? | Thanks. We have made those changes as advised in the manuscript. | Reviewer#9 |
| **Question 3**  Are the conclusions in the review supported by the data that were reviewed? | Yes. The conclusions are well supported, and the strength of the findings is carefully described. | Thank you for your comment. | Reviewer#10 |
| **Question 4**  Do you have any additional comments? | IT was a pleasure to review these reviews, and I look forward to their publication, as I can see much potential for practical use in my and my colleagues’ work. Thank you for the opportunity. | Thank you for your comment. | Reviewer#10 |
| **Question 1**  Are the objectives and methods of these evidence reviews clear? | Clear methods and ratings, appreciate description of AMSTAR 2, Grades of Evidence and reasons for exclusion. | Thank you for your comment. | Reviewer#11 |
| **Question 2**  Were the results clearly stated? | yes – based on strength of evidence, exclusions, and concerns of bias. As this document will be used to support decision making in primary care – is there any plan to update the reviews based on published systemic reviews from 2021 through 2023? | Thank you for your notes, but doing a search update is a significant point for future research. | Reviewer#11 |
| **Question 3**  Are the conclusions in the review supported by the data that were reviewed? | Initially I noted the difference in language from Cahill 2016 regarding varenicline, NRT, bupropion to placebo – compared to primary studies like Anthenelli 2016 (Eagles); however, reminded myself this was a review of systematic reviews and the data extracted supports the conclusions | Thank you for this note. | Reviewer#11 |
| **Question 4**  Do you have any additional comments? | I am curious why you chose to limit data to September 24^th^ 2020? Some minor flaws in how international guidelines are sourced I note you chose language of no conflicts vs authors do not report/or disclose conflict of interest | Thank you for your notes. The search limit depends on the day when the search strategy is being performed, i.e., the last search was performed on September 24^th^, 2020. We also checked the conflicts of interest (COI) reporting by different guideline groups; however, not all disclosed information on COI, except for two (i.e., USPSTF [2021] and the American Thoracic Society Clinical Practice Guideline (2020)). | Reviewer#11 |
| **Question 1**  Are the objectives and methods of these evidence reviews clear? | Yes, very clear as stated on the report. | Thank you for your comment. | Reviewer#12 |
| **Question 2**  Were the results clearly stated? | Yes, very clear as stated on the report. | Thank you for your comment. | Reviewer#12 |
| **Question 3**  Are the conclusions in the review supported by the data that were reviewed? | Yes, this review’s conclusions are supported by the data that were reviewed. | Thank you for your comment. | Reviewer#12 |
| **Question 4**  Do you have any additional comments? | - Search Strategy – I could not find on Appendix C – “Bupropion” as a search term (e.g. only "Zyban"). Please consider clarifying. - The CAN-ADDAPT guidelines also recommend NRT- combination (e.g. patch+ gum\lozenge\inhaler\spray) and Nortriptyline for smoking cessation. Unclear if these interventions were excluded or unable to pull data. I suggest further clarification. | Thanks. Zyban is the brand name for Bupropion.  We have also included data related to NRT but not Nortriptyline for smoking cessation. Please refer to Appendix K for more details. | Reviewer#12 |
| **Question 4**  Do you have any additional comments? | I am not an expert in assessing evidence reviews, but they seemed clear from my perspective. I understand the need to limit the smoking cessation intervention review to the general population and those with mental health issues, because of the vast scope of the studies available. However, since so many people who smoke have co-morbidities (cancer, heart disease, diabetes), I think it will be important to consider and articulate how the evidence for the general population could be extended/generalized to people with other diseases when making the recommendations. | Thank you, this is an important note to consider for future research. | Reviewer#13 |
