# Supplementary material for: Effectiveness of smoking cessation interventions among adults: an overview of systematic reviews
Source: Syst Rev. 2024 Jul 12;13:179. doi: 10.1186/s13643-024-02570-9 (PMC11242003; doi:10.1186/s13643-024-02570-9)
Supplement: Supplementary file 3 — Additional file 3. PRESS –peer review assessment of search strategy. [file 13643_2024_2570_MOESM3_ESM.docx]

**Additional file 3. PRESS –peer review assessment of search strategy**

# *PRESS Guideline* 2015— Search Submission & Peer Review Assessment

Reference: McGowan J, Sampson M, Salzwedel DM, Cogo E, Foerster V, Lefebvre C. PRESS Peer Review of Electronic Search Strategies: 2015 guideline statement. *J Clin Epidemiol* 2016;75:40-6. Available: <http://www.jclinepi.com/article/S0895-4356(16)00058-5/pdf>.

**Search submission: This section to be filled in by the searcher**

Searcher: Becky Skidmore	Email: [bskidmore@rogers.com](mailto:bskidmore@rogers.com)

Date submitted: 23 Oct 2018	Date requested by:	26 Oct 2018 AM

| 1. **Systematic Review Title** |  |
| --- | --- |

Effectiveness of stop smoking interventions among adults: an overview of systematic reviews and an updated systematic review

2. **This search strategy is …**

| X | My PRIMARY (core) database strategy — First time submitting a strategy for search question and database |
| --- | --- |
|  | My PRIMARY (core) strategy — Follow-up review NOT the first time submitting a strategy for search question and database. If this is a response to peer review, itemize the changes made to the review suggestions |
|  | SECONDARY search strategy— First time submitting a strategy for search question and database |
|  | SECONDARY search strategy — NOT the first time submitting a strategy for search question and database. If  this is a response to peer review, itemize the changes made to the review suggestions |

3. **Database** (e.g., MEDLINE, CINAHL)	*[mandatory]*

MEDLINE

4. **Interface** (e.g., Ovid, EbscoHost…)	*[mandatory]*

Ovid

5. **Research Question** (Describe the purpose of the search)	*[mandatory]*

What are the benefits and harms of interventions to promote cessation of tobacco smoking among adult smokers?

What is the comparative effectiveness (benefits and harms) of interventions to promote cessation of tobacco smoking among adult smokers?

What are the benefits and harms of behavioural change techniques or clusters of techniques to promote cessation of tobacco smoking among adult smokers?

6. **PICO Format** Outline the PICOs for your question — i.e., Patient, Intervention, Comparison, Outcome, and Study Design — as applicable

| **P** | Adults (≥18 years) who are current tobacco smokers (as defined by a given study/review) |
| --- | --- |
| **I / Exposure** | **KQ1a/b**: Interventions to promote abrupt (i.e., ‘all at once’) tobacco smoking cessation that can be directly delivered or referred to by primary care practitioners and are available in Canada^1^  Practitioner advice (of varying length/intensity, and by various provider types)  Very brief advice/minimal advice (as defined by a given review)  Brief advice (as defined by a given review)  Intensive individual counselling (of varying length, of varying number of sessions, and by various provider types)  Intensive group counselling (of varying length, of varying number of sessions, and by various provider types)  Self-help interventions^3^ (print-based or web/computer-based)  Internet or computer-based interventions with counselling/support^3^  Telephone-based interventions (e.g., mobile phone-based, quit lines/help lines) with counselling/support ^3^  Nicotine receptor partial agonists (varenicline and cytisine^4^)  Bupropion   - Nicotine replacement therapy^5^ (e.g., patch, gum, lozenge, mist, inhaler) - E-cigarettes^6^ - Exercise interventions - ‘Alternative’ therapies (e.g., acupuncture, acupressure, electrostimulation, hypnosis, St. John’s Wort^4^) - Combinations of interventions   Other interventions encountered in the literature will be assessed on a case-by-case basis in consultation with the WG.  The overview of reviews will seek information on the effects of variations in the delivery of stop smoking interventions (e.g., dose, duration of intervention, number of session) |
| **I / Exposure** | Interventions to promote abrupt (i.e., ‘all at once’) tobacco smoking cessation that can be directly delivered or referred to by primary care practitioners and are available in Canada^1^  **KQ1c**:  We will seek reviews which specifically examine the effectiveness of behavioural change techniques or cluster of techniques (e.g., explaining the consequences of smoking, strengthening ex-smoker identity, explaining the importance of abrupt cessation) which may be used as a component of the following behavioural change interventions:  Practitioner advice (of varying length/intensity, and by various provider types)  Very brief advice/minimal advice (as defined by a given review)  Brief advice (as defined by a given review)  Intensive individual counselling (of varying length, of varying number of sessions, and by various provider types)  Intensive group counselling (of varying length, of varying number of sessions, and by various provider types)  Self-help interventions^2^ (print-based or web/computer-based)  Internet or computer-based interventions with counselling/support^2^  Telephone-based interventions (e.g., mobile phone-based, quit lines/help lines) with counselling/support ^2^   - Combinations of interventions   Behavioural change techniques delivered as part of other behavioural change interventions (i.e., other than those listed above) will be assessed on a case-by-case basis in consultation with the Working Group.  We will seek information on intervention characteristics which may moderate the effectiveness of behavioural change techniques (e.g., duration of intervention, number of sessions) |
| **C** | **KQ1a:**   - Placebo - No intervention - Usual care - Waitlist - Minimal intervention   **KQ1b:**   - Other intervention (e.g., head-to-head comparisons, comparisons of types or intensities of advice/counselling) - Other combination of interventions - The same intervention, but used to promote cessation by reducing smoking to quit (as opposed to quitting abruptly)   **KQ1c:**   - No intervention - Usual care - Waitlist - Minimal intervention   Behavioural change techniques or cluster of techniques delivered as part of:   - Other behavioural change intervention (e.g., head-to-head comparisons, comparisons of types or intensities of advice/counselling) - Other combination of behavioural change interventions - The same behavioural change intervention, but used to promote cessation by reducing smoking to quit (as opposed to quitting abruptly) |
| **O** | Critical   - Tobacco use abstinence (as defined in a given review)   Important   - Reduction in tobacco smoking frequency/quantity - Relapse (**KQ1b only**)^7^ - Quality of life (using validated scales) - Adverse events due to treatment (as defined in a given review) - Possible adverse outcomes: - Weight gain - Changes in emotional state (e.g., increases in anxiety, changes in mood, irritability) - Loss of social group^8^ |
| **S** | Systematic reviews |

7. **Inclusion Criteria** (List criteria such as age groups, study designs, etc., to be included) *[optional]*
8.

**This search strategy is …**

Publication dates 2008-present

9. **Exclusion Criteria** (List criteria such as study designs, date limits, etc., to be excluded) **[optional]**

- Primary studies
- Editorials
- Commentaries

10. **Was a search filter applied?** No

**If YES, which one(s) (e.g., Cochrane RCT filter, PubMed Clinical Queries filter)? Provide the source if this is a published filter.** *[mandatory if YES to previous question* — *textbox]*

Modified CADTH

11. **Notes or comments you feel would be useful for the peer reviewer**	*[optional]*

Rather than itemizing all potentially available interventions, especially behavioural, the search searches generally on smoking cessation and then specifically utilizes terms that imply smoking cessation products, OR’d together.

12. **Please copy and paste your search strategy here, exactly as run, including the number of hits per line. [mandatory]**

Database: Ovid MEDLINE(R) and Epub Ahead of Print, In-Process & Other Non-Indexed Citations and Daily <1946 to September 10, 2018>

Search Strategy:

--------------------------------------------------------------------------------

1	Smoking Cessation/ (25950)

2	"Tobacco Use Cessation"/ (1027)

3	Smoking/dt [drug therapy] (961)

4	Smoking/th [therapy] (1650)

5	exp Tobacco Smoking/dt [drug therapy] (16)

6	exp Tobacco Smoking/th [therapy] (54)

7	"Tobacco Use Disorder"/dt [drug therapy] (993)

8	"Tobacco Use Disorder"/th [therapy] (1590)

9	((smoking or smoker* or tobacco* or nicotine or cigar? or cigarette* or cigarillo?) adj5 (abstain* or abstinen* or cease or ceased or ceases or cessation* or dehabituat* or desist* or discontinu* or end or ended or ending or ends or "give up" or "giving up" or "gives up" or "gave up" or halt* or quit* or stop*)).tw,kf. (37165)

10	or/1-9 [SMOKING CESSATION] (46128)

11	"Tobacco Use Cessation Products"/ (1495)

12	(nicotine adj2 replac*).tw,kf. (3113)

13	(nrt? and (abstain* or abstinen* or cease or ceased or ceases or cessation* or dehabituat* or desist* or discontinu* or end or ended or ending or ends or "give up" or "giving up" or "gives up" or "gave up" or halt* or quit* or stop*)).tw,kf. (1386)

14	(nrt? and (smoking or smoke? or smoker? or cigar? or cigarette* or cigarillo? or nicotine or tobacco*)).tw,kf. (1166)

15	(nicotine adj3 (gum? or lozenge? or mist* or patch* or spray* or tablet* or transdermal* or inhalant? or inhaler? or inhalator? or inhalation*)).tw,kf. (2890)

16	(nrt? adj3 (gum? or lozenge? or mist* or patch* or spray* or tablet* or transdermal* or inhalant? or inhaler? or inhalator? or inhalation*)).tw,kf. (142)

17	(nicoderm* or nicorette*).tw,kf. (116)

18	((commit$2 or thrive$2) adj5 (gum? or lozenge? or nicotine or nrt?)).tw,kf. (9)

19	Varenicline/ (1124)

20	(varenicline or champix* or chantix* or CP 526555-18 or UNII-82269ASB48).tw,kf. (1550)

21	(cytisine or baptitoxin? or cravv or cystisine or cytiton? or cytizin or desmoxan or laburnin or sophorin? or tabax or tabex or tsitizin or ulexin? or "BRN 0083882" or EINECS 207-616-0 or HSDB 3560 or NSC 407282 or UNII-53S5U404NU).tw,kf. (800)

22	(Zyban* or Zyntabac*).tw,kf. (126)

23	Electronic Nicotine Delivery Systems/ (1953)

24	(electronic adj (cigar* or nicotine* or smok* or tobacco*)).tw,kf. (2288)

25	(ecig* or e-cig*).tw,kf. (2668)

26	Vaping/ (182)

27	(vape or vaped or vapes or vaping).tw,kf. (544)

28	or/11-27 [SMOKING CESSATION PRODUCTS] (11435)

29	10 or 28 [SMOKING CESSATION, INCLUDING PRODUCTS] (50662)

30	limit 29 to systematic reviews (2703)

31	meta analysis.pt. (92353)

32	exp meta-analysis as topic/ (16897)

33	(meta-analy* or metanaly* or metaanaly* or met analy* or integrative research or integrative review* or integrative overview* or research integration or research overview* or collaborative review*).tw,kf. (138405)

34	(systematic review* or systematic overview* or evidence-based review* or evidence-based overview* or (evidence adj3 (review* or overview*)) or meta-review* or meta-overview* or meta-synthes* or "review of reviews" or technology assessment* or HTA or HTAs).tw,kf. (170726)

35	exp Technology assessment, biomedical/ (10488)

36	(cochrane or health technology assessment or evidence report).jw. (15713)

37	(network adj (MA or MAs)).tw,kf. (4)

38	(NMA or NMAs).tw,kf. (1708)

39	indirect* compar*.tw,kf. (1564)

40	(indirect treatment* adj1 compar*).tw,kf. (176)

41	(mixed treatment* adj1 compar*).tw,kf. (444)

42	(multiple treatment* adj1 compar*).tw,kf. (140)

43	(multi-treatment* adj1 compar*).tw,kf. (0)

44	simultaneous* compar*.tw,kf. (962)

45	mixed comparison?.tw,kf. (21)

46	or/31-45 (291926)

47	29 and 46 (2028)

48	30 or 47 (3143)

49	exp Child/ not (exp Child/ and exp Adult/) (1131536)

50	exp Child/ not (exp Child/ and Adolescent/) (928484)

51	exp Infant/ not (exp Infant/ and exp Adult/) (789467)

52	exp Infant/ not (exp Infant/ and Adolescent/) (781112)

53	or/49-52 (1809112)

54	48 not 53 [CHILD-/INFANT-ONLY REMOVED] (3032)

55	(editorial or news or newspaper article).pt. (675419)

56	(letter not (letter and randomized controlled trial)).pt. (994523)

57	54 not (55 or 56) [OPINION PIECES REMOVED] (2976)

58	limit 57 to yr="2008-current" (2061)

***************************

**Peer review assessment: this section to be filled in by the reviewer**

|  | Reviewer: Kaitryn Campbell | Email: [kcamlolo668@gmail.com](mailto:kcamlolo668@gmail.com) | Date completed: 26 Oct 2018 |
| --- | --- | --- | --- |
|  |  |  |  |

Do you wish to be acknowledged? (If yes, the review team will be advised to add an acknowledgement to any publications related to this work). No – unless your organization requires it

The suggested acknowledgement is “ We thank Xxxxx Yyyyyy, MLIS, AHIP (xxxxx Health Sciences Library, University of xxxxxx) for peer review of the MEDLINE search strategy.” [please edit to indicate your name, postnomials and institutional affiliation as you would like them presented].

|  | **1. TRANSLATION** |  |  | | |
| --- | --- | --- | --- | --- | --- |
|  | |  | |  |  |
|  | | A -­‐No revisions | | X |  |
|  | | B -­‐ Revision(s) suggested | |  |  |
|  | | C -­‐ Revision(s) required | |  |  |

If “B” or “C,” please provide an explanation or example:

**2. BOOLEAN AND PROXIMITY OPERATORS**

| A -­‐No revisions | X |
| --- | --- |
| B -­‐ Revision(s) suggested |  |
| C -­‐ Revision(s) required |  |

If “B” or “C,” please provide an explanation or example:

**3. SUBJECT HEADINGS**

| A -­‐No revisions | X |
| --- | --- |
| B -­‐ Revision(s) suggested |  |
| C -­‐ Revision(s) required |  |

If “B” or “C,” please provide an explanation or example:

**4. TEXT WORD SEARCHING**

| A -­‐No revisions | X |
| --- | --- |
| B -­‐ Revision(s)suggested |  |
| C -­‐ Revision(s) required |  |

If “B” or “C,” please provide an explanation or example:

**5. SPELLING, SYNTAX, AND LINE NUMBERS**

| A -­‐No revisions | X |
| --- | --- |
| B -­‐ Revision(s)suggested |  |
| C -­‐ Revision(s) required |  |

If “B” or “C,” please provide an explanation or example:

**6. LIMITS AND FILTERS**

| A -­‐No revisions | X |
| --- | --- |
| B -­‐ Revision(s) suggested |  |
| C -­‐ Revision(s) required |  |

If “B” or “C,” please provide an explanation or example:

OVERALL EVALUATION (Note: If one or more “revision required” is noted above, the response below must be “revisions required”.)

| A -­‐No revisions | X |
| --- | --- |
| B -­‐ Revision(s) suggested |  |
| C -­‐ Revision(s) required |  |

Additional comments:

Solid, comprehensive search with no errors or omissions. I agree that using “tobacco use/cessation” concept combined with the filter is the most reasonable/logical method of proceeding, especially given the size of the final search result set.
